# Intrathalline Fungal and Bacterial Diversity Is Uncovered in Antarctic Lichen Symbioses

**DOI:** 10.1111/1758-2229.70080

**Published:** 2025-05-05

**Authors:** Gerardo A. Stoppiello, Roberto De Carolis, Claudia Coleine, Mauro Tretiach, Lucia Muggia, Laura Selbmann

**Affiliations:** ^1^ Department of Life Sciences University of Trieste Trieste Italy; ^2^ Largo Dell' Università, Department of Ecological and Biological Sciences University of Tuscia Viterbo Italy; ^3^ Italian Antarctic National Museum (MNA) Mycological Section Genoa Italy

**Keywords:** biodiversity, community composition, endemism, microbiota, Victoria land

## Abstract

Although the Antarctic continent represents one of the most hostile environments on earth, microbial life has adapted to cope with these extreme conditions. Lichens are one of the most successful groups of organisms in Antarctica, where they serve as unique niches for microbial diversification. We have selected eight epilithic lichen species growing in Victoria Land (three cosmopolitan and five endemic to Antarctica) to describe with amplicon sequencing the diversity of the associated fungal and bacterial communities. The lichen mycobiota is predominantly composed of *Ascomycota* belonging to the classes *Chaetothyriomycetes* and *Dothideomycetes*, while a few key representative taxa were recognised as basidiomycetous yeasts. Bacteria associated with lichens were represented by *Pseudomonadota*, *Cyanobacteria*, and *Bacteroidota* in which psychrophilic genera were identified. The microbiota was diverse among the lichen species, and their variation was driven by the lichen species itself and their endemic or cosmopolitan distribution. There was a strong association of the microbial communities linked to the lichen itself, rather than to the specific characteristics of the collecting site. The lichen thallus, thus, plays an important role in microbial diversification and may potentially act as a selective biodiversity filter in which different fungal and bacterial communities thrive in it.

## Graphical abstract

Antarctic endemic lichens host a greater biodiversity and rare species of fungi and bacteria within the thallus than cosmopolitan lichens.

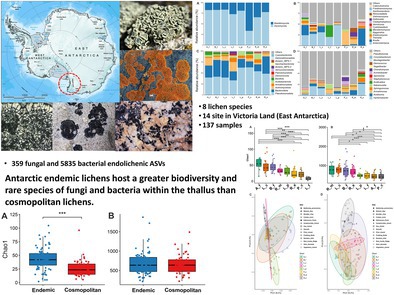

## 1 Introduction

Antarctica represents one of the most hostile environments on Earth (Øvstedal and Lewis Smith 2001; Selbmann et al. 2013). Here, vascular plants do not settle and are replaced by cryptogamic communities composed of mosses and, above all, lichens. Mosses and lichens spread along the coasts on rocks and soil, but lichens are the only ones able to settle even throughout the interior areas of the continent. As a result, lichens represent an important group of organisms in this extreme environment, with a high percentage of endemism (33%–50% in the continental Antarctica): 130 endemic lichen species have been reported so far from a total Antarctic flora of 393 species (Øvstedal and Lewis Smith 2001; Castello and Nimis 1997, 2000).

Lichens are indeed notable examples of self‐sustaining, long‐living, symbiotic systems that derive from the mutualistic associations between biotrophic fungi (the mycobionts) and phototrophic green microalgae or cyanobacteria (the photobionts, i.e. chlorobionts and the cyanobionts, respectively; Hawksworth and Honegger 1994). In addition, lichen thalli host a multiplicity of other microorganisms (Honegger 2012; Hawksworth and Grube 2020). The two main lichen symbionts have co‐evolved into peculiar phenotypes–building up three‐dimensional structures, that is, the lichen thalli–and have been able to adapt to the harshest environments on Earth (Lutzoni et al. 2001; Onofri et al. 2007). Despite the ecological roles of the lichen‐inhabiting fungi (i.e., the lichen mycobiota; Fernández‐Mendoza et al. 2017) and bacteria being still largely unknown, their presence has been correlated with the growth form and the ecology of the lichen hosts (Harutyunyan et al. 2008; Arnold et al. 2009; U'Ren et al. 2010, 2012, 2014; Muggia et al. 2016; Fernández‐Mendoza et al. 2017; Muggia and Grube 2018; Smith et al. 2020; Spribille 2018). So far, these lichen‐associated microorganisms were supposed to be active players for the ecological success of lichen symbioses, also in extreme environments (Grube et al. 2009, 2015; Grube and Berg 2009; Muggia and Grube 2018), but still, it has not been possible to report on the practical evidence of their potential roles.

To date, studies have mainly focused on non‐lichenized fungi and bacteria associated with the endolithic communities and were based on culture isolation and genetic identification of the grown strains (i.e., de la Torre et al. 2003; Selbmann et al. 2005, 2008; Egidi et al. 2014). More recently, thanks to the advent of high‐throughput sequencing techniques (HTS; Greco et al. 2021; Ji et al. 2013; Smith and Peay 2014; Tedersoo et al. 2015), a more accurate census of the viral, eukaryotic, and prokaryotic assemblages of Antarctic microbial communities has been reached (de la Torre et al. 2003; Archer et al. 2017; Coleine et al. 2018a, 2018b, 2019, 2021; Coleine, Gevi et al. 2020; Coleine, Masonjones et al. 2020; Albanese et al. 2021; Mezzasoma et al. 2022; Ettinger et al. 2023). On the other hand, only two studies have focused on microbial diversity from epilithic lichens in Antarctica. These correspond to the first pioneer study from Selbmann et al. (2013), who considered 13 species of epilithic lichens collected in Northern and Southern Victoria Land. Later, Santiago et al. (2015) reported ascomycetes, basidiomycetes, and taxa formerly recognised as zygomycetes from multiple thalli of the two endemic macrolichen species *Usnea antarctica* and *U. aurantiaco‐atra* from the Antarctic Peninsula.

To address this knowledge gap, in the present study we collected Antarctic epilithic lichens, both endemic and cosmopolitan, with the main aim to characterise by HTS their inhabiting microbial diversity comprising microfungi and bacteria. In particular, we studied five Antarctic endemic and three worldwide distributed (hereon referred to as ‘cosmopolitan’) lichen species from 14 localities in the Victoria Land to: (i) define the fungal and bacterial diversity associated with the lichen thalli; (ii) verify which factor is determinant for shaping the associated assemblages; (iii) determine the presence of a potential group of taxa that are uniquely associated either with cosmopolitan or endemic lichen species.

## 2 Material and Methods

### 2.1 Study Area and Sampling

**FIGURE 1:**
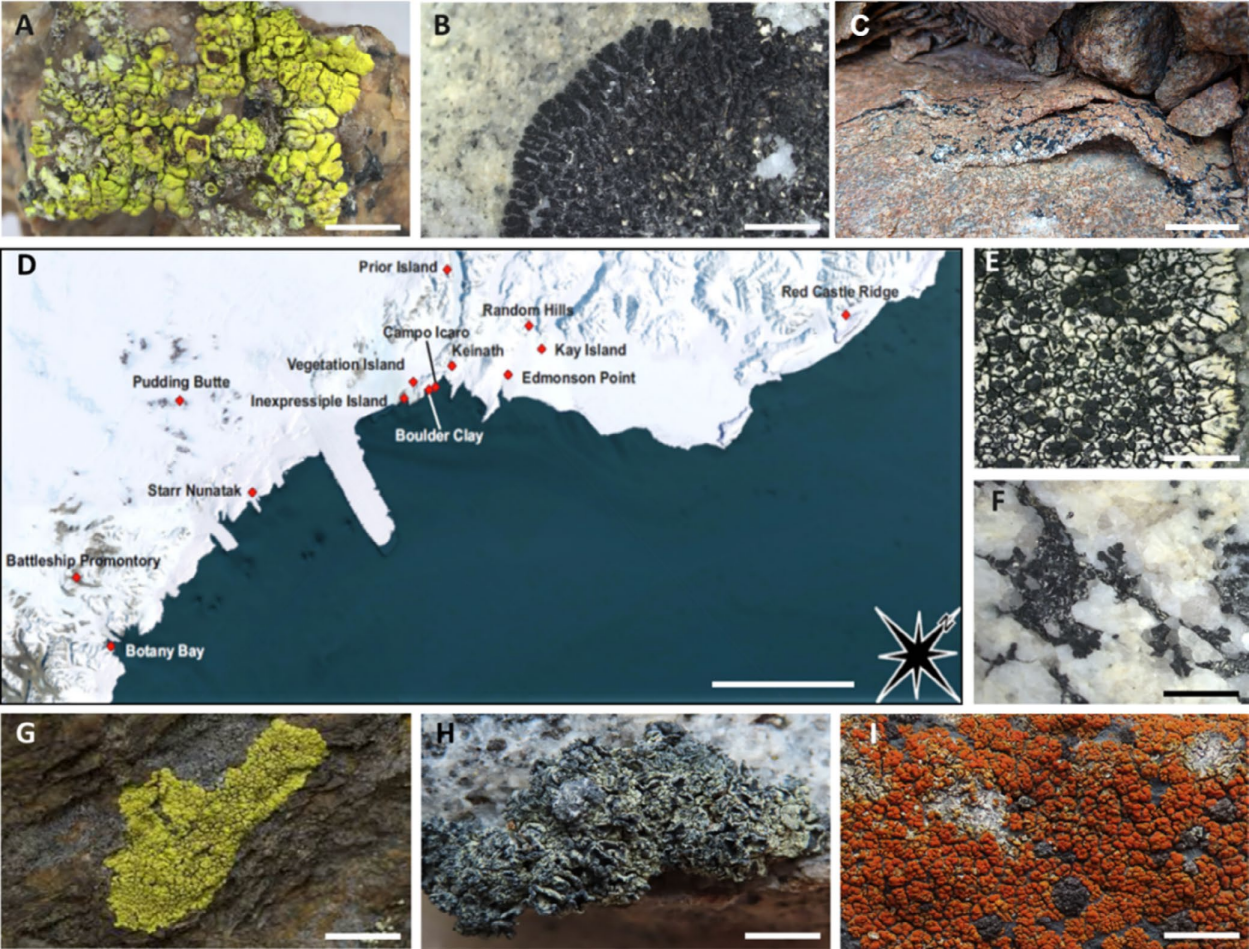
Lichen species and sampling area. (A) *A. flavocordia*; (B) *B. frigida* ; (C) *L. fuscobrunnea*, (D) collecting sites in Victoria Land (Antarctica) visited for the sampling of the eight lichen species here analysed; (E) *L. physciella*; (F) *L. cancriformis*; (G) *P. chlorophanum*; (H) *R. melanophthalma* ; (I) *R. elegans* . Scale bars: (A–C, E–H) 1 cm; (I) 2 cm.

The sampling campaign took place during the 37° Antarctic expedition, November 2021–February 2022. In this campaign, 14 selected localities have been visited in Northern Victoria Land, within the south latitudinal range from the 72° to 77° parallel (Figure 1, Table 1). In each locality, when present and feasible to collect, at least three individuals of each selected lichen species were sampled (Table 1). The variability of lichen species and thalli collected in each locality is primarily due to the availability of lichen specimens in the field and the sampling conditions in which the operator (i.e., one of the authors) had to collect. Sampling conditions are determined by the extreme environmental conditions; the logistic and climatic constraints often limit the time available for searching, identifying, and collecting samples at the localities. Thus, a homogeneity of the number of samples, the same number and type of specimens in all sampling sites could not be guaranteed. Indeed, there are cases in which the lichens were extremely rare, and thus less than three thalli for each occurring species were collected and used for the molecular analyses (Table 1; for example in Key Island only one thallus of *Rhizoplaca melanophthalma* was collected and used for the amplicon sequences analyses). The samples were aseptically collected with the rock substrate using a geological hammer, placed into sterile plastic bags, and stored dry at −20°C, also during the transportation to the laboratories, until downstream analyses.

**TABLE 1:** Sampling information of localities and the collected lichen species.

Locality	Coordinates	Endemic lichens	Cosmopolitan lichens	Samples used for amplicon sequencing analyses (endemics; cosmopolitan)
Botany Bay	77°00′00″ S 162°35′00″ E	*B. frigida* (6), *L. cancriformis* (6)	*R. elegans* (4)	*B. frigida* (3), *L. cancriformis* (3); *R. elegans* (3)
Inexpressible Island	74°53′21.5″S 163°44′43.8″ E	*B. frigida* (8), *L. cancriformis* (5)	*R. elegans* (6)	*B. frigida* (3), *L. cancriformis* (3); *R. elegans* (3)
Vegetation Island	74°47′03″ S 163°39′35″ E	*B. frigida* (5), *L. physciella* (3)	*P. chlorophanum* (3), *R. elegans* (6)	*B. frigida* (3), *L. physciella* (3); *P. chlorophanum* (3), *R. elegans* (3)
Prior Island	75°41′30″ S 162°52′43″ E	*B. frigida* (6), *L. cancriformis* (5)	—	*B. frigida* (3), *L. cancriformis* (3)
Key Island	74°04′13″ S 165°18′57″ E	*A. flavocordia* (12), *B. frigida* (5), *L. cancriformis* (7), *L. physciella* (7)	*R. elegans* (7), *R. melanophthalma* (1)	*A. flavocordia* (3), *B. frigida* (3), *L. cancriformis* (3), *L. physciella* (3); *R. elegans* (3), *R. melanophthalma* (1)
Starr Nunatak	75°53′54.7″S 162°35′34.4″ E	*B. frigida* (11), *L. cancriformis* (15)	*R. elegans* (12)	*B. frigida* (3), *L. cancriformis* (3); *R. elegans* (3)
Mt Keinath	74°55′S 164°00′ E	*L. cancriformis* (1)	*P. chlorophanum* (6)	*L. cancriformis* (1); *P. chlorophanum* (3)
Battleship Promontory (McMurdo Dry Valleys)	76°54′S 160°50′00.0″ E	*L. fuscobrunnea* (7)	—	*L. fuscobrunnea* (3)
Edmonson Point	74°19,902′S 165°08,223′ E	—	*R. elegans* (6)	*R. elegans* (3)
Random Hills site 2	74°0.1′0.15″S 164°21′23.4″ E	*A. flavocordia* (8), *B. frigida* (4), L. *cancriformis* (6)	*R. elegans* (5)	*A. flavocordia* (3), *B. frigida* (3), L. *cancriformis* (3); *R. elegans* (3)
Random Hills site 3	74°0.0′10.9″S 164°28′56.4″ E	*A. flavocordia* (1), *B. frigida* (6)	*P. chlorophanum* (7), *R. elegans* (6), *R. melanophthalma* (2)	*A. flavocordia* (1), *B. frigida* (3); *P. chlorophanum* (3), *R. elegans* (3), *R. melanophthalma* (2)
Random Hills site 4	74°0.1′42.2″S 164°44′44.5″ E	*A. flavocordia* (11), *B. frigida* (5), *L. physciella* (8)	*R. elegans* (6), *R. melanophthalma* (21)	*A. flavocordia* (3), *B. frigida* (3), *L. physciella* (3); *R. elegans* (3), *R. melanophthalma* (3)
Random Hills site 5	74°0.4′33.4″S 164°48′32.5″ E	*A. flavocordia* (8), *L. physciella* (15)	*R. melanophthalma* (3)	*A. flavocordia* (3), *L. physciella* (3); *R. melanophthalma* (3)
Pudding Butte	75°51′30.2″S 159°58′25.7″ E	*L. fuscobrunnea* (2)	—	*L. fuscobrunnea* (2)
Red Castle Ridge	72°26′00″ S 169°57′00″ E	*B. frigida* (5), *L. cancriformis* (5)	*R. elegans* (7)	*B. frigida* (3), *L. cancriformis* (3); *R. elegans* (3)
Campo Icaro	74°42′33.5″S 164°05′45.9″ E	*B. frigida* (6), *L. cancriformis* (6)	—	*B. frigida* (3), *L. cancriformis* (3)
Boulder Clay	74°44′6.8″S 164°02′22.50″ E	*L. fuscobrunnea* (1)	*P. chlorophanum* (16)	*L. fuscobrunnea* (1); *P. chlorophanum* (3)

*Note:* The number of thalli collected and used for amplicon sequencing analyses from each site and lichen species is shown in parentheses.

The collected and analysed lichen species are all chlorolichens (i.e., forming symbiosis with green trebouxioid photobionts): the endemic Antarctic *Acarospora flavocordia, Buellia frigida, Lecanora fuscobrunnea, Lecanora physciella*, and *Lecidea cancriformis*, and the cosmopolitan *Pleopsidium chlorophanum, Rhizoplaca melanophthalma*, and *Rusavskia elegans*. The lichen species were chosen based on literature knowledge and their availability at the sampling sites.

### 2.2 DNA Extraction, Amplification and Sequencing of Fungal and Bacterial Communities

For the amplicon sequencing analyses, up to three thalli of each species from each locality were used. Thus, the amplicon sequencing analysis was performed on a total of 140 environmental samples belonging to the eight lichen species (*A. flavocordia*, *B. frigida* , *L. fuscobrunnea*, *L. physciella*, *L. cancriformis*, *P. chlorophanum*, *R. melanophthalma*, *R. elegans* ; Table 1). Metagenomic DNA extraction was performed on a 0.5 cm^2^ fragment of the thallus, devoid of any symptoms of external infection or damage, which was removed from the substrate using a sterile razor blade and transferred into a 1.5 mL reaction tube. The fragments underwent a series of washes: they were rinsed three times for 15 min with sterile water, followed by a 30 min cleaning step using a 2% Tween 80 solution. A final wash was performed for 15 min with sterile water. The DNA extraction from the cleaned fragments followed the CTAB protocol of Cubero et al. (1999), with minor adjustments. Potential contaminants during the DNA extraction process were checked by establishing negative control samples for each lichen species. These negative controls consisted of a 1.5 mL tube kept open during the whole extraction process and processed in the same way as the lichen samples for the amplification and sequencing steps.

To study the diversity of fungal communities, the nuclear ribosomal internal transcribed spacer 1 region (ITS1) was targeted. The ITS1 region was amplified using the barcoded primers ITS1F (CTTGGTCATTTAGAGGAAGTAA) and ITS2 (GCTGCGTTCTTCATCGATGC; White et al. 1990; Smith and Peay 2014). The PCR amplification protocol for the ITS1 fragments was run with the following conditions: an initial denaturation at 94°C for 3 min, followed by 35 cycles of denaturation at 94°C for 45 s, annealing at 55°C for 45 s, extension at 72°C for 1 min, and a final extension step at 72°C for 5 min.

The V4 variable region of the small ribosomal subunit 16S rDNA was used to assess bacterial diversity. This variable region was amplified using the primers F515 (GTGCCAGCMGCCGCGGTAA) and R806 (GGACTACHVGGGTWTCTAAT) as described by Caporaso et al. (2012). The PCR amplification of the V4 variable region was performed with the following protocol: an initial denaturation at 94°C for 3 min, followed by 35 cycles of denaturation at 94°C for 45 s, annealing at 50°C for 1 min, extension at 72°C for 90 s, and a final extension step at 72°C for 10 min.

Samples were then sequenced in paired ends (2 × 300 bp) using the Illumina MiSeq platform at the Edmund Mach Foundation (San Michele all'Adige, Italy).

### 2.3 Bioinformatics Analyses

The samples were processed after checking for extraction contamination with Decontam (Davis et al. 2018). Raw reads were analysed using the Amplicon ToolKit (AMPtk) for Next Generation Sequence (NGS) v1.2.1 (Palmer et al. 2018), USEARCH (Edgar 2010) and VSEARCH (Rognes et al. 2016). The reads were trimmed, resulting in sequences with a length of 250 bp; reads shorter than 100 bp were discarded, and chimera removal was performed using USEARCH v. 9.2.64 with default parameters. Sequence quality filtering was performed with the expected error parameter of < 1.0 (Palmer et al. 2018). The dataset was clustered with DADA2 v1.6.0 using a 99% identity parameter to generate the Amplicon Sequence Variants (ASVs). Further filtering was performed in which rare ASVs (i.e., ASVs having less than five reads), singletons, and chimeras were discarded and not considered for the subsequent analyses according to Lindahl et al. (2013). The taxonomy assignment was performed via the UNITE v10.5 (2023) and RDP release 11.7.2023 (Cole et al. 2014) database, which uses the hybrid SYNTAX algorithm (Abarenkov et al. 2020; Edgar 2010); the sequences were aligned, and the taxonomy was assigned to the corresponding ASVs of the ITS and 16S. Regarding the bacteria dataset, we excluded both mitochondria and chloroplasts from the analysis.

### 2.4 Statistical Analyses

To test the effectiveness of the sampling effort of the overall lichen‐associated fungal and bacterial diversity in the studied area, species accumulation curves were calculated using the specaccum function in the ‘vegan’ package (Oksanen et al. 2013). Because of the dishomogeneous number of species and samples collected for each species in each locality, we calculated species accumulation curves for bacterial and fungal reads both for the entire dataset and for individual species.

The Linear discriminant Effect Size (LEfSe) analysis was performed to identify those taxa that explain the differences between endemic and cosmopolitan lichens myco‐ and microbiota (Segata et al. 2011).

To explore fungal and bacterial diversity, the analyses were done by clustering the ASVs by lichen mycobiont species (eight species) and by their ecological distribution (i.e., Antarctic endemic vs. cosmopolitan). Alpha diversity was calculated with the Chao1 index, beta diversity was calculated using the Bray– Curtis dissimilarity index, and PCoA analyses were performed and tested with PERMANOVA. All analyses were performed using R packages *microeco* (Liu et al. 2021) and *phyloseq* (McMurdie and Holmes 2013). Kruskal–Wallis and Wilcoxon tests (Kruskal and Wallis 1952; Wilcoxon 1945) were used to assess significant diversity in differences among lichen species and between groups with different ecological distributions. To explore the correlation in terms of Shannon diversity between fungi and bacteria, we conducted a Linear Regression Analysis calculated with the ‘vegan’ package (Oksanen et al. 2013). The sequencing depth, defined as the total number of reads per sample after quality filtering, was calculated using the *phyloseq* package (McMurdie and Holmes 2013) in R for both individual samples (for each species) and types of ecological groups (endemic and cosmopolitan).

## 3 Results

### 3.1 Taxonomic Composition of Myco‐ and Microbiota

The outcomes of sequencing returned 10,603,745 reads for fungi and 18,438,804 for bacteria, which were clustered at 99% identity and collapsed into 2073 and 24,784 ASVs, respectively. To specifically investigate the lichen mycobiota, a further filtering was applied: all ASVs matching any lichen‐forming fungi (i.e., any lichen mycobionts known) were excluded from the analysis (Table S1). This resulted in a final dataset that exclusively comprised fungal taxa classified as not lichen‐forming fungi. The complete datasets for fungi and bacteria were finally obtained by removing chimeras, singletons, contaminants, and archaeal sequences, resulting in a total of 359 fungal and 5835 bacterial ASVs. Unassigned ASVs were not included in the analyses, but they are presented and available in the (Tables S7 and S8, Supporting Informations 1 and 2). The sequencing depth showed no significant differences between the endemic and cosmopolitan samples analyzed for both fungal and bacterial data, *p* value > 0.05 (Table S9).

We performed accumulation curves for bacteria and fungal reads both for the entire dataset and for individual species (Figures S1 and S2). When examining individual lichen species, the species accumulation curves for fungi and bacteria level off for only a few lichen species (Figures S2A,B). This suggests that individual lichen species alone do not account for the full diversity observed in the dataset. Conversely, when considering all species collectively (Figures S1), we observe that the species accumulation curves gradually levelled off for both fungi and bacteria, indicating that the number of samples analysed approaches an adequate number to encompass the biodiversity of the studied communities (Figure 1).

**FIGURE 2:**
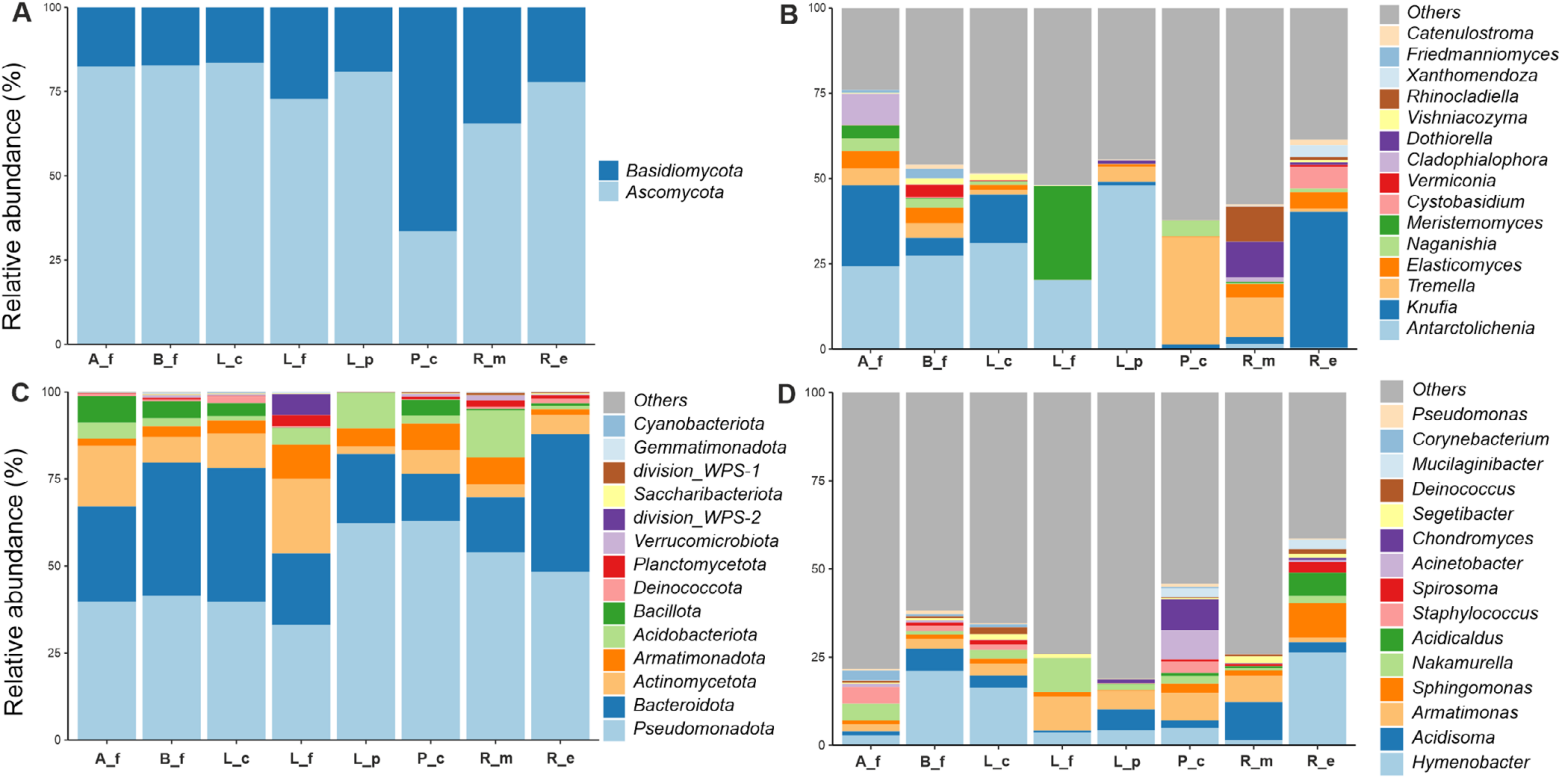
Relative abundance plots of fungi (A, C) and bacteria (B, D) reported for each lichen species; the 15 most frequently recovered genera are shown. Lichen species names were abbreviated as follows: *Acarospora flavocordia* (A_f), *Buellia frigida* (B_f), *Lecidea cancriformis* (L_c), *Lecanora fuscobrunnea* (L_f), *Lecanora physciella* (L_p), *Pleopsidium chlorophanum* (P_c), *Rusavskia elegans* (R_e), *Rhizoplaca melanophthalma* (R_m). Abundance data are shown in percentage. “*Others*” includes all the other genera and higher taxonomic ranks which were not among the 15 most frequently recovered ones.

The relative abundance of the community analyses (Figure 2) of the mycobiota revealed *Ascomycota* and *Basidiomycota* as the dominant phyla (Figure 2A). In seven (e.g., *A. flavocordia*, *B. frigida* , *L. physciella*, *L. cancriformis*, *R. melanophthalma* , *R. elegans, L. fuscobrunnea*) out of the eight lichen species, the phylum *Ascomycota* constituted the majority of ASVs, whereas the phylum *Basidiomycota* was predominant only in the thallus of *P. chlorophanum*. Based on relative abundances (Figure 2C), it emerged that the most represented genera were *Antarctolichenia* in *A. flavocordia* (24,3%), *B. frigida* (27,4%), *L. cancriformis* (31,14%), *L. fuscobrunnea* (20,3%), *L. physciella* (48%); *Knufia in R. elegans (40%)* and *Tremella* in *P. chlorophanum* (31,5%), *R. melanophthalma* (11,5%).

The analyses of the bacterial communities revealed *Pseudomonadota* as the dominant phylum in all the lichen species (Figure 2B). Looking at the relative abundance of bacteria at the genus level (Figure 2D), we found that most of the genera were: *Hymenobacter* in *R. elegans* (26,3%), *B. frigida* (21%), *L. cancriformis* (16,2%); *Acidisoma* in *R. melanophthalma* (10%) and *L. physciella* (6%); *Armatomonas* in *L. fuscobrunnea* (9,69%) and *P. chlorophanum* (7,64%); and *Staphylococcus* (4,81%) in *A. flavocordia*. Under “*Others*” we included all the other genera and higher taxonomic ranks that were not among the 15 most frequently recovered ones.

### 3.2 Alpha Diversity Analyses

**FIGURE 3:**
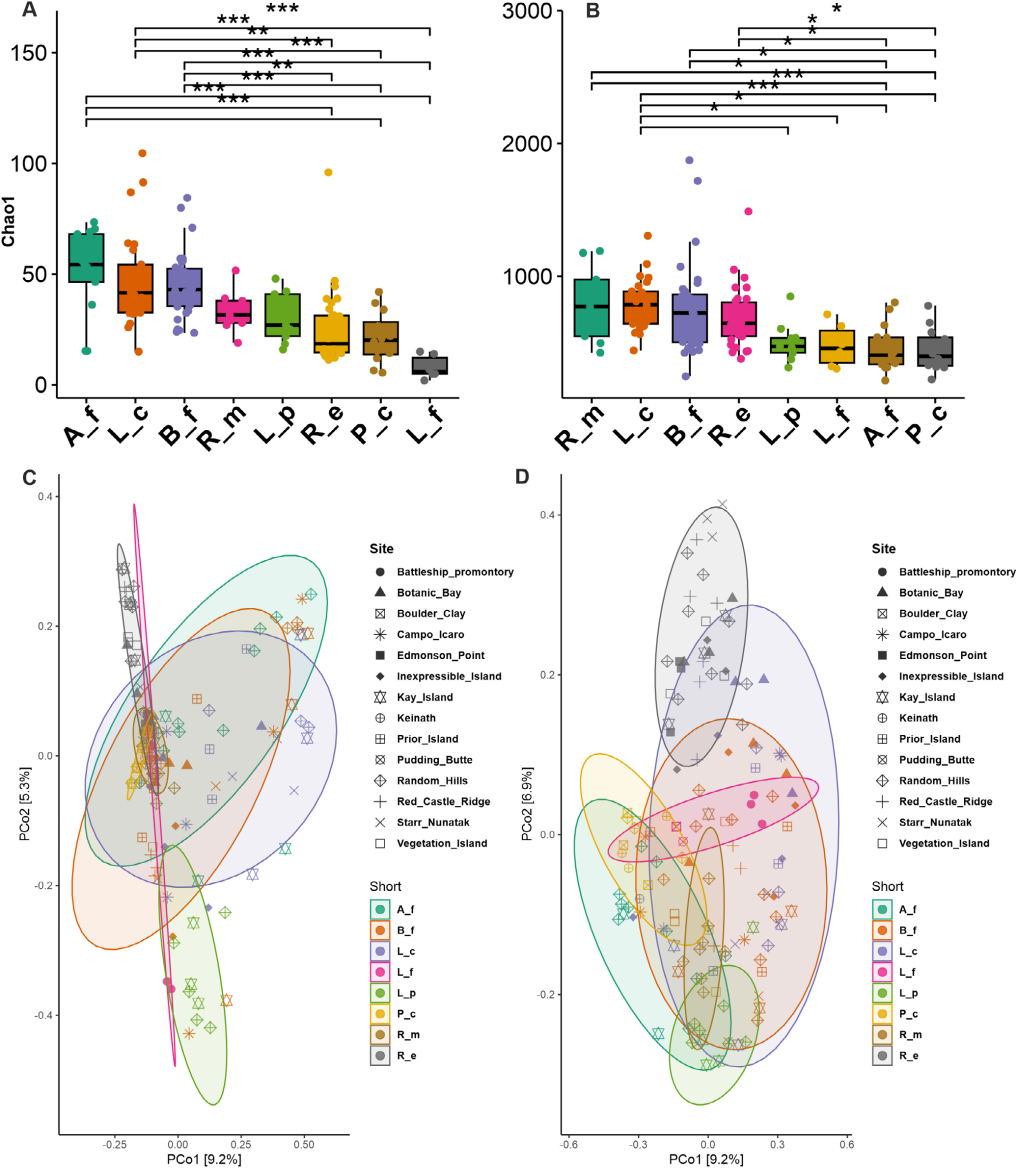
(A, B) Alpha diversity and Chao1 index plots of fungal (A) and bacteria (B) communities. Statistical differences between groups, tested by Kruskal‐Wallis test were marked with ***p < 0.001, **p < 0.01, *p < 0.05. (C, D) Beta diversity and principal component analysis (PCoA) based on Bray‐Curtis distance including ellipse of 95% confidence interval of fungal (C) and bacteria (D) communities. On the axes and legends lichen species names were abbreviated as follow: *Acarospora flavocordia* (A_f), *Buellia frigida* (B_f), *Lecidea cancriformis* (L_c), *Lecanora fuscobrunne*a (L_f), *Lecanora physciella* (L_p), *Pleopsidium chlorophanum* (P_c), *Rusavskia elegans* (R_e), *Rhizoplaca melanophthalma* (R_m).

The Chao1 indexes for fungal and bacterial diversity are shown in Figure 3. Mycobiota of *A. flavocordia* (Chao1 = 52) and *L*. *cancriformis* (Chao1 = 47.43) presented the highest Chao1 values among the lichen species, while *L. fuscobrunnea* displayed the lowest value (Chao1 = 8; Figure 3A). The Kruskal–Wallis Dunn test, conducted for multiple comparisons within lichen species, revealed significant statistical differences between *L. fuscobrunnea* and *A. flavocordia*, *B. frigida* , and *L. cancriformis* (*p* value < 0.001). Moreover, statistical differences were observed between *P. chlorophanum* and *A. flavocordia* (*p* value < 0.001), *B. frigida* (*p* value < 0.01), and *L. cancriformis* (*p* value < 0.01). *R. elegans* displayed statistical differences with *A. flavocordia* (*p* value < 0.001), *B. frigida* (*p* value < 0.001), and *L. cancriformis* (*p* value < 0.001; Tables [Link], [Link], [Link], [Link] report the detailed statistic values).

Looking at the bacterial assemblages, *R. melanophtalma, L. cancriformis*, and *B. frigida* displayed the highest Chao1 values (797, 789, and 765, respectively), while *P. chlorophanum* (Chao1 = 447) the lowest (Figure 3B). *L. cancriformis* showed statistical differences (*p* value < 0.001) with *A. flavocordia* and *P. chlorophanum; with L. fuscobrunnea* and *L. physciella* (*p* value < 0.05). *B. frigida* shows statistical differences (*p* value < 0.05) with *A. flavocordia* and *P. chlorophanum*. *R. melanophtalma* showed differences (*p* value < 0.05) with *A. flavocordia* and *P. chlorophanum. Also*, statistical differences were shown for *R. elegans* against *A. flavocordia* and *P. chlorophanum* (*p* value < 0.05; Figure 3B). Moreover, to understand the relationship between the microbial diversity of bacteria and fungi in our Antarctic epilithic communities, we conducted a linear regression analysis between the Shannon indices of the bacterial and fungal communities (Figure S3). The scatter plot clearly shows the linear relationship between the two indices, indicating that an increase in bacterial diversity is associated with an increase in fungal diversity in our sampled communities (Figure S3).

### 3.3 Beta Diversity Analyses

The Bray‐Curtis (incidence and relative abundance data) distance was used to assess the beta diversity and the differences in fungal and bacterial assemblages among lichen species. The results were presented through PCoA plots along with PERMANOVA statistical analysis. The effects of the lichen species and ecological distribution (i.e., endemic vs. cosmopolitan) were found to be significant (*p* value < 0.001), although the ecological distribution seemed to have a lesser impact on the community ordination than that of the lichen species (Figure 3C,D).

### 3.4 Cosmopolitan Versus Endemic Lichen Species

**FIGURE 4:**
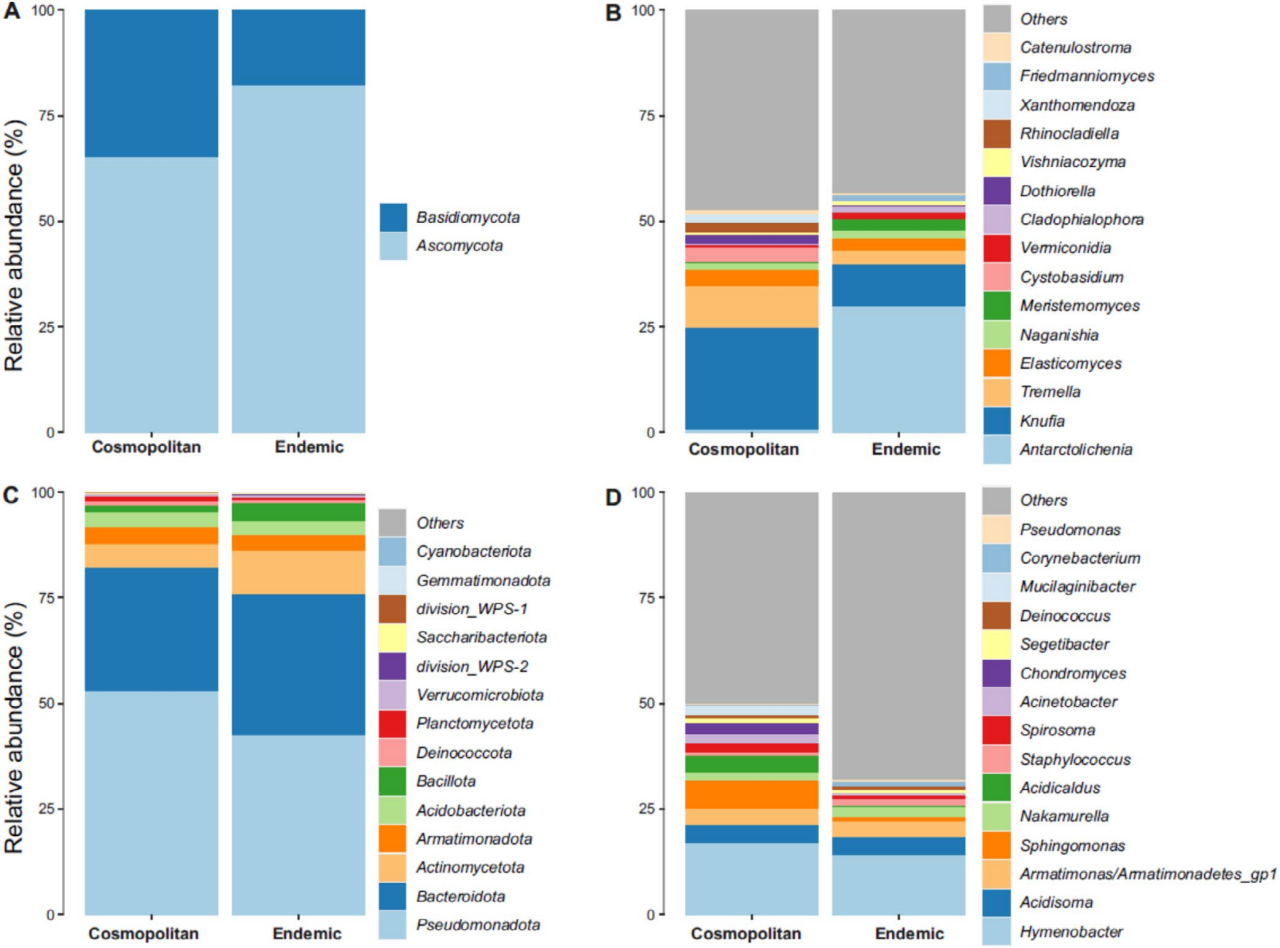
Relative abundance plots according to lichen distribution, that is endemic versus cosmopolitan species: Composition of phyla (A) and genera (C) of fungi; composition of phyla (B) and genera (D) of bacteria. “*Others*” includes all the other genera and higher taxonomic ranks which were not among the 15 most frequently recovered ones.

When the lichen species were grouped according to their endemic and cosmopolitan distribution, no significant differences in the relative abundances of fungal phyla were found. For fungi, in both endemic and cosmopolitan lichens, the phylum *Ascomycota* was the dominant one (82% and 65%, respectively), followed by *Basidiomycota* (18% in endemic and 35% in cosmopolitan) (Figure 4A). The genera *Antarctolichenia*, *Meristemomyces*, and *Vermiconidia* were more abundant in endemic lichens, while *Cystobasidium*, *Rhinocladiella*, *Dothiorella*, and *Knufia* were more abundant in the cosmopolitan lichens (Figure 4C).

Concerning bacteria, the most abundant phyla were *Pseudomonadota* and *Bacteroidota*. Specifically, *Pseudomonadota* was the main represented phylum in both endemic (42%) and cosmopolitan (53%) species. *Bacteroidota* was the second most abundant phylum in cosmopolitan (29%) and in endemic (33%) lichens (Figure 4B). Looking at the genus level, the majority of the fungi are represented by the ascomycetous genera *Antarctolichenia*, *Knufia*, and Elasticomyces, followed by the basidiomycetous yeast *Tremella* and *Naganishia*. Among the bacteria, the most abundant genera were *Hymenobacter* (14%), *Armatimonas* (3.65%), *Acidisoma* (4.1%) and *Nakamurella* (2.65%) in endemic lichens, and *Hymenobacter* (17%), *Sphingomonas* (6.66%), *Acidisoma* (4.12%), *Acidicaldus* (4%) and *Armatimonas* (3.86%) in cosmopolitan lichens (Figure 4D).

### 3.5 Cosmopolitan Versus Endemic Diversity

**FIGURE 5:**
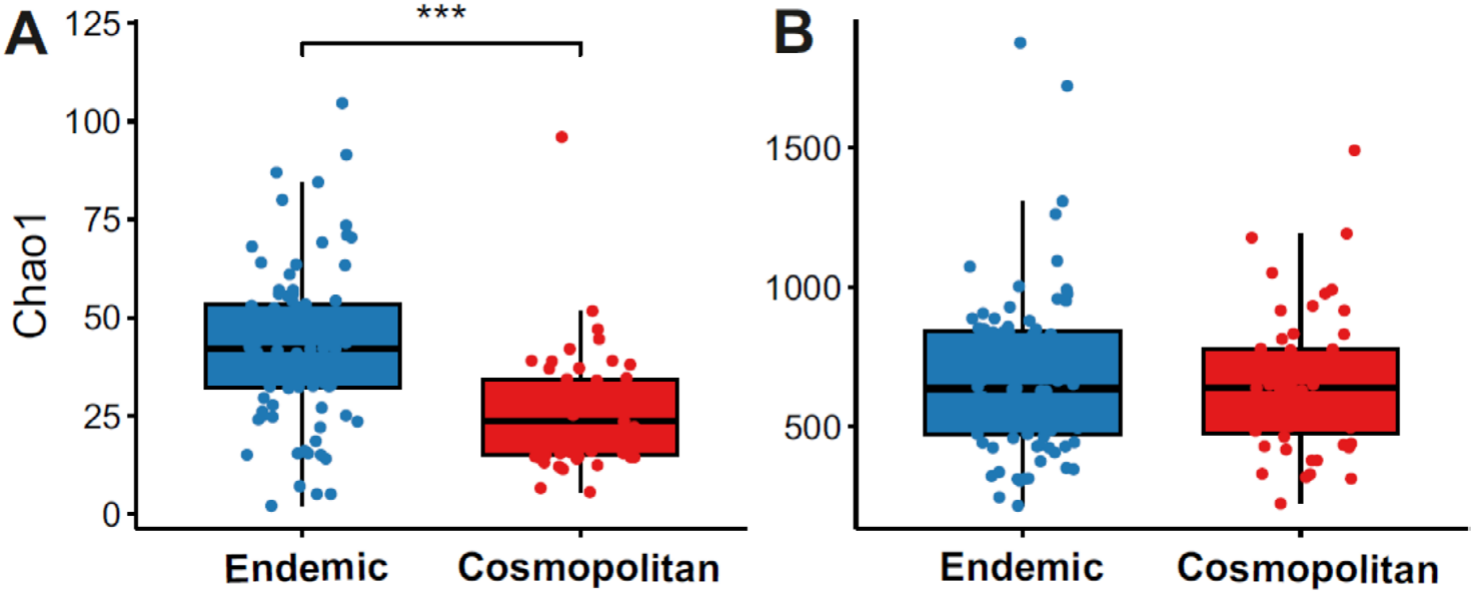
Comparison of alpha diversity using the Chao1 index of fungal (A) and bacterial (B) diversity between endemic and cosmopolitan lichens. The statistical support was tested by the Wilcoxon test and is indicated as ***p < 0.001.

The Chao1 index indicated that the fungal and bacterial diversity values were higher for the endemic than for the cosmopolitan lichens (*p* value < 0.001; Figure 5A). The Wilcoxon test conducted on the analysis did not indicate a significant difference in alpha diversity between the two lichen groups regarding bacterial diversity (Figure 5B).

### 3.6 Species Distribution

**FIGURE 6:**
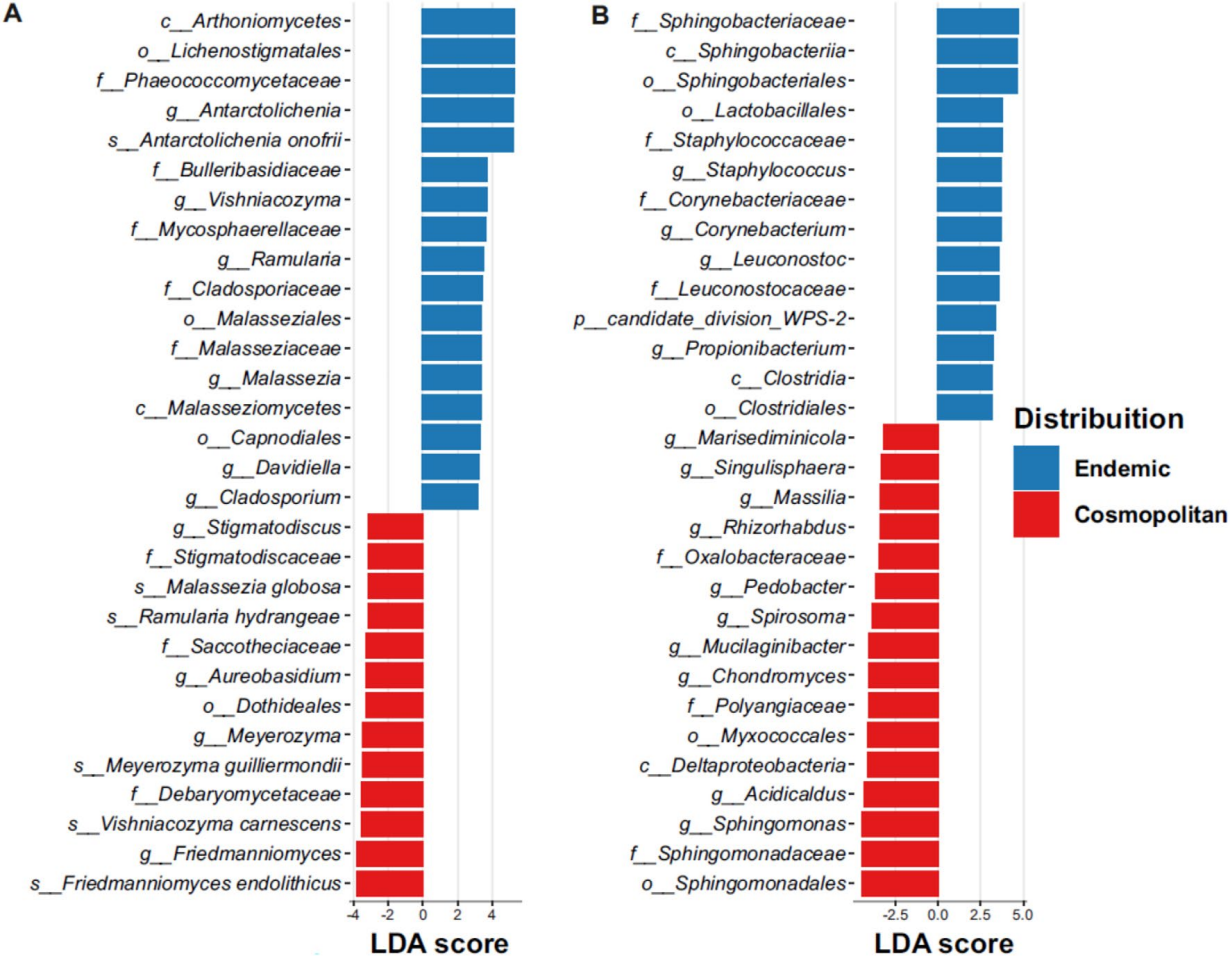
LEfSe analysis between endemic and cosmopolitan lichens relative to the fungal (A) and bacterial diversity (B). The 30 taxa with the highest LDA score are shown.

The effect size of the separation (LefSe) between the endemic and cosmopolitan fungal and bacterial diversity analyses showed taxa with the highest discriminatory power between the two groups of lichen species. The statistically significant results (LDA > 2, *p* value < 0.001) were reported in Table S6. The fungal taxa with the highest segregative values were the members of *Arthoniomycetes, Lichenostigmatales*, and *Phaeococcomycetaceae* for the endemic lichens, whereas the species *Friedmanniomyces endoliticus* was for cosmopolitan lichens (Figure 6A). Regarding the bacterial diversity, the taxa with the highest segregative values were represented by the *Sphingobacteriales* for endemics and the *Sphingomonadales* for cosmopolitan lichens (Figure 6B).

## 4 Discussion

In the present study, we have uncovered the diversity of fungi and bacteria associated with thalli of eight lichen species growing in Victoria Land, which themselves represent peculiar microniches and may act as cradles for the diversification of microbial life (Selbmann et al. 2010, 2013; Arnold et al. 2009; Santiago et al. 2015). While substantial progress has been made in investigating the diversity of fungi and bacteria inhabiting lichen thalli at boreal and arctic latitudes (Cardinale et al. 2006; Liba et al. 2006; Selbmann et al. 2010; Lee et al. 2014; Grube et al. 2009; Hodkinson and Lutzoni 2009; Bates et al. 2011; Spribille et al. 2020; Timling et al. 2014; Klarenberg et al. 2020), so far there have been fewer possibilities to analyse the intrathalline diversity of lichens from Antarctica (Grimm et al. 2021).

### 4.1 The Lichen Mycobiota: Composition and Diversity

In general, the lichen‐associated fungal diversity in the investigated species predominantly comprised *Ascomycota* taxa, and these results are in line with previous studies which have either analysed lichen‐dominated endolithic communities (Selbmann et al. 2005, 2008; Egidi et al. 2014; Coleine et al. 2018a) or fruticose lichens growing on soils with different deglaciation times (Beck et al. 2023), as well as the diversity of moss‐associated fungi in the ice‐free coastal outcrops in Antarctica (Cox et al. 2016; Ji et al. 2016; Yung et al. 2014; Hirose et al. 2016). The prevalence of *Chaetothyriomycetes* and *Dothideomycetes* is another important outcome that supports the frequent occurrence of these groups of fungi in epilithic lichens from dry and cold habitats (Muggia et al. 2016; Muggia and Grube 2018; Cometto et al. 2023, 2024), and as common representatives in lichen‐dominated endolithic communities (Selbmann et al. 2005, 2008; Egidi et al. 2014). *Knufia* (*Chaetothyriales*) is reported here as the most abundant genus present in the cosmopolitan lichens, characterised by its capacity to adapt to different ecologies, such as rock‐inhabiting fungus in extremophilic conditions (Isola et al. 2016) or as a lichen‐associated taxon (Untereiner et al. 2011; Cometto et al. 2023, 2024).

The ASVs analysis at the genus level revealed the presence of a recently described fungal species in the endemic type of distribution, *Antarctolichenia onofrii*, which was originally isolated multiple times from Antarctic rocks as a rock‐dwelling fungus (Muggia et al. 2021). This species is a meristematic melanized fungus and was shown to be a peculiar representative of the Antarctic mycobiota. It also emerges here as part of the lichen mycobiota in Antarctica, as it has never been found elsewhere so far (i.e., from rock or lichen thalli form other geographic origins). *A. onofrii*, albeit clearly free‐living, still seems to maintain a loose relation to and interdependence with microalgae, as it was isolated together with microalgae and it is not possible to propagate it in axenic culture for a long time (Muggia et al. 2021). I ts constant occurrence in nature with lichens (both epilithic and endolithic) supports the hypothesis for a possible link between a free‐living (rock associated) and symbiotic lifestyle.

Our findings, however, also unveil a significant presence of *Basidiomycota* in all examined thalli, even though their abundances were, in general, much lower than *Ascomycota*. Notably, only in the case of the lichen *P. chlorophanum* was a higher abundance of *Basidiomycota* detected. *Basidiomycota* were found to be particularly abundant in the Antarctic region only in two studies conducted in the waters of the Antarctic Peninsula (Garmendia et al. 2021) and in snow‐ice near the Italian–French station Concordia, respectively (Stoppiello et al. 2023). In lichens from other regions of the world, *Basidiomycota* have also been shown to be quite ubiquitous but in much lower amounts than *Ascomycota*, and they rarely showed specificity for the host lichen (Smith et al. 2020; Lendemer et al. 2019; Cometto et al. 2022). The three most abundant basidiomycetous yeast genera were recovered in both the Antarctic endemic and the cosmopolitan lichens, that is, *Tremella*, *Cystobasidium*, and *Naganishia*. These three genera are known to be extremophilic yeasts (Buzzini et al. 2018), which are even able to develop pathogenicity toward humans (Rush et al. 2023; Nizovoy et al. 2021). Environmental strains of *Tremella*, *Cystobasidium*, and *Naganishia* were also found in the two cosmopolitan lichens *T. atra* and *R. melanophthalma* collected across the other six continents (Cometto et al. 2022, 2024); however, only *R. melanophthalma* was included in the present study, as it was found in the sampling localities. The genus *Tremella* emerges here because it is a well‐known lichen parasite (Millanes et al. 2011), which has been studied for its phenotypic traits on many host lichen thalli (Millanes et al. 2012, 2014, 2015). Here, we present an additional case of its occurrence as cryptic species within the thalli (Tuovinen et al. 2021), as no symptoms of its infection could be found on any of the analysed lichen specimens. Cometto et al. (2022, 2024) already reported *Tremella* as cryptically present in lichens and succeeded in isolating it in culture (Cometto et al. 2022), as we did for samples included in the dataset presented here (De Carolis et al. in preparation). The high abundance of basidiomycetous yeasts compared to ascomycetous yeasts in Antarctic lichens could be due to the different organisation of the yeast cells. Indeed, the thicker cell wall and presence of capsules in basidiomycetous yeasts may represent an adaptive advantage in the Antarctic environment (Garmendia et al. 2021; Stoppiello et al. 2023), as well as their tendency to reside in the lichen thalli as resting cells/spores.

Eventually, species diversity analysis performed between the mycobiota of endemic and cosmopolitan lichens revealed that a few fungal genera that are endemic to Antarctica are also elements of only the endemic lichen mycobiota. One example is *Friedmanniomyces*, which has been frequently reported as a component of endolithic antarctic communities (Onofri et al. 1999).

### 4.2 The Lichen‐Associated Bacterial Communities: Composition and Diversity

*Pseudomonadota*, *Bacteroidota*, and *Actinomycetota* were recovered as the most abundant bacterial phyla. *Pseudomonadota* is in general widely distributed in different habitats on Earth (Nemergut et al. 2011) and in bioaerosols in the atmosphere (Fang et al. 2020; Tang et al. 2018), while *Bacteroidota* were also already found as predominant phyla among bacteria living in Antarctic lakes (Michaud et al. 2012). According to previous studies, it was found that heterotrophic *Pseudomonadota* and *Bacteroidota* coexist with Antarctic cyanobacteria (Cornet et al. 2018) in glaciers and cold deserts in Antarctica, for example in the King George and James Ross Islands, in the Arctic (Alaska and Greenland) and in Asia (Himalaya Mountains, Pamir, Then‐Shan and Qilian; Segawa et al. 2017). *Actinomycetota* were isolated from Antarctic soils (Silva et al. 2020; Araujo et al. 2020).

*Hymenobacter, Acidisoma, Armatimona/Armatimonadetes*, and *Sphingomonas* were among the most present genera identified in this study, and they are also the most frequently isolated genera from East Antarctica (Lambrechts et al. 2019; Peeters et al. 2011; Benaud et al. 2022; Pudasaini et al. 2017). These bacteria are all presented as chemoheterotrophic, strictly aerobic, and UV‐resistant. These findings are consistent with their ability to survive the harsh environmental conditions of the region. Members belonging to *Acidisoma* are among the most abundant, as also evidenced by previous studies of endolithic communities (Coleine et al. 2019). This genus has rarely been observed in the ice‐free areas of Victoria Land but is common in cold regions such as the Alps (Nakai et al. 2013), suggesting that it may have evolved unique adaptations for cold resistance. *Acidisoma* species are acidophilic and psychrotolerant heterotrophic bacteria. They can use a wide range of organic substrates, particularly carbohydrates and amino acids (Belova et al. 2009). The predominant genus analysed was *Hymenobacter*, which is found in several Arctic coastal glaciers. Glaciers, indeed, have been identified as a possible vehicle for bacterial transport to downstream marine waters. Several taxa belonging to this group have been retrieved in the McMurdo Dry Valleys in Antarctica (Hirsch et al. 1998). In addition, it has also been identified in coastal ice samples in southern Shetland (Garcia‐Lopez et al. 2021). These locations are all oligotrophic, which could suggest that the genus *Hymenobacter* owns the genetic setup to grow using a limited number of carbon sources, such as some sugars, sugar alcohols, organic acids, and some amino acids (Marizcurrena et al. 2019). On the other hand, the chemoheterotrophic genus *Armatimonas* has not been reported in Antarctic areas but has been found in alpine glaciers (Tolotti et al. 2020). These bacteria are members of the phylum *Armatimonadetes*, which is still poorly studied. Many of the *Armatimonas* bacteria have a wide range of enzymes that can be activated at different temperatures, allowing them to use the available resources (Tamaki et al. 2011).

A taxonomic peculiarity concerns the phylum *Planctomycetota*, which includes bacterial species known for their ability to adapt to extreme environmental conditions, such as low temperatures, high UV radiation, and freeze–thaw cycles, which are common in Antarctic environments (Jiya et al. 2024; Bradley et al. 2023). During this study, they were found to be associated with *L. fuscobrunnea* thalli. The survival capabilities of the *Planctomycetota bacteria* under extreme conditions make this phylum an interesting subject of study for astrobiological research.

### 4.3 Lichen Symbioses Select Their Own Fungal and Bacterial Diversity

The low Chao1 values for the mycobiota approached those found in the mycobiota of two lichen species collected worldwide in extreme, high altitudinal environments ( *R. melanophthalma* and *T. atra* ) by Cometto et al. (2024). On the other hand, Chao1 values were relatively high for the bacteria and showed extremely significant differences in the composition of the communities (e.g., Hodkinson et al. 2012; Bates et al. 2011; Cardinale et al. 2006) suggesting that a considerable proportion of the lichen‐associated bacteria are specific to the lichen species. Indeed, the statistically significant differences that we found between the lichen fungal and bacterial diversity among the analysed species suggest that the lichen itself influences most of the diversity associated with the thalli. This is supported further by the PCoA analysis concerning beta‐diversity, in which the lichen species mostly explained the community variation. Instead, the ‘site’ factor contributed less to the overall variation in the fungal and bacterial community, contrasting the results reported previously by Coleine et al. (2019). This observation may be explained by the fact that in the lichen symbioses, the mycobionts (more than the photobionts) shape, also in unique ways, the phenotype of the lichen thallus itself, with their morpho‐anatomical structures and the production of secondary metabolites. However, our analyses showed that the investigated lichen species, although differing only slightly in their external phenotypes, most of them being crustose epilithic (with the exception of the fruticose *R. melanophthalma* , and the partially foliose *R. elegans* ), and either Antarctic endemics or cosmopolitans, shared only part of their fungal and bacterial diversity. Lichens, thus, seem to act as environmental filters for microorganisms in general (Hodkinson et al. 2012; Cardinale et al. 2006; Almendras et al. 2018) but also in Antarctica. Interestingly, the influence of the lichen on the whole microbial diversity does not emerge when only different populations of a single or a few lichen species are compared (Cometto et al. 2024), or when only a few specimens would be investigated for a certain species. It seems, instead, that when a wide spectrum of species is collected in different localities, though under the same (or highly similar) environmental conditions, the lichen identity, together with the key ecological traits of its thallus, lets emerge the intrathalline fungal and bacterial diversity.

This study clearly highlighted that some fungal and bacterial taxa are more predominant in cosmopolitan or endemic lichen species. There are only a few taxa, for example, Antarctolichenia or Sphingobacteriales, for which we could assume that their stronger association with endemic lichens would relate to the physical and/or biotic barriers that limit the dispersal of the endemic lichen species outside the continent. This would promote the independent evolution of isolated populations of the lichens themselves and their associated fungi and bacteria (Olden et al. 2004; Armstrong 2017). Regarding cosmopolitan lichens, it is observed that the species *Friedmanniomyces endolithicus* is the most associated with this type of distribution. *F. endolithicus* is exclusively associated with endolithic microbial communities in the ice‐free areas of Victoria Land, including the McMurdo Dry Valleys, which are characterised by high UV irradiation, low temperatures, and strict oligotrophy. Among the black meristematic fungi in these communities, *F. endolithicus* is the most widespread and frequently isolated, further supporting its high degree of adaptation to the prohibitive environmental conditions of this area (Selbmann et al. 2015). It may be speculated that also inside the lichen thallus of the cosmopolitan lichens, *F. endolithicus* helps the symbionts in resisting UV irradiation as it does for endolithic communities. Prolonged isolation could be a structuring factor for the biogeography of microorganisms in Antarctica, and this could explain why only a few certain genera are detected as more abundant in endemic than in cosmopolitan lichens.

## 5 Conclusions

This study reports in detail the comparison of fungal and bacterial diversity associated with lichen thalli in Antarctica, by considering either Antarctic endemic and cosmopolitan species. We found a higher biodiversity associated with endemic than with cosmopolitan lichen species, representing also some endemic microbial taxa. This could be due to physical or biotic barriers that limit the dispersal of species and thus promote the independent evolution of isolated microbial assemblages. Observing the data, we found two distinct groups within the endemic lichens: one characterised by low diversity and another by high diversity. This variation in diversity among endemic lichens could be attributed to their specific adaptations to the unique microhabitats and environmental conditions present in Antarctic ecosystems. Endemic lichens may have evolved distinct ecological strategies, leading to a greater variability in their microbial communities. In contrast, among cosmopolitan lichens, there is less variability in microbial diversity. This consistent pattern suggests that cosmopolitan lichens might possess more generalised microbial communities, possibly due to their ability to thrive in a wider range of environmental conditions and their broader geographical distribution. The association between fungi and bacteria provides a robust understanding of the intricate associations between these two microbial communities within the lichen thalli. The significant correlation underscores the potential interdependence and co‐evolution of bacterial and fungal communities in these unique Antarctic ecosystems.

## Author Contributions

**Lucia Muggia:** conceptualization, methodology, investigation, funding acquisition, project administration, writing – review and editing, writing – original draft, resources, formal analysis, supervision. **Gerardo A. Stoppiello:** methodology, data curation, investigation, visualization, writing – original draft, formal analysis. **Roberto De Carolis:** methodology, data curation, visualization, investigation, writing – original draft, formal analysis. **Claudia Coleine:** conceptualization, methodology, data curation, writing – original draft, formal analysis. **Mauro Tretiach:** conceptualization, supervision, writing – review and editing, resources. **Laura Selbmann:** conceptualization, methodology, investigation, funding acquisition, writing – original draft, writing – review and editing, project administration, resources, formal analysis, supervision.

## Conflicts of Interest

The authors declare no conflicts of interest.

## Supporting information


**Figure S1.** Species accumulation curves of fungal (A) and bacterial (B) dataset.

**Figure S2.** Species accumulation curves performed for each lichen species on the fungal (A) and the bacterial (B) datasets.

**Figure S3.** Linear regression analysis between the Shannon indices of the bacterial and fungal communities.

**Table S1.** Lichen species genotypes removed from the final dataset.

**Table S2.** Alpha and beta diversity for the fungal dataset: (A) Alpha diversity indexes for type of distribuition, alpha diversity indexes comparision for type of distribuition, Alpha diversity indexes for type of mycobiont, Alpha diversity indexes comparision for type of mycobiont. (B) Beta diversity for mycobiont (Bray‐Curtis), Beta diversity mycobiont comparision (Bray‐curtis), Beta diversity for mycobiont (Jaccard), Beta diversity mycobiont comparision (Jaccard), Beta diversity for type of distribuition.

**Table S3.** Alpha and beta diversity for the bacterial dataset: (A) Alpha diversity indexes and multiple comparision; (B) Beta diversity scores for Bray‐Curtis and Jaccard matrixes.

**Table S4.** (A) Alpha diversity and alpha diversity indexes comparision of not rarefied data; (B) alpha diversity and alpha diversity indexes comparision of rarefied data.

**Table S5.** Linear regression analyses indexes.

**Table S6.** Linear discriminant Effect Size (LEfSe) analysis: (A) LEfSe score for type of distribuition of Fungi (LDA > 2); (B) LEfSe score for type of distribuition of Bacteria (LDA > 2).

**Table S7.** Original table of abundance and taxonomy of the sequenced fungal ASVs.

**Table S8.** Original table of abundance and taxonomy of the sequenced bacterial ASVs.

**Table S9.** Sequencing depth calculated for each sample and the ecological groups (endemics and cosmopolitan) for both fungi and bacteria.

**Supporting Information 1.** List of the sequenced fungal ASVs and their nucleotide sequence.

**Supporting Information 2.** List of the sequenced bacterial ASVs and their nucleotide sequence.

## Data Availability

The data have been deposited in NCBI under the bioproject accession number PRJNA1042949. It is also available as Supporting Informations 1 and 2.

## References

[emi470080-bib-0001] Abarenkov, K. , A. Zirk , T. Piirmann , et al. 2020. “The UNITE Database for Molecular Identification of Fungi–Recent Updates and Future Perspectives.” New Phytologist 186, no. 2: 281. 10.1111/j.1469-8137.2009.03160.x. 20409185

[emi470080-bib-0002] Albanese, D. , C. Coleine , O. Rota‐Stabelli , et al. 2021. “Pre‐Cambrian Roots of Novel Antarctic Cryptoendolithic Bacterial Lineages.” Microbiome 9: 1–15. 33741058 10.1186/s40168-021-01021-0 PMC7980648

[emi470080-bib-0003] Almendras, K. , J. García , M. Carú , and J. Orlando . 2018. “Nitrogen‐Fixing Bacteria Associated With *Peltigera* Cyanolichens and *Cladonia* Chlorolichens.” Molecules 23: 3077. 30477264 10.3390/molecules23123077 PMC6320784

[emi470080-bib-0004] Araujo, R. , V. V. Gupta , F. Reith , A. Bissett , P. Mele , and C. M. Franco . 2020. “Biogeography and Emerging Significance of Actinobacteria in Australia and Northern Antarctica Soils.” Soil Biology and Biochemistry 146: 107805.

[emi470080-bib-0005] Archer, S. D. , A. De los Ríos , K. C. Lee , et al. 2017. “Endolithic Microbial Diversity in Sandstone and Granite From the McMurdo Dry Valleys, Antarctica.” Polar Biology 40: 997–1006.

[emi470080-bib-0006] Armstrong, R. A. 2017. “Adaptation of Lichens to Extreme Conditions.” In Plant Adaptation Strategies in Changing Environment, edited by V. Shukla , S. Kumar , and N. Kumar . Springer.

[emi470080-bib-0007] Arnold, A. E. , J. Miadlikowska , K. L. Higgins , et al. 2009. “A Phylogenetic Estimation of Trophic Transition Networks for Ascomycetous Fungi: Are Lichens Cradles of Symbiotrophic Fungal Diversification?. Systematic Biology.” 58: 283–297. 10.1093/sysbio/syp001 20525584

[emi470080-bib-0009] Bates, S. T. , G. W. Cropsey , J. G. Caporaso , R. Knight , and N. Fierer . 2011. “Bacterial Communities Associated With the Lichen Symbiosis.” Applied and Environmental Microbiology 77: 1309–1314. 21169444 10.1128/AEM.02257-10 PMC3067232

[emi470080-bib-0010] Beck, A. , A. Casanova‐Katny , and J. Gerasimova . 2023. “Metabarcoding of Antarctic Lichens From Areas With Different Deglaciation Times Reveals a High Diversity of Lichen‐Associated Communities.” Genes 14: 1019. 37239380 10.3390/genes14051019 PMC10218603

[emi470080-bib-0011] Belova, S. E. , T. A. Pankratov , E. N. Detkova , E. N. Kaparullina , and S. N. Dedysh . 2009. “ *Acidisoma tundrae* Gen. Nov., Sp. Nov. and *Acidisoma sibiricum* Sp. Nov., Two Acidophilic, Psychrotolerant Members of the Alphaproteobacteria From Acidic Northern Wetlands.” International Journal of Systematic and Evolutionary Microbiology 59: 2283–2290. 19620354 10.1099/ijs.0.009209-0

[emi470080-bib-0012] Benaud, N. , D. S. Chelliah , S. Y. Wong , and B. C. Ferrari . 2022. “Soil Substrate Culturing Approaches Recover Diverse Members of Actinomycetota From Desert Soils of Herring Island, East Antarctica.” Extremophiles 26: 24. 35829965 10.1007/s00792-022-01271-2 PMC9279279

[emi470080-bib-0013] Bradley, J. A. , C. B. Trivedi , M. Winkel , et al. 2023. “Active and Dormant Microorganisms on Glacier Surfaces.” Geobiology 21, no. 2: 244–261. 36450703 10.1111/gbi.12535 PMC10099831

[emi470080-bib-0014] Buzzini, P. , B. Turchetti , and A. Yurkov . 2018. “Extremophilic Yeasts: The Toughest Yeasts Around?” Yeast 35: 487–497. 29577430 10.1002/yea.3314

[emi470080-bib-0015] Caporaso, J. G. , C. L. Lauber , W. A. Walters , et al. 2012. “Ultra‐High‐Throughput Microbial Community Analysis on the Illumina HiSeq and MiSeq Platforms.” ISME Journal 6: 1621–1624. 22402401 10.1038/ismej.2012.8 PMC3400413

[emi470080-bib-0016] Cardinale, M. , A. M. Puglia , and M. Grube . 2006. “Molecular Analysis of Lichen‐Associated Bacterial Communities.” FEMS Microbiology Ecology 57: 484–495. 16907761 10.1111/j.1574-6941.2006.00133.x

[emi470080-bib-0017] Castello, M. , and P. L. Nimis . 1997. “Diversity of Lichens in Antarctica.” In Antarctic Communities Species, Structure and Survival, edited by B. Battaglia , J. Valencia , and D. W. H. Walton , 15–21. Cambridge University Press.

[emi470080-bib-0018] Castello, M. , and P. L. Nimis . 2000. “A Key to the Lichens of Terra Nova Bay (Victoria Land, Continental Antarctica).” Italian Journal of Zoology 67: 175–184.

[emi470080-bib-0019] Cole, J. R. , Q. Wang , J. A. Fish , et al. 2014. “Ribosomal Database Project: Data and Tools for High Throughput rRNA Analysis.” Nucleic Acids Research 42: D633–D642. 24288368 10.1093/nar/gkt1244 PMC3965039

[emi470080-bib-0021] Coleine, C. , F. Gevi , G. Fanelli , S. Onofri , A. M. Timperio , and L. Selbmann . 2020a. “Specific Adaptations Are Selected in Opposite Sun Exposed Antarctic Cryptoendolithic Communities as Revealed by Untargeted Metabolomics.” PLoS One 15: e0233805. 32460306 10.1371/journal.pone.0233805 PMC7253227

[emi470080-bib-0022] Coleine, C. , S. Masonjones , K. Sterflinger , S. Onofri , L. Selbmann , and J. E. Stajich . 2020b. “Peculiar Genomic Traits in the Stress‐Adapted Cryptoendolithic Antarctic Fungus Friedmanniomyces Endolithicus.” Fungal Biology 124, no. 5: 458–467. 32389308 10.1016/j.funbio.2020.01.005

[emi470080-bib-0023] Coleine, C. , J. E. Stajich , N. Pombubpa , et al. 2019. “Altitude and Fungal Diversity Influence the Structure of Antarctic Cryptoendolithic Bacteria Communities.” Environmental Microbiology Reports 11: 718–726. 31393667 10.1111/1758-2229.12788 PMC8057506

[emi470080-bib-0024] Coleine, C. , J. E. Stajich , A. de Los Ríos , and L. Selbmann . 2021. “Beyond the Extremes: Rocks as Ultimate Refuge for Fungi in Drylands.” Mycologia 113: 108–133. 33232202 10.1080/00275514.2020.1816761

[emi470080-bib-0025] Coleine, C. , J. E. Stajich , L. Zucconi , et al. 2018a. “Antarctic Cryptoendolithic Fungal Communities Are Highly Adapted and Dominated by Lecanoromycetes and Dothideomycetes.” Frontiers in Microbiology 9: 1392. 30008702 10.3389/fmicb.2018.01392 PMC6033990

[emi470080-bib-0026] Coleine, C. , L. Zucconi , S. Onofri , N. Pombubpa , J. E. Stajich , and L. Selbmann . 2018b. “Sun Exposure Shapes Functional Grouping of Fungi in Cryptoendolithic Antarctic Communities.” Lifestyles 8: 19. 10.3390/life8020019 PMC6027399 29865244

[emi470080-bib-0028] Cometto, A. , C. G. Ametrano , S. D. Leavitt , M. Grube , A. Pallavicini , and L. Muggia . 2024. “Highly Heterogeneous Mycobiota Shape Fungal Diversity in Two Globally. Distributed Lichens.” Fungal Ecology 69: 101331.

[emi470080-bib-0029] Cometto, A. , S. D. Leavitt , M. Grube , S. De Hoog , and L. Muggia . 2023. “Tackling Fungal Diversity in Lichen Symbioses: Molecular and Morphological Data Recognize New Lineages in Chaetothyriales (Eurotiomycetes, Ascomycota).” Mycological Progress 22: 53.

[emi470080-bib-0030] Cometto, A. , S. D. Leavitt , A. M. Millanes , M. Wedin , M. Grube , and L. Muggia . 2022. “The Yeast Lichenosphere: High Diversity of Basidiomycetes From the Lichens *Tephromela Atra* and *Rhizoplaca melanophthalma* .” Fungal Biology 126: 587–608. 36008051 10.1016/j.funbio.2022.07.004

[emi470080-bib-0031] Cornet, L. , A. R. Bertrand , M. Hanikenne , E. J. Javaux , A. Wilmotte , and D. Baurain . 2018. “Metagenomic Assembly of New (Sub) Polar Cyanobacteria and Their Associated Microbiome From Non‐Axenic Cultures.” Microbial Genomics 4: e000212. 30136922 10.1099/mgen.0.000212 PMC6202449

[emi470080-bib-0032] Cox, F. , K. K. Newsham , R. Bol , J. A. Dungait , and C. H. Robinson . 2016. “Not Poles Apart: Antarctic Soil Fungal Communities Show Similarities to Those of the Distant Arctic.” Ecology Letters 19: 528–536. 26932261 10.1111/ele.12587

[emi470080-bib-0033] Cubero, O. F. , A. N. A. Crespo , J. Fatehi , and P. D. Bridge . 1999. “DNA Extraction and PCR Amplification Method Suitable for Fresh, Herbarium‐Stored, Lichenized, and Other Fungi.” Plant Systematics and Evolution 216: 243–249.

[emi470080-bib-0035] Davis, N. M. , D. M. Proctor , S. P. Holmes , D. A. Relman , and B. J. Callahan . 2018. “Simple Statistical Identification and Removal of Contaminant Sequences in Marker‐Gene and Metagenomics Data.” Microbiome 6: 1–14. 30558668 10.1186/s40168-018-0605-2 PMC6298009

[emi470080-bib-0036] de la Torre, J. R. , B. M. Goebel , E. I. Friedmann , and N. R. Pace . 2003. “Microbial Diversity of Cryptoendolithic Communities From the McMurdo Dry Valleys, Antarctica.” Applied and Environmental Microbiology 69: 3858–3867. 12839754 10.1128/AEM.69.7.3858-3867.2003 PMC165166

[emi470080-bib-0037] Edgar, R. C. 2010. “Search and Clustering Orders of Magnitude Faster Than BLAST.” Bioinformatics 26: 2460–2461. 20709691 10.1093/bioinformatics/btq461

[emi470080-bib-0038] Egidi, E. , G. S. De Hoog , D. Isola , et al. 2014. “Phylogeny and Taxonomy of Meristematic Rock‐Inhabiting Black Fungi in the Dothideomycetes Based on Multi‐Locus Phylogenies.” Fungal Diversity 65: 127–165.

[emi470080-bib-0039] Ettinger, C. L. , M. Saunders , L. Selbmann , et al. 2023. “Highly Diverse and Unknown Viruses May Enhance Antarctic Endoliths' Adaptability.” Microbiome 11: 1–8. 37158954 10.1186/s40168-023-01554-6 PMC10165816

[emi470080-bib-0040] Fang, J. , Q. Dong , W. Shen , et al. 2020. “Variation in Near‐Surface Airborne Bacterial Communities Among Five Forest Types.” Forests 11: 561.

[emi470080-bib-0041] Fernández‐Mendoza, F. , A. Fleischhacker , T. Kopun , M. Grube , and L. Muggia . 2017. “ITS 1 Metabarcoding Highlights Low Specificity of Lichen Mycobiomes at a Local Scale.” Molecular Ecology 26: 4811–4830. 28771869 10.1111/mec.14244

[emi470080-bib-0042] Garcia‐Lopez, E. , A. Moreno , M. Bartolomé , M. Leunda , C. Sancho , and C. Cid . 2021. “Glacial Ice Age Shapes Microbiome Composition in a Receding Southern European Glacier.” Frontiers in Microbiology 12: 714537. 34867842 10.3389/fmicb.2021.714537 PMC8636055

[emi470080-bib-0043] Garmendia, G. , A. Alvarez , R. Villarreal , A. Martínez‐Silveira , M. Wisniewski , and S. Vero . 2021. “Fungal Diversity in the Coastal Waters of King George Island (Maritime Antarctica).” World Journal of Microbiology and Biotechnology 37: 1–12. 10.1007/s11274-021-03112-4 34322842

[emi470080-bib-0130] Greco, S. , E. D’Agostino , C. Manfrin , A. S. Gaetano , G. Furlanis , and F. Capanni . 2021. “RNA‐Sequencing Indicates High Hemocyanin Expression as a Key Strategy for Cold Adaptation in the Antarctic Amphipod Eusirus cf. Giganteus Clade g3.” Biocell 46: 1611–1619.

[emi470080-bib-0045] Grimm, M. , M. Grube , U. Schiefelbein , D. Zühlke , J. Bernhardt , and K. Riedel . 2021. “The Lichens' Microbiota, Still a Mystery?” Frontiers in Microbiology 12: 714. 10.3389/fmicb.2021.623839 PMC8042158 33859626

[emi470080-bib-0046] Grube, M. , and G. Berg . 2009. “Microbial Consortia of Bacteria and Fungi With Focus on the Lichen Symbiosis.” Fungal Biology Reviews 23: 72–85.

[emi470080-bib-0047] Grube, M. , M. Cardinale , J. V. de Castro , H. Müller , and G. Berg . 2009. “Species‐Specific Structural and Functional Diversity of Bacterial Communities in Lichen Symbioses.” ISME Journal 3: 1105–1115. 19554038 10.1038/ismej.2009.63

[emi470080-bib-0048] Grube, M. , T. Cernava , J. Soh , et al. 2015. “Exploring Functional Contexts of Symbiotic Sustain Within Lichen‐Associated Bacteria by Comparative Omics.” ISME Journal 9: 412–424. 25072413 10.1038/ismej.2014.138 PMC4303634

[emi470080-bib-0049] Harutyunyan, S. , L. Muggia , and M. Grube . 2008. “Black Fungi in Lichens From Seasonally Arid Habitats.” Studies in Mycology 61: 83–90. 19287530 10.3114/sim.2008.61.08 PMC2610299

[emi470080-bib-0050] Hawksworth, D. L. , and M. Grube . 2020. “Lichens Redefined as Complex Ecosystems.” New Phytologist 227: 1281–1283. 32484275 10.1111/nph.16630 PMC7497170

[emi470080-bib-0051] Hawksworth, D. , and R. Honegger . 1994. “The Lichen Thallus: A Symbiotic Phenotype of Nutritionally Specialized Fungi and Its Response to Gall Producers.” In Plant Galls: Organisms, Interactions, Populations, edited by M. A. J. Williams , 77–98. Clarendon Press.

[emi470080-bib-0052] Hirose, D. , S. Hobara , S. Matsuoka , et al. 2016. “Diversity and Community Assembly of Moss‐Associated Fungi in Ice‐Free Coastal Outcrops of Continental Antarctica.” Fungal Ecology 24: 94–101.

[emi470080-bib-0053] Hirsch, P. , W. Ludwig , C. Hethke , M. Sittig , B. Hoffmann , and C. A. Gallikowski . 1998. “ *Hymenobacter roseosalivarius* Gen. Nov., Sp. Nov. From Continental Antarctic Soils and Sandstone: Bacteria of the *Cytophaga/Flavobacterium/Bacteroides* Line of Phylogenetic Descent.” Systematic and Applied Microbiology 21: 374–383. 9841127 10.1016/s0723-2020(98)80047-7

[emi470080-bib-0054] Hodkinson, B. P. , N. R. Gottel , C. W. Schadt , and F. Lutzoni . 2012. “Photoautotrophic Symbiont and Geography Are Major Factors Affecting Highly Structured and Diverse Bacterial Communities in the Lichen Microbiome.” Environmental Microbiology 14: 147–161. 21906220 10.1111/j.1462-2920.2011.02560.x

[emi470080-bib-0055] Hodkinson, B. P. , and F. Lutzoni . 2009. “A Microbiotic Survey of Lichen‐Associated Bacteria Reveals a New Lineage From the Rhizobiales.” Symbiosis 49: 163–180.

[emi470080-bib-0056] Honegger, R. 2012. “15 the Symbiotic Phenotype of Lichen‐Forming Ascomycetes and Their Endo‐ and Epibionts.” In Fungal Associations, edited by B. Hock , vol. 9, 287–339. Springer.

[emi470080-bib-0129] Isola, D. , L. Zucconi , S. Onofri , G. Caneva , G. S. de Hoog , and L. Selbmannal . 2016. “Extremotolerant Rock Inhabiting Black Fungi from Italian Monumental Sites.” Fungal Diversity 76: 75–96.

[emi470080-bib-0057] Ji, M. , J. van Dorst , A. Bissett , et al. 2016. “Microbial Diversity at Mitchell Peninsula, Eastern Antarctica: A Potential Biodiversity “Hotspot”.” Polar Biology 39: 237–249.

[emi470080-bib-0059] Ji, Y. , L. Ashton , S. M. Pedley , et al. 2013. “Reliable, Verifiable and Efficient Monitoring of Biodiversity via Metabarcoding.” Ecology Letters 16: 1245–1257. 23910579 10.1111/ele.12162

[emi470080-bib-0060] Jiya, N. , R. Ghosh , P. Shede , and A. Sharma . 2024. “Comparative Analysis of Bacterial Diversity in Accumulated Snow and Exposed Sediments Across Antarctic Islands.” Brazilian Journal of Microbiology 55: 2355–2362. 38748395 10.1007/s42770-024-01360-8 PMC11405587

[emi470080-bib-0061] Klarenberg, I. , C. Keuschnig , D. Warshan , I. Jónsdóttir , and O. Vilhelmsson . 2020. “The Total and Active Bacterial Community of the Chlorolichen *Cetraria Islandica* and Its Response to Long‐Term Warming in Sub‐Arctic Tundra.” Frontiers in Microbiology 11: 540404. 33391192 10.3389/fmicb.2020.540404 PMC7775390

[emi470080-bib-0062] Kruskal, W. H. , and W. A. Wallis . 1952. “Use of Ranks in One‐Criterion Variance Analysis.” Journal of the American Statistical Association 47: 583–621.

[emi470080-bib-0063] Lambrechts, S. , A. Willems , and G. Tahon . 2019. “Uncovering the Uncultivated Majority in Antarctic Soils: Toward a Synergistic Approach.” Frontiers in Microbiology 10: 242. 30828325 10.3389/fmicb.2019.00242 PMC6385771

[emi470080-bib-0064] Lee, Y. M. , E. H. Kim , H. K. Lee , and S. G. Hong . 2014. “Biodiversity and Physiological Characteristics of Antarctic and Arctic Lichens‐Associated Bacteria.” World Journal of Microbiology and Biotechnology 30: 2711–2721. 25001073 10.1007/s11274-014-1695-z

[emi470080-bib-0065] Lendemer, J. C. , K. G. Keepers , E. A. Tripp , C. S. Pogoda , C. M. McCain , and N. C. Kane . 2019. “A Taxonomically Broad Metagenomic Survey of 339 Species Spanning 57 Families Suggests Cystobasidiomycete Yeasts Are Not Ubiquitous Across all Lichens.” American Journal of Botany 106: 1090–1095. 31397894 10.1002/ajb2.1339

[emi470080-bib-0066] Liba, C. M. , F. I. D. S. Ferrara , G. P. Manfio , et al. 2006. “Nitrogen‐Fixing Chemo‐Organotrophic Bacteria Isolated From Cyanobacteria‐Deprived Lichens and Their Ability to Solubilize Phosphate and to Release Amino Acids and Phytohormones.” Journal of Applied Microbiology 101: 1076–1086. 17040231 10.1111/j.1365-2672.2006.03010.x

[emi470080-bib-0067] Lindahl, B. D. , R. H. Nilsson , L. Tedersoo , et al. 2013. “Fungal Community Analysis by High‐Throughput Sequencing of Amplified Markers–a User's Guide.” New Phytologist 199, no. 1: 288–299. 23534863 10.1111/nph.12243 PMC3712477

[emi470080-bib-0068] Liu, C. , Y. Cui , X. Li , and M. Yao . 2021. “Microeco: An R Package for Data Mining in Microbial Community Ecology.” FEMS Microbiology Ecology 97: fiaa255. 33332530 10.1093/femsec/fiaa255

[emi470080-bib-0069] Lutzoni, F. , M. Pagel , and V. Reeb . 2001. “Major Fungal Lineages Are Derived From Lichen Symbiotic Ancestors.” Nature 411: 937–940. 11418855 10.1038/35082053

[emi470080-bib-0070] Marizcurrena, J. J. , L. M. Herrera , A. Costabile , et al. 2019. “Validating Biochemical Features at the Genome Level in the Antarctic Bacterium *Hymenobacter* Sp. Strain UV11.” FEMS Microbiology Letters 366: fnz177. 31397847 10.1093/femsle/fnz177

[emi470080-bib-0071] McMurdie, P. J. , and S. Holmes . 2013. “Phyloseq: An R Package for Reproducible Interactive Analysis and Graphics of Microbiome Census Data.” PLoS One 8: e61217. 23630581 10.1371/journal.pone.0061217 PMC3632530

[emi470080-bib-0072] Mezzasoma, A. , C. Coleine , C. Sannino , and L. Selbmann . 2022. “Endolithic Bacterial Diversity in Lichen‐Dominated Communities Is Shaped by Sun Exposure in McMurdo Dry Valleys, Antarctica.” Microbial Ecology 83: 328–339. 34081148 10.1007/s00248-021-01769-w PMC8891110

[emi470080-bib-0073] Michaud, L. , C. Caruso , S. Mangano , F. Interdonato , V. Bruni , and A. Lo Giudice . 2012. “Predominance of *Flavobacterium*, *Pseudomonas*, and *Polaromonas* Within the Prokaryotic Community of Freshwater Shallow Lakes in the Northern Victoria Land, East Antarctica.” FEMS Microbiology Ecology 82: 391–404. 22512730 10.1111/j.1574-6941.2012.01394.x

[emi470080-bib-0074] Millanes, A. M. , P. Diederich , S. Ekman , and M. Wedin . 2011. “Phylogeny and Character Evolution in the Jelly Fungi (Tremellomycetes, Basidiomycota, Fungi).” Molecular Phylogenetics and Evolution 61: 12–28. 10.1016/j.ympev.2011.05.014. 21664282

[emi470080-bib-0075] Millanes, A. M. , P. Diederich , M. Westberg , T. Knutsson , and M. Wedin . 2014. “ *Tremella Rhizocarpicola* Sp. Nov. and Other Interesting Tremellales and Filobasidiales in the Nordic Countries.” MycoKeys 8: 31–41.

[emi470080-bib-0076] Millanes, A. M. , P. Diederich , M. Westberg , E. Pippola , and M. Wedin . 2015. “ *Tremella cetrariellae* (Tremellales, Basidiomycota, Fungi), A New Lichenicolous Fungus On Cetrariella delisei.” Lichenologist 47: 359–368.

[emi470080-bib-0077] Millanes, A. M. , M. Westberg , M. Wedin , and P. Diederich . 2012. “ *Tremella diploschistina* (Tremellales, Basidiomycota, Fungi), a New Lichenicolous Species Growing on Diploschistes.” Lichenologist 44: 321–332.

[emi470080-bib-0078] Muggia, L. , A. Fleischhacker , T. Kopun , and M. Grube . 2016. “Extremotolerant Fungi From Alpine Rock Lichens and Their Phylogenetic Relationships.” Fungal Diversity 76: 119–142. 26877720 10.1007/s13225-015-0343-8 PMC4739527

[emi470080-bib-0079] Muggia, L. , and M. Grube . 2018. “Fungal Diversity in Lichens: From Extremotolerance to Interactions With Algae.” Life 8: 15. 29789469 10.3390/life8020015 PMC6027233

[emi470080-bib-0080] Muggia, L. , Y. Quan , C. Gueidan , A. M. Al‐Hatmi , M. Grube , and S. de Hoog . 2021. “Sequence Data From Isolated Lichen‐Associated Melanized Fungi Enhance Delimitation of Two New Lineages Within Chaetothyriomycetidae.” Mycological Progress 20: 911–927.

[emi470080-bib-0081] Nakai, R. , E. Shibuya , A. Justel , et al. 2013. “Phylogeographic Analysis of Filterable Bacteria With Special Reference to Rhizobiales Strains That Occur in Cryospheric Habitats.” Antarctic Science 25: 219–228.

[emi470080-bib-0082] Nemergut, D. R. , E. K. Costello , M. Hamady , et al. 2011. “Global Patterns in the Biogeography of Bacterial Taxa.” Environmental Microbiology 13: 135–144. 21199253 10.1111/j.1462-2920.2010.02315.x PMC5834236

[emi470080-bib-0083] Nizovoy, P. , N. Bellora , S. Haridas , et al. 2021. “Unique Genomic Traits for Cold Adaptation in *Naganishia Vishniacii*, a Polyextremophile Yeast Isolated From Antarctica.” FEMS Yeast Research 21: foaa056. 33232451 10.1093/femsyr/foaa056

[emi470080-bib-0084] Oksanen, J. , F. G. Blanchet , R. Kindt , et al. 2013. “Package ‘Vegan’. Community Ecology Package, Version 2. The Comprehensive R Network (CRAN).”

[emi470080-bib-0085] Olden, J. D. , N. L. Poff , M. R. Douglas , M. E. Douglas , and K. D. Fausch . 2004. “Ecological and Evolutionary Consequences of Biotic Homogenization.” Trends in Eecology and Evolution 19: 18–24. 10.1016/j.tree.2003.09.010 16701221

[emi470080-bib-0086] Onofri, S. , S. Pagano , L. Zucconi , and S. Tosi . 1999. “ *Friedmanniomyces Endolithicus* (Fungi, Hyphomycetes), Anam‐Gen and Sp Nov, From Continental Antarctica.” Nova Hedwigia 68: 175–182.

[emi470080-bib-0087] Onofri, S. , L. Selbmann , G. S. de Hoog , et al. 2007. “Evolution and Adaptation of Fungi at Boundaries of Life.” Advances in Space Research 40: 1657–1664.

[emi470080-bib-0088] Øvstedal, D. O. , and R. I. Lewis Smith . 2001. Lichens of Antarctica and South Georgia. A Guide to Their Identification and Ecology, 1351–5721. Cambridge University Press.

[emi470080-bib-0089] Palmer, J. M. , M. A. Jusino , M. T. Banik , and D. L. Lindner . 2018. “Non‐Biological Synthetic Spike‐In Controls and the AMPtk Software Pipeline Improve Mycobiome Data.” PeerJ 6: e4925. 29868296 10.7717/peerj.4925 PMC5978393

[emi470080-bib-0091] Peeters, K. , D. Ertz , and A. Willems . 2011. “Culturable Bacterial Diversity at the Princess Elizabeth Station (Utsteinen, Sør Rondane Mountains, East Antarctica) Harbours Many New Taxa.” Systematic and Applied Microbiology 34: 360–367. 21501941 10.1016/j.syapm.2011.02.002

[emi470080-bib-0093] Pudasaini, S. , J. Wilson , M. Ji , et al. 2017. “Microbial Diversity of Browning Peninsula, Eastern Antarctica Revealed Using Molecular and Cultivation Methods.” Frontiers in Microbiology 8: 591. 28439263 10.3389/fmicb.2017.00591 PMC5383709

[emi470080-bib-0095] Rognes, T. , T. Flouri , B. Nichols , C. Quince , and F. Mahé . 2016. “VSEARCH: A Versatile Open Source Tool for Metagenomics.” PeerJ 4: e2584. 27781170 10.7717/peerj.2584 PMC5075697

[emi470080-bib-0097] Rush, R. E. , C. B. Blackwood , A. R. Lemons , B. J. Green , and T. L. Croston . 2023. “Persisting *Cryptococcus* Yeast Species *Vishniacozyma victoriae* and *Cryptococcus neoformans* Elicit Unique Airway Inflammation in Mice Following Repeated Exposure.” Frontiers in Cellular and Infection Microbiology 13: 1067475. 36864880 10.3389/fcimb.2023.1067475 PMC9971225

[emi470080-bib-0098] Santiago, I. F. , M. A. Soares , C. A. Rosa , and L. H. Rosa . 2015. “Lichensphere: A Protected Natural Microhabitat of the Non‐Lichenised Fungal Communities Living in Extreme Environments of Antarctica.” Extremophiles 19: 1087–1097. 26400492 10.1007/s00792-015-0781-y

[emi470080-bib-0099] Segata, N. , J. Izard , L. Waldron , et al. 2011. “Metagenomic Biomarker Discovery and Explanation.” Genome Biology 12: 1–18. 10.1186/gb-2011-12-6-r60 PMC3218848 21702898

[emi470080-bib-0100] Segawa, T. , T. Yonezawa , A. Edwards , et al. 2017. “Biogeography of Cryoconite Forming Cyanobacteria on Polar and Asian Glaciers.” Journal of Biogeography 44: 2849–2861.

[emi470080-bib-0101] Selbmann, L. , G. S. De Hoog , A. Mazzaglia , E. I. Friedmann , and S. Onofri . 2005. “Fungi at the Edge of Life: Cryptoendolithic Black Fungi From Antarctic Desert.” Studies in Mycology 51: 1–32.

[emi470080-bib-0102] Selbmann, L. , G. S. De Hoog , L. Zucconi , et al. 2008. “Drought Meets Acid: Three New Genera in a Dothidealean Clade of Extremotolerant Fungi.” Studies in Mycology 61: 1–20. 19287523 10.3114/sim.2008.61.01 PMC2610311

[emi470080-bib-0103] Selbmann, L. , E. Egidi , D. Isola , et al. 2013. “Biodiversity, Evolution and Adaptation of Fungi in Extreme Environments.” Plant Biosystems‐An International Journal Dealing With all Aspects of Plant Biology 14: 237–246.

[emi470080-bib-0104] Selbmann, L. , L. Zucconi , D. Isola , and S. Onofri . 2015. “Rock Black Fungi: Excellence in the Extremes, From the Antarctic to Space.” Current Genetics 61: 335–345. 25381156 10.1007/s00294-014-0457-7

[emi470080-bib-0105] Selbmann, L. , L. Zucconi , S. Ruisi , M. Grube , M. Cardinale , and S. Onofri . 2010. “Culturable Bacteria Associated With Antarctic Lichens: Affiliation and Psychrotolerance.” Polar Biology 33: 71–83.

[emi470080-bib-0106] Silva, L. J. , E. J. Crevelin , D. T. Souza , et al. 2020. “Actinobacteria From Antarctica as a Source for Anticancer Discovery.” Scientific Reports 10: 13870. 32807803 10.1038/s41598-020-69786-2 PMC7431910

[emi470080-bib-0107] Smith, D. P. , and K. G. Peay . 2014. “Sequence Depth, Not PCR Replication, Improves Ecological Inference From Next Generation DNA Sequencing.” PLoS One 9: e90234. 24587293 10.1371/journal.pone.0090234 PMC3938664

[emi470080-bib-0108] Smith, H. B. , F. Dal Grande , L. Muggia , et al. 2020. “Metagenomic Data Reveal Diverse Fungal and Algal Communities Associated With the Lichen Symbiosis.” Symbiosis 82: 133–147.

[emi470080-bib-0109] Spribille, T. 2018. “Relative Symbiont Input and the Lichen Symbiotic Outcome.” Current Oopinion in Plant Biology 44: 57–63. 10.1016/j.pbi.2018.02.007 29529531

[emi470080-bib-0110] Spribille, T. , A. Fryday , S. Pérez‐Ortega , et al. 2020. “Lichens and Associated Fungi From Glacier Bay National Park, Alaska.” Lichenologist 52: 61–181. 32788812 10.1017/S0024282920000079 PMC7398404

[emi470080-bib-0111] Stoppiello, G. A. , C. Coleine , R. Moeller , C. Ripa , D. Billi , and L. Selbmann . 2023. “Seasonality Is the Main Determinant of Microbial Diversity Associated to Snow/Ice Around Concordia Station on the Antarctic Polar Plateau.” Biology 12: 1193. 37759592 10.3390/biology12091193 PMC10525097

[emi470080-bib-0112] Tamaki, H. , Y. Tanaka , H. Matsuzawa , et al. 2011. “ *Armatimonas rosea* Gen. Nov., Sp. Nov., of a Novel Bacterial Phylum, Armatimonadetes Phyl. Nov., Formally Called the Candidate Phylum OP10.” International Jjournal of Systematic and Evolutionary Microbiology 61: 1442–1447. 10.1099/ijs.0.025643-0 20622056

[emi470080-bib-0113] Tang, K. , Z. Huang , J. Huang , et al. 2018. “Characterization of Atmospheric Bioaerosols Along the Transport Pathway of Asian Dust During the Dust‐Bioaerosol 2016 Campaign.” Atmospheric Chemistry and Physics 18: 7131–7148.

[emi470080-bib-0114] Tedersoo, L. , S. Anslan , M. Bahram , et al. 2015. “Shotgun Metagenomes and Multiple Primer Pair‐Barcode Combinations of Amplicons Reveal Biases in Metabarcoding Analyses of Fungi.” MycoKeys 10: 1–43.

[emi470080-bib-0115] Timling, I. , D. Walker , C. Nusbaum , N. Lennon , and D. Taylor . 2014. “Rich and Cold: Diversity, Distribution and Drivers of Fungal Communities in Patterned‐Ground Ecosystems of the North American Arctic.” Molecular Ecology 23: 3258–3272. 10.1111/mec.12743. 24689939

[emi470080-bib-0116] Tolotti, M. , L. Cerasino , C. Donati , et al. 2020. “Alpine Headwaters Emerging From Glaciers and Rock Glaciers Host Different Bacterial Communities: Ecological Implications for the Future.” Science of the Total Environment 717: 137101. 32065887 10.1016/j.scitotenv.2020.137101

[emi470080-bib-0117] Tuovinen, V. , A. M. Millanes , S. Freire‐Rallo , A. Rosling , and M. Wedin . 2021. “ *Tremella macrobasidiata* and *Tremella variae* Have Abundant and Widespread Yeast Stages in *Lecanora* Lichens.” Environmental Microbiology 23: 2484–2498. 33684261 10.1111/1462-2920.15455

[emi470080-bib-0118] Untereiner, W. A. , C. Gueidan , M. J. Orr , and P. Diederich . 2011. “The Phylogenetic Position of the Lichenicolous Ascomycete *Capronia peltigerae* .” Fungal Diversity 49: 225–233.

[emi470080-bib-0119] U'Ren, J. M. , F. Lutzoni , J. Miadlikowska , and A. E. Arnold . 2010. “Community Analysis Reveals Close Affinities Between Endophytic and Endolichenic Fungi in Mosses and Lichens.” Microbial Ecology 60: 340–353. 20625714 10.1007/s00248-010-9698-2

[emi470080-bib-0120] U'Ren, J. M. , F. Lutzoni , J. Miadlikowska , A. D. Laetsch , and A. E. Arnold . 2012. “Host and Geographic Structure of Endophytic and Endolichenic Fungi at a Continental Scale.” American Journal of Botany 99: 898–914. 22539507 10.3732/ajb.1100459

[emi470080-bib-0121] U'Ren, J. M. , J. M. Riddle , J. T. Monacell , I. Carbone , J. Miadlikowska , and A. E. Arnold . 2014. “Tissue Storage and Primer Selection Influence Pyrosequencing‐Based Inferences of Diversity and Community Composition of Endolichenic and Endophytic Fungi.” Molecular Ecology Resources 14: 1032–1048. 24628864 10.1111/1755-0998.12252

[emi470080-bib-0123] White, T. J. , T. Bruns , S. J. W. T. Lee , and J. Taylor . 1990. “Amplification and Direct Sequencing of Fungal Ribosomal RNA Genes for Phylogenetics.” PCR Protocols: A Guide to Methods and Applications 18: 315–322.

[emi470080-bib-0124] Wilcoxon, F. 1945. “Individual Comparisons by Ranking Methods.” Biometrics Bulletin 1: 80–83.

[emi470080-bib-0126] Yung, C. C. , Y. Chan , D. C. Lacap , et al. 2014. “Characterization of Chasmoendolithic Community in Miers Valley, McMurdo Dry Valleys, Antarctica.” Microbial Ecology 68: 351–359. 24671755 10.1007/s00248-014-0412-7

