# Supplementary figures and images for: Intrathalline Fungal and Bacterial Diversity Is Uncovered in Antarctic Lichen Symbioses
Source: Environ Microbiol Rep. 2025 May 5;17(3):e70080. doi: 10.1111/1758-2229.70080 (PMC12052756; doi:10.1111/1758-2229.70080)

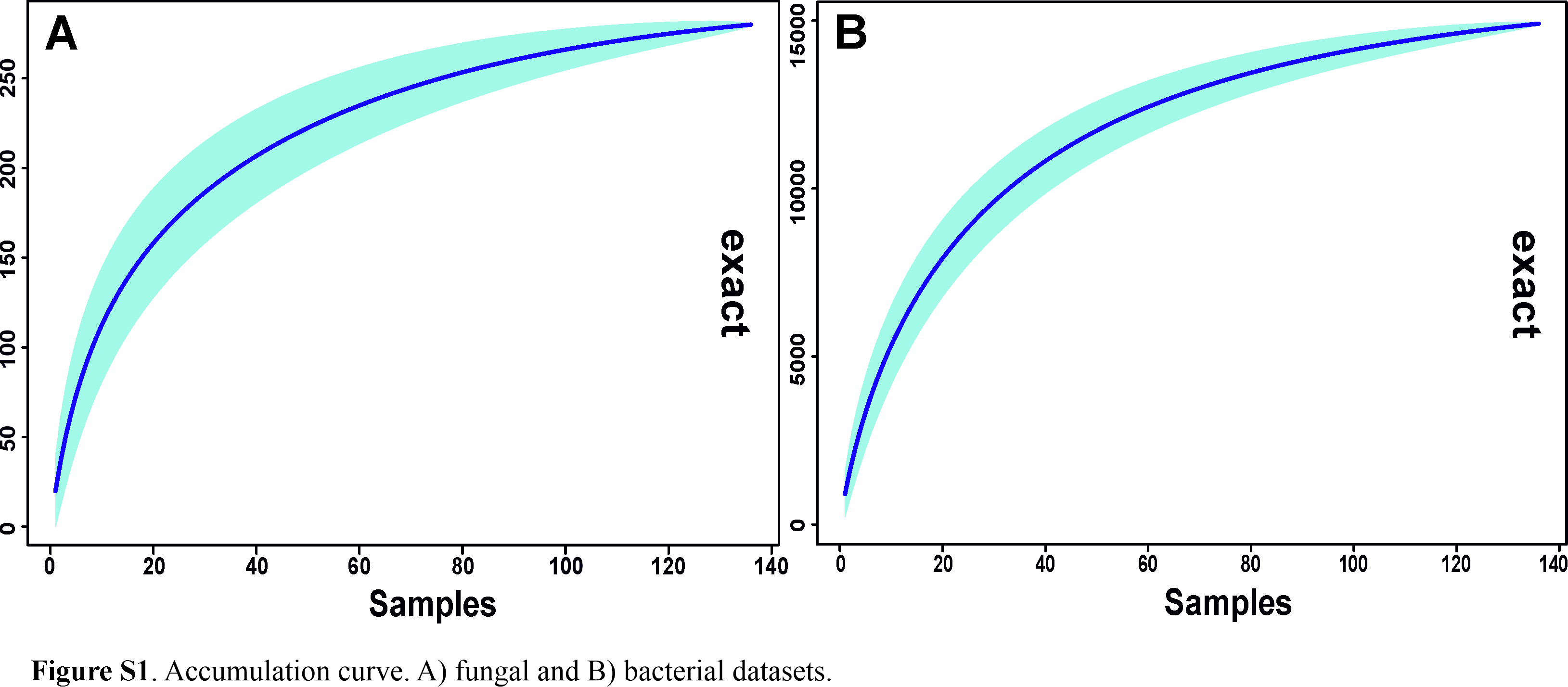

Supplement: Supplementary file 1 — Figure S1. Species accumulation curves of fungal (A) and bacterial (B) dataset. [file EMI4-17-e70080-s009.tif]

Linear Regression of Shannon Diversity Indices

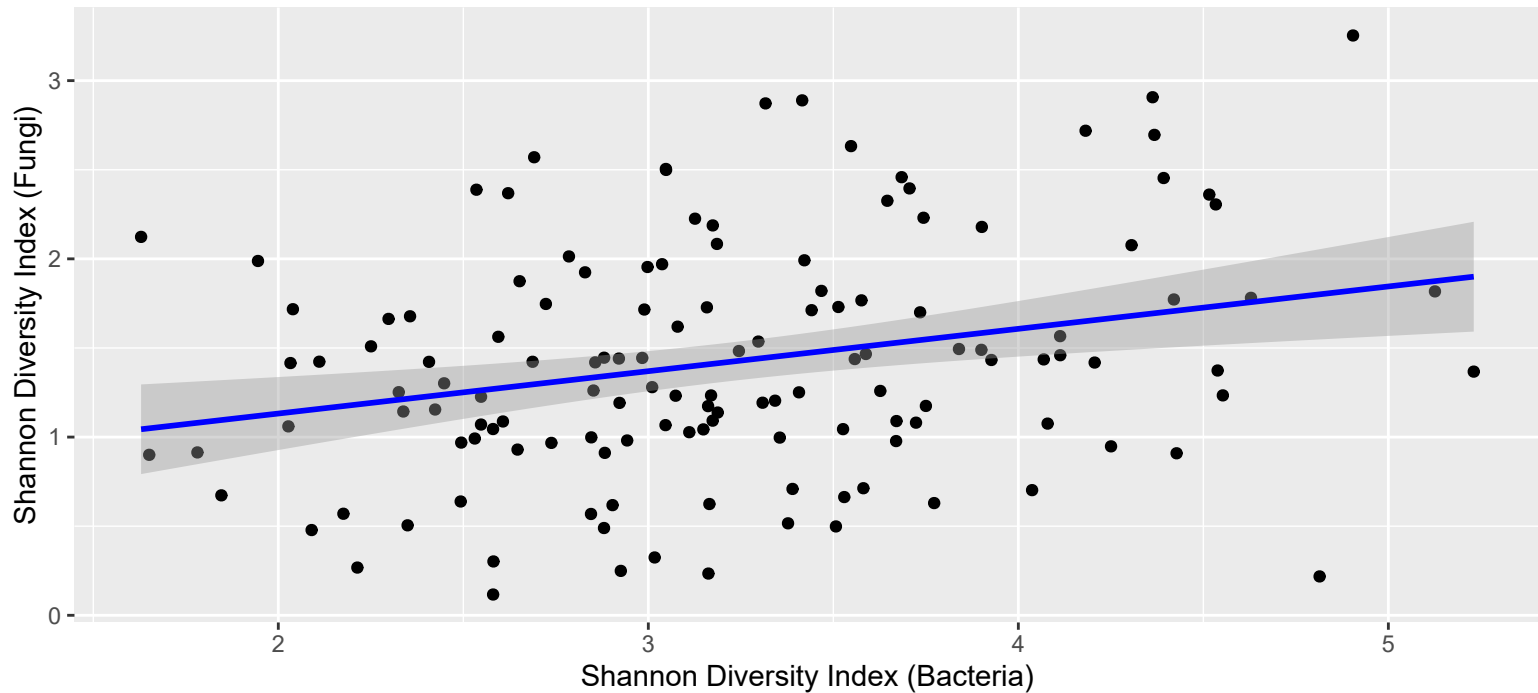

Supplement: Supplementary file 3 — Figure S3. Linear regression analysis between the Shannon indices of the bacterial and fungal communities. [file EMI4-17-e70080-s010.pdf]
