# Supplementary material for: Intrathalline Fungal and Bacterial Diversity Is Uncovered in Antarctic Lichen Symbioses
Source: Environ Microbiol Rep. 2025 May 5;17(3):e70080. doi: 10.1111/1758-2229.70080 (PMC12052756; doi:10.1111/1758-2229.70080)
Supplement: Supplementary file 2 — Figure S2. Species accumulation curves performed for each lichen species on the fungal (A) and the bacterial (B) datasets. [file EMI4-17-e70080-s001.pdf]

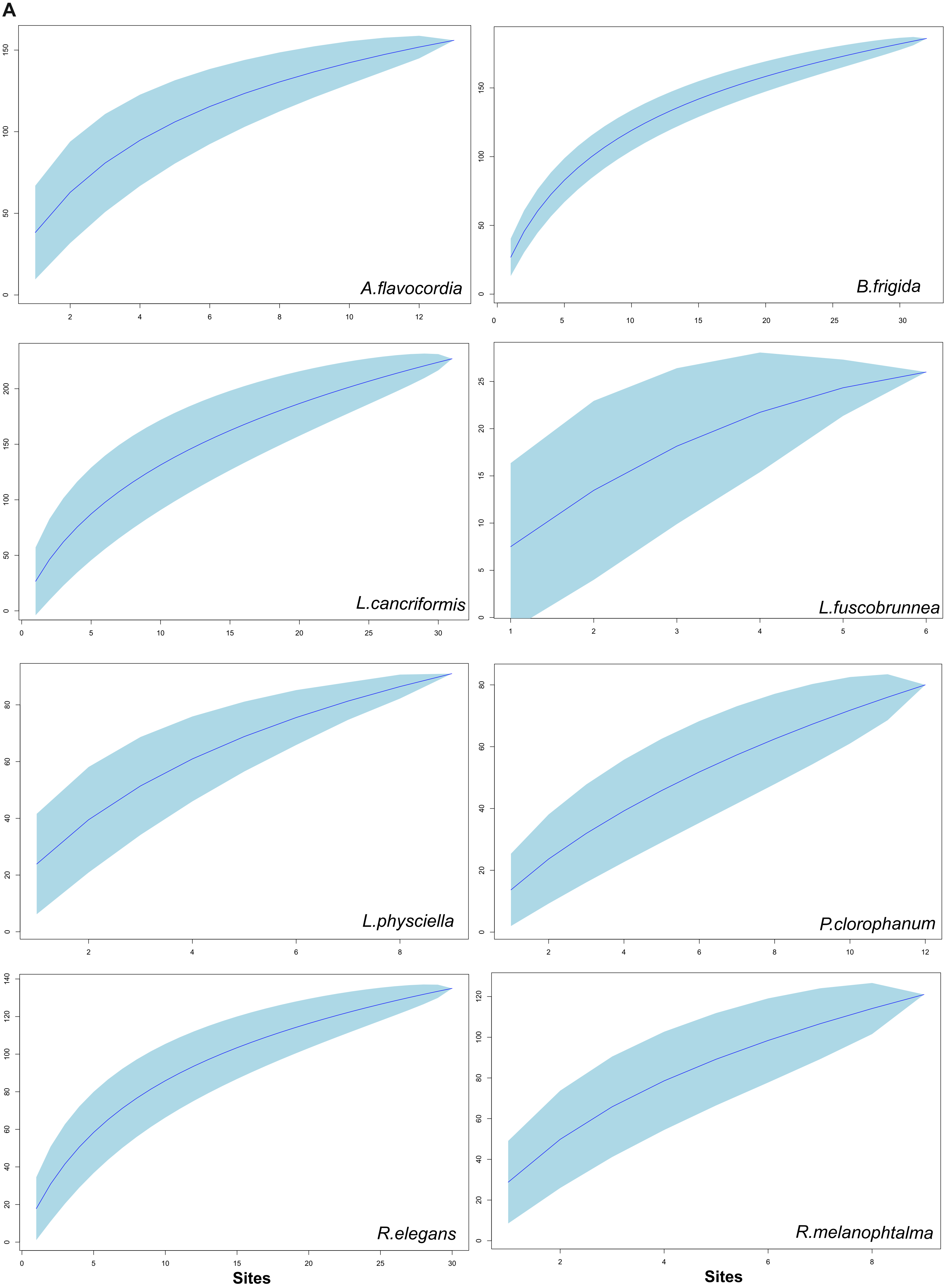

A) Fungal species accumulation curves performed on each lichen spieces

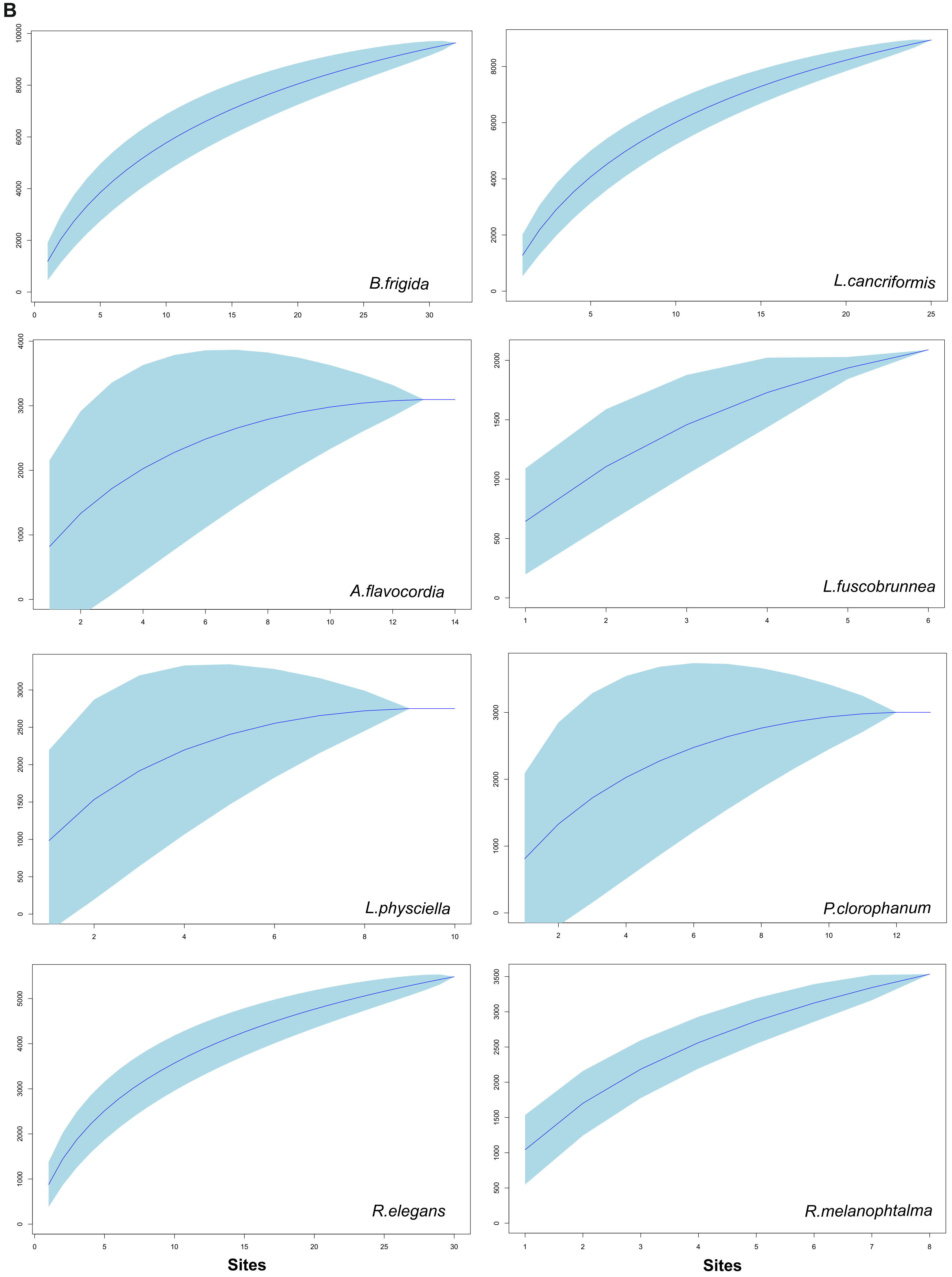

B) Bacterial species accumulation curves performed on each lichen species
