# Supplementary material for: Intrathalline Fungal and Bacterial Diversity Is Uncovered in Antarctic Lichen Symbioses
Source: Environ Microbiol Rep. 2025 May 5;17(3):e70080. doi: 10.1111/1758-2229.70080 (PMC12052756; doi:10.1111/1758-2229.70080)
Supplement: Supplementary file 13 — Supporting Information 1. List of the sequenced fungal ASVs and their nucleotide sequence. [file EMI4-17-e70080-s005.docx]

>ASV1 GS|100.0|AY667583|SH0954634.09FU;k:Fungi,p:Ascomycota,c:Lecanoromycetes,o:Caliciales,f:Caliciaceae,g:Buellia,s:Buellia frigida

AAGTCGTAACAAGGTTTCCGTAGGTGAACCTGCGGAAGGATCATTACCGAGAGACGGGGT

CGCTTCGGCCCCACTCTTCACCCGTGTCTACTTACCTTTGTTGCTTTGGCGGGCCTTCGG

GCTTGACCGTTCGACGCGGGTGGGAGGCTTTCGCCTCTCGCCTCGCGAGCGCCCGCCAAA

GGCCCTGTTAACTCTGTTTTAGTGTCTTCCGAGCAACCATGTAATAGTTAAAACTTTCAA

CAACGGATCT

>ASV2 GS|100.0|AF281306|SH1300522.09FU;k:Fungi,p:Ascomycota,c:Lecanoromycetes,o:Teloschistales,f:Teloschistaceae,g:Xanthoria,s:Xanthoria elegans

AAGTCGTAACAAGGTTTCCGTAGGTGAACCTGCGGAAGGATCATTACTAAGAGAGGGATG

TACGCTTCCAGCCGAGTCCCGGGGGGCTGCGCCCCTCACCTCTTCAACCCTGTGTCTACC

AACCGCTGTTGCTTCGGCGAGCGTCGGGGCGTCCGCGCCCCGGCCCCGGCTTCGGTCGGT

GAGCTCTCGCAGAGGCCTATCTTTATTCTGTTTTGCAGTGACGTCCGAGAATACCAATAT

AATCAATCAA

>ASV3 GS|100.0|JN873879|SH1123218.09FU;k:Fungi,p:Ascomycota,c:Lecanoromycetes,o:Lecanorales,f:Lecanoraceae,g:Lecanora,s:Lecanora physciella

AAGTCGTAACAAGGTTTCCGTAGGTGAACCTGCGGAAGGATCATTACCGAGAGCGGGGCT

AACCCCCCAAACTCCGCCGCCGAAAGGGGTACTCTCCACCCTATGTATACATATACCACT

CTCGCTTTGGCGGGCTGAAGGCTCTTGCCCTACGCCCGCCAGTGGCTCAAAAAATTCTGT

TTATCAGTGATGTCCGAGTAAAAACCTAATAGTTAAAACTTTCAACAACGGATCTCTTGG

TTCTG

>ASV4 GS|100.0|DQ525481|SH0954363.09FU;k:Fungi,p:Ascomycota,c:Lecanoromycetes,o:Acarosporales,f:Acarosporaceae,g:Pleopsidium,s:Pleopsidium chlorophanum

AAGTCGTAACAAGGTTTCCTTCCGGGTGTAGCACCTGCCGAAGCCTCCCGCAGCGACTCT

AAAGAAACCGCGCAGTCTGCCCCCCTGCAGGCAACACTGTCACTGTGCTGGGAGCCAGTC

ACGCCTGGTTCAGGCGCCGCTACCAGCAACCTGGGCAACAACGCCCAGGCTCACAGATCA

GATGATTGTGGCCACACCGTGGTTAAGATATGACCGGTCCCCGCTGTGGACAGCTGGGAG

CTTACGTTCC

>ASV5 GSL|100.0|AF278757|SH1300510.09FU;k:Fungi,p:Ascomycota,c:Lecanoromycetes,o:Teloschistales,f:Teloschistaceae,g:Caloplaca,s:Caloplaca rouxii

AAGTCGTAACAAGGTTTCCGTAGGTGAACCTGCGGAAGGATCATTACCGAGAGGGACATG

CGCCTCGCGGCCATGTCCCGGGGGGTTCCGCCCCCATCTCTTCAACCCTTGCCTATCTAC

CTCTGTTGCTTCGGCGAGCGCTCGGGTGCCATTGCGCCCGGGCCCCGGCTTCGGTCGGTG

CGCTCTCGCCGGAGGCCATATTGAACCTGTCTGTAGTGATGTCTGAGTGTACCGATTAAA

ATCAAAACTT

>ASV6 SS|1.0000|GU184109|SH0954388.09FU;k:Fungi,p:Ascomycota,c:Lecanoromycetes,o:Acarosporales,f:Acarosporaceae

AAGTCGTAACAAGGTTTCCATGCGGAGGGACATTCATCTTACTCGATCTTCACATCTGTA

AGCAAACCACCTCTGAAGCCTTGAAATCATGAGTCTGCATCTTTGCAGGCGACACTAGTC

CGTTGCTGGGAGTCTCCTGGAGATAATCAGCAGCCACGATACTGGATTGTGGTTCACAGA

TCAAACACTAGTGGCCCGTTGGTGGGTTAAAATATGACCGGCTCCATCTGGAAATAGATG

GACGATTGCG

>ASV7 GS|99.6|AY667583|SH0954634.09FU;k:Fungi,p:Ascomycota,c:Lecanoromycetes,o:Caliciales,f:Caliciaceae,g:Buellia,s:Buellia frigida

AAGTCGTAACAAGGTTTCCGTAGGTGAACCTGCGGAAGGATCATTACCGAGAGACGGGGT

CGCTTCGGCCCCACTCTTCACCCGTGTCTACTTACCTTTGTTGCTTTGGCGGGCCTTCGG

GCTTGACCGTTCGACGCGGGTGGGAGGCTTTCGCCTGTCGCCTCGCGAGCGCCCGCCAAA

GGCCCTGTTAACTCTGTTTTAGTGTCTTCCGAGCAACCATGTAATAGTTAAAACTTTCAA

CAACGGATCT

>ASV8 GS|99.6|AY667583|SH0954634.09FU;k:Fungi,p:Ascomycota,c:Lecanoromycetes,o:Caliciales,f:Caliciaceae,g:Buellia,s:Buellia frigida

AAGTCGTAACAAGGTTTCCGTAGGTGAACCTGCGGAAGGATCATTACCGAGAGACGGGGT

CGCTTCGGCCCCACTCTTCACCCGTGTCTACTTACCTTTGTTGCTTTGGCGGGCCTTCGG

GCTTGACCGTTCGACGCGGGTGGGAGGCTTTCGCCTCTCGCCTCGCGAGCGCCCGCCAAA

GGCCGTGTTAACTCTGTTTTAGTGTCTTCCGAGCAACCATGTAATAGTTAAAACTTTCAA

CAACGGATCT

>ASV9 GS|100.0|JN873872|SH0942719.09FU;k:Fungi,p:Ascomycota,c:Lecanoromycetes,o:Lecanorales,f:Lecanoraceae,g:Lecanora,s:Lecanora fuscobrunnea

AAGTCGTAACAAGGTTTCCGTAGGTGAACCTGCGGAAGGATCATTATCGAGAGGGGTCCC

CGGACTCCGGGGGCTTCGGCCCCCTACTCTTCACCCTATGTCTACACACCTTTGTTGCTT

TGGCGGGCCTCGGGTTCGCCCCGTACCGGCCGTGGGCTTCCATACCCCGGCCGTCCGTGC

CCGTCAGAGGCCCATGAACCCTCGTTTATCAGTGTCGTCCGAGTCCAACCATAATAGTAA

AAACTTTCAA

>ASV10 GS|99.5|JN873879|SH1123218.09FU;k:Fungi,p:Ascomycota,c:Lecanoromycetes,o:Lecanorales,f:Lecanoraceae,g:Lecanora,s:Lecanora physciella

AAGTCGTAACAAGGTTTCCGTAGGTGAACCTGCGGAAGGATCATTACCGAGAGCGGGGCT

AACCCCCCAAACTCCGCCGCCGAAAGGGGTACTCTCCACCCTATGTATACATATACCACT

CTCGCTTTGGCGGGCTGAAGGCTCTTGCCCTACGCCCGCCAGTGGCTCAAAAAATTCTGT

TCATCAGTGATGTCCGAGTAAAAACCTAATAGTTAAAACTTTCAACAACGGATCTCTTGG

TTCTG

>ASV11 GS|99.2|MW465660|SH1261156.09FU;k:Fungi,p:Ascomycota,c:Lecanoromycetes,o:Lecanorales,f:Lecanoraceae,g:Rhizoplaca,s:Rhizoplaca melanophthalma

AAGTCGTAACAAGGTTTCCGTAGGTATGCAAGACTGCACGTTTGCCTACGGGAGCCCTCG

CAGCGACTCTAAACAAGTGCCTCAGCCTGCGCCCCGAGTGGGACTGGCGACGCCGTCAAT

CTGCGCTGGGAGGACCCCCTTGGGGCTCAACCAGAAGCTCTTGAATGAGTTCACAGATCA

GACGATGGCGGCCACTTGCGAGTGGTTTAGATATGACCGGCCCCGGCCCTTTATCGGCCG

GCGAATCTGC

>ASV12 GS|99.2|AY667583|SH0954634.09FU;k:Fungi,p:Ascomycota,c:Lecanoromycetes,o:Caliciales,f:Caliciaceae,g:Buellia,s:Buellia frigida

AAGTCGTAACAAGGTTTCCGTAGGTGAACCTGCGGAAGGATCATTACCGAGAGACGGGGT

CGCTTCGGCCCCACTCTTCACCCGTGTCTACTTACCTTTGTTGCTTTGGCGGGCCTTCGG

GCTTGACCGTTCGACGCGGGTGGGAGGCTTTCGCCTCTCGCCTCGCGAGCGCCCGCCAAA

GACTCTGTTAACTCTGTTTTAGTGTCTTCCGAGCAACCATGTAATAGTTAAAACTTTCAA

CAACGGATCT

>ASV13 SS|0.8800|MN592663|SH1107827.09FU;k:Fungi,p:Ascomycota,c:Lecanoromycetes,o:Teloschistales,f:Teloschistaceae

AAGTCGTAACAAGGTTTCCGTAGGTGAACCTGCGGAAGGATCATTATCGAGAGGGGGGCT

CCATGCCCCGGGGCTCTGTCCCCGTACCTTTTCACCCTGTGTGTATTTTTCCCCCGTTGC

TTTGGCGGGCCCCGGGTCTTCCCCCGGCGTTGGCCCCCTCGCGGGGTTCGCGAGCGCCCG

CCGAAGGCTCATCGAAACTCTGTTGATCAGTGCAGTCTGAGCGTACGAACAATAAATCAA

AACTTTCAAC

>ASV14 GS|98.8|AY667583|SH0954634.09FU;k:Fungi,p:Ascomycota,c:Lecanoromycetes,o:Caliciales,f:Caliciaceae,g:Buellia,s:Buellia frigida

AAGTCGTAACAAGGTTTCCGTAGGTGAACCTGCGGAAGGATCATTACCGAGAGACGGGGT

CGCTTCGGCCCCACACTCTTCACCCGTGTCTACTTACCTTTGTTGCTTTGGCGGGCCTTC

GGGCTTGACCGTTCGACGCGGGTGGGAGGCTTTCGCCTCTCGCCTCGCGAGCGCCCGCCA

AAGGCCCTGTTAACTCTGTTTTAGTGTCTTCCGAGCAACCATGTAATATTTAAAACTTTC

AACAACGGAT

>ASV15 GS|97.6|AY667583|SH0954634.09FU;k:Fungi,p:Ascomycota,c:Lecanoromycetes,o:Caliciales,f:Caliciaceae,g:Buellia,s:Buellia frigida

AAGTCGTAACAAGGTTTCCGTAGGTGAACCTGCGGAAGGATCATTACCGAGAGACGGGGT

CGCTTCGGCCCCACTCTTCACCTTCACCCGTGTCTACTTACCTTTGTTGCTTTGGCGGGC

CTTCGGGCTTGACCGTTCGACGCGGGTGGGAGGCTTTCGCCTCTCGCCTCGCGAGCGCCC

GCCAAAGGCCCTGTTAACTCTGTTTTAGTGTCTTCCGAGCAACCATGTAATAGTTAAAAC

TTTCAACAAC

>ASV16 GS|99.2|AY667583|SH0954634.09FU;k:Fungi,p:Ascomycota,c:Lecanoromycetes,o:Caliciales,f:Caliciaceae,g:Buellia,s:Buellia frigida

AAGTCGTAACAAGGTTTCCGTAGGTGAACCTGCGGAAGGATCATTACCGAGAGACGGGGT

CGCTTCGGCCCCACTCTTCACCCGTGTCTACTTACCTTTGTTGCTTTGGCGGGCCTTCGG

GCTTGACCGTTCGACGCGGGTGGGAGGCTTTCGCCTCTCGCCTCGCGAGCGCCCGCCAAA

GGCCCTGTTAACTCTGTTTTAGTGTCTTCCGAGCAGCCATGTAATTGTTAAAACTTTCAA

CAACGGATCT

>ASV17 GS|99.6|AY667583|SH0954634.09FU;k:Fungi,p:Ascomycota,c:Lecanoromycetes,o:Caliciales,f:Caliciaceae,g:Buellia,s:Buellia frigida

AAGTCGTAACAAGGTTTCCGTAGGTGAACCTGCGGAAGGATCATTACCGAGAGACGGGGT

CGCTTCGGCCCCACTCTTCACCCGTGTCTACTTACCTTTGTTGCTTTGGCGGGCCTTCGG

GCTTGACCGTTCGACGCGGGTGGGAGGCTTTCGCCTCTCGCCTCGCGAGCGCCCGCCAAA

GGCCCTGTTAACTCTGTTTTAGTGTTTTCCGAGCAACCATGTAATAGTTAAAACTTTCAA

CAACGGATCT

>ASV18 GS|100.0|UDB06367067|SH0986024.09FU;k:Fungi,p:Ascomycota,c:Lecanoromycetes,o:Rhizocarpales,f:Rhizocarpaceae,g:Rhizocarpon

AAGTCGTAACAAGGTTTCCGTAGGTGAACCTGCGGAAGGATCATTACCGAGATAGGGGCT

CCGGCCCCGAACCTCCGACCCCTGTCTACCCACCTCTGTTGCTTTGGCGGGCCCGTGCCT

GGGCTGGTCCCGGCACCTCCGCCCCCGGGGTGTGCCCGCCGAAGGCCCTTTCAAAACTCG

TTTACCGGTGCAGTCTGAGCAAATAATCAATAATTAAAACTTTCAACAACGGATCTCTTG

GTTCTG

>ASV19 SS|0.8600|KU672610|SH0991977.09FU;k:Fungi,p:Ascomycota,c:Lecanoromycetes,o:Trapeliales,f:Trapeliaceae,g:Trapelia

AAGTCGTAACAAGGTTTCCGTAGGTGAACCTGCGGAAGGATCATTACCGAGATAGGGTCC

CCCGGGCCCGACCCTCCACCCGCTGCGTACCTACCTTTTGTTGCTTTGGCGGGCCGCGGG

GCCCCCGGCCCCCCGTCGACCCCGGTGGGCGAGCGCCCGCCAGAGACCCCCCCAACCCGG

TTGATCAGTGACGTCCGAGCCCCGATGAAAATCAATTAAAACTTTCAACAACGGATCTCT

TGGTTCTG

>ASV20 GS|99.6|AY667583|SH0954634.09FU;k:Fungi,p:Ascomycota,c:Lecanoromycetes,o:Caliciales,f:Caliciaceae,g:Buellia,s:Buellia frigida

AAGTCGTAACAAGGTTTCCGTAGGTGAACCTGCGGAAGGATCATTACCGAGAGACGGGGT

CGCTTCGGCCCCACTCTTCACCCGTGTCTACTTACCTTTGTTGCTTTGGCGGGCCTTCGG

GCTTGACCGTTCGACGCGGGTGGGAGGCTTTTGCCTCTCGCCTCGCGAGCGCCCGCCAAA

GGCCCTGTTAACTCTGTTTTAGTGTCTTCCGAGCAACCATGTAATAGTTAAAACTTTCAA

CAACGGATCT

>ASV21 GS|99.6|JX036119|SH0942719.09FU;k:Fungi,p:Ascomycota,c:Lecanoromycetes,o:Lecanorales,f:Lecanoraceae,g:Rhizoplaca

AAGTCGTAACAAGGTTTCCGTAGGTGAACCTGCGGAAGGATCATTATCGAGAGGGGTCCT

CGGACTCCGGGGGCTTCGGCCCCCTACTCTTCACCCTATGTCTACACACCTTTGTTGCTT

TGGCGGGCCTCGGGTTCGCCCCGTACCGGCCGTGGGCTTCCATACCCCGGCCGTCCGTGC

CCGTCAGAGGCCCATGAACCCTCGTTTATCAGTGTCGTCCGAGTCCAACCATAATAGTAA

AAACTTTCAA

>ASV22 GS|99.2|AY667583|SH0954634.09FU;k:Fungi,p:Ascomycota,c:Lecanoromycetes,o:Caliciales,f:Caliciaceae,g:Buellia,s:Buellia frigida

AAGTCGTAACAAGGTTTCCGTAGGTGAACCTGCGGAAGGATCATTACCGAGAGACGGGGT

CGCTTCGGCCCCACTCTTCACCCGTGTCTACTTACCTTTGTTGCTTTGGCGGGCCTTCGG

GCGCGACCGTTCGACGCGGGTGGGAGGCTTTCGCCTCTCGCCTCGCGAGCGCCCGCCAAA

GGCCCTGTTAACTCTGTTTTAGTGTCTTCCGAGCAACCATGTAATAGTTAAAACTTTCAA

CAACGGATCT

>ASV23 SS|0.9700|MT809271|SH0954085.09FU;k:Fungi

AAGTCGTAACAAGGTTTCCGTAGGTGAACCTGCGGAAGGATCATTACCGAGCTAGGGCCT

TCTAGGTCTGACCTCCCACCCTATGTTTATCAGCTCCTTGTTGCTTCGGCGGACCGTTGG

GGTCAAACCCGCCGCAGGCTTTCGGGCTTGTGAGCGTCCGTCGGAGGATACTTTTAACGC

GTTTAATTCATGTGGTCTGAGTGGGTATTTAATCACCTTAAAACTTTCAACAACGGATCT

CTTGGTTCTG

>ASV24 SS|1.0000|KF274227|SH0911134.09FU;k:Fungi

AAGTCGTAACAAGGTTTCCGTAGGTGAACCTGCGGAAGGATCATTACCGAGTTAGGGTCC

TCTGGGCTCGATCTCCAACCCTGTGTCTAAATAACCACGTTGCTTTGGCGGGCCCGCCTG

TAATGGGCCGCCGGGGGTGCCTTCGGCCCCCTGGTCCGCGCCTGCCAGTAGCCATCTCAA

ACTCTTCTTAATCGTGACGTCTGAGTAAAAATTATAAATTAAACAAAACTTTCAACAACG

GATCTCTTGG

>ASV25 GS|99.6|AY667583|SH0954634.09FU;k:Fungi,p:Ascomycota,c:Lecanoromycetes,o:Caliciales,f:Caliciaceae,g:Buellia,s:Buellia frigida

AAGTCGTAACAAGGTTTCCGTAGGTGAACCTGCGGAAGGATCATTACCGAGAGACGGGGT

CGCTTCGGCCCCACTCTTCACCCGTGTCTACTTACCTTTGTTGCTTTGGCGGGCCTTCGG

GCTTGACCGTTCGACGCGGGTGGGAGGCTTTCGCCTCTCGCCTCGCGAGCGCCCGCCAAA

GGCCCTGTTAACTCTGTTTTAGTGTCTTCCGACCAACCATGTAATAGTTAAAACTTTCAA

CAACGGATCT

>ASV26 GS|99.6|AY667583|SH0954634.09FU;k:Fungi,p:Ascomycota,c:Lecanoromycetes,o:Caliciales,f:Caliciaceae,g:Buellia,s:Buellia frigida

AAGTCGTAACAAGGTTTCCGTAGGTGAACCTGCGGAAGGATCATTACCGAGAGACGGGGT

CGCTTCGGCCCCACTCTTCACCCGTGCCTACTTACCTTTGTTGCTTTGGCGGGCCTTCGG

GCTTGACCGTTCGACGCGGGTGGGAGGCTTTCGCCTCTCGCCTCGCGAGCGCCCGCCAAA

GGCCCTGTTAACTCTGTTTTAGTGTCTTCCGAGCAACCATGTAATAGTTAAAACTTTCAA

CAACGGATCT

>ASV27 SS|0.9900|KF823589|SH1091255.09FU;k:Fungi

AAGTCGTAACAAGGTTTCCGTAGGTGAACCTGCGGAAGGATCATTAGGGATTTGGTCTCC

GGACCTTTTTCATATCCATAATACCCCTGTGAACCTGTCGGTCCTCGGGCCAGCGTTTCC

AAACCATGTGTAATGAACGTGAATGTGTATGAAACCCTAGTAAAACTTTCAACAACGGAT

CTCTTGGCTCTC

>ASV28 GS|99.2|DQ525481|SH0954363.09FU;k:Fungi,p:Ascomycota,c:Lecanoromycetes,o:Acarosporales,f:Acarosporaceae,g:Pleopsidium,s:Pleopsidium chlorophanum

AAGTCGTAACAAGGTTTCCTTCCGGGTGTAGCACCCGCCGAAGCCTCCCGCAGCGACTCT

AAAGAAACCGCGCAGTCTGCCCCCCTGCAGGCAACACTGTCACTGTGCTGGGAGCCTGTC

ACGCCTGGTTCAGGCGCCGCTACCAGCAACCTGGGCAACAACGCCCAGGCTCACAGATCA

GATGATTGTGGCCACACCGTGGTTAAGATATGACCGGTCCCCGCTGTGGACAGCTGGGAG

CTTACGTTCC

>ASV29 SS|1.0000|MT237054|SH0978826.09FU;k:Fungi

AAGTCGTAACAAGGTTTCCGTAGGTGAACCTGCGGAAGGATCATTACCGAGTTCCTGCCC

TTCGGGGTAGACCTCCCACCCTTGCTCAACCTGCCCTGTTGCTTTGGCGGGCCGTCCCCA

CGGCCACCGGCTTCGGCTGGTGCGTGCCCGCCAGAGACCCCAAACGCTGAATCTCGAGTG

TCGTCTGAGTCCTATATAATCGTTAAAACTTTCAACAACGGATCTCTTGGCTCTG

>ASV30 SS|0.9900|MT237021|SH1031428.09FU;k:Fungi

AAGTCGTAACAAGGTTTCCGTAGGTGAACCTGCGGAAGGATCATTACCGAGTTCATGCCC

CCCGGGGTAGATCTCCCACCCTTGCTTGAATTACCTTGTTGCTTTGGCGGGCCGCTCTCT

TTGAAAGCCACCGGCTCCCCGCTGGTGAGTGCCCGCCGAAGACCAACCAACGTCGTGTCA

CTGCAGCAGTCTAAGTAATATACAAATTGAAATAAAACTTTCAACAACGGATCTCTTGGC

TCTG

>ASV31 GS|85.8|JX036103|SH1123214.09FU;k:Fungi,p:Ascomycota,c:Lecanoromycetes,o:Lecanorales,f:Catillariaceae,g:Austrolecia

AAGTCGTAACAAGGTTTCCGTAGGTAGCATGTTTGCCTTCGGAGCCCCCGCAGCGACCTT

AAATAAATGCTCTAGTCGGTCCCCTCCAGGGCCGGCGACGCTATCAATCTGTGCTGGGAG

CGCCCTCTGGGGTGCGATCAGCAGCTCTCCCCCCCCGGGCAAGAGTTCACAGATCAAACG

ATAGCGGCCGCCCGGTGCGGTTCAGATATGATCGGCCTCAGCTGCACACCGGCTGGCGAC

TTTGCGGAAC

>ASV32 GS|99.5|UDB01588125|SH0913118.09FU;k:Fungi,p:Ascomycota,c:Lecanoromycetes,o:Caliciales,f:Caliciaceae

AAGTCGTAACAAGGTTTCCGTAGGTGCTGCAGCCTCCCAAGGTTCGCCTACCGAAGCCCC

GGGCGACTCTAAACAATCCGCCCAGTCAGTCGCCACTGGCGGCTGGCCACGCAGCCATTC

TGGCTTGGAAGCCCCCCGAGGAGCCCCCTCGGCGGCTACCAGCAGCTCCGGCACCGTCGA

GTGCCGGAGTCCACAGATCAAATACCTGCGGCCACATCTGTGGTTCTGATATGACCGACC

GCCGCCCGCA

>ASV33 GS|99.0|MK208766|SH1123218.09FU;k:Fungi,p:Ascomycota,c:Lecanoromycetes,o:Lecanorales,f:Lecanoraceae,g:Lecanora,s:Lecanora physciella

AAGTCGTAACAAGGTTTCCGTAGGTGAACCTGCGGAAGGATCATTACCGAGAGCGGGGCT

AACCCCCCAAACTCCGCCGCCGAAAGGGGTACTCTCCACCCTATGTATACATATACCACT

CTCGCTTTGGCGGGCTGAAGGCTCTTGCCCTACGCGCCCGCCAGTGGCTCAAAAAATTCT

GTTTATTAGTGATGTCCGAGTAAAAACCTAATAGTTAAAACTTTCAACAACGGATCTCTT

GGTTCTG

>ASV34 SS|0.8200|MW580898|SH1196660.09FU;k:Fungi,p:Basidiomycota,c:Tremellomycetes,o:Tremellales,f:Tremellaceae,g:Tremella

AAGTCGTAACAAGGTTTCCGTAGGTGAACCTGCGGAAGGATCATTAGTGATTCGGCCCTC

ACGGGTCTATAAAAGACACCTCTGTGAACCTGTCGGCCTCCGGGCCCACCTGCAAACACT

GTGTAACGAGCGTTGATGTATCATAAGCATAATAAAACTTTCAACAACGGATCTCTTGGC

TCTC

>ASV35 GS|100.0|FJ392865|SH1071027.09FU;k:Fungi,p:Ascomycota,c:Eurotiomycetes,o:Chaetothyriales,f:Trichomeriaceae,g:Knufia

AAGTCGTAACAAGGTTTCCGTAGGTGAACCTGCGGAAGGATCATTACCGAGTTAGGGTCT

CTTCGGAGCCCGAACCTCCCAACCCTTTGTCTAATTTACCTTGTCGTTGCTTCGGCGGAC

CGGTTGACCAACTGGTCTTGACCGCCGGGGGTCCCGTACCCCTGGAGAGCGTCCGCCGAC

GGCCCAACCACAAACTCTTGTACTAAACCATGTCGTCTGAATGTCCTTGATATTAATCAA

AAAACAAAAC

>ASV36 GS|0.0|None;No hit

AAGTCGTAACAAGGTTTTCGTAGGTGAACCTGCGGAAGGATCATTACCAGATGGGGCCTC

CGGGCCTTTTTCTCCTTACCACGTGCACCGAGTTGTCGGGAGACGCTTGTTTCCCGACCA

ATTCTTCACGCTTGTTTTGTTGTCTGAGTCGAATGACCATAATTGAAAAGAAAACTATGA

ACAACGGATCTCTAGGCTCTT

>ASV37 GS|100.0|MW991425|SH0964445.09FU;k:Fungi,p:Ascomycota,c:Arthoniomycetes,o:Lichenostigmatales,f:Phaeococcomycetaceae,g:Antarctolichenia,s:Antarctolichenia onofrii

AAGTCGTAACAAGGTCTCCGTAGGTGAACCTGCGGAGGGATCATTAATGAGATAGGGTCT

TCACGGCCCGACCTCCAACCCAATGTCTACCATACCTCTGTTGCCTCGGCGGGCCGCCGG

CGCCCTTTGTTGGGCGCCGCCGTCGGCTTTAGGGCTTTCGAGCGCCCGCCGCAGGACCGA

TTAAACTCTTTTTAAAACAAGTCTTCTGAGTGGGAAATCAAATTTATTAAAACTTTTAAC

AACGGATCTC

>ASV38 GS|99.6|AY667583|SH0954634.09FU;k:Fungi,p:Ascomycota,c:Lecanoromycetes,o:Caliciales,f:Caliciaceae,g:Buellia,s:Buellia frigida

AAGTCGTAACAAGGTTTCCGTAGGTGAACCTGCGGAAGGATCATTACCGAGAGACGAGGT

CGCTTCGGCCCCACTCTTCACCCGTGTCTACTTACCTTTGTTGCTTTGGCGGGCCTTCGG

GCTTGACCGTTCGACGCGGGTGGGAGGCTTTCGCCTCTCGCCTCGCGAGCGCCCGCCAAA

GGCCCTGTTAACTCTGTTTTAGTGTCTTCCGAGCAACCATGTAATAGTTAAAACTTTCAA

CAACGGATCT

>ASV39 GS|100.0|KP174857|SH1071027.09FU;k:Fungi,p:Ascomycota,c:Eurotiomycetes,o:Chaetothyriales,f:Trichomeriaceae,g:Knufia,s:Knufia separata

AAGTCGTAACAAGGTTTCCGTAGGTGAACCTGCGGAAGGATCATTACCGAGTTAGGGTCT

CTTCGGAGCCCGAACCTCCCAACCCTTTGTCTAATTTACCTTGTCGTTGCTTCGGCGGAC

CGGTTGACCAACTGGTCTTGACCGCCGGGGGTCCCGCACCCCTGGAGAGCGTCCGCCGAC

GGCCCAACCACAAACTCTTGTACTAAACCATGTCGTCTGAATGTACTTGATATTAATCAA

AAAACAAAAC

>ASV40 GS|98.2|UDB0479157|SH0953972.09FU;k:Fungi,p:Ascomycota,c:Dothideomycetes,o:Botryosphaeriales,f:Botryosphaeriaceae,g:Dothiorella

AAGTCGTAACAAGGTTTCCGTAGGTGAACCTGCGGAAGGATCATTACCGGGCTCAGGGGG

GGAAACCCCCCGAACTCCCAACCCTTGCTTACCTACCACGTTGCTTCGGCGGGTTCGCGC

CCGCCGGAGGTCAATCAAACTATTTTATACCATAGTCAGAGCGAATGCGAAAATAAGTAA

AAACTTTCAACAACGGATCTCTTGGTTCTG

>ASV41 SS|0.9900|KF274227|SH0911134.09FU;k:Fungi

AAGTCGTAACAAGGTTTCCGTAGGTGAACCTGCGGAAGGATCATTACCGAGTTAGGGTCC

TCTGGGCTCGATCTCCAACCCTGTGTCTAAATAACCACACGTTGCTTTGGCGGGCCCGCC

TGTAATGGGCCGCCGGGGGTGCCTTCGGCCCCCTGGTCAGCGCCTGCCAGTAGCCATCTC

AAACTCTTCTTAATCGTGACGTCTGAGTAAAAATTATAAATTAAACAAAACTTTCAACAA

CGGATCTCTT

>ASV42 SS|0.8700|MN592663|SH1107827.09FU;k:Fungi,p:Ascomycota,c:Lecanoromycetes,o:Teloschistales,f:Teloschistaceae

AAGTCGTAACAAGGTTTCCGTAGGTGAACCTGCGGAAGGATCATTATCGAGAGGGGGGCT

CCATGCCCCGGGGCTCTGTCCCCGTACCTTTTCACCCTGTGTGTATTTTCCCCCCGTTGC

TTTGGCGGGCCCCGGGTCTTCCCCCGGCGTTGGCCCCCTCGCGGGGTTCGCGAGCGCCCG

CCGAAGGCTCATCGAAACTCTGTTGATCAGTGCAGTCTGAGCGTACGAACAATAAATCAA

AACTTTCAAC

>ASV43 GS|99.6|AY667583|SH0954634.09FU;k:Fungi,p:Ascomycota,c:Lecanoromycetes,o:Caliciales,f:Caliciaceae,g:Buellia,s:Buellia frigida

AAGTCGTAACAAGGTTTCCGTAGGTGAACCTGCGGAAGGATCATTACCGAGAGACGGGGT

CGCTTCGGCCCCACTCTTCACCCGTGTCTACTTACCTTCGTTGCTTTGGCGGGCCTTCGG

GCTTGACCGTTCGACGCGGGTGGGAGGCTTTCGCCTCTCGCCTCGCGAGCGCCCGCCAAA

GGCCCTGTTAACTCTGTTTTAGTGTCTTCCGAGCAACCATGTAATAGTTAAAACTTTCAA

CAACGGATCT

>ASV44 SS|1.0000|JX036043|SH0954629.09FU;k:Fungi,p:Ascomycota,c:Lecanoromycetes,o:Caliciales,f:Caliciaceae,g:Buellia

AAGTCGTAACAAGGTTTCCGTAGGTGTCACGACCCGCCCGGTTCGCCTACATCGCCTCGG

AGCGACTATAAAGAAAGGTTTAGTCGGGCGCTCTTGCGTCCGGCCACGCGGCTATTCTGC

CCTGGGAGCAAACCAATCTCTTCGGAGGCTCGGGTGTAACCAGCAGCCCATACCTCTCTG

GCGGTACTGGGTTCACAGATCAAATGCCTGCGGCCACGCCTTAGTGGTTCTTATATGACC

GACCTCCGCC

>ASV45 SS|0.8200|MZ229889|SH0913114.09FU;k:Fungi,p:Ascomycota,c:Lecanoromycetes,o:Caliciales

AAGTCGTAACAAGGTTTCCGTAGGTGAACCTGCGGAAGGATCATTATCGAGAGACGGAGT

CTAACCGGCCCCACTCTTCACCCGTGTATACCTACCCTTTGTTGCTTTGGCGGGCGGTCG

GTCTTTACCGCCCGACGTCGGTCGGAAGGCTCGCGCCTTCCACTCACCGAACGCCCGCCA

GAGGCCCCCAAAAGCCGTCCATCTGTGTCCGAGCGAGTGAAGCAAATAGTTAAAAACTTT

CAACAACGGA

>ASV46 GS|99.2|AY667583|SH0954634.09FU;k:Fungi,p:Ascomycota,c:Lecanoromycetes,o:Caliciales,f:Caliciaceae,g:Buellia,s:Buellia frigida

AAGTCGTAACAAGGTTTCCGTAGGTGAACCTGCGGAAGGATCATTACCGAGAAACGGGGT

CGCTTGGGCCCCACTCTTCACCCGTGTCTACTTACCTTTGTTGCTTTGGCGGGCCTTCGG

GCTTGACCGTTCGACGCGGGTGGGAGGCTTTCGCCTCTCGCCTCGCGAGCGCCCGCCAAA

GGCCCTGTTAACTCTGTTTTAGTGTCTTCCGAGCAACCATGTAATAGTTAAAACTTTCAA

CAACGGATCT

>ASV47 SS|0.8100|KF823589|SH1091255.09FU;k:Fungi,p:Basidiomycota,c:Tremellomycetes,o:Tremellales

AAGTCGTAACAAGGTTTCCGTAGGTGAACCTGCGGAAGGATCATTAGTGATTGGCCTCCG

GGCTCTTTCGTTAATCCACACACCTCTGTGAACCTGTCGGCCTCCTGGCCTTCTTTTCCA

AACAATGTGTAACGAACGTAAGGCATACGATTAACCTAGTAAAACTTTCAACAACGGATC

TCTTGGCTCTC

>ASV48 GS|100.0|MW991423|SH0964445.09FU;k:Fungi,p:Ascomycota,c:Arthoniomycetes,o:Lichenostigmatales,f:Phaeococcomycetaceae,g:Antarctolichenia,s:Antarctolichenia onofrii

AAGTCGTAACAAGGTCTCCGTAGGTGAACCTGCGGAGGGATCATTAATGAGATAGGGTCT

TCACGGCCCGACCTCCAACCCAATGTCTACCATACCTCTGTTGCCTCGGCGGGCCGCCGG

CGTCCTTTGTTGGGCGCCGCCGTCGGCTTTAGGGCTTTCGAGCGCCCGCCGCAGGACCGA

TTAAACTCTTTTTAAAACAAGTCTTCTGAGTGGGAAATCAAATTTATTAAAACTTTTAAC

AACGGATCTC

>ASV49 SS|1.0000|KF274227|SH0911134.09FU;k:Fungi

AAGTCGTAACAAGGTTTCCGTAGGTGAACCTGCGGAAGGATCATTACCGAGTTAGGGTCC

TCTGGGCTCGATCTCCAACCCTGTGTCTAAATAACCACGTTGCTTTGGCGGGCCCGCCTG

TCATGGGCCGCCGGGGGTGCCTTCGGCCCCCTGGTCCGCGCCTGCCAGTAGCCATCTCAA

ACTCTTCTTAATCGTGACGTCTGAGTAAAAATTATAAATTAAACAAAACTTTCAACAACG

GATCTCTTGG

>ASV50 GS|98.0|MK208763|SH0942716.09FU;k:Fungi,p:Ascomycota,c:Lecanoromycetes,o:Lecanorales,f:Lecanoraceae,g:Lecidella

AAGTCGTAACAAGGTTTCCGTAGGTGAACCTGCGGAAGGATCATTAATGAGAGAGGGGCT

TCGCGCTCCCGGGGGCTCCGGCCCCCAACTCTTCACCCTCTGTCTACCTACCTTTGTTGC

TTTGGCGCGCCCTGGGGTTCCGCCCCACGCCGGCCTCAGGCCTTCGGCTTGGGCTGGTGA

GAGCCCGTCAGAGGCCCATTCTAATCCTTATATCAGTGATGTCCGAGTACAATTTTAATA

AATAAAACTT

>ASV51 GS|87.3|AF250779|SH0954634.09FU;k:Fungi,p:Ascomycota,c:Lecanoromycetes,o:Caliciales,f:Caliciaceae,g:Buellia,s:Buellia frigida

AAGTCGTAACAAGATTTTCGTAGATGAACTTGCGAAAGAATCATTATCGAGAGACGAGAT

CGCTTCGATCTCACTCTTCACTCGTATCTACTTATCTTTGTTGCTTTGACGAGTCTTCGA

GCTTGATCGTTCGACGCGAGTGGGAGGCTTTCGTCTCTCGTCTCGCGAGCGCTCGTCAAA

GGTCTTGTTAACTCTATTTTAATGTCTTTCGAGCAATCATGTAATAGTTAAAACTTTCAA

CAACGAATCT

>ASV52 GS|98.0|MZ243488|SH0916476.09FU;k:Fungi,p:Ascomycota,c:Lecanoromycetes,o:Caliciales,f:Physciaceae,g:Tetramelas,s:Tetramelas papillatus

AAGTCGTAACAAGGTTTCCGTAGGTGAACCTGCGGAAGGATCATTACCGAGAGACGGGGC

CTAATCGGCCCCACTCTTCACCCGTGTATACCTATCCTTTGTTGCTTTGGCGGGCCGTCG

AGCCTTACCGCTCGGCGCCAGTCGGAAGGCTAGCGTCCTCCGCCCGGCGAGCGTCCGCCA

GAGGCCTTCAATACTCCATCTCAGTGATGTCCGAGCAAACTAAGCAATAGTTAAAACTTT

CAACAACGGA

>ASV53 GS|98.9|KF309966|SH1084317.09FU;k:Fungi,p:Ascomycota,c:Dothideomycetes,o:Mycosphaerellales,f:Teratosphaeriaceae,g:Meristemomyces,s:Meristemomyces frigidus

AAGTCGTAACAAGGTCTCCGTAGGTGAACCTGCGGAGGGATCATTACTGAGTGAGGGTGC

TTGCGCCCGACCTCCAACCCCATGTTTTCCGACTCTGTTGCCTCGGGGGCGACCCGGCCC

TCTCGTGGGGCCCGGGGCCCTCGGTGGACCGCTCAACTCTGCATCTGTGCGTCTGAGTCA

ATATTTGAATTAATCAAAACTTTTAACAACGGATCTCTTGGTTCTG

>ASV54 SS|1.0000|GU184109|SH0954388.09FU;k:Fungi,p:Ascomycota,c:Lecanoromycetes,o:Acarosporales,f:Acarosporaceae

AAGTCGTAACAAGGTTTCCATGCGGAGGGACATCCATCTTACTCGATCTTCACATCTGTA

AGCAAACCACCTCTGAAGCCTTGAAATCATGAGTCTGCATCTTTGCAGGCGACACTAGTC

CGTTGCTGGGAGTCTCCTGGAGATAATCAGCAGCCACGATACTGGATTGTGGTTCACAGA

TCAAACACTAGTGGCCCGTTGGTGGGTTAAAATATGACCGGCTCCATCTGGAAATAGATG

GACGATTGCG

>ASV55 SS|0.9100|UDB0700338|SH1084495.09FU;k:Fungi,p:Ascomycota

AAGTCGTAACAAGGTCTCCGTAGGTGAACCTGCGGAGGGATCATTACCGAGTGAGGGCCT

CCGGGCTCGACCTCCAACCCTTTGTTGTACCAACCACAGTTGCCTCGGGGGCGACCCGGC

CTCCGCGTCGGGGCCCCCGGTGGACCCAACCAAACAACTCTGCGTCTTTGCGTCTGAGTA

TTAAAGTAAATCAATTAAAACTTTCAACAACGGATCTCTTGGTTCTG

>ASV56 SS|1.0000|MZ229889|SH0913114.09FU;k:Fungi

AAGTCGTAACAAGGTTTCCGTAGGTGAACCTGCGGAAGGATCATTATCGAGAGACGGAGT

CTAACCGGCCCCACTCTTCACCCGTGTATACCTACCCTTTGTTGCTTTGGCGGGCGGTCG

GTCTTTACCGCCCGACGTCGGTCGGAAGGCTCGCGCCTTCCACTCACCGAACGCCCGCCA

GAGGCCCCCAAAACCCGTCCATCTGTGTCCGAGCGAGTGAAGCAAATAGTTAAAAACTTT

CAACAACGGA

>ASV57 GS|100.0|FJ392867|SH1071027.09FU;k:Fungi,p:Ascomycota,c:Eurotiomycetes,o:Chaetothyriales,f:Trichomeriaceae,g:Knufia

AAGTCGTAACAAGGTTTCCGTAGGTGAACCTGCGGAAGGATCATTACCGAGTTAGGGTCT

CTTCGGAGCCCGAACCTCCCAACCCTTTGTCTAATTTACCTTGTCGTTGCTTCGGCGGAC

CGGTTGACCAACTGGTCTTGACCGCCGGGGGTCCCGTACCCCTGGAGAGCGTCCGCCGAC

GGCCTAACCACAAACTCTTGTACTAAACCATGTCGTCTGAATGTACTTGATATTAATCAA

AAAACAAAAC

>ASV58 GS|99.0|UDB03337334|SH0989105.09FU;k:Fungi,p:Ascomycota,c:Leotiomycetes,o:Helotiales

AAGTCGTAACAAGGTTTCCGTAGGTGAACCTGCGGAAGGATCATTACCGAGTTTGTGCCC

TCTGGGGTAGATCTCCCACCCTGTTTATTTTACTTCATGTTGCTTTGGCGGGTCGACTGT

CCAGGCCACCGGCGACTCGCTGGTGTGTGCCCGCCAAAGGCTCCTAAACCCGGATATGTA

ACCTGCCGTCCGAGTCCTATATAATACTTAAAACTTTCAACAACGGATCTCTTGGTTCTG

>ASV59 GS|99.2|AY667583|SH0954634.09FU;k:Fungi,p:Ascomycota,c:Lecanoromycetes,o:Caliciales,f:Caliciaceae,g:Buellia,s:Buellia frigida

AAGTCGTAACAAGGTTTCCGTAGGTGAACCTGCGGAAGGATCATTACCGAGAGACGAGGT

CGCTTCGGCCCCACTCTTCACCCGTGTCTACTTACCTTTGTTGCTTTGGCGGGCCTTCGG

GCTTGACCGTTCGACGCGGGTGGGAGGCTTTCGCCTCTCGCCTCGCGAGCGCCCGCCAAA

GGCCCTGTTAACTCTGTTTTAGTGTCTTCCGAGCAACCATGTAACAGTTAAAACTTTCAA

CAACGGATCT

>ASV60 GS|87.3|AF250779|SH0954634.09FU;k:Fungi,p:Ascomycota,c:Lecanoromycetes,o:Caliciales,f:Caliciaceae,g:Buellia,s:Buellia frigida

AAGTCGTAACAAGATTTTCGTAGATGAATTTGCGAAAGAATCATTATCGAGAGATGAGAT

CGCTTCGATCTCACTCTTTACTCGTGTCTACTTATCTTTGTTGCTTTGACGAGTCTTCGA

GCTTGATCGTTCGACGCGAGTGAGAGACTTTCGCTTGTCGTCTCGCGAGCGCTCGTCAAA

GACTTTGTTAACTCTGTTTTAGTGTCTTCCGAGCAACCATGTAATAGTTAAAACTTTCAA

CAACGGATCT

>ASV61 GS|98.8|UDB05295061|SH0991924.09FU;k:Fungi,p:Ascomycota,c:Dothideomycetes,o:Mycosphaerellales,f:Teratosphaeriaceae,g:Elasticomyces,s:Elasticomyces elasticus

AAGTCGTAACAAGGTCTCCGTAGGTGAACCTGCGGAGGGATCATTACCGAGTGAGGGCCC

CCGGGCCCGACCTCCAACCCCATGTTAACCGACCCTGTTGCCTCGGGGGCGACCCGGCCT

CGTGCCGGGGCCCCCGATGGACCTTCCAACACTGCATCTTTGCGTCCGAGTCAATATTTG

AATAAATCAAAACTTTCAACAACGGATCTCTTGGTTCTG

>ASV62 SS|0.9000|MN592663|SH1107827.09FU;k:Fungi,p:Ascomycota,c:Lecanoromycetes,o:Teloschistales,f:Teloschistaceae

AAGTCGTAACAAGGTTTCCGTAGGTGAACCTGCGGAAGGATCATTATCGAGAGGGGGGTT

CCATGCCCCGGGGCTCTGTCCCCGTACCTTTTCACCCTGTGTGTATTTTTCCCCCGTTGC

TTTGGCGGGCCCCGGGTCTTCCCCCGGCGTTGGCCCCCTCGCGGGGTTCGCGAGCGCCCG

CCGAAGGCTCATCGAAACTCTGTTGGTCAGTGCAGTCTGAGCGTACGAATAATAAATCAA

AACTTTCAAC

>ASV63 SS|0.9900|MF976578|SH1193052.09FU;k:Fungi

AAGTCGTAACAAGGTTTCCGTAGGTGAACCTGCGGAAGGATCATTACCGAGTCAGGGTCC

CCCCGGGGGCCCGACCTCCAACCCGCTGCCTACAAACCCCTGTTGCCTCGGCGGCTCCGG

GCTCCGGCCCCCTGTCGGTGGACCATTGAAAGCCCGTTCGAACCCCATTCTGAGCCCGAA

ATCAAGTTCACAAAACTTTCAACAACGGATCTCTTGGTTCCC

>ASV64 GS|0.0|None;No hit

AAGTCGTAACAAGGTTTTCGTAGGTGAACCTGCGGAAGGATCATTACCAGATGAGGCCTC

CGGGCCTTTTTCTCCTTACCACGTGCACCGAGTTGTCGGGAGATGCTTGTTTCCCGACCA

ATTCTTCACGCTTGTTTTGTTGTCTGAGTCGAATGACCATAATTGAAAAGAAAACTATGA

ACAACGGATCTCTAGGCTCTT

>ASV65 GS|100.0|MK958550|SH1072914.09FU;k:Fungi,p:Ascomycota,c:Sordariomycetes,o:Hypocreales,g:Acremonium

AAGTCGTAACAAGGTCTCCGTTGGTGAACCAGCGGAGGGATCATTACAGAGTGTAAAAAC

TCCCAAAACCCCTGCGAACATACCAGTCGTTGCCTCGGCGGGACCGCCCCGGGCGCCATC

GCGTGCCCCGGAACCAGGCGCCCGCCGGGGGACTCAAACTCTTGTTTTCACCCCAGTGGC

ATTATCTGAGTGGGCTTTAAGCAAACAAATGAATCAAAACTTTCAACAACGGATCTCTTG

GTTCTG

>ASV66 GS|84.8|AY667583|SH0954634.09FU;k:Fungi,p:Ascomycota,c:Lecanoromycetes,o:Caliciales,f:Caliciaceae,g:Buellia,s:Buellia frigida

AAGTCGTAACAAGGTTTTCGTAGATGAATTTGCGAAAGAATCATTATCGAGAGACGAGAT

CGCTTCGATCTCACTCTTCACTCGTGTCTACTTACCTTTGTTGCTTTGGCGAGTCTTTGA

ACTTGATCGTTCGATGCGAGTGAAAGACTTTCGTCTCTCGTTTCGCGAACGCTCGTCAAA

GGTCTTGTTAACTTTGTTTTAGTGTCTTTTGAGCAATCATGTAATAATTAAAACTTTCAA

CAACGAATCT

>ASV67 GS|99.0|UDB01588125|SH0913118.09FU;k:Fungi,p:Ascomycota,c:Lecanoromycetes,o:Caliciales,f:Caliciaceae

AAGTCGTAACAAGGTTTCCGTAGGTGCTGCAGCCTCCCAAGGTTCGCCTACCGAAGCCCC

GGGCGACTCTAAACAATCCGCCCAGTCAGTCGCCACTGGCGGCTGGCCACGCAGCCATTC

TGGCTTGGAAGCCCCCCGAGGAGCCCCCTCGGCGGCCACCAGCAGCTCCGGCACCGTCGA

GTGCCGGAGTCCACAGATCAAATACCTGCGGCCACATCTGTGGTTCTGATATGACCGACC

GCCGCCCGCA

>ASV68 SS|1.0000|MG982528|SH1003748.09FU;k:Fungi

AAGTCGTAACAAGGTTTCCGTAGGTGAACCTGCGGAAGGATCATTAGTGATTCGGCCCTC

ACGGGTCTATAAAAGACACCTCTGTGAACCTGTCGGCCTCCGGGCCCACCTGCAAACACT

GTGTGACGAGCGTCGATGTATCATAAGCATAATAAAACTTTCAACAACGGATCTCTTGGC

TCTC

>ASV69 SS|0.8800|KF823589|SH1091255.09FU;k:Fungi,p:Basidiomycota

AAGTCGTAACAAGGTTTCCGTAGGTGAACCTGCGGAAGGATCATTAGGGATTTGGTCTCC

GGACCTTTTTCATATCCATAATACCCCTGTGAACTGTCGGTCCTCGGGCCAGCGTTTCCA

AACCATGTGTAATGAACGTGAATGTGTATGAAACCCTAGTAAAACTTTCAACAACGGATC

TCTTGGCTCTC

>ASV70 SS|0.9900|AY667583|SH0954634.09FU;k:Fungi,p:Ascomycota,c:Lecanoromycetes,o:Caliciales,f:Caliciaceae,g:Buellia,s:Buellia frigida

AAGTCGTAACAAGGTTTTCGTAGGTGAATCTGCGGAAGGATCATTATCGAGAGACGAGGT

CGCTTCGACCTCACTCTTCACTCGTGTCTACTTACCTTTGTTGCTTTGGCGGGCCTTCGG

GCTTGATCGTTCGACGCGGGTGGGAGGCTTTCGTCTCTCGCCTCGCGAGCGCTCGTCAAA

GGCCCTCTTAACTTTGTTTTAGTGTCTTTCGAGCAATTATGTAATAGTTAAAACTTTCAA

CAATGAATCT

>ASV71 SS|0.9700|AF250779|SH0954634.09FU;k:Fungi,p:Ascomycota,c:Lecanoromycetes,o:Caliciales,f:Caliciaceae,g:Buellia,s:Buellia frigida

AAGTCGTAACAAGATTTCCATAGATGAACCTACGAAAGGATCATTATCGAGAGACGAGAT

CGCTTCGACCCCACTCTTCACTCGTGTCTACTTACCTTTGTTGCTTTGACGAGCCTTCGA

GCTTGATCGTTCGATGCGAGTGGAAGGCTTTCGCCTCTCGCCTCGCGAGCGCTCGCCAAA

GGCCCTGTTAACTCTGTTTTAGTGTCTTTCGAGCAATTATGTAATAGTTAAAACTTTCAA

CAACGAATCT

>ASV72 SS|1.0000|KY103451|SH1020764.09FU;k:Fungi

AAGTCGTAACAAGGTTTCCGTAGGTGAACCTGCGGAAGGATCATTAGTGATTTTGACCTC

TGGGTCTAACAAATCTCATCCACATACCCCCGTGAACCGTTTGAGCCTTCGGGTCTATCT

TAACAAACATCAGTGTAACGGATGTCAAATCTTAATAAAATTAATAAAACTTTCAACAAC

GGATCTCTTGGCTCTC

>ASV73 SS|0.9900|AY667583|SH0954634.09FU;k:Fungi,p:Ascomycota,c:Lecanoromycetes,o:Caliciales,f:Caliciaceae,g:Buellia,s:Buellia frigida

AAGTCGTAACAAGGTTTCCGTAGGTGAACCTGCGAAAGGATCATTATCGAGAGACGGGGT

CGCTTCGGCCCCACTCTTCACTCGTGTCTACTTATCTTTGTTGCTTTGGCGGGCCTTTGG

GCTTGATCGTTCGACGCGAGTGGGAGGCTTTCGTCTCTCGCCTCGCGAGCGCCCGCCAAA

GGTCCTGTTAACTCTGTTTTAGTGTCTTTCGAGCAATCATGTAATAGTTAAAACTTTCAA

CAACGAATCT

>ASV74 SS|1.0000|GU184109|SH0954388.09FU;k:Fungi,p:Ascomycota,c:Lecanoromycetes,o:Acarosporales,f:Acarosporaceae

AAGTCGTAACAAGGTTTCCATGCGGAGGGACATTCATCTTACTCGATCTTCACATCTGTA

AGCAAACCACCTCTGAAGCCTTGAAATCATGAGTCTGCATCTTTGCAGGCGACACTAGTC

CGTTGCTGGGAGTCTCCTGGAGATAATCAGCAGCCATGATACTGGATTGTGGTTCACAGA

TCAAACACTAGTGGCCCGTTGGTGGGTTAAAATATGACCGGCTCCATCTGGAAATAGATG

GACGATTGCG

>ASV75 GS|82.6|UDB01720327|SH1085256.09FU;k:Rhizaria,p:Cercozoa

AAGTCGTAACAAGGTCTTCGTAGGTGAACCTGCGAAGGGATCATTAACACGTTCCAAACC

TCATTATCAACCCATCTGTGAACTCTTGTTACCGCCGATATGACTGACTACAATGTGTAT

GGCGCATACGATCGACACTACGATGTTGCATAGTCTAAACCACTGTGCATGGTCCAGTAG

TATGCCTCCTGTGTCTTTACCGACAGCTATCCTTGTACGTTGGTCCGGCCCCCATATATG

CGCTTCGACA

>ASV76 GS|100.0|UDB02347716|SH1071027.09FU;k:Fungi,p:Ascomycota,c:Eurotiomycetes,o:Chaetothyriales,f:Trichomeriaceae,g:Knufia

AAGTCGTAACAAGGTTTCCGTAGGTGAACCTGCGGAAGGATCATTACCGAGTTAGGGTCT

CTTCGGAGCCCGAACCTCCCAACCCTTTGTCTAATTTACCTTGTCGTTGCTTCGGCGGAC

CGGTTGACCAACTGGTCTTGACCGCCGGGGGTCCCGTACCCCTGGAGAGCGTCCGCCGAC

GGCCCAACCACAAACTCTTGTACTAAACCATGTCGTCTGAATGTACTTGATATTAATCAA

AAAACAAAAC

>ASV77 GS|99.0|KF516956|SH0998103.09FU;k:Fungi,p:Ascomycota,c:Lecanoromycetes,o:Umbilicariales,f:Umbilicariaceae,g:Umbilicaria

AAGTCGTAACAAGGTTTCCGTAGGTGAACCTGCGGAAGGATCATTAATGAGATAGGGTCC

TTCGGGGCCCGAACCTCCAACCCTCTGTGTATCTTACCTTTGTTGCTTTGGCGGGCCTTT

GCTCACGCAGCGTACCCCCGGGTGCGAGTGCCCGCCCGAGGCCATTCAAATTCCGATTAT

CAGTGACGTCTGAGTACTATATAATAGTTAAAACTTTCAACAACGGATCTCTTGGTTCTG

>ASV78 GS|90.2|AF250779|SH0954634.09FU;k:Fungi,p:Ascomycota,c:Lecanoromycetes,o:Caliciales,f:Caliciaceae,g:Buellia,s:Buellia frigida

AAGTCGTAACAAGATTTTCGTAGATGAATTTGCGAAAGAATCATTATCGAGAGACGAGAT

CGCTTCGATCCCACTCTTCACTCGTGTCTACTTACCTTTGTTGCTTTGGCGAGCCTTCGA

GCTTGATCGTTCGACGCGAATGAGAGACTTTCGCCTCTCGTCTCGCGAGCGCTCGCCAAA

GACCGTGTTAACTCTATTTTAGTGTCTTCCGAGCAATCATGTAATAGTTAAAACTTTCAA

CAACGAATCT

>ASV79 SS|0.9500|AF250779|SH0954634.09FU;k:Fungi,p:Ascomycota,c:Lecanoromycetes,o:Caliciales,f:Caliciaceae,g:Buellia,s:Buellia frigida

AAGTCGTAACAAGGTTTTCGTAGGTGAACTTGCGAAAAAATCATTATCGAGAGACGAGGT

CGCTTCGGCTCCACTCTTCACTCGTGTCTACTTATCTTTGTTGCTTTGGCGAGTCTTCGA

GCTTGATCGTTCGACGCGAGTGGGAGGCTTTCGTCTCTCGCCTCGCGAGCGCTCGTCAAA

GATCCTGTTAACTCTGTTTTAGTGTCTTTCGAGCAATCATGTAATAGTTAAAACTTTCAA

CAACGAATCT

>ASV80 SS|1.0000|AF250779|SH0954634.09FU;k:Fungi,p:Ascomycota,c:Lecanoromycetes,o:Caliciales,f:Caliciaceae,g:Buellia,s:Buellia frigida

AAGTCGTAACAAGATTTCCGTAGGTGAATTTGCGAAAGGATCATTATCGAGAGATGGGGT

CGCTTCGGCCCCACTCTTCACTCGTGTCTACTTACCTTTGTTGCTTTGACGGGCCTTCGA

GCTTGATCGTTCGACGCGGGTGAGAGGCTTTCGTCTCTCGTCTCGCGAGCGCTCGTCAAA

GGCCCTGTTAACTCTGTTTTAGTGTCTTTCGAGCAATCATGTAATAGTTAAAACTTTCAA

CAACGAATCT

>ASV81 SS|1.0000|AF250779|SH0954634.09FU;k:Fungi,p:Ascomycota,c:Lecanoromycetes,o:Caliciales,f:Caliciaceae,g:Buellia,s:Buellia frigida

AAGTCGTAACAAGATTTTCGTAGATGAACTTGCGAAAGAATCATTATCGAGAGACGAGGT

CGCTTCGATTTCACTCTTCACTCGTGTCTACTTACCTTTGTTGCTTTGACGGGCCTTCGA

ACTTGATCGTTCGACGCGGGTGAGAGGCTTTCGCCTCTCGCCTCGCGAGCGCTCGCCAAA

GGCCCTGTTAACTCTGTTTTAGTGTCTTTCGAGCAATCATGTAATAGTTAAAACTTTCAA

CAACGAATCT

>ASV82 GS|100.0|JX036088|SH0954084.09FU;k:Fungi,p:Ascomycota,c:Lecanoromycetes,o:Acarosporales,f:Acarosporaceae,g:Acarospora

AAGTCGTAACAAGGTTTCCTTCCAGGCGGTGCATCCGCTGAAGCCTCCCGCAGCGACTAT

AAACAAAACGCGTCAGTCCGCCTCGCGGGCGACACCACGATCCTACTGGGACTCCTGCAT

GCATCTTTGATCATGCAGGTACCAGTGGCCTGTGCAACAGGTTCACAGATCAAACAGTGG

TGGCCATTGTGGTTAAGATATGACCGGTCTCCGCTGTAACCAGCCGGAGAGAACACGTTC

CGTAGGTGAA

>ASV83 SS|0.8600|FJ265755|SH1071004.09FU;k:Fungi,p:Ascomycota

AAGTCGTAACAAGGTTTCCGTAGGTGAACCTGCGGAAGGATCATTACCGAGTTAGGGTTC

GCTCGTCGAGCCCGACCTCCCAACCCTTTGTTTATTATACCTTGTCGTTGCTTCGGCGGA

CCGGTCTCTCGACCGCCGGGGGTTATCGCCCCTGGAACGTGTCCGCCGACGGCCCAACCA

CAAACTCTTGCCCCAAACCATGTCGCCTGAATTTACTTGATTAAAATCAAAAACAAAACT

TTCAACAACG

>ASV84 GS|99.6|MN103156|SH0903303.09FU;k:Fungi,p:Ascomycota,c:Lecanoromycetes,o:Caliciales,f:Physciaceae,g:Physcia,s:Physcia caesia

AAGTCGTAACAAGGTTTCCGTAGGTGAACCTGCGGAAGGATCATTACCGAGAGACGGGGT

CTAACCGGCCCCACCTCTTCATCCGTGTCTACCAACCAATGTTGCTTTGGCGAGCCTCGG

GCTTGTTCCGGGGAGTTTACGCTCCTTCGCCCGCCAAAGGCCCATACAAAACCTGTCATT

AGTGTCGTCCGAGTAAACATTTAATAGTTAAAACTTTCAACAACGGATCTCTTGGTTCTG

>ASV85 SS|1.0000|AY667583|SH0954634.09FU;k:Fungi,p:Ascomycota,c:Lecanoromycetes,o:Caliciales,f:Caliciaceae,g:Buellia,s:Buellia frigida

AAGTCGTAACAAGGTTTTCGTAGGTGAACCTGCGGAAGGATCATTATCGAGAGACGGGGT

CGCTTCGGCCTCACTCTTCACCCGTGTCTACTTACCTTTGTTGCTTTGGCGGGCCTTCGG

GCTTGATCGTTCGACGCGGGTGGGAGGCTTTCGTCTCTCGTCTCGCGAGCGCCCGCCAAA

GGCCCTGTTAACTTTGTTTTAGTGTCTTTCGAGCAACCATGTAATAGTTAAAACTTTCAA

CAACGGATCT

>ASV86 GS|99.0|UDB01588125|SH0913118.09FU;k:Fungi,p:Ascomycota,c:Lecanoromycetes,o:Caliciales,f:Caliciaceae

AAGTCGTAACAAGGTTTCCGTAGGTGCTGCAGCCTCCCAAGGTTCGCCTACCGAAGCCCC

GGGCGACTCTAAACAATCCGCCCAGTCAGTCGCCACCGGCGGCTGGCCACGCAGCCATTC

TGGCTTGGAAGCCCCCCGAGGAGCCCCCTCGGCGGCTACCAGCAGCTCCGGCACCGTCGA

GTGCCGGAGTCCACAGATCAAATACCTGCGGCCACATCTGTGGTTCTGATATGACCGACC

GCCGCCCGCA

>ASV87 GS|0.0|None;No hit

AAGTCGTAACAAGGTTTTCGTAGGTGAACCTGCGGAAGGATCATTACCAGATGAGGCCTC

CGGGCCTTTTTCTCCTTACCACGTGCACCGAGTTGTCGGGAGACGCTTGTTTCCCGACCA

ATTCTTCACGCTTGTTTTGTTGTCTGAGTCGAATGACCATAATTGAAAAGAAAACTATGA

ACAACGGATCTCTAGGCTCTT

>ASV88 GS|91.2|AF250779|SH0954634.09FU;k:Fungi,p:Ascomycota,c:Lecanoromycetes,o:Caliciales,f:Caliciaceae,g:Buellia,s:Buellia frigida

AAGTCGTAACAAGGTTTTCGTAGATGAATCTACGAAAGAATCATTATCGAGAGACGAGGT

CGCTTCGACCCCACTCTTCACTCGTGTCTACTTATCTTTGTTGCTTTGACGGGCCTTCGG

GCTTGATCATTCGACGCGAGTGGGAGGCTTTCGTCTCTCGTCTCGCGAGCGCTCGTCAAA

GGCCCTGTTAACTCTGTTTTAATGTCTTCCAAGCAATCATGTAATAGTTAAAACTTTTAA

CAACGAATCT

>ASV89 GS|99.0|AF250779|SH0954634.09FU;k:Fungi,p:Ascomycota,c:Lecanoromycetes,o:Caliciales,f:Caliciaceae,g:Buellia,s:Buellia frigida

AAGTCGTAACAAGGTTTTCGTAGGTGAACCTGCGGAAGGATCATTATCGAGAGACGGGGT

CGCTTCGGCCCCACTCTTCACCCGTGTCTACTTACCTTTGTTGCTTTGGCGGGCCTTCGG

GCTTGACCGTTCGACGCGGGTGGGAGGCTTTCGCCTGTCGCCTCGCGAGCGCCCGCCAAA

GGCCCTGTTAACTCTGTTTTAGTGTCTTCCGAGCAACCATGTAATAGTTAAAACTTTCAA

CAACGGATCT

>ASV90 SS|0.9900|AF250779|SH0954634.09FU;k:Fungi,p:Ascomycota,c:Lecanoromycetes,o:Caliciales,f:Caliciaceae,g:Buellia,s:Buellia frigida

AAGTCGTAACAAGGTTTCCGTAGGTGAATTTGCGAAAGGATCATTATCGAGAGACGAGGT

CGCTTCGACCCCACTCTTCACTCGTGTCTACTTATCTTTGTTGCTTTGGCGGGTCTTCGG

GCTTGATCGTTCGACGCGGGTGGGAGGCTTTCGCCTCTCGCCTCGCGAGCGCTCGCCAAA

GGCCTTGTTAACTCTGTTTTAGTGTCTTTCGAGCAATCATGTAATAGTTAAAACTTTCAA

CAACGGATCT

>ASV91 GS|100.0|MK970660|SH0942719.09FU;k:Fungi,p:Ascomycota,c:Lecanoromycetes,o:Lecanorales,f:Lecanoraceae,g:Lecanora,s:Lecanora fuscobrunnea

AAGTCGTAACAAGGTTTCCGTAGGTGAACCTGCGGAAGGATCATTATCGAGAGGGGTCCC

CGGACTCCGGGGGCTTCGGCCCCCTACTCTTCACCCTATGTCTACACACCTTTGTTGCTT

TGGCGGGCCTCGGGTTCGCCCCGTACCGGTCGTGGGCTTCCATACCCCGGCCGTCCGTGC

CCGTCAGAGGCCCATGAACCCTCGTTTATCAGTGTCGTCCGAGTCCAACCATAATAGTAA

AAACTTTCAA

>ASV92 GS|100.0|UDB01604532|SH1277830.09FU;k:Fungi,p:Ascomycota,c:Candelariomycetes,o:Candelariales,f:Candelariaceae,g:Candelariella,s:Candelariella flava

AAGTCGTAACAAGGTTTCCGTAGGTGTAAGTAATCCAACGTCCCAAACATTCACCATCCA

ATGCGTGATCATGCTAACATGTGATTTAGGAACCTGCGGAAGGATCATTAAAGAGCAAGG

GTCTTCTAGGCCCGACCTCCAACCCTTTGTATATCTACCTCTGTTGCTTTGGCGGGCCCG

TTGGGGTGACCCACCGCCGGCTTTCAGCCGGTGAGTGCCCGTCAGAGTCCACGTAAACTC

TGTCTATCTA

>ASV93 SS|1.0000|AY667583|SH0954634.09FU;k:Fungi,p:Ascomycota,c:Lecanoromycetes,o:Caliciales,f:Caliciaceae,g:Buellia,s:Buellia frigida

AAGTCGTAACAAGATTTCCGTAGATGAACCTGCGAAAGGATCATTATCGAGAGACGGGGT

CGCTTCGACCTCACTCTTCACTCGTGTCTACTTATCTTTGTTGCTTTGGCGGGCCTTCGG

GCTTGATCGTTCGACGCGGGTGGGAGGCTTTCGCCTCTCGCCTCGCGAGCGCTCGTCAAA

GATCTTGTTAACTTTGTTTTAGTGTCTTTCGAGCAATCATGTAATAGTTAAAACTTTCAA

CAACGGATCT

>ASV94 GS|98.0|AF250779|SH0954634.09FU;k:Fungi,p:Ascomycota,c:Lecanoromycetes,o:Caliciales,f:Caliciaceae,g:Buellia,s:Buellia frigida

AAGTCGTAACAAGGTTTTCGTAGGTGAACCTGCGAAAGGATCATTATCGAGAGACGGGGT

CGCTTCGGCCCCACTCTTCACCCGTGTCTACTTACCTTTGTTGCTTTGGCGGGCCTTCGG

GCTTGATCGTTCGACGCGGGTGGGAGGCTTTCGCCTCTCGCCTCGCGAGCGCTCGCCAAA

GGCTCTGTTAACTCTGTTTTAGTGTCTTCCGAGCAACCATGTAATAGTTAAAACTTTCAA

CAACGGATCT

>ASV95 GS|99.6|AY667583|SH0954634.09FU;k:Fungi,p:Ascomycota,c:Lecanoromycetes,o:Caliciales,f:Caliciaceae,g:Buellia,s:Buellia frigida

AAGTCGTAACAAGGTTTCCGTAGGTGAACCTGCGGAAGGATCATTACCGAGAGACGGGGT

CGCTTCGGCCCCACTCTTCACCCGTGTCTACTTACCTTTGTTGCTTTGGCGGGCCTTCGG

GCCTGACCGTTCGACGCGGGTGGGAGGCTTTCGCCTCTCGCCTCGCGAGCGCCCGCCAAA

GGCCCTGTTAACTCTGTTTTAGTGTCTTCCGAGCAACCATGTAATAGTTAAAACTTTCAA

CAACGGATCT

>ASV96 GS|99.6|AY667583|SH0954634.09FU;k:Fungi,p:Ascomycota,c:Lecanoromycetes,o:Caliciales,f:Caliciaceae,g:Buellia,s:Buellia frigida

AAGTCGTAACAAGGTTTCCGTAGGTGAACCTGCGGAAGGATCATTACCGAGAGACGGGGT

CGCTTCGGCCCCACTCTTCACCCGTGTCTACTTACCTTTGTTGCTTTGGCGGGCCTTCGG

GCTTGACCGTTCGACGCGGGTGGGAGGCTTTCGCCTCTCGCCGCGCGAGCGCCCGCCAAA

GGCCCTGTTAACTCTGTTTTAGTGTCTTCCGAGCAACCATGTAATAGTTAAAACTTTCAA

CAACGGATCT

>ASV97 GS|100.0|UDB03378640|SH0910872.09FU;k:Fungi,p:Ascomycota,c:Eurotiomycetes,o:Chaetothyriales,f:Herpotrichiellaceae

AAGTCGTAACAAGGTTTCCGTAGGTGAACCTGCGGAAGGATCATTATCGAGTTAGGGTCT

TTACTGGCCCGATCTCCAACCCTGTGTCTATAATACCATGTTGCTTTGGCGGGCCCGCCT

TTAACGGGCCGCCGAGGGTCTTCGGACCCTTGGTCAGTGCCCGCCAGTAGCCAAATTAAA

TTCTTCTTAACTGTGTCGTCTGAGTAAATATTTAAAATAAACAAAACTTTCAACAACGGA

TCTCTTGGTT

>ASV98 SS|0.9600|OK576250|SH1225643.09FU;k:Fungi

AAGTCGTAACAAGGTTTCCGTAGGTGAACCTGCGGAAGGATCATTACCGAGCTAGGGCCT

TCTAGGTCTGACCTCCCACCCTATGTTTACCAGCTCCTTGTTGCTTCGGCGGACCGTTGG

GGTCAAACCCGCCGCAGGCTTTCGGGCTTGTGAGCGTCCGTCGGAGGATACTTTTAACGC

GTTTAATTCATGTGGTCTGAGTGGGTATTTAATCACCTTAAAACTTTCAACAACGGATCT

CTTGGTTCTG

>ASV99 SS|0.9600|JN885566|SH1084447.09FU;k:Fungi,p:Ascomycota,c:Dothideomycetes

AAGTCGTAACAAGGTCTCCGTAGGTGAACCTGCGGAGGGATCATTACCGAGCGAGGGCCT

CCGGGTCCGACCTCCCACCCCATGTTATCCGACCACTGTTGCCTCGGGGGCGACCCGGCC

TTCGGGCGTCGGGGCCCCCGGCGGACCCGTAACCCTGCATCTGTGCGTCCGAGTCAAACG

ATTGAATCGATCAAAACTTTCAACAACGGATCTCTTGGTTCTG

>ASV100 GS|100.0|MF138060|SH0954356.09FU;k:Fungi,p:Ascomycota,c:Lecanoromycetes,o:Acarosporales,f:Acarosporaceae,g:Acarospora,s:Acarospora gwynnii

AAGTCGTAACAAGGTTTCCGTAGGTGAACCTGCGGAAGGATCATTACAGAGTTAGGGTCT

TTCCAGGCCCGATCTCCAACCCTATGTGTACTACCTTTGTTGCTTTGGCGGGCCCGTTGG

GGTGACCCACCGGTGGCCTTCTGGCTCCCGAGTGCCCGTCAGAGACCCATCAAAACCTGT

TAATTGTGTCGTCTGAGTACCATTTTAATAATTAAAACTTTCAACAACGGATCTCTTGGT

TCTG

>ASV101 SS|0.9300|GU073019|SH1194723.09FU;k:Fungi

AAGTCGTAACAAGGTTTCCGTAGGTGAACCTGCGGAAGGATCATTAATGAGCAAGTAGGC

CCGCCTTTACTAGGGTGGGTCTCCTACCTCTAAACCCCATATCTACCGACCTCATCATAA

ACTCAGTACAAACGAAAAGTCGTCCCTAGCGGGGCGCAGCCAATCAAATGTACAAAAATC

AAAACTTTCAACAACGGATCTCTTGGTTCTG

>ASV102 GS|99.6|MK782318|SH1188820.09FU;k:Fungi,p:Basidiomycota,c:Tremellomycetes,o:Filobasidiales,f:Filobasidiaceae,g:Naganishia,s:Naganishia friedmannii

AAGTCGTAACAAGGTTTCCGTAGGTGAACCTGCGGAAGGATCATTAATGAATACAGATGC

CTGTCGAGCTTGCTCACGGGCTTTCTATCATATCCATAACACCTGTGCACTTGTTGGATG

TTCTAGAGACTTAGAGTTAAACCTGCAGTCAATAGTCATCCACTTACACTAAACAATAAT

GTAACAAATGTAGTCTTATTATAACAAAATAAAACTTTCAACAACGGATCTCTTGGCTCT

C

>ASV103 GS|100.0|UDB01571356|SH0913113.09FU;k:Fungi,p:Ascomycota,c:Lecanoromycetes,o:Caliciales,f:Caliciaceae,g:Buellia

AAGTCGTAACAAGGTTTCCGTAGGTGAACCTGCGGAAGGATCATTACCGAGAGACGGGGT

CCAACCGGCCCCACTCTTCACCCGTGCCTACCTACCTATGTTGCTTTGGCGGGCCTCCTG

CTCGGCGTCGGTCCGGGGCCTTTCGGGCTTCTTCCCGGCGAGTGCCCGCCGAAGGCTCTG

CAAACTCTTGTTTCGTGTCGTCCGAGTACAAACCTAATAGTTAAAACTTTCAACAACGGA

TCTCTTGGTT

>ASV104 GS|90.2|AF250779|SH0954634.09FU;k:Fungi,p:Ascomycota,c:Lecanoromycetes,o:Caliciales,f:Caliciaceae,g:Buellia,s:Buellia frigida

AAGTCGTAACAAGATTTTCGTAGATGAATCTACGAAAGAATCATTATCGAGAGACGAGGT

CGCTTCGATCTCACTCTTCACTCGTGTCTACTTATCTTTGTTGCTTTGGCGGGTCTTCGA

GCTTGATCGTTCGACGCGAGTGAGAGACTTTCGCCTCTCGTCTCGCGAGCGCTCGTCAAA

GGTCTTGTTAACTCTGTTTTAGTGTCTTCCGAGCAATCATGTAATAGTTAAAACTTTCAA

CAACGAATCT

>ASV105 SS|0.9200|AY667583|SH0954634.09FU;k:Fungi,p:Ascomycota,c:Lecanoromycetes,o:Caliciales,f:Caliciaceae,g:Buellia,s:Buellia frigida

AAGTCGTAACAAGGTTTTCGTAGGTGAATCTGCGAAAGGATCATTATCGAGAGACGAGGT

CGCTTCGGTCTCACACTCTTCACCCGTGTCTACTTATCTTTGTTGCTTTGGCGGGCCTTC

GGGCTTGATCGTTCGACGCGAGTGGGAGGCTTTCGTCTCTCGCCTCGCGAGCGCTCGTCA

AAGGCTTTGTTAACTCTGTTTTAGTGTCTTCCGAGCAATCATGTAATATTTAAAACTTTC

AACAATGGAT

>ASV106 GS|85.4|JX036103|SH1123214.09FU;k:Fungi,p:Ascomycota,c:Lecanoromycetes,o:Lecanorales,f:Catillariaceae,g:Austrolecia

AAGTCGTAACAAGGTTTCCGTAGGTAGCATGTTTGCCTTCGGAGCCCCCGCAGCGACCTT

AAATAAATGCTCTAGTCGGTCCCCTCCAGGGCCGGCGACGCTATCAATCTGTGCTGGGAG

CGCCCTCTGGGGTGCGATCAGCAGCTCTCCCCCCCGGGTATAGAGTTCACAGATCAAACG

ATAGCGGCCGCCCGGTGCGGTTCAGATATGATCGGCCTCAGCTGCACACCGGCTGGCGAC

TTTGCGGAAC

>ASV107 GS|86.3|AF250779|SH0954634.09FU;k:Fungi,p:Ascomycota,c:Lecanoromycetes,o:Caliciales,f:Caliciaceae,g:Buellia,s:Buellia frigida

AAGTCGTAACAAGATTTTCGTAGATAAATCTGCGAAAGAATCATTATCGAGAGACGAGAT

CGCTTCGATCTCACTCTTCACTCGTATCTACTTATCTTTGTTGCTTTGACGAGTCTTCGA

GCGCGATCGTTCGACGCGAGTGAGAGACTTTCGTCTCTCGTCTCGCGAGCGCTCGCCAAA

GACTCTGTTAACTTTGTTTTAGTGTCTTTCGAGCAATCATGTAATAGTTAAAACTTTCAA

CAACGAATCT

>ASV108 GS|97.9|UDB01571642|SH1041881.09FU;k:Fungi,p:Basidiomycota,c:Tremellomycetes,o:Tremellales,f:Bulleraceae

AAGTCGTAACAAGGTTTCCGTAGGTGAACCTGCGGAAGGATCATTAATGTAAACCCCTTG

TGGGGAAATACAAATCCACATACCTCTGTGAACCGTTGACCTCCGGGTCGTCTTCACAAA

CATCAGTGTAACGAACGTATACAAACATAAACAAAACAAAACTTTCAACAACGGATCTCT

TGGCTCTC

>ASV109 SS|1.0000|FJ265755|SH1071004.09FU;k:Fungi

AAGTCGTAACAAGGTTTCCGTAGGTGAACCTGCGGAAGGATCATTACCGAGTTAGGGTTC

GCTCGTCGAGCCCGACCTCCCAACCCTTTGTTTATCATACCTTGTCGTTGCTTCGGCGGA

CCGGTCTCTCGACCGCCGGGGGTTATCGCCCCTGGAACGCGTCCGCCGACGGCCCAACCA

CAAACTCTTGCCCCAAACCATGTCGTCTGAATTTACTTGATTAAAATCAAAAACAAAACT

TTCAACAACG

>ASV110 GS|99.0|KF823589|SH1091255.09FU;k:Fungi,p:Basidiomycota,c:Tremellomycetes,o:Tremellales,f:Sirobasidiaceae

AAGTCGTAACAAGGTTTCCGTAGGTGAACCTGCGGAAGGATCATTAGTGATTTGGCCTCC

GGGCCTTCAATCCCATCCTCATACCTCTGTGAACCAGTTGGGCCCTCGGGCCTACCCTTT

CAAACACTGTGTAACGAACGTGATGTATATTATAAACCTAATAAAACTTTCAACAACGGA

TCTCTTGGCTCTC

>ASV111 GS|98.4|KF823589|SH1091255.09FU;k:Fungi,p:Basidiomycota,c:Tremellomycetes,o:Tremellales,f:Sirobasidiaceae

AAGTCGTAACAAGGTTTCCGTAGGTGAACCTGCGGAAGGATCATTAGTGATTTGGCCTCC

GGGCCTTCAATCCCATCCTATACCTCTGTGAACCAGTTGGGCCCTCGGGCCTACCCTTTC

AAACACTGTGTAACGAACGTGATGTATATTATAAACCTAATAAAACTTTCAACAACGGAT

CTCTTGGCTCTC

>ASV112 GS|99.6|DQ534471|SH0942704.09FU;k:Fungi,p:Ascomycota,c:Lecanoromycetes,o:Lecanorales,f:Lecanoraceae,g:Lecidella,s:Lecidella carpathica

AAGTCGTAACAAGGTTTCCGTAGGTGGTTTGCCTGTCGGATCCCCCCGCAGCGACTCTAA

AGAACTGCGCCAGTCGGGCTCCCATCTCGAAGCCTGGCGACGCCATCAGTCTGGCTGGGA

GCCCCCCTACGCGGGGGGCCACCAGCAGCTCCTTCGGGAGTCCACAGATCAAACGATGAG

CGGCCGCTTCACTGCGGTTCAGATATGACCGGCCCCAGCCTGCATCGGCTGGTGACTCCT

CGTGGAACCT

>ASV113 GS|99.2|AY667583|SH0954634.09FU;k:Fungi,p:Ascomycota,c:Lecanoromycetes,o:Caliciales,f:Caliciaceae,g:Buellia,s:Buellia frigida

AAGTCGTAACAAGGTTTCCGTAGGTGAACCTGCGGAAGGATCATTACCGAGAGACGGGGT

CGCTTCGGCCCCACTCTTCACCCGTGTCTACTTACCTTTGTTGCTTTGGCGGGCCTTCGG

GCTTGACCGTTCGACGCGTGGGGAGGCTTTCGCCTCTCGCCTCGCGAGCGCCCGCCAAAG

GCCCTGTTAACTCTGTTTTAGTGTCTTCCGAGCAACCATGTAATAGTTAAAACTTTCAAC

AACGGATCTC

>ASV114 GSL|100.0|DQ028268|SH0956600.09FU;k:Fungi,p:Ascomycota,c:Leotiomycetes,o:Thelebolales,f:Thelebolaceae,g:Thelebolus

AAGTCGTAACAAGGTTTCCGTAGGTGAACCTGCGGAAGGATCATTAAAGAGACGTTGCCC

TTCGGGGTATACCTCCCACCCTTTGTTTATTTATACCTTTGTTGCTTTGGCAGACCCGGC

TTCGGCCCACCGGCTCCGGCTGGTCAGTGTCTGCCAGAGGACCTAAAACTCTGTTTGTTA

ATATTGTCTGAGTACTATATAATAGTTAAAACTTTCAACAACGGATCTCTTGGTTCTG

>ASV115 GS|81.4|AF250779|SH0954634.09FU;k:Fungi,p:Ascomycota,c:Lecanoromycetes,o:Caliciales,f:Caliciaceae,g:Buellia,s:Buellia frigida

AAGTCGTAACAAGATTTTCGTAGATGAATTTACGAAAAAATCATTATCGAGAGATGAGAT

CGCTTCGATCTCACTCTTCACTCGTATTTACTTATCTTTGTTGCTTTGACGAGTCTTCGA

GCTTAATCGTTCGACGCGAGTGAAAGACTTTCGTCTCTCGTCTTGCGAGCGCTCGTCAAA

GATCGTATTAACTTTATTTTAATGTCTTTCGAGCAATCATGTAATAGTTAAAACTTTCAA

TAACGAATTT

>ASV116 GS|89.2|AF250779|SH0954634.09FU;k:Fungi,p:Ascomycota,c:Lecanoromycetes,o:Caliciales,f:Caliciaceae,g:Buellia,s:Buellia frigida

AAGTCGTAACAAGGTTTTCGTAGGTGAATTTGCGAAAGAATCATTATCGAGAGACGAAAT

CGCTTCGGTCCCACTCTTCACTCGTGTCTACTTACCTTTGTTGCTTTGGCGAGCCTTCGA

GCTTGACCATTCGACGCGAGTGAGAGACTTTCGCCTCTCGTCTCGCGAGCGCTCGTCAAA

GGTCGTATTAACTCTATTTTAGTGTCTTTCGAGCAATCATGTAATAGTTAAAACTTTCAA

CAACGAATCT

>ASV117 GS|98.8|AY667583|SH0954634.09FU;k:Fungi,p:Ascomycota,c:Lecanoromycetes,o:Caliciales,f:Caliciaceae,g:Buellia,s:Buellia frigida

AAGTCGTAACAAGGTTTCCGTAGGTGAACCTGCGGAAGGATCATTACCGAGAGACGAGGT

CGCTTCGGCCCCACTCTTCACCCGTGTCTACTTACCTTTGTTGCTTTGGCGGGCCTTCGG

GCTTGACCGTTCGACGCGGGTGGGAGGCTTTCGCCTCTCGCCTCGCGAGCGCCCGCCAAA

GGCCCTGTTAACTCTGTTTTAGTGTCTTCCGAGCAACTGTAATAGTTAAAACTTTCAACA

ACGGATCTCT

>ASV118 GS|99.5|FJ265747|SH1280101.09FU;k:Fungi,p:Ascomycota,c:Eurotiomycetes,o:Chaetothyriales,f:Herpotrichiellaceae,g:Cladophialophora

AAGTCGTAACAAGGTTTCCGTAGGTGAACCTGCGGAAGGATCATTAAAGAGTGAGGGTCT

TCTCAGGCCCGACCTCCCAACCCTTTGTTTATCAACCCTTTTGTTGCTTCGGCGGGCCCG

TCTCACGACCGCCAGAGGATGTACCCCATCCTCTGGCCCGCGCCCGCCGATGGCCAACCA

TTTAAATTTCTTGAATGAATCGTGTCTTGTCCCTAAGTAATGATTAAATCAAAAACAAAA

CTTTCAACAA

>ASV119 GS|87.3|AF250779|SH0954634.09FU;k:Fungi,p:Ascomycota,c:Lecanoromycetes,o:Caliciales,f:Caliciaceae,g:Buellia,s:Buellia frigida

AAGTCGTAACAAGATTTTCGTAAGTGAATCTACGAAAGAATCATTATCGAGAGACGAAGT

CGCTTCGATCTCACTCTTCACTCATGTCTACTTATCTTTGTTGCTTTGACGAGTCTTCGA

GCTTGATCATTCGACGCGAGTGAAAGACTTTCGCCTCTCGTCTCGCGAGCGCTCGTCAAA

GGCCGTGTTAACTCTGTTTTAGTGTCTTTCGAGCAATCATGTAATAGTTAAAACTTTCAA

CAACGAATCT

>ASV120 SS|1.0000|EU257674|SH1256610.09FU;k:Fungi,p:Ascomycota,c:Lecanoromycetes,o:Lecideales,f:Lecideaceae,g:Lecidea

AAGTCGTAACAAGGTTTCCGTAGGTGAACCTGCGGAAGGATCATTAACGAGAGAGGGGCC

TCGTGCCTCGGGGGCTTCGGCCCCGCCTCTTCACCCCGTGCGTACCTACTTTGTTGCTTT

GGCGGGCCTCGGGTTTTCCCGACGCCGGCCCGGGGGCCACCCCGGGCTCTACCCCGGGCT

CTGCGCGCGCCCGCCGAAGGCCATGCCCAAACTCTGTTCATCAGTGTCGTCTGAGTACCA

ATTCAATAAC

>ASV121 GS|99.5|JN873879|SH1123218.09FU;k:Fungi,p:Ascomycota,c:Lecanoromycetes,o:Lecanorales,f:Lecanoraceae,g:Lecanora,s:Lecanora physciella

AAGTCGTAACAAGGTTTCCGTAGGTGAACCTGCGGAAGGATCATTACCGAGAGCGGGGCT

AACCCTCCAAACTCCGCCGCCGAAAGGGGTACTCTCCACCCTATGTATACATATACCACT

CTCGCTTTGGCGGGCTGAAGGCTCTTGCCCTACGCCCGCCAGTGGCTCAAAAAATTCTGT

TTATCAGTGATGTCCGAGTAAAAACCTAATAGTTAAAACTTTCAACAACGGATCTCTTGG

TTCTG

>ASV122 GS|99.1|FJ392866|SH1071027.09FU;k:Fungi,p:Ascomycota,c:Eurotiomycetes,o:Chaetothyriales

AAGTCGTAACAAGGTTTCCGTAGGTGAACCTGCGGAAGGATCATTACCGAGTTAGGGTCT

CTTCGGAGCCCGAACCTCCCAACCCTTTGTCTAATTTACCTTGTCGTTGCTTCGGCGGAC

CGGTTGACCAACTGGTCTTGACCGCCGGGGGTCCCGTACCCCTGGAGAGCGTCCGCCGAC

GGCCCAACCACAAACTCTTGTACTATACCATGTCGTCTGAATGTACTTGATATTAATCAA

CAAACAAAAC

>ASV123 GS|71.4|UDB0243452|SH1241595.09FU;k:Fungi,p:Basidiomycota,c:Agaricomycetes,o:Agaricales,f:Inocybaceae,g:Inocybe

AAGTCGTAACAAGGTTTCCGTAGGTGAACCTGCGGAAGGATCATTAGTGAATCAAGGGTG

ACTCTGTCAAGAGAATCCCGCGTACACTTTTTCATTACACTTGTGCACCGAATTTTTGAA

TGAGTTCAGTCTGGACTTACGGTTTTGTTAAAGGAGACTCGCGCAAAATTAGAAGCCTAA

GCTGAACTTGTATCAAACGCTTGATGAAATAGTTTGTGATGTCACATATAATAACGAAAG

AAAAACTTTC

>ASV124 GS|99.1|FJ392866|SH1071027.09FU;k:Fungi,p:Ascomycota,c:Eurotiomycetes,o:Chaetothyriales

AAGTCGTAACAAGGTTTCCGTAGGTGAACCTGCGGAAGGATCATTACCGAGTTAGGGTCT

CTTCGGAGCCCGAACCTCCCAACCCTTTGTCTAATTTACCTTGTCGTTGCTTCGGCGGAC

CGGTTGACCAACTGGTCTTGACCGCCGGGGGTCCCGTACCCTTGGAGAGCGTTCGCCGAC

GGCCCAACCACAAACTCTTGTACTAAACCATGTCGTCTGAATGTACTTGATATTAATCAA

AAAACAAAAC

>ASV125 SS|1.0000|AF250779|SH0954634.09FU;k:Fungi,p:Ascomycota,c:Lecanoromycetes,o:Caliciales,f:Caliciaceae,g:Buellia,s:Buellia frigida

AAGTCGTAACAAGATTTCCGTAGATGAATCTACGAAAGAATCATTATCGAGAGACGAGGT

CGCTTCGACCCCACTCTTCACCCGTGTCTACTTACCTTTGTTGCTTTGACGAGCCTTCGA

GCTTGATCGTTCGACGCGAGTGAGAGGCTTTCGCCTCTCGCCTCGCGAGCGCCCGCCAAA

GACCCTGTTAACTCTGTTTTAGTGTCTTTCGAGCAACCATGTAATAGTTAAAACTTTCAA

CAACGAATCT

>ASV126 GS|86.4|AY667583|SH0954634.09FU;k:Fungi,p:Ascomycota,c:Lecanoromycetes,o:Caliciales,f:Caliciaceae,g:Buellia,s:Buellia frigida

AAGTCGTAACAAGATTTTCGTAGGTGAATTTGCGAAAGAATCATTATCGAGAGACGAGAT

CGCTTCGATCTCACTCTTCACTCGTGTCTACTTATCTTTGTTGCTTTGACGAGTCTTCGA

GCTTAATCGTTCGACGCGAGTGAGAGACTTTCGTCTCTCGTTTCGCGAGCGCTCGTCAAA

GACTTTGTTAACTCTGTTTTAGTGTCTTTCGAGCAATCATGTAATAGTTAAAACTTTCAA

CAACGAATCT

>ASV127 GS|0.0|None;No hit

GTGTCAGCAGCCGCGGTAAGACGGAGGATGCAAGCGTTATCCGGAATGATTGGGCGTAAA

GCGTCTGTAGGTGGATTGTAAAGTCCTCTGTTAAAGATCTGGGCTTAACCCAGTTCAAGC

AGTGGAAACTTATAATCTAGAGTACGGTAGGGGCAGAGGGAATTCCCGGTGTAGCGGTGA

AATGCGTAGATATCGGGAAGAACACCGACAGCGAAAGCACTCTGCTGGGCCGAAACTGAC

ACTGAGAGAC

>ASV128 GS|99.6|FJ392866|SH1071027.09FU;k:Fungi,p:Ascomycota,c:Eurotiomycetes,o:Chaetothyriales,f:Trichomeriaceae,g:Knufia

AAGTCGTAACAAGGTTTCCGTAGGTGAACCTGCGGAAGGATCATTACCGAGTTAGGGTCT

CTTCGGAGCCCGAACCTCCCAACCCTTTGTCTAATTTACCTTGTCGTTGCTTCGGCGGAC

CGGTTGACCAACTGGTCTTGACCGCCGGGGGTCCCGTACCCCTGGAGAGCGTCCGCCGAC

GGCCCAACCACAAACTCTTGTACTAAACCATGTCGTCTGAATGTACTTTGATATTAATCA

AAAAACAAAA

>ASV129 GS|87.7|AF250779|SH0954634.09FU;k:Fungi,p:Ascomycota,c:Lecanoromycetes,o:Caliciales,f:Caliciaceae,g:Buellia,s:Buellia frigida

AAGTCGTAACAAGATTTTCGTAGATGAATCTACGAAAGAATCATTATCGAGAGACGAGGT

CGCTTCGACCTCACTCTTCACTCGTATCTACTTATCTTTGTTGCTTTGACGAGCCTTCGA

GCTTGATCGTTCGACGCGAATGAAAGGCTTTCGTCTCTCGTCTCGCGAGCGCTCGCCAAA

GATCGTGTTAACTCTATTTTAGTGTCTTTCGAGCAATCATGTAATAGTTAAAACTTTCAA

CAACGAATCT

>ASV130 GS|100.0|KT291473|SH1302832.09FU;k:Fungi,p:Ascomycota,c:Lecanoromycetes,o:Teloschistales,f:Teloschistaceae,g:Xanthomendoza,s:Xanthomendoza borealis

AAGTCGTAACAAGGTTTCCTTGGTGCCTACCGAAGCCTTGTAGAAATAGCAGTCCGCCTG

CGGGCGGGCGACATTCGCCTGTTGCTGGAAATCATCTCCTTTCGCGGGGGGTCAACCAGC

TGCTGCATGGCCCGCATGTAGTCCACAGATCAAACGCGAGTGGCCTGCGCCCCCGGCTCC

CCTGAGAAGGCGACCGTCGGCGCCGGTTAAGATATGACCGTAGGAAGTTCTGTAAAGAAC

CCGAAACTAG

>ASV131 SS|0.8100|UDB0700338|SH1084495.09FU;k:Fungi,p:Ascomycota,c:Dothideomycetes

AAGTCGTAACAAGGTCTCCGTAGGTGAACCTGCGGAGGGATCATTACCGAGTGAGGGCCT

CCGGGCTCGACCTCCAACCCTTTTGTCGTACCAACCACAGTTGCCTCGGGGGCGACCCGG

CCTCCGCGTCGGGGCCCCCGGTGGACCCAACCGAACAACTCTGCGTCTTTGCGTCTGAGT

ATTAAAGTAAATCAATTAAAACTTTCAACAACGGATCTCTTGGTTCTG

>ASV132 GS|100.0|KF309963|SH1084061.09FU;k:Fungi,p:Ascomycota,c:Dothideomycetes

AAGTCGTAACAAGGTCTCCGTAGGTGAACCTGCGGAGGGATCATTACCGAGTGAGGGCCT

CCGGGTCCGACCTCCAACCCCATGTTATCTGACCCTGTTGCCTCGGGGGCGACCCGGCCT

TCGGGCGTCTTGGGGCCCCCGGTGGACCACTCAACACTGCATCTTTGCGTCTGAGTTATA

TTTTGAATCAATCAAAACTTTCAACAACGGATCTCTTGGTTCTG

>ASV133 SS|0.9400|MK208766|SH1123218.09FU;k:Fungi,p:Ascomycota

AAGTCGTAACAAGGTTTCCGTAGGTGAACCTGCGGAAGGATCATTACCGAGAGCGGGGCT

AACCCCCTAAACTCCGCCGCCGAAAGGGGTACTCTCCACCCTATGTATATATATACCACT

CTCGCTTTGGCGGGTTGAGGGCTTTTACCCCGCGCCCGCCGGCGGCTTATTAAAATCTGT

TTGTTAGTGATGTCCGAGTAAAAACATAATAGTTAAAACTTTCAACAACGGATCTCTTGG

TTCTG

>ASV134 SS|0.8500|UDB0700338|SH1084495.09FU;k:Fungi,p:Ascomycota

AAGTCGTAACAAGGTCTCCGTAGGTGAACCTGCGGAGGGATCATTACCGAGTGAGGGCCT

CCGGGCTCGACCTCCAACCCTTTGTCGTACCAACCACAGTTGCCTCGGGGGCGACCCGGC

CTCCGCGTCGGGGCCCCCGGTGGACCCAACCAAACAACTCTGCGTCTTTGCGTCTGAGTA

TTAAAGTAAATCAATTAAAACTTTCAACAACGGATCTCTTGGTTCTG

>ASV135 GS|82.0|AY667583|SH0954634.09FU;k:Fungi,p:Ascomycota,c:Lecanoromycetes,o:Caliciales,f:Caliciaceae,g:Buellia,s:Buellia frigida

AAGTCGTAACAAGATTTTCGTAGATGAATTTGCGAAAGAATCATTATCGAGAGACGAGAT

CGCTTCGATCTCATTCTTCATTCGTATTTACTTATCTTTGTTGCTTTGACGAGTCTTCGA

ATTTGATCGTTCGACGCGAATGAGAGACTTTCGTCTCTCGTTTCGCGAACGCTCGTCAAA

GATCTTGTTAACTTTGTTTTAGTGTCTTTCGAGTAATCATGTAATAGTTAAAACTTTCAA

TAACGAATCT

>ASV136 SS|0.8300|UDB02859585|SH0920406.09FU;k:Fungi,p:Ascomycota,c:Eurotiomycetes,o:Chaetothyriales

AAGTCGTAACAAGGTTTCCGTAGGTCAGTAATAAACCCTCCAATTCCCCTCCACTATTCT

TTTGTTATGACATACTAATATAGTTTCTCTTAGGTGAACCTGCGGAAGGATCATTACTGA

GTTAGGGTCTTTCTAGGCTCGATCTCCAACCCTTTGTATAACTACCATGTTGCTTTGGCG

GGCCCGTCTCTAACCGGACCGCCGGGGGTCTTTGACCTCTGGCCAGCGCTCGCCAGTAGC

CCACCACAAA

>ASV137 SS|1.0000|KF274260|SH1023760.09FU;k:Fungi,p:Ascomycota

AAGTCGTAACAAGGTTTCCGTAGGTGAACCTGCGGAAGGATCATTAATGGGATTGACCTC

TAAGGTCTAACCTTCAACCCTTTGTCTTAACAACCCTTGTTTCTTTGGCGGGTTCGCCCG

TCAGAAGAATTCTCAAACTCATATAATTATGTCTTCTGAAATCAAAAAACAAATAAAATT

AAAACTTTCAACAACGGATCTCTTGGTTCTG

>ASV138 SS|0.9400|MN592663|SH1107827.09FU;k:Fungi,p:Ascomycota,c:Lecanoromycetes,o:Teloschistales,f:Teloschistaceae

AAGTCGTAACAAGGTTTCCGTAGGTGAACCTGCGGAAGGATCATTATCGAGAGGGGGGCT

CCATGCCCCGGGGCTCCGTCCCCGTACCTTTTCACCCTGTGTGTATTTTTCCCCCGTTGC

TTTGGCGGGCCCCGGGTCTTCCCCCGGCGTTGGCCCCCTCGCGGGGTTCGCGAGCGCCCG

CCGAAGGCTCATCGAAACTCTGTTGATCAGTGCAGTCTGAGCGTACGAACAATAAATCAA

AACTTTCAAC

>ASV139 SS|1.0000|MK970652|SH1123214.09FU;k:Fungi,p:Ascomycota,c:Lecanoromycetes,o:Lecanorales,f:Catillariaceae,g:Austrolecia

AAGTCGTAACAAGGTTTCCGTAGGTGAACCTGCGGAAGGATCATTACCGAGAGAGGGGGG

GCGACCCCCCAATCTTACCGCCGGGCTCCGCTGTTCGCAGCCGACCCGGGGTCTCTCCAC

CCTATGTATACCCTACCATTGTTGCTTTGGCGGGCTCGAGGCTGTTGCCCCGCGCTCGCC

AGAGGCCCATTAAATACTATTCATTAGTGATGTCTGAGTCAAAAACACAATAGTTAAAAC

TTTCAACAAC

>ASV140 GS|99.6|AY667583|SH0954634.09FU;k:Fungi,p:Ascomycota,c:Lecanoromycetes,o:Caliciales,f:Caliciaceae,g:Buellia,s:Buellia frigida

AAGTCGTAACAAGGTTTCCGTAGGTGAACCTGCGGAAGGATCATTACCGAGAGATGGGGT

CGCTTCGGCCCCACTCTTCACCCGTGTCTACTTACCTTTGTTGCTTTGGCGGGCCTTCGG

GCTTGACCGTTCGACGCGGGTGGGAGGCTTTCGCCTCTCGCCTCGCGAGCGCCCGCCAAA

GGCCCTGTTAACTCTGTTTTAGTGTCTTCCGAGCAACCATGTAATAGTTAAAACTTTCAA

CAACGGATCT

>ASV141 GS|99.2|KU948792|SH0918900.09FU;k:Fungi,p:Basidiomycota,c:Cystobasidiomycetes,o:Cyphobasidiales

AAGTCGTAACAAGGTTTCCGTAGGTGAACCTGCGGAAGGATCATTAATGAAATTCAAGGA

TGCTCTTTTTAGAGATCCGACCTTTTTCATCTCACACTGTGCACACACACTTTTACACCT

TTTTCACTCGTAGAGTTTATAAGAATGTAACTGTCTCTTAATTGAGCATAAATTAGTAAA

ACTTTCAGCAACGGATCTCTTGGCTCTC

>ASV142 SS|0.9500|None;k:Fungi

AAGTCATAACAAGGTTTCCGTAGGTGAACCTGCGGAAGGATCATTCGAGAATAGGGCCTC

TGGCCTTAATCCATTATATACACCGTGAAAACATAACCTTGGGACCTCGCAAGGGGGACC

TCGGATTACACTTTTAAACAGAGTCAAGAAAGGAAACATTATAAATACAAATAACTTTTA

ACAACGGATCTCTTGGCTCTC

>ASV143 SS|1.0000|AF250779|SH0954634.09FU;k:Fungi,p:Ascomycota,c:Lecanoromycetes,o:Caliciales,f:Caliciaceae,g:Buellia,s:Buellia frigida

AAGTCGTAACAAGATTTCCGTAGGTGAATCTGCGAAAGGATCATTATCGAGAGACGAAGT

CGCTTCGACCCCACTCTTCACCCGTGTCTACTTACCTTTGTTGCTTTGGCGAGCCTTCGG

GCTTGATCGTTCGACGCGAGTGGGAGGCTTTCGCCTCTCGCCTCGCGAGCGCCCGCCAAA

GGCCCTGTTAACTTTGTTTTAGTGTCTTTCGAGCAACCATGTAATAGTTAAAACTTTCAA

CAACGGATCT

>ASV144 SS|0.8500|KF274388|SH1185377.09FU;k:Fungi,p:Basidiomycota,c:Tremellomycetes

AAGTCGTAACAAGGTTTCCGTAGGTGAACCTGCGGAAGGATCATTAGTGAATGTCTTCGG

ACTTTAACATATTCAAACCTTTGTGAACTGTGGCCTCCGGGCCGATCTTCAAACAATGTG

TAATGAACGTATAACAAATAAAAACAAATAAAACTTTCAACAACGGATCTCTTGGCTCTC

>ASV145 GSL|100.0|KF309980|SH0993867.09FU;k:Fungi,p:Ascomycota,c:Dothideomycetes,o:Mycosphaerellales,f:Extremaceae

AAGTCGTAACAAGGTCTCCGTAGGTGAACCTGCGGAGGGATCATTACCGAGTGAGGGCCC

TCGCGCCCGACCTCCAACCCTTTGTCGATCAATATCTGTTGCCTCGGGGGGCGACCCGGC

CGTCCGCGGGCGGGGGTCCCCCAGAGGACCAATCAACTCTGCATCTTTGCGTCGAGTATT

GAATACAAATCAATCAAAACTTTTAACAACGGATCTCTTGGTTCTG

>ASV146 GS|88.2|AF250779|SH0954634.09FU;k:Fungi,p:Ascomycota,c:Lecanoromycetes,o:Caliciales,f:Caliciaceae,g:Buellia,s:Buellia frigida

AAGTCGTAACAAGATTTTCGTAGATGAATTTGCGAAAGAATCATTATCGAGAGACGAGAT

CGCTTCGATCTCACACTCTTCACTCGTGTCTACTTATCTTTGTTGCTTTGACGAGTCTTC

GAGCTTGATCGTTCGACGCGAGTGAGAGACTTTCGCCTCTCGCCTCGCGAGCGCTCGTCA

AAGGCCCTGTTAACTCTGTTTTAGTGTCTTTCGAGCAATCATGTAATATTTAAAACTTTC

AACAACGAAT

>ASV147 SS|0.9700|KC965541|SH0962007.09FU;k:Fungi

AAGTCGTAACAAGGTTTCCGTAGGGTACGTTCACCCCTATGAGCCCCCCTTGCTACCTCG

AAAATGATTTGCTAACTCTCATTCTAGTGAACCTGCGGAAGGATCATTAAAGAGAAATCG

GGGTATCGCCGCGAGGCTTTACCCCATCTCCCTAAACCCCCTGTCTACCGACCTCACACA

AACCACCGTACAAATATCAAGTCGTCCCTAGCGGGGCGCAATACCATAAATGTACAAAAA

ACAAAACTTT

>ASV148 GS|0.0|None;No hit

GTGTCAGCAGCCGCGGTAATACGGGGGGGGCAAGCGTTATTCGAAATGATTGGGCGTAAA

GGGCACGTAGACGGTTTTTTAAGTGGCCATCCTTGTTTTTGTTTTTCCCTTTCAACTCTA

TTATAAATAAAATACATAGAAGGGAAACGGGAAAAGGGAAGCAAAGATTAAAATGGAGTG

TGGATTCTCTTTATTTTTATAATCCCTTATTTGTATAAATTATTTATAATGAAAACAAAT

AAGGGATAAC

>ASV149 SS|1.0000|KF673749|SH0992234.09FU;k:Fungi

AAGTCGTAACAAGGTTTCCGTAGGTGAACCTGCGGAAGGATCATTACTGAGTTAGGGTCT

TCTAGGCCCGACCTCCAACCCTATGTCTACCTTACCATGTTGCTTTGGCGGGCCCGTCGT

TAGTTCGACCGCCTCCGGGTCCGTGCCCGCCAGAAGCCCAAATTAAATTCTTGATGAAAC

TTGTCGTCTTAGTATACAAGCAATAATAAAAAACTTTCAACAACGGATCTCTTGGTTCTG

>ASV150 SS|1.0000|AY667583|SH0954634.09FU;k:Fungi,p:Ascomycota,c:Lecanoromycetes,o:Caliciales,f:Caliciaceae,g:Buellia,s:Buellia frigida

AAGTCGTAACAAGGTTTCCGTAGGTGAATCTACGAAAGGATCATTATCGAGAGACGAGGT

CGCTTCGACCTCACTCTTCACTCGTGTCTACTTACCTTTGTTGCTTTGGCGGGCCTTCGG

GCTTGATCGTTCGACGCGAGTGAAAGGCTTTCGCCTCTCGCCTCGCGAGCGCTCGCCAAA

GGCCCTGTTAACTCTGTTTTAGTGTCTTTCGATCAATCATGTAATAGTTAAAACTTTCAA

CAACGAATCT

>ASV151 SS|1.0000|KF274227|SH0911134.09FU;k:Fungi

AAGTCGTAACAAGGTTTCCGTAGGTGAACCTGCGGAAGGATCATTACCGAGTTAGGGTCC

TCTGGGCTCGATCTCCAACCCTGTGTCTAAATAACCACGTTGCTTTGGCGGGCCCGCCTG

TAATGGGCCGCCGGGGGTGCCTTCGGCCCCCTGGTCCGCGCCTGCCAGTAGCCATCTCAT

ACTCTTCTTAATCGTGACGTCTGAGTAAAAATTATAAATTAAACAAAACTTTCAACAACG

GATCTCTTGG

>ASV152 GS|99.6|JX036043|SH0954629.09FU;k:Fungi,p:Ascomycota,c:Lecanoromycetes,o:Caliciales,f:Caliciaceae,g:Buellia

AAGTCGTAACAAGGTTTCCGTAGGTGTCACGACCCGCCCGGTTCGCCTACATCGCCTCGG

AGCGACTATAAAGAAAGATTTAGTCGGGTGCTCTTGCGTCCGGCCACGCGGCTATTCTGC

CTGGGAGCAAACCAACCTCTCTGGAGGCTCGGGTGTAACCAGCAGCCCGTACCGCGCTGG

CGGTACTGGGTTCACAGATCAAATGCCTGCGGCCACGCCTTAGTGGTTCTAATATGACCG

ACCTTCGCCA

>ASV153 GS|100.0|UDB02904352|SH1048210.09FU;k:Fungi,p:Basidiomycota,c:Tremellomycetes,o:Tremellales,f:Bulleraceae,g:Genolevuria

AAGTCGTAACAAGGTTTCCGTAGGTGAACCTGCGGAAGGATCATTAGTGATTGGCTTCGG

CCTCATATCCTTTCAAACCTGTGCACCTCTTGGCTCCGGCCAATTCAAACATGTGTCATG

AATGTACATAAACAAAAACATAAACAAAACTTTCAACAACGGATCTCTTGGCTCTC

>ASV154 SS|1.0000|AF250779|SH0954634.09FU;k:Fungi,p:Ascomycota,c:Lecanoromycetes,o:Caliciales,f:Caliciaceae,g:Buellia,s:Buellia frigida

AAGTCGTAACAAGGTTTCCGTAGGTGAATCTACGAAAGAATCATTATCGAGAGACGGGGT

CGCTTCGACCCCACTCTTCACTCGTGTTTACTTACCTTTGTTGCTTTGGCGAGCCTTCGA

GCTTGATCGTTCGACGCGAGTGGGAGACTTTCGCCTCTCGTCTCGCGAGCGCCCGCCAAA

GGTCTTGTTAACTCTGTTTTAGTGTCTTCCGACCAACCATGTAATAGTTAAAACTTTCAA

CAACGAATCT

>ASV155 SS|0.9500|AM901805|SH0999915.09FU;k:Fungi

AAGTCATAACAAGGTTTCCGTAGGTGAACCTGCGGAAGGATCATTCGAGAATAGGGCCTC

TGGCCTTAATCCAAAACCCACATCGTGAACCGTACCTTGGGACCTCGCAAGGGGGACCTT

GGATTACACTTTATAACTGAGTCTTGAAAGGAAGAATATGACAAACAGAAAATAACTTTT

AACAACGGATCTCTTGGCTCTC

>ASV156 GS|100.0|AF250779|SH0954634.09FU;k:Fungi,p:Ascomycota,c:Lecanoromycetes,o:Caliciales,f:Caliciaceae,g:Buellia,s:Buellia frigida

AAGTCGTAACAAGGTTTCCGTAGGTGAACCTACGGAAGGATCATTACCGAGAGACGGGGT

CGCTTCGGCCCCACTCTTCACCCGTGTCTACTTACCTTTGTTGCTTTGGCGGGCCTTCGG

GCTTGACCGTTCGACGCGGGTGGGAGGCTTTCGCCTCTCGCCTCGCGAGCGCCCGCCAAA

GGCCCTGTTAACTCTGTTTTAGTGTCTTCCGAGCAACCATGTAATAGTTAAAACTTTCAA

CAACGGATCT

>ASV157 GS|100.0|GU170842|SH1256610.09FU;k:Fungi,p:Ascomycota,c:Lecanoromycetes,o:Lecideales,f:Lecideaceae,g:Lecidea,s:Lecidea cancriformis

AAGTCGTAACAAGGTTTCCGTAGGTGAACCTGCGGAAGGATCATTAACGAGAGAGGGGCC

TCGTGCCTCGGGGGCTTCGGCCCCGCCTCTTCACCCCGTGCGTACCTACTTTGTTGCTTT

GGCGGGCCTCGGGTTTTCCCGACGCCGGCCCGGGGGCTACCCCGGGCTCTGCGCGCGCCC

GCCGCAGGCCATTCCGAAACTCTGTTCATCAGTGTCGTCCGAGTACCAATTCAATAACTA

AAACTTTCAA

>ASV158 GS|84.4|AY667583|SH0954634.09FU;k:Fungi,p:Ascomycota,c:Lecanoromycetes,o:Caliciales,f:Caliciaceae,g:Buellia,s:Buellia frigida

AAGTCGTAACAAGATTTTCGTAGATGAATTTGCGAAAGAATCATTATCGAGAGACGAGAT

CGCTTCGATTTCACTCTTCATTCGTGTCTACTTATCTTTGTTGCTTTGACGAGTCTTCGA

GCTTGATCGTTCGACGCGAGTGAGAGACTTTCGTCTCTCGTCTTGCGAGCGCTCGTCAAA

GACTCTATTAACTTTATTTTAGTGTCTTTCGAACAATCATGTAATAGTTAAAACTTTCAA

CAACGAATCT

>ASV159 GS|100.0|JX092259|SH1188820.09FU;k:Fungi,p:Basidiomycota,c:Tremellomycetes,o:Filobasidiales,f:Filobasidiaceae,g:Naganishia,s:Naganishia friedmannii

AAGTCGTAACAAGGTTTCCGTAGGTGAACCTGCGGAAGGATCATTAATGAATACAGATGC

CTGTCGAGCTTGCTCACGGGCTTTCTATCATATCCATAACACCTGTGCACTTGTTGGATG

TTCTAGAGACTTAGGGTTAAACCTGCAGTCAATAGTCATCCACTTACACTAAACAATAAT

GTAACAAATGTAGTCTTATTATAACAAAATAAAACTTTCAACAACGGATCTCTTGGCTCT

C

>ASV160 GS|98.4|KF823589|SH1091255.09FU;k:Fungi,p:Basidiomycota,c:Tremellomycetes,o:Tremellales,f:Sirobasidiaceae

AAGTCGTAACAAGGTTTCCGTAGGTGAACCTGCGGAAGGATCATTAGTGATTTGGCCTCC

GGGCCTTCAATCCCATCCTCATACCTCTGTGAACCAGTTGGGCCCTCGGGCCTACCCTTC

CAAACACTGTGTAACGAACGTGATGTATATTATAAACCTAATAAAACTTTCAACAACGGA

TCTCTTGGCTCTC

>ASV161 GS|98.5|UDB03337334|SH0989105.09FU;k:Fungi,p:Ascomycota,c:Leotiomycetes,o:Helotiales

AAGTCGTAACAAGGTTTCCGTAGGTGAACCTGCGGAAGGATCATTACCGAGTTTGTGCCC

TCTGGGGTAGATCTCCCACCCTGTTTATTTTACTTCATGTTGCTTTGGCGGGTCGACTGT

CCAGGCCACCGGCGACTCGCTGGTGTGTGCCCGCCAAAGGCTCCTAAACTCGAGTATGTA

ACCTGCCGTCCGAGTCCTATATAATACTTAAAACTTTCAACAACGGATCTCTTGGTTCTG

>ASV162 GS|100.0|AF250779|SH0954634.09FU;k:Fungi,p:Ascomycota,c:Lecanoromycetes,o:Caliciales,f:Caliciaceae,g:Buellia,s:Buellia frigida

AAGTCGTAACAAGGTTTCCGTAGGTGAACCTGCGGAAGAATCATTACCGAGAGACGGGGT

CGCTTCGGCCCCACTCTTCACCCGTGTCTACTTACCTTTGTTGCTTTGGCGGGCCTTCGG

GCTTGACCGTTCGACGCGGGTGGGAGGCTTTCGCCTCTCGCCTCGCGAGCGCCCGCCAAA

GGCCCTGTTAACTCTGTTTTAGTGTCTTCCGAGCAACCATGTAATAGTTAAAACTTTCAA

CAACGGATCT

>ASV163 GS|0.0|None;No hit

AAGTCGTAACAAGATTTTCGTAGATGAATTTGCGAAAGAATCATTATTGAGAAACGAAAT

CGCTTCGACTTCACTCTTCATTCGTATCTATTTATTTTTGTTGCTTTGACGAATTTTCGA

ACTTGATCGTTCGACGCGAGTGAGAGACTTTCGTCTCTCGTTTTGCGAGCGCTCGTCAAA

GACTTTGTTAACTTTGTTTGAATGTCTTTCGAACAATTATATAATAATTAAAACTTTCAA

TAGCGAATTT

>ASV164 SS|0.8300|MG733135|SH1107635.09FU;k:Fungi,p:Ascomycota,c:Lecanoromycetes,o:Teloschistales,f:Teloschistaceae

AAGTCGTAACAAGGTTTCCGTAGGTGAACCTGCGGAAGGATCATTATCGAGAGGGGGGTT

CCATGCCCCGGGGCTCTGTCCCCGTACCTTTTCACCCTGTGTGTATTTTTCCCCCGTTGC

TTTGGCGGGCCCCGGGTCTTCCTCCCGGCGTTGGCCCCCTCGCGGGGGTTCGCGAGCGCC

CGCCGAATGCTCATCCAAACTCTGTTGACCAGTGCAGTCTGAGCGTACGAACAATAAATC

AAAACTTTCA

>ASV165 GS|100.0|MK208787|SH1097880.09FU;k:Fungi,p:Ascomycota,c:Lecanoromycetes,o:Lecanorales,f:Lecanoraceae,g:Carbonea,s:Carbonea vorticosa

AAGTCGTAACAAGGTTTCCGTAGGTGAACCTGCGGAAGGATCATTAATGGGAGGGGTTTT

CGGACCCCGGGGACTCCGGTCCCCCCCCCTTACCCTATGCCTACCTACCTTCGTTGCCTT

GACGGGCCCGAGGAATACCCTTCTTGCACCCCCGTCAGTGGCCCCTCCAACTTCGTCCAT

CAGCGTCGTCCGAGTAGTACTATAATCGTAAAAACTTTCAACAACGGATCTCTTGGTTCT

G

>ASV166 GS|85.8|AF250779|SH0954634.09FU;k:Fungi,p:Ascomycota,c:Lecanoromycetes,o:Caliciales,f:Caliciaceae,g:Buellia,s:Buellia frigida

AAGTCGTAACAAGATTTTCGTAGATGAATTTGCGAAAGAATCATTATCGAGAGATGAGAT

CGCTTTGATCTCACTCTTCACTCGTGTCTACTTATCTTTGTTGCTTTGACGAGTCTTCGA

GCTTGATCGTTCGACGCGAATGAGAGGCTTTCGTCTCTCGTCTCGCGAGCGCTCGCCAAA

GATCTTGTTAACTCTATTTTAGTGTCTTTCAAGCAATCATGTAATAGTTAAAACTTTCAA

CAACGAATCT

>ASV167 GSL|100.0|KU057816|SH1048210.09FU;k:Fungi,p:Basidiomycota,c:Tremellomycetes,o:Tremellales

AAGTCGTAACAAGGTTTCCGTAGGTGAACCTGCGGAAGGATCATTAGTGATTGGCTTCGG

CCTCATATCCTTTCAAACCTGTGCACCTCTTGGCTCCGGCCAATTCAAACATGTGTCATG

AATGTATATAAACAAAAACATAAACAAAACTTTCAACAACGGATCTCTTGGCTCTC

>ASV168 SS|0.8300|KF274277|SH0999504.09FU;k:Fungi,p:Basidiomycota,c:Tremellomycetes,o:Tremellales

AAGTCGTAACAAGGTTTCCGTAGGTGAACCTGCGGAAGGATCATTAGTGATTGGTCTCTG

GACCATTTTCATCATCCATATATACCTCTGTGAACCTGTCGGCCCTCGGGCCAGCTTTTC

CAAACAAAGTGTAATGAACGTGTGTGATTATAACCTAGTAAAACTTTCAACAACGGATCT

CTTGGCTCTC

>ASV169 GS|85.2|AY667583|SH0954634.09FU;k:Fungi,p:Ascomycota,c:Lecanoromycetes,o:Caliciales,f:Caliciaceae,g:Buellia,s:Buellia frigida

AAGTCGTAACAAGATTTTCGTAGATGAACTTGCGAAAGAATCATTATCGAGAGACGAAAT

CGCTTCGATCTCACTCTTCACTCGTGTCTACTTATCTTTGTTGCTTTGACGAGTTTTCGA

ATTTGATCGTTCGACGCGAGTGAGAGACTTTCGTCTCTCGTCTCGCGAGCGCTCGTCAAA

GACTTTGTTAACTTTGTTTTAGTGTCTTTCGAGCAATCATGTAATAGTTAAAACTTTCAA

CAACGAATCT

>ASV170 GS|83.6|AY667583|SH0954634.09FU;k:Fungi,p:Ascomycota,c:Lecanoromycetes,o:Caliciales,f:Caliciaceae,g:Buellia,s:Buellia frigida

AAGTCGTAACAAGATTTTCGTAGATGAACTTGCGAAAGAATCATTATTAAGAGACGAGAT

CGCTTCGATCTCACTCTTCATTCGTATCTACTTATCTTTGTTGCTTTGACGAGCTTTCGA

GCTTGATCGTTCGACGCGAGTGAGAGACTTTCGTCTCTCGTTTTGCGAGCGCTCGTCAAA

GATCTTGTTAACTCTATTTTAGTGTCTTTCGAACAATCATGTAATAGTTAAAACTTTTAA

CAACGAATCT

>ASV171 GS|72.3|KF535949|SH1174631.09FU;k:Fungi,p:Basidiomycota,c:Agaricomycetes,o:Agaricales,f:Amanitaceae,g:Amanita,s:Amanita exitialis

AAGTCGTAACAAGGTTTTCGTAGGTGAACCTGCGGAAGGATCATTACCAGATGGGCCTCC

GGGCCTTTTCTCCCAACCACGTGCACCGAGTTGCCGGGAGACGCGCAAGCCTCTCCCGAC

CAATTCTTCACGCTTGTTTTGTTGTCTGAGTCGAATGACCATAATTGAAAAGAAAACTAT

GAACAACGGATCTCTAGGCTCTT

>ASV172 GS|99.5|KC179119|SH1085530.09FU;k:Fungi,p:Ascomycota,c:Lecanoromycetes,o:Teloschistales,f:Teloschistaceae,g:Shackletonia,s:Shackletonia insignis

AAGTCGTAACAAGGTTTCCGTAGGTGAACCTGCGGAAGGATCATTATCGAGAGCGGGGCT

TCGCGCCCCCGGGGGGCTTCGGCCTCCTACCTCTTCACCCTGTGTATATCAACCTCTGTT

GCTTTGGCGAGCCCTGGGTTTACCCCCCGGCACGGTTTCGGGCGATCCTGCCCGGAGTCG

CGGGTGCTCGCCCGAGGCCTTTAACCATCTGTTTACGTGGAACGTCCGAATGACCCAAAA

ATTAAATAAA

>ASV173 GS|0.0|None;No hit

GTGTCAGCAGCCGCGGTAATACGTAGAAGACAAGTGTTATTCATCTTTAATAGGTTTAAA

GGGTACTTAGACGGTAAATAAAGCCTCCAAAAGGTACTAGTTTGCTAGAGTTTTATATGA

AGGAGTTTTAAAGTACTACTGGTGTAGAGATGAAATTCAGTTATACCTTACATGGCACAG

GTTAAGGCGAAAGCATCTTCTTATGTAAAAACTGACGTTGAAGGACGAAGGCTTTGTGTC

TCGAACAGGA

>ASV174 GS|99.0|JN873879|SH1123218.09FU;k:Fungi,p:Ascomycota,c:Lecanoromycetes,o:Lecanorales,f:Lecanoraceae,g:Lecanora,s:Lecanora physciella

AAGTCGTAACAAGGTTTCCGTAGGTGAACCTGCGGAAGGATCATTACCGAGAGCGGGGCT

AACCCCCCAAACTCCGCCGCCGAAAGGGGTACTCTCCACCCTATGTATACATATACCACT

CTCGCTTTGGCGGGCTGAAGGCTCTTGCCCTACGCCCGCCAGTGGCTCAAAAAATTCTGT

TCATCAGTGATGTCCGAGTAAAAACCTAATATTTAAAACTTTCAACAACGGATCTCTTGG

TTCTG

>ASV175 GS|86.3|AF250779|SH0954634.09FU;k:Fungi,p:Ascomycota,c:Lecanoromycetes,o:Caliciales,f:Caliciaceae,g:Buellia,s:Buellia frigida

AAGTCGTAACAAGATTTTCGTAGATGAATTTACGAAAGAATTATTATCGAGAGACGAGAT

CGCTTCGACCTCACTTTTCACTCGTATCTACTTACCTTTGTTGCTTTGGCGAGTCTTCGA

ACTTGATCGTTCGACGCGAGTGAGAGACTTTCGTCTCTCGTCTTGCGAGCGCTCGTCAAA

GGCTTTGTTAACTCTATTTTAGTGTCTTTCGAGCAATCATGTAATAGTTAAAACTTTCAA

CAATGAATCT

>ASV176 GS|100.0|FJ415476|SH0991924.09FU;k:Fungi,p:Ascomycota,c:Dothideomycetes,o:Mycosphaerellales,f:Teratosphaeriaceae,g:Elasticomyces,s:Elasticomyces elasticus

AAGTCGTAACAAGGTCTCCGTAGGTGAACCTGCGGAGGGATCATTACCGAGTGAGGGCCT

CCGGGTCCGACCTCCAACCCCATGTTAACCGACCCTGTTGCCTCGGGGGCGACCCGGACT

CGTGCCGGGGCCCCCGATGGACCTTCAAACACTGCATCTTTGCGTCCGAGTCAATATTTT

GAATAAATCAAAACTTTCAACAACGGATCTCTTGGTTCTG

>ASV177 SS|1.0000|KF617804|SH1034547.09FU;k:Fungi

AAGTCATAACAAGGTTTCCGTAGGTGAACCTGCGGAAGGATCATTCGAGAATAGGGCCTC

TGGCCTTAACCCAATACACACACATTGTGAACTGTGCCTCGGGACCTCACGAGGGGGACC

TTGGATTTACACTATAACAAAGTCAGTCTTGAAAGGAAGGACATTATAAATACAAATATA

ACTTTCAACAACGGATCTCTTGGCTCTC

>ASV178 SS|0.8100|MN592663|SH1107827.09FU;k:Fungi,p:Ascomycota,c:Lecanoromycetes,o:Teloschistales,f:Teloschistaceae

AAGTCGTAACAAGGTTTCCGTAGGTGAACCTGCGGAAGGATCATTATCGAGAGGGGGGTT

CCATGCCCCGGGGCTCTGTCCCCATACCTTTTCACCCTGTGTGTATTTTTCCCCCGTTGC

TTTGGCGGGCCCCGGGTCTTCCCCCGGCGTTGGCCCCCTCGCGGGGTTCGCGAGCGCCCG

CCGAAGGCTCATCGAAACTCTGTTGGTCAGTGCAGTCTGAGCGTACGAATAATAAATCAA

AACTTTCAAC

>ASV179 SS|0.9100|KC346301|SH1107756.09FU;k:Fungi,p:Ascomycota,c:Lecanoromycetes,o:Teloschistales,f:Teloschistaceae

AAGTCGTAACAAGGTTTCCGTAGGTGAACCTGCGGAAGGATCATTATCGAGAGGGGGGCT

CCATGCCCCGGGGCTCTGTCCCCGTACCTTTTCACCCTGTGTGTATTTTTCCCCCGTTGC

TTTGGCGGGCCCCGGGTCTTCCCCCGGCGCTGGCCCCCTCGCGAGGTTCGCGAGCGCCCG

CCGAAGGCTCTTCGAAACTCTGTTGATCAGTGCAGTCTGAGCGTACGAACAATAAATCAA

AACTTTCAAC

>ASV180 GS|87.3|AF250779|SH0954634.09FU;k:Fungi,p:Ascomycota,c:Lecanoromycetes,o:Caliciales,f:Caliciaceae,g:Buellia,s:Buellia frigida

AAGTCGTAACAAGATTTTCGTAGATGAACTTGCGAAAGAATCATTATCGAGAGACGAGGT

CGCTTCGATCTCACTCTTCACTCGTATCTACTTATCTTTGTTGCTTTGACGAGTCTTCGA

GCTTGATCATTCGACGCGAGTGAGAGGCTTTCGTCTCTCGTCTCGCGAGCGCTCGTCAAA

GGCTTTGTTAACTCTGTTTTAGTGTCTTTCGAGCAATCATGTAATAGTTAAAACTTTCAA

CAACGAATTT

>ASV181 GS|99.6|AY667583|SH0954634.09FU;k:Fungi,p:Ascomycota,c:Lecanoromycetes,o:Caliciales,f:Caliciaceae,g:Buellia,s:Buellia frigida

AAGTCGTAACAAGGTTTCCGTAGGTGAACCTGCGGAAGGATCATTACCGAGAGACGGGGC

CGCTTCGGCCCCACTCTTCACCCGTGTCTACTTACCTTTGTTGCTTTGGCGGGCCTTCGG

GCTTGACCGTTCGACGCGGGTGGGAGGCTTTCGCCTCTCGCCTCGCGAGCGCCCGCCAAA

GGCCCTGTTAACTCTGTTTTAGTGTCTTCCGAGCAACCATGTAATAGTTAAAACTTTCAA

CAACGGATCT

>ASV182 GS|83.3|AF250779|SH0954634.09FU;k:Fungi,p:Ascomycota,c:Lecanoromycetes,o:Caliciales,f:Caliciaceae,g:Buellia,s:Buellia frigida

AAATCGTAACAAGATTTTCGTAAATGAATTTGCGAAAGAATCATTATCGAGAGACGAAAT

CGCTTCGATCTCATTCTTCACTCGTATCTACTTATCTTTGTTGCTTTGACGAGTCTTCGA

ACTTGATCGTTCGATGCGAGTGAGAGACTTTCGTCTCTCGTCTCGCGAGCGCTCGTCAAA

GATCGTATTAACTTTGTTTTAATGTCTTTCGAGCAATCATGTAATAGTTAAAACTTTCAA

CAACGAATCT

>ASV183 GS|87.3|AF250779|SH0954634.09FU;k:Fungi,p:Ascomycota,c:Lecanoromycetes,o:Caliciales,f:Caliciaceae,g:Buellia,s:Buellia frigida

AAGTCGTAACAAGATTTTCGTAGATGAATTTGCGAAAGAATCATTATCGAGAGACGAGAT

CGCTTCGATCTCACTCTTCACTCGTGTCTACTTATCTTTGTTGCTTTGACGAGTCTTCGA

GCTTGATCGTTCGACGCGAGTGAGAGACTTTCGTCTCTCGTCTCGCGAGCGCTCGTCAAA

GGTCGTGTTAACTTTGTTTTAGTGTCTTTCGAGCAATCATGTAATAGTTAAAACTTTCAA

CAACGAATCT

>ASV184 GS|99.6|AY667583|SH0954634.09FU;k:Fungi,p:Ascomycota,c:Lecanoromycetes,o:Caliciales,f:Caliciaceae,g:Buellia,s:Buellia frigida

AAGTCGTAACAAGGTTTCCGTAGGTGAACCTGCGGAAGGATCATTACCGAGAGACGGGGT

CGCTTGGGCCCCACTCTTCACCCGTGTCTACTTACCTTTGTTGCTTTGGCGGGCCTTCGG

GCTTGACCGTTCGACGCGGGTGGGAGGCTTTCGCCTCTCGCCTCGCGAGCGCCCGCCAAA

GGCCCTGTTAACTCTGTTTTAGTGTCTTCCGAGCAACCATGTAATAGTTAAAACTTTCAA

CAACGGATCT

>ASV185 GS|98.4|MW991426|SH0964453.09FU;k:Fungi,p:Ascomycota,c:Arthoniomycetes,o:Lichenostigmatales,f:Phaeococcomycetaceae

AAGTCGTAACAAGGTCTCCGTAGGTGAACCTGCGGAGGGATCATTAATGAGATAGGGTCT

TCACGGCCCGACCTCCAACCCAATGTCTACCATACCTCTGTTGCCTCGGCGGGCCGCCGG

CGTCCTTTACCGGGCGTCGCCGTCGGCTCTAGGGCTCTCGAGCGCCCGCCGCAGGACCGA

TTAAACTCTTTTTAAAACAAGTCTTCTGAGTGGGAAATTAAATTTATTAAAACTTTTAAC

AACGGATCTC

>ASV186 GS|86.3|AF250779|SH0954634.09FU;k:Fungi,p:Ascomycota,c:Lecanoromycetes,o:Caliciales,f:Caliciaceae,g:Buellia,s:Buellia frigida

AAGTCGTAACAAGATTTTCGTTGATGAATTTGCGAAAGAATCATTATCGAGAGACGAGAT

CGCTTCGATCTCACTCTTCACTCGTGTCTACTTATCTTTATTGCTTTGACGAGTCTTCGA

GCTTGATCGTTCGACGCGAATGAGAGACTTTCGTCTCTCGTCTCGCGAGCGCTCGCCAAA

GACTTTGTTAACTCTATTTTAGTGTCTTTCGAGCAATCATGTAATAGTTAAAACTTTCAA

CAACGAATCT

>ASV187 GS|82.8|AF250779|SH0954634.09FU;k:Fungi,p:Ascomycota,c:Lecanoromycetes,o:Caliciales,f:Caliciaceae,g:Buellia,s:Buellia frigida

AGGTCGTAACAAGATTTTCGTAGATGAATTTGCGAAAGAATCATTATCGAGAGACGAGAT

CGCTTCGATTTCACTCTTCACTCGTGTCTACTTATCTTTGTTGCTTTGACGAGTCTTCGA

ACTTGATCGTTCGACGCAAGTGAAAGACTTTCGTCTCTCGTCTTGCGAGCGCTCGTCAAA

GATCGTGTTAACTTTGTTTTAGTGTCTTTCGAGCAATCATATAATAATTAAAACTTTCAA

CAACGAATTT

>ASV188 SS|0.9600|OL603994|SH0942717.09FU;k:Fungi,p:Ascomycota,c:Lecanoromycetes,o:Lecanorales

AAGTCGTAACAAGGTTTCCGTAGGTGCAGTCCAACGTTCGCCTGTGGGAACCGTGCAGGA

TCGACTCTAAATCAACTGCTCCAGTCCGCCCGTGGTTAGGGTTGGCGACGCCATCAGTCT

GCGCTGGGAGGGTCCTTTCGGGGTCAACCAGCAGCTCCTCTAGGGAGTCCACAGATCAAA

CGACGGCGGCCACAAGATCGTGGTTTAGATATGATCGGCCCCAGCCATCATTGGCTGGCG

ATATGATAGC

>ASV189 GS|0.0|None;No hit

GTGTCAGCAGCCGCGGTAATACGGAGGATGCAAGCGTTATCCGGAATGATTGGGCGTAAA

GCGTCTGTAGGTGGGTTGTAAAGTCTTCTGTTAAAGATCGGGGCTTAACCCAGTTTAAGC

AGTGGAAACTTATAACCTAGAGTACGGTAGGGGCAGAGGGAATTCCCGGTGTAGCGGTGA

AATGCGTAGATATCGGGAAGAACACCGACGGCGAAAGCACTCTGCTGGGCCGAAACTGAC

ACTGAGAGAC

>ASV190 GS|98.0|KP903330|SH0989164.09FU;k:Fungi,p:Ascomycota,c:Dothideomycetes,o:Mycosphaerellales,f:Teratosphaeriaceae,g:Catenulostroma

AAGTCGTAACAAGGTCTCCGTAGGTGAACCTGCGGAGGGATCATTACCGAGCGAGGGTCC

TCCGGGCCCGACCTCTCAACCCCATGTGACCCGACACCGTTGCCTCGGGGGCGACCCGGC

CGTCTCGGCCGGGGCCCCCGGTGGACCGACCAAACACTGCATCTGTGCGTCCGAGTCCCA

TGATAAATCAATCAAAACTTTCAACAACGGATCTCTTGGTTCTG

>ASV191 GS|89.2|AF250779|SH0954634.09FU;k:Fungi,p:Ascomycota,c:Lecanoromycetes,o:Caliciales,f:Caliciaceae,g:Buellia,s:Buellia frigida

AAGTCGTAACAAGGTTTTCGTAGGTGAATCTACGAAAGAATCATTATCGAGAGACGAGGT

CGCTTCGACCCCACTCTTCATTCGTGTCTACTTACCTTTGTTGCTTTGGCGAGTTTTCGA

GCTTGATCGTTCGACGCGGGTGGAAGACTTTCGTCTCTCGCCTCGCGAGCGCTCGTCAAA

GGTCTTGTTAACTCTATTTTAGTGTCTTTCGAGCAATCATGTAATAATTAAAACTTTCAA

CAACGAATCT

>ASV192 SS|0.8300|JX042758|SH1087522.09FU;k:Fungi,p:Basidiomycota,c:Tremellomycetes,o:Tremellales,f:Cryptococcaceae,g:Cryptococcus

AAGTCGTAACAAGGTTTCCGTAGGTGAACCTGCGGAAGGATCATTAGTGATTTGGCCTCT

GGCCATTTTCTTGACCAACCTCTGTGAACCGTGGCCTCCGGGCCGTATTTAAACATCAGT

GTAAAGAATGTAATCTATCGAAACAAAAACAAAACTTTCAACAACGGATCTCTTGGCTCT

C

>ASV193 GSL|100.0|OL334731|SH1223465.09FU;k:Fungi,p:Basidiomycota,c:Cystobasidiomycetes,o:Cystobasidiales,f:Cystobasidiaceae,g:Cystobasidium,s:Cystobasidium laryngis

AAGTCGTAACAAGGTTTCCGTAGGTGAACCTGCGGAAGGATCATTAATGAATTTTAGGAC

TCTCTTTTTAGAGGTCCGACCCTTTCATTTCCATACACTGTGCACACACTTCTTTTCACA

CATTTTAACACTATAGTATAAGAATGTAACAGTCTCTTTATTGAGCATAAATAAAAATAA

AACTTTCAGCAACGGATCTCTTGGCTCTC

>ASV194 SS|0.9900|AY667583|SH0954634.09FU;k:Fungi,p:Ascomycota,c:Lecanoromycetes,o:Caliciales,f:Caliciaceae,g:Buellia,s:Buellia frigida

AAGTCGTAACAAGGTTTCCGTAGATGAACCTGCGAAAGAATCATTATCGAGAGACGGGGT

CGCTTCGACCTCACTCTTCACTCGTGTCTACTTATCTTTGTTGCTTTGGCGGGCCTTCGA

GCTTGATCGTTCGACGCGAATGGGAGGCTTTCGCCTCTCGCCTCGCGAGCGCTCGTCAAA

GGCCTTGTTAACTTTGTTTTAGTGTCTTCCGACCAATCATGTAATAATTAAAACTTTCAA

CAACGAATCT

>ASV195 GS|85.3|AF250779|SH0954634.09FU;k:Fungi,p:Ascomycota,c:Lecanoromycetes,o:Caliciales,f:Caliciaceae,g:Buellia,s:Buellia frigida

AAGTCGTAACAAGATTTTCGTAGATGAATTTGCGAAAGAATCATTATCGAGAGACGAGAT

CGCTTCGATCTCACTCTTCATTCGTGTCTACTTATCTTTGTTGCTTTGACGAGTCTTCGA

ACTTGATCGTTCGACGCGAGTGAGAGGCTTTCGTCTCTCGTTTCGCGAGCGCTCGTCAAA

GATTTTGTTAACTCTATTTTAGTGTCTTTCGAGCAATCATGTAATAGTTAAAACTTTCAA

CAACGAATCT

>ASV196 SS|1.0000|AF250779|SH0954634.09FU;k:Fungi,p:Ascomycota,c:Lecanoromycetes,o:Caliciales,f:Caliciaceae,g:Buellia,s:Buellia frigida

AAGTCGTAACAAGGTTTTCGTAGATGAATTTGCGAAAGAATCATTATCGAGAGACGAAAT

CGCTTCGATCCCACTCTTCACTCGTGTCTACTTACCTTTGTTGCTTTGACGAGCCTTCGA

GCTTGATCGTTCAACGCGAGTGGAAGGCTTTCGCCTCTCGCCTCGCGAGCGCTCGCCAAA

GGCCCTGTTAACTTTGTTTTAGTGTCTTTCGACCAACCATGTAATAGTTAAAACTTTCAA

CAACGAATCT

>ASV197 GSL|100.0|KF309982|SH1001330.09FU;k:Fungi,p:Ascomycota,c:Dothideomycetes,o:Mycosphaerellales,f:Extremaceae,g:Vermiconia,s:Vermiconia antarctica

AAGTCGTAACAAGGTCTCCGTAGGTGAACCTGCGGAGGGATCATTACCGAGTGAGGGTGG

AAACACCCGACCTCCAACCCCATGTCGTTACAACCTTTGTTGCCTCGGGGGCGACCCGGC

CTCGCGCCGGGGCCCCCGATGGACCATCTCACTCTGCGTCTTTGCGTCGGAGTCACAAGT

AAATTGAATCAAAACTTTTAACAACGGATCTCTTGGTTCTG

>ASV198 SS|0.8900|JF357909|SH1107865.09FU;k:Fungi,p:Ascomycota,c:Lecanoromycetes

AAGTCGTAACAAGGTTTCCGTAGGTGAACCTGCGGAAGGATCATTATCGAGAGGGGGGCT

CCATGCCCCGGGGCTCTGTCCCCGTACCTTTTCACCCTGTGTGTATTTTTCCCCCGTTGC

TTTGGCGGGCCCCGGGTCTTCCCCCGGCGTTGGCCCCCTCGCGGGGTTCGCGAGCGCCCG

CCGAAGGCTCATCAAAACTCTGTTGATCAGTGCAGTCTGAGCGTACGAACAATAAATTAA

AACTTTCAAC

>ASV199 GS|90.7|AF250779|SH0954634.09FU;k:Fungi,p:Ascomycota,c:Lecanoromycetes,o:Caliciales,f:Caliciaceae,g:Buellia,s:Buellia frigida

AAGTCGTAACAAGATTTTCGTAGATGAATTTGCGAAAGAATCATTATCGAGAGACGAGAT

CGCTTCGATCTCACTCTTCACTCGTGTCTACTTATCTTTGTTGCTTTGACGAGTCTTCGG

GCTTGATCGTTCGACGCGGGTGGGAGGCTTTCGTCTCTCGTCTCGCGAGCGCTCGTCAAA

GACCCTGTTAACTCTGTTTTAGTGTCTTTCGAGCAATCATGTAATAGTTAAAACTTTCAA

CAACGGATCT

>ASV200 GS|89.7|AF250779|SH0954634.09FU;k:Fungi,p:Ascomycota,c:Lecanoromycetes,o:Caliciales,f:Caliciaceae,g:Buellia,s:Buellia frigida

AAGTCGTAACAAGATTTTCGTAGATGAACTTGCGAAAGAATCATTATCGAGAGACGAGAT

CGCTTCGATCTCACTCTTCACTCGTGTCTACTTACCTTTGTTGCTTTGACGAGCCTTCGA

ACTTGATCGTTCGACGCGAGTGGAAGGCTTTCGTCTCTCGTCTCGCGAGCGCTCGCCAAA

GGCCTTGTTAACTCTGTTTTAGTGTCTTTCGAGCAATCATGTAATAGTTAAAACTTTCAA

CAACGAATCT

>ASV201 GS|88.2|AF250779|SH0954634.09FU;k:Fungi,p:Ascomycota,c:Lecanoromycetes,o:Caliciales,f:Caliciaceae,g:Buellia,s:Buellia frigida

AAGTCGTAACAAGATTTTCGTAGATGAATCTGCGAAAGAATTATTATCGAGAGACGAGAT

CGCTTCGATCCCACTCTTCACTCGTGTCTACTTATCTTTGTTGCTTTGACGAACCTTCGA

GCTTGATCGTTCGACGCGAGTGAGAGGCTTTCGTCTCTCGTCTCGCGAGCGCTCGTCAAA

GATCTTGTTAACTCTGTTTTAGTGTCTTTCGAGCAATCATGTAATAGTTAAAACTTTCAA

CAACGAATCT

>ASV202 GS|99.6|AY667583|SH0954634.09FU;k:Fungi,p:Ascomycota,c:Lecanoromycetes,o:Caliciales,f:Caliciaceae,g:Buellia,s:Buellia frigida

AAGTCGTAACAAGGTTTCCGTAGGTGAACCTGCGGAAGGATCATTACCGAGAGACGGGGT

CGCTTCGGCCCCACTCTTCACCCGTGTCTACTTACCTTTGTTGCTTTGGCGGGCCTTCGG

GCTTGACCGTTCGACGCGGGTAGGAGGCTTTCGCCTCTCGCCTCGCGAGCGCCCGCCAAA

GGCCCTGTTAACTCTGTTTTAGTGTCTTCCGAGCAACCATGTAATAGTTAAAACTTTCAA

CAACGGATCT

>ASV203 GS|100.0|MZ919293|SH0903303.09FU;k:Fungi,p:Ascomycota,c:Lecanoromycetes,o:Caliciales,f:Physciaceae,g:Physcia,s:Physcia caesia

AAGTCGTAACAAGGTTTCCGTAGGTGAAGGTTTGCCTACCGAAACCCCTCGCAGCATCGA

CTCTAAATACATGCGCGGCTTGCACCTTTAGGTGCAAGCGACACCGTTCAAGTGCTGAGA

GGGTTTCGGGCTGTGAGCCCGATCCGACCAGCAGCCAGCTTTCACAGTTGGTTCACAGAT

CAATCGACGGTGGCCTTATGGCTAAAATATGACCGGTCCCCGGCCTCTGGCCCTGGATTT

TGCGGAACCT

>ASV204 GS|97.1|MF138060|SH0954356.09FU;k:Fungi,p:Ascomycota,c:Lecanoromycetes,o:Acarosporales,f:Acarosporaceae,g:Acarospora,s:Acarospora gwynnii

AAGTCGTAACAAGGTTTCCGTAGGTGAACCTGCGGAAGGATCATTACAGAGTTAGGGTCT

TTCCAGGCCCGACCTCCAACCCTATGTGTACCTACCTTTGTTGCTTTGGCGGGCCCGTTG

GGTGACCCACCGGTGGCCTCTGGCTCCCGAGTGCCCGTCAGAGACCCATCAAAACCCGTT

AATTGTGTCGTCTGAGTACCACTTTAATAATTAAAACTTTCAACAACGGATCTCTTGGTT

CTG

>ASV205 GS|88.2|AF250779|SH0954634.09FU;k:Fungi,p:Ascomycota,c:Lecanoromycetes,o:Caliciales,f:Caliciaceae,g:Buellia,s:Buellia frigida

AAGTCGTAACAAGATTTTCGTAGATGAACTTGCGAAAGAATCATTATCGAGAGACGAGGT

CGCTTCGACCTCACTCTTCACTCGTGTCTACTTATCTTTGTTGCTTTGGCGAGCCTTCGA

GCTTGATCGTTCGACCTGAGTGAAAGACTTTCGTCTCTCGTCTCGCGAGCGCTCGTCAAA

GATCTTGTTAACTCTGTTTTAGTGTCTTTCGAGCAACCATGTAATAGTTAAAACTTTCAA

CAACGAATCT

>ASV206 GS|99.5|JN873879|SH1123218.09FU;k:Fungi,p:Ascomycota,c:Lecanoromycetes,o:Lecanorales,f:Lecanoraceae,g:Lecanora,s:Lecanora physciella

AAGTCGTAACAAGGTTTCCGTAGGTGAACCTGCGGAAGGATCATTACCGAGAGCGGGGCT

AACCCCCTAAACTCCGCCGCCGAAAGGGGTACTCTCCACCCTATGTATACATATACCACT

CTCGCTTTGGCGGGCTGAAGGCTCTTGCCCTACGCCCGCCAGTGGCTCAAAAAATTCTGT

TTATCAGTGATGTCCGAGTAAAAACCTAATAGTTAAAACTTTCAACAACGGATCTCTTGG

TTCTG

>ASV207 GS|79.2|AY667583|SH0954634.09FU;k:Fungi,p:Ascomycota,c:Lecanoromycetes,o:Caliciales,f:Caliciaceae,g:Buellia,s:Buellia frigida

AAATCGTAACAAAATTTTCGTAAATGAATTTGCGAAAGAATCATTATCGAAAGACGAAGT

CGCTTCGATTTCACTCTTCACTCGTATCTACTTATCTTTGTTACTTTGACGAGTTTTCGA

ATTTGATTATTCGACGCGAGTGAGAGACTTTCGTCTCTCGTTTCGCAAGCGTTCGTCAAA

GATCTTGTTAACTTTGTTTTAATGTCTTTCAAGCAATCATGTAATAGTTAAAACTTTTAA

CAACGAATCT

>ASV208 SS|0.9700|KT714247|SH1142646.09FU;k:Fungi,p:Ascomycota,c:Lecanoromycetes,o:Caliciales,f:Caliciaceae,g:Buellia

AAGTCGTAACAAGGTTTCCGTAGGTGAAGTGACCTCCTTTGGTTCGCCTGCATCAACCCT

CGAGCGACTATAAAAAAGTGTTAGTCGGGTGCTTCGTCCCCGGCCACGCGGCTATTCTGC

CCTGGGAGCAAGCTTCGTCGCAAGATCGAGTTGCAACCAGCAGCCCAATTCTCTCTACGA

CGGGACTGGGTTCACAGATCAAATGCCTGCGGCCACGCTTCAGTGGTTCTAATATGACCG

ATTCCCGCCA

>ASV209 GSL|100.0|KC455918|SH1223465.09FU;k:Fungi,p:Basidiomycota,c:Cystobasidiomycetes,o:Cystobasidiales,f:Cystobasidiaceae,g:Cystobasidium,s:Cystobasidium laryngis

AAGTCGTAACAAGGTTTCCGTAGGTGAACCTGCGGAAGGATCATTAATGAATTTTAGGAC

TCTCTTTTTAGAGGTCCGACCCCTTCATTTCCATACACTGTGCACACACTTCTTTTCACA

CATTTTAACACTATAGTATAAGAATGTAACAGTCTCTTTATTGAGCATAAATAAAAATAA

AACTTTCAGCAACGGATCTCTTGGCTCTC

>ASV210 GS|0.0|None;No hit

AAGTCGTAACAAGGTTTTCGTAGGTGAACCTGCGGAAGGATCATTACCAGATGGGGCCTG

CGGGCCTTTTTCTCCTTACCACGTGCACCGAGTTGTCGGGAGACGCTTGTTTCCCGACCA

ATTCTTCACGCTTGTTTTGTTGTCTGAGTCGAATGACCATAATTGAAAAGAAAACTATGA

ACAACGGATCTCTAGGCTCTT

>ASV211 GS|99.2|AY667583|SH0954634.09FU;k:Fungi,p:Ascomycota,c:Lecanoromycetes,o:Caliciales,f:Caliciaceae,g:Buellia,s:Buellia frigida

AAGTCGTAACAAGGTTTCCGTAGGTGAACCTGCGGAAGGATCATTACCGAGAGACGGGGT

CGCTTCGGCCCCACTCTTCACCCGTGTCTACTTACCTTTGTTGCTTTGGCGGGCCTTCGG

GCTTGACCGTGCGACGCGGGTGGGAGGCTTTCGCCTCTCGCCTCGCGAGCGCCCGCCAAA

GGCCCTGTTAACTCTGTTTTAGTGTCTTCCGAGCAACCATGTAATAGTTAAAACTTTCAA

CAACGAATCT

>ASV212 SS|0.9900|AF250779|SH0954634.09FU;k:Fungi,p:Ascomycota,c:Lecanoromycetes,o:Caliciales,f:Caliciaceae,g:Buellia,s:Buellia frigida

AAGTCGTAACAAGGTTTTCGTAGATGAACTTGCGAAAGAATCATTATCGAGAGACGAGAT

CGCTTCGACCCCACTCTTCACTCGTGTCTACTTATCTTTGTTGCTTTGACGAGCCTTCGG

GCTTGACCATTCGACGCGAGTGAGAGGCTTTCGTCTCTCGCCTCGCGAGCGCCCGCCAAA

GGCCCTGTTAACTCTATTTTAGTGTCTTTCGAGCAACCATGTAATAGTTAAAACTTTCAA

CAACGAATCT

>ASV213 SS|0.9100|MT809481|SH1325656.09FU;k:Fungi,p:Ascomycota

AAGTCGTAACAAGGTTTCCGTAGGTGAACCTGCGGAAGGATCATTACCGAGCTAGGGTCT

CTGGCCCGACCTCCAACCCCACGTGTACAGAACCTTTGTTGCTTTGGCGGGCCGTTGGGT

GCAAACCCGCCCCGGGCCTTCGTGGCTCGCGTGTGCCCGCCAGGGGACCCTTTGAACCCG

ATATGTCCCTGCCGTCTGAGTGGGATTCAAATCAAGCAAAACTTTCAACAACGGATCTCT

TGGTTCTG

>ASV214 GS|0.0|None;No hit

AAGTCGTAACAAGGTTTCCGTAGGTAAGTTATCGAAACACCCCCCCAATATATCCCCTAT

ACGCTTATGCTAACCCCCCTACCCAGGTGAACCTGCGGAAGGATCATTAAAGAATAAACG

CTCGTTAGGGGGGGTCCCCCGGGGCCCCCTCCCGCGTCCGTATACTCTAAACCCCCTGTC

TACCGACCTCACACAAAACCAGTACAACCTCACAAAAGTCGTCCCTAGCGGGGCGCGCTA

CACTGAATGT

>ASV215 GS|99.5|UDB01890890|SH1188820.09FU;k:Fungi,p:Basidiomycota,c:Tremellomycetes,o:Filobasidiales,f:Filobasidiaceae,g:Naganishia

AAGTCGTAACAAGGTTTCCGTAGGTGAACCTGCGGAAGGATCATTAATGAATACAGATGC

CTGTCGAGCTTGCTCACGGGCTTTCTATCATATCCATAACACCTGTGCACTTGTTGGATG

TTCTAGAGACTTAGGGTTAAACCTGCAGGTAATAGTCATCCACTTACACTAAACAATAAT

GTAACAAATGTAGTCTTATTATAACAAAATAAAACTTTCAACAACGGATCTCTTGGCTCT

C

>ASV216 SS|1.0000|AF250779|SH0954634.09FU;k:Fungi,p:Ascomycota,c:Lecanoromycetes,o:Caliciales,f:Caliciaceae,g:Buellia,s:Buellia frigida

AAGTCGTAACAAGGTTTTCGTAGATGAATTTGCGAAAGGATCATTATCGAGAGACGAGAT

CGCTTCGACCCCACACTCTTCACTCGTGTCTACTTACCTTTGTTGCTTTGGCGGGCCTTC

GGGCTTGATCGTTCGACGCGAGTGGGAGGCTTTCGCCTCTCGCCTCGCGAGCGCTCGTCA

AAGACCCTGTTAACTCTGTTTTAGTGTCTTTCGAGCAACCATGTAATATTTAAAACTTTC

AACAACGGAT

>ASV217 SS|1.0000|KC965222|SH0962008.09FU;k:Fungi

AAGTCGTAACAAGGTTTCCGTAGGTGAACCTGCGGAAGGATCATTAAAGAGTCGGCCCCC

CCAACGTGGGAGGTCTCGACCCTACACCCCCCTGTTTACCGACCTCACACGAACTCTGTA

TACAGACACGAAGTCGTCCTTAGCGGGGCGCACGACCACAAAAACGTATAACAAAATTAA

AACTTTCAACAACGGATCTCTTGGTTCTG

>ASV218 GS|99.0|AF250779|SH0954634.09FU;k:Fungi,p:Ascomycota,c:Lecanoromycetes,o:Caliciales,f:Caliciaceae,g:Buellia,s:Buellia frigida

AAGTCGTAACAAGATTTCCGTAGGTGAACCTGCGAAAGAATCATTACCGAGAGACGGGGT

CGCTTCGGCCCCACTCTTCACCCGTGTCTACTTACCTTTGTTGCTTTGGCGGGCCTTCGG

GCTTGACCGTTCGACGCGGGTGGAAGGCTTTCGCCTGTCGCCTCGCGAGCGCCCGCCAAA

GGCCCTGTTAACTCTGTTTTAGTGTCTTCCGAGCAACCATGTAATAGTTAAAACTTTCAA

CAACGGATCT

>ASV219 GS|0.0|None;No hit

GTGTCAGCAGCCGCGGTAATACGTGGAAGACTAGTGTTATTCATCTTTAATAGGTTTAAA

GGGTACCTAGACGGTATTTCTAGCCCAAAAAGGGGTACGGATTTACTAGAGTTTTATGTG

AGGAGGGGAGTACTTGTGGAGTAAAGATGAAATTTTTTTATACTATGAGGAAACTGGTAG

CGGCGAAAGCAACCTTCTATGTATAAACTGACGTTGAGGGACGAAGGCTTGGGTAGCAAA

TAGGATTAGA

>ASV220 GS|99.6|AF281306|SH1300522.09FU;k:Fungi,p:Ascomycota,c:Lecanoromycetes,o:Teloschistales,f:Teloschistaceae,g:Xanthoria,s:Xanthoria elegans

AAGTCGTAACAAGGTTTCCGTAGGTGAACCTGCGGAAGGATCATTACTAAGAGAGGGATG

TACGCTTCCAGCCGAGTCCCGGGGGGCTGCGCCCTTCACCTCTTCAACCCTGTGTCTACC

AACCGCTGTTGCTTCGGCGAGCGTCGGGGCGTCCGCGCCCCGGCCCCGGCTTCGGTCGGT

GAGCTCTCGCAGAGGCCTATCTTTATTCTGTTTTGCAGTGACGTCCGAGAATACCAATAT

AATCAATCAA

>ASV221 GS|100.0|UDB05166137|SH0954236.09FU;k:Fungi,p:Ascomycota,c:Lecanoromycetes,o:Acarosporales,f:Acarosporaceae,g:Polysporina,s:Polysporina subfuscescens

AAGTCGTAACAAGGTTTCCGTAGGTGAACCTGCGGAAGGATCATTACAGAGTTAGGGTCT

CTTCAGGCCCGATCTCCAACCCTTTGTGTACCTACCTTTGTTGCTTTGGCGGGCCCGCTG

GGGCGACCCACCGGCGGCCTTTGGCTCCCGAGTGCCCGTCAGAGACCCTTTCAAACCTGT

CAATTGTGTCGTCTGAGTACCAACTTAATAATTAAAACTTTCAACAACGGATCTCTTGGT

TCTG

>ASV222 GSL|100.0|FJ236008|SH1188820.09FU;k:Fungi,p:Basidiomycota,c:Tremellomycetes,o:Filobasidiales,f:Filobasidiaceae,g:Naganishia

AAGTCGTAACAAGGTTTCCGTAGGTGAACCTGCGGAAGGATCATTAATGAATACAGATGC

CTGTCGAGCTTGCTCACGGGCTTTCTATCATATCCATAACACCTGTGCACTTGTTGGATG

TCTAGAGACGTAAAGTCGACAGTCATCCACTTACACTAAACAATAATGTAACAAATGTAG

TCTTATTATAACAAAATAAAACTTTCAACAACGGATCTCTTGGCTCTC

>ASV223 GS|89.2|AF250779|SH0954634.09FU;k:Fungi,p:Ascomycota,c:Lecanoromycetes,o:Caliciales,f:Caliciaceae,g:Buellia,s:Buellia frigida

AAGTCGTAACAAGATTTTCGTAGATGAACTTGCGAAAGAATCATTATCGAGAGACGAGGT

CGCTTCGACCTCACTCTTCACTCGTGTCTACTTATCTTTGTTGCTTTGGCGAGCCTTCGA

GCTTGATCGTTCGACGCGAGTGAAAGACTTTCGTCTCTCGTCTCGCGAGCGCTCGTCAAA

GATCTTGTTAACTCTGTTTTAGTGTCTTTCGAGCAACCATGTAATAGTTAAAACTTTCAA

CAACGAATCT

>ASV224 GS|97.1|KC179119|SH1085530.09FU;k:Fungi,p:Ascomycota,c:Lecanoromycetes,o:Teloschistales,f:Teloschistaceae,g:Shackletonia,s:Shackletonia insignis

AAGTCGTAACAAGGTTTCCGTAGGTGAACCTGCGGAAGGATCATTATCGAGAGCGGAGCT

TCGCGCCCCCGGGGGGCTTCGGCCTCCTACCTCTTCACCCTGTGTATATCAACCTCTGTT

GCTTTGGCGAGCCCTGGGTTTATACCCGGCACGGTTCCGGGCGATCCTGCCCGGAGTCGC

GGGTGCTCGCCCGAGGCCTTTAACCATCTGTTTACGTGGAACGTCCGAATGACCCAAAAA

TTAAATAAAA

>ASV225 GS|85.3|AF250779|SH0954634.09FU;k:Fungi,p:Ascomycota,c:Lecanoromycetes,o:Caliciales,f:Caliciaceae,g:Buellia,s:Buellia frigida

AAGTCGTAACAAGATTTTCGTAAATGAATCTACGAAAGAATCATTATCGAGAGACGAGAT

CGCTTCGATCTCACACTCTTCACTCGTGTCTACTTATCTTTGTTGCTTTGACGAGTCTTC

GAGCTTGATCGTTCGACGCGAGTGAGAGATTTTCGTCTCTCGTCTCGCGAGCGCTCGTCA

AAGGCTCTATTAACTTTGTTTTAGTGTCTTTCGAGCAATCATGTAATATTTAAAACTTTC

AACAACGAAT

>ASV226 SS|0.9000|MZ229889|SH0913114.09FU;k:Fungi,p:Ascomycota,c:Lecanoromycetes

AAGTCGTAACAAGGTTTCCGTAGGTGAACCTGCGGAAGGATCATTATCGAGAGACGGAGT

CTAACCGGCCCCACTCTTCACCCGTGTATACCTACCCTTTGTTGCTTTGGCGGGCGGTCG

GTCTTTACCGCCCGACGTCGGTCGGAAGGCTCGCGCCTTCCACTCACCGAACGCCCGCCA

GAGGCCCCCCAAACCCGTCCATCTGTGTCCGAGCGAGTGAAGCAAATAGTTAAAAACTTT

CAACAACGGA

>ASV227 SS|1.0000|UDB06403837|SH1047739.09FU;k:Fungi

AAGTCGTAACAAGGTTTCCGTAGGTGAACCTGCGGAAGGATCATTAGTGAACGCCTCCGG

GCTTATCCAACCAACCTCTGTGAACCGTGGCCTCCGGGCCCTTACAAACATCAGTGTCCT

GAACGTAACAATGTATAATCAAAACAAAACTTTCAACAACGGATCTCTTGGCTCTC

>ASV228 GS|87.3|AF250779|SH0954634.09FU;k:Fungi,p:Ascomycota,c:Lecanoromycetes,o:Caliciales,f:Caliciaceae,g:Buellia,s:Buellia frigida

AAGTCGTAACAAGATTTCCGTAGATGAATCTACGAAAGAATCATTATCGAGAGACGAGAT

CGCTTCGATCCCACTCTTCACTCGTATCTACTTATCTTTGTTGCTTTGACGAGTCTTTGA

GCTTGATCGTTCGACGCGAATGGGAGGCTTTCGTCTCTCGCCTCGCGAGCGCTCGTCAAA

GACTCTATTAACTTTGTTTTAGTGTCTTTCGAGCAATCATGTAATAGTTAAAACTTTCAA

CAACGAATCT

>ASV229 GS|99.0|KC179119|SH1085530.09FU;k:Fungi,p:Ascomycota,c:Lecanoromycetes,o:Teloschistales,f:Teloschistaceae,g:Shackletonia,s:Shackletonia insignis

AAGTCGTAACAAGGTTTCCGTAGGTGAACCTGCGGAAGGATCATTATCGAGAGTGGGGCT

TCGCGCCCCCGGGGGGCTTCGGCCTCCTACCTCTTCACCCTGTGTATATCAACCTCTGTT

GCTTTGGCGAGCCCTGGGTTTACCCCCCGGCACGGTTTCGGGCGATCCTGCCCGGAGTCG

CGGGTGCTCGCCCGAGGCCTTTAACCATCTGTTTACGTGGAACGTCCGAATGACCCAAAA

ATTAAATAAA

>ASV230 SS|1.0000|FJ265755|SH1071004.09FU;k:Fungi

AAGTCGTAACAAGGTTTCCGTAGGTGAACCTGCGGAAGGATCATTACCGAGTTAGGGTTC

GCTCGTCGAGCCCGACCTCCCAACCCTTTGTTTATTATACCTTGTCGTTGCTTCGGCGGA

CCGGTCTCTCGACCGCCGGGGGTTATCGCCCCTGGAACGTGTCCGCCGACGGCCCAACCA

CAAACTCTTGCCCCCAAACCATGTCGCCTGAATTTACTTGATTAAAATCAAAAACAAAAC

TTTCAACAAC

>ASV231 GS|88.8|AY667583|SH0954634.09FU;k:Fungi,p:Ascomycota,c:Lecanoromycetes,o:Caliciales,f:Caliciaceae,g:Buellia,s:Buellia frigida

AAGTCGTAACAAGATTTCCGTAGGTGAACCTACGAAAGAATCATTATCGAGAGACGAGAT

CGCTTCGACCTCACTCTTCACTCGTGTCTACTTATCTTTGTTGCTTTGACGAGCCTTCGA

ACTTGATCGTTCGACGCGAATGAGAGACTTTCGTCTCTCGTCTCGCGAGCGCTCGTCAAA

GGTCCTGTTAACTCTGTTTTAGTGTCTTTCGAGCAATCATGTAATAGTTAAAACTTTCAA

CAACGAATCT

>ASV232 GS|99.2|AY667583|SH0954634.09FU;k:Fungi,p:Ascomycota,c:Lecanoromycetes,o:Caliciales,f:Caliciaceae,g:Buellia,s:Buellia frigida

AAGTCGTAACAAGGTTTCCGTAGGTGAACCTGCGGAAGGATCATTACCGAGAGACGGGGT

CGCTTCGGCCCCACTCTTCACCCGTGTCTACTTACCTTTGTTGCTTTGGCAGGCCTTCGG

GCTTGACCGTTCGACGCGGGTGGGAGGCTTTCGCCTCTCGCCTCGCGAGCGCCCGCCAAA

GGCCCTGTTAACTCTGTTTTAGTGTCTTCCGACCAACCATGTAATAGTTAAAACTTTCAA

CAACGGATCT

>ASV233 GS|81.2|AY667583|SH0954634.09FU;k:Fungi,p:Ascomycota,c:Lecanoromycetes,o:Caliciales,f:Caliciaceae,g:Buellia,s:Buellia frigida

AAGTCGTAACAAGATTTTCGTAAATGAATTTACGAAAAAATCATTATCGAGAGACGAAAT

CGCTTCGATCTCATTCTTCATTCGTATCTACTTATCTTTGTTGCTTTGACGAGTCTTCGA

GCTTGATCGTTCGACGCGAGTGAAAGACTTTCGTCTCTCGTTTCGCGAGCGCTCGTCAAA

GATTCTATTAACTTTGTTTTAGTGTCTTTCGAGCAATCATATAATAATTAAAATTTTCAA

CAACGAATCT

>ASV234 GS|84.8|AF250779|SH0954634.09FU;k:Fungi,p:Ascomycota,c:Lecanoromycetes,o:Caliciales,f:Caliciaceae,g:Buellia,s:Buellia frigida

AAATCGTAACAAGATTTTCATAAATGAATCTACGAAAGAATCATTATCGAGAGACGAGAT

CGCTTCGATTTCACTCTTCATTCGTATCTACTTATCTTTGTTGCTTTGACGAGTCTTCGA

GCTTGATCGTTCGACGCGAGTGAGAGACTTTCGTCTCTCGTTTCGCGAGCGCTCGTCAAA

GACTCTATTAACTCTATTTTAGTGTCTTTCGAGCAATCATGTAATAGTTAAAACTTTCAA

CAACGAATCT

>ASV235 GS|99.0|UDB01571356|SH0913113.09FU;k:Fungi,p:Ascomycota,c:Lecanoromycetes,o:Caliciales,f:Caliciaceae,g:Buellia

AAGTCGTAACAAGGTTTCCGTAGGTGAACCTGCGGAAGGATCATTACCGAGAGACGGGGT

CCAACCGGCCCCACTCTTCACCCGTGCGAACCTACCTATGTTGCTTTGGCGGGCCTCCTG

CTCGGCGTCGGTCCGGGGCCTTTCGGGCTTCTTCCCGGCGAGTGCCCGCCGAAGGCTCTG

CAAACTCTTGTTTCGTGTCGTCCGAGTACAAACCTAATAGTTAAAACTTTCAACAACGGA

TCTCTTGGTT

>ASV236 GS|100.0|HQ287871|SH0942705.09FU;k:Fungi,p:Ascomycota,c:Lecanoromycetes,o:Lecanorales,f:Lecanoraceae,g:Lecidella,s:Lecidella greenii

AAGTCGTAACAAGGTTTCCGTAGGTGGTTTGCCTGTCGGATTCCCCCGCAGCGACTCTAA

AGAACTGCGCCAGTCGGGCTTCACCTCAGAGCCTGGCGACGCCATCAGTCTGGCTGGGAG

CCCCCTACGCGGGGGCTACCAGCAGCTCCTTCGGGAGTCCACAGATCAAACGATGAGCGG

CCGCAACACTGCGGTTCAGATATGACCGGCCCCAGCCTTCATCGGCTGGTGACTTCTTGC

GGAACCTGCG

>ASV237 GS|98.1|KP174855|SH1071027.09FU;k:Fungi,p:Ascomycota,c:Eurotiomycetes,o:Chaetothyriales,f:Trichomeriaceae,g:Knufia,s:Knufia separata

AAGTCGTAACAAGGTTTCCGTAGGTGAACCTGCGGAAGGATCATTACCGAGTTAGGGTCT

CTTCGGAGCCCGAACCTCCCAACCCTTTGTCTAATTTACCTTGTCGTTGCTTCGGCGGAC

CGGTTGACCAGCTGGTCTTGACCGCCGGGGGTCCCGCACCCCTGGAGAGCGTCCGCCGAC

GGCCCAACCACAAACTCTTGTACTAAACCATGTCGTCTGAATGTACTTGATATTAATCAA

AAAAACAAAA

>ASV238 GS|97.0|JN873879|SH1123218.09FU;k:Fungi,p:Ascomycota,c:Lecanoromycetes,o:Lecanorales,f:Lecanoraceae,g:Lecanora,s:Lecanora physciella

AAGTCGTAATAAGGTTTCCGTAGGTAAACCTGCGGAAGGATCATTACCGAGAGCGGGGCT

AACCCCCTAAACTCCGCCGCCGAAAGGGGTACTCTCCACCCTATATATACATATACCACT

CTCGCTTTGGCGGGCTGAAGGCTCTTACCCTACGCCCGCCAGTAGCTCAAAAAATTCTGT

TTATCAGTAATATCCGAGTAAAAACCTAATAGTTAAAACTTTCAACAACGGATCTCTTGG

TTCTG

>ASV239 SS|0.8100|KF274472|SH0981989.09FU;k:Fungi,p:Ascomycota,c:Lecanoromycetes

AAGTCGTAACAAGGTTTCCGTAGGTGAACCTGCGGAAGGATCATTAAAGAGAAGGGGAGT

CTCGCGCTCCCCGGGGTCTCGGCCCCAACTCTTCACCCTCTGTATACCTACCTTTGTTGC

TTTGGCGGGCCTGGGGTCAAACTCATGCTGGTAACGTGCTTCGGCCCGTCGCCGGCGAGC

GCCCGCCAGAGGTCCTCAAAACTCCGATTTCAATGCAGTCTGAGCAAATATATATAAATA

AATAAAACTT

>ASV240 SS|1.0000|JN053511|SH1189082.09FU;k:Fungi,p:Basidiomycota,c:Tremellomycetes,o:Tremellales

AAGTCGTAACAAGGTTTCCGTAGGTGAACCTGCGGAAGGATCATTAGTGAACGGCCGGGA

GGCCTTAAACCCATCAAACCTCTGTGAACAGCGCCTTCGGGCATGCCCCTCGGGGCGATT

ACAAACAAAGAAGTCCAGAACGTAGACATTTTAAATGAAACACAACTTTCAACAACGGAT

CTCTTGGCTCTC

>ASV241 GS|84.3|AF250779|SH0954634.09FU;k:Fungi,p:Ascomycota,c:Lecanoromycetes,o:Caliciales,f:Caliciaceae,g:Buellia,s:Buellia frigida

AAGTCGTAATAAGATTTTCGTAGATGAATTTGCGAAAAAATCATTATCGAGAGACGAAAT

CGCTTCGATCTCATTCTTCACTCGTATCTACTTATCTTTGTTGCTTTGACGAGCTTTCGA

ATTTGATCGTTCGACGCAAGTGAAAGACTTTCGTCTCTCGTCTCGCGAGCGCTCGCCAAA

GGCTCTATTAACTCTATTTTAGTGTCTTTCGAGCAATCATGTAATAGTTAAAACTTTCAA

CAACGAATCT

>ASV242 SS|1.0000|KC965541|SH0962007.09FU;k:Fungi

AAGTCGTAACAAGGTTTCCGTAGGGTACGTTCACCCCTATAAACCCCCCCTTGCTACGTC

GAAAACGATTTGCTAACCCCCATTCTAGTGAACCTGCGGAAGGATCATTAAAGAGAAATC

GGGGTGTCGCCGCGAGGCTTTACCCCATCTCCCTAAACCCCCTGTCTACCGACCTCACAC

AAAACCACAGTACAAATATCAAGTCGTCCCTAGCGGGGCGCAACACCATAAATGTACAAA

AAACAAAACT

>ASV243 SS|0.9800|None;k:Fungi,p:Ascomycota,c:Lecanoromycetes,o:Lecanorales,f:Parmeliaceae,g:Neoprotoparmelia,s:Neoprotoparmelia corallifera

GTGTCAGCAGCCGCGGTAATACGTAGAAGACTAGTGTTATTCATCTTTAATAGGTTTAAA

GGGTACCTAGACGGTAAATCAAGCCCATATGGGGACTACTTTACTAGAGTTACTTATGAG

GGGGTATTAAAGTACTGCTGGTGTAGAGATGAAATTCTGTCATACCTCTTTCGTGGGAAA

ATTAATGGCACAGGTATAGGCGAAAGCATCCCCTTATGTGATAACTGACGTTGAAGGACG

AAGGCTTTGT

>ASV244 SS|0.8100|None;k:Fungi,p:Basidiomycota

AAGTCATAACAAGGTTTCCGTAGGTGAACCTGCGGAAGGATCATTCGAGAATAAGGCTTT

GGCCTTAATCCAATACAAACACCGTGAACCGACCCTCGGGACCTCGCAAGGGGGACCTTG

GATTACACTTTTAAACAGAGTTAAGAAAGGAAACATTATAAATACAAATAACTTTTAACA

ACGGATCTCTTGGCTCTC

>ASV245 GS|100.0|JX036093|SH0954224.09FU;k:Fungi,p:Ascomycota,c:Lecanoromycetes,o:Acarosporales,f:Acarosporaceae,g:Polysporina,s:Polysporina frigida

AAGTCGTAACAAGGTTTCCGTAGGTGAACCTGCGGAAGGATCATTACAGAGTTAGGGTCT

TTCAGGCCCGATCTCCAACCCTATGTGTATTTACCTCTGTTGCTTTGGCGGGCCCGCTGG

GAACCCCCACCGGTGGCCACTGGCTCCCGAGTGCCCGTCAGAGACCCATCAAATCCTGTT

AAATGTGTAGTCGGAGTATGAATACAATAATCAAAACTTTCAACAACGGATCTCTTGGTT

CTG

>ASV246 SS|0.9900|AF250779|SH0954634.09FU;k:Fungi,p:Ascomycota,c:Lecanoromycetes,o:Caliciales,f:Caliciaceae,g:Buellia,s:Buellia frigida

AAGTCGTAACAAGATTTCCGTAGGTGAATTTGCGAAAGAATCATTATCGAGAGACGAGAT

CGCTTCGACCTCACACTCTTCACTCGTGTCTACTTACCTTTGTTGCTTTGGCGGGCCTTC

GGGCTTGATCGTTCGACGCGGGTGAGAGACTTTCGCCTCTCGCCTCGCGAGCGCTCGTCA

AAGGCTCTGTTAACTCTGTTTTAGTGTCTTCCGAGCAATCATGTAATATTTAAAACTTTC

AACAACGGAT

>ASV247 GS|99.6|MW991411|SH0964445.09FU;k:Fungi,p:Ascomycota,c:Arthoniomycetes,o:Lichenostigmatales,f:Phaeococcomycetaceae,g:Antarctolichenia,s:Antarctolichenia onofrii

AAGTCGTAACAAGGTCTCCGTAGGTGAACCTGCGGAGGGATCATTAATGAGATAGGGTCT

TCACGGCCCGACCTCCAACCCAATGTCTACCATACCTCTGTTGCCTCGGCGGGCCGCCGG

CGTCCTTTGTTGGGCGCCGCCGTCGGCTTTAGGGCTTTCGAGCGCCCGCCGCAGGACCGA

TTAAACTCTTTTTAAAACAGGTCTTCTGAGTGGGAAATCAAATTTATTAAAACTTTTAAC

AACGGATCTC

>ASV248 GS|83.2|AF281307|SH0954634.09FU;k:Fungi,p:Ascomycota,c:Lecanoromycetes,o:Caliciales,f:Caliciaceae,g:Buellia,s:Buellia frigida

AAGTCGTAACAAGATTTTCGTAGATGAATCTACGAAAGAATCATTATCGAAAGACGAGAT

CGCTTCGACTTCACTCTTCACTCGTGTTTACTTATCTTTGTTGCTTTGACGAGTCTTCGA

GCTTGATCGTTCGACGCGAGTGAGAGACTTTCGTCTCTCGTTTTGCGAGCGCTCGTCAAA

GATCGTATTAACTTTGTTTTAATGTCTTTCGAGCAATTATGTAATAATTAAAACTTTCAA

CAACGAATCT

>ASV249 SS|0.9900|AF250779|SH0954634.09FU;k:Fungi,p:Ascomycota,c:Lecanoromycetes,o:Caliciales,f:Caliciaceae,g:Buellia,s:Buellia frigida

AAGTCGTAACAAGGTTTTCGTAGGTGAATCTACGAAAGGATCATTATCGAGAGACGGGGT

CGCTTCGGCCCCACACTCTTCACTCGTGTCTACTTATCTTTGTTGCTTTGACGAGCCTTC

GAGCTTGACCGTTCGACGCGAGTGGGAGGCTTTCGCCTCTCGTCTCGCGAGCGCCCGCCA

AAGATCCTGTTAACTCTGTTTTAGTGTCTTTCAAGCAATCATGTAATATTTAAAACTTTC

AACAACGAAT

>ASV250 GS|98.4|KF823589|SH1091255.09FU;k:Fungi,p:Basidiomycota,c:Tremellomycetes,o:Tremellales,f:Sirobasidiaceae

AAGTCGTAACAAGGTTTCCGTAGGTGAACCTGCGGAAGGATCATTAGTGATTTGGCCTCC

GGGCCTTCAATCCCATCCTCATACCTCTGTGAACCAGTTGGGCCCTCGGGCCTACCCTTT

CAAACACTGTGTAACGGACGTGATGTATATTATAAACCTAATAAAACTTTCAACAACGGA

TCTCTTGGCTCTC

>ASV251 GS|97.1|AF250779|SH0954634.09FU;k:Fungi,p:Ascomycota,c:Lecanoromycetes,o:Caliciales,f:Caliciaceae,g:Buellia,s:Buellia frigida

AAGTCGTAACAAGATTTCCGTAGGTGAACCTGCGAAAGAATCATTACCGAGAGACGAGAT

CGCTTCGACCCCACTCTTCACCCGTGTCTACTTACCTTTGTTGCTTTGACGGGCCTTCGG

GCTTGACCGTTCGACGCGAGTGGGAGGCTTTCGCCTCTCGCCTCGCGAGCGCCCGCCAAA

GGCCCTGTTAACTCTATTTTAGTGTCTTCCGAGCAACCATGTAATAGTTAAAACTTTCAA

CAACGGATCT

>ASV252 GS|0.0|None;No hit

GTGTCAGCAGCCGCGGTAATACGGGGGGGGCGAGCGTTATTCGAAATGATTGGGCGTAAA

GAGCACGTAGACGGTTTTTTAAGTGGACATTATATCTTTTTTGTTCTCTAAGGATAAAAA

AGAAAGGATTATGGAAATATTTCTGTACTCGGGAAAAAGACCAAGGCTCAACCATGGTGT

TTCCCGCCAAACTAAAAAACTAGAGTAAGTTAGAGGAAAGTGGAATTCCTGGAGGAAAGG

TTAAATTTTA

>ASV253 SS|0.9900|AF250779|SH0954634.09FU;k:Fungi,p:Ascomycota,c:Lecanoromycetes,o:Caliciales,f:Caliciaceae,g:Buellia,s:Buellia frigida

AAGTCGTAACAAGGTTTTCGTAGGTGAACTTGCGAAAGAATCATTATCGAGAGACGGGGT

CGCTTCGACCCCACTCTTCACTCGTGTCTACTTATCTTTGTTGCTTTGACGGGCCTTCGG

GCTTGATCGTTCGACGCGGGTGGGAGACTTTCGTCTCTCGTCTCGCGAGCGCTCGCCAAA

GGCCCTATTAACTCTGTTTTAGTGTCTTTCGAGCAACCATGTAATAGTTAAAACTTTTAA

CAACGAATCT

>ASV254 GS|97.5|AF250779|SH0954634.09FU;k:Fungi,p:Ascomycota,c:Lecanoromycetes,o:Caliciales,f:Caliciaceae,g:Buellia,s:Buellia frigida

AAGTCGTAACAAGATTTCCGTAGGTGAACCTGCGAAAGAATCATTACCGAGAGACGAGAT

CGCTTCGACCCCACTCTTCACCCGTGTCTACTTACCTTTGTTGCTTTGACGGGCCTTCGG

GCTTGACCGTTCGACGCGAGTGGGAGGCTTTCGCCTCTCGCCTCGCGAGCGCCCGCCAAA

GGCCCTGTTAACTCTGTTTTAGTGTCTTCCGAGCAACCATGTAATAGTTAAAACTTTCAA

CAACGGATCT

>ASV255 SS|0.9900|KF823589|SH1091255.09FU;k:Fungi

AAGTCGTAACAAGGTTTCCGTAGGTGAACCTGCGGAAGGATCATTAGGGATTTGGTCTCC

GGACCTTTTTCATATCCATAATACCCCTGTGAACTGTCGGTCCTCGGGCCAGCGTTTCCA

AACCATGTGTAATGAACGTGAATGTGTATGAAACCCTAGTAAAACTTTCAACAACGGATC

TCTTGGTTCTG

>ASV256 GS|99.6|MW991425|SH0964445.09FU;k:Fungi,p:Ascomycota,c:Arthoniomycetes,o:Lichenostigmatales,f:Phaeococcomycetaceae,g:Antarctolichenia,s:Antarctolichenia onofrii

AAGTCGTAACAAGGTCTCCGTAGGTGAACCTGCGGAGGGATCATTAATGAGATAGGGTCT

TCACGGCCCGACCTCCAACCCAATGTCTACCACACCTCTGTTGCCTCGGCGGGCCGCCGG

CGCCCTTTGTTGGGCGCCGCCGTCGGCTTTAGGGCTTTCGAGCGCCCGCCGCAGGACCGA

TTAAACTCTTTTTAAAACAAGTCTTCTGAGTGGGAAATCAAATTTATTAAAACTTTTAAC

AACGGATCTC

>ASV257 GS|84.8|AF250779|SH0954634.09FU;k:Fungi,p:Ascomycota,c:Lecanoromycetes,o:Caliciales,f:Caliciaceae,g:Buellia,s:Buellia frigida

AAGTCGTAACAAGATTTTCGTAGATGAATTTACGAAAAAATCATTATCGAAAGACGAAAT

CGCTTCGATCTCATTCTTCACTCGTATCTACTTATCTTTGTTGCTTTGACGAGTCTTCGA

GCTTGATCGTTCGACGCGAGTGAGAGACTTTCGTCTCTCGTCTTGCGAGCGCTCGTCAAA

GATCTTGTTAACTCTGTTTTAGTGTCTTTCGAGCAATCATGTAATAGTTAAAACTTTCAA

CAACGAATCT

>ASV258 GS|85.3|AF250779|SH0954634.09FU;k:Fungi,p:Ascomycota,c:Lecanoromycetes,o:Caliciales,f:Caliciaceae,g:Buellia,s:Buellia frigida

AAGTCGTAACAAGATTTTCGTAGATGAATCTACGAAAAGATCATTATCGAGAGACGAGAT

CGCTTCGATCTTACTCTTCACTCGTGTCTACTTATCTTTGTTGCTTTGACGAGTCTTCGA

GCTTGATCGTTCGACGCGAGTGAGAGACTTTCGTCTCTCGTCTCGCGAGCGCTCGTCAAA

GACTTTGTTAACTTTGTTTTAATGTCTTTCGAGCAATCATATAATAGTTAAAACTTTCAA

CAACGAATCT

>ASV259 GS|0.0|None;No hit

AAGTCGTAACAAGGTTTCGATCAGGTCGAACGGGAGATCCTTCCGTATGTACGCCTCGGA

AGCCTTTGCAGCCCCGCAAGGGGTCATGTCTGGCGCGACTTCCAAAATAATGACAGGCTC

CAGTCATCTTCTTCGTGGAGATCTGGATATGCAAGTCAGCTTGATGGCTGGCGACACTTT

CGAATTGCGGGGATCCCCTAAAGCTCTCCTCTACCAACCCATTCGAGAAATCGGATGGGC

ACTTGTGCCA

>ASV260 SS|0.8700|MK778057|SH1261224.09FU;k:Fungi,p:Ascomycota

AAGTCGTAACAAGGTTTCCGTAGGTGAACCTGCGGAAGGATCATTAACGAGAGGGGTCTC

CGGACTCTAGGGGCCTCGGCCCCTACCTCTTCACCCCATGTTGACCTACCTTTCTGCTTT

GGCGGGCCTTGAGGGTTCGCCTCATGTCGCCCGAGGGTACTCCTACCCCGGTGGCCCGTG

CCCGCCCGAGGCCTCTCCAACCCAGTTTATCCGCGACGTCCGAGCACACAGATAACAGTC

AAAACTTTCA

>ASV261 GS|98.6|UDB01571642|SH1041881.09FU;k:Fungi,p:Basidiomycota,c:Tremellomycetes,o:Tremellales,f:Bulleraceae

AAGTCGTAACAAGGTTTCCGTAGGTGAACCTGCGGAAGGATCATTAATGTAAACCCCTTG

TGGGGAAATACAAATCCACATACCTCTGTGAACCGTTGACCTCCGGGTCGTCTTTACAAA

CATCAGTGTAACGAACGTATACAAACATAAACAAAACAAAACTTTCAACAACGGATCTCT

TGGCTCTC

>ASV262 GS|88.7|AF250779|SH0954634.09FU;k:Fungi,p:Ascomycota,c:Lecanoromycetes,o:Caliciales,f:Caliciaceae,g:Buellia,s:Buellia frigida

AAGTCGTAACAAGGTTTTCGTAGATGAATCTACGAAAGAATCATTATCGAGAGACGAGAT

CGCTTCGATCTCACTCTTCACTCGTGTCTACTTATCTTTGTTGCTTTGACGAGTCTTCGA

ACTTGATCGTTCGACGCGAGTGAGAGGCTTTCGCCTCTCGTCTCGCGAACGCTCGTCAAA

GGCTCTGTTAACTCTGTTTTAGTGTCTTCCGAGCAATCATGTAATAGTTAAAACTTTCAA

CAACGAATCT

>ASV263 GS|98.4|MT236857|SH1289168.09FU;k:Fungi,p:Ascomycota,c:Eurotiomycetes,o:Chaetothyriales,f:Herpotrichiellaceae,g:Rhinocladiella

AAGTCGTAACAAGGTTTCCGTAGGTGAACCTGCGGAAGGATCATTACCGAGTTAGGGTCC

TCTGGGCTCGACCTCCTACCCTGTGTCTACCTTACCTTTTGTTGCTTCGGCGGATCCGTC

CCTTAGGGGACCGCCGGAGGGTTTCACCCCCCTCTGGCCCGCGTCCGCCGGTGGCCCACA

ACCAAAACTCTTGTTTAAACGTGTCGTCTAAGTAGAAAAACAAATAAGTAAAAACTTTCA

ACAACGGATC

>ASV264 GS|99.2|AY667583|SH0954634.09FU;k:Fungi,p:Ascomycota,c:Lecanoromycetes,o:Caliciales,f:Caliciaceae,g:Buellia,s:Buellia frigida

AAGTCGTAACAAGGTTTCCGTAGGTGAACCTGCGGAAGGATCATTACCGAGAGACGAGGT

CGCTTCGGCCCCACTCTTCACCCGTGTCTACTTACCTTTGTTGCTTTGGCGGGCCTTCGG

GCTTGACCGTTCGACGCGGGTGGGAGGCTTTCGCCTCTCGCCTCGCGAGCGCCCGCCAAA

GGCCCTGTTAACTCTGTTTTAGTGTCTTTCGAGCAACCATGTAATAGTTAAAACTTTCAA

CAACGGATCT

>ASV265 GS|98.5|UDB01588125|SH0913118.09FU;k:Fungi,p:Ascomycota,c:Lecanoromycetes,o:Caliciales,f:Caliciaceae

AAGTCGTAACAAGGTTTCCGTAGGTGCTGCAGCCTCCCAAGGTTCGCCTACCGAAGCCCC

GGGCGACTCTCTAAACAATCCGCCCAGTCAGTCGCCACTGGCGGCTGGCCACGCAGCCAT

TCTGGCTTGGAAGCCCCCCGAGGAGCCCCCTCGGCGGCTACCAGCAGCTCCGGCACCGTC

GAGTGCCGGAGTCCACAGATCAAATACCTGCGGCCACATCTGTGGTTCTGATATGACCGA

CCGCCGCCCG

>ASV266 GSL|100.0|FJ473371|SH1188820.09FU;k:Fungi,p:Basidiomycota,c:Tremellomycetes,o:Filobasidiales,f:Filobasidiaceae,g:Naganishia

AAGTCGTAACAAGGTTTCCGTAGGTGAACCTGCGGAAGGATCATTAATGAATGCAGATTC

CTGTCGAGCTTGCTCACGGGATTTCAATCATATCCATAACACCTGTGCACTTGTTGGATG

GTATAACAGAGCCCTAGTGGTGAAGTTTTCCATCTAATTTACACTAAACAATCCTGTAAC

AAATGTAGTCTTATTATAACATAATAAAACTTTCAACAACGGATCTCTTGGCTCTC

>ASV267 GS|97.5|UDB01588134|SH0913118.09FU;k:Fungi,p:Ascomycota,c:Lecanoromycetes,o:Caliciales,f:Caliciaceae

AAGTCGTAACAAGGTTTCCGTAGGTGAACCTGCGGAAGGATCATTACCGAGAGACGGGGT

CTAACCGGCCCCACTCTTCACCCGTGTATACCTACCCATGTTGCTTTGGCGGGCCTCCTG

CTAGGCGCCGGTCAGAGGCCGTTCGTGCCTCTTCCCGGCGAGTGCCCGCCGAAGGCTCTG

CAAACTCTTGTTTTAGTGTCGTCCGAGTAAAAACCTAATAGTTAAAACTTTCAACAACGG

ATCTCTTGGT

>ASV268 GS|98.0|AF250779|SH0954634.09FU;k:Fungi,p:Ascomycota,c:Lecanoromycetes,o:Caliciales,f:Caliciaceae,g:Buellia,s:Buellia frigida

AAGTCGTAACAAGGTTTCCGTAGGTGAACCTACGAAAGAATCATTATCGAGAGACGGGGT

CGCTTCGGCCCCACTCTTCACCCGTGTCTACTTACCTTTGTTGCTTTGGCGGGCCTTCGA

GCTTGACCGTTCGACGCGAGTGGGAGGCTTTCGCCTCTCGCCTCGCGAGCGCCCGCCAAA

GGCCCTGTTAACTCTGTTTTAGTGTCTTCCGAGCAACCATGTAATAGTTAAAACTTTCAA

CAACGAATCT

>ASV269 GS|97.5|AF250779|SH0954634.09FU;k:Fungi,p:Ascomycota,c:Lecanoromycetes,o:Caliciales,f:Caliciaceae,g:Buellia,s:Buellia frigida

AAGTCGTAACAAGGTTTCCGTAGGTGAACCTGCGAAAGAATCATTACCGAGAGACGAAGT

CGCTTCGACCCCACTCTTCACCCGTGTCTACTTACCTTTGTTGCTTTGGCGGGCCTTCGG

GCTTGACCGTTCGACGCGAGTGGGAGGCTTTCGCCTCTCGCCTCGCGAGCGCCCGCCAAA

GGCCCTGTTAACTCTGTTTTAGTGTCTTCCGAGCAACCATGTAATAGTTAAAACTTTCAA

CAACGAATCT

>ASV270 GS|88.7|AF250779|SH0954634.09FU;k:Fungi,p:Ascomycota,c:Lecanoromycetes,o:Caliciales,f:Caliciaceae,g:Buellia,s:Buellia frigida

AAGTCGTAACAAGATTTTCGTAGATGAACTTGCGAAAGAATCATTATCGAGAGACGAGAT

CGCTTCGACCCCACTCTTCACTCGTGCCTACTTATCTTTGTTGCTTTGACGAGCCTTCGA

GCTTGATCGTTCGACGCGAGTGGAAGGCTTTCGCCTCTCGTCTCGCGAGCGCTCGTCAAA

GGCTTTGTTAACTCTGTTTTAGTGTCTTTCGAACAATCATGTAATAATTAAAACTTTCAA

CAACGAATCT

>ASV271 GS|84.8|AF250779|SH0954634.09FU;k:Fungi,p:Ascomycota,c:Lecanoromycetes,o:Caliciales,f:Caliciaceae,g:Buellia,s:Buellia frigida

AAGTCGTAACAAGATTTTCATAGATGAATCTACGAAAGAATCATTATCGAGAGTCGAGGT

CGCTTCGATCTCACTCTTCACTCGTGTCTACTTATCTTTGTTGCTTTGACGAGTCTTCGA

ACTTGATCGTTCGACGCGAGTGAGAGACTTTCGTCTCTCGTCTCGCGAGCGTTCGTCAAA

AGTCGTGTTAACTTTGTTTTAGTGTCTTTCGAGCAATCATATAATAATTAAAACTTTCAA

CAACGAATCT

>ASV272 GS|100.0|DQ028272|SH1084019.09FU;k:Fungi,p:Ascomycota,c:Dothideomycetes,o:Mycosphaerellales,f:Teratosphaeriaceae,g:Friedmanniomyces,s:Friedmanniomyces endolithicus

AAGTCGTAACAAGGTCTCCGTAGGTGAACCTGCGGAGGGATCATTACTGAGCGAGGGCCT

CCGGGTCCGACCTCCAACCCCCTGTCATCCGACCACTTGCCTTGGGGGCGACCCGGCCTT

CGGGCGTCGGGGCCCCCAATGGACCCGCCAACCCTGCATCTGTGCGTCCGAGTCAACGAT

TGAATCAATCAAAACTTTCAACAACGGATCTCTTGGTTCTG

>ASV273 GS|85.8|AF250779|SH0954634.09FU;k:Fungi,p:Ascomycota,c:Lecanoromycetes,o:Caliciales,f:Caliciaceae,g:Buellia,s:Buellia frigida

AAGTCGTAACAAGATTTTCGTAGATGAATTTGCGAAAGAATCATTATCGAGAGACGAAGT

CGCTTCGATCTCACTCTTCACTCGTGTCTACTTATCTTTGTTGCTTTGACGAGTCTTCGA

GCTTGATCGTTCGACGCGAGTGAAAGACTTTCGTCTCTCGTCTCGCGAGCGCTCGTCAAA

GATCTTGTTAACTCTGTTTTAGTGTCTTTCGAGCAATCATGTAATAATTAAAACTTTCAA

CAATGAATCT

>ASV274 GSL|100.0|MN489437|SH0985300.09FU;k:Fungi,p:Ascomycota,c:Dothideomycetes,o:Pleosporales,f:Didymellaceae

AAGTCGTAACAAGGTTTCCGTAGGTGAACCTGCGGAAGGATCATTACCTAGAGTTTGTGG

GCTTTGCCTGCTATCTCTTACCCATGTCTTTTGAGTACTTACGTTTCCTCGGTGGGTTCG

CCCGCCGATTGGACAATTTAAACCCTTTGCAGTTGCAATCAGCGTCTGAAAAACATAATA

GTTACAACTTTCAACAACGGATCTCTTGGTTCTG

>ASV275 GS|98.4|AY667583|SH0954634.09FU;k:Fungi,p:Ascomycota,c:Lecanoromycetes,o:Caliciales,f:Caliciaceae,g:Buellia,s:Buellia frigida

AAGTCGTAACAAGGTTTCCGTAGGTGAACCTGCGGAAGGATCATTACCGAGAGACGGGGT

CGCTTCGGCCCCACTCTTCACCCGTGTCTACTTACCTTTGTTGCTTTGGCGGGCCTTCGG

GCTTGACCGATTGACGCGGGTGGGAGGCTTTCGCCTCTCGCCTCGCGAGCGCCCGCCAAA

GGCCCTGTTAACTCTGTTTTAGTGTCTTCCGAGCAGCCATGTAATTGTTAAAACTTTCAA

CAACGGATCT

>ASV276 GS|0.0|None;No hit

GTGTCAGCAGCCGCGGTAAGACGGAGGATGCAAGCGTTATCCGGAATGATTGGGCGTAAA

GCGTCTGTAGGTGGGTTGTGAAGTCTTCTGTTAAAGATCAGGGCTTAACCCTGTTTAGGC

AGTGGAAACTCATAACCTAGAGTACGGTAGGGGCAGAGGGAATTCCCGGTGTAGCGGTGA

AATGCGTAGATATCGGGAAGAACACCGACGGCGAAAGCACTCTGCTGGGCCGAAACTGAC

ACTGAGAGAC

>ASV277 SS|0.9500|KC346301|SH1107756.09FU;k:Fungi,p:Ascomycota,c:Lecanoromycetes,o:Teloschistales,f:Teloschistaceae

AAGTCGTAACAAGGTTTCCGTAGGTGAACCTGCGGAAGGATCATTATCGAGAGGGGGGCT

CCATGCCCCGGGGCTCTGTCCCCGTACCTTTTCACCCTGTGTGTATTTTTCCCCCGTTGC

TTTGGCGGGCCCCGGGTCTTCCCCCGGCGCTGGCCCCCTCGCGGGGTTCGCGAGCGCCCG

CCGAAGGCTCTTCGAAACTCTGTTGATCAGTGCAGTCTGAGCGTACGAACAATAAATCAA

AACTTTCAAC

>ASV278 GS|86.4|AY667583|SH0954634.09FU;k:Fungi,p:Ascomycota,c:Lecanoromycetes,o:Caliciales,f:Caliciaceae,g:Buellia,s:Buellia frigida

AAGTCGTAACAAGATTTTCGTAGATGAACCTACGAAAGAATCATTATCGAGAGACGAAAT

CGCTTCGATCTCACTCTTCACTCGTGTCTACTTATCTTTGTTGCTTTGACGAGTCTTCGA

GCTTGATCGTTCGACGCGAGTGAGAGACTTTCGCCTCTCGTCTCGCGAGCGCTCGTCAAA

GATTTTGTTAACTCTATTTTAGTGTCTTTCGAGCAATCATGTAATAGTTAAAACTTTCAA

CAACGAATCT

>ASV279 GS|99.6|EU139141|SH1289185.09FU;k:Fungi,p:Ascomycota,c:Eurotiomycetes,o:Chaetothyriales,f:Herpotrichiellaceae,g:Rhinocladiella

AAGTCGTAACAAGGTTTCCGTAGGTGAACCTGCGGAAGGATCATTACCGAGTTAGGGTCC

TCTGGGCTCGACCTCCTACCCTTTGTCTACCTTACCTTTTGTTGCTTCGGCGGACCCGTC

TCTTAGGGGACCGCCGGAGGGTTGACCCCCTCTGGCCCGCGTCCGCCGGTGGCCCACAAC

CAAAACTCTTGTTTAAACGTGTCGTCTAAGTACAAAAACAAATAAGTAAAAACTTTCAAC

AACGGATCTC

>ASV280 GS|86.3|AF250779|SH0954634.09FU;k:Fungi,p:Ascomycota,c:Lecanoromycetes,o:Caliciales,f:Caliciaceae,g:Buellia,s:Buellia frigida

AAGTCGTAACAAGATTTTCGTAGATGAATCTACGAAAGAATCATTATCGAGAGACGAGAT

CGCTTCGACTCCACTCTTCACTCGTGTCTACTTATCTTTGTTGCTTTGACGAGTCTTCGA

ACTTGATCGTTCGACGCGAGTGAGAGACTTTCGTCTCTCGTTTCGCGAGCGCTCGTCAAA

GACCCTATTAACTTTGTTTTAGTGTCTTTCGAGCAATCATGTAATAGTTAAAACTTTTAA

CAACGAATCT

>ASV281 GS|99.6|FJ392866|SH1071027.09FU;k:Fungi,p:Ascomycota,c:Eurotiomycetes,o:Chaetothyriales,f:Trichomeriaceae,g:Knufia

AAGTCGTAACAAGGTTTCCGTAGGTGAACCTGCGGAAGGATCATTACCGAGTTAGGGTCT

CTTCGGAGCCCGAACCTCCCAACCCTTTGTCTAATTTACCTTGTCGTTGCTTCGGCGGAC

CGGTTGACCAACTGGTCTTGACCGCTGGGGGTCCCGTACCCCTGGAGAGCGTCCGCCGAC

GGCCCAACCACAAACTCTTGTACTAAACCATGTCGTCTGAATGTACTTGATATTAATCAA

AAAACAAAAC

>ASV282 GS|100.0|OL614726|SH0994206.09FU;k:Fungi,p:Basidiomycota,c:Tremellomycetes,o:Tremellales,f:Bulleribasidiaceae,g:Vishniacozyma,s:Vishniacozyma victoriae

AAGTCATAACAAGGTTTCCGTAGGTGAACCTGCGGAAGGATCATTAATAGTGCCCTCTGA

TGCAAATCATTGGGTTAGATCTGCTCTCTTCGCAAGAAGAGGGTTTCCATACACACCGTG

AACTGTGGCTTCGGCCATCACAAACTGTTAGTAATGAATGTAATATCATAACAAAAACAA

AACTTTTAACAACGGATCTCTTGGCTCTC

>ASV283 GS|71.4|UDB0243452|SH1241595.09FU;k:Fungi,p:Basidiomycota,c:Agaricomycetes,o:Agaricales,f:Inocybaceae,g:Inocybe

AAGTCGTAACAAGGTTTCCGTAGGTGAACCTGCGGAAGGATCATTATTGAATCTGCTTGG

TGAGGTCATTCTGTGGCCTCGCTGGGTAACCTCTCTTCTTCTCCCTGTGAACTGTTGAGA

TGTGTATGGTCGTCTGACGACTATTACTGCTCATAATTTTTAGAAACCACGTCAAAAGAA

TGTTTTTACACGATGGCAGCTTAAACACTGTCGTCCAAACCAAAATACAACTTTTAGCAA

CGGATCTCTT

>ASV284 GS|99.6|FJ392867|SH1071027.09FU;k:Fungi,p:Ascomycota,c:Eurotiomycetes,o:Chaetothyriales,f:Trichomeriaceae,g:Knufia

AAGTCGTAACAAGGTTTCCGTAGGTGAACCTGCGGAAGGATCATTACCGAGTTAGGGTCT

CTTCGGAGCCCGAACCTCCCAACCCTTTGTCTAATTTACCTTGTCGTTGCTTCGGCGGAC

CGGTTGACCAACTGGTCTTGACCGCCGGGGGTCCCGTACCCCTGGAGAGCGTCCGCCGAC

GGCCTAACCACAAACTCTTGTACTAAACCATGTCGTCTGAATGTACTTGATATCAATCAA

AAAACAAAAC

>ASV285 GS|83.2|AY667583|SH0954634.09FU;k:Fungi,p:Ascomycota,c:Lecanoromycetes,o:Caliciales,f:Caliciaceae,g:Buellia,s:Buellia frigida

AAGTCGTAACAAGATTTTCGTAGATGAACTTGTGAAAGAATCATTATCGAGAGATGAGAT

CGCTTCGATCTTACTCTTCACTCGTGTCTACTTATCTTTGTTGCTTTGACGAGTTTTCGA

GCTTAATCGTTCGACGCGAATGAGAGACTTTCGTCTTTCGTCTCGCGAGCGTTCGTCAAA

GACTCTATTAACTTTGTTTTAGTGTCTTTCGAGCAATCATGTAATAATTAAAACTTTCAA

CAACGAATCT

>ASV286 GS|83.6|AY667583|SH0954634.09FU;k:Fungi,p:Ascomycota,c:Lecanoromycetes,o:Caliciales,f:Caliciaceae,g:Buellia,s:Buellia frigida

AAGTCGTAACAAGATTTTCGTAGATGAATTTGCGAAAGAATCATTATCGAGAGACGAGAT

CGCTTCGATTTCACTCTTCACTCGTATCTACTTATCTTTGTTGCTTTGACGAGTCTTCGA

GCTTGATCGTTCGACGCGAGTGAAAGACTTTCGTCTCTCGTCTTGCGAGCGTTCGTCAAA

GACTTTGTTAACTTTGTTTTAATGTCTTTCGAGCAATCATGTAATAATTAAAACTTTCAA

CAACGAATCT

>ASV287 GS|100.0|KY947809|SH0998157.09FU;k:Fungi,p:Ascomycota,c:Lecanoromycetes,o:Umbilicariales,f:Umbilicariaceae,g:Umbilicaria,s:Umbilicaria decussata

AAGTCGTAACAAGGTTTCCGTAGGTGAACCTGCGGAAGGATCATTACTGAGATAGGGTCC

TCCGGGCCCGAACCTCCAACCCTTTGTGTACCTTACCTTTGTTGCTTTGGCGGGCCCGCT

GGGGAAACCCACCGCCGGCGTCGAGCCGGTGAGCGCCCGCCGGAGGCCCCCAAAACTCCG

TCTTGTCAGTGTCGTCTGAGTACTATACAATAGCTAAAACTTTCAACAACGGATCTCTTG

GTTCTG

>ASV288 GS|0.0|None;No hit

AAGTCATAACAAGATTTTCGTAAATGAATTTGCGAAAGAATCATTATCGAGAGACGAAAT

CGCTTCGACTTCATTCTTCACTCGTATCTACTTATCTTTGTTGCTTTAACGAGTCTTCGA

ACTTGATCGTTCAACGCAAATGAGAGACTTTCGTCTCTCGTCTTGCGAGCGCTCGTCAAA

GACTCTATTAACTCTATTTTAGTGTTTTTCGAGCAATCATGTAATAATTAAAACTTTCAA

CAATAAATCT

>ASV289 GS|86.4|AY667583|SH0954634.09FU;k:Fungi,p:Ascomycota,c:Lecanoromycetes,o:Caliciales,f:Caliciaceae,g:Buellia,s:Buellia frigida

AAGTCGTAACAAGATTTTCGTAGGTGAATTTGCGAAAGAATCATTATCGAGAGACGAGAT

CGCTTCGATCTCACTCTTCACTCGTGTTTACTTATCTTTGTTGCTTTGACGAGTCTTCGA

GCTTGATCGTTCGACGCGAATGAGAGACTTTCGTCTCTCGTCTCGCGAGCGCTCGTCAAA

GGTCTTGTTAACTCTATTTTAGTGTCTTTCGAGCAATCATGTAATAGTTAAAACTTTCAA

CAACGAATCT

>ASV290 GS|97.0|AY756489|SH1199759.09FU;k:Fungi,p:Ascomycota,c:Lecanoromycetes,o:Lecanorales,f:Micareaceae,g:Micarea,s:Micarea nitschkeana

AAGTCGTAACAAGGTTTCCGTAGGTGAACCTGCGGAAGGATCATTAATGAGAACGTGCCC

TCCGGGGTAGATCTCCAACCCTCTGTCTACCTATCCATGTTGCTTTGACGGGCCCGTCCC

CCGGGACCGCCGGCTCCGGCTGGCCCGTGCCCGTCAGAGGTCCACCAAACCCTATCAAAT

GTACTGTCCGAGTAACACATAATCGTTAAAACTTTCAACAACGGATCTCTTGGTTCTG

>ASV291 GS|99.6|KC965446|SH0947923.09FU;k:Fungi,p:Ascomycota,c:Sordariomycetes,o:Hypocreales

AAGTCGTAACAAGGTCTCCGTTGGTGAACCAGCGGAGGGATCATTACCGAGTTTTCAACT

CCCAAACCCATGTGAACATACCACTGTTGCTTCGGCGGACCGCCCCGGGTGCTGCGTGCC

CCGCAACCAGGCGCCCGCCGGAGGCCTAAACTCTTGTTTTTACCATGAATCTTCTGAGTA

GCGCAAGCAAAAAAAATAAATCAAAACTTTCAACAACGGATCTCTTGGTTCTG

>ASV292 GS|98.5|KF516956|SH0998103.09FU;k:Fungi,p:Ascomycota,c:Lecanoromycetes,o:Umbilicariales,f:Umbilicariaceae,g:Umbilicaria

AAGTCGTAACAAGGTTTCCGTAGGTGAACCTGCGGAAGGATCATTAATGAGATAGGGTCC

TTCGGGGCCCGAACCTCCCAACCCTCTGTGTATCTTACCTTTGTTGCTTTGGCGGGCCTT

TGCTCACGCAGCGTACCCCCGGGTGCGAGTGCCCGCCCGAGGCCATTCAAATTCCGATTA

TCAGTGACGTCTGAGTACTATATAATAGTTAAAACTTTCAACAACGGATCTCTTGGTTCT

G

>ASV293 GS|88.2|AF250779|SH0954634.09FU;k:Fungi,p:Ascomycota,c:Lecanoromycetes,o:Caliciales,f:Caliciaceae,g:Buellia,s:Buellia frigida

AAGTCGTAACAAGGTTTTCGTAGGTGAATCTACGAAAAAATCATTATCGAGAGACGGGGT

CGCTTCGATTTCACTCTTCACTCGTATCTACTTATCTTTGTTGCTTTGACGAGCCTTCGA

GCTTGATCGTTCGACGCGAGTGAGAGGCTTTCGCCTCTCGTTTTGCGAGCGCTCGTCAAA

GGCCTTGTTAACTTTGTTTTAGTGTCTTTCGAGCAATCATGTAATAGTTAAAACTTTCAA

CAACGAATCT

>ASV294 GS|99.6|AY667583|SH0954634.09FU;k:Fungi,p:Ascomycota,c:Lecanoromycetes,o:Caliciales,f:Caliciaceae,g:Buellia,s:Buellia frigida

AAGTCGTAACAAGGTTTCCGTAGGTGAACCTGCGGAAGGATCATTACCGAGAGACGGGGT

CGCTTCGGCCCCACTCTTCACCCGTGTCTACTTACCTTTGTTGCTTTGGCGAGCCTTCGG

GCTTGACCGTTCGACGCGGGTGGGAGGCTTTCGCCTCTCGCCTCGCGAGCGCCCGCCAAA

GGCCCTGTTAACTCTGTTTTAGTGTCTTCCGAGCAACCATGTAATAGTTAAAACTTTCAA

CAACGGATCT

>ASV295 GS|99.6|AY667583|SH0954634.09FU;k:Fungi,p:Ascomycota,c:Lecanoromycetes,o:Caliciales,f:Caliciaceae,g:Buellia,s:Buellia frigida

AAGTCGTAACAAGGTTTCCGTAGGTGAACCTGCGGAAGGATCATTACCGAGAGACGGGGT

CGCTTCGGCCCCACTCTTCACCCGTGTCTACTTACCTGTGTTGCTTTGGCGGGCCTTCGG

GCTTGACCGTTCGACGCGGGTGGGAGGCTTTCGCCTCTCGCCTCGCGAGCGCCCGCCAAA

GGCCCTGTTAACTCTGTTTTAGTGTCTTCCGAGCAACCATGTAATAGTTAAAACTTTCAA

CAACGGATCT

>ASV296 GS|82.8|AY667583|SH0954634.09FU;k:Fungi,p:Ascomycota,c:Lecanoromycetes,o:Caliciales,f:Caliciaceae,g:Buellia,s:Buellia frigida

AAGTCGTAACAAGATTTTCGTAGATGAATCTACGAAAGAATCATTATCGAGAGACGAAAT

CGCTTCGATTTCACTCTTCACTCGTATCTACTTATCTTTGTTGCTTTGGCGAGTCTTCGA

GCTTGATCGTTCGATGCGAGTGAAAGACTTTCGTCTCTCGTTTTGCGAGCGTTCGTCAAA

GATCGTGTTAACTTTGTTTTAGTATCTTTCGAACAATCATGTAATAGTTAAAACTTTCAA

CAACGAATCT

>ASV297 SS|1.0000|KF274145|SH0988164.09FU;k:Fungi

AAGTCGTAACAAGGTTTCCGTAGGTGAACCTGCGGAAGGATCATTAATGAATGCAAGGAT

ACTCTTTTTAGAGGTCCGACTCATCATTTTCCAACCCTGTGCACATACACTGTTGTTTTA

CACATCATTTTTAACACAAAGTCTATAAGAATGTATAAAAGTCTCTTGATTGAGCACATA

AATTAAACAAAACTTTCAGCAACGGATCTCTTGGCTCTC

>ASV298 GS|88.7|AF250779|SH0954634.09FU;k:Fungi,p:Ascomycota,c:Lecanoromycetes,o:Caliciales,f:Caliciaceae,g:Buellia,s:Buellia frigida

AAGTCGTAACAAGATTTTCGTAGATGAACTTGCGAAAGAATCATTATCGAGAGACGAAAT

CGCTTCGACCTCACTCTTCACTCGTGTCTACTTACCTTTGTTGCTTTGGCGAGTTTTCGA

GCTTGATCGTTCGACGCGAGTGAGAGACTTTCGCCTCTCGCCTCGCGAGCGCTCGTCAAA

GGTTCTGTTAACTCTATTTTAGTGTCTTTCGAGCAATCATGTAATAGTTAAAACTTTCAA

CAACGAATCT

>ASV299 GS|89.2|AF250779|SH0954634.09FU;k:Fungi,p:Ascomycota,c:Lecanoromycetes,o:Caliciales,f:Caliciaceae,g:Buellia,s:Buellia frigida

AAGTCGTAACAAGATTTTCGTAGATGAATCTACGAAAGAATCATTATCGAGAGACGAGGT

CGCTTCGATCTCACTCTTCACTCGTGTCTACTTACCTTTGTTGCTTTGGCGAGTCTTTGG

GCTTGATCGTTCGACGCGAGTGAGAGGCTTTCGTCTCTCGCCTTGCGAGCGTTCGCCAAA

GACTCTGTTAACTTTGTTTTAGTGTCTTTCGAGCAATCATGTAATAGTTAAAACTTTCAA

CAACGAATCT

>ASV300 GS|100.0|UDB02641368|SH0922156.09FU;k:Fungi,p:Ascomycota,c:Orbiliomycetes,o:Orbiliales

AAGTCGTAACAAGGTTTCCGTAGGTGAACCTGCGGAAGGATCATTAAAAATCCTTCACCC

TGGATCCAGTATCCGGGTGGACCAGCCTCCGGGGCTCTAATCCGTACGTAATGCCACCCG

CGGAGCCCCTTCATGGGGATGCGGTGGTACTCGTTACTTCAAACCATTGTGAACCTTCCC

GTTGCTTCGGCGGCATGCCTTGTGCTGTCAGCCCGCCCGAGGCCCACCATGAAACCATTT

CTGTAAACTT

>ASV301 GS|0.0|None;No hit

AAGTCGTAACAAGATTTTCGTAAATGAATTTGCGAAAGAATCATTATCGAGAGACGAAAT

CGCTTCGACTTCACTCTTCACTCGTATCTACTTATCTTTGTTGCTTTAACGAGTCTTCGA

ATTTGATCGTTCAATGCGAATGAGAGACTTTCGTCTCTCGTCTTGCGAGCGCTCGTCAAA

GACTCTATTAACTCTATTTTAGTGTTTTTCGAACAATCATGTAATAATTAAAATTTTTAA

CAATAAATCT

>ASV302 GS|90.2|AF250779|SH0954634.09FU;k:Fungi,p:Ascomycota,c:Lecanoromycetes,o:Caliciales,f:Caliciaceae,g:Buellia,s:Buellia frigida

AAGTCGTAACAAGATTTTCGTAGGTGAACCTACGAAAGAATCATTATCGAGAGACGAGGT

CGCTTCGACCTCACTCTTCACTCGTGTCTACTTACCTTTGTTGCTTTGACGGGCCTTCGA

GCTTGATCGTTCGACGCGAGTGGGAGACTTTCGTCTCTCGTCTCGCGAGCGCTCGTCAAA

GACTTTGTTAACTCTGTTTTAGTGTCTTTCGAGCAATCATGTAATAGTTAAAACTTTCAA

CAACGAATCT

>ASV303 SS|0.9600|JN885566|SH1084447.09FU;k:Fungi,p:Ascomycota,c:Dothideomycetes

AAGTCGTAACAAGGTCTCCGTAGGTGAACCTGCGGAGGGATCATTACCGAGCGAGGGCCT

CCGGGTCCGACCTCCCACCCCATGTTATCCGACCACTGTTGCCTCGGGGGCGACCCGGCC

TTCGGGCGTCGGGGCCCCCGGCGGACCCGTAACCCTGCATCTGTGCGTCCGAGTCAAACG

ATTGAATCAATCAAAACTTTCAACAACGGATCTCTTGGTTCTG

>ASV304 GS|84.4|AY667583|SH0954634.09FU;k:Fungi,p:Ascomycota,c:Lecanoromycetes,o:Caliciales,f:Caliciaceae,g:Buellia,s:Buellia frigida

AAGTCGTAACAAGATTTTCGTAGATGAATTTGCGAAAGAATCATTATCGAGAGACGAGAT

CGCTTCGATCTCACTCTTCACTCGTATCTACTTATCTTTGTTGCTTTGACGAGTCTTCGA

GCTTGATCGTTCGACGCGAGTGAAAGACTTTCGCTTGTCGTCTTGCGAGCGCTCGTCAAA

GACTTTGTTAACTCTATTTTAGTGTCTTTCGAGCAATTATGTAATAGTTAAAACTTTCAA

CAACGAATCT

>ASV305 GS|89.2|AF250779|SH0954634.09FU;k:Fungi,p:Ascomycota,c:Lecanoromycetes,o:Caliciales,f:Caliciaceae,g:Buellia,s:Buellia frigida

AAGTCGTAACAAGATTTTCGTAGATGAATCTACGAAAGAATCATTATCGAGAGACGAGAT

CGCTTCGATCTCACTCTTCACTCGTATCTACTTATCTTTGTTGCTTTGGCGAGCCTTCGA

GCTTGATCGTTCGACGCGAGTGGGAGGCTTTCGTCTATCGTCTCGCGAGCGCCCGCCAAA

GACCTTGTTAACTCTATTTTAGTGTCTTTCAAGCAACCATGTAATAGTTAAAACTTTCAA

CAACGAATCT

>ASV306 GS|88.2|AF250779|SH0954634.09FU;k:Fungi,p:Ascomycota,c:Lecanoromycetes,o:Caliciales,f:Caliciaceae,g:Buellia,s:Buellia frigida

AAGTCGTAACAAGATTTTCGTAGATGAACTTGCGAAAGAATCATTATCGAGAGACGAGGT

CGCTTCGATCTCACTCTTCACTCGTGTCTACTTATCTTTGTTGCTTTGGCGAATCTTCGA

ACTTGATCGTTCGACGCGAGTGAGAGGCTTTCGCCTCTCGTCTCGCGAGCGTTCGCCAAA

GACCCTATTAACTCTATTTTAGTGTCTTTCGAGCAATCATGTAATAGTTAAAACTTTCAA

CAACGAATCT

>ASV307 GS|0.0|None;No hit

AAGTCGTAACAAGATTTTCGTAAATGAATTTGCGAAAAAATCATTATTGAGAAACGAAAT

CGCTTCGACTTCACTCTTCATTCGTATCTATTTATTTTTGTTGCTTTGACGAATTTTCGA

ACTTGATCGTTCGACGCGAGTGAGAGACTTTCGTCTCTCGTTTTGCGAGCGCTCGTCAAA

GACTTTGTTAACTTTGTTTGAATGTCTTTCGAACAATTATATAATAATTAAAACTTTCAA

TAGCGAATTT

>ASV308 GS|100.0|JN835193|SH0956695.09FU;k:Fungi,p:Ascomycota,c:Leotiomycetes,o:Thelebolales,f:Pseudeurotiaceae,g:Pseudeurotium

AAGTCGTAACAAGGTTTCCGTAGGTGAACCTGCGGAAGGATCATTAAAGAGACGTTGCCC

TTCGGGGTATACCTCCCACCCTTTGTTTATACATACCATTGTTGCTTTGGCAGGCCCGGC

TTCGGCCCACCGGCTCCGGCTGGTCCGCGTCTGCCAGAGGAAACCCAAACTCTGTTTGTT

AATATTGTCTGAGTACTATATAATAGTTAAAACTTTCAACAACGGATCTCTTGGTTCTG

>ASV309 SS|1.0000|UDB01588134|SH0913118.09FU;k:Fungi,p:Ascomycota,c:Lecanoromycetes,o:Caliciales,f:Caliciaceae

AAGTCGTAACAAGGTTTCCGTAGGTGAACCTGCGGAAGGATCATTACCGAGAGACGGGGT

CGCTTCGGCCCCACTCTTCACCCGTGTATACCTACCCATGTTGCTTTGGCGGGCCTCCTG

CTAGGCGCCGGTCAGAGGCCGTTCGTGCCTCTTCCCGGCGAGTGCCCGCCGAAGGCTCTG

CAAACTCTTGTTTTAGTGTCGTCCGAGTAAAAACCTAATAGTTAAAACTTTCAACAACGG

ATCTCTTGGT

>ASV310 GSL|100.0|MF473171|SH1081689.09FU;k:Fungi,p:Ascomycota,c:Dothideomycetes,o:Capnodiales,f:Cladosporiaceae,g:Davidiella

AAGTCGTAACAAGGTCTCCGTAGGTGAACCTGCGGAGGGATCATTACAAGAACGCCCGGG

CTTCGGCCTGGTTATTCATAACCCTTTGTTGTCCGACTCTGTTGCCTCCGGGGCGACCCT

GCCTTCGGGCGGGGGCTCCGGGTGGACACTTCAAACTCTTGCGTAACTTTGCAGTCTGAG

TAAACTTAATTAATAAATTAAAACTTTTAACAACGGATCTCTTGGTTCTG

>ASV311 GS|75.3|DQ534454|SH0954616.09FU;k:Fungi,p:Ascomycota,c:Lecanoromycetes,o:Caliciales,f:Caliciaceae,g:Buellia,s:Buellia russa

AAGTCGTAACAAGATTTTCGTAAATGAATTTACGAAAAAATCATTATCGAGAGACGAAAT

CGCTTCGATCTCACTCTTCACTCGTATTTACTTATCTTTGTTGTTTTGACGAGTCTTCGA

ACTTGATCGTTCGACGCGAATGAAAGACTTTCGTCTCTCGTTTCGCGAGCGTTCGTCAAA

GATCTTGTTAACTTTGTTTTAATGTCTTTCGAGCAATCATGTAATAATTAAAACTTTTAA

CAATGAATCT

>ASV312 GS|85.8|AF250779|SH0954634.09FU;k:Fungi,p:Ascomycota,c:Lecanoromycetes,o:Caliciales,f:Caliciaceae,g:Buellia,s:Buellia frigida

AAGTCGTAACAAGATTTTCGTAGATGAATTTGCGAAAGAATCATTATCGAGAGACGAGAT

CGCTTCGATTCCACTCTTCACTCGTGTCTACTTATCTTTGTTGCTTTGACGAGTCTTCGA

GCTTGATCGTTCGACGCGAATGAGAGACTTTCGTCTCTCGTCTCGCGAGCGCTCGTCAAA

GATTCTATTAACTTTGTTTTAGTGTCTTTCGAGCAATCATGTAATAGTTAAAACTTTCAA

CAACGAATCT

>ASV313 GS|100.0|MW991427|SH0964445.09FU;k:Fungi,p:Ascomycota,c:Arthoniomycetes,o:Lichenostigmatales,f:Phaeococcomycetaceae,g:Antarctolichenia,s:Antarctolichenia onofrii

AAGTCGTAACAAGGTCTCCGTAGGTGAACCTGCGGAGGGATCATTAATGAGATAGGGTCT

TCACGGCCCGACCTCCAACCCAATGTCTACCATACCTCTGTTGCCTCGGCGGGCCGCCGG

CGCCCTTTGTTGGGCGCCGCCGTCGGCTTTAGGGCTCTCGAGCGCCCGCCGCAGGACCGA

TTAAACTCTTTTTAAAACAAGTCTTCTGAGTGGGAAATCAAATTTATTAAAACTTTTAAC

AACGGATCTC

>ASV314 GS|88.7|AF250779|SH0954634.09FU;k:Fungi,p:Ascomycota,c:Lecanoromycetes,o:Caliciales,f:Caliciaceae,g:Buellia,s:Buellia frigida

AAGTCGTAACAAGATTTTCGTAGATGAATCTACGAAAGAATCATTATCGAGAGACGAGAT

CGCTTCGACCCCACTCTTCACTCGTATCTACTTACCTTTGTTGCTTTGACGAATCTTCGA

GCTTGATCGTTCGACGCGAGTGAGAGACTTTCGTCTCTCGTCTCGCGAGCGCTCGTCAAA

GGCCCTGTTAACTCTATTTTAGTGTCTTTCGAGCAATCATGTAATAGTTAAAACTTTCAA

CAACGAATCT

>ASV315 GS|98.0|KX550105|SH0903330.09FU;k:Fungi,p:Ascomycota,c:Lecanoromycetes,o:Caliciales,f:Physciaceae,g:Physcia,s:Physcia dubia

AAGTCGTAACAAGGTTTCCGTAGGTACAAATAGGTTTGCCTGCCGAAACCCCCCGCAACG

ACGTTAAAAAAACTGCGCGGTCTGCGCCCTTGTGGCGTGGGCAACACCGTTCAAGTGCTG

AGAATGCCCGGCAACAGCCCCGCACAACCAGCAGCCAGCGTGTCACTGCTGGTCCACAGA

TCAATCGACGGTGACCTTTATCGGTTAAGCTATGACCGGTCCAGAGCCCCAACGGGCCCT

GGAACGACGG

>ASV316 GS|85.8|AF250779|SH0954634.09FU;k:Fungi,p:Ascomycota,c:Lecanoromycetes,o:Caliciales,f:Caliciaceae,g:Buellia,s:Buellia frigida

AAGTCGTAACAAGATTTTCGTAGATGAATCTACGAAAGAATCATTATCGAGAGACGAGAT

CGCTTCGATCTCACTCTTCACTCGTGTCTACTTATCTTTGTTGCTTTGACGAGTCTTCGA

GCTTGATCGTTCGACGCGAATGAGAGACTTTCGTCTCTCGTCTCGCGAGCGCTCGTCAAA

GACTTTGTTAACTTTGTTTTAGTGTCTTTCGAGCAATCATATAATAGTTAAAACTTTCAA

CAACGAATCT

>ASV317 GS|84.8|AF250779|SH0954634.09FU;k:Fungi,p:Ascomycota,c:Lecanoromycetes,o:Caliciales,f:Caliciaceae,g:Buellia,s:Buellia frigida

AAGTCGTAACAAGATTTTCGTAGATGAATTTGCGAAAGAATCATTATCGAGAGACGAGAT

CGCTTCGACTTCACTCTTCACTCGTATCTACTTATCTTTGTTGCTTTGACGAGTCTTCGA

GCTTGATCATTCGATGCGAGTGAGAGACTTTCGTCTCTCGTTTCGCGAGCGCTCGTCAAA

GACTCTATTAACTTTGTTTTAGTGTCTTTCGAGCAATCATGTAATAGTTAAAACTTTCAA

CAACGAATCT

>ASV318 SS|0.9800|EU516901|SH0993795.09FU;k:Fungi,p:Ascomycota,c:Dothideomycetes,o:Mycosphaerellales,f:Teratosphaeriaceae

AAGTCGTAACAAGGTCTCCGTAGGTGAACCTGCGGAGGGATCATTACCGAGCGAGGGCCT

CCGCGCCCGACCTCCAACCCTTTGTCGATTCATATCTGTTGCCTCGGGGGGCGACCCGGC

CGTCCGCGGGCGGGCGTCCCCCAGAGGGCCAATCAACTCTGCATCTTTGCGTCGAGTATT

GAATACAAATCAATCAAAACTTTTAACAACGGATCTCTTGGTTCTG

>ASV319 GSL|100.0|JQ074197|SH1299503.09FU;k:Fungi,p:Ascomycota,c:Lecanoromycetes,o:Teloschistales,f:Teloschistaceae

AAGTCGTAACAAGGTTTCCGTAGGTGAACCTGCGGAAGGATCATTACCGAGAGAGGGGTT

TCGCGCCCCCGGGGGGGTTTCGGCCCCCCTTACCTCTTCAACCCTGTGTCTACCAACCTT

TGTTGCTTTGGCGAGCGCCGGGGTGCCTTGCGCCTCGGCCCCGGCTTCGGTCGGTGCGCT

CTCGTCAGAGGCCCTTTTCCACGCTGTTTTCAGTGACGTCCGAGTGAAACAAACAATAAA

TTAAAACTTT

>ASV320 SS|0.8400|KY102927|SH1003748.09FU;k:Fungi,p:Basidiomycota,c:Tremellomycetes,o:Tremellales

AAGTCGTAACAAGGTTTCCGTAGGTGAACCTGCGGAAGGATCATTAGTGATTCGGCCCTC

ACGGGTCTATACAAGACACCTCTGTGAACCTGTCGGCCTCCGGGCCAACCTGCAAACACT

GTGTAAGGAGCGTTGATGTATCATAAGCGTAATAAAACTTTCAACAACGGATCTCTTGGC

TCTC

>ASV321 GS|99.0|KC179119|SH1085530.09FU;k:Fungi,p:Ascomycota,c:Lecanoromycetes,o:Teloschistales,f:Teloschistaceae,g:Shackletonia,s:Shackletonia insignis

AAGTCGTAACAAGGTTTCCGTAGGTGAACCTGCGGAAGGATCATTATCGAGAGCGGGGCT

TCGCGCCCCCGGGGGGCTTCGGCCTCCTACCTCTTCACCCTGTGTATATCAACCTCTGTT

GCTTTGGCGAGCCCTGGGTTTACCCCCCGGCACGGTTTCGGGCGATCCTGCCCGGAGTCG

CGGGTGCTCGCCCGAGGCCTTTAACCATCTGTTTACGTGCAACGTCCGAATGACCCAAAA

ATTAAATAAA

>ASV322 GSL|100.0|MW880910|SH1309305.09FU;k:Fungi

AAGTCGTAACAAGGTCTCCGTAGGTGAACCTGCGGAGGGATCATTACAAGTGACCCCGGT

CTAACCACCGGGATGTTCATAACCCTTTGTTGTCCGACTCTGTTGCCTCCGGGGCGACCC

TGCCTTCGGGCGGGGGCTCCGGGTGGACACTTCAAACTCTTGCGTAACTTTGCAGTCTGA

GTAAACTTAATTAATAAATTAAAACTTTTAACAACGGATCTCTTGGTTCTG

>ASV323 GS|85.3|AF250779|SH0954634.09FU;k:Fungi,p:Ascomycota,c:Lecanoromycetes,o:Caliciales,f:Caliciaceae,g:Buellia,s:Buellia frigida

AAGTCGTAACAAGATTTTCGTAGATGAATCTACGAAAGAATCATTATCGAGAGACGAGAT

CGCTTCGATCTCACACTCTTCACTCGTGTCTACTTATCTTTGTTGCTTTGACGAGTCTTC

GAGCTTGATCGTTCGACGCGAGTGAGAGACTTTCGTCTCTCGTTTCGCGAGCGCTCGTCA

AAGACTTTGTTAACTTTGTTTTAGTGTCTTTCGAGCAATCATGTAATATTTAAAACTTTC

AACAACGGAT

>ASV324 GS|89.7|AF250779|SH0954634.09FU;k:Fungi,p:Ascomycota,c:Lecanoromycetes,o:Caliciales,f:Caliciaceae,g:Buellia,s:Buellia frigida

AAATCGTAACAAGATTTTCGTAGATGAACCTACGAAAAAATCATTATCGAGAGACGAGAT

CGCTTCGACCCCACTCTTCACTCGTGTCTACTTACCTTTGTTGCTTTGACGAGCCTTCGA

GCTTGATCGTTCGACGCGAGTGAGAGACTTTCGTCTCTCGCCTCGCGAGCGCTCGCCAAA

GACCCTATTAACTTTGTTTTAGTGTCTTTCGAGCAATCATGTAATAGTTAAAACTTTCAA

CAATGAATCT

>ASV325 GS|88.7|AF250779|SH0954634.09FU;k:Fungi,p:Ascomycota,c:Lecanoromycetes,o:Caliciales,f:Caliciaceae,g:Buellia,s:Buellia frigida

AAGTTGTAACAAGATTTTCGTAGGTGAATCTACGAAAGAATCATTATCGAGAGACGAGGT

CGCTTCGATTTCACTCTTCACTCGTGTCTACTTATTTTTGTTGCTTTGGCGAATCTTCGA

GCTTGATCGTTCGACGCGAGTGGAAGGCTTTCGCCTCTCGCCTCGCGAGCGCTCGTCAAA

GACCCTATTAACTCTGTTTTAGTGTCTTTCGAGCAATCATGTAATAGTTAAAACTTTCAA

CAACGAATCT

>ASV326 GS|80.6|MZ224659|SH0916480.09FU;k:Fungi,p:Ascomycota,c:Lecanoromycetes,o:Caliciales,f:Caliciaceae,g:Buellia,s:Buellia insignis

AAGTCGTAACAAGATTTCCGTAGGTGAATCTGCGGAAGAATCATTATCGAGAAACGGAGT

CTAACCGGCCTCACTCTTCACCCGTGTATATCTATCCTTTGTTGCTTTGGCGGGCGGTCG

GTCTTTACCGCCCGACGTCGGTCGGAAGGCTCGCGCCTTCCACTCATCGAACGCCCGCCA

GAGGCCCTCAAAAGTCGTCCATCTGTGTCCGAGCGAGTGAAGCAAATAGTTAAAAACTTT

CAACAACGGA

>ASV327 SS|1.0000|MH301303|SH1020897.09FU;k:Fungi,p:Ascomycota,c:Candelariomycetes,o:Candelariales,f:Candelariaceae,g:Candelaria,s:Candelaria murrayi

AAGTCGTAACAAGGTTTCCGTAGGTGTAAGTAACCCCATATCCCCCTGCACACCCTCAAG

TGTCACCCCACTAACATCCACCAGGAACCTGCGGAAGGATCATTAAAGAGTAAGGGTCCA

CTCGGGCCCGACCTCCAACCCTCTGTATATCTACCTCTGTTGCTTTGGCGGGCCCGCTGG

GCTCAACCCCCACCGCCGGCCTAGGCTGGTGAGTGCCCGCCAGAGACCGCCTAAATTCTC

CTCAACAGTG

>ASV328 GS|85.3|AF250779|SH0954634.09FU;k:Fungi,p:Ascomycota,c:Lecanoromycetes,o:Caliciales,f:Caliciaceae,g:Buellia,s:Buellia frigida

AAGTCGTAACAAGATTTTCGTAGATGAATCTACGAAAAAATCATTATCGAGAGACGAGAT

CGCTTCGATCTCACTCTTCACTCGTGTCTACTTATCTTTGTTGCTTTGACGAGTCTTCGA

GCTTGATCATTCGACGCGAGTGAGAGACTTTCGTCTCTCGTCTCGCGAGCGTTCGTCAAA

GACTTTGTTAACTTTGTTTTAGTGTCTTTCGAGCAATCATGTAATAATTAAAACTTTCAA

CAACGAATCT

>ASV329 GS|88.4|MK970687|SH0954634.09FU;k:Fungi,p:Ascomycota,c:Lecanoromycetes,o:Caliciales,f:Caliciaceae,g:Buellia,s:Buellia frigida

AAGTCGTAACAAGATTTTCGTAGATGAATTTGCGAAAGGATCATTATCGAGAGATGGGAT

CGCTTCGACCTCACTCTTCACTCGTGTTTACTTATCTTTGTTGCTTTGACGAGTCTTCGA

GCTTGATCGTTCGACGCGAGTGAGAGGCTTTCGTCTCTCGTCTCGCGAGCGCTCGCCAAA

GGTCTTGTTAACTTTGTTTTAGTGTCTTTCGAGCAATCATGTAATAGTTAAAACTTTCAA

CAACGAATCT

>ASV330 GS|99.6|AY667583|SH0954634.09FU;k:Fungi,p:Ascomycota,c:Lecanoromycetes,o:Caliciales,f:Caliciaceae,g:Buellia,s:Buellia frigida

AAGTCGTAACAAGGTTTCCGTAGGTGAACCTGCGGAAGGATCATTACCGAGAGACGGGGT

CGCTTCGGCCCCACTCTTCACCCGTGTCTACTTACCTTTGTTGCTTTGGCGGGCCTTCGG

GCTTGACCGTTCGACGCGGGTGGGAGGCTTTCGCCTCTCGCCTCGCGAGCGCCCGCGAAA

GGCCCTGTTAACTCTGTTTTAGTGTCTTCCGAGCAACCATGTAATAGTTAAAACTTTCAA

CAACGGATCT

>ASV331 GS|99.6|JQ342183|SH0948228.09FU;k:Fungi,p:Ascomycota,c:Eurotiomycetes,o:Chaetothyriales,f:Herpotrichiellaceae,g:Cladophialophora

AAGTCGTAACAAGGTTTCCGTAGGTGAACCTGCGGAAGGATCATTATCGAGTTAGGGTTC

CTTCACGGGCCCGACCTCCAACCCGGTGTCTAAACTTACCACTGTTGCTTCGGCGGGCCC

GCCTGTAAAAGGGCCGCCGGGGGTGCTCCGTCACCTCTGGCCCGCGCCCGTCGATAGCCC

ACAACAAACTCTTTTAAACCGTGTCCTGTCTGAATAAACCAAAAAATTAAGCACAAAACT

TTCAACAACG

>ASV332 SS|0.9900|AF250779|SH0954634.09FU;k:Fungi,p:Ascomycota,c:Lecanoromycetes,o:Caliciales,f:Caliciaceae,g:Buellia,s:Buellia frigida

AAGTCGTAACAAGGTTTCCGTAGGTGAATTTGCGAAAGAATCATTATCGAGAGACGGGGT

CGCTTCGACCTCACACTCTTCACTCGTGTCTACTTACCTTTGTTGCTTTGGCGGGCCTTC

GGGCTTGATCGTTCGACGCGGGTGAGAGACTTTCGCCTCTCGCCTCGCGAGCGCTCGTCA

AAGGCTCTGTTAACTCTGTTTTAGTGTCTTCCGAGCAATCATGTAATATTTAAAACTTTC

AACAACGGAT

>ASV333 GS|85.3|AF250779|SH0954634.09FU;k:Fungi,p:Ascomycota,c:Lecanoromycetes,o:Caliciales,f:Caliciaceae,g:Buellia,s:Buellia frigida

AAGTCGTAATAAGATTTTCGTAGATGAACTTGCGAAAGAATCATTATCGAGAGACGAGAT

CGCTTCGATCTCACTCTTCACTCGTGTCTACTTATCTTTGTTGCTTTGACGAGTCTTCGA

GCTTGATCGTTCGACGCGAGTGAAAGACTTTCGTCTCTCGTCTCGCGAGCGTTCGTCAAA

GACTTTGTTAACTCTATTTTAGTGTCTTTCGAGCAATCATGTAATAGTTAAAACTTTTAA

CAACGAATCT

>ASV334 SS|0.8700|MF976002|SH1083909.09FU;k:Fungi,p:Ascomycota,c:Dothideomycetes

AAGTCGTAACAAGGTCTCCGTAGGTGAACCTGCGGAGGGATCATTACCGAGCGAGGGCGT

CCCGCCCAACCTCCCAACCCTCTGCGACCCGACTCGATTGCCTCGGGGGCGACCCGGCCC

TTTTGGTTCGGGGCCCCCGGTGGACCCCTCCTACTCTGCGTCTTTGCGTCCGAGTACCGT

ACAAATCAATCAAAACTTTCAACAACGGATCTCTTGGTTCTG

>ASV335 SS|1.0000|AY667583|SH0954634.09FU;k:Fungi,p:Ascomycota,c:Lecanoromycetes,o:Caliciales,f:Caliciaceae,g:Buellia,s:Buellia frigida

AAGTCGTAACAAGGTTTTCGTAGATGAACCTGCGGAAGGATCATTATCGAGAGACGAGGT

CGTTTCGGCCCCACTCTTCACCCGTGTCTACTTATCTTTGTTGCTTTGGCGAGCCTTCGG

GCCTGATCGTTCGACGCGGGTGGGAGGCTTTCGCCTCTCGTCTCGCGAGCGCTCGCCAAA

GACCCTGTTAACTCTGTTTTAGTGTCTTTCGAGCAACCATGTAATAGTTAAAACTTTCAA

CAACGGATCT

>ASV336 GS|84.0|AY667583|SH0954634.09FU;k:Fungi,p:Ascomycota,c:Lecanoromycetes,o:Caliciales,f:Caliciaceae,g:Buellia,s:Buellia frigida

AAGTCGTAACAAGATTTTCGTAAGTGAACTTGCGAAAGAATCATTATCGAGAGACGAAAT

CGCTTCGATCTCACTTTTCACTCGTGTCTACTTATCTTTGTTGCTTTGACGAGTCTTCGA

GTTTGATCATTCGACGCGAGTGAGAGACTTTCGTCTATCGTCTCGCGAGCGCTCGTCAAA

GATCTTGTTAACTCTATTTTAGTGTCTTTCGAGCAATCATATAATAGTTAAAACTTTCAA

CAATGAATCT

>ASV337 GS|98.3|MT108257|SH0985456.09FU;k:Fungi,p:Ascomycota,c:Lecanoromycetes,o:Rhizocarpales,f:Rhizocarpaceae,g:Rhizocarpon,s:Rhizocarpon furax

AAGTCGTAACAAGGTTTCCGTAGGTGAACCTGCGGAAGGATCATTACTGAGATAGGGGCT

CCCGGCCCCGAACCTCCACCCGTGCATACCGACCTTTTGTTGCTTTGGCGGGTCTGCGGT

CGGATCCGTTCCCGGCCGCCGCAGCGTGCCCGCCGAAAGCCCACCCAAACTCGCTTACCG

GTGATGTCTGAGTAACGAAACAATAATCAAAACTTTCAACAACGGATCTCTTGGTTCTG

>ASV338 GSL|100.0|MK036062|SH1029393.09FU;k:Fungi,p:Ascomycota,c:Saccharomycetes,o:Saccharomycetales,f:Debaryomycetaceae,g:Meyerozyma,s:Meyerozyma guilliermondii

AAGTCGTAACAAGGTTTCCGTAGGTGAACCTGCGGAAGGATCATTACAGTATTCTTTTGC

CAGCGCTTAACTGCGCGGCGAAAAACCTTACACACAGTGTCTTTTTGATACAGAACTCTT

GCTTTGGTTTGGCCTAGAGATAGGTTGGGCCAGAGGTTTAACAAAACACAATTTAATTAT

TTTTACAGTTAGTCAAATTTTGAATTAATCTTCAAAACTTTCAACAACGGATCTCTTGGT

TCTC

>ASV339 SS|1.0000|None;k:Fungi,p:Ascomycota,c:Lecanoromycetes,o:Lecanorales,f:Parmeliaceae,g:Neoprotoparmelia,s:Neoprotoparmelia corallifera

GTGTCAGCAGCCGCGGTAATACGTAGAAGACTAGTGTTATTCATCTTTAATAGGTTTAAA

GGGTACCTAGACGGTAAATCAAGCCCTTAGGGGGACTACTTTACTAGAGTTACTTATGAG

GGGGTATTAAAGTACTGCTGGTGTAGAGATGAAATTCTGTCATACCTCTTTTCGTGGGAA

AAATAATGGCACAGGTATAGGCGAAAGCATCCCCTTATGTGATAACTGACGTTGAAGGAC

GAAGGCTTTG

>ASV340 GS|84.8|AF250779|SH0954634.09FU;k:Fungi,p:Ascomycota,c:Lecanoromycetes,o:Caliciales,f:Caliciaceae,g:Buellia,s:Buellia frigida

AAGTCGTAACAAGATTTTCGTAGATGAATCTACGAAAGAATCATTATCGAGAGACGAGAT

CGCTTCGATCTCACACTCTTCACTCGTGTCTACTTATCTTTGTTGCTTTGACGAGTCTTC

GAGCTTGATCGTTCGACGCGAGTGAGAGACTTTCGTCTCTCGTTTCGCGAGCGCTCGTCA

AAGACTTTGTTAACTTTGTTTTAGTGTCTTTCGAGCAATCATGTAATATTTAAAACTTTC

AACAACGAAT

>ASV341 GS|100.0|UDB01523021|SH0947923.09FU;k:Fungi,p:Ascomycota,c:Sordariomycetes,o:Hypocreales,f:Bionectriaceae

AAGTCGTAACAAGGTCTCCGTTGGTGAACCAGCGGAGGGATCATTACCGAGTTTTCAACT

CCCAAACCCATGTGAACATACCACTGTTGCTTCGGCGGACCGCCCCGGGTGCTGCGTGCC

CCGGAACCAGGCGCCCGCCGGAGGCCTAAACTCTTGTTTTTACCATGAATCTTCTGAGTA

GCGCAAGCAAAAAAAATAAATCAAAACTTTCAACAACGGATCTCTTGGTTCTG

>ASV342 GS|99.6|AY667583|SH0954634.09FU;k:Fungi,p:Ascomycota,c:Lecanoromycetes,o:Caliciales,f:Caliciaceae,g:Buellia,s:Buellia frigida

AAGTCGTAACAAGGTTTCCGTAGGTGAACCTGCGGAAGGATCATTACCGAGAGACGGGGT

CGCTTCGGCCCCACTCTTCACCCGTGTCTACTTACCTTTGTTGCTTTGGCGGGCCTTCGG

GCTTGACCGTTCGACGCGGGTGGGAGGCTTTCGCCTCTCGCCTCGCGAGCGCTCGCCAAA

GGCCCTGTTAACTCTGTTTTAGTGTCTTCCGAGCAACCATGTAATAGTTAAAACTTTCAA

CAACGGATCT

>ASV345 GS|75.1|UDB01703013|SH1008128.09FU;k:Rhizaria,p:Cercozoa

AAGTCGTAACAAGGTCTTCGTAGGTGAACCTGCGAAGGGATCATTAACACGTTCCAAACC

TATTATCAACCCATCTGTGAACTCATGTTACCGCCGAAATGGCTGGCAACAAGTGTATGG

CGCATACGATCGACTCTATGATGTGAGGTCTTAGCTGCGAGTTAGTGTAACAACTAGCTC

GTTCTTTAGCCCTGGTCTAGTATGCCTCGTGCTTCTACTCATCTCTCGTCTCGCACGAGA

GTTGAGTCAG

>ASV346 SS|1.0000|AF250779|SH0954634.09FU;k:Fungi,p:Ascomycota,c:Lecanoromycetes,o:Caliciales,f:Caliciaceae,g:Buellia,s:Buellia frigida

CTTTTTCCTTTCTCTTCCTTCCCCTTCTTCCCCCTTTTTCCGTATTTTCCCCTGCGGACT

TCTCCTTACCGATCTCCTTGGTCGCTTCTTCCCCACTCTTCCCCCTTGTCTACTTACCTT

TGTTGCTTTGGCGGGCCTTCGGGCTTGACCGTTCGCCGCGGGTGGGAGGCTTTCGCCTCT

CGCCTCGCGCGCGCCCGCCAAAGGCCCTGTTAACTCTGTTTTAGTGTCTTCCGAGCAACC

ATGTAATAGT

>ASV347 GS|89.2|AF250779|SH0954634.09FU;k:Fungi,p:Ascomycota,c:Lecanoromycetes,o:Caliciales,f:Caliciaceae,g:Buellia,s:Buellia frigida

AAGTCGTAACAAGATTTTCGTAGATGAATCTACGAAAGAATCATTATCGAGAGACGAGGT

CGCTTCGACCCCACTCTTCACTCGTATCTACTTACCTTTGTTGCTTTGGCGAGTCTTCGA

GCTTGATCGTTCGACGCGAGTGAGAGACTTTCGTCTCTCGCCTTGCGAGCGCTCGCCAAA

GGCTTTGTTAACTTTGTTTTAGTGTCTTCCGAACAATCATGTAATAGTTAAAACTTTCAA

CAACGAATTT

>ASV348 SS|0.9100|MN592663|SH1107827.09FU;k:Fungi,p:Ascomycota,c:Lecanoromycetes,o:Teloschistales,f:Teloschistaceae

AAGTCGTAACAAGGTTTCCGTAGGTGAACCTGCGGAAGGATCATTATCGAGAGGGGGGCT

CCATGCCCCGGGCTTCTGTCCCCGTACCTTTTCACCCTGTGTGTATTTTTCCCCCGTTGC

TTTGGCGGGCCCCGGGTCTTCCCCCGGCGTTGGCCCCCTCGCGGGGTTCGCGAGCGCCCG

CCGAAGGCTCATCGAAACTCTGTTGATCAGTGCAGTCTGAGCGTACGAACAATAAATCAA

AACTTTCAAC

>ASV349 GS|97.9|KF618067|SH0955201.09FU;k:Fungi,p:Ascomycota,c:Sordariomycetes,o:Hypocreales

AAGTCGTAACAAGGTCTCCGTTGGTGAACCAGCGGAGGGATCATTACCGAGTCTTTACAA

CTCCCAAACCCCTGTGAACATACCATCGTTGCTTCGGCGGATCACCCCGGGCGCCTCGGC

GCCCCGGGCCCAGGTGCCCGCCGGAGGACCCAAACTCTTGTTTTAGCAGTGGCATCTTCT

GAGTAATTTTATAAATAAATCAAAACTTTCAACAACGGATCTCTTGGTTCTG

>ASV350 SS|1.0000|MT237021|SH1031428.09FU;k:Fungi

AAGTCGTAACAAGGTTTCCGTAGGTGAACCTGCGGAAGGATCATTACCGAGTTCATGCCC

CCCGGGGTAGATCTCCCACCCTTGCTTGAATTACCTTGTTGCTTTGGCGGGCCGCTCTCT

TTGAAAGCCACCGGCTCCCCGCTGGTGAGTGCCCGCCGAAGACCAACCAACCTCGTGTCA

CTGCAGCAGTCTAAGTAATATACAAATTGAAATAAAACTTTCAACAACGGATCTCTTGGC

TCTG

>ASV351 GS|85.8|AF250779|SH0954634.09FU;k:Fungi,p:Ascomycota,c:Lecanoromycetes,o:Caliciales,f:Caliciaceae,g:Buellia,s:Buellia frigida

AAGTCGTAACAAAATTTTCGTAGATGAATTTGCGAAAGAATCATTATCGAGAGACGAGAT

CGCTTCGATCTCACTCTTCACTCGTATCTACTTATCTTTGTTGCTTTGACGAGCCTTCGA

ACTTGATCGTTCGACGCGAGTGAGAGGCTTTCGCCTCTCGTCTCGCGAGCGTTCGTCAAA

GACTTTGTTAACTTTGTTTTAGTGTCTTTCGAGCAATCATGTAATAATTAAAACTTTCAA

CAATGAATCT

>ASV352 SS|0.9900|AF250779|SH0954634.09FU;k:Fungi,p:Ascomycota,c:Lecanoromycetes,o:Caliciales,f:Caliciaceae,g:Buellia,s:Buellia frigida

AAGTCGTAACAAGATTTTCGTAAATGAATCTGCGGAAGAATCATTATCGAGAGACGGGGT

CGCTTCGACCTCACTCTTCACTCGTGTCTACTTATCTTTGTTGCTTTGGCGGGCCTTCGG

GCTTGATCGTTCGACGCGGGTGGGAGGCTTTCGCCTCTCGTCTCGCGAGCGCTCGTCAAA

GACCCTGTTAACTCTGTTTTAGTGTCTTCCGAGCAATCATGTAATAGTTAAAACTTTCAA

CAACGGATCT

>ASV353 SS|1.0000|FR682176|SH1068012.09FU;k:Fungi

AAGTCGTAACAAGGTTTCCGTAGGTGAACCTGCGGAAGGATCATTACTGAGATAGGGTCC

TCCGGGGCCCGACCTCCAACCCTTTGTCTACCTTACCACTGTTGCCTCGGCGGGTCGCCG

GCGCTCTCACGGGCGTCGCCGTCGGCCTCCGCGCTCTCGAGTGCCCGCCGCTAGGCCCCC

TAAATCCTTTATAAATCAGTCTTCTGAGAGTATATTCAATACTTAAAACTTTCAACAACG

GATCTCTTGG

>ASV354 GS|80.5|MK970687|SH0954634.09FU;k:Fungi,p:Ascomycota,c:Lecanoromycetes,o:Caliciales,f:Caliciaceae,g:Buellia,s:Buellia frigida

AAGTCGTAATAAGATTTTCGTAAATAAATCTACGAAAGAATCATTATCGAGAAACGAAAT

CGCTTCGATCTCACTCTTCACTCGTGTCTACTTATCTTTGTTGTTTTGACGAGTTTTCGA

ACTTGATCGTTCGATGCGAGTGAGAGACTTTCGTTTCTCGTTTTGCGAGCGCTCGTCAAA

GATTTTGTTAACTTTGTTTTAGTGTTTTTCGAACAATCATGTAATAGTTAAAACTTTCAA

CAATGAATCT

>ASV355 SS|1.0000|AF250779|SH0954634.09FU;k:Fungi,p:Ascomycota,c:Lecanoromycetes,o:Caliciales,f:Caliciaceae,g:Buellia,s:Buellia frigida

AAGTCGTAACAAGGTTTCCGTAAGTGAACCTACGAAAGAATCATTATCGAGAGACGAGGT

CGCTTCGACCCCACTCTTCACTCGTGTCTACTTACCTTTGTTGCTTTGGCGAGCCTTCGA

GCTTGATCGTTCGACGCGGGTGAGAGGCTTTCGCTTGTCGCCTCGCGAGCGCTCGCCAAA

GGCCTTGTTAACTCTGTTTTAGTGTCTTCCGAGCAATCATGTAATAGTTAAAACTTTCAA

CAACGGATCT

>ASV356 GS|99.2|AY667583|SH0954634.09FU;k:Fungi,p:Ascomycota,c:Lecanoromycetes,o:Caliciales,f:Caliciaceae,g:Buellia,s:Buellia frigida

AAGTCGTAACAAGGTTTCCGTAGGTGAACCTGCGGAAGGATCATTACCGAGAGACGGGGT

CGCTTCGGCCCCACTCTTCACCCGTGTCTACTTACCTTTGTTGCTTTGGCGGGCCTTCGA

GCTTGACCGTTCGACGCGGGTGGGAGGCTTTCGCCTCTCGCCTCGCGAGCGCCCGCCAAA

GGCCCTATTAACTCTGTTTTAGTGTCTTCCGAGCAACCATGTAATAGTTAAAACTTTCAA

CAACGGATCT

>ASV357 GS|98.0|KC179119|SH1085530.09FU;k:Fungi,p:Ascomycota,c:Lecanoromycetes,o:Teloschistales,f:Teloschistaceae,g:Shackletonia,s:Shackletonia insignis

AAGTCGTAACAAGGTTTCCGTAGGTGAACCTGCGGAAGGATCATTATCGAGAGCGGGGCT

TCGCGCCCCCGGGGGGCTTCGGCCTCCTACCTCTTCACCCTGTGTATATCAACCTCTGTT

GCTTTGGCGAGCCCTGGGTTTACACCCGGCACGGTTCCGGGCGATCCTGCCCGGAGTCGC

GGGTGCTCGCCCGAGGCCTTTAACCATCTGTTTACGTGGAACGTCCGAATGACCCAAAAA

TTAAATAAAA

>ASV358 GS|99.2|AY667583|SH0954634.09FU;k:Fungi,p:Ascomycota,c:Lecanoromycetes,o:Caliciales,f:Caliciaceae,g:Buellia,s:Buellia frigida

AAGTCGTAACAAGGTTTCCGTAGGTGAACCTGCGGAAGGATCATTACCGAGAGACGGGGT

CGCTTCGGCCCCACTCTTCACCCGTGTCTACCTACCTTTGTTGCTTTGGCGGGCCTTCGG

GCTTGACCGTTCGACGCGGGTGGGAGGCTTTCGCCTGTCGCCTCGCGAGCGCCCGCCAAA

GGCCCTGTTAACTCTGTTTTAGTGTCTTCCGAGCAACCATGTAATAGTTAAAACTTTCAA

CAACGGATCT

>ASV359 GS|84.0|AY667583|SH0954634.09FU;k:Fungi,p:Ascomycota,c:Lecanoromycetes,o:Caliciales,f:Caliciaceae,g:Buellia,s:Buellia frigida

AAGTCGTAACAAGATTTTCGTAGATGAATTTGCGAAAGAATCATTATCGAGAGACGAAAT

CGCTTCGATCTCACTCTTCACTCGTGTCTACTTATCTTTGTTGCTTTGACGAATCTTCGA

GCTTGATCATTCGACGCGAGTGAGAGACTTTCGTCTCTCGTCTTGCGAGCGCTCGTCAAA

GATTTTGTTAACTCTATTTTAGTGTCTTTCGAGTAATCATGTAATAGTTAAAACTTTCAA

CAACGAATCT

>ASV360 GS|100.0|UDB05295406|SH0991924.09FU;k:Fungi,p:Ascomycota,c:Dothideomycetes,o:Mycosphaerellales,f:Teratosphaeriaceae,g:Elasticomyces,s:Elasticomyces elasticus

AAGTCGTAACAAGGTCTCCGTAGGTGAACCTGCGGAGGGATCATTACCGAGTGAGGGCCT

CCGGGCCCGACCTCCAACCCCATGTTAACCGACCCTGTTGCCTCGGGGGCGACCCGGACT

CGTGCCGGGGCCCCCGATGGACCTTCCAACACTGCATCTTTGCGTCCGAGTCAATATTTG

AATAAATCAAAACTTTCAACAACGGATCTCTTGGTTCTG

>ASV361 GS|0.0|None;No hit

GTGTCAGCAGCCGCGGTAATACGAAGGGGGCTAGCGTTGCTCGGAATGACTGGGCGTAAA

GGGCGCGTAGGCGGATGTTTTAGTCAGGCGTGAAAGTCCTGGGCTCAACCTGGGGATTGC

GTTTGATACGGGGCATCTAGAGTTGGGAAGAGGGTCGTGGAATTCCCAGTGTAGAGGTGA

AATTCGTAGATATTGGGAAGAACACCGGTGGCGAAGGCGGCGACCTGGTCCTTGACTGAC

GCTGAGGCGC

>ASV362 GS|0.0|None;No hit

GTGTCAGCAGCCGCGGTAATACGAAGGGGGCTAGCGTTGCTCGGAATGACTGGGCGTAAA

GGGCGCGTAGGCGGATTGGTCAGTCAGACGTGAAATTCCTGGGCTTAACCTGGGGGCTGC

GTTTGAGACGGCTGGTCTAGAGTTTGGAAGAGGGTCGTGGAATTCCCAGTGTAGAGGTGA

AATTCGTAGATATTGGGAAGAACACCGGTGGCGAAGGCGGCGACCTGGTCCTGGACTGAC

GCTGAGGCGC

>ASV363 GS|84.8|AF250779|SH0954634.09FU;k:Fungi,p:Ascomycota,c:Lecanoromycetes,o:Caliciales,f:Caliciaceae,g:Buellia,s:Buellia frigida

AAGTCGTAACAAGATTTTCGTAAGTGAATCTACGAAAGAATCATTATCGAGAGACGAGAT

CGCTTCGATCTCACTCTTCACTCGTATCTACTTATCTTTGTTGCTTTGACGAGTCTTCGA

ATCTAATCGTTCGACGCGAGTGAAAGACTTTCGTCTCTCGCCTCGCGAGCGCTCGTCAAA

GACTCTATTAACTCTGTTTTAGTGTCTTTCGAGCAATCATGTAATAGTTAAAACTTTCAA

CAACGAATCT

>ASV364 GS|99.6|AY667583|SH0954634.09FU;k:Fungi,p:Ascomycota,c:Lecanoromycetes,o:Caliciales,f:Caliciaceae,g:Buellia,s:Buellia frigida

AAGTCGTAACAAGGTTTCCGTAGGTGAACCTGCGGAAGGATCATTACCGAGAGACGGGGT

CGCTTCGGCCCCACTCTTCACCCGTGTCTACTTACCTTTGTTGCTTTGGCGGGCCTTCGG

GCTTGACCGTTCGACGCGGGTGGGAGGCTTTCGCCTCTCGCCTCGCGAGCGCCCGCCAAA

GGCCCTGTCAACTCTGTTTTAGTGTCTTCCGAGCAACCATGTAATAGTTAAAACTTTCAA

CAACGGATCT

>ASV365 GS|84.8|AF250779|SH0954634.09FU;k:Fungi,p:Ascomycota,c:Lecanoromycetes,o:Caliciales,f:Caliciaceae,g:Buellia,s:Buellia frigida

AAATCGTAACAAGATTTTCGTAGATGAACTTGCGAAAGAATCATTATCGAGAGACGAAAT

CGCTTCGATTTCACTCTTCACTCGTGTCTACTTATCTTTGTTGCTTTGACGAGTCTTCGA

GCTTGATCGTTCGACGCGAGTGAGAGACTTTCGTCTTTCGTCTCGCGAGCGTTCGTCAAA

GACTTTGTTAACTCTATTTTAGTGTCTTTCGAGCAATCATGTAATAGTTAAAACTTTCAA

CAACGAATCT

>ASV366 GS|99.1|FJ392866|SH1071027.09FU;k:Fungi,p:Ascomycota,c:Eurotiomycetes,o:Chaetothyriales,f:Trichomeriaceae,g:Knufia

AAGTCGTAACAAGGTTTCCGTAGGTGAACCTGCGGAAGGATCATTACCGAGTTAGGGTCT

CTTCGGAGCCCGAACCTCCCAACCCTTTGTCTAATTTACCTGGTCGTTGCTTCGGCGGAC

CGGTTGACCAACTGGTCTTGACCGCCGGGGGTCCCGTACCCCTGGAGAGCGTCCGCCGAC

GGCCCAACCACAAACTCTTGTACTAAACCATGTCGTCTGAATGTACTTTGATATTAATCA

AAAAACAAAA

>ASV367 GS|99.6|MW991411|SH0964445.09FU;k:Fungi,p:Ascomycota,c:Arthoniomycetes,o:Lichenostigmatales,f:Phaeococcomycetaceae,g:Antarctolichenia,s:Antarctolichenia onofrii

AAGTCGTAACAAGGTCTCCGTAGGTGAACCTGCGGAGGGATCATTAATGAGATAGGGTCT

TCACGGCCCGACCTCCAACCCAATGTCTACCATACCTCTGTTGCCTCGGCGGGCCGCCGG

CGTCCTTTGTTGGGCGCCGCCGTCGGCTTTAGGGCTTTCGAGCGCCCGCCGCAGGACCGA

TTAAACTCTTTTTAAAACAAGTCTTCTGAGTGGGGAAATCAAATTTATTAAAACTTTTAA

CAACGGATCT

>ASV368 GS|78.8|AY667583|SH0954634.09FU;k:Fungi,p:Ascomycota,c:Lecanoromycetes,o:Caliciales,f:Caliciaceae,g:Buellia,s:Buellia frigida

AAGTCGTAACAAAATTTTCGTAAATGAACTTGCGAAAAAATCATTATCGAAAGACGAAAT

CGCTTCGATCTTACTTTTCACTCGTGTCTACTTATCTTTGTTGCTTTGACGAATCTTCGA

ATTTGATCGTTCGATGCGAATGAGAGATTTTCGTCTTTCGTTTCGCGAGCGTTCGTTAAA

GACTCTATTAATTTTGTTTTAGTGTCTTTCGAACAATCATGTAATAGTTAAAACTTTTAA

CAACGAATCT

>ASV369 GS|83.3|AF250779|SH0954634.09FU;k:Fungi,p:Ascomycota,c:Lecanoromycetes,o:Caliciales,f:Caliciaceae,g:Buellia,s:Buellia frigida

AAGTCGTAACAAGATTTTCGTAGATGAATCTACGAAAAAATCATTATCGAGAGACGAGAT

CGCTTCGATCTCATTCTTCACTCGTATCTACTTATCTTTGTTGCTTTGACGAGTCTTTGA

ATTTGATCGTTCGACGCGAGTGAAAGGCTTTCGTCTATCGTTTCGCGAGCGCTCGTCAAA

GACCTTGTTAACTTTGTTTTAATGTCTTTCGAGCAATCATGTAATAGTTAAAACTTTCAA

CAACGAATCT

>ASV370 GS|86.8|AF250779|SH0954634.09FU;k:Fungi,p:Ascomycota,c:Lecanoromycetes,o:Caliciales,f:Caliciaceae,g:Buellia,s:Buellia frigida

AAGTCGTAACAAGATTTTCGTAAGTGAATTTGCGAAAGAATCATTATCGAGAGACGAGGT

CGCTTCGACTTCACTCTTCACTCGTGTCTACTTATCTTTGTTGCTTTGACGAGTCTTCGA

ACTTGATCGTTCGACGCGAGTGAGAGACTTTCGTCTCTCGTCTCGCGAGCGCTCGTCAAA

GACTTTGTTAACTTTGTTTTAGTGTCTTTCGAGCAATCATGTAATAGTTAAAACTTTCAA

CAACGAATCT

>ASV371 GS|99.6|AY667583|SH0954634.09FU;k:Fungi,p:Ascomycota,c:Lecanoromycetes,o:Caliciales,f:Caliciaceae,g:Buellia,s:Buellia frigida

AAGTCGTAACAAGGTTTCCGTAGGTGAACCTGCGGAAGGATCATTACCGAGAGACGGGGT

CGCTTCGGCCCCACTCTTCACCCGTGTCTACTTACCTTTGTTGCTTTGGCGGGCCTTCGG

GCTTGACCGTTCGACGCGGGTGGGAGGCTTTCGCCTCCCGCCTCGCGAGCGCCCGCCAAA

GGCCCTGTTAACTCTGTTTTAGTGTCTTCCGAGCAACCATGTAATAGTTAAAACTTTCAA

CAACGGATCT

>ASV372 GS|100.0|AF279772|SH1300522.09FU;k:Fungi,p:Ascomycota,c:Lecanoromycetes,o:Teloschistales,f:Teloschistaceae,g:Xanthoria,s:Xanthoria elegans

AGGTCGTAACAAGGTTTCCGTAGGTGAACCTGCGGAAGGATCATTACTAAGAGAGGGATG

TACGCTTCCAGCCGAGTCCCGGGGGGCTGCGCCCCTCACCTCTTCAACCCTGTGTCTACC

AACCGCTGTTGCTTCGGCGAGCGTCGGGGCGTCCGCGCCCCGGCCCCGGCTTCGGTCGGT

GAGCTCTCGCAGAGGCCTATCTTTATTCTGTTTTGCAGTGACGTCCGAGAATACCAATAT

AATCAATCAA

>ASV373 SS|1.0000|KF274227|SH0911134.09FU;k:Fungi

AAGTCGTAACAAGGTTTCCGTAGGTGAACCTGCGGAAGGATCATTACCGAGTTAGGGTCC

TCTGGGCTCGATCTCCAACCCTGTGTCTAAATAACCACGTTGCTTTGGCGGGCCCGCCTG

TAATGGGCCGCCGGGGGTGCCTTCGGCCCCCTGGTCAGCGCCTGCCAGTAGCCATCTCAA

ACTCTTCTTAATCGTGACGTCTGAGTAAAAATTATAAATTAAACAAAACTTTCAACAACG

GATCTCTTGG

>ASV374 GS|88.2|AF250779|SH0954634.09FU;k:Fungi,p:Ascomycota,c:Lecanoromycetes,o:Caliciales,f:Caliciaceae,g:Buellia,s:Buellia frigida

AAATCGTAACAAGATTTCCGTAGATGAATCTACGAAAGAATCATTATCGAGAGACGAGAT

CGCTTCGATCTCACTCTTCACTCGTGCCTACTTATCTTTGTTGCTTTGACGAGTCTTCGA

GCTTGACCATTCGACGCGAGTGAGAGACTTTCGTCTCTCGCCTCGCGAGCGCTCGCCAAA

GGCTCTGTTAACTCTATTTTAGTGTCTTCCAAGCAATCATGTAATAGTTAAAACTTTCAA

CAACGAATCT

>ASV375 GS|86.4|AY667583|SH0954634.09FU;k:Fungi,p:Ascomycota,c:Lecanoromycetes,o:Caliciales,f:Caliciaceae,g:Buellia,s:Buellia frigida

AAGTCGTAACAAGGTTTTCGTAGATGAATTTGCGAAAGAATCATTATCGAGAGACGAGAT

CGCTTCGATCTCACTCTTCACTCGTGTCTACTTATCTTTGTTGCTTTGACGGGTCTTTGG

GCTTGATCGTTCGACGCGAGTGAGAGACTTTCGTCTCTCGCTTTGCGAGCGCTCGTCAAA

GATCTTGTTAACTTTGTTTTAGTGTCTTTCGAGCAATCATGTAATAGTTAAAACTTTCAA

CAATGAATCT

>ASV376 GS|85.8|AF250779|SH0954634.09FU;k:Fungi,p:Ascomycota,c:Lecanoromycetes,o:Caliciales,f:Caliciaceae,g:Buellia,s:Buellia frigida

AAGTCGTAACAAGATTTTCGTAGATGAATTTGCGAAAAAATCATTATCGAGAGACGAAAT

CGCTTCGACTTCACTCTTCACTCGTGTCTACTTATCTTTGTTGCTTTGACGAGTCTTCGA

GCTTGATCGTTCGACGCGAGTGAGAGACTTTCGTCTCTCGTCTCGCGAGCGTTCGTCAAA

GACTTTGTTAACTTTGTTTTAGTGTCTTTCGAGCAATCATGTAATAGTTAAAACTTTCAA

CAACGAATCT

>ASV377 SS|1.0000|KF274145|SH0988164.09FU;k:Fungi

AAGTCGTAACAAGGTTTCCGTAGGTGAACCTGCGGAAGGATCATTAATGAATGCAAGGAT

ACTCTTTTTAGAGGTCCGACTCATCATTTTCCAACCCTGTGCACATACACTGTTGTTTTA

CACATCATTTTTAAACACAAAGTCTATAAGAATGTATGAAAGTCTCTTGATTGAGCACAT

AAATTAAACAAAACTTTCAGCAACGGATCTCTTGGCTCTC

>ASV378 GS|84.4|AY667583|SH0954634.09FU;k:Fungi,p:Ascomycota,c:Lecanoromycetes,o:Caliciales,f:Caliciaceae,g:Buellia,s:Buellia frigida

AAGTCGTAACAAGATTTTCGTAGATGAATCTACGAAAGAATCATTATCGAGAGACGAGAT

CGCTTTGATCTCACTCTTCATTCGTGCCTACTTATCTTTGTTGCTTTGACGAGCCTTCGA

GCTTGATCGTTCGACGCGAGTGAGAGGCTTTCGTCTCTCGTCTCGCGAACGCTCGTCAAA

GATTTTGTTAACTTTGTTTTAGTGTCTTTCAAGCAATCATGTAATAGTTAAAACTTTCAA

CAACGAATTT

>ASV379 GS|85.8|AF250779|SH0954634.09FU;k:Fungi,p:Ascomycota,c:Lecanoromycetes,o:Caliciales,f:Caliciaceae,g:Buellia,s:Buellia frigida

AAGTCGTAACAAGATTTTCGTAGATGAATTTGCGAAAGAATCATTATCGAGAGACGAGAT

CGCTTCGATCTCACTCTTCACTCATGTCTACTTATCTTTGTTGCTTTGACGAGTTTTCGA

GCTTGATCGTTCGACGCGAGTGAGAGGCTTTCGTCTCTCGTTTCGCGAGCGCTCGTCAAA

GACTTTGTTAACTCTATTTTAGTGTCTTTCGAGCAATCATGTAATAGTTAAAACTTTCAA

CAACGAATCT

>ASV380 GS|82.4|AY667583|SH0954634.09FU;k:Fungi,p:Ascomycota,c:Lecanoromycetes,o:Caliciales,f:Caliciaceae,g:Buellia,s:Buellia frigida

AAGTCGTAACAAGATTTTCGTAAATGAATTTGCGAAAGAATCATTATCGAGAGACGAAAT

CGCTTCGATCTCATTCTTCACTCGTGTCTACTTATCTTTGTTGCTTTGACGAATTTTCGA

ACTTGATCGTTCGACGCGAGTGAGAGACTTTCGTCTCTCGTTTCGCGAGCGTTCGTCAAA

GATCTTGTTAACTTTGTTTTAGTGTCTTTCGAACAATCATGTAATAATTAAAACTTTCAA

CAACGAATCT

>ASV381 GSL|100.0|DQ534488|SH0920692.09FU;k:Fungi,p:Ascomycota,c:Lecanoromycetes,o:Lecanorales,f:Parmeliaceae,g:Usnea,s:Usnea aurantiacoatra

AAGTCGTAACAAGGTTTCCGTAGGCGGAACATTGCGTCCTTTCGGGTACATCTGCTGAAA

CCTTAGTGAATTGATTAGGCGACTCTAAACAAGTATAATCCTCGGGGCTTGCCCTTGAGC

ACTAGCCATTTCTTTTAGATGGCAACATTACCAGTATGCGGGAACATTCAACTAAGTTGG

ATAACCCGCAGTCACATTTGTGGCCCACAGGCTAAATGGTAATGGGCATAGATGCAAGGC

AGCTATGCTT

>ASV382 SS|0.8400|MZ229889|SH0913114.09FU;k:Fungi,p:Ascomycota,c:Lecanoromycetes,o:Caliciales

AAGTCGTAACAAGGTTTCCGTAGGTGAACCTGCGAAAGGATCATTATCGAGAGACGGAGT

CTAACCGGCCCCACTCTTCACCCGTGTATACCTACCCTTTATTGCTTTGGCGGGCGGTCG

GTCTTTACCGCCCGACGTCGGTCGGAAGGCTCGCGCCTTCCACTCACCGAACGCCCGCCA

GAGGCCCCCAAAAGCCGTCCATCTGTGTCCGAGCGAGTGAAGCAAATAGTTAAAAACTTT

CAACAACGGA

>ASV383 GS|84.8|AF250779|SH0954634.09FU;k:Fungi,p:Ascomycota,c:Lecanoromycetes,o:Caliciales,f:Caliciaceae,g:Buellia,s:Buellia frigida

AAGTCGTAACAAGATTTTCGTAGATGAATTTACGAAAGAATCATTATCGAGAGATGAAAT

CGCTTCGATCTCACTCTTCACTCGTGTCTACTTATCTTTGTTGCTTTGACGAGTCTTCGA

GCTTGATCGTTCGACGCGAGTGAGAGACTTTCGTTTCTCGTCTCGCGAGCGCTCGTCAAA

GATTCTATTAACTTTGTTTTAGTGTCTTTCGAGCAATCATGTAATAGTTAAAACTTTCAA

CAACGAATCT

>ASV384 SS|0.8700|MZ229889|SH0913114.09FU;k:Fungi,p:Ascomycota,c:Lecanoromycetes,o:Caliciales

AAGTCGTAACAAGGTTTCCGTAGGTGAACCTGCGGAAGGATCATTATCGAGAGACGGGGT

CTAACCGGCCCCACTCTTCACCCGTGTATACCTACCCTTTGTTGCTTTGGCGGGCGGTCG

GTCTTTACCGCCCGACGTCGGTCGGAAGGCTCGCGCCTTCCACTCACCGAACGCCCGCCA

GAGGCCCCCAAAACCCGTCCATCTGTGTCCGAGCGAGTGAAGCAAATAGTTAAAAACTTT

CAACAACGGA

>ASV385 GS|98.0|KX550105|SH0903330.09FU;k:Fungi,p:Ascomycota,c:Lecanoromycetes,o:Caliciales,f:Physciaceae,g:Physcia,s:Physcia dubia

AAGTCGTAACAAGGTTTCCGTAGGTACAAATAGGTTTGCCTGCCGAAACCCCCCGCAACG

ACGTTAAAAAACTGCGCGGTCTGCGCCCTTGTGGCGTGGGCAACACCGTCCAAGTGCTGA

GAATGCCCGGCAACAGCCCCGCACAACCAGCAGCCAGCGTGTCACCGCTGGTCCACAGAT

CAATCGACGGTGACCTTTATCGGTTAAGCTATGACCGGTCCAGAGCCCCAACGGGCCCTG

GAGCGACGGA

>ASV386 GS|100.0|KP903330|SH0989164.09FU;k:Fungi,p:Ascomycota,c:Dothideomycetes,o:Mycosphaerellales,f:Teratosphaeriaceae,g:Catenulostroma

AAGTCGTAACAAGGTCTCCGTAGGTGAACCTGCGGAGGGATCATTACCGAGCGAGGGTCC

TCCAGGCCCGACCTCTCAACCCCATGTGACCCGACACCGTTGCCTCGGGGGCGACCCGGC

CGTCTCGGCCGGGGCCCCCGGTGGACCGACCAAACACTGCGTCCGTGCGTCCGAGTCCCA

TGACAAATCAATCAAAACTTTCAACAACGGATCTCTTGGTTCTG

>ASV387 GS|77.8|AF224403|SH0903213.09FU;k:Fungi,p:Ascomycota,c:Lecanoromycetes,o:Caliciales,f:Physciaceae,g:Physcia,s:Physcia dubia

AAGTCGTAACAAGGTTTCCGTAGGTGAGAACAGGTTTGCCTCCGGAAAACCCCCGCAACG

ACGTTAAACAAGTGCGTGGTCTGCACCTTAGCAGTGCAGGCAACACCGTTCAAATGCTGA

GAGCACCGAGACACTCGGGGTAACCAGCAGCCAGCACGCTCGCGTCTGGTGCACAGATCA

TTCGATGGTGACCTTATGGTTGAGATATGACCGGCTCCAGGCCTTGAAAGCCCCGGAACC

TGCGGAACCT

>ASV388 SS|0.9900|None;k:Fungi,p:Ascomycota,c:Lecanoromycetes,o:Lecanorales,f:Parmeliaceae,g:Neoprotoparmelia,s:Neoprotoparmelia corallifera

GTGTCAGCAGCCGCGGTAATACGTAGAAGACTAGTGTTATTCATCTTTAATAGGTTTAAA

GGGTACCTAGACGGTAAATCAAGCCTGCAATATGGGACTAATGTACTAGAGTTACTTACG

AGGGGGTATTAAAGTACTGCTGGTGTAGAGATGAAATTCTGTCATACCTCATTTCATAAG

AAATACTATGGCACAGGTATAGGCGAAAGCATCCCCTTATGTGATAACTGACGTTGAAGG

ACGAAGGCTT

>ASV389 GS|99.0|UDB03378640|SH0910872.09FU;k:Fungi,p:Ascomycota,c:Eurotiomycetes,o:Chaetothyriales,f:Herpotrichiellaceae

AAGTCGTAACAAGGTTTCCGTAGGTGAACCTGCGGAAGGATCATTATCGAGTTAGGGTCT

TTACTGGCCCGATCTCCAACCCTGTGTCTATAATACCATGTTGCTTTGGCGGGCCCGCCT

TTAACGGGCCGCCGAGGGTCTTCGGACCCTTGGTCAGTGCCCGCCAGTAGCCAAACTAAA

TTCTTCTTAACTGTGTCGTCTGAGTAAATATTTAAATAAACAAAACTTTCAACAACGGAT

CTCTTGGTTC

>ASV390 GS|99.4|KF309963|SH1084061.09FU;k:Fungi,p:Ascomycota,c:Dothideomycetes

AAGTCGTAACAAGGTCTCCGTAGGTGAACCTGCGGAGGGATCATTACTGAGTGAGGGCCT

CCGGGTCCGACCTCCAACCCCATGTTATCTGACCCTGTTGCCTCGGGGGCGACCCGGCCT

TCGGGCGTCTTGGGGCCCCCGGTGGACCACTCAACACTGCATCTTTGCGTCTGAGTTATA

TTTTGAATCAATCAAAACTTTCAACAACGGATCTCTTGGTTCTG

>ASV391 GS|80.9|AF250779|SH0954634.09FU;k:Fungi,p:Ascomycota,c:Lecanoromycetes,o:Caliciales,f:Caliciaceae,g:Buellia,s:Buellia frigida

AAGTCGTAATAAAATTTTTATAAATGAATTTGCGAAAGAGATATTATCGAGAGACGAAAT

CGCTTCGATCTCACTCTTCATTCGTATCTACTTATCTTTGTTGCTTTGACGAATTTTCGA

ACTTGATCGTTCGACGCGAGTGAGAGACTTTCGTCTCTCGTTTTGCGAGCGTTCGTCAAA

GATTTTGTTAACTTTGTTTTAGTGTCTTTCGAGCAATCATGTAATAGTTAAAACTTTTAA

CAACGAATTT

>ASV392 GS|75.0|MN103133|SH0913114.09FU;k:Fungi,p:Ascomycota,c:Lecanoromycetes,o:Caliciales,f:Caliciaceae,g:Dimelaena,s:Dimelaena oreina

AAGTCGTAATAAGATTTTCGTAAGTGAATCTGCGAAAGGATCATTATCGAGAGACGAAAT

CTAACCGGCCCCACTCTTCACTCGTGTATATTTATTCTTTGTTGTTTTGACGGGCGGTCG

GTCTTTATCGCTCGACGTCGGTCGAAAAACTCGCGCCTTTCACTCATCGAACGCCCGCCA

GAGATTTTCAAAACTCGTTTATTTGTGTTCGAGCGAGTGAAATAAATAATTAAAAACTTT

CAACAACGGA

>ASV393 GS|88.7|AF250779|SH0954634.09FU;k:Fungi,p:Ascomycota,c:Lecanoromycetes,o:Caliciales,f:Caliciaceae,g:Buellia,s:Buellia frigida

AAGTCGTAACAAGATTTTCGTAAATGAATTTACGAAAGAATCATTATCGAGAGACGAGAT

CGCTTCGATCTCACTCTTCACTCGTGTCTACTTATCTTTGTTGCTTTGACGAGTCTTCGA

GCTTGATCGTTCGACGCGAGTGAGAGGCTTTCGCCTCTCGTCTCGCGAGCGCTCGTCAAA

GACTCTGTTAACTCTGTTTTAGTGTCTTTCGAGCAATCATGTAATAGTTAAAACTTTCAA

CAACGAATCT

>ASV394 GS|87.3|AF250779|SH0954634.09FU;k:Fungi,p:Ascomycota,c:Lecanoromycetes,o:Caliciales,f:Caliciaceae,g:Buellia,s:Buellia frigida

AAGTCGTAACAAGATTTTCGTAGATGAACTTGCGAAAGAATCATTATCGAGAGACGAAGT

CGCTTCGATCTCACTCTTCACTCGTGTCTACTTATCTTTGTTGCTTTGACGAGCCTTCGA

GCTTGATCGTTCGACGCGAGTGAGAGACTTTCGTCTCTCGTCGCGCGAGCGCTCGTCAAA

GACTCTGTTAACTTTGTTTTAGTGTCTTTCGAGCAATCATGTAATAGTTAAAACTTTCAA

CAACGAATCT

>ASV395 GS|72.6|UDB0243452|SH1241595.09FU;k:Fungi,p:Basidiomycota,c:Agaricomycetes,o:Agaricales,f:Inocybaceae,g:Inocybe

AAGTCGTAACAAGGTTTCCGTAGGTGAACCTGCGGAAGGATCATTATGAATGCGTTTAGC

AATTAACTGTGTATTATCTATATTTGTCATTTGCGAGCTAGCGTAGCTAGTGAGCAGTGA

TTGTGTCGATGGTTGATTATTTATAGTCGATCACGATTCAATCGGTGACAGTCTTACTAT

TACAATTGTCTGAATGAGAATAATTAAAAACTCTCAACAATGGATCTCTTGGCTCTG

>ASV396 GS|85.3|AF250779|SH0954634.09FU;k:Fungi,p:Ascomycota,c:Lecanoromycetes,o:Caliciales,f:Caliciaceae,g:Buellia,s:Buellia frigida

AAGTCGTAACAAGATTTTCGTAGATGAACTTGCGAAAAAATCATTATCGAGAGACGAGAT

CGCTTCGATTTCACTCTTCACTCGTGTCTACTTATCTTTGTTGCTTTGACGAATCTTCGA

GCTTGATCGTTCGACGCGAGTGAAAGGCTTTCGTCTCTCGTCTTGCGAGCGCTCGTCAAA

GACTTTGTTAACTTTGTTTTAGTGTCTTTCGAGCAATCATGTAATAGTTAAAACTTTCAA

CAACGAATCT

>ASV397 SS|0.9000|MZ229889|SH0913114.09FU;k:Fungi,p:Ascomycota,c:Lecanoromycetes

AAGTCGTAACAAGGTTTCCGTAGGTGAACCTGCGGAAGGATCATTATCGAGAGACGGAGT

CTAACCGGCCCCACTCTTCACCCGTGTATACCTACCCTTTGTTGCTTTGGCGGGCGGTCG

GTCTTTACCGCCCGACGTCGGTCGGAAGGCTCGCGCCTTCCACTCACCGAACGCCCGCCA

GAGGTTCCCAAAACCCGTCCATCTGTGTCCGAGCGAGTGAAGCAAATAGTTAAAAACTTT

CAACAACGGA

>ASV398 SS|0.8800|UDB01720327|SH1085256.09FU;k:Rhizaria,p:Cercozoa

AAGTCGTAACAAGGTCTTCGTAGGTGAACCTGCGAAGGGATCATTAACACGTTCCATACC

TCAACTATCAACCCATCTGTGAACCTTTGTTACCGCCGAATGGCTAACTACAAGTGTATG

GCGCATACGATCGACTCAACGATGTTGTAGGACTCACTGTTCTGCATGGTCTAGTAGTAT

GCCCTCTGTGTCCGTCACACGACAGCTATCCTTGTATGTTGGTCCGGCCACTATATATGC

GCTTCGACGG

>ASV399 GS|99.2|JX036119|SH0942719.09FU;k:Fungi,p:Ascomycota,c:Lecanoromycetes,o:Lecanorales,f:Lecanoraceae,g:Rhizoplaca

AAGTCGTAACAAGGTTTCCGTAGGTGAACCTGCGGAAGGATCATTATCGAGAGGGGTCCC

CGGACTCCGGGGGCTTCGGCCCCCTACTCTTCACCCTATGTCTACACACCTTTGTTGCTT

TGGCGGGCCTCGGGTTCGCCCCGTACCTGCCGTGGGCTTCCATACCCCGGCCGTCCGTGC

CCGTCAGAGGCCCATGAACCCTCGTTTATCAGTGTCGTCCGAGTCCAACCATAATAGCAA

AAACTTTCAA

>ASV400 SS|1.0000|AF250779|SH0954634.09FU;k:Fungi,p:Ascomycota,c:Lecanoromycetes,o:Caliciales,f:Caliciaceae,g:Buellia,s:Buellia frigida

AAGTCGTAACAAGGTTTTCGTAGGTGAATCTACGAAAGAATCATTATCGAGAGACGGGGT

CGCTTCGGTCTCACACTCTTCACTCGTGTCTACTTATCTTTGTTGCTTTGGCGGGCCTTC

GAGCTTGATCGTTCGACGCGAGTGGGAGGCTTTCGCCTCTCGTCTCGCGAGCGCTCGTCA

AAGATTCTGTTAACTCTGTTTTAGTGTCTTTCGAGCAATCATGTAATATTTAAAACTTTC

AACAACGAAT

>ASV401 GS|90.2|MK970687|SH0954634.09FU;k:Fungi,p:Ascomycota,c:Lecanoromycetes,o:Caliciales,f:Caliciaceae,g:Buellia,s:Buellia frigida

AAGTCGTAACAAGATTTTCGTAGATGAACCTACGAAAGGATCATTATCGAGAGACGAGGT

CGCTTCGACCCCACTCTTCACTCGTGTCTACTTACCTTTGTTGCTTTGACGAGCCTTCGA

GCTTGATCGTTCGACGCAAGTGAGAGACTTTCGTCTCTCGCCTCGCGAGCGCTCGTCAAA

GGCCTTGTTAACTCTATTTTAGTGTCTTTCGACCAACCATGTAATAGTTAAAACTTTCAA

CAACGAATCT

>ASV402 GS|0.0|None;No hit

AAGTCGTAACAAAATTTTCGTAAATGAATTTACAAAAAAATCATTATTGAGAGACGAAAT

CGTTTCGATCTCACTCTTTATTCGTATCTACTTATCTTTGTTGCTTTAACGAATCTTCGA

ACTTAATCGTTCGACGCGAATGAGAGACTTTCGTCTTTCGTTTCGCGAGCGTTTGTCAAA

GACTTTGTTAACTTTGTTTTAATGTCTTTCAAGCAATCATGTAATAATTAAAACTTTTAA

TAACGAATCT

>ASV403 GS|87.3|AF250779|SH0954634.09FU;k:Fungi,p:Ascomycota,c:Lecanoromycetes,o:Caliciales,f:Caliciaceae,g:Buellia,s:Buellia frigida

AAGTCGTAACAAGGTTTTCGTAAGTGAATTTGCGAAAGAATCATTATTGAGAGACGAGAT

CGCTTCGATCTCACTCTTCACTCGTGTCTACTTATCTTTGTTGCTTTGACGAGTTTTCGA

GCTTGATCGTTCGACGCGGGTGAGAGGCTTTCGTCTCTCGTCTCGCGAGCGCTCGTCAAA

GGTCTTGTTAACTTTGTTTTAGTGTCTTTCGAGCAATCATGTAATAGTTAAAACTTTCAA

CAACGAATCT

>ASV404 GS|0.0|None;No hit

GTGTCAGCAGCCGCGGTAATACGGAGGGAGCTAGCGTTATTCGGAATTACTGGGCGTAAA

GCGCACGTAGGCGGCTTTGTAAGTAAGAGGTGAAAGCCCAGAGCTCAACTCTGGAATTGC

CTTTTAGACTGCATCGCTTGAATCATGGAGAGGTCAGTGGAATTCCGAGTGTAGAGGTGA

AATTCGTAGATATTCGGAAGAACACCAGTGGCGAAGGCGGCTGACTGGACATGTATTGAC

GCTGAGGTGC

>ASV405 SS|0.8700|FJ265754|SH1070903.09FU;k:Fungi,p:Ascomycota,c:Eurotiomycetes,o:Chaetothyriales,f:Trichomeriaceae,g:Knufia

AAGTCGTAACAAGGTTTCCGTAGGTGAACCTGCGGAAGGATCATTACTAAGAGAGGGATG

TACGCTTCCAGCCGAGTCCCGGGGGGCTGCGCCCCTCACCTCTTCAACCCTGTGTCTACC

AACCGCTGTTGCTTCGGCGGACCGGTTGACCAACTGGTCTTGACCGCCGGGGGTCCCGTA

CCCCTGGAGAGCGTCCGCCGACGGCCCAACCACAAACTCTTGTACTAAACCATGTCGTCT

GAATGTCCTT

>ASV406 GS|86.3|AF250779|SH0954634.09FU;k:Fungi,p:Ascomycota,c:Lecanoromycetes,o:Caliciales,f:Caliciaceae,g:Buellia,s:Buellia frigida

AAGTCGTAACAAGATTTTCGTAGATAAATCTACGAAAAAATCATTATCGAGAGACGAGAT

CGCTTCGATCTCACTCTTCACTCGTACCTACTTATCTTTGTTGCTTTGACGAGTCTTCGA

GCTTGATCGTTCGACGCGAGTGAGAGACTTTCGTCTCTCGTCTCGCGAGCGCTCGTCAAA

GGTCTTGTTAACTTTGTTTTAGTGTCTTTCGAGCAATCATGTAATAGTTAAAACTTTCAA

CAACGAATCT

>ASV407 SS|0.8800|MG982528|SH1003748.09FU;k:Fungi,p:Basidiomycota

AAGTCGTAACAAGGTTTCCGTAGGTGAACCTGCGGAAGGATCATTAGTGATTTGGCCCTC

ACGGGTCTATAAAAGACACCTCTGTGAACCTGTCGGCCTCCGGGCCCACCTGCAAACACT

GTGTAACGAGCGTTGATGTATCATAAGCATAATAAAACTTTCAACAACGGATCTCTTGGC

TCTC

>ASV408 GS|87.7|AF250779|SH0954634.09FU;k:Fungi,p:Ascomycota,c:Lecanoromycetes,o:Caliciales,f:Caliciaceae,g:Buellia,s:Buellia frigida

AAGTCGTAACAAGATTTTCGTAAATGAATTTGCGAAAGAATCATTATCGAGAGACGAGAT

CGCTTCGATCTCACTCTTCACTCGTGTCTACTTATCTTTGTTGCTTTGACGAGTCTTCGG

GCTTGATCGTTCGACGCGAGTGGAAGACTTTCGTCTCTCGTCTCGCGAGCGCTCGTCAAA

GATTCTGTTAACTCTGTTTTAGTGTCTTTCGAGCAATCATGTAATAGTTAAAACTTTCAA

CAACGAATCT

>ASV409 GS|85.8|AF250779|SH0954634.09FU;k:Fungi,p:Ascomycota,c:Lecanoromycetes,o:Caliciales,f:Caliciaceae,g:Buellia,s:Buellia frigida

AAGTCGTAACAAGATTTTCGTAGATGAATTTGCGAAAGAATCATTATCGAGAGACGAGAT

CGCTTCGACTTCACTCTTCACTCGTGTCTACTTATCTTTGTTGCTTTGACGAGTCTTCGA

GCTTGATCGTTCGACGCGAGTGAAAGACTTTCGTCTCTCGTCTTGCGAGCGCTCGTCAAA

GACTCTGTTAACTTTGTTTTAGTGTCTTTCGAGCAATCATGTAATAGTTAAAACTTTCAA

CAACGAATTT

>ASV410 SS|0.9500|AY667583|SH0954634.09FU;k:Fungi,p:Ascomycota,c:Lecanoromycetes,o:Caliciales,f:Caliciaceae,g:Buellia,s:Buellia frigida

AAGTCGTAACAAGGTTTCCGTAGATGAACCTGCGAAAGAATCATTATCGAGAGACGAGGT

CGCTTCGGTTCCACTTTTCATCCGTGTCTATTTATCTTTGTTGCTTTGGCGGGTTTTCGA

GCTTGATCGTTCAACGCGGGTGGGAGGCTTTCGTCTCTCGCCTCGCGAGCGCTCGTCAAA

GACTTTGTTAACTCTGTTTTAGTGTCTTTCGAGCAACCATGTAATAGTTAAAACTTTCAA

CAACGGATCT

>ASV411 GS|98.4|AY667583|SH0954634.09FU;k:Fungi,p:Ascomycota,c:Lecanoromycetes,o:Caliciales,f:Caliciaceae,g:Buellia,s:Buellia frigida

AAGTCGTAACAAGGTTTCCGTAGGTGAACCTGCGGAAGGATCATTACCGAGAGACGAGGT

CGCTTCGGCCCCACTCTTCACCCGTGTCTACTTACCTTTGTTGCTTTGACGAGCCTTCGG

GCTTGACCGTTCGACGCGGGTGGGAGACTTTCGCCTCTCGCCTCGCGAGCGCCCGCCAAA

GGCCCTGTTAACTCTGTTTTAGTGTCTTCCGAGCAACCATGTAATAGTTAAAACTTTCAA

CAACGGATCT

>ASV412 GS|99.6|FJ392865|SH1071027.09FU;k:Fungi,p:Ascomycota,c:Eurotiomycetes,o:Chaetothyriales,f:Trichomeriaceae,g:Knufia

AAGTCGTAACAAGGTTTCCGTAGGTGAACCTGCGGAAGGATCATTACCGAGTTAGGGTCT

CTTCGGAGCCCGAACCTCCCAACCCTTTGTCTAATTTACCTTGTCGTTGCTTCGGCGGAC

CGGTTGACCAACTGGTCTTGACCGCCGGGGGTCCCGTACCCCTGGAGAGCGTCTGCCGAC

GGCCCAACCACAAACTCTTGTACTAAACCATGTCGTCTGAATGTCCTTGATATTAATCAA

AAAACAAAAC

>ASV413 GS|99.0|AF250779|SH0954634.09FU;k:Fungi,p:Ascomycota,c:Lecanoromycetes,o:Caliciales,f:Caliciaceae,g:Buellia,s:Buellia frigida

AAGTCGTAACAAGGTTTCCGTAGGTGAACCTGCGAAAGAATCATTACCGAGAGACGGGGT

CGCTTCGGCCCCACACTCTTCACCCGTGTCTACTTACCTTTGTTGCTTTGGCGGGCCTTC

GGGCTTGACCGTTCGACGCGGGTGGGAGGCTTTCGCCTCTCGCCTCGCGAGCGCCCGCCA

AAGGCCCTGTTAACTCTGTTTTAGTGTCTTCCGAGCAACCATGTAATAGTTAAAACTTTC

AACAACGGAT

>ASV415 GS|88.7|AF250779|SH0954634.09FU;k:Fungi,p:Ascomycota,c:Lecanoromycetes,o:Caliciales,f:Caliciaceae,g:Buellia,s:Buellia frigida

AAGTCGTAACAAGATTTTCGTAGATGAATCTACGAAAAAATCATTATCGAGAGACGAAGT

CGCTTCGACCCCACTCTTCACTCGTGTCTACTTATCTTTGTTGCTTTGACGAGCCTTCGA

GCTTAATCGTTCGACGCGAGTGAGAGACTTTCGTCTCTCGTCTCGCGAGCGCTCGCCAAA

GACCTTGTTAACTCTATTTTAGTGTCTTTCGAGCAATCATGTAATAGTTAAAACTTTCAA

CAACGAATCT

>ASV416 GS|100.0|UDB05295223|SH0991924.09FU;k:Fungi,p:Ascomycota,c:Dothideomycetes,o:Mycosphaerellales,f:Teratosphaeriaceae,g:Elasticomyces,s:Elasticomyces elasticus

AAGTCGTAACAAGGTCTCCGTAGGTGAACCTGCGGAGGGATCATTACCGAGTGAGGGCCT

CCGGGCCCGACCTCCAACCCCATGTTAACCGACCCTGTTGCCTCGGGGGCGACCCGGACT

CGTGCCGGGGCCCCCGATGGACCTCCAAACACTGCATCTTTGCGTCCGAGTCAATATTTG

AATAAATCAAAACTTTCAACAACGGATCTCTTGGTTCTG

>ASV417 GS|100.0|UDB05295243|SH0991924.09FU;k:Fungi,p:Ascomycota,c:Dothideomycetes,o:Mycosphaerellales,f:Teratosphaeriaceae,g:Elasticomyces,s:Elasticomyces elasticus

AAGTCGTAACAAGGTCTCCGTAGGTGAACCTGCGGAGGGATCATTACCGAGTGAGGGCCT

CCGGGCCCGACCTCCAACCCCATGTTAACTGACCCTGTTGCCTCGGGGGCGACCCGGACT

CGTGCCGGGGCCCCCGATGGACCTTCAAACACTGCATCTTTGCGTCCGAGTCAATATTTG

AATAAATCAAAACTTTCAACAACGGATCTCTTGGTTCTG

>ASV418 SS|1.0000|AF250779|SH0954634.09FU;k:Fungi,p:Ascomycota,c:Lecanoromycetes,o:Caliciales,f:Caliciaceae,g:Buellia,s:Buellia frigida

AAGTCGTAACAAGGTTTTCGTAGATGAACCTGCGGAAGGATCATTATCGAGAGACGGGGT

CGCTTCGGCCCCACTCTTCACCCGTGTCTACTTATCTTTGTTGCTTTGGCGGGCCTTCGG

GCCTGACCGTTCGACGCGGGTGGGAGGCTTTCGCCTCTCGCCTCGCGAGCGCTCGCCAAA

GACCCTGTTAACTCTGTTTTAGTGTCTTCCGAGCAATCATGTAATAGTTAAAACTTTCAA

CAATGGATCT

>ASV419 GS|98.5|AF250779|SH0954634.09FU;k:Fungi,p:Ascomycota,c:Lecanoromycetes,o:Caliciales,f:Caliciaceae,g:Buellia,s:Buellia frigida

AAGTCGTAACAAGGTTTCCGTAGGTGAACCTGCGAAAGAATCATTACCGAGAGACGGGGT

CGCTTCGGCCCCACACTCTTCACCCGTGTCTACTTACCTTTGTTGCTTTGGCGGGCCTTC

GGGCTTGACCGTTCGACGCGGGTGGGAGGCTTTCGCCTCTCGCCTCGCGAGCGCCCGCCA

AAGGCCCTGTTAACTCTGTTTTAGTGTCTTCCGAGCAACCATGTAATATTTAAAACTTTC

AACAACGGAT

>ASV420 SS|0.9700|AF250779|SH0954634.09FU;k:Fungi,p:Ascomycota,c:Lecanoromycetes,o:Caliciales,f:Caliciaceae,g:Buellia,s:Buellia frigida

AAGTCGTAACAAGGTTTTCGTAGGTGAACCTACGAAAGGATCATTATCGAGAGACGAGGT

CGCTTCGGCCCCACACTCTTCACCCGTGTCTACTTACCTTTGTTGCTTTGGTGGGCCTTC

GGGCTTGATCGTTCGACGCGGGTGGGAGACTTTCGCCTCTCGCCTCGCGAGCGCTCGCCA

AAGGCCTTGTTAACTCTGTTTTAGTGTCTTTCGAGCAATCATGTAATATTTAAAACTTTC

AACAACGAAT

>ASV421 GSL|99.5|KF309980|SH0993867.09FU;k:Fungi,p:Ascomycota,c:Dothideomycetes,o:Mycosphaerellales,f:Extremaceae

AAGTCGTAACAAGGTCTCCGTAGGTGAACCTGCGGAGGGATCATTACCGAGTGAGGGCCC

TCGCGCCCGACCTCCAACCCTTTGTCGATCAATATCTGTTGCCTCGGGGGGCGACCCGGC

CGTCCGCGGGCGGGCGTCCCCCAGAGGACCAATCAACTCTGCATCTTTGCGTCGAGTATT

GAATACAAATCAATCAAAACTTTTAACAACGGATCTCTTGGTTCTG

>ASV422 GS|0.0|None;No hit

GTGTCAGCAGCCGCGGTAATACGGGGGGGGCAAGCGTTATTCGAAATGATTGGGCGTAAA

GAGCACGTAGACGGTTTTTATAGTAGACATAGTGGCAATATCTCTGTATTAAACATCTTT

CACATTTCAAACATCTACATCTTTGACATTTCTCATAAATGTTTAGGATGTTAGGATGTC

AGAGAAATGTGAAAGATGTTTACTTCTATATATTTGCATACTTTTGATGGAGTATCTCTT

TGTTCATGTA

>ASV423 GS|0.0|None;No hit

AAGTCGTAACAAGGTTTCCGTAGATGAATCTGCGAAAGAATTATTATCGAGAGATGAAAT

CTAATCGGCCTCACTTTTCATTCGTGTATATTTACTCTTTATTGCTTTGACGGGCGGTCG

GTCTTTACCGCCCGACGTCGGTCGAAAGACTCGCGTCTTTCACTCATCGAACGCCCGTTA

AAGATTTTCAAAACCCGTTCATTTATATTCGAGCGAGTGAAACAAATAATTAAAAACTTT

TAACAACGAA

>ASV424 SS|1.0000|AF250779|SH0954634.09FU;k:Fungi,p:Ascomycota,c:Lecanoromycetes,o:Caliciales,f:Caliciaceae,g:Buellia,s:Buellia frigida

AAGTCGTAACAAGGTTTTCGTAGGTGAATCTACGAAAGAATCATTATCGAGAGATGGGGT

CGCTTCGACCCCACTCTTCACTCGTATCTACTTACCTTTGTTGCTTTGACGAGCCTTCGA

GCATGATCGTTCGACGCGGGTGGGAGGCTTTCGCCTCTCGCCTCGCGAGCGCTCGTCAAA

GGCCCTGTTAACTCTATTTTAGTGTCTTCCGAGCAATCATGTAATAGTTAAAACTTTCAA

CAACGAATCT

>ASV425 GS|87.3|AF250779|SH0954634.09FU;k:Fungi,p:Ascomycota,c:Lecanoromycetes,o:Caliciales,f:Caliciaceae,g:Buellia,s:Buellia frigida

AAGTCGTAACAAGATTTTCGTAGATGAATCTACAAAAGAATCATTATCGAGAGACGAGAT

CGCTTCGACTTCACTCTTCACTCGTGTCTACTTATCTTTGTTGCTTTGACGAGTCTTCGA

GCGCGATCGTTCGACGCGAGTGAGAGACTTTCGTCTCTCGCCTCGCGAGCGCTCGTCAAA

GACCCTGTTAACTCTGTTTTAGTGTTTTTCGAGCAATCATGTAATAGTTAAAACTTTCAA

CAACGAATCT

>ASV426 SS|0.9900|AY667583|SH0954634.09FU;k:Fungi,p:Ascomycota,c:Lecanoromycetes,o:Caliciales,f:Caliciaceae,g:Buellia,s:Buellia frigida

AAGTCGTAACAAGGTTTCCGTAGGTGAACCTGCGAAAGAATCATTATCGAGAGACGAGGT

CGCTTCGACCCCACTCTTCACTCGTGTCTACTTACCTTTGTTGCTTTGACGAGCCTTCGG

GCTTGACCGTTCGACGCGGGTGGGAGGCTTTCGTCTCTCGCCTCGCGAGCGCCCGCCAAA

GGCTTTGTTAACTCTGTTTTAGTGTCTTCCGAGCAACCATGTAATAGTTAAAACTTTCAA

CAACGAATCT

>ASV427 GS|99.5|JN873879|SH1123218.09FU;k:Fungi,p:Ascomycota,c:Lecanoromycetes,o:Lecanorales,f:Lecanoraceae,g:Lecanora,s:Lecanora physciella

AAGTCGTAACAAGGTTTCCGTAGGTGAACCTGCGGAAGGATCATTACCGAGAGCGGGGCT

AACCCCCCAAACTCCGCCGCCGAAAGGGGTACTCTCCACCCTATGTATACATATACCACT

CTCGCTTTGGCGGGCTGAAGGCTCTTGCCCTACGCCCGCCAGTGGCTCAAAAAATTCTGT

TTATCAGTGATGTCCGAGTAAAAACCTAATAGTTTAAAACTTTCAACAACGGATCTCTTG

GTTCTG

>ASV428 GS|77.3|HM537068|SH1323168.09FU;k:Fungi,p:Ascomycota,c:Sordariomycetes,o:Magnaporthales,f:Magnaporthaceae

AAGTCGTAACAAGGTTTCGAGCAACGATGGCCGGTTCCTTCCCTGGCCAGGCCTATTGGA

AGCCTTTGCCACCCCGCAAGGGGTACGCGCCGCGACTGTAAATAATAGCGTGCATTAAAT

AGCAAGTTGGCCCCGGCGGCCGGCGACACTTTCGAACTGCGGGGACACCTTAAAGCCCGG

CTCTACCAACCCAGCGGAGAAATCCAGCTGGGGGCCCATGTTAACCGCATGGGGTATGGT

AACAATGAGC

>ASV429 GS|0.0|None;No hit

GTGTCAGCAGCCGCGGTAATACGAAGGGGGCTAGCGTTGCTCGGAATGACTGGGCGTAAA

GGGCGCGTAGGCGGATTTGTCAGTCGGGCGTGAAATTCCTGGGCTTAACCTGGGGGCTGC

GTTCGAGACGGCGGGTCTTGAGTTTGGAAGAGGGTCGTGGAATTCCCAGTGTAGAGGTGA

AATTCGTAGATATTGGGAAGAACACCGGTGGCGAAGGCGGCGACCTGGTCCTGGACTGAC

GCTGAGGCGC

>ASV430 GS|98.5|UDB03337334|SH0989105.09FU;k:Fungi,p:Ascomycota,c:Leotiomycetes,o:Helotiales

AAGTCGTAACAAGGTTTCCGTAGGTGAACCTGCGGAAGGATCATTACCGAGTTTGTGCCC

TCTGGGGTAGATCTCCCACCCTGTTTATTTTACTTCTTGTTGCTTTGGCGGGTCGACTGT

CCAGGCCACCGGCGACTCGCTGGTGTGTGCCCGCCAAAGGCTCCTAAACCCGGATATGTA

ACCTGCCGTCCGAGTCCTATATAATACTTAAAACTTTCAACAACGGATCTCTTGGTTCTG

>ASV431 GS|100.0|FR682222|SH0966251.09FU;k:Fungi,p:Basidiomycota,c:Tremellomycetes,o:Holtermanniales,f:Holtermanniaceae,g:Holtermanniella

AAGTCGTAACAAGGTTTCCGTAGGTGAACCTGCGGAAGGATCATTAGTGAATTGAGCTGG

CTTCGGCCGCTCTGATCTATACACACCTGTGAACTGTTGGCCTTCGGGTCTTTAACTACA

ACTGTGACGAACGTAAATTATTATAATCAATAAAACTTTTAACAACGGATCTCTTGGCTC

TC

>ASV432 GS|97.2|UDB01571642|SH1041881.09FU;k:Fungi,p:Basidiomycota,c:Tremellomycetes,o:Tremellales,f:Bulleraceae

AAGTCGTAACAAGGTTTCCGTAGGTGAACCTGCGGAAGGATCATTAATGTAAACCCCTTG

TGGGGAAATACAAGTCCACATACCTCTGTGAACCGTTGACCTCCGGGTCGTCTTCACAAA

CATCAGTGTAACGAACGTATACAAACATAAACAAAACAAAACTTTCAACAACGGATCTCT

TGGCTCTC

>ASV433 GS|86.0|AY667583|SH0954634.09FU;k:Fungi,p:Ascomycota,c:Lecanoromycetes,o:Caliciales,f:Caliciaceae,g:Buellia,s:Buellia frigida

AAGTCGTAACAAGATTTTCGTAGATGAACTTGCGAAAGAATCATTATCGAGAGACGAGAT

CGCTTCGATCTCACTCTTCACTCGTGTTTACTTATCTTTGTTGCTTTGACGAGCCTTCGA

GCTTGATCGTTCGACGCGAGTGGGAGACTTTCGTCTCTCGTCTCGCGAGCGTTCGTCAAA

GATCTTGTTAACTCTATTTTAGTGTCTTTCGAGCAATCATGTAATAGTTAAAACTTTCAA

CAATGAATTT

>ASV434 SS|1.0000|UDB0747173|SH1194722.09FU;k:Fungi

AAGTCGTAACAAGGTTTCCGTAGGTGAACCTGCGGAAGGATCATTAAAAGAGAAAACGGG

AGGCCCCTCTCAGGGGGTCGCCCTCATCTCCCAAACCCCCCGTCTACCGACCTCACAAAA

CCCAAGTACAAGTAAACAAGTCGTCCCTAGCGGGGCGCACTACCTTAAATGTACAGAAAT

CAAAACTTTTAACAACGGATCTCTTGGTTCTG

>ASV435 SS|0.8600|MK116454|SH1304331.09FU;k:Fungi,p:Ascomycota,c:Eurotiomycetes,o:Chaetothyriales,f:Herpotrichiellaceae

AAGTCGTAACAAGGTTTCCGTAGGTATGTAAACGATCAAACCTCTCACCCCCCGAAACGC

GACACATGCTAACTACTTTTATAGGTGAACCTGCGGAAGGATCATTAAAGAGTTAGGGTT

CCCTTCGGGGGCCCGACCTCCCAACCCTCTGTTTATTATACCTTGTGTTGCTTCGGTAGG

CCTGGATAGCTCCTGCCGGGGGGCCCTGTCGAGAGACACCCCTCTGGAGAGCGCTTACCG

ATAGCCTCCA

>ASV436 GS|99.6|AY667583|SH0954634.09FU;k:Fungi,p:Ascomycota,c:Lecanoromycetes,o:Caliciales,f:Caliciaceae,g:Buellia,s:Buellia frigida

AAGTCGTAACAAGGTTTCCGTAGGTGAACCTGCGGAAGGATCATTATCGAGAGACGGGGT

CGCTTCGGCCCCACTCTTCACCCGTGTCTACTTACCTTTGTTGCTTTGGCGGGCCTTCGG

GCTTGACCGTTCGACGCGGGTGGGAGGCTTTCGCCTCTCGCCTCGCGAGCGCCCGCCAAA

GGCCCTGTTAACTCTGTTTTAGTGTCTTCCGAGCAACCATGTAATAGTTAAAACTTTCAA

CAACGGATCT

>ASV437 GS|86.8|AF250779|SH0954634.09FU;k:Fungi,p:Ascomycota,c:Lecanoromycetes,o:Caliciales,f:Caliciaceae,g:Buellia,s:Buellia frigida

AAGTCGTAACAAGATTTTCGTAGATGAATTTGCGAAAGAATCATTATCGAGAGACGAGGT

CGCTTCGACTTCACTCTTCATTCGTGTCTACTTATCTTTGTTGCTTTGACGAGTCTTCGA

GCTTGATCGTTCGACGCGAGTGAGAGACTTTCGTCTCTCGTCTCGCGAGCGCTCGTCAAA

GACTTTGTTAACTCTGTTTTAGTGTCTTTCGAGCAATCATGTAATAGTTAAAACTTTCAA

TAACGAATCT

>ASV438 GS|99.6|HM161510|SH0998157.09FU;k:Fungi,p:Ascomycota,c:Lecanoromycetes,o:Umbilicariales,f:Umbilicariaceae,g:Umbilicaria,s:Umbilicaria decussata

AAGTCGTAACAAGGTTTCCGTAGGTGAACCTGCGGAAGGATCATTACTGAGATAGGGTCC

TCCGGGCCCGAACCTCCAACCCTTTGCGTACCTTACCTTTGTTGCTTTGGCGGGCCCGCT

GGGGAAACCCACCGCCGGCGTCGAGCCGGTGAGCGCCCGCCGGAGGCCCTCAAAACTCCG

TCTTGTCAGTGTCGTCTGAGTACTATACAATAGCTAAAACTTTCAACAACGGATCTCTTG

GTTCTG

>ASV439 GS|99.5|UDB06703551|SH0954586.09FU;k:Fungi,p:Ascomycota,c:Lecanoromycetes,o:Lecanorales,f:Scoliciosporaceae

AAGTCGTAACAAGGTTTCCGTAGGTGAACCTGCGGAAGGATCATTAATGAGAAACGGCCT

CGCAGCCGTGGGGGTTTCGGTCCCCAACTCAACCCTTTGCGTATATACCTTTGTTGCTTT

GGCGAGCTTTGAGCCTTCCAGCTCTGATCCCCTGCCGGTGCACCGGTGGGTGTTCGCCAG

AGGCCCTACATCTTCTGTTGTATTATTATCGTCGGAGCGAAAATCAAAACAATCAAAACT

TTCAACAACG

>ASV440 GS|100.0|UDB01571340|SH0954610.09FU;k:Fungi,p:Ascomycota,c:Lecanoromycetes,o:Caliciales,f:Caliciaceae,g:Buellia

AAGTCGTAACAAGGTTTCCGTAGGTGAACCTGCGGAAGGATCATTACCGAGAGACGGGGT

CGCTTCGGCCCCACTCTTCACCCGTGTCTATTTACCTTTGTTGCTTTGGCGGGCCTTCGG

GCTTGATCGTTTGGCGCTGGTTGGGGGGCGTTGGCCTCTCGCCCAGCGAGTGCCTGCCAA

AGACCTTGTTAACTCCGTTCTTAATGTCCTCTGAGCCACCATATAATAGTTAAAACTTTC

AACAACGGAT

>ASV441 GS|98.8|AY667583|SH0954634.09FU;k:Fungi,p:Ascomycota,c:Lecanoromycetes,o:Caliciales,f:Caliciaceae,g:Buellia,s:Buellia frigida

AAGTCGTAACAAGGTTTCCGTAGGTGAACCTGCGGAAGGATCATTACCGAGAGACGGGGT

CGCTTCGGCCCCACTCTTCACCCGTGTCTACTTACCTTTGTTGCTTTGGCGGGCCTTCGG

GCTTGATCGTTCGACGCGGGTGGGAGGCTTTCGCCTCTCGCCTCGCGAGCGCTCGCCAAA

GGCCTTGTTAACTCTGTTTTAGTGTCTTCCGAGCAACCATGTAATAGTTAAAACTTTCAA

CAACGGATCT

>ASV442 GS|79.8|MN103133|SH0913114.09FU;k:Fungi,p:Ascomycota,c:Lecanoromycetes,o:Caliciales,f:Caliciaceae,g:Dimelaena,s:Dimelaena oreina

AAGTCGTAACAAGGTTTTCGTAGGTGAATTTGCGGAAGGATCATTATCGAGAGACGGAGT

CTAACCGGCCCCACTCTTCACTCGTGTATACCTACCCTTTGTTGCTTTGGCGGGCGGTCG

GTCTTTACCGCCCGACGTCGGTCGGAAGGCTCGCGCCTTCCACTCACCGAACGCCCGCCA

GAGGTCTCCAAAACCCGTCCATTTGTGTTCGAGCGAGTGAAGCAAATAATTAAAAACTTT

CAATAACGGA

>ASV443 GS|97.2|AY667583|SH0954634.09FU;k:Fungi,p:Ascomycota,c:Lecanoromycetes,o:Caliciales,f:Caliciaceae,g:Buellia,s:Buellia frigida

AAGTCGTAACAAGGTTTCCGTAGGTGAACCTGCGGAAGGATCATTATCGAGAGACGGGGT

CGCTTCGGCCCCACTCTTCACTCGTGTCTACTTACCTTTGTTGCTTTGGCGGGCCTTCGG

GCTTGACCGTTCGACGCGGGTGGGAGGCTTTCGTCTCTCGCCTCGCGAGCGCCCGCCAAA

GGCTTTGTTAACTTTGTTTTAGTGTCTTCCGAGCAATCATGTAATAGTTAAAACTTTCAA

CAACGGATCT

>ASV444 GSL|100.0|OK576236|SH1309305.09FU;k:Fungi,p:Ascomycota,c:Dothideomycetes,o:Capnodiales,f:Cladosporiaceae,g:Cladosporium

AAGTCGTAACAAGGTCTCCGTAGGTGAACCTGCGGAGGGATCATTACAAGTGACCCCGGT

CTTACCACCGGGATGTTCATAACCCTTTGTTGTCCGACTCTGTTGCCTCCGGGGCGACCC

TGCCTTCGGGCGGGGGCTCCGGGTGGACACTTCAAACTCTTGCGTAACTTTGCAGTCTGA

GTAAACTTAATTAATAAATTAAAACTTTTAACAACGGATCTCTTGGTTCTG

>ASV445 GS|99.2|AY667583|SH0954634.09FU;k:Fungi,p:Ascomycota,c:Lecanoromycetes,o:Caliciales,f:Caliciaceae,g:Buellia,s:Buellia frigida

AAGTCGTAACAAGGTTTCCGTAGGTGAACCTGCGGAAGGATCATTACCGAGAGACGGGGT

CGCTTCGGCCCCACTCTTCACCCGTGTCTACTTACCTGTGTTGCTTTGGCGGGCCCTCGG

GCTTGACCGTTCGACGCGGGTGGGAGGCTTTCGCCTCTCGCCTCGCGAGCGCCCGCCAAA

GGCCCTGTTAACTCTGTTTTAGTGTCTTCCGAGCAACCATGTAATAGTTAAAACTTTCAA

CAACGGATCT

>ASV446 GS|86.4|AY667583|SH0954634.09FU;k:Fungi,p:Ascomycota,c:Lecanoromycetes,o:Caliciales,f:Caliciaceae,g:Buellia,s:Buellia frigida

AAGTCGTAACAAGATTTTCGTAGATGAACTTGCGAAAGAATCATTATCGAGAGACGAGAT

CGCTTCGATCTCACTTTTCATTCGTGTCTACTTATCTTTGTTGCTTTGACGAGCCTTCGA

GCTTGATCGTTCGACGCGGGTAGAAGGCTTTCGCCTCTCGTCTCGCGAGCGCTCGTCAAA

GATTCTGTTAACTTTGTTTTAGTGTCTTTCGAGCAATCATGTAATAATTAAAACTTTCAA

CAATGAATCT

>ASV447 GS|86.8|AF250779|SH0954634.09FU;k:Fungi,p:Ascomycota,c:Lecanoromycetes,o:Caliciales,f:Caliciaceae,g:Buellia,s:Buellia frigida

AAGTCGTAACAAGATTTTCGTAGATGAATTTGCGAAAGAATCATTATCGAGAGACGAGGT

CGCTTCGATTTCACTCTTCACTCGTGTCTACTTATCTTTGTTGCTTTGGCGAGTTTTCGA

GCTTGATCGTTCGACGCGAGTGAGAGACTTTCGTCTCTCGTCTCGCGAGCGCTCGTCAAA

GACTTTGTTAACTTTGTTTTAGTGTCTTTCGAGCAATCATGTAATAGTTAAAACTTTCAA

CAACGAATCT

>ASV448 GS|81.2|AY667583|SH0954634.09FU;k:Fungi,p:Ascomycota,c:Lecanoromycetes,o:Caliciales,f:Caliciaceae,g:Buellia,s:Buellia frigida

AAGTCGTAACAAGATTTTCGTAGATGAATCTACGAAAGAATTATTATCGAGAGACGAAAT

CGTTTCGATCTCACTCTTCACTCGTATCTACTTATCTTTGTTGCTTTGACGAGTCTTCGA

GCTTGATCGTTCGACGCGAGTGAGAGACTTTCATCTCTCGTTTCGCGAGCGTTCATCAAA

GATCTTGTTAACTTTGTTTTAATGTCTTTCGAACAATCATGTAATAATTAAAACTTTCAA

TAACGAATTT

>ASV449 SS|0.9200|MZ229889|SH0913114.09FU;k:Fungi,p:Ascomycota

AAGTCGTAACAAGGTTTCCGTAGGTGAACCTGCGGAAGGATCATTATCGAGAGACGGAGT

CTAACCGGCCCCACTCTTCATTCGTGTATACCTACCCTTTGTTGCTTTGGCGGGCGGTCG

GTCTTTACCGCCCGACGTCGGTCGGAAGGCTCGCGCCTTCCACTCACCGAACGCCCGCCA

GAGGCCCCCAAAACCCGTTCATCTGTGTCCGAGCGAGTGAAGCAAATAGTTAAAAACTTT

CAACAACGGA

>ASV450 GS|99.2|AY667583|SH0954634.09FU;k:Fungi,p:Ascomycota,c:Lecanoromycetes,o:Caliciales,f:Caliciaceae,g:Buellia,s:Buellia frigida

AAGTCGTAACAAGGTTTCCGTAGGTGAACCTGCGGAAGGATCATTACCGAGAGACGGGGT

CGCTTCGGCCCCACTCTTCACCCGTGTCTACTTACCTTTGTTGCTTTGGCGGGCCTTCGG

GCTTGACCGTTCGACGCGGGTGGGAGGCTTTCGCCTCTCGCCTCGCGAGCGCCCGCCAAA

GGTCCTGTTACCTCTGTTTTAGTGTCTTCCGAGCAACCATGTAATAGTTAAAACTTTCAA

CAACGGATCT

>ASV451 GS|99.0|MK208763|SH0942716.09FU;k:Fungi,p:Ascomycota,c:Lecanoromycetes,o:Lecanorales,f:Lecanoraceae,g:Lecidella

AAGTCGTAACAAGGTTTCCGTAGGTGAACCTGCGGAAGGATCATTAATGAGAGAGGGGCT

TCGCGCTCCCGGGGGCTCCGGCCCCCAACTCTTCACCCTCTGTTTACCTACCTTTGTTGC

TTTGGCGCGCCCTGGGGTTCCGCCCCACGCCGGCCCCAGGCCTTCGGCTTGGGCTGGTGA

GAGCCCGTCAGAGGCCCATTCCAACCCTTATATCAGTGATGTCCGAGTACAATCTTAATA

GATAAAACTT

>ASV452 GS|97.1|KY266860|SH0954363.09FU;k:Fungi,p:Ascomycota,c:Lecanoromycetes,o:Acarosporales,f:Acarosporaceae,g:Pleopsidium,s:Pleopsidium chlorophanum

AAGTCGTAACAAGGTTTCCGTAGGTGAACCTGCGGAAGGATCATTACAGAGTTAGGGTCT

TCCGGGCCCGATCTCCAACCCTATGTCTACCTACCTTTGTTCTTTGGCGGGCCCGCTGGG

GGAGACCCACCGGTGGCTCCGGCTGCCGAGCGCCCGTCAGAGACCCATCGAACCCTGTTA

ATCATGTAGTCTGAGTACCAATACAATAGTTAAAACTTTCAACAACGGATCTCTTGGTTC

TG

>ASV453 GSL|99.5|KF309980|SH0993867.09FU;k:Fungi,p:Ascomycota,c:Dothideomycetes,o:Mycosphaerellales,f:Extremaceae

AAGTCGTAACAAGGTCTCCGTAGGTGAACCTGCGGAGGGATCATTACCGAGTGAGGGCCC

TCGCGCCCGACCTCCAACCCTTTGTCGATCAATATCTGTTGCCTCGGGGGGCGACCCGGC

CGTCCGCGGGCGGGGGTCCCCCAGAGGACCAATCAACTCTGCATCTTTGCGTCGAGTATT

GAATACAAATCAAGCAAAACTTTTAACAACGGATCTCTTGGTTCTG

>ASV454 SS|0.9900|KF823589|SH1091255.09FU;k:Fungi

AAGTCGTAACAAGGTTTCCGTAGGTGAACCTGCGGAAGGATCATTAGGGATTTGGTCTCC

GGACCTTTTTCATATCCATAATACCCCTGTGAACCTGTCGGTCCTCGGGCCAGCGTTTCC

AAACCATGTGTAATGAACGTGAATGTGTATGAAACCCTAGTAAAACTTTCAACAACGGAT

CTCTTGGTTCTG

>ASV455 GS|99.0|UDB03337332|SH0989105.09FU;k:Fungi,p:Ascomycota,c:Leotiomycetes,o:Helotiales

AAGTCGTAACAAGGTTTCCGTAGGTGAACCTGCGGAAGGATCATTACCGAGTTTGTGCCC

TCTGGGGTAGATCTCCCACCCTGTTTATTTTACTTCATGTTGCTTTGGCGGGTCGACTGT

CCAGGCCACCGGCGACTCGCTGGTGTGTGCCCGCCAAAGGCCCCCAAACCCGGATATGTA

ACCTGCCGTCCGAGTCCTATATAATATTTAAAACTTTCAACAACGGATCTCTTGGTTCTG

>ASV456 SS|1.0000|KM504479|SH1164905.09FU;k:Fungi,p:Basidiomycota,c:Agaricostilbomycetes,o:Agaricostilbales,f:Chionosphaeraceae,g:Kurtzmanomyces

AAGTCGTAACAAGGTTTTCGTAGGTGAACCTGCGGAAGGATCATTAGTGAATTGCTTTTG

GGAGTCCCTCTTTACCGGAGAAGGCCCATCTCTTCATTACACCCACACACTTGTGCATTT

GTCCCCTTTTCTTCAATGAAAAATTACCTTTTTGACCCTTTATGCATTTGAAACGGTTGT

TGTTGCATATCAAAATGATAAACGAAAGAAAAACTTTCAGCAATGGATCTCTTGGCTCTC

>ASV457 GS|97.2|AY667583|SH0954634.09FU;k:Fungi,p:Ascomycota,c:Lecanoromycetes,o:Caliciales,f:Caliciaceae,g:Buellia,s:Buellia frigida

AAGTCGTAACAAGGTTTCCGTAGGTGAATCTGCGGAAGGATCATTATCGAGAGACGAGGT

CGCTTCGGCCCCACTCTTCACCCGTGTCTACTTATCTTTGTTGCTTTGGCGGGCCTTCGG

GCTTGATCGTTCGACGCGGGTGGGAGGCTTTCGCCTCTCGCCTCGCGAGCGCCCGCCAAA

GGCTCTGTTAACTCTGTTTTAGTGTCTTCCGAGCAATCATGTAATAGTTAAAACTTTCAA

CAACGGATCT

>ASV458 SS|0.9900|OK576250|SH1225643.09FU;k:Fungi

AAGTCGTAACAAGGTTTCCGTAGGTGAACCTGCGGAAGGATCATTACCGAGCTAGGGCCT

TCTAGGTCTGACCTCCCACCCTATGTTTATCAGCTCCTTGTTGCTTCGGCGGACCGTTGG

GGTCAAACCCGCCGCAGGCTTTCGGGCTTGTGAGCGTCCGTCGGAGGATACTTTTAACGC

GTTCAATTCATGTGGTCTGAGTGGGTATTTAATCACCTTAAAACTTTCAACAACGGATCT

CTTGGTTCTG

>ASV459 GS|98.1|AY843176|SH0964452.09FU;k:Fungi,p:Ascomycota

AAGTCGTAACAAGGTTTCCGTAGGTGAACCTGCGGAAGGATCATTACCGAGATAGGGTTT

CTTCGGAGCCCGACCTCCAACCCATTGTCTACCATACCTCTGTTGCCTCGGCGGGCCGCC

GGCGCCTTTACGGGCGTCGCCGTTGGTCTTTACGGGCTTTCGAGCGCCCGCCGCAGGACT

TATAAACTCTTTTTTAAAGTAGTATTCTGAGTGGGAATTTAATAACTTCAAAACTTTCAA

CAACGGATCT

>ASV460 SS|0.8100|JX043001|SH0956956.09FU;k:Fungi,p:Ascomycota,c:Lecanoromycetes,o:Lecanorales,f:Lecanoraceae

AAGTCGTAACAAGGTTTCCGTAGGTGAACCTGCGGAAGGATCATTACTGAGAGGGGTTCT

CGGACCCCGGGGGCCCCGGCCCCCAACTCATCACCCTGTGCATACCTACCTTTGTTCCTT

TGGCGGGCCTTGGAGCCTGCTCCATGGTGGCTTCGGGCTTCACTGTCCGGCCGTCCCGCG

CCCGTCAGAGACCTATCCAACCTCTTTTATCCGTATCGTCCGAGTCAAACCACAATTGTA

AAAAACTTTC

>ASV461 GS|84.8|AF250779|SH0954634.09FU;k:Fungi,p:Ascomycota,c:Lecanoromycetes,o:Caliciales,f:Caliciaceae,g:Buellia,s:Buellia frigida

AAGTCGTAACAAGATTTTCGTAAATGAATTTGCGAAAGAATCATTATCGAGAGACGAAAT

CGCTTCGATTTCACTCTTCACTCGTGTCTACTTATCTTTGTTGCTTTGACGAATCTTCGA

GCTTAATCGTTCGATGCGAGTGAGAGGCTTTCGTCTCTCGTCTCGCGAGCGCTCGTCAAA

GACTTTGTTAACTTTGTTTTAGTGTCTTTCGAGCAATCATGTAATAGTTAAAACTTTCAA

CAACGAATCT

>ASV462 GS|90.2|AF250779|SH0954634.09FU;k:Fungi,p:Ascomycota,c:Lecanoromycetes,o:Caliciales,f:Caliciaceae,g:Buellia,s:Buellia frigida

AAGTCGTAACAAGGTTTTCGTAGATGAACCTACGAAAGAATCATTATCGAGAGACGAGAT

CGCTTCGACCCCACTCTTCACCCGTGTCTACTTATCTTTGTTGCTTTGACGAGCCTTCGA

ACTTGATCGTTCGACGCGAGTGGGAGGCTTTCGTCTCTCGCCTCGCGAGCGCTCGCCAAA

GACTTTGTTAACTCTGTTTTAGTGTCTTTCGAGCAATTATGTAATAGTTAAAACTTTCAA

CAACGAATCT

>ASV463 SS|1.0000|AF250779|SH0954634.09FU;k:Fungi,p:Ascomycota,c:Lecanoromycetes,o:Caliciales,f:Caliciaceae,g:Buellia,s:Buellia frigida

AAGTCGTAACAAGGTTTGCGTAGATGAATCTGCGAAAGGATCATTATCGAGAGACGAGGT

CGCTTCGGCCCCACTCTTCACTCGTGTCTACTTACCTTTGTTGCTTTGACGAGCCTTCGA

GCTTGATCGTTCGACGCGGGTGGGAGGCTTTCGCCTGTCGCCTCGCGAGCGCCCGCCAAA

GGCCCTGTTAACTCTGTTTTAGTGTCTTCCGAGCAACCATGTAATAGTTAAAACTTTCAA

CAACGGATCT

>ASV464 SS|1.0000|AF250779|SH0954634.09FU;k:Fungi,p:Ascomycota,c:Lecanoromycetes,o:Caliciales,f:Caliciaceae,g:Buellia,s:Buellia frigida

AAGTCGTAACAAGGTTTTCGTAGGTGAATCTGCGAAAGGATCATTATCGAGAGACGAGGT

CGCTTCGGCCTCACTCTTCACTCGTGTCTACTTATCTTTGTTGCTTTGGCGGGCCTTCGG

GCTTAATCGTTCGACGCGGGTGGGAGGCTTTCGCCTCTCGCCTCGCGAGCGCCCGCCAAA

GGCCCTGTTAACTCTGTTTTAGTGTCTTCCGAGCAACCATGTAATAGTTAAAACTTTCAA

CAACGGATCT

>ASV465 GS|98.6|AF279772|SH1300522.09FU;k:Fungi,p:Ascomycota,c:Lecanoromycetes,o:Teloschistales,f:Teloschistaceae,g:Xanthoria,s:Xanthoria elegans

CTTGTTCCTTTCTCTTCCTTCCCCTTCTTCCCCCTTTTTCCGTAGGTGAACCTGCGGAAG

GATCATTACTCCGAGAGGGATGTACGCTTCCAGCCGAGTCCCGGGGGGCTGCGCCCCTCA

CCTCTTCCACCCTGTGTCTACCAACCGCTGTTGCTTCGGCGAGCGTCGGGGCGTCCGCGC

CCCGGCCCCGGCTTCGGTCGGTGAGCTCTCGCAGAGGCCTATCTTTATTCTGTTTTGCAG

TGACGTCCGA

>ASV466 SS|0.9500|MF138063|SH0954212.09FU;k:Fungi,p:Ascomycota,c:Lecanoromycetes,o:Acarosporales,f:Acarosporaceae,g:Acarospora

AAGTCGTAACAAGGTTTCCGTAGGTGAACCTGCGGAAGGATCATTACCGAGATAGGGTTT

CTTCGGAGCCCGACCTCCAACCCTATGTGTACCTACCTTTGTTGCTTTGGCGGGCCCGTT

GGGTGACCCACCGGTGGCCTCTGGCTCCCGAGTGCCCGTCAGAGACCCATCAAAACCCGT

TAATTGTGTCGTCTGAGTACCACTTTAATAATTAAAACTTTCAACAACGGATCTCTTGGT

TCTG

>ASV467 SS|0.9200|MZ224659|SH0916480.09FU;k:Fungi,p:Ascomycota,c:Lecanoromycetes

AAGTCGTAACAAGGTTTCCGTAGGTGAACCTGCGAAAGGATCATTATCGAGAGACGGAGT

CTAACCGGCCCCACTCTTCACCCGTGTATACCTACCCTTTGTTGCTTTGGCGGGCGGTCG

GTCTTTACCGCCCGACGTCGGTCGGAAGGCTCGCGCCTTCCACTCACCGAACGCCCGCCA

AAGACCTTCAAAAGCCGTCCATCTGTGTCCGAGCGAGTGAAGTAAATAGTTAAAAACTTT

CAACAACGGA

>ASV468 SS|0.8000|UDB04294373|SH1084495.09FU;k:Fungi,p:Ascomycota,c:Dothideomycetes

AAGTCGTAACAAGGTCTCCGTAGGTGAACCTGCGGAGGGATCATTACCGAGTGAGGGCCT

CCGGGCTCGACCTCCAACCCTCTGTTGTACCAACCACAGTTGCCTCGGGGGCGACCCGGC

CTCCGCGTCGGGGCCCCCGGTGGACCCAACCAAACAACTCTGCGTCTTTGCGTCTGAGTA

TTAAAGTAAATCAATTAAAACTTTCAACAACGGATCTCTTGGTTCTG

>ASV469 GS|86.0|AY667583|SH0954634.09FU;k:Fungi,p:Ascomycota,c:Lecanoromycetes,o:Caliciales,f:Caliciaceae,g:Buellia,s:Buellia frigida

AAGTCGTAACAAGATTTTCGTAAGTGAACTTGCGAAAGAATCATTATCGAGAGACGAGAT

CGCTTCGATCTCACTCTTCACTCGTGTCTACTTATCTTTGTTGCTTTGACGAGTCTTCGA

GCTTGATCGTTTGATGCGAGTGAAAGACTTTCGCCTCTCGTCTCGCGAGCGCTCGTCAAA

GATCTTGTTAACTTTGTTTTAGTGTCTTTCGAGCAATCATGTAATAGTTAAAACTTTCAA

CAACGAATCT

>ASV470 GS|0.0|None;No hit

AAGTCGTAACAAGGTTAACTGATCCTTTCGTACATCTTAAGGAAGCCTTAGCAGCCGATA

CTGGTGCAGTCTAGACGACTTTTACTTAAATCTTAACTAGACTCTTGAATGCTAGTTGAT

CCTAGAGATCAGCGATACTACCAAATTGCGGGGAACTCTTAAAGCTTTTACTACCAAACG

TCTCAGAGGAAACTCGGGCGTGGCCAGGTTAATGACCTTGGGTACGGTAATAATGTAGAA

GATTTACATT

>ASV471 GS|89.2|AF250779|SH0954634.09FU;k:Fungi,p:Ascomycota,c:Lecanoromycetes,o:Caliciales,f:Caliciaceae,g:Buellia,s:Buellia frigida

AAGTCGTAACAAGATTTTCGTAGATGAATCTACGAAAGAATCATTATCGAGAGACGAGGT

CGCTTCGACCCCATTCTTCACTCGTGTCTACTTACCTTTGTTGCTTTGGCGAGTCTTCGA

ACTTGATCGTTCGACGCGAGTGAGAGACTTTCGTCTCTCGCCTCGCGAGCGCTCGTCAAA

GGCCTTGTTAACTTTGTTTTAATGTCTTTCGAGCAATCATGTAATAGTTAAAACTTTCAA

CAACGAATCT

>ASV472 GS|99.5|UDB03302459|SH0905560.09FU;k:Fungi,p:Ascomycota,c:Leotiomycetes,o:Helotiales,f:Helotiaceae

AAGTCGTAACAAGGTTTCCGTAGGTGAACCTGCGGAAGGATCATTACAGTGTTCCCTGCC

CTCACGGGTAGAAACGCCCACCCTTTGTATATTATATCTTTGTTGCTTTGGCAGGCCGCC

CTCGGGCGCTGGCTCCGGCTGGATCGCGCCTGCCAGAGGAAACCCAAACTCTGAATGTTA

GTGTCGTCCGAGTACTATCTAATAGTTAAAACTTTCAACAACGGATCTCTTGGTTCTG

>ASV473 SS|0.9600|MZ229889|SH0913114.09FU;k:Fungi,p:Ascomycota,c:Lecanoromycetes

AAGTCGTAACAAGGTTTCCGTAGGTGAACCTGCGGAAGAATCATTATCGAGAGACGGAGT

CTAACCGGCCCCACTCTTCACCCGTGTATACCTACCCTTTGTTGCTTTGGCGGGCGGTCG

GTCTTTACCGTCCGACGTCGGTCGGAAGGCTCGCGCCTTCCACTCACCGAACGCCCGTCA

GAGGCTCTCAAAAGCCGTCCATCTGTGTCCGAGCGAGTGAAGCAAATAGTTAAAAACTTT

CAACAACGGA

>ASV474 SS|1.0000|KU164637|SH1223443.09FU;k:Fungi

AAGTCGTAACAAGGTTTCCGTAGGTGAACCTGCGGAAGGATCATTAATGAAATGAAAGGA

TGCTCTTTTTAGAGGTCCGACCCATTACATTTCCAACACTGTGCACAAACACATTTTTAC

ACCCCTTTTTAACGCATTAGTTATAAGAATGTATAAAGTCTCTTAATTGAGCATAAAATA

AGCAAAACTTTCAGCAACGGATCTCTTGGCTCTC

>ASV475 GS|99.5|AF276067|SH0954634.09FU;k:Fungi,p:Ascomycota,c:Lecanoromycetes,o:Caliciales,f:Caliciaceae,g:Buellia,s:Buellia frigida

CTTTTTCCTTTCTCTTCCTTCCCCGTCGTCCCCCGGTTTCCGTAGGTGAACCTGCGGAAG

GATCATTACCGAGAGCCGGGGTCGCTTCGGCCCCACTCTTCACCCGTGTCTACTTACCTT

TGTTGCTTTGGCGGGCCTTCGGGCTTGACCGTTCGACGCGGGTGGGAGGCTTTCGCCTCT

CGCCTCGCGAGCGCCCGCCAAAGGCCCTGTTAACTCTGTTTTAGTGTCTTCCGAGCAACC

ATGTAATAGT

>ASV476 SS|0.8500|MK539964|SH0985721.09FU;k:Fungi,p:Ascomycota,c:Dothideomycetes,o:Pleosporales

AAGTCGTAACAAGGTTTCCGTAGGTGAACCTGCGGAAGGATCATTACCGTGGGGATCCGT

CCCCATTGAGATAGCACCCTTTGTTCACGAGTACCCTTGTTTCCTCGGCCGGTTCGCCGG

CCACGAGGACACGTAAAACCCTTTGCAGTAGCAGTCAATTCAGTCGTAAACAAAAAACAT

TAAAACTTTCAACAACGGATCTCTTGGTTCTG

>ASV477 GS|85.3|AF250779|SH0954634.09FU;k:Fungi,p:Ascomycota,c:Lecanoromycetes,o:Caliciales,f:Caliciaceae,g:Buellia,s:Buellia frigida

AAGTCGTAACAAGATTTTCATAAGTGAATCTACGAAAGAATCATTATCGAGAGACGAAAT

CGCTTCGACTTCACTCTTCACTCGTGTCTACTTATCTTTGTTGCTTTAACGAGTCTTCGA

GCTTGATCGTTCGACGCGAGTGAGAGACTTTCGTCTCTCGTCTCGCAAGCGCTCGTCAAA

GATCGTGTTAACTCTATTTTAGTGTCTTTCGAGCAATCATGTAATAGTTAAAACTTTCAA

CAACGAATCT

>ASV478 GS|99.6|AY667583|SH0954634.09FU;k:Fungi,p:Ascomycota,c:Lecanoromycetes,o:Caliciales,f:Caliciaceae,g:Buellia,s:Buellia frigida

AAGTCGTAACAAGGTTTCCGTAGGTGAACCTGCGGAAGGATCATTACCGAGAGACGGGGT

CGCTTCGGCCCCACTCTTCACCCGTGTCTACTTACCTTTGTTGCTTTGGCGGGCCTTCGG

GCTTGACCGTTCGACGCGGGTGGGAGGCTTTCGCCTCTCGCCTCGCGAGCGCCCGCCAAG

GGCCCTGTTAACTCTGTTTTAGTGTCTTCCGAGCAACCATGTAATAGTTAAAACTTTCAA

CAACGGATCT

>ASV479 SS|1.0000|AY667583|SH0954634.09FU;k:Fungi,p:Ascomycota,c:Lecanoromycetes,o:Caliciales,f:Caliciaceae,g:Buellia,s:Buellia frigida

AAGTCGTAACAAGGTTTTCGTAGGTGAACTTGCGGAAGGATCATTATCGAGAGACGGGGT

CGCTTTGGCCCCACTCTTCACTCGTGTTTACTTACCTTTGTTGCTTTGGCGGGCCTTCGG

GCTTGACCGTTCGACGCGGGTGGGAGGCTTTCGCCTCTCGTCTCGCGAGCGCTCGTCAAA

GGCCTTGTTAACTCTGTTTTAGTGTCTTTCGAGCAATCATGTAATAGTTAAAACTTTCAA

CAATGGATCT

>ASV480 SS|0.9000|KC346301|SH1107756.09FU;k:Fungi,p:Ascomycota,c:Lecanoromycetes,o:Teloschistales,f:Teloschistaceae

AAGTCGTAACAAGGTTTCCGTAGGTGAACCTGCGGAAGGATCATTATCGAGAGGGGGGCT

CCATGCCCCGGGGCTCTGTCCCCGTACCTTTTCACCCTGTGTGTATTTTTCCCCCGTTGC

TTTGGCGGGCCCCGGGTCTTCCCCCGGCGCTGGCTCCCTCGCGGGGTTCGCGAGCGCCCG

CCGAAGGCTCTTCGAAACTCTGTTGATCAGTGCAGTCTGAGCGTACGAACAATAAATCAA

AACTTTCAAC

>ASV481 GS|100.0|KF309966|SH1084317.09FU;k:Fungi,p:Ascomycota,c:Dothideomycetes,o:Mycosphaerellales,f:Teratosphaeriaceae,g:Meristemomyces,s:Meristemomyces frigidus

AAGTCGTAACAAGGTCTCCGTAGGTGAACCTGCGGAGGGATCATTACTGAGTGAGGGTGC

TTGCGCCCGACCTCCAACCCCATGTTTTCCGACTCTGTTGCCTCGGGGGCGACCCGGCCC

CCTCGTGGGGCTCGGGGCCCTCGGTGGACCGCTCAACTCTGCATCTGTGCGTCTGAGTCA

ATATTTGAATTAATCAAAACTTTTAACAACGGATCTCTTGGTTCTG

>ASV482 GS|84.3|AF250779|SH0954634.09FU;k:Fungi,p:Ascomycota,c:Lecanoromycetes,o:Caliciales,f:Caliciaceae,g:Buellia,s:Buellia frigida

AAGTCGTAACAAGATTTTCGTAAATGAATTTGCGAAAGAATCATTATCGAGAGACGAGAT

CGCTTCGACTTCACTCTTCACTCGTGTTTACTTATCTTTGTTGCTTTGACGAATCTTCGA

GCTTGATCGTTCGACGCGAGTGAGAGACTTTCGTCTCTCGTTTTGCGAGCGCTCGTCAAA

GATCTTGTTAACTTTGTTTTAGTGTCTTTCGAGCAATCATGTAATAATTAAAACTTTCAA

CAACGAATCT

>ASV483 SS|0.9900|AF250779|SH0954634.09FU;k:Fungi,p:Ascomycota,c:Lecanoromycetes,o:Caliciales,f:Caliciaceae,g:Buellia,s:Buellia frigida

AAGTCGTAACAAGGTTTTCGTAGGTGAATTTGCGAAAGGATCATTATCGAGAGACGAAGT

CGCTTCGGCCCCACTCTTCACCCGTGTCTACTTATCTTTGTTGCTTTGGCGGGCCTTCGA

GCTTGATCGTTCGACGCGGGTGGGAGGCTTTCGCCTCTCGCCTCGCGAGCGCTCGCCAAA

GGTCGTGTTAACTCTGTTTTAGTGTCTTCCGAGCAACCATGTAATAGTTAAAACTTTCAA

CAACGGATCT

>ASV484 GS|86.3|AF250779|SH0954634.09FU;k:Fungi,p:Ascomycota,c:Lecanoromycetes,o:Caliciales,f:Caliciaceae,g:Buellia,s:Buellia frigida

AAGTCGTAACAAGATTTTCGTAGATGAACTTGCGAAAAAATCATTATCGAGAGACGAGAT

CGCTTCGATCTCACTTTTCATTCGTGTCTACTTATCTTTGTTGCTTTGACGAGCCTTCGA

GCTTGATCGTTCGACGCGGGTAGAAGGCTTTCGCCTCTCGTCTCGCGAGCGCTCGTCAAA

GATTCTGTTAACTTTGTTTTAGTGTCTTTCGAGCAATCATGTAATAATTAAAACTTTCAA

CAATGAATCT

>ASV485 GS|0.0|None;No hit

GTGTCAGCAGCCGCGGTAATACGAAGGGGGCTAGCGTTGCTCGGAATGACTGGGCGTAAA

GGGCGCGTAGGCGGGCATCTTAGTCAGGCGTGAAATTCCCGGGCTTAACCTGGGGGCTGC

GTTTGATACGGGGTGCCTAGAGTTTGGAAGAGGGTCGTGGAATTCCCAGTGTAGAGGTGA

AATTCGTAGATATTGGGAAGAACACCGGTGGCGAAGGCGGCGACCTGGTCCTTGACTGAC

GCTGAGGCGC

>ASV486 GS|87.7|AF250779|SH0954634.09FU;k:Fungi,p:Ascomycota,c:Lecanoromycetes,o:Caliciales,f:Caliciaceae,g:Buellia,s:Buellia frigida

AAGTCGTAACAAGATTTTCGTAGATGAACTTGCGAAAGAATCATTATCGAGAGACGAGAT

CGCTTCGATTTCACTCTTCACTTGTGTCTACTTATCTTTGTTGCTTTGACGAATCTTTGA

GCTTGATCGTTCGACGCGAGTGAGAGACTTTCGCCTCTCGCCTCGCGAGCGCTCGCCAAA

GACCCTGTTAACTTTGTTTTAGTGTCTTCCAAGCAACCATGTAATAGTTAAAACTTTCAA

CAACGAATCT

>ASV487 GS|98.8|AY667583|SH0954634.09FU;k:Fungi,p:Ascomycota,c:Lecanoromycetes,o:Caliciales,f:Caliciaceae,g:Buellia,s:Buellia frigida

AAGTCGTAACAAGGTTTCCGTAGGTGAACCTGCGGAAGGATCATTACCGAGAGACGGGGT

CGCTTCGGCCCCACTCTTCACCCGTGTCTACTTACCTTTGTTGCTTTGGCGGGCCTTCGG

GCTTGATCGTTCGACGCGGGTGGGAGGCTTTCGCCTCTCGCCTCGCGAGCGCCCGCCAAA

GACTCTGTTAACTCTGTTTTAGTGTCTTCCGAGCAACCATGTAATAGTTAAAACTTTCAA

CAACGGATCT

>ASV488 GS|88.2|AF250779|SH0954634.09FU;k:Fungi,p:Ascomycota,c:Lecanoromycetes,o:Caliciales,f:Caliciaceae,g:Buellia,s:Buellia frigida

AAGTCGTAACAAGATTTTCGTAGGTAAATTTGCGAAAGAATCATTATCGAGAGACGAAGT

CGCTTCGATCTCACTCTTCACTCGTGTCTACTTATCTTTGTTGCTTTGGCGAGTCTTCGA

GCTTGATCGTTCGACGCGAGTGAGAGGCTTTCGTCTCTCGTCTCGCGAGCGTTCGCCAAA

GGTCTTGTTAACTTTGTTTTAGTGTCTTTCGAGCAATCATGTAATAGTTAAAACTTTCAA

CAACGAATCT

>ASV489 GS|98.1|AY843176|SH0964452.09FU;k:Fungi,p:Ascomycota

AAGTCGTAACAAGGTTTCCGTAGGTGAACCTGCGGAAGGATCATTACCGAGATAGGGTTT

CTTCGGAGCCCGACCTCCAACCCATTGTCTACCATACCTCTGTTGCCTCGGCGGGCCACC

GGCGCCTTTACGGGCGTCGCCGTCGGTCTTTACGGGCTTTCGAGCGCCCGCCGCAGGACT

TATAAACTCTTTTTTAAAGTAGTATTCTGAGTGGGAATTTAATAACTTCAAAACTTTCAA

CAACGGATCT

>ASV490 GS|82.0|AY667583|SH0954634.09FU;k:Fungi,p:Ascomycota,c:Lecanoromycetes,o:Caliciales,f:Caliciaceae,g:Buellia,s:Buellia frigida

AAGTCGTAACAAGATTTTCGTAGATGAATTTGCGAAAGAATCATTATCGAGAGACGAAAT

CGCTTCGATCTCACTCTTCACTCGTGTCTACTTATCTTTGTTGCTTTGACGAATCTTCGA

ATTTGATCGTTCGACGCGAATGAGAGACTTTCGTTTCTCATTTCGCGAGCGCTCGTCAAA

GACTTTGTTAACTCTATTTTAGTGTCTTTTGAGCAATCATGTAATAATTAAAACTTTCAA

TAACGAATCT

>ASV491 GS|86.3|AF250779|SH0954634.09FU;k:Fungi,p:Ascomycota,c:Lecanoromycetes,o:Caliciales,f:Caliciaceae,g:Buellia,s:Buellia frigida

AAGTCGTAACAAGATTTTCGTAAATGAATTTGCGAAAGAATCATTATCGAGAGACGAGAT

CGCTTCGATCTCACTCTTCACTCGTGTCTACTTATCTTTGTTGCTTTGACGAGTCTTCGA

ACTTGATCGTTCGACGCGAGTGAGAGACTTTCGTCTCTCGTCTCGCGAGCGCTCGTCAAA

GATCTTGTTAACTCTATTTTAGTGTCTTTCGAGCAATCATGTAATAGTTAAAACTTTCAA

CAACGAATCT

>ASV492 SS|0.9000|MZ229869|SH0913119.09FU;k:Fungi,p:Ascomycota,c:Lecanoromycetes

AAGTCGTAACAAGGTTTCCGTAGGTGAACCTGCGGAAGGATCATTATCGAGAGACGGAGT

CTAACCGGCCCCACTCTTCACCCGTGTATACCTACCCTTTGTTGCTTTGGCGGGCGGTCG

GTCTTTATCGCCCGACGTCGGTCGGAAGGCTCGCGCCTTCCATTCACCGAACGCCCGCCA

GAGGCCCCCAAAAGCCGTCCATCTGTGTCCGAGCGAGTGAAGCAAATAGTTAAAAACTTT

CAACAACGGA

>ASV493 SS|0.9700|MF138060|SH0954356.09FU;k:Fungi,p:Ascomycota,c:Lecanoromycetes,o:Acarosporales,f:Acarosporaceae,g:Acarospora

AAGTCGTAACAAGGTTTCCGTAGGTGAACCTGCGGAAGGATCATTACAGAGTTAGGGTCT

TTCCAGGCCCGACCTCCAACCCTATGTGTACCTACCTTTGTTGCTTTGGCGGGCCCGTCG

GGTGACCCACCGGTGGCCTCTGGCTCCCGAGTGCCCGTCAGAGACCCATCAAAACCCGTT

AATTGTGTCGTCTGAGTACCACTTTAATAATTAAAACTTTCAACAACGGATCTCTTGGTT

CTG

>ASV494 GS|90.0|AY667583|SH0954634.09FU;k:Fungi,p:Ascomycota,c:Lecanoromycetes,o:Caliciales,f:Caliciaceae,g:Buellia,s:Buellia frigida

AAGTCGTAACAAGGTTTTCGTAGGTGAATTTGCGAAAGGATCATTATCGAGAGACGAGGT

CGCTTCGACTCCACTCTTCACTCGTGTCTACTTACCTTTGTTGCTTTAGTGGGCCTTCGA

GCTTGATCGTTCGATGCGAGTGGGAGACTTTCGTCTCTCGTCTCGCGAGCGCTCGCCAAA

GGCTTTGTTAACTCTATTTTAGTGTCTTTCGAGCAATCATGTAATAGTTAAAACTTTCAA

CAACGAATCT

>ASV495 GS|0.0|None;No hit

GTGTCAGCAGCCGCGGTAATACGGAGGGTGCGAGCGTTGTCCGGATTTATTGGGTTTAAA

GGGTGCGTAGGCGGCCGTTTAAGTCTGGGGTGAAAGCCCGCTGCTCAACAGCGGAACTGC

CCTGGATACTGGATGGCTTGAATACAGTGGAGGTTGGCGGAATGGACCGAGTAGCGGTGA

AATGCATAGATACGGTCCAGAACCCCGATTGCGAAGGCAGCTGACTACACTGGTATTGAC

GCTGAGGCAC

>ASV496 SS|0.8200|MZ229889|SH0913114.09FU;k:Fungi,p:Ascomycota,c:Lecanoromycetes,o:Caliciales,f:Caliciaceae

AAGTCGTAACAAGGTTTCCGTAGGTGAACCTGCGGAAGGATCATTATCGAGAGACGGAGT

CTAACCGGCCCCACTCTTCACCCGTGTATACCTACCCTTTATTGCTTTGGCGGGCGGTCG

GTCTTTACCGCCCGACGTCGGTCGGAAGGCTCGCGCCTTCCACTCACCGAACGCCCGCCA

GAGGCCCCCAAAAGCCGTCCATCTGTGTCCGAGCGAGTGAAGCAAATAGTTAAAAACTTT

CAACAACGGA

>ASV497 GS|83.3|AF250779|SH0954634.09FU;k:Fungi,p:Ascomycota,c:Lecanoromycetes,o:Caliciales,f:Caliciaceae,g:Buellia,s:Buellia frigida

AAGTCGTAACAAGATTTTCGTAAATGAATCTACGAAAGAATCATTATCGAAAGACGAAAT

CGCTTCGATCTCACTCTTCACTCGTGTCTACTTATCTTTGTTACTTTGACGAGTCTTCGA

GCTTGATCGTTCGACGCGAGTGAGAGACTTTCGTCTCTCGTCTTGCGAGCGCTCGTCAAA

GATTTTGTTAACTCTATTTTAGTGTCTTTCGAGCAATTATGTAATAGTTAAAACTTTCAA

CAACAAATCT

>ASV498 SS|0.9900|AY667583|SH0954634.09FU;k:Fungi,p:Ascomycota,c:Lecanoromycetes,o:Caliciales,f:Caliciaceae,g:Buellia,s:Buellia frigida

AAGTCGTAACAAGGTTTTCGTAGGTGAACCTGCGGAAGGATCATTATCGAGAGACGGGGT

CGCTTCGGCCTCACTCTTCACTCGTGTCTACTTATCTTTGTTGCTTTGGCGGGCCTTCGG

GCTTGATCGTTCGACGCGGGTGGGAGGCTTTCGCCTCTCGTCTCGCGAGCGCTCGTCAAA

GGCCTTGTTAACTCTGTTTTAGTGTCTTCCGAGCAACCATGTAATAGTTAAAACTTTCAA

CAACGGATCT

>ASV500 SS|1.0000|KF274277|SH0999504.09FU;k:Fungi

AAGTCGTAACAAGGTTTCCGTAGGTGAACCTGCGGAAGGATCATTAGTGATTGGTCTCCG

GACCATTTTCATCATCCACAAACCTCTGTGAACCTGTCGGCCCTCGGGCCAGCTTTTCCA

AACAAAGTGTAACGAACGTGTGATATTATAATCTAGTAAAACTTTCAACAACGGATCTCT

TGGCTCTC

>ASV501 GSL|100.0|OK644453|SH1301428.09FU;k:Fungi

AAGTCGTAACAAGGTCTCCGTAGGTGAACCTGCGGAGGGATCATTACACAAATATGAAGG

CGGGCTGGAACCTCTCGGGGTTACAGCCTTGCTGAATTATTCACCCTTGTCTTTTGCGTA

CTTCTTGTTTCCTTGGTGGGTTCGCCCACCACTAGGACAAACATAAACCTTTTGTAATTG

CAATCAGCGTCAGTAACAAATTAATAATTACAACTTTCAACAACGGATCTCTTGGTTCTG

>ASV502 GS|85.3|AF250779|SH0954634.09FU;k:Fungi,p:Ascomycota,c:Lecanoromycetes,o:Caliciales,f:Caliciaceae,g:Buellia,s:Buellia frigida

AAGTCGTAACAAGATTTTCGTAGATGAATCTACGAAAGAATCATTATCGAGAGACGAAAT

CGCTTCGATCTCACTCTTCACTCGTATCTACTTATCTTTGTTGCTTTGACGAGTCTTCGA

GCTTGATCGTTCGACGCGAGTAAGAGACTTTCGTCTCTCGTCTCGCGAGCGCTCGTCAAA

GACTTTGTTAACTCTATTTTAGTGTCTTTCGAGCAATCATGTAATAGTTAAAACTTTCAA

CAACGAATCT

>ASV503 GS|86.4|AY667583|SH0954634.09FU;k:Fungi,p:Ascomycota,c:Lecanoromycetes,o:Caliciales,f:Caliciaceae,g:Buellia,s:Buellia frigida

AAGTCGTAACAAGATTTTCGTAAGTGAACTTGCGAAAGAATCATTATCGAGAGACGAGAT

CGCTTCGATCTCACTCTTCACTCGTGTCTACTTATCTTTGTTGCTTTGACGAGTCTTCGA

GCTTGATCGTTTGATGCGAGTGAAAGACTTTCGCCTCTCGTCTCGCGAGCGCTCGTCAAA

GATCTTGTTAACTTTGTTTTAGTGTCTTTCGAGCAATCATGTAATAGTTAAAACTTTCAA

CAACGGATCT

>ASV504 GS|82.4|AF250779|SH0954634.09FU;k:Fungi,p:Ascomycota,c:Lecanoromycetes,o:Caliciales,f:Caliciaceae,g:Buellia,s:Buellia frigida

AAGTCGTAACAAGATTTTCGTAAATGAATTTGCGAAAGAATTATTATCGAGAGACGAGAT

CGCTTCGATTTCACTCTTCACTCGTATCTACTTATCTTTGTTGCTTTAACGAATCTTCGA

GCTTGATCGTTCGACGCGAATGAGAGACTTTCGTCTCTCGTTTCGCGAGCGCTCGTTAAA

GATTTTGTTAACTCTATTTTAGTGTCTTTCGAGCAATTATGTAATAGTTAAAACTTTCAA

CAACGAATCT

>ASV505 GS|0.0|None;No hit

GTGTCAGCAGCCGCGGTAATACGGAGGGTGCGAGCGTTGTCCGGATTTATTGGGTTTAAA

GGGTGCGTAGGCGGCCGTTTAAGTCTGGGGTGAAAGCCCGCTGCTCAACAGCGGAACTGC

CCTGGATACTGGATGGCTTGAATACAGTGGAGGTTGGCGGAATGGACTGAGTAGCGGTGA

AATGCATAGATACAGTCCAGAACCCCGATTGCGAAGGCAGCTGACTACACTGGTATTGAC

GCTGAGGCAC

>ASV506 GS|0.0|None;No hit

GTGTCAGCAGCCGCGGTAATACGGAGGGTGCGAGCGTTGTCCGGATTTATTGGGTTTAAA

GGGTGCGTAGGCGGCCGATTAAGTCTGGGGTGAAAGCCCGCTGCTCAACAGCGGAACTGC

CCTGGATACTGGTTGGCTTGAGTACAGACGAGGTTGGCGGAATGGACCGAGTAGCGGTGA

AATGCATAGATACGGTCCAGAACCCCGATTGCGAAGGCAGCTGACTAGGCTGTTACTGAC

GCTGAGGCAC

>ASV507 GS|82.8|AF250779|SH0954634.09FU;k:Fungi,p:Ascomycota,c:Lecanoromycetes,o:Caliciales,f:Caliciaceae,g:Buellia,s:Buellia frigida

AAGTCGTAACAAGATTTTCATAGATGAATCTACGAAAAAATCATTATCGAGAGACGAGAT

CGCTTCGATTTCACTCTTCATTCGTATCTACTTATCTTTGTTGCTTTGACGAGTCTTCGA

GCTTAATCGTTCGACGCGAGTGAGAGACTTTCGTCTCTCGTCTCGCGAGCGCTCGTCAAA

GATCTTATTAACTTTGTTTTAGTGTCTTTCAAGTAGTCATGTAATAGTTAAAACTTTCAA

CAACGAATCT

>ASV508 SS|0.8600|MN103133|SH0913114.09FU;k:Fungi,p:Ascomycota,c:Lecanoromycetes,o:Caliciales

AAGTCGTAACAAGGTTTCCGTAGGTGAACCTGCGGAAGGATCATTATCGAGAGACGGAGT

CTAACCGGCCCCACTCTTCACCCGTGTATACCTACCCTTTGTTGCTTTGGCGGGCGGTCG

GTCTTTACCGCCCGACGTCGGTCGGAAGGCTCGCGCCTTTCACTCACCGAACGCCCGCCA

GAGGCTTCCAAAAGCCGTCCATCTGTGTTCGAGCGAGTGAAGCAAATAGTTAAAAACTTT

CAACAACGGA

>ASV509 GS|88.7|AF250779|SH0954634.09FU;k:Fungi,p:Ascomycota,c:Lecanoromycetes,o:Caliciales,f:Caliciaceae,g:Buellia,s:Buellia frigida

AAGTCGTAACAAGGTTTTCGTAAATGAATTTGCGAAAGAATCATTATCGAGAGACGAAAT

CGCTTCGACCTCACTCTTCACTCGTGTCTACTTACCTTTGTTGCTTTGGCGAGTCTTCGA

ACTTGATCGTTCGACGCGAGTGAGAGACTTTCGCCTCTCGTCTTGCGAGCGCTCGCCAAA

GGCCTTGTTAACTTTGTTTTAGTGTCTTTCGAGCAATCATGTAATAGTTAAAACTTTCAA

CAACGAATCT

>ASV510 SS|1.0000|AF250779|SH0954634.09FU;k:Fungi,p:Ascomycota,c:Lecanoromycetes,o:Caliciales,f:Caliciaceae,g:Buellia,s:Buellia frigida

AAGTCGTAACAAGATTTTCGTAGATGAACCTGCGGAAGGATCATTATCGAGAGACGGGAT

CGCTTCGGCCCCACTCTTCACTCGTGTCTACTTACCTTTGTTGCTTTGACGAGTCTTCGA

GCTTGATCGTTCGACGCGGGTGGGAGGCTTTCGCCTCTCGCCTCGCGAGCGCCCGCCAAA

GGCCCTGTTAACTCTGTTTTAGTGTCTTCCGAGCAACCATGTAATAGTTAAAACTTTCAA

CAACGGATCT

>ASV511 GS|99.2|GU170840|SH0942719.09FU;k:Fungi,p:Ascomycota,c:Lecanoromycetes,o:Lecanorales,f:Lecanoraceae,g:Lecanora,s:Lecanora fuscobrunnea

AAGTCGTAACAAGGTTTCCGTAGGTGAACCTGCGGAAGGATCATTATCGAGAGGGGTCCC

CGGACTCCGGGGGCTTCGGCCCCCTACTCTTCACCCTATGTCTACACACCTTTGCTGCTT

TGGCGGGCCTCGGGTTCGCCCCGTACCGGCCGTGGGCTTCCATACCCCGGCTGTCCGTGC

CCGTCAGAGGCCCATGAACCCTCGTTTATCAGTGTCGTCCGAGTCCAACCACAATAGTAA

AAACTTTCAA

>ASV512 GS|74.9|DQ534454|SH0954616.09FU;k:Fungi,p:Ascomycota,c:Lecanoromycetes,o:Caliciales,f:Caliciaceae,g:Buellia,s:Buellia russa

AAGTCGTAACAAGATTTTCGTAAATGAATTTACGAAAAAATCATTATCGAGAGACGAAAT

CGCTTCGATCTCACTCTTCACTCGTATTTACTTATCTTTGTTGTTTTGACGAATCTTCGA

ACTTGATCGTTCGACGCGAATGAAAGACTTTCGTCTCTCGTTTCGCGAGCGTTCGTCAAA

GATCTTGTTAACTTTGTTTTAATGTCTTTCGAGCAATCATGTAATAATTAAAACTTTTAA

CAATGAATCT

>ASV513 GS|87.3|AF250779|SH0954634.09FU;k:Fungi,p:Ascomycota,c:Lecanoromycetes,o:Caliciales,f:Caliciaceae,g:Buellia,s:Buellia frigida

AAGTCGTAACAAGGTTTTCGTAGATGAATCTACGAAAGAATCATTATCGAAAGACGAGAT

CGCTTCGACCTCACTCTTCACTCGTGTCTACTTATCTTTGTTGCTTTGACGAGTCTTCGA

GCTTGATCGTTCGACGCGAGTGAGAGACTTTCGTCTCTCGTTTCGCGAGCGTTCGTCAAA

GGCCCTGTTAACTTTGTTTTAGTGTCTTTCGAGCAATCATGTAATAGTTAAAACTTTCAA

CAACGAATCT

>ASV514 SS|0.9600|AF250779|SH0954634.09FU;k:Fungi,p:Ascomycota,c:Lecanoromycetes,o:Caliciales,f:Caliciaceae,g:Buellia,s:Buellia frigida

AAGTCGTAACAAGGTTTTCGTAGGTGAATCTGCGAAAGGATCATTATCGAGAGACGAGGT

CGCTTCGGCCTCACTCTTCACTCGTGTCTACTTATCTTTGTTGCTTTGGCGGGCCTTCGG

GCTTAATCGTTCGACGCGGGTGGGAGGCTTTCGCCTCTCGCCTCGCGAGCGCTCGCCAAA

GGCCTTGTTAACTTTGTTTTAGTGTCTTTCGAGCAACCATGTAATAGTTAAAACTTTCAA

CAACGGATCT

>ASV515 GS|97.4|KP903330|SH0989164.09FU;k:Fungi,p:Ascomycota,c:Dothideomycetes,o:Mycosphaerellales,f:Teratosphaeriaceae,g:Catenulostroma

AAGTCGTAACAAGGTCTCCGTAGGTGAACCTGCGGAGGGATCATTACCGAGCGAGGGTCC

TCCGGGCCCGACCTCTCAACCCCATGTGACCCGACACCGTTGCCTCGGGGGCGACCCGGC

CGCTTCGGCCGGGGCCCCCGGTGGACCGACCAAACACTGCATCCGTGCGTCCGAGTCCCA

TGATAAATCAATCAAAACTTTCAACAACGGATCTCTTGGTTCTG

>ASV516 SS|1.0000|KC965541|SH0962007.09FU;k:Fungi

AAGTCGTAACAAGGTTTCCGTAGGGTACGTTCACCCGCATAAACCCCCCCTTGCTACGTC

GAAAATGATTTGCTAACCCCTATTCTAGTGAACCTGCGGAAGGATCATTAAAGAGAAATC

GGGGTATCGCCGCGAGGCTTTACCCCATCTCCCTAAACCCCCTGTCTACCGACCTCACAC

AAACCATAGTACAAATATCAAGTCGTCCCTAGCGGGGCGCAATACCATAAATGTACAAAA

AAACAAAAAA

>ASV517 GS|85.8|AF250779|SH0954634.09FU;k:Fungi,p:Ascomycota,c:Lecanoromycetes,o:Caliciales,f:Caliciaceae,g:Buellia,s:Buellia frigida

AAGTCGTAACAAGATTTTCGTAGATGAACTTGCGAAAAAATCATTATCGAGAGACGAAAT

CGCTTCGATTTCACTCTTCACTCGTGTCTACTTATCTTTGTTGCTTTGACGAGTCTTCGA

GCTTGATCGTTCGACGCGAGTGAGAGACTTTCGTCTCTCGTCTCGCGAGCGCTCGTCAAA

GGTCTTGTTAACTCTATTTTAGTGTCTTTCGAACAATCATGTAATAGTTAAAACTTTCAA

CAACGAATCT

>ASV518 GS|98.4|AF250779|SH0954634.09FU;k:Fungi,p:Ascomycota,c:Lecanoromycetes,o:Caliciales,f:Caliciaceae,g:Buellia,s:Buellia frigida

CTTTTTCCTTTCTCTTCCTTCCCCTTCGTCCCCCGGTTTCCGTAGGTGCCCCTGCGGACG

TCTCCTTACCGAGCGCCTGGGTCGCTTCGGCCCCACTCTTCACCCGTGTCTACTTACCTT

TGTTGCTTTGGCGGGCCTTCGGGCTTGACCGTTCGACGCGGGTGGGAGGCTTTCGCCTCT

CGCCTCGCGAGCGCCCGCCAAAGGCCCTGTTAACTCTGTTTTAGTGTCTTCCGAGCAACC

ATGTAATAGT

>ASV519 SS|1.0000|AY667583|SH0954634.09FU;k:Fungi,p:Ascomycota,c:Lecanoromycetes,o:Caliciales,f:Caliciaceae,g:Buellia,s:Buellia frigida

AAGTCGTAACAAGGTTTCCGTAGGTGAATCTGCGGAAGGATCATTATCGAGAGACGGGGT

CGCTTCGGCCCCACTCTTCACCCGTGTCTACTTACCTTTGTTGCTTTGGCGGGTCTTCGG

GCTTGATCGTTCGACGCGGGTGGGAGGCTTTCGCCTCTCGTCTCGCGAGCGCTCGCCAAA

GGCCCTGTTAACTCTGTTTTAGTGTCTTTCGAGCAATCATGTAATAGTTAAAACTTTCAA

CAACGGATCT

>ASV520 SS|0.9300|MW991425|SH0964445.09FU;k:Fungi,p:Ascomycota,c:Arthoniomycetes,o:Lichenostigmatales,f:Phaeococcomycetaceae,g:Antarctolichenia,s:Antarctolichenia onofrii

AAGTCGTAACAAGGTTTCCGTAGGTGAACCTGCGGAAGGATCATTACCGAGATAGGGTTT

CTTCGGAGCCCGACCTCCAACCCATTGTCTACCATACCTCTGTTGCCTCGGCGGGCCGCC

GGCGCCCTTTGTTGGGCGCCGCCGTCGGCTTTAGGGCTTTCGAGCGCCCGCCGCAGGACC

GATTAAACTCTTTTTAAAACAAGTCTTCTGAGTGGGAAATCAAATTTATTAAAACTTTTA

ACAACGGATC

>ASV521 GS|0.0|None;No hit

GTGTCAGCAGCCGCGGTAATACGGAGGGAGCTAGCGTTATTCGGAATTACTGGGCGTAAA

GCGCACGTAGGCGGCTTTGTAAGTTAGAGGTGAAAGCCCAGAGCTCAACTCTGGAATTGC

CTTTAAGACTGCATCGCTTGAATCCAGGAGAGGTGAGTGGAATTCCGAGTGTAGAGGTGA

AATTCGTAGATATTCGGAAGAACACCAGTGGCGAAGGCGGCTCACTGGACTGGTATTGAC

GCTGAGGTGC

>ASV522 GS|0.0|None;No hit

GTGTCAGCAGCCGCGGTAATACGGAGGGTGCGAGCGTTGTCCGGATTTATTGGGTTTAAA

GGGTGCGTAGGCGGTCGATTAAGTCTGGGGTGAAAGCCCGCTGCTCAACAGCGGAACTGC

CCTGGATACTGGTTGACTTGAGTACAGACGAGGTTGGCGGAATGGACGGAGTAGCGGTGA

AATGCATAGATACCGTCCAGAACCCCGATTGCGAAGGCAGCTGACTAGGCTGTTACTGAC

GCTGAGGCAC

>ASV523 SS|1.0000|MF976578|SH1193052.09FU;k:Fungi

AAGTCGTAACAAGGTTTCCGTAGGTGAACCTGCGGAAGGATCATTACCGAGCCAGGGTCC

CCCCGGGGGCCCGACCTCCAACCCGCTGCCTACAAACCCCTGTTGCCTCGGCGGCTCCGG

GCTCCGGCCCCCCGTCGGTGGACCACTGAAAGTCCGTTCGAACCCCATTCTGAGCCCGAA

ATCAAGTTTACAAAACTTTCAACAACGGATCTCTTGGTTCCC

>ASV524 SS|1.0000|AF250779|SH0954634.09FU;k:Fungi,p:Ascomycota,c:Lecanoromycetes,o:Caliciales,f:Caliciaceae,g:Buellia,s:Buellia frigida

AAGTCGTAACAAGGTTTTCGTAGGTGAATCTGCGGAAGGATCATTACCGAGAGACGAGGT

CGCTTCGGTCTCACTCTTCACTCGTGTCTACTTATCTTTGTTGCTTTGGCGGGCCTTCGG

GCTTGACCGTTCGACGCGGGTGGGAGGCTTTCGCCTCTCGCCTCGCGAGCGCCCGCCAAA

GGTTCTGTTAACTCTGTTTTAGTGTCTTTCGAGCAACCATGTAATAGTTAAAACTTTCAA

CAACGGATCT

>ASV525 GS|85.8|AF250779|SH0954634.09FU;k:Fungi,p:Ascomycota,c:Lecanoromycetes,o:Caliciales,f:Caliciaceae,g:Buellia,s:Buellia frigida

AAGTCGTAATAAGATTTTCGTAGATGAATTTGCGAAAGAATCATTATCGAGAGACGAGAT

CGCTTCGATCTCACTCTTTACTCGTGTCTACTTATCTTTGTTGCTTTGACGAGTCTTTGA

GCTTGATCGTTCGACGCGAGTGAAAGACTTTCGTCTCTCGTCTCGCGAGCGCTCGTCAAA

GACTCTATTAACTCTGTTTTAGTGTCTTTCGAGCAATCATGTAATAGTTAAAACTTTCAA

CAACGAATCT

>ASV526 SS|1.0000|AF250779|SH0954634.09FU;k:Fungi,p:Ascomycota,c:Lecanoromycetes,o:Caliciales,f:Caliciaceae,g:Buellia,s:Buellia frigida

AAGTCGTAACAAGGTTTTCGTAGGTGAACTTGCGGAAGGATCATTATCGAGAGACGGGGT

CGCTTCGGCCCCACTCTTCACTCGTGTCTACTTACCTTTGTTGCTTTGGCGGGTCTTCGG

GCTTGATCGTTCGACGCGGGTGGGAGGCTTTCGCCTCTCGCCTTGCGAGCGCCCGCCAAA

GGTCGTGTTAACTCTGTTTTAGTGTCTTCCGAGCAATCATGTAATAGTTAAAACTTTCAA

CAACGGATCT

>ASV527 SS|0.9900|MK970687|SH0954634.09FU;k:Fungi,p:Ascomycota,c:Lecanoromycetes,o:Caliciales,f:Caliciaceae,g:Buellia,s:Buellia frigida

AAGTCGTAACAAGGTTTTCATAGATGAATTTGCGAAAGGATCATTATCGAGAGACGGGGT

CGCTTCGATCTCATTCTTCACTCGTGTCTACTTATCTTTGTTGCTTTGGCGAGCCTTCGG

GCTTGATCGTTCGACGCGGGTGGGAGGCTTTCGTTTGTCGTCTCGCGAGCGCTCGTCAAA

GGCCCTGTTAACTTTGTTTTAGTGTCTTTCGAGCAATCATGTAATAGTTAAAACTTTCAA

CAATGAATCT

>ASV528 GS|97.1|AF250779|SH0954634.09FU;k:Fungi,p:Ascomycota,c:Lecanoromycetes,o:Caliciales,f:Caliciaceae,g:Buellia,s:Buellia frigida

AAGTCGTAACAAGGTTTTCGTAGGTGAACTTGCGAAAGGATCATTATCGAGAGACGGGGT

CGCTTCGGCCCCACTCTTCACTCGTGTCTACTTACCTTTGTTGCTTTGGCGGGCCTTCGG

GCTTGATCGTTCGACGCGGGTGGAAGGCTTTCGCCTCTCGCCTCGCGAGCGCCCGCCAAA

GGCCCTGTTAACTCTGTTTTAGTGTCTTTCGATCAACCATGTAATAGTTAAAACTTTCAA

CAACGGATCT

>ASV529 SS|0.9900|AY081157|SH1302831.09FU;k:Fungi,p:Ascomycota,c:Lecanoromycetes,o:Teloschistales,f:Teloschistaceae

AAGTCGTAACAAGGTTTCCGTAGGTGAACCTGCGGAAGGATCATTACTAAGAGAGGGATG

TACGCTTCCAGCCGAGTCCCGGGGGGCTGCGCCCCTCACCTCTTCAACCCTGTGTATATC

AATCCATGTTGCTTTGGCGAGCGCCGGGGAGCGATTCCCGGCCCTGGCTTCGGTCAGTCA

GCCCTCGTCAGAGGCCCATCCAAATTCTGTTTCAGTGACGTCCGAGTATACCAGCAAATA

AATTAAAACT

>ASV530 GS|100.0|JN885556|SH1084060.09FU;k:Fungi,p:Ascomycota,c:Dothideomycetes,o:Mycosphaerellales,f:Teratosphaeriaceae,g:Friedmanniomyces,s:Friedmanniomyces endolithicus

AAGTCGTAACAAGGTCTCCGTAGGTGAACCTGCGGAGGGATCATTACTGAGCGAGGGCCT

CCGGGTCCGACCTCCAACCCCCTGTTATCCGACCACTGTTGCCTTGGGGGCGACCCGGCC

TTCGGGCGTCGGGGCCCCCAACGGACCCGCCAACCCTGCATCTGTGCGTCCGAGTCAACG

ATTGAATCAATCAAAACTTTCAACAACGGATCTCTTGGTTCTG

>ASV531 GS|0.0|None;No hit

GTGTCAGCAGCCGCGGTAATACGAAGGGGGCTAGCGTTGCTCGGAATGACTGGGCGTAAA

GGGCGCGTAGGCGGACATTTTAGTCGGGCGTGAAATTCCTGGGCTTAACCTGGGGGCTGC

GTTCGATACGGGGTGTCTAGAGTTTGGCAGAGGGTCGTGGAATTCCCAGTGTAGAGGTGA

AATTCGTAGATATTGGGAAGAACACCGGTGGCGAAGGCGGCGACCTGGTCCTTGACTGAC

GCTGAGGCGC

>ASV532 GSL|100.0|KF861806|SH1188820.09FU;k:Fungi,p:Basidiomycota,c:Tremellomycetes,o:Filobasidiales,f:Filobasidiaceae,g:Naganishia

AAGTCGTAACAAGGTTTCCGTAGGTGAACCTGCGGAAGGATCATTAATGAATACAGATGC

CTGTCGAGCTTGCTCACGGGCTTTCTATCATATCCATAACACCTGTGCACTTGTTGGATG

ATCTAAAGACATAGGGTAAAACCTAAAGTCAATAGTCATCCACTTACACTAAACAATAAT

GTAACAAATGTAGTCTTATTATAACAAAATAAAACTTTCAACAACGGATCTCTTGGCTCT

C

>ASV533 GS|83.6|AY667583|SH0954634.09FU;k:Fungi,p:Ascomycota,c:Lecanoromycetes,o:Caliciales,f:Caliciaceae,g:Buellia,s:Buellia frigida

AAGTCGTAACAAGATTTTCGTAGATGAATTTGCGAAAGAATCATTATCGAGAGACGAAAT

CGCTTCGATCTCACTCTTCACTCGTATTTACTTATCTTTGTTGCTTTGACGAGTCTTCGA

GCTTGATCGTTCGACGCGAGTGAGAGACTTTTGTTTCTCGTCTCGCGAGCGTTCGTCAAA

GACTTTGTTAACTTTGTTTTAATGTCTTTCGAGCAATCATGTAATAGTTAAAACTTTCAA

CAACGAATCT

>ASV534 GS|80.3|OL603994|SH0942717.09FU;k:Fungi,p:Ascomycota,c:Lecanoromycetes,o:Lecanorales,f:Lecanoraceae,g:Lecanora,s:Lecanora intricata

AAGTCGTAACAAGGTTTCCGTAGGTGCAGTCCAACGTTCGCCTGTGGGAACCGTGCAGGA

TCGACTCTAAATCAACTGCTCCAGTCCGCCCGTGGTTAGGGTCGGCGACGCCATCAGTCT

GCGCTGGGAGGGTCCTTTCGGGGTCAACCAGCAGCTCCTCTAGGGAGTCCACAGATCAAA

CGACGGCGGCCACAAGATCGTGGTTTAGATATGATCGGCCCCAGCCATCATTGGCTGGCG

ATATGATAGC

>ASV535 GS|99.6|FJ392865|SH1071027.09FU;k:Fungi,p:Ascomycota,c:Eurotiomycetes,o:Chaetothyriales,f:Trichomeriaceae,g:Knufia

AAGTCGTAACAAGGTTTCCGTAGGTGAACCTGCGGAAGGATCATTACCGAGTTAGGGTCT

CTTCGGAGCCCGAACCTCCCAACCCTTTGTCTAATTTACCTTGTCGTTGCTTCGGCGGAC

CGGTTGACCAACTGGTCTTGACCGCCGGGGGTCCCGTACCCCTGGAGAGCGTCCGCCGAC

GGCCCAACCACAAACTCTTGTACTAAACCATGTCGTCTGAATGTCCTGGATATTAATCAA

AAAACAAAAC

>ASV536 SS|1.0000|AY081152|SH1300501.09FU;k:Fungi,p:Ascomycota,c:Lecanoromycetes,o:Teloschistales,f:Teloschistaceae

CTTTTTCCTTTCTCTTCCTTCCCCTTCTTCCCCCTTTTTCCTTCGGTGAACCTGCGGCAG

GATCATTCCTCCGAGAGGGATGTCCGCTTCCAGCCGAGTCCCGGGGGGCTTCTCCCCTCA

CCTCTTCCACCCTGTGTCTACCAACCGCTGTTGCTTCGGCGAGCGTCGGGGCGTCCGCGC

CCCGGCCCCGGCTTCGGTCGGTGAGCTCTCGCAGAGGCCTATCTTTATTCTGTTTTGCCG

TGACGTCCGC

>ASV537 GS|97.1|AF250779|SH0954634.09FU;k:Fungi,p:Ascomycota,c:Lecanoromycetes,o:Caliciales,f:Caliciaceae,g:Buellia,s:Buellia frigida

AAGTCGTAACAAGGTTTTCGTAGATGAACCTACGAAAGAATCATTATCGAGAGACGAGGT

CGCTTCGATCCCACTCTTCACCCGTGTCTACTTACCTTTGTTGCTTTGGCGAGCCTTCGG

GCTTGATCGTTCGACGCGGGTGGGAGGCTTTCGCCTCTCGCCTCGCGAGCGCCCGCCAAA

GGCCCTGTTAACTCTGTTTTAGTGTCTTCCGAGCAACCATGTAATAGTTAAAACTTTCAA

CAACGGATCT

>ASV538 GS|85.2|AY667583|SH0954634.09FU;k:Fungi,p:Ascomycota,c:Lecanoromycetes,o:Caliciales,f:Caliciaceae,g:Buellia,s:Buellia frigida

AAGTCGTAACAAGGTTTTCGTAGATGAATTTGCGAAAGAATCATTATCGAGAGACGAGGT

CGCTTCGATCTCACTCTTCACTCGTATCTACTTATCTTTGTTGCTTTGACGAGTCTTCGA

GCTTAATCGTTCGACGCGAATGAGAGGCTTTCGTTTTTCGTCTCGCGAGCGTTCGTCAAA

GACTTTGTTAACTTTGTTTTAGTGTCTTTCGAGCAATCATGTAATAGTTAAAACTTTCAA

CAACGAATCT

>ASV539 GS|78.4|AY667583|SH0954634.09FU;k:Fungi,p:Ascomycota,c:Lecanoromycetes,o:Caliciales,f:Caliciaceae,g:Buellia,s:Buellia frigida

AAGTCGTAACAAAATTTTCGTAAATGAACTTGCGAAAAAATCATTATCGAAAGACGAAAT

CGCTTCGATCTTACTTTTCACTCGTGTCTACTTATCTTTGTTGCTTTGACGAATCTTCGA

ATTTGATCGTTCGATGCGAATGAGAGATTTTCGTCTTTCGTTTCGCGAGCGTTCGTTAAA

GACTCTATTAATTTTGTTTTAGTGTCTTTCGAACAATCATGTAATAATTAAAACTTTTAA

CAACGAATCT

>ASV540 GS|97.1|UDB0262447|SH0956955.09FU;k:Fungi,p:Ascomycota,c:Lecanoromycetes

AAGTCGTAACAAGGTTTCCGTAGGTGAACCTGCGGAAGGATCATTACTGAGAGGGGTTTT

CGGACCCCGGGGGCTCCGGCCCCCAACTCATCACCCTTTGTGTATCTACCTTTGTTGCTT

TGGCGGGCCTTGAGGTTCGCCTCATGGCGGCTCCGGGCTTTGTTGCCCCGGCCGTCTCGT

GCTCGTCAGAGACCCATCAAACTCCATTCTATCAGTATCGTCCGAGTAAAAACACAATAG

TAAAAAACTT

>ASV541 SS|0.8600|MG982528|SH1003748.09FU;k:Fungi,p:Basidiomycota,c:Tremellomycetes,o:Tremellales

AAGTCGTAACAAGGTTTCCGTAGGTGAACCTGCGGAAGGATCATTAGTGATTCGGCCCTC

ACGGGTCTATAAAAGACACCTCTGTGAACCTGTCGGCCTCCGGGCCCACCTGCAAACACT

GTGTGACAAGCGTCGATGTATCATAAGCATAATAAAACTTTCAACAACGGATCTCTTGGC

TCTC

>ASV542 GS|100.0|OK051379|SH1071219.09FU;k:Fungi,p:Basidiomycota,c:Tremellomycetes,o:Cystofilobasidiales,f:Mrakiaceae,g:Mrakia,s:Mrakia frigida

AAGTCGTAACAAGGTTTCCGTAGGTGAACCTGCGGAAGGATCACTAGTGATTAAATCGAG

AGTGTCTTCATTGACCTCTCACCCTTCACATCCACATACACCTGTGCACCGTTTGGCTCT

TTTAAAAGACGCAAGTCTGCAAAGAGAGTCATCAATTTTATACATACCCCAGTCTTATGA

ATGTAACAGTTTTAATAAACATAATAAAACTTTTAACAACGGATCTCTTGGTTCTC

>ASV543 GS|0.0|None;No hit

AAGTCGTAACAAGATTTCCGTAAATGAATTTGCGAAAGAATCATTATCGAGAAACGGAGT

TTAACCGATTCCACTCTTCACTCGTGTATATTTACTCTTTATTGTTTTAACGGGCGATCG

GTCTTTATCGCTCGACGTCGGTCGGAAAACTCGCGTCTTTCACTCACCGAACGCTCGTCA

AAGATTCTCAAAATCCGTTCATTTATGTTCGAGCGAATGAAATAAATAATTAAAAACTTT

CAACAACGGA

>ASV544 GS|83.3|AF250779|SH0954634.09FU;k:Fungi,p:Ascomycota,c:Lecanoromycetes,o:Caliciales,f:Caliciaceae,g:Buellia,s:Buellia frigida

AAGTCGTAACAAGATTTTCGTAAATGAATTTGCGAAAGAATCATTATCGAAAGACGAAAT

CGCTTCGATCTCACTCTTCATTCGTGTCTACTTATCTTTGTTGCTTTGACGAGTCTTTGA

GCTTGATCGTTCGATGCGAGTGAGAGACTTTCGTCTCTCGTCTCGCGAGCGCTCGTCAAA

GACTTTGTTAACTTTGTTTTAGTGTCTTTCGAACAATCATGTAATAATTAAAACTTTCAA

CAACGAATCT

>ASV545 GS|80.4|AY667583|SH0954634.09FU;k:Fungi,p:Ascomycota,c:Lecanoromycetes,o:Caliciales,f:Caliciaceae,g:Buellia,s:Buellia frigida

AAGTCGTAACAAGATTTTCGTAGATGAATTTACGAAAAAATCATTATCGAAAGACGAAAT

CGCTTCGATTTCACTCTTCACTCGTGTTTACTTATCTTTGTTGCTTTAACGAGTTTTCGA

ACTTGATCGTTCGACGCGAATGAGAGACTTTCGTCTCTCGTTTCGCGAGCGTTCGTCAAA

AATCTTGTTAACTTTGTTTTAATGTCTTTCGAGCAATCATGTAATAGTTAAAACTTTCAA

CAACGAATTT

>ASV546 SS|0.8700|KF823589|SH1091255.09FU;k:Fungi,p:Basidiomycota,c:Tremellomycetes,o:Tremellales

AAGTCGTAACAAGGTTTCCGTAGGTGAACCTGCGGAAGGATCATTAGTGAATGGCCTCCG

GGCTCTTTCGTTAATCCACACACCTCTGTGAACCTGTCGGCCTCCTGGCCTTCTTTTCCA

AACAATGTGTAACGAACGTAAGGCATACGATTAACCTAGTAAAACTTTCAACAACGGATC

TCTTGGCTCTC

>ASV547 SS|0.8400|AY667583|SH0954634.09FU;k:Fungi,p:Ascomycota,c:Lecanoromycetes

AAGTCGTAACAAGGTTTCCGTAGGTGAACCTGCGGAAGGATCATTACCGAGAGACGGGGT

CGCTGCGGCCCCACTCTTCACCCGTGTATACATACCTTTGTTGCTTTGGCGCGCCCTCGG

GCCTGACCGTCTGGCGCTGGTAGGGGGGTCCCGCCCCTCGCCCAGCGAGCGCCGGCCAAA

GACCCCTAAAATTCTGTCTTCCATGTCGTCCGAGCAACCATATAATAGTTAAAACTTTCA

ACAACGGATC

>ASV548 GS|81.4|MK970687|SH0954634.09FU;k:Fungi,p:Ascomycota,c:Lecanoromycetes,o:Caliciales,f:Caliciaceae,g:Buellia,s:Buellia frigida

AAATCGTAACAAGATTTTCGTAGATGAATTTACGAAAGAATCATTATTGAGAGACGAAAT

CGCTTCGATCTCACTCTTCACTCGTATTTATTTATCTTTGTTGCTTTGACGAATCTTCGA

ACTTGATCATTCGACGCGAGTGAGAGACTTTCGTCTCTCGTCTCGCGAGCGCTCGTCAAA

GATTCTATTAACTTTATTTTAGTGTCTTTCGAACAATCATGTAATAGTTAAAACTTTCAA

CAACGAATCT

>ASV549 SS|1.0000|UDB0700338|SH1084495.09FU;k:Fungi

AAGTCGTAACAAGGTCTCCGTAGGTGAACCTACGGAGGGATCATTACCGAGTAAGGGCCT

CCGGGCTCGACCTCCAACCCTTTGTCGTACCCACTACAGTTGCCGCGGGGGCGACCCGGC

CTCCGCGTCGGGGCCCCCGGTGGACCCAACTAAACAACTCTGCGTCTTTGCGTCTGAGTA

TTAAAGTAAATCAATTAAAACTTTCAACAACGGATCTCTTAGTTCTA

>ASV550 SS|0.9300|MZ229889|SH0913114.09FU;k:Fungi,p:Ascomycota,c:Lecanoromycetes

AAGTCGTAACAAGGTTTCCGTAGGTGAATCTGCGGAAGAATCATTATCGAGAGACGGAGT

TTAACCGGCCCCACTCTTCACCCGTGTATACCTACCCTTTGTTGCTTTGGCGGGCGGTCG

GTCTTTACCGTCCGACGTCGGTCGGAAGGCTCGCGCCTTCCACTCACCGAACGCCCGCCA

GAGGCTCTCAAAAGCCGTCCATCTGTGTCCGAGCGAGTGAAGCAAATAGTTAAAAACTTT

CAACAACGGA

>ASV551 GS|84.0|AY667583|SH0954634.09FU;k:Fungi,p:Ascomycota,c:Lecanoromycetes,o:Caliciales,f:Caliciaceae,g:Buellia,s:Buellia frigida

AAGTCGTAACAAGATTTTCGTAGATGAATTTGCGAAAGAATCATTATCGAGAGACGAAAT

CGCTTCGATCTCACTCTTCACTCGTATTTACTTATCTTTGTTGCTTTGACGAGTCTTCGA

GCTTGATCGTTCGACGCGAGTGAGAGACTTTTGTTTCTCGTCTCGCGAGCGTTCGTCAAA

GACTTTGTTAACTTTGTTTTAATGTCTTTCGAGCAATCATGTAATAGTTAAAACTTTCAA

CAACGGATCT

>ASV552 GS|97.6|MF138060|SH0954356.09FU;k:Fungi,p:Ascomycota,c:Lecanoromycetes,o:Acarosporales,f:Acarosporaceae,g:Acarospora,s:Acarospora gwynnii

AAGTCGTAACAAGGTTTCCGTAGGTGAACCTGCGGAAGGATCATTACAGAGTTAGGGTCT

TTCCAGGCCCGACCTCCAACCCTATGTGTACCTACCTTTGTTGCTTTGGCGGGCCCGTTG

GGTGACCCACCGGTGGCCTCTGGCTCCCGAGTGCCCGTCAGAGACCCATCAAAACCTGTT

AATTGTGTCGTCTGAGTACCACTTTAATAATTAAAACTTTCAACAACGGATCTCTTGGTT

CTG

>ASV553 SS|0.8200|AF250779|SH0954634.09FU;k:Fungi,p:Ascomycota,c:Lecanoromycetes,o:Caliciales,f:Caliciaceae,g:Buellia,s:Buellia frigida

AAGTCGTAACAAGGTTTTCGTAGATGAATTTGCGAAAGAATCATTATCGAGAGACGAGGT

CGCTTCGGCCTCACTCTTCACTCGTGTCTACTTATCTTTGTTGCTTTGACGGGCCTTCGG

GCTTGATCGTTCGACGCGGGTGGAAGGCTTTCGCCTCTCGTCTCGCGAGCGCTCGTCAAA

GGCCCTATTAACTTTGTTTTAGTGTCTTTCGAGCAATCATGTAATAGTTAAAACTTTCAA

CAACGAATCT

>ASV555 GS|78.4|AY667583|SH0954634.09FU;k:Fungi,p:Ascomycota,c:Lecanoromycetes,o:Caliciales,f:Caliciaceae,g:Buellia,s:Buellia frigida

AAGTCGTAACAAGATTTTCGTAAATGAATTTACGAAAGAATCATTATCGAAAGACGAAAT

CGCTTCGATCTCACTCTTCACTTGTATTTACTTATCTTTGTTACTTTGACGAATCTTCAA

ACTTGATCGTTCGACGCGAGTGAGAGACTTTCGTCTCTCGTTTTGCGAACGTTCGTCAAA

GATTTTGTTAATTCTATTTTAATGTCTTTCGAGTAATCATGTAATAGTTAAAACTTTCAA

TAACGAATCT

>ASV556 GS|83.3|AF250779|SH0954634.09FU;k:Fungi,p:Ascomycota,c:Lecanoromycetes,o:Caliciales,f:Caliciaceae,g:Buellia,s:Buellia frigida

AAGTCGTAACAAGATTTTCGTAAATGAATTTACGAAAGAATCATTATCGAAAGACGAGAT

CGCTTCGACTTCACTCTTTACTCGTGTCTACTTATCTTTGTTGCTTTGACGAATCTTTGA

GCTTGATCGTTCGACGCGAGTGAGAGACTTTCGTCTCTCGTCTCGCGAGCGTTCGTCAAA

GATCTTGTTAACTCTATTTTAGTGTCTTTCGAGCAATCATGTAATAGTTAAAACTTTCAA

CAACAAATTT

>ASV557 GS|99.5|JN873879|SH1123218.09FU;k:Fungi,p:Ascomycota,c:Lecanoromycetes,o:Lecanorales,f:Lecanoraceae,g:Lecanora,s:Lecanora physciella

AAGTCGTAACAAGGTTTCCATAGGTGAACCTGCGGAAAGATCATTACCGAGAGCGGGGCT

AACCCCCCAAACTCCGCCGCCGAAAGGGGTACTCTCCACCCTATGTATACATATACCACT

CTCGCTTTGGCGGGCTGAAGGCTCTTGCCCTACGCCCGCCAGTGGCTCAAAAAATTCTGT

TCATCAGTGATGTCCGAGTAAAAACCTAATAGTTAAAACTTTCAACAACGGATCTCTTGG

TTCTG

>ASV558 GS|99.6|AY603122|SH0998157.09FU;k:Fungi,p:Ascomycota,c:Lecanoromycetes,o:Umbilicariales,f:Umbilicariaceae,g:Umbilicaria,s:Umbilicaria decussata

AAGTCGTAACAAGGTTTCCGTAGGTGAACCTGCGGAAGGATCATTACTGAGATAGGGTCC

TCCGGGCCCGAACCTCCAACCCTTTGCGTACCTTACCTTTGTTGCTTTGGCGGGCCCGCT

GGGGAAACCCACCGCCGGCGTTGAGCCGGTGAGCGCCCGCCGGAGGCCCCCAAAACTCCG

TCTTGTCAGTGTCGTCTGAGTACTATACAATAGCTAAAACTTTCAACAACGGATCTCTTG

GTTCTG

>ASV559 GSL|100.0|OK041528|SH1309305.09FU;k:Fungi,p:Ascomycota,c:Dothideomycetes,o:Capnodiales,f:Cladosporiaceae,g:Cladosporium

AAGTCGTAACAAGGTCTCCGTAGGTGAACCTGCGGAGGGATCATTACAAGAACTTCCGGG

CTTCGGCCTGGTTATTCATAACCCTTTGTTGTCCGACTCTGTTGCCTCCGGGGCGACCCT

GCCTTCGGGCGGGGGCTCCGGGTGGACACTTCAAACTCTTGCGTAACTTTGCAGTCTGAG

TAAACTTAATTAATAAATTAAAACTTTTAACAACGGATCTCTTGGTTCTG

>ASV560 SS|0.8900|MZ229889|SH0913114.09FU;k:Fungi,p:Ascomycota,c:Lecanoromycetes

AAGTCGTAACAAGGTTTCCGTAGGTGAACCTGCGGAAGGATCATTATCGAGAGACGGAGT

CTAACCGGCCCCACTCTTCACCCGTGTATACCTACCCTTTGTTGCTTTGGCGGGCGGTCG

GTCTTTACCGCCCGACGTCGGTCGGAAGGCTCGCGCCTTCCACTCACCGAGCGCCCGCCA

GAGGCCCCCAAAAGCCGTCCATCTGTGTCCGAGCGAGTGAAGCAAATAGTTAAAAACTTT

CAACAACGGA

>ASV561 GS|89.7|AF250779|SH0954634.09FU;k:Fungi,p:Ascomycota,c:Lecanoromycetes,o:Caliciales,f:Caliciaceae,g:Buellia,s:Buellia frigida

AAGTCGTAACAAGATTTTCGTAGATGAATCTACGAAAGAATCATTATCGAGAGACGAGAT

CGCTTCGACCCCACTCTTCACTCGTGTCTACTTATCTTTGTTGCTTTGACGAGCCTTCGA

ACTTGATCGTTCGACGCGAGTGAGAGGCTTTCGTCTCTCGTCTCGCGAGCGCTCGTCAAA

GGCCCTGTTAACTCTATTTTAGTGTCTTTCGAGCAATCATGTAATAGTTAAAACTTTCAA

CAACGAATCT

>ASV562 GS|76.5|DQ534454|SH0954616.09FU;k:Fungi,p:Ascomycota,c:Lecanoromycetes,o:Caliciales,f:Caliciaceae,g:Buellia,s:Buellia russa

AAGTCGTAACAAGATTTTCGTAAATGAACTTGCGAAAGAATCATTATCGAGAGACGAGAT

CGCTTCGATTTCACTCTTTATTCGTGTTTACTTATTTTTGTTGCTTTGACGAGCTTTCGA

GCTTGATCGTTCAACGCGAGTGAGAGACTTTCGTCTCTCGTTTCGCGAACGCTCGTCAAA

GATTTTGTTAATTTTGTTTTAATGTCTTTCGAGCAATCATGTAATTGTTAAAACTTTTAA

CAACGAATCT

>ASV563 GS|89.7|AF250779|SH0954634.09FU;k:Fungi,p:Ascomycota,c:Lecanoromycetes,o:Caliciales,f:Caliciaceae,g:Buellia,s:Buellia frigida

AAGTCGTAACAAGGTTTTCGTAGATGAATTTGCGAAAGAATCATTATCGAGAGACGGGGT

CGCTTCGGCCCCACTCTTCACTCGTGTCTACTTATCTTCGTTGCTTTGGCGAGCCTTCGA

GCTTGATCGTTCGACGCGAGTGAAAGGCTTTCGCCTCTCGTCTCGCGAGCGCTCGTCAAA

GATTCTATTAACTCTGTTTTAGTGTCTTTCGAACAACCATGTAATAGTTAAAACTTTTAA

CAACGAATCT

>ASV564 GS|100.0|UDB01571335|SH0954626.09FU;k:Fungi,p:Ascomycota,c:Lecanoromycetes,o:Caliciales,f:Caliciaceae,g:Buellia

AAGTCGTAACAAGGTTTCCGTAGGTGAACCTGCGGAAGGATCATTACCGAGAGACGGGGT

CGCTTCGGCCCCACTCTTCACCCGTGTCTATTTATCTTTGTTGCTTTGGCGGGCCTTCGG

GCTTGATCGTTCGACGCTGGCTGGGGGTTAGCCTCGCGCCCAGCGAGCGCCTGCCAAAGA

CCTTGTTAACTCTGTTCTTAGTGTCCTCTGAGCAACCACATAATAGTTAAAACTTTCAAC

AACGGATCTC

>ASV565 SS|0.9000|MZ229889|SH0913114.09FU;k:Fungi,p:Ascomycota,c:Lecanoromycetes

AAGTCGTAACAAGGTTTCCGTAGGTGAACCTGCGGAAGGATCATTATCGAGAGACGGAGT

CTAACCGGCCCCACTCTTCACCCGTGTATACCTACCCTTTGTTGCTTTGGCGGGCGGTCG

GTCTTTACCGCCCGACGTCGGTCGGAAGGCTCGCGTCTTCCACTCACCGAACGCCCGCCA

GAGGCCCCCAAAAGCCGTCCATCTGTGTTCGAGCGAGTGAAGCAAATAGTTAAAAACTTT

CAACAACGGA

>ASV566 GS|86.3|AF250779|SH0954634.09FU;k:Fungi,p:Ascomycota,c:Lecanoromycetes,o:Caliciales,f:Caliciaceae,g:Buellia,s:Buellia frigida

AAGTCGTAACAAGATTTTCGTAGATGAATTTGCGAAAGAATCATTATCGAGAGACGAGGT

CGCTTCGATCTCACTCTTCATTCGTGTCTACTTATCTTTGTTGCTTTGACGAGTCTTCGA

GCTTGATCGTTCGACGCGAGTGAGAGACTTTCGCCTCTCGTCTCGCGAGCGCTCGTCAAA

GATTTTGTTAACTCTATTTTAGTGTCTTTCAAGCAATCATGTAATAGTTAAAACTTTCAA

CAACGAATCT

>ASV567 GS|99.1|AF279772|SH1300522.09FU;k:Fungi,p:Ascomycota,c:Lecanoromycetes,o:Teloschistales,f:Teloschistaceae,g:Xanthoria,s:Xanthoria elegans

CTTTTTCCTTTCTCTTCCTTCCCCTTCTTCCCCCGGTTTCCGTAGGTGAACCTGCGGCAG

GATCATTACTACGAGAGGGATGTACGCTTCCAGCCGAGTCCCGGGGGGCTGCGCCCCTCA

CCTCTTCAACCCTGTGTCTACCAACCGCTGTTGCTTCGGCGAGCGTCGGGGCGTCCGCGC

CCCGGCCCCGGCTTCGGTCGGTGAGCTCTCGCAGAGGCCTATCTTTATTCTGTTTTGCAG

TGACGTCCGA

>ASV568 GS|100.0|UDB01890890|SH1188820.09FU;k:Fungi,p:Basidiomycota,c:Tremellomycetes,o:Filobasidiales,f:Filobasidiaceae,g:Naganishia

AAGTCGTAACAAGGTTTCCGTAGGTGAACCTGCGGAAGGATCATTAATGAATACAGATGC

CTGTCGAGCTTGCTCACGGGCTTTCTATCATATCCATAACACCTGTGCACTTGTTGGATG

TTCTAGAGACTTAGGGTTAAACCTGCAGTTAATAGTCATCCACTTACACTAAACAATAAT

GTAACAAATGTAGTCTTATTATAACAAAATAAAACTTTCAACAACGGATCTCTTGGCTCT

C

>ASV569 GS|82.4|AY667583|SH0954634.09FU;k:Fungi,p:Ascomycota,c:Lecanoromycetes,o:Caliciales,f:Caliciaceae,g:Buellia,s:Buellia frigida

AAGTCGTAACAAGATTTTCGTAGATGAATTTACGAAAAGATCATTATCGAGAGACAAAGT

CGCTTCGATCTCACTCTTCATTCGTATCTACTTATCTTTGTTGCTTTGACGAGTTTTCGA

GCTTGATCGTTCGACGCGAGTGAGAGACTTTCGTCTCTCGTCTTGCGAACGTTCGTCAAA

GACTTTGTTAACTTTGTTTTAATGTCTTTCAAGCAATCATGTAATAGTTAAAACTTTCAA

CAACGAATCT

>ASV570 GS|0.0|None;No hit

GTGTCAGCAGCCGCGGTAATACGGAGGATCCAAGCGTTATCCGGATTTATTGGGTTTAAA

GGGTGCGTAGGCGGCCTGTTAAGTCAGGGGTGAAATTTTCCGGCTCAACCGGGACATTGC

CTTTGATACTGACGGGCTTGAATGCAGCTGAGGTAGGCGGAATGTGACAAGTAGCGGTGA

AATGCATAGATATGTCACAGAACACCAATTGCGAAGGCAGCTTACTAAAGTGTGATTGAC

GCTGAGGCAC

>ASV571 GS|71.6|AM901805|SH0999915.09FU;k:Fungi,p:Basidiomycota,c:Tremellomycetes,o:Tremellales,f:Bulleribasidiaceae,g:Vishniacozyma

AAGTCATAACAAGGTTTCCGTAGGTGAACCTGCGGAAGGATCATTCGAGAATAGGGCCTC

TGGCCTTAATCCAAAACCCACATCGTGAACCGTGCCTTGGGACCTCGCAAGGGGGACCTT

GGATCACACTTTATAACTGAGTCTTGAAAGGAAGAATATGACAAACAGAAAATAACTTTT

AACAACGGATCTCTTGGCTCTC

>ASV572 SS|1.0000|AF276067|SH0954634.09FU;k:Fungi,p:Ascomycota,c:Lecanoromycetes,o:Caliciales,f:Caliciaceae,g:Buellia,s:Buellia frigida

AAGTCGTAACAAGATTTCCGTAGGTGAACCTGCGAAAGGATCATTATCGAGAGACGGGGT

CGCTTCGACCCCACTCTTCACCCGTGTCTACTTACCTTTGTTGCTTTGACGAGCCTTCGA

GCTTGACCGTTCGACGCGAGTGGGAGGCTTTCGCCTCTCGCCTCGCGAGCGCCCGCCAAA

GGCCCTGTTAACTCTGTTTTAGTGTCTTCCGAGCAACCATGTAATAGTTAAAACTTTCAA

CAACGAATCT

>ASV573 SS|1.0000|AY667583|SH0954634.09FU;k:Fungi,p:Ascomycota,c:Lecanoromycetes,o:Caliciales,f:Caliciaceae,g:Buellia,s:Buellia frigida

AAGTCGTAACAAGGTTTTCGTAGGTGAACCTGCGGAAGGATCATTATCGAGAGACGGGGT

CGCTTCGGCCCCACTCTTCACTCGTGTCTACTTACCTTTGTTGCTTTGGCGGGCCTTCGG

GCTTGATCGTTCGACGCGGGTGGGAGGCTTTCGCCTGTCGCCTCGCGAGCGCTCGTCAAA

GGCCCTGTTAACTCTGTTTTAGTGTCTTCCGAGCAACCATGTAATAGTTAAAACTTTCAA

CAACGAATCT

>ASV574 SS|0.9800|AY667583|SH0954634.09FU;k:Fungi,p:Ascomycota,c:Lecanoromycetes,o:Caliciales,f:Caliciaceae,g:Buellia,s:Buellia frigida

AAGTCGTAACAAGGTTTTCGTAGGTGAATTTGCGGAAGGATCATTATCGAGAGACGGGGT

CGCTTCGACTTCACTCTTCACTCGTGTCTACTTACCTTTGTTGCTTTGGCGGGTCTTCGA

GCTTGATCGTTCGACGTGGGTGGGAGGCTTTCGCCTGTCGTCTCGCGAGCGCTCGCCAAA

GGCCCTGTTAACTTTGTTTTAGTGTCTTTCGAGCAATCATGTAATAGTTAAAACTTTCAA

CAACGAATCT

>ASV575 GS|84.4|AY667583|SH0954634.09FU;k:Fungi,p:Ascomycota,c:Lecanoromycetes,o:Caliciales,f:Caliciaceae,g:Buellia,s:Buellia frigida

AAGTCGTAACAAGATTTTCGTAGATGAATTTGCGAAAGAATCATTATCGAGAGACGAGAT

CGCTTTGGTCTCACACTCTTCACTCGTATCTACTTATCTTTGTTGCTTTGACGAGTCTTC

GAGCTTAATCGTTCGACGCGAGTGAAAGGCTTTCGTCTCTCGTTTCGCGAGCGCTCGTCA

AAGGCTCTGTTAACTCTATTTTAGTGTCTTTCGAGCAATCATGTAATATTTAAAACTTTC

AATAACGAAT

>ASV576 GS|87.3|AF250779|SH0954634.09FU;k:Fungi,p:Ascomycota,c:Lecanoromycetes,o:Caliciales,f:Caliciaceae,g:Buellia,s:Buellia frigida

AAGTCGTAACAAGGTTTTCGTAGATGAATCTACGAAAGAATCATTATCGAGAGACGAGAT

CGCTTCGATCTCACTCTTCACTCGTGTCTACTTATCTTTGTTGCTTTGACGAGTCTTCGA

GCTTGATCGTTCGACGCGTGAGAAGGCTTTCGCCTCTCGCCTCGCGAGCGCTCGTCAAAG

GTCCTATTAACTCTATTTTAGTGTCTTTCGAGCAATCATGTAATAATTAAAACTTTCAAC

AACGAATCTC

>ASV577 GS|83.6|AY667583|SH0954634.09FU;k:Fungi,p:Ascomycota,c:Lecanoromycetes,o:Caliciales,f:Caliciaceae,g:Buellia,s:Buellia frigida

AAGTCGTAACAAGATTTTCGTAGATGAATTTGCGAAAGAATCATTATCGAGAGACGAAAT

CGCTTCGACTTCACTCTTCACTCGTATCTACTTATCTTTGTTGCTTTGACGAGTCTTCGA

GCTTGATCATTCGATGCGAGTGAGAGACTTTCGTCTCTCGTTTCGCGAGCGCTCGTCAAA

GATCTTGTTAACTTTGTTTTAATGTCTTTCGAGCAATCATGTAATAGTTAAAACTTTCAA

CAATGAATCT

>ASV578 GS|98.8|AY667583|SH0954634.09FU;k:Fungi,p:Ascomycota,c:Lecanoromycetes,o:Caliciales,f:Caliciaceae,g:Buellia,s:Buellia frigida

AAGTCGTAACAAGGTTTCCGTAGGTGAACCTGCGGAAGGATCATTACCGAGAGACGGGGT

CGCTTCGGCCCCACTCTTCACCCGTGTCTACTTACCTTCGTTGCTTTGGCGGGCCTTCGG

GCTTGACCGTTCGACGCGGGTGGGAGGCTTTCGCCTCTCGCCTCGCGAGCGCCCGCCAAA

GGCCCTGTTAACTCTGTTTTAAGTGTCTTCCGAGCGACCATGTAATAGTTAAAACTTTCA

ACAACGGATC

>ASV579 SS|1.0000|UDB01571642|SH1041881.09FU;k:Fungi

AAGTCGTAACAAGGTTTCCGTAGGTGAACCTGCGGAAGGATCATTAATGTAACCCCCCGA

TCGGCTCACGCCGTGGTGGGAAATACAAATCCACATACCTCTGTGGACCGTTGACCTCCG

GGTCGTCTTTACAAACATCAGTGTAATGAACGTATACAAACATAAACAAAACAAAACTTT

CAACAACGGATCTCTTGGCTCTC

>ASV580 SS|0.8900|KF297104|SH1316584.09FU;k:Fungi

AAGTCGTAACAAGGTTTCCGTAGGTGAACCTGCGGAAGGATCATTAGCGAATCGGAACGG

CGGCCTCCCGGGGCCCGCCTTCTCTTCGTCACCCTGTGCACCGTGCCCCTTTTTCAAACG

CCGTACCCGTCGAGGATGTCAACCAATAACGAAAGGCACAAAATGAAGCAACGGATCTCT

TGGCTCTC

>ASV581 SS|0.8600|AF281306|SH1300522.09FU;k:Fungi,p:Ascomycota,c:Lecanoromycetes,o:Teloschistales,f:Teloschistaceae

AAGTCGTAACAAGGTTTCCGTAGGTGAACCTGCGGAAGGATCATTACTAAGAGAGGGATG

TACGCTTCCAGCCGAGTCCCGGGGGGCTGCGCCCCTCACCTCTTCAACCCTGTGTCTACC

AACCGCTGTTGCTTCGGCGAGCGTCGGGGCGTCCGCGCCCCGGCCCCGGCTTCGGTCAGT

CAGCCCTCGTCAGAGGCCCATCCAAATTCTGTTTCAGTGACGTCCGAGTATACCAGCAAA

TAAATTAAAA

>ASV582 GS|99.6|HQ650628|SH0913122.09FU;k:Fungi,p:Ascomycota,c:Lecanoromycetes,o:Caliciales,f:Caliciaceae,g:Buellia,s:Buellia frigida

AAGTCGTAACAAGGTTTCCGTAGGTGAACCTGCGGAAGGATCATTACCGAGAGACGGGGT

CGAACCGGCCCCACTCTTCACCCGTGCCTACCTACCTATGTTGCTTTGGCGGGCCTCCTG

CTCGGCGTCGGTCCGAGGCCTTTCGTGCCTCTTCCCGGCGAGTGCCCGCCGAAGGCTCTG

CAAACTCTTGTTCAGTGTCGTCCGAGTAACAACCTAATAGTTAAAACTTTCAACAACGGA

TCTCTTGGTT

>ASV583 GS|86.8|AF250779|SH0954634.09FU;k:Fungi,p:Ascomycota,c:Lecanoromycetes,o:Caliciales,f:Caliciaceae,g:Buellia,s:Buellia frigida

AAGTCGTAACAAGATTTTCGTAGATGAATTTGCGAAAGAATCATTATCGAGAGACGAGAT

CGCTTCGATCTCACTCTTCATTCGTATCTACTTATCTTTGTTGCTTTGACGAGTCTTCGA

GCTTGATCGTTCGACGCGAGTGAGAGGCTTTCGCCTCTCGTCTCGCGAGCGCTCGTCAAA

GATCTTGTTAACTCTATTTTAGTGTCTTTCGAGCAATCATGTAATAGTTAAAACTTTCAA

CAACGAATCT

>ASV584 SS|0.8300|MZ229889|SH0913114.09FU;k:Fungi,p:Ascomycota,c:Lecanoromycetes,o:Caliciales

AAGTCGTAACAAGGTTTCCGTAGGTGAACCTGCGGAAGGATCATTATCGAGAGACGGAGT

CTAACCGGCCCCACTCTTCACCCGTGTATACCTACCCGTTGTTGCTTTGGCGGGCGGTCG

GTCTTTACCGCCCGACGTCGGTCGGAAGGCTCGCGCCTTCCACTCACCGAACGCCCGCCA

GAGGCCCCCAAAAGCCGTCCATCTGTGTCCGAGCGAGTGAAGCAAATAGTTAAAAACTTT

CAACAACGGA

>ASV585 SS|0.8400|UDB01765780|SH0980138.09FU;k:Fungi,p:Ascomycota,c:Eurotiomycetes

AAGTCGTAACAAGGTTTCCGTAGGTGAACCTGCGGAAGGATCATTATCGAGCAAGGGTCC

TCCGGGGCCCGACCTCCCAACCCTGTGTCTACCGCACCACGTTGCTTTGGCGGGCCCGTC

CACTGGGCCGCCGGCGGTCCCTCACCGGACCCCTGGCCCGCGCCCGCCAACGGCCCCACA

TCAAAACCGAACAGAACCGTGACCGTCTCAGTTAGAATATAAAATGACCAAAACTTTCAA

CAACGGATCT

>ASV586 SS|1.0000|AY667583|SH0954634.09FU;k:Fungi,p:Ascomycota,c:Lecanoromycetes,o:Caliciales,f:Caliciaceae,g:Buellia,s:Buellia frigida

AAGTCGTAACAAGGTTTTCGTAGGTGAACCTGCGGAAGGATCATTATCGAGAGACGGGGT

CGCTTCGGTCTCACTCTTCACTCGTGTCTACTTACCTTTGTTGCTTTGGCGGGCCTTCGG

GCTTGATCGTTCGACGTGGGTGGGAGGCTTTCGCCTCTCGCCTCGCGAGCGCTCGTCAAA

GGCTCTGTTAACTCTGTTTTAGTGTCTTTCGACCAATCATGTAATAGTTAAAACTTTCAA

CAACGGATCT

>ASV587 GS|85.8|AF250779|SH0954634.09FU;k:Fungi,p:Ascomycota,c:Lecanoromycetes,o:Caliciales,f:Caliciaceae,g:Buellia,s:Buellia frigida

AAGTCGTAACAAGATTTTCGTAAATGAATCTACGAAAGAATCATTATCGAGAGACGAGAT

CGCTTCGACCTCACACTCTTCACTCGTGTCTACTTATCTTTGTTGCTTTGACGAGTCTTC

GAGCTTGATCGTTCGACGCGAGTGAGAGATTTTCGTCTCTCGTCTCGCGAGCGCTCGTCA

AAGGCTCTATTAACTTTGTTTTAGTGTCTTTCGAGCAATCATGTAATATTTAAAACTTTC

AACAACGAAT

>ASV588 SS|0.8000|MF138060|SH0954356.09FU;k:Fungi,p:Ascomycota,c:Lecanoromycetes,o:Acarosporales,f:Acarosporaceae,g:Acarospora

AAGTCGTAACAAGGTTTCCGTAGGTGAACCTGCGGAAGGATCATTAATGAGATAGGGTCC

TTCGGGGCCCGAACCTCCAACCCTCTGTGTATCTTACCTTTGTTGCTTTGGCGGGCCCGT

TGGGTGACCCACCGGTGGCCTCTGGCTCCCGAGTGCCCGTCAGAGACCCATCAAAACCCG

TTAATTGTGTCGTCTGAGTACCACTTTAATAATTAAAACTTTCAACAACGGATCTCTTGG

TTCTG

>ASV589 GS|100.0|AF250779|SH0954634.09FU;k:Fungi,p:Ascomycota,c:Lecanoromycetes,o:Caliciales,f:Caliciaceae,g:Buellia,s:Buellia frigida

CTTTTTCCTTTCTCTTCCTTCCCCTTCGTCCCCAGGTTTCCGTAGGTGAACCTGCGGAAG

GATCATTACCGAGAGACGGGGTCGCTTCGGCCCCACTCTTCACCCGTGTCTACTTACCTT

TGTTGCTTTGGCGGGCCTTCGGGCTTGACCGTTCGACGCGGGTGGGAGGCTTTCGCCTCT

CGCCTCGCGAGCGCCCGCCAAAGGCCCTGTTAACTCTGTTTTAGTGTCTTCCGAGCAACC

ATGTAATAGT

>ASV590 SS|1.0000|AF250779|SH0954634.09FU;k:Fungi,p:Ascomycota,c:Lecanoromycetes,o:Caliciales,f:Caliciaceae,g:Buellia,s:Buellia frigida

AAGTCGTAACAAGGTTTTCGTAGGTGAATCTGCGAAAGGATCATTATCGAGAGACGAGGT

CGCTTCGGCCTCACTCTTCACTCGTGTCTACTTATCTTTGTTGCTTTGGCGGGCCTTCGG

GCTTAATCGTTCGACGCGGGTGGGAGGCTTTCGCCTCTCGCCTCGCGAGCGCTCGCCAAA

GGCCTTGTTAACTTTGTTTTAGTGTCTTTCGAGCAACCATGTAATAGTTAAAACTTTCAA

CAACGAATCT

>ASV591 GS|80.0|AY667583|SH0954634.09FU;k:Fungi,p:Ascomycota,c:Lecanoromycetes,o:Caliciales,f:Caliciaceae,g:Buellia,s:Buellia frigida

AAGTCGTAACAAGATTTTCGTAAATGAACTTATGAAAAAATCATTATCGAAAGACGAAAT

CGCTTCGATTTCATTCTTCATTCGTATCTACTTATCTTTGTTGCTTTGACGAGTCTTCGA

GCTTGATCGTTCGATGCGAGTGAGAGACTTTCGTCTCTCGTTTTGCGAACGCTCGTCAAA

GATCGTATTAACTCTATTTTAGTGTCTTTCGAGCAATCATGTAATAATTAAAACTTTCAA

CAACGAATTT

>ASV593 GS|100.0|AF278752|SH0934140.09FU;k:Fungi,p:Ascomycota,c:Lecanoromycetes,o:Caliciales,f:Physciaceae,g:Phaeophyscia

AAGTCGTAACAAGGTTTCGCCCGTCACGGAAGCCTTGCACCACCAGTCTGCTCCCCGGAG

CGGGCAACGCTTGATTGATGCTGGGAGAACGCCTCGCCCGGGGCCTAACCAGCAGCCGTC

GTGCCTAGCACTTCGGTCCACAGATCATCTTCAGGCGGTCCTCAGGGATCAAGATATGAC

CGGAACCGCCCGTCACAGGGTGGGAGCCTCGTTCCGTAGGTGGGCAATTCGCCTACCCAA

GCCCCAGCAG

>ASV594 GS|100.0|AM279269|SH1006616.09FU;k:Fungi,p:Ascomycota,c:Saccharomycetes,o:Saccharomycetales,f:Dipodascaceae,g:Yarrowia,s:Yarrowia alimentaria

AAGTCGTAACAAGGTTTCCGTAGGTGAACCTGCGGAAGGATCATTATGAATATATACATT

ATTGTGGAATTACTTATATCTAACTAATCTATTTTTTAATTACAACTATCAACAACGGAT

CTCTTGGCTCTC

>ASV595 GS|0.0|None;No hit

AAGTCGTAACAAGATTTCCGTAAATGAATTTGCGAAAGAATCATTATCGAGAAACGGAGT

TTAACCGATCCCACTCTTCACTCGTGTATATTTACTCTTTATTGTTTTAACGGGCGATCG

GTCTTTATCGCTCGACGTCGGTCGGAAAACTCGCGTCTTTCACTCACCGAACGCTCGTTA

AAGATTCTCAAAATCCGTTCATTTATGTTCGAGCGAATGAAATAAATAATTAAAAACTTT

CAACAACGGA

>ASV596 GS|84.8|AY667583|SH0954634.09FU;k:Fungi,p:Ascomycota,c:Lecanoromycetes,o:Caliciales,f:Caliciaceae,g:Buellia,s:Buellia frigida

AAGTCGTAACAAGATTTTCGTAGATGAACTTGCGAAAGAATCATTATCGAGAGACGAAAT

CGCTTCGACCTCACTCTTCACTCGTGTCTATTTATCTTTGTTGCTTTGACGAGTCTTCGA

GCTTGATCGTTCGACGCGAGTGAGAGACTTTCGTCTCTCGTCTCGCGAGCGTTCGTCAAA

GACTCTATTAACTTTGTTTTAGTGTCTTTCGAACAATCATGTAATAGTTAAAACTTTTAA

CAACGAATTT

>ASV597 SS|0.8300|MN592663|SH1107827.09FU;k:Fungi,p:Ascomycota,c:Lecanoromycetes,o:Teloschistales,f:Teloschistaceae

AAGTCGTAACAAGGTTTCCGTAGGTGAACCTGCGGAAGGATCATTATCGAGAGGGGGGGC

TCTATGCCCCGGGGCTCTGTCCCCGTACCTTTTCACCCTGTGTGTATTTTTCCCCCGTTG

CTTTGGCGGGCCCCGGGTCTTCCCCCGGCGTTGGCCCCCTCGCGGGGTTCGCGAGCGCCC

GCCGGAAGGCTTATCGAAACTCTAATCAGTGCAGTCTGAGCGTACGAATAATAAATTAAA

ACTTTTAATA

>ASV598 GS|83.8|AF250779|SH0954634.09FU;k:Fungi,p:Ascomycota,c:Lecanoromycetes,o:Caliciales,f:Caliciaceae,g:Buellia,s:Buellia frigida

AAGTCGTAACAAGATTTTCGTAAATGAACTTGCGAAAAAATCATTATCGAGAAACGAGAT

CGCTTCGATCTCACTCTTCACTCGTATCTACTTATCTTTGTTGCTTTGACAAGTCTTCGA

GCTTGATCGTTCGACGCGAATGAGAGACTTTCGTCTCTCGTCTTGCGAGCGCTCGTCAAA

GACTCTATTAACTCTATTTTAGTGTCTTTCGAGCAATCATGTAATAGTTAAAACTTTCAA

TAACGAATCT

>ASV599 SS|1.0000|FR682176|SH1068012.09FU;k:Fungi

AAGTCGTAACAAGGTTTCCGTAGGTGAACCTGCGGAAGGATCATTACTGAGATAGGGTCC

TCCGGGGCCCGACCTCCAACCCTTTGTCTACCTTACCACTGTTGCCTCGGCGGGTCGCCG

GCGCTCTCACGGGCGTCGCCGTCGGCCTCCGCACTCTCGAGTGCCCGCCGCTAGGCCCCC

TAAATCCTTTATAAATCAGTCTTCTGAGAGTATATTCAATACTTAAAACTTTCAACAACG

GATCTCTTGG

>ASV600 SS|0.8300|AY667583|SH0954634.09FU;k:Fungi,p:Ascomycota,c:Lecanoromycetes,o:Caliciales,f:Caliciaceae

AAGTCGTAACAAGGTTTCCGTAGGTGAACCTGCGGAAGGATCATTACCGAGAGACGGGGT

CGCTTCGGCCCCACTCTTCACCCGTGTATACATACCTTTGTTGCTTTGGCGCGCCCTCGG

GCCTGACCGTCTGGCGCTGGTAGGGGGGTCCCGCCCCTCGCCCAGCGAGCGCCGGCCAAA

GACCCCTAAAATTCTGTCTTCCATGTCGTCCGAGCAACCATATAATAGTTAAAACTTTCA

ACAACGGATC

>ASV601 GS|90.7|AF250779|SH0954634.09FU;k:Fungi,p:Ascomycota,c:Lecanoromycetes,o:Caliciales,f:Caliciaceae,g:Buellia,s:Buellia frigida

AAGTCGTAACAAGATTTCCGTAGATGAACTTGCGAAAGAATCATTATCGAGAGACGAGGT

CGCTTCGACCCCACTCTTCACTCGTGCCTACTTATCTTTGTTGCTTTGACGAGCCTTCGA

GCTTGATCGTTCGACGCGAGTGAGAGACTTTCGCCTCTCGTCTCGCGAGCGCTCGTCAAA

GGCTCTGTTAACTCTGTTTTAGTGTCTTTCGAGCAACCATGTAATAGTTAAAACTTTCAA

CAACGAATCT

>ASV602 GS|85.3|AF250779|SH0954634.09FU;k:Fungi,p:Ascomycota,c:Lecanoromycetes,o:Caliciales,f:Caliciaceae,g:Buellia,s:Buellia frigida

AAGTCGTAACAAGATTTTCGTAGATGAATTTGCGAAAGAATCATTATCGAGAGACGAGAT

CGCTTCGACTTCACTCTTCACTCGTGTCTACTTATCTTTGTTGCTTTGACGAGTCTTCGA

GCTTGATCGTTCGACGCGAGTGAAAGACTTTCGTCTCTCGTTTCGCGAGCGCTCGTTAAA

GACTCTATTAACTTTGTTTTAGTGTCTTTCGAGCAATCATGTAATAGTTAAAACTTTCAA

CAACGAATCT

>ASV603 SS|1.0000|AF250779|SH0954634.09FU;k:Fungi,p:Ascomycota,c:Lecanoromycetes,o:Caliciales,f:Caliciaceae,g:Buellia,s:Buellia frigida

CTTTTTCCTTTCTCTTCCTTCCCCTTCTTCCCCCTTTTTCCGTATTTTCCCCTGCGGACT

TCTCCTTACCGATCTCCTTGGTCGCTTCTTCCCCACTCTTCCCCCGTGTCTACTTACCTT

TGTTGCTTTGGCGGGCCTTCGGGCTTGACCGTTCGCCGCGGGTGGGAGGCTTTCGCCTCT

CGCCTCGCGAGCGCCCGCCAAAGGCCCTGTTAACTCTGTTTTAGTGTCTTCCGAGCAACC

ATGTAATAGT

>ASV604 GS|84.8|AF250779|SH0954634.09FU;k:Fungi,p:Ascomycota,c:Lecanoromycetes,o:Caliciales,f:Caliciaceae,g:Buellia,s:Buellia frigida

AAGTCGTAACAAGATTTTCGTAGATGAATCTACGAAAGAATCATTATCGAGAGACGAGAT

CGCTTCGATTTCACTCTTCACTCGTGTTTACTTATCTTTGTTACTTTGACGAGTCTTCGA

GCTTGATCGTTCGACGCGAGTGAGAGACTTTCGTCTCTCGTTTCGCGAGCGCTCGTCAAA

GACTCTGTTAACTCTATTTTAGTGTCTTTCGAACAATCATGTAATAGTTAAAACTTTCAA

CAACGAATCT

>ASV605 GS|97.2|AY667583|SH0954634.09FU;k:Fungi,p:Ascomycota,c:Lecanoromycetes,o:Caliciales,f:Caliciaceae,g:Buellia,s:Buellia frigida

AAGTCGTAACAAGGTTTCCGTAGGTGAACTTGCGGAAGGATCATTATCGAGAGACGGGGT

CGCTTCGGCCTCATTCTTCACTCGTGTCTACTTACCTTTGTTGCTTTGGCGGGCCTTCGG

GCTTGATCGTTCGACGCGGGTGGGAGGCTTTCGCCTCTCGCCTCGCGAGCGCCCGCCAAA

GGCCCTGTTAACTCTGTTTTAGTGTCTTCCGAGCAACCATGTAATAGTTAAAACTTTCAA

CAATGGATCT

>ASV606 SS|0.9600|AY667583|SH0954634.09FU;k:Fungi,p:Ascomycota,c:Lecanoromycetes,o:Caliciales,f:Caliciaceae,g:Buellia,s:Buellia frigida

AAGTCGTAACAAGATTTCCGTAGGTGAACTTGCGAAAGAATCATTATCGAGAGACGAGGT

CGCTTCGACCTCACTCTTCACTCGTGTCTACTTATCTTTGTTGCTTTGGCGAGCTTTCGA

GCTTGATCGTTCGACGCGAGTGAGAGGCTTTCGCCTCTCGCCTCGCGAGCGCTCGCCAAA

GGCTTTGTTAACTTTGTTTTAGTGTCTTTCGAGCAATCATGTAATAGTTAAAACTTTCAA

CAACGAATCT

>ASV607 GS|0.0|None;No hit

AAGTCGTAACAAGGTCTCCGTAGGTGAACCTGCGGAGGGATCATTAACACGTTCCATACC

AACTATCAACCCAACCTGTGAAGTTTCGTATCTGCCACAAGCTCGTCCAGGTGCTCTCTG

TACGGACTCTTGTCTGCCTTTGTGCAGTCAGAGCCACAACAGGAATACTACGGTGTCGAC

TGAGATCCAGGATGAAGGTGATGAGATGCTTGACTCTTGTGGCACTCTGTGTTAACAACG

CAGAGAGCTC

>ASV608 GSL|100.0|OL691165|SH0985300.09FU;k:Fungi,p:Ascomycota

AAGTCGTAACAAGGTTTCCGTAGGTGAACCTGCGGAAGGATCATTACCTAGAGTTGTAGG

CTTTGCCTGCTATCTCTTACCCATGTCTTTTAAGTACCTTACGTTTCCTCGGCGGGTCCG

CCCGCCGATTGGACAATTTAAACCATTTGCAGTTGCAATCAGCGTCTGAAAAAACTTAAT

AGTTACAACTTTCAACAACGGATCTCTTGGTTCTG

>ASV609 GS|0.0|None;No hit

AAGTCGTAACAAAATTTTCGTAAATGAATTTACGAAAAAATCATTATCGAGAGACGAAAT

CGCTTTGATCTCACTCTTCACTCGTGTTTACTTATCTTTGTTACTTTGACGAGTCTTCGA

ACTTAATCGTTCGACGCGAATGAGAGACGTTTGTCTCTCGTTTCGCGAGTGTTCGTTAAA

GACTCTATTAACTCTATTTTAATATTTTTCGAACAATCATGTAATAATTAAAACTTTTAA

CAACGAATCT

>ASV610 GS|0.0|None;No hit

AAGTCGTAACAAGATTTTCGTAAATGAATTTACGAAAAAATCATTATCGAAAGACGAAAT

CGCTTCGATCTCACTCTTCACTCGTATTTACTTATCTTTGTTGCTTTGACGAGTCTTCGA

ACTTGATCGTTCAACGCGAGTAAGAGACTTTCGTCTCTCGTTTCGCAAGCGCTCGTTAAA

GATTCTATTAACTTTATTTTAATGTCTTTCGAATAATCATGTAATAATTAAAATTTTCAA

CAACGAATCT

>ASV611 GS|99.5|AF250779|SH0954634.09FU;k:Fungi,p:Ascomycota,c:Lecanoromycetes,o:Caliciales,f:Caliciaceae,g:Buellia,s:Buellia frigida

AAGTCGTAACAAGGTTTCCGTAGATGAACCTGCGGAAGAATCATTACCGAGAGACGAGGT

CGCTTCGGCCCCACTCTTCACCCGTGTCTACTTACCTTTGTTGCTTTGGCGGGCCTTCGG

GCTTGACCGTTCGACGCGGGTGGGAGGCTTTCGCCTCTCGCCTCGCGAGCGCCCGCCAAA

GGCCCTGTTAACTCTGTTTTAGTGTCTTCCGAGCAACCATGTAATAGTTAAAACTTTCAA

CAACGGATCT

>ASV612 GS|83.3|AF250779|SH0954634.09FU;k:Fungi,p:Ascomycota,c:Lecanoromycetes,o:Caliciales,f:Caliciaceae,g:Buellia,s:Buellia frigida

AAATTGTAACAAGATTTTCGTAGATGAATTTGCGAAAGAATCATTATCGAGAGACGAAAT

CGCTTCGATCTCATTCTTCACTCGTGTCTACTTATCTTTGTTGCTTTGACGAGTCTTCGA

GCTTGATCGTTCGACGCGAGTGAGAGACTTTCGTCTCTCGTCTTGCGAGCGCTCGTCAAA

GATTTTATTAACTTTATTTTAGTGTCTTTCGAGCAATCATGTAATAGTTAAAACTTTCAA

CAATGAATCT

>ASV613 GS|82.0|AY667583|SH0954634.09FU;k:Fungi,p:Ascomycota,c:Lecanoromycetes,o:Caliciales,f:Caliciaceae,g:Buellia,s:Buellia frigida

AAGTCGTAACAAGATTTTCGTAGATGAATCTACGAAAAAATCATTATCGAAAGACGAGAT

CGCTTCGATCTTACTCTTCACTCGTATCTACTTATCTTTGTTGCTTTGACGAGTCTTCGA

ACTTGATCGTTCGATGCGAGTAAGAGACTTTCGTTTGTCGTTTCGCGAGCGCTCGTTAAA

GACTTTGTTAACTCTATTTTAGTGTCTTTCGAGCAATCATGTAATAGTTAAAACTTTCAA

CAACGAATCT

>ASV614 GS|73.7|MZ229889|SH0913114.09FU;k:Fungi,p:Ascomycota,c:Lecanoromycetes,o:Caliciales,f:Caliciaceae,g:Dimelaena

AAGTCGTAACAAGATTTTCGTAAATGAATTTGCGAAAGGATCATTATCAAGAGACGAAGT

CTAACCGGCCCCACTCTTCACCCGTGTATATCTACCCTTTATTGCTTTAACGAGCGGTCG

ATCTTTACCGCCCGACGTCGGTCGAAAGACTCGCGCCTTTCATTCATCGAACGCCCGCCA

GAGATTCTTCAAACCCGTTCATTTATGTTCGAGCGAGTGAAGTAAATAATTAAAAACTTT

CAACAACGGA

>ASV615 GS|89.7|AF250779|SH0954634.09FU;k:Fungi,p:Ascomycota,c:Lecanoromycetes,o:Caliciales,f:Caliciaceae,g:Buellia,s:Buellia frigida

AAGTCGTAACAAGATTTTCGTAGATGAACTTGCGAAAGAATCATTATCGAGAGACGAGGT

CGCTTCGACCTCACTCTTCACTCGTGTCTACTTATCTTTGTTGCTTTGGCGAGCCTTCGA

GCTTGATCGTTCGACGCGAGTGAAAGACTTTCGTCTCTCGCCTCGCGAGCGCTCGTCAAA

GATCTTGTTAACTCTGTTTTAGTGTCTTTCGAGCAACCATGTAATAGTTAAAACTTTCAA

CAACGAATCT

>ASV616 SS|1.0000|AY667583|SH0954634.09FU;k:Fungi,p:Ascomycota,c:Lecanoromycetes,o:Caliciales,f:Caliciaceae,g:Buellia,s:Buellia frigida

AAGTCGTAACAAGGTTTCCGTAGGTGAACCTGCGAAAGGATCATTATCGAGAGACGGGGT

CGCTTCGACCCCACTCTTCACTCGTGTCTACTTACCTTTGTTGCTTTGGCGGGCCTTCGG

GCTTGATCGTTCGACGCGGGTGAGAGGCTTTCGCCTCTCGCCTCGCGAGCGCTCGCCAAA

GGCCCTATTAACTCTGTTTTAGTGTCTTTCGACCAACCATGTAATAGTTAAAACTTTCAA

CAACGGATCT

>ASV617 GS|79.3|MG954222|SH1107724.09FU;k:Fungi,p:Ascomycota,c:Lecanoromycetes,o:Teloschistales,f:Teloschistaceae,g:Caloplaca,s:Caloplaca nivalis

AAGTCGTAATAAGGTTTCCGTAGGTGAACCTGCGGAAGGATTATTATCGAGAGGGGGGGC

TTTATACCCCGGGGCTCTGTCCCCGTACCTTTTCACCCTGTGTGTATTTTTCCCCCGTTG

CTTTAGCGGGCCCCGGGTCTTCCCCCGGCGTTGGCCCCCTCGCGGGGTTCGCGAGCGCCC

GCCGGAAGGCTTATCGAAACTCTAATTAGTGTAGTCTAAGCGTACGAATAATAAATCAAA

ACTTTCAACA

>ASV618 GS|73.8|MN103133|SH0913114.09FU;k:Fungi,p:Ascomycota,c:Lecanoromycetes,o:Caliciales,f:Caliciaceae,g:Dimelaena,s:Dimelaena oreina

AAGTCGTAATAAGGTTTTCGTAAGTGAATCTGCGAAAGGATCATTATCGAGAGACAAAAT

CTAACCGGCCCCACTCTTTACTCGTGTATATTTATTCTTTATTGTTTTGACGGGCGGTCG

GTCTTTACCGCCCGACGTCGGTCGAAAGACTCGCGCCTTTCACTCATCGAACGCCCGTCA

AAGATTTTCAAAACTCGTTTATTTATGTTCGAGCGAGTGAAATAAATAATTAAAAACTTT

CAACAACGGA

>ASV619 GS|70.9|MN103133|SH0913114.09FU;k:Fungi,p:Ascomycota,c:Lecanoromycetes,o:Caliciales,f:Caliciaceae,g:Dimelaena,s:Dimelaena oreina

AAGTCGTAACAAGATTTTCATAAATGAATTTGCGAAAGAATCATTATCGAGAAACGGAGT

CTAATCGATCCCACTTTTCACTCGTGTATATTTACTCTTTATTGTTTTGACGGGCGATCG

GTCTTTATCGCCCGACGTCGGTCGGAAAACTCGCGCCTTTCACTCACCGAACGCCCGTCA

AAGATCCTCAAAATCCGTTCATTTATATTCGAGCGAATAAAGCAAATAATTAAAAATTTT

CAACAACGAA

>ASV620 GS|88.7|AF250779|SH0954634.09FU;k:Fungi,p:Ascomycota,c:Lecanoromycetes,o:Caliciales,f:Caliciaceae,g:Buellia,s:Buellia frigida

AAGTCGTAACAAGATTTTCGTAGATGAACTTGCGAAAGAATCATTATCGAGAGACGAGAT

CGCTTCGATCTCACTCTTCACTCGTGTCTACTTACCTTTGTTGCTTTGACGAGTCTTCGA

GCTTGATCGTTCGACGCAAATGAGAGGCTTTCGCCTCTCGTCTCGCGAGCGCTCGTCAAA

GGCCTTGTTAACTCTGTTTTAGTGTCTTTCGAGCAATCATGTAATAGTTAAAACTTTCAA

CAACGAATCT

>ASV621 GS|75.8|MK778643|SH0916486.09FU;k:Fungi,p:Ascomycota,c:Lecanoromycetes,o:Caliciales,f:Physciaceae,g:Tetramelas

AAGTCGTAACAAGATTTCTGTAGATGAATTTGCGGAAGGATCATTATCGAGAGACGGAGT

CTAACCGGCCCCACTCTTCACCCGTGTATATCTACCCTTTATTACTTTGACGGGCGGTCG

ATCTTTACCGCCCGACGTCGATCGAAAGGCTCGCGTCTTCCACTCACCGAACGCCCGTCA

AAGATCCTCAAAAGTCGTTCATCTATGTTCGAGCGAGTGAAGCAAATAATTAAAAACTTT

CAACAACGGA

>ASV622 SS|1.0000|AF250779|SH0954634.09FU;k:Fungi,p:Ascomycota,c:Lecanoromycetes,o:Caliciales,f:Caliciaceae,g:Buellia,s:Buellia frigida

AAGTCGTAACAAGGTTTGCGTAGATGAATCTGCGAAAGGATCATTATCGAGAGACGAGGT

CGCTTCGGCCCCACTCTTCACTCGTGTCTACTTACCTTTGTTGCTTTGACGAGCCTTCGA

GCTTGATCGTTCGACGCGGGTGGGAGGCTTTCGCCTGTCGCCTCGCGAGCGCCCGCCAAA

GGCCCTGTTAACTCTGTTTTAGTGTCTTTCGAGCAACCATGTAATAGTTAAAACTTTCAA

CAACGAATCT

>ASV623 GS|80.4|MN592663|SH1107827.09FU;k:Fungi,p:Ascomycota,c:Lecanoromycetes,o:Teloschistales,f:Teloschistaceae,g:Caloplaca,s:Caloplaca haematites

AAGTCGTAATAAGGTTTCCGTAGGTAAACCTGCGGAAGGATTATTATCGAGAGGGGGGGC

TCTATGCCCCGGGGCTCTGTCCCCGTACCTTTTCACCCTGTGTGTATTTTTCCCCCGTTG

CTTTAGCGGGCCCCGGGTCTTCCCCCGGCGTTGGCCCCCTCGCGGGGTTCGCGAGCGCCC

GCCGGAAGGCTTATCGAAACTCTGATTAGTACAGTCTGAGCGTACGAATAATAAATTAAA

ACTTTTAATA

>ASV624 SS|0.9200|MG982528|SH1003748.09FU;k:Fungi,p:Basidiomycota,c:Tremellomycetes,o:Tremellales

AAGTCGTAACAAGGTTTCCGTAGGTGAACCTGCGGAAGGATCATTAGTGATTCGGCCCTC

ACGGGTCTATAAAAGACACCTCTGTGAACCTGTCGGCCTCCGGGCCCACCTGCAAACACT

GTGTAACGAGCGTTGATGTATCATAAGCATAATAACACTTTCAACAACGGATCTCTTGGC

TCTC

>ASV625 GS|85.6|AY667583|SH0954634.09FU;k:Fungi,p:Ascomycota,c:Lecanoromycetes,o:Caliciales,f:Caliciaceae,g:Buellia,s:Buellia frigida

AAGTCGTAACAAGATTTTCGTAGATGAACTTGCGAAAGAATCATTATCGAGAGACGAAAT

CGCTTCGATCTCACTCTTCACTCGTGTCTACTTATCTTTGTTGCTTTGACGAGTCTTCGA

ACTTGATCGTTCGACGCGAGTGAGAGACTTTCGCTTGTCGTCTCGCGAGCGCTCGCCAAA

GATTCTATTAACTTTGTTTTAGTGTCTTTCGAGCAATCATGTAATAGTTAAAACTTTCAA

CAACGAATCT

>ASV626 GS|82.8|AF250779|SH0954634.09FU;k:Fungi,p:Ascomycota,c:Lecanoromycetes,o:Caliciales,f:Caliciaceae,g:Buellia,s:Buellia frigida

AAGTCGTAACAAGATTTTCGTAAATGAACTTGCGAAAAAATCATTATCGAGAGACGAAAT

CGCTTCGATCTCACTCTTCACTCGTATCTACTTATTTTTGTTGCTTTGACGAGTCTTCGA

GCTTGATCGTTCGACGCGAGTGAGAGACTTTCGCTTGTCGTCTTGCGAGCGTTCGTCAAA

GACTCTATTAACTCTATTTTAGTGTCTTTCGAACAATTATGTAATAGTTAAAACTTTCAA

CAACGAATCT

>ASV627 SS|1.0000|AF250779|SH0954634.09FU;k:Fungi,p:Ascomycota,c:Lecanoromycetes,o:Caliciales,f:Caliciaceae,g:Buellia,s:Buellia frigida

AAGTCGTAACAAGGTTTTCGTAGATGAACCTACGAAAGAATCATTATCGAGAGACGAGGT

CGCTTCGATCCCACTCTTCACCCGTGTCTACTTACCTTTGTTGCTTTGGCGAGCCTTCGG

GCTTGATCGTTCGACGCGGGTGGGAGGCTTTCGCCTCTCGCCTCGCGAGCGCCCGTCAAA

GGCCCTGTTAACTCTATTTTAGTGTCTTTCGAGCAATCATGTAATAGTTAAAACTTTCAA

CAACGAATCT

>ASV628 GS|78.4|AY667583|SH0954634.09FU;k:Fungi,p:Ascomycota,c:Lecanoromycetes,o:Caliciales,f:Caliciaceae,g:Buellia,s:Buellia frigida

AAGTCGTAACAAGATTTTCATAAATGAATCTACGAAAGAATCATTATCGAGAGACGAAAT

CGCTTCGATTTCATTCTTCATTCGTATCTACTTATTTTTGTTACTTTGACGAGTTTTCGA

GCTTGATCGTTCGATGCGAATGAAAGATTTTCGTCTATCGTTTTGCGAACGCTCGTCAAA

GACTCTATTAACTCTATTTTAGTGTCTTTCGAACAATCATGTAATAGTTAAAACTTTTAA

CAACGAATCT

>ASV629 GS|85.8|AF250779|SH0954634.09FU;k:Fungi,p:Ascomycota,c:Lecanoromycetes,o:Caliciales,f:Caliciaceae,g:Buellia,s:Buellia frigida

AAGTCGTAACAAGGTTTTCGTAAATGAATTTGCGAAAGAATCATTATCGAGAGACGAGAT

CGCTTCGATTTCACTCTTCACTCGTGTCTACTTATCTTTGTTGCTTTGACGAGTCTTCGA

GCTTGATCGTTCGACGCGAATGAGAGGCTTTCGTCTCTCGTTTCGCGAGCGCTCGTCAAA

GATCTTGTTAACTTTGTTTTAGTGTCTTTCGAGCAATCATGTAATAGTTAAAACTTTCAA

CAACGAATCT

>ASV630 SS|0.9800|MZ919298|SH1277830.09FU;k:Fungi,p:Ascomycota,c:Candelariomycetes,o:Candelariales,f:Candelariaceae,g:Candelariella,s:Candelariella flava

AAGTCGTAACAAGGTTTCCGTAGGTGTAAGTAATCCAACGTCCCAAACATTCACCATCCA

ATGCGTGATCATGCTAACATGTGATTTAGGAACCTGCGGAAGGATCATTAAAGAGCAAGG

GTCTTCTAGGCCCGACCTCCAACCCTTTGTATATCTACCTCTGTTGCTTTGGCGGGCCCG

TCGGGTGACCCACCGGTGGCCTCTGGCTCCCGAGTGCCCGTCAGAGACCCATCAAAACCC

GTTAATTGTG

>ASV631 GS|88.8|AY667583|SH0954634.09FU;k:Fungi,p:Ascomycota,c:Lecanoromycetes,o:Caliciales,f:Caliciaceae,g:Buellia,s:Buellia frigida

AAGTCGTAACAAGGTTTCCGTAGATGAACTTGCGAAAGAATCATTATCGAGAGACGAGAT

CGCTTCGACCTCACTCTTCACTCGTGTCTACTTATCTTTGTTGCTTTGACGAGTCTTCGA

ACTTGATCGTTCGACGCGAGTGAGAGGCTTTCGCTTGTCGTCTCGCGAGCGCTCGCCAAA

GATCTTGTTAACTCTGTTTTAGTGTCTTTCGAGCAACCATGTAATAGTTAAAACTTTCAA

CAACGAATCT

>ASV632 GS|79.4|AF250779|SH0954634.09FU;k:Fungi,p:Ascomycota,c:Lecanoromycetes,o:Caliciales,f:Caliciaceae,g:Buellia,s:Buellia frigida

AAGTCGTAACAAAATTTTCATAGATGAATCTACGAAAGAATTATTATTGAGAGACGAAAT

CGCTTCGATTTCACTTTTCATTCGTATCTACTTATCTTTGTTATTTTGACGAGTCTTCGA

GCGCGATCGTTCGACGCAAGTGAGAAACTTTCGTCTCTCGTTTTGCGAGCGCTCGTCAAA

GATTTTGTTAACTCTATTTTAGTGTCTTTCGAGCAATCATGTAATAGTTAAAACTTTCAA

CAACGAATCT

>ASV633 SS|0.8100|JN873879|SH1123218.09FU;k:Fungi,p:Ascomycota,c:Lecanoromycetes

AAGTCGTAACAAGGTTTCCGTAGGTGAACCTGCGGAAGGATCATTACCGAGAGCGGGGCT

CACCCCCCCAAACTCCGCCGCCGAAAGGGTACTCTCCACCCTATGTATACATACCACTCT

CGCTTTGGCGGGCGCGGAAGGCTTTATGCCCTGCGGCCGCCGGCGGCTCATTCAAATTCG

GTTTATCAGTGACGTCCGAGTAAAAACGCAACAGTTAAAACTTTCAACAACGGATCTCTT

GGTTCTG

>ASV634 SS|0.9000|KU672610|SH0991977.09FU;k:Fungi,p:Ascomycota,c:Lecanoromycetes,o:Trapeliales,f:Trapeliaceae,g:Trapelia

AAGTCGTAACAAGGTTTCCGTAGGTGAACCTGCGGAAGGATCATTACCGAGATAGGGTCC

CCCGGGCCCGACCCTCCACCCGCTGCGTACCTACCTTTTGTTGCTTTGGCGGGCCGCGGG

GCCCCCGGCCCCCCGTCGACCCCGGTGGGCGAGCGCCTGCCAGAGACCCCCCCAACCCGG

TTGATCAGTGACGTCCGAGCCCCGATGAAAATCAGTTAAAACTTTCAACAACGGATCTCT

TGGTTCTG

>ASV635 GS|99.5|MH863220|SH0991924.09FU;k:Fungi,p:Ascomycota,c:Dothideomycetes,o:Mycosphaerellales,f:Teratosphaeriaceae,g:Elasticomyces,s:Elasticomyces elasticus

AAGTCGTAACAAGGTCTCCGTAGGTGAACCTGCGGAGGGATCATTACCGAGTGAGGGCCT

CCGGGTCCGACCTCCAACCCCATGTTAACCGACCCTGTTGCCTCGGGGGCGACCCGGACT

CGTGCCGGGGCCCCCGATGGACCTTCAAACACTGCATCTTTGGGTCCGAGTCAATATTTT

GAATAAATCAAAACTTTCAACAACGGATCTCTTGGTTCTG

>ASV636 GS|97.1|KY266860|SH0954363.09FU;k:Fungi,p:Ascomycota,c:Lecanoromycetes,o:Acarosporales,f:Acarosporaceae,g:Pleopsidium,s:Pleopsidium chlorophanum

AAGTCGTAACAAGGTTTTCGTAGGTGAACCTGCGGAAGGATCATTACAGAGTTAGGGTCT

TCCGGGCCCGATCTCCAACCCTATGTCTACCTACCTTTGTTCTTTGGCGGGCCCGCTGGG

GGAGACCCACCGGTGGCTCCGGCTGCCGAGCGCCCGTCAGAGACCCATCGAACCCTGTTA

ATCATGTAGTCTGAGTACCAATACAATAGTTAAAACTTTCAACAACGGATCTCTTGGTTC

TG

>ASV637 GS|74.2|MN103133|SH0913114.09FU;k:Fungi,p:Ascomycota,c:Lecanoromycetes,o:Caliciales,f:Caliciaceae,g:Dimelaena,s:Dimelaena oreina

AAGTCGTAACAAGGTTTTCGTAAATGAACCTGCGGAAGGATCATTATCGAGAGACGAAGT

TTAACCGGCCCCACTCTTCACTCGTGTATATTTACCCTTTATTGTTTTGGCGAGCGGTCG

ATCTTTATCGCCCGACGTCGATCGGAAGACTCGCGCCTTCCACTCATCGAACGCTCGTTA

AAGATTTTTAAAAGTCGTTCATTTGTGTTCGAGCGAGTGAAACAAATAATTAAAAACTTT

CAACAACGGA

>ASV638 GS|80.6|MZ229869|SH0913119.09FU;k:Fungi,p:Ascomycota,c:Lecanoromycetes,o:Caliciales,f:Caliciaceae,g:Dimelaena

AAGTCGTAACAAGGTTTTCGTAGATGAATTTGCGGAAGAATCATTATCGAGAGACGGAGT

CTAACCGGCCCCACTCTTCACCCGTGTATATCTACCCTTTGTTGCTTTGGCGGGCGGTCG

GTCTTTATCGCCCGACGTCGGTCGGAAGGCTCGCGCCTTCCACTCACCGAACGCCCGCCA

GAGGCCCCCAAAACCCGTCCATCTGTGTCCGAGCGAGTGAAGCAAATAATTAAAAACTTT

CAACAACGGA

>ASV639 SS|0.8500|AY667583|SH0954634.09FU;k:Fungi,p:Ascomycota,c:Lecanoromycetes,o:Caliciales,f:Caliciaceae,g:Buellia,s:Buellia frigida

AAGTCGTAACAAGGTTTCCGTAGGTGAACCTGCGGAAGGATCATTACCGAGAGACGGGGT

CGCTTCGGCCCCACTCTTCACCCGTGTCTACTTACCTTTGTTGCTTTGGCGCGCCCTCGG

GCCTGACCGTCTGGCGCTGGTAGGGGGGTCCCGCCCCTCGCCCAGCGAGCGCCGGCCAAA

GACCCCTAAAATTCTGTCTTCCATGTCGTCCGAGCAACCATATAATAGTTAAAACTTTCA

ACAACGGATC

>ASV640 SS|1.0000|KF673749|SH0992234.09FU;k:Fungi

AAGTCGTAACAAGGTTTCCGTAGGTGAACCTGCGGAAGGATCATTACTGAGTTAGGGTCT

TTTAGGCCCGACCTCCAACCCTATGTCTACCTTACCATGTTGCTTTGGCGGGCCCGTCGT

TAGTTCGACCGCCTCCGGGTCCGTGCCCGCCAGAAGCCCAATTAAATTCTTGAAGAAACT

TGTCGTCTTAGTATACAAGCAATAATAAAAAACTTTCAACAACGGATCTCTTGGTTCTG

>ASV641 SS|0.8700|AY143410|SH0954621.09FU;k:Fungi,p:Ascomycota,c:Lecanoromycetes,o:Caliciales,f:Caliciaceae,g:Buellia

AAGTCGTAACAAGGTTTCCGTAGGTGAACCTGCGGAAGGATCATTACCGAGAGACGGGGT

CGTTTCGGCCCCACTCTTCACCCGTGCACATATATCTCTGTTGCTTTGGCGCGCCCTCGG

GCCTAACCGTCCGGCGCTGGTAGGGGGCTCTGTCCCTCGCCCCGCGAGTGCCCGCCAAAG

ACCCCCATAACTCTGTACTTAGTGCTGTCTGAGCAACCATACAATAGTTAAAACTTTCAA

CAACGGATCT

>ASV642 SS|1.0000|FR682176|SH1068012.09FU;k:Fungi

AAGTCGTAACAAGGTTTCCGTAGGTGAACCTGCGGAAGGATCATTACTGAGATAGGGTCC

TCCGGGGCCCGACCTCCAACCCTTTGTCTACCTTACCACTGTTGCCTCGGCGGGTCGCCG

GCGCTCTCACGGGCGTCGCCGTCGGCCTCCGCGCTCTCGAGTGCCCGCCGTTAGGCCCCC

TAAATCCTTTATAAATCAGTCTTCTGAGAGTATATTCAATACTTAAAACTTTCAACAACG

GATCTCTTGG

>ASV643 GS|78.5|AY667583|SH0954634.09FU;k:Fungi,p:Ascomycota,c:Lecanoromycetes,o:Caliciales,f:Caliciaceae,g:Buellia,s:Buellia frigida

AAATCGTAACAAGATTTTCGTAGATGAATCTACGAAAGAATCATTATCGAAAGACGAGAT

CGCTTCGATCTCACTCTTCATTCGTATCTACTTATCTTTGTTGCTTTGACGAATCTTCGA

GCTTGATCGTTCGACGCGAGTAAGAAACTTTCGTCTCTCGTTTCGCGAGCGCTCGTCAAA

GACTCTATAGTGTCTTTCGAGCAATTATGTAATAGTTAAAACTTTCAACAACGAATCTCT

TGATTCTA

>ASV644 SS|1.0000|AF250779|SH0954634.09FU;k:Fungi,p:Ascomycota,c:Lecanoromycetes,o:Caliciales,f:Caliciaceae,g:Buellia,s:Buellia frigida

AAGTCGTAACAAGATTTTCGTAGATGAATTTGCGAAAGAATCATTATCGAGAGACGAAAT

CGCTTCGATCTCACTCTTCACTCGTATTTACTTATCTTTGTTGCTTTGACGAGTCTTCGA

GCTTGATCGTTCGACGCGGGTGGGAGGCTTTTGCCTCTCGCCTCGCGAGCGCCCGCCAAA

GGCCCTGTTAACTCTGTTTTAGTGTCTTCCGAGCAACCATGTAATAGTTAAAACTTTCAA

CAACGGATCT

>ASV645 GS|81.9|AF250779|SH0954634.09FU;k:Fungi,p:Ascomycota,c:Lecanoromycetes,o:Caliciales,f:Caliciaceae,g:Buellia,s:Buellia frigida

AAGTCGTAACAAGATTTTCGTAAATGAATTTGCGAAAAAATCATTATCGAGAGACGAAAT

CGCTTCGATCTCACTCTTCATTCGTATCTACTTATCTTTGTTGCTTTGACGAATCTTCGA

ACTTGATCGTTCGACGCGAATGAAAAACTTTCGCTTGTCGTCTCGCGAACGCTCGTCAAA

GACTCTATTAACTTTGTTTTAGTGTCTTTCGAGCAATCATGTAATAGTTAAAACTTTCAA

CAACGAATCT

>ASV646 GS|84.4|AY667583|SH0954634.09FU;k:Fungi,p:Ascomycota,c:Lecanoromycetes,o:Caliciales,f:Caliciaceae,g:Buellia,s:Buellia frigida

AAGTCGTAACAAGATTTTCGTAGATGAATTTGCGAAAGAATCATTATCGAGAGACGAAAT

CGCTTCGATCTCACTCTTCACTCGTATCTACTTATCTTTGTTGCTTTGACGAGTCTTCGA

GCTTGATCGTTCGACGCGAGTAAGAGACTTTCGTCTCTCGTCTCGCGAGCGCTCGTCAAA

GACTTTGTTAACTTTGTTTTAATGTCTTTCGAGCAATCATGTAATAATTAAAACTTTCAA

CAACGAATCT

>ASV647 GS|82.4|AY667583|SH0954634.09FU;k:Fungi,p:Ascomycota,c:Lecanoromycetes,o:Caliciales,f:Caliciaceae,g:Buellia,s:Buellia frigida

AAATCGTAACAAGATTTTCGTAGATGAATCTACGAAAGAATCATTATCGAGAGACGAAAT

CGCTTCGATCTCACTCTTCACTCGTATCTATTTATCTTTGTTGCTTTGACGAATTTTCGA

ATTTGATCGTTCGACGCGAGTGAAAGACTTTCGTCTCTCGTCTCGCGAGCGCTCGTCAAA

GATCGTGTTAACTCTATTTTAATGTCTTTCGAGCAATCATGTAATAGTTAAAACTTTCAA

CAACGAATCT

>ASV648 SS|0.9500|KF274388|SH1185377.09FU;k:Fungi,p:Basidiomycota,c:Tremellomycetes,o:Tremellales

AAGTCGTAACAAGGTTTCCGTAGGTGAACCTGCGGAAGGATCATTAATGAATGTCTTCGG

ACTTAAACCATATTCAAACCTTTGTGAACCGTGGCCTCCGGGCCGTCTTTCAAACACTGT

GTAATGAACGTCAATTGATACAAAACAAGTAAAACTTTCAACAACGGATCTCTTGGCTCT

C

>ASV649 GS|0.0|None;No hit

GTGTCAGCAGCCGCGGTAATACGGAGGATCCGAGCGTTATCCGGATTTATTGGGTTTAAA

GGGTGCGTAGGCGGCCTGTTAAGTCAGGGGTGAAAGACGGTAGCTCAACTATCGCAGTGC

CTTTGATACTGACGGGCTTGAATGCAGCTGAGGTAGGCGGAATGTGACAAGTAGCGGTGA

AATGCATAGATATGTCACAGAACACCAATTGCGAAGGCAGCTTACTAAAGTGTGATTGAC

GCTGAGGCAC

>ASV650 GS|100.0|KP825473|SH1102553.09FU;k:Fungi,p:Basidiomycota,c:Malasseziomycetes,o:Malasseziales,f:Malasseziaceae,g:Malassezia,s:Malassezia restricta

AAGTCGTAACAAGGTTTCTGTAGGTGAACCTGCAGAAGGATCATTAGTGAAGATTTGGGC

AGGCCATACGGACGCCAAAAAGTGTCCCTGGCCGCCTACACCCACTATACATCCACAAAC

CCGTGTGCACTGTCTTGGAGAAAGGCTTCAGAGAAGTTTTTTGTGGCCTCTCTTGGGGTC

TTTCTTCGCTACAAACTCGAATGGTTAGTATGAACGTGGAACTTGGTTGGACCGTCACTG

GCCAACAAAC

>ASV651 GS|75.2|MN615681|SH0916491.09FU;k:Fungi,p:Ascomycota,c:Lecanoromycetes,o:Caliciales,f:Caliciaceae,g:Buellia,s:Buellia insignis

AAGTCGTAACAAGGTTTCCGTAAGTGAACCTGCGAAAGAATCATTATCGAGAGACGAAGT

CTAACCGGTCCCACTCTTCACCCGTGTATATCTACCCTTTATTACTTTAACGAGCGATCG

ATCTTTATCGCCCGACGTCGATCGGAAGACTCGCGCCTTTCACTCACCGAACGCTCGCCA

AATATTTTCAAAACCCGTTCATCTGTGTCCGAGCGAGTGAAGCAAATAATTAAAAACTTT

CAACAACGAA

>ASV652 GS|86.8|AY667583|SH0954634.09FU;k:Fungi,p:Ascomycota,c:Lecanoromycetes,o:Caliciales,f:Caliciaceae,g:Buellia,s:Buellia frigida

AAGTCGTAACAAGGTTTTCGTAGATGAATTTGCGAAAGAATCATTATCGAGAGACGAGAT

CGCTTCGATTTCACTCTTCACTCGTGTCTACTTATCTTTGTTGCTTTGACGAATCTTCGA

GCTTGATCGTTCGACGCGAGTGAGAGACTTTCGCCTGTCGTCTCGCGAGCGCTCGTCAAA

GGTCTTGTTAACTCTGTTTTAGTGTCTTTCGAGCAATCATGTAATAGTTAAAACTTTCAA

CAACGAATCT

>ASV653 GS|85.8|AF250779|SH0954634.09FU;k:Fungi,p:Ascomycota,c:Lecanoromycetes,o:Caliciales,f:Caliciaceae,g:Buellia,s:Buellia frigida

AAGTCGTAACAAGATTTTCGTAGATGAATCTACGAAAGAATCATTATCGAGAGACGAGAT

CGCTTCGATCTCACTCTTCACTCGTGTTTACTTATCTTTGTTGCTTTGACGAATCTTCGA

GCTTGATCGTTCGACGCGAGTGAGAGACTTTCGTCTCTCGTCTCGCGAGCGCTCATCAAA

GACTCTGTTAACTCTATTTTAGTGTCTTTCGAGCAATCATGTAATAGTTAAAACTTTCAA

CAACGAATCT

>ASV654 GS|85.2|AY667583|SH0954634.09FU;k:Fungi,p:Ascomycota,c:Lecanoromycetes,o:Caliciales,f:Caliciaceae,g:Buellia,s:Buellia frigida

AAGTCGTAACAAGATTTTCGTAGATGAATCTGCGAAAGAATCATTATCGAGAGATGAGGT

CGCTTCGATCCCACTCTTCACTCGTGTCTACTTATCTTTGTTGCTTTGACGAATCTTCGA

ACTTGATCGTTCGACGCGAGTGAAAGGCTTTCGCTTGTCGTTTCGCGAGCGCTCGTCAAA

GATTCTATTAACTCTATTTTAGTGTCTTTCGAGCAATCATGTAATAGTTAAAACTTTCAA

CAACGGATTT

>ASV655 SS|0.8300|KP744437|SH0940275.09FU;k:Fungi,p:Ascomycota

AAGTCGTAACAAGGTTTCCGTAGGTGAGTAAATCGAACACACCCCCGGCGCTCGTGAACG

ACGGATCGCTAACCGTGTCTCTAGGTGAACCTGCGGAAGGATCATTATCGAGTAGGGTCC

CTCGGGGCCCGACCTCCAACCCTTGTGTCTACCACACCACGTTGCTTTGGCGGGCCCGCC

CTCTGGGCCGCCGGTGGTCCCCCCGGACCCCTGGCCCGTGCCCGTCAATGGCCCACACAA

AACCTGAACA

>ASV656 SS|1.0000|UDB02020877|SH1083901.09FU;k:Fungi

AAGTCGTAACAAGGTCTCCGTAGGTGAACCTGCGGAGGGATCATTACCGAGTGAGGGCCT

CCGGGCCCGACCTCCAACCCTCTGTTGTTAATACCACTGTTGCCTCGGGGGCGACCCGGC

CTCCGCGTCGGGGCCCCCGGTGGACCCAACCCAAACCCTGCGTCTTTGCGTCGGAGTATT

AAAGTAAATCAATTAAAACTTTCAACAACGGATCTCTTGGTTCTG

>ASV657 GS|72.3|KF535949|SH1174631.09FU;k:Fungi,p:Basidiomycota,c:Agaricomycetes,o:Agaricales,f:Amanitaceae,g:Amanita,s:Amanita exitialis

AAGTCGTAACAAGGTTTTCGTAGGTGAACCTGCGGAAGGATCATTACCAGATGGGCCTCC

GGGCCTTTTCTCCCACCCACGTGCACCGAGTTGCCGGGAGAGACGCAAGCCTCTCCCGGC

CAATTCTTCACGCTTGTTTTGTTGTCTGAGTCGAATGACCATAATTGAAAAGAAAACTAT

GAACAACGGATCTCTAGGCTCTT

>ASV658 GS|98.8|DQ534471|SH0942704.09FU;k:Fungi,p:Ascomycota,c:Lecanoromycetes,o:Lecanorales,f:Lecanoraceae,g:Lecidella,s:Lecidella carpathica

AAGTCGTAACAAGGTTTCCGTAGGTGGTTTGCCTGTCGGATCCCCCCCGCAGCGACTCTA

AAGAACTGCGCCAGTCGGGCTCCCATCTCCAAGCCTGGCGACGCCATCAGTCTGGCTGGG

AGCCCCCCTACGCGGGGGGCCACCAGCAGCTCCTTCGGGAGTCCACAGATCAAACGATGA

GCGGCCGCTTCACTGCGGTTCAGATATGACCGGCCCCAGCCTGCATCGGCTGGTGACTCC

TCGTGGAACC

>ASV659 GS|79.7|MZ229889|SH0913114.09FU;k:Fungi,p:Ascomycota,c:Lecanoromycetes,o:Caliciales,f:Caliciaceae,g:Dimelaena

AAGTCGTAACAAGGTTTTCGTAGGTGAATTTGCGGAAGGATCATTATCGAGAGACGGAGT

CTAACCGGCCCCACTCTTCACCCGTGTATACCTACCCTTTGTTGCTTTGGCGGGCGATCG

GTCTTTACCGCCCGACGTCGGTCGGAAGGCTCGCGCCTTCCACTCATCGAACGCCCGTCA

GAGACTCCCAAAACCCGTTCATTTGTGTTCGAGCGAGTGAAGCAAATAATTAAAAACTTT

CAACAACGGA

>ASV660 GS|97.3|KF309966|SH1084317.09FU;k:Fungi,p:Ascomycota,c:Dothideomycetes,o:Mycosphaerellales,f:Teratosphaeriaceae,g:Meristemomyces,s:Meristemomyces frigidus

AAGTCGTAACAAGGTCTCCGTAGGTGAACCTGCGGAGGGATCATTACTGAGTGAGGGTGC

TCGCGCCCGACCTCCAACCCCATGTTTTCCGACTCTGTTGCCTCGGGGGCGACCCGACCC

TCTCGTAGGGCCCGGGGCCCTCGGTGGACCGCTCAACTCTGCATCTGTGCGTCTGAGTCA

ATATTTGAATTAATCAAAACTTTTAACAACGGATCTCTTGGTTCTG

>ASV661 GS|99.4|UDB01817331|SH1035452.09FU;k:Fungi,p:Ascomycota,c:Dothideomycetes,o:Mycosphaerellales,f:Mycosphaerellaceae

AAGTCGTAACAAGGTCTCCGTAGGTGAACCTGCGGAGGGATCATTACCGAGTGAGGGCCT

CTGGCCCGACCTCCAACCCTTTTGTGAACCGACCTCTGTTGCCTCGGGGGCGACCCGGAC

CTTCGGGTGTTGGGTCCCCCCGGTGGACCAAACCAACTCTGCATCTCTGCGTCTGAGTAC

AAAGTAAATCAATTAAAACTTTTAACAACGGATCTCTTGGTTCTG

>ASV662 GS|80.8|AY667583|SH0954634.09FU;k:Fungi,p:Ascomycota,c:Lecanoromycetes,o:Caliciales,f:Caliciaceae,g:Buellia,s:Buellia frigida

AAGTCGTAACAAGATTTTCGTAAATGAATTTGCGAAAGAATCATTATCGAGAGACGAAAT

CGCTTCGATTTCACTCTTCACTCGTATCTACTTATCTTTGTTGCTTTGACGAATTTTCGA

ACTTGATCGTTCGATGCGAATGAGAGACTTTCGTCTCTCGTTTCGCGAGCGTTCGTCAAA

GACTCTATTAACTCTATTTTAATATCTTTCGAACAATCATGTAATAGTTAAAACTTTCAA

CAACGAATCT

>ASV663 GS|81.2|AY667583|SH0954634.09FU;k:Fungi,p:Ascomycota,c:Lecanoromycetes,o:Caliciales,f:Caliciaceae,g:Buellia,s:Buellia frigida

AAGTCGTAACAAGATTTTCGTAAATGAATTTGCGAAAGAATCATTATCGAGAGACGAAAT

CGCTTCGATCTCACTTTTCACTCGTATCTACTTATTTTTGTTGCTTTAACGAGTCTTCGA

GCTTGATCGTTCGATGCGAATGAGAGACTTTCGCTTGTCGTCTCGCGAGCGCTCGTCAAA

AATTTTATTAACTTTGTTTTAGTGTCTTTCGAACAATCATGTAATAGTTAAAACTTTCAA

CAACGAATCT

>ASV664 GS|90.2|AF250779|SH0954634.09FU;k:Fungi,p:Ascomycota,c:Lecanoromycetes,o:Caliciales,f:Caliciaceae,g:Buellia,s:Buellia frigida

AAGTCGTAACAAGATTTTCGTAGATGAATCTGCGAAAGAATCATTATCGAGAGACGAGGT

CGCTTCGACCCCACTCTTCACTCGTGTCTACTTATCTTTGTTGCTTTGACGAGTCTTCGA

GCTTGATCGTTCGACGCGAGTGAGAGGCTTTCGTCTCTCGCCTCGCGAGCGCTCGCCAAA

GATCCTGTTAACTCTGTTTTAGTGTCTTTCAAGCAATCATGTAATAGTTAAAACTTTCAA

CAACGAATCT

>ASV665 GS|76.3|UDB01716342|SH1085252.09FU;k:Rhizaria,p:Cercozoa

AAGTCGTAACAAGGTCTTCGTAGGTGAACCTGCGAAGGGATCATTAACACGTTCCAAACC

TATTATCAACCCATCTGTGAACTCTTGTTACCGCCGATATGGCTGACCACAAGTGTATGG

CGCATACGATCGACTCTACGATGTAAGGTCATAGGCCGCAGTAGGTAAACACTACTCGAC

TCTATGCCTGGTCTAGTATGCCTCCGTGTCCCTACTATTCTTAATACGATAGTCTGGACA

GCTATTCTCG

>ASV666 GS|97.6|AY667583|SH0954634.09FU;k:Fungi,p:Ascomycota,c:Lecanoromycetes,o:Caliciales,f:Caliciaceae,g:Buellia,s:Buellia frigida

AAGTCGTAACAAGGTTTCCGTAGGTGAACCTGCGGAAGGATCATTACCGAGAGACGGGGT

CGCTTCGGCCCCACTCTTTACCCGTGTCTACTTACCTTTGTTGCTTTGGCGGGCCTTCGA

GCTTGACCGTTCGACGCGGGTGGGAGGCTTTCGCCTCTCGCCTCGCGAGCGCCCGCCAAA

GACTTTGTTAACTCTGTTTTAGTGTCTTTCGAGCAACCATGTAATAGTTAAAACTTTCAA

CAACGGATCT

>ASV667 GS|78.2|MN103133|SH0913114.09FU;k:Fungi,p:Ascomycota,c:Lecanoromycetes,o:Caliciales,f:Caliciaceae,g:Dimelaena,s:Dimelaena oreina

AAGTCGTAACAAGATTTCCGTAGATGAACCTGCGGAAGAATCATTATTGAGAGACGAAAT

CTAATCGGCCCCACTCTTCACCCGTGTATATCTACTCTTTATTGCTTTGGCGGGCGGTCG

GTCTTTACCGCCCGACGTCGGTCGAAAGGCTCGCGCCTTCCACTCATCGAACGCCCGTCA

GAGATCTCCAAAACCCGTTCATTTGTGTTCGAGCGAGTGAAGCAAATAATTAAAAACTTT

CAACAACGGA

>ASV668 GS|99.6|JX036119|SH0942719.09FU;k:Fungi,p:Ascomycota,c:Lecanoromycetes,o:Lecanorales,f:Lecanoraceae,g:Rhizoplaca

AAGTCGTAACAAGGTTTCCGTAGGTGAACCTGCGGAAGGATCATTATCGAGAGGGGTCCC

CGGACTCCGGGGGCTTCGGCCCCCTACTCTTCACCCCATGTCTACACACCTTTGTTGCTT

TGGCGGGCCTCGGGTTCGCCCCGTACCGGCCGTGGGCTTCCATACCCCGGCCGTCCGTGC

CCGTCAGAGGCCCATGAACCCTCGTTTATCAGTGTCGTCCGAGTCCAACCATAATAGTAA

AAACTTTCAA

>ASV669 SS|1.0000|AY667583|SH0954634.09FU;k:Fungi,p:Ascomycota,c:Lecanoromycetes,o:Caliciales,f:Caliciaceae,g:Buellia,s:Buellia frigida

AAGTCGTAACAAGGTTTCCGTAGATGAACCTGCGAAAGGATCATTATCGAGAGACGAGGT

CGCTTCGACCCCACTCTTCATTCGTGTCTACTTACCTTTGTTGCTTTGACGAGCCTTCGA

GCTTGACCGTTCGACGCGAGTGGGAGACTTTCGCCTCTCGCCTCGCGAGCGCCCGTCAAA

GGCCCTGTTAACTCTGTTTTAGTGTCTTTCGAGCAATCATGTAATAGTTAAAACTTTCAA

CAACGGATCT

>ASV670 GS|97.6|AY667583|SH0954634.09FU;k:Fungi,p:Ascomycota,c:Lecanoromycetes,o:Caliciales,f:Caliciaceae,g:Buellia,s:Buellia frigida

AAGTCGTAACAAGGTTTCCGTAGGTGAACTTGCGGAAGGATCATTATCGAGAGACGGGGT

CGCTTCGGCCTCATTCTTCACTCGTGTCTACTTACCTTTGTTGCTTTGGCGGGCCTTCGG

GCTTGATCGTTCGACGCGGGTGGGAGGCTTTCGCCTCTCGCCTCGCGAGCGCCCGCCAAA

GGCCCTGTTAACTCTGTTTTAGTGTCTTCCGAGCAACCATGTAATAGTTAAAACTTTCAA

CAACGGATCT

>ASV671 GS|88.2|AF250779|SH0954634.09FU;k:Fungi,p:Ascomycota,c:Lecanoromycetes,o:Caliciales,f:Caliciaceae,g:Buellia,s:Buellia frigida

AAGTCGTAACAAGATTTTCGTAGATGAATCTACGAAAGAATCATTATCGAGAGACGAGGT

CGCTTCGACCTCACTCTTCACTCGTATCTACTTATCTTTGTTGCTTTGACGGGTCTTCGA

GCTTGATCGTTCGATGTGGGTGGAAGGCTTTCGTCTCTCGCCTCGCGAGCGCTCGTCAAA

GACTTTGTTAACTTTGTTTTAGTGTCTTTCGAGCAATCATGTAATAGTTAAAACTTTCAA

CAACGAATCT

>ASV672 SS|0.8100|MN592663|SH1107827.09FU;k:Fungi,p:Ascomycota,c:Lecanoromycetes,o:Teloschistales,f:Teloschistaceae

AAGTCGTAACAAGGTTTCCGTAGGTGAACCTGCGGAAGGATTATTATCGAGAGGGGGGGC

TCTATACCCCGGGGCTCTGTCCCCGTACCTTTTCACCCTGTGTGTATTTTTCCCCCGTTG

CTTTGGCGGGCCCCGGGTCTTCCCCCGGCGTTGGCCCCCTCGCGGGGTTCGCGAGCGCCC

GCCGGAAGGCTTATCGAAACTCTGATTAGTGCAGTCTGAGCGTACGAACAATAAATTAAA

ACTTTTAACA

>ASV673 SS|0.9500|None;k:Fungi

AAGTCATAACAAGGTTTCCGTAGGTGAACCTGCGGAAGGATCATTCGAGAATAGGGCTTC

TGGCCTTAATCCATTATATACACCGTGAAAACATAACCTTGGGACCTCGCAAGGGGGACC

TCGGATTACACTTTTAAACAGAGTCAAGAAAGGAAACATTATAAATACAAATAACTTTTA

ACAACGGATCTCTTGGCTCTC

>ASV674 GS|100.0|AF279772|SH1300522.09FU;k:Fungi,p:Ascomycota,c:Lecanoromycetes,o:Teloschistales,f:Teloschistaceae,g:Xanthoria,s:Xanthoria elegans

CTTTTTCCTTTCTCTTCCTTCCCAGTCTTCCCCCGGTTTCCGTAGGTGAACCTGCGGAAG

GATCATTACTAAGAGAGGGATGTACGCTTCCAGCCGAGTCCCGGGGGGCTGCGCCCCTCA

CCTCTTCAACCCTGTGTCTACCAACCGCTGTTGCTTCGGCGAGCGTCGGGGCGTCCGCGC

CCCGGCCCCGGCTTCGGTCGGTGAGCTCTCGCAGAGGCCTATCTTTATTCTGTTTTGCAG

TGACGTCCGA

>ASV675 GS|98.8|DQ534471|SH0942704.09FU;k:Fungi,p:Ascomycota,c:Lecanoromycetes,o:Lecanorales,f:Lecanoraceae,g:Lecidella,s:Lecidella carpathica

AAGTCGTAACAAGGTTTCCGTAGGTGGTTTGCCTGTCGGATCCCCCCGCAGCGACTCTAA

AGAACTGCGCCAGTCGGGCTCCCATCTCCAAGCCTGGCGACGCCATCAGTCTGGCTGGGA

GGCCCCCCTACGCGGGGGGCCACCAGCAGCTCCTTCGGGAGTCCACAGATCAAACGATGA

GCGGCCGCTTCACTGCGGTTCAGATATGACCGGCCCCAGCCTGCATCGGCTGGTGACTCC

TCGTGGAACC

>ASV676 GS|84.8|AF250779|SH0954634.09FU;k:Fungi,p:Ascomycota,c:Lecanoromycetes,o:Caliciales,f:Caliciaceae,g:Buellia,s:Buellia frigida

AAGTCGTAACAAGATTTTCGTAGATGAATCTACGAAAGAATCATTATCGAGAGACGAGAT

CGCTTCGATTTCACTCTTCACTCGTGTCTACTTATCTTTGTTGCTTTGACGAGTCTTCGA

ACTTGATCGTTCGACGCGAGTGAAAGACTTTCGCCTCTCGTCTCGCGAGCGCCCATCAAA

GATCTTGTTAACTTTGTTTTAGTGTCTTTCGAGCAATCATGTAATAATTAAAACTTTCAA

CAATGAATCT

>ASV677 GS|98.0|AF250779|SH0954634.09FU;k:Fungi,p:Ascomycota,c:Lecanoromycetes,o:Caliciales,f:Caliciaceae,g:Buellia,s:Buellia frigida

AAGTCGTAACAAGATTTTCGTAGGTGAACTTGCGAAAGAATCATTATCGAGAGACGAGGT

CGCTTCGACCCCACTCTTCACTCGTGTCTACTTACCTTTGTTGCTTTGGCGGGCCTTCGG

GCTTGACCGTTCGACGCGGGTGGGAGGCTTTCGCCTCTCGCCTCGCGAGCGCCCGCCAAA

GGCCCTGTTAACTCTGTTTTAGTGTCTTCCGAGCAACCATGTAATAGTTAAAACTTTCAA

CAACGGATCT

>ASV678 GS|75.3|MZ229889|SH0913114.09FU;k:Fungi,p:Ascomycota,c:Lecanoromycetes,o:Caliciales,f:Caliciaceae,g:Dimelaena

AAGTCGTAACAAGATTTCCGTAAATGAATTTGCGGAAGAATCATTATCGAAAAACGGAGT

CTAACCGACCCCACTCTTCACTCGTGTATATCTACCCTTTATTGCTTTAACGGGCGGTCG

ATCTTTACCGCCCGACGTCGGTCGGAAGGCTCGCGCCTTCCACTCACCGAACGCCCGCCA

GAGACCCCCAAAAGCCGTTCATCTGTGTTCGAGCGAGTGAAGTAAATAATTAAAAACTTT

CAACAACGAA

>ASV679 GS|86.8|AF250779|SH0954634.09FU;k:Fungi,p:Ascomycota,c:Lecanoromycetes,o:Caliciales,f:Caliciaceae,g:Buellia,s:Buellia frigida

AAGTCGTAACAAGATTTTCGTAGATGAATCTACGAAAGAATCATTATCGAGAGACGAGAT

CGCTTCGATCTCACACTCTTCACTCGTGTCTACTTACCTTTGTTGCTTTGACGAGTCTTC

GAGCTTGATCGTTCGACGCGAGTGAGAGACTTTCGCCTCTCGTCTCGCGAGCGCTCGTCA

AAGACTTTGTTAACTTTGTTTTAGTGTCTTTCGAGCAATCATGTAATATTTAAAACTTTC

AACAACGGAT

>ASV680 GS|97.7|FJ552767|SH0991945.09FU;k:Fungi,p:Ascomycota,c:Dothideomycetes,o:Mycosphaerellales,f:Teratosphaeriaceae,g:Elasticomyces

AAGTCGTAACAAGGTCTCCGTAGGTGAACCTGCGGAGGGATCATTACCGAGTGAGGGCCC

CCGGGCCCGACCTCCAACCCCGTTAACCGACCCTGTTGCCTCGGGGGCGACCCGGCCTCG

TGCCGGGGCCCCCGATGGACCTTCCAACACTGCATCTTTGCGTCCGAGTCAATATTTGAA

TAAATCAAAACTTTCAACAACGGATCTCTTGGTTCTG

>ASV681 GS|85.3|AF250779|SH0954634.09FU;k:Fungi,p:Ascomycota,c:Lecanoromycetes,o:Caliciales,f:Caliciaceae,g:Buellia,s:Buellia frigida

AAGTCGTAATAAGATTTTCGTAAATGAATCTATGAAAGAATCATTATCGAGAGACGAGAT

CGCTTCGATCTCACTCTTCACTCGTATCTACTTATCTTTGTTGCTTTGACGAGTCTTCGA

GCTTGATCGTTCGACGCGAGTAGGAGGCTTTCGTCTCTCGTCTCGCGAACGCTCGTCAAA

GATCTTGTTAACTCTATTTTAGTGTCTTTCGAGCAATCATGTAATAGTTAAAACTTTCAA

CAATGAATTT

>ASV682 GS|84.4|AY667583|SH0954634.09FU;k:Fungi,p:Ascomycota,c:Lecanoromycetes,o:Caliciales,f:Caliciaceae,g:Buellia,s:Buellia frigida

AAGTCGTAACAAGATTTTCGTAGATGAATCTACGAAAGAATCATTATCGAGAGACGAGAT

CGCTTCGATCTCACTCTTCATTCGTGTCTACTTATCTTTGTTGCTTTGACGAGTTTTTGA

GCTTGATCGTTCGACGCGAGTGAGAGACTTTCGTTTCTCGTTTCGCGAGCGCTCGTCAAA

GACTTTGTTAACTTTGTTTTAGTGTCTTTCGAGCAATCATGTAATAGTTAAAACTTTCAA

CAACGAATCT

>ASV683 GS|91.7|AF250779|SH0954634.09FU;k:Fungi,p:Ascomycota,c:Lecanoromycetes,o:Caliciales,f:Caliciaceae,g:Buellia,s:Buellia frigida

AAGTCGTAACAAGATTTTCGTAGATGAACTTGCGAAAGAATCATTATCGAGAGACGAGGT

CGCTTCGACCCCACTCTTCACTCGTGTCTACTTATCTTTGTTGCTTTGGCGAGCCTTCGA

GCTTGATCGTTCGACGCGAGTGAGAGACTTTCGCCTCTCGTCTCGCGAGCGCTCGCCAAA

GACTCTGTTAACTCTGTTTTAGTGTCTTTCGAGCAACCATGTAATAGTTAAAACTTTCAA

CAACGAATCT

>ASV684 GS|85.8|AF250779|SH0954634.09FU;k:Fungi,p:Ascomycota,c:Lecanoromycetes,o:Caliciales,f:Caliciaceae,g:Buellia,s:Buellia frigida

AAGTCGTAACAAGATTTTCGTAGATGAATCTACGAAAGAATCATTATCGAGAGACGAGAT

CGCTTCGATCTCACTCTTCACTCGTGTCTACTTATCTTTGTTGCTTTGACGAGTCTTCGA

GCTTAATCATTCGACGCGAGTGGAAGACTTTCGTCTCTCGTCTCGCGAGCGCTCGTCAAA

GGTCTTGTTAACTCTATTTTAGTGTCTTTCGAGCAATCATGTAATAGTTAAAACTTTCAA

CAATGAATCT

>ASV685 GS|82.8|AF250779|SH0954634.09FU;k:Fungi,p:Ascomycota,c:Lecanoromycetes,o:Caliciales,f:Caliciaceae,g:Buellia,s:Buellia frigida

AAGTCGTAACAAGATTTTCGTAGATGAATTTACGAAAGAATCATTATCGAGAGACGAGAT

CGCTTCGATCTCACTCTTTATTCGTATCTACTTATCTTTGTTGCTTTGACGAGTCTTCGA

GCTTGATCGTTCGACGCGAATGAGAGACTTTCGTCTCTCGTTTCGCGAGCGCTCGTCAAA

GATCTTGTTAACTCTATTTTAGTGTCTTTCAAGCAATCATGTAATAGTTAAAATTTTCAA

CAATGAATCT

>ASV686 GS|86.8|AF250779|SH0954634.09FU;k:Fungi,p:Ascomycota,c:Lecanoromycetes,o:Caliciales,f:Caliciaceae,g:Buellia,s:Buellia frigida

AAGTCGTAACAAGATTTTCGTAGATGAATCTACGAAAGAATCATTATCGAGAGACGAGGT

CGCTTCGACCCCACTCTTCACTCGTGTTTACTTACCTTTGTTGTTTTGACGAGCTTTCGA

GCTTGATCGTTCGACGCGAGTGAGAGACTTTCGTCTCTCGTCTTGCGAGCGCTCGTCAAA

GATCTTGTTAACTCTATTTTAATGTCTTTCGAGCAATCATGTAATAGTTAAAACTTTCAA

CAACGAATCT

>ASV687 GS|100.0|KU948788|SH0941383.09FU;k:Fungi,p:Basidiomycota,c:Cystobasidiomycetes

AAGTCGTAACAAGGTTTCCGTAGGTGAACCTGCGGAAGGATCATTAATGAAATGCAAGGG

CGCTCTTTTTAGAGGTCCAACCTATTCATTTTCTCACACTGTGCACACACTATTCACACC

TTTTTAACACTATAGTATAAGAATGTCAACAGTCTCTTAATTGAGCATCAATTGTAATAA

AACTTTCGGCAACGGATCTCTTGGCTCCC

>ASV688 SS|0.8600|MZ229889|SH0913114.09FU;k:Fungi,p:Ascomycota,c:Lecanoromycetes

AAGTCGTAACAAGGTTTCCGTAGGTGAACCTGCGGAAGGATCATTATCGAGAGATGGAGT

CTAACCGGCCCCACTCTTCACCCGTGTATACCTACCCTTTGTTGCTTTGGCGGGCGGTCG

GTCTTTACCGCCCGACGTCGGTCGGAAGGCTCGCGCCTTCCACTCACCGAACGCCCGCCA

GAGGCCCCCAAAACCCGTCCATCTGTGTCCGAGCGAGTGAAGCAAATAGTTAAAAACTTT

CAACAACGGA

>ASV689 GS|85.8|AF250779|SH0954634.09FU;k:Fungi,p:Ascomycota,c:Lecanoromycetes,o:Caliciales,f:Caliciaceae,g:Buellia,s:Buellia frigida

AAGTCGTAACAAGATTTTCGTAAATGAATCTACGAAAGAATCATTATCGAGAGACGAGAT

CGCTTCGATCTCACTCTTCACTCGTGTCTACTTATCTTTGTTGCTTTGACGAGTCTTCGA

GCTTGATCGTTCGACGCGAGTGAAAGACTTTCGTCTCTCGTCTCGCGAGCGCTCGTCAAA

GACTTTGTTAACTCTATTTTAGTGTCTTTCGAGCAATCATGTAATAGTTAAAACTTTCAA

CAATGAATCT

>ASV690 GS|81.4|AF250779|SH0954634.09FU;k:Fungi,p:Ascomycota,c:Lecanoromycetes,o:Caliciales,f:Caliciaceae,g:Buellia,s:Buellia frigida

AAGTCGTAACAAGATTTTCGTAAATGAATTTGCGAAAAAATCATTATCGAGAGACGAAAT

CGCTTCGATCTCACTTTTCACTCGTATCTACTTATCTTTGTTGCTTTGACGAGTCTTCGA

ACTTAATCGTTCGACGCAAATGAAAGACTTTCGTCTCTCGTTTCGCGAGCGCTCGTCAAA

GATCTTGTTAACTTTGTTTTAGTGTCTTTCGAGCAATCATGTAATAATTAAAACTTTCAA

CAATGAATCT

>ASV691 GS|83.3|AF250779|SH0954634.09FU;k:Fungi,p:Ascomycota,c:Lecanoromycetes,o:Caliciales,f:Caliciaceae,g:Buellia,s:Buellia frigida

AAGTCGTAACAAGATTTTCGTAAATGAACTTGCGAAAAAATCATTATCGAGAGACGAAAT

CGCTTCGATCTCACTCTTCACTCGTATCTACTTATCTTTGTTGCTTTGACGAGTCTTCGA

GCTTGATCGTTCGACGCGAGTGAAAGACTTTCGTCTCTCGTCTCGCGAGCGTTCGTCAAA

GATCTTGTTAACTCTATTTTAGTGTCTTTCAAGCAATCATGTAATAATTAAAATTTTCAA

CAACGAATCT

>ASV692 GS|82.0|AY667583|SH0954634.09FU;k:Fungi,p:Ascomycota,c:Lecanoromycetes,o:Caliciales,f:Caliciaceae,g:Buellia,s:Buellia frigida

AAGTCGTAACAAGATTTTCGTAAATGAATTTGCGAAAGAATCATTATCGAGAGACGAGAT

CGCTTCGATCTCACTCTTCATTTGTATCTACTTATCTTTGTTGCTTTGACGAGTCTTCGA

GCGCAATCGTTCGACGCGAGTGAGAGATTTTCGTCTCTCGTCTTGCGAACGCTCGTCAAA

GACTTTGTTAACTTTGTTTTAGTGTCTTTCGAGCAATCATGTAATAGTTAAAATTTTCAA

CAACGAATCT

>ASV693 GS|78.2|AF224403|SH0903213.09FU;k:Fungi,p:Ascomycota,c:Lecanoromycetes,o:Caliciales,f:Physciaceae,g:Physcia,s:Physcia dubia

AAGTCGTAACAAGGTTTCCGTAGGTGAGAACAGGTTTGCCTCCGGAAAACCCCCGCAACG

ACGTTAAACAAGTGCGTGGTCTGCACCTTAGCAGTGCAGGCAACACCGTTCAAATGCTGA

GAGCACCGAGACACTCGGGGTAACCAGCAGCCAGCACGCTCGCGTCTGGTGCACAGATCA

TTCGACGGTGACCTTATGGTTGAGATATGACCGGCTCCAGGCCTTGAAAGCCCCGGAACC

TGCGGAACCT

>ASV694 GS|100.0|UDB01720327|SH1085256.09FU;k:Rhizaria,p:Cercozoa

AAGTCGTAACAAGGTCTTCGTAGGTGAACCTGCGAAGGGATCATTAACACGTTCCACACC

TCAATTATCAACCCATCTGTGAACCTTTGTTACCGCCGAATGGTCAACTACAGGTGTATG

GCGCATACGATCGACTCAATGATGTTGTAGAATTTATTCTGCATGGTCTAGTAGTATGCC

TCTGTGTCGTTCACACGACAGCTATCCTTGTATGTTGGTCCGGCCACTATATATGCGCTT

CGACGGCCGT

>ASV695 SS|1.0000|AY667583|SH0954634.09FU;k:Fungi,p:Ascomycota,c:Lecanoromycetes,o:Caliciales,f:Caliciaceae,g:Buellia,s:Buellia frigida

AAGTCGTAACAAGGTTTCCGTAGGTGAACCTGCGAAAGGATCATTATCGAGAGATGGGGT

CGCTTCGGCCTCACTCTTCACCCGTGTCTACTTACCTTTGTTGCTTTGGCGGGTCTTCGG

GCTTGATCGTTCGACGCGGGTGGGAGACTTTCGCCTCTCGCCTCGCGAGCGCTCGCCAAA

GGCCCTGTTAACTCTGTTTTAGTGTCTTCCGAGCAATCATGTAATAGTTAAAACTTTCAA

CAACGAATCT

>ASV696 GS|82.8|AY667583|SH0954634.09FU;k:Fungi,p:Ascomycota,c:Lecanoromycetes,o:Caliciales,f:Caliciaceae,g:Buellia,s:Buellia frigida

AAGTCGTAACAAGATTTTCGTAGATGAATTTGCGAAAGAATCATTATCGAGAGACGAAAT

CGCTTCGATCTTACTCTTCACTCGTGTCTACTTATCTTTGTTGCTTTGACGAATCTTTGA

GCTTGATCGTTCGACGCGAGTGAGAGACTTTCGTCTCTCGTCTCGCAAGCGCTCGTCAAA

GACTTTGTTAACTTTGTTTTAGTGTTTTTCGAACAATCATGTAATAGTTAAAACTTTCAA

CAATGAATTT

>ASV697 GS|85.3|AF250779|SH0954634.09FU;k:Fungi,p:Ascomycota,c:Lecanoromycetes,o:Caliciales,f:Caliciaceae,g:Buellia,s:Buellia frigida

AAGTCGTAACAAGATTTTCGTAAATGAATTTGCGAAAGAATCATTATCGAGAGATGAGAT

CGCTTCGATCTCACTCTTCACTCGTATTTACTTATCTTTGTTGCTTTGACGAGTCTTCGA

ACTTGATCGTTCGACGCGAGTAAGAGACTTTCGTCTCTCGCCTCGCGAGCGCTCGTCAAA

GACTCTATTAACTCTGTTTTAGTGTTTTTCGAGCAATCATGTAATAGTTAAAACTTTCAA

CAACGGATCT

>ASV698 SS|1.0000|KF823589|SH1091255.09FU;k:Fungi

AAGTCGTAACAAGGTTTCCGTAGGTGAACCTGCGGAAGGATCATTAGTGATTGGTCTCCG

GACCATTTTCATCATCCACAAACCTCTGTGAACCTGTCGGCCCTCGGGCCAGCTTTTCCA

AACAAAGTGTAACGAACGTGTGATATTATAATCTAGTCTAGTAAAACTTTCAACAACGGA

TCTCTTGGCTCTC

>ASV699 GS|0.0|None;No hit

GTGTCAGCAGCCGCGGTAATACGAAGGGGGCTAGCGTTGCTCGGAATGACTGGGCGTAAA

GGGCGCGTAGGCGGATTGATCAGTCAGATGTGAAATTCCTGGGCTTAACCTGGGGGCTGC

ATTTGAGACGGTTAGTCTAGAGTGTGAAAGAGGGTCGTGGAATTCCCAGTGTAGAGGTGA

AATTCGTAGATATTGGGAAGAACACCGGTGGCGAAGGCGGCGACCTGGTTCATAACTGAC

GCTGAGGCGC

>ASV700 GS|100.0|AY603122|SH0998157.09FU;k:Fungi,p:Ascomycota,c:Lecanoromycetes,o:Umbilicariales,f:Umbilicariaceae,g:Umbilicaria,s:Umbilicaria decussata

AAGTCGTAACAAGGTTTCCGTAGGTGAACCTGCGGAAGGATCATTACTGAGATAGGGTCC

TCCGGGCCCGAACCTCCAACCCTTTGCGTACCTTACCTTTGTTGCTTTGGCGGGCCCGCT

GGGGAAACCCACCGCCGGCGTCGAGCCGGTGAGCGCCCGCCGGAGGCCCCCAAAACTCCG

TCTTGTCAGTGTCGTCTGAGTACTATACAATAGCTAAAACTTTCAACAACGGATCTCTTG

GTTCTG

>ASV701 GS|99.5|AM697878|SH1302893.09FU;k:Fungi,p:Ascomycota,c:Lecanoromycetes,o:Teloschistales,f:Teloschistaceae,g:Xanthoria,s:Xanthoria mawsonii

AAGTCGTAACAAGGTTTCCGTAGGTGAACCTGCGGAAGGATCATTATCGAGAGAGGGACT

TCGCGTCCCCCGGGGGGTCCGGCCCCCCCACCTCTTCAACCCTGTGTATATCAATCCATG

TTGCTTTGGCGAGCGCCGGGGAGCGATTCCCGGCCCTGGCTTCGGTCAGTCAGCCCTCGT

CAGAGGCCCATCCAAATTCTGTTTCAGTGACGTCCGAGTATACCAGCAAATAAATTAAAA

CTTTCAACAA

>ASV702 GS|82.8|AF250779|SH0954634.09FU;k:Fungi,p:Ascomycota,c:Lecanoromycetes,o:Caliciales,f:Caliciaceae,g:Buellia,s:Buellia frigida

AAGTCGTAACAAGATTTTCGTAAATGAATTTGCGAAAAAATCATTATCGAGAGACGAGAT

CGCTTCGATCTCATTCTTCACTCGTATCTATTTATCTTTGTTGCTTTGACGAGTCTTCGA

ACTTGATCGTTCGACGCGAATGAGAGACTTTCGTCTCTCGTCTCGCGAGCGCTCGTCAAA

GATTCTATTAACTTTGTTTTAGTGTCTTTCGAACAATCATGTAATAGTTAAAACTTTCAA

CAATGAATCT

>ASV703 GS|88.2|AF250779|SH0954634.09FU;k:Fungi,p:Ascomycota,c:Lecanoromycetes,o:Caliciales,f:Caliciaceae,g:Buellia,s:Buellia frigida

AAGTCGTAACAAGATTTTCGTAGATGAATTTGCGAAAGAATCATTACCAAGAGACGAGGT

CGCTTCGATTCCACTCTTCACTCGTGTCTACTTATCTTTGTTGCTTTGGCGAGTCTTCGA

GCTTGATCGTTCGACGCGAGTGGGAGGCTTTCGTTTCTCGTCTCGCGAGCGCTCGTCAAA

GACTTTGTTAACTTTGTTTTAGTGTCTTCCAAGCAATCATGTAATAGTTAAAACTTTCAA

CAACGAATCT

>ASV704 GS|78.2|MZ224659|SH0916480.09FU;k:Fungi,p:Ascomycota,c:Lecanoromycetes,o:Caliciales,f:Caliciaceae,g:Buellia,s:Buellia insignis

AAGTCGTAACAAAGTTTCCGTAGGTGAACCTGCGGAAGAATCATTATCAAGAGACGAAGT

CTAACCGGCCCCACTCTTCACCCGTGTATATCTACCCTTTATTACTTTGGCGGGCGATCG

GTCTTTACCGCCCGACGTCGGTCGGAAGACTCGCGCCTTCCACTCACCGAACGCCCGCCA

GAGATCTCCAAAACCCGTTCATTTGTGTTCGAACGAGTGAAACAAATAGTTAAAAATTTT

CAACAACGGA

>ASV705 GS|86.3|AF250779|SH0954634.09FU;k:Fungi,p:Ascomycota,c:Lecanoromycetes,o:Caliciales,f:Caliciaceae,g:Buellia,s:Buellia frigida

AAGTCGTAACAAGATTTTCGTAGATGAACTTGCGAAAAAATCATTATCGAAAGACGAGAT

CGCTTCGATCTCACTTTTCACTCGTGTCTACTTATCTTTGTTGCTTTGACGAGTCTTCGA

GCTTGATCGTTCGACGCGAGTGAGAGGCTTTCGTCTCTCGCCTCGCGAGCGCTCGTCAAA

GACTTTGTTAACTTTGTTTTAGTGTCTTTCGAGCAATCACGTAATAGTTAAAACTTTCAA

CAACGAATCT

>ASV706 SS|1.0000|MF138063|SH0954212.09FU;k:Fungi,p:Ascomycota

AAGTCGTAACAAGGTTTCCGTAGGTGTAAGTAATCCAACGTCCCAAACATTCACCATCCA

ATGCGTGATCATGCTAACATGTGATTTAGGAACCTGCGGAAGGATCATTAAAGAGCAAGG

GTCTTCTAGGCCCGACCTCCAACCCTATGTGTACCTACCTTTGTTGCTTTGGCGGGCCCG

TCGGGTGACCCACCGGTGGCCTCTGGCTCCCGAGTGCCCGTCAGAGACCCATCAAAACCC

GTTAATTGTG

>ASV707 GS|0.0|None;No hit

AAGTCGTAACAAGATTTTCGTAAATGAATTTGCGAAAAAATCATTATCGAGAGACGAAAT

CGCTTCGATCTCACTTTTCATTCGTATCTACTTATCTTTATTACTTTGACGAGTCTTCGA

ACTTAATCGTTCGACGCGAATGAGAGACTTTCGTCTCTCGTTTTACGAACGCTCGTCAAA

GACTTTGTTAACTTTATTTTAATGTTTTTCGAACAATCATATAATAATTAAAACTTTCAA

CAACGAATCT

>ASV708 SS|1.0000|UDB02845207|SH0910250.09FU;k:Fungi

AAGTCGTAACAAGGTTTCCGTAGGTGAACCTGCGGAAGGATCATTACCGAGTTAGGGTCT

TTCAGGCCCGACCTCCAACCCTTTGTTTAAACTACCATGTTGCTTTGGCGGGCCCGTCCT

TCGGGACCGCTGGGGGCCTCACAGCCCCTGGTCAGCGCTCGCCAGTAGCCTTCTCAAACT

CTTTATAAACTATGTTGTCTGAGTATAAATATAAATCGTTAAAACTTTCAACAACGGATC

TCTTGGTTCT

>ASV709 GS|82.8|AF250779|SH0954634.09FU;k:Fungi,p:Ascomycota,c:Lecanoromycetes,o:Caliciales,f:Caliciaceae,g:Buellia,s:Buellia frigida

AAGTCGTAACAAAGTTTTCGTAAGTGAATTTGCGAAAGAATTATTATCGAGAGACGAAAT

CGCTTCGATCTTACTCTTCACTCGTGTCTACTTATTTTTGTTGCTTTGACGAGTTTTCGA

GCTTGATCGTTCAACGCGAGTGAGAGACTTTCGTCTCTCGTTTCGCGAACGCTCGTCAAA

GACTTTGTTAACTTTGTTTTAGTGTCTTTCGAGCAATCATGTAATAATTAAAACTTTCAA

CAACGAATCT

>ASV710 SS|1.0000|MK208763|SH0942716.09FU;k:Fungi,p:Ascomycota,c:Lecanoromycetes

AAGTCGTAACAAGGTTTCCGTAGGTGAACCTGCGGAAGGATCATTACCGAGAGACGGGGT

CGCTTCGGCCCCACTCTTCACCCGTGTCTACTTACCTTTGTTGCTTTGGCGCGCCCTGGG

GTTCCGCCCCACGCCGGCCTCAGGCCTTCGGCTTGGGCTGGTGAGAGCCCGTCAGAGGCC

CATTCTAATCCTTATATCAGTGATGTCCGAGTACAATTTTAATAAATAAAACTTTCAACA

ACGGATCTCT

>ASV711 GS|0.0|None;No hit

AAGTCGTAACAAGATTTTCGTAAATGAATCTACGAAAAAATCATTATCGAGAGACGAAAT

CGCTTCGATCTCACTCTTTATTCGTATCTACTTATTTTTGTTACTTTGACGAATCTTCGA

ACTTGATCGTTCGATGCGAGTAAGAGACTTTCGTCTCTCGTTTCGCAAGCGCTCGTTAAA

GACTTTGTTAATTTTGTTTTAATGTTTTTCGAGCAATCATGTAATAATTAAAATTTTTAA

TAACGAATCT

>ASV712 GS|86.3|AF250779|SH0954634.09FU;k:Fungi,p:Ascomycota,c:Lecanoromycetes,o:Caliciales,f:Caliciaceae,g:Buellia,s:Buellia frigida

AAGTCGTAATAAGATTTTCGTAGATGAATTTGTGAAAGAATCATTATCGAGAGACGAGGT

CGCTTCGATCTCACTCTTCACTCGTGTCTACTTATCTTTGTTACTTTGACGAATTTTCGA

GCTTGATCGTTCGACGCGAGTGAGAGGCTTTCGTCTCTCGTCTCGCGAGCGCTCGTCAAA

GACTTTGTTAACTCTGTTTTAGTGTCTTTCGAGCAATTATGTAATAGTTAAAACTTTCAA

CAACGAATCT

>ASV713 GS|82.8|AY667583|SH0954634.09FU;k:Fungi,p:Ascomycota,c:Lecanoromycetes,o:Caliciales,f:Caliciaceae,g:Buellia,s:Buellia frigida

AAGTCGTAACAAGATTTTCGTAGATGAATCTACGAAAGAATCATTATCGAGAGACGAGGT

CGCTTCGACTTCACTTTTCACTCGTACTTACTTATCTTTGTTGCTTTGACGAGTCTTCGA

GCTTGATCGTTCGATGCGAATGAGAGACTTTCGTCTCTCGTCTTGCGAGCGCTCATCAAA

GATTCTATTAACTTTGTTTTAGTGTTTTTCGAGCAATCATGTAATAGTTAAAACTTTCAA

CAACGAATCT

>ASV714 GS|86.0|AY667583|SH0954634.09FU;k:Fungi,p:Ascomycota,c:Lecanoromycetes,o:Caliciales,f:Caliciaceae,g:Buellia,s:Buellia frigida

AAGTCGTAACAAGATTTTCGTAGATGAACTTGCGAAAGAATCATTATCAAGAGACGAGAT

CGCTTCGATCTCACTCTTCACTCGTGTTTACTTATCTTTGTTGCTTTGACGAGTCTTCGA

GCTTGATCGTTCGACGCGAGTGAGAGACTTTCGTCTATCGTCTCGCGAGCGCTCGTCAAA

GACTCTGTTAACTTTGTTTTAGTGTCTTTCGAGCAATCATGTAATAGTTAAAACTTTCAA

CAACGAATCT

>ASV715 GS|81.4|AF250779|SH0954634.09FU;k:Fungi,p:Ascomycota,c:Lecanoromycetes,o:Caliciales,f:Caliciaceae,g:Buellia,s:Buellia frigida

AAGTCGTAACAAGATTTTCGTAAATGAATTTGCGAAAAAATCATTATCGAGAGACGAAAT

CGCTTCGACTTCACTCTTCATTCGTGTCTACTTATCTTTGTTGCTTTGACGAATCTTCGA

ACTTGATCATTCGATGCGAGTGAGAGACTTTCGTCTCTCGTTTCGCGAGCGCTCGTCAAA

GACTTTGTTAACTCTATTTTAGTGTTTTTCGAGTAATCATGTAATAATTAAAATTTTCAA

CAACGAATCT

>ASV716 GS|85.8|AF250779|SH0954634.09FU;k:Fungi,p:Ascomycota,c:Lecanoromycetes,o:Caliciales,f:Caliciaceae,g:Buellia,s:Buellia frigida

AAGTCGTAACAAGATTTTCGTAGATGAATCTACGAAAAAATCATTATCGAGAGACGAGAT

CGCTTCGACCTCACTCTTCACTCGTGTCTACTTACCTTTGTTGCTTTGACGAGTCTTCGA

ACTTAATCGTTCGACGCGAATGAAAGACTTTCGCCTCTCGTTTCGCGAGCGCTCGTCAAA

GACTCTATTAACTTTGTTTTAGTGTCTTTCGAGCAATCATGTAATAGTTAAAACTTTCAA

CAACGAATCT

>ASV717 SS|0.8400|AY081152|SH1300501.09FU;k:Fungi,p:Ascomycota,c:Lecanoromycetes,o:Teloschistales,f:Teloschistaceae

AAGTCGTAACAAGGTTTCCGTAGGTGAACCTGCGGAAGGATCATTACCGAGTTAGGGTCT

CTTCGGAGCCCGAACCTCCCAACCCTTTGTCTAATTTACCTTGTCGTTGCTTCGGCGAGC

GTCGGGGCGTCCGCGCCCCGGCCCCGGCTTCGGTCGGTGAGCTCTCGCAGAGGCCTATCT

TTATTCTGTTTTGCAGTGACGTCCGAGAATACCAATATAATCAATCAAAACTTTCAACAA

CGGATCTCTT

>ASV718 GS|100.0|KF309971|SH0991911.09FU;k:Fungi,p:Ascomycota,c:Dothideomycetes,o:Mycosphaerellales,f:Teratosphaeriaceae,g:Meristemomyces,s:Meristemomyces frigidus

AAGTCGTAACAAGGTCTCCGTAGGTGAACCTGCGGAGGGATCATTACTGAGTGAGGGCGC

GAGCCCGACCTCCAACCCTTTGTCACCCGACTCTGTTGCCTCGGGGGCGACCCGGCCTTC

GGGCGTCGGGGCCCCCGGCGGACACGTAACCCTGCATCTTTGCGTCCGAGTAAACATTTG

AATCAATCAAAACTTTTAACAACGGATCTCTTGGTTCTG

>ASV719 GS|97.9|MW991424|SH0964445.09FU;k:Fungi,p:Ascomycota,c:Arthoniomycetes,o:Lichenostigmatales,f:Phaeococcomycetaceae

AAGTCGTAACAAGGTCTCCGTAGGTGAACCTGCGGAGGGATCATTAATGAGATAGGGTCT

TCACGGCCCGACCTCCAACCCAATGTCTACCATACCTCTGTTGCCTCGGCGGACCGCCGG

CGCCCTTTATTGGGCGTCGCCGTCGGCTCTAGGGCTCTCGAGCGCCCGCCGCAGGACCGA

TTAAACTCTTTTTAAAACAAGTCTTCTGAGTGGGAAATTAAATTTATTAAAACTTTTAAC

AACGGATCTC

>ASV720 GS|100.0|OL614722|SH0994206.09FU;k:Fungi,p:Basidiomycota,c:Tremellomycetes,o:Tremellales,f:Bulleribasidiaceae,g:Vishniacozyma,s:Vishniacozyma carnescens

AAGTCATAACAAGGTTTCCGTAGGTGAACCTGCGGAAGGATCATTAATAATGCTCTCTGG

CTTCGGTCAGTTGAGTTCAATGAGTGCCTTCTCTTCGGAGTTGGCCATCCATACACACCG

TGAACTGTGGCTTCGGCCATCACAAACTGTTAGTAATGAATGTAATATCATAACAAAAAC

AAAACTTTTAACAACGGATCTCTTGGCTCTC

>ASV721 GS|0.0|None;No hit

AAATCGTAACAAGATTTTCGTAAATAAACTTGCGAAAAAATCATTATTGAGAGACGAAAT

CGCTTCGATTTTACTCTTCACTCGTATCTACTTATCTTTGTTGCTTTAACGAATTTTCGA

ATTTAATCGTTCGACGCGAATGAGAGACTTTCGTCTCTCGTTTTGCGAACGCTCGTCAAA

GACTTTGTTAATTCTATTTTAGTGTCTTTCGAGCAATCATGTAATAATTAAAATTTTTAA

CAATAAATCT

>ASV722 GS|0.0|None;No hit

AAGTCGTAACAAGATTTTCGTAAATGAATTTACGAAAAAATCATTATCGAGAGACGAAAT

CGCTTCGATCTCACTCTTCACTCGTATCTATTTATTTTTGTTGCTTTAACGAGTCTTTGA

ACTTGATCGTTCAACGCGAGTGAGAGACTTTCGTCTCTCGTTTCGCGAGCGCTCGTTAAA

GATTTTGTTAACTTTGTTTTAATATCTTTCGAACAATCATGTAATAATTAAAACTTTTAA

CAACGAATCT

>ASV723 SS|1.0000|AY667583|SH0954634.09FU;k:Fungi,p:Ascomycota,c:Lecanoromycetes,o:Caliciales,f:Caliciaceae,g:Buellia,s:Buellia frigida

AAGTCGTAACAAGGTTTCCGTAGGTGAACCTGCGAAAGGATCATTATCGAGAGACGGGGT

CGCTTCGACCCCACTCTTCACTCGTGTCTACTTACCTTTGTTGCTTTGGCGGGCCTTCGG

GCTTGATCGTTCGACGCGGGTGGGAGGCTTTCGCCTCTCGCCTCGCGAGCGCCCGTCAAA

GGCCCTGTTAACTTTGTTTTAGTGTCTTCCGAGCAACCATGTAATAGTTAAAACTTTCAA

CAACGAATCT

>ASV724 GS|86.8|AF250779|SH0954634.09FU;k:Fungi,p:Ascomycota,c:Lecanoromycetes,o:Caliciales,f:Caliciaceae,g:Buellia,s:Buellia frigida

AAGTCGTAACAAGATTTTCATAGATGAACCTACGAAAGAATCATTATCGAGAGACGAGAT

CGCTTCGATCTCACTCTTCACTCGTGTCTACTTATCTTTGTTGCTTTGACGAGTCTTCGA

GCTTGATCGTTCGACGCGAGTGAGAGGCTTTCGTCTCTCGTCTCGCGAGCGTTCGTCAAA

GATCGTGTTAACTCTGTTTTAGTGTCTTTCGAGCAATCATGTAATAGTTAAAACTTTTAA

CAACGAATCT

>ASV725 SS|0.9900|KF274227|SH0911134.09FU;k:Fungi

AAGTCGTAACAAGGTTTCCGTAGGTGAACCTGCGGAAGGATCATTACCGAGTTAGGGTCC

TCTGGGCTCGATCTCCAACCCTGTGTCTAAATAACCACGTTGCTTTGGCGGGCCCGCCTG

TAATGGGCCGCCGGGGCCGCCTTCGGCCCCCTGGTCCGCGCCTGCCAGTAGCCATCTCAA

ACTCTTCTTAATCGTGACGTCTGAGTAAAAATTATAAATTAAACAAAACTTTCAACAACG

GATCTCTTGG

>ASV726 GS|87.7|AF250779|SH0954634.09FU;k:Fungi,p:Ascomycota,c:Lecanoromycetes,o:Caliciales,f:Caliciaceae,g:Buellia,s:Buellia frigida

AAGTCGTAACAAGATTTTCGTAAATGAACTTGCGAAAGAATTATTATCGAGAGACGAGAT

CGCTTCGATCTCACTCTTCACTCGTGTCTACTTATCTTTGTTGCTTTGACGAATCTTCGA

GCTTGATCGTTCGACGCGAGTGAGAGGCTTTCGCCTCTCGTCTCGCGAGCGCTCGTCAAA

GACTCTATTAACTTTGTTTTAGTGTCTTTCGAGCAATCATGTAATAGTTAAAACTTTCAA

CAACGGATCT

>ASV727 GS|84.3|AF250779|SH0954634.09FU;k:Fungi,p:Ascomycota,c:Lecanoromycetes,o:Caliciales,f:Caliciaceae,g:Buellia,s:Buellia frigida

AAGTCGTAACAAGATTTTCGTAAATGAATTTGCGAAAGAATCATTATCGAGAGATGAGAT

CGCTTCGATCTCACTCTTCACTCGTATTTACTTATCTTTGTTGCTTTGACGAGTCTTCGA

ACTTGATCGTTCGACGCGAGTAAGAGACTTTCGTCTCTCGCCTCGCGAGCGCTCGTCAAA

GACTCTATTAACTCTGTTTTAGTGTTTTTCGAGCAATCATGTAATAGTTAAAACTTTCAA

CAATGAATCT

>ASV728 GS|97.6|UDB0479157|SH0953972.09FU;k:Fungi,p:Ascomycota,c:Dothideomycetes,o:Botryosphaeriales,f:Botryosphaeriaceae,g:Dothiorella

AAGTCGTAACAAGGTTTCCGTAGGTGAACCTGCGGAAGGATCATTACCGGGCTCAGGGGG

GGAAACCCCCCGAACTCCCAACCCTTGCTTACCTACCACGTTGCTTCGGCGGGCTCGCGC

CCGCCGGAGGTCAATCAAACTATTTTATACCATAGTCAGAGCGAATGCGAAAATAAGTAA

AAACTTTCAACAACGGATCTCTTGGTTCTG

>ASV729 SS|0.8800|MK948452|SH0954310.09FU;k:Fungi,p:Ascomycota,c:Lecanoromycetes,o:Acarosporales,f:Acarosporaceae,g:Acarospora

AAGTCGTAACAAGGTTTCCGTAGGTGAACCTGCGGAAGGATCATTACCGAGAGACGGGGT

CGCTTCGGCCCCACTCTTCACCCGTGTCTACTTACCTTTGTTGCTTTGGCGGGCCCGTTG

GGGCGACCCACCGGTGGCCTTTGGCTCCCGAGTGCCCGTCAGAGATCCATCAAAACCTTT

CAATTGTGTCGTCTGAGTACCAACATAATAATTAAAACTTTCAACAACGGATCTCTTGGT

TCTG

>ASV730 SS|0.9600|JF519086|SH0993791.09FU;k:Fungi,p:Ascomycota,c:Dothideomycetes,o:Mycosphaerellales,f:Teratosphaeriaceae

AAGTCGTAACAAGGTCTCCGTAGGTGAACCTGCGGAGGGATCATTACCGAGCGAGGGCCC

CCGCGCCCGACCTCCAACCCTTTGTCGATTCATATCTGTTGCCTCGGGGGGCGACCCGGC

CGTCCGCGGGCGGGCGTCCCCCAGAGGGTCAATCAACTCTGCATCTTTGCGTCGAGTATT

GAATACAAATCAATCAAAACTTTTAACAACGGATCTCTTGGTTCTG

>ASV731 GS|84.8|AF250779|SH0954634.09FU;k:Fungi,p:Ascomycota,c:Lecanoromycetes,o:Caliciales,f:Caliciaceae,g:Buellia,s:Buellia frigida

AAGTCGTAACAAGATTTTCGTAGATGAATTTGCGAAAGAATCATTATCGAGAGACGAGAT

CGCTTCGATTTCATTCTTCATTCGTGTCTACTTATCTTTGTTGCTTTGACGAGTCTTCGA

GCTTGATCGTTCGACGCGAGTGAGAGACTTTCGTCTCTCGTCTCGCGAGCGCTCGTCAAA

GACTTTGTTAACTCTATTTTAGTGTCTTTCGAGCAATCATGTAATAGTTAAAATTTTCAA

CAACGAATCT

>ASV732 GS|76.1|MZ229889|SH0913114.09FU;k:Fungi,p:Ascomycota,c:Lecanoromycetes,o:Caliciales,f:Caliciaceae,g:Dimelaena

AAGTCGTAACAAAGTTTTCGTAAGTGAATTTGCGAAAGGATCATTATCGAGAGACGGAGT

CTAACCGGCCCCACTCTTCACTCGTGTATATCTATTCTTTGTTGCTTTGGCGAGCGGTCG

GTCTTTACCGCCCGACGTCGGTCGGAAGATTCGCGTCTTTCACTCACTGAACGCCCGCTA

GAGGTTCTCAAAAGTCGTTCATCTGTGTTCGAGCGAGTGAAGCAAATAATTAAAAACTTT

TAACAACGGA

>ASV733 GS|86.3|AF250779|SH0954634.09FU;k:Fungi,p:Ascomycota,c:Lecanoromycetes,o:Caliciales,f:Caliciaceae,g:Buellia,s:Buellia frigida

AAGTCGTAACAAGGTTTTCGTAGATGAATCTACGAAAAAATCATTATCGAGAGACGAGAT

CGCTTCGATTTCACTCTTCACTCGTATCTACTTATCTTTGTTGCTTTGACGAGCCTTCGA

GCTTGATCGTTCGACGCGAGTGAGAGACTTTCGCCTCTCGTTTTGCGAGCGCTCGTCAAA

GACCTTGTTAACTTTGTTTTAGTGTCTTTCGAGCAATCATGTAATAGTTAAAACTTTCAA

CAACGAATCT

>ASV734 GS|86.8|AF250779|SH0954634.09FU;k:Fungi,p:Ascomycota,c:Lecanoromycetes,o:Caliciales,f:Caliciaceae,g:Buellia,s:Buellia frigida

AAGTCGTAATAAGATTTTCGTAGATGAATTTGTGAAAGAATCATTATCGAGAGACGAGGT

CGCTTCGATCTCACTCTTCACTCGTGTCTACTTATCTTTGTTACTTTGACGAATTTTCGA

GCTTGATCGTTCGACGCGAGTGAGAGGCTTTCGTCTCTCGTCTCGCGAGCGCTCGTCAAA

GACTTTGTTAACTCTGTTTTAGTGTCTTTCGAGCAATTATGTAATAGTTAAAACTTTCAA

CAACGGATCT

>ASV735 GS|82.8|AF250779|SH0954634.09FU;k:Fungi,p:Ascomycota,c:Lecanoromycetes,o:Caliciales,f:Caliciaceae,g:Buellia,s:Buellia frigida

AAGTCGTAACAAAATTTTCGTAGATGAATTTGCGAAAGAATCATTATCAAGAGACGAGAT

CGCTTCGATCTCACTCTTCACTCGTGTCTACTTATCTTTATTGCTTTGACGAGTCTTCGA

ACTTGATCGTTCGACGCGAGTGAGAGACTTTCGTCTCTCGTCTCGCGAGCGCTCGTCAAA

GATTCTATTAACTTTGTTTTAGTGTCTTTCGAGCAATCATGTAATAGTTAAAACTTTCAA

TAATAAATTT

>ASV736 GS|84.8|AF250779|SH0954634.09FU;k:Fungi,p:Ascomycota,c:Lecanoromycetes,o:Caliciales,f:Caliciaceae,g:Buellia,s:Buellia frigida

AAGTCGTAACAAGATTTTCGTAGATGAATCTACGAAAGAATCATTATCGAGAGACGAGAT

CGCTTCGATCTCACTCTTCACTCGTGTTTACTTATTTTTGTTGCTTTGACGAGTCTTCGA

GCTTGATCGTTCGACGCGAGTGAGAGATTTTCGTCTCTCGTCTCGCGAGCGCTCGTCAAA

GACTTTGTTAACTTTGTTTTAGTGTCTTTCGAGCAATCATATAATAGTTAAAACTTTCAA

CAACGAATCT

>ASV737 GS|99.5|UDB03311447|SH1045652.09FU;k:Fungi,p:Ascomycota,c:Leotiomycetes,o:Helotiales

AAGTCGTAACAAGGTCTCCGTAGGTGAACCTGCGGAGGGATCATTACAGAGTTCATGCCC

TTCGGGGTAGACCTCCCACCCGTGTGAACGATACCTTTGTTGCTTTGGTTGGCCGCGGGC

CCGCCCGCTACCGGCTCTGCTAGTACGCGCCAACCAGAGGCCCTAACTCCTGTCTTCTGT

GCCGTCTAAGTCCCATATAATCGTTAAAACTTTCAACAACGGATCTCTTGGTTCTG

>ASV738 SS|0.9400|MT809481|SH1325656.09FU;k:Fungi,p:Ascomycota

AAGTCGTAACAAGGTTTCCGTAGGTGAACCTGCGGAAGGATCATTACCGAGCTAGGGTCT

CTGGCCCGACCTCCAACCCTCTGTGTACCTACCTTTGTTGCTTTGGCGGGCCGTCGGGCC

ACCATGGCCTGCCGAGGACCTCTGAGGTCCGCGAGCGCCCGTCAGAAGCCTTCCTAAAAA

CTCCCATGTACAGTTGCAGTCTGAGTAGGCATTTAATCTAGTTAAAACTTTCAACAACGG

ATCTCTTGGT

>ASV739 GS|86.3|AF250779|SH0954634.09FU;k:Fungi,p:Ascomycota,c:Lecanoromycetes,o:Caliciales,f:Caliciaceae,g:Buellia,s:Buellia frigida

AAGTCGTAACAAGATTTTCGTAAATGAATTTGCGAAAGAATCATTATCGAGAGATGAGAT

CGCTTCGATCTCACTCTTCACTCGTATTTACTTATCTTTGTTGCTTTGACGAGTCTTCGA

ACTTGATCGTTCGACGCGAGTAAGAGACTTTCGTCTCTCGCCTCGCGAGCGCTCGTCAAA

GACTCTATTAACTCTGTTTTAGTGTTTTCCGAGCAACCATGTAATAGTTAAAACTTTCAA

CAACGGATCT

>ASV740 SS|1.0000|AF250779|SH0954634.09FU;k:Fungi,p:Ascomycota,c:Lecanoromycetes,o:Caliciales,f:Caliciaceae,g:Buellia,s:Buellia frigida

CTTTTTCCTTTCTCTTCCTTCCCCTTCTTCCCCCTTTTTCCGTATTTTCCCCTGCGGACT

TCTCCTTACCGATCTCCTTGGTCGCTTCTTCCCCACTCTTCCCCCTTGTCTACTTCCCTT

TGTTGCTTTGGCGGGCCTTCGGGCTTGACCTTTCTCCGCGGGTGGGAGGCTTTCGCCTCT

CGCCTCTCTCTCGCCCGCCAAAGGCCCTGTTAACTCTGTTTTAGTGTCTTCCGAGCAACC

ATGTAATAGT

>ASV741 GS|82.4|AY667583|SH0954634.09FU;k:Fungi,p:Ascomycota,c:Lecanoromycetes,o:Caliciales,f:Caliciaceae,g:Buellia,s:Buellia frigida

AAGTCGTAACAAGATTTTCGTAGATGAACTTGCGAAAGAATCATTATCGAGAGACGAAAT

CGCTTCGACTTCACTTTTCACTCGTATTTACTTATCTTTATTGCTTTGACGAGTCTTCGA

GCTTGATCGTTCGACGCGAGTGAAAGACTTTCGTCTCTCGTTTCGCGAGCGTTCGTCAAA

GATCTTGTTAACTTTGTTTTAGTGTCTTTCGAGCAATCATGTAATAATTAAAACTTTCAA

CAATGAATTT

>ASV742 SS|1.0000|AF250779|SH0954634.09FU;k:Fungi,p:Ascomycota,c:Lecanoromycetes,o:Caliciales,f:Caliciaceae,g:Buellia,s:Buellia frigida

CTTTTTCCTTTCTCTTCCTTCCCCTTCTTCCCCCTTTTTCCGTATTTTCCCCTGCGGACT

TCTCCTTACCGATCTCCTTGGTCGCTTCTTCCCCACTCTTCCCCCTTGTCTACTTACCTT

TGTTGCTTTGGCGGGCCTTCGGGCTTGACCGTTCGCCGCGGGTGGGAGGCTTTTGCCTCT

CGCCTCGCGCGCGCCCGCCAAAGGCCCTGTTAACTCTGTTTTAGTGTCTTCCGAGCAACC

ATGTAATAGT

>ASV743 GS|0.0|None;No hit

AAGTCGTAACAAGGTTTTCGTAGGTGAACCTGCGGAAGGATCATTACCAGATGGGGCCTC

CGGGCCTTTTTCTCCTTACCACGTGCACCGAGTTGTCGGGAGATGTTTGTTTCCCGACCA

ATTCTTCACGCTTGTTTTGTTGTCTGAGTCGAATGACCATAAATTGAAAAGAAAACTATG

AACAACGGATCTCTAGGCTCTT

>ASV744 GS|74.8|MN615681|SH0916491.09FU;k:Fungi,p:Ascomycota,c:Lecanoromycetes,o:Caliciales,f:Caliciaceae,g:Buellia,s:Buellia insignis

AAGTCGTAACAAGATTTTCGTAAATGAATTTGCGGAAGAATCATTATTGAGAGACGGAGT

CTAACCGGCCCCACTTTTCACCCGTGTATATTTATTCTTTATTGTTTTAACGGGCGATCG

GTTTTTATCGCCCGACGTCGGTCGAAAGACTCGCGCCTTCCACTCATCGAACGCTCGTCA

AAGATCTCCAAAAGTCGTTCATCTGTGTTCGAGCGAGTGAAGTAAATAGTTAAAAACTTT

CAACAACGGA

>ASV745 GS|78.5|MZ229889|SH0913114.09FU;k:Fungi,p:Ascomycota,c:Lecanoromycetes,o:Caliciales,f:Caliciaceae,g:Dimelaena

AAGTCGTAACAAGGTTTCCGTAGATGAACCTGCGGAAGAATCATTATCGAGAGACGAAGT

CTAACCGGCCCCACTCTTCATCCGTGTATATCTACTCTTTATTGCTTTGGCGGGCGGTCG

ATCTTTACCGCCCGACGTCGGTCGGAAGACTCGCGCCTTTCACTCACCGAACGCCCGCCA

AAGACTCCCAAAAACCGTTCATCTATGTCCGAGCGAGTGAAGCAAATAATTAAAAACTTT

CAACAACGGA

>ASV746 GS|84.3|AF250779|SH0954634.09FU;k:Fungi,p:Ascomycota,c:Lecanoromycetes,o:Caliciales,f:Caliciaceae,g:Buellia,s:Buellia frigida

AAGTCGTAACAAGATTTTCGTAAATGAATTTGCGAAAGAATCATTATCGAGAGACGAGAT

CGCTTCGATCTCACTCTTCACTCGTGTCTACTTATCTTTGTTGCTTTGACGAGTCTTTGA

ACTTGATCGTTCGACGCGAGTGAAAGACTTTCGTCTCTCGTTTCGCGAGCGTTCGTCAAA

GACTCTATTAACTTTGTTTTAGTGTCTTTCGAGCAATCATGTAATAGTTAAAACTTTCAA

CAACGAATCT

>ASV747 SS|1.0000|AF250779|SH0954634.09FU;k:Fungi,p:Ascomycota,c:Lecanoromycetes,o:Caliciales,f:Caliciaceae,g:Buellia,s:Buellia frigida

AAGTCGTAACAAGGTTTCCGTAGATGAATCTACGAAAGAATCATTATCGAGAGACGAGGT

CGCTTCGACCCCACTCTTCACTCGTGTCTACTTACCTTTGTTGCTTTGACGAGCCTTCGA

GCTTGATCGTTCGACGCGAGTGGGAGGCTTTCGCCTCTCGCCTCGCGAGCGCCCGCCAAA

GGCCCTGTTAACTCTGTTTTAGTGTCTTCCGAGCAACCATGTAATAGTTAAAACTTTCAA

CAACGGATCT

>ASV748 SS|0.8500|UDB07672048|SH0991995.09FU;k:Fungi,p:Ascomycota,c:Lecanoromycetes,o:Trapeliales,f:Trapeliaceae,g:Trapelia

AAGTCGTAACAAGGTTTCCGTAGGTGAACCTGCGGAAGGATCATTACCGAGATAGGGTCC

CCCGGGCCCGACCCTCCACCCGCTGCGTACCTACCTTTTGTTGCTTTGGCGGGCCGCGGG

GCCCCGGGCCCCCCGTCGACCCCGGTGGGCGAGCGCCCGCCAGAGACCCCCCCAACCCGG

TTGATCAGTGACGTCCGAGCCCCGATGAAAATCAATTAAAACTTTCAACAACGGATCTCT

TGGTTCTG

>ASV749 GS|97.1|AF250779|SH0954634.09FU;k:Fungi,p:Ascomycota,c:Lecanoromycetes,o:Caliciales,f:Caliciaceae,g:Buellia,s:Buellia frigida

AAGTCGTAACAAGATTTCCGTAGGTGAACCTGCGAAAGGATCATTACCGAGAGACGAGGT

CGCTTCGACCCCACTCTTCACCCGTGTCTACTTACCTTTGTTGCTTTGACGGGCCTTCGG

GCTTGACCGTTCGACGCGGGTGGAAGACTTTCGCCTCTCGCCTCGCGAGCGCCCGCCAAA

GGCCCTGTTAACTCTGTTTTAGTGTCTTCCGAGCAATCATGTAATAGTTAAAACTTTCAA

CAACGGATCT

>ASV750 GS|0.0|None;No hit

AAGTCGTAATAAAATTTTCGTAAATGAATTTACGAAAGAATCATTATCGAAAGACGAAAT

CGCTTCGATTTCATACTTTTCACTCGTATTTACTTATCTTTGTTGCTTTGACGAATCTTC

GAACTTGATCGTTCGACGCGAGTGAGAGACTTTCGTTTCTCGTTTCGCGAGCGTTCGTCA

AAGACTTCATTAACTTTGTTTCAATGTCTTTCGAACAATTATGTAATATTTAAAATTTTC

AACAACGAAT

>ASV751 SS|0.8000|LC669673|SH0934230.09FU;k:Fungi,p:Ascomycota,c:Lecanoromycetes,o:Caliciales,f:Physciaceae

AAGTCGTAACAAGGTTTCCGTAGGTGAACCTGCGGAAGGATCATTACCGAGAGCCTGGGA

CCTAACCGGCCCCAACTCTTCACCCGTGTATACGAAAACAAATGTTGCTTTGGCGGGTCA

GGGATTCATCTCGGTCCCTGGATGGGCGTCTACCCATTCCGCGCCCGCCGAAGGCCCAAC

CAAACCCTGTTTATCCGTGATGTCCGAGTAACCATACAATAACTAAAACTTTCAACAACG

GATCTCTTGG

>ASV752 GS|87.3|AF250779|SH0954634.09FU;k:Fungi,p:Ascomycota,c:Lecanoromycetes,o:Caliciales,f:Caliciaceae,g:Buellia,s:Buellia frigida

AAGTCGTAACAAGATTTTCGTAGATGAACTTGCGAAAGAATCATTATCGAGAGACGAGAT

CGCTTCGACCTCACTCTTCACTCGTATTTACTTATCTTTGTTGCTTTGGCGAGTCTTTGA

GCTTGATCGTTCGACGCGAGTGAGAGGCTTTCGTCTCTCGCCTCGCGAGCGCTCGCCAAA

GGCTTTGTTAACTCTATTTTAGTGTCTTTCGAACAATCATGTAATAGTTAAAATTTTCAA

CAACGAATCT

>ASV753 GS|82.4|AY667583|SH0954634.09FU;k:Fungi,p:Ascomycota,c:Lecanoromycetes,o:Caliciales,f:Caliciaceae,g:Buellia,s:Buellia frigida

AAGTCGTAACAAGATTTTCGTAAATGAACTTGCGAAAGAATCATTATCGAGAGACGAGAT

CGCTTCGATCTCACTTTTCATTCGTATCTACTTATCTTTGTTGCTTTGACGAGTCTTCGA

ACGCGATCGTTCGACGCGAATGAAAGGCTTTCGTCTCTCGTCTCGCGAGCGCTCGTCAAA

GATTTTGTTAACTCTGTTTTAATGTCTTTCGAGCAATCATATAATAGTTAAAACTTTCAA

CAATGAATCT

>ASV754 GS|79.2|MN592663|SH1107827.09FU;k:Fungi,p:Ascomycota,c:Lecanoromycetes,o:Teloschistales,f:Teloschistaceae,g:Caloplaca,s:Caloplaca haematites

AAGTCGTAATAAGGTTTCCGTAGGTAAACCTGCGGAAGGATTATTATCGAGAGGGGGGGC

TCTATGCCCCGGGGCTCTGTCCCCGTACCTTTTTACCCTGTATATATTTTTCCCCCGTTG

CTTTAGCGGGCCCCGGGTCTTCCCCCGGCGTTAGCCCCCTCGCGGGGTTCGCGAGCGCCC

GCCGGAAGGCTTATCGAAACTCTGATTAGTGCAGTCTGAGCGTACGAATAATAAATCAAA

ACTTTTAACA

>ASV755 SS|0.8700|UDB02705663|SH0920237.09FU;k:Fungi,p:Ascomycota,c:Eurotiomycetes,o:Chaetothyriales

AAGTCGTAACAAGGTTTCCGTAGGTGAACCTGCGGAAGGATCATTACTGAGTTAGGGTCT

TTCTAGGCTCGATCTCCAACCCTTTGTATAACTACCATGTTGCTTTGGCGGGCCCGTCTC

TAACCGGACCGCCGGGGGTCTTTGACCTCTGGCCAGCGCTCGCCAGTAGCCCACCACAAA

TTCTTTTTAACCATGTTAAATCTGAAATTAGTTAAATAAACTAAAACTTTCAACAACGGA

TCTCTTGGTT

>ASV756 GS|0.0|None;No hit

GTGTCAGCAGCCGCGGTAATACGGAGGATCCAAGCGTTATCCGGATTTATTGGGTTTAAA

GGGTGCGTAGGCGGCCTGTTAAGTCAGGGGTGAAAGACGGTGGCTCAACCATCGCAGTGC

CTTTGATACTGACGGGCTTGAATGCAGTTGAGGTAGGCGGAATGTGGCAAGTAGCGGTGA

AATGCATAGATATGCCACAGAACACCAATTGCGAAGGCAGCTTACCAAAGTGCGATTGAC

GCTGAGGCAC

>ASV757 GS|0.0|None;No hit

AAGTCGTAACAAGGTCTCCGTAGGTGAACCTGCGGAGGGATCATTAATGAAGTTCGGGTG

GGTTCCTGTGTGCGACCGCGCGGGCCAGTGTCTAGCTGGCCCGTGCGGCACCACATCGGG

CCCCCCAGCCCTCTCATCCACAATACCCCTGTGCACTGTTGGTCGTCCTTCGTCTTGCGC

CTCGGCGCTCGACAAGCACCCCTTTCTATCAATATACAAACCATGTCTGAAGAACGTCAC

GTCTGTCTTT

>ASV758 GS|88.2|AF250779|SH0954634.09FU;k:Fungi,p:Ascomycota,c:Lecanoromycetes,o:Caliciales,f:Caliciaceae,g:Buellia,s:Buellia frigida

AAGTCGTAATAAGATTTTCGTAGATGAATTTGTGAAAGAATCATTATCGAGAGACGAGGT

CGCTTCGATCTCACTCTTCACTCGTGTCTACTTATCTTTGTTACTTTGACGAATTTTCGA

GCTTGATCGTTCGACGCGAGTGAGAGGCTTTCGTCTCTCGTCTCGCGAGCGCTCGTCAAA

GACTTTGTTAACTCTGTTTTAGTGTCTTCCGAGCAACCATGTAATAGTTAAAACTTTCAA

CAACGGATCT

>ASV759 SS|1.0000|AF250779|SH0954634.09FU;k:Fungi,p:Ascomycota,c:Lecanoromycetes,o:Caliciales,f:Caliciaceae,g:Buellia,s:Buellia frigida

AAGTCGTAACAAGATTTCCGTAGGTGAACCTGCGAAAGAATCATTACCGAGAGACGAGGT

CGCTTCGACCCCACTCTTCACCCGTGTCTACTTACCTTTGTTGCTTTGGCGAGCCTTCGG

GCTTGACCGTTCGACGCGAATGAAAGACTTTCGCCTCTCGCCTCGCGAGCGCTCGCCAAA

GACCCTGTTAACTCTGTTTTAGTGTCTTCCGAGCAACCATGTAATAGTTAAAACTTTCAA

CAACGGATCT

>ASV760 GS|90.2|AF250779|SH0954634.09FU;k:Fungi,p:Ascomycota,c:Lecanoromycetes,o:Caliciales,f:Caliciaceae,g:Buellia,s:Buellia frigida

AAGTCGTAACAAGATTTTCGTAGGTGAATCTGCGAAAGAATCATTATCGAGAGACGAGAT

CGCTTCGACCCCACTCTTCACCCGTGTCTACTTATCTTTGTTGCTTTGACGAGTCTTCGG

GCTTGATCGTTCGACGCGAGTGAGAGGCTTTCGCCTGTCGCCTCGCGAGCGTTCGCCAAA

GGCTTTGTTAACTTTGTTTTAGTGTCTTTCGAGCAATCATGTAATAGTTAAAACTTTCAA

CAACGAATCT

>ASV761 GS|80.0|MN592663|SH1107827.09FU;k:Fungi,p:Ascomycota,c:Lecanoromycetes,o:Teloschistales,f:Teloschistaceae,g:Caloplaca,s:Caloplaca haematites

AAGTCGTAACAAGGTTTCCGTAGGTGAACCTGCGGAAGGATTATTATCGAGAGGGGGGGC

TCTATACCCCGGGGCTCTGTCCCCGTACCTTTTCACCCTATATATATTTTTCCCCCGTTG

CTTTAGCGGGCCCCGGGTCTTCCCCCGGCGTTGGCCCCCTCGCGGGGTTCGCGAGCGCCC

GCCGGAAGGCTTATCGAAACTCTGATTAGTGCAGTCTAAGCGTACGAATAATAAATTAAA

ACTTTTAATA

>ASV762 GS|0.0|None;No hit

GTGTCAGCAGCCGCGGTAATACGAGGGGGGCAAGCGTTGTTCGGAATTATTGGGCGTAAA

GGGTGCGTAGGCGGCCCCGCAAGTCTTGTGTGAAAGCCTCAAGCTCAACTTGAGGACTGC

ACAGGAAACTGCTGGGCTGGAGTATGGGAGAGGTGAGTGGAATTCCTGGTGTAGCGGTGA

AATGCGTAGATATCAGGAGGAACACCTGTGGCGAAAGCGGCTCACTGGACCATAACTGAC

GCTGAGGCAC

>ASV763 GS|0.0|None;No hit

AAGTCGTAACAAGGTTATCCTTCTCGGTTTACCGGCCGAAGCCTTTTTCCACACTTCGAC

TGAAAATATTAGTGTGCAGTCTGCTTCACGGCAGGCAACGTCACGTCTGTGCTGGGACTA

CTAAACATGTAGTACCAGCAGCCGAGTCAAGACAGACTCGGTTCACAGATCATGCAGTGG

CGGCCAGGTCCCGAGACCTTCTCAGGACATGGTTAAGATATGACCGGTCCAGCCTATCAC

TGGTTGGAGG

>ASV764 GS|100.0|UDB02377110|SH1084406.09FU;k:Fungi,p:Ascomycota,c:Dothideomycetes,o:Mycosphaerellales,f:Teratosphaeriaceae,g:Oleoguttula

AAGTCGTAACAAGGTCTCCGTAGGTGAACCTGCGGAGGGATCATTACCGAGTGAGGGTCC

TCTGGGCCCGACCTCCAACCCTTTGATATCTGACTCTGTTGCCTCGGGGGCGACCCGGCC

CTCACGGGCGTCGGGGCCCCCGGTGGACCACACCAACCCTGTATCCATACGTCCGAGTAA

CAACCGAATAAGTTAAAACTTTCAACAACGGATCTCTTGGTTCTG

>ASV765 GS|0.0|None;No hit

AAGTCGTAACAAGGTCTTCGTAGGTACATCATTCATATGATCCTAGGCCTTGAACAACAT

CACATCGATGTTAGTCTGGTATGAGCCATGCAACAGCGGACTCAACAGGCAACACTCTTA

AATTGCGAGGACCCCCTGACTGTGTTGAATACCGCTCCTTCCCGAGCAGTCAAGAAGAGA

GATCTTGATGATGGTAACAACTTCAACAAAGGACAATGTTTATTTGGTTTAGCAGTTTTC

TAATACAGAA

>ASV766 SS|0.9400|MT809481|SH1325656.09FU;k:Fungi,p:Ascomycota

AAGTCGTAACAAGGTTTCCGTAGGTGAACCTGCGGAAGGATCATTACCGAGCTAGGGTCT

CTGGCCCCACCTCCAACCCTCTGTGTACCTACCTTTGTTGCTTTGGCGGGCCGTCGGGCC

ACCATGGCCTGCCGAGGACCTCTGAGGTCCGCGAGCGCCCGTCAGAAGCCTTCCTAAAAA

CTCCCATGTACAGTTGCAGTCTGAGTAGGCATTTAATCTAGTTAAAACTTTCAACAACGG

ATCTCTTGGT

>ASV767 GS|84.3|AF250779|SH0954634.09FU;k:Fungi,p:Ascomycota,c:Lecanoromycetes,o:Caliciales,f:Caliciaceae,g:Buellia,s:Buellia frigida

AAGTCGTAACAAGATTTTCGTAGATGAATCTACAAAAGAATCATTATCGAAAGACGAAAT

CGCTTCGATCTCACTCTTCACTCGTGTCTACTTATCTTTGTTGCTTTGACGAGTCTTTGA

GCTTAATCGTTCGACGCGAGTGAGAGACTTTCGTCTCTCGTCTCGCGAGCGCTCGTCAAA

GACTCTATTAACTTTGTTTTAGTGTCTTTCGAGCAATCATGTAATAGTTAAAATTTTCAA

CAACGAATCT

>ASV768 SS|1.0000|AF250779|SH0954634.09FU;k:Fungi,p:Ascomycota,c:Lecanoromycetes,o:Caliciales,f:Caliciaceae,g:Buellia,s:Buellia frigida

CTTTTTCCTTTCTCTTCCTTCCCCTTCTTCCCCCTTTTTCCGTAGTTTCCCCTGCGGACT

TCTCCTTACCGAGCTCCTGGGTCGCTTCGGCCCCACTCTTCCCCCGTGTCTACTTACCTT

TGTTGCTTTGGCGGGCCTTCGGGCTTGACCGTTCGCCGCGGGTGGGAGGCTTTCGCCTCT

CGCCTCGCGCGCGCCCGCCAAAGGCCCTGTTAACTCTGTTTTAGTGTCTTCCGAGCAACC

ATGTAATAGT

>ASV769 SS|0.8900|MZ224659|SH0916480.09FU;k:Fungi,p:Ascomycota,c:Lecanoromycetes,o:Caliciales

AAGTCGTAACAAGGTTTCCGTAGGTGAACCTGCGGAAGGATCATTATCGAGAGACGGAGT

CTAACCGGCCCCACTCTTCACCCGTGTATACCTACCCTTTATTGCTTTGGCGGGCGGTCG

GTCTTTACCGCCCGACGTCGGTCGGAAGGCTCGCGCCTTCCACTCACCGAACGCCCGCCA

GAGGCCTTCAAAAGCCGTCCATCTGTGTCCGAGCGAGTGAAGTAAATAGTTAAAAACTTT

CAACAACGGA

>ASV770 GS|99.0|AF250779|SH0954634.09FU;k:Fungi,p:Ascomycota,c:Lecanoromycetes,o:Caliciales,f:Caliciaceae,g:Buellia,s:Buellia frigida

AAGTCGTAATAAGATTTTCGTAGATGAATTTGTGAAAGAATCATTATCGAGAGACGGGGT

CGCTTCGGCCCCACTCTTCACCCGTGTCTACTTACCTTTGTTGCTTTGGCGGGCCTTCGG

GCTTGACCGTTCGACGCGGGTGGGAGGCTTTTGCCTCTCGCCTCGCGAGCGCCCGCCAAA

GGCCCTGTTAACTCTGTTTTAGTGTCTTCCGAGCAACCATGTAATAGTTAAAACTTTCAA

CAACGGATCT

>ASV771 SS|1.0000|AF250779|SH0954634.09FU;k:Fungi,p:Ascomycota,c:Lecanoromycetes,o:Caliciales,f:Caliciaceae,g:Buellia,s:Buellia frigida

CTTTTTCCTTTCTCTTCCTTCCCCTTCTTCCCCCTTTTTCCGTATTTTCCCCTGCGGACT

TCTCCTTACCGATCTCCTTGGTCGCTTCTTCCCCACTCTTCCCCTTCACCCGTGTCTACT

TACCTTTGTTGCTTTGGCGGGCCTTCGGGCTTGCCCGTTCGACGCGGGTGGGAGGCTTTC

GCCTCTCGCCTCGCGAGCGCCCGCCAAAGGCCCTGTTAACTCTGTTTTAGTGTCTTCCGA

GCAACCATGT

>ASV772 SS|0.9500|MZ224659|SH0916480.09FU;k:Fungi,p:Ascomycota,c:Lecanoromycetes

AAGTCGTAACAAGGTTTCCGTAGATGAATTTGCGGAAGGATCATTATCGAGAGACGGAGT

CTAACCGGCCCCACTCTTCACCCGTGTATATTTACCCTTTGTTGCTTTGGCGAGCGGTCG

GTCTTTACCGCCCGACGTCGGTCGGAAGGCTCGCGTCTTCCACTCATCGAACGCCCGCCA

GAGGCCTCCAAAAGCCGTTCATTTGTGTCCGAGCGAGTGAAGCAAATAATTAAAAACTTT

CAACAACGGA

>ASV773 GS|77.9|MZ229888|SH0913119.09FU;k:Fungi,p:Ascomycota,c:Lecanoromycetes,o:Caliciales,f:Caliciaceae,g:Dimelaena

AAGTCGTAACAAGGTTTCCGTAAATGAACCTGCGAAAGGATCATTATCGAGAGACGAAGT

CTAACCGACCCCACTCTTCACCCGTGTATACCTACCCTTTATTGCTTTGACGAACGATCG

ATCTTTACCGCCCGACGTCGGTCGGAAGGCTCGCGCCTTCCACTCATCGAACGCCCGCCA

GAGGCCCCCAAAAGCCGTCCATTTGTGTCCGAGCGAGTGAAACAAATAATTAAAAACTTT

CAACAACGGA

>ASV774 GS|78.4|AY667583|SH0954634.09FU;k:Fungi,p:Ascomycota,c:Lecanoromycetes,o:Caliciales,f:Caliciaceae,g:Buellia,s:Buellia frigida

AAGTCGTAACAAAATTTTCGTAAATGAATTTGCGAAAGAATCATTATCGAGAGACGAAAT

CGTTTCGATCTCATTCTTTATTCGTATCTACTTATCTTTGTTGCTTTGACGAATCTTCGA

ACGCGATCATTCGATGCGAGTGAGAGACTTTCGTTTCTCGTTTCGCAAGCGCTCGTCAAA

GATTTTGTTAACTCTATTTTAGTGTCTTTCGAGCAATTATGTAATAGTTAAAATTTTCAA

CAACGAATCT

>ASV775 GS|80.9|AF250779|SH0954634.09FU;k:Fungi,p:Ascomycota,c:Lecanoromycetes,o:Caliciales,f:Caliciaceae,g:Buellia,s:Buellia frigida

AAGTCGTAACAAGATTTTCGTAAATGAATTTACGAAAGAATCATTATCGAGAGACGAAAT

CGCTTCGATCTCATTCTTCACTCGTATTTACTTATCTTTGTTGCTTTGACGAGTCTTCGA

ACTTAATCGTTCGACGCGAATGAAAAACTTTCGTCTCTCGTTTTGCGAGCGCTCGTCAAA

GATCTTGTTAACTCTATTTTAGTGTCTTTCAAGTAATCATGTAATAGTTAAAACTTTCAA

CAACGGATCT

>ASV776 GS|0.0|None;No hit

GTGTCAGCAGCCGCGGTAATACGAAGGGGGCTAGCGTTGCTCGGAATGACTGGGCGTAAA

GGGCGCGTAGGCGGATCACACAGTCAGGCGTGAAATTCCTGGGCTTAACCTGGGGGCTGC

GTTTGAGACGTGGGGTCTGGAGTGGGGAAGAGGGTCGTGGAATTCCCAGTGTAGAGGTGA

AATTCGTAGATATTGGGAAGAACACCGGTGGCGAAGGCGGCGACCTGGTCCTTGACTGAC

GCTGAGGCGC

>ASV777 GS|0.0|None;No hit

GTGTCAGCAGCCGCGGTAATACGGAGGGTGCGAGCGTTGTCCGGATTTATTGGGTTTAAA

GGGTGCGTAGGCGGCCGATTAAGTCTGGGGTGAAAGCCCGCTGCTCAACAGCGGAACTGC

CCTGGATACTGGCTGGCTTGAGTACAGACGAGGTTGGCGGAATGGACCGAGTAGCGGTGA

AATGCATAGATACGGTCCAGAACCCCGATTGCGAAGGCAGCTGACTAGGCTGTTACTGAC

GCTGAGGCAC

>ASV778 GS|100.0|MN006700|SH1223465.09FU;k:Fungi,p:Basidiomycota,c:Cystobasidiomycetes,o:Cystobasidiales,f:Cystobasidiaceae,g:Cystobasidium,s:Cystobasidium laryngis

AAGTCGTAACAAGGTTTCCGTAGGTGAACCTGCGGAAGGATCATTAATGAATTTTAGGAC

TCTCTCTTTAGAGGTCCGACCCCTTCATTTCCTTACACTGTGCACACACTTCTTTTCACA

CATTTTAACACTATAGTATAAGAATGTAACAGTCTCTTTATTGAGCATAAATAAAAATAA

AACTTTCAGCAACGGATCTCTTGGCTCTC

>ASV779 GS|0.0|None;No hit

AAGTCGTAACAAGATTTCCGTAGATGAATCTGCGAAAGAATTATTATCGAGAGATGAAAT

CTAATCGGCCTCACTTTTCATTCGTGTATATTTACTCTTTATTGCTTTGACGGGCGGTCG

GTCTTTACCGCCCGACGTCGGTCGAAAGACTCGCGTCTTTCACTCATCGAACGCCCGTTA

AAGATTTTCAAAACCCGTTCATTTATATTCGAGCGAGTGAAACAAATAATTAAAAACTTT

TAACAACGAA

>ASV780 GSL|100.0|OK576228|SH1240491.09FU;k:Fungi,p:Ascomycota,c:Dothideomycetes,o:Dothideales,f:Saccotheciaceae,g:Aureobasidium

AAGTCGTAACAAGGTTTCCGTAGGTGAACCTGCGGAAGGATCATTAAAGAGTAAGGGTGC

TCAGCGCCCGACCTCCAACCCTTTGTTGTTAAAACTACCTTGTTGCTTTGGCGGGACCGC

TCGGTCTCGAGCCGCTGGGGATTCGTCCCAGGCGAGCGCCCGCCAGAGTTAAACCAAACT

CTTGTTATTTAACCGGTCGTCTGAGTTAAAATTTTGAATAAATCAAAACTTTCAACAACG

GATCTCTTGG

>ASV781 GS|97.5|AF250779|SH0954634.09FU;k:Fungi,p:Ascomycota,c:Lecanoromycetes,o:Caliciales,f:Caliciaceae,g:Buellia,s:Buellia frigida

AAGTCGTAACAAGGTTTTCGTAGGTGAACCTGCGAAAGGATCATTATTGAGAGACGGGGT

CGCTTCGGCCCCACTCTTCACTCGTGTCTACTTACCTTTGTTGCTTTGGCGGGCCTTCGG

GCTTGATCGTTCGACGCGGGTGGGAGGCTTTCGCCTCTCGCCTCGCGAGCGCCCGCCAAA

GGCCTTGTTAACTCTGTTTTAGTGTCTTCCGAGCAACCATGTAATAGTTAAAACTTTCAA

CAACGGATCT

>ASV782 GS|82.4|AF250779|SH0954634.09FU;k:Fungi,p:Ascomycota,c:Lecanoromycetes,o:Caliciales,f:Caliciaceae,g:Buellia,s:Buellia frigida

AAGTCGTAACAAAATTTTCGTAGATGAATTTGCGAAAAAATCATTATCGAGAGACGAAAT

CGCTTCGATCTCACTTTTCACTCGTATCTACTTATCTTTGTTGCTTTGACGAATCTTCGA

GCTTGATCGTTCGACGCGAATGAGAGACTTTCGTCTCTCGTTTCGCGAGCGCTCGTCAAA

AATCTTGTTAACTTTGTTTTAGTGTCTTTCGAGCAATCATGTAATAATTAAAATTTTCAA

CAACGAATCT

>ASV783 GS|76.1|UDB01571335|SH0954626.09FU;k:Fungi,p:Ascomycota,c:Lecanoromycetes,o:Caliciales,f:Caliciaceae,g:Buellia

AAGTCGTAATAAGATTTTCGTAAATGAATTTACGAAAAAATCATTATCGAGAGACGAAAT

CGTTTCGATCTCACTCTTCACTCGTATCTACTTATCTTTGTTGCTTTGACGAATCTTCGA

GCTTGATCGTTCGACGCGAATGAAAGACTTTTGTCTCTCGTTTTGCGAGCGCTTGTCAAA

GACTTTGTTAACTCTATTTTAATGTCTTTCGAACAATCATGTAATAATTAAAACTTTTAA

CAACGAATCT

>ASV784 GS|0.0|None;No hit

AAGTCGTAACAAGGTTTTCGTAGGTGAACCTGCGGAAGGATCATTACCAGATGGGGCCTC

CGGGCCCTTTTCTCCTTACCACGTGCACCGAGTTGTCGGGAGATGTTTGTTTCCCGACCA

ATTCTTCACGCTTGTTTTGTTGTCTGAGTCGAATGACCATAAATTGAAAAGAAAACTATG

AACAACGGATCTCTAGGCTCTT

>ASV785 GSL|78.2|MZ224659|SH0916480.09FU;k:Fungi,p:Ascomycota,c:Lecanoromycetes,o:Caliciales,f:Caliciaceae

AAGTCGTAACAAGATTTCCGTAGATGAACCTGCGGAAGAATCATTATCGAAAGACGGAGT

CTAACCGGCCCCACTCTTCACCCGTGTATATCTATCCTTTGTTGCTTTAACGGGCGATCG

GTCTTTACCGCCCGACGTCGGTCGAAAGACTCGCGCCTTCCACTCATCGAACGCCCGCCA

GAGACCTCCAAAAGCCGTTCATCTATGTTCGAGCGAGTGAAGCAAATAATTAAAAACTTT

CAACAACGGA

>ASV786 GS|83.8|AF250779|SH0954634.09FU;k:Fungi,p:Ascomycota,c:Lecanoromycetes,o:Caliciales,f:Caliciaceae,g:Buellia,s:Buellia frigida

AAGTCGTAACAAGATTTTCGTAGATGAATTTACGAAAAAATCATTATCGAGAGACGAAAT

CGCTTCGATTTCACTCTTCACTCGTGTCTACTTATCTTTGTTGCTTTGACGAGTCTTCGA

ACTTGATCGTTCGATGCGAGTGAGAGACTTTCGTCTCTCGTCTCGCGAGCGCTCGTCAAA

GACTTTGTTAACTCTATTTTAGTGTTTTTCGAGCAATCATGTAATAGTTAAAATTTTCAA

CAACGAATCT

>ASV787 GS|81.4|MK970687|SH0954634.09FU;k:Fungi,p:Ascomycota,c:Lecanoromycetes,o:Caliciales,f:Caliciaceae,g:Buellia,s:Buellia frigida

AAGTCGTAACAAAATTTTCATAAATAAATCTACGAAAGAATCATTATCGAGAGACGAAAT

CGCTTCGATCTCACTCTTCACTCGTATCTACTTATCTTTGTTGCTTTGACGAATCTTCGA

ACTTGATCGTTCGACGCGAATGAGAGACTTTCGTCTCTCGTTTTGCGAGCGTTCGTCAAA

GATCTTGTTAACTTTGTTTTAGTGTCTTTCGAACAATCATGTAATAATTAAAATTTTCAA

CAACGAATCT

>ASV788 GS|82.8|AY667583|SH0954634.09FU;k:Fungi,p:Ascomycota,c:Lecanoromycetes,o:Caliciales,f:Caliciaceae,g:Buellia,s:Buellia frigida

AAGTCGTAACAAGATTTTCGTAAATGAACTTGCGAAAGAATCATTATCGAAAGACGAGAT

CGCTTCGATTTCACTCTTCACTCGTATCTACTTATTTTTGTTGCTTTGACGAATCTTCGA

GCTTGATCGTTCGACGCGAGTGAGAGACTTTCGTTTCTCGTCTTGCGAGCGTTCGTCAAA

GACTCTATTAACTCTATTTTAGTGTCTTTCGAGCAATCATGTAATAATTAAAACTTTCAA

CAACGAATCT

>ASV789 SS|0.9900|MZ243488|SH0916476.09FU;k:Fungi,p:Ascomycota,c:Lecanoromycetes

AAGTCGTAACAAGGTTTCCGTAGGTGAACCTGCGGAAGGATCATTACTAAGAGAGGGATG

TACGCTTCCAGCCGAGTCCCGGGGGGCTGCGCCCCTCACCTCTTCAACCCTGTGTCTACC

AACCGCTGTTGCTTTGGCGGGCCGTCGAGCCTTACCGCTCGGCGCCAGTCGGAAGGCTAG

CGTCCTCCGCCCGGCGAGCGTCCGCCAGAGGCCTTCAATACTCCATCTCAGTGATGTCCG

AGCAAACTAA

>ASV790 SS|0.8400|KY266860|SH0954363.09FU;k:Fungi,p:Ascomycota,c:Lecanoromycetes,o:Acarosporales,f:Acarosporaceae,g:Pleopsidium,s:Pleopsidium chlorophanum

AAGTCGTAACAAGGTTTCCGTAGGTGAACCTGCGGAAGGATCATTACAGAGTTAGGGTCT

TCCGGGCCCGATCTCCAACCCTATGTCTACCTACCTTTGTTCCTTGGCGGGCCCGCTGGG

GGAGACCCACCGGTGGCTCCGGCTGCCGAGCGCCCGTCAGAGACCCATCGAACCCTGTTA

ATCATGTAGTCTGAGTACCAATACAATAGTTAAAACTTTCAACAACGGATCTCTTGGTTC

TG

>ASV791 SS|0.9300|KP822310|SH1067818.09FU;k:Fungi,p:Ascomycota,c:Lecanoromycetes

AAGTCGTAACAAGGTTTCCGTAGGTGAACCTGCGGAAGGATCATTACCGAGAGACGGGCT

TCGCGCTCCGGGGGGTTTCGGCCCCCGCCTCTTCACCCTATGTCTACTTACTTTTGTTGC

TTTGGCGGACCTGGGGCCTTGCCCCCACGTCAATCCCAGGCTTCACGGCCGGGGTCGGTG

CGCGCCCGCCAGAGGCTCATTATATTCTGCTTACCAGTGACGTCCGAGTAACAACACAAT

AGTTAAAACT

>ASV792 GS|0.0|None;No hit

AAGTCGTAACAAGGTTGTCCTTCTCGGTGAATCGGCCGAAGCCTTTTTCCACACGTCGAC

TGAAAATACAAGTGTGCAGTCTGCTCTACGGCAGGCAACGTCACGTCTATGCTGGGACTA

CCAAACATGTAGTACCAGCAGCCGAGTCAAAACAGACTCGGTTCACAGATCATGCAGTGG

CGGCCAGGTCCCGAGACCTTCTCGGGACATGGTTAAGATATGACCGGTCCAGCCTATTAC

TGGTTGGAGG

>ASV793 GS|100.0|MK268132|SH1223355.09FU;k:Fungi,p:Basidiomycota,c:Tremellomycetes,o:Trichosporonales,f:Trichosporonaceae,g:Apiotrichum,s:Apiotrichum gracile

AAGTCGTAACAAGGTTTCCGTAGGTGAACCTGCGGAAGGATCATTAATGAAATTCAAGGA

CGCTCTTTTTAGAGGTCCGACCAATTACATTTTACCTTTACTCTGTGCACACACTACTCT

TTACACCATTTTTTAACTCTATAGTTTTAAGAATGTAAAACAAGTCTCGAAAGAGCAACA

TAACAAAACAAAACTTTCGGCAACGGATCTCTTGGCTCTC

>ASV794 GS|88.7|AF250779|SH0954634.09FU;k:Fungi,p:Ascomycota,c:Lecanoromycetes,o:Caliciales,f:Caliciaceae,g:Buellia,s:Buellia frigida

AAGTCGTAACAAGATTTTCGTAGATGAATTTGCGAAAGAATCATTATCGAGAGACGAGGT

CGCTTCGATCTCACTCTTCACTCGTGTCTACTTATCTTTGTTGCTTTGACGAGTCTTCGA

GCTTGATCGTTCGACGCGAGTGAGAGGCTTTCGTCTCTCGTCTCGCGAGCGCTCGCCAAA

GACTCTATTAACTTTGTTTTAGTGTCTTTCGAGCAATCATGTAATAGTTAAAACTTTCAA

CAACGGATCT

>ASV795 SS|1.0000|AY667583|SH0954634.09FU;k:Fungi,p:Ascomycota,c:Lecanoromycetes,o:Caliciales,f:Caliciaceae,g:Buellia,s:Buellia frigida

AAGTCGTAACAAGGTTTCCGTAGGTGAACCTGCGGAAGGATCATTACCGAGAGACGGGGT

CGCTTCGGTCTCACTCTTCACTCGTGTCTACTTATCTTTGTTGCTTTGGCGGGTCTTCGG

GCTTGACCGTTCGACGCGAGTGGGAGGCTTTCGCCTCTCGCCTCGCGAGCGCTCGCCAAA

GGCCCTGTTAACTCTGTTTTAGTGTCTTCCGAGCAATCATGTAATAGTTAAAACTTTCAA

CAACGGATCT

>ASV796 SS|0.9900|AF250779|SH0954634.09FU;k:Fungi,p:Ascomycota,c:Lecanoromycetes,o:Caliciales,f:Caliciaceae,g:Buellia,s:Buellia frigida

AAGTCGTAACAAGATTTTCGTAGGTGAACTTGCGAAAGAATCATTATCGAGAGACGAGGT

CGCTTCGACCCCACTCTTCACTCGTGTCTACTTACCTTTGTTGCTTTGGCGAGCCTTCGA

ACTTGATCGTTCGACGCGGGTGGGAGGCTTTCGTCTCTCGTCTCGCGAGCGCTCGCCAAA

GGCCCTATTAACTCTGTTTTAGTGTCTTCCAAGCAACCATGTAATAGTTAAAACTTTCAA

CAACGAATCT

>ASV797 GS|98.8|AY667583|SH0954634.09FU;k:Fungi,p:Ascomycota,c:Lecanoromycetes,o:Caliciales,f:Caliciaceae,g:Buellia,s:Buellia frigida

AAGTCGTAACAAGGTTTCCGTAGGTGAACCTGCGGAAGGATCATTACCGAGAGACGGGGT

CGCTTCGGCCCCACTCTTCACCCGTGTCTACTTACCTTTGTTGCTTTGGCGGGCCTTCGA

GCTTGACCGTTCGACGCGGGTGGGAGGCTTTCGCCTGTCGCCTCGCGAGCGCCCGCCAAA

GACCCTGTTAACTCTGTTTTAGTGTCTTCCGAGCAACCATGTAATAGTTAAAACTTTCAA

CAACGGATCT

>ASV798 SS|0.8000|MZ229889|SH0913114.09FU;k:Fungi,p:Ascomycota,c:Lecanoromycetes,o:Caliciales,f:Caliciaceae

AAGTCGTAACAAGGTTTCCGTAGGTGAACCTGCGGAAGGATCATTATCGAGAGACGGAGT

CTAACCGGCCCCACTCTTCACCCGTGTATATCTACTCTTTGTTGCTTTGGCGGGCGGTCG

GTCTTTACCGCCCGACGTCGGTCGGAAGGCTCGCGCCTTCCACTCATCGAACGCCCGCCA

GAGGCCCCCAAAAGCCGTTCATCTGTGTTCGAGCGAGTGAAGCAAATAGTTAAAAACTTT

CAACAACGGA

>ASV799 SS|0.9000|MZ224659|SH0916480.09FU;k:Fungi,p:Ascomycota

AAGTCGTAACAAGGTTTCCGTAGATGAACCTGCGGAAGGATCATTATCGAGAGACGGAGT

TTAACCGGCCCCACTCTTCACCCGTGTATATTTACCCTTTGTTGCTTTGGCGGGCGGTCG

GTCTTTACCGCCCGACGTCGGTCGGAAGGCTCGCGTTTTTCACTCACCGAACGCCCGCCA

GAGGTCTTCAAAAGCCGTTCATCTGTGTCCGAGCGAGTGAAACAAATAGTTAAAAACTTT

CAACAACGGA

>ASV800 GS|77.7|MZ229889|SH0913114.09FU;k:Fungi,p:Ascomycota,c:Lecanoromycetes,o:Caliciales,f:Caliciaceae,g:Dimelaena

AAGTCGTAATAAGGTTTCCGTAAATGAATCTGCGGAAGGATCATTATCGAGAGACGAAGT

CTAACCGGCCCCACTCTTCACCCGTGTATATCTACCCTTTATTGCTTTGGCGGGCGGTCG

GTCTTTACCGCCCGACGTCGGTCGAAAAACTCGCGCCTTTCACTCATCGAACGCCCGTCA

GAGACCCCCAAAAGCCGTCCATTTGTGTCCGAGCGAGTGAAGCAAATAATTAAAAACTTT

CAACAACGGA

>ASV801 GS|84.3|AF250779|SH0954634.09FU;k:Fungi,p:Ascomycota,c:Lecanoromycetes,o:Caliciales,f:Caliciaceae,g:Buellia,s:Buellia frigida

AAGTCGTAACAAGATTTTCGTAGATGAATTTACGAAAGAATCATTATCGAGAGACGAGAT

CGCTTCGATCTCACTCTTTATTCGTATCTACTTATCTTTGTTGCTTTGACGAGTCTTCGA

GCTTGATCGTTCGACGCGAATGAGAGACTTTCGTCTCTCGTTTCGCGAGCGCTCGTCAAA

GATCTTGTTAACTCTATTTTAGTGTCTTTCAAGCAATCATGTAATAGTTAAAACTTTCAA

CAACGGATCT

>ASV802 GS|83.8|AF250779|SH0954634.09FU;k:Fungi,p:Ascomycota,c:Lecanoromycetes,o:Caliciales,f:Caliciaceae,g:Buellia,s:Buellia frigida

AAGTCGTAACAAGATTTTCGTAAATGAATTTGCGAAAGAATTATTATCGAGAGACGAAGT

CGCTTCGATCTCACTCTTCATTCGTGTCTACTTATTTTTGTTGCTTTGACGAGTCTTCGA

GCGCGATCGTTCGACGCGAGTGAAAGACTTTCGTCTCTCGTCTCGCGAGCGTTCGTCAAA

GACTTTGTTAACTCTATTTTAGTGTCTTTCGAGCAATCATGTAATAGTTAAAACTTTCAA

CAACGAATCT

>ASV803 GS|84.3|AF250779|SH0954634.09FU;k:Fungi,p:Ascomycota,c:Lecanoromycetes,o:Caliciales,f:Caliciaceae,g:Buellia,s:Buellia frigida

AAGTCGTAACAAGATTTTCATAGATGAATTTGCGAAAGAATCATTATCGAGAGACGAGAT

CGCTTCGATCTCACTCTTCATTCGTATCTACTTATCTTTGTTGCTTTGACGAGTCTTCGA

GCTTGATCATTCGACGCGAGTGAGAGACTTTCGTCTCTCGTCTTGCGAGCGCTCGTCAAA

GACTTTGTTAACTTTGTTTTAGTGTCTTTCAAGCAATCATGTAATAGTTAAAACTTTCAA

CAACGAATCT

>ASV804 GS|0.0|None;No hit

GTGTCAGCAGCCGCGGTGATACGTAGGGTCCGAGCGTTGTCCGGAATTATTGGGCGTAAA

GGGCTTGTAGGCGGTCCGTCGCGTCAGGAGTGAAAACTCGGGGCTTAACCCCGAGCCTGC

TTTTGATACGGGCGGACTAGAGGGATGCAGGGGAGAACGGAATTCCTGGTGGAGCGGTGG

AATGCGCAGATATCAGGAGGAACACCGGTGGCGAAGGCGGTTCTCTGGGCATTTCCTGAC

GCTGAGAAGC

>ASV805 GS|98.5|UDB03337334|SH0989105.09FU;k:Fungi,p:Ascomycota,c:Leotiomycetes,o:Helotiales

AAGTCGTAACAAGGTTTCCGTAGGTGAACCTGCGGAAGGATCATTACCGAGTTTGTGCCC

TCTGGCGTAGATCTCCCACCCTGTTTATTTTACTTCATGTTGCTTTGGCGGGTCGACTGT

CCAGGCCACCGGCGACTCGCTGGTGTGTGCCCGCCAAAGGCTCCTAAACCCGGATATGTA

ACCTGCCGTCCGAGTCCTATATAATACTTAAAACTTTCAACAACGGATCTCTTGGTTCTG

>ASV806 GS|99.5|UDB06665463|SH0903291.09FU;k:Fungi,p:Ascomycota,c:Lecanoromycetes,o:Caliciales,f:Physciaceae,g:Rinodina,s:Rinodina olivaceobrunnea

AAGTCGTAACAAGGTTTCCGTAGGTTTGAGATGGTTTGCCTACCGAAGCCCCCCGCAGCG

ACTGTAAAAAACTGCGCGGTCTACGTCCCCGAGATGTAGGCAACACCGTCCAAGTGCTGA

AAGGGCCCGGCGAAAGCCCGCGCCTAACCAGCAGCCAGTGCGTGGGAACGCTGGTGCACA

GATCAATCGACGGTGACCTTTTACGGTTAAAATATGACCGGTCCTAGGCCTCGTGGCTTT

TGGAAGTGTC

>ASV807 GS|97.6|UDB0479157|SH0953972.09FU;k:Fungi,p:Ascomycota,c:Dothideomycetes,o:Botryosphaeriales,f:Botryosphaeriaceae,g:Dothiorella

AAGTCGTAACAAGGTTTCCGTAGGTGAACCTGCGGAAGGATCATTACCGGGCTCAGGGGG

GGAAACCCCCCGAACTCCCAACCCTTGCTTACCTACGACGTTGCTTCGGCGGGTTCGCGC

CCGCCGGAGGTCAATCAAACTATTTTATACCATAGTCAGAGCGAATGCGAAAATAAGTAA

AAACTTTCAACAACGGATCTCTTGGTTCTG

>ASV808 GS|78.2|MN103133|SH0913114.09FU;k:Fungi,p:Ascomycota,c:Lecanoromycetes,o:Caliciales,f:Caliciaceae,g:Dimelaena,s:Dimelaena oreina

AAGTCGTAACAAGATTTCCGTAGGTGAACCTGCGAAAGGATCATTATCGAAAGATGAAGT

CTAACCGGCCCCACTCTTCACCCGTGTATATCTACCCTTTGTTGCTTTGACGGGCGGTCG

GTCTTTATCGCCCGACGTCGGTCGAAAGACTCGCGCCTTTCATTCATCGAACGCCCGTCA

GAGGCTTCCAAAACCCGTCCATCTATGTTCGAGCGAGTGAAGCAAATAATTAAAAACTTT

CAACAACGGA

>ASV809 GS|0.0|None;No hit

AAGTCGTAACAAAATTTTCGTAAATGAATCTACGAAAGAATCATTATTGAGAGACGAAAT

CACTTCGATCTCATTCTTCACTCGTATCTACTTATCTTTGTTGTTTTGACGAGTTTTCGA

ACTTGATCGTTCGACGCGAGTGAGAGACTTTCGTTTCTCGTCTTGCGAGCGCTCGTCAAA

GATTTTATTAACTCTATTTTAGTGTCTTTCGAGCAATCATATAATAATTAAAACTTTCAA

TAACGAATCT

>ASV810 SS|1.0000|AF250779|SH0954634.09FU;k:Fungi,p:Ascomycota,c:Lecanoromycetes,o:Caliciales,f:Caliciaceae,g:Buellia,s:Buellia frigida

AAGTCGTAACAAGGTTTTCGTAGGTGAATCTGCGGAAGGATCATTATCGAGAGACGGGGT

CGCTTCGGCCCCACTCTTCACTCGTGTCTACTTACCTTTGTTGCTTTGGCGGGCCTTCGG

GCTTGATCGTTCGACGCGGGTGGGAGGCTTTCGCCTCTCGCCTCGCGAGCGTCCGCCAAA

GGCTCTGTTAACTCTGTTTTAGTGTCTTTCGAGCAATCATGTAATAGTTAAAACTTTCAA

CAACGGATCT

>ASV811 GS|84.8|AF250779|SH0954634.09FU;k:Fungi,p:Ascomycota,c:Lecanoromycetes,o:Caliciales,f:Caliciaceae,g:Buellia,s:Buellia frigida

AAGTCGTAACAAGATTTTCGTAGATAAATTTGCGAAAGAATCATTATCGAGAGACGAGAT

CGCTTCGATCTCACTCTTCACTCATGTCTACTTATCTTTGTTGCTTTGACGAATTTTTGA

GCTTGATCGTTCGACGCGAGTGAGAGACTTTCGTCTATCGTCTCGCGAGCGCTCGTCAAA

GATTCTGTTAACTCTGTTTTAGTGTCTTTCGAGCAATCATGTAATAGTTAAAACTTTCAA

CAACGAATCT

>ASV812 GS|82.8|AF250779|SH0954634.09FU;k:Fungi,p:Ascomycota,c:Lecanoromycetes,o:Caliciales,f:Caliciaceae,g:Buellia,s:Buellia frigida

AAGTCGTAACAAGATTTTCGTAGATAAATCTACGAAAGAATCATTATCGAGAGACGAAAT

CGCTTCGATCTCACTCTTTACTCGTGTCTACTTATCTTTGTTGTTTTGACGAGTCTTCGA

ACTTGATCGTTCGACGCGAATGAAAGACTTTCGTCTCTCGCTTCGCGAGCGTTCGTCAAA

GACTTTGTTAACTCTATTTTAGTGTCTTTCGAGCAATCATGTAATAATTAAAACTTTCAA

CAACGAATCT

>ASV813 SS|0.9400|AY081162|SH1302838.09FU;k:Fungi,p:Ascomycota,c:Lecanoromycetes,o:Teloschistales,f:Teloschistaceae

AAGTCGTAACAAGGTTTCCGTAGGTGAACCTGCGGAAGGATCATTACTAAGAGAGGGATG

TACGCTTCCAGCCGAGTCCCGGGGGGCTGCGCCCCTCACCTCTTCAACCCTGTGTCTACC

AACCGCTGTTGCTTCGGCGAGCGCCGGGGAGCGATTCCCGGCCCTGGCTTCGGTCAGTCA

GCCCTCGTCAGAGGCCCATCCAAATTCTGTTTCAGTGACGTCCGAGTATACCAGCAAATA

AATTAAAACT

>ASV814 GS|99.5|UDB01571356|SH0913113.09FU;k:Fungi,p:Ascomycota,c:Lecanoromycetes,o:Caliciales,f:Caliciaceae,g:Buellia

AAGTCGTAACAAGGTTTCCGTAGGTGAACCTGCGGAAGGATCATTACCGAGAGACGGGGT

CCAACCGGCCCCACTCTTCACCCGTGCCTACCTACCTATGTTGCTTTGGCGGGCCTCCTG

CTCGGCGTCGGTCCGGGGCCTTTCGGGCTTCTTCCCGGCGAGTGCCCGCCGAAGGCTCTG

CAAACTCCTGTTTCGTGTCGTCCGAGTACAAACCTAATAGTTAAAACTTTCAACAACGGA

TCTCTTGGTT

>ASV815 GS|98.6|KU057816|SH1048210.09FU;k:Fungi,p:Basidiomycota,c:Tremellomycetes,o:Tremellales,f:Cryptococcaceae,g:Cryptococcus

AAGTCGTAACAAGGTTTCCGTAGGTGAACCTGCGGAAGGATCATTAGTGATTGGCTTCGG

CCTCATATCCTTTCAAACCTGTGCACCTCTTGGCTCCGGCCAATTCAAACATGTGTCATG

AATGTATATAAACAAAAACATAAACAAAACTTTCAACAACGGATCTCTTGGTTCTG

>ASV816 GS|84.6|KU613347|SH1126450.09FU;k:Fungi,p:Ascomycota

AAGTCGTAACAAGGTTTCCGTAGGTGCATTGTCCCCCCAAGGCTCGCCTACAGGCCCCCG

ACCGACTATAAAGAAATCTACCTAGTTGACTCGCGTCTTCGCGGTCGGCCACGCAGCTAT

TCTGCCCTGGAAGCCGTCCACCTCGTGGAAAGTAACCAGCAGCTCAAAGGATTCACAGAG

AACCTTGAGTCTACAGATCAAATGCCTGCGGCCATGGGCTTCGTGCCTTGGTGTTGATAT

GACCGAACCC

>ASV817 GS|83.3|AF250779|SH0954634.09FU;k:Fungi,p:Ascomycota,c:Lecanoromycetes,o:Caliciales,f:Caliciaceae,g:Buellia,s:Buellia frigida

AAATCGTAACAAGATTTTCGTAAATGAATTTACGAAAAAATCATTATCGAGAGACGAAAT

CGCTTCGATCTCACTCTTCATTCGTATCTACTTATCTTTGTTGCTTTGACGAATCTTCGA

ACTTGATCGTTCGACGCGAGTGAGAGACTTTCGTCTCTCGTTTCGCGAACGCTCGTCAAA

GACTTTGTTAACTCTATTTTAGTGTCTTTCGAGCAATCATGTAATAGTTAAAACTTTCAA

CAACGAATCT

>ASV818 GSL|100.0|AF145328|SH1188866.09FU;k:Fungi,p:Basidiomycota,c:Tremellomycetes,o:Filobasidiales,f:Filobasidiaceae,g:Naganishia

AAGTCGTAACAAGGTTTCCGTAGGTGAACCTGCGGAAGGATCATTAATGATTGAACGTCT

GTCGAGCTTGCTCACAGGCACATCATATCCATAACACCTGTGCACTTGTCGGATGGCTTA

GTGAAGACCGCAAGGTTGGATCTATCCATCTACTTTACATAACAATTCTGTAACAAATGT

AGTCTTATTATAACATAATAAAACTTTCAACAACGGATCTCTTGGCTCTC

>ASV819 GS|99.0|AF250779|SH0954634.09FU;k:Fungi,p:Ascomycota,c:Lecanoromycetes,o:Caliciales,f:Caliciaceae,g:Buellia,s:Buellia frigida

AAGTCGTAACAAGGTTTTCGTAGATGAACCTGCGAAAGGATCATTATCGAGAGACGGGGT

CGCTTCGGCCCCACTCTTCACCCGTGTCTACTTACCTTTGTTGCTTTGGCGGGCCTTCGG

GCTTGACCGTTCGACGCGAGTGGGAGGCTTTCGCCTCTCGCCTCGCGAGCGCCCGCCAAA

GGCCCTGTTAACTCTGTTTTAGTGTCTTCCGAGCAACCATGTAATAGTTAAAACTTTCAA

CAACGGATCT

>ASV820 SS|1.0000|AF250779|SH0954634.09FU;k:Fungi,p:Ascomycota,c:Lecanoromycetes,o:Caliciales,f:Caliciaceae,g:Buellia,s:Buellia frigida

AAGTCGTAACAAGATTTTCGTAGATGAACCTGCGGAAGGATCATTATCGAGAGACGGGAT

CGCTTCGGCCCCACTCTTCACTCGTGTCTACTTACCTTTGTTGCTTTGACGAGTCTTCGA

GCTTGATCGTTCGACGCGGGTGGGAGGCTTTCGCCTCTCGCCTCGCGAGCGCCCGCCAAA

GGCCCTGTTAACTCTGTTTTAGTGTCTTTCGAGCAACCATGTAATAGTTAAAACTTTCAA

CAACGGATCT

>ASV821 GS|82.8|AF250779|SH0954634.09FU;k:Fungi,p:Ascomycota,c:Lecanoromycetes,o:Caliciales,f:Caliciaceae,g:Buellia,s:Buellia frigida

AAATCGTAACAAGATTTTCGTAAATGAATCTACGAAAGAATCATTATCGAGAGACGAAAT

CGCTTCGATCTCACTCTTCATTCGTATCTACTTACCTTCGTTGCTTTGACGAGTCTTCGA

ACTTAATCGTTCGACGCGAGTGAGAGACTTTCGTCTCTCGTTTCGCGAGCGCTCGTCAAA

GACTCTATTAACTCTATTTTAGTGTCTTTCGAGCAATCATGTAATAATTAAAATTTTCAA

CAACGAATCT

>ASV822 GS|84.0|AY667583|SH0954634.09FU;k:Fungi,p:Ascomycota,c:Lecanoromycetes,o:Caliciales,f:Caliciaceae,g:Buellia,s:Buellia frigida

AAGTCGTAACAAGATTTTCGTAGATGAACTTGCGAAAGAATCATTATCGAGAGACGAAAT

CGCTTCGATCTCACTCTTCATTCGTATCTACTTATCTTTGTTGCTTTGACGAGTCTTCGA

GCTTGATCGTTCGACGCGAGTGAGAGACTTTCGTCTCTCGTCTTGCGAGCGCTCGTCAAA

GATTTTGTTAACTCTATTTTAGTGTCTTTCGAACAATCATGTAATAATTAAAACTTTCAA

CAACGAATCT

>ASV823 SS|1.0000|AF250779|SH0954634.09FU;k:Fungi,p:Ascomycota,c:Lecanoromycetes,o:Caliciales,f:Caliciaceae,g:Buellia,s:Buellia frigida

CTTTTTCCTTTCTCTTCCTTCCCCTTCTTCCCCCTTTTTCCGTATTTTCCCCTGCGGACT

TCTCCTTACCGATCTCCTTGGTCGCTTCGGCCCCACTCTTCACCCGTGTCTACTTACCTT

TGTTGCTTTGGCGGGCCTTCGGGCTTGACCGTTCGCCGCGGGTGGGAGGCTTTCGCCTCT

CGCCTCGCTCGCGCCCGCCAAAGGCCCTGTTAACTCTGTTTTAGTGTCTTCCGAGCAACC

ATGTAATAGT

>ASV824 SS|0.9800|UDB0700338|SH1084495.09FU;k:Fungi

AAGTCGTAACAAGGTCTCCGTAGGTGAACCTACGGAGGGATCATTACCGAGTAAGGGCCT

CCGGGCTCGACCTCTAACCCTTTGTTGTACCCACTACAGTTGCCGCGGGGGCGACCCGGC

CTCCGCGTCGGGGCCCCCGGTGGACCCAACTAAACAACTCTGCGTCTTTGCGTCTAAGTA

TTAAAGTAAATCAATTAAAACTTTCAACAACGGATCTCTTAGTTCTA

>ASV825 SS|1.0000|AF250779|SH0954634.09FU;k:Fungi,p:Ascomycota,c:Lecanoromycetes,o:Caliciales,f:Caliciaceae,g:Buellia,s:Buellia frigida

AAGTCGTAACAAGATTTTCGTAGATGAATCTACGAAAGAATCATTATCGAGAGACGAGAT

CGCTTCGATCTCACACTCTTCACTCGTGTCTACTTACCTTTGTTGCTTTGGCGGGCCTTC

GGGCTTGACCGTTCGACGCGGGTGGGAGGCTTTCGCCTCTCGCCTCGCGAGCGCCCGCCA

AAGGCCCTGTTAACTCTGTTTTAGTGTCTTCCGAGCAACCATGTAATAGTTAAAACTTTC

AACAACGGAT

>ASV826 GS|73.7|DQ534454|SH0954616.09FU;k:Fungi,p:Ascomycota,c:Lecanoromycetes,o:Caliciales,f:Caliciaceae,g:Buellia,s:Buellia russa

AAGTCGTAACAAAATTTTCGTAAATGAATTTGCGAAAAAATCATTATCGAGAGACGAAAT

CGATTCGATCTCACTCTTCATTCGTGTCTACTTATCTTTGTTATTTTGACGAGTCTTCGA

ACTTGATCGTTCGACACGAATGAGAGACTTTCGTTTCTCGTTTCGCGAGCGCTCGTCAAA

GATTCTATTAACTCTATTTTAATGTCTTTCGAGCAATCATATAATAATTAAAACTTTCAA

TAACGAATCT

>ASV827 GS|100.0|UDB06397502|SH1003737.09FU;k:Fungi,p:Basidiomycota,c:Tremellomycetes,o:Tremellales,f:Tremellaceae,g:Tremella

AAGTCGTAACAAGGTTTCCGTAGGTGAACCTGCGGAAGGATCATTAGTGAGCCGAGAGGC

GTAAAATCCAAACCTCTGTGAACCGTGCCTTCGGGCTTTACAACAAACCAGTGTGATGAA

CGTAATACAATAGAAACAAAACTTTCAACAACGGATCTCTTGGCTCTC

>ASV828 GSL|100.0|MK216328|SH0966251.09FU;k:Fungi,p:Basidiomycota,c:Tremellomycetes

AAGTCGTAACAAGGTTTCCGTAGGTGAACCTGCGGAAGGATCATTAGTGAATTGAGCTGG

CTTCGGCCGCTCTGATCTATACACACCTGTGAACTGTTGGCCTTCGGGTCTTTAATTACA

ACTGTGACGAACGTAAATTATTATAACAAATAAAACTTTTAACAACGGATCTCTTGGCTC

TC

>ASV829 GS|75.5|UDB01703015|SH1008128.09FU;k:Rhizaria,p:Cercozoa

AAGTCGTAACAAGGTCTTCGTAGGTGAACCTGCGAAGGGATCATTAACACGTTCCAAACC

TATTATCCACCCATCTGTGAACTCATGTAACCGCCGATATGGCTGGCAACAAGTGTATGG

CGCATACGATCGACTCTATTGATGTGAGGTGTAGTGCGAGTCAGTACCAACTGCTCGTAC

TACCCCTGGTCTAGTATGCCCCGTGCTCCTCTACTCCCACTCTTCGGAGTGAGGCTAGCA

GAGCAGCTGT

>ASV830 GS|100.0|KF309957|SH0991924.09FU;k:Fungi,p:Ascomycota,c:Dothideomycetes,o:Mycosphaerellales,f:Teratosphaeriaceae,g:Elasticomyces,s:Elasticomyces elasticus

AAGTCGTAACAAGGTCTCCGTAGGTGAACCTGCGGAGGGATCATTACCGAGTGAGGGCCT

CCGGGTCCGACCTCCAACCCCATGTTAACCGACCCTGTTGCCTCGGGGGCGACCCGGACT

CGTGCCGGGGCCCCCGATGGACCTTCAAACACTGCATCTTTGCGTCCGAGTCAATATTTG

AATAAATCAAAACTTTCAACAACGGATCTCTTGGTTCTG

>ASV831 SS|1.0000|AY667583|SH0954634.09FU;k:Fungi,p:Ascomycota,c:Lecanoromycetes,o:Caliciales,f:Caliciaceae,g:Buellia,s:Buellia frigida

AAGTCGTAACAAGGTTTTCGTAGGTGAACCTGCGGAAGGATCATTATCGAGAGACGGGGT

CGCTTCGGTCTCACTCTTCACCCGTGTCTACTTATCTTTGTTGCTTTGGCGGGCCTTCGA

GCTTGATCGTTCGACGCGGGTGGGAGGCTTTCGCCTCTCGTCTCGCGAGCGCTCGCCAAA

GGCCCTGTTAACTCTGTTTTAGTGTCTTTCGAGCAACCATGTAATAGTTAAAACTTTCAA

CAACGGATCT

>ASV832 SS|1.0000|AF250779|SH0954634.09FU;k:Fungi,p:Ascomycota,c:Lecanoromycetes,o:Caliciales,f:Caliciaceae,g:Buellia,s:Buellia frigida

CTTTTTCCTTTCTCTTCCTTCCCCTTCTTCCCCCTTTTTCCGTATTTTCCCCTGCGGACT

TCTCCTTACCGATCTCCTTGGTCGCTTCTTCCCCACTCTTCCCCCGTGTCTACTTACCTT

TGTTGCTTTGGCGGGCCTTCGGGCTTGACCGTTCGCCGCGGGTGGGAGGCTTTCGCCTGT

CGCCTCGCGAGCGCCCGCCAAAGGCCCTGTTAACTCTGTTTTAGTGTCTTCCGAGCAACC

ATGTAATAGT

>ASV833 SS|0.9300|KC965634|SH0916490.09FU;k:Fungi,p:Ascomycota,c:Lecanoromycetes

AAGTCGTAACAAGGTTTCCGTAGGTGAACCTGCGGAAGGATCATTATCGAGAGACGAAGT

CTAACCGGCCCCACTCTTCACCCGTGTATACCTACCCTTTGTTGCTTTGGCGGGCGGTCG

ATCTTTACCGCCCGACGTCGGTCGGAAGGCTCGCGCCTTCCACTCACCGAACGCCCGCCA

GAGACCCCCAAAACCCGTCCATCTGTGTCCGAGCGAGTGAAGCAAATAATTAAAAACTTT

CAACAACGGA

>ASV834 SS|0.9200|MZ229869|SH0913119.09FU;k:Fungi,p:Ascomycota,c:Lecanoromycetes

AAGTCGTAACAAGGTTTCCGTAGGTGAACCTGCGGAAGGATCATTATCGAGAGACGGAGT

CTAACCGGCCCCACTCTTCACCCGTGTATACCTACCCTTTGTTGCTTTGGCGGGCGGTCG

GTCTTTACCGCCCGACGTCGGTCGGAAGGCTCGCGCCTTCCACTCACCGAACGCCCGCCA

GAGGCCCCCCAAACCCGTCCATCTGTGTCCGAGCGAGTGAAGCAAATAATTAAAAACTTT

CAACAACGGA

>ASV835 GS|87.0|MK970687|SH0954634.09FU;k:Fungi,p:Ascomycota,c:Lecanoromycetes,o:Caliciales,f:Caliciaceae,g:Buellia,s:Buellia frigida

AAGTCGTAACAAGATTTTCGTAGATGAATTTGCGAAAGGATCATTATCGAGAGACGAGGT

CGCTTCGATCTCACTCTTCATTCGTGTCTACTTATCTTTGTTGCTTTGATGAGTCTTCGA

GCTTGATCGTTCGATGCGAGTGGGAGACTTTCGTCTCTCGTCTCGCGAGCGCTCGTCAAA

GACTTTGTTAACTTTGTTTTAGTGTCTTTCGAGCAATCATGTAATAGTTAAAACTTTCAA

CAACGGATCT

>ASV836 GS|86.3|AF250779|SH0954634.09FU;k:Fungi,p:Ascomycota,c:Lecanoromycetes,o:Caliciales,f:Caliciaceae,g:Buellia,s:Buellia frigida

AAGTCGTAACAAGATTTTCGTAGATGAATTTGCGAAAGAATCATTATCGAGAGACGAAAT

CGCTTCGATCTCACTCTTCATTCGTGTCTACTTATCTTTGTTGCTTTGACGAGTCTTCGA

GCTTGATCGTTCGACGCGAGTGAAAGACTTTCGTCTCTCGCCTCGCGAGCGCTCGTCAAA

GACTCTGTTAACTTTGTTTTAGTGTCTTTCGAGCAATCATGTAATAGTTAAAACTTTCAA

CAACGAATCT

>ASV837 GS|98.1|KP174855|SH1071027.09FU;k:Fungi,p:Ascomycota,c:Eurotiomycetes,o:Chaetothyriales,f:Trichomeriaceae,g:Knufia,s:Knufia separata

AAGTCGTAACAAGGTTTCCGTAGGTGAACCTGCGGAAGGATCATTACCGAGTCAGGGTCT

CTTCGGAGCCCGAACCTCCCAACCCTTTGTCTAATTTACCTTGTCGTTGCTTCGGCGGAC

CGGTTGACCAACTGGTCTTGACCGCCGGGGGTCCCGCACCCCTGGAGAGCGTCCGCCGAC

GGCCCAACCACAAACTCTTGTACTAAACCATGTCGTCTGAATGTACTTGATATTAATCAA

AAAAACAAAA

>ASV838 GS|81.9|AF250779|SH0954634.09FU;k:Fungi,p:Ascomycota,c:Lecanoromycetes,o:Caliciales,f:Caliciaceae,g:Buellia,s:Buellia frigida

AAGTCGTAACAAGATTTTCGTAAATGAATTTGCGAAAAAATCATTATCGAAAGACGAAAT

CGCTTCGATCTCACTCTTCACTCGTATTTACTTATCTTTGTTGCTTTGACGAGTTTTCGA

ATTTGATCGTTCGACGCGAGTGAGAGACTTTCGTCTCTCGTCTTGCGAGCGCTCGTCAAA

GACTTTATTAACTCTATTTTAGTGTCTTTCGAACAATCATGTAATAGTTAAAACTTTCAA

CAACGAATCT

>ASV839 GS|86.4|AY667583|SH0954634.09FU;k:Fungi,p:Ascomycota,c:Lecanoromycetes,o:Caliciales,f:Caliciaceae,g:Buellia,s:Buellia frigida

AAGTCGTAACAAGATTTTCGTAGATGAACTTGCGAAAGAATCATTATCGAGAGACGAGAT

CGCTTCGATCTCACTCTTCACTCGTGTCTACTTACCTTTGTTGCTTTGGCGAGTCTTTGA

ACTTGATCGTTCGACGCGAATGAAAGGCTTTCGTTTCTCGTCTCGCGAGCGCTCGTCAAA

GGCCTTGTTAACTCTATTTTAGTGTCTTCCGACCAATCATGTAATAATTAAAACTTTCAA

CAACGAATCT

>ASV840 SS|1.0000|AY667583|SH0954634.09FU;k:Fungi,p:Ascomycota,c:Lecanoromycetes,o:Caliciales,f:Caliciaceae,g:Buellia,s:Buellia frigida

AAGTCGTAACAAGGTTTCCGTAGGTGAACCTGCGAAAGAATCATTATCGAGAGACGGGGT

CGCTTCGACCCCACTCTTCACCCGTGTCTACTTACCTTTGTTGCTTTGGCGGGTCTTCGA

GCTTGACCGTTCGACGCGAGTGGAAGACTTTCGTCTCTCGCCTCGCGAGCGCCCGTCAAA

GACCCTGTTAACTCTATTTTAGTGTCTTCCGACCAACCATGTAATAGTTAAAACTTTCAA

CAACGAATCT

>ASV841 GS|85.3|AF250779|SH0954634.09FU;k:Fungi,p:Ascomycota,c:Lecanoromycetes,o:Caliciales,f:Caliciaceae,g:Buellia,s:Buellia frigida

AAGTCGTAACAAGATTTTCGTAGATGAATTTGCGAAAGAATCATTATCGAGAGACGAAAT

CGCTTCGATCTTACTCTTCACTCGTGTCTACTTATCTTTGTTGCTTTGACGAATCTTTGA

GCTTGATCGTTCGACGCGAGTGAGAGACTTTCGTCTCTCGTCTCGCAAGCGCTCGTCAAA

GACTTTGTTAACTTTGTTTTAGTGTTTTCCGAGCAACCATGTAATAGTTAAAACTTTCAA

CAACGGATCT

>ASV842 SS|1.0000|AF250779|SH0954634.09FU;k:Fungi,p:Ascomycota,c:Lecanoromycetes,o:Caliciales,f:Caliciaceae,g:Buellia,s:Buellia frigida

AAGTCGTAACAAGATTTTCGTAAATGAATTTGCGAAAGAATCATTATCGAGAGATGAGAT

CGCTTCGATCTCACTCTTCACTCGTATTTACTTATCTTTGTTGCTTTGACGAGTCTTCGA

ACTTGATCGTTCGACGCGAGTAAGAGACTTTCGTCTCTCGCCTCGCGAGCGCCCGCCAAA

GGCCCTGTTAACTCTGTTTTAGTGTTTTCCGAGCAACCATGTAATAGTTAAAACTTTCAA

CAACGGATCT

>ASV843 SS|0.8700|MK970689|SH0954625.09FU;k:Fungi,p:Ascomycota,c:Lecanoromycetes,o:Caliciales,f:Caliciaceae,g:Buellia

AAGTCGTAACAAGGTTTCCGTAGGTGAACCTGCGGAAGGATCATTATCGAGAGGGGTCCC

CGGACTCCGGGGGCTTCGGCCCCCTACTCTTCACCCTATGTCTACACACCTTTGTTGCTT

TGGCGGGCCTTCGGGTCTTACCACCCGGGGCTGGTCGGGGGCCTTTTGCCCCTCGCTCCG

CGAGTGCCTGCCAATGATTTTGTCAACTCTGTTCTTAGTGTCCTCTGAGCAACCATACAA

TAGTTAAAAC

>ASV844 GS|98.4|FJ553294|SH0920183.09FU;k:Fungi,p:Ascomycota,c:Eurotiomycetes,o:Chaetothyriales,f:Herpotrichiellaceae,g:Cladophialophora,s:Cladophialophora humicola

AAGTCGTAACAAGGTTAAATATCGATGTAGGTAAACCACCTCCGAAGCCTTGAACTCTAG

TCTATTCTTAATAGGCTACACCGGTCTGTTGCTAGGCTTAACATTTATATTAGTTAAATG

TTTAACTAGCAGTCGTTGATACGATTCACAGGTCAAACACCGGTGGTGAGCAAATTTCGC

GAATTTAGATATGATCGGTCAGTCTTTGTTAAGAAGTTTGATAAGCGTTCCGTAGGTGAA

CCTGCGGAAG

>ASV845 GSL|100.0|EF394861|SH0995195.09FU;k:Fungi,p:Ascomycota,c:Dothideomycetes,o:Mycosphaerellales,f:Mycosphaerellaceae,g:Ramularia

AAGTCGTAACAAGGTCTCCGTAGGTGAACCTGCGGAGGGATCATTACTGAGTGAGGGAGC

AATCCCGACCTCCAACCCTTTGTGAACGCATCATGTTGCTTTGGGGGCGACCCTGCCGTT

CGCGGCATTCCCCCCGAAGGTCATCAAAACACTGCATTCTTACGTCGGAGTATAAAGTTA

ATTTAATAAAACTTTCAACAACGGATCTCTTGGTTCTG

>ASV847 GS|99.2|JX036119|SH0942719.09FU;k:Fungi,p:Ascomycota,c:Lecanoromycetes,o:Lecanorales,f:Lecanoraceae,g:Rhizoplaca

AAGTCGTAACAAGGTTTCCGTAGGTGAACCTGCGGAAGGATCATTATCGAGAGGGGTCCC

CGGACTCCGGGGGCTTCGGCCCCCTACTCTTCACCCTATGTCTACACACCTTTGTTGCTT

TGGCGGGCCTCGGGTTCGCCCCGTACCTGCCGTGGGCTTCCATACCCCGGCCGTCCGTGC

CCGTCAGAGGCCCACGAACCCTCGTTTATCAGTGTCGTCCGAGTCCAACCATAATAGTAA

AAACTTTCAA

>ASV848 GS|81.9|AF250779|SH0954634.09FU;k:Fungi,p:Ascomycota,c:Lecanoromycetes,o:Caliciales,f:Caliciaceae,g:Buellia,s:Buellia frigida

AAGTCGTAACAAGATTTTCGTAAATGAATTTGCGAAAAAATCATTATCGAGAGACGAAAT

CGCTTCGATCTCACTCTTCACTCGTATCTACTTATCTTTGTTGCTTTGACGAGTTTTCGA

GCTTGATCGTTCGACGCGAGTGAAAGACTTTCGTCTCTCGTCTTGCGAGCGTTCGTCAAA

GATCTTGTTAACTCTATTTTAGTGTCTTTCAAGCAATCATGTAATAATTAAAATTTTCAA

TAACGAATCT

>ASV849 GS|85.8|AF250779|SH0954634.09FU;k:Fungi,p:Ascomycota,c:Lecanoromycetes,o:Caliciales,f:Caliciaceae,g:Buellia,s:Buellia frigida

AAGTCGTAATAAGATTTTCGTAGATGAATTTGCGAAAGAATCATTATCGAGAGACGAAAT

CGCTTCGATTTCACTTTTCACTCGTGTCTACTTATCTTTGTTGCTTTGACGAGCCTTCGA

GCTTGATCGTTCGACGCGAGTGAGAGACTTTCGTCTCTCGTCTCGCGAGCGTTCGTCAAA

GACTCTATTAACTCTATTTTAGTGTCTTTCGAGCAATCATGTAATAGTTAAAACTTTCAA

CAACGGATCT

>ASV850 GS|73.8|JF794067|SH0954616.09FU;k:Fungi,p:Ascomycota,c:Lecanoromycetes,o:Caliciales,f:Caliciaceae,g:Buellia,s:Buellia russa

AAATCGTAACAAAATTTTTATAAATGAATTTGCAAAAGAATCATTATCGAGAGACGAAAT

CGTTTCGATTTTACTCTTCACTCGTATCTATTTATCTTTGTTGCTTTGACGAGTCTTCGA

ACTTGATCATTCGATGCGAATAAGAGACTTTCGTCTCTCGTTTCGCGAACGTTCGTCAAA

GACTTTATTAACTTTGTTTTAATGTTTTTCGAGCAATCATGTAATAGTTAAAACTTTCAA

TAACGAATCT

>ASV851 GS|0.0|None;No hit

GTGTCAGCAGCCGCGGTAATACGAAGGGGGCTAGCGTTGCTCGGAATGACTGGGCGTAAA

GGGCGCGTAGGCGGTATGGACAGTCAGGTGTGAAATTCCCGGGCTTAACCTGGGGACTGC

ATTTGATACGTTCAAACTAGAGTGTGGAAGAGGGTCGTGGAATTCCCAGTGTAGAGGTGA

AATTCGTAGATATTGGGAAGAACACCGGTGGCGAAGGCGGCGACCTGGTCCATTACTGAC

GCTGAGGCGC

>ASV852 SS|0.8700|AF279770|SH1300516.09FU;k:Fungi,p:Ascomycota,c:Lecanoromycetes,o:Teloschistales,f:Teloschistaceae

AAGTCGTAACAAGGTTTCCGTAGGTGAACCTGCGGAAGGATCATTACCGAGAGACGGGGC

CTAATCGGCCCCACTCTTCACCCGTGTATACCTATCCTTTGTTGCTTCGGCGAGCGTCGG

GGCGTCCGCGCCCCGGCCCCGGCTTCGGTCGGTGAGCTCTCGCAGAGGCCTATCTTTATT

CTGTTTTGCAGTGACGTCCGAGAATACCAATATAATCAATCAAAACTTTCAACAACGGAT

CTCTTGGTTC

>ASV853 GS|0.0|None;No hit

GTGTCAGCAGCCGCGGTAATACAGAGGGTGCGAGCGTTAATCGGATTTACTGGGCGTAAA

GCGTGCGTAGGCGGCTTCTTAAGTCGGATGTGAAATCCCTGAGCTTAACTTAGGAATTGC

ATTCGATACTGGGAAGCTAGAGTATGGGAGAGGATGGTAGAATTCCAGGTGTAGCGGTGA

AATGCGTAGAGATCTGGAGGAATACCGATGGCGAAGGCAGCCATCTGGCCTAATACTGAC

GCTGAGGTAC

>ASV854 GS|99.5|UDB01604532|SH1277830.09FU;k:Fungi,p:Ascomycota,c:Candelariomycetes,o:Candelariales,f:Candelariaceae,g:Candelariella,s:Candelariella flava

AAGTCGTAACAAGGTTTCCGTAGGTGTAAGTAATCCAACGTCCCAAACATTCACCATCCA

ATGCGTGATCATGCTAACATGTGATTTAGGAACCTGCGGAAGGATCATTAAAGAGCAAGG

GTCTTCTAGGCCCGACCTCCAACCCTTTGTATATCTACCTCTGTTGCTTTGGCGGGCCCG

TTGGGGTGACCCACCGCCGGCTTTCAGCCGGTGGGTGCCCGTCAGAGTCCACGTAAACTC

TGTCTATCTA

>ASV855 SS|0.8500|UDB01720327|SH1085256.09FU;k:Rhizaria,p:Cercozoa

AAGTCGTAACAAGGTCTTCGTAGGTGAACCTGCGAAGGGATCATTAACACGTTCCATACC

TCCATTATCAACCCATCTGTGAACTTTTGTTACCGCCGAATGGCTAACTACAAGTGTATG

GCGCATACGATCGACTCAACGATGTTGTAGGACTCACTGTTCTGCATGGTCTAGTAGTAT

GCCCTCTGTGTCCGTCACACGACAGCTATCCTTGTTTGTTGGTCCGGCCACTATATATGC

GCTTCGACGG

>ASV856 SS|0.8400|MG982528|SH1003748.09FU;k:Fungi,p:Basidiomycota,c:Tremellomycetes,o:Tremellales

AAGTCGTAACAAGGTTTCCGTAGGTGAACCTGCGGAAGGATCATTAGTGATTCGGCCCTC

ACGGGTCTATAAAAGACACCTCTGTGAACCTGTCGGCCTCCGGGCCCACCTGCAAACACT

GTGTAACGAGCGTTGATGTATCATAAGCATAATAAAACTCTCAACAACGGATCTCTTGGC

TCTC

>ASV857 SS|0.8800|MT809481|SH1325656.09FU;k:Fungi,p:Ascomycota

AAGTCGTAACAAGGTTTCCGTAGGTGAACCTGCGGAAGGATCATTACCGAGCTAGGGTCT

CTGGCCCAACCTCCAACCCCACGTGTACAGAACCTTTGTTGCTTTGGCGGGCCGTTGGGT

GCAAACCCGCCCCGGCCTTTCGTGGCTCGCGTGTGCCTGCCAGGGGACCCTTTGAACCCG

ATATGTCCGTGCCGTCTGAGTGGGATTCAAATCAAGCAAAACTTTCAACAACGGATCTCT

TGGTTCTG

>ASV858 SS|1.0000|MF138063|SH0954212.09FU;k:Fungi,p:Ascomycota,c:Lecanoromycetes,o:Acarosporales,f:Acarosporaceae,g:Acarospora

AAGTCGTAACAAGGTTTCCGTAGGTGAACCTGCGGAAGGATCATTACAGAGTTAGGGTCT

TTTCAGGCTCGATCTCCAACCCTATGTGTACCTACCTTTGTTGCTTTGGCGGGCCCGTTG

GGGCGACCCACCGGTGGCCTTTGGCTCCCGAGTGCCCGTCAGAGATCCATCAAAACCTTT

CAATTGTGTCGTCTGAGTACCAACATAATAATTAAAACTTTCAACAACGGATCTCTTGGT

TCTG

>ASV859 SS|1.0000|AF250779|SH0954634.09FU;k:Fungi,p:Ascomycota,c:Lecanoromycetes,o:Caliciales,f:Caliciaceae,g:Buellia,s:Buellia frigida

CTTTTTCCTTTCTCTTCCTTCCCCTTCTTCCCCCTTTTTCCGTATTTTCCCCTGCGGACT

TCTCCTTACCGATCTCCTTGGTCGCTTCTTCCCCACTCTTCCCCCTTGTCTACTTACCTT

TGTTGCTTTGGCGGGCCTTCGGGCTTGACCTTTCTCCGCGGGTGGGAGGCTTTCGCCTCT

CGCCTCTCTCTCGCCCGCCAAAGGCCCTGTTAACTCTGTTTTAGTGTCTTCCGAGCAACC

ATGTAATAGT

>ASV860 SS|0.8900|AY667583|SH0954634.09FU;k:Fungi,p:Ascomycota,c:Lecanoromycetes,o:Caliciales,f:Caliciaceae,g:Buellia,s:Buellia frigida

AAGTCGTAACAAGATTTCCGTAGATGAACCTGCGAAAGGATCATTATCGAGAGACGAGGT

CGCTTCGGCCTCACTCTTCACCCGTGTCTACTTATCTTTGTTGCTTTGATGGACCTTCGG

GCTTGATCGTTCGACGCGGGTGGGAGGCTTTCGTCTCTCGCCTCGCGAGCGCTCGTCAAA

GATTCTGTTAACTCTGTTTTAGTGTCTTTCGAGCAATCATGTAATAGTTAAAACTTTCAA

CAACGAATCT

>ASV861 GS|79.4|AF250779|SH0954634.09FU;k:Fungi,p:Ascomycota,c:Lecanoromycetes,o:Caliciales,f:Caliciaceae,g:Buellia,s:Buellia frigida

AAGTCGTAACAAGATTTTCATAAATGAATTTACGAAAGAATCATTATCGAGAGACGAAAT

CGCTTCGATTTCATTTTTCATTCGTATCTACTTATCTTTGTTACTTTGACGAGTTTTCGA

GCTTGATCGTTCGATGCGAGTGAAAGACTTTCGTCTATCGTTTTGCGAACGCTCGTCAAA

GACTCTATTAACTCTATTTTAGTGTCTTTCGAACAATCATGTAATAGTTAAAACTTTCAA

CAACGAATCT

>ASV862 GS|87.3|AF250779|SH0954634.09FU;k:Fungi,p:Ascomycota,c:Lecanoromycetes,o:Caliciales,f:Caliciaceae,g:Buellia,s:Buellia frigida

AAGTCGTAACAAGATTTTCGTAAGTGAATTTGCGAAAGAATCATTATCGAGAGACGAGGT

CGCTTCGACCTCACTCTTCACTCGTGCCTACTTATCTTTGTTGCTTTGACGAGTCTTCGA

ACTTGATCGTTCGACGCGAGTGGGAGACTTTCGTCTCTCGCCTCGCGAGCGCTCGTCAAA

GACCTTGTTAACTTTATTTTAGTGTCTTTCGAGCAATCATGTAATAATTAAAACTTTCAA

CAACGAATCT

>ASV863 GSL|100.0|MT535828|SH0884485.09FU;k:Fungi,p:Ascomycota,c:Eurotiomycetes,o:Eurotiales,f:Aspergillaceae,g:Penicillium

AAGTCGTAACAAGGTTTCCGTAGGTGAACCTGCGGAAGGATCATTACCGAGTGAGGGCCC

TCTGGGTCCAACCTCCCACCCGTGTTTATTTTACCTTGTTGCTTCGGCGGGCCCGCCTTA

ACTGGCCGCCGGGGGGCTTACGCCCCCGGGCCCGCGCCCGCCGAAGACACCCTCGAACTC

TGTCTGAAGATTGTAGTCTGAGTGAAAATATAAATTATTTAAAACTTTCAACAACGGATC

TCTTGGTTCC

>ASV864 GS|81.2|AY667583|SH0954634.09FU;k:Fungi,p:Ascomycota,c:Lecanoromycetes,o:Caliciales,f:Caliciaceae,g:Buellia,s:Buellia frigida

AAGTCGTAACAAGATTTTCGTAAATGAATTTGCGAAAGAATCATTATCGAGAAACGAAAT

CGCTTCGATCTCACTCTTCACTCGTGTTTACTTATCTTTGTTGCTTTGACGAGTCTTCGA

GCTTGATCGTTCGACGCGAATGAGAGACTTTCGTCTCTCGTTTCGCGAGCGTTCGTCAAA

GATTTTGTTAACTCTATTTTAGTATCTTTCAAGTAATTATGTAATAATTAAAACTTTCAA

CAACGAATCT

>ASV865 SS|1.0000|AY667583|SH0954634.09FU;k:Fungi,p:Ascomycota,c:Lecanoromycetes,o:Caliciales,f:Caliciaceae,g:Buellia,s:Buellia frigida

AAGTCGTAACAAGGTTTCCGTAGATGAACCTACGAAAGGATCATTACCGAGAGACGAGAT

CGCTTCGGCCCCACACTCTTCATTCGTGTCTACTTACCTTTGTTGCTTTGACGAGCCTTC

GAGCTTGACCGTTCGACGCGAGTGGGAGACTTTCGTCTCTCGCCTCGCGAGCGCCCGCCA

AAGACCCTATTAACTCTGTTTTAATGTCTTCCGAGCAACCATGTAATATTTAAAACTTTC

AACAACGAAT

>ASV866 SS|0.9000|KF617359|SH0989107.09FU;k:Fungi,p:Ascomycota,c:Leotiomycetes,o:Helotiales

AAGTCGTAACAAGGTTTCCGTAGGTGAACCTGCGGAAGGATCATTACCGAGTTCATGCCC

CCCGGGGTAGCTCTCCCACCCTGTTTACTTTACTCATGTTGCTTTGGCGGGCCGACCGCC

CAGGCCATCGGCGTCTCGCTGGTGTGTGCCCGCCAGAGGCCCTACAACTCTGATATTGAA

CGTGCCGTCCGAGTACTATACAAAATTTTAAAACTTTCAACAACGGATCTCTTGGTTCTG

>ASV867 SS|0.8700|MN592663|SH1107827.09FU;k:Fungi,p:Ascomycota,c:Lecanoromycetes

AAGTCGTAACAAGGTTTCCGTAGGTGAACCTGCGGAAGGATCATTATCGAGAGGGGGGGC

TCCATGCCCCGGGGCTCTGTCCCCGTACCTTTTCACCCTGTGTGTATTTTTCCCCCGTTG

CTTTGGCGGGCCCCGGGTCTTCCCCCGGCGTTAGCCCCCTCGCGGGGTTCGCGAGCGCCC

GCCGGAAGGCTTATCGAAACTCTGATTAGTACAGTCTGAGCGTACGAATAATAAATTAAA

ACTTTTAACA

>ASV868 GS|0.0|None;No hit

GTGTCAGCAGCCGCGGTAATACGGAGGATCCGAGCGTTATCCGGATTTATTGGGTTTAAA

GGGTGCGTAGGCGGCCTGTTAAGTCAGGGGTGAAATTTTCCGGCTCAACCGGGGACTTGC

CTTTGATACTGACGGGCTTGAATGCAGCTGAGGTAGGCGGAATGTGACAAGTAGCGGTGA

AATGCATAGATATGTCACAGAACACCGATTGCGAAGGCAGCTTACCAAAGTGCGATTGAC

GCTGAGGCAC

>ASV869 GS|100.0|JN873899|SH0942704.09FU;k:Fungi,p:Ascomycota,c:Lecanoromycetes,o:Lecanorales,f:Lecanoraceae,g:Lecidella,s:Lecidella siplei

AAGTCGTAACAAGGTTTCCGTAGGTGAACCTGCGGAAGGATCATTAATGAGAGAGGGGCT

TTGCGCTCCGGCGGCTTCGCGGCTGCCGACTCTTCACCCTGTGTCTAACCTACCCATGTT

GCTTTGGCGGGCCCTGGGGGTAACCTCACGCCAACCTTGGGCTTCGCGGTCCGGGTCGGT

CAGCGCCCGTCGAAGGCCCCCATCGACTCTGTTTATCAGTGATGTCCGAGTACAAACACA

ATAATCAAAA

>ASV870 GS|80.4|AY667583|SH0954634.09FU;k:Fungi,p:Ascomycota,c:Lecanoromycetes,o:Caliciales,f:Caliciaceae,g:Buellia,s:Buellia frigida

AAGTCGTAACAAAATTTTCGTAAATGAATTTGCGAAAAAATCATTATCGAGAGACGAAAT

CGTTTCGATTTCACTCTTCACTCGTATTTACTTATCTTTGTTGCTTTGACGAATCTTCGA

GCTTGATCGTTCGACGCGAGTGAGAGATTTTCGTCTCTCGTCTCGCGAGCGCTCGTCAAA

AATCTTGTTAACTTTGTTTTAATGTCTTTCGAGCAATCATATAATAGTTAAAACTTTTAA

CAACGAATTT

>ASV871 GS|100.0|KM062081|SH1001330.09FU;k:Fungi,p:Ascomycota,c:Dothideomycetes,o:Mycosphaerellales,f:Extremaceae,g:Vermiconia,s:Vermiconia calcicola

AAGTCGTAACAAGGTCTCCGTAGGTGAACCTGCGGAGGGATCATTACCGAGTGAGGGTGG

AAACACCCGACCTCCAACCCCATGTCGTTACAACCTTTGTTGCCTCGGGGGCGACCCGGC

CTCGCGCCGGGGCCCCCGACGGACCAGCTCACTCTGCGTCTTTGCGTCGGAGTCACAAGT

AAATTGAATCAAAACTTTTAACAACGGATCTCTTGGTTCTG

>ASV872 SS|1.0000|AY667583|SH0954634.09FU;k:Fungi,p:Ascomycota,c:Lecanoromycetes,o:Caliciales,f:Caliciaceae,g:Buellia,s:Buellia frigida

AAGTCGTAACAAGATTTCCGTAGGTGAACCTGCGAAAGAATCATTATCGAGAGACGAAAT

CGCTTCGATTTCACTCTTCACTCGTGCCTACTTACCTTTGTTGCTTTGACGAGCCTTCGG

GCTTGATCGTTCGACGCGAGTGAGAGGCTTTCGCCTCTCGCCTCGCGAGCGCTCGTCAAA

GGCCCTGTTAACTCTGTTTTAGTGTCTTTCGAGCAATCATGTAATAGTTAAAACTTTCAA

CAACGAATCT

>ASV873 GS|75.6|MN615681|SH0916491.09FU;k:Fungi,p:Ascomycota,c:Lecanoromycetes,o:Caliciales,f:Caliciaceae,g:Buellia,s:Buellia insignis

AAGTCGTAACAAGGTTTCCGTAAGTGAACCTGCGAAAGAATCATTATCGAGAGACGAAGT

CTAACCGGTCCCACTCTTCACCCGTGTATATCTACCCTTTATTACTTTAACGAGCGGTCG

ATCTTTATCGCCCGACGTCGATCGGAAGACTCGCGCCTTTCACTCACCGAACGCCCGCCA

AATATTTCCAAAACCCGTTCATCTGTGTCCGAGCGAGTGAAGCAAATAATTAAAAACTTT

CAACAACGAA

>ASV874 GS|83.8|AF250779|SH0954634.09FU;k:Fungi,p:Ascomycota,c:Lecanoromycetes,o:Caliciales,f:Caliciaceae,g:Buellia,s:Buellia frigida

AAGTCGTAACAAGATTTTCATAGATGAATCTACGAAAAAATCATTATCGAAAGACGAAAT

CGCTTCGACTCCACTCTTCATTCGTGTCTACTTATCTTTGTTGCTTTGACGAGTCTTCGA

GCTTGATCGTTCGATGCGAGTGAAAGACTTTCGTCTCTCGTCTCGCGAGCGCTCGTCAAA

GATCTTGTTAACTTTGTTTTAGTGTCTTTCGAGCAATCATATAATAATTAAAACTTTCAA

CAACGAATCT

>ASV875 GSL|100.0|KC959216|SH0995195.09FU;k:Fungi,p:Ascomycota,c:Dothideomycetes,o:Mycosphaerellales,f:Mycosphaerellaceae,g:Ramularia,s:Ramularia hydrangeae-macrophyllae

AAGTCGTAACAAGGTCTCCGTAGGTGAACCTGCGGAGGGATCATTACTGAGTTAGGGAGC

AATCCCGAACCTCCAACCCTTTGTGAACACATCTTGTTGCTTCGGGGGCGACCCTGCCGT

TTCGACGGCATTCCCCCCGGAGGTCATCAAAACACTGCATTCTTACGTCGGAGTATAAAG

TTAATTTAATAAAACTTTCAACAACGGATCTCTTGGTTCTG

>ASV876 GS|98.0|AY667583|SH0954634.09FU;k:Fungi,p:Ascomycota,c:Lecanoromycetes,o:Caliciales,f:Caliciaceae,g:Buellia,s:Buellia frigida

AAGTCGTAACAAGGTTTCCGTAGGTGAACCTGCGGAAGGATCATTATCGAGAGACGGGGT

CGCTTCGGCCCCACTCTTCACTCGTGTCTACTTATCTTTGTTGCTTTGGCGGGCCTTCGG

GCTTGATCGTTCGACGCGGGTGGGAGGCTTTCGCCTCTCGCCTCGCGAGCGCTCGCCAAA

GGCCCTGTTAACTCTGTTTTAGTGTCTTCCGAGCAACCATGTAATAGTTAAAACTTTCAA

CAACGGATCT

>ASV877 GS|89.2|AF250779|SH0954634.09FU;k:Fungi,p:Ascomycota,c:Lecanoromycetes,o:Caliciales,f:Caliciaceae,g:Buellia,s:Buellia frigida

AAGTCGTAATAAGATTTCCGTAGATGAACTTGCGAAAGAATCATTATCGAGAGACGAGAT

CGCTTCGATCTCACTCTTCACTCGTATCTACTTATCTTTGTTGCTTTGACGAGCCTTCGA

GCTTGACCGTTCGACGCGAGTGAGAGACTTTCGCCTCTCGTCTCGCGAGCGCTCGTCAAA

GGCCCTATTAACTCTATTTTAGTGTCTTCCGAGCAATCATGTAATAGTTAAAACTTTCAA

CAACGAATCT

>ASV878 GS|85.8|AF250779|SH0954634.09FU;k:Fungi,p:Ascomycota,c:Lecanoromycetes,o:Caliciales,f:Caliciaceae,g:Buellia,s:Buellia frigida

AAGTCGTAACAAGATTTTCGTAGATGAATTTGCGAAAGAATCATTATCGAGAGACGAGAT

CGCTTCGATCTCACTCTTCATTCGTATCTACTTATCTTTGTTGCTTTGACGAGTCTTCGA

GCTTGATCGTTCGACGCGAGTAAGAGACTTTCGTCTCTCGTCTCGCGAGCGCTCGCCAAA

GACTTTGTTAACTTTGTTTTAGTGTCTTTCGAGCAATCATGTAATAGTTAAAACTTTCAA

CAACGAATCT

>ASV879 GS|98.1|AY843176|SH0964452.09FU;k:Fungi,p:Ascomycota

AAGTCGTAACAAGGTTTCCGTAGGTGAACCTGCGGAAGGATCATTACCGAGATAGGGTTT

CTTCGGAGCCCGACCTCCAACCCATTGTCTACCATACCTCTGTTGCCTCGGCGGACCGCC

GGCGCCTTTACGGGCGTCGCCGTCGGTCTTTACGGGCTTTCGAGCGCCCGCCGCAGGACT

TATAAACTCTTTTTTAAAGTAGTATTCTGAGTGGGAATTTAATAACTTCAAAACTTTCAA

CAACGGATCT

>ASV880 GS|82.4|AY667583|SH0954634.09FU;k:Fungi,p:Ascomycota,c:Lecanoromycetes,o:Caliciales,f:Caliciaceae,g:Buellia,s:Buellia frigida

AAGTCGTAACAAGATTTTCGTAGATGAATCTACGAAAGAATCATTATCGAGAGACGAGAT

CGCTTCGATCTCACTCTTCACTCGTATCTATATATCTTTGTTGCTTTGACGAGTCTTCGA

GCTTGATCGTTCGACGCGAGTGAAAGACTTTCGTCTCTCGTCTCGCGAGCGTTCGTCAAA

GACTCTATTAACTTTGTTTTAATGTCTTTCGAGCAATCATGTAATAATTAAAATTTTCAA

CAATGAATTT

>ASV881 SS|0.9900|UDB01604565|SH1277830.09FU;k:Fungi,p:Ascomycota,c:Candelariomycetes,o:Candelariales,f:Candelariaceae,g:Candelariella,s:Candelariella flava

AAGTCGTAACAAGGTTTCCGTAGGTGAACCTGCGGAAGGATCATTAAAGAGCAAGGGTCT

TCTAGGCCCGACCTCCAACCCTTTGTATATCTACCTCTGTTGCTTTGGCGGGCCCGTTGG

GGTGACCCACCGCCGGCTTTCAGCCGGTGAGTGCCCGTCAGAGTCCACGTAAACTCTGTC

TATCTATGCAGTCTGAGCACCATATAATAATTAAAACTTTCAACAACGGATCTCTTGGTT

CTG

>ASV882 SS|0.8000|UDB0243452|SH1241595.09FU;k:Fungi

AAGTCGTAACAAGGTTTCCGTAGGTGAACCTGCGGAAGGATCATTATATTAGCAAGTAAT

TGCGATAACTGTGCACTACCTATATTTGTCATTGCGAGTTATTTTAGTAACGGGCGTGAT

TGTGTCGTGGTTGATTTATTATCAATCACGATTCAATCGGTGACAGTCTTACCATTACAA

TTGTCTGACTAAAAAGAGAATAAAACTCTCAACAATGGATCTCTTGGCTCTG

>ASV883 GS|81.4|AF250779|SH0954634.09FU;k:Fungi,p:Ascomycota,c:Lecanoromycetes,o:Caliciales,f:Caliciaceae,g:Buellia,s:Buellia frigida

AAGTCGTAACAAGATTTTCATAAATGAATTTGCGAAAGAATCATTATCGAGAGACGAAAT

CGCTTCGATCTCATTCTTCACTCGTATCTACTTATCTTTGTTGCTTTGACGAGTCTTCGA

ACTTGATCGTTCGACGCGAGTGAGAGACTTTCGTCTCTCGTCTTGCGAACGCTCGTCAAA

GATTTTGTTAACTTTGTTTTAGTGTCTTTCGAGTAATCATGTAATAATTAAAACTTTCAA

CAATAAATCT

>ASV884 GS|75.1|UDB01571335|SH0954626.09FU;k:Fungi,p:Ascomycota,c:Lecanoromycetes,o:Caliciales,f:Caliciaceae,g:Buellia

AAGTCGTAACAAGATTTTCGTAAATAAATTTACGAAAAAATTATTATCGAGAGACGAAAT

CGCTTCGATCTCACTCTTCATTCGTATCTATTTATCTTTGTTGCTTTGACGAGTTTTCGA

GTTTGATCGTTCGATGCGAGTGAGAGACTTTCGTCTCTCGTTTCGCGAACGTTCGTCAAA

GACTTTGTTAACTTTATTTTAGTGTCTTTCGAGCAATCATATAATAATTAAAATTTTCAA

TAACGAATCT

>ASV885 SS|0.9900|AF250779|SH0954634.09FU;k:Fungi,p:Ascomycota,c:Lecanoromycetes,o:Caliciales,f:Caliciaceae,g:Buellia,s:Buellia frigida

AAGTCGTAACAAGGTTTCCGTAGATGAATCTACGAAAGAATCATTATCGAGAGACGAGGT

CGCTTCGACCCCACTCTTCACTCGTGTCTACTTACCTTTGTTGCTTTGACGAGCCTTCGA

GCTTGATCGTTCGACGCGAGTGGGAGGCTTTCGCCTCTCGCCTCGCGAGCGCTCGCCAAA

GGCCCTGTTAACTCTATTTTAGTGTCTTTCGAGCAACCATGTAATAGTTAAAACTTTCAA

CAACGGATCT

>ASV886 SS|0.8000|MN592663|SH1107827.09FU;k:Fungi,p:Ascomycota,c:Lecanoromycetes,o:Teloschistales,f:Teloschistaceae

AAGTCGTAACAAGGTTTCCGTAGGTGAACCTGCGGAAGGATCATTATCGAGAGGGGGGTT

CCATGCCCCGGGGCTCTGTCCCCATACCTTTTCACCCTGTGTGTATTTCTCCCCCGTTGC

TTTGGCGGGCCCCGGGTCTTCCCCCGGCGTTGGCCCCCTCGCGGGGTTCGCGAGCGCCCG

CCGAAGGCTCATCGAAACTCTGTTGGTCAGTGCAGTCTGAGCGTACGAATAATAAATCAA

AACTTTCAAC

>ASV887 GS|98.0|JX036119|SH0942719.09FU;k:Fungi,p:Ascomycota,c:Lecanoromycetes,o:Lecanorales,f:Lecanoraceae,g:Rhizoplaca

AAGTCGTAACAAGGTTTCCGTAGGTGAACCTGCGGAAGGATCATTATCGAGAGGGGTCCC

CGGACTCCGGGGGCTTCGGCCCCCTACTCTTCACCCTATGTCTACACACCTTTGTTGCTT

TGGCGGGCCTCGGGTTCGCCCCGTACCGGCCGTGGGCTTCCATACCCCGGCCGTCCGTGC

CCGTCAGAGGCCCATGAACCCTCGTTTATCAGTGTCGTCCGAGTAGTACTATAATCGTAA

AAACTTTCAA

>ASV888 GS|0.0|None;No hit

GTGTCAGCAGCCGCGGTAATACGTAGGGGGCGAGCGTTGTCCGAAGTTACTGGGCGTAAA

GAGCGCGTAGGCGGGTTCTTAAGTGAGGGGTGAAATTCCGAGGCTTAACCTCGGAACTGC

CTTTCATACTGGGAACCTTGAGTGTGGGAGAGGCGAGTGGAATGGTCGGTGTAGCGGTGA

AATGCGTAGATATCGATCGGAACACCCATGGCGAAGGCAGCTCGCTGGCCTATAACTGAC

GCTGAGGCGC

>ASV889 GS|0.0|None;No hit

GTGTCAGCAGCCGCGGTAATACGGAGGGGGCTAGCGTTGTTCGGAATTACTGGGCGTAAA

GCGTACGTAGGCGGTTTTGTAAGTTAGAGGTGAAAGCCCGGAGCTCAACTTCGGAATTGC

CTTTAAGACTGCATCACTTGAACGTCGGAGAGGTGAGTGGAATTCCGAGTGTAGAGGTGA

AATTCGTAGATATTCGGAAGAACACCAGTGGCGAAGGCGGCTCACTGGACGACTGTTGAC

GCTGAGGTAC

>ASV890 SS|0.9300|AY081162|SH1302838.09FU;k:Fungi,p:Ascomycota,c:Lecanoromycetes,o:Teloschistales,f:Teloschistaceae

AAGTCGTAACAAGGTTTCCGTAGGTGAACCTGCGGAAGGATCATTACTAAGAGAGGGATG

TACGCTTCCAGCCGAGTCCCGGGGGGCTGCGCCCCTCACCTCTTCAACCCTGTGTCTACC

AACCGCTGTTGCTTCGGCGAGCGTCGGGGAGCGATTCCCGGCCCTGGCTTCGGTCAGTCA

GCCCTCGTCAGAGGCCCATCCAAATTCTGTTTCAGTGACGTCCGAGTATACCAGCAAATA

AATTAAAACT

>ASV891 GS|77.8|MK778643|SH0916486.09FU;k:Fungi,p:Ascomycota,c:Lecanoromycetes,o:Caliciales,f:Physciaceae,g:Tetramelas

AAGTCGTAACAAGGTTTCCGTAGGTGAACCTGCGAAAGAATCATTATCGAGAGACGGAGT

TTAACCGGCCCCACTCTTCACCCGTGTATATCTACCCTTTATTGCTTTGACGGGCGATCG

ATCTTTACCGCCCGACGTCGGTCGAAAGACTCGCGCCTTCCACTCACCGAACGCCCGTCA

GAGATCCTCAAAACCCGTCCATCTGTGTTCGAGCGAGTGAAACAAATAATTAAAAACTTT

CAACAACGGA

>ASV892 GS|86.3|AF250779|SH0954634.09FU;k:Fungi,p:Ascomycota,c:Lecanoromycetes,o:Caliciales,f:Caliciaceae,g:Buellia,s:Buellia frigida

AAGTCGTAACAAGATTTTCGTAGATGAATTTGCGAAAGAATCATTATCGAGAGACGAGAT

CGCTTGAGTCTCACTCTTCACTCGTGTCTACTTATCTTTGTTGCTTTGACGAGTCTTCGA

GCTTGATCGTTCGACGCGAGTGAGAGACTTTCGTCTCTCGTCTCGCGAGCGCTCGTCAAA

GACTCTATTAACTCTGTTTTAGTGTCTTTCGAGCAATCATGTAATAGTTAAAACTTTCAA

TAACGAATCT

>ASV893 GS|77.2|MN615681|SH0916491.09FU;k:Fungi,p:Ascomycota,c:Lecanoromycetes,o:Caliciales,f:Caliciaceae,g:Buellia,s:Buellia insignis

AAGTCGTAACAAGGTTTCCGTAAATGAACCTGCGAAAGAATCATTATCGAGAGACGGAGT

CTAACCGGCCCCACTCTTCACCCGTGTATATCTACTCTTTATTACTTTGACGAGCGATCG

ATCTTTATCGCCCGACGTCGGTCGGAAGGCTCGCGCCTTCCACTTATCGAACGTCCGCCA

GAGATCTCCAAAAGCCGTCCATTTGTGTTCGAGCGAGTGAAGCAAATAATTAAAAACTTT

CAACAACGGA

>ASV894 GS|86.8|AF250779|SH0954634.09FU;k:Fungi,p:Ascomycota,c:Lecanoromycetes,o:Caliciales,f:Caliciaceae,g:Buellia,s:Buellia frigida

AAGTCGTAACAAGATTTTCGTAAATGAATCTACGAAAAAATCATTATCGAGAGACGAGGT

CGCTTCGACCCCACTCTTCACTCGTATCTACTTATTTTTGTTGCTTTGACGAGTCTTCGA

GCTTGATCGTTCGACGCGAGTGAGAGACTTTCGTCTCTCGTCTCGCGAGCGTTCGTCAAA

GATCTTGTTAACTCTATTTTAGTGTCTTTCAAGCAATCATGTAATAGTTAAAACTTTCAA

CAACGGATCT

>ASV895 SS|0.9300|JN885566|SH1084447.09FU;k:Fungi,p:Ascomycota,c:Dothideomycetes

AAGTCGTAACAAGGTCTCCGTAGGTGAACCTGCGGAGGGATCATTACCGAGCGAGGGCCT

CCGGGTCCGACCTCCCACCCCATGTTATCCGACCACTGTTGCCTCGGGGGCGACCCGGCC

TTCGGGCGTCGGGGCCCCCGGCGGACGCGTAACCCTGCATCTGTGCGTCCGAGTCAAACG

ATTGAATCAATCAAAACTTTCAACAACGGATCTCTTGGTTCTG

>ASV896 GS|98.7|MK782318|SH1188820.09FU;k:Fungi,p:Basidiomycota,c:Tremellomycetes,o:Filobasidiales,f:Filobasidiaceae,g:Naganishia,s:Naganishia friedmannii

AAGTCGTAACAAGGTTTCCGTAGGTGAACCTGCGGAAGGATCATTAATGAATACAGATGC

CTGTCGAGCTTGCTCACGGGCTTTCTATCATATCCATAACACCTGTGCACTTGTTGGATG

TTCTAGAGACTTAGAGTTAAACCTGCAGTCAATAGTCATCCACTTACACTAAACAATAAT

GTAACAAATGTAGTCTTATTATAACAAAATAAAACTTTCAACAACGGATCTCTTGGTTCT

G

>ASV897 SS|1.0000|KF274227|SH0911134.09FU;k:Fungi

AAGTCGTAACAAGGTTTCCGTAGGTGAACCTGCGGAAGGATCATTACCGAGTTAGGGTCC

TCTGGGCTCGATCTCCAACCCTGTGTCTAAATAACCACGTTGCTTTGGCGGGCCCGCCTG

TAATGGGCCGCCGGGGGTGCCTTCGGCCCCCTGGTCAGCGCCTGCCAGTAGCCATCTCAA

ACTCTTCTTAATCGTGACGTCTGAGTCAAAATTATAAATTAAACAAAACTTTCAACAACG

GATCTCTTGG

>ASV898 GS|84.4|AY667583|SH0954634.09FU;k:Fungi,p:Ascomycota,c:Lecanoromycetes,o:Caliciales,f:Caliciaceae,g:Buellia,s:Buellia frigida

AAGTCGTAACAAGATTTTCGTAGATGAATTTGCGAAAGAATCATTATCGAGAGACGAGAT

CGCTTCGACCTCACTCTTCATTCGTGTCTATTTATCTTTGTTGCTTTGACGAGTCTTCGA

ACTTGATCGTTCGACGCGAGTGAGAGGCTTTCGCTTGTCGCCTTGCGAGCGCTCGTCAAA

AATTTTGTTAACTTTGTTTTAATGTCTTTCGAGCAATCATGTAATAGTTAAAACTTTCAA

CAACGAATCT

>ASV899 GS|71.5|MZ229888|SH0913119.09FU;k:Fungi,p:Ascomycota,c:Lecanoromycetes,o:Caliciales,f:Caliciaceae,g:Dimelaena

AAGTCGTAACAAGATTTCCGTAAATGAACCTGCGAAAGAATTATTATCGAAAGACGAAGT

TTAATCGATCTCATTCTTCACTCGTGTATATTTATTCTTTATTACTTTGACGGGCGGTTG

ATCTTTACCGCCCGACGTCGATTGAAAGACTCGCGTCTTTCATTCATCGAACGCCCGCCA

GAGACCTTCAAAATCCGTTCATTTATGTTCGAACGAGTGAAACAAATAATTAAAAACTTT

CAACAACGAA

>ASV900 GS|78.4|AF250779|SH0954634.09FU;k:Fungi,p:Ascomycota,c:Lecanoromycetes,o:Caliciales,f:Caliciaceae,g:Buellia,s:Buellia frigida

AAATCGTAACAAAATTTTCATAAATGAATTTGCGAAAGAATCATTATCGAGAGACGAAAT

CGTTTCGATTTTACTCTTCACTCGTATCTATTTATCTTTGTTGCTTTGACGAGTCTTCGA

ACTTGATCATTCGATGCGAATAAGAGACTTTCGTCTCTCGTTTCGCGAACGTTCGTCAAA

GACTTTATTAACTTTGTTTTAGTGTTTTTCGAGCAATCATGTAATAGTTAAAACTTTCAA

TAACGAATCT

>ASV901 GS|85.8|AF250779|SH0954634.09FU;k:Fungi,p:Ascomycota,c:Lecanoromycetes,o:Caliciales,f:Caliciaceae,g:Buellia,s:Buellia frigida

AAGTCGTAACAAGATTTTCGTAGATGAATCTACGAAAGAATCATTATCGAGAGACGAGAT

CGCTTCGACCTCACTCTTCACTCGTGTCTACTTATCTTTGTTGCTTTGACGAGTCTTCGA

GCTTGATCGTTCGACGCAAATGAAAGACTTTCGTCTCTCGTCTCGCGAGCGCTCGTCAAA

GACTCTGTTAACTCTGTTTTAGTGTCTTTCGAGCAATCATGTAATAATTAAAACTTTCAA

CAATGAATCT

>ASV902 GS|82.8|AY667583|SH0954634.09FU;k:Fungi,p:Ascomycota,c:Lecanoromycetes,o:Caliciales,f:Caliciaceae,g:Buellia,s:Buellia frigida

AAGTCGTAACAAGATTTTCGTAAATGAACTTGCGAAAGAATCATTATCGAAAGACGAAAT

CGCTTCGATTTCACTCTTCACTCGTATCTACTTATTTTTGTTGCTTTGACGAATCTTCGA

GCTTGATCGTTCGACGCGAGTGAGAGACTTTCGTCTCTCGTCTTGCGAGCGTTCGTCAAA

GACTCTATTAACTCTATTTTAGTGTCTTTCGAGCAATCATGTAATAATTAAAACTTTCAA

CAACGAATCT

>ASV903 GS|87.3|AF250779|SH0954634.09FU;k:Fungi,p:Ascomycota,c:Lecanoromycetes,o:Caliciales,f:Caliciaceae,g:Buellia,s:Buellia frigida

AAGTCGTAACAAGATTTTCGTAGATGAATTTGCGAAAGAATCATTATCGAGAGACGAGGT

CGCTTCGATCTCACTCTTCACTCGTGTCTACTTATCTTTGTTGCTTTGACGAGTCTTCGA

GCTTGATCGTTCGACGCGAGTGAGAGACTTTCGTCTCTCGTCTCGCGAGCGCTCGTCAAA

GACTCTATTAACTCTATTTTAGTGTCTTTCGAGCAATCATGTAATAGTTAAAACTTTCAA

CAACGAATCT

>ASV904 GS|99.5|UDB01720327|SH1085256.09FU;k:Rhizaria,p:Cercozoa

AAGTCGTAACAAGGTCTTCGTAGGTGAACCTGCGAAGGGATCATTAACACGTTCCACACC

TCAATTATCAACCCATCTGTGAACCTTTGTTACCGCCGAATGGTCAACTACAGGTGTATG

GCGCATACGATCGACTCAATGATGTTGCAGAATTTATTCTGCATGGTCTAGTAGTATGCC

TCTGTGTCGTTCACACGACAGCTATCCTTGTATGTTGGTCCGGCCACTATATATGCGCTT

CGACGGCCGT

>ASV905 SS|0.9900|AF250779|SH0954634.09FU;k:Fungi,p:Ascomycota,c:Lecanoromycetes,o:Caliciales,f:Caliciaceae,g:Buellia,s:Buellia frigida

AAGTCGTAACAAGATTTTCGTAGGTGAACTTGCGAAAGAATCATTATCGAGAGACGAGGT

CGCTTCGACCCCACTCTTCACTCGTGTCTACTTACCTTTGTTGCTTTGGCGAGCCTTCGA

ACTTGATCGTTCGACGCGGGTGGGAGGCTTTCGTCTCTCGTCTCGCGAGCGCCCGCCAAA

GGCCCTGTTAACTCTGTTTTAGTGTCTTCCGAGCAACCATGTAATAGTTAAAACTTTCAA

CAACGGATCT

>ASV906 GS|97.9|UDB01571642|SH1041881.09FU;k:Fungi,p:Basidiomycota,c:Tremellomycetes,o:Tremellales,f:Bulleraceae

AAGTCGTAACAAGGTTTCCGTAGGTGAACCTGCGGAAGGATCATTAATGTAAACCCCTTG

TGGGGAAATACAAATCCACATACCTCTGTGAACCGTTGACCTCCGGGTCGTCTTTACAAA

CATCAGTGTAACGAACGTATACAAACATAAACAAAACAAAACTTTCAACAACAGATCTCT

TGGCTCTC

>ASV907 GS|78.3|MZ229880|SH0913105.09FU;k:Fungi,p:Ascomycota,c:Lecanoromycetes,o:Caliciales,f:Caliciaceae,g:Dimelaena,s:Dimelaena oreina

AAGTCGTAACAAGGTTTTCGTAAGTGAATCTGCGGAAGGATCATTATCGAGAGACGGAGT

CTAACCGGCCCCACTCTTCACCCGTGTATATTTACCCTTTATTGCTTTGGCGGGCGGTCG

GTCTTTACCGCCCGACGTCGATCGGAAGACTCGCGCCTTCCACTCACCGAACGCCCGCCA

AAGACTCCCAAAAACCGTTCATCTGTGTTCGAGCGAGTGAAGCAAATAATTAAAAACTTT

CAACAACGGA

>ASV908 GS|85.8|AF250779|SH0954634.09FU;k:Fungi,p:Ascomycota,c:Lecanoromycetes,o:Caliciales,f:Caliciaceae,g:Buellia,s:Buellia frigida

AAGTCGTAACAAGATTTTCGTAGATGAATTTGCGAAAGAATCATTATCGAGAGATGAGAT

CGCTTCGATCTCACTCTTCACTCGTGTCTACTTATCTTTGTTGCTTTGACGAGTCTTCGA

GCTTGATCGTTCGACGCGAGTGAGAGACTTTCGTCTCTCGTCTCGCGAGCGCTCGTCAAA

GACTCTATTAACTTTGTTTTAGTGTCTTTCGAGCAATCATGTAATAGTTAAAATTTTCAA

CAACGAATCT

>ASV909 GS|100.0|AF279772|SH1300522.09FU;k:Fungi,p:Ascomycota,c:Lecanoromycetes,o:Teloschistales,f:Teloschistaceae,g:Xanthoria,s:Xanthoria elegans

CTTTGTCCTTTCTCTTCCTTCCCCGTCTTCACAAGGTTTCCGTAGGTGAACCTGCGGAAG

GATCATTACTAAGAGAGGGATGTACGCTTCCAGCCGAGTCCCGGGGGGCTGCGCCCCTCA

CCTCTTCAACCCTGTGTCTACCAACCGCTGTTGCTTCGGCGAGCGTCGGGGCGTCCGCGC

CCCGGCCCCGGCTTCGGTCGGTGAGCTCTCGCAGAGGCCTATCTTTATTCTGTTTTGCAG

TGACGTCCGA

>ASV910 GS|99.5|UDB03378640|SH0910872.09FU;k:Fungi,p:Ascomycota,c:Eurotiomycetes,o:Chaetothyriales,f:Herpotrichiellaceae

AAGTCGTAACAAGGTTTCCGTAGGTGAACCTGCGGAAGGATCATTATCGAGTTAGGGTCT

TTACTGGCCCGATCTCCAACCCTGTGTCTATAATACCATGTTGCTTTGGCGGGCCCGCCT

TTAACGGGCCGCCGAGGGTCTTCGGACCCTTGGTCAGTGCCCGCCAGTAGCCAAATTAAA

TTCTTCTTAACTGTGTCGTCTGAGTAAATATTTAAAATAAACACAACTTTCAACAACGGA

TCTCTTGGTT

>ASV911 SS|1.0000|DQ525485|SH0954363.09FU;k:Fungi,p:Ascomycota,c:Lecanoromycetes,o:Acarosporales,f:Acarosporaceae,g:Pleopsidium,s:Pleopsidium chlorophanum

CTTGTTCCTTTCTCTTCCTTCCCCTTCTTCCCCCTTTTTCCTTCCGGGTGTAGCACCTGC

CGAAGCCTCCCGCAGCGACTCTAAAGAAACCGCGCAGTCTGCCCCCCTGCCGGCAACACT

GTCCCTGTGCTGGGAGCCAGTCACGCCTGGTTCAGGCGCCGCTACCAGCAACCTGGGCAA

CAACGCCCAGGCTCACAGATCAGATGATTGTGGCCACACCGTGGTTAAGATATGCCCGGT

CCCCGCTGTG

>ASV912 GS|99.0|KF823589|SH1091255.09FU;k:Fungi,p:Basidiomycota,c:Tremellomycetes,o:Tremellales,f:Sirobasidiaceae

AAGTCGTAACAAGGTTTCCGTAGGTGAACCTGCGGAAGGATCATTAGTGATTTGGCCTCC

GGGCCTTCAATCCCATCCTCATACCTCTGTGAACCAGTTGGGCCCTCGGGCCTACCCTTT

CAAACACTGTGTAACCAACGTGATGTATATTATAAACCTAGTAAAACTTTCAACAACGGA

TCTCTTGGCTCTC

>ASV914 SS|0.9800|MF138060|SH0954356.09FU;k:Fungi,p:Ascomycota,c:Lecanoromycetes,o:Acarosporales,f:Acarosporaceae,g:Acarospora

AAGTCGTAACAAGGTCTCCGTAGGTGAACCTGCGGAAGGATCATTACAGAGTTAGGGTCT

TTCCAGGCCCGACCTCCAACCCTATGTGTACCTACCTTTGTTGCTTTGGCGGGCCCGTCG

GGTGACCCACCGGTGGCCTCTGGCTCCCGAGTGCCCGTCAGAGACCCATCAAAACCCGTT

AATTGTGTCGTCTGAGTACCACTTTAATAATTAAAACTTTCAACAACGGATCTCTTGGTT

CTG

>ASV915 GS|84.8|AF250779|SH0954634.09FU;k:Fungi,p:Ascomycota,c:Lecanoromycetes,o:Caliciales,f:Caliciaceae,g:Buellia,s:Buellia frigida

AAGTCGTAACAAGATTTTCGTAGATGAATTTACGAAAGAATCATTATCGAGAGACGAGAT

CGCTTCGATCTCACTCTTCACTCGTATCTATTTATCTTTGTTGCTTTGACGAGTCTTCGA

GCATGATCGTTCGACGCGAGTGAGAGACTTTCGTCTCTCGTCTCGCGAGCGCTCGTCAAA

GGCTTTGTTAACTTTGTTTTAGTGTCTTTCGAACAATTATGTAATAGTTAAAACTTTCAA

CAACGAATCT

>ASV916 GS|100.0|LT746245|SH1079201.09FU;k:Fungi,p:Ascomycota,c:Sordariomycetes,o:Hypocreales,f:Nectriaceae,g:Fusarium,s:Fusarium citricola

AAGTCGTAACAAGGTCTCCGTTGGTGAACCAGCGGAGGGATCATTACCGAGTTTACAACT

CCCAAACCCCTGTGAACATACCTTTAATGTTGCCTCGGCGGATCAGCCCGCGCCCCGTAA

AACGGGACGGCCCGCCAGAGGACCCAAACTCTAATGTTTCTTATTGTAACTTCTGAGTAA

AACAAACAAATAAATCAAAACTTTCAACAACGGATCTCTTGGTTCTG

>ASV917 GS|75.0|MN103133|SH0913114.09FU;k:Fungi,p:Ascomycota,c:Lecanoromycetes,o:Caliciales,f:Caliciaceae,g:Dimelaena,s:Dimelaena oreina

AAGTCGTAATAAGATTTTCGTAAGTGAATCTGCGAAAGGATCATTATCGAGAGACGAAAT

CTAACCGGCCCCACTCTTCACTCGTGTATATTTATCCTTTGTTGTTTTGACGGGCGGTCG

GTCTTTATCGCTCGACGTCGGTCGAAAAACTCGCGCCTTTCACTCATCGAACGCCCGCCA

GAGATTTTCAAAACTCGTTTATTTGTGTTCGAGCGAGTGAAATAAATAATTAAAAACTTT

CAACAACGGA

>ASV918 GS|84.8|AF250779|SH0954634.09FU;k:Fungi,p:Ascomycota,c:Lecanoromycetes,o:Caliciales,f:Caliciaceae,g:Buellia,s:Buellia frigida

AAGTCGTAACAAGATTTTCGTAGATGAATTTACGAAAAAATCATTATCGAGAGACGAAAT

CGCTTCGATTTCACTCTTCACTCGTGTCTACTTATCTTTGTTGCTTTGACGAGTCTTCGA

ACTTGATCGTTCGATGCGAGTGAGAGACTTTCGTCTCTCGTCTCGCGAGCGCTCGTCAAA

GACTTTGTTAACTCTATTTTAGTGTTTTTCGAGCAATCATGTAATAGTTAAAACTTTCAA

CAACGGATCT

>ASV919 GS|88.2|AF250779|SH0954634.09FU;k:Fungi,p:Ascomycota,c:Lecanoromycetes,o:Caliciales,f:Caliciaceae,g:Buellia,s:Buellia frigida

AAGTCGTAACAAGATTTTCGTAGATGAATCTGCGAAAGAATCATTATCGAGAGACGAGAT

CGCTTCGATCTCACTCTTCACTCGTGTCTACTTATCTTTGTTGCTTTGACGAGTCTTCGA

GCTTGATCGTTCGACGCGGGTGAGAGGCTTTCGCCTCTCGTCTCGCGAGCGCTCGTCAAA

GATCGTGTTAACTTTGTTTTAGTGTCTTTCGAGCAATCATGTAATAGTTAAAACTTTCAA

CAACGAATCT

>ASV920 GS|86.8|AF250779|SH0954634.09FU;k:Fungi,p:Ascomycota,c:Lecanoromycetes,o:Caliciales,f:Caliciaceae,g:Buellia,s:Buellia frigida

AAGTCGTAACAAGATTTTCGTAGATGAATCTACGAAAAAATCATTATCGAGAGACGAGGT

CGCTTCGATCTCACTCTTCATTCGTATCTACTTATCTTTGTTGCTTTGGCGAATCTTCGA

GCTTGATCGTTCGACGCGAGTGAGAGGCTTTCGCCTCTCGTCTCGCGAGCGCTCGTCAAA

GATCTTGTTAACTTTGTTTTAGTGTCTTTCGAGCAATCATGTAATAGTTAAAACTTTCAA

CAATGAATCT

>ASV921 SS|0.9900|MK970689|SH0954625.09FU;k:Fungi,p:Ascomycota,c:Lecanoromycetes,o:Caliciales,f:Caliciaceae,g:Buellia

AAGTCGTAACAAGGTTTCCGTAGGTGAACCTGCGGAAGGATCATTACTGAGACGGGGTCT

CATCGACCCCACTCTTCACCCATGTCTACTATACCCTGTTGCTTTGGCGGGCCTTCGGGT

CTTACCACCCGGGGTTGGTCGGGGGCCTCATGCCCCTCGCTCAGCGAGTGCCCGCCAATG

ACCCTGTCAACTCTATTCTTAGTGTCCTCTGAGCAACCACACAATAGTCAAAACTTTCAA

CAACGGATCT

>ASV922 GSL|100.0|FN394680|SH0881518.09FU;k:Fungi,p:Ascomycota,c:Sordariomycetes,o:Sordariales,f:Chaetomiaceae,g:Dichotomopilus

AAGTCGTAACAAGGTCTCCGTTGGTGAACCAGCGGAGGGATCATTACAGAGTTGCAAAAC

TCCCAAACCATCGTGAACGTTACCTATATCGTTGCTTCGGCGGGTGGCTCGGCCTCGGCC

CTGCCCTAGGCCCCTCTCGGGGGCCCGCCGGAGGTCTACCAAACTCTTGAATTTACATGG

CCTCTCTGAGTCTTCTGTACTGAATAAGTCAAAACTTTCAACAACGGATCTCTTGGTTCT

G

>ASV923 GS|99.5|UDB01983871|SH1165401.09FU;k:Fungi,p:Ascomycota,c:Eurotiomycetes,o:Chaetothyriales,f:Herpotrichiellaceae,g:Exophiala

AAGTCGTAACAAGGTTTCCGTAGGTGAACCTGCGGAAGGATCATTAACGAGTTAGGGTCT

TCCAAGGCCCGACCTCCCAACCCTATGTTTATTGTACCATGTGTTGCTTCGGCGGGACCG

TCTTCACGACCGCCGGAGGGCCGTAAAACGTCCTCTGGCCCGCGCCCGCCGATGGCCCCC

CTTCAAAAACTCTTGAATAATCATGCCTATATTGTCTGAGTAATACACAAAAATTAAAAG

CAAAACTTTC

>ASV924 SS|1.0000|KC965541|SH0962007.09FU;k:Fungi

AAGTCGTAACAAGGTTTCCGTAGGGTACGTTCACCCCTATAAACCCCCCCTTGCTACGTC

GAAAACGATTTGCTAACCCTCATTCCAGTGAACCTGCGGAAGGATCATTAAAGAGAAATC

GGGGTGTCGCCGCGAGGCTTTACCCCATCTCCCTAAACCCCCTGTCTACCGACCTCACAC

AAAACCACAGTACAAATATCAAGTCGTCCCTAGCGGGGCGCAACACCATAAATGTACAAA

AAACAAAACT

>ASV925 GS|84.0|AY667583|SH0954634.09FU;k:Fungi,p:Ascomycota,c:Lecanoromycetes,o:Caliciales,f:Caliciaceae,g:Buellia,s:Buellia frigida

AAGTCGTAACAAGATTTTCGTAGATGAACTTGCGAAAGAATCATTATCGAGAGACGAGAT

CGCTTCGATTTCATTCTTCACTCGTATCTACTTATCTTTGTTGTTTTAACGAGTCTTCGA

GCTTGATCGTTCGACGCGAGTGAGAGACTTTCGTCTCTCGTCTCGCGAGCGCTCGTCAAA

GATCTTGTTAACTTTGTTTTAATGTCTTTCGAGCAATCATGTAATAGTTAAAACTTTCAA

CAATGAATCT

>ASV926 GS|85.3|AF250779|SH0954634.09FU;k:Fungi,p:Ascomycota,c:Lecanoromycetes,o:Caliciales,f:Caliciaceae,g:Buellia,s:Buellia frigida

AAGTCGTAACAAGATTTTCGTAGATGAATTTGCGAAAGAATCATTATCGAGAGACGAAGT

CGCTTCGATCTCACTCTTCATTCGTGTCTACTTATCTTTGTTGCTTTGACGAGTCTTCGA

GCTTGATCGTTCGACGCGAGTGAGAGACTTTCGTCTCTCGTCTTGCGAGCGCTCGTCAAA

GACTCTATTAACTTTGTTTTAGTGTCTTTCGAACAATCATGTAATAGTTAAAACTTTCAA

CAACGAATCT

>ASV927 SS|1.0000|AF250779|SH0954634.09FU;k:Fungi,p:Ascomycota,c:Lecanoromycetes,o:Caliciales,f:Caliciaceae,g:Buellia,s:Buellia frigida

CTTTTTCCTTTCTCTTCCTTCCCCTTCTTCCCCCTTTTTCCGTATTTTCCCCTGCGGACT

TCTCCTTACCGATCTCCTTGGTCGCTTCTTCCCCACTCTTCCCCCGTGTCTACTTACCTT

TGTTGCTTTGGCGGGCCTTCGGGCTTGACCGTTCGCCGCGGGTGGGAGGCTTTCGCCTCT

CGCCTCTCTCTCGCCCGCCAAAGGCCCTGTTAACTCTGTTTTAGTGTCTTCCGAGCAACC

ATGTAATAGT

>ASV928 GS|87.6|AY667583|SH0954634.09FU;k:Fungi,p:Ascomycota,c:Lecanoromycetes,o:Caliciales,f:Caliciaceae,g:Buellia,s:Buellia frigida

AAGTCGTAACAAGATTTTCGTAGATGAATCTGCGAAAGGATCATTATCGAGAGACGAGGT

CGCTTCGATCTCACTCTTCACTCGTGTCTACTTATCTTTGTTGCTTTGACGAGTCTTCGA

GCTTGATCGTTCGACGCGAGTGAGAGACTTTCGTCTCTCGCTTCGCGAGCGCTCGTCAAA

GGCTTTGTTAACTCTATTTTAGTGTCTTTCGAGCAATCATGTAATTGTTAAAACTTTCAA

CAACGAATCT

>ASV929 GS|87.3|AF250779|SH0954634.09FU;k:Fungi,p:Ascomycota,c:Lecanoromycetes,o:Caliciales,f:Caliciaceae,g:Buellia,s:Buellia frigida

AAGTCGTAACAAGATTTTCGTAGATGAATTTACGAAAGAATCATTATCGAGAGACGAGAT

CGCTTCGATCTCACTCTTCACTCGTGTCTACTTATCTTTGTTGCTTTAACGAGCCTTCGA

GCTTGATCGTTCGACGCGAGTGAGAGGCTTTCGTCTGTCGTCTCGCGAGCGCTCGTCAAA

GACCCTATTAACTCTATTTTAGTGTCTTTCGAGCAATCATGTAATAGTTAAAACTTTCAA

CAACGAATCT

>ASV930 SS|1.0000|AY667583|SH0954634.09FU;k:Fungi,p:Ascomycota,c:Lecanoromycetes,o:Caliciales,f:Caliciaceae,g:Buellia,s:Buellia frigida

AAGTCGTAACAAGGTTTCCGTAGATGAACCTGCGGAAGGATCATTACCGAGAGACGAGAT

CGCTTCGACCCCACTCTTCACCCGTGTCTACTTACCTTTGTTGCTTTGACGAGCCTTCGG

GCTTGACCGTTCGACGCGGATGAGAGGCTTTCGCCTGTCGCCTCGCGAGCGCCCGCCAAA

GGCCCTGTTAACTCTGTTTTAGTGTCTTCCGAGCAACCATGTAATAGTTAAAACTTTCAA

CAACGGATCT

>ASV931 GS|80.8|AY667583|SH0954634.09FU;k:Fungi,p:Ascomycota,c:Lecanoromycetes,o:Caliciales,f:Caliciaceae,g:Buellia,s:Buellia frigida

AAGTCGTAACAAGATTTTCGTAAATGAATTTGCGAAAGAATCATTATCGAGAAACGAAAT

CGCTTCGATCTCACTCTTCACTCGTATTTACTTATCTTTGTTGCTTTGACGAGTCTTCGA

GCTTGATCGTTCGACGCGAATGAGAGACTTTCGTCTCTCGTTTCGCGAGCGTTCGTCAAA

GATTTTGTTAACTCTATTTTAGTATCTTTCAAGTAATTATGTAATAATTAAAACTTTCAA

CAACGAATCT

>ASV932 GS|83.8|AF250779|SH0954634.09FU;k:Fungi,p:Ascomycota,c:Lecanoromycetes,o:Caliciales,f:Caliciaceae,g:Buellia,s:Buellia frigida

AAGTCGTAATAAGATTTTCGTAGATGAATTTGCGAAAGAATCATTATCGAGAGACGAAAT

CGCTTCGATCTCACTCTTCACTCGTATCTACTTATCTTTGTTGCTTTGACGAGTCTTCGA

GCTTGATCGTTCGACGCGAGTAAGAGACTTTCGTCTCTCGTCTCGCGAGCGCTCGTCAAA

GATTTTGTTAACTTTGTTTTAGTGTCTTTCGAGCAATCATGTAATAATTAAAACTTTCAA

CAACGAATTT

>ASV933 GS|0.0|None;No hit

AAGTCGTAACAAGGTTGAACCAGCGTGTGAATCAATATCAAGTATCGCATGATAAGATAT

GATATGATCCCTCTGAAGGCCTTGAACAGCATCACACACACAATGATGATGCTAGTCTGG

TATGAGCCATGCAACAGCAGACTCAACAGGCAACACTCTTCAATTGCGAGGACCCCCTGA

CTAGTGTGTTGGATACCGCTCTGCCTTTTGCAGCTGGAGCAGTCAAGAAGAGAGATCTTG

ATGATGGTAA

>ASV934 GS|99.2|JX171190|SH0981986.09FU;k:Fungi,p:Ascomycota,c:Lecanoromycetes,o:Lecanorales,f:Psilolechiaceae,g:Psilolechia,s:Psilolechia leprosa

AAGTCGTAACAAGGTTTCCGTAGGTGAACCTGCGGAAGGATCATTAATGAGCGAGGGGCC

CCGTGCTCCCGGGGGCTTCGGCCCCCACTCCTCACCCTTTGTATACATACCTTTGTTGCT

TTGGCGGGCCTTGGGGACCTCCCCATGCCGGCTCCGGGCTTCGGCCCCGCCGGCGAGCCG

CCCGCCCGAGGCCTATCAAATTCCGTTTTATCAGTGTTGTCCGAGTCAGATATAACAATC

AAAACTTTCA

>ASV935 GS|0.0|None;No hit

GTGTCAGCAGCCGCGGTAATACGAAGGGGGCTAGCGTTGCTCGGAATGACTGGGCGTAAA

GGGCGCGTAGGCGGCTTGGTGAGTTAGACGTGAAATTCCTGGGCTCAACCTGGGGGCTGC

GTTTGATACAGCTAGGCTAGAGTGGGGAAGAGGGTTGTGGAATTCCCAGTGTAGAGGTGA

AATTCGTAGATATTGGGAAGAACACCGGTGGCGAAGGCGGCAACCTGGTCCTTGACTGAC

GCTGAGGCGC

>ASV936 GS|99.0|AF278757|SH1300510.09FU;k:Fungi,p:Ascomycota,c:Lecanoromycetes,o:Teloschistales,f:Teloschistaceae,g:Xanthoria,s:Xanthoria elegans

CTTGTTCCTTTCTCTTCCTTCCCCTTCTTCCCCCTTTTTCCGTAGGTGAACCTGCGGAAG

GATCATTACCGCGAGGGACATGCGCCTCGCGGCCCTGTCCCGGGGGGTTCCGCCCCCATC

TCTTCAACCCTTGCCTATCTACCTCTGTTGCTTCGGCGAGCGCTCGGGTGCCATTGCGCC

CGGGCCCCGGCTTCGGTCGGTGCGCTCTCGCCGGAGGCCATATTGAACCTGTCTGTAGTG

ATGTCTGAGT

>ASV937 GS|100.0|EF179806|SH1075344.09FU;k:Fungi,p:Ascomycota,c:Lecanoromycetes,o:Lecanorales,f:Parmeliaceae,g:Usnea,s:Usnea subantarctica

AAGTCGTAACAAGGTTTCCGTAGGTGAACCTGCGGAAGGATCATTACCGAGAGCGGGGCT

TCGCGCTCCCGGGGGTTTCGGCCTCCACCTCTTCACCCATTGTGTACCTACCTTTGTTGC

TTTGGCGGGCCATGGGGTTCGCCTCACGCCGGCCTCCGGGCTGGTGAGCGCCCGTCGGAG

GCCCTTCAAATTCTGTTTATATCGACGTCCGAGTTAAACACAAACAGTAAAAACTTTCAA

CAACGGATCT

>ASV938 GS|0.0|None;No hit

AAGTCGTAACAAGGTTCTCCGGACATAGGTCAGATGAAGAGTTTCCCACTTCATCATCCA

ACGGGAGCCTTGTAAGTAATGGTCTGCTCTCTGCAGGCGACACTCGTCTGTTGCTGGAAA

TAACCCCTTTTAGGGAGTCAATCAGCCGCTATTTGCGAAACCGCAGGTAGTCCACAGATC

AAACACGAGTGGCCCTTTCCCCTTCTGCCAGCGCCGTTTCGACCTTGAAGTAGCTCTCGA

TCGGAAGCCG

>ASV939 GS|79.5|MN615681|SH0916491.09FU;k:Fungi,p:Ascomycota,c:Lecanoromycetes,o:Caliciales,f:Caliciaceae,g:Buellia,s:Buellia insignis

AAGTCGTAACAAGGTTTTCGTAGGTGAATCTGCGGAAGAATCATTATCGAGAGACGGAGT

TTAACCGGCTCCACTTTTCACCCGTGTATATTTATTCTTTGTTGCTTTGGCGGGCGGTCG

GTCTTTATCGCCCGACGTCGGTCGGAAGGCTCGCGCCTTTCACTCACCGAACGCCCGCCA

GAGATTCCCAAAACCCGTTTATCTATGTCCGAGCGAGTGAAGCAAATAGTTAAAAACTTT

CAACAACGGA

>ASV940 SS|0.8300|AY667583|SH0954634.09FU;k:Fungi,p:Ascomycota,c:Lecanoromycetes

AAGTCGTAACAAGGTTTCCGTAGGTGAACCTGCGGAAGGATCATTATCGAGAGACGGGGT

CTTACCGGCCCCACTCTTCACCCATGTATACTTACCCTGTTGCTTTGACGGGCCTTCGGG

CCTTATCGCTCGGCGCTGGTCGAGAGATTTCCCTCTCTCGCTCGGCGAGCGCCCGTCAGA

GGCCTTGTAAACTCCAGTTTTTGTCTCCTCCGAGCCAGCATATAATAGTTAAAACTTTCA

ACAACGGATC

>ASV941 GSL|100.0|MG020726|SH0885168.09FU;k:Fungi,p:Ascomycota,c:Dothideomycetes,o:Pleosporales,f:Pleosporaceae,g:Stemphylium

AAGTCGTAACAAGGTCTCCGTAGGTGAACCTGCGGAGGGATCATTACACAATATGAAAGC

GGGTTGGGACCTCATCTCGGTGAGGGCTCCAGCTTGTCTGAATTATTCACCCATGTCTTT

TGCGCACTTCTTGTTTCCTGGGCGGGTTCGCCCGCCACCAGGACCCAACCATAAACCTTT

TTTTGTAATTGCAATCAGCGTCAGTACACAATGTAATTATTACAACTTTCAACAACGGAT

CTCTTGGTTC

>ASV942 GS|0.0|None;No hit

AAGTCGTAACAAGGTCTCCGTAGGTGAACCTGCGGAGGGATCATTAGCAAATGCCAAATG

GATGGCGAACGACGTGCGGGCTGCGGAACGTAAAACACGCCTGCCGCTCTCCGTCGCCCT

GCCTCCATCTCTCATTTTCATCCACACAACCCCTGTGCACTGTCGGATTGGTAGATGAAT

CCCGTCTGTAGCTATCTAGTCTGGTATTATGTACCTTGCTTTGCACACAGTTCCGGTCCA

CATCCGCCTC

>ASV944 GS|99.4|UDB05021827|SH1223442.09FU;k:Fungi,p:Basidiomycota,c:Cystobasidiomycetes,o:Cystobasidiales,f:Cystobasidiaceae,g:Cystobasidium

AAGTCGTAACAAGGTTTCCGTAGGTGAACCTGCGGAAGGATCATTAATGAATTTTAGGAC

TCTCTCTTTAGAGGTCCGACCCCTTCATTTCCTTACACTGTGCACACACTTCTTTTCACA

CATTTTAACACTATAGTATAAGAATGTAACAGTCTCTTTATTGAGCATAAATAAAAATAA

AACTTCCAGCAACGGATCTCTTGGCTCTC

>ASV945 GS|87.3|AF250779|SH0954634.09FU;k:Fungi,p:Ascomycota,c:Lecanoromycetes,o:Caliciales,f:Caliciaceae,g:Buellia,s:Buellia frigida

AAGTCGTAACAAGATTTTCGTAAATGAATTTGCGAAAGAATCATTATCGAGAGACGAAAT

CGCTTCGACCTCACTCTTCACTCGTGTCTACTTATCTTTGTTGCTTTGGCGAGTCTTCGA

ACTTGATCGTTCGACGCGAGTGAGAGACTTTCGTCTCTCGTTTTGCGAGCGCTCGCCAAA

GGCCTTGTTAACTTTGTTTTAGTGTCTTTCGAGCAATCATGTAATAGTTAAAACTTTCAA

CAACGAATCT

>ASV946 SS|1.0000|AF250779|SH0954634.09FU;k:Fungi,p:Ascomycota,c:Lecanoromycetes,o:Caliciales,f:Caliciaceae,g:Buellia,s:Buellia frigida

CTTTTTCCTTTCTCTTCCTTCCCCTTCTTCCCCCTTTTTCCGTATTTTCCCCTGCGGACT

TCTCCTTACCGATCTCCTTGGTCGCTTCTTCCCCACACTCTTCCCCCGTGTCTACTTACC

TTTGTTGCTTTGGCGGGCCTTCGGGCTTGCCCGTTCGACGCGGGTGGGAGGCTTTCGCCT

CTCGCCTCGCGAGCGCCCGCCAAAGGCCCTGTTAACTCTGTTTTAGTGTCTTCCGAGCAA

CCATGTAATA

>ASV947 SS|1.0000|AF250779|SH0954634.09FU;k:Fungi,p:Ascomycota,c:Lecanoromycetes,o:Caliciales,f:Caliciaceae,g:Buellia,s:Buellia frigida

CTTTTTCCTTTCTCTTCCTTCCCCTTCTTCCCCCTTTTTCCGTATTTTCCCCTGCGGACT

TCTCCTTACCGATCTCCTTGGTCGCTTCTTCCCCACTCTTCCCCTTCACCCGTGTCTCCT

TCCCTTTGTTGCTTTGGCGGGCCTTCGGGCTTGCCCGTTCGACGCGGGTGGGAGGCTTTC

GCCTCTCTCCTCGCGAGCGCCCGCCAAAGGCCCTGTTAACTCTGTTTTAGTGTCTTCCGA

GCAACCATGT

>ASV948 GS|85.3|AF250779|SH0954634.09FU;k:Fungi,p:Ascomycota,c:Lecanoromycetes,o:Caliciales,f:Caliciaceae,g:Buellia,s:Buellia frigida

AAGTCGTAATAAGATTTTCGTAGATGAATTTGCGAAAGAATCATTATCGAGAGACGAAAT

CGCTTCGATCTCACTCTTCACTCGTGTCTACTTATCTTTGTTGCTTTGACGAATCTTCGA

GCTTGATCGTTCGACGCGAGTGAAAGACTTTCGTCTCTCGCCTCGCGAGCGCTCGTCAAA

GATCTTGTTAACTTTGTTTTAGTGTCTTTCGAACAATCATGTAATAGTTAAAACTTTCAA

CAACGAATCT

>ASV949 GS|85.3|AF250779|SH0954634.09FU;k:Fungi,p:Ascomycota,c:Lecanoromycetes,o:Caliciales,f:Caliciaceae,g:Buellia,s:Buellia frigida

AAGTCGTAACAAGATTTTCGTAGATGAATTTGCGAAAGAATCATTATCGAGAGACGAAAT

CGCTTCGATCTCACTCTTCACTCGTGTCTACTTATCTTTGTTGCTTTGACGAGTCTTCGA

ACTTGATCGTTCGACGCGAGTGAGAGACTTTCGTCTCTCGTCTCGCGAGCGCTCGTCAAA

GATTTTGTTAACTCTATTTTAGTGTCTTTCGAGCAATCATGTAATAGTTAAAACTTTCAA

CAACGAATCT

>ASV950 SS|0.8200|MN592663|SH1107827.09FU;k:Fungi,p:Ascomycota,c:Lecanoromycetes,o:Teloschistales,f:Teloschistaceae

AAGTCGTAATAAGGTTTCCGTAGGTGAACCTGCGGAAGGATTATTATCGAGAGGGGGGGC

TCTATGCCCCGGGGCTCTATCCCCGTACCTTTTCACCCTGTGTGTATTTTTCCCCCGTTG

CTTTGGCGGGCCCCGGGTCTTCCCCCGGCGTTAGCCCCCTCGCGGGGTTCGCGAGCGCCC

GCCGGAAGGCTTATCGAAACTCTGATTAGTGCAGTCTGAGCGTACGAATAATAAATTAAA

ACTTTCAATA

>ASV951 GSL|98.9|KF309980|SH0993867.09FU;k:Fungi,p:Ascomycota,c:Dothideomycetes,o:Mycosphaerellales,f:Extremaceae

AAGTCGTAACAAGGTCTCCGTAGGTGAACCTGCGGAGGGATCATTACCGAGTGAGGGCCC

TCGCGCCCGACCTCCAACCCTTTGTCGATCAATATCTGTTGCCTCGGGGGGCGACCCGGC

CGTCCGCGGGCGGGGGTCCCCCAGAGGACCAATCAACTCTGCATCTTTGCGTCGAGTATT

GAGTACGAATCAATCAAAACTTTTAACAACGGATCTCTTGGTTCTG

>ASV952 GS|0.0|None;No hit

GTGTCAGCAGCCGCGGTAATACGGAGGGTGCGAGCGTTGTCCGGATTTATTGGGTTTAAA

GGGTGCGTAGGTGGGGTTCTAAGTCTGGTTTGAAAGCAGGCGGCTCAACCGTCTGATGTG

GCTGGAAACTGGGGTTCTTGAATGGGTTGGCGGTAGCCGGAACGGGTCATGTAGCGGTGA

AATGCATAGATATGACCCAGAACACCGATTGCGAAGGCAGGCTACTACGACTTGATTGAC

ACTGAGGCAC

>ASV953 SS|1.0000|KF617635|SH1319225.09FU;k:Fungi

AAGTCGTAACAAGGTTTCCGTAGGTGAACCTGCGGAAGGATCATTACAGAGAAAAAACGG

GCTACGCGGGGAGGGCAACCTCACCCGCGGGCTCACATCTCATAAACCCCCTGTCTACCG

ACCTCAACACAAACCCAGTACAAACACCAAAGTCGTCCCTAGCGGGGCGCGCCAAACTGA

ATGTACAAAAAACAAAACTTTCAACAACGGATCTCTTGGTTCTG

>ASV954 GS|100.0|JQ074199|SH1299503.09FU;k:Fungi,p:Ascomycota,c:Lecanoromycetes,o:Teloschistales,f:Teloschistaceae,g:Austroplaca,s:Austroplaca darbishirei

AAGTCGTAACAAGGTTTCCGTAGGTGAACCTGCGGAAGGATCATTACCGAGAGAGGGGTT

TCGCGCCCCCGGGGGGGTTTCGGCCCCCCTTACCTCTTCAACCCTGTGCCTACCAACCTT

TGTTGCTTTGGCGAGCGCCGGGGTGCCTTGCGCCTCGGCCCCGGCTTCGGTCGGTGCGCT

CTCGTCAGAGGCCCTTTTCCACGCTGTTTTCAGTGACGTCCGAGTGAAACAAACAATAAA

TTAAAACTTT

>ASV955 GS|0.0|None;No hit

GTGTCAGCAGCCGCGGTAATACGTAGGGGGCGAGCGTTGTCCGAAGTTACTGGGCGTAAA

GAGCGCGTAGGCGGGTTCTTAAGTGAGGGGTGAAAGTCCGAGGCTCAACCTCGGAACTGC

CTTTCATACTGGGAACCTTGAGTGTGGGAGAGGCGAGTGGAATGGTCGGTGTAGCGGTGA

AATGCGTAGATATCGATCGGAACACCCATGGCGAAGGCAGCTCGCTGGCCTATAACTGAC

GCTGAGGCGC

>ASV956 GS|78.9|KT714247|SH1142646.09FU;k:Fungi,p:Basidiomycota,c:Agaricomycetes,o:Polyporales,f:Irpicaceae,g:Flavodon,s:Flavodon flavus

AAGTCGTAACAAGGTCTCCGTAGGTGAACCTGCGGAGGGATCATTAACACGCTCCAAACC

CCAATATTAACCCAATCTGTGAAGTTTTGTATCTGCCATCGGCTTGAATTCGAGCACTCT

TTCTTGTTAGAGTGCAACGCGGTGTCAGCTGAGATCCAGGATGAAGGTGATGAGATGCTT

GACTGCTGCTGTTCTTTTCTCGAAGGAGAGCTTCAGTCCTCTCGTCATACGGGTCGAGTT

GACTTTTGGT

>ASV957 GS|0.0|None;No hit

GTGTCAGCAGCCGCGGTAATACGAAGGGGGCTAGCGTTGCTCGGAATGACTGGGCGTAAA

GGGCGCGTAGGCGGATCGGATAGTCAGGCGTGAAATTCCTGGGCTCAACCTGGGGGCTGC

GTTTGATACGTTTGGTCTAGAGTGGGGAAGAGGGTTGTGGAATTCCCAGTGTAGAGGTGA

AATTCGTAGATATTGGGAAGAACACCGGTGGCGAAGGCGGCAACCTGGTCCTTGACTGAC

GCTGAGGCGC

>ASV958 SS|1.0000|KC965222|SH0962008.09FU;k:Fungi

AAGTCGTAACAAGGTTTCCGTAGGTGAACCTGCGGAAGGATCATTAAAGAGAAATCGGGG

TATCGCCGCGAGGCTTTACCCCATCTCCCTAAACCCCCTGTCTACCGACCTCACACAAAC

CACCGTACAAATATCAAGTCGTCCCTAGCGGGGCGCAATACCATAAATGTACAAAAAACA

AAACTTTTAACAACGGATCTCTTGGTTCTG

>ASV959 GS|72.7|UDB01571335|SH0954626.09FU;k:Fungi,p:Ascomycota,c:Lecanoromycetes,o:Caliciales,f:Caliciaceae,g:Buellia

AAATCGTAACAAGATTTTCGTAAATGAATTTATGAAAAAATTATTATCGAGAAACGAAAT

CGTTTCGATCTCATTTTTCACTCGTATCTACTTATCTTTGTTGCTTTGACGAATTTTCGA

ATTTGATCGTTCGACGCGAGTGAGAGACTTTCGTCTCTCGTTTCGCGAGCGCTCGTCAAA

GACTCTATTAACTCTATTTTAATGTCTTTCGAACAATTATATAATAGTTAAAATTTTCAA

CAACGAATCT

>ASV960 GS|99.2|GU170840|SH0942719.09FU;k:Fungi,p:Ascomycota,c:Lecanoromycetes,o:Lecanorales,f:Lecanoraceae,g:Lecanora,s:Lecanora fuscobrunnea

AAGTCGTAACAAGGTTTCCGTAGGTGAACCTGCGGAAGGATCATTATCGAGAGGGGTCCC

CGGACTCCGGGGGCTTCGGCCCCCTACTCGTCACCCTATGTCTACACACCTTTGTTGCTT

TGGCGGGCCTCGGGTTCGCCCCGTACCGGCCGTGGGCTTCCATACCCCGGCTGTCCGTGC

CCGTCAGAGGCCCATGAACCCTCGTTTATCAGTGTCGTCCGAGTCCAACCACAATAGTAA

AAACTTTCAA

>ASV961 GS|72.7|MZ229888|SH0913119.09FU;k:Fungi,p:Ascomycota,c:Lecanoromycetes,o:Caliciales,f:Caliciaceae,g:Dimelaena

AAGTCGTAACAAGATTTCCGTAAATGAATTTGCGAAAGAATCATTATCGAGAAACGGAGT

TTAACCGATCCCACTCTTCACTCGTGTATATTTACTCTTTATTGTTTTGACGGGCGATCG

GTCTTTATCGCCCGACGTCGGTCGGAAAACTCGCGTCTTTCACTCACCGAACGCTCGTCA

AAGATTCTCAAAATCCGTTCATTTATGTTCGAGCGAATGAAGCAAATAATTAAAAACTTT

CAACAACGGA

>ASV962 SS|1.0000|AF250779|SH0954634.09FU;k:Fungi,p:Ascomycota,c:Lecanoromycetes,o:Caliciales,f:Caliciaceae,g:Buellia,s:Buellia frigida

CTTTTTCCTTTCTCTTCCTTCCCCTTCTTCCCCCTTTTTCCGTATTTTCCCCTGCGGACT

TCTCCTTACCGATCTCCTTGGTCGCTTCTTCCCCACTCTTCCCCCTTGTCTACTTACCTT

TGTTGCTTTGGCGGGCCTTCGGGCTTGACCGTTCGCCGCGGGTGGGAGGCTTTCGCCTCT

CGCCTCGCGCGCGCCCGCCAAAGGCCGTGTTAACTCTGTTTTAGTGTCTTCCGAGCAACC

ATGTAATAGT

>ASV963 GS|88.2|AF250779|SH0954634.09FU;k:Fungi,p:Ascomycota,c:Lecanoromycetes,o:Caliciales,f:Caliciaceae,g:Buellia,s:Buellia frigida

AAGTCGTAACAAGATTTTCGTAGATGAATCTACGAAAGAATCATTATCGAGAGACGAGAT

CGCTTCGACCTCACTCTTCACTCGTGTCTACTTATCTTTGTTGCTTTGACGAGCCTTTGA

GCTTGATCGTTCGACGCGAGTGGAAGACTTTCGTCTCTCGTCTCGCGAGCGCTCGTCAAA

GACCTTGTTAACTCTGTTTTAGTGTCTTTCGAGCAATCATGTAATAGTTAAAACTTTCAA

CAACGAATCT

>ASV964 GS|88.4|AY667583|SH0954634.09FU;k:Fungi,p:Ascomycota,c:Lecanoromycetes,o:Caliciales,f:Caliciaceae,g:Buellia,s:Buellia frigida

AAGTCGTAACAAGATTTCCGTAGATGAACTTGCGAAAGAATCATTATCGAGAGACGAAAT

CGCTTCGACCTCACTCTTCACTCGTGTCTACTTATCTTTGTTGCTTTGGCGAGTCTTCGA

GCTTGATCGTTCGACGCGAGTGGGAGGCTTTCGCCTCTCGTCTCGCGAGCGCTCGTCAAA

GGCTTTGTTAACTCTATTTTAGTGTCTTTCGAGCAATCATGTAATAATTAAAATTTTCAA

CAACGAATCT

>ASV965 GS|85.3|AF250779|SH0954634.09FU;k:Fungi,p:Ascomycota,c:Lecanoromycetes,o:Caliciales,f:Caliciaceae,g:Buellia,s:Buellia frigida

AAGTCGTAACAAGATTTTCGTAGATGAATTTGCGAAAGAATCATTATCGAGAGATGAAAT

CGCTTCGATCTCACTCTTCACTCGTGTCTACTTATCTTTGTTGCTTTGACGAGTCTTCGA

GCTTGATCGTTCGACGCGAGTGAGAGACTTTCGTCTCTCGTCTCGCGAGCGCTCGTCAAA

GACTTTGTTAACTTTGTTTTAGTGTCTTTCAAGCAATCATGTAATAGTTAAAACTTTCAA

CAACGAATCT

>ASV966 SS|1.0000|KM504479|SH1164905.09FU;k:Fungi,p:Basidiomycota,c:Agaricostilbomycetes,o:Agaricostilbales,f:Chionosphaeraceae,g:Kurtzmanomyces

AAGTCGTAACAAGGTTTTCGTAGGTGAACCTGCGGAAGGATCATTAGTGAATTGCTTTTG

GGAGTCCCTCTTTACCGGAGAAGGCCCATCTCTTCATTACACCCACACACTTGTGCATTT

GTCCCCTTTTCTTCAATGAAAAATTACGTTTTTGACCCTTTATGCATTTGAAACGGTTGT

TGTTGCATATCAAAATGATAAACGAAAGAAAAACTTTCAGCAATGGATCTCTTGGCTCTC

>ASV967 GS|98.0|AF250779|SH0954634.09FU;k:Fungi,p:Ascomycota,c:Lecanoromycetes,o:Caliciales,f:Caliciaceae,g:Buellia,s:Buellia frigida

AAGTCGTAACAAGGTTTTCGTAGGTGAACCTGCGGAAGGATCATTATCGAGAGACGGGGT

CGCTTCGGCCCCACTCTTCACCCGTGTCTACTTACCTTTGTTGCTTTGGCGGGCCTTCGG

GCTTGATCGTTCGACGCGGGTGGGAGGCTTTCGCCTGTCGCCTCGCGAGCGCTCGCCAAA

GGCCCTGTTAACTCTGTTTTAGTGTCTTCCGAGCAACCATGTAATAGTTAAAACTTTCAA

CAACGGATCT

>ASV968 GS|86.8|AF250779|SH0954634.09FU;k:Fungi,p:Ascomycota,c:Lecanoromycetes,o:Caliciales,f:Caliciaceae,g:Buellia,s:Buellia frigida

AAGTCGTAACAAGATTTCCGTAGATGAATCTACGAAAAAATCATTATCGAGAGATGAGAT

CGCTTCGATTTCACTCTTCACTCGTGTCTACTTATCTTTGTTGCTTTGACGAGCCTTCGA

GCTTGATCGTTCGACGCGAGTGAGAGACTTTCGTCTCTCGTCTCGCGAGCGCTCGTCAAA

GGCTTTGTTAACTTTGTTTTAGTGTCTTTCGAGCAATCATGTAATAGTTAAAACTTTCAA

CAACGAATCT

>ASV969 GS|90.2|AF250779|SH0954634.09FU;k:Fungi,p:Ascomycota,c:Lecanoromycetes,o:Caliciales,f:Caliciaceae,g:Buellia,s:Buellia frigida

AAGTCGTAACAAGATTTTCGTAGATGAATCTGCGAAAGAATCATTATCGAGAGACGAGGT

CGCTTCGGCTTCACTCTTCACTCGTGTCTACTTATCTTTGTTGCTTTGGCGAGTCTTCGA

GCTTGATCGTTCGACGCGAGTGGGAGGCTTTCGTCTCTCGTCTCGCGAGCGCTCGTCAAA

GGTCGTGTTAACTTTGTTTTAGTGTCTTTCGAGCAACCATGTAATAGTTAAAACTTTCAA

CAACGAATCT

>ASV970 GS|88.2|AF250779|SH0954634.09FU;k:Fungi,p:Ascomycota,c:Lecanoromycetes,o:Caliciales,f:Caliciaceae,g:Buellia,s:Buellia frigida

AAGTCGTAATAAGATTTTCGTAGATGAACCTACGAAAGAATCATTATCGAGAGACGAGAT

CGCTTCGACCCCACTCTTCACTCGTATCTACTTATCTTTGTTGCTTTGACGAGTCTTTGA

GCTTGATCGTTCGACGCGAATGGGAGGCTTTCGTCTCTCGCCTCGCGAGCGCTCGTCAAA

GGCTCTATTAACTTTGTTTTAGTGTCTTTCGAGCAATCATGTAATAGTTAAAACTTTCAA

CAACGAATCT

>ASV971 GS|80.4|AY667583|SH0954634.09FU;k:Fungi,p:Ascomycota,c:Lecanoromycetes,o:Caliciales,f:Caliciaceae,g:Buellia,s:Buellia frigida

AAGTCGTAACAAGATTTTCGTAGATGAATCTACGAAAAAATTATTATCGAGAGACGAAAT

CGCTTCGATCTCACTTTTTATTCGTATCTACTTATCTTTGTTGCTTTGACGAGTCTTCGA

ACTTAATCGTTCGACGCAAATAAGAGACTTTCGTCTCTCGTTTTGCGAACGCTCGTCAAA

GATCTTGTTAACTCTATTTTAGTGTCTTTCGAGCAATTATGTAATAGTTAAAACTTTCAA

CAACGGATCT

>ASV972 SS|0.9300|MT809481|SH1325656.09FU;k:Fungi,p:Ascomycota

AAGTCGTAACAAGGTTTCCGTAGGTGAACCTGCGGAAGGATCATTACCGAGTTAGGGTCT

TTGGCCCGACCTCCAACCCTATGTGTACCTACCTTTGTTGCTTCGGCGGGCCGGCAGATG

CTCTGGCCGCTGCGTCCCTCTCCCGGGGGCCAGCGAGTGCCCGCCCGAAGGTCTCTTGAA

CCCGTTTCCATGTGCCGTCTGAGTGAGCCTTTAATCGTTAAAACTTTCAACAACGGATCT

CTTGGTTCTG

>ASV973 SS|1.0000|AY667583|SH0954634.09FU;k:Fungi,p:Ascomycota,c:Lecanoromycetes,o:Caliciales,f:Caliciaceae,g:Buellia,s:Buellia frigida

AAGTCGTAACAAGGTTTCCGTAGGTGAATCTACGAAAGGATCATTATCGAGAGACGAGGT

CGCTTCGACCTCACTCTTCACTCGTGTCTACTTACCTTTGTTGCTTTGGCGGGCCTTCGG

GCTTGATCGTTCGACGCGAGTGAAAGGCTTTCGCCTCTCGCCTCGCGAGCGCTCGCCAAA

GGCCCTGTTAACTCTGTTTTAGTGTCTTTCGATCAATCATGTAATAGTTAAAAATTTCAA

CAACGAATCT

>ASV974 GS|78.4|MN592663|SH1107827.09FU;k:Fungi,p:Ascomycota,c:Lecanoromycetes,o:Teloschistales,f:Teloschistaceae,g:Caloplaca,s:Caloplaca haematites

AAGTCGTAATAAGGTTTCCGTAGGTAAACCTGCGGAAGGATTATTATCGAGAGGGGGGGC

TCTATACCCCGGGGCTCTATCCCCGTACCTTTTCACCCTGTATATATTTTTCCCCCGTTG

CTTTAGCGGGCCCCGGGTCTTCCCCCGGCGTTAGCCCCCTCGCGGGGTTCGCGAGCGCCC

GCCGGAAGGCTTATCGAAACTTTAATTAGTGCAGTCTGAGCGTACGAACAATAAATTAAA

ACTTTTAATA

>ASV975 GS|100.0|MK208769|SH0942716.09FU;k:Fungi,p:Ascomycota,c:Lecanoromycetes,o:Lecanorales,f:Lecanoraceae,g:Lecidella

AAGTCGTAACAAGGTTTCCGTAGGTGAACCTGCGGAAGGATCATTAATGAGAGAGGGGCT

TCGCGCTCCCGGGGGCTCCGGCCCCCAACTCTTCACCCTCTGTTTAACCTACCTTTGTTG

CTTTGGCGCGCCCTGGGGTTCCGCTCCACGCCGGCCTCAGGCCTTCGGCTTGGGCTGGTG

AGAGCCCGTCAGAGGCCTATTCCAACCCTTATATCAGTGATGTCCGAGTACAATCTTAAT

AAATAAAACT

>ASV976 GS|0.0|None;No hit

GTGTCAGCAGCCGCGGTAATACGAAGGGGGCTAGCGTTGCTCGGAATGACTGGGCGTAAA

GGGCGCGTAGGCGGAATGTACAGTCAGACGTGAAATTCCTGGGCTCAACCTGGGGACTGC

GTTTGAGACGTGCGTTCTAGAGTTTGGAAGAGGGTCGTGGAATTCCCAGTGTAGAGGTGA

AATTCGTAGATATTGGGAAGAACACCGGTGGCGAAGGCGGCGACCTGGTCCTTGACTGAC

GCTGAGGCGC

>ASV977 SS|0.9000|MH063156|SH1070726.09FU;k:Fungi,p:Ascomycota

AAGTCGTAACAAGGTTTCCGTAGGTGAACCTGCGGAAGGATCATTACTAAGAGAGGGATG

TACGCTTCCAGCCGAGTCCCGGGGGGCTGCGCCCCTCACCTCTTCAACCCTGTGTCTACC

AACCGCTGTTGCTTCGGCGGACCGGTCTCTCGACCGCCGGGGGTTATCGCCCCTGGAACG

TGTCCGCCGACGGCCCAACCACAAACTCTTGCCCCAAACCATGTCGCCTGAATTTACTTG

ATTAAAATCA

>ASV978 GS|99.1|FJ392866|SH1071027.09FU;k:Fungi,p:Ascomycota,c:Eurotiomycetes,o:Chaetothyriales,f:Trichomeriaceae,g:Knufia

AAGTCGTAACAAGGTTTCCGTAGGTGAACCTGCGGAAGGATCATTACCGAGTTAGGGTCT

CTTCGGAGCCCGAACCTCCCAACCCTTTGTCTAATTTACCTTGTCGTTGCTTCGGCGGAC

CGGTTGACCAACTGGTCTTGACCGCCGGGGGTCCCGTACCCCTGGAGAGCGTCCGCCGAC

GGCCCAACCACAAATCCTTGTACTAAACCATGTCGTCTGAATGTACTTGATATTAATCAA

AAAACAAAAC

>ASV979 SS|0.9400|FJ554179|SH0942566.09FU;k:Fungi,p:Ascomycota

AAGTCGTAACAAGGTTTCCGTAGGTGAACCTGCGGAAGGATCATTACTGAGTTAGGGTCC

TCTGGGCTCGACCTCCTACCCTTTGTCTACCTTACCATTTGTTGCTTCGGCGGATCCGTC

CCCTCGGCCCGCGTCCGCCGGTGGCCCAAAACCAAAACTCTTGTTTAAGCGTGTCGTCTA

AGTACAAAACAAATAAGTAAAAACTTTCAACAACGGATCTCTTGGTTCTG

>ASV980 SS|0.9500|UDB0243452|SH1241595.09FU;k:Fungi

AAGTCGTAACAAGGTTTCCGTAGGTGAACCTGCGGAAGGATCATTACTGTATTCTTGCGA

GAATGCCTTCTTTTGTGTCTCTTTTTTTCTTTTCTTTTACACCATTATCTTTTTTCTTTC

ATATTCAAAAGTTTTTAAATAAATACAAAACAAAAAACTTTCAGCAACGGATCTCTTGGC

TCTC

>ASV981 SS|0.9900|MF971624|SH1020757.09FU;k:Fungi

AAGTCGTAACAAGGTTTCCGTAGGTGAACCTGCGGAAGGATCATTAGTGATTGACCTCCG

GGTCCAAACATCATAATCCACACACCTCTGTGAACCGTTGGTCTTTGGACCTACAACCAA

CACAAACATCAGCGTAATGAACGTCAAACGTATTAAACATAATAAAACTTTCAACAACGG

ATCTCTTGGCTCTC

>ASV983 GS|87.6|AY667583|SH0954634.09FU;k:Fungi,p:Ascomycota,c:Lecanoromycetes,o:Caliciales,f:Caliciaceae,g:Buellia,s:Buellia frigida

AAGTCGTAACAAGGTTTTCGTAGATGAATCTACGAAAGGATCATTATCGAGAGACGAGAT

CGCTTCGACTTCACTCTTCACTCGTGTCTACTTATCTTTGTTGCTTTGGCGAGTCTTCGA

GCTTGATCGTTCGACGCGAGTGAAAGACTTTCGTCTCTCGTTTCGCGAGCGCTCGTCAAA

GGTCTTGTTAACTCTGTTTTAGTGTCTTTCGAGCAATCATGTAATAGTTAAAACTTTCAA

CAACGAATCT

>ASV984 GS|99.5|AF276069|SH0954634.09FU;k:Fungi,p:Ascomycota,c:Lecanoromycetes,o:Caliciales,f:Caliciaceae,g:Buellia,s:Buellia frigida

CTTTTTCCTTTCTCTTCCTTCCCCTTCGTCACAAGGTTTCCGTAGGTGAACCTGCGGAAG

GATCATTACCGAGAGACGGGGTCGCTTCGGCCCCACTCTTCACCCGTGTCTACTTACCTT

TGTTGCTTTGGCGGGCCTTCGGGCTTGACCGTTCGACGCGGGTGGGAGGCTTTCGCCTCT

CGCCTCGCGCGCGCCCGCCAAAGGCCCTGTTAACTCTGTTTTAGTGTCTTCCGAGCAACC

ATGTAATAGT

>ASV985 GS|78.5|MZ229889|SH0913114.09FU;k:Fungi,p:Ascomycota,c:Lecanoromycetes,o:Caliciales,f:Caliciaceae,g:Dimelaena

AAGTCGTAACAAGATTTTCGTAAGTGAACCTGCGAAAGGATCATTATCGAGAGACGGAGT

TTAACCGGCCCCACTCTTCACTCGTGTATATCTATCCTTTGTTGCTTTGGCGGACGGTCG

ATCTTTACCGTCCGACGTCGGTCGGAAGGCTCGCGCCTTTCACTCATCGAACGCCCGCCA

GAGACCCTCAAAATTCGTTCATCTATGTTCGAGCGAGTGAAGCAAATAATTAAAAACTTT

CAACAACAGA

>ASV986 GS|0.0|None;No hit

AAGTCGTAACAAGATTTCCGTAAATGAATTTGCGAAAGAATCATTATCGAGAAACGGAGT

TTAACCGATCCCACTCTTCACTCGTGTATATTTACTCTTTATTGTTTTAACGGGCGATCG

GTCTTTATCGCTCGACGTCGGTCGGAAAACTCGCGTCTTTCACTCACCGAACGCTCGTCA

AAGATTCTCAAAATCCGTTCATTTATGTTCGAGCGAATGAAATAAATAATTAAAAACTTT

CAACAACGGA

>ASV987 GS|97.8|AF250779|SH0954634.09FU;k:Fungi,p:Ascomycota,c:Lecanoromycetes,o:Caliciales,f:Caliciaceae,g:Buellia,s:Buellia frigida

CTTTTTCCTTTCTCTTCCTTCCCCTTCGTCCCCCGGTTTCCGTAGGTGCCCCTGCGGACG

TCTCCTTACCGAGCGCCTGGGTCGCTTCGGCCCCACTCTTCACCCGTGTCTACTTACCTT

TGTTGCTTTGGCGGGCCTTCGGGCTTGACCGTTCGACGCGGGTGGGAGGCTTTCGCCTCT

CGCCTCGCGAGCGCCCGCCAAAGGCCGTGTTAACTCTGTTTTAGTGTCTTCCGAGCAACC

ATGTAATAGT

>ASV988 GS|87.3|AF250779|SH0954634.09FU;k:Fungi,p:Ascomycota,c:Lecanoromycetes,o:Caliciales,f:Caliciaceae,g:Buellia,s:Buellia frigida

AAGTCGTAACAAGATTTTCGTAGATGAACTTGCGAAAGAATCATTATCGAGAGACGAGAT

CGCTTCGATCTCACTCTTCACTCGTGTTTACTTATCTTTGTTGCTTTGACGAGTCTTCGA

GCTTGATCGTTCGACGCGAGTGAGAGACTTTCGTCTCTCGTCTCGCGAGCGCTCGTCAAA

GGCTTTGTTAACTCTGTTTTAGTGTCTTTCGAGCAATCATGTAATAGTTAAAACTTTCAA

CAACGAATCT

>ASV989 SS|1.0000|MF138060|SH0954356.09FU;k:Fungi,p:Ascomycota

AAGTCGTAACAAGGTTTCCGTAGGTGTAAGTAATCCAACGTCCCAAACATTCACCATCCA

ATGCGTGATCATGCTAACATGTGATTTAGGAACCTGCGGAAGGATCATTACAGAGTTAGG

GTCTTTCCAGGCCCGACCTCCAACCCTATGTGTACCTACCTTTGTTGCTTTGGCGGGCCC

GTCGGGTGACCCACCGGTGGCCTCTGGCTCCCGAGTGCCCGTCAGAGACCCATCAAAACC

CGTTAATTGT

>ASV990 SS|0.9700|AF250779|SH0954634.09FU;k:Fungi,p:Ascomycota,c:Lecanoromycetes,o:Caliciales,f:Caliciaceae,g:Buellia,s:Buellia frigida

AAGTCGTAACAAGGTTTCCGTAGGTGAATTTGCGGAAGAATCATTATCGAGAGACGAGGT

CGCTTCGGTCCCACTCTTCACCCGTGTCTACTTATCTTTGTTGCTTTGACGAGCCTTCGG

GCTTGATCGTTCGACGCGGGTGGGAGGCTTTCGTCTCTCGTCTCGCGAGCGCCCGCCAAA

GGCCGTGTTAACTCTGTTTTAGTGTCTTTCGAGCAACCATGTAATAGTTAAAACTTTCAA

CAACGGATCT

>ASV991 GS|98.2|MT321782|SH0967927.09FU;k:Fungi,p:Ascomycota,c:Leotiomycetes,o:Helotiales,f:Hyaloscyphaceae

AAGTCGTAACAAGGTCTCCGTAGGTGAACCTGCGGAGGGATCATTACAGAGTTTTTGCCC

GCAAGGGTAGATCTCCCACCCTATGTTATACTGTCTATGTTGCTTTGGCGGGCCGTCAGG

CCTCGGTCAGGCTACCGGCTTCGGCTGGTAAGCGCCCGCCAGAGGACCCCAAACCCTGAA

TATTAGTGTCGTCTGAGTACTATATAATAGTTAAAACTTTCAACAACGGATCTCTTGGTT

CTG

>ASV992 GS|97.5|AF250779|SH0954634.09FU;k:Fungi,p:Ascomycota,c:Lecanoromycetes,o:Caliciales,f:Caliciaceae,g:Buellia,s:Buellia frigida

AAGTCGTAACAAGGTTTTCGTAGGTGAACCTGCGGAAGAATCATTACCGAGAGACGGGGT

CGCTTCGACCCCACTCTTCACCCGTGTCTACTTACCTTTGTTGCTTTGGCGGGCCTTCGA

GCTTGACCGTTCGACGCGAGTGGGAGACTTTCGCCTCTCGCCTCGCGAGCGCCCGCCAAA

GACCCTGTTAACTCTGTTTTAGTGTCTTCCGAGCAACCATGTAATAGTTAAAACTTTCAA

CAACGGATCT

>ASV993 GS|83.8|AF250779|SH0954634.09FU;k:Fungi,p:Ascomycota,c:Lecanoromycetes,o:Caliciales,f:Caliciaceae,g:Buellia,s:Buellia frigida

AAGTCGTAACAAGATTTTCGTAAATGAATTTGCGAAAAAATCATTATCGAGAGACGAAAT

CGCTTCGACTTCACTTTTCACTCGTATCTACTTATCTTTGTTGCTTTGACGAATCTTCGA

GCTTGATCGTTCGACGCGAGTGAGAGACTTTCGTCTCTCGTCTCGCGAGCGTTCGTCAAA

GACTCTATTAACTCTATTTTAGTGTCTTTCGAGCAATCATGTAATAGTTAAAACTTTCAA

CAACGAATTT

>ASV994 SS|0.8900|MN592663|SH1107827.09FU;k:Fungi,p:Ascomycota,c:Lecanoromycetes,o:Teloschistales,f:Teloschistaceae

AAGTCGTAACAAGGTTTCCGTAGGTGAACCTGCGGAAGGATCATTATCGAGAGGGGGGCT

CCATGCCCCGGGGCTCTGTCCCCGTACCTTTTCACCCTGTGTGTATTTTTCCCCCGTTGC

TTTAGCGGGCCCCGGGTCTTCCCCCGGCGTTGGCCCCCTCGCGGGGTTCGCGAGCGCCCG

CCGAAGGCTCATCGAAACTCTGTTAATCAGTGCAGTCTAAGCGTACGAATAATAAATCAA

AACTTTCAAT

>ASV995 GS|100.0|MW798744|SH0907557.09FU;k:Fungi,p:Basidiomycota,c:Tremellomycetes,o:Filobasidiales,f:Filobasidiaceae,g:Filobasidium,s:Filobasidium wieringae

AAGTCGTAACAAGGTTTCCGTAGGTGAACCTGCGGAAGGATCATTAATGAAAATGTGTTG

CCGGGGCCCATAATCCCGGCACTAACCTTCTTATCCATAACACCTGTGCACTGTTGGATG

CTTGCATCCACTTTTATACTAAACAATTTGTAACAAATGTAGTCTTATTATAATTAATAA

AACTTTTAACAACGGATCTCTTGGCTCTC

>ASV996 GS|0.0|None;No hit

GTGTCAGCAGCCGCGGTAATACGAAGGGGGCTAGCGTTGCTCGGAATGACTGGGCGTAAA

GGGCGCGTAGGCGGATGTCTTAGTCAGGCGTGAAATTCCTGGGCTTAACCTGGGGGCTGC

GTTTGATACGGGATGTCTAGAGTTTGGCAGAGGGTCGTGGAATTCCCAGTGTAGAGGTGA

AATTCGTAGATATTGGGAAGAACACCGGTGGCGAAGGCGGCGACCTGGTCCTTGACTGAC

GCTGAGGCGC

>ASV997 GS|99.5|JN873909|SH1261156.09FU;k:Fungi,p:Ascomycota,c:Lecanoromycetes,o:Lecanorales,f:Lecanoraceae,g:Rhizoplaca,s:Rhizoplaca melanophthalma

AAGTCGTAACAAGGTTTCCGTAGGTGAACCTGCGGAAGGATCATTACTGAGAGGGGTTTT

CGGACCCCGGGGGCTTCGGCCCCCATCTCTTCACCCTATGTTTACGTACCTTCGTTGCTT

TGGCGGGTCTTGGGGCTCGCCCCCTACCGGCTCCTGGCCTAGCGCCCCGGCCGGTCCGTG

CCCGCCTGAGGCCCATCAAATTCTGTTCATCAAGTGACGTCCGAGCAAAAACACAATAGT

AAAAACTTTC

>ASV998 GS|98.3|JN873879|SH1123218.09FU;k:Fungi,p:Ascomycota,c:Lecanoromycetes,o:Lecanorales,f:Lecanoraceae,g:Lecanora,s:Lecanora physciella

CTTTTTCCTTTCTCTTCCTTCCCCTTCTTCCCCCGGTTTCCGTAGGTGAACCTGCGGCAG

GATCATTACCGCGAGCGGGGCTAACCCCCCAAACTCCGCCGCCGAAAGGGGTCCTCTCCA

CCCTATGTATACATATACCACTCTCGCTTTGGCGGGCTGAAGGCTCTTGCCCTACGCCCG

CCAGTGGCTCAAAAAATTCTGTTTATCAGTGATGTCCGAGTAAAAACCTAATAGTTTAAA

ACTTTCAACA

>ASV999 GS|100.0|UDB0746256|SH0918856.09FU;k:Fungi

AAGTCGTAACAAGGTTTCCGTAGGTGAACCTGCGGAAGGATCATTAATGAATACACTGGA

CGCTCTTTTTAGAGGTCCGACCAAATCATTACCTTCACACTGTGCACACACTTCTTTTTC

ACCCATTTTAAACACCTTAGTATAAGAATGTACCAGTCTCTTAATTGAGCATAAATATAA

AATAAAACTTTCAGCAACGGATCTCTTGGCTCTC

>ASV1000 GS|79.4|AF250779|SH0954634.09FU;k:Fungi,p:Ascomycota,c:Lecanoromycetes,o:Caliciales,f:Caliciaceae,g:Buellia,s:Buellia frigida

AAGTCGTAACAAAATTTTCGTAAATGAATTTGCGAAAGAATTATTATCGAGAGACGAAAT

CGCTTCGATCTCACTTTTCATTCGTATCTATTTATCTTTGTTATTTTGACGAATCTTCGA

GTTTGATCGTTCGACGCGAGTGAGAGACTTTCGTCTCTCGTTTCGCGAGCGCTCGTTAAA

GATTTTGTTAATTCTATTTTAGTGTCTTTCGAGCAATCATGTAATAGTTAAAACTTTCAA

TAACGAATTT

>ASV1002 GS|83.8|AF250779|SH0954634.09FU;k:Fungi,p:Ascomycota,c:Lecanoromycetes,o:Caliciales,f:Caliciaceae,g:Buellia,s:Buellia frigida

AAGTCGTAACAAGATTTTTGTAGATGAATCTACGAAAGAATCATTATCGAGAGACGAGAT

CGCTTCGATCTCACTCTTCACTCGTATTTACTTATCTTTGTTGCTTTGACGAATCTTCGA

GCTTAATCGTTCGACGCGAGTAAGAGACTTTCGTCTCTCGTCTCGCGAGCGCTCGTCAAA

GACTCTATTAACTTTGTTTTAGTGTCTTTCGAGCAATCATGTAATAGTTAAAACTTTTAA

CAACGAATCT

>ASV1003 GS|84.3|AF250779|SH0954634.09FU;k:Fungi,p:Ascomycota,c:Lecanoromycetes,o:Caliciales,f:Caliciaceae,g:Buellia,s:Buellia frigida

AAGTCGTAACAAGATTTTCGTAGATGAATCTACGAAAGAATTATTATCGAGAGACGAGAT

CGCTTCGATCTCATTCTTCATTCGTATCTACTTATCTTTGTTGCTTTGACGAGTCTTTGA

GCTTGATCGTTCGACGCGAGTGAGAGACTTTCGTCTCTCGTCTCGCGAGCGCTCGTCAAA

GATCTTGTTAACTTTGTTTTAGTGTCTTTCGAGCAATCATGTAATAATTAAAACTTTCAA

CAACGAATCT

>ASV1004 SS|1.0000|AF250779|SH0954634.09FU;k:Fungi,p:Ascomycota,c:Lecanoromycetes,o:Caliciales,f:Caliciaceae,g:Buellia,s:Buellia frigida

AAGTCGTAACAAGATTTTCGTAGATGAATTTGCGAAAGGATCATTATCGAGAGACGGGAT

CGCTTCGGCCTCACTCTTCACTCGTGTCTATTTACCTTTGTTGCTTTGACGAGTCTTCGA

GCTTGATCGTTCGACGCGGGTGGGAGGCTTTCGCCTCTCGCCTCGCGAGCGCCCGCCAAA

GGCCCTGTTAACTCTGTTTTAGTGTCTTCCGAGCAACCATGTAATAGTTAAAACTTTCAA

CAACGGATCT

>ASV1005 GS|80.9|AF250779|SH0954634.09FU;k:Fungi,p:Ascomycota,c:Lecanoromycetes,o:Caliciales,f:Caliciaceae,g:Buellia,s:Buellia frigida

AAGTCGTAACAAGATTTTCATAGATGAATTTGCGAAAAAATCATTATCGAGAGACGAAAT

CGCTTCGATCTCACTTTTCACTCGTATCTACTTATTTTTGTTGCTTTGACGAGTTTTCGA

GCTTGATTATTCGATGCGAGTGAAAGACTTTTGTCTCTCGTCTCGCGAGCGCTCGTCAAA

GATCTTGTTAATTCTATTTTAGTGTCTTTCGAGCAATCATGTAATAGTTAAAATTTTCAA

CAACGAATCT

>ASV1006 GS|84.0|AY667583|SH0954634.09FU;k:Fungi,p:Ascomycota,c:Lecanoromycetes,o:Caliciales,f:Caliciaceae,g:Buellia,s:Buellia frigida

AAGTCGTAACAAGATTTTCGTAGATGAATTTGCGAAAGAATCATTATCGAGAGACGAAGT

CGCTTCGATCTCACTCTTCACTCGTGTCTACTTATCTTTGTTGCTTTAACGAGTCTTCGA

GTTTGATCGTTCGACGCAAGTGAAAGACTTTCGTCTCTCGTCTCGCGAGCGCTCGTCAAA

GACTTTGTTAACTTTGTTTTAGTGTCTTTCGAACAATCATGTAATAATTAAAACTTTCAA

CAACGAATCT

>ASV1007 GS|82.4|AF250779|SH0954634.09FU;k:Fungi,p:Ascomycota,c:Lecanoromycetes,o:Caliciales,f:Caliciaceae,g:Buellia,s:Buellia frigida

AAGTCGTAACAAGATTTTCGTAAATGAATCTACGAAAGAATTATTATCGAAAGACGAGAT

CGCTTCGATCTCACTCTTTATTCGTATCTACTTATCTTTGTTGCTTTGACGAGTTTTCGA

ACTTGATCGTTCGACGCGAGTGAGAGACTTTCGTCTCTCGTCTCGCGAGCGCTCGTCAAA

GATTTTGTTAACTCTATTTTAGTGTCTTTCAAGCAATCATGTAATAATTAAAACTTTCAA

CAACGAATCT

>ASV1008 SS|0.8200|KU164637|SH1223443.09FU;k:Fungi,p:Basidiomycota

AAGTCGTAACAAGGTTTCCGTAGGTGAACCTGCGGAAGGATCATTAATGAAATGAAAGGA

TGCTCTTTTTAGAGGTCCGACCCATTACATTTCCAACACTGTGCACAAACACATTTTTAC

ACCCCTTTTTAACGCATTAGTTATAAGAATGTATAGAGTCTCTTAATTGAGCATAAAATA

AGCAAAACTTTCAGCAACGGATCTCTTGGCTCTC

>ASV1009 SS|0.9700|AF276067|SH0954634.09FU;k:Fungi,p:Ascomycota,c:Lecanoromycetes,o:Caliciales,f:Caliciaceae,g:Buellia,s:Buellia frigida

AAGTCGTAACAAGATTTCCGTAGGTGAACCTGCGAAAGGATCATTATCGAGAGACGAGGT

CGCTTCGATCTCACTCTTCACCCGTGTCTACTTACCTTTGTTGCTTTGGCGGGTCTTCGG

GCTTGATCGTTCGACGCGGGTGGAAGGCTTTCGTCTCTCGCCTCGCGAGCGCTCGTCAAA

GACCCTGTTAACTCTGTTTTAGTGTTTTCCGAGCAATCATGTAATAGTTAAAACTTTCAA

CAACGGATCT

>ASV1010 GS|100.0|MN006700|SH1223465.09FU;k:Fungi,p:Basidiomycota,c:Cystobasidiomycetes,o:Cystobasidiales,f:Cystobasidiaceae,g:Cystobasidium,s:Cystobasidium laryngis

AAGTCGTAACAAGGTTTCCGTAGGTGAACCTGCGGAAGGATCATTAATGAATTTTAGGAC

TCTCTTTTTAGAGGTCCGACCCCTTCATTTCCTTACACTGTGCACACACTTCTTTTCACA

CATTTTAACACTATAGTATAAGAATGTAACAGTCTCTTTATTGAGCATAAATAAAAATAA

AACTTTCAGCAACGGATCTCTTGGCTCTC

>ASV1011 SS|0.9100|MH104936|SH1107788.09FU;k:Fungi,p:Ascomycota,c:Lecanoromycetes,o:Teloschistales,f:Teloschistaceae

AAGTCGTAACAAGGTTTCCGTAGGTGAACCTGCGGAAGGATTATTATCGAGAGGGGGGCT

CCATGCCCCGGGGCTCTGTCCCCGTACCTTTTCACCCTGTGTGTATTTTTCCCCCGTTGC

TTTGGCGGGCCCCGGGTCTTCCCCCGGCGTTGGCCCCCTCGCGGGGTTCGCGAGCGCCCG

CCGAAGGCTCATCGAAACTCTGTTGATCAGTGCAGTCTGAGCGTACGAATAATAAATCAA

AACTTTCAAC

>ASV1012 GSL|100.0|MZ300864|SH1081689.09FU;k:Fungi,p:Ascomycota,c:Dothideomycetes,o:Capnodiales,f:Cladosporiaceae,g:Cladosporium

AAGTCGTAACAAGGTCTCCGTAGGTGAACCTGCGGAGGGATCATTACAAGTGACCCCGGC

TACGGCCGGGATGTTCATAACCCTTTGTTGTCCGACTCTGTTGCCTCCGGGGCGACCCTG

CCTTCGGGCGGGGGCTCCGGGTGGACACTTCAAACTCTTGCGTAACTTTGCAGTCTGAGT

AAACTTAATTAATAAATTAAAACTTTTAACAACGGATCTCTTGGTTCTG

>ASV1013 SS|0.8500|JN873879|SH1123218.09FU;k:Fungi,p:Ascomycota,c:Lecanoromycetes

AAGTCGTAACAAGGTTTCCGTAGGTGAACCTGCGGAAGGATCATTACCGAGAGCGGGGCT

AACCCCCTAAACTCCGCCGCCGAAAGGGGTACTCTCCACCCTATGTATACATACCACTCT

CGCTTTGGCGGGCGCGGAAGGCTTTATGCCCTGCGGCCGCCGGCGGCTCATTCAAATTCG

GTTTATCAGTGACGTCCGAGTAAAAACGCAACAGTTAAAACTTTCAACAACGGATCTCTT

GGTTCTG

>ASV1014 GS|0.0|None;No hit

GTGTCAGCAGCCGCGGTAATACGAAGGGGGCTAGCGTTGCTCGGAATGACTGGGCGTAAA

GGGCGCGTAGGCGGAATGCTTTGTCGGGCGTGAAATTCCAGGGCTTAACCTTGGGACTGC

GTTCGAGACGGGTATTCTAGAGTGGAGAAGAGGGTCGTGGAATTCCCAGTGTAGAGGTGA

AATTCGTAGATATTGGGAAGAACACCGGTGGCGAAGGCGGCGACCTGGTCTTTTACTGAC

GCTGAGGCGC

>ASV1015 GDL|100.0|MW222220|SH1001330.09FU;k:Fungi,p:Ascomycota,c:Dothideomycetes

AAGTCGTAACAAGGTCTCCGTAGGTGAACCTGCGGAGGGATCATTACCGAGTGAGGGTGG

AAACACCCGACCTCCAACCCCATGTCGTTACAACCTTTGTTGCCTCGGGGGCGACCCGGC

CTCGCGCCGGGGCCCCCGATGGACCATCTCACTCTGCGTCTTTGCGTCGGAGTCACAAGT

AAACTGAATCAAAACTTTTAACAACGGATCTCTTGGTTCTG

>ASV1016 GS|0.0|None;No hit

GTGTCAGCAGCCGCGGTAATACGGAGGGTGCGAGCGTTGTCCGGATTTATTGGGTTTAAA

GGGTGCGTAGGTGGGGTTCTAAGTCTGGTTTGAAAGCAGGTGGCTCAACCATCTGATGTG

GCTGGAAACTGGGGTTCTTGAATGGGTTGGCGGTAGCCGGAACGGGTCATGTAGCGGTGA

AATGCATAGATATGACCCAGAACACCGATTGCGAAGGCAGGCTACTACGACTTGATTGAC

ACTGAGGCAC

>ASV1017 GS|99.0|UDB03379129|SH0909821.09FU;k:Fungi,p:Ascomycota,c:Eurotiomycetes,o:Chaetothyriales,f:Herpotrichiellaceae

AAGTCGTAACAAGGTTTCCGTAGGTGAACCTGCGGAAGGATCATTATCGAGTTAGGGTCT

TTATTGGCCCGATCTCCAACCCTGTGTCTATAATACCATGTTGCTTTGGCGGGCCCGCCT

TTAACGGGCCGCCGAGGGTTTTCGGACCCTTGGTCAGTGTCCGCCAGTAGCCAAATCAAA

TTCTTCTTAACTGTGTCGTCTGAGTAAATATTTAAATAAACAAAACTTTCAACAACGGAT

CTCTTGGTTC

>ASV1018 GS|0.0|None;No hit

AAGTCGTAACAAGGTTCCGTAGAACGAGTTCTAGGGATAATGACCCTTCTGCATGTACAC

TTGCCGAAGCCTTGTAGAGCAGCCTGAGAAGGTAGTCTGCGCGACTGTAAATAACGCAGC

TTAAATGCTAGTCTCTTCACCCCCGGGTGATGGGCAACACTTTCAAATTGCGGGGATACC

CTAAAGGCTTTAGATACCAAGCACCGCTAGAGATAGTGGTGTGGCCGGGCTAGTAACCCC

GGGTACGGTG

>ASV1019 GS|0.0|None;No hit

AAGTCGTAACAAGGTTTCCGTAGGTGAACCTGCGGAAGGATCATTACCGAGAGCGGGGCT

AACCCCCCAAACTCCGCCGCCGAAAGGGGTACTCTCCACCCTATGTTTATCAGCTCCTTG

TTGCTTCGGCGGACCGTTGGGGTCAAACCCGCCGCAGGCTTTCGGGCTTGTGAGCGTCCG

TCGGAGGATACTTTTAACGCGTTTAATTCATGTGGTCTGAGTGGGTATTTAATCACCTTA

AAACTTTCAA

>ASV1020 SS|1.0000|HM161461|SH0886713.09FU;k:Fungi,p:Ascomycota,c:Lecanoromycetes,o:Acarosporales,f:Acarosporaceae

CTTTTTCCTTTCTCTTCCTTCCCCTTCGTCCCCCGGTTTCCATGCGGCGGGACATTCATC

TTCCTCGATCTTCCCCTCTGTAAGCAAACCCCCTCTGAAGCCTTGAAATCATGAGTCTGC

ATCTTTGCAGGCGACACTAGTCCGTTGCTGGGAGTCTCCTGGAGATAATCAGCAGCCACG

ATACTGGATTGTGGTTCACAGATCAAACACTAGTGGCCCGTTGGTGGGTTAAAATATGAC

CGGCTCCATC

>ASV1021 GS|74.8|MN615681|SH0916491.09FU;k:Fungi,p:Ascomycota,c:Lecanoromycetes,o:Caliciales,f:Caliciaceae,g:Buellia,s:Buellia insignis

AAGTCGTAACAAGATTTCCGTAAGTGAATCTGCGAAAGGATTATTATCGAGAGACGAAGT

CTAATCGACCTCACTCTTCACCCGTGTATATTTATTCTTTATTGCTTTAACGAGCGATTA

ATCTTTATCGCCCGACGTCGGTCGAAAGACTCGCGTCTTTCACTCATCGAACGCCCGTCA

AAGATCTTCAAAACCCGTTCATTTATGTTCGAGCGAGTGAAGCAAATAATTAAAAACTTT

CAACAACGAA

>ASV1022 SS|1.0000|AF250779|SH0954634.09FU;k:Fungi,p:Ascomycota,c:Lecanoromycetes,o:Caliciales,f:Caliciaceae,g:Buellia,s:Buellia frigida

CTTTTTCCTTTCTCTTCCTTCCCCTTCTTCCCCCTTTTTCCGTATTTTCCCCTGCGGACT

TCTCCTTACCGATCTCCTTGGTCGCTTCTTCCCCACTCTTCCCCCTTGTCTACTTACCTT

TGTTGCTTTGGCGGGCCTTCGGGCTTGACCGTTCGCCGCGGGTGGGAGGCTTTCGCCTCT

CGCCTCGCGCGCGCCCGCCAAAGACTCTGTTAACTCTGTTTTAGTGTCTTCCGAGCAACC

ATGTAATAGT

>ASV1023 GS|77.2|MN615681|SH0916491.09FU;k:Fungi,p:Ascomycota,c:Lecanoromycetes,o:Caliciales,f:Caliciaceae,g:Buellia,s:Buellia insignis

AAGTCGTAACAAGATTTCCGTAAGTGAATCTGCGAAAGAATCATTATCGAAAGACGAAGT

CTAATCGGTCCCACTCTTCACTCGTGTATATTTACTCTTTGTTGCTTTGGCGGGCGGTCG

ATCTTTATCGCCCGACGTCGGTCGGAAGACTCGCGTCTTTCACTCACCGAACGTCCGCCA

AAGGTTTTCAAAACCCGTTCATCTATGTTCGAGCGAGTGAAGTAAATAATTAAAAATTTT

CAACAACGGA

>ASV1024 GS|84.8|AY667583|SH0954634.09FU;k:Fungi,p:Ascomycota,c:Lecanoromycetes,o:Caliciales,f:Caliciaceae,g:Buellia,s:Buellia frigida

AAGTCGTAACAAGATTTTCGTAAGTGAACTTGCGAAAGAATCATTATCGAGAGACGAGAT

CGCTTCGATCTCACTCTTCATTCGTGTCTACTTATCTTTGTTGCTTTGACGAGTCTTCGA

ACTTGATCATTCGACGCGAGTGAGAGACTTTCGTCTCTCGTCTCGCGAGCGCTCGTCAAA

GACTTTGTTAACTTTGTTTTAGTGTCTTTCGAGCAATCATGTAATAATTAAAACTTTCAA

CAACGAATTT

>ASV1025 GS|84.4|AY667583|SH0954634.09FU;k:Fungi,p:Ascomycota,c:Lecanoromycetes,o:Caliciales,f:Caliciaceae,g:Buellia,s:Buellia frigida

AAGTCGTAACAAGATTTTCGTAGATGAATTTGCGAAAGAATCATTATCGAGAGACGAAAT

CGCTTCGACTTCACTCTTCACTCGTGTCTACTTATCTTTGTTGCTTTGACGAATCTTCGA

ACTTAATCGTTCGACGCGAGTGAGAGACTTTCGTCTCTCGTCTCGCGAACGCTCGTCAAA

GATCTTGTTAACTCTATTTTAGTGTCTTTCGAGCAATCATGTAATAGTTAAAACTTTCAA

CAACGAATCT

>ASV1026 GS|86.8|AF250779|SH0954634.09FU;k:Fungi,p:Ascomycota,c:Lecanoromycetes,o:Caliciales,f:Caliciaceae,g:Buellia,s:Buellia frigida

AAGTCGTAACAAGATTTTCGTAGATGAATTTGCGAAAGAATCATTATCGAGAGACGAGAT

CGCTTCGATCTCACTCTTCATTCGTGTCTACTTATCTTTGTTGCTTTGACGAGTCTTCGA

GCTTGATCGTTCGACGCGAGTGGGAGACTTTCGTCTCTCGTCTCGCGAGCGCTCGTCAAA

GACTCTATTAACTTTGTTTTAGTGTCTTTCGAGCAATCATGTAATAGTTAAAACTTTCAA

CAACGAATCT

>ASV1027 GS|85.8|AF250779|SH0954634.09FU;k:Fungi,p:Ascomycota,c:Lecanoromycetes,o:Caliciales,f:Caliciaceae,g:Buellia,s:Buellia frigida

AAGTCGTAACAAGATTTTCGTAGATGAATTTGCGAAAGAATCATTATCGAGAGACGAGAT

CGCTTCGATCTCACTCTTCACTCGTGTCTATTTATCTTTGTTGCTTTGGCGAATCTTCGA

GCTTGATCGTTCGACGCGAGTGAAAGGCTTTCGCCTCTCGTCTCGCGAGCGCTCGTCAAA

GATTTTGTTAACTTTGTTTTAGTGTCTTTCGAGCAATCATGTAATAGTTAAAACTTTCAA

CAATGAATCT

>ASV1028 SS|0.8400|HQ701748|SH0952740.09FU;k:Fungi,p:Ascomycota,c:Leotiomycetes

AAGTCGTAACAAGGTCTCCGTAGGTGAACCTGCGGAGGGATCATTAATGAGAACTTGCCC

TTCGGGGTAGATCTCCCACCCTTTGTTTATTATACCTTTGTTGCTTTGTCGGGCCCGTCT

CTCGGGACCGCCGGCTCCGGCTGGCCAGCGCCCGGCAGAGGACTCAAACTCTTGTTTTCA

AAGCCGTCAGAGTACTATATCAATAGTTAAAACTTTCAACAACGGATCTCTTGGTTCTG

>ASV1029 SS|0.8100|UDB01571642|SH1041881.09FU;k:Fungi,p:Basidiomycota

AAGTCGTAACAAGGTTTCCGTAGGTGAACCTGCGGAAGGATCATTAATGTAAACCCCCCG

ATCGGCTCACGCCGTGGTGGGAAATACAAATCCACATACCTCTGTGAACCGTTGACCTCC

GGGTCGTCTTTACAAACATCAGTGTAATGAACGTAAACAAACATAAACAACACAAAACTT

TCAACAACGGATCTCTTGGCTCTC

>ASV1030 GS|81.9|AF250779|SH0954634.09FU;k:Fungi,p:Ascomycota,c:Lecanoromycetes,o:Caliciales,f:Caliciaceae,g:Buellia,s:Buellia frigida

AAGTCGTAACAAGATTTTCGTAAATGAATTTACGAAAAAATCATTATCGAGAGACGAAAT

CGCTTCGATCTCACTCTTCACTCGTATCTACTTATCTTTGTTGCTTTGACGAATCTTCGA

GCTTGATCGTTCGACGCGAATGAAAGACTTTCGTCTCTCGTCTCGCGAACGTTCGTCAAA

GATCTTGTTAACTTTGTTTTAGTGTCTTTCGAACAATCATATAATAATTAAAACTTTCAA

CAACGAATCT

>ASV1031 GS|99.2|KU948788|SH0941383.09FU;k:Fungi,p:Basidiomycota,c:Cystobasidiomycetes

AAGTCGTAACAAGGTTTCCGTAGGTGAACCTGCGGAAGGATCATTAATGAAATGCAAGGG

CGCTCTTTTTAGAGGTCCAACCTATTCATTTTCTCACACTGTGCACACACTATTCACACC

TTTTTAACACTATAGTATAAGAATGTCAACAGTCTCTTAATTGAGCATCAATTGTAATAA

AACTTTAGGCAACGGATCTCTTGGCTCCC

>ASV1033 SS|0.9100|KF007916|SH1107664.09FU;k:Fungi,p:Ascomycota,c:Lecanoromycetes,o:Teloschistales,f:Teloschistaceae

AAGTCGTAACAAGGTTTCCGTAGGTGAACCTGCGGAAGGATCATTATCGAGAGGGGGGTT

CCATGCCCCGGGGCTCTGTCCCCGTACCTTTTCACCCTGTGTGTATTTTTCCCCCGTTGC

TTTGGCGGGCCCCGGGTCTTCCCCCGGCGTCGGCCCCCTCGCGGGGTTCGCGAGCGCCCG

CCGAAGGCTCATCGAAACTCTGCTGGTCAGTGCAGTCTGAGCGTACGAATAATAAATCAA

AACTTTCAAC

>ASV1034 GS|98.0|AF281306|SH1300522.09FU;k:Fungi,p:Ascomycota,c:Lecanoromycetes,o:Teloschistales,f:Teloschistaceae,g:Xanthoria,s:Xanthoria elegans

AAGTCGTAACAAGGTTTCCGTAGGTGAACCTGCGGAAGGATCATTACTAAGAGAGGGATG

TACGCTTCCAGCCGAGTCCCGGGGGGCTGCGCCCCTCACCTCTTCAACCCTGTGTCTACC

AACCGCTGTTGCTTCGGCGAGCGTCGGGGCGTCCGCGCCCCGGCCCCGGCTTCGGTCGGT

GAGCTCTCGCAGAGGCCTATCTTTATTCTGTTTTGCAGTGACGTCCGAGTATACCAACAA

ATAAATTAAA

>ASV1035 SS|1.0000|MF976691|SH1154274.09FU;k:Fungi

AAGTCGTAACAAGGTTTCCGTAGGTGAACCTGCGGAAGGATCATTAAAGATGCGGCGCAA

GCCGTTTAACCAAACACACTGTGAACCGTGGCTTCGGCCTCTTCAAACCATGTAGTACAG

AACGTAATCATGATAATCATCATAACTTTCAACAACGGATCTCTTGGCTCTC

>ASV1036 GS|73.3|KF823589|SH1091255.09FU;k:Fungi,p:Basidiomycota,c:Tremellomycetes,o:Tremellales,f:Sirobasidiaceae

AAGTCGTAACAAGGTTTCCGTAGGTGAAGGATCATTACCGATCGGGCCTTCCTTGGCCTT

CATCCCTCCTACCTGTGTGAACCTGTCCGGGCCCTCGGGCCCGCATTTCAAACAATGTGT

GACGAACGTGGGAAAGGATAAACAGTAAGACTTTCTACGACGGATCTCTTGGCTCTC

>ASV1037 SS|0.9900|KY950299|SH1189084.09FU;k:Fungi,p:Basidiomycota,c:Tremellomycetes,o:Tremellales

AAGTCGTAACAAGGTTTCCGTAGGTGAACCTGCGGAAGGATCATTATTGAACGGCCGGGA

GGCCTAAAACCCATCAAACCTCTGTGAACAACAGCGCCTTCGGGCATGCCCTCGGGCGAT

TACAAACAAAGAAGTCCAGAACGTTAAACATTAAAGAAACATAACTTTCAACAACGGATC

TCTTGGCTCTC

>ASV1038 SS|0.9800|MT981780|SH0943484.09FU;k:Fungi

AAGTCGTAACAAGGTTTCCGTAGGTGAACCTGCGGAAGGATCATTACCGAGATGGGGTCC

TCCGGGGCCCGACCTCCAACCCTTTGTCTACAAACCTCTGTTGCCTCGGCGAGTCGCCGG

GCGGTCTTTAACTAGGACGCCCGCCGTCGGCCCCCCTGGGGCCCTCGAGTGCTCGCCGGT

GGGATTTTATCAAACTCCGAGAAACGAGTCTTCTGAGTGAATTTACAATAACTTCAAAAC

TTTCAACAAC

>ASV1039 GSL|99.5|KF309980|SH0993867.09FU;k:Fungi,p:Ascomycota,c:Dothideomycetes,o:Mycosphaerellales,f:Extremaceae

AAGTCGTAACAAGGTCTCCGTAGGTGAACCTGCGGAGGGATCATTACCGAGTGAGGGCCC

TCGCGCCCGACCTCCAACCCTTTGTCGATCAATATCTGTTGCCTCGGGGGGCGACCCGGC

CGTCCGCGGGCGGGGGTCCCCCAGAGGACCAATCAACTCTGCATCTTTGCGTCGAGTATT

GAATACGAATCAATCAAAACTTTTAACAACGGATCTCTTGGTTCTG

>ASV1041 SS|0.8200|MW580898|SH1196660.09FU;k:Fungi,p:Basidiomycota,c:Tremellomycetes,o:Tremellales,f:Tremellaceae,g:Tremella,s:Tremella wirthii

AAGTCGTAACAAGGTTTCCGTAGGTGAAAATTCGGCCCTCACGGGTCTATAAAAGACACC

TCTGTGAACCTGTCGGCCTCCGGGCCCACCTGCAAACACTGTGTAACGAGCGTTGATGTA

TCATAAGCATAATAAAACTTTCAACAACGGATCTCTTGGCTCTC

>ASV1042 GS|74.5|UDB01721735|SH1098689.09FU;k:Rhizaria,p:Cercozoa

AAGTCGTAACAAGGTCTCCGTAGGTGAACCTGCGGAGGGATCATTAACACGTTCCATACC

AACATATCAACCCAACCTGTGAAGTTTCGTATCTGCCACAAGCTCGTCCAGGTGCTCTCT

TGTCTCTGCTCTGTCTGCCTTTTGTGCAGTCAGTGTGGTGGACAGGGGATACCACGGTGT

CGACTGAGATCCAGGATGAAGGTGATGAGATGCTTGACTCTTGTGGCACTTTGTGTGTTA

ACAACACGCA

>ASV1043 GS|74.2|MN103133|SH0913114.09FU;k:Fungi,p:Ascomycota,c:Lecanoromycetes,o:Caliciales,f:Caliciaceae,g:Dimelaena,s:Dimelaena oreina

AAGTCGTAATAAGATTTTCGTAAGTGAATCTGCGAAAGAATTATTATCGAGAGACGAAAT

CTAACCGGCCCCACTCTTCACTCGTGTATATTTATCCTTTGTTGTTTTGACGGGCGGTCG

GTCTTTATCGCTCGACGTCGGTCGAAAAACTCGCGCCTTTCACTCATCGAACGCCCGCCA

GAGATTTTCAAAACTCGTTTATTTGTGTTCGAGCGAGTGAAATAAATAATTAAAAACTTT

CAACAACGGA

>ASV1044 SS|1.0000|AY667583|SH0954634.09FU;k:Fungi,p:Ascomycota,c:Lecanoromycetes,o:Caliciales,f:Caliciaceae,g:Buellia,s:Buellia frigida

AAGTCGTAACAAGGTTTCCGTAGGTGAACCTGCGGAAGGATCATTACCGAGAGACGGGGT

CGCTTCGGCCCCACTCTTCACCCGTGTCTACTTACCTTTGTTGCTTTGGCGGGCGCTCGC

GAGGCGAGAGGCGAAAGCCTCCCACCCGCGTCGAACGGTCAAGCCCGAAGGCCCTGTTAA

CTCTGTTTTAGTGTCTTCCGAGCAACCATGTAATAGTTAAAACTTTCAACAACGGATCTC

TTGGTTCTG

>ASV1045 GS|73.2|MN615681|SH0916491.09FU;k:Fungi,p:Ascomycota,c:Lecanoromycetes,o:Caliciales,f:Caliciaceae,g:Buellia,s:Buellia insignis

AAGTCGTAACAAGGTTTTCGTAAATGAACCTGCGAAAGAATCATTATTGAGAAACGAAGT

CTAATCGACCTCACTCTTCACCCGTGTATATTCATTCTTTATTGCTTTGACGAGCGATCG

ATCTTTATCGCCCGACGTCGGTCGAAAGACTCGCGCCTTTCACTCATCGAACGCCCGCCA

GAAATTCTCAAAACCCGTTCATTTATGTCCGAGCGAGTAAAATAAATAATTAAAAATTTT

TAATAACGGA

>ASV1046 GS|82.8|AF250779|SH0954634.09FU;k:Fungi,p:Ascomycota,c:Lecanoromycetes,o:Caliciales,f:Caliciaceae,g:Buellia,s:Buellia frigida

AAGTCGTAACAAGATTTTCGTAAATGAATCTACGAAAGAATCATTATCGAGAGACGAAAT

CGCTTCGATCTCACTCTTCACTCGTGTCTACTTATCTTTGTTGCTTTGACGAATCTTCGA

GCATGATCGTTCGACACGAATGAGAGACTTTCGTCTCTCGTTTCGCGAGCGCTCGTCAAA

GACTCTATTAACTTTATTTTAATGTCTTTCGAGCAATCATGTAATAGTTAAAACTTTCAA

CAACGAATCT

>ASV1047 GS|84.3|AF250779|SH0954634.09FU;k:Fungi,p:Ascomycota,c:Lecanoromycetes,o:Caliciales,f:Caliciaceae,g:Buellia,s:Buellia frigida

AAGTCGTAATAAGATTTTCGTAGATGAATTTGCGAAAGAATCATTATCGAGAGACGAAAT

CGCTTCGATCTCACTCTTCACTCGTGTCTACTTATCTTTGTTGCTTTGACGAATCTTCGA

GCTTAATCGTTCGACGCGAGTGAGAGACTTTCGTCTCTCGTTTCGCGAGCGCTCGTCAAA

GATCTTGTTAACTTTGTTTTAGTGTCTTTCGAGCAATCATATAATAGTTAAAACTTTCAA

CAACGAATCT

>ASV1048 SS|0.8500|MZ229889|SH0913114.09FU;k:Fungi,p:Ascomycota,c:Lecanoromycetes,o:Caliciales

AAGTCGTAACAAGATTTCCGTAGGTGAACCTGCGGAAGGATCATTATCGAGAGACGGAGT

CTAACCGGCCCCACTCTTCACTCGTGTATACCTACTCTTTGTTGCTTTGGCGGGCGGTCG

GTCTTTACCGCCCGACGTCGGTCGGAAGGCTCGCGCCTTCCACTCACCGAACGCCCGCCA

GAGGCCCCCAAAAGCCGTTCATCTGTGTCCGAGCGAGTGAAGCAAATAGTTAAAAACTTT

CAACAACGGA

>ASV1049 GS|88.7|AF250779|SH0954634.09FU;k:Fungi,p:Ascomycota,c:Lecanoromycetes,o:Caliciales,f:Caliciaceae,g:Buellia,s:Buellia frigida

AAGTCGTAACAAGATTTTCGTAGATGAATTTACGAAAAAATCATTATCGAGAGACGAGAT

CGCTTCGACCCCACTCTTCACTCGTGTCTACTTATCTTTGTTGCTTTGGCGAGCCTTCGA

ACTTGATCGTTCGACGCGGGTGAGAGACTTTCGCCTCTCGTTTCGCGAGCGCTCGTCAAA

GGTTTTGTTAACTTTGTTTTAGTGTCTTTCGAGCAATCATGTAATAGTTAAAACTTTCAA

CAACGAATCT

>ASV1050 GS|87.3|AF250779|SH0954634.09FU;k:Fungi,p:Ascomycota,c:Lecanoromycetes,o:Caliciales,f:Caliciaceae,g:Buellia,s:Buellia frigida

AAGTCGTAACAAAATTTTCGTAGATGAACTTGCGAAAGAATCATTATCGAGAGACGAGAT

CGCTTCGATCTCACTCTTCACTCGTGTCTACTTATCTTTGTTGCTTTGACGAGTCTTCGA

ACTTGATCGTTCGACGCGAGTGAGAGGCTTTCGTCTCTCGTCTCGCGAGCGCTCGTCAAA

GACTTTGTTAACTCTGTTTTAGTGTCTTTCGAGCAATCATGTAATAGTTAAAACTTTCAA

CAACGAATCT

>ASV1051 SS|0.9900|AF250779|SH0954634.09FU;k:Fungi,p:Ascomycota,c:Lecanoromycetes,o:Caliciales,f:Caliciaceae,g:Buellia,s:Buellia frigida

AAGTCGTAACAAGATTTCCGTAGATGAACTTGCGAAAGAATCATTATCGAGAGACGAAAT

CGCTTCGACCTCACTCTTCACTCGTGTCTACTTATCTTTGTTGCTTTGGCGAGTCTTCGA

GCTTGATCGTTCGACGCGAGTGGGAGGCTTTCGCCTCTCGTCTCGCGAGCGCCCGCCAAA

GGCCCTGTTAACTCTGTTTTAGTGTCTTCCGAGCAACCATGTAATAGTTAAAACTTTCAA

CAACGGATCT

>ASV1052 SS|1.0000|AF250779|SH0954634.09FU;k:Fungi,p:Ascomycota,c:Lecanoromycetes,o:Caliciales,f:Caliciaceae,g:Buellia,s:Buellia frigida

CTTTTTCCTTTCTCTTCCTTCCCCTTCTTCCCCCTTTTTCCGTATTTTCCCCTGCGGACT

TCTCCTTACCGATCTCCTTGGTCGCTTCTTCCCCACTCTTCCCCCTTGTCTACTTACCTT

TTTTGCTTTGGCGGGCCTTCGGGCTTGACCTTTCTCCGCGGGTGGGAGGCTTTCGCCTGT

CGCCTCTCTCTCGCCCGCCAAAGGCCCTGTTAACTCTGTTTTAGTGTCTTCCGAGCAACC

ATGTAATAGT

>ASV1053 SS|1.0000|AF250779|SH0954634.09FU;k:Fungi,p:Ascomycota,c:Lecanoromycetes,o:Caliciales,f:Caliciaceae,g:Buellia,s:Buellia frigida

AAGTCGTAACAAGGTTTTCGTAGATAAACCTACGAAAGAATCATTATCGAGAGACGAGGT

CGCTTCGGCCCCACACTCTTCACTCGTGTCTACTTACCTTTGTTGCTTTGACGGGCCTTC

GAGCTTGATCGTTCGACGCGGGTGGGAGGCTTTCGTCTCTCGCCTCGCGAACGTCCGTCA

AAGATCCTGTTAACTCTATTTTAGTGTCTTTCGAGCAACCATGTAATATTTAAAACTTTC

AACAACGAAT

>ASV1054 SS|1.0000|AF250779|SH0954634.09FU;k:Fungi,p:Ascomycota,c:Lecanoromycetes,o:Caliciales,f:Caliciaceae,g:Buellia,s:Buellia frigida

AAGTCGTAACAAGATTTCCGTAGATGAACTTGCGAAAGAATCATTATCGAGAGACGAAAT

CGCTTCGACCCCACTCTTCACTCGTGTCTACTTACCTTTGTTGCTTTGACGAGCCTTCGA

GCTTGATCGTTCGACGCGAGTGAGAGGCTTTCGCCTCTCGTCTCGCGAGCGCCCGCCAAA

GACCCTGTTAACTCTGTTTTAGTGTCTTTCGAGCAACCATGTAATAGTTAAAACTTTCAA

CAACGAATCT

>ASV1055 SS|1.0000|AF250779|SH0954634.09FU;k:Fungi,p:Ascomycota,c:Lecanoromycetes,o:Caliciales,f:Caliciaceae,g:Buellia,s:Buellia frigida

AAGTCGTAACAAGATTTTCGTAGATGAATTTACGAAAGAATCATTATCGAGAGACGAGAT

CGCTTCGATCTCACTCTTCACTCGTGTCTACTTATCTTTGTTGCTTTAACGAGCCTTCGA

GCTTGATCGTTCGACGCGAGTGAGAGGCTTTCGTCTGTCGTCTCGCGAGCGCTCGTCAAA

GGCCGTGTTAACTCTGTTTTAGTGTCTTCCGAGCAACCATGTAATAGTTAAAACTTTCAA

CAACGGATCT

>ASV1056 GS|84.8|AF250779|SH0954634.09FU;k:Fungi,p:Ascomycota,c:Lecanoromycetes,o:Caliciales,f:Caliciaceae,g:Buellia,s:Buellia frigida

AAGTCGTAACAAGATTTTCGTAGATGAATTTGCGAAAGAATCATTATCGAGAGACGAGAT

CGCTTCGATTTCACTCTTCACTCGTGTCTACTTATCTTTGTTGCTTTGACGAGTCTTCGA

GCTTGATCGTTCGACGCGAGTGAGAGACTTTCGTCTCTCGTCTCGCGAGCGCTCGTCAAA

GATTTTGTTAACTCTGTTTTAGTGTTTTTCGAGCAATCATGTAATAATTAAAATTTTCAA

CAACGAATCT

>ASV1057 GS|85.3|AF250779|SH0954634.09FU;k:Fungi,p:Ascomycota,c:Lecanoromycetes,o:Caliciales,f:Caliciaceae,g:Buellia,s:Buellia frigida

AAGTCGTAACAAGATTTTCGTAGATGAATCTACGAAAAAATCATTATCGAGAGATGAGAT

CGCTTCGATCTCACTCTTCACTCGTATCTACTTATCTTTGTTGCTTTGACGAGTCTTCGA

GCTTGATCGTTCGACGCGAGTGAGAGACTTTCGCCTCTCGTCTTGCGAGCGCTCGTCAAA

GATCTTGTTAACTTTGTTTTAGTGTTTTTCGAGCAATCATGTAATAGTTAAAACTTTCAA

CAACGAATCT

>ASV1058 GS|99.4|JN053103|SH1084083.09FU;k:Fungi,p:Ascomycota,c:Dothideomycetes,o:Capnodiales

AAGTCGTAACAAGGTCTCCGTAGGTGAACCTGCGGAGGGATCATTACTGAGTGGAGGGCC

TCCGGGTCCGACCTCCAACCCCATGTTATCCGACTCTGTTGCCTCGGGGGCGACCCGGCC

TTCGGGTGTCGGGGCCCCCGGTGGACCACTCAACTCTGCATCTTTGCGTCCGAGTCAAAT

GATAAATCAATCAAAACTTTCAACAACGGATCTCTTGGTTCTG

>ASV1059 GS|85.3|AF250779|SH0954634.09FU;k:Fungi,p:Ascomycota,c:Lecanoromycetes,o:Caliciales,f:Caliciaceae,g:Buellia,s:Buellia frigida

AAGTCGTAACAAGATTTTCGTAGATGAATTTGCGAAAGAATCATTATCGAGAGACGAGAT

CGCTTCGATCTCACTCTTCACTCGTGTCTACTTATCTTTGTTGCTTTGACGAATCTTCGA

GCTTGATCGTTCGACGCGAGTGAAAGACTTTCGTCTCTCGTCTCGCGAGCGCTCGTCAAA

GATTTTGTTAACTCTATTTTAGTGTCTTTCGAGCAATCATGTAATAGTTAAAACTTTCAA

CAACGAATCT

>ASV1060 GS|84.0|AY667583|SH0954634.09FU;k:Fungi,p:Ascomycota,c:Lecanoromycetes,o:Caliciales,f:Caliciaceae,g:Buellia,s:Buellia frigida

AAGTCGTAACAAGATTTTCGTAGATGAATTTGCGAAAGAATCATTATCGAGAGACGAAAT

CGCTTCGATCTCACTCTTCATTCATGTCTACTTATCTTTGTTGCTTTGATGAGTCTTCGA

GCTTGATCGTTCGACGCGAGTGAGAGACTTTCGTCTCTCGTCTCGCGAGCGCTCGTCAAA

GATCTTGTTAACTTTGTTTTAATGTCTTTCGAGCAATCATATAATAGTTAAAACTTTCAA

CAACGAATCT

>ASV1061 SS|1.0000|AF276067|SH0954634.09FU;k:Fungi,p:Ascomycota,c:Lecanoromycetes,o:Caliciales,f:Caliciaceae,g:Buellia,s:Buellia frigida

AAGTCGTAACAAGATTTCCGTAGGTGAACCTGCGAAAGGATCATTATCGAGAGACGAGGT

CGCTTCGATCTCACTCTTCACCCGTGTCTACTTACCTTTGTTGCTTTGGCGGGTCTTCGG

GCTTGATCGTTCGACGCGGGTGGGAGGCTTTCGCCTCTCGCCTCGCGAGCGCCCGCCAAA

GGCCCTGTTAACTCTGTTTTAGTGTCTTCCGAGCAACCATGTAATAGTTAAAACTTTCAA

CAACGGATCT

>ASV1062 GS|80.9|AF250779|SH0954634.09FU;k:Fungi,p:Ascomycota,c:Lecanoromycetes,o:Caliciales,f:Caliciaceae,g:Buellia,s:Buellia frigida

AAGTCGTAACAAGATTTTCGTAAATGAATTTGCGAAAAAATCATTATCGAGAGACGAAAT

CGCTTCGATCTCACTTTTCACTCGTATCTACTTATCTTTGTTGCTTTGACGAGTCTTCGA

ACTTGATCGTTCGACGCAAATGAAAGACTTTCGTCTCTCGTTTCGCGAGCGTTCGTCAAA

AATCTTGTTAACTTTGTTTTAGTGTCTTTCGAGCAATCATGTAATAATTAAAACTTTCAA

CAATGAATCT

>ASV1063 SS|1.0000|AF250779|SH0954634.09FU;k:Fungi,p:Ascomycota,c:Lecanoromycetes,o:Caliciales,f:Caliciaceae,g:Buellia,s:Buellia frigida

AAGTCGTAACAAGATTTTCGTAGATGAATTTACGAAAGAATCATTATCGAGAGACGAAAT

CGCTTCGATCTCACTCTTCACTCGTGTCTACTTACCTTTGTTGCTTTGACGAATCTTCGA

ACTTGATCGTTCGACGCGAGTGGGAGGCTTTCGCCTCTCGTCTCGCGAGCGCTCGTCAAA

GATCTTGTTAACTCTGTTTTAGTGTCTTTCGAGCAATCATGTAATAGTTAAAACTTTCAA

CAACGAATCT

>ASV1064 SS|0.8400|MF976691|SH1154274.09FU;k:Fungi,p:Basidiomycota

AAGTCGTAACAAGGTTTCCGTAGGTGAACCTGCGGAAGGATCATTAGAGATTTGCGGCGA

AAGCCGTCAAACCCCCAAACACACCGTGAACCGTGGGCCTCGGCCCCTTACAAACCTGTC

GTAAAGAACGTGATCTATGATAACAAAACAAAACTTTCAACAACGGATCTCTTGGCTCTC

>ASV1065 GS|81.6|AY667583|SH0954634.09FU;k:Fungi,p:Ascomycota,c:Lecanoromycetes,o:Caliciales,f:Caliciaceae,g:Buellia,s:Buellia frigida

AAGTCGTAACAAGATTTTCGTAGATGAATCTACGAAAGAATCATTATCGAGAGACGAAAT

CGCTTCGATCTTACTCTTCATTCGTATTTACTTATCTTTGTTGCTTTGACGAGTCTTCGA

GCTTGATCGTTCGATGCGAATGAAAGACTTTCGTCTCTCGTTTCGCGAGCGTTCGTCAAA

GACTTTGTTAACTTTGTTTTAATGTCTTTCGAGCAATCATGTAATAGTTAAAACTTTTAA

CAACGAATCT

>ASV1066 GS|82.4|AY667583|SH0954634.09FU;k:Fungi,p:Ascomycota,c:Lecanoromycetes,o:Caliciales,f:Caliciaceae,g:Buellia,s:Buellia frigida

AAGTCGTAACAAAATTTTCGTAGATGAATTTGCGAAAGAATCATTATCGAGAGACGAAAT

CGCTTCGATCTCACTTTTCACTCGTGTCTACTTATCTTTGTTGCTTTGACGAGTCTTCGA

ACTTGATCATTCGATGCGAGTGAGAGACTTTCGTCTCTCGTTTCGCAAGCGTTCGTCAAA

GACTCTATTAACTTTGTTTTAGTGTCTTTCGAGCAATCATGTAATAATTAAAACTTTCAA

CAACGAATCT

>ASV1067 SS|1.0000|AY667583|SH0954634.09FU;k:Fungi,p:Ascomycota,c:Lecanoromycetes,o:Caliciales,f:Caliciaceae,g:Buellia,s:Buellia frigida

AAGTCGTAACAAGGTTTTCGTAGGTGAATCTGCGAAAGGATCATTATCGAGAGACGGGGT

CGCTTCGGCCTCACTCTTCACTCGTGTCTACTTATCTTTGTTGCTTTGGCGAGCCTTCGA

GCTTGATCGTTCGACGCGAGTGGGAGGCTTTCGTCTCTCGCCTCGCGAGCGCTCGTCAAA

GGTTCTGTTAACTCTGTTTTAGTGTCTTTCGAGCAATCATGTAATAGTTAAAACTTTCAA

CAACGAATCT

>ASV1068 GSL|100.0|OK236399|SH0985300.09FU;k:Fungi,p:Ascomycota

AAGTCGTAACAAGGTTTCCGTAGGTGAACCTGCGGAAGGATCATTACCTAGAGTTTGTAG

ACTTCGGTCTGCTACCTCTTACCCATGTCTTTTGAGTACCTTCGTTTCCTCGGCGGGTCC

GCCCGCCGATTGGACAACATTCAAACCCTTTGCAGTTGCAATCAGCGTCTGAAAAAACAT

AATAGTTACAACTTTCAACAACGGATCTCTTGGTTCTG

>ASV1069 GS|80.4|MN592663|SH1107827.09FU;k:Fungi,p:Ascomycota,c:Lecanoromycetes,o:Teloschistales,f:Teloschistaceae,g:Caloplaca,s:Caloplaca haematites

AAGTCGTAATAAGGTTTCCGTAGGTAAACCTGCGGAAGGATTATTATCGAGAGGGGGGGC

TCTATACCCCGGGGCTCTGTCCCCGTACCTTTTCACCCTGTGTGTATTTTTCCCCCGTTG

CTTTGGCGGGCCCCGGGTCTTCCCCCGGCGTTAGCCCCCTCGCGGGGTTCGCGAGCGCCC

GCCGGAAGGCTTATCGAAACTCTGATTAGTACAGTCTGAGCGTACGAATAATAAATTAAA

ACTTTTAACA

>ASV1070 SS|1.0000|KF309963|SH1084061.09FU;k:Fungi,p:Ascomycota,c:Dothideomycetes

AAGTCGTAATAAGGTCTCCGTAGGTAAACCTACGGAGGGATTATTACCGAGTAAGGGCCT

CCGGGTCCGACCTCTAACCCCATGTTATCTGACCCTATTGCCTTAGGGGCGACCCGGCCT

TCGGGCGTCTTAGGGCCCCCGGTAGACTACTTAACACTACATCTTTGCATCTAAGTTATA

TTTTGAATCAATCAAAACTTTCAATAACGGATCTCTTGGTTCTA

>ASV1071 GS|100.0|JX171190|SH0981986.09FU;k:Fungi,p:Ascomycota,c:Lecanoromycetes,o:Lecanorales,f:Psilolechiaceae,g:Psilolechia,s:Psilolechia leprosa

AAGTCGTAACAAGGTTTCCGTAGGTGAACCTGCGGAAGGATCATTAATGAGCGAGGGGCC

TCGTGCTCCCGGGGGCTTCGGCCCCCACTCCTCACCCTTTGTATACATACCTTTGTTGCT

TTGGCGGGCCTTGGGGACCTCCCCATGCCGGCTCCGGGCTTCGGTCCCGCCGGCGAGCCG

CCCGCCCGAGGCCTATCAAATTCCGTTTTATCAGTGTTGTCCGAGTCAGATATAACAATC

AAAACTTTCA

>ASV1072 GS|0.0|None;No hit

GTGTCAGCAGCCGCGGTAATACGGAGGATCCAAGCGTTATCCGGATTTATTGGGTTTAAA

GGGTGCGTAGGCGGCCTGTTAAGTCAGGGGTGAAATTTTTCGGCTCAACCGGAAACTTGC

CTTTGATACTGATGGGCTTGAATGCAGCTGAGGTAGGCGGAATGTGACAAGTAGCGGTGA

AATGCATAGATATGTCACAGAACACCAATTGCGAAGGCAGCTTACCAAAGTGTGATTGAC

GCTGAGGCAC

>ASV1073 SS|1.0000|KF274145|SH0988164.09FU;k:Fungi

AAGTCGTAACAAGGTTTCCGTAGGTGAACCTGCGGAAGGATCATTAATGAATGCAAGGAT

GCTCTTTTTAGAGGTCCGACCCATCATTTTCCAACCCTGTGCACATACACTGATATTTTA

CACATCATTTTTAAACACTTGAAGTACAAAAGAATGTCAAAAGTCTCTTGATTGAGCATA

TAATTAAACAAAACTTTCAGCAACGGATCTCTTGGCTCTC

>ASV1074 GS|100.0|KP974240|SH1008712.09FU;k:Fungi,p:Basidiomycota,c:Microbotryomycetes,o:Sporidiobolales,f:Sporidiobolaceae,g:Rhodosporidiobolus,s:Rhodosporidiobolus colostri

AAGTCGTAACAAGGTTTCCGTAGGTGAACCTGCGGAAGGATCATTAGTGAATAACAGGAC

GTTCAATTTAACTTGAAGTCCGAACTCTCACTTTCTAACCCTGTGCATTTGTTTTGGTTA

GTAGAATGCTTGCGTTCGAACACCTCTTCATTTTACAAACACTAAGTCAAAGAATGTTTA

AATTTTATAACAAAACAAAACTTTCAACAACGGATCTCTTGGCTCTC

>ASV1075 SS|0.8600|KJ152485|SH1146347.09FU;k:Fungi,p:Ascomycota,c:Lecanoromycetes,o:Lecanorales,f:Lecanoraceae,g:Palicella

AAGTCGTAACAAGGTTTCCGTAGGTGAACCTGCGGAAGGATCATTAATGAGAGAGGGGCT

TCGCGCTCCCGGGGGTTTCGGCCTCCGACTCTTCACCCTGTGTATACCTTACCTTCGTTG

CTTTGGCGGGCCTTGGAGTTCGTCTCCTGCCGGCCCCGGGCTTCGGTTCGGGTCGGAGCG

CGCCCGTCGGAGGCTCATTCAACTCCGTTTATCAGTGACGTCCGAGTAAAACCATAATAG

TAAAAACTTT

>ASV1076 GS|0.0|None;No hit

GTGTCAGCAGCCGCGGTAATACGGAGGATCCGAGCGTTATCCGGATTTATTGGGTTTAAA

GGGTGCGTAGGCGGCCTGTTAAGTCAGGGGTGAAAGACGGTGGCTCAACCATCGCAGTGC

CTTTGATACTGACGGGCTTGAATGCAGTTGAGGTAGGCGGAATGTGGCAAGTAGCGGTGA

AATGCATAGATATGCCACAGAACACCAATTGCGAAGGCAGCTTACCAAAGTGCGATTGAC

GCTGAGGCAC

>ASV1077 GSL|100.0|MZ540271|SH1008722.09FU;k:Fungi,p:Basidiomycota

AAGTCGTAACAAGGTTTCCGTAGGTGAACCTGCGGAAGGATCATTAGTGAATATAGGACG

TCCAACTTAACTTGGAGTCCGAACTCTCACTTTCTAACCCTGTGCACTTGTTTGGGATAG

TAACTCTCGCAAGAGAGCGAACTCCTATTCACTTATAAACACAAAGTCTATGAATGTATT

AAATTTTATAACAAAATAAAACTTTCAACAACGGATCTCTTGGCTCTC

>ASV1078 GS|71.3|MN103133|SH0913114.09FU;k:Fungi,p:Ascomycota,c:Lecanoromycetes,o:Caliciales,f:Caliciaceae,g:Dimelaena,s:Dimelaena oreina

AAGTCGTAACAAGATTTTCGTAAATGAATTTGCGAAAGAATCATTATCGAGAAACGGAGT

TTAACCGATCCCACTCTTCACTCGTGTATATTTACTCTTTATTGTTTTGACGGGCGATCG

GTTTTTATCGCCCGACGTCGGTCGGAAAACTCGCGTCTTTCACTCACCGAACGCTCGTCA

AAGATTCTCAAAATCCGTTCATTTATGTTCGAGCGAATGAAGCAAATAATTAAAAACTTT

CAACAACGGA

>ASV1079 GS|82.0|AY667583|SH0954634.09FU;k:Fungi,p:Ascomycota,c:Lecanoromycetes,o:Caliciales,f:Caliciaceae,g:Buellia,s:Buellia frigida

AAGTCGTAACAAGATTTTCGTAAATGAATTTGCGAAAGAATCATTATCGAGAGACGAGAT

CGCTTCGATCTCACTCTTCACTCGTATCTACTTATCTTTGTTGCTTTGACGAGTCTTCGA

ATTTGATCGTTCGACGCAAGTGAAAGACTTTCGTCTATCGTTTCGCGAGCGCTCGTCAAA

GATTCTATTAACTCTATTTTAGTGTCTTTCGAGCAATTATGTAATAGTTAAAACTTTTAA

CAACGAATCT

>ASV1080 SS|1.0000|AY667583|SH0954634.09FU;k:Fungi,p:Ascomycota,c:Lecanoromycetes,o:Caliciales,f:Caliciaceae,g:Buellia,s:Buellia frigida

AAGTCGTAACAAGGTTTCCGTAGATGAACCTGCGGAAGGATCATTACCGAGAGACGAAGT

CGCTTCGACCCCACTCTTCACCCGTGTCTACTTACCTTTGTTGCTTTGACGGGCCTTCGA

GCTTGACCGTTCGACGCGAGTGAGAGGCTTTCGCCTGTCGCCTCGCGAGCGCCCGCCAAA

GACCCTGTTAACTCTGTTTTAGTGTCTTCCGAGCAACCATGTAATAGTTAAAACTTTCAA

CAACGGATCT

>ASV1081 SS|0.8200|KU164637|SH1223443.09FU;k:Fungi,p:Basidiomycota,c:Cystobasidiomycetes

AAGTCGTAACAAGGTTTCCGTAGGTGAACCTGCGGAAGGATCATTAATGAAATGAAAGGA

TGCTCTTTTTAGAGGTCCGACCCATTACATTTCCAACACTGTGCACAAACACATTTTTAC

ACCCCTTTTTAACGCATTAGTTATAAGAATGTATAAGGTCTCTTAATTGAGCATAAAATA

AGCAAAACTTTCAGCAACGGATCTCTTGGCTCTC

>ASV1082 GS|79.2|AY667583|SH0954634.09FU;k:Fungi,p:Ascomycota,c:Lecanoromycetes,o:Caliciales,f:Caliciaceae,g:Buellia,s:Buellia frigida

AAGTCGTAACAAGATTTTCGTAGATGAATCTACGAAAAAATTATTATCGAGAGACGAAAT

CGCTTCGATCTCACTTTTTATTCGTATCTACTTATCTTTGTTGCTTTGACGAGTCTTCGA

ACTTAATCGTTCGACGCAAATAAGAGACTTTCGTCTCTCGTTTTGCGAACGCTCGTCAAA

GATCTTGTTAACTCTATTTTAGTGTCTTTCGAGCAATTATGTAATAGTTAAAATTTTCAA

CAACGAATTT

>ASV1083 GS|85.2|AY667583|SH0954634.09FU;k:Fungi,p:Ascomycota,c:Lecanoromycetes,o:Caliciales,f:Caliciaceae,g:Buellia,s:Buellia frigida

AAGTCGTAACAAGATTTTCGTAGATGAACTTGCGAAAGAATCATTATCGAGAGACGAGAT

CGCTTCGATCTCACTCTTCACTCGTGTCTACTTATCTTTGTTGTTTTGACGAGTCTTCGA

GCTTGATCGTTCGACGCGAGTGAAAGACTTTCGTCTCTCGTCTCGCGAGCGCTCGTCAAA

GACTCTATTAACTCTATTTTAGTGTCTTTCGAGCAATCATGTAATAGTTAAAACTTTCAA

CAATGAATTT

>ASV1084 GS|83.8|AF250779|SH0954634.09FU;k:Fungi,p:Ascomycota,c:Lecanoromycetes,o:Caliciales,f:Caliciaceae,g:Buellia,s:Buellia frigida

AAGTCGTAATAAGATTTTCGTAAATGAATTTGCGAAAAAATCATTATCGAGAGACGAGAT

CGCTTCGACTTCACTCTTCACTCGTGTCTACTTATCTTTGTTGCTTTGACGAGTTTTCGA

ACTTGATTGTTCGACGCGAGTGAGAGACTTTCGTCTCTCGTTTCGCGAGCGCTCGTCAAA

GACTCTATTAACTTTGTTTTAGTGTCTTTCAAGCAATCATATAATAGTTAAAACTTTCAA

CAACGAATCT

>ASV1085 GS|79.8|JF357909|SH1107865.09FU;k:Fungi,p:Ascomycota,c:Lecanoromycetes,o:Teloschistales,f:Teloschistaceae,g:Caloplaca,s:Caloplaca bicolor

AAGTCGTAACAAGGTTTCCGTAAGTAAACCTGCGGAAGGATTATTATCGAGAGGGGGGCT

CTATACCCCGGGGCTCCGTCCCCGTACCTTTTCACCCTATATATATTTTTCCCCCGTTGC

TTTAGCGGGCCCCGGGTCTTCCCCCGGCGTTAGCCCCCTCGCGGGGTTCGCGAGCGCCCG

CCGAAGGCTTATCGAAACTCTATTAATTAGTGCAGTCTAAGCGTACGAATAATAAATTAA

AACTTTCAAC

>ASV1086 GS|0.0|None;No hit

GTGTCAGCAGCCGCGGTAATACGGAGGATCCAAGCGTTATCCGGATTTATTGGGTTTAAA

GGGTGCGTAGGCGGCCTGTTAAGTCAGGGGTGAAATTTTCCGGCTCAACCGGGACATTGC

CTTTGATACTGACGGGCTTGAATGCAGCTGAGGTAGGCGGAATGTGACAAGTAGCGGTGA

AATGCATAGATATGTCACAGAACACCAATTGCGAAGGCAGCTTACCAAAGTGTGATTGAC

GCTGAGGCAC

>ASV1087 GS|0.0|None;No hit

AAGTCGTAACAAGGTCTCCGTAGGTGAACCTGCGGAGGGATCATTCACATGAATCCAACC

TATCAACCAACTGTGAACTTGTTATTGCCGTGTGAGATAAGAATAATGGAGTTAGTTAAG

TTGAAGAGGAATGGAATTATTGGTAAAACAATAATTGAAACTTGCTAATCATTAATTGTT

TTATTATTAGTAAAATAATAATAAAGGCTATTGACTGTTTTCTTATTGATCCGGCCCTAT

CTCTGTATCA

>ASV1088 SS|0.8200|AY081152|SH1300501.09FU;k:Fungi,p:Ascomycota,c:Lecanoromycetes,o:Teloschistales,f:Teloschistaceae

AAGTCGTAACAAGGTTTCCGTAGGTGAACCTGCGGAAGGATCATTACCGAGTTAGGGTCT

CTTCGGAGCCCGAACCTCCCAACCCTTTGTCTACCAACCGCTGTTGCTTCGGCGAGCGTC

GGGGCGTCCGCGCCCCGGCCCCGGCTTCGGTCGGTGAGCTCTCGCAGAGGCCTATCTTTA

TTCTGTTTTGCAGTGACGTCCGAGAATACCAATATAATCAATCAAAACTTTCAACAACGG

ATCTCTTGGT

>ASV1089 GS|0.0|None;No hit

GTGTCAGCAGCCGCGGTAATACGAAGGGGGCTAGCGTTGCTCGGAATGACTGGGCGTAAA

GGGCGCGTAGGCGGATTGGTCAGTCAGATGTGAAATTCCTGGGCTTAACCTGGGGGCTGC

ATTTGAGACGGCAGGTCTAGAGTGTGAAAGAGGGTCGTGGAATTCCCAGTGTAGAGGTGA

AATTCGTAGATATTGGGAAGAACACCGGTGGCGAAGGCGGCGACCTGGTTCATAACTGAC

GCTGAGGCGC

>ASV1090 SS|0.9200|None;k:Fungi

AAGTCGTAACAAGGTTTCCGTAGGTGAACCTGCGGAAGGATCATTACTGTATTCACTCTT

GAATGCTTTCTTTTGTGGCCCGCCTTCTTGCCCAACACACAAACCCCCTTCTTCACCCTT

CTTGCCCAACAACAACAACCAAAAGTTTAAAACAAAAACTTTCAGCAACGGATCTCTTGG

CTCTC

>ASV1091 SS|1.0000|KF673749|SH0992234.09FU;k:Fungi

AAGTCGTAACAAGGTTTCCGTAGGTGAACCTGCGGAAGGATCATTACTGAGTTAGGGTCT

TCTAGGCCCGACCTCCAACCCTATGTCTACCTTACCATGTTGCTTTGGCGGGCCCGTCGT

TAGTTCGACCGCCTCCGGGTCAGTGCCCGCCAGAAGCCCAAATTAAATTCTTGATGAAAC

TTGTCGTCTTAGTATACAAGCAATAATAAAAAACTTTCAACAACGGATCTCTTGGTTCTG

>ASV1092 GS|84.4|AY667583|SH0954634.09FU;k:Fungi,p:Ascomycota,c:Lecanoromycetes,o:Caliciales,f:Caliciaceae,g:Buellia,s:Buellia frigida

AAGTCGTAACAAGATTTTCGTAGATGAATTTGCGAAAGAATCATTATCGAGAGACGAGAT

CGCTTCGATCTCACTCTTCACTCGTGTCTACTTATCTTTGTTGCTTTGACGAATTTTCGA

GCTTGATCGTTCGACGCGAGTGAAAGACTTTCGTCTCTCGTCTCGCGAGCGCTCGTCAAA

GACTTTGTTAACTTTGTTTTAGTGTTTTTCGAGCAATCATGTAATAGTTAAAACTTTTAA

CAACGAATCT

>ASV1093 SS|1.0000|AF250779|SH0954634.09FU;k:Fungi,p:Ascomycota,c:Lecanoromycetes,o:Caliciales,f:Caliciaceae,g:Buellia,s:Buellia frigida

CTTTTTCCTTTCTCTTCCTTCCCCTTCTTCCCCCTTTTTCCGTATTTTCCCCTGCGGACT

TCTCCTTACCGATCTCCTTGGTCGCTTCTTCCCCACTCTTCCCCCTTGTCTACTTACCTT

TGTTGCTTTGGCGGGCCTTCGGGCTTGACCTTTCTCCGCGGGTGGGAGGCTTTCGCCTCT

CGCCTCTCTCTCGCCCGCCAAAGGCCGTGTTAACTCTGTTTTAGTGTCTTCCGAGCAACC

ATGTAATAGT

>ASV1094 SS|1.0000|AF250779|SH0954634.09FU;k:Fungi,p:Ascomycota,c:Lecanoromycetes,o:Caliciales,f:Caliciaceae,g:Buellia,s:Buellia frigida

AAGTCGTAACAAGATTTCCGTAGATGAATCTGCGAAAGAATCATTACCGAGAGACGAGAT

CGCTTCGGCTCCACTCTTCACCCGTGTCTACTTACCTTTGTTGCTTTGACGGACCTTCGA

GCCTGATCGTTCGACGCGGGTGGGAGACTTTCGCCTCTCGCCTCGCGAGCGCCCGCCAAA

GACCCTGTTAACTCTGTTTTAGTGTCTTTCGAGCAATCATGTAATAGTTAAAACTTTCAA

CAACGAATCT

>ASV1095 GS|85.3|AF250779|SH0954634.09FU;k:Fungi,p:Ascomycota,c:Lecanoromycetes,o:Caliciales,f:Caliciaceae,g:Buellia,s:Buellia frigida

AAGTCGTAACAAGATTTTCGTAGATGAATTTGCGAAAGAATCATTATCGAGAGACGAAAT

CGCTTCGATCTCACTCTTCATTCGTGTCTACTTATCTTTGTTGCTTTGACGAGTCTTCGA

GCTTGATCGTTCGACGCGAGTGAGAGACTTTCGTCTCTCGTCTCGCGAGCGTTCGTCAAA

GACTTTGTTAACTTTGTTTTAGTGTCTTTCGAGCAATCATGTAATAGTTAAAACTTTCAA

CAACGAATCT

>ASV1096 GS|97.2|AY667583|SH0954634.09FU;k:Fungi,p:Ascomycota,c:Lecanoromycetes,o:Caliciales,f:Caliciaceae,g:Buellia,s:Buellia frigida

AAGTCGTAACAAGGTTTCCGTAGGTGAACCTGCGAAAGGATCATTACCGAGAGACGAGAT

CGCTTCGACCCCACTCTTCACCCGTGTCTACTTACCTTTGTTGCTTTGGCGGGCCTTCGA

GCTTGACCGTTCGACGCGGGTGAGAGGCTTTCGCCTCTCGCCTCGCGAGCGCCCGCCAAA

GGCCCTGTTAACTCTGTTTTAGTGTCTTCCGAGCAACCATGTAATAGTTAAAACTTTCAA

CAACGAATCT

>ASV1097 GS|84.8|AF250779|SH0954634.09FU;k:Fungi,p:Ascomycota,c:Lecanoromycetes,o:Caliciales,f:Caliciaceae,g:Buellia,s:Buellia frigida
[truncated: 241,514 more chars]
