# Supplementary material for: Intrathalline Fungal and Bacterial Diversity Is Uncovered in Antarctic Lichen Symbioses
Source: Environ Microbiol Rep. 2025 May 5;17(3):e70080. doi: 10.1111/1758-2229.70080 (PMC12052756; doi:10.1111/1758-2229.70080)
Supplement: Supplementary file 14 — Supporting Information 2. List of the sequenced bacterial ASVs and their nucleotide sequence. [file EMI4-17-e70080-s002.docx]

>ASV1 SS|1.0000|DQ422812_S001020357;k:Bacteria,p:Cyanobacteria/Chloroplast

GACGGAGGATGCAAGCGTTATCCGGAATGATTGGGCGTAAAGCGTCTGTAGGTGGATTGT

AAAGTCCTCTGTTAAAGATCTGGGCTTAACCCAGTTCAAGCAGTGGAAACTTATAATCTA

GAGTACGGTAGGGGCAGAGGGAATTCCCGGTGTAGCGGTGAAATGCGTAGATATCGGGAA

GAACACCGACAGCGAAAGCACTCTGCTGGGCCGAAACTGACACTGAGAGACGAAAGCTAG

GGGAGCAAAA

>ASV2 GS|0.0|None;No hit

TACGGGGGGGGCAAGCGTTATTCGAAATGATTGGGCGTAAAGGGCACGTAGACGGTTTTT

TAAGTGGCCATCCTTGTTTTTGTTTTTCCCTTTCAACTCTATTATAAATAAAATACATAG

AAGGGAAACGGGAAAAGGGAAGCAAAGATTAAAATGGAGTGTGGATTCTCTTTATTTTTA

TAATCCCTTATTTGTATAAATTATTTATAATGAAAACAAATAAGGGATAACAAGATTCTA

CACTTGGGAA

>ASV3 SS|0.9900|AF166114_S000498654;k:Bacteria,p:Cyanobacteria/Chloroplast

TACGGAGGATGCAAGCGTTATCCGGAATGATTGGGCGTAAAGCGTCTGTAGGTGGGTTGT

AAAGTCTTCTGTTAAAGATCGGGGCTTAACCCAGTTTAAGCAGTGGAAACTTATAACCTA

GAGTACGGTAGGGGCAGAGGGAATTCCCGGTGTAGCGGTGAAATGCGTAGATATCGGGAA

GAACACCGACGGCGAAAGCACTCTGCTGGGCCGAAACTGACACTGAGAGACGAAAGCTAG

GGGAGCAAAA

>ASV4 SS|1.0000|AF166114_S000498654;k:Bacteria,p:Cyanobacteria/Chloroplast

GACGGAGGATGCAAGCGTTATCCGGAATGATTGGGCGTAAAGCGTCTGTAGGTGGGTTGT

GAAGTCTTCTGTTAAAGATCAGGGCTTAACCCTGTTTAGGCAGTGGAAACTCATAACCTA

GAGTACGGTAGGGGCAGAGGGAATTCCCGGTGTAGCGGTGAAATGCGTAGATATCGGGAA

GAACACCGACGGCGAAAGCACTCTGCTGGGCCGAAACTGACACTGAGAGACGAAAGCTAG

GGGAGCGAAA

>ASV5 GS|0.0|None;No hit

TACGGGGGGGGCGAGCGTTATTCGAAATGATTGGGCGTAAAGAGCACGTAGACGGTTTTT

TAAGTGGACATTATATCTTTTTTGTTCTCTAAGGATAAAAAAGAAAGGATTATGGAAATA

TTTCTGTACTCGGGAAAAAGACCAAGGCTCAACCATGGTGTTTCCCGCCAAACTAAAAAA

CTAGAGTAAGTTAGAGGAAAGTGGAATTCCTGGAGGAAAGGTTAAATTTTATGATATCAG

GAGGAACGCC

>ASV6 SS|1.0000|JX949238_S003747851;k:Bacteria,p:"Bacteroidetes",c:Sphingobacteriia,o:"Sphingobacteriales",f:Sphingobacteriaceae

TACGGAGGATCCAAGCGTTATCCGGATTTATTGGGTTTAAAGGGTGCGTAGGCGGCCTGT

TAAGTCAGGGGTGAAATTTTCCGGCTCAACCGGGACATTGCCTTTGATACTGACGGGCTT

GAATGCAGCTGAGGTAGGCGGAATGTGACAAGTAGCGGTGAAATGCATAGATATGTCACA

GAACACCAATTGCGAAGGCAGCTTACTAAAGTGTGATTGACGCTGAGGCACGAAAGCGTG

GGGATCAAAC

>ASV7 GS|0.0|None;No hit

TACGTAGAAGACAAGTGTTATTCATCTTTAATAGGTTTAAAGGGTACTTAGACGGTAAAT

AAAGCCTCCAAAAGGTACTAGTTTGCTAGAGTTTTATATGAAGGAGTTTTAAAGTACTAC

TGGTGTAGAGATGAAATTCAGTTATACCTTACATGGCACAGGTTAAGGCGAAAGCATCTT

CTTATGTAAAAACTGACGTTGAAGGACGAAGGCTTTGTGTCTCGAACAGG

>ASV8 GS|0.0|None;No hit

TACGTGGAAGACTAGTGTTATTCATCTTTAATAGGTTTAAAGGGTACCTAGACGGTATTT

CTAGCCCAAAAAGGGGTACGGATTTACTAGAGTTTTATGTGAGGAGGGGAGTACTTGTGG

AGTAAAGATGAAATTTTTTTATACTATGAGGAAACTGGTAGCGGCGAAAGCAACCTTCTA

TGTATAAACTGACGTTGAGGGACGAAGGCTTGGGTAGCAAATAGG

>ASV9 GS|0.0|None;No hit

TACGGGGGGGGCAAGCGTTATTCGAAATGATTGGGCGTAAAGAGCACGTAGACGGTTTTT

ATAGTAGACATAGTGGCAATATCTCTGTATTAAACATCTTTCACATTTCAAACATCTACA

TCTTTGACATTTCTCATAAATGTTTAGGATGTTAGGATGTCAGAGAAATGTGAAAGATGT

TTACTTCTATATATTTGCATACTTTTGATGGAGTATCTCTTTGTTCATGTATAAACATGA

ACAAATACTC

>ASV10 GS|0.0|None;No hit

TACGTAGAAGACTAGTGTTATTCATCTTTAATAGGTTTAAAGGGTACCTAGACGGTAAAT

CAAGCCCATATGGGGACTACTTTACTAGAGTTACTTATGAGGGGGTATTAAAGTACTGCT

GGTGTAGAGATGAAATTCTGTCATACCTCTTTCGTGGGAAAATTAATGGCACAGGTATAG

GCGAAAGCATCCCCTTATGTGATAACTGACGTTGAAGGACGAAGGCTTTGTGTCGCGAAC

AGG

>ASV11 SS|0.8700|AM947653_S001093907;k:Bacteria,p:"Proteobacteria",c:Alphaproteobacteria,o:Rhodospirillales,f:Acetobacteraceae,g:Acidisoma

TACGAAGGGGGCTAGCGTTGCTCGGAATGACTGGGCGTAAAGGGCGCGTAGGCGGATGTT

TTAGTCAGGCGTGAAAGTCCTGGGCTCAACCTGGGGATTGCGTTTGATACGGGGCATCTA

GAGTTGGGAAGAGGGTCGTGGAATTCCCAGTGTAGAGGTGAAATTCGTAGATATTGGGAA

GAACACCGGTGGCGAAGGCGGCGACCTGGTCCTTGACTGACGCTGAGGCGCGAAAGCGTG

GGGAGCAAAC

>ASV12 SS|1.0000|EF368368_S000806277;k:Bacteria,p:"Proteobacteria",c:Alphaproteobacteria,o:Rhodospirillales,f:Acetobacteraceae

TACGAAGGGGGCTAGCGTTGCTCGGAATGACTGGGCGTAAAGGGCGCGTAGGCGGATTGG

TCAGTCAGACGTGAAATTCCTGGGCTTAACCTGGGGGCTGCGTTTGAGACGGCTGGTCTA

GAGTTTGGAAGAGGGTCGTGGAATTCCCAGTGTAGAGGTGAAATTCGTAGATATTGGGAA

GAACACCGGTGGCGAAGGCGGCGACCTGGTCCTGGACTGACGCTGAGGCGCGAAAGCGTG

GGGAGCAAAC

>ASV13 SS|1.0000|AM947653_S001093907;k:Bacteria,p:"Proteobacteria",c:Alphaproteobacteria,o:Rhodospirillales,f:Acetobacteraceae

TACGAAGGGGGCTAGCGTTGCTCGGAATGACTGGGCGTAAAGGGCGCGTAGGCGGGCATC

TTAGTCAGGCGTGAAATTCCCGGGCTTAACCTGGGGGCTGCGTTTGATACGGGGTGCCTA

GAGTTTGGAAGAGGGTCGTGGAATTCCCAGTGTAGAGGTGAAATTCGTAGATATTGGGAA

GAACACCGGTGGCGAAGGCGGCGACCTGGTCCTTGACTGACGCTGAGGCGCGAAAGCGTG

GGGAGCAAAC

>ASV14 GS|0.0|None;No hit

TACGTGGAAGACTAGTGTTATTCATCTTTATTAGGTTTAAAGGGTACCTAGACGGTATTT

CTAGCCCAAAATAGGGTACGGATTTACTAGAGTTTTATGTGAGGAGGGGAGTACTCGTGG

AGTAGAGATGAAATTTTGTTATACTATGAGGTAACTGGTGGCGGCGAAAGCAACCTTCTA

TGTATAAACTGACGTTGAGGGACGAAGGCTTGGGTAGCAAATAGG

>ASV15 SS|1.0000|AY140238_S000397413;k:Bacteria,p:"Proteobacteria",c:Alphaproteobacteria,o:Rhodospirillales,f:Acetobacteraceae

TACGAAGGGGGCTAGCGTTGCTCGGAATGACTGGGCGTAAAGGGCGCGTAGGCGGACATT

TTAGTCGGGCGTGAAATTCCTGGGCTTAACCTGGGGGCTGCGTTCGATACGGGGTGTCTA

GAGTTTGGCAGAGGGTCGTGGAATTCCCAGTGTAGAGGTGAAATTCGTAGATATTGGGAA

GAACACCGGTGGCGAAGGCGGCGACCTGGTCCTTGACTGACGCTGAGGCGCGAAAGCGTG

GGGAGCAAAC

>ASV16 GS|0.0|None;No hit

TACGGGGGGGGCGAGCGTTATTCGAAATGATTGGGCGTAAAGGGCACGTAGACGGTTTTT

TAAGTGGACATCTTCTCTTTTTTGTTCTCTAAGGATAAAAAAGAAAGGATTATGGAAATC

TTTCTGTACTCGGGAAAAAGACCAAGGCTCAACCATGGTGTTTCCCACCAAACTAAAAAA

CTAGAGTAAGTTAGAGGAAAGTGGAATTCCTGGAGGAAAGGTTAAATTTTATGATATCAG

GAGGAACGCC

>ASV17 GS|0.0|None;No hit

TACGTAGAAGACTAGTGTTATTCATCTTTAATAGGTTTAAAGGGTACCTAGACGGTAAAT

CAAGCCCTTAGGGGGACTACTTTACTAGAGTTACTTATGAGGGGGTATTAAAGTACTGCT

GGTGTAGAGATGAAATTCTGTCATACCTCTTTTCGTGGGAAAAATAATGGCACAGGTATA

GGCGAAAGCATCCCCTTATGTGATAACTGACGTTGAAGGACGAAGGCTTTGTGTCGCGAA

CAGG

>ASV18 SS|1.0000|HM032897_S002167665;k:Bacteria,p:"Bacteroidetes",c:Cytophagia,o:Cytophagales,f:Cytophagaceae,g:Hymenobacter

TACGGAGGGTGCGAGCGTTGTCCGGATTTATTGGGTTTAAAGGGTGCGTAGGCGGCCGTT

TAAGTCTGGGGTGAAAGCCCGCTGCTCAACAGCGGAACTGCCCTGGATACTGGATGGCTT

GAATACAGTGGAGGTTGGCGGAATGGACCGAGTAGCGGTGAAATGCATAGATACGGTCCA

GAACCCCGATTGCGAAGGCAGCTGACTACACTGGTATTGACGCTGAGGCACGACAGCGTG

GGGAGCGAAC

>ASV19 SS|1.0000|AF127407_S000387324;k:Bacteria,p:"Proteobacteria",c:Alphaproteobacteria,o:Rhodospirillales,f:Acetobacteraceae

TACGAAGGGGGCTAGCGTTGCTCGGAATGACTGGGCGTAAAGGGCGCGTAGGCGGATTGG

TCAGTCAGATGTGAAATTCCTGGGCTTAACCTGGGGGCTGCATTTGAGACGGCAGGTCTA

GAGTGTGAAAGAGGGTCGTGGAATTCCCAGTGTAGAGGTGAAATTCGTAGATATTGGGAA

GAACACCGGTGGCGAAGGCGGCGACCTGGTTCATAACTGACGCTGAGGCGCGAAAGCGTG

GGGAGCAAAC

>ASV20 GS|0.0|None;No hit

TTCCAGCTCCAATAGCGTATATTAAAGTTGTTGCAGTTAAAAAGCTCGTAGTTGAAACTT

GGGCCTGGCTGTCCGGTCCGCCTCACCGCGTGCACTGGTTCGGCCGGGCCTTTCCTTCTG

GGGAGCCGCATGCCCTTCATTGGGTGTGCCGGGGAACCAGGACTTTTACTTTGAAAAAAT

TAGAGTGTTCAAAGCAGGCCTATGCTCGAATACATTAGCATGGAATAATAGAATAGGACG

CGTGGTTCTA

>ASV21 SS|1.0000|DQ422812_S001020357;k:Bacteria,p:Cyanobacteria/Chloroplast

GACGGAGGATGCAAGCGTTATCCGGAATGATTGGGCGTAAAGCGTCTGTAGGTGGATTGT

AAAGTCTTCTGTTAAAGATCGGGGCTTAACCCAGTTCAAGCAGTGGAAACTTATAATCTA

GAGTACGGTAGGGGCAGAGGGAATTCCCGGTGTAGCGGTGAAATGCGTAGATATCGGGAA

GAACACCGACAGCGAAAGCACTCTGCTGGGCCGAAACTGACACTGAGAGACGAAAGCTAG

GGGAGCAAAA

>ASV22 SS|0.9800|AF166114_S000498654;k:Bacteria,p:Cyanobacteria/Chloroplast,c:Chloroplast,f:Chloroplast

GACGGGGGATGCAAGTGTTATCCGGAATAATTGGGCGTAAAGCGTCTGTAGGTGGTTTAC

CAAGTCTGTTGTTAAAAATCAGGGCTTAACCCTGATCCGGCAACAGAAACTAGTAGGCTG

GAGTACGGTAGGGGCAGAGGGAATTCTCGGTGTAGTGGTGAAATACGTAGATATCGAGAA

GAACACCAATAGCGAAAGCACTCTGCTGGGCCGAAACTGACATTGAGAGACGAAAGCTAG

GGGAGCGAAA

>ASV23 SS|1.0000|JX949238_S003747851;k:Bacteria,p:"Bacteroidetes",c:Sphingobacteriia,o:"Sphingobacteriales",f:Sphingobacteriaceae

TACGGAGGATCCAAGCGTTATCCGGATTTATTGGGTTTAAAGGGTGCGTAGGCGGCCTGT

TAAGTCAGGGGTGAAATTTTCCGGCTCAACCGGGACATTGCCTTTGATACTGACGGGCTT

GAATGCAGCTGAGGTAGGCGGAATGTGACAAGTAGCGGTGAAATGCATAGATATGTCACA

GAACACCAATTGCGAAGGCAGCTTACCAAAGTGTGATTGACGCTGAGGCACGAAAGCGTG

GGGATCAAAC

>ASV24 SS|1.0000|X75617_S000020692;k:Bacteria,p:"Proteobacteria",c:Alphaproteobacteria,o:Rhodospirillales,f:Acetobacteraceae

TACGAAGGGGGCTAGCGTTGCTCGGAATGACTGGGCGTAAAGGGCGCGTAGGCGGTATGG

ACAGTCAGGTGTGAAATTCCCGGGCTTAACCTGGGGACTGCATTTGATACGTTCAAACTA

GAGTGTGGAAGAGGGTCGTGGAATTCCCAGTGTAGAGGTGAAATTCGTAGATATTGGGAA

GAACACCGGTGGCGAAGGCGGCGACCTGGTCCATTACTGACGCTGAGGCGCGAAAGCGTG

GGGAGCAAAC

>ASV25 GS|0.0|None;No hit

TACGTAGAAGACTAGTGTTATTCATCTTTAATAGGTTTAAAGGGTACCTAGACGGTAAAT

CAAGCCTGCAATATGGGACTAATGTACTAGAGTTACTTACGAGGGGGTATTAAAGTACTG

CTGGTGTAGAGATGAAATTCTGTCATACCTCATTTCATAAGAAATACTATGGCACAGGTA

TAGGCGAAAGCATCCCCTTATGTGATAACTGACGTTGAAGGACGAAGGCTTTGTGTAGCG

AACAGG

>ASV26 GS|99.2|HE599560_S003258029;k:Bacteria,p:"Actinobacteria",c:Actinobacteria,o:Actinomycetales,f:Nakamurellaceae,g:Nakamurella;

TACGTAGGGTGCAAGCGTTGTCCGGAATTATTGGGCGTAAAGAGCTCGTAGGCGGTCTGT

CGCGTCGAATGTGAAAATCCGGGGCTCAACCCCGGACCTGCATTCGATACGGGCAGACTA

GAGTTCGGTAGGGGAGTCTGGAATTCCTGGTGTAGCGGTGAAATGCGCAGATATCAGGAG

GAACACCGGTGGCGAAGGCGGGACTCTGGGCCGATACTGACGCTGAGGAGCGAAAGCGTG

GGGAGCAAAC

>ASV27 SS|1.0000|HM032897_S002167665;k:Bacteria,p:"Bacteroidetes",c:Cytophagia,o:Cytophagales,f:Cytophagaceae,g:Hymenobacter

TACGGAGGGTGCGAGCGTTGTCCGGATTTATTGGGTTTAAAGGGTGCGTAGGCGGCCGTT

TAAGTCTGGGGTGAAAGCCCGCTGCTCAACAGCGGAACTGCCCTGGATACTGGATGGCTT

GAATACAGTGGAGGTTGGCGGAATGGACTGAGTAGCGGTGAAATGCATAGATACAGTCCA

GAACCCCGATTGCGAAGGCAGCTGACTACACTGGTATTGACGCTGAGGCACGACAGCGTG

GGGAGCGAAC

>ASV28 GS|100.0|AJ429239_S000145113;k:Bacteria,p:"Proteobacteria",c:Alphaproteobacteria,o:Sphingomonadales,f:Sphingomonadaceae,g:Sphingomonas;

TACGGAGGGAGCTAGCGTTATTCGGAATTACTGGGCGTAAAGCGCACGTAGGCGGCTTTG

TAAGTAAGAGGTGAAAGCCCAGAGCTCAACTCTGGAATTGCCTTTTAGACTGCATCGCTT

GAATCATGGAGAGGTCAGTGGAATTCCGAGTGTAGAGGTGAAATTCGTAGATATTCGGAA

GAACACCAGTGGCGAAGGCGGCTGACTGGACATGTATTGACGCTGAGGTGCGAAAGCGTG

GGGAGCAAAC

>ASV29 SS|0.8300|AY140238_S000397413;k:Bacteria,p:"Proteobacteria",c:Alphaproteobacteria,o:Rhodospirillales,f:Acetobacteraceae,g:Acidicaldus

TACGAAGGGGGCTAGCGTTGCTCGGAATGACTGGGCGTAAAGGGCGCGTAGGCGGATTTG

TCAGTCGGGCGTGAAATTCCTGGGCTTAACCTGGGGGCTGCGTTCGAGACGGCGGGTCTT

GAGTTTGGAAGAGGGTCGTGGAATTCCCAGTGTAGAGGTGAAATTCGTAGATATTGGGAA

GAACACCGGTGGCGAAGGCGGCGACCTGGTCCTGGACTGACGCTGAGGCGCGAAAGCGTG

GGGAGCAAAC

>ASV30 GS|0.0|None;No hit

CACGTAGAAGACTAGTGTTATTCATCTTTAGTAGGTTTAAAGGGTACCTAGACGGGAGAT

TAAGCCATAGTAGGTACTGATTTTCTAGAGTTTTATGAGAAAAGGTTCGAATTTTCGGAG

TAGAGTTAAAATTTTTTGATACCGAAGAGACGGGTAACGGCGAAGGCAACCTTTTATGTA

AAAACTGACGTTGAGGGACGAAGCCTTGGGTAACGATAAGG

>ASV31 SS|1.0000|AY140238_S000397413;k:Bacteria,p:"Proteobacteria",c:Alphaproteobacteria,o:Rhodospirillales,f:Acetobacteraceae

TACGAAGGGGGCTAGCGTTGCTCGGAATGACTGGGCGTAAAGGGCGCGTAGGCGGAATGC

TTTGTCGGGCGTGAAATTCCAGGGCTTAACCTTGGGACTGCGTTCGAGACGGGTATTCTA

GAGTGGAGAAGAGGGTCGTGGAATTCCCAGTGTAGAGGTGAAATTCGTAGATATTGGGAA

GAACACCGGTGGCGAAGGCGGCGACCTGGTCTTTTACTGACGCTGAGGCGCGAAAGCGTG

GGGAGCAAAC

>ASV32 SS|1.0000|AF523990_S000431354;k:Bacteria,p:"Acidobacteria",c:Acidobacteria_Gp1

TACGAGGGGGGCAAGCGTTGTTCGGAATTATTGGGCGTAAAGGGTGCGTAGGCGGCCCCG

CAAGTCTTGTGTGAAAGCCTCAAGCTCAACTTGAGGACTGCACAGGAAACTGCTGGGCTG

GAGTATGGGAGAGGTGAGTGGAATTCCTGGTGTAGCGGTGAAATGCGTAGATATCAGGAG

GAACACCTGTGGCGAAAGCGGCTCACTGGACCATAACTGACGCTGAGGCACGAAAGCTAG

GGGAGCAAAC

>ASV33 GS|98.4|AB018439_S000439484;k:Bacteria,p:"Proteobacteria",c:Alphaproteobacteria,o:Sphingomonadales,f:Sphingomonadaceae,g:Sphingomonas;

TACGGAGGGAGCTAGCGTTATTCGGAATTACTGGGCGTAAAGCGCACGTAGGCGGCTTTG

TAAGTTAGAGGTGAAAGCCCAGAGCTCAACTCTGGAATTGCCTTTAAGACTGCATCGCTT

GAATCCAGGAGAGGTGAGTGGAATTCCGAGTGTAGAGGTGAAATTCGTAGATATTCGGAA

GAACACCAGTGGCGAAGGCGGCTCACTGGACTGGTATTGACGCTGAGGTGCGAAAGCGTG

GGGAGCAAAC

>ASV34 SS|1.0000|JN090860_S002916046;k:Bacteria,p:"Bacteroidetes",c:Cytophagia,o:Cytophagales,f:Cytophagaceae,g:Hymenobacter

TACGGAGGGTGCGAGCGTTGTCCGGATTTATTGGGTTTAAAGGGTGCGTAGGCGGCCGAT

TAAGTCTGGGGTGAAAGCCCGCTGCTCAACAGCGGAACTGCCCTGGATACTGGTTGGCTT

GAGTACAGACGAGGTTGGCGGAATGGACCGAGTAGCGGTGAAATGCATAGATACGGTCCA

GAACCCCGATTGCGAAGGCAGCTGACTAGGCTGTTACTGACGCTGAGGCACGAAAGCGTG

GGGAGCGAAC

>ASV35 SS|1.0000|AB778531_U010573768;k:Bacteria,p:"Proteobacteria",c:Alphaproteobacteria,o:Rhodospirillales,f:Acetobacteraceae

TACGAAGGGGGCTAGCGTTGCTCGGAATGACTGGGCGTAAAGGGCGCGTAGGCGGATTGA

TCAGTCAGATGTGAAATTCCTGGGCTTAACCTGGGGGCTGCATTTGAGACGGTTAGTCTA

GAGTGTGAAAGAGGGTCGTGGAATTCCCAGTGTAGAGGTGAAATTCGTAGATATTGGGAA

GAACACCGGTGGCGAAGGCGGCGACCTGGTTCATAACTGACGCTGAGGCGCGAAAGCGTG

GGGAGCAAAC

>ASV36 GS|0.0|None;No hit

TACGGGGGGGGCGAGCGTTATTCGAAATGATTGGGCGTAAAGGGCACGTAGACGGTTTTT

TGAGTTGACATGCTTGAGGTCGTACAAAGAATACAAAATAAATATTCTTTCGTACGTCTA

AAAGATTAAAATGGAGTGTGGATTTTCACCCTGTATTCTTTGCTTGATAAAAGAAAAGGT

ATTCGGTTTGCTTTACACTTGGGAAAAAGGCCAAGGCTCAACCATGGTGTTTCCCGCTAT

ACTATAAAAC

>ASV37 SS|1.0000|D86512_S000010801;k:Bacteria,p:"Proteobacteria",c:Alphaproteobacteria,o:Rhodospirillales,f:Acetobacteraceae

TACGAAGGGGGCTAGCGTTGCTCGGAATGACTGGGCGTAAAGGGCGCGTAGGCGGATCAC

ACAGTCAGGCGTGAAATTCCTGGGCTTAACCTGGGGGCTGCGTTTGAGACGTGGGGTCTG

GAGTGGGGAAGAGGGTCGTGGAATTCCCAGTGTAGAGGTGAAATTCGTAGATATTGGGAA

GAACACCGGTGGCGAAGGCGGCGACCTGGTCCTTGACTGACGCTGAGGCGCGAAAGCGTG

GGGAGCAAAC

>ASV38 SS|1.0000|HM032898_S002167666;k:Bacteria,p:"Bacteroidetes",c:Cytophagia,o:Cytophagales,f:Cytophagaceae,g:Hymenobacter

TACGGAGGGTGCGAGCGTTGTCCGGATTTATTGGGTTTAAAGGGTGCGTAGGCGGCTTTT

TAAGTCTGGGGTGAAAGCCCGCTGCTCAACAGCGGAACTGCCCTGGATACTGGAGAGCTT

GAGTACAGACGAGGTTGGCGGAATGGACGGAGTAGCGGTGAAATGCATAGATACCGTCCA

GAACCCCGATTGCGAAGGCAGCTGACTAGGCTGATACTGACGCTGAGGCACGACAGCGTG

GGGAGCGAAC

>ASV39 SS|1.0000|D86513_S000011943;k:Bacteria,p:"Proteobacteria",c:Alphaproteobacteria,o:Rhodospirillales,f:Acetobacteraceae

TACGAAGGGGGCTAGCGTTGCTCGGAATGACTGGGCGTAAAGGGCGCGTAGGCGGTTCGG

ACAGTCAGGTGTGAAATTCCCGGGCTTAACCTGGGGACTGCATTTGATACGTCCGGGCTA

GAGTGCGGAAGAGGGTCGTGGAATTCCCAGTGTAGAGGTGAAATTCGTAGATATTGGGAA

GAACACCGGTGGCGAAGGCGGCGACCTGGTCCGTGACTGACGCTGAGGCGCGAAAGCGTG

GGGAGCAAAC

>ASV40 SS|1.0000|JN090860_S002916046;k:Bacteria,p:"Bacteroidetes",c:Cytophagia,o:Cytophagales,f:Cytophagaceae,g:Hymenobacter

TACGGAGGGTGCGAGCGTTGTCCGGATTTATTGGGTTTAAAGGGTGCGTAGGCGGCCGCG

TAAGTCCGGGGTGAAAGCCCGTTGCTCAACAACGGAACTGCCCTGGAAACTGCGCGGCTT

GAGTCCAGACGAGGTTGGCGGAATGGGCGGTGTAGCGGTGAAATGCATAGATACCGTCCA

GAACCCCGATTGCGAAGGCAGCTGACTAGGCTGGTACTGACGCTGAGGCACGAAAGCGTG

GGGAGCGAAC

>ASV41 SS|1.0000|JN090860_S002916046;k:Bacteria,p:"Bacteroidetes",c:Cytophagia,o:Cytophagales,f:Cytophagaceae,g:Hymenobacter

TACGGAGGGTGCGAGCGTTGTCCGGATTTATTGGGTTTAAAGGGTGCGTAGGCGGTCGAT

TAAGTCTGGGGTGAAAGCCCGCTGCTCAACAGCGGAACTGCCCTGGATACTGGTTGACTT

GAGTACAGACGAGGTTGGCGGAATGGACGGAGTAGCGGTGAAATGCATAGATACCGTCCA

GAACCCCGATTGCGAAGGCAGCTGACTAGGCTGTTACTGACGCTGAGGCACGAAAGCGTG

GGGAGCGAAC

>ASV42 GS|0.0|None;No hit

TACGGGGGGGGCAAGCGTTATTCGAAATGATTGGGCGTAAAGGGCACGTAGACGGTTTTT

TAAGTGGCCATCCTTGTTTTTGTTTTTGCCTTTCAACTCTATTATAAATAAAATACATAG

AAGGGAAACGGGAAAAGGGAAGCAAAGATTAAAATGGAGTGTGGATTCTCTTTATTTTTA

TAATCCCTTATTTGTATAAATTATTTATAATGAAAACAAATAAGGGATAACAAGATTCTA

CACTTGGGAA

>ASV43 GS|98.0|JN090860_S002916046;k:Bacteria,p:"Bacteroidetes",c:Cytophagia,o:Cytophagales,f:Cytophagaceae,g:Hymenobacter;

TACGGAGGGTGCGAGCGTTGTCCGGATTTATTGGGTTTAAAGGGTGCGTAGGCGGCCTTA

TAAGTCCGGGGTGAAAGCCCGTTGCTCAACAACGGAACTGCCCTGGATACTGTGAGGCTT

GAGTACAGACGAGGTTGGCGGAATGGACCGAGTAGCGGTGAAATGCATAGATACGGTCCA

GAACCCCGATTGCGAAGGCAGCTGACTAGGCTGTTACTGACGCTGAGGCACGACAGCGTG

GGGAGCGAAC

>ASV44 SS|1.0000|EU861928_S001148233;k:Bacteria,p:"Armatimonadetes",c:Armatimonadia,o:Armatimonadales,f:Armatimonadaceae,g:Armatimonas/Armatimonadetes_gp1

TACGTAGGGGGCGAGCGTTGTTCGAAGTTACTGGGCGTAAAGAGCGCGTAGGCGGGTCTT

TAAGTGAGGGGTGAAATTCCGAGGCTCAACCTCGGAACTGCCTTTTATACTGGGGACCTT

GAGTGTGGGAGAGGCGAGTGGAATGGTCGGTGTAGCGGTGAAATGCGTAGATATCGATCG

GAACACCCATGGCGAAGGCAGCTCGCTGGCCTATAACTGACGCTGAGGCGCGAAAGCGTG

GGGAGCAAAC

>ASV45 SS|0.9900|AM231587_S000650722;k:Bacteria,p:"Proteobacteria",c:Alphaproteobacteria,o:Rhodospirillales,f:Acetobacteraceae

TACGAAGGGGGCTAGCGTTGCTCGGAATGACTGGGCGTAAAGGGCGCGTAGGCGGCTTGG

TGAGTTAGACGTGAAATTCCTGGGCTCAACCTGGGGGCTGCGTTTGATACAGCTAGGCTA

GAGTGGGGAAGAGGGTTGTGGAATTCCCAGTGTAGAGGTGAAATTCGTAGATATTGGGAA

GAACACCGGTGGCGAAGGCGGCAACCTGGTCCTTGACTGACGCTGAGGCGCGAAAGCGTG

GGGAGCAAAC

>ASV46 SS|1.0000|JN090860_S002916046;k:Bacteria,p:"Bacteroidetes",c:Cytophagia,o:Cytophagales,f:Cytophagaceae,g:Hymenobacter

TACGGAGGGTGCGAGCGTTGTCCGGATTTATTGGGTTTAAAGGGTGCGTAGGCGGCCGAT

TAAGTCTGGGGTGAAAGCCCGCTGCTCAACAGCGGAACTGCCCTGGATACTGGCTGGCTT

GAGTACAGACGAGGTTGGCGGAATGGACCGAGTAGCGGTGAAATGCATAGATACGGTCCA

GAACCCCGATTGCGAAGGCAGCTGACTAGGCTGTTACTGACGCTGAGGCACGAAAGCGTG

GGGAGCGAAC

>ASV47 SS|1.0000|JX949238_S003747851;k:Bacteria,p:"Bacteroidetes",c:Sphingobacteriia,o:"Sphingobacteriales",f:Sphingobacteriaceae

TACGGAGGATCCGAGCGTTATCCGGATTTATTGGGTTTAAAGGGTGCGTAGGCGGCCTGT

TAAGTCAGGGGTGAAATTTTTCGGCTCAACCGGAAACTTGCCTTTGATACTGATGGGCTT

GAATGCAGCTGAGGTAGGCGGAATGTGACAAGTAGCGGTGAAATGCATAGATATGTCACA

GAACACCAATTGCGAAGGCAGCTTACTAAAGTGTGATTGACGCTGAGGCACGAAAGCGTG

GGGATCAAAC

>ASV48 SS|1.0000|JN090860_S002916046;k:Bacteria,p:"Bacteroidetes",c:Cytophagia,o:Cytophagales,f:Cytophagaceae,g:Hymenobacter

TACGGAGGGTGCGAGCGTTGTCCGGATTTATTGGGTTTAAAGGGTGCGTAGGCGGCCGTT

TAAGTCTGGGGTGAAAGCCCGCTGCTCAACAGCGGAACTGCCCTGGATACTGGATGGCTT

GAGTACAGACGAGGTTGGCGGAATGGACGGAGTAGCGGTGAAATGCATAGATACCGTCCA

GAACCCCGATTGCGAAGGCAGCTGACTAGGCTGTTACTGACGCTGAGGCACGAAAGCGTG

GGGAGCGAAC

>ASV49 SS|1.0000|AY140238_S000397413;k:Bacteria,p:"Proteobacteria",c:Alphaproteobacteria,o:Rhodospirillales,f:Acetobacteraceae

TACGAAGGGGGCTAGCGTTGCTCGGAATGACTGGGCGTAAAGGGCGCGTAGGCGGATTGA

TCAGTCGGACGTGAAATTCCTGGGCTTAACCTGGGGGCTGCGTTCGAGACGGTTGGTCTT

GAGTTTGGAAGAGGGTCGTGGAATTCCCAGTGTAGAGGTGAAATTCGTAGATATTGGGAA

GAACACCGGTGGCGAAGGCGGCGACCTGGTCCTGGACTGACGCTGAGGCGCGAAAGCGTG

GGGAGCAAAC

>ASV50 GS|99.2|DQ321750_S000636629;k:Bacteria,p:"Actinobacteria",c:Actinobacteria,o:Actinomycetales,f:Nakamurellaceae,g:Nakamurella;

TACGTAGGGTGCAAGCGTTGTCCGGAATTATTGGGCGTAAAGAGCTCGTAGGCGGTTTGT

CGCGTCGAATGTGAAAATCCGGGGCTCAACCCCGGACCTGCATTCGATACGGGCAGACTA

GAGTTCGGTAGGGGAGTCTGGAATTCCTGGTGTAGCGGTGAAATGCGCAGATATCAGGAG

GAACACCGGTGGCGAAGGCGGGACTCTGGGCCGATACTGACGCTGAGGAGCGAAAGCGTG

GGGAGCAAAC

>ASV51 SS|0.9800|AM887759_S000941902;k:Bacteria,p:"Acidobacteria",c:Acidobacteria_Gp1,g:Granulicella

TACGAGGGGGGCAAGCGTTGTTCGGATTTATTGGGCGTAAAGGGTGCGTAGGCGGTTTGG

CAAGTCTTATGTGAAATCTATGGGCTCAACCCATAGTCTGCATGAGAAACTACCGGGCTT

GAGTATTGGAGAGGTGAGTGGAATTTCCGGTGTAGCGGTGAAATGCGTAGATATCGGAAG

GAACACCTGTGGCGAAAGCGGCTCACTGGACAATAACTGACGCTGATGCACGAAAGCTAG

GGGAGCAAAC

>ASV52 SS|1.0000|D86513_S000011943;k:Bacteria,p:"Proteobacteria",c:Alphaproteobacteria,o:Rhodospirillales,f:Acetobacteraceae

TACGAAGGGGGCTAGCGTTGCTCGGAATGACTGGGCGTAAAGGGCGCGTAGGCGGATTGG

TCAGTCGGGCGTGAAATTCCTGGGCTTAACCTGGGGGCTGCGTTCGAGACGGCTGGTCTG

GAGTGGGGAAGAGGGTCGTGGAATTCCCAGTGTAGAGGTGAAATTCGTAGATATTGGGAA

GAACACCGGTGGCGAAGGCGGCGACCTGGTCCTTGACTGACGCTGAGGCGCGAAAGCGTG

GGGAGCAAAC

>ASV53 SS|1.0000|JX949238_S003747851;k:Bacteria,p:"Bacteroidetes",c:Sphingobacteriia,o:"Sphingobacteriales",f:Sphingobacteriaceae

TACGGAGGATCCAAGCGTTATCCGGATTTATTGGGTTTAAAGGGTGCGTAGGCGGCCTGT

TAAGTCAGGGGTGAAATTTTCCGGCTCAACCGGGACATTGCCTTTGATACTGATGGGCTT

GAATGCAGCTGAGGTAGGCGGAATGTGACAAGTAGCGGTGAAATGCATAGATATGTCACA

GAACACCAATTGCGAAGGCAGCTTACTAAAGTGTGATTGACGCTGAGGCACGAAAGCGTG

GGGATCAAAC

>ASV54 SS|1.0000|EU861928_S001148233;k:Bacteria,p:"Armatimonadetes",c:Armatimonadia,o:Armatimonadales,f:Armatimonadaceae,g:Armatimonas/Armatimonadetes_gp1

TACGTAGGGGGCGAGCGTTGTCCGAAGTTACTGGGCGTAAAGAGCGCGTAGGCGGGTTCT

TAAGTGAGGGGTGAAATTCCGAGGCTTAACCTCGGAACTGCCTTTCATACTGGGAACCTT

GAGTGTGGGAGAGGCGAGTGGAATGGTCGGTGTAGCGGTGAAATGCGTAGATATCGATCG

GAACACCCATGGCGAAGGCAGCTCGCTGGCCTATAACTGACGCTGAGGCGCGAAAGCGTG

GGGAGCAAAC

>ASV55 SS|1.0000|AY788950_S000610642;k:Bacteria,p:"Proteobacteria",c:Alphaproteobacteria,o:Rhodospirillales,f:Acetobacteraceae

TACGAAGGGGGCTAGCGTTGCTCGGAATGACTGGGCGTAAAGGGCGCGTAGGCGGCTTGT

ACAGTCAGATGTGAAATTCCTGGGCTTAACCTGGGGGCTGCATTTGATACGTGCGGGCTA

GAGTGTGAAAGAGGGTCGTGGAATTCCCAGTGTAGAGGTGAAATTCGTAGATATTGGGAA

GAACACCGGTGGCGAAGGCGGCGACCTGGTTCATGACTGACGCTGAGGCGCGAAAGCGTG

GGGAGCAAAC

>ASV56 GS|0.0|None;No hit

TACGTAAAAGACTAGTGTTATTCATCTTTAATAGGTTTAAAGGGTACTTAGACGGTAAAT

AAAGCCCGAATAGGGAACTAATATACTAGAGTTTGATGTAAGGAAGCATGAAAGTACTGT

TGGTGTAGAGATAAAATTCTATCATACCAAATATGGCACAGGTTTAGGCGAAAGCATCTT

CTTATGTAATAACTGACGTTGAAGGACGAAGGCTTTGTGTCTCGAACAGG

>ASV57 GS|0.0|None;No hit

TTCCAGCTCCAATAGCGTATATTAAAGTTGTTGCAGTTAAAAAGCTCGTAGTTGAACCTT

GGGCCTGGCTGGCCGGTCCGCCTCACCGCGTGCACTGGTCCGGCCGGGCCTTTCCTTCTG

GGGATCCACATGCCCTTCACTGGGTGTGCCGGGGAACCAGGACTTTTACTTTGAAAAAAT

TAGAGTGTTCAAAGCAGGCCTATGCTCGAATACATTAGCATGGAATAATAGAATAGGACG

TGTGGTTCTA

>ASV58 SS|1.0000|EU861928_S001148233;k:Bacteria,p:"Armatimonadetes",c:Armatimonadia,o:Armatimonadales,f:Armatimonadaceae,g:Armatimonas/Armatimonadetes_gp1

TACGTAGGGGGCGAGCGTTGTCCGAAGTTACTGGGCGTAAAGAGCGCGTAGGCGGGTTCT

TAAGTGAGGGGTGAAAGTCCGAGGCTCAACCTCGGAACTGCCTTTCATACTGGGAACCTT

GAGTGTGGGAGAGGCGAGTGGAATGGTCGGTGTAGCGGTGAAATGCGTAGATATCGATCG

GAACACCCATGGCGAAGGCAGCTCGCTGGCCTATAACTGACGCTGAGGCGCGAAAGCGTG

GGGAGCAAAC

>ASV59 SS|1.0000|KF999686_S004084195;k:Bacteria,p:"Bacteroidetes",c:Cytophagia,o:Cytophagales,f:Cytophagaceae,g:Spirosoma

TACGGAGGGTGCGAGCGTTGTCCGGATTTATTGGGTTTAAAGGGTGCGTAGGTGGGGTTC

TAAGTCTGGTTTGAAAGCAGGTGGCTCAACCATCTGATGTGGCTGGAAACTGGGGTTCTT

GAATGGGTTGGCGGTAGCCGGAACGGGTCATGTAGCGGTGAAATGCATAGATATGACCCA

GAACACCGATTGCGAAGGCAGGCTACTACGACTTGATTGACACTGAGGCACGAGAGCCGG

GGTAGCGAAC

>ASV60 GS|97.6|JN695632_S002960974;k:Bacteria,p:"Bacteroidetes",c:Sphingobacteriia,o:"Sphingobacteriales",f:Sphingobacteriaceae,g:Mucilaginibacter;

TACGGAGGATCCGAGCGTTATCCGGATTTATTGGGTTTAAAGGGTGCGTAGGCGGCCTGT

TAAGTCAGGGGTGAAAGACGGTAGCTCAACTATCGCAGTGCCTTTGATACTGACGGGCTT

GAATGCAGCTGAGGTAGGCGGAATGTGACAAGTAGCGGTGAAATGCATAGATATGTCACA

GAACACCAATTGCGAAGGCAGCTTACTAAAGTGTGATTGACGCTGAGGCACGAAAGCGTG

GGGATCAAAC

>ASV61 SS|1.0000|JN090860_S002916046;k:Bacteria,p:"Bacteroidetes",c:Cytophagia,o:Cytophagales,f:Cytophagaceae,g:Hymenobacter

TACGGAGGGTGCGAGCGTTGTCCGGATTTATTGGGTTTAAAGGGTGCGTAGGCGGCCGCG

TAAGTCCGGGGTGAAAGCCCGTTGCTCAACAACGGAACTGCCCTGGAAACTGCGCGGCTT

GAGTCCAGACGAGGTCGGCGGAATGGGCGGTGTAGCGGTGAAATGCATAGATACCGTCCA

GAACCCCGATTGCGAAGGCAGCTGACTAGGCTGGTACTGACGCTGAGGCACGAAAGCGTG

GGGAGCGAAC

>ASV62 GS|100.0|L37599_S000414713;k:Bacteria,p:Firmicutes,c:Bacilli,o:Bacillales,f:Staphylococcaceae,g:Staphylococcus;

TACGTAGGTGGCAAGCGTTATCCGGAATTATTGGGCGTAAAGCGCGCGTAGGCGGTTTTT

TAAGTCTGATGTGAAAGCCCACGGCTCAACCGTGGAGGGTCATTGGAAACTGGAAAACTT

GAGTGCAGAAGAGGAAAGTGGAATTCCATGTGTAGCGGTGAAATGCGCAGAGATATGGAG

GAACACCAGTGGCGAAGGCGACTTTCTGGTCTGTAACTGACGCTGATGTGCGAAAGCGTG

GGGATCAAAC

>ASV63 SS|1.0000|JF999998_S002914924;k:Bacteria,p:"Bacteroidetes",c:Sphingobacteriia,o:"Sphingobacteriales",f:Sphingobacteriaceae

TACGGAGGATCCAAGCGTTATCCGGATTTATTGGGTTTAAAGGGTGCGTAGGCGGCCTGT

TAAGTCAGGGGTGAAAGACGGTGGCTCAACCATCGCAGTGCCTTTGATACTGACGGGCTT

GAATGCAGCTGAGGTAGGCGGAATGTGACAAGTAGCGGTGAAATGCATAGATATGTCACA

GAACACCAATTGCGAAGGCAGCTTACTAAAGTGTGATTGACGCTGAGGCACGAAAGCGTG

GGGATCAAAC

>ASV64 SS|1.0000|HQ436503_S002339683;k:Bacteria,p:"Proteobacteria",c:Alphaproteobacteria,o:Rhodospirillales,f:Acetobacteraceae

TACGAAGGGGGCTAGCGTTGCTCGGAATGACTGGGCGTAAAGGGCGCGTAGGCGGCTTTG

TTTGTCAGACGTGAAAGTCCTGGGCTTAACCTGGGGATTGCGTATGGGACGGCAGGGCTA

GAGTGGGGAAGAGGGTTGTGGAATTCCCAGTGTAGAGGTGAAATTCGTAGATATTGGGAA

GAACACCGGTGGCGAAGGCGGCGACCTGGTCCTTTTACTGACGCTGAGGCGCGAGAGCGT

GGGGAGCAAA

>ASV65 GS|0.0|None;No hit

TTCCAGCTCCAATAGCGTATATTAAAGTTGTTGCAGTTAAAAAGCTCGTAGTTGAAACTT

GGGCCTGGCTGGCCGGTCCGCCTCACCGCGTGCACTGGTCCGGCCGGGCCTTTCCTTCTG

GGGAGCCGCATGCCCTTCACTGGGCGTGCCGGGGAACCAGGACTTTTACTTTGAAAAAAT

TAGAGTGTTCAAAGCAGGCCTATGCTCGAATACATTAGCATGGAATAATAGAATAGGACG

TGTGGTTCTA

>ASV66 SS|1.0000|JQ436923_S003312544;k:Bacteria,p:"Proteobacteria",c:Alphaproteobacteria,o:Rhodospirillales,f:Acetobacteraceae

TACGAAGGGGGCTAGCGTTGCTCGGAATGACTGGGCGTAAAGGGCGCGTAGGCGGTTTGT

ACAGTCGGATGTGAAATTCCTGGGCTTAACCTGGGGGCTGCATTCGATACGTGCAGGCTT

GAGTGTGGAAGAGGGTTGTGGAATTCCCAGTGTAGAGGTGAAATTCGTAGATATTGGGAA

GAACACCGGTGGCGAAGGCGGCAACCTGGTCCATGACTGACGCTGAGGCGCGAAAGCGTG

GGGAGCAAAC

>ASV67 SS|1.0000|AB377116_S000994777;k:Bacteria,p:"Actinobacteria",c:Actinobacteria,o:Actinomycetales

TACGTAGGGTGCAAGCGTTGTCCGGAATTATTGGGCGTAAAGAGCTCGTAGGCGGTTCGT

CGCGTCTGCTGTGAAAACCTGGGGCTCAACCCCGGGCGTGCAGTGGGTACGGGCGGGCTA

GAGTGCAGTAGGGGAGACTGGAATTCCTGGTGTAGCGGTGAAATGCGCAGATATCAGGAG

GAACACCGGTGGCGAAGGCGGGTCTCTGGGCTGTTACTGACGCTGAGGAGCGAAAGCGTG

GGGAGCGAAC

>ASV68 SS|1.0000|DQ422812_S001020357;k:Bacteria,p:Cyanobacteria/Chloroplast

GACGGAGGATGCAAGCGTTATCCGGAATGATTGGGCGTAAAGCGTCTGTAGGTGGATTGT

AAAGTCCTCTGTTAAAGATCGGGGCTTAACCCAGTTCAAGCAGTGGAAACTTATAATCTA

GAGTACGGTAGGGGCAGAGGGAATTCCCGGTGTAGCGGTGAAATGCGTAGATATCGGGAA

GAACACCGACAGCGAAAGCACTCTGCTGGGCCGAAACTGACACTAAGAGACGAAAGCTAG

GGGAGCAAAA

>ASV69 GS|0.0|None;No hit

TACGTAGAAGACAAGTGTTATTCATCTTTAATAGGTTTAAAGGGTACTTAGACGGTAAAT

CAAGCCTTAATAAGGGACTAATTTACTAGAGTTTTATGTGAAAAAGTATTAAAGTACTGC

TGGTGTAGAGATGAAATTCTGTCATACCTTAAATGGCACAGGTTAAGGCGAAAGCATCTT

TTTATGTAAAAACTGACGTTGAAGGACGAAGGCTTTGTGTCTCGAACAGG

>ASV70 SS|1.0000|D86513_S000011943;k:Bacteria,p:"Proteobacteria",c:Alphaproteobacteria,o:Rhodospirillales,f:Acetobacteraceae

TACGAAGGGGGCTAGCGTTGCTCGGAATGACTGGGCGTAAAGGGCGCGTAGGCGGTTCGG

ACAGTCAGGTGTGAAATTCCCGGGCTTAACCTGGGGACTGCATTTGATACGTCCGGACTA

GAGTGCGGAAGAGGGTCGTGGAATTCCCAGTGTAGAGGTGAAATTCGTAGATATTGGGAA

GAACACCGGTGGCGAAGGCGGCGACCTGGTCCGTGACTGACGCTGAGGCGCGAAAGCGTG

GGGAGCAAAC

>ASV71 SS|1.0000|AB362219_S001043921;k:Bacteria,p:"Proteobacteria",c:Alphaproteobacteria,o:Rhodospirillales,f:Acetobacteraceae

TACGAAGGGGGCTAGCGTTGCTCGGAATGACTGGGCGTAAAGGGCGCGTAGGCGGAATGT

ACAGTCAGACGTGAAATTCCTGGGCTCAACCTGGGGACTGCGTTTGAGACGTGCGTTCTA

GAGTTTGGAAGAGGGTCGTGGAATTCCCAGTGTAGAGGTGAAATTCGTAGATATTGGGAA

GAACACCGGTGGCGAAGGCGGCGACCTGGTCCTTGACTGACGCTGAGGCGCGAAAGCGTG

GGGAGCAAAC

>ASV72 SS|1.0000|AB778530_U010573767;k:Bacteria,p:"Proteobacteria",c:Alphaproteobacteria,o:Rhodospirillales,f:Acetobacteraceae

TACGAAGGGGGCTAGCGTTGCTCGGAATGACTGGGCGTAAAGGGCGCGTAGGCGGTTCAC

GCAGTCAGATGTGAAATTCCTGGGCTTAACCTGGGGGCTGCATTTGAGACGCGTGGGCTT

GAGTGTGAAAGAGGGTCGTGGAATTCCCAGTGTAGAGGTGAAATTCGTAGATATTGGGAA

GAACACCGGTGGCGAAGGCGGCGACCTGGTTCACAACTGACGCTGAGGCGCGAAAGCGTG

GGGAGCAAAC

>ASV73 SS|1.0000|HM032898_S002167666;k:Bacteria,p:"Bacteroidetes",c:Cytophagia,o:Cytophagales,f:Cytophagaceae,g:Hymenobacter

TACGGAGGGTGCGAGCGTTGTCCGGATTTATTGGGTTTAAAGGGTGCGTAGGCGGCTTTT

TAAGTCTGGGGTGAAAGCCCGCTGCTCAACAGCGGAACGGCCCTGGATACTGGGGAGCTT

GAGTACAGACGAGGTTGGCGGAATGGACGGAGTAGCGGTGAAATGCATAGATACCGTCCA

GAACCCCGATTGCGAAGGCAGCTGACTAGGCTGCTACTGACGCTGAGGCACGACAGCGTG

GGGAGCGAAC

>ASV74 SS|1.0000|D86513_S000011943;k:Bacteria,p:"Proteobacteria",c:Alphaproteobacteria,o:Rhodospirillales,f:Acetobacteraceae

TACGAAGGGGGCTAGCGTTGCTCGGAATGACTGGGCGTAAAGGGCGCGTAGGCGGTTCGG

ACAGTCAGGTGTGAAATTCCCGGGCTTAACCTGGGGACTGCATTTGATACGTCTGGGCTA

GAGTGCGGAAGAGGGTCGTGGAATTCCCAGTGTAGAGGTGAAATTCGTAGATATTGGGAA

GAACACCGGTGGCGAAGGCGGCGACCTGGTCCGTGACTGACGCTGAGGCGCGAAAGCGTG

GGGAGCAAAC

>ASV75 GS|98.4|AM229669_S000643543;k:Bacteria,p:"Proteobacteria",c:Alphaproteobacteria,o:Sphingomonadales,f:Sphingomonadaceae,g:Sphingomonas;

TACGGAGGGAGCTAGCGTTATTCGGAATTACTGGGCGTAAAGCGCACGTAGGCGGCTTTG

TAAGTTAGAGGTGAAAGCCCAGAGCTCAACTCTGGAATTGCCTTTAAGACTGCATCGCTC

GAATCCAGGAGAGGTGAGTGGAATTCCGAGTGTAGAGGTGAAATTCGTAGATATTCGGAA

GAACACCAGTGGCGAAGGCGGCTCACTGGACTGGTATTGACGCTGAGGTGCGAAAGCGTG

GGGAGCAAAC

>ASV76 SS|1.0000|AY140238_S000397413;k:Bacteria,p:"Proteobacteria",c:Alphaproteobacteria,o:Rhodospirillales,f:Acetobacteraceae

TACGAAGGGGGCTAGCGTTGCTCGGAATGACTGGGCGTAAAGGGCGCGTAGGCGGATGTC

TTAGTCAGGCGTGAAATTCCTGGGCTTAACCTGGGGGCTGCGTTTGATACGGGATGTCTA

GAGTTTGGCAGAGGGTCGTGGAATTCCCAGTGTAGAGGTGAAATTCGTAGATATTGGGAA

GAACACCGGTGGCGAAGGCGGCGACCTGGTCCTTGACTGACGCTGAGGCGCGAAAGCGTG

GGGAGCAAAC

>ASV77 SS|1.0000|Z37138_S000001649;k:Bacteria,p:"Actinobacteria",c:Actinobacteria,o:Actinomycetales

TACGTAGGGTGCAAGCGTTGTCCGGAATTACTGGGCGTAAAGAGCTCGTAGGCGGTGTGT

CGCGTCGTCTGTGAAATCCCGTGGCTCAACCACGGGCTTGCAGGCGATACGGGCAGACTT

GAGTATTGCAGGGGAGACTGGAATTCCTGGTGTAGCGGTGAAATGCGCAGATATCAGGAG

GAACACCGGTGGCGAAGGCGGGTCTCTGGGCAAATACTGACGCTGAGGAGCGAAAGCATG

GGTAGCAAAC

>ASV78 SS|1.0000|KC213491_S003715425;k:Bacteria,p:"Bacteroidetes",c:Cytophagia,o:Cytophagales,f:Cytophagaceae,g:Hymenobacter

TACGGAGGGTGCGAGCGTTGTCCGGATTTATTGGGTTTAAAGGGTGCGTAGGCGGCTTTT

TAAGTCTGGGGTGAAAGCCCGCTGCTCAACAGCGGAACGGCCCTGGATACTGGGGAGCTT

GAATACAGTGGAGGTTGGCGGAATGGACGGAGTAGCGGTGAAATGCATAGATACCGTCCA

GAACCCCGATTGCGAAGGCAGCTGACTACACTGGTATTGACGCTGAGGCACGACAGCGTG

GGGAGCGAAC

>ASV79 SS|1.0000|JN090860_S002916046;k:Bacteria,p:"Bacteroidetes",c:Cytophagia,o:Cytophagales,f:Cytophagaceae,g:Hymenobacter

TACGGAGGGTGCGAGCGTTGTCCGGATTTATTGGGTTTAAAGGGTGCGTAGGCGGCCGCG

TAAGTCTGGGGTGAAAGCCCGTTGCTCAACAACGGAACTGCCCTGGAAACTGCGCGGCTT

GAGTCCAGACGAGGTTGGCGGAATGGGCGGTGTAGCGGTGAAATGCATAGATACCGTCCA

GAACACCGATTGCGAAGGCAGCTGACTAGGCTGGTACTGACGCTGAGGCACGAAAGCGTG

GGGAGCGAAC

>ASV80 SS|1.0000|AF166114_S000498654;k:Bacteria

TACGGAGGATGCAAGCGTTGTCCGGAATCATTGGGCGTAAAGGGTCCTGAGGTGGTTTGC

TTAGTCCAGTGTGAAAGTTCAGGGCTTACCCTTGAAAATGCGGTGGAAACTAGCAGGCTA

GAGTACGGTGGGGGCAGAGGGAATTCCCGATGTAGCGGTGAAATGCATAGATATCGGGAA

GAACACCGATGGCGAAGGCACTCTGCTGGGCCGAAACTGACACTGAAAGACGAAAGCCAG

GGGAGCGAAT

>ASV81 GS|0.0|None;No hit

TACGTAGAAGACTAGTGTTATTCATCTTTAATAGGTTTAAAGGGTACCTAGACGGTAAAT

CAAGCCCATATGGGGACTACTTTACTAGAGTTACTTATGAGGGGGTATTAAAGTACTGCT

GGTGTAGAGATGAAATTCTGTCATACCTCTTTTCGCGGGAAAAATAATGGCACAGGTATA

GGCGAAAGCATCCCCTTATGTGATAACTGACGTTGAAGGACGAAGGCTTTGTGTCGCGAA

CAGG

>ASV82 GS|79.6|EU046270_S000903110;k:Bacteria,p:Firmicutes,c:Bacilli,o:Bacillales,f:Paenibacillaceae_1,g:Saccharibacillus;

TACGTAGGTGGCAAGCGTTGTCCGGATTTACTGGGCGTAAAGGGCAAGCAGGCGGACTGT

TAAGTAGGAAGTGAAAGGTCGGAGCTCAACTCCAACATTGCTTCCTATACTGGCAGTCTT

GAGTCTCGGAGAGGAAAGCGGAACGATACGTGTAGCGGTGAAATGCGTTGATATGTATCG

GAACACCAATGGCGAAGGCAGCTTTCTGGACGAGAACTGACGCTCATTTGCGAAAGCCGA

GGTAGCGAAC

>ASV83 GS|100.0|Z93440_S000381585;k:Bacteria,p:"Proteobacteria",c:Gammaproteobacteria,o:Pseudomonadales,f:Moraxellaceae,g:Acinetobacter;

TACAGAGGGTGCGAGCGTTAATCGGATTTACTGGGCGTAAAGCGTGCGTAGGCGGCTTTT

TAAGTCGGATGTGAAATCCCTGAGCTTAACTTAGGAATTGCATTCGATACTGGGAAGCTA

GAGTATGGGAGAGGATGGTAGAATTCCAGGTGTAGCGGTGAAATGCGTAGAGATCTGGAG

GAATACCGATGGCGAAGGCAGCCATCTGGCCTAATACTGACGCTGAGGTACGAAAGCATG

GGGAGCAAAC

>ASV84 SS|1.0000|JN090860_S002916046;k:Bacteria,p:"Bacteroidetes",c:Cytophagia,o:Cytophagales,f:Cytophagaceae,g:Hymenobacter

TACGGAGGGTGCGAGCGTTGTCCGGATTTATTGGGTTTAAAGGGTGCGTAGGCGGCCGCG

TAAGTCCGGGGTGAAAGCCCGTTGCTCAACAACGGAACGGCCCTGGAAACTGCGCGGCTT

GAGTCCAGGCGAGGTCGGCGGAATGGGCGGTGTAGCGGTGAAATGCATAGATACCGTCCA

GAACCCCGATTGCGAAGGCAGCTGACTAGGCTGGTACTGACGCTGAGGCACGAAAGCGTG

GGGAGCGAAC

>ASV85 GS|0.0|None;No hit

TACGTAGAAGACAAGTGTTATTCATCTTTAACAGGTTTAAAGGGTACCTAGACGGAAAAT

CAAGCCATAGTAGGGACTAATTTTCTAGAGTTTTATGTGTGAAGATCGAATTACCTGAAG

AGCAATAAAATGCATTGACACAGGGAAGACGGGTAGCAGCGAAGGCAATCTTCTATGTAA

AAACTGACGTTGAGGGACGAAGCCTTGGGGAGCGAGAAGG

>ASV86 GS|0.0|None;No hit

TACGGGGGGGGCGAGCGTTATTCGAAATGATTGGGCGTAAAGGGCACGTAGACGGTTTTT

TGAGTTGACATGCTTGAGGTCGTAGAAAGAATACAAAATAAATATTCTTTCGTACGTCTA

AAAGATTAAAATGGAGTGTGGATTTTCACCCTGTATTCTTTGCTTGACAAAAGAAAAGGT

ATTCGGTTTGCTTTACACTTGGGAAAAAGGCCAAGGCTCAACCATGGTGTTTCCCGCTAT

ACTATAAAAC

>ASV87 SS|1.0000|HM032897_S002167665;k:Bacteria,p:"Bacteroidetes",c:Cytophagia,o:Cytophagales,f:Cytophagaceae,g:Hymenobacter

TACGGAGGGTGCGAGCGTTGTCCGGATTTATTGGGTTTAAAGGGTGCGTAGGCGGCCGTT

TAAGTCCGGGGTGAAAGCCCGCTGCTCAACAGCGGAACTGCCCTGGATACTGGATGGCTT

GAATACAGTGGAGGTTGGCGGAATGGACCGAGTAGCGGTGAAATGCATAGATACGGTCCA

GAACCCCGATTGCGAAGGCAGCTGACTACACTGGTATTGACGCTGAGGCACGACAGCGTG

GGGAGCGAAC

>ASV88 SS|1.0000|JN695632_S002960974;k:Bacteria,p:"Bacteroidetes",c:Sphingobacteriia,o:"Sphingobacteriales",f:Sphingobacteriaceae

TACGGAGGATCCGAGCGTTATCCGGATTTATTGGGTTTAAAGGGTGCGTAGGCGGCCTGT

TAAGTCAGGGGTGAAATTTTCCGGCTCAACCGGGGACTTGCCTTTGATACTGACGGGCTT

GAATGCAGCTGAGGTAGGCGGAATGTGACAAGTAGCGGTGAAATGCATAGATATGTCACA

GAACACCGATTGCGAAGGCAGCTTACCAAAGTGCGATTGACGCTGAGGCACGAAAGCGTG

GGGATCAAAC

>ASV89 SS|1.0000|AB778531_U010573768;k:Bacteria,p:"Proteobacteria",c:Alphaproteobacteria,o:Rhodospirillales,f:Acetobacteraceae

TACGAAGGGGGCTAGCGTTGCTCGGAATGACTGGGCGTAAAGGGCGCGTAGGCGGTTTGT

ACAGTCGGATGTGAAATTCCTGGGCTTAACCTGGGGGCTGCATTCGATACGTGCAGGCTT

GAGTGTGGAAGAGGGTTGTGGAATTCCCAGTGTAGAGGTGAAATTCGTAGATATTGGGAA

GAACACCGGTGGCGAAGGCGGCAACCTGGTCCATAACTGACGCTGAGGCGCGAAAGCGTG

GGGAGCAAAC

>ASV90 GS|100.0|HE599560_S003258029;k:Bacteria,p:"Actinobacteria",c:Actinobacteria,o:Actinomycetales,f:Nakamurellaceae,g:Nakamurella;

TACGTAGGGTGCAAGCGTTGTCCGGAATTATTGGGCGTAAAGAGCTCGTAGGCGGTCTGT

CGCGTCGAATGTGAAAATCCGAGGCTCAACCTCGGACCTGCATTCGATACGGGCAGACTA

GAGTTCGGTAGGGGAGTCTGGAATTCCTGGTGTAGCGGTGAAATGCGCAGATATCAGGAG

GAACACCGGTGGCGAAGGCGGGACTCTGGGCCGATACTGACGCTGAGGAGCGAAAGCGTG

GGGAGCAAAC

>ASV91 GS|0.0|None;No hit

TACGGGGGGGGCAAGCGTTATTCGAAATGATTGGGCGTAAAGGGCACGTAGACGGTTTTA

TGAGTTTTCATTTTTAAAAGTACACAAAATGGCTTTTAAAAATTATAATGGAGTGTGTTT

TCTTTTATTGCAATTTTTTCTTTCTTTCCTTTTAAAACAGAAAAGAAAGTTAAAGATTCC

GATTACACTTGGGAAAAAGGCCAAGGCCTAACCATGGTGTTCCCACTATACTATAAAACT

AGAGTAAGTT

>ASV92 SS|0.8300|KM044053_S004224125;k:Bacteria,p:"Actinobacteria",c:Actinobacteria,o:Actinomycetales,f:Nocardiaceae

TACGTAGGGTGCGAGCGTTGTCCGGAATTACTGGGCGTAAAGAGCTCGTAGGCGGTTTGT

CACGTCGGCTGTGAAATCCCATCGCTCAACGGTGGGCTTGCAGTCGATACGGGCTGACTT

GAGTACTGCAGGGGAGACTGGAATTCCTGGTGTAGCGGTGAAATGCGCAGATATCAGGAG

GAACACCGGTGGCGAAGGCGGGTCTCTGGGCAGTAACTGACGCTGAGGAGCGAAAGCGTG

GGTAGCAAAC

>ASV93 SS|1.0000|JN090860_S002916046;k:Bacteria,p:"Bacteroidetes",c:Cytophagia,o:Cytophagales,f:Cytophagaceae,g:Hymenobacter

TACGGAGGGTGCGAGCGTTGTCCGGATTTATTGGGTTTAAAGGGTGCGTAGGCGGCCGCG

TAAGTCTGGGGTGAAAGCCCGTTGCTCAACAACGGAACTGCCCTGGAAACTGCGCGGCTT

GAGTCCAGACGAGGTTGGCGGAATGGGCGGTGTAGCGGTGAAATGCATAGATACCGTCCA

GAACCCCGATTGCGAAGGCAGCTGACTAGGCTGGTACTGACGCTGAGGCACGAAAGCGTG

GGGAGCGAAC

>ASV94 SS|1.0000|EU861928_S001148233;k:Bacteria,p:"Armatimonadetes",c:Armatimonadia,o:Armatimonadales,f:Armatimonadaceae,g:Armatimonas/Armatimonadetes_gp1

TACGTAGGGGGCGAGCGTTGTCCGAAGTTACTGGGCGTAAAGAGCGCGTAGGCGGGTTCT

TAAGTGAGGGGTGAAAGTCCGAGGCTCAACCTCGGAACTGCCTTTCATACTGGGAACCTT

GAGTATGGGAGAGGCGAGTGGAATGGTCGGTGTAGCGGTGAAATGCGTAGATATCGATCG

GAACACCCATGGCGAAGGCAGCTCGCTGGCCTATAACTGACGCTGAGGCGCGAAAGCGTG

GGGAGCAAAC

>ASV95 SS|1.0000|D86513_S000011943;k:Bacteria,p:"Proteobacteria",c:Alphaproteobacteria,o:Rhodospirillales,f:Acetobacteraceae

TACGAAGGGGGCTAGCGTTGCTCGGAATGACTGGGCGTAAAGGGCGCGTAGGCGGATCGG

ATAGTCAGGCGTGAAATTCCTGGGCTCAACCTGGGGGCTGCGTTTGATACGTTTGGTCTA

GAGTGGGGAAGAGGGTTGTGGAATTCCCAGTGTAGAGGTGAAATTCGTAGATATTGGGAA

GAACACCGGTGGCGAAGGCGGCAACCTGGTCCTTGACTGACGCTGAGGCGCGAAAGCGTG

GGGAGCAAAC

>ASV96 SS|1.0000|AJ009456_S000115949;k:Bacteria

TACGTAGGTGGCAAGCGTTGTCCGGATTTACTGGGCGTAAAGCGCGCGCAGGCGGACTGT

TAAGTAGAAAGTGAAAGGTTGGAGCTCAACTCCAACATTGCTTCCTATACTGGCAGTCTT

GAGTCCCGGAGGGGAGAGCGGAACAATACGTGTAGCGGTGAAATGCGTTGATATGTATTG

GAACACCAATGGCGAAGGCAGCTCTCTGGACGGGAACTGACGCTCAGGCGCGAAAGCCGA

GGTAGCGAAC

>ASV97 GS|98.0|AB267478_S000721192;k:Bacteria,p:"Bacteroidetes",c:Sphingobacteriia,o:"Sphingobacteriales",f:Chitinophagaceae,g:Segetibacter;

TACGGAGGGTGCAAGCGTTATCCGGATTCACTGGGTTTAAAGGGTGCGTAGGTGGGGATG

TAAGTCAGTGGTGAAATCTCCGTGCTTAACATGGAAACTGCCATTGATACTATGTTTCTT

GAATTTTCTGGAGGTCAGCGGAATATGTCATGTAGCGGTGAAATGCTTAGATATGACATA

GAACACCAATTGCGAAGGCAGCTGGCTACAGGGATATTGACACTGAGGCACGAAAGCGTG

GGGATCAAAC

>ASV98 GS|99.6|EF635408_S001095322;k:Bacteria,p:"Deinococcus-Thermus",c:Deinococci,o:Deinococcales,f:Deinococcaceae,g:Deinococcus;

TACGGAGGGTGCAAGCGTTACCCGGAATCACTGGGCGTAAAGGGCGTGTAGGCGGCCCGC

CAAGTCTGACTTTAAAGACCGAAGCTCAACTTCGGGCATGGGTTGGAAACTGACGGGCTA

GACGGATGGAGAGGTCACTGGAATTCCTGGTGTAGCGGTGGAATGCGTAGATACCAGGAG

GAACACCAACGGCGAAGGCAGGTGACTGGACATTTAGTGACGCTGAGGCGCGAAAGTGTG

GGGAGCGAAC

>ASV99 SS|1.0000|EF635408_S001095322;k:Bacteria,p:"Deinococcus-Thermus",c:Deinococci,o:Deinococcales,f:Deinococcaceae,g:Deinococcus

TACGGAGGGTGCAAGCGTTACCCGGAATCACTGGGCGTAAAGGGCGTGTAGGCGGTTTGC

CAAGTCTGACTTTAAAGACCGAAGCTCAACTTCGGGCATGGGTTGGAGACTGGCAGACTA

GACGGATGGAGAGGTCACTGGAATTCCTGGTGTAGCGGTGGAATGCGTAGATACCAGGAG

GAACACCAACGGCGAAGGCAGGTGACTGGACATTTAGTGACGCTGAGGCGCGAAAGTGTG

GGGAGCAAAC

>ASV100 GS|97.2|AB267478_S000721192;k:Bacteria,p:"Bacteroidetes",c:Sphingobacteriia,o:"Sphingobacteriales",f:Chitinophagaceae,g:Segetibacter;

TACGGAGGGTGCAAGCGTTATCCGGATTTACTGGGTTTAAAGGGTGCGTAGGTGGGAATG

TAAGTCAGTGGTGAAATCTCCGTGCTTAACATGGAAACTGCCATTGATACTATGTTTCTT

GAATTTTCTGGAGGTTAGCGGAATATGTCATGTAGCGGTGAAATGCTTAGATATGACATA

GAACACCAATTGCGAAGGCAGCTGGCTACAGGAAAATTGACACTGATGCACGAAAGCGTG

GGGATCAAAC

>ASV101 SS|1.0000|HM032898_S002167666;k:Bacteria,p:"Bacteroidetes",c:Cytophagia,o:Cytophagales,f:Cytophagaceae,g:Hymenobacter

TACGGAGGGTGCGAGCGTTGTCCGGATTTATTGGGTTTAAAGGGTGCGTAGGCGGCTTTT

TAAGTCTGGGGTGAAAGCCCGCTGCTCAACAGCGGAACTGCCCTGGATACTGGAGAGCTT

GAGTACAGACGAGGTTGGCGGAATGGACGGAGTAGCGGTGAAATGCATAGATACCGTCCA

GAACCCCGATTGCGAAGGCAGCTGACTAGGCTGTTACTGACGCTGAGGCACGAAAGCGTG

GGGAGCGAAC

>ASV102 GS|0.0|None;No hit

TACGGGGGGGGCAAGCGTTATTCGAAATGATTGGGCGTAAAGAGCACGTAGACGGTTTTT

ATAGTAGACATAGTGGCAATATCTCTGTATTAAACATCTTTCACATTTCAAACATCTATG

GATGTCAGAGAAATGTGAAAGATGTTTACTTCTATATATTTGCATACTTTTGATGGAGTA

TCTCTTTGTTCATGTATAAACATGAACAAATACTCGGGAAAAAGACCAAGGCTCAACCAT

GGTGTTTCCC

>ASV103 GS|97.6|AJ292684_S000017061;k:Bacteria,p:candidate_division_WPS-2,g:WPS-2_genera_incertae_sedis;

GACGTAGGGGGCAAGCGTTATTCGGAATTATTGGGCGTAAAGCGCTCGTAGGCGGGACAG

GAAGTCCGTGAAGAAAGACCTGGGCTCAACTCAGGGAACGGCACGGATACTCTTGTTCTT

GAGGCAATCAGAGGGTGATGGAATTCCCGGTGTAGCGGTGAAATGCGTAGATATCGGGAG

GAACACCAGTGGCGAAGGCGATCACCTGGGGTTGTTCTGACGCTGAGGAGCGAAAGCTAG

GGGAGCAAAC

>ASV104 SS|0.9200|AY902680_S000576842;k:Bacteria,p:"Proteobacteria",c:Alphaproteobacteria,o:Sphingomonadales,f:Sphingomonadaceae

TACGGAGGGGGCTAGCGTTGTTCGGAATTACTGGGCGTAAAGCGTTCGTAGGCGGCTTGC

CAAGTCAGAGGTGAAATCCCACGACTCAATCGTGGAACTGCCTTTGAGACTGGTAGGCTT

GAACACGGGAGAGGTGAGTGGAATTCCGAGTGTAGAGGTGAAATTCGTAGATATTCGGAA

GAACACCAGTGGCGAAGGCGGCTCACTGGACCGTTGTTGACGCTGAGGAACGAAAGCGTG

GGGAGCAAAC

>ASV105 SS|1.0000|JX949238_S003747851;k:Bacteria,p:"Bacteroidetes",c:Sphingobacteriia,o:"Sphingobacteriales",f:Sphingobacteriaceae

TACGGAGGATCCAAGCGTTATCCGGATTTATTGGGTTTAAAGGGTGCGTAGGCGGCCTGT

TAAGTCAGGGGTGAAATTTTTCGGCTCAACCGGAAACTTGCCTTTGATACTGACGGGCTT

GAATGCAGCTGAGGTAGGCGGAATGTGACAAGTAGCGGTGAAATGCATAGATATGTCACA

GAACACCAATTGCGAAGGCAGCTTACTAAAGTGTGATTGACGCTGAGGCACGAAAGCGTG

GGGATCAAAC

>ASV106 SS|1.0000|JF999998_S002914924;k:Bacteria,p:"Bacteroidetes",c:Sphingobacteriia,o:"Sphingobacteriales",f:Sphingobacteriaceae

TACGGAGGATCCAAGCGTTATCCGGATTTATTGGGTTTAAAGGGTGCGTAGGCGGCCTGT

TAAGTCAGGGGTGAAAGACGGTGGCTCAACCATCGCAGTGCCTTTGATACTGACGGGCTT

GAATGCAGTTGAGGTAGGCGGAATGTGGCAAGTAGCGGTGAAATGCATAGATATGCCACA

GAACACCAATTGCGAAGGCAGCTTACCAAAGTGCGATTGACGCTGAGGCACGAAAGCGTG

GGGATCAAAC

>ASV107 GS|98.0|KF483876_S003921123;k:Bacteria,p:"Bacteroidetes",c:Sphingobacteriia,o:"Sphingobacteriales",f:Sphingobacteriaceae,g:Mucilaginibacter;

TACGGAGGATCCAAGCGTTATCCGGATTTATTGGGTTTAAAGGGTGCGTAGGTGGCCTGT

TAAGTCAGGGGTGAAAGACGGTGGCTCAACCATCGCAGTGCCTTTGATACTGACGGGCTT

GAATACACTAGAGGTAGGCGGAATGTGACAAGTAGCGGTGAAATGCATAGATATGTCACA

GAACACCGATTGCGAAGGCAGCTTACTATGGTGTTATTGACACTGAGGCACGAAAGCGTG

GGGATCAAAC

>ASV108 SS|1.0000|JX949238_S003747851;k:Bacteria,p:"Bacteroidetes",c:Sphingobacteriia,o:"Sphingobacteriales",f:Sphingobacteriaceae

TACGGAGGATCCAAGCGTTATCCGGATTTATTGGGTTTAAAGGGTGCGTAGGCGGCCTGT

TAAGTCAGGGGTGAAATTTTCCGGCTCAACCGGGACATTGCCTTTGATACTGACGGGCTT

GAATGCAGCTGAGGTAAGCGGAATGTGACAAGTAGCGGTGAAATGCATAGATATGTCACA

GAACACCAATTGCGAAGGCAGCTTACTAAAGTGTGATTGACGCTGAGGCACGAAAGCGTG

GGGATCAAAC

>ASV109 GS|0.0|None;No hit

TTCCAGCTCCAATAGCGTATATTAAAGTTGTTGCAGTTAAAAAGCTCGTAGTTGAACCTT

GGGCCTGGCTGGCCGGTCCGCCTCACCGCGTGCACCGGTCCGGCCGGGCCTTTCCTTCTG

GGGATCCACATGCCCTTCACTGGGTGTGCCGGGGAACCAGGACTTTTACTTTGAAAAAAT

TAGAGTGTTCAAAGCAGGCCTATGCTCGAATACATTAGCATGGAATAATAGAATAGGACG

TGTGGTTCTA

>ASV110 SS|1.0000|HM032897_S002167665;k:Bacteria,p:"Bacteroidetes",c:Cytophagia,o:Cytophagales,f:Cytophagaceae,g:Hymenobacter

TACGGAGGGTGCGAGCGTTGTCCGGATTTATTGGGTTTAAAGGGTGCGTAGGCGGCCGTT

TAAGTCTGGGGTGAAAGCCCGTTGCTCAACAGCGGAACTGCCCTGGATACTGGATGGCTT

GAATACAGTGGAGGTTGGCGGAATGGACCGAGTAGCGGTGAAATGCATAGATACGGTCCA

GAACCCCGATTGCGAAGGCAGCTGACTACACTGGTATTGACGCTGAGGCACGACAGCGTG

GGGAGCGAAC

>ASV111 SS|1.0000|AB739062_S003286967;k:Bacteria,p:"Proteobacteria",c:Alphaproteobacteria,o:Rhodospirillales,f:Acetobacteraceae

TACGAAGGGGGCTAGCGTTGCTCGGAATGACTGGGCGTAAAGGGCGCGTAGGCGGAGATA

TCAGTCAGATGTGAAATTCCTGGGCTTAACCTGGGGGCTGCATTTGAGACGGTATGTCTA

GAGTGTGAAAGAGGGTCGTGGAATTCCCAGTGTAGAGGTGAAATTCGTAGATATTGGGAA

GAACACCGGTGGCGAAGGCGGCGACCTGGTTCATAACTGACGCTGAGGCGCGAAAGCGTG

GGGAGCAAAC

>ASV112 SS|1.0000|GQ342559_S003611109;k:Bacteria,p:"Bacteroidetes",c:Cytophagia,o:Cytophagales,f:Cytophagaceae,g:Spirosoma

TACGGAGGGTGCAAGCGTTGTCCGGATTTATTGGGTTTAAAGGGTGCGTAGGTGGTTTCT

TAAGTCTGGTTTGAAAGCAGGCGGCTCAACCGTGTGATGTGGCTGGAAACTGGGGAACTT

GAATGGGATGGCGGTAGCCGGAACGGGTCATGTAGCGGTGAAATGCATAGATATGACCCA

GAACACCGATTGCGAAGGCAGGCTACTAGGTCCTGATTGACACTGAGGCACGAGAGCATG

GGGAGCCAAC

>ASV113 SS|1.0000|AM231587_S000650722;k:Bacteria,p:"Proteobacteria",c:Alphaproteobacteria,o:Rhodospirillales,f:Acetobacteraceae

TACGAAGGGGGCTAGCGTTGCTCGGAATGACTGGGCGTAAAGGGCGCGTAGGCGGCTTGG

TTAGTTAGACGTGAAATTCCTGGGCTCAACCTGGGGGCTGCGTTTGATACAGCTAGGCTA

GAGTGGGGAAGAGGGTTGTGGAATTCCCAGTGTAGAGGTGAAATTCGTAGATATTGGGAA

GAACACCGGTGGCGAAGGCGGCAACCTGGTCCTTGACTGACGCTGAGGCGCGAAAGCGTG

GGGAGCAAAC

>ASV114 SS|1.0000|EU861928_S001148233;k:Bacteria,p:"Armatimonadetes",c:Armatimonadia,o:Armatimonadales,f:Armatimonadaceae,g:Armatimonas/Armatimonadetes_gp1

TACGTAGGGGGCGAGCGTTGTCCGAAGTTACTGGGCGTAAAGAGCGCGTAGGCGGGTTCT

TAAGTGAGGGGTGAAAGTCCGGGGCTCAACCCCGGAACTGCCTTTCATACTGGGAACCTT

GAGTGTGGGAGAGGCGAGTGGAATGGTCGGTGTAGCGGTGAAATGCGTAGATATCGATCG

GAACACCCATGGCGAAGGCAGCTCGCTGGCCTATAACTGACGCTGAGGCGCGAAAGCGTG

GGGAGCAAAC

>ASV115 SS|1.0000|EU861876_S001148181;k:Bacteria,p:"Armatimonadetes",c:Armatimonadia,o:Armatimonadales,f:Armatimonadaceae,g:Armatimonas/Armatimonadetes_gp1

TACGTAGGGGGCCAGCGTTGTCCGAAGTTACTGGGCGTAAAGAGCGCGTAGGCGGGTCTT

TAAGTGAGGGGTGAAAGGTTCGGGCTCAACCCGGACACTGCCTTTCATACTGGGGGCCTT

GAGTATGGGAGAGGCGAGTGGAATTCTTGGTGTAGCGGTGAAATGCGTAGATATCAAGAG

GAACACCCATGGCGAAGGCAGCTCGCTGGCCTATAACTGACGCTGAGGCGCGAAAGCTGG

GGGAGCAAAC

>ASV116 SS|1.0000|EU861928_S001148233;k:Bacteria,p:"Armatimonadetes",c:Armatimonadia,o:Armatimonadales,f:Armatimonadaceae,g:Armatimonas/Armatimonadetes_gp1

TACGTAGGGGGCGAGCGTTGTCCGAAGTTACTGGGCGTAAAGAGCGCGTAGGCGGGTTTT

TAAGTGAGGGGTGAAATTCCGAGGCTTAACCTCGGAACTGCCTTTCATACTGGGAACCTT

GAGTGTGGGAGAGGCGAGTGGAATGGTCGGTGTAGCGGTGAAATGCGTAGATATCGATCG

GAACACCCATGGCGAAGGCAGCTCGCTGGCCTATAACTGACGCTGAGGCGCGAAAGCGTG

GGGAGCAAAC

>ASV117 SS|0.8000|AM231587_S000650722;k:Bacteria,p:"Proteobacteria",c:Alphaproteobacteria,o:Rhodospirillales,f:Acetobacteraceae,g:Acidisoma

TACGAAGGGGGCTAGCGTTGCTCGGAATGACTGGGCGTAAAGGGCGCGTAGGCGGATTGG

TTAGTCAGACGTGAAATTCCTGGGCTCAACCTGGGGGCTGCGTTTGAGACGGCTAATCTA

GAGTTTGGAAGAGGGTCGTGGAATTCCCAGTGTAGAGGTGAAATTCGTAGATATTGGGAA

GAACACCGGTGGCGAAGGCGGCGACCTGGTCCTTGACTGACGCTGAGGCGCGAAAGCGTG

GGGAGCAAAC

>ASV118 GS|0.0|None;No hit

TACAAGGAAGACTAGTGTTATTCATCTTTAATAGGTTTAAAGGGTACCTAGACGGTATTT

TTAGCCCAAAACAGGGTACGGATTTACTAGAGTTTTATGTGAGAAGGGGAGTACTTATGG

AGTAGGGATGAAATTCTTTTATACTATGGGGTAACTGGTAGAGGCGAAAGCAACCTTTTA

TGTAGAAACTGACGTTGAGGGACGAAGGCCTGGGTAGCAAACAGG

>ASV119 GS|99.6|Z93440_S000381585;k:Bacteria,p:"Proteobacteria",c:Gammaproteobacteria,o:Pseudomonadales,f:Moraxellaceae,g:Acinetobacter;

TACAGAGGGTGCGAGCGTTAATCGGATTTACTGGGCGTAAAGCGTGCGTAGGCGGCTTCT

TAAGTCGGATGTGAAATCCCTGAGCTTAACTTAGGAATTGCATTCGATACTGGGAAGCTA

GAGTATGGGAGAGGATGGTAGAATTCCAGGTGTAGCGGTGAAATGCGTAGAGATCTGGAG

GAATACCGATGGCGAAGGCAGCCATCTGGCCTAATACTGACGCTGAGGTACGAAAGCATG

GGGAGCAAAC

>ASV120 SS|1.0000|EF516412_S000840854;k:Bacteria,p:"Armatimonadetes",c:Armatimonadia,o:Armatimonadales,f:Armatimonadaceae,g:Armatimonas/Armatimonadetes_gp1

TACGTAGGGGGCCAGCGTTGTTCGAAGTTACTGGGCGTAAAGAGCGCGTAGGCGGACTTT

TAAGTGAGGGGTGAAAGGTTCAGGGCTTAACCCGGACACTGCCTTTTATACTGGGAGTCT

TGAGTGTTGGAGAGGCGAGTGGAATGGTCGGTGTAGCGGTGAAATGCGTAGATATCGATC

GGAACACCCATGGCGAAGGCAGCTCGCTGGCCAACAACTGACGCTGAGGCGCGAAAGCGT

GGGGAGCAAA

>ASV121 SS|0.9900|AJ292581_S000022859;k:Bacteria,p:"Acidobacteria",c:Acidobacteria_Gp1

TACGAGGGGGGCAAGCGTTGTTCGGAATTATTGGGCGTAAAGGGTGCGTAGGCGGCCCCG

CAAGTCTCGTGTGAAATCCTCAAGCTCAACTTGAGGTCTGCACGGGAAACTGCTGGGCTG

GAGTATGGGAGAGGTGAGTGGAATTCCTGGTGTAGCGGTGAAATGCGTAGATATCAGGAG

GAACACCTGTGGCGAAAGCGGCTCACTGGACCATAACTGACGCTGAGGCACGAAAGCTAG

GGGAGCAAAC

>ASV122 SS|0.9900|AB739062_S003286967;k:Bacteria,p:"Proteobacteria",c:Alphaproteobacteria,o:Rhodospirillales,f:Acetobacteraceae

TACGAAGGGGGCTAGCGTTGCTCGGAATGACTGGGCGTAAAGGGCGCGTAGGCGGAGATA

TCAGTCAGATGTGAAATTCCTGGGCTTAACCTGGGGGCTGCATTTGAGACGGTATTTCTA

GAGTGTGAAAGAGGGTCGTGGAATTCCCAGTGTAGAGGTGAAATTCGTAGATATTGGGAA

GAACACCGGTGGCGAAGGCGGCGACCTGGTTCATAACTGACGCTGAGGCGCGAAAGCGTG

GGGAGCAAAC

>ASV123 SS|1.0000|AY140238_S000397413;k:Bacteria,p:"Proteobacteria",c:Alphaproteobacteria,o:Rhodospirillales,f:Acetobacteraceae

TACGAAGGGGGCTAGCGTTGCTCGGAATGACTGGGCGTAAAGGGCGCGTAGGCGGTTTGC

ACAGTCGGGTGTGAAATTCCTGGGCTTAACCTGGGGACTGCATTCGATACGTGTGGGCTT

GAGTGTGGAAGAGGGTTGTGGAATTCCCAGTGTAGAGGTGAAATTCGTAGATATTGGGAA

GAACACCGGTGGCGAAGGCGGCAACCTGGTCCATGACTGACGCTGAGGCGCGAAAGCGTG

GGGAGCAAAC

>ASV124 SS|1.0000|FR666706_S002222816;k:Bacteria,p:"Acidobacteria",c:Acidobacteria_Gp1

TACGAGGGGGGCAAGCGTTGTTCGGAATTATTGGGCGTAAAGGGTGCGTAGGCGGTTTGA

TAAGTCTTATGTGAAATCTATGGGCTCAACCCATAGTCTGCATAGGAAACTGTCGGGCTT

GAGGATGGGAGAGGTGAGTGGAATTTCCGGTGTAGCGGTGAAATGCGTAGATATCGGAAG

GAACACCTGTGGCGAAAGCGGCTCACTGGACCATTTCTGACGCTGATGCACGAAAGCTAG

GGGAGCAAAC

>ASV125 SS|0.9900|Y18947_S000088565;k:Bacteria,p:"Proteobacteria",c:Alphaproteobacteria,o:Rhizobiales

TACGAAGGGGGCTAGCGTTGTTCGGAATCACTGGGCGTAAAGGGTGCGTAGGCGGATTTT

TAAGTCAGGGGTGAAATCCCGAGGCTCAACCTCGGAACTGCCTTTGATACTGGGGATCTT

GAGTCCGGAAGAGGTGAGTGGAACTGCGAGTGTAGAGGTGAAATTCGTAGATATTCGCAA

GAACACCAGTGGCGAAGGCGGCTCACTGGTCCGGAACTGACGCTGAGGCACGACAGCGTG

GGGAGCAAAC

>ASV126 SS|1.0000|AY140238_S000397413;k:Bacteria,p:"Proteobacteria",c:Alphaproteobacteria,o:Rhodospirillales,f:Acetobacteraceae

TACGAAGGGGGCTAGCGTTGCTCGGAATGACTGGGCGTAAAGGGCGCGTAGGCGGATTTG

TCAGTTGGGCGTGAAATTCCTGGGCTTAACCTGGGGGCTGCGTTCGAGACGGCGGGTCTA

GAGTTTGGAAGAGGGTCGTGGAATTCCCAGTGTAGAGGTGAAATTCGTAGATATTGGGAA

GAACACCGGTGGCGAAGGCGGCGACCTGGTCCTGGACTGACGCTGAGGCGCGAAAGCGTG

GGGAGCAAAC

>ASV127 SS|1.0000|EU861928_S001148233;k:Bacteria,p:"Armatimonadetes",c:Armatimonadia,o:Armatimonadales,f:Armatimonadaceae,g:Armatimonas/Armatimonadetes_gp1

TACGTAGGGGGCGAGCGTTGTTCGAAGTTACTGGGCGTAAAGAGCGCGTAGGCGGGTTCT

TAAGTGAGAGGTGAAATTCCGGGGCTCAACCCCGGAACTGCCTTTCATACTGGGAACCTT

GAGTGTGGGAGAGGCGAGTGGAATGGTCGGTGTAGCGGTGAAATGCGTAGATATCGATCG

GAACACCCATGGCGAAGGCAGCTCGCTGGCCTATAACTGACGCTGAGGCGCGAAAGCGTG

GGGAGCAAAC

>ASV128 GS|97.2|JQ346802_S003290742;k:Bacteria,p:"Actinobacteria",c:Actinobacteria,o:Actinomycetales,f:Cryptosporangiaceae,g:Jatrophihabitans;

TACGTAGGGTGCAAGCGTTGTCCGGAATTATTGGGCGTAAAGAGCTCGTAGGCGGTTTGT

CGCGTCGGCTGTGAAAACATGGGGCTCAACCCTGTGCCTGCAGCCGATACGGGCAGACTA

GAATTCGGTAGGGGAGACTGGAATTCCTGGTGTAGCGGTGAAATGCGCAGATATCAGGAG

GAACACCGGTGGCGAAGGCGGGTCTCTGGGCCGATATTGACGCTGAGGAGCGAAAGCGTG

GGGAGCAAAC

>ASV129 GS|100.0|FR733685_S002304642;k:Bacteria,p:"Actinobacteria",c:Actinobacteria,o:Actinomycetales,f:Micromonosporaceae,g:Actinoplanes;

GACGTAGGGCGCGAGCGTTGTCCGGATTTATTGGGCGTAAAGAGCTCGTAGGCGGCTTGT

CGCGTCGACTGTGAAAACCCGCGGCTCAACCGCGGGCCTGCAGCCGATACGGGCAGGCTA

GAGTTCGGTAGGGGAGACTGGAATTCCTGGTGTAGCGGTGAAATGCGCAGATATCAGGAG

GAACACCGATGGCGAAGGCAGGTCTCTGGGCCGATACTGACGCTGAGGAGCGAAAGCGTG

GGGAGCGAAC

>ASV130 GS|100.0|AB018439_S000439484;k:Bacteria,p:"Proteobacteria",c:Alphaproteobacteria,o:Sphingomonadales,f:Sphingomonadaceae,g:Sphingomonas;

TACGGAGGGAGCTAGCGTTATTCGGAATTACTGGGCGTAAAGCGCACGTAGGCGGCTTTG

TAAGTTAGAGGTGAAAGCCTGGAGCTCAACTCCAGAATTGCCTTTAAGACTGCATCGCTT

GAATCCAGGAGAGGTGAGTGGAATTCCGAGTGTAGAGGTGAAATTCGTAGATATTCGGAA

GAACACCAGTGGCGAAGGCGGCTCACTGGACTGGTATTGACGCTGAGGTGCGAAAGCGTG

GGGAGCAAAC

>ASV131 GS|0.0|None;No hit

GTGTCAGCAGCCGCGGTGTGTACGGCAGCGACTGTACTAGCAGTCGGTGATGCGAGAGAG

GTAAGTCCAGCGTACTGTCGGGTGATGGCATTGTCTGCTATGTTCGGAAAGACTCTGCCT

ATGATGTTTGGCCGTCCAACGGACTTGACCTGAGAAGGCGTAGCTTCACCTTTCTCCTGG

GTGGTTGAGATGGATGGTAAAGATGTGGTAGTTGTAGATTGGTGGGCATGCGGCTGTGAG

TGTGCGGAGT

>ASV132 GS|98.0|KF437572_S004053197;k:Bacteria,p:"Proteobacteria",c:Alphaproteobacteria,o:Sphingomonadales,f:Sphingomonadaceae,g:Rhizorhabdus;

TACGGAGGGGGCTAGCGTTGTTCGGAATTACTGGGCGTAAAGCGTACGTAGGCGGTTTTG

TAAGTTAGAGGTGAAAGCCCGGAGCTCAACTTCGGAATTGCCTTTAAGACTGCATCACTT

GAACGTCGGAGAGGTGAGTGGAATTCCGAGTGTAGAGGTGAAATTCGTAGATATTCGGAA

GAACACCAGTGGCGAAGGCGGCTCACTGGACGACTGTTGACGCTGAGGTACGAAAGCGTG

GGGAGCAAAC

>ASV133 SS|1.0000|JN695632_S002960974;k:Bacteria,p:"Bacteroidetes",c:Sphingobacteriia,o:"Sphingobacteriales",f:Sphingobacteriaceae

TACGGAGGATCCGAGCGTTATCCGGATTTATTGGGTTTAAAGGGTGCGTAGGCGGCCTGT

TAAGTCAGGGGTGAAAGACGGTGGCTCAACCATCGCAGTGCCTTTGATACTGACGGGCTT

GAATGCAGTTGAGGTAGGCGGAATGTGGCAAGTAGCGGTGAAATGCATAGATATGCCACA

GAACACCAATTGCGAAGGCAGCTTACCAAAGTGCGATTGACGCTGAGGCACGAAAGCGTG

GGGATCAAAC

>ASV134 SS|1.0000|HM032898_S002167666;k:Bacteria,p:"Bacteroidetes",c:Cytophagia,o:Cytophagales,f:Cytophagaceae,g:Hymenobacter

TACGGAGGGTGCGAGCGTTGTCCGGATTTATTGGGTTTAAAGGGTGCGTAGGCGGCTTTT

TAAGTCTGGGGTGAAAGCCCGCTGCTCAACAGCGGAACTGCCCTGGATACTGGGAAGCTT

GAGTACAGACGAGGTTGGCGGAATGGACGGAGTAGCGGTGAAATGCATAGATACCGTCCA

GAACCCCGATTGCGAAGGCAGCTGACTAGGCTGTTACTGACGCTGAGGCACGAAAGCGTG

GGGAGCGAAC

>ASV135 SS|1.0000|GQ342559_S003611109;k:Bacteria,p:"Bacteroidetes",c:Cytophagia,o:Cytophagales,f:Cytophagaceae,g:Spirosoma

TACGGAGGGTGCGAGCGTTGTCCGGATTTATTGGGTTTAAAGGGTGCGTAGGTGGGGTTC

TAAGTCTGGTTTGAAAGCAGGCGGCTCAACCGTCTGATGTGGCTGGAAACTGGGGTTCTT

GAATGGGTTGGCGGTAGCCGGAACGGGTCATGTAGCGGTGAAATGCATAGATATGACCCA

GAACACCGATTGCGAAGGCAGGCTACTACGACTTGATTGACACTGAGGCACGAGAGCCGG

GGTAGCGAAC

>ASV136 SS|0.9200|D86513_S000011943;k:Bacteria,p:"Proteobacteria",c:Alphaproteobacteria,o:Rhodospirillales,f:Acetobacteraceae,g:Acidisoma

TACGAAGGGGGCTAGCGTTGCTCGGAATGACTGGGCGTAAAGGGCGCGTAGGCGGATTCT

ACAGTCAGGCGTGAAATTCCTGGGCTCAACCTGGGGGCTGCGTTTGAGACGTGGGGTCTT

GAGTGGGGAAGAGGGTCGTGGAATTCCCAGTGTAGAGGTGAAATTCGTAGATATTGGGAA

GAACACCGGTGGCGAAGGCGGCGACCTGGTCCTTTTACTGACGCTGAGGCGCGAAAGCGT

GGGGAGCAAA

>ASV137 GSL|99.2|AJ938026_S000539945;k:Bacteria,p:"Proteobacteria",c:Betaproteobacteria,o:Burkholderiales,f:Comamonadaceae

TACGTAGGGTGCAAGCGTTAATCGGAATTACTGGGCGTAAAGCGTGCGCAGGCGGTGATA

TAAGACAGATGTGAAATCCCCGGGCTCAACCTGGGAACTGCATTTGTGACTGTATCGCTG

GAGTGCGGCAGAGGGGGATGGAATTCCGCGTGTAGCAGTGAAATGCGTAGATATGCGGAG

GAACACCGATGGCGAAGGCAATCCCCTGGGCCTGCACTGACGCTCATGCACGAAAGCGTG

GGGAGCAAAC

>ASV138 SS|1.0000|EU861928_S001148233;k:Bacteria,p:"Armatimonadetes",c:Armatimonadia,o:Armatimonadales,f:Armatimonadaceae,g:Armatimonas/Armatimonadetes_gp1

TACGTAGGGGGCGAGCGTTGTCCGAAGTTACTGGGCGTAAAGAGCGCGTAGGCGGGTTCT

TAAGTGAGGGGTGAAATTCCGAGGCTTAACCTCGGAACTGCCTCTCATACTGGGAACCTT

GAGTGTGGGAGAGGCGAGTGGAATGGTCGGTGTAGCGGTGAAATGCGTAGATATCGATCG

GAACACCCATGGCGAAGGCAGCTCGCTGGCCTATAACTGACGCTGAGGCGCGAAAGCGTG

GGGAGCAAAC

>ASV139 GS|97.2|EF457481_S000834906;k:Bacteria,p:"Acidobacteria",c:Acidobacteria_Gp4,g:Gp4;

TACGTAGGGACCAAGCGTTGTTCGGATTTACTGGGCGTAAAGGGCGCGTAGGCGGCGTGT

CAAGTCAATTGTGAAATCTCCGAGCTTAACTCGGAACGGTCAATTGATACTGATGTGCTA

GAGTGCAGAAGGGGCAATCGGAATTCTTGGTGTAGCGGTGAAATGCGTAGATATCAAGAG

GAACACCTGAGGTGAAGACGGGTTGCTGGGCTGACACTGACGCTGAGGCGCGAAAGCTAG

GGTAGCAAAC

>ASV140 SS|1.0000|D86513_S000011943;k:Bacteria,p:"Proteobacteria",c:Alphaproteobacteria,o:Rhodospirillales,f:Acetobacteraceae

TACGAAGGGGGCTAGCGTTGCTCGGAATGACTGGGCGTAAAGGGCGCGTAGGCGGCATGG

ACAGTCAGGTGTGAAATTCCCGGGCTTAACCTGGGGACTGCATTTGATACGTTCGGGCTA

GAGTGTGGAAGAGGGTCGTGGAATTCCCAGTGTAGAGGTGAAATTCGTAGATATTGGGAA

GAACACCGGTGGCGAAGGCGGCGACCTGGTCCATTACTGACGCTGAGGCGCGAAAGCGTG

GGGAGCAAAC

>ASV141 GS|0.0|None;No hit

TACGGGGGGGGCGAGCGTTATTCGAAATGATTGGGCGTAAAGGGCACGTAGACGGTTTTT

TGAGTTGACATGCTTGAGGTCGTAGAAAGAATAGAAAATAAATATTCTTTCGTACGTCTA

AAAGATTAAAATGGAGTGTGGATTTTCACCCTGTATTCTTTGCTTGACAAAAGAAAAGGT

ATTCGGTTTGCTTTACACTTGGGAAAAAGGCCAAGGCTCAACCATGGTGTTTCCCGCTAT

ACTATAAAAC

>ASV142 SS|1.0000|HM032898_S002167666;k:Bacteria,p:"Bacteroidetes",c:Cytophagia,o:Cytophagales,f:Cytophagaceae,g:Hymenobacter

TACGGAGGGTGCGAGCGTTGTCCGGATTTATTGGGTTTAAAGGGTGCGTAGGCGGCCGCG

TAAGTCCGGGGTGAAAGCCCGTTGCTCAACAACGGAACTGCCCTGGAAACTGTGCGGCTT

GAGTCCAGACGAGGTTGGCGGAATGGGCGGTGTAGCGGTGAAATGCATAGATACCGTCCA

GAACCCCGATTGCGAAGGCAGCTGACTAGGCTGGTACTGACGCTGAGGCACGAAAGCGTG

GGGAGCGAAC

>ASV143 SS|1.0000|HM032897_S002167665;k:Bacteria,p:"Bacteroidetes",c:Cytophagia,o:Cytophagales,f:Cytophagaceae,g:Hymenobacter

TACGGAGGGTGCGAGCGTTGTCCGGATTTATTGGGTTTAAAGGGTGCGTAGGCGGCCGTT

TAAGTCTGGGGTGAAAGCCCGCTGCTCAACAGCGGAACTGCCCTGGATACTGGATGGCTT

GAATACAGTGGAGGTTGGCGGAATGGACCGAGTAGCGGTGAAATGCATAGATACGGTCCA

GAACCCCGATTGCGAAGGCAGCTGACTACACTGGTATTGACGCTGAGGCACGATAGCGTG

GGGAGCGAAC

>ASV144 SS|1.0000|D86513_S000011943;k:Bacteria,p:"Proteobacteria",c:Alphaproteobacteria,o:Rhodospirillales,f:Acetobacteraceae

TACGAAGGGGGCTAGCGTTGCTCGGAATGACTGGGCGTAAAGGGCGCGTAGGCGGTTCGG

ACAGTCAGGTGTGAAATTCCCGGGCTTAACCTGGGGACTGCATTTGATACGTCTGGACTA

GAGTGCGGAAGAGGGTCGTGGAATTCCCAGTGTAGAGGTGAAATTCGTAGATATTGGGAA

GAACACCGGTGGCGAAGGCGGCGACCTGGTCCGTGACTGACGCTGAGGCGCGAAAGCGTG

GGGAGCAAAC

>ASV145 GS|99.6|AF175402_S000388588;k:Bacteria,p:Firmicutes,c:Bacilli,o:Lactobacillales,f:Leuconostocaceae,g:Leuconostoc;

TACGTATGTCCCGAGCGTTATCCGGATTTATTGGGCGTAAAGCGAGCGCAGACGGTTGGT

TAAGTCTGATGTGAAAGCCCGGAGCTCAACTCCGGAAAGGCATTGGAAACTGGTCAACTT

GAGTGCAGTAGAGGTAAGTGGAACTCCATGTGTAGCGGTGGAATGCGTAGATATATGGAA

GAACACCAGCGGCGAAGGCGGCTTACTGGACTGTAACTGACGTTGAGGCTCGAAAGTGTG

GGTAGCAAAC

>ASV146 SS|1.0000|EU861928_S001148233;k:Bacteria,p:"Armatimonadetes",c:Armatimonadia,o:Armatimonadales,f:Armatimonadaceae,g:Armatimonas/Armatimonadetes_gp1

TACGTAGGGGGCGAGCGTTGTCCGAAGTTACTGGGCGTAAAGAGCGCGTAGGCGGGTTCT

TAAGTGAGGGGTGAAAGTCCGAGGCTTAACCTCGGAACTGCCTTTCATACTGGGAACCTT

GAGTATGGGAGAGGCGAGTGGAATGGTCGGTGTAGCGGTGAAATGCGTAGATATCGATCG

GAACACCCATGGCGAAGGCAGCTCGCTGGCCTATAACTGACGCTGAGGCGCGAAAGCGTG

GGGAGCAAAC

>ASV147 GS|100.0|AB041885_S000002852;k:Bacteria,p:"Proteobacteria",c:Gammaproteobacteria,o:Pseudomonadales,f:Pseudomonadaceae,g:Pseudomonas;

TACAGAGGGTGCAAGCGTTAATCGGAATTACTGGGCGTAAAGCGCGCGTAGGTGGTTTGT

TAAGTTGAATGTGAAATCCCCGGGCTCAACCTGGGAACTGCATCCAAAACTGGCAAGCTA

GAGTATGGTAGAGGGTAGTGGAATTTCCTGTGTAGCGGTGAAATGCGTAGATATAGGAAG

GAACACCAGTGGCGAAGGCGACTACCTGGACTGATACTGACACTGAGGTGCGAAAGCGTG

GGGAGCAAAC

>ASV148 SS|0.9900|AB739062_S003286967;k:Bacteria,p:"Proteobacteria",c:Alphaproteobacteria,o:Rhodospirillales,f:Acetobacteraceae

TACGAAGGGGGCTAGCGTTGCTCGGAATGACTGGGCGTAAAGGGCGCGTAGGCGGTTGGT

ACAGTCGGATGTGAAATGCCTGGGCTTAACCTGGGGACTGCATTCGATACGTGCTGGCTT

GAGTGTGGAAGAGGGTTGTGGAATTCCCAGTGTAGAGGTGAAATTCGTAGATATTGGGAA

GAACACCGGTGGCGAAGGCGGCAACCTGGTCCATTACTGACGCTGAGGCGCGAAAGCGTG

GGGAGCAAAC

>ASV149 GS|0.0|None;No hit

TACGGGGGGGGCAAGCGTTATTCGAAATGATTGGGCGTAAAGGGCACGTAGACGGTTTTT

TAAGTGGCCATCCTTGTTTTTGTTTTTCCCTTTCAACTCTATTATAAATAAAATACATAG

AAGGGAAACGGGAAAAGGGAAGCAAAGATTAAAATGGAGTGTGGATTCTCTTTATTTTTA

TACACCCTTATTTGTATAAATTATTTATAATGAAAACAAATAAGGGATAACAAGATTCTA

CACTTGGGAA

>ASV150 SS|0.8300|AB267478_S000721192;k:Bacteria,p:"Bacteroidetes",c:Sphingobacteriia,o:"Sphingobacteriales",f:Chitinophagaceae,g:Segetibacter

TACGGAGGGTGCAAGCGTTATCCGGATTTACTGGGTTTAAAGGGTGCGTAGGTGGGAATG

TAAGTCAGTGGTGAAATCTTCATGCTTAACATGGAAACTGCCATTGATACTATATTTCTT

GAATTTTCTGGAGGTTAGCGGAATATGTCATGTAGCGGTGAAATGCTTAGATATGACATA

GAACACCAATTGCGAAGGCAGCTGGCTACAGGAAAATTGACACTGATGCACGAAAGCGTG

GGGATCAAAC

>ASV151 SS|0.9800|AF013554_S000336303;k:Bacteria,p:"Acidobacteria",c:Acidobacteria_Gp4,g:Gp4

TACGAGGGGAGCAAGCGTTGTTCGGATTTACTGGGCGTAAAGGGCGCGTAGGCGGCTCTA

CAAGTCACTTGTGAAATCTCCGGGCTTAACTCGGAACGGTCAAGTGATACTGTCGAGCTA

GAGTGCAGAAGGGGCAATTGGAATTCTCGGTGTAGCGGTGAAATGCGTAGATATCGAGAG

GAACACCAGAGGCGAAGGCGGATTGCTGGGCTGACACTGACGCTGAGGCGCGAAAGCTAG

GGGAGCGAAC

>ASV152 GS|100.0|EF363714_S000806231;k:Bacteria,p:"Proteobacteria",c:Alphaproteobacteria,o:Sphingomonadales,f:Sphingomonadaceae,g:Sphingomonas;

TACGGAGGGAGCTAGCGTTGTTCGGAATTACTGGGCGTAAAGCGCACGTAGGCGGCTTTG

TAAGTTAGAGGTGAAAGCCTGGAGCTCAACTCCAGAATTGCCTTTAAGACTGCATCGCTT

GAATCCAGGAGAGGTGAGTGGAATTCCGAGTGTAGAGGTGAAATTCGTAGATATTCGGAA

GAACACCAGTGGCGAAGGCGGCTCACTGGACTGGTATTGACGCTGAGGTGCGAAAGCGTG

GGGAGCAAAC

>ASV153 SS|1.0000|Z37138_S000001649;k:Bacteria,p:"Actinobacteria",c:Actinobacteria,o:Actinomycetales

TACGTAGGGTGCAAGCGTTGTCCGGAATTACTGGGCGTAAAGAGCTCGTAGGCGGTTTGT

CGCGTCGTCTGTGAAAACCCGTGGCTCAACCACGGGCTTGCAGGCGATACGGGCAGACTT

GAGTATTGCAGGGGAGACTGGAATTCCTGGTGTAGCGGTGAAATGCGCAGATATCAGGAG

GAACACCGGTGGCGAAGGCGGGTCTCTGGGCAAATACTGACGCTGAGGAGCGAAAGCATG

GGTAGCAAAC

>ASV154 SS|1.0000|AM947653_S001093907;k:Bacteria,p:"Proteobacteria",c:Alphaproteobacteria,o:Rhodospirillales,f:Acetobacteraceae

TACGAAGGGGGCTAGCGTTGCTCGGAATGACTGGGCGTAAAGGGCGCGTAGGCGGGCATC

TTAGTCAGGCGTGAAATTCCCGGGCTTAACCTGGGGGCTGCGTTTGATACGGGGTGCCTA

GAGTTTGGCAGAGGGTCGTGGAATTCCCAGTGTAGAGGTGAAATTCGTAGATATTGGGAA

GAACACCGGTGGCGAAGGCGGCGACCTGGGCCTTGACTGACGCTGAGGCGCGAAAGCGTG

GGGAGCAAAC

>ASV155 SS|0.8000|KJ155688_S004086269;k:Bacteria,p:"Bacteroidetes",c:Cytophagia,o:Cytophagales,f:Cytophagaceae,g:Spirosoma

TACGGAGGGTGCAAGCGTTGTCCGGATTTATTGGGTTTAAAGGGTGCGTAGGTGGGTCAT

CAAGTCTGATTTGAAAGCGTGTCGCTTAACGATACGATGTGGTTGGAAACTGGTGGTCTT

GAATGGGGTAGCGGTAGCCGGAATGGGTCATGTAGCGGTGAAATGCATAGATATGACCCG

GAACACCGATTGCGAAGGCAGGCTACTGGGCCTTAATTGACACTGAGGCACGAGAGCATG

GGTAGCGAAC

>ASV156 SS|0.9900|AY140238_S000397413;k:Bacteria,p:"Proteobacteria",c:Alphaproteobacteria,o:Rhodospirillales,f:Acetobacteraceae

TACGAAGGGGGCTAGCGTTGCTCGGAATGACTGGGCGTAAAGGGCGCGTAGGCGGACGGC

TTAGTCGGGCGTGAAAGTCCTGGGCTCAACCTGGGGACGGCGTTCGATACGGGTTGTCTA

GAGTGGGGCAGAGGGTCGTGGAATTCCCAGTGTAGAGGTGAAATTCGTAGATATTGGGAA

GAACACCGGTGGCGAAGGCGGCGACCTGGTCCTTGACTGACGCTGAGGCGCGAAAGCGTG

GGGAGCAAAC

>ASV157 GS|99.6|AM491368_S000804721;k:Bacteria,p:"Bacteroidetes",c:Sphingobacteriia,o:"Sphingobacteriales",f:Sphingobacteriaceae,g:Pedobacter;

TACGGAGGATCCAAGCGTTATCCGGATTTATTGGGTTTAAAGGGTGCGTAGGCGGCTTAT

TAAGTCAGGGGTGAAAGACGGTGGCTCAACCATCGCAGTGCCTTTGATACTGATGAGCTT

GAATACACTAGAGGTAGGCGGAATGTGACAAGTAGCGGTGAAATGCATAGATATGTCACA

GAACACCGATTGCGAAGGCAGCTTACTATGGTGTCATTGACGCTGAGGCACGAAAGCGTG

GGGATCAAAC

>ASV158 SS|1.0000|AB778530_U010573767;k:Bacteria,p:"Proteobacteria",c:Alphaproteobacteria,o:Rhodospirillales,f:Acetobacteraceae

TACGAAGGGGGCTAGCGTTGCTCGGAATGACTGGGCGTAAAGGGCGCGTAGGCGGATCAC

GCAGTCAGATGTGAAATTCCTGGGCTTAACCTGGGGGCTGCATTTGAGACGCGTGGTCTA

GAGTGTGAAAGAGGGTCGTGGAATTCCCAGTGTAGAGGTGAAATTCGTAGATATTGGGAA

GAACACCGGTGGCGAAGGCGGCGACCTGGTTCATTACTGACGCTGAGGCGCGAAAGCGTG

GGGAGCAAAC

>ASV159 SS|1.0000|JX294485_S003614212;k:Bacteria,p:"Bacteroidetes",c:Cytophagia,o:Cytophagales,f:Cytophagaceae,g:Hymenobacter

TACGGAGGGTGCGAGCGTTGTCCGGATTTATTGGGTTTAAAGGGTGCGTAGGCGGTTTTA

TAAGTCTGGGGTGAAAGCCCGCTGCTCAACAGCGGAACTGCCCTGGATACTGTAGGACTT

GAGGACAGACGAGGTTGGCGGAATGGAGGGTGTAGCGGTGAAATGCATAGATACCCTCCA

GAACCCCGATTGCGAAGGCAGCTGACTAGACTGTAACTGACGCTGAGGCACGAAAGCGTG

GGGAGCGAAC

>ASV160 SS|1.0000|HM032898_S002167666;k:Bacteria,p:"Bacteroidetes",c:Cytophagia,o:Cytophagales,f:Cytophagaceae,g:Hymenobacter

TACGGAGGGTGCGAGCGTTGTCCGGATTTATTGGGTTTAAAGGGTGCGTAGGCGGCTCGG

TAAGTCCGGGGTGAAAGCCCGTTGCTCAACAACGGAACTGCCCTGGAAACTGCGGGGCTT

GAGTCCAGACGAGGTCGGCGGAATGGGCGGTGTAGCGGTGAAATGCATAGATACCGTCCA

GAACCCCGATTGCGAAGGCAGCTGACTAGGCTGGTACTGACGCTGAGGCACGAAAGCGTG

GGGAGCGAAC

>ASV161 GS|0.0|None;No hit

TTCCAGCTCCAATAGCGTATATTAAAGTTGTTGCAGTTAAAAAGCTCGTAGTTGAAACTT

GGGCCTGGCTGACCGGTCCGCCTCACCGCGTGTACTGGTTCGGCCGGGCCTTTCCTTCTG

GGGAGCCGCATGGCCTTCATTGGTCGTGTTGGGGATCCAGGACTTTTACTTTGAAAAAAT

TAGAGTGTTCAAAGCAGGCCTATGCTCGAATACATTAGCATGGAATAATAGAATAGGACG

TGTGGTTCTA

>ASV162 GSL|98.0|DQ528761_S000701761;k:Bacteria,p:"Acidobacteria",c:Acidobacteria_Gp1

TACGAGGGGGGCAAGCGTTGTTCGGAATTATTGGGCGTAAAGGGTGCGTAGGCGGTTTGA

CAAGTCTTATGTGAAATCTCTGGGCTCAACCCAGAGTCTGCATGGGAAACTGTCGGGCTT

GAGTATGGGAGAGGTGAGTGGAATTTCCGGTGTAGCGGTGAAATGCGTAGATATCGGAAG

GAACACCTGTGGCGAAAGCGGCTCACTGGACCATAACTGACGCTGAGGCACGAAAGCTAG

GGGAGCAAAC

>ASV163 SS|1.0000|HM032898_S002167666;k:Bacteria,p:"Bacteroidetes",c:Cytophagia,o:Cytophagales,f:Cytophagaceae,g:Hymenobacter

TACGGAGGGTGCGAGCGTTGTCCGGATTTATTGGGTTTAAAGGGTGCGTAGGCGGCTTTG

TAAGTCCGGGGTGAAAGCCCGTTGCTCAACAACGGAACTGCCCTGGAAACTGTAGGGCTT

GAGTCCAGACGAGGTCGGCGGAATGGGCGGTGTAGCGGTGAAATGCATAGATACCGTCCA

GAACCCCGATTGCGAAGGCAGCTGACTAGGCTGGTACTGACGCTGAGGCACGAAAGCGTG

GGGAGCGAAC

>ASV164 GS|99.2|HM777013_S002233062;k:Bacteria,p:"Proteobacteria",c:Betaproteobacteria,o:Burkholderiales,f:Oxalobacteraceae,g:Massilia;

TACGTAGGGTGCAAGCGTTAATCGGAATTACTGGGCGTAAAGCGTGCGCAGGCGGTTTTG

TAAGTCTGTCGTGAAATCCCCGGGCTCAACCTGGGAATGGCGATGGAGACTGCAAGGCTA

GAATCTGGCAGAGGGGGGTAGAATTCCACGTGTAGCAGTGAAATGCGTAGATATGTGGAG

GAACACCGATGGCGAAGGCAGCCCCCTGGGCTAAGATTGACGCTCATGCACGAAAGCGTG

GGGAGCAAAC

>ASV165 SS|1.0000|EF635408_S001095322;k:Bacteria,p:"Deinococcus-Thermus",c:Deinococci,o:Deinococcales,f:Deinococcaceae,g:Deinococcus

TACGGAGGGTGCAAGCGTTACCCGGAATCACTGGGCGTAAAGGGCGTGTAGGCGGCACTC

CAAGTCTGGCTTTAAAGACCGAAGCTCAACTTCGGGACTGGGCCGGAAACTGGAGCGCTA

GACGGATGGAGAGGTCACTGGAATTCCTGGTGTAGCGGTGGAATGCGTAGATACCAGGAG

GAACACCAACGGCGAAGGCAGGTGACTGGACATTTTGTGACGCTGAGGCGCGAAAGTGTG

GGGAGCGAAC

>ASV166 SS|1.0000|AM231587_S000650722;k:Bacteria,p:"Proteobacteria",c:Alphaproteobacteria,o:Rhodospirillales,f:Acetobacteraceae

TACGAAGGGGGCTAGCGTTGCTCGGAATGACTGGGCGTAAAGGGCGCGTAGGCGGATTGG

TCAGTCAGACGTGAAATTCCTGGGCTTAACCTGGGGGCTGCGTTTGAGACGGCTAATCTA

GAGTTTGGAAGAGGGTCGTGGAATTCCCAGTGTAGAGGTGAAATTCGTAGATATTGGGAA

GAACACCGGTGGCGAAGGCGGCGACCTGGTCCTGGACTGACGCTGAGGCGCGAAAGCGTG

GGGAGCAAAC

>ASV167 SS|1.0000|D86513_S000011943;k:Bacteria,p:"Proteobacteria",c:Alphaproteobacteria,o:Rhodospirillales,f:Acetobacteraceae

TACGAAGGGGGCTAGCGTTGCTCGGAATGACTGGGCGTAAAGGGCGCGTAGGCGGATTGC

GTAGTCAGATGTGAAAATCCTGGGCTCAACCTGGGGACTGCATTTGATACGCGCAGTCTA

GAGTTTGGAAGAGGGTCGTGGAATTCCCAGTGTAGAGGTGAAATTCGTAGATATTGGGAA

GAACACCGGTGGCGAAGGCGGCGACCTGGTCCTTGACTGACGCTGAGGCGCGAAAGCGTG

GGGAGCAAAC

>ASV168 SS|1.0000|HM032897_S002167665;k:Bacteria,p:"Bacteroidetes",c:Cytophagia,o:Cytophagales,f:Cytophagaceae,g:Hymenobacter

TACGGAGGGTGCGAGCGTTGTCCGGATTTATTGGGTTTAAAGGGTGCGTAGGCGGCCGTT

TAAGTCTGGGGTGAAAGCCCGTTGCTCAACAGCGGAACTGCCCTGGATACTGGACGGCTT

GAATACAGTGGAGGTTGGCGGAATGGACCGAGTAGCGGTGAAATGCATAGATACGGTCCA

GAACCCCGATTGCGAAGGCAGCTGACTACACTGGTATTGACGCTGAGGCACGACAGCGTG

GGGAGCGAAC

>ASV169 SS|1.0000|AJ519370_S000102021;k:Bacteria

TACGTAGGAGGCGAGCGTTGTCCGGAATTACTGGGCGTAAAGAGCGCGTAGGCGGCGCTG

TACGACACGGGTGAAAGCCCCCGGCTCAACTGGGGAGGGTCCCGTGTAACGGCAGTGCTA

CGAGTGCAGGAGAGGGAAGTGGAACTCCGGGAGTAGCGGTGAAATGCGTAGAGACCCGGA

GGAACACCAGTGGCGAAGGCGGCTTCCTGGTCTGCGACTGACGCTGTGGCGCGAAAGCTA

GGGGAGCAAA

>ASV170 SS|0.9900|FN433469_S001575418;k:Bacteria,p:"Proteobacteria",c:Alphaproteobacteria,o:Rhizobiales

TACGAAGGGGGCTAGCGTTGTTCGGATTTACTGGGCGTAAAGGGTGCGTAGGCGGATTGT

TAAGTCAGGGGTGAAATGCCGGGGCTCAACCTCGGAACTGCCTTTGATACTGGCGATCTT

GAGTCCGGGAGAGGTGAGTGGAACTGCGAGTGTAGAGGTGAAATTCGTAGATATTCGCAA

GAACACCGGTGGCGAAGGCGGCTCACTGGCCCGGAACTGACGCTGAGGCACGAAAGCGTG

GGGAGCAAAC

>ASV171 SS|1.0000|AY788950_S000610642;k:Bacteria,p:"Proteobacteria",c:Alphaproteobacteria,o:Rhodospirillales,f:Acetobacteraceae

TACGAAGGGGGCTAGCGTTGCTCGGAATGACTGGGCGTAAAGGGCGCGTAGGCGGTTGGT

ACAGTCGGATGTGAAATGCCTGGGCTTAACCTGGGGACTGCATTCGATACGTGCTGGCTT

GAGTGCGGAAGAGGGTTGTGGAATTCCCAGTGTAGAGGTGAAATTCGTAGATATTGGGAA

GAACACCGGTGGCGAAGGCGGCAACCTGGTCCGTGACTGACGCTGAGGCGCGAAAGCGTG

GGGAGCAAAC

>ASV172 GS|0.0|None;No hit

TACGGGGGGGGCAAGCGTTATTCGAAATGATTGGGCGTAAAGGGCACGTAGACGGTTTTT

TAAGTGGCCATCCTTGTTTTTGTTTTTCCCTTTCAACTCTATTATAAATAAAATACATAG

AAGGGAAACGGGAAAAGGGAAGCAAAGATTCAAATGGAGTGTGGATTCTCTTTATTTTTA

TAATCCCTTATTTGTATAAATTATTTATAATGAAAACAAATAAGGGATAACAAGATTCTA

CACTTGGGAA

>ASV173 SS|0.9700|JQ309130_S003619637;k:Bacteria,p:"Acidobacteria",c:Acidobacteria_Gp4,g:Blastocatella

TACGTAGGGACCAAGCGTTGTTCGGATTTACTGGGCGTAAAGGGCGCGTAGGCGGCGTGA

CAAGTCAGTTGTGAAATCTCCGAGCTTAACTCGGAACGGTCAACTGATACTGTTGTGCTA

GAGTACAGAAGGGGCAATCGGAATTCTTGGTGTAGCGGTGAAATGCGTAGATATCAAGAG

GAACACCTGAGGTGAAGACGGGTTGCTGGGCTGATACTGACGCTGAGGCGCGAAAGCTAG

GGTAGCAAAC

>ASV174 SS|0.8200|FN391026_S001418752;k:Bacteria,p:"Planctomycetes",c:Planctomycetia,o:Planctomycetales,f:Planctomycetaceae,g:Singulisphaera

GACGAACCGTGCGAACGTTATTCGGAATCACTGGGCTTAAAGCGCGTGTAGGCGGGTTCG

AACGTCCGATGTTGAAAGCCCCCGGCTCAACCGGGGAAGTGGCACGGATACGGCGAGCCT

GGAGGGGGGTAGGGGGAGCTGGAACTTCCGGTGGAGCGGTGAAATGCGTTGAGATCGGAA

GGAACGCCCGTGGCGAAAGCGAGCTCCTGGACCCTTACTGACGCTGAGACGCGAAAGCCA

GGGGAGCGAA

>ASV175 SS|0.8500|Y18189_S000001545;k:Bacteria,p:Firmicutes

TACGTAGGTGGCAAGCGTTGTCCGGATTTACTGGGCGTAAAGCGAACGCAGGCGGATACT

TAAGTAGAGAGTGAAAGGCGGGAGCTCAACTCCTGGACTGCTCCCTATACTGGGTGTCTT

GAGTGGCGGAGAGGAAGATGGAACAAATCGTGTAGCGGTGAAATGCGTAGATATGATTTG

GAACACCAATGGCGAAAGCAATCTTCTGGACGCATACTGACGCTGAGGTTCGAAAGCCAT

GGTAGCGAAC

>ASV176 GS|0.0|None;No hit

CACGTAGAAGACTAGTGTTATTCATCTTTAGTAGGTTTAAAGGGTACCTAGACGGGAAAT

CAAGCCATCTTTGGGACTAATTTTCTAGAGTTTTATAAGAGAAGGGGTGAATTTCCGGAG

GAAAGTTGATATTTTGTGATACCGGAAGGACGGGTAACAGCGAAGGCGACCTTCTATGTA

AAAACTGACGTTGAGGGACGAAGCCTTGGGTAACGATAAGG

>ASV177 GS|71.2|GU207875_S002224490;k:Bacteria,p:"Proteobacteria",c:Deltaproteobacteria,o:Myxococcales,f:Polyangiaceae,g:Chondromyces;

CTTGGTCATTTAGAGGAAGTAAAAGTCGTAACAAGGTTTCCGTAGGTGAACCTGCGGAAG

GATCATTACTAAGAGAGGGATGTACGCTTCCAGCCGAGTCCCGGGGGGCTGCGCCCCTCA

CCTCTTCAACCCTGTGTCTACCAACCGCTGTTGCTTCGGCGAGCGTCGGGGCGTCCGCGC

CCCGGCCCCGGCTTCGGTCGGTGAGCTCTCGCAGAGGCCTATCTTTATTCTGTTTTGCAG

TGACGTCCGA

>ASV178 SS|1.0000|JN090860_S002916046;k:Bacteria,p:"Bacteroidetes",c:Cytophagia,o:Cytophagales,f:Cytophagaceae,g:Hymenobacter

TACGGAGGGTGCGAGCGTTGTCCGGATTTATTGGGTTTAAAGGGTGCGTAGGCGGCCCGT

TAAGTCTGGGGTGAAAGCCCGCTGCTCAACAGCGGAACTGCCCTGGATACTGACGGGCTT

GAGTACAGACGAGGTTGGCGGAATGGACGGAGTAGCGGTGAAATGCATAGATACCGTCCA

GAACCCCGATTGCGAAGGCAGCTGACTAGGCTGCTACTGACGCTGAGGCACGACAGCGTG

GGGAGCGAAC

>ASV179 SS|1.0000|JN090860_S002916046;k:Bacteria,p:"Bacteroidetes",c:Cytophagia,o:Cytophagales,f:Cytophagaceae,g:Hymenobacter

TACGGAGGGTGCGAGCGTTGTCCGGATTTATTGGGTTTAAAGGGTGCGTAGGCGGCCGTT

TAAGTCTGGGGTGAAAGCCCGCTGCTCAACAGCGGAACTGCCCTGGATACTGGATGGCTT

GAGTACAGACGAGGTTGGCGGAATGGACGGAGTAGCGGTGAAATGCATAGATACCGTCCA

GAACCCCGATTGCGAAGGCAGCTGACTAGGCTGATACTGACGCTGAGGCACGACAGCGTG

GGGAGCGAAC

>ASV180 GS|99.6|DQ664244_S000712592;k:Bacteria,p:"Proteobacteria",c:Betaproteobacteria,o:Burkholderiales,f:Burkholderiales_incertae_sedis,g:Piscinibacter;

TACGTAGGGTGCAAGCGTTAATCGGAATTACTGGGCGTAAAGCGTGCGCAGGCGGCTTTG

CAAGACAGATGTGAAATCCCCGGGCTCAACCTGGGAACTGCATTTGTGACTGCATGGCTA

GAGTGCGGCAGAGGGGGATGGAATTCCGCGTGTAGCAGTGAAATGCGTAGATATGCGGAG

GAACACCGATGGCGAAGGCAATCCCCTGGGCCTGCACTGACGCTCATGCACGAAAGCGTG

GGGAGCAAAC

>ASV181 SS|0.9800|AJ416411_S000018317;k:Bacteria,p:"Proteobacteria",c:Alphaproteobacteria,o:Sphingomonadales,f:Sphingomonadaceae

TACGGAGGGGGCTAGCGTTGTTCGGAATTACTGGGCGTAAAGCGCACGTAGGCGGTTATT

CAAGTCAGAGGTGAAAGCCCGGAGCTCAACTCCGGAACTGCCTTTGAAACTAGATAACTC

GAATCATGGAGAGGCGAGTGGAATTCCGAGTGTAGAGGTGAAATTCGTAGATATTCGGAA

GAACACCAGTGGCGAAGGCGGCTCGCTGGACATGTATTGACGCTGAGGTGCGAAAGCGTG

GGGAGCAAAC

>ASV182 SS|1.0000|AY140238_S000397413;k:Bacteria,p:"Proteobacteria",c:Alphaproteobacteria,o:Rhodospirillales,f:Acetobacteraceae

TACGAAGGGGGCTAGCGTTGCTCGGAATGACTGGGCGTAAAGGGCGCGTAGGCGGAATGC

TTTGTCGGGCGTGAAATTCCAGGGCTTAACCTTGGGGCTGCGTTCGAGACGGGTATTCTA

GAGTGGAGAAGAGGGTCGTGGAATTCCCAGTGTAGAGGTGAAATTCGTAGATATTGGGAA

GAACACCGGTGGCGAAGGCGGCGACCTGGTCTTTTACTGACGCTGAGGCGCGAAAGCGTG

GGGAGCAAAC

>ASV183 SS|1.0000|AJ292581_S000022859;k:Bacteria,p:"Acidobacteria",c:Acidobacteria_Gp1

TACGAGGGGGGCAAGCGTTGTTCGGAATTATTGGGCGTAAAGGGTGCGTAGGCGGCCCCG

CAAGTCTTGTGTGAAAGCCTCAAGCTCAACTTGAGGACTGCACGGGAAACTGCTGGGCTG

GAGTATGGGAGAGGTGAGTGGAATTCCTGGTGTAGCGGTGAAATGCGTAGATATCAGGAG

GAACACCTGTGGCGAAAGCGGCTCACTGGACCATAACTGACGCTGAGGCACGAAAGCTAG

GGGAGCAAAC

>ASV184 SS|1.0000|EF635408_S001095322;k:Bacteria,p:"Deinococcus-Thermus",c:Deinococci,o:Deinococcales,f:Deinococcaceae,g:Deinococcus

TACGGAGGGTGCAAGCGTTACCCGGAATCACTGGGCGTAAAGGGCGTGTAGGCGGTTTGC

CAAGTCTGACTTTAAAGACCGAAGCTCAACTTCGGGCATGGGTTGGATACTGGCAGACTA

GACGGATGGAGAGGTCACTGGAATTCCTGGTGTAGCGGTGGAATGCGTAGATACCAGGAG

GAACACCAACGGCGAAGGCAGGTGACTGGACATTTAGTGACGCTGAGGCGCGAAAGTGTG

GGGAGCAAAC

>ASV185 SS|1.0000|EU861928_S001148233;k:Bacteria,p:"Armatimonadetes",c:Armatimonadia,o:Armatimonadales,f:Armatimonadaceae,g:Armatimonas/Armatimonadetes_gp1

TACGTAGGGGGCGAGCGTTGTTCGAAGTTACTGGGCGTAAAGAGCGCGTAGGCGGGTCTT

TAAGTGAGGGGTGAAAGTCCGAGGCTCAACCTCGGAACTGCCTTTTATACTGGGGACCTT

GAGTGTGGGAGAGGCGAGTGGAATGGTCGGTGTAGCGGTGAAATGCGTAGATATCGATCG

GAACACCCATGGCGAAGGCAGCTCGCTGGCCTATAACTGACGCTGAGGCGCGAAAGCGTG

GGGAGCAAAC

>ASV186 SS|1.0000|EF368368_S000806277;k:Bacteria,p:"Proteobacteria",c:Alphaproteobacteria,o:Rhodospirillales,f:Acetobacteraceae

TACGAAGGGGGCTAGCGTTGCTCGGAATGACTGGGCGTAAAGGGCGCGTAGGCGGATTGA

TTAGTCAGGCGTGAAATTCCCGGGCTTAACCTGGGGGCTGCGTTTGATACGGTTGGTCTA

GAGTTTGGCAGAGGGTCGTGGAATTCCCAGTGTAGAGGTGAAATTCGTAGATATTGGGAA

GAACACCGGTGGCGAAGGCGGCGACCTGGTCCTTGACTGACGCTGAGGCGCGAAAGCGTG

GGGAGCAAAC

>ASV187 SS|1.0000|JX294485_S003614212;k:Bacteria,p:"Bacteroidetes",c:Cytophagia,o:Cytophagales,f:Cytophagaceae,g:Hymenobacter

TACGGAGGGTGCGAGCGTTGTCCGGATTTATTGGGTTTAAAGGGTGCGTAGGCGGCCTTA

TAAGTCTGGGGTGAAAGCCCGCTGCTCAACAGCGGAACTGCCCTGGATACTGTGGGGCTT

GAGGACAGACGAGGTTGGCGGAATGGAGGGTGTAGCGGTGAAATGCATAGATACCCTCCA

GAACCCCGATTGCGAAGGCAGCTGACTAGACTGTACCTGACGCTGAGGCACGAAAGCGTG

GGGAGCGAAC

>ASV188 GS|100.0|GQ496083_S001612437;k:Bacteria,p:"Actinobacteria",c:Actinobacteria,o:Actinomycetales,f:Microbacteriaceae,g:Marisediminicola;

TACGTAGGGTGCAAGCGTTGTCCGGAATTATTGGGCGTAAAGAGCTCGTAGGCGGTTTGT

CGCGTCTGCTGTGAAATCCGGAGGCTCAACCTCCGGCCTGCAGTGGGTACGGGCAGACTA

GAGTGCGGTAGGGGAGATTGGAATTCCTGGTGTAGCGGTGGAATGCGCAGATATCAGGAG

GAACACCAATGGCGAAGGCAGATCTCTGGGCCGTAACTGACGCTGAGGAGCGAAAGCATG

GGGAGCGAAC

>ASV189 SS|1.0000|D86513_S000011943;k:Bacteria,p:"Proteobacteria",c:Alphaproteobacteria,o:Rhodospirillales,f:Acetobacteraceae

TACGAAGGGGGCTAGCGTTGCTCGGAATGACTGGGCGTAAAGGGCGCGTAGGCGGATGGC

TTAGTCAGGTGTGAAATTCCTGGGCTTAACCTGGGGGCTGCATTTGAGACGGGTTGTCTA

GAGTTTGGAAGAGGGTCGTGGAATTCCCAGTGTAGAGGTGAAATTCGTAGATATTGGGAA

GAACACCGGTGGCGAAGGCGGCGACCTGGTCCTTGACTGACGCTGAGGCGCGAAAGCGTG

GGGAGCAAAC

>ASV190 SS|1.0000|D30775_S000413730;k:Bacteria,p:"Proteobacteria",c:Alphaproteobacteria,o:Rhodospirillales,f:Acetobacteraceae

TACGAAGGGGGCTAGCGTTGCTCGGAATGACTGGGCGTAAAGGGCGCGTAGGCGGATCGG

ATAGTCAGGCGTGAAATTCCTGGGCTCAACCTGGGGGCTGCGTTTGATACGTTTGGTCTA

GAGTTTGGAAGAGGGTTGTGGAATTCCCAGTGTAGAGGTGAAATTCGTAGATATTGGGAA

GAACACCGGTGGCGAAGGCGGCAACCTGGTCCTTGACTGACGCTGAGGCGCGAAAGCGTG

GGGAGCAAAC

>ASV191 GS|97.6|AB267478_S000721192;k:Bacteria,p:"Bacteroidetes",c:Sphingobacteriia,o:"Sphingobacteriales",f:Chitinophagaceae,g:Segetibacter;

TACGGAGGGTGCAAGCGTTATCCGGATTCACTGGGTTTAAAGGGTGCGTAGGTGGGAATG

TAAGTCAGTGGTGAAATCTCCGTGCTTAACATGGAAACTGCCATTGATACTATGTTTCTT

GAATTTTCCGGAGGTAAGCGGAATATGTCATGTAGCGGTGAAATGCTTAGATATGACATA

GAACACCAATTGCGAAGGCAGCTTACTACAGGAACATTGACACTGAGGCACGAAAGCGTG

GGGATCAAAC

>ASV192 GS|100.0|EF174497_S000776520;k:Bacteria,p:"Proteobacteria",c:Alphaproteobacteria,o:Rhizobiales,f:Methylobacteriaceae,g:Methylobacterium;

TACGAAGGGGGCTAGCGTTGCTCGGAATCACTGGGCGTAAAGGGCGCGTAGGCGGCCATT

CAAGTCGGGGGTGAAAGCCTGTGGCTCAACCACAGAATTGCCTTCGATACTGTTTGGCTT

GAGACCGGAAGAGGTAAGTGGAACTGCGAGTGTAGAGGTGAAATTCGTAGATATTCGCAA

GAACACCAGTGGCGAAGGCGGCTTACTGGTCCGGTTCTGACGCTGAGGCGCGAAAGCGTG

GGGAGCAAAC

>ASV193 SS|1.0000|AB859260_U010573780;k:Bacteria,p:"Bacteroidetes",c:Cytophagia,o:Cytophagales,f:Cytophagaceae,g:Hymenobacter

TACGGAGGGTGCGAGCGTTGTCCGGATTTATTGGGTTTAAAGGGTGCGTAGGCGGCTTGG

TAAGTCTGGGGTGAAAGCCCGCTGCTCAACAGCGGAACTGCCCTGGATACTGCCCAGCTT

GAGGACAGACGAGGTTGGCGGAATGGAGGGTGTAGCGGTGAAATGCATAGATACCCTCCA

GAACCCCGATTGCGAAGGCAGCTGACTAGACTGTAACTGACGCTGAGGCACGAAAGCGTG

GGGAGCGAAC

>ASV194 GS|99.6|DQ660892_S000712490;k:Bacteria,p:"Acidobacteria",c:Acidobacteria_Gp1,g:Terriglobus;

TACGAGGGGGGCAAGCGTTGTTCGGAATTATTGGGCGTAAAGGGCGCGTAGGCGGTTTGG

CAAGTTTCGTGTGAAATCTTCGGGCTCAACTCGAAGTCTGCACGGAAAACTGCCGGGCTT

GAGTATGGGAGAGGTGAGTGGAATTTCCGGTGTAGCGGTGAAATGCGTAGATATCGGAAG

GAACACCTGTGGCGAAAGCGGCTCACTGGACCATAACTGACGCTGAGGCGCGAAAGCTAG

GGGAGCAAAC

>ASV195 SS|1.0000|AY140238_S000397413;k:Bacteria,p:"Proteobacteria",c:Alphaproteobacteria,o:Rhodospirillales,f:Acetobacteraceae

TACGAAGGGGGCTAGCGTTGCTCGGAATGACTGGGCGTAAAGGGCGCGTAGGCGGATTTG

TCAGTCGGGCGTGAAATTCCTGGGCTCAACCTGGGGGCTGCGTTCGAGACGGCGGGTCTT

GAGTGGGGAAGAGGGTCGTGGAATTCCCAGTGTAGAGGTGAAATTCGTAGATATTGGGAA

GAACACCGGTGGCGAAGGCGGCGACCTGGTCCTTGACTGACGCTGAGGCGCGAAAGCGTG

GGGAGCAAAC

>ASV196 SS|1.0000|EU861928_S001148233;k:Bacteria,p:"Armatimonadetes",c:Armatimonadia,o:Armatimonadales,f:Armatimonadaceae,g:Armatimonas/Armatimonadetes_gp1

TACGTAGGGGGCGAGCGTTGTCCGAAGTTACTGGGCGTAAAGAGCGCGTAGGCGGGTTCT

TAAGTGAGGGGTGAAATTCCGAGGCTCAACCTCGGAACTGCCTTTCATACTGGGAACCTT

GAGTGTGGGAGAGGCGAGTGGAATGGTCGGTGTAGCGGTGAAATGCGTAGATATCGATCG

GAACACCCATGGCGAAGGCAGCTCGCTGGCCTATAACTGACGCTGAGGCGCGAAAGCGTG

GGGAGCAAAC

>ASV197 SS|1.0000|EU861928_S001148233;k:Bacteria,p:"Armatimonadetes",c:Armatimonadia,o:Armatimonadales,f:Armatimonadaceae,g:Armatimonas/Armatimonadetes_gp1

TACGTAGGGGGCGAGCGTTGTCCGAAGTTACTGGGCGTAAAGAGCGCGTAGGCGGGTTCT

TAAGTGAGGGGTGAAATTCCGAGGCTTAACCTCGGAACTGCCTCTCATACTGGGAACCTT

GAGTGTGGGAGAGGCGAGTGGAATGGTCGGTGTAGCGGTGAAATGCGTAGATATCGATCG

GAACACCTATGGCGAAGGCAGCTCGCTGGCCTATAACTGACGCTGAGGCGCGAAAGCGTG

GGGAGCAAAC

>ASV198 GS|0.0|None;No hit

TACGTAGAAGCCAAGTGTTATTCATGTTTAATCGGTTTAAAGGGTACCTAGACGGTAAAT

CAAGCCTATAAATAAAAGTAATAAAGGGACTAATTTGCTAGAGTTACTTATGAGGGGGTA

TTAAAGTACTGCTGGTGTAGAGATGAAATTCTGTTATACCTCTTTTCAATAGAAAAATTA

TGGCACAGGTATAGGCGAAGGCATCCCCTTATGTGATAACTGACGTTGAGGGACGAAGGC

TTTGTGTAGC

>ASV199 SS|1.0000|JN090860_S002916046;k:Bacteria,p:"Bacteroidetes",c:Cytophagia,o:Cytophagales,f:Cytophagaceae,g:Hymenobacter

TACGGAGGGTGCGAGCGTTGTCCGGATTTATTGGGTTTAAAGGGTGCGTAGGCGGCCGCG

TAAGTCTGGGGTGAAAGCCCGTTGCTCAACAACGGAACTGCCCTGGAAACTGCGCGGCTT

GAGTCCAGACGAGGTTGGCGGAATGGGCGGTGTAGCGGTGAAATGCATAGATACCGTCCA

GAACTCCGATTGCGAAGGCAGCTGACTAGGCTGGTACTGACGCTGAGGCACGAAAGCGTG

GGGAGCGAAC

>ASV200 SS|1.0000|AB362219_S001043921;k:Bacteria,p:"Proteobacteria",c:Alphaproteobacteria,o:Rhodospirillales,f:Acetobacteraceae

TACGAAGGGGGCTAGCGTTGCTCGGAATGACTGGGCGTAAAGGGCGCGTAGGCGGCTTGG

TCGGTCAGACGTGAAATTCCTGGGCTCAACCTGGGGGCTGCGTTTGATACAGCCTGGCTA

GAGTGGGGAAGAGGGTTGTGGAATTCCCAGTGTAGAGGTGAAATTCGTAGATATTGGGAA

GAACACCGGTGGCGAAGGCGGCAACCTGGTCCTTGACTGACGCTGAGGCGCGAAAGCGTG

GGGAGCAAAC

>ASV201 GS|0.0|None;No hit

TACGAGGGGGGCAAGCGTTATTCGAAATGATTGGGCGTAAAGGGCATCTAGGCGGTTTAT

TTTATTTATTTTCCTGAGCTAAAAAAGTCAAGGATGAAAAAATACCAGAGCTTAACTGTG

GTAGTTGGATAAGTTAAGATAAACTAGAGTAAGTAAGAGGAAAATGGAATTTCTGGAGGA

GAGGTAAAATTTGTAGATTTCAGAAGGAACATCAAAAGCAAAGGCAATTTTCTGGGACTT

TACTGACGCT

>ASV202 GS|99.2|EU861902_S001148207;k:Bacteria,p:"Armatimonadetes",c:Armatimonadia,o:Armatimonadales,f:Armatimonadaceae,g:Armatimonas/Armatimonadetes_gp1;

TACGTAGGGGGCCAGCGTTGTCCGAAGTTACTGGGCGTAAAGAGCGCGTAGGCGGGTCTT

TAAGTGGGGGGTGAAATTCTGAGGCTCAACCTCGGAACTGCCTTCCAAACTGGGGATCTT

GAGTGCGGGAGAGGCGAGTGGAATGGTCGGTGTAGCGGTGAAATGCGTAGATATCGATCG

GAACACCCATGGCGAAGGCAGCTCGCTGGCCTGCAACTGACGCTGAGGCGCGAAAGCCGG

GGGAGCAAAC

>ASV203 SS|1.0000|JN090860_S002916046;k:Bacteria,p:"Bacteroidetes",c:Cytophagia,o:Cytophagales,f:Cytophagaceae,g:Hymenobacter

TACGGAGGGTGCGAGCGTTGTCCGGATTTATTGGGTTTAAAGGGTGCGTAGGCGGCCGTT

TAAGTCCGGGGTGAAAGCCCGCTGCTCAACAGCGGAACGGCCCTGGATACTGGATGGCTT

GAGTACAGACGAGGTTGGCGGAATGGACCGAGTAGCGGTGAAATGCATAGATACGGTCCA

GAACCCCGATTGCGAAGGCAGCTGACTAGGCTGCTACTGACGCTGAGGCACGACAGCGTG

GGGAGCGAAC

>ASV204 GS|100.0|JQ946365_S003299030;k:Bacteria,p:"Proteobacteria",c:Betaproteobacteria,o:Burkholderiales,f:Comamonadaceae,g:Acidovorax;

TACGTAGGGTGCGAGCGTTAATCGGAATTACTGGGCGTAAAGCGTGCGCAGGCGGTGATG

TAAGACAGATGTGAAATCCCCGGGCTCAACCTGGGAACTGCATTTGTGACTGCATCGCTG

GAGTGCGGCAGAGGGGGATGGAATTCCGCGTGTAGCAGTGAAATGCGTAGATATGCGGAG

GAACACCGATGGCGAAGGCAATCCCCTGGGCCTGCACTGACGCTCATGCACGAAAGCGTG

GGGAGCAAAC

>ASV205 SS|0.8900|KJ528316_S004225669;k:Bacteria,p:"Proteobacteria",c:Alphaproteobacteria,o:Sphingomonadales,f:Sphingomonadaceae

TACGGAGGGGGCTAGCGTTGTTCGGAATTACTGGGCGTAAAGCGCACGTAGGCGGCTATT

CAAGTCAGGGGTGAAAGCCCGGAGCTCAACTCCGGAACTGCCTCTGAAACTAGGTAGCTT

GAATCATGGAGAGGCGAGTGGAATTCCGAGTGTAGAGGTGAAATTCGTAGATATTCGGAA

GAACACCAGTGGCGAAGGCGGCTCGCTGGACATGTATTGACGCTGAGGTGCGAAAGCGTG

GGGAGCAAAC

>ASV206 SS|1.0000|AB089482_S000415016;k:Bacteria,p:"Proteobacteria",c:Betaproteobacteria

TACGTAGGGTGCAAGCGTTAATCGGAATTACTGGGCGTAAAGCGTGCGCAGGCGGTTCCG

CAAGTCAGATGTGAAATCCCCGGGCTCAACCTGGGAACTGCATTTGAAACTACGGGGCTA

GAGTGTGTCAGAGGGGGGTGGAATTCCACGTGTAGCAGTGAAATGCGTAGAGATGTGGAG

GAACACCAATGGCGAAGGCAGCCCCCTGGGATGACACTGACGCTCATGCACGAAAGCGTG

GGGAGCAAAC

>ASV207 SS|1.0000|JF834159_S002913810;k:Bacteria,p:"Bacteroidetes",c:Sphingobacteriia,o:"Sphingobacteriales",f:Chitinophagaceae

TACGGAGGGTGCAAGCGTTATCCGGATTCACTGGGTTTAAAGGGTGCGTAGGTGGGCAGT

TAAGTCAGTGGTGAAATCTCCGGGCTCAACCCGGAAACTGCCATTGATACTATCTGTCTT

GAATATCGTGGAGGTAAGCGGAATATGTCATGTAGCGGTGAAATGCTTAGATATGACATA

GAACACCAATTGCGAAGGCAGCTTACTACCCGAATATTGACACTGAGGCACGAAAGCGTG

GGGATCAAAC

>ASV208 SS|1.0000|D86513_S000011943;k:Bacteria,p:"Proteobacteria",c:Alphaproteobacteria,o:Rhodospirillales,f:Acetobacteraceae

TACGAAGGGGGCTAGCGTTGCTCGGAATGACTGGGCGTAAAGGGCGCGTAGGCGGATGGC

TTAGTCAGGTGTGAAATTCCCGGGCTTAACCTGGGGGCTGCATTTGAGACGGGTTGTCTA

GAGTTTGGAAGAGGGTCGTGGAATTCCCAGTGTAGAGGTGAAATTCGTAGATATTGGGAA

GAACACCGGTGGCGAAGGCGGCGACCTGGTCCTTGACTGACGCTGAGGCGCGAAAGCGTG

GGGAGCAAAC

>ASV209 GSL|99.6|AF144383_S000388105;k:Bacteria,p:"Proteobacteria",c:Betaproteobacteria,o:Burkholderiales,f:Comamonadaceae

TACGTAGGGTGCAAGCGTTAATCGGAATTACTGGGCGTAAAGCGTGCGCAGGCGGTGATG

TAAGACAGATGTGAAATCCCCGGGCTCAACCTGGGAACTGCATTTGTGACTGCATCGCTG

GAGTGCGGCAGAGGGGGATGGAATTCCGCGTGTAGCAGTGAAATGCGTAGATATGCGGAG

GAACACCGATGGCGAAGGCAATCCCCTGGGCCTGCACTGACGCTCATGCACGAAAGCGTG

GGGAGCAAAC

>ASV210 SS|1.0000|AY140238_S000397413;k:Bacteria,p:"Proteobacteria",c:Alphaproteobacteria,o:Rhodospirillales,f:Acetobacteraceae

TACGAAGGGGGCTAGCGTTGCTCGGAATGACTGGGCGTAAAGGGCGCGTAGGCGGTTTGC

ACAGTCGGGTGTGAAATTCCTGGGCTTAACCTGGGGGCTGCATTCGATACGTGTGGGCTT

GAGTGTGGAAGAGGGTTGTGGAATTCCCAGTGTAGAGGTGAAATTCGTAGATATTGGGAA

GAACACCGGTGGCGAAGGCGGCAACCTGGTCCATGACTGACGCTGAGGCGCGAAAGCGTG

GGGAGCAAAC

>ASV211 SS|1.0000|EU861928_S001148233;k:Bacteria,p:"Armatimonadetes",c:Armatimonadia,o:Armatimonadales,f:Armatimonadaceae,g:Armatimonas/Armatimonadetes_gp1

TACGTAGGGGGCGAGCGTTGTCCGAAGTTACTGGGCGTAAAGAGCGCGTAGGCGGGTTCT

TAAGTGAGGGGTGAAAGTCCGAGGCTCAACCTCGGAACTGCCTTTCATACTGGGAACCTT

GAGTGCGGGAGAGGCGAGTGGAATGGTCGGTGTAGCGGTGAAATGCGTAGATATCGATCG

GAACACCCATGGCGAAGGCAGCTCGCTGGCCTGTAACTGACGCTGAGGCGCGAAAGCGTG

GGGAGCAAAC

>ASV212 SS|1.0000|HM032897_S002167665;k:Bacteria,p:"Bacteroidetes",c:Cytophagia,o:Cytophagales,f:Cytophagaceae,g:Hymenobacter

TACGGAGGGTGCGAGCGTTGTCCGGATTTATTGGGTTTAAAGGGTGCGTAGGCGGCCGTT

TAAGTCTGGGGTGAAAGCCCGCTGCTCAACAGCGGAACTGCCCTGGATACTGGATGGCTT

GAATACAGTGGAGGTTGGCGGAATGGACCGAGTAGCGGTGAAATGCATAGATACGGTCCA

GAACCCCGATTGCGAAGGCAGCTGACTACACTGGAATTGACGCTGAGGCACGACAGCGTG

GGGAGCGAAC

>ASV213 SS|1.0000|EU861928_S001148233;k:Bacteria,p:"Armatimonadetes",c:Armatimonadia,o:Armatimonadales,f:Armatimonadaceae,g:Armatimonas/Armatimonadetes_gp1

TACGTAGGGGGCGAGCGTTGTTCGAAGTTACTGGGCGTAAAGAGCGCGTAGGCGGGTTCT

TAAGTGAGGGGTGAAAGTCCGGGGCTCAACCCCGGAACTGCCTTTTATACTGGGAACCTT

GAGTGTGGGAGAGGCGAGTGGAATGGTCGGTGTAGCGGTGAAATGCGTAGATATCGATCG

GAACACCCATGGCGAAGGCAGCTCGCTGGCCTATAACTGACGCTGAGGCGCGAAAGCGTG

GGGAGCAAAC

>ASV214 SS|1.0000|JX949238_S003747851;k:Bacteria,p:"Bacteroidetes",c:Sphingobacteriia,o:"Sphingobacteriales",f:Sphingobacteriaceae

TACGGAGGATCCAAGCGTTATCCGGATTTATTGGGTTTAAAGGGTGCGTAGGCGGCCTGT

TAAGTCAGGGGTGAAATTTTTCGGCTCAACCGGAAACTTGCCTTTGATACTGATGGGCTT

GAATGCAGCTGAGGTAGGCGGAATGTGACAAGTAGCGGTGAAATGCATAGATATGTCACA

GAACACCAATTGCGAAGGCAGCTTACCAAAGTGTGATTGACGCTGAGGCACGAAAGCGTG

GGGATCAAAC

>ASV215 SS|1.0000|JX949238_S003747851;k:Bacteria,p:"Bacteroidetes",c:Sphingobacteriia,o:"Sphingobacteriales",f:Sphingobacteriaceae

TACGGAGGATCCAAGCGTTATCCGGATTTATTGGGTTTAAAGGGTGCGTAGGCGGCCTGT

TAAGTCAGGGGTGAAATTTTTCGGCTCAACCGGGACATTGCCTTTGATACTGACGGGCTT

GAATACAGTTGAGGTAGGCGGAATGTGACAAGTAGCGGTGAAATGCATAGATATGTCACA

GAACACCGATTGCGAAGGCAGCTTACCAAAGTGTGATTGACGCTGAGGCACGAAAGCGTG

GGGATCAAAC

>ASV216 SS|1.0000|D86513_S000011943;k:Bacteria,p:"Proteobacteria",c:Alphaproteobacteria,o:Rhodospirillales,f:Acetobacteraceae

TACGAAGGGGGCTAGCGTTGCTCGGAATGACTGGGCGTAAAGGGCGCGTAGGCGGATTGA

TCAGTCAGGCGTGAAATTCCTGGGCTTAACCTGGGGGCTGCGTTTGAGACGGTTGGTCTA

GAGTGGGGAAGAGGGTCGTGGAATTCCCAGTGTAGAGGTGAAATTCGTAGATATTGGGAA

GAACACCGGTGGCGAAGGCGGCGACCTGGTCCTTGACTGACGCTGAGGCGCGAAAGCGTG

GGGAGCAAAC

>ASV217 SS|1.0000|HM032897_S002167665;k:Bacteria,p:"Bacteroidetes",c:Cytophagia,o:Cytophagales,f:Cytophagaceae,g:Hymenobacter

TACGGAGGGTGCGAGCGTTGTCCGGATTTATTGGGTTTAAAGGGTGCGTAGGCGGCCGTT

TAAGTCTGGGGTGAAAGCCCGCTGCTCAACAGCGGAACTGCCCTGGATACTGGATAGCTT

GAATACAGTGGAGGTTGGCGGAATGGACCGAGTAGCGGTGAAATGCATAGATACGGTCCA

GAACCCCGATTGCGAAGGCAGCTGACTACACTGGTATTGACGCTGAGGCACGACAGCGTG

GGGAGCGAAC

>ASV218 GS|0.0|None;No hit

TACGTGGAAGACTAGTGTTATTCATCTTTATTAGGTTTAAAGGGTACCTAGACGGTATTT

CTAGCCCGCAAAGGGTACGGATTTACTAGAGTTTTATGTAAGAGGCTACTGTTAGTACTA

TTGGTGTAGAGATGAAATTCTTTGATACTATTAGGACTGGTGGCGGCGAAAGCAACCCTT

TATGTATAAACTGACGTTGAGGGACGAAGGCTTGGGTAGCGAACAGG

>ASV219 SS|1.0000|D86512_S000010801;k:Bacteria,p:"Proteobacteria",c:Alphaproteobacteria,o:Rhodospirillales,f:Acetobacteraceae

TACGAAGGGGGCTAGCGTTGCTCGGAATGACTGGGCGTAAAGGGCGCGTAGGCGGACGGC

TTAGTCAGGCGTGAAATTCCCGGGCTTAACCTGGGGGCTGCGTTTGATACGGGTTGTCTA

GAGTTTGGCAGAGGGTCGTGGAATTCCCAGTGTAGAGGTGAAATTCGTAGATATTGGGAA

GAACACCGGTGGCGAAGGCGGCGACCTGGTCCTTGACTGACGCTGAGGCGCGAAAGCGTG

GGGAGCAAAC

>ASV220 SS|1.0000|JN090860_S002916046;k:Bacteria,p:"Bacteroidetes",c:Cytophagia,o:Cytophagales,f:Cytophagaceae,g:Hymenobacter

TACGGAGGGTGCGAGCGTTGTCCGGATTTATTGGGTTTAAAGGGTGCGTAGGCGGCTTGG

TAAGTCTGGGGTGAAAGCCCGCTGCTCAACAGCGGAACTGCCCTGGATACTGCCAGGCTT

GAGGACAGACGAGGTTAGCGGAATGGACGGTGTAGCGGTGAAATGCATAGATACCGTCCA

GAACCCCGATTGCGAAGGCAGCTGACTAGACTGTAACTGACGCTGAGGCACGAAAGCGTG

GGGAGCGAAC

>ASV221 GS|97.2|KF999686_S004084195;k:Bacteria,p:"Bacteroidetes",c:Cytophagia,o:Cytophagales,f:Cytophagaceae,g:Spirosoma;

TACGGAGGGTGCAAGCGTTGTCCGGATTTATTGGGTTTAAAGGGTGCGTAGGTGGGTGTT

TAAGTCTGGTTTGAAAGCAGGCGGCTCAACCGTCTGATGTGGCTGGAGACTGGGCATCTT

GAATGGGTTGGCGGTAGCCGGAATGGGTCATGTAGCGGTGAAATGCATAGATATGACCCG

GAACACCGATTGCGAAGGCAGGCTACTACGACCTGATTGACACTGAGGCACGAGAGCATG

GGTAGCGAAC

>ASV222 GS|99.6|AB594446_S002949232;k:Bacteria,p:"Actinobacteria",c:Actinobacteria,o:Actinomycetales,f:Dermacoccaceae,g:Branchiibius;

TACGTAGGGTGCGAGCGTTGTCCGGAATTATTGGGCGTAAAGAGCTTGTAGGCGGTTTGT

CGCGTCTGCTGTGAAAGCCCGGGGCTTAACTCCGGGTCTGCAGTGGGTACGGGCAGGCTA

GAGTGTGGTAGGGGAGACTGGAATTCCTGGTGTAGCGGTGAAATGCGCAGATATCAGGAG

GAACACCGATGGCGAAGGCAGGTCTCTGGGCCATTACTGACGCTGAGAAGCGAAAGCATG

GGGAGCAAAC

>ASV223 GS|98.0|AB267478_S000721192;k:Bacteria,p:"Bacteroidetes",c:Sphingobacteriia,o:"Sphingobacteriales",f:Chitinophagaceae,g:Segetibacter;

TACGGAGGGTGCAAGCGTTATCCGGATTCACTGGGTTTAAAGGGTGCGTAGGTGGGAATG

TAAGTCAGTGGTGAAATCTCCGTGCTTAACATGGAAACTGCCATTGATACTATGTTTCTT

GAATTTTCCGGAGGTAAGCGGAATATGTCATGTAGCGGTGAAATGCTTAGATATGACATA

GAACACCAATTGCGAAGGCAGCTTACTACAGGAATATTGACACTGAGGCACGAAAGCGTG

GGGATCAAAC

>ASV224 SS|1.0000|AM887757_S000941900;k:Bacteria,p:"Acidobacteria",c:Acidobacteria_Gp1

TACGAGGGGGGCAAGCGTTGTTCGGAATTATTGGGCGTAAAGGGTGCGTAGGCGGTTTGA

TAAGTCTTGTGTGAAATCTATGGGCTCAACCCATAGTCTGCACAGGAAACTGTCGGGCTT

GAGAGTGGGAGAGGTGAGTGGAATTTCCGGTGTAGCGGTGAAATGCGTAGATATCGGAAG

GAACACCTGTGGCGAAAGCGGCTCACTGGACCATTTCTGACGCTGATGCACGAAAGCTAG

GGGAGCAAAC

>ASV225 GS|97.2|AJ292684_S000017061;k:Bacteria,p:candidate_division_WPS-2,g:WPS-2_genera_incertae_sedis;

GACGTAGGGGGCAAGCGTTATTCGGAATTATTGGGCGTAAAGCGCTCGTAGGCGGGACAG

GAAGTCCGTGAAGAAAGACCTGGGCTTAACTCAGGGAACGGCACGGATACTCTTGTTCTT

GAGGCAATCAGAGGGTGATGGAATTCCCGGTGTAGCGGTGAAATGCGTAGATATCGGGAG

GAACACCAGTGGCGAAGGCGATCACCTGGGGTTGTTCTGACGCTGAGGAGCGAAAGCTAG

GGGAGCAAAC

>ASV226 GS|97.6|AB245356_S000627892;k:Bacteria,p:"Proteobacteria",c:Gammaproteobacteria,o:Pseudomonadales,f:Pseudomonadaceae,g:Rhizobacter;

TACGTAGGGTGCAAGCGTTAATCGGAATTACTGGGCGTAAAGCGTGCGCAGGCGGCTTTG

CAAGACAGATGTGAAATCCCCGGGCTCAACCTGGGAACTGCATTTGTGACTGCAAGGCTA

GAGTACGGTAGAGGGGAGTGGAATTCCGCGTGTAGCAGTGAAATGCGTAGATATGCGGAG

GAACACCGATGGCGAAGGCAGCTCCCTGGACCTGTACTGACGCTCATGCACGAAAGCGTG

GGGAGCAAAC

>ASV227 GSL|78.4|AJ009456_S000115949;k:Bacteria

TACGTAGGTGGCAAGCGTTGTCCGGATTTACTGGGCGTAAAGCGAACGCAGGCGGACTGT

TAAGTAGAAAGTGAAAGGTCGGAGCTCAACTCCAACATTGCTTTCTATACTGGCAGTCTT

GAGTAGCGGAGAGGAAGGGGGAACGACACGTGTAGCGGTGAAATGCGTTGATATGTGTCG

GAACACCCATGGCGAAAGCACCCTTCTGGACGCAAACTGACGCTCAGGTTCGAAAGCCAA

GGTAGCGAAC

>ASV228 GS|0.0|None;No hit

TACGTGGAAGACTAGTGTTATTCATCTTTAATAGGTTTAAAGGGTACCTAGACGGTATTT

CTAGCCCCAAAAGGGTACGGATTACTAGAGTTTTATGTGAGAGGAAAAATTGTTAGTACT

ATTGGTGTAGAGATGAAATTCTTTGATACTTCTAGGACTGGTGGCGGCGAAAGCAGCCCT

CTAAGTATAAACTGACGTTGAGGGACGAAGGCTTGGGTAGCGAACAGG

>ASV229 SS|1.0000|EF407879_S000824637;k:Bacteria,p:"Bacteroidetes",c:Sphingobacteriia,o:"Sphingobacteriales",f:Chitinophagaceae

TACGGAGGGTGCAAGCGTTATCCGGATTCACTGGGTTTAAAGGGAGCGTAGGCGGGCAGG

TAAGTCAGTGGTGAAATCTTCGAGCTTAACTCGGAAACTGCCATTGATACTATCTATCTT

GAATATTGTGGAGGTAAGCGGAATATGTCATGTAGCGGTGAAATGCTTAGAGATGACATA

GAACACCTATTGCGAAGGCAGCTTACTACGCATATATTGACGCTGAGGCTCGAAAGCGTG

GGGATCAAAC

>ASV230 GS|0.0|None;No hit

TTCCAGCTCCAATAGCGTATATTAAAGTTGTTGCAGTTAAAAAGCTCGTAGTTGAAACTT

GGGCCTGGCTGGCCGGTCCGCCTCACCGCGTGCACTGGTCCGGCCGGGCCTTTCCTTCTG

GGGAGCCGTATGCCCTTCACTGGGCGTGCCGGGGAACCAGGACTTTTACTTTGAAAAAAT

TAGAGTGTTCAAAGCAGGCCTATGCTCGAATACATTAGCATGGAATAATAGAATAGGACG

TGTGGTTCTA

>ASV231 SS|0.9400|AJ416411_S000018317;k:Bacteria,p:"Proteobacteria",c:Alphaproteobacteria,o:Sphingomonadales,f:Sphingomonadaceae

TACGGAGGGGGCTAGCGTTGTTCGGAATTACTGGGCGTAAAGCGCACGTAGGCGGCTATT

CAAGTCAGGGGTGAAAGCCCGGAGCTCAACTCCGGAACTGCCTCTGAAACTAGGTAGCTC

GAATCATGGAGAGGCGAGTGGAATTCCGAGTGTAGAGGTGAAATTCGTAGATATTCGGAA

GAACACCAGTGGCGAAGGCGGCTCGCTGGACATGTATTGACGCTGAGGTGCGAAAGCGTG

GGGAGCAAAC

>ASV232 GS|0.0|None;No hit

GTGTCAGCAGCCGCAAGGACAAAGACCAGCTCTTAGGGAAACGACCTGCTTTCTATGGCG

ACGAGGCTTGCCGATTACTGGAGATTGCTGGGGATGAGCTTATTTCAGAGGCTACCGGAG

CGGCACCTGGCGACAACGTAGTCC

>ASV233 SS|1.0000|D86513_S000011943;k:Bacteria,p:"Proteobacteria",c:Alphaproteobacteria,o:Rhodospirillales,f:Acetobacteraceae

TACGAAGGGGGCTAGCGTTGCTCGGAATGACTGGGCGTAAAGGGCGCGTAGGCGGATTGA

TCAGTCAGGCGTGAAATTCCTGGGCTTAACCTGGGGGCTGCGTTTGAGACGGTTGGTCTA

GAGTTTGGAAGAGGGTTGTGGAATTCCCAGTGTAGAGGTGAAATTCGTAGATATTGGGAA

GAACACCGGTGGCGAAGGCGGCGACCTGGTCCTTGACTGACGCTGAGGCGCGAAAGCGTG

GGGAGCAAAC

>ASV234 SS|1.0000|HM032898_S002167666;k:Bacteria,p:"Bacteroidetes",c:Cytophagia,o:Cytophagales,f:Cytophagaceae,g:Hymenobacter

TACGGAGGGTGCGAGCGTTGTCCGGATTTATTGGGTTTAAAGGGTGCGTAGGCGGCTTTT

TAAGTCTGAGGTGAAAGCCCGCTGCTCAACAGCGGAACGGCCCTGGATACTGGAGAGCTT

GAGTACAGACGAGGTTGGCGGAATGGACGGAGTAGCGGTGAAATGCATAGATACCGTCCA

GAACCCCGATTGCGAAGGCAGCTGACTAGGCTGTTACTGACGCTGAGGCACGAAAGCGTG

GGGAGCGAAC

>ASV235 SS|1.0000|JN695632_S002960974;k:Bacteria,p:"Bacteroidetes",c:Sphingobacteriia,o:"Sphingobacteriales",f:Sphingobacteriaceae

TACGGAGGATCCAAGCGTTATCCGGATTTATTGGGTTTAAAGGGTGCGTAGGCGGCCTGT

TAAGTCAGGGGTGAAATTTTCCGGCTCAACCGGGGCATTGCCTTTGATACTGACGGGCTT

GAATGCAGCTGAGGTAGGCGGAATGTGACAAGTAGCGGTGAAATGCATAGATATGTCACA

GAACACCAATTGCGAAGGCAGCTTACCAAAGTGTGATTGACGCTGAGGCACGAAAGCGTG

GGGATCAAAC

>ASV236 SS|1.0000|D30775_S000413730;k:Bacteria,p:"Proteobacteria",c:Alphaproteobacteria,o:Rhodospirillales,f:Acetobacteraceae

TACGAAGGGGGCTAGCGTTGCTCGGAATGACTGGGCGTAAAGGGCGCGTAGGCGGATTGG

TCAGTCAGACGTGAAATTCCTGGGCTCAACCTGGGGGCTGCGTTTGAGACGGCTAATCTA

GAGTTTGGAAGAGGGTTGTGGAATTCCCAGTGTAGAGGTGAAATTCGTAGATATTGGGAA

GAACACCGGTGGCGAAGGCGGCAACCTGGTCCTTGACTGACGCTGAGGCGCGAAAGCGTG

GGGAGCAAAC

>ASV237 SS|1.0000|EF368368_S000806277;k:Bacteria,p:"Proteobacteria",c:Alphaproteobacteria,o:Rhodospirillales,f:Acetobacteraceae

TACGAAGGGGGCTAGCGTTGCTCGGAATGACTGGGCGTAAAGGGCGCGTAGGCGGATTGA

TTAGTCAGGCGTGAAATTCCTGGGCTTAACCTGGGGGCTGCGTTTGATACGGTTGGTCTA

GAGTTTGGAAGAGGGTCGTGGAATTCCCAGTGTAGAGGTGAAATTCGTAGATATTGGGAA

GAACACCGGTGGCGAAGGCGGCGACCTGGTCCTTGACTGACGCTGAGGCGCGAAAGCGTG

GGGAGCAAAC

>ASV238 SS|0.9800|KJ155688_S004086269;k:Bacteria,p:"Bacteroidetes",c:Cytophagia,o:Cytophagales,f:Cytophagaceae,g:Spirosoma

TACGGAGGGTGCAAGCGTTGTCCGGATTTATTGGGTTTAAAGGGTGCGCAGGTGGTTCTG

TAAGTCTGATTTGAAAGCTAGCGGCTTAACCGTTAGATGTGGTTGGAAACTGTGGAACTT

GAATGGCGTAGCGGGAGCCGGAATGGGTCATGTAGCGGTGAAATGCATAGATATGACCCG

GAACACCGATTGCGAAGGCAGGCTCCTGGGCGCTGATTGACACTGAGGCACGAAAGCATG

GGTAGCGAAC

>ASV239 GS|78.8|AJ009456_S000115949;k:Bacteria,p:"Armatimonadetes",c:Chthonomonadetes,o:Chthonomonadales,f:Chthonomonadaceae,g:Chthonomonas/Armatimonadetes_gp3;

TACGTAGGTGGCAAGCGTTGTCCGGATTTACTGGGCGTAAAGGGCAAGCAGGCGGACTGT

TAAGTAGAAAGTGAAAGGTTGGGGCTCAACCCCAACATTGCTTTCTATACTGGCAGTCTT

GAATCCCGGAGGGGAAAGCGGAACAATACGTGTAGCGGTGAAATGCGTTGATATGTATTG

GAACACCAATGGCGAAGGCAGCTTTCTGGACGGGGATTGACGCTCATTTGCGAAAGCCGA

GGTAGCGAAC

>ASV240 GSL|100.0|AJ310412_S000351463;k:Bacteria,p:"Actinobacteria",c:Actinobacteria,o:Actinomycetales,f:Microbacteriaceae

TACGTAGGGTGCAAGCGTTGTCCGGAATTATTGGGCGTAAAGAGCTCGTAGGCGGTTTGT

CGCGTCTGCTGTGAAAACTGGAGGCTCAACCTCCAGCCTGCAGTGGGTACGGGCAGACTA

GAGTGCGGTAGGGGAGATTGGAATTCCTGGTGTAGCGGTGGAATGCGCAGATATCAGGAG

GAACACCGATGGCGAAGGCAGATCTCTGGGCCGTAACTGACGCTGAGGAGCGAAAGCATG

GGGAGCGAAC

>ASV241 GSL|100.0|AJ007800_S000002983;k:Bacteria,p:"Proteobacteria",c:Alphaproteobacteria,o:Sphingomonadales,f:Sphingomonadaceae,g:Sphingomonas

TACGGAGGGAGCTAGCGTTGTTCGGAATTACTGGGCGTAAAGCGCACGTAGGCGGCTTTG

TAAGTCAGAGGTGAAAGCCTGGAGCTCAACTCCAGAACTGCCTTTGAGACTGCATCGCTT

GAATCCAGGAGAGGTGAGTGGAATTCCGAGTGTAGAGGTGAAATTCGTAGATATTCGGAA

GAACACCAGTGGCGAAGGCGGCTCACTGGACTGGTATTGACGCTGAGGTGCGAAAGCGTG

GGGAGCAAAC

>ASV242 GS|99.6|EF363714_S000806231;k:Bacteria,p:"Proteobacteria",c:Alphaproteobacteria,o:Sphingomonadales,f:Sphingomonadaceae,g:Sphingomonas;

TACGGAGGGAGCTAGCGTTGTTCGGAATTACTGGGCGTAAAGCGCACGTAGGCGGCTTTG

TAAGTTAGAGGTGAAAGCCTGGAGCTTAACTCCAGAATTGCCTTTAAGACTGCATCGCTT

GAATCCAGGAGAGGTGAGTGGAATTCCGAGTGTAGAGGTGAAATTCGTAGATATTCGGAA

GAACACCAGTGGCGAAGGCGGCTCACTGGACTGGTATTGACGCTGAGGTGCGAAAGCGTG

GGGAGCAAAC

>ASV243 SS|1.0000|EU861928_S001148233;k:Bacteria,p:"Armatimonadetes",c:Armatimonadia,o:Armatimonadales,f:Armatimonadaceae,g:Armatimonas/Armatimonadetes_gp1

TACGTAGGGGGCGAGCGTTGTCCGAAGTTACTGGGCGTAAAGAGCGCGTAGGCGGGTTCT

TAAGTGAGGGGTGAAATTCCGGGGCTCAACCCCGGAACTGCCTTTCATACTGGGGACCTT

GAGTGCGGGAGAGGCGAGTGGAATGGTCGGTGTAGCGGTGAAATGCGTAGATATCGATCG

GAACACCCATGGCGAAGGCAGCTCGCTGGCCTGTAACTGACGCTGAGGCGCGAAAGCGTG

GGGAGCAAAC

>ASV244 GS|98.4|EF067920_S001020492;k:Bacteria,p:Cyanobacteria/Chloroplast,c:Chloroplast,f:Chloroplast,g:Bacillariophyta;

GACGGAGGATGCAAGTGTTATCCGGAATCACTGGGCGTAAAGCGTCTGTAGGTGGTCAAA

TAAGTCAACTGTTAAATCTTGAGGCTCAACCTCAAAATCGCAGTCGAAACTATTAGACTA

GAGTATAGTAGGGGTAAAGGGAATTTCCAGTGGAGCGGTGAAATGCGTAGATATTGGAAA

GAACACCGATGGCGAAGGCACTTTACTGGGCTATTACTAACACTCAGAGACGAAAGCTAG

GGTAGCAAAT

>ASV245 GS|98.0|HQ687087_S002989302;k:Bacteria,p:"Acidobacteria",c:Acidobacteria_Gp1,g:Granulicella;

TACGAGGGGGGCAAGCGTTGTTCGGAATTATTGGGCGTAAAGGGTGCGTAGGCGGTTTGA

CAAGTCTCATGTGAAATCTTCGGGCTCAACCCGAAGCCTGCATGGGAAACTGTCGGGCTT

GAGTATGGGAGAGGTGAGTGGAATTTCCGGTGTAGCGGTGAAATGCGTAGATATCGGAAG

GAACACCTGTGGCGAAAGCGGCTCACTGGACCATAACTGACGCTGAGGCACGAAAGCTAG

GGGAGCAAAC

>ASV246 GS|100.0|AY543023_S000369470;k:Bacteria,p:Firmicutes,c:Bacilli,o:Bacillales,f:Listeriaceae,g:Brochothrix;

TACGTAGGTGGCAAGCGTTGTCCGGAATTATTGGGCGTAAAGCGCGCGCAGGCGGTCTCT

TAAGTCTGATGTGAAAGCCCCCGGCTCAACCGGGGAGGGTCATTGGAAACTGGGAGACTT

GAGGACAGAAGAGGAGAGTGGAATTCCAAGTGTAGCGGTGAAATGCGTAGATATTTGGAG

GAACACCAGTGGCGAAGGCGGCTCTCTGGTCTGTTACTGACGCTGAGGCGCGAAAGCGTG

GGGAGCAAAC

>ASV247 GS|97.2|EF451725_S000979715;k:Bacteria,p:"Bacteroidetes",c:Cytophagia,o:Cytophagales,f:Cytophagaceae,g:Spirosoma;

TACGGAGGGTGCAAGCGTTGTCCGGATTTATTGGGTTTAAAGGGTGCGTAGGTGGTTTTT

TAAGTCTGGTTTGAAAGCAAGTGGCTCAACCATTTGATGTGGCTGGAAACTGGGGAACTT

GAATGGATTGGCGGTAGCCGGAACGGGTCATGTAGCGGTGAAATGCATAGATATGACCCA

GAACACCGATTGCGAAGGCAGGCTACTACGATTTGATTGACACTGAGGCACGAGAGCATG

GGTAGCGAAC

>ASV248 GS|100.0|AJ227785_S000112772;k:Bacteria,p:"Proteobacteria",c:Alphaproteobacteria,o:Caulobacterales,f:Caulobacteraceae,g:Brevundimonas;

TACGAAGGGGGCTAGCGTTGCTCGGAATTACTGGGCGTAAAGGGAGCGTAGGCGGACATT

TAAGTCAGAGGTGAAATCCCGGAGCTTAACTTCGGAACTGCCTTTGATACTGGGTGTCTT

GAGTGTGAGAGAGGTATGTGGAACTCCGAGTGTAGAGGTGAAATTCGTAGATATTCGGAA

GAACACCAGTGGCGAAGGCGACATACTGGCTCATTACTGACGCTGAGGCTCGAAAGCGTG

GGGAGCAAAC

>ASV249 GS|97.2|AB377116_S000994777;k:Bacteria,p:"Actinobacteria",c:Actinobacteria,o:Actinomycetales,f:Kineosporiaceae,g:Kineosporia;

TACGTAGGGTGCAAGCGTTGTCCGGAATTATTGGGCGTAAAGAGCTCGTAGGCGGTTCGT

CGCGTCTGCTGTGAAAACCTGGGGCTCAACTCCGGGCGTGCAGTGGGTACGGGCGGGCTA

GAGTGCAGTAGGGGAGACTGGAATTCCTGGTGTAGCGGTGAAATGCGCAGATATCAGGAG

GAACACCGGTGGCGAAGGCGGGTCTCTGGGCTGTTACTGACGCTGAGGAGCGAAAGCGTG

GGGAGCGAAC

>ASV250 SS|0.8200|DQ229107_S001020348;k:Bacteria,p:Cyanobacteria/Chloroplast,c:Chloroplast,f:Chloroplast,g:Streptophyta

GACAGGTGATGCAAGTGTTATCCGGAATGATTGGGCGTAAAGCGTCTGTAGGTGGTTTAT

CCAGTCTGTTGTTAAAACTTAGGGCTTAACCCTAATCCGGCAACAGAAACTAATAGACTA

GAGTACGGTAGGGGCAGAGGGAATTCTCGGTGTAGTGGTGAAATACGTAGATATCGAGAA

GAACACCAATAGCGAAAGCACTCTGCTGGGCCGAAACTGACATTCAGAGACGAAAGCTAG

GGGAGCGAAA

>ASV251 GS|97.6|DQ321750_S000636629;k:Bacteria,p:"Actinobacteria",c:Actinobacteria,o:Actinomycetales,f:Nakamurellaceae,g:Nakamurella;

TACGTAGGGTGCAAGCGTTGTCCGGAATTATTGGGCGTAAAGAGCTCGTAGGCGGTCTGT

CGCGTCGACTGTGAAAACCCGGGGCTCAACTCCGGGCCTGCAGTCGATACGGGCAGACTA

GAGTTCGGTAGGGGAGACTGGAATTCCTGGTGTAGCGGTGAAATGCGCAGATATCAGGAG

GAACACCGGTGGCGAAGGCGGGTCTCTGGGCCGATACTGACGCTGAGGAGCGAAAGCGTG

GGGAGCAAAC

>ASV252 GS|0.0|None;No hit

GTGTCAGCAGCCACTTTCTGCCATGACCGTAACCCAAAGGCGTACCATTGGGTCCACCAA

TTTGACTCCGTTATCATCTGGCGGATTGTGGCTCGCAGTGATCATTACTCCAACGATCTG

ACCATTCAACTTTCTTGAGCGTAGAGCTGCCAGCAACCCAACTCGAAAGACGACTGAGTC

GAGAAGCGCCCTGCGTCCGGTTAAGGTTGAGGCTGATTACTGTACAGACCGCATCGGACG

AACTGCAATG

>ASV253 SS|1.0000|EF635408_S001095322;k:Bacteria,p:"Deinococcus-Thermus",c:Deinococci,o:Deinococcales,f:Deinococcaceae,g:Deinococcus

TACGGAGGGTGCAAGCGTTACCCGGAATCACTGGGCGTAAAGGGCGTGTAGGCGGCTTGC

CAAGTCTGACTTTAAAGACCGACGCTCAACGTCGGGCATGGGTTGGAGACTGGCAGGCTA

GACGGATGGAGAGGTCGCTGGAATTCCTGGTGTAGCGGTGGAATGCGTAGATACCAGGAG

GAACACCCATGGCGAAGGCAGGCGACTGGACATTTGGTGACGCTGAGGCGCGAAAGTGTG

GGGAGCAAAC

>ASV254 SS|1.0000|D30774_S000413729;k:Bacteria,p:"Proteobacteria",c:Alphaproteobacteria,o:Rhodospirillales,f:Acetobacteraceae

TACGAAGGGGGCTAGCGTTGCTCGGAATGACTGGGCGTAAAGGGCGCGTAGGCGGCTTGG

TCGGTCAGACGTGAAATTCCTGGGCTCAACCTGGGGGCTGCGTTTGATACAGCTAGGCTA

GAGTGGGGAAGAGGGTTGTGGAATTCCCAGTGTAGAGGTGAAATTCGTAGATATTGGGAA

GAACACCGGTGGCGAAGGCGGCAACCTGGTCCTTGACTGACGCTGAGGCGCGAAAGCGTG

GGGAGCAAAC

>ASV255 SS|1.0000|JN090860_S002916046;k:Bacteria,p:"Bacteroidetes",c:Cytophagia,o:Cytophagales,f:Cytophagaceae,g:Hymenobacter

TACGGAGGGTGCGAGCGTTGTCCGGATTTATTGGGTTTAAAGGGTGCGCAGGCGGCCGCG

TAAGTCCGGGGTGAAAGCCCGTTGCTCAACAACGGAACTGCCCCGGAAACTGCGCGGCTT

GAGTCCAGACGAGGTCGGCGGAATGGGCGGTGTAGCGGTGAAATGCATAGATACCGTCCA

GAACCCCGATTGCGAAGGCAGCTGACTAGGCTGGTACTGACGCTGAGGCACGAAAGCGTG

GGGAGCGAAC

>ASV256 SS|1.0000|HM032898_S002167666;k:Bacteria,p:"Bacteroidetes",c:Cytophagia,o:Cytophagales,f:Cytophagaceae,g:Hymenobacter

TACGGAGGGTGCGAGCGTTGTCCGGATTTATTGGGTTTAAAGGGTGCGTAGGCGGCTTTT

TAAGTCTGGGGTGAAAGCCCGTTGCTTAACAACGGAACTGCCCTGGATACTGGAGAGCTT

GAGTACAGACGAGGTTGGCGGAATGGACCGAGTAGCGGTGAAATGCATAGATACGGTCCA

GAACCCCGATTGCGAAGGCAGCTGACTAGGCTGATACTGACGCTGAGGCACGACAGCGTG

GGGAGCGAAC

>ASV257 SS|0.9700|EU861902_S001148207;k:Bacteria,p:"Armatimonadetes",c:Armatimonadia,o:Armatimonadales,f:Armatimonadaceae,g:Armatimonas/Armatimonadetes_gp1

TACGTAGGGGGCGAGCGTTGTCCGAAGTTACTGGGCGTAAAGGGCGCGTAGGCGGGTCCT

TAAGTGTGGGGTGAAATCCTGTGGCTCAACCTCAGGACTGCCCTGCAAACTGGGGGCCTT

GAGTGTCGGAGGGGCGAGTGGAATGGTCGGTGTAGCGGTGAAATGCGTAGATATCGATCG

GAACACCCATGGCGAAGGCAGCTCGCTGGCCGACAACTGACGCTGAGGCGCGAAAGCGTG

GGGAGCAAAC

>ASV258 SS|0.8600|AB001684_S000495320;k:Bacteria,p:Cyanobacteria/Chloroplast,c:Chloroplast,f:Chloroplast

GACGGAGGATGCAAGCGTTATCCGGAATGATTGGGCGTAAAGCGTCTGTAGGTGGTTTAT

TAAGTCTACTGTTAAATATCAGGGCTTAACCCTGAATCGGCAGTAGAAACTCATAAGCTT

GAGTACGGTAGGGGCAGAGGGAATTCCCGGTGTAGCGGTGAAATGCGTAGAGATCGGGAA

GAACACCGATGGCGAAAGCACTCTGCTGGGCCGAAACTGACACTGAGAGACGAAAGCTAG

GGGAGCAAAA

>ASV259 GS|0.0|None;No hit

TACGTAGAAGACTAGTGTTATTCATCTTTAATAGGTTTAAAGGGTACCTAGACGGTAAAT

CAAGCCTTAATTGGGACTAATTTGCTAGAGTTACTTATGAGGGGGCATTAATGTACTAGT

GGTGTAGAGATGAAATTTTGTGATACCTTTTCTTGAAAAAGAACTGATGGCACAGGTATA

GGCGAAAGCATCCCCTTATGTGATAACTGACGTTGAGGGACGAAGGCTTTGTGTAGCGAA

CAGG

>ASV260 GS|0.0|None;No hit

TTCCAGCTCCAATAGCGTATATTAAAGTTGTTGCAGTTAAAAAGCTCGTAGTTGAACCTT

GGGCCTGGCCGCCCGGTCCGCCTCACCGCGTGCACTGGTTCGGCCGGGCCTTTCCTCCTG

GGGAACCGCATGCCCTTCACTGGGTGTGCCGGGGAATCAGGACTTTTACTTTGAAAAAAT

TAGAGTGTTCAAAGCAGGCATTTGCTCGAATACATTAGCATGGAATAATAGAATAGGACG

TCGTGGTTCT

>ASV261 GS|98.4|AB594446_S002949232;k:Bacteria,p:"Actinobacteria",c:Actinobacteria,o:Actinomycetales,f:Dermacoccaceae,g:Branchiibius;

TACGTAGGGTGCGAGCGTTGTCCGGAATTATTGGGCGTAAAGAGCTTGTAGGCGGTTTGT

CGCGTCTGCTGTGAAAGCCCGGGGCTTAACTCCGGGTCTGCAGTGGGTACGGGCAGGCTA

GAGTATGGTAGGGGAGACTGGAATTCCTAGTGTAGCGGTGAAATGCGCAGATATTAGGAG

GAACACCGATGGCGAAGGCAGGTCTCTGGGCCATTACTGACGCTGAGAAGCGAAAGCATG

GGGAGCAAAC

>ASV262 GS|99.2|EU861876_S001148181;k:Bacteria,p:"Armatimonadetes",c:Armatimonadia,o:Armatimonadales,f:Armatimonadaceae,g:Armatimonas/Armatimonadetes_gp1;

TACGTAGGGGGCCAGCGTTGTCCGAAGTTACTGGGCGTAAAGAGCGCGTAGGCGGGCCTC

TAAGTGGGGGGTGAAAGGTCGGGGCTCAACCCCGACACTGCCTCTCATACTGGGGGCCTT

GAGTGCGGGAGAGGCGAGTGGAATGGTCGGTGTAGCGGTGAAATGCGTAGATATCGATCG

GAACACCCATGGCGAAGGCAGCTCGCTGGCCTGCAACTGACGCTGAGGCGCGAAAGCCGG

GGGAGCGAAC

>ASV263 GS|0.0|None;No hit

GTGTCAGCAGCCACGTTCAATCCGCGGAGATGCATAACCAAGCCGTCAACTCCCTGGCAT

TCATCTCCACCCGCTACACGCTCGAAAGTGCAGACCTGGTGTCGCTGATGAGCGCTTCTT

ACCTGTATGCCGTTTGTCAAGCCTTGGACCTGCGTGTGCTCCAGAAGATATTCTTCCAGC

ATCTCGAACCCGCACTCTTTGCGATCAACCTCGAGGTCCTCGGCGAATACCTCTCTCCGG

CCGCGATTAA

>ASV264 SS|1.0000|HM032898_S002167666;k:Bacteria,p:"Bacteroidetes",c:Cytophagia,o:Cytophagales,f:Cytophagaceae,g:Hymenobacter

TACGGAGGGTGCGAGCGTTGTCCGGATTTATTGGGTTTAAAGGGTGCGTAGGCGGCGGCG

TAAGTCCGGGGTGAAAGCCCGTTGCTCAACAACGGAACTGCCCTGGAAACTGCGCGGCTT

GAGTCCAGACGAGGTTGGCGGAATGGGCGGTGTAGCGGTGAAATGCATAGATACCGTCCA

GAACCCCGATTGCGAAGGCAGCTGACTAGGCTGGTACTGACGCTGAGGCACGAAAGCGTG

GGGAGCGAAC

>ASV265 SS|1.0000|HM032898_S002167666;k:Bacteria,p:"Bacteroidetes",c:Cytophagia,o:Cytophagales,f:Cytophagaceae,g:Hymenobacter

TACGGAGGGTGCGAGCGTTGTCCGGATTTATTGGGTTTAAAGGGTGCGTAGGCGGCCGTT

TAAGTCTGGGGTGAAAGCCCGCTGCTCAACAGCGGAACTGCCCTGGATACTGGATGGCTT

GAGTACAGACGAGGTTGGCGGAATGGACTGAGTAGCGGTGAAATGCATAGATACAGTCCA

GAACCCCGATTGCGAAGGCAGCTGACTAGGCTGTTACTGACGCTGAGGCACGAAAGCGTG

GGGAGCGAAC

>ASV266 SS|1.0000|JX294485_S003614212;k:Bacteria,p:"Bacteroidetes",c:Cytophagia,o:Cytophagales,f:Cytophagaceae,g:Hymenobacter

TACGGAGGGTGCGAGCGTTGTCCGGATTTATTGGGTTTAAAGGGTGCGTAGGCGGCCAAA

TAAGTCTGGGGTGAAAGCCCGCTGCTCAACAGCGGAACTGCCCTGGATACTGTATGGCTT

GAGGACAGACGAGGTTGGCGGAATGGAGGGTGTAGCGGTGAAATGCATAGATACCCTCCA

GAACCCCGATTGCGAAGGCAGCTGACTAGACTGTATCTGACGCTGAGGCACGAAAGCGTG

GGGAGCGAAC

>ASV267 SS|0.8300|AB649056_S002949821;k:Bacteria,p:"Proteobacteria",c:Alphaproteobacteria,o:Sphingomonadales,f:Sphingomonadaceae

TACGGAGGGGGCTAGCGTTGTTCGGAATTACTGGGCGTAAAGCGTCCGTAGGCGGCTTGC

CAAGTCAGAGGTGAAATCCCACGACTCAATCGTGGAACTGCCTTTGAGACTGGTTCGCTT

GAAGATGGGAGAGGTTAGTGGAATTCCGAGTGTAGAGGTGAAATTCGTAGATATTCGGAA

GAACACCAGTGGCGAAGGCGGCTAACTGGACCATTCTTGACGCTGAGGGACGAAAGCGTG

GGGAGCAAAC

>ASV268 GS|100.0|AY152673_S000398317;k:Bacteria,p:"Proteobacteria",c:Gammaproteobacteria,o:Pseudomonadales,f:Pseudomonadaceae,g:Pseudomonas;

TACAGAGGGTGCAAGCGTTAATCGGAATTACTGGGCGTAAAGCGCGCGTAGGTGGTTTGT

TAAGTTGAATGTGAAATCCCCGGGCTCAACCTGGGAACTGCATCCAAAACTGGCAAGCTA

GAGTAGGGCAGAGGGTGGTGGAATTTCCTGTGTAGCGGTGAAATGCGTAGATATAGGAAG

GAACACCAGTGGCGAAGGCGACCACCTGGGCTCATACTGACACTGAGGTGCGAAAGCGTG

GGGAGCAAAC

>ASV269 SS|1.0000|AF127407_S000387324;k:Bacteria,p:"Proteobacteria",c:Alphaproteobacteria,o:Rhodospirillales,f:Acetobacteraceae

TACGAAGGGGGCTAGCGTTGCTCGGAATGACTGGGCGTAAAGGGCGCGTAGGCGGATTGG

TCAGTCAGATGTGAAATTCCTGGGCTTAACCTGGGGGCTGCATTTGAGACGGCAGGTCTA

GAGTGTGAGAGAGGGTCGTGGAATTCCCAGTGTAGAGGTGAAATTCGTAGATATTGGGAA

GAACACCGGTGGCGAAGGCGGCGACCTGGTTCATAACTGACGCTGAGGCGCGAAAGCGTG

GGGAGCAAAC

>ASV270 GS|100.0|AB022925_S000382657;k:Bacteria,p:Firmicutes,c:Bacilli,o:Lactobacillales,f:Leuconostocaceae,g:Leuconostoc;

TACGTATGTCCCGAGCGTTATCCGGATTTATTGGGCGTAAAGCGAGCGCAGACGGTTGGT

TAAGTCTGATGTGAAAGCCCGGAGCTCAACTCCGGAAAGGCATTGGAAACTGGTTAACTT

GAGTGCAGTAGAGGTAAGTGGAACTCCATGTGTAGCGGTGGAATGCGTAGATATATGGAA

GAACACCAGCGGCGAAGGCGGCTTACTGGACTGTAACTGACGTTGAGGCTCGAAAGTGTG

GGTAGCAAAC

>ASV271 GS|98.8|JQ309130_S003619637;k:Bacteria,p:"Acidobacteria",c:Acidobacteria_Gp4,g:Blastocatella;

TACGTAGGGACCAAGCGTTGTTCGGATTTACTGGGCGTAAAGGGCGCGTAGGCGGCAATT

CAAGTCAGTTGTGAAATCTCCGAGCTTAACTCGGAACGGTCAACTGATACTGCTTTGCTA

GAGTACAGAAGGGGCAATCGGAATTCTTGGTGTAGCGGTGAAATGCGTAGATATCAAGAG

GAACACCAGAGGTGAAGACGGGTTGCTGGGCTGATACTGACGCTGAGGCGCGAAAGCTAG

GGTAGCAAAC

>ASV272 SS|1.0000|AJ519370_S000102021;k:Bacteria

TACGTAGGATGCGAGCGTTGTCCGGAATTACTGGGCGTAAAGAGCGCGTAGGCGGCGCTG

TACGACCGCGGTGAAAGCCCCCGGCTCAACTGGGGAGGGTCCGTGGTAACGGCAGTGCTC

TGAGTGCAGGAGAGGGAAGTGGAACTCCGGGAGTAGCGGTGAAATGCGTAGAGACCCGGA

GGAACACCAGTGGCGAAGGCGGCTTCCTGGTCTGCAACTGACGCTGTGGCGCGAAAGCTA

GGGGAGCAAA

>ASV273 SS|0.8400|AB267478_S000721192;k:Bacteria,p:"Bacteroidetes",c:Sphingobacteriia,o:"Sphingobacteriales",f:Chitinophagaceae,g:Segetibacter

TACGGAGGGTGCAAGCGTTATCCGGATTTACTGGGTTTAAAGGGTGCGTAGGTGGGAATG

TAAGTCAGTGGTGAAATCTTCATGCTTAACATGGAAACTGCCATTGATACTATGTTTCTT

GAATTTTCTGGAGGTTAGCGGAATATGTCATGTAGCGGTGAAATGCTTAGATATGACATA

GAACACCAATTGCGAAGGCAGCTGGCTACAGGAAAATTGACACTGATGCACGAAAGCGTG

GGGATCAAAC

>ASV274 SS|1.0000|JN090860_S002916046;k:Bacteria,p:"Bacteroidetes",c:Cytophagia,o:Cytophagales,f:Cytophagaceae,g:Hymenobacter

TACGGAGGGTGCGAGCGTTGTCCGGATTTATTGGGTTTAAAGGGTGCGTAGGCGGCCTTG

TAAGTCTGGGGTGAAAGCCCGTTGCTCAACAACGGAACTGCCCTGGAAACTGCGGGGCTT

GAGTCCAGACGAGGTCGGCGGAATGGGCGGTGTAGCGGTGAAATGCATAGATACCGTCCA

GAACCCCGATTGCGAAGGCAGCTGACTAGGCTGGTACTGACGCTGAGGCACGAAAGCGTG

GGGAGCGAAC

>ASV275 GS|98.4|AM887757_S000941900;k:Bacteria,p:"Acidobacteria",c:Acidobacteria_Gp1,g:Granulicella;

TACGAGGGGGGCAAGCGTTGTTCGGAATTATTGGGCGTAAAGGGTGCGTAGGCGGTTTGA

CAAGTCTTATGTGAAATCTCTGGGCTCAACCCAGAGTCTGCATGGGAAACTGTCGGGCTT

GAGTATGGGAGAGGTGAGTGGAATTTCCGGTGTAGCGGTGAAATGCGTAGATATCGGAAG

GAACACCTGTGGCGAAAGCGGCTCACTGGACCATAACTGACGCTGATGCACGAAAGCTAG

GGGAGCAAAC

>ASV276 GS|100.0|AB245368_S000627904;k:Bacteria,p:"Bacteroidetes",c:Sphingobacteriia,o:"Sphingobacteriales",f:Sphingobacteriaceae,g:Pedobacter;

TACGGAGGATCCAAGCGTTATCCGGATTTATTGGGTTTAAAGGGTGCGTAGGCGGCTTAT

TAAGTCAGGGGTGAAAGACGGTGGCTCAACCATCGCAGTGCCTTTGATACTGATGAGCTT

GAATGAACTAGAGGTAGGCGGAATGTGACAAGTAGCGGTGAAATGCATAGATATGTCACA

GAACACCGATTGCGAAGGCAGCTTACTATGGTTTTATTGACGCTGAGGCACGAAAGCGTG

GGGATCAAAC

>ASV277 SS|1.0000|JN090860_S002916046;k:Bacteria,p:"Bacteroidetes",c:Cytophagia,o:Cytophagales,f:Cytophagaceae,g:Hymenobacter

TACGGAGGGTGCGAGCGTTGTCCGGATTTATTGGGTTTAAAGGGTGCGTAGGCGGCCGAT

TAAGTCCGGGGTGAAAGCCCGCTGCTCAACAGCGGAACGGCCCTGGATACTGGCTGGCTT

GAGTACAGACGAGGTTGGCGGAATGGACGGAGTAGCGGTGAAATGCATAGATACCGTCCA

GAACCCCGATTGCGAAGGCAGCTGACTAGGCTGGTACTGACGCTGAGGCACGACAGCGTG

GGGAGCGAAC

>ASV278 GS|98.8|JQ772481_S003313779;k:Bacteria,p:"Proteobacteria",c:Alphaproteobacteria,o:Sphingomonadales,f:Sphingomonadaceae,g:Sphingomonas;

TACGGAGGGGGCTAGCGTTGTTCGGAATTACTGGGCGTAAAGCGCACGTAGGCGGCTTTG

TAAGTTAGAGGTGAAAGCCCGGGGCTCAACTCCGGAATTGCCTTTAAGACTGCATCGCTT

GAACATCGGAGAGGTAAGTGGAATTCCGAGTGTAGAGGTGAAATTCGTAGATATTCGGAA

GAACACCAGTGGCGAAGGCGACTTACTGGACGATTGTTGACGCTGAGGTGCGAAAGCGTG

GGGAGCAAAC

>ASV279 GSL|100.0|AJ294412_S000017300;k:Bacteria,p:"Actinobacteria",c:Actinobacteria,o:Actinomycetales,f:Intrasporangiaceae

TACGTAGGGTGCGAGCGTTGTCCGGAATTATTGGGCGTAAAGAGCTTGTAGGCGGTTTGT

CGCGTCTGCTGTGAAAATCCGGGGCTCAACCCCGGACTTGCAGTGGGTACGGGCAGACTA

GAGTGTGGTAGGGGAGACTGGAATTCCTGGTGTAGCGGTGAAATGCGCAGATATCAGGAG

GAACACCGATGGCGAAGGCAGGTCTCTGGGCCACTACTGACGCTGAGAAGCGAAAGCATG

GGGAGCGAAC

>ASV280 GS|0.0|None;No hit

TACGAGGGGGGCGAGCGTTATTCGGAATGATTGGGCGTAAAGGGCATCTAGTTGGTATAA

TCTATTTGAATCATAAAATACCAGAGCTACACTGTGGTTGGTGATTTAAAATAAGGACTT

TTTTGCTTCTATGTAAGTAAAATCAGTATTTATACTATAGATTATACTAGAGTAAAATAA

AGGAAAAAGGAATTTCTGTTGGAGAGGTAAAATTTGTAGATATCAGAAGGAACACCGAAA

GCGAAGGCAT

>ASV281 SS|1.0000|EF457349_S000834774;k:Bacteria

TACGTAGGGGGCGAGCGTTGTCCGGATTTATTGGGCGTAAAGGGCGCGTAGGCGGCCTGG

CGCATCCGGCGTGAAATCTCCCCGCTCAACGGGGAGGGGTCGGCGGAGATGGCCGGGCTT

GAGGGCGGTAGAGGCAGATGGAAGTCCCGAAGTAGTGGTGAAATGCGTAGAGATCGGGAG

GAACACCAGAGGCGAAGGCGATCTGCTGGGCCGACCCTGACGCTGAGGCGCGACAGCCAG

GGGAGCGAAC

>ASV282 SS|0.9100|AJ292687_S000017618;k:Bacteria,p:candidate_division_WPS-1,g:WPS-1_genera_incertae_sedis

GACAGAGGTGCCAAGCGTTAGGCGGAATCACTGGGCTTAAAGCGTGTGTAGGCGGATGTT

TAAGTACTTTGTGAAATCCCACGGCTCAACCGTGGAACTGCTCGGTATACTGGACATCTT

GAGGCAATTAGGGGTTACCGGAACAAGTGGTGGAGCGGTGAAATGCGTAGATATCACTTG

GAACGCCAATGGCGAAGGCAGGTAACTGGGATTGTCCTGACGCTGAGACACGAAAGCTAG

GGGAGCAAAC

>ASV283 GS|97.2|HM051286_S002167842;k:Bacteria,p:"Bacteroidetes",c:Sphingobacteriia,o:"Sphingobacteriales",f:Sphingobacteriaceae,g:Pedobacter;

TACGGAGGATCCAAGCGTTATCCGGATTTATTGGGTTTAAAGGGTGCGTAGGCGGCCTGT

TAAGTCAGGGGTGAAAGACGGTGGCTCAACCATCGCAGTGCCTTTGATACTGACGGGCTT

GAATACAGCTGAGGTAGGCGGAATGTGACAAGTAGCGGTGAAATGCATAGATATGTCACA

GAACACCAATTGCGAAGGCAGCTTACTAAAGTGTGATTGACGCTGAGGCACGAAAGCGTG

GGGATCAAAC

>ASV284 GSL|98.4|AJ717391_S000544176;k:Bacteria,p:"Proteobacteria",c:Alphaproteobacteria,o:Caulobacterales,f:Caulobacteraceae

TACGAAGGGGGCTAGCGTTGCTCGGAATTACTGGGCGTAAAGGGCGCGTAGGCGGACAGT

TTAGTCAGAGGTGAAAGCCCAGGGCTCAACCTTGGAACTGCCTTTGATACTGGCTGTCTT

GAGTACGGGAGAGGTGTGTGGAACTCCGAGTGTAGAGGTGAAATTCGTAGATATTCGGAA

GAACACCGGTGGCGAAGGCGACACACTGGCCCGTTACTGACGCTGAGGCGCGAAAGCGTG

GGGAGCAAAC

>ASV285 GS|98.8|AB166881_S000386315;k:Bacteria,p:"Proteobacteria",c:Alphaproteobacteria,o:Caulobacterales,f:Caulobacteraceae,g:Phenylobacterium;

TACGAAGGGGGCTAGCGTTGCTCGGAATTACTGGGCGTAAAGGGCGCGTAGGCGGACAGT

TTAGTCAGAGGTGAAAGCCCAGGGCTCAACCTTGGAACTGCCTTTGATACTGGCTGTCTT

GAGTTCGGGAGAGGTGAGTGGAACTCCGAGTGTAGAGGTGAAATTCGTAGATATTCGGAA

GAACACCAGTGGCGAAGGCGACTCACTGGCCCGATACTGACGCTGAGGCGCGAAAGCGTG

GGGAGCAAAC

>ASV286 SS|1.0000|AY788950_S000610642;k:Bacteria,p:"Proteobacteria",c:Alphaproteobacteria,o:Rhodospirillales,f:Acetobacteraceae

TACGAAGGGGGCTAGCGTTGCTCGGAATGACTGGGCGTAAAGGGCGCGTAGGCGGCTTGC

ATAGTCAGATGTGAAATTCCCGGGCTTAACCTGGGGGCTGCATTTGATACGTGCGGGCTA

GAGTGTGAAAGAGGGTCGTGGAATTCCCAGTGTAGAGGTGAAATTCGTAGATATTGGGAA

GAACACCGGTGGCGAAGGCGGCGACCTGGTTCATGACTGACGCTGAGGCGCGAAAGCGTG

GGGAGCAAAC

>ASV287 SS|0.8500|JF834159_S002913810;k:Bacteria,p:"Bacteroidetes",c:Sphingobacteriia,o:"Sphingobacteriales",f:Chitinophagaceae

TACGGAGGGTGCAAGCGTTATCCGGATTTACTGGGTTTAAAGGGTGCGCAGGCGGACTTT

TAAGTCAGAGGTGAAATCTCCGGGCTCAACCCGGAAACTGCCCCTGATACTATTGGTCTT

GAATATCGTTGAGGTAGGCGGAATACATCATGTAGCGGTGAAATGCTTAGATATGATGTA

GAACACCAATTGCGAAGGCAGCTTACTAAACGATTATTGACGCTCAGGCACGAAAGCGTG

GGGAGCAAAC

>ASV288 GS|99.6|DQ664244_S000712592;k:Bacteria,p:"Proteobacteria",c:Betaproteobacteria,o:Burkholderiales,f:Burkholderiales_incertae_sedis,g:Piscinibacter;

TACGTAGGGTGCAAGCGTTAATCGGAATTACTGGGCGTAAAGCGTGCGCAGGCGGCTTTG

CAAGACAGATGTGAAATCCCCGGGCTTAACCTGGGAACTGCATTTGTGACTGCATGGCTG

GAGTGCGGCAGAGGGGGATGGAATTCCGCGTGTAGCAGTGAAATGCGTAGATATGCGGAG

GAACACCGATGGCGAAGGCAATCCCCTGGGCCTGCACTGACGCTCATGCACGAAAGCGTG

GGGAGCAAAC

>ASV289 SS|1.0000|D86513_S000011943;k:Bacteria,p:"Proteobacteria",c:Alphaproteobacteria,o:Rhodospirillales,f:Acetobacteraceae

TACGAAGGGGGCTAGCGTTGCTCGGAATGACTGGGCGTAAAGGGCGCGTAGGCGGATTGT

GTAGTCAGATGTGAAATTCCTGGGCTCAACCTGGGGACTGCATTTGATACGCGCAGTCTA

GAGTTTGGAAGAGGGTCGTGGAATTCCCAGTGTAGAGGTGAAATTCGTAGATATTGGGAA

GAACACCGGTGGCGAAGGCGGCGACCTGGTCCTTGACTGACGCTGAGGCGCGAAAGCGTG

GGGAGCAAAC

>ASV290 SS|1.0000|DQ422812_S001020357;k:Bacteria,p:Cyanobacteria/Chloroplast

GACGGAGGATGCAAGCGTTATCCGGAATGATTGGGCGTAAAGCGTCTGTAGGTGGATTGT

AAAGTCCTCTGTTAAAGATCTGGGCTTAACCCAGTTCAAGCAGTGGAAACTTATAATCTA

GAGTACGGTAGGGGCAGAGGGAATTCCCGGTGTAGCGGTGAAATGCGTAGATATCGGGAA

GAACACCGACAGCGAAAGCACTCTGCTGGGCCGAAACTGACACTGAGAGACGAAAGCATG

GGGATCAAAG

>ASV291 SS|1.0000|EU741687_S001046429;k:Bacteria

TACGGGGGGTGCAAGCGTTGCTCGGAATTATTGGGCGTAAAGGGTAGGTAGGTGGTCTCA

TTTGTCTGGGGTGAAAGCCTTGAGCTTAACTCAAGAAGTGCCTCAGAAACGGTGAGACTA

GAGTTCTGGAGAGGGTCGTGGAATTCCCGGTGTAGCGGTGAAATGCGTAGAGATCGGGAG

GAACACCAGAGGCGAAGGCGGCGACCTGGACAGTGACTGACACTGAACTACGAAAGCGTG

GGGAGCAAAC

>ASV292 SS|1.0000|KF999686_S004084195;k:Bacteria,p:"Bacteroidetes",c:Cytophagia,o:Cytophagales,f:Cytophagaceae,g:Spirosoma

TACGGAGGGTGCGAGCGTTGTCCGGATTTATTGGGTTTAAAGGGTGCGTAGGTGGGTGGC

TAAGTCTGGTTTGAAAGCAGGTGGCTCAACCATCTGATGTGGCTGGAAACTGGCGATCTT

GAATGGGTTGGCGGTTGCCGGAACGGGTCATGTAGCGGTGAAATGCATAGATATGACCCA

GAACACCGATTGCGAAGGCAGGCAACTAGGACTTGATTGACACTGAGGCACGAGAGCCGG

GGTAGCGAAC

>ASV293 SS|1.0000|KJ504175_S004089758;k:Bacteria,p:"Actinobacteria",c:Actinobacteria,o:Actinomycetales

TACGTAGGGTGCAAGCGTTGTCCGGAATTATTGGGCGTAAAGAGCTCGTAGGCGGTTTGT

TACGTCGGCTGTGAAAACCTGGGGCTCAACTCCGGGCCTGCAGCTGATACGGGCAGACTA

GAGTTCGGCAGGGGAGACTGGAATTCCTGGTGTAGCGGTGAAATGCGCAGATATCAGGAG

GAACACCGGTGGCGAAGGCGGGTCTCTGGGCCGATACTGACGCTGAGGAGCGAAAGCGTG

GGGAGCAAAC

>ASV294 SS|1.0000|EF635408_S001095322;k:Bacteria,p:"Deinococcus-Thermus",c:Deinococci,o:Deinococcales,f:Deinococcaceae,g:Deinococcus

TACGGAGGGTGCAAGCGTTACCCGGAATCACTGGGCGTAAAGGGCGTGTAGGCGGCTCAG

TCAGTCTGACTTTAAAGACCGAAGCTCAACTTCGGGCCTGGGTTGGATACTGCTGAGCTA

GACGGATGGAGAGGTCACTGGAATTCCTGGTGTAGCGGTGGAATGCGTAGATACCAGGAG

GAACACCAACGGCGAAGGCAGGTGACTGGACATTTGGTGACGCTGAGGCGCGAAAGTGTG

GGGAGCGAAC

>ASV295 SS|1.0000|JN090860_S002916046;k:Bacteria,p:"Bacteroidetes",c:Cytophagia,o:Cytophagales,f:Cytophagaceae,g:Hymenobacter

TACGGAGGGTGCGAGCGTTGTCCGGATTTATTGGGTTTAAAGGGTGCGTAGGCGGCCGTT

TAAGTCTGGGGTGAAAGCCCGCTGCTCAACAGCGGAACGGCCCTGGATACTGGATGGCTT

GAGTACAGACGAGGTTGGCGGAATGGACCGAGTAGCGGTGAAATGCATAGATACGGTCCA

GAACCCCGATTGCGAAGGCAGCTGACTAGGCTGATACTGACGCTGAGGCACGACAGCGTG

GGGAGCGAAC

>ASV296 SS|1.0000|JX949238_S003747851;k:Bacteria,p:"Bacteroidetes",c:Sphingobacteriia,o:"Sphingobacteriales",f:Sphingobacteriaceae

TACGGAGGATCCAAGCGTTATCCGGATTTATTGGGTTTAAAGGGTGCGTAGGCGGCCTGT

TAAGTCAGGGGTGAAATTTTTCGGCTCAACCGGAAACTTGCCTTTGATACTGACGGGCTT

GAATGCAGCTGAGGTAAGCGGAATGTGACAAGTAGCGGTGAAATGCATAGATATGTCACA

GAACACCAATTGCGAAGGCAGCTTACTAAAGTGTGATTGACGCTGAGGCACGAAAGCGTG

GGGATCAAAC

>ASV297 SS|0.8300|AY140238_S000397413;k:Bacteria,p:"Proteobacteria",c:Alphaproteobacteria,o:Rhodospirillales,f:Acetobacteraceae,g:Acidicaldus

TACGAAGGGGGCTAGCGTTGCTCGGAATGACTGGGCGTAAAGGGCGCGTAGGCGGATTTG

TCAGTCGGGCGTGAAATTCCTGGGCTTAACCTGGGGGCTGCGTTCGAGACGGCGGGTCTA

GAGTTTGGAAGAGGGTCGTGGAATTCCCAGTGTAGAGGTGAAATTCGTAGATATTGGGAA

GAACACCGGTGGCGAAGGCGGCGACCTGGTCCTGGACTGACGCTGAGGCGCGAAAGCGTG

GGGAGCAAAC

>ASV298 GS|100.0|JQ417431_S003290754;k:Bacteria,p:"Proteobacteria",c:Betaproteobacteria,o:Burkholderiales,f:Oxalobacteraceae,g:Undibacterium;

TACGTAGGGTGCAAGCGTTAATCGGAATTACTGGGCGTAAAGCGTGCGCAGGCGGTTATA

TAAGTCAGATGTGAAATCCCCGGGCTCAACCTGGGAACTGCATTTGAGACTGTATGGCTA

GAGTGTGTCAGAGGGGGGTAGAATTCCACGTGTAGCAGTGAAATGCGTAGATATGTGGAG

GAATACCGATGGCGAAGGCAGCCCCCTGGGATAACACTGACGCTCATGCACGAAAGCGTG

GGGAGCAAAC

>ASV299 GS|100.0|EU583729_S001097493;k:Bacteria,p:"Actinobacteria",c:Actinobacteria,o:Actinomycetales,f:Micrococcaceae,g:Arthrobacter;

TACGTAGGGCGCAAGCGTTATCCGGAATTATTGGGCGTAAAGAGCTCGTAGGCGGTTTGT

CGCGTCTGCCGTGAAAGTCCGGGGCTCAACCCCGGATCTGCGGTGGGTACGGGCAGACTA

GAGTGATGTAGGGGAGACTGGAATTCCTGGTGTAGCGGTGAAATGCGCAGATATCAGGAG

GAACACCGATGGCGAAGGCAGGTCTCTGGGCATTAACTGACGCTGAGGAGCGAAAGCATG

GGGAGCGAAC

>ASV300 GSL|99.6|AB245358_S000627894;k:Bacteria,p:"Proteobacteria",c:Betaproteobacteria,o:Burkholderiales,f:Comamonadaceae

TACGTAGGGTGCGAGCGTTAATCGGAATTACTGGGCGTAAAGCGTGCGCAGGCGGTTATG

TAAGACAGTTGTGAAATCCCCGGGCTCAACCTGGGAACTGCATCTGTGACTGCATAGCTA

GAGTACGGTAGAGGGGGATGGAATTCCGCGTGTAGCAGTGAAATGCGTAGATATGCGGAG

GAACACCGATGGCGAAGGCAATCCCCTGGACCTGTACTGACGCTCATGCACGAAAGCGTG

GGGAGCAAAC

>ASV301 GS|0.0|None;No hit

TTCCAGCTCCAATAGCGTATATTAAAGTTGTTGCAGTTAAAAAGCTCGTAGTTGAAACTT

GGGCCTGGCTGACCGGTCCGCCTCACCGCGTGCACTGGTTCGGCCGGGCCTTTCCTTCTG

GGGATGCGCATGGCCTTCATTGGTCGTGTTTGCCGATCCAGGACTTTTACTTTGAAAAAA

TTAGAGTGTTCAAAGCAGGCATATGCTCGAATACATTAGCATGGAATAATAGAATAGGAC

GTGTGGTTCT

>ASV302 SS|1.0000|AM231587_S000650722;k:Bacteria,p:"Proteobacteria",c:Alphaproteobacteria,o:Rhodospirillales,f:Acetobacteraceae

TACGAAGGGGGCTAGCGTTGCTCGGAATGACTGGGCGTAAAGGGCGCGTAGGCGGATTGG

TTAGTCAGACGTGAAATTCCTGGGCTCAACCTGGGGGCTGCGTTTGAGACGGCTAATCTA

GAGTTTGGAAGAGGGTTGTGGAATTCCCAGTGTAGAGGTGAAATTCGTAGATATTGGGAA

GAACACCGGTGGCGAAGGCGGCAACCTGGTCCTTGACTGACGCTGAGGCGCGAAAGCGTG

GGGAGCAAAC

>ASV303 GS|98.4|EF507900_S000859439;k:Bacteria,p:"Bacteroidetes",c:Cytophagia,o:Cytophagales,f:Cytophagaceae,g:Spirosoma;

TACGGAGGGTGCAAGCGTTGTCCGGATTTATTGGGTTTAAAGGGTGCGTAGGTGGGTAGC

TAAGTCTGGTTTGAAAGCAGGTGGCTCAACCATCTGATGTGGCTGGAAACTGGTTATCTT

GAATGGGTTGGCGGTAGCCGGAATGGGTCATGTAGCGGTGAAATGCATAGATATGACCCG

GAACACCGATTGCGAAGGCAGGCTACTACGACTTGATTGACACTGAGGCACGAGAGCATG

GGTAGCGAAC

>ASV304 SS|1.0000|HM032898_S002167666;k:Bacteria,p:"Bacteroidetes",c:Cytophagia,o:Cytophagales,f:Cytophagaceae,g:Hymenobacter

TACGGAGGGTGCGAGCGTTGTCCGGATTTATTGGGTTTAAAGGGTGCGTAGGCGGCTTTT

TAAGTCTGGGGTGAAAGCCCGCTGCTCAACAGCGGAACTGCCCTGGATACTGGAGAGCTT

GAGTACAGACGAGGTTGGCGGAATGGACAGAGTAGCGGTGAAATGCATAGATACTGTCCA

GAACCCCGATTGCGAAGGCAGCTGACTAGGCTGTTACTGACGCTGAGGCACGAAAGCGTG

GGGAGCGAAC

>ASV305 GSL|99.2|EF553529_S000860302;k:Bacteria,p:"Actinobacteria",c:Actinobacteria,o:Actinomycetales,f:Intrasporangiaceae

TACGTAGGGTGCGAGCGTTGTCCGGAATTATTGGGCGTAAAGAGCTTGTAGGCGGTTTGT

CGCGTCTGCTGTGAAAATCCGGGGCTCAACCCCGGACTTGCAGTGGGTACGGGCAGGCTA

GAGTGTGGTAGGGGAGACTGGAATTCCTGGTGTAGCGGTGGAATGCGCAGATATCAGGAG

GAACACCGATGGCGAAGGCAGGTCTCTGGGCCATTACTGACGCTGAGAAGCGAAAGCATG

GGGAGCGAAC

>ASV306 SS|1.0000|AM231587_S000650722;k:Bacteria,p:"Proteobacteria",c:Alphaproteobacteria,o:Rhodospirillales,f:Acetobacteraceae

TACGAAGGGGGCTAGCGTTGCTCGGAATGACTGGGCGTAAAGGGCGCGTAGGCGGCTTGG

TTAGTTAGGCGTGAAATTCCTGGGCTCAACCTGGGGGCTGCGTTTAATACAGCTAGGCTA

GAGTGGGGAAGAGGGTTGTGGAATTCCCAGTGTAGAGGTGAAATTCGTAGATATTGGGAA

GAACACCGGTGGCGAAGGCGGCAACCTGGTCCTTGACTGACGCTGAGGCGCGAAAGCGTG

GGGAGCAAAC

>ASV307 GS|98.4|DQ660892_S000712490;k:Bacteria,p:"Acidobacteria",c:Acidobacteria_Gp1,g:Terriglobus;

TACGAGGGGGGCAAGCGTTGTTCGGAATTATTGGGCGTAAAGGGCGCGTAGGCGGTTTGG

CAAGTTTGGTGTGAAATCTTCGGGCTCAACTCGAAGTCTGCATCGAAAACTGCCGGGCTT

GAGTATGGGAGAGGTGAGTGGAATTTCCGGTGTAGCGGTGAAATGCGTAGATATCGGAAG

GAACACCTGTGGCGAAAGCGGCTCACTGGACCATAACTGACGCTGAGGCGCGAAAGCTAG

GGGAGCAAAC

>ASV308 SS|0.8000|AB072735_S000251887;k:Bacteria,p:"Gemmatimonadetes",c:Gemmatimonadetes,o:Gemmatimonadales,f:Gemmatimonadaceae,g:Gemmatimonas

TACGGAGGGTGCAAGCGTTGTCCGGAATCACTGGGCGTAAAGGGCGCGTAGGTGGCGCGG

TAAGCGTGCGGTGAAAGCCCGGGGCTCAACCCCGGGTCTGCCGTGCGAACTGCTGGGCTG

GAGCATCGTAGAGGCAGGTGGAATTTCGGGTGTAGCGGTGGAATGCGTAGAGATCCGAAA

GAACACCAGTGGCGAAGGCGGCCTGCTGGGCGATTGCTGACACTGAGGCGCGACAGCGTG

GGGAGCAAAC

>ASV309 SS|1.0000|EF457480_S000834905;k:Bacteria

TACGTAGGGGTCGAGCGTTGTCCGGAGTTACTGGGCGTAAAGCGTGTGCAGGCGGCTCAT

TGCGCCCGGCGTGAAAGCCCCCGGCTCAACCGGGGAGGGTCGTCGGGGACGGGTGAGCTT

GAGGGTATCAGGGGCTGGTGGAATTCCCGGTGTAGTGGTGAAATGCGTAGATATCGGGAG

GAACACCCGTGGCGAAGGCGGCCAGCTGGGATACACCTGACGCTGAGACACGAAGGCGTG

GGGAGCGAAC

>ASV310 SS|0.9600|AJ292687_S000017618;k:Bacteria,p:candidate_division_WPS-1,g:WPS-1_genera_incertae_sedis

GACAGAGGTGCCGAGCGTTAGGCGGAATCACTGGGCTTAAAGCGTGTGTAGGCGGATGTC

TAAGTACCTTGTGAAATCCCACGGCTCAACCGTGGAACTGCTCGGTATACTGGACATCTT

GAGACAACTAGGGGCTACCGGAACAAGTGGTGGAGCGGTGAAATGCGTAGATATCACTTG

GAACGCCAATGGCGAAGGCAGGTGGCTGGGGTTGTTCTGACGCTGAGACACGAAAGCCAG

GGGAGCAAAC

>ASV311 GS|100.0|JF799985_S002913220;k:Bacteria,p:"Proteobacteria",c:Betaproteobacteria,o:Burkholderiales,f:Oxalobacteraceae,g:Massilia;

TACGTAGGGTGCAAGCGTTAATCGGAATTACTGGGCGTAAAGCGTGCGCAGGCGGTTTTG

TAAGTCTGTCGTGAAATCCCCGGGCTCAACCTGGGAATTGCGATGGAGACTGCAAGGCTA

GAATCTGGCAGAGGGGGGTAGAATTCCACGTGTAGCAGTGAAATGCGTAGAGATGTGGAG

GAACACCGATGGCGAAGGCAGCCCCCTGGGTCAAGATTGACGCTCATGCACGAAAGCGTG

GGGAGCAAAC

>ASV312 SS|0.8300|EF451726_S000979716;k:Bacteria,p:"Bacteroidetes",c:Cytophagia,o:Cytophagales,f:Cytophagaceae,g:Spirosoma

TACGGAGGGTGCAAGCGTTGTCCGGATTTATTGGGTTTAAAGGGTGCGTAGGTGGGCTAT

CAAGTCTGATTTGAAAGCGAGTCGCTTAACGATTCGATGTGGTTGGAAACTGGTGGTCTT

GAATGGGGTAGCGGTAGCCGGAATGGGTCATGTAGCGGTGAAATGCATAGATATGACCCG

GAACACCGATTGCGAAGGCAGGCTACTGGGCCTTAATTGACACTGAGGCACGAGAGCATG

GGTAGCGAAC

>ASV313 SS|1.0000|AY140238_S000397413;k:Bacteria,p:"Proteobacteria",c:Alphaproteobacteria,o:Rhodospirillales,f:Acetobacteraceae

TACGAAGGGGGCTAGCGTTGCTCGGAATGACTGGGCGTAAAGGGCGCGTAGGCGGACATG

TCAGTCGGACGTGAAATTCCTGGGCTTAACCTGGGGGCTGCGTTCGAGACGGTGTGTCTA

GAGTTTGGAAGAGGGTCGTGGAATTCCCAGTGTAGAGGTGAAATTCGTAGATATTGGGAA

GAACACCGGTGGCGAAGGCGGCGACCTGGTCCTGGACTGACGCTGAGGCGCGAAAGCGTG

GGGAGCAAAC

>ASV314 SS|1.0000|JN090860_S002916046;k:Bacteria,p:"Bacteroidetes",c:Cytophagia,o:Cytophagales,f:Cytophagaceae,g:Hymenobacter

TACGGAGGGTGCGAGCGTTGTCCGGATTTATTGGGTTTAAAGGGTGCGTAGGCGGCCACT

TAAGTCTGGGGTGAAAGCCCGCTGCTCAACAGCGGAACGGCCCTGGATACTGGGTGGCTT

GAGTACAGACGAGGTTGGCGGAATGGACCGAGTAGCGGTGAAATGCATAGATACGGTCCA

GAACCCCGATTGCGAAGGCAGCTGACTAGGCTGTTACTGACGCTGAGGCACGAAAGCGTG

GGGAGCGAAC

>ASV315 SS|1.0000|AJ420142_S000145622;k:Bacteria

GACGTAGGAGGCGAGCGTTGTCCGGAGTTACTGGGCGTAAAGCGCGCGCGGGCGGTCGCG

CAGGTCGCCTGTGAAAGCCCCCGGCTCAACCGGGCGGAGGCGGGCGAAACCGCGCGACTG

GAGGGCGGCAGAGGGTCGTGGAATTCCCGGTGTAGTGGTGAAATGCGTAGAGATCGGGAG

GAACACCCGCGGCGAAGGCGGCGACCTGGACCGACCCTGACGCTGAGGCGCGAAGGCCGG

GGGAGCGAAC

>ASV316 GS|99.6|DQ344632_S000640797;k:Bacteria,p:"Actinobacteria",c:Actinobacteria,o:Actinomycetales,f:Pseudonocardiaceae,g:Pseudonocardia;

TACGTAGGGTGCGAGCGTTGTCCGGAATTATTGGGCGTAAAGAGCTCGTAGGCGGTCTGT

CGCGTCGGTCGTGAAAACTTGGGGCTTAACCCTGAGCTTGCGGTCGATACGGGCATGACT

GGAGTTCGGCAGGGGAGACTGGAATTCCTGGTGTAGCGGTGAAATGCGCAGATATCAGGA

GGAACACCGGTGGCGAAGGCGGGTCTCTGGGCCGATACTGACGCTGAGGAGCGAAAGCGT

GGGGAGCAAA

>ASV317 SS|1.0000|KC213491_S003715425;k:Bacteria,p:"Bacteroidetes",c:Cytophagia,o:Cytophagales,f:Cytophagaceae,g:Hymenobacter

TACGGAGGGTGCGAGCGTTGTCCGGATTTATTGGGTTTAAAGGGTGCGTAGGCGGCTTTG

TAAGTCTGGGGTGAAAGCCCGCTGCTCAACAGCGGAACTGCCCTGGATACTGCGGAGCTT

GAGGACAGACGAGGTTGGCGGAATAGAGGGTGTAGCGGTGAAATGCATAGATACCCTCTA

GAACCCCGATTGCGAAGGCAGCTGACTAGACTGTATCTGACGCTGAGGCACGAAAGCGTG

GGGAGCGAAC

>ASV318 GS|0.0|None;No hit

TTCCAGCTCCAATAGCGTATATTAAAGTTGTTGCAGTTAAAAAGCTCGTAGTTGAAACTT

GGGCCTGGCTGACCGGTCCGCCTCACCGCGTGCACTGGTTCGGCCGGGCCTTTCCTTCTG

GGGATGCGCATGGCCTTCATTGGTCGTGTTGCCGATCCAGGACTTTTACTTTGAAAAAAT

TAGAGTGTTCAAAGCAGGCATATGCTCGAATACATTAGCATGGAATAATAGAATAGGACG

TGTGGTTCTA

>ASV319 SS|1.0000|FN391026_S001418752;k:Bacteria,p:"Planctomycetes",c:Planctomycetia,o:Planctomycetales,f:Planctomycetaceae

GACGAACCGTGCGAACGTTATTCGGAATCACTGGGCTTAAAGCGCGTGTAGGCGGGCCGC

CGCGTCGGCTGCTGAAATCCCCCGGCTCAACCGGGGAACGGGCACCGATACGGGCGGCCT

GGAGGGGGGTAGGGGGGACTGGAACTTCCGGTGGAGCGGTGAAATGCGTTGAGATCGGAA

GGAACGCCCGTGGCGAAAGCGAGTCCCTGGACCCTTTCTGACGCTGAGACGCGAAAGCCA

GGGGAGCGAA

>ASV320 GS|100.0|AB245363_S000627899;k:Bacteria,p:"Proteobacteria",c:Gammaproteobacteria,o:Xanthomonadales,f:Xanthomonadaceae,g:Lysobacter;

TACGAAGGGTGCAAGCGTTACTCGGAATTACTGGGCGTAAAGCGTGCGTAGGTGGTTTGT

TAAGTCTGATGTGAAAGCCCTGGGCTCAACCTGGGAATTGCATTGGATACTGGCAGGCTA

GAGTGCGGTAGAGGATGGCGGAATTCCCGGTGTAGCAGTGAAATGCGTAGAGATCGGGAG

GAACATCTGTGGCGAAGGCGGCCATCTGGACCAGCACTGACACTGAGGCACGAAAGCGTG

GGGAGCAAAC

>ASV321 SS|1.0000|EF457480_S000834905;k:Bacteria

TACGTAGGGTCCGAGCGTTGTCCGGAATTACTGGGCGTAAAGCGTGCGCAGGCGGCCTTC

CAAGCGCCACGTGAAAGCCCCGGGCTCAACCCGGGAGGGTCGTGGCGAACTGGGAGACTT

GAGGCCGGTAGAGGTGGGTGGAACTCCTGGTGGAGTGGTGAAATGCGTAGAGATCAGGAA

GAACACCCGTGGCGAAGGCGGCCCACTGGGCCGGTACTGACGCTGAGGCACGAAGGCGTG

GGGAGCGAAC

>ASV322 GS|100.0|AJ536198_S000110436;k:Bacteria,p:"Actinobacteria",c:Actinobacteria,o:Actinomycetales,f:Micrococcaceae,g:Micrococcus;

TACGTAGGGTGCGAGCGTTATCCGGAATTATTGGGCGTAAAGAGCTCGTAGGCGGTTTGT

CGCGTCTGTCGTGAAAGTCCGGGGCTTAACCCCGGATCTGCGGTGGGTACGGGCAGACTA

GAGTGCAGTAGGGGAGACTGGAATTCCTGGTGTAGCGGTGGAATGCGCAGATATCAGGAG

GAACACCGATGGCGAAGGCAGGTCTCTGGGCTGTAACTGACGCTGAGGAGCGAAAGCATG

GGGAGCGAAC

>ASV323 SS|1.0000|AB362219_S001043921;k:Bacteria,p:"Proteobacteria",c:Alphaproteobacteria,o:Rhodospirillales,f:Acetobacteraceae

TACGAAGGGGGCTAGCGTTGCTCGGAATGACTGGGCGTAAAGGGCGCGTAGGCGGATTGC

ATAGTCAGACGTGAAATTCCTGGGCTCAACCTGGGGGCTGCGTTTGATACGTGCGGTCTA

GAGTTTGGAAGAGGGTTGTGGAATTCCCAGTGTAGAGGTGAAATTCGTAGATATTGGGAA

GAACACCGGTGGCGAAGGCGGCAACCTGGTCCTTGACTGACGCTGAGGCGCGAAAGCGTG

GGGAGCAAAC

>ASV324 SS|1.0000|AY140238_S000397413;k:Bacteria,p:"Proteobacteria",c:Alphaproteobacteria,o:Rhodospirillales,f:Acetobacteraceae

TACGAAGGGGGCTAGCGTTGCTCGGAATGACTGGGCGTAAAGGGCGCGTAGGCGGATGGC

TTTGTCGGGCGTGAAATTCCAGGGCTTAACCTTGGGACTGCGTTCGAGACGGGTTGTCTA

GAGTGGAGAAGAGGGTCGTGGAATTCCCAGTGTAGAGGTGAAATTCGTAGATATTGGGAA

GAACACCGGTGGCGAAGGCGGCGACCTGGTCTTTTACTGACGCTGAGGCGCGAAAGCGTG

GGGAGCAAAC

>ASV325 SS|1.0000|JQ309130_S003619637;k:Bacteria,p:"Acidobacteria",c:Acidobacteria_Gp4

TACGTAGGGACCAAGCGTTGTTCGGATTTACTGGGCGTAAAGGGCGCGTAGGCGGCGTGA

CAAGTCACTTGTGAAATCTCCGGGCTTAACTCGGAACGGTCAAGTGATACTGTTGTGCTA

GAGTATAGAAGGGGCAATCGGAATTCTCGGTGTAGCGGTGAAATGCGTAGATATCGAGAG

GAACACCAGAGGCGAAGGCGGATTGCTAGGCTAATACTGACGCTGAGGCGCGAAAGCTAG

GGTAGCAAAC

>ASV326 SS|1.0000|DQ244076_S000620069;k:Bacteria,p:"Bacteroidetes",c:Sphingobacteriia,o:"Sphingobacteriales",f:Chitinophagaceae

TACGGAGGGTGCAAGCGTTATCCGGATTCACTGGGTTTAAAGGGTGCGTAGGTGGGCAGG

TAAGTCAGTGGTGAAATCTCCGGGCTTAACCCGGAAACTGCCGTTGATACTATTTGTCTT

GAATATTGTGGAGGTAAGCGGAATATGTCATGTAGCGGTGAAATGCTTAGATATGACATA

GAACACCAATTGCGAAGGCAGCTTACTACACAATGATTGACGCTGAGGCACGAAAGCGTG

GGGATCAAAC

>ASV327 SS|1.0000|KJ528316_S004225669;k:Bacteria,p:"Proteobacteria",c:Alphaproteobacteria,o:Sphingomonadales,f:Sphingomonadaceae

TACGGAGGGGGCTAGCGTTGTTCGGAATTACTGGGCGTAAAGCGCACGTAGGCGGTTATT

CAAGTCAGAGGTGAAAGCCCGGAGCTCAACTCCGGAACTGCCTTTGAAACTAGATAACTT

GAATCATGGAGAGGCGAGTGGAATTCCGAGTGTAGAGGTGAAATTCGTAGATATTCGGAA

GAACACCAGTGGCGAAGGCGGCTCGCTGGACATGTATTGACGCTGAGGTGCGAAAGCGTG

GGGAGCAAAC

>ASV328 GS|0.0|None;No hit

CACAAGTAAGATTAGTGTTATTCATCTTTATTAGGTTTAAAGGGTACCTAGACAGCATTT

TGAACTCCTAAGGAGAAAGGATTTGCTAGAGTTTAATGTGAGAGGTAAATATTAGGACTA

TTGGTGTAAAGATGAAATTTTTTGATACTAATAGAATGTGTAATGGCGAAGACAACCCTC

TATGTATAAACTGACGTTAAGGGACGAAGGCTTGGGGAGCAAATAGG

>ASV329 GS|0.0|None;No hit

TACGTAGAAGACAAGTGTTATTCATCTTTAGCAGGTTTAAAGCGTACCTAGACGGGAAAT

CAAGCCATAGAAGGAACTAATTTTCTAGAGTTTTATGTGTGAAGATCGAATTACCTGAAG

AGCAATAAAATGCATTGACACAGGGAAGACGGATAGCAGCGAAGGCAATCTTCTATGTAA

AAACTGACGTTGAGGGACGAAGCCTTGGGTAGCGAGAAGG

>ASV330 GS|97.6|HQ687087_S002989302;k:Bacteria,p:"Acidobacteria",c:Acidobacteria_Gp1,g:Granulicella;

TACGAGGGGGGCAAGCGTTGTTCGGAATTATTGGGCGTAAAGGGTGCGTAGGCGGTTTGA

CAAGTCTTATGTGAAATCTTCGGGCTCAACTCGAAGTCTGCATGGGAAACTGTCGGGCTT

GAGTATGGGAGAGGTGAGTGGAATTTCCGGTGTAGCGGTGAAATGCGTAGATATCGGAAG

GAACACCTGTGGCGAAAGCGGCTCACTGGACCATAACTGACGCTGAGGCACGAAAGCTAG

GGGAGCAAAC

>ASV331 GS|99.2|JQ309130_S003619637;k:Bacteria,p:"Acidobacteria",c:Acidobacteria_Gp4,g:Blastocatella;

TACGTAGGGACCAAGCGTTGTTCGGATTTACTGGGCGTAAAGGGCGCGTAGGCGGCAATT

CAAGTCAGTTGTGAAATCTCCGAGCTTAACTCGGAACGGTCAACTGATACTGCTTTGCTA

GAGTACAGAAGGGGCAATCGGAATTCTTGGTGTAGCGGTGAAATGCGTAGATATCAAGAG

GAACACCTGAGGTGAAGACGGGTTGCTGGGCTGATACTGACGCTGAGGCGCGAAAGCTAG

GGTAGCAAAC

>ASV332 GS|0.0|None;No hit

TACAAGGAAGACTAGTGTTATTCATCTTTATTAGGTTTAAAGGGTACCTAGACGGTTTTG

ATAGCTCCAAAAGAGTACGTTAAAACTAGAGTTTGATAGGAGAGGTAAATATTAGGGCTA

TTGGTGTAGAGATGAAATTCTTTGATACTAATAGAACGGATAAAGGCGAAGGCAACCCTC

TATATATAAACTGACGTTGAGGGACGAAGGCTTGGGGAGCAAATAGG

>ASV333 GSL|100.0|X77434_S000003090;k:Bacteria,p:"Actinobacteria",c:Actinobacteria,o:Actinomycetales,f:Microbacteriaceae,g:Clavibacter

TACGTAGGGTGCAAGCGTTGTCCGGAATTATTGGGCGTAAAGAGCTCGTAGGCGGTTTGT

CGCGTCTGCTGTGAAATCCCGAGGCTCAACCTCGGGTCTGCAGTGGGTACGGGCAGACTA

GAGTGCGGTAGGGGAGATTGGAATTCCTGGTGTAGCGGTGGAATGCGCAGATATCAGGAG

GAACACCGATGGCGAAGGCAGATCTCTGGGCCGTAACTGACGCTGAGGAGCGAAAGCATG

GGGAGCGAAC

>ASV334 SS|1.0000|JF803808_S002913330;k:Bacteria,p:"Bacteroidetes",c:Sphingobacteriia,o:"Sphingobacteriales",f:Chitinophagaceae

TACGGAGGGTGCAAGCGTTATCCGGATTCACTGGGTTTAAAGGGTGCGTAGGTGGGTGAG

TAAGTCAGTGGTGAAATCTCCGGGCTTAACCCGGAAACTGCCATTGATACTATTTGTCTT

GAATGTCGTGGAGGTAAGCGGAATATGTCATGTAGCGGTGAAATGCTTAGATATGACATA

GAACACCAATTGCGAAGGCAGCTTACTACACGGATATTGACACTGAGGCACGAAAGCGTG

GGGATCAAAC

>ASV335 SS|1.0000|GQ368824_S001577957;k:Bacteria,p:"Actinobacteria",c:Actinobacteria,o:Actinomycetales

TACGTAGGGTGCGAGCGTTGTCCGGAATTACTGGGCGTAAAGAGCTCGTAGGCGGTTTGT

CACGTCGTCTGTGAAATCCTAGGGCTTAACCCTGGACGTGCAGGCGATACGGGCTGACTT

GAGTACTACAGGGGAGACTGGAATTTCTGGTGTAGCGGTGGAATGCACAGATATCAGGAA

GAACACCGATGGCGAAGGCAGGTCTCTGGGTAGTAACTGACGCTGAGGAGCGAAAGCATG

GGTAGCGAAC

>ASV336 GS|100.0|AJ438050_S000128467;k:Bacteria,p:"Actinobacteria",c:Actinobacteria,o:Actinomycetales,f:Corynebacteriaceae,g:Corynebacterium;

TACGTAGGGTGCGAGCGTTGTCCGGAATTACTGGGCGTAAAGGGCTCGTAGGTGGTTTGT

CGCGTCGTCTGTGAAATTCCGGGGCTTAACTCCGGGCGTGCAGGCGATACGGGCATAACT

TGAGTACTGTAGGGGTAACTGGAATTCCTGGTGTAGCGGTGAAATGCGCAGATATCAGGA

GGAACACCGATGGCGAAGGCAGGTTACTGGGCAGTTACTGACGCTGAGGAGCGAAAGCAT

GGGTAGCGAA

>ASV337 GS|99.6|AM887757_S000941900;k:Bacteria,p:"Acidobacteria",c:Acidobacteria_Gp1,g:Granulicella;

TACGAGGGGGGCAAGCGTTGTTCGGAATTATTGGGCGTAAAGGGTGCGTAGGCGGCTTGA

TAAGTCTCGTGTGAAATCTCTGGGCTCAACCCAGAGTCTGCACGGGAAACTGTCGGGCTT

GAGTATGGGAGAGGTGAGTGGAATTTCCGGTGTAGCGGTGAAATGCGTAGATATCGGAAG

GAACACCTGTGGCGAAAGCGGCTCACTGGACCATAACTGACGCTGATGCACGAAAGCTAG

GGGAGCAAAC

>ASV338 SS|0.8600|KF694752_S003923295;k:Bacteria,p:"Bacteroidetes",c:Sphingobacteriia,o:"Sphingobacteriales",f:Chitinophagaceae

TACGGAGGGTGCAAGCGTTATCCGGATTTACTGGGTTTAAAGGGTGTGTAGGCGGACTTT

TAAGTCAGAGGTGAAATCCTGAGGCTCAACCTCAGAACTGCCCCTGATACTATTTGTCTT

GAATACTGATGAGGTGGGCGGAACGGGTCATGTAGCGGTGAAATGCTTAGAGATGACCCG

GAACACCGATTGCGAAGGCAGCTCACTGGGCAGTTATTGACGCTGAGGCACGAAAGCGTG

GGGATCAAAC

>ASV339 GS|0.0|None;No hit

TTCCAGCTCCAATAGCGTATATTAAAGTTGTTGCAGTTAAAAAGCTCGTAGTTGAAACTT

GGGCCTGGCTGTCCGGTCCGCCTCACCGCGTGCACTGGTTCGGCCGGGCCTTTCCTTCTG

GGGAGCCGCATGCCCTTCATTGGGTGTGCCGGGGAACCAGGACTTTTACTTTGAAAAAAT

TAGAGTGTTCAAAGCAGGCCTATGCTCGAATACATTAGCATGGAATAATAGAATAGGACG

TGTGGTTCTA

>ASV340 SS|1.0000|HM032897_S002167665;k:Bacteria,p:"Bacteroidetes",c:Cytophagia,o:Cytophagales,f:Cytophagaceae,g:Hymenobacter

TACGGAGGGTGCGAGCGTTGTCCGGATTTATTGGGTTTAAAGGGTGCGTAGGCGGCCGTT

TAAGTCCGGGGTGAAAGCCCGCTGCTCAACAGCGGAACTGCCCTGGATACTGGATGGCTT

GAATACAGTGGAGGGTGGCGGAATGGACCGAGTAGCGGTGAAATGCATAGATACGGTCCA

GAACCCCGATTGCGAAGGCAGCTGCCTACACTGGTATTGACGCTGAGGCACGACAGCGTG

GGGAGCGAAC

>ASV341 SS|1.0000|Z37138_S000001649;k:Bacteria,p:"Actinobacteria",c:Actinobacteria,o:Actinomycetales

TACGTAGGGTGCAAGCGTTGTCCGGAATTACTGGGCGTAAAGAGCTCGTAGGCGGTGTGT

CGCGTCGTCTGTGAAAACCCGTGGCTCAACCACGGGCTTGCAGGCGATACGGGCAGACTT

GAGTATTGCAGGGGAGACTGGAATTCCTGGTGTAGCGGTGAAATGCGCAGATATCAGGAG

GAACACCGGTGGCGAAGGCGGGTCTCTGGGCAAATACTGACGCTGAGGAGCGAAAGCATG

GGTAGCAAAC

>ASV342 SS|1.0000|JQ309130_S003619637;k:Bacteria,p:"Acidobacteria",c:Acidobacteria_Gp4

TACGTAGGGACCAAGCGTTGTTCGGATTTACTGGGCGTAAAGGGCGCGTAGGCGGCGTGA

CAAGTCAATTGTGAAATCTCCGGGCTTAACTCGGAACGGTCAATTGATACTGTTGTGCTA

GAGTACAGAAGGGGCAATCGGAATTCTTGGTGTAGCGGTGAAATGCGTAGATATCAAGAG

GAACACCTGAGGTGAAGACGGGTTGCTGGGCTGATACTGACGCTGAGGCGCGAAAGCTAG

GGTAGCAAAC

>ASV343 GS|100.0|AB042288_S000264635;k:Bacteria,p:"Actinobacteria",c:Actinobacteria,o:Actinomycetales,f:Propionibacteriaceae,g:Propionibacterium;

TACGTAGGGTGCGAGCGTTGTCCGGATTTATTGGGCGTAAAGGGCTCGTAGGTGGTTGAT

CGCGTCGGAAGTGTAATCTTGGGGCTTAACCCTGAGCGTGCTTTCGATACGGGTTGACTT

GAGGAAGGTAGGGGAGAATGGAATTCCTGGTGGAGCGGTGGAATGCGCAGATATCAGGAG

GAACACCAGTGGCGAAGGCGGTTCTCTGGGCCTTTCCTGACGCTGAGGAGCGAAAGCGTG

GGGAGCGAAC

>ASV344 SS|1.0000|EU861928_S001148233;k:Bacteria,p:"Armatimonadetes",c:Armatimonadia,o:Armatimonadales,f:Armatimonadaceae,g:Armatimonas/Armatimonadetes_gp1

TACGTAGGGGGCGAGCGTTGTCCGAAGTTACTGGGCGTAAAGAGCGCGTAGGCGGGTTTT

TAAGTGAGGGGTGAAATTCCGAGGCTTAACCTCGGAACTGCCTTTCATACTGGGAATCTT

GAGTGTGGGAGAGGCGAGTGGAATGGTCGGTGTAGCGGTGAAATGCGTAGATATCGATCG

GAACACCCATGGCGAAGGCAGCTCGCTGGCCCATAACTGACGCTGAGGCGCGAAAGCGTG

GGGAGCAAAC

>ASV345 SS|1.0000|AB267476_S000721190;k:Bacteria,p:"Bacteroidetes",c:Sphingobacteriia,o:"Sphingobacteriales",f:Chitinophagaceae

TACGGAGGGTGCAAGCGTTATCCGGATTCACTGGGTTTAAAGGGTGCGTAGGTGGAGAGG

TAAGTCAGTGGTGAAATCTCCGGGCTTAACCCGGAAACTGCCATTGATACTATCTTTCTT

GAATACCGTGGAGGTGAGCGGAATATGTCATGTAGCGGTGAAATGCTTAGATATGACATA

GAACACCAATTGCGAAGGCAGCTCACTACCCGAATATTGACACTGAGGCACGAAAGCGTG

GGGATCAAAC

>ASV346 GS|0.0|None;No hit

TTCCAGCTCCAATAGCGTATATTAAAGTTGTTGCAGTTAAAAAGCTCGTAGTTGAAACTT

GGGCCTGGCTGGCCGGTCCGCCTAACCGCGTGTACTGGTCCGGCCGGGCCTTTCCTTCTG

GGAAGCCCCATGCCCTTCATTGGGTGTGCGGGGGAACCAGGACTTTTACTTTGAAAAAAT

TAGAGTGTTCAAAGCAGGCCTATGCTCGGATACATTAGCATGGAATAATAGAATAGGACG

TGTGGTTCTA

>ASV347 SS|0.9100|AY902680_S000576842;k:Bacteria,p:"Proteobacteria",c:Alphaproteobacteria,o:Sphingomonadales,f:Sphingomonadaceae

TACGGAGGGGGCTAGCGTTGTTCGGAATTACTGGGCGTAAAGCGTTCGTAGGCGGCTTGC

CAAGTCAGAGGTGAAATCCCACGACTCAATCGTGGAACTGCCTTTGAGACTGGTTGGCTT

GAACACGGGAGAGGTGAGTGGAATTCCGAGTGTAGAGGTGAAATTCGTAGATATTCGGAA

GAACACCAGTGGCGAAGGCGGCTCACTGGACCGTTGTTGACGCTGAGGAACGAAAGCGTG

GGGAGCAAAC

>ASV348 SS|1.0000|AM947653_S001093907;k:Bacteria,p:"Proteobacteria",c:Alphaproteobacteria,o:Rhodospirillales,f:Acetobacteraceae

TACGAAGGGGGCTAGCGTTGCTCGGAATGACTGGGCGTAAAGGGCGCGTAGGCGGACATA

TTAGTCAGGCGTGAAATTCCTGGGCTTAACCTGGGGGCTGCGTTTGATACGGTGTGTCTT

GAGTGGGGAAGAGGGTCGTGGAATTCCCAGTGTAGAGGTGAAATTCGTAGATATTGGGAA

GAACACCGGTGGCGAAGGCGGCGACCTGGTCCTTGACTGACGCTGAGGCGCGAAAGCGTG

GGGAGCAAAC

>ASV349 GS|98.4|AB267478_S000721192;k:Bacteria,p:"Bacteroidetes",c:Sphingobacteriia,o:"Sphingobacteriales",f:Chitinophagaceae,g:Segetibacter;

TACGGAGGGTGCGAGCGTTATCCGGATTCACTGGGTTTAAAGGGTGCGTAGGTGGGAATG

TAAGTCAGTGGTGAAATCTCCGTGCTTAACACGGAAACTGCCATTGATACTATGTTTCTT

GAATTTTCTGGAGGTCAGCGGAATATGTCATGTAGCGGTGAAATGCTTAGATATGACATA

GAACACCAATTGCGAAGGCAGCTGGCTACAGGGATATTGACACTGAGGCACGAAAGCGTG

GGGATCAAAC

>ASV350 GS|0.0|None;No hit

CACGCAGAAGACTAGTGTTATTCATCTTTATTAGGTATAAAGGGTACCTAGACGGATTAT

CAAGCCAAATGAAGGGACTAATTTTCTAGAGTTTTATGGAAGAATATCGAATTGCTGGAG

GAAAGTCAACATTTTTTGATACCAGTGAGACGGACAACTGCGAAGGCGTTATTCTATGTA

AACAACTGACGTTGAGGGACGAAGCCTGGGGGAGCGATAAGG

>ASV351 SS|1.0000|AF268998_S000340459;k:Bacteria,p:Candidatus_Saccharibacteria,g:Saccharibacteria_genera_incertae_sedis

TACGTAGGGCCCGAGCGTTATCCGGAGTGACTGGGCGTAAAGAGTTGCGTAGGTGGCTTG

TAAAGTGAATAGTGAAACCTGGTGGCTCAACCATTCAGACTATTATTCAAACTCACAAGC

TCGAGAGTAGCAGAGGTAACTGGAATTTCTTGTGTAGGAGTGAAATCCGTAGATATAAGA

AGGAACACCAATGGCGTAGGCAGGTTACTGGGCTATTTCTGACACTGAGGCACGAAAGCG

TGGGGAGCGA

>ASV352 SS|0.9400|AJ289983_S000020777;k:Bacteria,p:"Verrucomicrobia",c:Spartobacteria,g:Spartobacteria_genera_incertae_sedis

TACAGAGGCCTCAAGCGTTGTTCGGATTCATTGGGCGTAAAGGGTGCGTAGGCGGTCGGG

TAAGTCGGATGTGAAATCCTGGGGCTCAACCTCAGAACTGCATTCGATACTGCTTGGCTA

GAGGACTGGAGAGGAGATCGGAATTCACGGTGTAGCAGTGAAATGCGTAGATATCGTGAG

GAAGACCAGTGGCGAAGGCGGATCTCTGGACAGTTCCTGACGCTGATGCACGAAGGCTAG

GGGAGCAAAC

>ASV353 SS|0.9900|FN391026_S001418752;k:Bacteria,p:"Planctomycetes",c:Planctomycetia,o:Planctomycetales,f:Planctomycetaceae

GACGAACCGTCCGAACGTTATTCGGAATTACTGGGCTTAAAGCGTGTGTAGGCGGCTCGG

TACGTCCGTTGCTGAAATCCCCCGGCTTAACCGGGGAAGTGGCGTGGATACGGCCAAGCT

GGAGGGAGGTAGGGGGGTCTGGAACTTCCGGTGGAGCGGTGAAATGCGTTGAGATCGGAA

GGAACGCCCGTGGCGAAAGCGAGGCCCTAGACCTTTTCTGACGCTGAGACACGAAAGCTA

GGGGAGCGAA

>ASV354 GS|100.0|AY485602_S000420023;k:Bacteria,p:Firmicutes,c:Bacilli,o:Lactobacillales,f:Streptococcaceae,g:Streptococcus;

TACGTAGGTCCCGAGCGTTGTCCGGATTTATTGGGCGTAAAGCGAGCGCAGGCGGTTAGA

TAAGTCTGAAGTTAAAGGCTGTGGCTTAACCATAGTACGCTTTGGAAACTGTTTAACTTG

AGTGCAAGAGGGGAGAGTGGAATTCCATGTGTAGCGGTGAAATGCGTAGATATATGGAGG

AACACCGGTGGCGAAAGCGGCTCTCTGGCTTGTAACTGACGCTGAGGCTCGAAAGCGTGG

GGAGCAAACA

>ASV355 GS|99.2|EU808006_S001153808;k:Bacteria,p:"Proteobacteria",c:Betaproteobacteria,o:Burkholderiales,f:Oxalobacteraceae,g:Massilia;

TACGTAGGGTGCAAGCGTTAATCGGAATTACTGGGCGTAAAGCGTGCGCAGGCGGTTTTG

TAAGTTTGTCGTGAAAGCCCCGGGCTCAACCTGGGAATTGCGATGAAGACTGCAAGGCTT

GAATCTGGCAGAGGGGGGTAGAATTCCACGTGTAGCAGTGAAATGCGTAGAGATGTGGAG

GAACACCGATGGCGAAGGCAGCCCCCTGGGTCAAGATTGACGCTCATGCACGAAAGCGTG

GGGAGCAAAC

>ASV356 SS|0.9400|EU781657_S001046746;k:Bacteria,p:"Proteobacteria",c:Alphaproteobacteria,o:Sphingomonadales,f:Sphingomonadaceae

TACGGAGGGGGCTAGCGTTATTCGGAATTACTGGGCGTAAAGCGTACGTAGGCGGTGGTT

CAAGTCAGAGGTGAAAGCCCGGAGCTCAACTCCGGAACTGCCTTTGAAACTAGATCGCTA

GAACATCGGAGAGGTAAGTGGAATTCCGAGTGTAGAGGTGAAATTCGTAGATATTCGGAA

GAACACCAGTGGCGAAGGCGGCTTACTGGACGATTGTTGACGCTGAGGTACGAAAGCGTG

GGGAGCAAAC

>ASV357 SS|1.0000|JN090860_S002916046;k:Bacteria,p:"Bacteroidetes",c:Cytophagia,o:Cytophagales,f:Cytophagaceae,g:Hymenobacter

TACGGAGGGTGCGAGCGTTGTCCGGATTTATTGGGTTTAAAGGGTGCGTAGGCGGCCGAA

TAAGTCTGGGGTGAAAGCCCGCTGCTCAACAGCGGAACTGCCCTGGATACTGCTTGGCTT

GAGTACAGACGAGGTTGGCGGAATGGACGGAGTAGCGGTGAAATGCATAGATACCGTCCA

GAACCCCGATTGCGAAGGCAGCTGACTAGGCTGATACTGACGCTGAGGCACGACAGCGTG

GGGAGCGAAC

>ASV358 SS|1.0000|AM947652_S001093906;k:Bacteria,p:"Proteobacteria",c:Alphaproteobacteria,o:Rhodospirillales,f:Acetobacteraceae

TACGAAGGGGGCTAGCGTTGCTCGGAATGACTGGGCGTAAAGGGCGCGTAGGCGGCTTTT

CTAGTCAGGCGTGAAATTCCTGGGCTCAACCTGGGGGCTGCGCTTGATACAGGGAGGCTA

GAGTGGGGAAGAGGGTTGTGGAATTCCCAGTGTAGAGGTGAAATTCGTAGATATTGGGAA

GAACACCGGTGGCGAAGGCGGCAACCTGGTCCTTGACTGACGCTGAGGCGCGAAAGCGTG

GGGAGCAAAC

>ASV359 GS|98.0|KF999686_S004084195;k:Bacteria,p:"Bacteroidetes",c:Cytophagia,o:Cytophagales,f:Cytophagaceae,g:Spirosoma;

TACGGAGGGTGCGAGCGTTGTCCGGATTTATTGGGTTTAAAGGGTGCGTAGGTGGGTGAC

TAAGTCTGGTTTGAAAGCAGGTGGCTCAACCATCTGATGTGGCTGGAAACTGGTGATCTT

GAATGGGTTGGCGGTAGCCGGAACGGGTCATGTAGCGGTGAAATGCATAGATATGACCCA

GAACACCGATTGCGAAGGCAGGCTACTACGACTTGATTGACACTGAGGCACGAGAGCATG

GGTAGCGAAC

>ASV360 SS|1.0000|JX294485_S003614212;k:Bacteria,p:"Bacteroidetes",c:Cytophagia,o:Cytophagales,f:Cytophagaceae,g:Hymenobacter

TACGGAGGGTGCGAGCGTTGTCCGGATTTATTGGGTTTAAAGGGTGCGTAGGCGGCCTCA

TAAGTCTGGGGTGAAAGCCCGCTGCTCAACAGCGGAACTGCCCTGGATACTGTGGGGCTT

GAGGACAGACGAGGTTGGCGGAATGGAGGGTGTAGCGGTGAAATGCATAGATACCCTCCA

GAACCCCGATTGCGAAGGCAGCTGACTAGACTGTAACTGACGCTGAGGCACGAAAGCGTG

GGGAGCGAAC

>ASV361 SS|0.8400|AB072735_S000251887;k:Bacteria,p:"Gemmatimonadetes",c:Gemmatimonadetes,o:Gemmatimonadales,f:Gemmatimonadaceae,g:Gemmatimonas

TACAGAGGGTGCGAGCGTTGTCCGGAATCACTGGGCGTAAAGGGCGCGTAGGTGGCTGAG

TGCGCGTGCCGTGAAAGCCCGGGGCTTAACCCCGGGTCGGCGGTGCGAACGGCTGAGCTG

GAGCATGCGAGAGGCAGGCGGAATTCCGGGTGTAGCGGTGGAATGCGTAGAGATCCGGAA

GAACACCGGGGGCGAAGGCGGCCTGCTGGCGCAGTAGCTGACACTGAGGCGCGACAGCGT

GGGGAGCAAA

>ASV362 SS|1.0000|D86513_S000011943;k:Bacteria,p:"Proteobacteria",c:Alphaproteobacteria,o:Rhodospirillales,f:Acetobacteraceae

TACGAAGGGGGCTAGCGTTGCTCGGAATGACTGGGCGTAAAGGGCGCGTAGGCGGCTTGG

CCAGTTAGGCGTGAAATTCCTGGGCTCAACCTGGGGGCTGCGCTTGATACAGCTAGGCTA

GAGTGGGGAAGAGGGTTGTGGAATTCCCAGTGTAGAGGTGAAATTCGTAGATATTGGGAA

GAACACCGGTGGCGAAGGCGGCAACCTGGTCCTTGACTGACGCTGAGGCGCGAAAGCGTG

GGGAGCAAAC

>ASV363 SS|1.0000|AM947653_S001093907;k:Bacteria,p:"Proteobacteria",c:Alphaproteobacteria,o:Rhodospirillales,f:Acetobacteraceae

TACGAAGGGGGCTAGCGTTGCTCGGAATGACTGGGCGTAAAGGGCGCGTAGGCGGACATT

TTAGTCAGGCGTGAAATTCCTGGGCTTAACCTGGGGGCTGCGTTTGATACGGGGTGTCTA

GAGTGGGGCAGAGGGTCGTGGAATTCCCAGTGTAGAGGTGAAATTCGTAGATATTGGGAA

GAACACCGGTGGCGAAGGCGGCGACCTGGTCCTTGACTGACGCTGAGGCGCGAAAGCGTG

GGGAGCAAAC

>ASV364 SS|1.0000|HM032897_S002167665;k:Bacteria,p:"Bacteroidetes",c:Cytophagia,o:Cytophagales,f:Cytophagaceae,g:Hymenobacter

TACGGAGGGTGCGAGCGTTGTCCGGATTTATTGGGTTTAAAGGGTGCGTAGGCGGCCGTT

TAAGTCTGGGGTGAAAGCCCGTTGCTCAACAGCGGAACTGCCCTGGATACTGGATGGCTT

GAATACAGTGGAGGTTGGCGGAATGGACTGAGTAGCGGTGAAATGCATAGATACAGTCCA

GAACCCCGATTGCGAAGGCAGCTGACTACACTGGTATTGACGCTGAGGCACGACAGCGTG

GGGAGCGAAC

>ASV365 GS|97.6|KF999686_S004084195;k:Bacteria,p:"Bacteroidetes",c:Cytophagia,o:Cytophagales,f:Cytophagaceae,g:Spirosoma;

TACGGAGGGTGCGAGCGTTGTCCGGATTTATTGGGTTTAAAGGGTGCGTAGGTGGGTGAC

TAAGTCTGGTTTGAAAGCAGGTGGCTCAACCATCTGATGTGGCTGGAAACTGGTGATCTT

GAATGGGTTGGCGGTAGCCGGAACGGGTCATGTAGCGGTGAAATGCATAGATATGACCCA

GAACACCGATTGCGAAGGCAGGCTACTAGGACTTGATTGACACTGAGGCACGAGAGCATG

GGTAGCGAAC

>ASV366 SS|1.0000|AB072735_S000251887;k:Bacteria

TACGGGGGGTGCAAGCGTTGTCCGGAATCACTGGGCGTAAAGGGCGCGTAGGTGGTCTTG

TAAGTCTGTGGTGAAAGCCCGGGGCTCAACCCCGGGTCTGCCGTGGATACTGCGAGACTT

GAGTACTGTAGAGGCAGGTAGAATATCGGGTGTAGCGGTGGAATGCGTAGAGATCCGATA

GAAGACCGGTGGCGAAGGCGGCCTGCTGGGCAGTAACTGACACTGAGGCGCGACAGCGTG

GGGAGCAAAC

>ASV367 SS|1.0000|JX949238_S003747851;k:Bacteria,p:"Bacteroidetes",c:Sphingobacteriia,o:"Sphingobacteriales",f:Sphingobacteriaceae

TACGGAGGATCCAAGCGTTATCCGGATTTATTGGGTTTAAAGGGTGCGTAGGCGGCCTGT

TAAGTCAGGGGTGAAATTTTTCGGCTCAACCGGGACATTGCCTTTGATACTGACGGGCTT

GAATACAGTTGAGGTAGGCGGAATGTGACAAGTAGCGGTGAAATGCATAGATATGTCACA

GAACACCAATTGCGAAGGCAGCTTACCAAAGTGTGATTGACGCTGAGGCACGAAAGCGTG

GGGATCAAAC

>ASV368 SS|1.0000|EF516412_S000840854;k:Bacteria,p:"Armatimonadetes",c:Armatimonadia,o:Armatimonadales,f:Armatimonadaceae,g:Armatimonas/Armatimonadetes_gp1

TACGTAGGGGGCCAGCGTTGTTCGAAGTTACTGGGCGTAAAGAGCGCGTAGGCGGACTTT

TAAGTGAGGGGTGAAAGGTTCAGGGCTTAACCCGGACACTGCCTTTTATACTGGAAGTCT

TGAGTGTAGGAGAGGCGAGTGGAATGGTCGGTGTAGCGGTGAAATGCGTAGATATCGATC

GGAACACCCATGGCGAAGGCAGCTCGCTGGCCTATAACTGACGCTGAGGCGCGAAAGCGT

GGGGAGCAAA

>ASV369 GS|98.4|AJ429239_S000145113;k:Bacteria,p:"Proteobacteria",c:Alphaproteobacteria,o:Sphingomonadales,f:Sphingomonadaceae,g:Sphingomonas;

TACGGAGGGAGCTAGCGTTATTCGGAATTACTGGGCGTAAAGCGCACGTAGGCGGCTTTG

TAAGTAAGAGGTGAAAGCCCGGGGCTCAACCCCGGAATTGCCTTTTAGACTGCATCGCTT

GAATCATGGAGAGGTCAGTGGAATTCCGAGTGTAGAGGTGAAATTCGTAGATATTCGGAA

GAACACCAGTGGCGAAGGCGGCTGACTGGACATGTATTGACGCTGAGGTGCGAAAGCGTG

GGGAGCAAAC

>ASV370 SS|0.9900|D86511_S000414138;k:Bacteria,p:"Proteobacteria",c:Alphaproteobacteria,o:Rhodospirillales,f:Acetobacteraceae

TACGAAGGGGGCTAGCGTTGCTCGGAATGACTGGGCGTAAAGGGCGCGTAGGCGGTTTGT

TTAGTTAGGCGTGAAATTCCTGGGCTTAACCTGGGGACTGCGTTTAATACAGGCAGACTT

GAGTTCGGAAGAGGGTTGTGGAATTCCCAGTGTAGAGGTGAAATTCGTAGATATTGGGAA

GAACACCGGTGGCGAAGGCGGCAACCTGGTCCGATACTGACGCTGAGGCGCGAAAGCGTG

GGGAGCAAAC

>ASV371 GS|97.6|FJ194436_S001188468;k:Bacteria,p:"Proteobacteria",c:Alphaproteobacteria,o:Sphingomonadales,f:Sphingomonadaceae,g:Sphingomonas;

TACGGAGGGGGCTAGCGTTGTTCGGAATTACTGGGCGTAAAGCGCACGTAGGCGGCTTTG

TAAGTTAGAGGTGAAAGCCTGGAGCTCAACTCCAGAATTGCCTTTAAGACTGCATCGCTT

GAATCCGAGAGAGGTGAGTGGAATTCCGAGTGTAGAGGTGAAATTCGTAGATATTCGGAA

GAACACCAGTGGCGAAGGCGGCTCACTGGCTCGGTATTGACGCTGAGGTGCGAAAGCGTG

GGGAGCAAAC

>ASV372 GS|99.6|DQ347958_S000641302;k:Bacteria,p:Cyanobacteria/Chloroplast,c:Chloroplast,f:Chloroplast,g:Streptophyta;

TACAGAGGATGCAAGCGTTATCCGGAATGATTGGGCGTAAAGCGTCTGTAGGTGGCTTTT

TAAGTCCGCCGTCAAATCCCAGGGCTCAACCCTGGACAGGCGGTGGAAACTACCAAGCTG

GAGTACGGTAGGGGCAGAGGGAATTTCCGGTGGAGCGGTGAAATGCGTAGAGATCGGAAA

GAACACCAACGGCGAAAGCACTCTGCTGGGCCGACACTGACACTGAGAGACGAAAGCTAG

GGGAGCAAAT

>ASV373 SS|1.0000|HM032898_S002167666;k:Bacteria,p:"Bacteroidetes",c:Cytophagia,o:Cytophagales,f:Cytophagaceae,g:Hymenobacter

TACGGAGGGTGCGAGCGTTGTCCGGATTTATTGGGTTTAAAGGGTGCGTAGGCGGCTTTT

TAAGTCTGGGGTGAAAGCCCGCTGCTCAACAGCGGAACGGCCCTGGATACTGAGGAGCTT

GAATACAGTGGAGGTTGGCGGAATGGACGGAGTAGCGGTGAAATGCATAGATACCGTCCA

GAACCCCGATTGCGAAGGCAGCTGACTACACTGGTATTGACGCTGAGGCACGACAGCGTG

GGGAGCGAAC

>ASV374 GSL|97.2|AJ871304_S000460115;k:Bacteria,p:"Actinobacteria",c:Actinobacteria,o:Actinomycetales

TACGTAGGGTGCAAGCGTTGTCCGGAATTATTGGGCGTAAAGAGCTCGTAGGCGGTCTGT

CACGTCGGCTGTGAAAACCCGAGGCTCAACCTCGGGCCTGCAGTCGATACGGGCAGACTA

GAGTTCGGTAGGGGAGACTGGAATTCCTGGTGTAGCGGTGAAATGCGCAGATATCAGGAG

GAACACCGGTGGCGAAGGCGGGTCTCTGGGCCGAAACTGACGCTGAGGAGCGAAAGCGTG

GGGAGCAAAC

>ASV375 SS|0.9900|AM947653_S001093907;k:Bacteria,p:"Proteobacteria",c:Alphaproteobacteria,o:Rhodospirillales,f:Acetobacteraceae

TACGAAGGGGGCTAGCGTTGCTCGGAATGACTGGGCGTAAAGGGCGCGTAGGCGGATGTT

TTAGTCAGGCGTGAAAGTCCTGGGCTCAACCTGGGGATTGCGTTTGATACGGGGCATCTA

GAGTTGGGAAGAGGGTCGTGGAATTCCCAGTGTAGAGGTGAAATTCGTAGATATTGGGAA

GAACACCGGTGGCGAAGGCGGCGACCTGGTCTTTGACTGACGCTGAGGCGCGAAAGCGTG

GGGAGCAAAC

>ASV376 GS|0.0|None;No hit

GTGTCAGCAGCCCAACCGAAATCCTCCGCTACACCGAGCTCCAAAAACATGGCTGTCTCA

AAGACATCCATTCGTTCCTCAGTGGCGTCATCGTCAAGCTTTACCATCATTTCTTCCCAA

TGCGTATCATGCACTGTACGTGAAGGGAGCGGGCTGCCCAGCCGACAAGATAATATGCTA

GGAATTTTCGACTGCGCCACAGGTTCTCGTAGTCC

>ASV377 SS|0.9900|EU861876_S001148181;k:Bacteria,p:"Armatimonadetes",c:Armatimonadia,o:Armatimonadales,f:Armatimonadaceae,g:Armatimonas/Armatimonadetes_gp1

TACGTAGGGGGCGAGCGTTGTCCGAAGTTACTGGGCGTAAAGAGCGCGTAGGCGGCCTCT

TAAGTGTGGGGTGAAAGTCCGGGGCTCAACCCCGGAACTGCCTCGCAAACTGGGGGGCTT

GAGTGCGGGAGGGGCGAGTGGAATGGTCGGTGTAGCGGTGAAATGCGTAGATATCGATCG

GAACACCCATGGCGAAGGCAGCTCGCTGGCCTGTAACTGACGCTGAGGCGCGAAAGCGTG

GGGAGCAAAC

>ASV378 SS|1.0000|D86513_S000011943;k:Bacteria,p:"Proteobacteria",c:Alphaproteobacteria,o:Rhodospirillales,f:Acetobacteraceae

TACGAAGGGGGCTAGCGTTGCTCGGAATGACTGGGCGTAAAGGGCGCGTAGGCGGATGGC

ACAGTCAGATGTGAAATTCCCGGGCTTAACCTGGGGGCTGCATTTGATACGTGTTGTCTA

GAGTGAGGAAGAGGGTCGTGGAATTCCCAGTGTAGAGGTGAAATTCGTAGATATTGGGAA

GAACACCGGTGGCGAAGGCGGCGACCTGGTCCTTAGACTGACGCTGAGGCGCGAAAGCGT

GGGGAGCAAA

>ASV379 GS|98.4|DQ660892_S000712490;k:Bacteria,p:"Acidobacteria",c:Acidobacteria_Gp1,g:Terriglobus;

TACGAGGGGGGCAAGCGTTGTTCGGAATTATTGGGCGTAAAGGGCGCGTAGGCGGTTTGG

CAAGTTTCGTGTGAAATCTTCAGGCTCAACCTGAAGTCTGCACGGAAAACTGCCGGGCTT

GAGTATGGGAGAGGTGAGTGGAATTTCCGGTGTAGCGGTGAAATGCGTAGATATCGGAAG

GAACACCTGTGGCGAAAGCGGCTCACTGGACCATAACTGACGCTGAGGCGCGAAAGCTAG

GGGAGCAAAC

>ASV380 GS|0.0|None;No hit

TTCCAGCTCCAATAGCGTATATTAAAGTTGTTGCAGTTAAAAAGCTCGTAGTTGAAACTT

GGGCCTGGCTGTCCGGTCCGCCTCACCGCGTGCACTGGTTCGGCCGGGCCTTTCCTTCTG

GGGAGCCGCATGCCCTTCATTGGGTGTGCTGGTCAACCAGGACTTTTACTTTGAAAAAAT

TAGAGTGTTCAAAGCAGGCCTATGCTCGAATACATTAGCATGGAATAATAGAATAGGACG

TGTGGTTCTA

>ASV381 SS|0.9500|AM887756_S000941899;k:Bacteria,p:"Acidobacteria",c:Acidobacteria_Gp1,g:Granulicella

TACGAGGGGGGCAAGCGTTGTTCGGATTTATTGGGCGTAAAGGGTGCGTAGGCGGTTTGA

TAAGTCTTATGTGAAATCTATGGGCTCAACCCATAGTCTGCATGAGAAACTGTCGGGCTT

GAGTATTGGAGAGGTGAGTGGAATTTCCGGTGTAGCGGTGAAATGCGTAGATATCGGAAG

GAACACCTGTGGCGAAAGCGGCTCACTGGACAATAACTGACGCTGAGGCACGAAAGCTAG

GGGAGCAAAC

>ASV382 GS|0.0|None;No hit

GTGTCAGCAGCCGCGGTGGGCGAGAAAGCATCAGTAGAATACCGATGTGGTCCTCCACCG

TAGCTATTCCTGTTGTCGTCACTCTGTTGCCCCGCAAAGTCCGAAAAGTACACTTGCTGT

GAGTCCAACATGGTTGATTGCCTCGTCTGAATTCCATTCTGCGACCCGTAGTCC

>ASV383 SS|1.0000|DQ422812_S001020357;k:Bacteria,p:Cyanobacteria/Chloroplast

GACGGAGGATGCAAGCGTTATCCGGAATGATTGGGCGTAAAGCGTCTGTAGGTGGATTGT

AAAGTCCTCTGTTAAAGATCTGGGCTTAACCCAGTTCAAGCAGTGGAAACTTATAATCTA

GAGTACGGTAGGGGCAGAGGGAATTCCCGGTGTAGCGGTGAAATGCGTAGATATCGGGAA

GAACACCGACAGCGAAAGCACTCTGCTGGGCCGAAACTGACACTGAGAGACGAAAGCTAG

GGGATCAAAG

>ASV384 GS|98.4|AB267478_S000721192;k:Bacteria,p:"Bacteroidetes",c:Sphingobacteriia,o:"Sphingobacteriales",f:Chitinophagaceae,g:Segetibacter;

TACGGAGGGTGCAAGCGTTATCCGGATTCACTGGGTTTAAAGGGTGCGTAGGTGGGGATG

TAAGTCAGTGGTGAAATCTCCGTGCTTAACACGGAAACTGCCATTGATACTATGTTTCTT

GAATTTTCTGGAGGTCAGCGGAATATGTCATGTAGCGGTGAAATGCTTAGATATGACATA

GAACACCAATTGCGAAGGCAGCTGGCTACAGGGATATTGACACTGAGGCACGAAAGCGTG

GGGATCAAAC

>ASV385 SS|0.9700|X64380_S000749517;k:Bacteria,p:"Verrucomicrobia",c:Spartobacteria,g:Spartobacteria_genera_incertae_sedis

TACAGAGGTCCCAAGCGTTGTTCGGATTCATTGGGCGTAAAGGGTGCGCAGGCGGTTAGG

TAAGTCAGGTGTGAAATCTCAGGGCTCAACCCTGAAACTGCATTTGATACTGCTTGACTA

GAGGACTGGAGAGGTGATCGGAATTCACGGTGTAGCAGTGAAATGCGTAGATATCGTGAG

GAAGGCCAGCGGCGAAGGCGGATCACTGGACAGTTCCTGACGCTCAGGCACGAAGGCCAG

GGGAGCAAAC

>ASV386 GS|78.5|GQ487984_S001683783;k:Bacteria,p:"Armatimonadetes",c:Chthonomonadetes,o:Chthonomonadales,f:Chthonomonadaceae,g:Chthonomonas/Armatimonadetes_gp3;

TACGTAGGTGGCAAGCGTTGTCCGGATTTACTGGGTGTAAAGGGTGCGCAGGCGGGCCAT

TAAGTAGAAAGTGAAAGGTTGGAGCTCAACTCCGACACTGCTTTCTATACTGGTGGCCTT

GAGTCTTGGAGGGGGAAGCGGAACGACACGTGTAGCGGTGAAATGCGTTGATATGTGTCG

GAACACCAATGGCGAAAGCAGCTTCCTGGACAAGTACTGACGCTCAGGCACGAAAGCCAA

GGTAGCAAAC

>ASV387 SS|1.0000|D86512_S000010801;k:Bacteria,p:"Proteobacteria",c:Alphaproteobacteria,o:Rhodospirillales,f:Acetobacteraceae

TACGAAGGGGGCTAGCGTTGCTCGGAATGACTGGGCGTAAAGGGCGCGTAGGCGGATCAG

CCAGTCGGGCGTGAAATTCCTGGGCTTAACCTGGGGGCTGCGTTCGAGACGGTTGGTCTA

GAGTGGGGAAGAGGGTCGTGGAATTCCCAGTGTAGAGGTGAAATTCGTAGATATTGGGAA

GAACACCGGTGGCGAAGGCGGCGACCTGGTCCTTGACTGACGCTGAGGCGCGAAAGCGTG

GGGAGCAAAC

>ASV388 GS|78.8|AJ009456_S000115949;k:Bacteria,p:"Armatimonadetes",c:Chthonomonadetes,o:Chthonomonadales,f:Chthonomonadaceae,g:Chthonomonas/Armatimonadetes_gp3;

TACGTAGGTGGCAAGCGTTGTCCGGATTTACTGGGCGTAAAGCGAACGCAGGCGGACTGT

TAAGTAGAAAGTGAAAGGTCGGAGCTCAACTCCAACATTGCTTCCTATACTGGCAGTCTT

GAGTAGCGGAGAGGAAGGGGGAACGACACGTGTAGCGGTGAAATGCGTTGATATGTGTCG

GAACACCCATGGCGAAAGCACCCTTCTGGACGCAAACTGACGCTCAGGTTCGAAAGCCAA

GGTAGCGAAC

>ASV389 SS|1.0000|D86513_S000011943;k:Bacteria,p:"Proteobacteria",c:Alphaproteobacteria,o:Rhodospirillales,f:Acetobacteraceae

TACGAAGGGGGCTAGCGTTGCTCGGAATGACTGGGCGTAAAGGGCGCGTAGGCGGATCGA

TCAGTCGGGCGTGAAATTCCCGGGCTCAACCTGGGGACTGCGTTCGAGACGGTTGGTCTA

GAGTGGGGAAGAGGGTCGTGGAATTCCCAGTGTAGAGGTGAAATTCGTAGATATTGGGAA

GAACACCGGTGGCGAAGGCGGCGACCTGGTCCTTGACTGACGCTGAGGCGCGAAAGCGTG

GGGAGCAAAC

>ASV390 GS|97.2|AJ292684_S000017061;k:Bacteria,p:candidate_division_WPS-2,g:WPS-2_genera_incertae_sedis;

GACGTAGGGGGCAAGCGTTATTCGGAATTATTGGGCGTAAAGCGCTCGTAGGCGGGACAG

GAAGTCCGTGAAGAAAGGCCTGGGCTCAACTCAGGGAACGGCACGGATACTCTTGTTCTT

GAGGCAATCAGAGGGTGATGGAATTCCCGGTGTAGCGGTGAAATGCGTAGATATCGGGAG

GAACACCAGTGGCGAAGGCGATCACCTGGGGTTGTTCTGACGCTGAGGAGCGAAAGCTAG

GGGAGCAAAC

>ASV391 GS|97.2|HQ436503_S002339683;k:Bacteria,p:"Proteobacteria",c:Alphaproteobacteria,o:Rhodospirillales,f:Acetobacteraceae,g:Roseomonas;

TACGAAGGGGGCTAGCGTTGCTCGGAATTACTGGGCGTAAAGGGCGCGTAGGCGGCACTG

CTCGTCAGGCGTGAAATTCCTGGGCTCAACCTGGGGACTGCGCTTGATACGGCAGAGCTA

GAGGATGGAAGAGGGTCGTGGAATTCCCAGTGTAGAGGTGAAATTCGTAGATATTGGGAA

GAACACCGGTGGCGAAGGCGGCGACCTGGTCCATTACTGACGCTGAGGCGCGACAGCGTG

GGGAGCAAAC

>ASV392 SS|0.9900|AJ292581_S000022859;k:Bacteria,p:"Acidobacteria",c:Acidobacteria_Gp1

TACGAGGGGGGCAAGCGTTGTTCGGAATTATTGGGCGTAAAGGGTGCGTAGGCGGCCCCG

CAAGTCCTGTGTGAAATCCTCAAGCTCAACTTGAGGACTGCACGGGAAACTGCTGGGCTG

GAGTATGGGAGAGGTGAGTGGAATTCCTGGTGTAGCGGTGAAATGCGTAGATATCAGGAG

GAACACCTGTGGCGAAAGCGGCTCACTGGACCATAACTGACGCTGAGGCACGAAAGCTAG

GGGAGCAAAC

>ASV393 SS|1.0000|JX949238_S003747851;k:Bacteria,p:"Bacteroidetes",c:Sphingobacteriia,o:"Sphingobacteriales",f:Sphingobacteriaceae

TACGGAGGATCCAAGCGTTATCCGGATTTATTGGGTTTAAAGGGTGCGTAGGCGGCCTGT

TAAGTCAGGGGTGAAATTTTTCGGCTCAACCGGAACATTGCCTTTGATACTGACGGGCTT

GAATGCAGCTGAGGTAGGCGGAATGTGACAAGTAGCGGTGAAATGCATAGATATGTCACA

GAACACCAATTGCGAAGGCAGCTTACTAAAGTGTGATTGACGCTGAGGCACGAAAGCGTG

GGGATCAAAC

>ASV394 GS|0.0|None;No hit

GTGTCAGCAGCCGCCAGATAGCAGTCCCGTTAAGAAGACTGCACGGTCAAGAGGAAAGAG

CAAGGCTCGACCATCACCACCACCACCGCCGCCGGAAGGTGGAAACATGTCGCTGGATCA

GGCCGTTGCTCAAGGGAGAGGGTCATGCATTTTCCGGTATTTGAATGGTGCTGCGCCTGT

CGTGTATGGCATCCCTGTTTTTGTACAGTGAACGTGTCTTGGGTCTTGGGTTCGCTGGCG

GTGCATGAGA

>ASV395 SS|1.0000|AB362776_S000964290;k:Bacteria,p:"Bacteroidetes",c:Sphingobacteriia,o:"Sphingobacteriales",f:Chitinophagaceae

TACGGAGGGTGCAAGCGTTATCCGGATTCACTGGGTTTAAAGGGTGCGTAGGCGGGCAAG

TAAGTCAGTGGTGAAATCTCCAGGCTTAACCTGGAAACTGCCATTGATACTATTTGTCTT

GAATTACGTGGAGGTGAGCGGAATATGTCATGTAGCGGTGAAATGCTTAGATATGACATA

GAACACCAATTGCGAAGGCAGCTCACTACACGTTGATTGACGCTGAGGCACGAAAGCGTG

GGGATCAAAC

>ASV396 SS|0.9400|AJ292582_S000020307;k:Bacteria,p:"Acidobacteria",c:Acidobacteria_Gp3,g:Gp3

TACGTAGGCAGCGAGCGTTGTTCGGAATTACTGGGCGTAAAGAGTCTGTAGGCGGTGTTC

TAAGTTCGGTGTGAAATCTCCTGGCTTAACTGGGAGGGTGCGCCGAAAACTGGAATGCTT

GAACGTCGGAGAGGAAAGCGGAATTCCTGGTGTAGCGGTGAAATGCGTAGATATCAGGAG

GAACACCTGTGGTGTAGACGGCTTTCTGGACGATTGTTGACGCTGAGAGACGAAAGCGTG

GGTAGCAAAC

>ASV397 SS|1.0000|HM032898_S002167666;k:Bacteria,p:"Bacteroidetes",c:Cytophagia,o:Cytophagales,f:Cytophagaceae,g:Hymenobacter

TACGGAGGGTGCGAGCGTTGTCCGGATTTATTGGGTTTAAAGGGTGCGTAGGCGGCCGTT

TAAGTCTGGGGTGAAAGCCCGCTGCTCAACAGCGGAACTGCCCTGGATACTGGATGGCTT

GAGTACAGACGAGGTTGGCGGAATGGACTGAGTAGCGGTGAAATGCATAGATACAGTCCA

GAACCCCGATTGCGAAGGCAGCTGACTAGGCTGATACTGACGCTGAGGCACGACAGCGTG

GGGAGCGAAC

>ASV398 GS|75.9|X99562_S000381430;k:Archaea,p:"Crenarchaeota",c:Thermoprotei,o:Desulfurococcales,f:Desulfurococcaceae,g:Ignicoccus;

CTTGGTCATTTAGAGGAAGTAAAAGTCGTAACAAGGTTTCCGTAGGTGAACCTGCGGAAG

GATCATTACCGAGAGCGGGGCTAACCCCCCAAACTCCGCCGCCGAAAGGGGTACTCTCCA

CCCTATGTATACATATACCACTCTCGCTTTGGCGGGCTGAAGGCTCTTGCCCTACGCCCG

CCAGTGGCTCAAAAAATTCTGTTTATCAGTGATGTCCGAGTAAAAACCTAATAGTTAAAA

CTTTCAACAA

>ASV399 GSL|98.4|AB064317_S000384110;k:Bacteria,p:"Proteobacteria",c:Betaproteobacteria,o:Burkholderiales,f:Comamonadaceae

TACGTAGGGTGCAAGCGTTAATCGGAATTACTGGGCGTAAAGCGTGCGCAGGCGGTTTTG

TAAGACAGATGTGAAATCCCCGGGCTTAACCTGGGAATTGCATTTGTGACTGCAAGGCTG

GAGTGCGGCAGAGGGGGATGGAATTCCGCGTGTAGCAGTGAAATGCGTAGATATGCGGAG

GAACACCGATGGCGAAGGCAATCCCCTGGGCCTGCACTGACGCTCATGCACGAAAGCGTG

GGGAGCAAAC

>ASV400 GS|0.0|None;No hit

GTGTCAGCAGCCGCCCCGAAACATACTCATGCGACAGGCCATGAAAGCCGTAACGCCGCA

GGCCAAAGCGCTCCGACAGCGCACCCGGTAAGGCATAAGTGTACGCGACTTCGGGCAAAG

TCCGGTGAAACGCTGTATCAAAGCAAGCGACTTCCGGCACATCCGGCAGCAGCTTCTGCG

TCGCTTCAATCAGCCCGATTTCGCCGGGATTATGCAGCGGGTCCAGCCCTTCGAGTGCCT

TCAAGTCTTC

>ASV401 SS|1.0000|AY140238_S000397413;k:Bacteria,p:"Proteobacteria",c:Alphaproteobacteria,o:Rhodospirillales,f:Acetobacteraceae

TACGAAGGGGGCTAGCGTTGCTCGGAATGACTGGGCGTAAAGGGCGCGTAGGCGGATTTG

ACAGTCGGGCGTGAAATTCCTGGGCTTAACCTGGGGGCTGCGTTCGAGACGTTGGGTCTA

GAGTTTGGAAGAGGGTTGTGGAATTCCCAGTGTAGAGGTGAAATTCGTAGATATTGGGAA

GAACACCGGTGGCGAAGGCGGCGACCTGGTCCTTGACTGACGCTGAGGCGCGAAAGCGTG

GGGAGCAAAC

>ASV402 GS|100.0|AJ585986_S000247788;k:Bacteria,p:"Deinococcus-Thermus",c:Deinococci,o:Deinococcales,f:Deinococcaceae,g:Deinococcus;

TACGGAGGGTGCAAGCGTTACCCGGAATCACTGGGCGTAAAGGGCGTGTAGGCGGCATCA

CAAGTCTGGTTTTAAAGCCTGCGGCTCAACCGCAGAGATGGACTGGAGACTGTGAAGCTA

GACCTCTGGAGAGAGAACTGGAATTCCTGGTGTAGCGGTGGAATGCGTAGATACCAGGAG

GAACACCGATGGCGAAGGCAGGTTCTTGGACAGAAGGTGACGCTGAGGCGCGAAAGTGTG

GGGAGCAAAC

>ASV403 SS|1.0000|EF457312_S000834737;k:Bacteria

TACGTAGGATGCGAGCGTTGTCCGGAATTACTGGGCGTAAAGAGCGCGTAGGCGGCGCTG

TACGACCGCGGTGAAAGCCCCCGGCTCAACTGGGGAGGGTCCGTGGTAACGGCAGTGCTC

TGAGTGCAGGAGAGGGAAGTGGAACTCCGGGAGTAGCGGTGAAATGCGTAGAGACCCGGA

GGAACACCAGTGGCGAAGGCGGCTTCCTGGTCTGCGACTGACGCTGTGGCGCGAAAGCTA

GGGGAGCAAA

>ASV404 GS|0.0|None;No hit

TTCCAGCTCCAATAGCGTATATTAAAGTTGTTGCAGTTAAAAAGCTCGTAGTTGAACTTT

GGGCCTGGCTAGCCGGTCCGCCTCACCGCGTGTACTGGTCCGGCTGGGCCTTTCCTTCTG

GGGATCCGCATGCCCTTCACTGGGTGTGTCGGGGAACCAGGACTTTTACTTTGAAAAAAT

TAGAGTGTTCAAAGCAGGCATTTGCTCGAATACCTTAGCATGGAATAATAGAATAGGACG

TGCGGTTCTA

>ASV405 GS|97.6|AB033947_S000005649;k:Bacteria,p:"Proteobacteria",c:Alphaproteobacteria,o:Sphingomonadales,f:Sphingomonadaceae,g:Sphingomonas;

TACGGAGGGGGCTAGCGTTGTTCGGAATTACTGGGCGTAAAGCGCACGTAGGCGGCTTTG

TAAGTCAGAGGTGAAATCCCGGGGCTCAACCCCGGAACTGCCTTTGAGACTGCATCGCTC

GAATCCAGGAGAGGTGAGTGGAATTCCGAGTGTAGAGGTGAAATTCGTAGATATTCGGAA

GAACACCAGTGGCGAAGGCGGCTCACTGGACTGGTATTGACGCTGAGGTGCGAAAGCGTG

GGGAGCAAAC

>ASV406 GS|97.2|AB377116_S000994777;k:Bacteria,p:"Actinobacteria",c:Actinobacteria,o:Actinomycetales,f:Kineosporiaceae,g:Kineosporia;

TACGTAGGGTGCAAGCGTTGTCCGGAATTATTGGGCGTAAAGAGCTCGTAGGCGGTTCGT

CGCGTCTGCTGTGAAAACCTGGGGCTCAACCCCGGGCGTGCAGTGGGTACGGGCGGGCTA

GAGTGCAGTAGGGGAGACTGGAATTCCTGGTGTAGCGGTGAAATGCGCAGATATCAGGAG

GAACACCGGTGGCGAAGGCGGGTCTCTGGGCTGTTACTGACGCTGAGGAGCGAAAGCATG

GGGAGCGAAC

>ASV407 SS|1.0000|D86513_S000011943;k:Bacteria,p:"Proteobacteria",c:Alphaproteobacteria,o:Rhodospirillales,f:Acetobacteraceae

TACGAAGGGGGCTAGCGTTGCTCGGAATGACTGGGCGTAAAGGGCGCGTAGGCGGATTGG

TCAGTCAGGCGTGAAATTCCTGGGCTTAACCTGGGGTGTGCGTTTGAGACGGCTGGTCTG

GAGTGGGGAAGAGGGTCGTGGAATTCCCAGTGTAGAGGTGAAATTCGTAGATATTGGGAA

GAACACCGGTGGCGAAGGCGGCGACCTGGTCCTTGACTGACGCTGAGGCGCGAAAGCGTG

GGGAGCAAAC

>ASV408 SS|1.0000|AB369258_S001151836;k:Bacteria,p:"Proteobacteria",c:Alphaproteobacteria,o:Rhodospirillales,f:Acetobacteraceae

TACGAAGGGGGCTAGCGTTGCTCGGAATGACTGGGCGTAAAGGGCGCGTAGGCGGATCTG

TCAGTCAGGCGTGAAATTCCTGGGCTTAACCTGGGGGCTGCGTTTGAGACGGTGGGTCTA

GAGTTTGGAAGAGGGTCGTGGAATTCCCAGTGTAGAGGTGAAATTCGTAGATATTGGGAA

GAACACCGGTGGCGAAGGCGGCGACCTGGTCCTGGACTGACGCTGAGGCGCGAAAGCGTG

GGGAGCAAAC

>ASV409 SS|1.0000|FN391026_S001418752;k:Bacteria,p:"Planctomycetes",c:Planctomycetia,o:Planctomycetales,f:Planctomycetaceae

GACGAACCGTCCGAACGTTATTCGGAATTACTGGGCTTAAAGCGTGTGTAGGCGGCTCGG

TACGTCCGTTGCTGAAATCCCCCGGCTTAACCGGGGAAGTGGCGTGGATACGGCCGAGCT

GGAGGGAGGTAGGGGGGTCTGGAACTTCCGGTGGAGCGGTGAAATGCGTTGAGATCGGAA

GGAACGCCCGTGGCGAAAGCGAGGCCCTAGACCTTTTCTGACGCTGAGACACGAAAGCTA

GGGGAGCGAA

>ASV410 SS|1.0000|HM032898_S002167666;k:Bacteria,p:"Bacteroidetes",c:Cytophagia,o:Cytophagales,f:Cytophagaceae,g:Hymenobacter

TACGGAGGGTGCGAGCGTTGTCCGGATTTATTGGGTTTAAAGGGTGCGTAGGCGGCTTTT

TAAGTCTGGGGTGAAAGCCCGCTGCTCAACAGCGGAACTGCCCTGGATACTGGAGAGCTT

GAGTACAGACGAGGTTGGCGGAATGGACGGAGTAGCGGTGAAATGCATAGATACCGTCCA

GAACCCCGATTGCGAAGGCAGCTGACTAGGCTGATACTGACGCTGAGGCACGAAAGCGTG

GGGAGCGAAC

>ASV411 GS|0.0|None;No hit

GTGTCAGCAGCCCATCATCACCTCCTCGGATTGCGAAGGTGCCGGGGAGGTTTTCACACT

GGCAGCCAAGTCGGCTCAAGCCACACCTATTCCAGATTCCACATCATCACAGGCTACTCC

GGAATCGTTCTTCCGGACACCAAGATATCTCACGGTCTCATCGCAACTACATTTGGAGAC

ATTCATACCTGAGCATCAGGGTGTGTGGAGCTTATCGCCGACCTTCAGAGCAGAGAAGAG

TGACACGCCC

>ASV412 SS|1.0000|AB267478_S000721192;k:Bacteria,p:"Bacteroidetes",c:Sphingobacteriia,o:"Sphingobacteriales",f:Chitinophagaceae

TACGGAGGGTGCAAGCGTTATCCGGATTTACTGGGTTTAAAGGGTGCGTAGGTGGGAATA

TAAGTCAGTGGTGAAATCTTCATGCTTAACATGGAAACTGCCATTGATACTATGTTTCTT

GAATTTTCTGGAGGTTAGCGGAATATGTCATGTAGCGGTGAAATGCTTAGATATGACATA

GAACACCAATTGCGAAGGCAGCTGGCTACAGGAAAATTGACACTGATGCACGAAAGCGTG

GGGATCAAAC

>ASV413 SS|0.9700|D86511_S000414138;k:Bacteria,p:"Proteobacteria",c:Alphaproteobacteria,o:Rhodospirillales,f:Acetobacteraceae,g:Acidiphilium

TACGAAGGGGGCTAGCGTTGCTCGGAATGACTGGGCGTAAAGGGCGCGTAGGCGGCATGT

TCAGTTAGGCGTGAAATTCCTGGGCTCAACCTGGGGACTGCGTTTAATACAGGCAGGCTA

GAGTGTGAAAGAGGGTCGTGGAATTTCCAGTGTAGAGGTGAAATTCGTAGATATTGGAAA

GAACACCGGTGGCGAAGGCGGCGACCTGGTTCACAACTGACGCTGAGGCGCGAAAGCGTG

GGGAGCAAAC

>ASV414 SS|1.0000|JQ346802_S003290742;k:Bacteria,p:"Actinobacteria",c:Actinobacteria,o:Actinomycetales

TACGTAGGGTGCAAGCGTTGTCCGGAATTATTGGGCGTAAAGAGCTCGTAGGCGGTTTGT

CGCGTCGGCTGTGAAAACATGGGGCTCAACCCTGTGCCTGCAGCCGATACGGGCAGACTA

GAATTCGGTAGGGGAGACTGGAATTCCTGGTGTAGCGGTGAAATGCGCAGATATCAGGAG

GAACACCGGTGGCGAAGGCGGGTCTCTGGGCCGACATTGACGCTGAGGAGCGAAAGCGTG

GGGAGCAAAC

>ASV415 SS|1.0000|D86513_S000011943;k:Bacteria,p:"Proteobacteria",c:Alphaproteobacteria,o:Rhodospirillales,f:Acetobacteraceae

TACGAAGGGGGCTAGCGTTGCTCGGAATGACTGGGCGTAAAGGGCGCGTAGGCGGATCGG

ATAGTCAGGCGTGAAATTCCTGGGCTCAACCTGGGGGCTGCGTTTGATACGTTTGGTCTA

GAGTGGGGAAGAGGGTTGCGGAATTCCCAGTGTAGAGGTGAAATTCGTAGATATTGGGAA

GAACACCGGTGGCGAAGGCGGCAACCTGGTCCTTGACTGACGCTGAGGCGCGAAAGCGTG

GGGAGCAAAC

>ASV416 SS|1.0000|JF834159_S002913810;k:Bacteria,p:"Bacteroidetes",c:Sphingobacteriia,o:"Sphingobacteriales",f:Chitinophagaceae

TACGGAGGGTGCAAGCGTTATCCGGATTCACTGGGTTTAAAGGGTGCGTAGGTGGGCAGT

TAAGTCAGTGGTGAAATCTCCGGGCTCAACCCGGAAACTGCCATTGATACTATCTATCTT

GAATATCGTGGAGGTAAGCGGAATATGTCATGTAGCGGTGAAATGCTTAGATATGACATA

GAACACCAATTGCGAAGGCAGCTTACTACCCGAATATTGACACTGAGGCACGAAAGCGTG

GGGATCAAAC

>ASV417 SS|0.9500|X64380_S000749517;k:Bacteria,p:"Verrucomicrobia",c:Spartobacteria,g:Spartobacteria_genera_incertae_sedis

TACAGAGGCCTCAAGCGTTGTTCGGATTCATTGGGCGTAAAGGGAGCGTAGGCGGTCGGG

TAAGTCGGGCGTGAAATCCTGGGGCTCAACCTCAGAACTGCGTTCGATACTGCTCGGCTA

GAGGACTGGAGAGGAGATTGGAATTCACGGTGTAGCAGTGAAATGCGTAGATATCGTGAG

GAAGACCAGTGGCGAAGGCGGATCTCTGGACAGTATCTGACGCTGAGGCTCGAAGGCTAG

GGGAGCAAAC

>ASV418 SS|1.0000|AJ420142_S000145622;k:Bacteria

TACGTAGGGGTCGAGCGTTGTCCGGAGTTACTGGGCGTAAAGCGTGTGCAGGCGGCGCAT

CGCGCCCGGCGTGAAAGCCCCCGGCTCAACCGGGGAGGGTCGTCGGGGACGGGTGCGCTT

GAGGGTCGCAGGGGCTGGTGGAATTCCCGGTGTAGTGGTGAAATGCGTAGAGATCGGGAG

GAACACCCGTGGCGAAGGCGGCCAGCTGGGCGACACCTGACGCTGAGACACGAAGGCGTG

GGGAGCGAAC

>ASV419 SS|1.0000|JN090860_S002916046;k:Bacteria,p:"Bacteroidetes",c:Cytophagia,o:Cytophagales,f:Cytophagaceae,g:Hymenobacter

TACGGAGGGTGCGAGCGTTGTCCGGATTTATTGGGTTTAAAGGGTGCGTAGGCGGCCTGG

TAAGTCTGGGGTGAAAGCCCGCTGCTCAACAGCGGAACTGCCCTGGATACTGTCAGGCTT

GAGGACAGACGAGGTTGGCGGAATGGAGGGTGTAGCGGTGAAATGCATAGATACCCTCCA

GAACCCCGATTGCGAAGGCAGCTGACTAGACTGTAACTGACGCTGAGGCACGAAAGCGTG

GGGAGCGAAC

>ASV420 SS|1.0000|D86513_S000011943;k:Bacteria,p:"Proteobacteria",c:Alphaproteobacteria,o:Rhodospirillales,f:Acetobacteraceae

TACGAAGGGGGCTAGCGTTGCTCGGAATGACTGGGCGTAAAGGGCGCGTAGGCGGTTTGC

ACAGTCGGGTGTGAAATTCCTGGGCTTAACCTGGGGGCTGCATTCGATACGTGTGGGCTT

GAGTGCGGAAGAGGGTTGTGGAATTCCCAGTGTAGAGGTGAAATTCGTAGATATTGGGAA

GAACACCGGTGGCGAAGGCGGCAACCTGGTCCGTGACTGACGCTGAGGCGCGAAAGCGTG

GGGAGCAAAC

>ASV421 SS|1.0000|AF268998_S000340459;k:Bacteria,p:Candidatus_Saccharibacteria,g:Saccharibacteria_genera_incertae_sedis

TACGTAGGGCCCGAGCGTTATCCGGAGTGACTGGGCGTAAAGAGTTGCGTAGGTGGCTTG

TAAAGTGAATAGTGAAAGCTGGTGGCTCAACCATTCAGATTATTATTCAAACTCACAAGC

TCGAGAGTAGCAGAGGTAACTGGAATTTCTTGTGTAGGAGTGAAATCCGTAGATATAAGA

AGGAACACCAATGGCGTAGGCAGGTTACTGGGCTACTTCTGACACTGAGGCACGAAAGCG

TGGGGAGCGA

>ASV422 GS|98.0|AB267478_S000721192;k:Bacteria,p:"Bacteroidetes",c:Sphingobacteriia,o:"Sphingobacteriales",f:Chitinophagaceae,g:Segetibacter;

TACGGAGGGTGCAAGCGTTATCCGGATTCACTGGGTTTAAAGGGTGCGTAGGTGGGAATG

TAAGTCAGTGGTGAAATCTCCGTGCTTAACATGGAAACTGCCATTGATACTATGTTTCTT

GAATTTTCTGGAGGATAGCGGAATATGTCATGTAGCGGTGAAATGCTTAGATATGACATA

GAACACCAATTGCGAAGGCAGCTATCTACAGGAACATTGACACTGAGGCACGAAAGCGTG

GGGATCAAAC

>ASV423 SS|0.9500|AM231587_S000650722;k:Bacteria,p:"Proteobacteria",c:Alphaproteobacteria,o:Rhodospirillales,f:Acetobacteraceae,g:Acidisoma

TACGAAGGGGGCTAGCGTTGCTCGGAATGACTGGGCGTAAAGGGCGCGTAGGCGGATTGG

TTAGTCAGACGTGAAATTCCTGGGCTCAACCTGGGGGCTGCGTTTGAGACGGCTGATCTA

GAGTTTGGAAGAGGGTCGTGGAATTCCCAGTGTAGAGGTGAAATTCGTAGATATTGGGAA

GAACACCGGTGGCGAAGGCGGCGACCTGGTCCTTGACTGACGCTGAGGCGCGAAAGCGTG

GGGAGCAAAC

>ASV424 SS|0.9400|AF132781_S000387813;k:Bacteria,p:Cyanobacteria/Chloroplast,c:Cyanobacteria,f:Family_I,g:GpI

TACGGAGGATGCAAGCGTTATCCGGAATGATTGGGCGTAAAGCGTCCGCAGGTGGTTTAT

CAAGTTTGCGGTTAAAGGTTCTGGCTCAACCAGAGACAGGCCGTGAAAACTGATAGACTA

GAGTATGGTAGGGGCAGAGGGAATTCCCAGTGTAGCGGTGAAATGCGTAGAGATTGGGAA

GAACACCGGTGGCGAAAGCGCTCTGCTAGGCCAAAACTGACACTGAGGGACGAAAGCTAG

GGGAGCGAAT

>ASV425 SS|1.0000|JN090860_S002916046;k:Bacteria,p:"Bacteroidetes",c:Cytophagia,o:Cytophagales,f:Cytophagaceae,g:Hymenobacter

TACGGAGGGTGCGAGCGTTGTCCGGATTTATTGGGTTTAAAGGGTGCGTAGGCGGCCGTT

TAAGTCTGGGGTGAAAGCCCGCTGCTCAACAGCGGAACTGCCCTGGATACTGGATGGCTT

GAGTACAGACGAGGTTGGCGGAATGGACCGAGTAGCGGTGAAATGCATAGATACGGTCCA

GAACCCCGATTGCGAAGGCAGCTGACTAGGCTGTTACTGACGCTGAGGCACGAAAGCGTG

GGGAGCGAAC

>ASV426 SS|1.0000|EF457349_S000834774;k:Bacteria

TACGTAGGGGGCGAGCGTTGTCCGGATTTATTGGGCGTAAAGGGCGCGTAGGCGGCCCGG

CGCATCCGGCGTGAAATCTCCCCGCTCAACGGGGAGGGGTCGGCGGAGATGGCCGGGCTT

GAGGGCGGTAGAGGCAGATGGAAGTCCCGAAGTAGTGGTGAAATGCGTAGAGATCGGGAG

GAACACCAGAGGCGAAGGCGATCTGCTGGGCCGACCCTGACGCTGAGGCGCGACAGCCAG

GGGAGCGAAC

>ASV427 SS|1.0000|AY140238_S000397413;k:Bacteria,p:"Proteobacteria",c:Alphaproteobacteria,o:Rhodospirillales,f:Acetobacteraceae

TACGAAGGGGGCTAGCGTTGCTCGGAATGACTGGGCGTAAAGGGCGCGTAGGCGGGTTGC

ACAGTCGGGCGTGAAATTCCTGGGCTTAACCTGGGGGCTGCGTTCGAGACGTGTGGCCTA

GAGTGGGGAAGAGGGTCGTGGAATTCCCAGTGTAGAGGTGAAATTCGTAGATATTGGGAA

GAACACCGGTGGCGAAGGCGGCGACCTGGTCCTTGACTGACGCTGAGGCGCGAAAGCGTG

GGGAGCAAAC

>ASV428 SS|1.0000|AB369258_S001151836;k:Bacteria,p:"Proteobacteria",c:Alphaproteobacteria,o:Rhodospirillales,f:Acetobacteraceae

TACGAAGGGGGCAAGCGTTGCTCGGAATGACTGGGCGTAAAGGGCGCGTAGGCGGCTCGA

TTTGTCAGGCGTGAAAGTCCTGGGCTCAACCTGGGGATGGCGCTTGGGACGGTGGGGCTG

GAGTGGGGAAGAGGGTCGTGGAATTCCCAGTGTAGAGGTGAAATTCGTAGATATTGGGAA

GAACACCGGTGGCGAAGGCGGCGACCTGGTCCTTTTACTGACGCTGAGGCGCGAGAGCGT

GGGGAGCAAA

>ASV429 SS|1.0000|EF635408_S001095322;k:Bacteria,p:"Deinococcus-Thermus",c:Deinococci,o:Deinococcales,f:Deinococcaceae,g:Deinococcus

TACGGAGGGTGCAAGCGTTACCCGGAATCACTGGGCGTAAAGGGCGTGTAGGCGGTTTGC

CAAGTCTGACTTTAAAGACCGAAGCTCAACTTCGGGAATGGGCCGGAGACTGGCAGACTA

GACGGATGGAGAGGTCACTGGAATTCCTGGTGTAGCGGTGGAATGCGTAGATACCAGGAG

GAACACCAACGGCGAAGGCAGGTGACTGGACATTTAGTGACGCTGAGGCGCGAAAGTGTG

GGGAGCAAAC

>ASV430 SS|1.0000|DQ986200_S000736608;k:Bacteria,p:"Planctomycetes",c:Planctomycetia,o:Planctomycetales,f:Planctomycetaceae

GACGAACCGTGCGAACGTTATTCGGAATTACTGGGCTTAAAGCGCGTGTAGGCGGCTCGG

CACGTCTGATGTTGAAATCCCCCGGCTTAACCGGGGAAGTGGCACGGATACGACCGAGCT

GGAGGGAGGTAGGGGGGTCTGGAACTTCCGGTGGAGCGGTGAAATGCGTTGAGATCGGAA

GGAACGCCCGTGGCGAAAGCGAGGCCCTAGACCTTTTCTGACGCTGAGACGCGAAAGCCA

GGGGAGCGAA

>ASV431 GS|98.0|AB018439_S000439484;k:Bacteria,p:"Proteobacteria",c:Alphaproteobacteria,o:Sphingomonadales,f:Sphingomonadaceae,g:Sphingomonas;

TACGGAGGGAGCTAGCGTTATTCGGAATTACTGGGCGTAAAGCGCACGTAGGCGGCTTTG

TAAGTTAGAGGTGAAAGCCCAGAGCTCAACTCTGGAATTGCCTTTAAGACTGAATCGCTT

GAATCCAGGAGAGGTGAGTGGAATTCCGAGTGTAGAGGTGAAATTCGTAGATATTCGGAA

GAACACCAGTGGCGAAGGCGGCTCACTGGACTGGTATTGACGCTGAGGTGCGAAAGCGTG

GGGAGCAAAC

>ASV432 GS|99.2|HQ839787_S002910138;k:Bacteria,p:"Actinobacteria",c:Actinobacteria,o:Actinomycetales,f:Propionibacteriaceae,g:Friedmanniella;

TACGTAGGGTCCGAGCGTTGTCCGGAATTATTGGGCGTAAAGGGCTTGTAGGCGGTCCGT

CGCGTCAGGAGTGAAAACTCGGGGCTTAACCCCGAGCCTGCTTTTGATACGGGCGGACTA

GAGGGATGCAGGGGAGAACGGAATTCCTGGTGGAGCGGTGGAATGCGCAGATATCAGGAG

GAACACCGGTGGCGAAGGCGGTTCTCTGGGCATTTCCTGACGCTGAGAAGCGAAAGCGTG

GGGAGCAAAC

>ASV433 GS|97.2|EU861928_S001148233;k:Bacteria,p:"Armatimonadetes",c:Armatimonadia,o:Armatimonadales,f:Armatimonadaceae,g:Armatimonas/Armatimonadetes_gp1;

TACGTAGGGGGCGAGCGTTGTCCGAAGTTACTGGGCGTAAAGAGCGCGTAGGCGGGTCTT

TAAGTGAGGGGTGAAAGTCCGAGGCTCAACCTCGGAACTGCCTTTCATACTGGGGACCTT

GAGTGTGGGAGAGGCGAGTGGAATGGTCGGTGTAGCGGTGAAATGCGTAGATATCGATCG

GAACACCCATGGCGAAGGCAGCTCGCTGGCCTATAACTGACGCTGAGGCGCGAAAGCGTG

GGGAGCAAAC

>ASV434 GS|97.6|EU861928_S001148233;k:Bacteria,p:"Armatimonadetes",c:Armatimonadia,o:Armatimonadales,f:Armatimonadaceae,g:Armatimonas/Armatimonadetes_gp1;

TACGTAGGGGGCGAGCGTTGTCCGAAGTTACTGGGCGTAAAGAGCGCGTAGGCGGGTCTT

TAAGTGAGGGGTGAAAGTCCGAGGCTCAACCTCGGAACTGCCTTTCATACTGGAGACCTT

GAGTGTGGGAGAGGCGAGTGGAATGGTCGGTGTAGCGGTGAAATGCGTAGATATCGATCG

GAACACCCATGGCGAAGGCAGCTCGCTGGCCTATAACTGACGCTGAGGCGCGAAAGCGTG

GGGAGCAAAC

>ASV435 SS|1.0000|JN090860_S002916046;k:Bacteria,p:"Bacteroidetes",c:Cytophagia,o:Cytophagales,f:Cytophagaceae,g:Hymenobacter

TACGGAGGGTGCGAGCGTTGTCCGGATTTATTGGGTTTAAAGGGTGCGTAGGCGGCTTGG

TAAGTCTGGGGTGAAAGCCCGCTGCTCAACAGCGGAACTGCCCTGGATACTGCCAGGCTT

GAGGACAGACGAGGTTAGCGGAATGGACGGTGTAGCGGTGAAATGCATAGATACCGTCCA

GAACCCCGATTGCGAAGGCAGCTGACTAGACTGTACCTGACGCTGAGGCACGAAAGCGTG

GGGAGCGAAC

>ASV436 GS|100.0|GQ253122_S001576427;k:Bacteria,p:"Proteobacteria",c:Alphaproteobacteria,o:Sphingomonadales,f:Sphingomonadaceae,g:Sphingomonas;

TACGGAGGGAGCTAGCGTTATTCGGAATTACTGGGCGTAAAGCGCACGTAGGCGGCTTTG

TAAGTAAGAGGTGAAAGCCTGGTGCTCAACACCAGAACTGCCTTTTAGACTGCATCGCTT

GAATCCAGGAGAGGTGAGTGGAATTCCGAGTGTAGAGGTGAAATTCGTAGATATTCGGAA

GAACACCAGTGGCGAAGGCGGCTCACTGGACTGGTATTGACGCTGAGGTGCGAAAGCGTG

GGGAGCAAAC

>ASV437 SS|0.8300|FN421805_S001593419;k:Bacteria,p:"Armatimonadetes",c:Armatimonadia,o:Armatimonadales,f:Armatimonadaceae,g:Armatimonas/Armatimonadetes_gp1

TACGTAGGGGGCGAGCGTTGTCCGAAGTTACTGGGCGTAAAGCGCGTGTAGGCGGGTTTT

TAAGTTTGGGGTGAAAGGTTCACGGCTCAACCGGAACAGTGCCTTGAAAACTGGGAACCT

TGAATGTGGCAGGGGAAAGCGGAATTCCAGGTGTAGCGGTGAAATGCGTAGATATCTGGA

GGAACACCAATGGCGAAGGCAGCTTTCTGGGCTAACATTGACGCTGAGACGCGAAAGCGT

GGGGAGCGAA

>ASV438 GS|0.0|None;No hit

TTCCAGCTCCAATAGCGTATATTAAAGTTGTTGCAGTTAAAAAGCTCGTAGTTGAAACTT

GGGCCTGGCTGACCGGTCCGCCTCACCGCGTGTACTGGTTCGGCCGGGCCTTTCCTTCTG

GGGAGCCGCATGGCCTTCATTGGTCGTGTTGGGGATCCAGGACTTTTACTTTGAAAAAAT

TAGAGTGTTCAAAGCAGGCCTAGGCTCGAATACATTAGCATGGAATAATAGAATAGGACG

TGTGGTTCTA

>ASV439 GS|100.0|AF035052_S000427920;k:Bacteria,p:"Proteobacteria",c:Betaproteobacteria,o:Burkholderiales,f:Burkholderiales_incertae_sedis,g:Aquabacterium;

TACGTAGGGTGCGAGCGTTAATCGGAATTACTGGGCGTAAAGCGTGCGCAGGCGGCTTTG

CAAGACAGAGGTGAAATCCCCGGGCTCAACCTGGGAACTGCCTTTGTGACTGCAAGGCTA

GAGTACGGCAGAGGGGGATGGAATTCCGCGTGTAGCAGTGAAATGCGTAGATATGCGGAG

GAACACCAATGGCGAAGGCAATCCCCTGGGCCTGTACTGACGCTCATGCACGAAAGCGTG

GGGAGCAAAC

>ASV440 SS|1.0000|JX949238_S003747851;k:Bacteria,p:"Bacteroidetes",c:Sphingobacteriia,o:"Sphingobacteriales",f:Sphingobacteriaceae

TACGGAGGATCCAAGCGTTATCCGGATTTATTGGGTTTAAAGGGTGCGTAGGCGGCCTGT

TAAGTCAGGGGTGAAATTTTCCGGCTCAACCAGGACATTGCCTTTGATACTGACGGGCTT

GAATGCAGTTGAGGTAGGCGGAATGTGACAAGTAGCGGTGAAATGCATAGATATGTCACA

GAACACCAATTGCGAAGGCAGCTTACTAAAGTGTGATTGACGCTGAGGCACGAAAGCGTG

GGGATCAAAC

>ASV441 SS|1.0000|D86513_S000011943;k:Bacteria,p:"Proteobacteria",c:Alphaproteobacteria,o:Rhodospirillales,f:Acetobacteraceae

TACGAAGGGGGCTAGCGTTGCTCGGAATGACTGGGCGTAAAGGGCGCGTAGGCGGCACGG

ACAGTCAGGTGTGAAATTCCCGGGCTTAACCTGGGGACTGCATTTGATACGTTCGAGCTA

GAGTGCGGAAGAGGGTCGTGGAATTCCCAGTGTAGAGGTGAAATTCGTAGATATTGGGAA

GAACACCGGTGGCGAAGGCGGCGACCTGGTCCGTGACTGACGCTGAGGCGCGAAAGCGTG

GGGAGCAAAC

>ASV442 GS|97.2|AB267478_S000721192;k:Bacteria,p:"Bacteroidetes",c:Sphingobacteriia,o:"Sphingobacteriales",f:Chitinophagaceae,g:Segetibacter;

TACGGAGGGTGCAAGCGTTATCCGGATTCACTGGGTTTAAAGGGTGCGTAGGTGGGGATG

TAAGTCAGTGGTGAAATCTCCGTGCTTAACATGGAAACTGCCATTGATACTATGTTTCTT

GAATTTTCTGGAGGTCAGCGGAATATGTCATGTAGCGGTGAAATGCTTAGATATGACATA

GAACACCAATTGCGAAGGCAGCTGGCTACGGGAATATTGACACTGAGGCACGAAAGCGTG

GGGATCAAAC

>ASV443 GS|0.0|None;No hit

TACGTAAGAGACTAGTGTTATTCATCACAATTAGGTTTAAAGGGTAAGTAGACGGTCTAA

ATAGCATTTAAGATGTAACTATTTGACTAGAGTTTTATATAAGAGGGCAGTACCTGAGGT

GGAGAGATAATATTTGATGATACCAAAGGGACTGGTAAAGGCGAAGACAGCCCTTTATGT

AAGAACTGACGTTGAGGTACGAAGGCATAGGTCACGAACAGG

>ASV444 SS|1.0000|AJ009456_S000115949;k:Bacteria

TACGTAGGTGGCAAGCGTTGTCCGGATTTACTGGGCGTAAAGCGCGCGCAGGCGGACTGT

TAAGTAGGAAGTGAAAGGTTGGAGCTCAACTCCAACATTGCTTCCTAAACTGGCAGTCTT

GAGTCCCGGAGGGGAGAGCGGAACAATACGTGTAGCGGTGAAATGCGTTGATATGTATTG

GAACACCAATGGCGAAGGCAGCTCTCTGGACGGGAACTGACGCTCAGGCGCGAAAGCCGA

GGTAGCGAAC

>ASV445 GS|99.6|AB297501_S000870487;k:Bacteria,p:"Proteobacteria",c:Alphaproteobacteria,o:Rhodospirillales,f:Acetobacteraceae,g:Roseomonas;

TACGAAGGGGGCTAGCGTTACTCGGAATTACTGGGCGTAAAGGGCGCGTAGGCGGCTTGA

TAAGTCAGGCGTGAAATTCCCGGGCTCAACCTGGGGACTGCGCTTGAGACTGTTGGGCTA

GAGGATGGAAGAGGGTCGTGGAATTCCCAGTGTAGAGGTGAAATTCGTAGATATTGGGAA

GAACACCGGTGGCGAAGGCGGCGACCTGGTCCATTACTGACGCTGAGGCGCGACAGCGTG

GGGAGCAAAC

>ASV446 SS|0.9000|AB847449_S004125734;k:Bacteria,p:"Proteobacteria",c:Deltaproteobacteria,o:Myxococcales,f:Labilitrichaceae,g:Labilithrix

GACAGAGGGTGCAAACGTTGTTCGGAATTACTGGGCGTAAAGCGAGTGTAGGCGGTCTCA

AAAGTCAGGTGTGAAAGCCCTGGGCTCAACCCAGGAAGTGCACTTGAAACTGCGAGGCTA

GAGTACTGGAGAGGTTGGTGGAATTCTCGGTGTAGAGGTGAAATTCGTAGATATCGAGAG

GAACACCGGTGGCGAAGGCGGCCAACTGGACAGATACTGACGCTGAGACTCGAAAGCGTG

GGGAGCAAAC

>ASV447 SS|0.9700|FJ423552_S001241589;k:Bacteria,p:"Actinobacteria",c:Actinobacteria,o:Actinomycetales,f:Intrasporangiaceae

TACGTAGGGTGCAAGCGTTGTCCGGAATTATTGGGCGTAAAGAGCTTGTAGGTGGTTTGT

CACGTCCGCTGTGAAATTTCGAGGCTCAACCTCGAACTTGCAGTGGGTACGGGCAGACTA

GAGTGTGGTAGGGGAGACTGGAATTCCTGGTGTAGCGGTGAAATGCGCAGATATCAGGAG

GAACACCGATGGCGAAGGCAGGTCTCTGGGCCATAACTGACACTGAGAAGCGAAAGCATG

GGGAGCGAAC

>ASV448 SS|1.0000|EF457480_S000834905;k:Bacteria

TACGTGGGGACCAAGCGTTGTCCGGAATTACTGGGCGTAAAGGGCGTGCAGGTGGCTGCT

CAAGCTTGGCGTGAAAGCCCCCGGCCCAACCGGGGAGGGTCGTCGAGGACTGGGTGGCTT

GAGGGGCGCAGGGGTCGGTGGAATTCCGGGTGTAGTGGTGAAATGCGTAGAGATCCGGAG

GAACACCAGTGGCGAAGGCGGCCGACTGGGCGCACCCTGACACTGAGACGCGAAGGCGTG

GGGAGCGAAC

>ASV449 SS|1.0000|EF516412_S000840854;k:Bacteria,p:"Armatimonadetes",c:Armatimonadia,o:Armatimonadales,f:Armatimonadaceae,g:Armatimonas/Armatimonadetes_gp1

TACGTAGGGGGCCAGCGTTGTCCGAAGTTACTGGGCGTAAAGAGCGCGTAGGCGGGTCTT

TAAGTGAGGGGTGAAAGGTTTGGGCTTAACCCAGACACTGCCTTTCATACTGGGGACCTT

GAGTATGGGAGAGGCGAGTGGAATTCTTGGTGTAGCGGTGAAATGCGTAGATATCAAGAG

GAACACCCATGGCGAAGGCAGCTCGCTGGCCTATAACTGACGCTGAGGCGCGAAAGCTGG

GGGAGCAAAC

>ASV450 GS|72.9|M38637_S000436282;k:Archaea,p:"Euryarchaeota",c:Thermoplasmata,o:Thermoplasmatales,f:Thermoplasmataceae,g:Thermoplasma;

CTTGGTCATTTAGAGGAAGTAAAAGTCGTAACAAGGTTTCCGTAGGTGAACCTGCGGAAG

GATCATTACCGAGAGGGACATGCGCCTCGCGGCCATGTCCCGGGGGGTTCCGCCCCCATC

TCTTCAACCCTTGCCTATCTACCTCTGTTGCTTCGGCGAGCGCTCGGGTGCCATTGCGCC

CGGGCCCCGGCTTCGGTCGGTGCGCTCTCGCCGGAGGCCATATTGAACCTGTCTGTAGTG

ATGTCTGAGT

>ASV451 SS|1.0000|GQ421847_S001589376;k:Bacteria,p:"Bacteroidetes",c:Sphingobacteriia,o:"Sphingobacteriales",f:Chitinophagaceae

TACGGAGGGTGCGAGCGTTATCCGGATTCACTGGGTTTAAAGGGTGCGTAGGTGGGTTGG

TAAGTCAGTGGTGAAATCTCCGTGCTTAACATGGAAACTGCCATTGATACTACTGGTCTT

GAATTTTCCGGAGGTTAGCGGAATATGTCATGTAGCGGTGAAATGCTTAGATATGACATA

GAACACCAATTGCGAAGGCAGCTAACTACAGGGACATTGACACTGAGGCACGAAAGCGTG

GGGATCAAAC

>ASV452 SS|1.0000|EF457349_S000834774;k:Bacteria

TACGTAGGGGGCGAGCGTTGTCCGGATTTATTGGGCGTAAAGGGCGCGTAGGCGGCCTGG

CGCATCCGGCGTGAAATCTCCCCGCTCAACGGGGAGGGGTCGGCGGAGATGGCCGGGCTG

GAGGGCGGTAGAGGCAGATGGAAGTCCCGAAGTAGTGGTGAAATGCGTAGAGATCGGGAG

GAACACCAGAGGCGAAGGCGATCTGCTGGGCCGACCCTGACGCTGAGGCGCGACAGCCAG

GGGAGCGAAC

>ASV453 SS|1.0000|AY140238_S000397413;k:Bacteria,p:"Proteobacteria",c:Alphaproteobacteria,o:Rhodospirillales,f:Acetobacteraceae

TACGAAGGGGGCTAGCGTTGCTCGGAATGACTGGGCGTAAAGGGCGCGTAGGCGGAGTGC

TTTGTCGGGCGTGAAATTCCAGGGCTTAACCTTGGGGCTGCGTTCGAGACGGGTATTCTA

GAGTGTGGCAGAGGGTCGTGGAATTCCCAGTGTAGAGGTGAAATTCGTAGATATTGGGAA

GAACACCGGTGGCGAAGGCGGCGACCTGGTCCATTACTGACGCTGAGGCGCGAAAGCGTG

GGGAGCAAAC

>ASV454 SS|1.0000|AM887758_S000941901;k:Bacteria,p:"Acidobacteria",c:Acidobacteria_Gp1

TACGAGGGGGGCAAGCGTTGTTCGGAATTATTGGGCGTAAAGGGTGCGTAGGCGGTTTGA

CAAGTTCTATGTGAAATCTATGGGCTCAACCCATAGTCTGCATGGAAAACTGTCAGGCTT

GAGGATGGGAGAGGTGAGTGGAATTTCCGGTGTAGCGGTGAAATGCGTAGATATCGGAAG

GAACACCTGTGGCGAAAGCGGCTCACTGGACCATTACTGACGCTGATGCACGAAAGCTAG

GGGAGCAAAC

>ASV455 SS|0.9500|CP001854_S001872674;k:Bacteria,p:"Actinobacteria",c:Actinobacteria,o:Solirubrobacterales

TACGTAGGGGGCAAGCGTTGTCCGGAATTATTGGGCGTAAAGCGCGTGTAGGCGGCTCGA

TCAGTCCGCTCTGAAAGTCCGGGGCTCAACCCCGGGAGGCGGGTGGATACTGTCGGGCTC

GAGTCCGGAAGAGGCGAGTGGAATTCCTGGTGTAGCGGTGAAATGCGCAGATATCAGGAG

GAACACCAATGGCGAAGGCAGCTCGCTGGGACGGTACTGACGCTGAGACGCGAAAGCGTG

GGGAGCAAAC

>ASV456 GS|98.4|HQ687090_S002989305;k:Bacteria,p:"Acidobacteria",c:Acidobacteria_Gp1,g:Granulicella;

TACGAGGGGGGCAAGCGTTGTTCGGAATTATTGGGCGTAAAGGGTGCGTAGGCGGTTTGA

CAAGTCATCTGTGAAATCTATGGGCTCAACCCATAGTCTGCAGGCGAAACTGTCGGGCTT

GAGTATGGGAGAGGTGAGTGGAATTTCCGGTGTAGCGGTGAAATGCGTAGATATCGGAAG

GAACACCTGTGGCGAAAGCGGCTCACTGGACCATAACTGACGCTGAGGCACGAAAGCTAG

GGGAGCAAAC

>ASV457 SS|1.0000|DQ986200_S000736608;k:Bacteria,p:"Planctomycetes",c:Planctomycetia,o:Planctomycetales,f:Planctomycetaceae

GACGAACCGTGCGAACGTTATTCGGAATCACTGGGCTTAAAGCGCGTGTAGGCGGGTCGG

CGCGTCGGGAGCTGAAATCCCTCGGCTCAACCGGGGAAGTGGCACCGATACGGCCGGTCT

GGAGGGGGGTAGGGGGGCCTGGAACTTCCGGTGGAGCGGTGAAATGCGTTGAGATCGGAA

GGAACGCCCGTGGCGAAAGCGAGGCCCTGGACCTTTTCTGACGCTGAGACGCGAAAGCCA

GGGGAGCGAA

>ASV458 SS|1.0000|D86513_S000011943;k:Bacteria,p:"Proteobacteria",c:Alphaproteobacteria,o:Rhodospirillales,f:Acetobacteraceae

TACGAAGGGGGCTAGCGTTGCTCGGAATGACTGGGCGTAAAGGGCGCGTAGGCGGTTTGC

ATAGTCAGATGTGAAATTCCTGGGCTTAACCTGGGGGCTGCATTTGATACGTGTGGGCTT

GAGTGGGGAAGAGGGTCGTGGAATTCCCAGTGTAGAGGTGAAATTCGTAGATATTGGGAA

GAACACCGGTGGCGAAGGCGGCGACCTGGTCCTTTACTGACGCTGAGGCGCGAAAGCGTG

GGGAGCAAAC

>ASV459 GS|100.0|AB003935_S000381721;k:Bacteria,p:"Actinobacteria",c:Actinobacteria,o:Actinomycetales,f:Kineosporiaceae,g:Kineosporia;

TACGTAGGGTGCAAGCGTTGTCCGGAATTATTGGGCGTAAAGAGCTCGTAGGCGGTCTGT

CGCGTCTGCTGTGAAAACTCAGGGCTTAACCCTGAGCTTGCAGTGGGTACGGGCAGACTA

GAGTGCGGTAGGGGAGACTGGAATTCCTGGTGTAGCGGTGAAATGCGCAGATATCAGGAG

GAACACCGGTGGCGAAGGCGGGTCTCTGGGCCGTAACTGACGCTGAGGAGCGAAAGCATG

GGGAGCGAAC

>ASV460 SS|0.9400|AF013550_S000336299;k:Bacteria,p:"Acidobacteria",c:Acidobacteria_Gp4

TACGGGGGGGGCAAGCGTTGTTCGGATTTACTGGGCGTAAAGCGTATGTAGGCGGTTAGG

TAAGTTACTTGTGAAATCTCCGAGCTTAACTCGGAACGGCCAAGTAATACTGCCAAACTA

GAGTGCAGAAGGGGCAATTGGAATTCTTGGTGTAGCGGTGAAATGCGTAGATATCAAGAG

GAACACCAGAGGCGAAGGCGAATTGCTGGGCTGACACTGACGCTGAGATACGAAAGCTAG

GGGAGCAAAC

>ASV461 SS|1.0000|JN090860_S002916046;k:Bacteria,p:"Bacteroidetes",c:Cytophagia,o:Cytophagales,f:Cytophagaceae,g:Hymenobacter

TACGGAGGGTGCGAGCGTTGTCCGGATTTATTGGGTTTAAAGGGTGCGTAGGCGGCCGCT

TAAGTCTGGGGTGAAAGCCCGCTGCTCAACAGCGGAACGGCCCTGGATACTGGGTGGCTT

GAGTACAGACGAGGTTGGCGGAATGGACCGAGTAGCGGTGAAATGCATAGATACGGTCCA

GAACCCCGATTGCGAAGGCAGCTGACTAGGCTGTTACTGACGCTGAGGCACGAAAGCGTG

GGGAGCGAAC

>ASV462 SS|1.0000|EU861928_S001148233;k:Bacteria,p:"Armatimonadetes",c:Armatimonadia,o:Armatimonadales,f:Armatimonadaceae,g:Armatimonas/Armatimonadetes_gp1

TACGTAGGGGGCGAGCGTTGTCCGAAGTTACTGGGCGTAAAGAGCGCGTAGGCGGGTTCT

TAAGTGAGGGGTGAAATTCCGAGGCTTAACCTCGGAACTGCCTTTCATACTGGGAACCTT

GAGTGTGGGAGAGGCGAGTGGAATGGTCGGTGTAGCGGTGAAATGCGTAGATATCGATCG

GAACACCCATGGCGAAGGCAGCTCGCTGGCCCATAACTGACGCTGAGGCGCGAAAGCGTG

GGGAGCAAAC

>ASV463 GSL|98.4|EF532793_S000859919;k:Bacteria,p:"Proteobacteria",c:Betaproteobacteria,o:Burkholderiales,f:Comamonadaceae

TACGTAGGGTGCGAGCGTTAATCGGAATTACTGGGCGTAAAGCGTGCGCAGGCGGTTTTG

TAAGACAGGTGTGAAATCCCCGGGCTCAACCTGGGAATTGCATTTGTGACTGCAAGGCTG

GAGTGCGGCAGAGGGGGATGGAATTCCGCGTGTAGCAGTGAAATGCGTAGATATGCGGAG

GAACACCGATGGCGAAGGCAATCCCCTGGGCCTGCACTGACGCTCATGCACGAAAGCGTG

GGGAGCAAAC

>ASV464 SS|1.0000|D86513_S000011943;k:Bacteria,p:"Proteobacteria",c:Alphaproteobacteria,o:Rhodospirillales,f:Acetobacteraceae

TACGAAGGGGGCTAGCGTTGCTCGGAATGACTGGGCGTAAAGGGCGCGTAGGCGGTTCGT

ACAGTTGGGTGTGAAATTCCTGGGCTTAACCTGGGGGCTGCATTCGATACGTGCGGGCTA

GAGTGGGGAAGAGGGTCGTGGAATTCCCAGTGTAGAGGTGAAATTCGTAGATATTGGGAA

GAACACCGGTGGCGAAGGCGGCGACCTGGTCCTTGACTGACGCTGAGGCGCGAAAGCGTG

GGGAGCAAAC

>ASV465 SS|1.0000|JN695632_S002960974;k:Bacteria,p:"Bacteroidetes",c:Sphingobacteriia,o:"Sphingobacteriales",f:Sphingobacteriaceae

TACGGAGGATCCGAGCGTTATCCGGATTTATTGGGTTTAAAGGGTGCGTAGGCGGCCTGT

TAAGTCAGGGGTGAAAGACGGTGGCTCAACCATCGCAGTGCCTTTGATACTGACGGGCTT

GAATGCAGCTGAGGTAGGCGGAATGTGACAAGTAGCGGTGAAATGCATAGATATGTCACA

GAACACCAATTGCGAAGGCAGCTTACTAAAGTGTGATTGACGCTGAGGCACGAAAGCGTG

GGGATCAAAC

>ASV466 SS|0.9900|JN090860_S002916046;k:Bacteria,p:"Bacteroidetes",c:Cytophagia,o:Cytophagales,f:Cytophagaceae,g:Hymenobacter

TACGGAGGGTGCGAGCGTTGTCCGGATTTATTGGGTTTAAAGGGTGCGTAGGCGGCCGCG

TAAGTCCGGGGTGAAAGCCCGTTGCTCAACAACGGAACTGCCCCGGAAACTGCGCGGCTT

GAGTCCAGACGAGGTCGGCGGAATGGGCGGTGTAGCGGTGAAATGCATAGATACCGTCCA

GAACCCCGATTGCGAAGGCAGCTGACTAGGCTGGCACTGACGCTGAGGCACGAAAGCGTG

GGGAGCGAAC

>ASV467 SS|1.0000|AB778530_U010573767;k:Bacteria,p:"Proteobacteria",c:Alphaproteobacteria,o:Rhodospirillales,f:Acetobacteraceae

TACGAAGGGGGCTAGCGTTGCTCGGAATGACTGGGCGTAAAGGGCGCGTAGGCGGTTCAC

GCAGTCAGATGTGAAATTCCTGGGCTTAACCTGGGGGCTGCATTTGAGACGCGTGGGCTT

GAGTATGAAAGAGGGTCGTGGAATTCCCAGTGTAGAGGTGAAATTCGTAGATATTGGGAA

GAACACCGGTGGCGAAGGCGGCGACCTGGTTCATAACTGACGCTGAGGCGCGAAAGCGTG

GGGAGCAAAC

>ASV468 SS|1.0000|AJ420142_S000145622;k:Bacteria

GACGTAGGAGGCGAGCGTTGTCCGGAGTTACTGGGCGTAAAGCGCGCGCAGGCGGTCGCG

CAGGTCGCCCGTGAAAGCCCCCGGCTCAACCGGGCGGAGGCGGGCGAAACCGCGCGACTG

GAGGGCGGCAGAGGGTCGTGGAATTCCCGGTGTAGTGGTGAAATGCGTAGAGATCGGGAG

GAACACCCGCGGCGAAGGCGGCGACCTGGACCGACCCTGACGCTGAGGCGCGAAGGCCGG

GGGAGCGAAC

>ASV469 SS|1.0000|AY140238_S000397413;k:Bacteria,p:"Proteobacteria",c:Alphaproteobacteria,o:Rhodospirillales,f:Acetobacteraceae

TACGAAGGGGGCTAGCGTTGCTCGGAATGACTGGGCGTAAAGGGCGCGTAGGCGGATGTC

TTAGTCAGGTGTGAAATTCCTGGGCTTAACCTGGGGGCTGCATTTGAGACGGGATGTCTA

GAGTTTGGAAGAGGGTCGTGGAATTCCCAGTGTAGAGGTGAAATTCGTAGATATTGGGAA

GAACACCGGTGGCGAAGGCGGCGACCTGGTCCTTGACTGACGCTGAGGCGCGAAAGCGTG

GGGAGCAAAC

>ASV470 SS|0.8500|KM044053_S004224125;k:Bacteria,p:"Actinobacteria",c:Actinobacteria,o:Actinomycetales,f:Nocardiaceae

TACGTAGGGTGCGAGCGTTGTCCGGAATTACTGGGCGTAAAGAGCTCGTAGGCGGTTTGT

CACGTCGGCTGTGAAATCTCATCGCTCAACGGTGAGCTTGCAGTCGATACGGGCTGACTT

GAGTACTGCAGGGGAGACTGGAATTCCTGGTGTAGCGGTGAAATGCGCAGATATCAGGAG

GAACACCGGTGGCGAAGGCGGGTCTCTGGGCAGTAACTGACGCTGAGGAGCGAAAGCGTG

GGTAGCAAAC

>ASV471 SS|0.8700|EF067860_S000769040;k:Bacteria,p:"Bacteroidetes",c:Sphingobacteriia,o:"Sphingobacteriales",f:Chitinophagaceae

TACGGAGGGTGCAAGCGTTATCCGGATTTACTGGGTTTAAAGGGTGCGCAGGCGGTCTTT

TAAGTCAGTGGTGAAATCCCGGGGCTCAACCCCGGAACTGCCATTGATACTATTGGACTT

GAATATCGTTGAGGCTGGCGGAATACATCATGTAGCGGTGAAATGCTTAGATATGATGTA

GAACACCGATTGCGAAGGCAGCTGGCTAAGCGATGATTGACGCTCATGCACGAAAGCGTG

GGGATCAAAC

>ASV472 GS|100.0|X82054_S000011560;k:Bacteria,p:"Actinobacteria",c:Actinobacteria,o:Actinomycetales,f:Corynebacteriaceae,g:Corynebacterium;

TACGTAGGGTGCGAGCGTTGTCCGGAATTACTGGGCGTAAAGAGCTCGTAGGTGGTTTGT

CGCGTCGTTTGTGTAAGCCCGCAGCTTAACTGCGGGACTGCAGGCGATACGGGCATAACT

TGAGTGCTGTAGGGGAGACTGGAATTCCTGGTGTAGCGGTGGAATGCGCAGATATCAGGA

GGAACACCGATGGCGAAGGCAGGTCTCTGGGCAGTAACTGACGCTGAGGAGCGAAAGCAT

GGGTAGCGAA

>ASV473 GS|97.2|AB267478_S000721192;k:Bacteria,p:"Bacteroidetes",c:Sphingobacteriia,o:"Sphingobacteriales",f:Chitinophagaceae,g:Segetibacter;

TACGGAGGGTGCAAGCGTTATCCGGATTTACTGGGTTTAAAGGGTGCGTAGGTGGGAATG

TAAGTCAGTGGTGAAATCTCCGTGCTTAACATGGAAACTGCCATTGATACTATGTTTCTT

GAATTTTCCGGAGGTAAGCGGAATATGTCATGTAGCGGTGAAATGCTTAGATATGACATA

GAACACCAATTGCGAAGGCAGCTTACTACAGGAACATTGACACTGAGGCACGAAAGCGTG

GGGATCAAAC

>ASV474 GS|98.4|EU861894_S001148199;k:Bacteria,p:"Armatimonadetes",c:Armatimonadia,o:Armatimonadales,f:Armatimonadaceae,g:Armatimonas/Armatimonadetes_gp1;

TACGTAGGGGGCCAGCGTTGTCCGAAGTTACTGGGCGTAAAGAGCGCGTAGGCGGGCCCT

TAAGTGGGGGGTGAAAGTCTGAGGCTCAACCTCGGAACTGCCTTTCATACTGGGGGCCTT

GAGTGCGGGAGAGGCGAGTGGAATGGTCGGTGTAGCGGTGAAATGCGTAGATATCGATCG

GAACACCCATGGCGAAGGCAGCTCGCTGGCCTGCAACTGACGCTGAGGCGCGAAAGCCGG

GGGAGCGAAC

>ASV475 GS|97.6|AB561883_S002150744;k:Bacteria,p:"Proteobacteria",c:Alphaproteobacteria,o:Rhodospirillales,f:Acetobacteraceae,g:Acidiphilium;

TACGAAGGGGGCTAGCGTTGCTCGGAATGACTGGGCGTAAAGGGCGCGTAGGCGGCTTAC

ACAGTCAGGCGTGAAATTCCTGGGCTCAACCTGGGGACTGCGTCTGATACGTGTAGGCTT

GAGTAGGGAAGAGGGTCGTGGAATTTCCAGTGTAGAGGTGAAATTCGTAGATATTGGAAA

GAACACCGGTGGCGAAGGCGGCGACCTGGTCCTTTACTGACGCTGAGGCGCGAAAGCGTG

GGGAGCAAAC

>ASV476 SS|1.0000|AJ009456_S000115949;k:Bacteria

TACGTAGGTGGCAAGCGTTGTCCGGATTTACTGGGCGTAAAGCGCGCGCAGGCGGACTGT

TAAGTAGGAAGTGAAAGGTTGGGGCTTAACCCCAACATTGCTTCCTATACTGGCAGTCTT

GAGTCCCGGAGGGGAGAGCGGAACAATACGTGTAGCGGTGAAATGCGTTGATATGTATTG

GAACACCAATGGCGAAGGCAGCTCTCTGGACGGGTACTGACGCTCAGGCGCGAAAGCCGA

GGTAGCGAAC

>ASV477 SS|1.0000|D86513_S000011943;k:Bacteria,p:"Proteobacteria",c:Alphaproteobacteria,o:Rhodospirillales,f:Acetobacteraceae

TACGAAGGGGGCTAGCGTTGCTCGGAATGACTGGGCGTAAAGGGCGCGTAGGCGGATTGG

TCAGTCAGGCGTGAAATTCCTGGGCTTAACCTGGGGGCTGCGTTTGAGACGGCTGGTCTG

GAGTGGGGAAGAGGGTCGTGGAATTCCCAGTGTAGAGGTGAAATTCGTAGATATTGGGAA

GAACACCGGTGGCGAAGGCGGCGACCTGGTCCTTGACTGACGCTGAGGCGCGAAAGCGTG

GGGAGCAAAC

>ASV478 SS|0.9900|GQ342559_S003611109;k:Bacteria,p:"Bacteroidetes",c:Cytophagia,o:Cytophagales,f:Cytophagaceae,g:Spirosoma

TACGGAGGGTGCAAGCGTTGTCCGGATTTATTGGGTTTAAAGGGTGCGTAGGTGGTTTCT

TAAGTCTGGTTTGAAAGCAGGCGGCTCAACCGTGTGATGTGGCTGGAAACTGGGGAACTT

GAATGGGTTGGCGGCTGCCGGAACGGGTCATGTAGCGGTGAAATGCATAGATATGACCCA

GAACACCGATTGCGAAGGCAGGCAGCTAGGACCTGATTGACACTGAGGCACGAGAGCATG

GGGAGCCAAC

>ASV479 SS|1.0000|AB267476_S000721190;k:Bacteria,p:"Bacteroidetes",c:Sphingobacteriia,o:"Sphingobacteriales",f:Chitinophagaceae

TACGGAGGGTGCAAGCGTTATCCGGATTTACTGGGTTTAAAGGGTGCGTAGGCGGGTAGG

TAAGTCAGTGGTGAAATCTTCGAGCTTAACTCGGAAATTGCCGTTGATACTATTTATCTT

GAATATCGTTGAGGTTTGCGGAATATGTCATGTAGCGGTGAAATGCTTAGAGATGACATA

GAACACCTATTGCGAAGGCAGCAGGCTAAACGAACATTGACGCTGAGGCACGAAAGCGTG

GGGATCAAAC

>ASV480 GS|97.2|KF999686_S004084195;k:Bacteria,p:"Bacteroidetes",c:Cytophagia,o:Cytophagales,f:Cytophagaceae,g:Spirosoma;

TACGGAGGGTGCGAGCGTTGTCCGGATTTATTGGGTTTAAAGGGTGCGTAGGTGGGTGAC

TAAGTCTGGTTTGAAAGCAGGTGGCTCAACCATCTGATGTGGCTGGAAACTGGTGATCTT

GAATGGGTTGGCGGTAGCCGGAACGGGTCATGTAGCGGTGAAATGCATAGATATGACCCA

GAACACCGATTGCGAAGGCAGGCTACTACGACTTGATTGACACTGAGGCACGAGAGCCGG

GGTAGCGAAC

>ASV481 GS|100.0|AB037000_S000383150;k:Bacteria,p:"Actinobacteria",c:Actinobacteria,o:Actinomycetales,f:Micromonosporaceae,g:Actinoplanes;

GACGTAGGGCGCGAGCGTTGTCCGGATTTATTGGGCGTAAAGAGCTCGTAGGCGGCTTGT

CGCGTCGACTGTGAAAACCCGCGGCTCAACCGCGGGCCTGCATTCGATACGGGCAGGCTA

GAGTTCGGTAGGGGAGACTGGAATTCCTGGTGTAGCGGTGAAATGCGCAGATATCAGGAG

GAACACCGATGGCGAAGGCAGGTCTCTGGGCCGATACTGACGCTGAGGAGCGAAAGCGTG

GGGAGCGAAC

>ASV482 GS|98.8|JQ346802_S003290742;k:Bacteria,p:"Actinobacteria",c:Actinobacteria,o:Actinomycetales,f:Cryptosporangiaceae,g:Jatrophihabitans;

TACGTAGGGTGCAAGCGTTGTCCGGAATTATTGGGCGTAAAGAGCTCGTAGGCGGTTTGT

CGCGTCGGCTGTGAAAACCTGGGGCTCAACCCCGGGCCTGCAGCCGATACGGGCAAACTA

GAATTCGGTAGGGGAGACTGGAATTCCTGGTGTAGCGGTGAAATGCGCAGATATCAGGAG

GAACACCGGTGGCGAAGGCGGGTCTCTGGGCCGATATTGACGCTGAGGAGCGAAAGCGTG

GGGAGCAAAC

>ASV483 GS|0.0|None;No hit

GTGTCAGCAGCCACGTTCAATCCGCGGAGATGCATAACCAAGCCGTCAACTCCCTGGCAT

TCATCTCCACCCGCTACACGCTCGAAAGTGCAGACCTGGTGTCGCTGATGAGCGCCTCTT

ACCTGTATGCCGTTTGTCAAGCCTTGGACCTGCGTGTGCTCCAGAAGATATTCTTCCAGC

ATCTCGAACCCGCACTCTTTGCGATCAACCTCGAGGTCCTCGGCGAATACCTCTCTCCGG

CCGCGATTAA

>ASV484 SS|1.0000|HM032898_S002167666;k:Bacteria,p:"Bacteroidetes",c:Cytophagia,o:Cytophagales,f:Cytophagaceae,g:Hymenobacter

TACGGAGGGTGCGAGCGTTGTCCGGATTTATTGGGTTTAAAGGGTGCGTAGGCGGCTTGC

TAAGTCTGGGGTGAAAGCCCGTTGCTCAACAACGGAACTGCCCTGGATACTGGCGAGCTT

GAGGACAGGCGAGGTTGGCGGAATGGAGGGTGTAGCGGTGAAATGCATAGATACCCTCCA

GAACCCCGATTGCGAAGGCAGCTGACTAGGCTGTACCTGACGCTGAGGCACGAAAGCGTG

GGGAGCGAAC

>ASV485 SS|1.0000|AB377116_S000994777;k:Bacteria,p:"Actinobacteria",c:Actinobacteria,o:Actinomycetales

TACGTAGGGTGCAAGCGTTGTCCGGAATTATTGGGCGTAAAGAGCTCGTAGGCGGTTCGT

CGCGTCTGCTGTGAAAACCTGGGGCTCAACCCCGGGCGTGCAGTGGGTACGGGCGGGCTA

GAGTACAGTAGGGGAGACTGGAATTCCTGGTGTAGCGGTGAAATGCGCAGATATCAGGAG

GAACACCGGTGGCGAAGGCGGGTCTCTGGGCTGTTACTGACGCTGAGGAGCGAAAGCGTG

GGGAGCGAAC

>ASV486 GS|0.0|None;No hit

TACGAGGGGGGCAAGCGTTATTCGAAATGACTGGGCGTAAAGGGCATGTAGGCGGTCTAT

TAAGTTAGTTTTAAAAAAAATTAAAAGCATCAGCTATGCAGCCCTTACCTACTGGTAAGG

ACTAATGGGCAATTGTGTGAAAAATTCCAAAGCTTAACTTTGGTAAAACACCTAATACTG

ATAGACTAGAGTAAGTGAGAGGAAAGTGGAATTCCTGGAGGAGAGATAAAATTTGTAGAT

ATCAGGAGGA

>ASV487 GS|0.0|None;No hit

TACGTGGAAGACTAGTGTTATTCATCTTTAATTGGTTTAAAGGGTACTTAGACGGTTAAT

CAAACCCCTAAAGGGTACTAGTTGACTAGAGTTTTATATGAGAAGGGGAGTACTAAAGAG

TGTAGAGATGAAATTCTGTCATACCTGCCTTGGAACAGGCAACGGCGAAGGCAACCTTTT

ATGTAAAAACTGACGTTGAAGGACGAAGGCTTAGGACACCAACAGG

>ASV488 GS|98.4|AB267478_S000721192;k:Bacteria,p:"Bacteroidetes",c:Sphingobacteriia,o:"Sphingobacteriales",f:Chitinophagaceae,g:Segetibacter;

TACGGAGGGTGCAAGCGTTATCCGGATTTACTGGGTTTAAAGGGTGCGTAGGTGGGAATG

TAAGTCAGTGGTGAAATCTCCGTGCTTAACATGGAAACTGCCATTGATACTATGTTTCTT

GAATTTTCTGGAGGTAAGCGGAATATGTCATGTAGCGGTGAAATGCTTAGATATGACATA

GAACACCAATTGCGAAGGCAGCTTACTACAGGGATATTGACACTGAGGCACGAAAGCGTG

GGGATCAAAC

>ASV489 SS|1.0000|JF999998_S002914924;k:Bacteria,p:"Bacteroidetes",c:Sphingobacteriia,o:"Sphingobacteriales",f:Sphingobacteriaceae

TACGGAGGATCCAAGCGTTATCCGGATTTATTGGGTTTAAAGGGTGCGTAGGCGGCCTGT

TAAGTCAGAGGTGAAAGACGGTGGCTCAACCATCGCAGTGCCTTTGATACTGACGGGCTT

GAATGCAGCTGAGGTAGGCGGAATGTGACAAGTAGCGGTGAAATGCATAGATATGTCACA

GAACACCAATTGCGAAGGCAGCTTACTAAAGTGTGATTGACGCTGAGGCACGAAAGCGTG

GGGATCAAAC

>ASV490 SS|0.8700|KJ528316_S004225669;k:Bacteria,p:"Proteobacteria",c:Alphaproteobacteria,o:Sphingomonadales,f:Sphingomonadaceae

TACGGAGGGGGCTAGCGTTGTTCGGAATTACTGGGCGTAAAGCGCACGTAGGCGGTTATT

CAAGTCAGGGGTGAAAGCCCGGAGCTCAACTCCGGAACTGCCTCTGAAACTAGATAACTT

GAATCATGGAGAGGCGAGTGGAATTCCGAGTGTAGAGGTGAAATTCGTAGATATTCGGAA

GAACACCAGTGGCGAAGGCGGCTCGCTGGACATGTATTGACGCTGAGGTGCGAAAGCGTG

GGGAGCAAAC

>ASV491 GS|0.0|None;No hit

GTGTCAGCAGCCAAGGCTCGAAAGACTAACGAGCTTCAGTAGAAAGCCACGAGCAGGTCT

CGTCGCTTGTGCTATCCTCAAAGCTGGAGCGCCTTCTCTGTTCTACTCCGTTCAAGGTCA

ACATCCCTCCCGACTACAACAGATATATGGAGTGGGGTTTCTTCGACGGTAGTGTACGGT

TCTATGCTGCGGACAGTGGGAAGGTCCGCATTCCTTCGTACCGATGTTTGTTTTCGAGAG

GTTAACTTTC

>ASV492 SS|0.8000|X99980_S000012437;k:Bacteria,p:Firmicutes

GACGTAGGAAGCAAGCGTTGTCCGGAATTACTGGGCGTAAAGAGCTCGTAGGCGGGAGCG

TAAGTCTGAAATGAAATCTCAAGGCTCAACTTTGGGGCTATTTCAGATACTGCGTTTCTT

GAGGGTATCAGAGGATAGTAGAATTCCCGGTGTAGCGGTGAAATGCGTAGATATCGGGAG

GAATACCAGTGGCGAAGGCGACTGTCTGGGATATTCCTGACGCTGAGGAGCGAAAGCGTG

GGGAGCAAAC

>ASV493 SS|1.0000|FN391026_S001418752;k:Bacteria,p:"Planctomycetes",c:Planctomycetia,o:Planctomycetales,f:Planctomycetaceae

GACGAACCGTGCGAACGTTATTCGGAATCACTGGGCTTAAAGCGCGTGTAGGCGGGCCGC

CGCGTCGGCTGCTGAAATCCCCCGGCTCAACCGGGGAACGGGCACCGATACGGGCGGCCT

CGAGGGGGGTAGGGGGGACTGGAACTTCCGGTGGAGCGGTGAAATGCGTTGAGATCGGAA

GGAACGCCCGTGGCGAAAGCGAGTCCCTGGACCCTTTCTGACGCTGAGACGCGAAAGCCA

GGGGAGCGAA

>ASV494 SS|1.0000|KF437572_S004053197;k:Bacteria,p:"Proteobacteria",c:Alphaproteobacteria,o:Sphingomonadales,f:Sphingomonadaceae

TACGGAGGGGGCTAGCGTTGTTCGGAATTACTGGGCGTAAAGCGTACGTAGGCGGCTTTT

TAAGTCAGAGGTGAAATCCCGGTGCTCAACACCGGAACTGCCTTTGAGACTGGATCGCTT

GAACGTCGGAGAGGTGAGTGGAATTCCGAGTGTAGAGGTGAAATTCGTAGATATTCGGAA

GAACACCAGTGGCGAAGGCGGCTCACTGGACGACTGTTGACGCTGAGGTACGAAAGCGTG

GGGAGCAAAC

>ASV495 GS|97.6|GU295962_S001873908;k:Bacteria,p:"Bacteroidetes",c:Sphingobacteriia,o:"Sphingobacteriales",f:Chitinophagaceae,g:Flavitalea;

TACGGAGGGTGCAAGCGTTATCCGGATTCACTGGGTTTAAAGGGTGCGTAGGCGGGTTGG

TAAGTCCGTGGTGAAATCTCCGAGCTTAACTCGGAAACTGCCGTGGATACTATCAGTCTT

GAATATCGTTGAGGTAAGCGGAATATGTCATGTAGCGGTGAAATGCTTAGATATGACATA

GAACACCAATTGCGAAGGCAGCTTACTGAACGGTCATTGACGCTGAGGCACGAAAGCGTG

GGGAGCAAAC

>ASV496 SS|0.8700|AB597950_S002440613;k:Bacteria,p:"Actinobacteria",c:Actinobacteria,o:Solirubrobacterales,f:Conexibacteraceae,g:Conexibacter

TACGTAGGGGGCAAGCGTTGTCCGGAATCATTGGGCGTAAAGCGCGTGTAGGCGGGTCGG

TAAGTCTGCTCTGAAAGTCCAAGGCTCAACCTTGGGATGCGGGTGGATACTGCCGACCTC

GAGTCCGGAAGAGGCGAGTGGAATTCCTGGTGTAGCGGTGAAATGCGCAGATATCAGGAG

GAACACCAATGGCGAAGGCAGCTCGCTGGGACGTGACTGACGCTGAGACGCGAAAGCGTG

GGGAGCAAAC

>ASV497 GS|0.0|None;No hit

TACGTGGAAGACTAGTGTTATTCATCTTTATTAGGTTTAAAGGGTACCTAGACGGTATTT

CTAGCCCAAAATAGGGTACGGATTTACTAGAGTTTTATGTGAGGAGGGGAGTACTCGTGG

AGTAGAGATGAAATTTTGTTATACTATGAGGTAACTGATAGCGGCGAAAGCAACCTTCTA

TGTATAAACTGACGTTGAGGGACGAAGGCTCGGGTAGCAAATAGG

>ASV498 SS|0.9100|KF360052_S004051835;k:Bacteria,p:"Proteobacteria",c:Alphaproteobacteria,o:Caulobacterales,f:Caulobacteraceae,g:Caulobacter

TACGAAGGGGGCTAGCGTTGCTCGGAATTACTGGGCGTAAAGGGAGCGTAGGCGGATGTT

TAAGTCAGAGGTGAAAGCCCAGGGCTCAACCTTGGAACTGCCTTTGATACTGGGCATCTT

GAGTGTGGGAGAGGTAAGCGGAACTCCGAGTGTAGAGGTGAAATTCGTAGATATTCGGAA

GAACACCAGTGGCGAAGGCGGCTTACTGGCCCATTACTGACGCTGAGGCTCGAAAGCGTG

GGGAGCAAAC

>ASV499 SS|1.0000|AM947653_S001093907;k:Bacteria,p:"Proteobacteria",c:Alphaproteobacteria,o:Rhodospirillales,f:Acetobacteraceae

TACGAAGGGGGCTAGCGTTGCTCGGAATGACTGGGCGTAAAGGGCGCGTAGGCGGACATA

TTAGTCAGGCGTGAAATTCCCGGGCTTAACCTGGGGGCTGCGTTTGATACGGTGTGTCTA

GAGTTTGGAAGAGGGTCGTGGAATTCCCAGTGTAGAGGTGAAATTCGTAGATATTGGGAA

GAACACCGGTGGCGAAGGCGGCGACCTGGTCCTTGACTGACGCTGAGGCGCGAAAGCGTG

GGGAGCAAAC

>ASV500 GS|97.2|KJ528316_S004225669;k:Bacteria,p:"Proteobacteria",c:Alphaproteobacteria,o:Sphingomonadales,f:Sphingomonadaceae,g:Blastomonas;

TACGGAGGGGGCTAGCGTTGTTCGGAATTACTGGGCGTAAAGCGCACGTAGGCGGCTATT

CAAGTCAGAGGTGAAAGCCCGGAGCTCAACTCCGGAACTGCCTTTGAAACTAGGTAGCTT

GAATCATGGAGAGGCGAGTGGAATTCCGAGTGTAGAGGTGAAATTCGTAGATATTCGGAA

GAACACCAGTGGCGAAGGCGGCTCGCTGGACATGTATTGACGCTGAGGTGCGAAAGCGTG

GGGAGCAAAC

>ASV501 SS|1.0000|HM032897_S002167665;k:Bacteria,p:"Bacteroidetes",c:Cytophagia,o:Cytophagales,f:Cytophagaceae,g:Hymenobacter

TACGGAGGGTGCGAGCGTTGTCCGGATTTATTGGGTTTAAAGGGTGCGTAGGCGGCCATT

TAAGTCTGGGGTGAAAGCCCGCTGCTCAACAGCGGAACTGCCCTGGATACTGGATGGCTT

GAATACAGTGGAGGTTGGCGGAATGGACTGAGTAGCGGTGAAATGCATAGATACAGTCCA

GAACCCCGATTGCGAAGGCAGCTGACTACACTGGTATTGACGCTGAGGCACGACAGCGTG

GGGAGCGAAC

>ASV502 GS|100.0|X81665_S000003452;k:Bacteria,p:"Proteobacteria",c:Gammaproteobacteria,o:Pseudomonadales,f:Moraxellaceae,g:Acinetobacter;

TACAGAGGGTGCAAGCGTTAATCGGATTTACTGGGCGTAAAGCGCGCGTAGGTGGCCAAT

TAAGTCAAATGTGAAATCCCCGAGCTTAACTTGGGAATTGCATTCGATACTGGTTGGCTA

GAGTATGGGAGAGGATGGTAGAATTCCAGGTGTAGCGGTGAAATGCGTAGAGATCTGGAG

GAATACCGATGGCGAAGGCAGCCATCTGGCCTAATACTGACACTGAGGTGCGAAAGCATG

GGGAGCAAAC

>ASV503 SS|1.0000|Y07841_S000002016;k:Bacteria,p:Firmicutes,c:Clostridia,o:Clostridiales,f:Clostridiales_Incertae_Sedis_XI,g:Anaerococcus

TACGTAAGGACCGAGCGTTGTCCGGAATCATTGGGCGTAAAGGGTACGTAGGCGGCTAGA

AAAGTTAGAAGTCAAAGGCTATAGCTCAACTATAGTAAGCTTCTAAAACTATTTAGCTTG

AGAAATGGAAGGGAAAGTGGAATTCCTAGTGTAGCGGTGGAATGCGCAGATATTAGGAAG

AATACCGGTGGCGAAGGCGACTTTCTGGCCATTATCTGACGCTGAGGTACGAAAGCGTGG

GTAGCAAACA

>ASV504 SS|1.0000|JF803808_S002913330;k:Bacteria,p:"Bacteroidetes",c:Sphingobacteriia,o:"Sphingobacteriales",f:Chitinophagaceae

TACGGAGGGTGCAAGCGTTATCCGGATTCACTGGGTTTAAAGGGTGCGTAGGTGGGTTGG

TAAGTCAGTGGTGAAATCTCCGGGCTTAACCCGGAAACTGCCATTGATACTACTAGTCTT

GAATGTCGTGGAGGTGAGCGGAATATGTCATGTAGCGGTGAAATGCTTAGATATGACATA

GAACACCAATTGCGAAGGCAGCTCACTACACGGATATTGACACTGAGGCACGAAAGCGTG

GGGATCAAAC

>ASV505 GS|0.0|None;No hit

GTGTCAGCAGCCGCGGTGGGCGAGAAAGCATCAGTAGAATACCGATGTGGTCCTCCACCG

TAGCTATTCCTGTTGTCGTCACTCTGTTGCCCCGCAAAGTCCGAAAAGTACACTTGCTGT

GAGTCCAACATGGTTGATTGCCTCGTCTGAATTCCATTCTGCGACCAGTAGTCC

>ASV506 SS|1.0000|AB003935_S000381721;k:Bacteria,p:"Actinobacteria",c:Actinobacteria,o:Actinomycetales

TACGTAGGGTGCAAGCGTTGTCCGGAATTATTGGGCGTAAAGAGCTCGTAGGCGGTCTGT

CGCGTCGGCTGTGAAAACTTGGGGCTCAACCCCAAGCCTGCAGTCGATACGGGCAGACTA

GAGTGCGGTAGGGGAGACTGGAATTCCTGGTGTAGCGGTGAAATGCGCAGATATCAGGAG

GAACACCGGTGGCGAAGGCGGGTCTCTGGGCCGTAACTGACGCTGAGGAGCGAAAGCGTG

GGGAGCGAAC

>ASV507 SS|1.0000|JX294485_S003614212;k:Bacteria,p:"Bacteroidetes",c:Cytophagia,o:Cytophagales,f:Cytophagaceae,g:Hymenobacter

TACGGAGGGTGCGAGCGTTGTCCGGATTTATTGGGTTTAAAGGGTGCGTAGGCGGCCTTA

TAAGTCTGGGGTGAAAGCCCGCTGCTCAACAGCGGAACTGCCCTGGATACTGTGGGGCTT

GAGGACAGACGAGGTTGGCGGAATGGAGGGTGTAGCGGTGAAATGCATAGATACCCTCCA

GAACCCCGATTGCGAAGGCAGCTGACTAGACTGTAACTGACGCTGAGGCACGAAAGCGTG

GGGAGCGAAC

>ASV508 SS|0.9300|AB267478_S000721192;k:Bacteria,p:"Bacteroidetes",c:Sphingobacteriia,o:"Sphingobacteriales",f:Chitinophagaceae,g:Segetibacter

TACGGAGGGTGCAAGCGTTATCCGGATTTACTGGGTTTAAAGGGTGCGTAGGTGGGAATG

TAAGTCAGTGGTGAAATCTCCGTGCTTAACATGGAAACTGCCATTGATACTATATTTCTT

GAATTTTCTGGAGGTTAGCGGAATATGTCATGTAGCGGTGAAATGCTTAGATATGACATA

GAACACCAATTGCGAAGGCAGCTGGCTACAGGAAAATTGACACTGATGCACGAAAGCGTG

GGGATCAAAC

>ASV509 GS|100.0|AF131295_S000387421;k:Bacteria,p:"Proteobacteria",c:Alphaproteobacteria,o:Sphingomonadales,f:Sphingomonadaceae,g:Sphingomonas;

TACGGAGGGAGCTAGCGTTGTTCGGAATTACTGGGCGTAAAGCGCACGTAGGCGGCTTTG

TAAGTTAGAGGTGAAAGCCTGGAGCTCAACTCCAGAACTGCCTTTAAGACTGCATCGCTT

GAATCCAGGAGAGGTGAGTGGAATTCCGAGTGTAGAGGTGAAATTCGTAGATATTCGGAA

GAACACCAGTGGCGAAGGCGGCTCACTGGACTGGTATTGACGCTGAGGTGCGAAAGCGTG

GGGAGCAAAC

>ASV510 GS|100.0|AF468450_S000395040;k:Bacteria,p:"Proteobacteria",c:Gammaproteobacteria,o:Pseudomonadales,f:Pseudomonadaceae,g:Pseudomonas;

TACAGAGGGTGCAAGCGTTAATCGGAATTACTGGGCGTAAAGCGCGCGTAGGTGGTTTGT

TAAGTTGGATGTGAAATCCCCGGGCTCAACCTGGGAACTGCATTCAAAACTGACAAGCTA

GAGTATGGTAGAGGGTGGTGGAATTTCCTGTGTAGCGGTGAAATGCGTAGATATAGGAAG

GAACACCAGTGGCGAAGGCGACCACCTGGACTGATACTGACACTGAGGTGCGAAAGCGTG

GGGAGCAAAC

>ASV511 SS|1.0000|JN090860_S002916046;k:Bacteria,p:"Bacteroidetes",c:Cytophagia,o:Cytophagales,f:Cytophagaceae,g:Hymenobacter

TACGGAGGGTGCGAGCGTTGTCCGGATTTATTGGGTTTAAAGGGTGCGTAGGCGGCCGTT

TAAGTCTGGGGTGAAAGCCCGCTGCTCAACAGCGGAACGGCCCTGGATACTGGACGGCTT

GAGTACAGACGAGGTTGGCGGAATGGACCGAGTAGCGGTGAAATGCATAGATACGGTCCA

GAACCCCGATTGCGAAGGCAGCTGACTAGGCTGATACTGACGCTGAGGCACGACAGCGTG

GGGAGCGAAC

>ASV512 SS|1.0000|DQ986200_S000736608;k:Bacteria,p:"Planctomycetes",c:Planctomycetia,o:Planctomycetales,f:Planctomycetaceae

GACGAACCGTGCGAACGTTATTCGGAATTACTGGGCTTAAAGCGCGTGTAGGCGGACCGG

CACGTTTGATGCTGAAATCCCCCGGCTCAACCGGGGAAGTGGCACAGATACGACCGGTCT

GGAGGGAGGTAGGGGGGTCTGGAACTTCCGGTGGAGCGGTGAAATGCGTTGAGATCGGAA

GGAACGCCCGTGGCGAAAGCGAGGCCCTGGACCTTTTCTGACGCTGAGACGCGAAAGCCA

GGGGAGCGAA

>ASV513 GS|98.0|EF407879_S000824637;k:Bacteria,p:"Bacteroidetes",c:Sphingobacteriia,o:"Sphingobacteriales",f:Chitinophagaceae,g:Sediminibacterium;

TACGGAGGGTGCAAGCGTTATCCGGATTCACTGGGTTTAAAGGGTGCGTAGGCGGGCAGG

TAAGTCAGTGGTGAAATCTTTGAGCTTAACTCGAAAACTGCCATTGATACTATCTATCTT

GAATATTGTGGAGGTAAGCGGAATATGTCATGTAGCGGTGAAATGCTTAGATATGACATA

GAACACCTATTGCGAAGGCAGCTTACTACGCATATATTGACGCTGAGGCACGAAAGCGTG

GGGATCAAAC

>ASV514 SS|1.0000|JX294485_S003614212;k:Bacteria,p:"Bacteroidetes",c:Cytophagia,o:Cytophagales,f:Cytophagaceae,g:Hymenobacter

TACGGAGGGTGCGAGCGTTGTCCGGATTTATTGGGTTTAAAGGGTGCGTAGGCGGCCAAA

TAAGTCTGGGGTGAAAGCCCGCTGCTCAACAGCGGAACTGCCCTGGATACTGTATGGCTT

GAGGACAGACGAGGTTGGCGGAATAGAGGGTGTAGCGGTGAAATGCATAGATACCCTCTA

GAACCCCGATTGCGAAGGCAGCTGACTAGACTGTATCTGACGCTGAGGCACGAAAGCGTG

GGGAGCGAAC

>ASV515 SS|0.8300|DQ200983_S000735521;k:Bacteria,p:"Actinobacteria",c:Actinobacteria,o:Actinomycetales,f:Geodermatophilaceae

TACGTAGGGTGCAAGCGTTGTCCGGAATTATTGGGCGTAAAGAGCTCGTAGGCGGTCTGT

CACGTCGGCTGTGAAAACCCGAGGCTCAACCTCGGGCCTGCAGTCGATACGGGCAGACTA

GAGTTTGGTAGGGGAGACTGGAATTCCTGGTGTAGCGGTGAAATGCGCAGATATCAGGAG

GAACACCGGTGGCGAAGGCGGGTCTCTGGGCCAAAACTGACGCTGAGGAGCGAAAGCGTG

GGGAGCAAAC

>ASV516 GS|100.0|HQ439186_S002305818;k:Bacteria,p:"Proteobacteria",c:Alphaproteobacteria,o:Sphingomonadales,f:Sphingomonadaceae,g:Sphingomonas;

TACGGAGGGAGCTAGCGTTGTTCGGAATTACTGGGCGTAAAGCGCACGTAGGCGGCTTTG

TAAGTTAGAGGTGAAAGCCTGGAGCTCAACTCCAGAATTGCCTTTGATACTGCATGGCTT

GAATCCAGGAGAGGTGAGTGGAATTCCGAGTGTAGAGGTGAAATTCGTAGATATTCGGAA

GAACACCAGTGGCGAAGGCGGCTCACTGGACTGGTATTGACGCTGAGGTGCGAAAGCGTG

GGGAGCAAAC

>ASV517 SS|0.8700|JN674641_S002960635;k:Bacteria,p:"Bacteroidetes",c:Sphingobacteriia,o:"Sphingobacteriales",f:Chitinophagaceae

TACGGAGGGTGCAAGCGTTATCCGGATTTACTGGGTTTAAAGGGTGTGTAGGCGGACATT

TAAGTCAGAGGTGAAATCCTGAGGCTCAACCTCAGAACTGCCCCTGATACTATTTGTCTT

GAATACTGATGAGGTGGGCGGAACGGGTCATGTAGCGGTGAAATGCTTAGAGATGACCCG

GAACACCGATTGCGAAGGCAGCTCACTGGGCAGTTATTGACGCTGAGGCACGAAAGCGTG

GGGATCAAAC

>ASV518 SS|1.0000|AJ717391_S000544176;k:Bacteria,p:"Proteobacteria",c:Alphaproteobacteria,o:Caulobacterales,f:Caulobacteraceae

TACGAAGGGGGCTAGCGTTGCTCGGAATTACTGGGCGTAAAGGGCGCGTAGGCGGATCGT

TAAGTTAGAGGTGAAAGCCCAGGGCTCAACCCTGGAATTGCCTTTGATACTGGCGATCTT

GAGTACGGGAGAGGTGTGTGGAACTCCGAGTGTAGAGGTGAAATTCGTAGATATTCGGAA

GAACACCAGTGGCGAAGGCGACACACTGGCCCGTTACTGACGCTGAGGCGCGAAAGCGTG

GGGAGCAAAC

>ASV519 SS|0.9900|DQ022076_S000537030;k:Bacteria,p:"Deinococcus-Thermus",c:Deinococci,o:Deinococcales,f:Trueperaceae,g:Truepera

TACGGAGGGTGCAAGCGTTATCCGGAATCACTGGGCGTAAAGGGCGCGTAGGCGGTTTGT

TAAGTCCGATGTTAAAGACCGAGGCTCAACCTCGACACGGCGTTGGATACTGGCAAGCTG

GACGGTTGGAGAGGAAGGTAGAATTACCAGTGTAGCGGTGGAATGCGTAGATACTGGTAG

GAATACCCATTGCGAAGGCAGCCTTCTGGACAACACGTGACGCTGAGGCGCGAAAGTGTG

GGGAGCAAAC

>ASV520 GS|97.2|HM032898_S002167666;k:Bacteria,p:"Bacteroidetes",c:Cytophagia,o:Cytophagales,f:Cytophagaceae,g:Hymenobacter;

TACGGAGGGTGCGAGCGTTGTCCGGATTTATTGGGTTTAAAGGGTGCGTAGGCGGCTTTT

TAAGTCCGGGGTGAAAGCCCGTTGCTCAACAACGGAACTGCCCTGGATACTGGAGAGCTT

GAGTACAGACGAGGTTGGCGGAATGGACGGAGTAGCGGTGAAATGCATAGATACCGTCCA

GAACCCCGATTGCGAAGGCAGCTGACTAGGCTGTTACTGACGCTGAGGCACGAAAGCGTG

GGGAGCGAAC

>ASV521 SS|1.0000|HQ436503_S002339683;k:Bacteria,p:"Proteobacteria",c:Alphaproteobacteria,o:Rhodospirillales,f:Acetobacteraceae

TACGAAGGGGGCTAGCGTTGCTCGGAATGACTGGGCGTAAAGGGCGCGTAGGCGGATTTG

TCAGTCAGACGTGAAATTCCTGGGCTTAACCTGGGGGCTGCGTTTGAGACGGCGGGTCTA

GAGTTTGGAAGAGGGTCGTGGAATTCCCAGTGTAGAGGTGAAATTCGTAGATATTGGGAA

GAACACCGGTGGCGAAGGCGGCGACCTGGTCCTGGACTGACGCTGAGGCGCGAAAGCGTG

GGGAGCAAAC

>ASV522 GS|100.0|AJ635303_S000471776;k:Bacteria,p:"Proteobacteria",c:Alphaproteobacteria,o:Rhizobiales,f:Methylobacteriaceae,g:Methylobacterium;

TACGAAGGGGGCTAGCGTTGCTCGGAATCACTGGGCGTAAAGGGCGCGTAGGCGGCTGAT

TTAGTCGAGGGTGAAAGCCCGTGGCTCAACCACGGAATGGCCTTCGATACTGGTTGGCTT

GAGACCGGAAGAGGACAGCGGAACTGCGAGTGTAGAGGTGAAATTCGTAGATATTCGCAA

GAACACCAGTGGCGAAGGCGGCTGTCTGGTCCGGTTCTGACGCTGAGGCGCGAAAGCGTG

GGGAGCAAAC

>ASV523 GS|100.0|D32224_S000469010;k:Bacteria,p:"Proteobacteria",c:Alphaproteobacteria,o:Rhizobiales,f:Methylobacteriaceae,g:Methylobacterium;

TACGAAGGGGGCTAGCGTTGCTCGGAATCACTGGGCGTAAAGGGCGCGTAGGCGGCCGAT

TAAGTCGGGGGTGAAAGCCTGTGGCTCAACCACAGAATTGCCTTCGATACTGGTTGGCTT

GAGACCGGAAGAGGACAGCGGAACTGCGAGTGTAGAGGTGAAATTCGTAGATATTCGCAA

GAACACCAGTGGCGAAGGCGGCTGTCTGGTCCGGTTCTGACGCTGAGGCGCGAAAGCGTG

GGGAGCAAAC

>ASV524 GS|0.0|None;No hit

CTTGGTCATTTAGAGGAAGTAAAAGTCGTAACAAGGTTTCCTTCCGGGTGTAGCACCTGC

CGAAGCCTCCCGCAGCGACTCTAAAGAAACCGCGCAGTCTGCCCCCCTGCAGGCAACACT

GTCACTGTGCTGGGAGCCAGTCACGCCTGGTTCAGGCGCCGCTACCAGCAACCTGGGCAA

CAACGCCCAGGCTCACAGATCAGATGATTGTGGCCACACCGTGGTTAAGATATGACCGGT

CCCCGCTGTG

>ASV525 SS|1.0000|AM947653_S001093907;k:Bacteria,p:"Proteobacteria",c:Alphaproteobacteria,o:Rhodospirillales,f:Acetobacteraceae

TACGAAGGGGGCTAGCGTTGCTCGGAATGACTGGGCGTAAAGGGCGCGTAGGCGGACATT

TTAGTCAGACGTGAAATTCCTGGGCTCAACCTGGGGGCTGCGTTTGATACGGGATGTCTA

GAGTTTGGAAGAGGGTTGTGGAATTCCCAGTGTAGAGGTGAAATTCGTAGATATTGGGAA

GAACACCGGTGGCGAAGGCGGCAACCTGGTCCTTGACTGACGCTGAGGCGCGAAAGCGTG

GGGAGCAAAC

>ASV526 SS|1.0000|EF368368_S000806277;k:Bacteria,p:"Proteobacteria",c:Alphaproteobacteria,o:Rhodospirillales,f:Acetobacteraceae

TACGAAGGGGGCTAGCGTTGCTCGGAATGACTGGGCGTAAAGGGCGCGTAGGCGGCTTGG

TTAGTCAGACGTGAAATTCCTGGGCTCAACCTGGGGGCTGCGTTTGATACAGCTGGGCTG

GAGTGGGGAAGAGGGTTGTGGAATTCCCAGTGTAGAGGTGAAATTCGTAGATATTGGGAA

GAACACCGGTGGCGAAGGCGGCAACCTGGTCCTTGACTGACGCTGAGGCGCGAAAGCGTG

GGGAGCAAAC

>ASV527 SS|1.0000|AB841026_U010573774;k:Bacteria,p:"Actinobacteria",c:Actinobacteria,o:Actinomycetales

TACGTAGGGTGCAAGCGTTGTCCGGATTTATTGGGCGTAAAGAGCTCGTAGGCGGTTTGT

CGCGTCGGCTGTGAAAACTCGGGGCTCAACCCCGAGCCTGCAGTCGATACGGGCAGACTA

GAGTTCGGTAGGGGAGACTGGAATTCCTGGTGTAGCGGTGAAATGCGCAGATATCAGGAG

GAACACCGATGGCGAAGGCAGGTCTCTGGGCCGATACTGACGCTGAGGAGCGAAAGCGTG

GGGAGCGAAC

>ASV528 SS|1.0000|AM000023_S000629072;k:Bacteria,p:"Bacteroidetes",c:Cytophagia,o:Cytophagales,f:Cytophagaceae,g:Spirosoma

TACGGAGGGTGCAAGCGTTGTCCGGATTTATTGGGTTTAAAGGGTGCGCAGGTGGTTCTG

TAAGTCTGATTTGAAAGCCGGCGGCTCAACCGTCGGATGTGGTTGGAAACTGTGGAACTT

GAATTTGGTAGCGGTAGCCGGAATGGGTCATGTAGCGGTGAAATGCATAGATATGACCCG

GAACACCGATTGCGAAGGCAGGCTACTGGGCCGATATTGACACTGAGGCACGAGAGCATG

GGTAGCGAAC

>ASV529 SS|1.0000|DQ986200_S000736608;k:Bacteria,p:"Planctomycetes",c:Planctomycetia,o:Planctomycetales,f:Planctomycetaceae

GACGAACCGTGCGAACGTTATTCGGAATCACTGGGCTTAAAGCGCGTGTAGGCGGGCCGC

CGCGTCGGTCGCTGAAATCCCCCGGCTCAACCGGGGAAGTGGCGTCGAAACGGGCGGCCT

GGAGGGGGGTAGGGGGGCCTGGAACTTCCGGTGGAGCGGTGAAATGCGTTGAGATCGGAA

GGAACGCCCGTGGCGAAAGCGAGGCCCTGGACCCTTACTGACGCTGAGACGCGAAAGCCA

GGGGAGCGAA

>ASV530 GS|100.0|AY204889_S000417782;k:Bacteria,p:Firmicutes,c:Bacilli,o:Lactobacillales,f:Lactobacillaceae,g:Lactobacillus;

TACGTAGGTGGCAAGCGTTGTCCGGATTTATTGGGCGTAAAGCGAGCGCAGGCGGTTTCT

TAAGTCTGATGTGAAAGCCTTCGGCTCAACCGAAGAAGTGCATCGGAAACTGGGAAACTT

GAGTGCAGAAGAGGACAGTGGAACTCCATGTGTAGCGGTGAAATGCGTAGATATATGGAA

GAACACCAGTGGCGAAGGCGGCTGTCTGGTCTGTAACTGACGCTGAGGCTCGAAAGCATG

GGTAGCAAAC

>ASV531 SS|1.0000|FJ177532_S001188354;k:Bacteria,p:"Bacteroidetes",c:Sphingobacteriia,o:"Sphingobacteriales",f:Chitinophagaceae

TACGGAGGGTGCAAGCGTTATCCGGATTCACTGGGTTTAAAGGGTGCGTAGGTGGGTCTG

TAAGTCAGTGGTGAAATCTTCGAGCTTAACTCGGAAACTGCCATTGATACTATAGGTCTT

GAATTTTCTGGAGGTAAGCGGAATATGTCATGTAGCGGTGAAATGCTTAGATATGACATA

GAACACCAATTGCGAAGGCAGCTTACTACGGGAATATTGACACTGAGGCACGAAAGCGTG

GGGATCAAAC

>ASV532 GSL|99.2|AF131295_S000387421;k:Bacteria,p:"Proteobacteria",c:Alphaproteobacteria,o:Sphingomonadales,f:Sphingomonadaceae,g:Sphingomonas

TACGGAGGGAGCTAGCGTTGTTCGGAATTACTGGGCGTAAAGCGCACGTAGGCGGCTTTG

TAAGTAAGAGGTGAAAGCCTGGAGCTCAACTCCAGAACTGCCTTTTAGACTGCATCGCTT

GAATCCAGGAGAGGTGAGTGGAATTCCGAGTGTAGAGGTGAAATTCGTAGATATTCGGAA

GAACACCAGTGGCGAAGGCGGCTCACTGGACTGGTATTGACGCTGAGGTGCGAAAGCGTG

GGGAGCAAAC

>ASV533 GS|78.0|AY512588_S004009252;k:Bacteria,p:"Elusimicrobia",c:Endomicrobia,g:Candidatus_Endomicrobium;

TACGTAGGTGGCAAGCGTTGTCCGGATTTACTGGGCGTAAAGCGTATGCAGGCGGATACT

TAAGTAGGAAGTGAAAGGTTGCAGCTCAACTGCAACACTGCTTCCTATACTGGGTGTCTT

GAGTGGCGGAGAGGGAGATGGAACGACACGTGTAGCGGTGAAATGCGTTGATATGTGTCG

GAACACCAATGGCGAAAGCAATCTCCTGGACGCAAACTGACGCTGAGATACGAAAGCTAA

GGTAGCAAAC

>ASV534 SS|1.0000|DQ986200_S000736608;k:Bacteria,p:"Planctomycetes",c:Planctomycetia,o:Planctomycetales,f:Planctomycetaceae

GACGAACCGTGCGAACGTTATTCGGAATTACTGGGCTTAAAGCGCGTGTAGGCGGACCGG

CACGTCCGATGCTGAAATCCCCCGGCTCAACCGGGGAAGTGGCACAGATACGACCGGTCT

GGAGGGAGGTAGGGGGGTCTGGAACTTCCGGTGGAGCGGTGAAATGCGTTGAGATCGGAA

GGAACGCCCGTGGCGAAAGCGAGGCCCTGGACCTTTTCTGACGCTGAGACGCGAAAGCCA

GGGGAGCGAA

>ASV535 SS|0.8300|AB067734_S000384429;k:Bacteria,p:"Actinobacteria",c:Actinobacteria,o:Actinomycetales,f:Nocardiaceae

TACGTAGGGTGCGAGCGTTGTCCGGAATTACTGGGCGTAAAGAGCTCGTAGGCGGTTTGT

CACGTCGGCTGTGAAATCCCACCGCTCAACGGTGGGCTTGCAGTCGATACGGGCTGACTT

GAGTACTGCAGGGGAGACTGGAATTCCTGGTGTAGCGGTGAAATGCGCAGATATCAGGAG

GAACACCGGTGGCGAAGGCGGGTCTCTGGGCAGTAACTGACGCTGAGGAGCGAAAGCGTG

GGTAGCAAAC

>ASV536 GS|98.4|FM998003_S002355582;k:Bacteria,p:"Actinobacteria",c:Actinobacteria,o:Actinomycetales,g:Motilibacter;

TACGTAGGGTGCAAGCGTTGTCCGGAATTATTGGGCGTAAAGAGCTCGTAGGTGGCGAGT

CACGTCGGGTGTGAAAGCCCGGGGCTTAACCCCGGGTCTGCATTCGATACGGGCTTGCTA

GGGTCCGGCAGGGGAGACTGGAATTCCTGGTGTAGCGGTGAAATGCGCAGATATCAGGAG

GAACACCGGTGGCGAAGGCGGGTCTCTGGGCCGGTACCGACACTGAGGAGCGAAAGCATG

GGGAGCAAAC

>ASV537 SS|1.0000|JQ309130_S003619637;k:Bacteria,p:"Acidobacteria",c:Acidobacteria_Gp4

TACGTAGGGACCAAGCGTTGTTCGGATTTACTGGGCGTAAAGGGCGCGTAGGCGGCGTGA

CAAGTCACTTGTGAAATCTCCGGGCTTAACTCGGAACGGTCAAGTGATACTGTTGTGCTA

GAGTACAGAAGGGGCAATCGGAATTCTCGGTGTAGCGGTGAAATGCGTAGATATCGAGAG

GAACACCAGAGGCGAAGGCGGATTGCTGGGCTGATACTGACGCTGAGGCGCGAAAGCTAG

GGTAGCAAAC

>ASV538 SS|0.9600|JQ309130_S003619637;k:Bacteria,p:"Acidobacteria",c:Acidobacteria_Gp4,g:Blastocatella

TACGTAGGGACCAAGCGTTGTTCGGATTTACTGGGCGTAAAGGGCGCGTAGGCGGCGTGA

CAAGTCACTTGTGAAATCTCCGAGCTTAACTCGGAACGGTCAAGTGATACTGTTATGCTA

GAGTACAGAAGGGGTAATCGGAATTCTTGGTGTAGCGGTGAAATGCGTAGATATCAAGAG

GAACACCAGAGGCGAAGGCGGATTACTAGGCTGATACTGACGCTGAGGCGCGAAAGCTAG

GGTAGCAAAC

>ASV539 GS|97.2|KF437572_S004053197;k:Bacteria,p:"Proteobacteria",c:Alphaproteobacteria,o:Sphingomonadales,f:Sphingomonadaceae,g:Rhizorhabdus;

TACGGAGGGGGCTAGCGTTGTTCGGAATTACTGGGCGTAAAGCGTACGTAGGCGGCTTTT

TAAGTCAGAGGTGAAATCCCGGGGCTCAACTCCGGAACTGCCTTTGAGACTGGATCGCTT

GAACGTCGGAGAGGTGAGTGGAATTCCGAGTGTAGAGGTGAAATTCGTAGATATTCGGAA

GAACACCAGTGGCGAAGGCGGCTCACTGGACGACTGTTGACGCTGAGGTACGAAAGCGTG

GGGAGCAAAC

>ASV540 SS|1.0000|KF309176_S003920854;k:Bacteria,p:"Bacteroidetes",c:Sphingobacteriia,o:"Sphingobacteriales",f:Chitinophagaceae

TACGGAGGGTGCAAGCGTTATCCGGATTCACTGGGTTTAAAGGGTGCGTAGGTGGGTAGG

TAAGTCCGTGGTGAAATCTCTGAGCTTAACTCAGAAACTGCCGTGGATACTATTTATCTT

GAATATTGTGGAGGTGAGCGGAATATGTCATGTAGCGGTGAAATGCTTAGATATGACATA

GAACACCAATTGCGAAGGCAGCTCACTACACAATCATTGACGCTGAGGCACGAAAGCGTG

GGGATCAAAC

>ASV541 GS|98.0|HQ687089_S002989304;k:Bacteria,p:"Acidobacteria",c:Acidobacteria_Gp1,g:Granulicella;

TACGAGGGGGGCAAGCGTTGTTCGGAATTATTGGGCGTAAAGGGTGCGTAGGCGGTTTGA

CAAGTCTTATGTGAAATCTCTGGGCTCAACCCAGAGTCTGCATAGGAAACTGTCGGGCTT

GAGTATTGGAGAGGTGAGTGGAATTTCCGGTGTAGCGGTGAAATGCGTAGATATCGGAAG

GAACACCTGTGGCGAAAGCGGCTCACTGGACAATAACTGACGCTGATGCACGAAAGCTAG

GGGAGCAAAC

>ASV542 SS|0.8400|AM231587_S000650722;k:Bacteria,p:"Proteobacteria",c:Alphaproteobacteria,o:Rhodospirillales,f:Acetobacteraceae,g:Roseomonas

TACGAAGGGGGCTAGCGTTGCTCGGAATTACTGGGCGTAAAGGGCGCGTAGGCGGCACCG

CTCGTCAGGCGTGAAATTCCTGGGCTCAACCTGGGGACTGCGCTTGATACGGCGGAGCTA

GAGGATGGAAGAGGGTCGTGGAATTCCCAGTGTAGAGGTGAAATTCGTAGATATTGGGAA

GAACACCGGTGGCGAAGGCGGCGACCTGGTCCATTACTGACGCTGAGGCGCGACAGCGTG

GGGAGCAAAC

>ASV543 SS|1.0000|KF999686_S004084195;k:Bacteria,p:"Bacteroidetes",c:Cytophagia,o:Cytophagales,f:Cytophagaceae,g:Spirosoma

TACGGAGGGTGCGAGCGTTGTCCGGATTTATTGGGTTTAAAGGGTGCGTAGGTGGGTGGC

TAAGTCTGGTTTGAAAGCAGGCGGCTCAACCGTCTGATGTGGCTGGAAACTGGTTATCTT

GAATGGGTTGGCGGCTGCCGGAACGGGTCATGTAGCGGTGAAATGCATAGATATGACCCA

GAACACCGATTGCGAAGGCAGGCAGCTACGACTTGATTGACACTGAGGCACGAGAGCCGG

GGTAGCGAAC

>ASV544 SS|1.0000|D86513_S000011943;k:Bacteria,p:"Proteobacteria",c:Alphaproteobacteria,o:Rhodospirillales,f:Acetobacteraceae

TACGAAGGGGGCTAGCGTTGCTCGGAATGACTGGGCGTAAAGGGCGCGTAGGCGGATGGC

ACAGTCAGATGTGAAATTCCCGGGCTTAACCTGGGGGCTGCATTTGATACGTGTTGTCTA

GAGTGAGGAAGAGGGTCGTGGAATTCCCAGTGTAGAGGTGAAATTCGTAGATATTGGGAA

GAACACCGGTGGCGAAGGCGGCGACCTGGTCCTTGACTGACGCTGAGGCGCGAAAGCGTG

GGGAGCAAAC

>ASV545 SS|1.0000|AM947653_S001093907;k:Bacteria,p:"Proteobacteria",c:Alphaproteobacteria,o:Rhodospirillales,f:Acetobacteraceae

TACGAAGGGGGCTAGCGTTGCTCGGAATGACTGGGCGTAAAGGGCGCGTAGGCTGGCATC

TTAGTCAGGCGTGAAATTCCCGGGCTTAACCTGGGGGCTGCGTTTGATACGGGGTGCCTA

GAGTTTGGAAGAGGGTCGTGGAATTCCCAGTGTAGAGGTGAAATTCGTAGATATTGGGAA

GAACACCGGTGGCGAAGGCGGCGACCTGGTCCTTGACTGACGCTGAGGCGCGAAAGCGTG

GGGAGCAAAC

>ASV546 GS|100.0|AM231587_S000650722;k:Bacteria,p:"Proteobacteria",c:Alphaproteobacteria,o:Rhodospirillales,f:Acetobacteraceae,g:Roseomonas;

TACGAAGGGGGCTAGCGTTGCTCGGAATTACTGGGCGTAAAGGGCGCGTAGGCGGCTTAG

TTAGTCAGGCGTGAAATTCCTGGGCTCAACCTGGGGACTGCGCTTGATACGGCTAGGCTA

GAGGATGGAAGAGGGTCGCGGAATTCCCAGTGTAGAGGTGAAATTCGTAGATATTGGGAA

GAACACCGGTGGCGAAGGCGGCGACCTGGTCCATTACTGACGCTGAGGCGCGACAGCGTG

GGGAGCAAAC

>ASV547 SS|1.0000|JQ345500_S003290740;k:Bacteria

GACGTAGGAGGCGAGCGTTGTCCGGAGTTACTGGGCGTAAAGCGCGCGCAGGCGGTCCCG

CAGGTCACCTGTGAAAGCCCCCGGCTCAACCGGGCGGAGGCGGGTGAAACCGCGGGACTG

GAGGGCGGCAGAGGGTCGTGGAATTCCCGGTGTAGTGGTGAAATGCGTAGAGATCGGGAG

GAACACCCGCGGCGAAGGCGGCGACCTGGACCGACCCTGACGCTGAGGCGCGAAGGCCGG

GGGAGCGAAC

>ASV548 SS|1.0000|EU135017_S000933611;k:Bacteria

TACGTAGGGTCCGAGCGTTGTCCGGAATTACTGGGCGTAAAGCGTGCGCAGGCGGCCCGT

AAGGTGTCACGTGAAAGCCCCGGGCTCAACCCGGGAGGGTCGTGGCAAACCGGCGGGCTT

GAGGCCGGTAGAGGTGGGTGGAACTCCTGGTGGAGTGGTGAAATGCGTAGAGATCAGGAA

GAACACCCGTGGCGAAGGCGGCCCACTGGGCCGGTACTGACGCTGAGGCACGAAGGCGTG

GGGAGCGAAC

>ASV549 GS|0.0|None;No hit

GTGTCAGCAGCCGCAGTCCTGTACCATCGCTTCGCTTCGCTTCCCTTCCCTTGACCTCCC

TTCCCTTTCCTTCACTCAATTAGGTACGTACTCGACAGAAGAACAGGGCATACGACGCAT

CCGCATCCGCATCTGCATCTGCATGCAAGCACTCCGATGACGCCTCCCCGCACCTTCCGC

GTACTAACGGGACTAACCCCTCTCCCTACGCCTTACAAGGCTGCAGGCCAACCCCCGCAC

GAACTTACGA

>ASV550 SS|0.8900|CP001854_S001872674;k:Bacteria,p:"Actinobacteria",c:Actinobacteria,o:Solirubrobacterales

TACGTAGGGGGCAAGCGTTGTCCGGAATCATTGGGCGTAAAGAGCGTGTAGGCGGCCTGA

TAAGTCGGCTGTGAAAGTCAAGGGCTCAACCCTTGAATGCCGGCCGATACTGTCAGGCTA

GAGTCCGGAAGAGGCGAGTGGAATTCCTGGTGTAGCGGTGAAATGCGCAGATATCAGGAG

GAACACCAATGGCGAAGGCAGCTCGCTGGGACGGTACTGACGCTGAGACGCGAAAGCGTG

GGGAGCGAAC

>ASV551 SS|0.9800|X64380_S000749517;k:Bacteria,p:"Verrucomicrobia",c:Spartobacteria,g:Spartobacteria_genera_incertae_sedis

TACAGAGGCCTCAAGCGTTGTTCGGATTCATTGGGCGTAAAGGGAGCGTAGGCGGTCGGG

TAAGTCGGGCGTGAAATCCTGGGGCTCAACCCCAGAACTGCGTTCGATACTGCTCGGCTA

GAGGACTGGAGAGGAGATTGGAATTCACGGTGTAGCAGTGAAATGCGTAGATATCGTGAG

GAAGACCAGTGGCGAAGGCGGATCTCTGGACAGTATCTGACGCTGAGGCTCGAAGGCCAG

GGGAGCAAAC

>ASV552 GS|0.0|None;No hit

TACGGGGGGGGCGAGCGTTATTCGAAATGATTGGGCGTAAAGAGCACGTAGACGGTTTTT

TAAGTGGACATTATATCTTTTTTGTTCTCTAAGGATAAAAAAGAAAGGATTATGGAAATA

TTTCTGTACTCGGGAAAAGGACCAAGGCTCAACCATGGTGTTTCCCGCCAAACTAAAAAA

CTAGAGTAAGTTAGAGGAAAGTGGAATTCCTGGAGGAAAGGTTAAATTTTATGATATCAG

GAGGAACGCC

>ASV553 SS|1.0000|EF457480_S000834905;k:Bacteria

TACGTAGGGGTCGAGCGTTGTCCGGAGTTACTGGGCGTAAAGCGTGCGCAGGCGGCTCAT

TGCGCCCGGCGTGAAAGCCCCCGGCTCAACCGGGGAGGGTCGTCGGGGACGGGTGAGCTT

GAGGGTATCAGGGGCTGGTGGAATTCCCGGTGTAGTGGTGAAATGCGTAGAGATCGGGAG

GAACACCCGTGGCGAAGGCGGCCAGCTGGGATGCACCTGACGCTGAGGCACGAAGGCGTG

GGGAGCGAAC

>ASV554 GS|98.8|EF363714_S000806231;k:Bacteria,p:"Proteobacteria",c:Alphaproteobacteria,o:Sphingomonadales,f:Sphingomonadaceae,g:Sphingomonas;

TACGGAGGGAGCTAGCGTTGTTCGGAATTACTGGGCGTAAAGCGCACGTAGGCGGCTTTG

TAAGTTAGAGGTGAAAGCCTGGAGCTCAACTCCAGAATTGCCTTTAAGACTGCATCGCTT

GAATCCAAGAGAGGTGAGTGGAATTCCGAGTGTAGAGGTGAAATTCGTAGATATTCGGAA

GAACACCAGTGGCGAAGGCGGCTCACTGGCTTGGTATTGACGCTGAGGTGCGAAAGCGTG

GGGAGCAAAC

>ASV555 SS|1.0000|JQ309130_S003619637;k:Bacteria,p:"Acidobacteria",c:Acidobacteria_Gp4

TACGTAGGGACCAAGCGTTGTTCGGATTTACTGGGCGTAAAGGGCGCGTAGGCGGCGTGA

CAAGTCACTTGTGAAATCTCCGAGCTTAACTCGGAACGGTCAAGTGATACTGTTATGCTA

GAGTACAGAAGGGGTAATCGGAATTCTCGGTGTAGCGGTGAAATGCGTAGATATCGAGAG

GAACACCAGAGGCGAAGGCGGATTACTAGGCTGATACTGACGCTGAGGCGCGAAAGCTAG

GGTAGCAAAC

>ASV556 SS|1.0000|AY140238_S000397413;k:Bacteria,p:"Proteobacteria",c:Alphaproteobacteria,o:Rhodospirillales,f:Acetobacteraceae

TACGAAGGGGGCTAGCGTTGCTCGGAATGACTGGGCGTAAAGGGCGCGTAGGCGGATGTC

TTTGTCGGGCGTGAAATTCCAGGGCTCAACCTTGGGACTGCGTTCGAGACGGGATGTCTA

GAGTTTGGCAGAGGGTCGTGGAATTCCCAGTGTAGAGGTGAAATTCGTAGATATTGGGAA

GAACACCGGTGGCGAAGGCGGCGACCTGGTCCTTGACTGACGCTGATGCGCGAAAGCGTG

GGGAGCAAAC

>ASV557 GS|0.0|None;No hit

TTCCAGCTCCAATAGCGTATATTAAAGTTGTTGCAGTTAAAAAGCTCGTAGTTGAACTTT

GGGTCTGGCTGATCTGTCCTCCTAATCGAGCGCACGGATTCGGTCGGACCTTTCCTTCTG

GGGATCCCTATGCCCTTCACTGGGCGTAGTGGGGAACCAGGACTTTTACCTTGAAAAAAT

TAGAGTGTTCAAAGCAGGCCTTTGCTCGAATACATTAGCATGGAATAATAAAATAGGACA

TCGGTTCTAT

>ASV558 GS|0.0|None;No hit

GTGTCAGCAGCCCACACACGCAGAGCGCGCAAACAGTAATGATGAACCATCGTGGCTCGG

TTCCTCCTATACCGCCGCCACCTCAGCAGCCGCAGCATCAGCCATCACCTGAGGTCCAAG

CCAGTGGCGTTGAAGAAGCCCCTCTGTATGTGAACGCCAAACAATTTCACCGAATATTGA

AGCGGCGGGTCGCGCGACAAAAATTGGAAGAAGCCCTCCGCCTTACATCCAAGGGCCGAA

AACCATACCT

>ASV559 SS|0.9900|EU907898_S001151285;k:Bacteria,p:"Armatimonadetes",c:Chthonomonadetes,o:Chthonomonadales,f:Chthonomonadaceae,g:Chthonomonas/Armatimonadetes_gp3

TACGGAGGGGGCGAGCGTTGTCCGAAGTTACTGGGCGTAAAGGGCGCGTAGGCGGGTTGG

CAAGTCCGCTGTGAAAGCCCGGCGCTTAACGCCGGAGGGTCGGTGGATACTGCCAGTCTT

GAAGGTGCTAGGGACAGATGGAATTACCAGTGTAGCGGTGAAATGCGTAGATATTGGTAG

GAACACCAGTGGCGAAGGCGGTCTGTTGGAGCACACTTGACGCTGAGGCGCGAAAGCTGG

GGGAGCGAAC

>ASV560 GS|0.0|None;No hit

GTGTCAGCAGCCGAGAAAGCTTGTAGGGGAAGGAGGGCCAACGCTGTGAGCGTGGGATAG

AGGAGTTTGAGGGCCATGGTAGGGCTAAAGAGCAGCGTAGGGTAATCGAGGAGGCGGGAA

CGAAACCCTTGTAGTCC

>ASV561 GS|100.0|AB272165_S000824049;k:Bacteria,p:"Bacteroidetes",c:Cytophagia,o:Cytophagales,f:Cytophagaceae,g:Persicitalea;

TACGGAGGGTGCGAGCGTTGTCCGGATTTATTGGGCTTAAAGGGTGCGCAGGTGGCCCGC

CAAGCCAGCGGTGAAATCCCCCCGCTCAACGGGGGGCGTGCCGTTGGAACTGGCGGGCTT

GAGTTCGGCCGAGGCCGCCGGAACGGGCGGTGTAGCGGTGAAATGCATAGATATCGCCCA

GAACGCCGATTGCGAAGGCAGGCGGCCAGGCCGACACTGACACTGAGGCACGAAAGCGTG

GGGAGCGAAC

>ASV562 GS|98.8|AM778124_S000941857;k:Bacteria,p:"Actinobacteria",c:Actinobacteria,o:Actinomycetales,f:Nakamurellaceae,g:Nakamurella;

TACGTAGGGTGCAAGCGTTGTCCGGAATTATTGGGCGTAAAGAGCTCGTAGGCGGTCTGT

CGCGTCGAATGTGAAAACACGAGGCTCAACTTCGTGCCTGCATTCGATACGGGCAGACTA

GAGTTCGGTAGGGGAGTCTGGAATTCCTGGTGTAGCGGTGAAATGCGCAGATATCAGGAG

GAACACCGGTGGCGAAGGCGGGACTCTGGGCCGATACTGACGCTGAGGAGCGAAAGCGTG

GGGAGCAAAC

>ASV563 GS|98.4|AB267478_S000721192;k:Bacteria,p:"Bacteroidetes",c:Sphingobacteriia,o:"Sphingobacteriales",f:Chitinophagaceae,g:Segetibacter;

TACGGAGGGTGCAAGCGTTATCCGGATTCACTGGGTTTAAAGGGTGCGTAGGTGGGAATG

TAAGTCAGTGGTGAAATCTCCGTGCTTAACATGGAAACTGCCATTGATACTATGTTTCTT

GAATTTTCTGGAGGTCAGCGGAATATGTCATGTAGCGGTGAAATGCTTAGATATGACATA

GAACACCAATTGCGAAGGCAGCTGGCTACAGGGATATTGACACTGAGGCACGAAAGCGTG

GGGATCAAAC

>ASV564 GS|0.0|None;No hit

GTGTCAGCAGCCGTGAGGCAGTGTTGCCTGGAGCTGCTCACCATCTAGTGCCTTGATGCG

GCCAGGCATTGGTCGATTGCTATCTAGGCAGAACTCTCCAGCCCAGCCGAAGTCAAGCCT

CGACCGTTGCATATATATCCCG

>ASV565 SS|1.0000|JX294485_S003614212;k:Bacteria,p:"Bacteroidetes",c:Cytophagia,o:Cytophagales,f:Cytophagaceae,g:Hymenobacter

TACGGAGGGTGCAAGCGTTGTCCGGATTTATTGGGTTTAAAGGGTGCGTAGGCGGCCGCT

TAAGTCCGGGGTGAAAGCCCGTTGCTCAACAACGGAACTGCCCTGGAAACTGGTCGGCTT

GAGTACAGACGAGGGTGGCGGAATGGACGGTGTAGCGGTGAAATGCATAGATACCGTCCA

GAACCCCGATTGCGAAGGCAGCTGCCTAGACTGTAACTGACGCTGAGGCACGAAAGCGTG

GGGAGCGAAC

>ASV566 GSL|99.2|AF144383_S000388105;k:Bacteria,p:"Proteobacteria",c:Betaproteobacteria,o:Burkholderiales,f:Comamonadaceae

TACGTAGGGTGCAAGCGTTAATCGGAATTACTGGGCGTAAAGCGTGCGCAGGCGGTGATG

TAAGACAGATGTGAAATCCCCGGGCTCAACCTGGGAACTGCATTTGTGACTGCATCGCTC

GAGTGCGGCAGAGGGGGATGGAATTCCGCGTGTAGCAGTGAAATGCGTAGATATGCGGAG

GAACACCGATGGCGAAGGCAATCCCCTGGGCCTGCACTGACGCTCATGCACGAAAGCGTG

GGGAGCAAAC

>ASV567 SS|1.0000|EU861928_S001148233;k:Bacteria,p:"Armatimonadetes",c:Armatimonadia,o:Armatimonadales,f:Armatimonadaceae,g:Armatimonas/Armatimonadetes_gp1

TACGTAGGGGGCGAGCGTTGTTCGAAGTTACTGGGCGTAAAGAGCGCGTAGGCGGGTTCT

TAAGTGAGGGGTGAAATTCCGGGGCTCAACCCCGGAACTGCCTTTTATACTGGGAACCTT

GAGTGTGGGAGAGGCGAGTGGAATGGTCGGTGTAGCGGTGAAATGCGTAGATATCGATCG

GAACACCCATGGCGAAGGCAGCTCGCTGGCCTATAACTGACGCTGAGGCGCGAAAGCGTG

GGGAGCAAAC

>ASV568 SS|0.9900|AF166114_S000498654;k:Bacteria,p:Cyanobacteria/Chloroplast

TACGGAGGATGCAAGCGTTATCCGGAATGATTGGGCGTAAAGCGTCTGTAGGTGGGTTGT

AAAGTCTTCTGTTAAAGATCGGGGCTTAACCCAGTTTAAGCAGTGGAAACTTATAACCTA

GAGTACGGTAGGGGCAGAGGGAATTCCCGGTGTAGCGGTGAAATGCGTAGATATCGGGAA

GAACACCGACGGCGAAAGCACTCTGCTGGGCCGAAACTGACACTGAGAGACGAAAGCATG

GGGATCAAAG

>ASV569 GS|100.0|HF558376_S003722567;k:Bacteria,p:"Proteobacteria",c:Alphaproteobacteria,o:Sphingomonadales,f:Sphingomonadaceae,g:Sphingomonas;

TACGGAGGGGGCTAGCGTTGTTCGGAATTACTGGGCGTAAAGCGCACGTAGGCGGCTTTG

TAAGTCAGAGGTGAAAGCCTGGAGCTCAACTCCAGAACTGCCTTTGAGACTGCATCGCTT

GAATCCAGGAGAGGTCAGTGGAATTCCGAGTGTAGAGGTGAAATTCGTAGATATTCGGAA

GAACACCAGTGGCGAAGGCGGCTGACTGGACTGGTATTGACGCTGAGGTGCGAAAGCGTG

GGGAGCAAAC

>ASV570 GS|100.0|AY364020_S000572063;k:Bacteria,p:"Proteobacteria",c:Alphaproteobacteria,o:Rhizobiales,f:Methylobacteriaceae,g:Methylobacterium;

TACGAAGGGGGCTAGCGTTGCTCGGAATCACTGGGCGTAAAGGGCGCGTAGGCGGCCATT

CAAGTCGGGGGTGAAAGCCTGTGGCTCAACCACAGAATTGCCTTCGATACTGTTTGGCTT

GAGTATGGTAGAGGTCGGTGGAACTGCGAGTGTAGAGGTGAAATTCGTAGATATTCGCAA

GAACACCAGTGGCGAAGGCGGCCGACTGGACCATTACTGACGCTGAGGCGCGAAAGCGTG

GGGAGCAAAC

>ASV571 SS|0.9900|CP001854_S001872674;k:Bacteria,p:"Actinobacteria",c:Actinobacteria,o:Solirubrobacterales

TACGTAGGGGGCAAGCGTTGTCCGGAATCATTGGGCGTAAAGAGCGTGTAGGTGGCTCGG

TAAGTCCGCTGTGAAAGTCCAGGGCTCAACCCTGGAATGCCGGTGGAAACTGTCGGGCTA

GAGTCCGGAAGAGGCGAGTGGAATTCCTGGTGTAGCGGTGAAATGCGCAGATATCAGGAG

GAACACCAATGGCGAAGGCAGCTCGCTGGGACGGTACTGACACTGAGACGCGAAAGCGTG

GGGAGCAAAC

>ASV572 SS|1.0000|JX294485_S003614212;k:Bacteria,p:"Bacteroidetes",c:Cytophagia,o:Cytophagales,f:Cytophagaceae,g:Hymenobacter

TACGGAGGGTGCGAGCGTTGTCCGGATTTATTGGGTTTAAAGGGTGCGTAGGCGGCCAAA

TAAGTCTGGGGTGAAAGCCCGCTGCTCAACAGCGGAACTGCCCTGGATACTGTATGGCTT

GAGGACAGACGAGGTTGGCGGAATGGAGGGTGTAGCGGTGAAATGCATAGATACCCTCCA

GAACCCCGATTGCGAAGGCAGCTGACTAGACTGTAACTGACGCTGAGGCACGAAAGCGTG

GGGAGCGAAC

>ASV573 GS|100.0|X83804_S000010779;k:Bacteria,p:"Actinobacteria",c:Actinobacteria,o:Actinomycetales,f:Cellulomonadaceae,g:Cellulomonas;

TACGTAGGGCGCAAGCGTTGTCCGGAATTATTGGGCGTAAAGAGCTCGTAGGCGGTTTGT

CGCGTCTGCTGTGAAAACCTGAGGCTCAACCTCGGGCTTGCAGTGGGTACGGGCAGACTA

GAGTGCGGTAGGGGTGACTGGAATTCCTGGTGTAGCGGTGGAATGCGCAGATATCAGGAG

GAACACCGATGGCGAAGGCAGGTCACTGGGCCGCAACTGACGCTGAGGAGCGAAAGCATG

GGGAGCGAAC

>ASV574 GS|98.0|AJ563932_S000352294;k:Bacteria,p:"Proteobacteria",c:Alphaproteobacteria,o:Rhizobiales,f:Beijerinckiaceae,g:Beijerinckia;

TACGAAGGGGGCTAGCGTTGTTCGGATTTACTGGGCGTAAAGGGCGCGTAGGCGGATTTT

TAAGTCAGGGGTGAAATCCCAAGGCTCAACCTTGGAACTGCCTTTGATACTGGGGATCTT

GAGTCCGGGAGAGGTGAGTGGAACTGCGAGTGTAGAGGTGAAATTCGTAGATATTCGCAA

GAACACCAGTGGCGAAGGCGGCTCACTGGCCCGGCACTGACGCTGAGGCGCGAAAGCGTG

GGGAGCAAAC

>ASV575 SS|1.0000|HM032898_S002167666;k:Bacteria,p:"Bacteroidetes",c:Cytophagia,o:Cytophagales,f:Cytophagaceae,g:Hymenobacter

TACGGAGGGTGCGAGCGTTGTCCGGATTTATTGGGTTTAAAGGGTGCGTAGGCGGTCGAT

TAAGTCTGGGGTGAAAGCCCGCTGCTCAACAGCGGAACTGCCCTGGATACTGGTTGACTT

GAGTACAGACGAGGTTGGCGGAATGGACTGAGTAGCGGTGAAATGCATAGATACAGTCCA

GAACCCCGATTGCGAAGGCAGCTGACTAGGCTGATACTGACGCTGAGGCACGAAAGCGTG

GGGAGCGAAC

>ASV576 GS|98.0|EU861894_S001148199;k:Bacteria,p:"Armatimonadetes",c:Armatimonadia,o:Armatimonadales,f:Armatimonadaceae,g:Armatimonas/Armatimonadetes_gp1;

TACGTAGGGGGCCAGCGTTGTCCGAAGTTACTGGGCGTAAAGAGCGCGTAGGCGGGCCCT

TAAGTGGGGGGTGAAATCCCGAGGCTCAACCTCGGGACTGCCTCTCATACTGGGGGCCTT

GAGTGCGGGAGAGGCGAGTGGAATGGTCGGTGTAGCGGTGAAATGCGTAGATATCGATCG

GAACACCCATGGCGAAGGCAGCTCGCTGGCCTGCAACTGACGCTGAGGCGCGAAAGCCGG

GGGAGCGAAC

>ASV577 GS|98.8|AB166881_S000386315;k:Bacteria,p:"Proteobacteria",c:Alphaproteobacteria,o:Caulobacterales,f:Caulobacteraceae,g:Phenylobacterium;

TACGAAGGGGGCTAGCGTTGCTCGGAATTACTGGGCGTAAAGGGCGCGTAGGCGGACAGT

TTAGTCAGAGGTGAAAGCCCAGGGCTCAACCTTGGAAATGCCTTTGATACTGGCTGTCTT

GAGTTCGGGAGAGGTGAGTGGAACTCCGAGTGTAGAGGTGAAATTCGTAGATATTCGGAA

GAACACCAGTGGCGAAGGCGACTCACTGGCCCGATACTGACGCTGAGGCGCGAAAGCGTG

GGGAGCAAAC

>ASV578 GS|100.0|AM778124_S000941857;k:Bacteria,p:"Actinobacteria",c:Actinobacteria,o:Actinomycetales,f:Nakamurellaceae,g:Nakamurella;

TACGTAGGGTGCAAGCGTTGTCCGGAATTATTGGGCGTAAAGAGCTCGTAGGCGGTCTGT

CACGTCGAATGTGAAAACCCGAGGCTCAACTTCGGGCCTGCATTCGATACGGGCAGACTA

GAGTTCGGTAGGGGAGTCTGGAATTCCTGGTGTAGCGGTGAAATGCGCAGATATCAGGAG

GAACACCGGTGGCGAAGGCGGGACTCTGGGCCGATACTGACGCTGAGGAGCGAAAGCGTG

GGGAGCAAAC

>ASV579 GS|100.0|AJ439346_S000366402;k:Bacteria,p:"Actinobacteria",c:Actinobacteria,o:Actinomycetales,f:Corynebacteriaceae,g:Corynebacterium;

TACGTAGGGTGCGAGCGTTGTCCGGAATTACTGGGCGTAAAGGGCTCGTAGGTGGTTTGT

CGCGTCGTCTGTGAAATTCTGGGGCTTAACTCCGGGCGTGCAGGCGATACGGGCATAACT

TGAGTGCTGTAGGGGTAACTGGAATTCCTGGTGTAGCGGTGAAATGCGCAGATATCAGGA

GGAACACCGATGGCGAAGGCAGGTTACTGGGCAGTTACTGACGCTGAGGAGCGAAAGCAT

GGGTAGCGAA

>ASV580 GS|100.0|HM583567_S002988298;k:Bacteria,p:"Proteobacteria",c:Betaproteobacteria,o:Burkholderiales,f:Comamonadaceae,g:Polaromonas;

TACGTAGGGTGCGAGCGTTAATCGGAATTACTGGGCGTAAAGCGTGCGCAGGCGGTGATG

TAAGACAGTTGTGAAATCCCCGGGCTCAACCTGGGAATTGCATCTGTGACTGCATCGCTA

GAGTACGGTAGAGGGGGATGGAATTCCGCGTGTAGCAGTGAAATGCGTAGATATGCGGAG

GAACACCGATGGCGAAGGCAATCCCCTGGACCTGTACTGACGCTCATGCACGAAAGCGTG

GGGAGCAAAC

>ASV581 SS|1.0000|D86513_S000011943;k:Bacteria,p:"Proteobacteria",c:Alphaproteobacteria,o:Rhodospirillales,f:Acetobacteraceae

TACGAAGGGGGCTAGCGTTGCTCGGAATGACTGGGCGTAAAGGGCGCGTAGGCGGCTCAG

ATAGTCAGATGTGAAATTCCTGGGCTCAACCTGGGGGCTGCATTTGATACGTCTGGGCTA

GAGTGGGGAAGAGGGTCGTGGAATTCCCAGTGTAGAGGTGAAATTCGTAGATATTGGGAA

GAACACCGGTGGCGAAGGCGGCGACCTGGTCCTTTTACTGACGCTGAGGCGCGAAAGCGT

GGGGAGCAAA

>ASV582 SS|1.0000|AJ009456_S000115949;k:Bacteria

TACGTAGGTGGCAAGCGTTGTCCGGATTTACTGGGCGTAAAGCGCGCGCAGGCGGACTAT

TAAGTAGAAAGTGAAAGGTTGGAGCTCAACTCCAACATTGCTTCCTATACTGGCAGTCTT

GAGTCCCGGAGGGGAGAGCGGAACAATACGTGTAGCGGTGAAATGCGTTGATATGTATTG

GAACACCAATGGCGAAGGCAGCTCTCTGGACGGGAACTGACGCTCAGGCGCGAAAGCCGA

GGTAGCGAAC

>ASV583 SS|1.0000|Y18947_S000088565;k:Bacteria,p:"Proteobacteria",c:Alphaproteobacteria,o:Rhizobiales

TACGAAGGGGGCTAGCGTTGTTCGGAATCACTGGGCGTAAAGGGTGCGTAGGCGGATTCT

TAAGTCAGGGGTGAAATCCCGAGGCTCAACCTCGGAACTGCCTTTGATACTGGGGATCTT

GAGTCCGGAAGAGGTGAGTGGAACTGCGAGTGTAGAGGTGAAATTCGTAGATATTCGCAA

GAACACCGGTGGCGAAGGCGGCTCACTGGTCCGGAACTGACGCTGAGGCACGACAGCGTG

GGGAGCAAAC

>ASV584 SS|0.9000|DQ986200_S000736608;k:Bacteria,p:"Planctomycetes",c:Planctomycetia,o:Planctomycetales,f:Planctomycetaceae,g:Aquisphaera

GACGAACCGTGCGAACGTTATTCGGAATCACTGGGCTTAAAGCGCGTGTAGGCGGGACGG

CACGTCGGTCGTTGAAATCCCCCGGCTTAACCGGGGAAGTGGCGCCGATACGACCGTCCT

GGAGGGACGTAGGGGGGCCTGGAACTTCCGGTGGAGCGGTGAAATGCGTTGAGATCGGAA

GGAACGCCCGTGGCGAAAGCGAGGCCCTGGACGTCTACTGACGCTGAGACGCGAAAGCCA

GGGGAGCGAA

>ASV585 GS|97.6|AJ563932_S000352294;k:Bacteria,p:"Proteobacteria",c:Alphaproteobacteria,o:Rhizobiales,f:Beijerinckiaceae,g:Beijerinckia;

TACGAAGGGGGCTAGCGTTGTTCGGATTTACTGGGCGTAAAGGGTGCGTAGGCGGATTTT

TAAGTCAGGGGTGAAATCCCAAGGCTCAACCTTGGAACTGCCTTTGATACTGGGGATCTT

GAGTCCGGGAGAGGTGAGTGGAACTGCGAGTGTAGAGGTGAAATTCGTAGATATTCGCAA

GAACACCAGTGGCGAAGGCGGCTCACTGGCCCGGAACTGACGCTGAGGCACGAAAGCGTG

GGGAGCAAAC

>ASV586 GS|0.0|None;No hit

TACGGGGGGGGCAAGCGTTATTCGAAATGATTGGGCGTAAAGGGCACGTAGACGGTTTTA

TGAGTTTTCATTTTTAAAAGTACACAAAATGGCTTTTAAAAATTATAATGGAGTGTGTTT

TCTTTTATTGCAATTTTTTCTTTCTTTCCTTTTAAAAGAGAAAAGAAAGTTAAAGATTCC

GATTACACTTGGGAAAAAGGCCAAGGCCTAACCATGGTGTTCCCACTATACTATAAAACT

AGAGTAAGTT

>ASV587 GS|99.6|X93185_S000003791;k:Bacteria,p:"Actinobacteria",c:Actinobacteria,o:Actinomycetales,f:Micromonosporaceae,g:Actinoplanes;

GACGTAGGGCGCGAGCGTTGTCCGGATTTATTGGGCGTAAAGAGCTCGTAGGCGGCTTGT

CGCGTCGACCGTGAAAACTTGGGGCTCAACTCCAAGCCTGCGGTCGATACGGGCAGGCTC

GAGTTCGGTAGGGGAGACTGGAATTCCTGGTGTAGCGGTGAAATGCGCAGATATCAGGAG

GAACACCGGTGGCGAAGGCGGGTCTCTGGGCCGATACTGACGCTGAGGAGCGAAAGCGTG

GGGAGCGAAC

>ASV588 GS|0.0|None;No hit

GACGGTAAAGACTAGTGTTATTCATCTTTAATAGGTTTAAAGGGTACCTAGACGGTGGAT

CTTGCCCCTAAAAGGTACGGATCTTACTAGAGTTTTATGGAAGAGGTTAGATGTAGTATT

AACGGTGGAGAGATGAAATTCTTTGATACTGTTAGGGACTGGTAACGGCGAAGGCGATCC

TCTAAGTAAAAACTGACGTTGAGGGACGAAGGCTTGGGTAGCGAACAGG

>ASV589 GS|99.2|AB272165_S000824049;k:Bacteria,p:"Bacteroidetes",c:Cytophagia,o:Cytophagales,f:Cytophagaceae,g:Persicitalea;

TACGGAGGGTGCGAGCGTTGTCCGGATTTATTGGGCTTAAAGGGTGCGCAGGTGGCCCGC

CAAGCCAGTGGTGAAATCCCCCCGCTCAACGGGGGGCGTGCCATTGGAACTGGCGGGCTT

GAGTTCGGCCGAGGCCGCCGGAACGGGCGGTGTAGCGGTGAAATGCATAGATATCGCCCA

GAACGCCGATTGCGAAGGCAGGCGGCCAGGCCGACACTGACACTGAGGCACGAAAGCGTG

GGGAGCGAAC

>ASV590 SS|0.9600|AF013550_S000336299;k:Bacteria,p:"Acidobacteria",c:Acidobacteria_Gp4

TACGGGGGGGGCAAGCGTTGTTCGGATTTACTGGGCGTAAAGCGTATGTAGGCGGTTAGG

CAAGTTACTTGTGAAATCTCCGAGCTTAACTCGGAACGGCCAAGTAATACTGCCAAACTA

GAGTGCAGAAGGGGCAATTGGAATTCTTGGTGTAGCGGTGAAATGCGTAGATATCAAGAG

GAACACCAGAGGCGAAGGCGAATTGCTGGGCTGACACTGACGCTGAGATACGAAAGCTAG

GGGAGCAAAC

>ASV591 SS|0.9200|EU682685_S001044889;k:Bacteria,p:"Proteobacteria",c:Alphaproteobacteria,o:Sphingomonadales,f:Sphingomonadaceae

TACGGAGGGGGCTAGCGTTGTTCGGAATTACTGGGCGTAAAGCGTACGTAGGCGGCTTTT

TAAGTCAGAGGTGAAATCCCGGTGCTCAACACCGGAACTGCCTTTGAGACTGGATCGCTT

GATCACGGGAGAGGTTAGTGGAATTCCGAGTGTAGAGGTGAAATTCGTAGATATTCGGAA

GAACACCAGTGGCGAAGGCGGCTAACTGGACCGTTGATGACGCTGAGGTACGAAAGCGTG

GGGAGCAAAC

>ASV592 SS|0.9900|JN630810_S002958490;k:Bacteria,p:"Verrucomicrobia",c:Verrucomicrobiae,o:Verrucomicrobiales

TACGAAGGTCCCGAGCGTTATTCGGAATCACTGGGCGTAAAGGGAGCGTAGGCGGTTTGG

TAAGTCAGATGTGAAATCCCGGGGCTCAACCCCGGAACTGCATCCGATACTGCTGAGCTA

GAGGATTGGAGAGGTGAGTGGAATTCTCGGTGTAGCAGTGAAATGCGTAGATATCGAGAG

GAAGACCAGTTGCGAAAGCGACTCACTGGACAATTCCTGACGCTGAGGCTCGAAGGCTAG

GGGAGCAAAA

>ASV593 SS|0.9300|JQ309130_S003619637;k:Bacteria,p:"Acidobacteria",c:Acidobacteria_Gp4,g:Blastocatella

TACGTAGGGACCAAGCGTTGTTCGGATTTACTGGGCGTAAAGGGCGCGTAGGCGGCGTGA

CAAGTCAGTTGTGAAATCTCCGAGCTTAACTCGGAACGGTCAACTGATACTGTTGTGCTA

GAGTACAGAAGGGGCAATCGGAATTCTTGGTGTAGCGGTGAAATGCGTAGATATCAAGAG

GAACACCAGAGGCGAAGGCGGATTGCTAGGCTGATACTGACGCTGAGGCGCGAAAGCTAG

GGTAGCAAAC

>ASV594 SS|0.9000|AM231587_S000650722;k:Bacteria,p:"Proteobacteria",c:Alphaproteobacteria,o:Rhodospirillales,f:Acetobacteraceae,g:Roseomonas

TACGAAGGGGGCTAGCGTTGCTCGGAATTACTGGGCGTAAAGGGCGCGTAGGCGGCACGG

TCAGTCAGGCGTGAAATCCCGGGGCTCAACCCTGGGGCTGCGCTTGATACGGCCGGGCTA

GAGGGTAGGAGAGGGTCGCGGAATTCCCAGTGTAGAGGTGAAATTCGTAGATATTGGGAA

GAACACCGGTGGCGAAGGCGGCGACCTGGCCTATTACTGACGCTGAGGCGCGACAGCGTG

GGGAGCAAAC

>ASV595 SS|1.0000|EF368368_S000806277;k:Bacteria,p:"Proteobacteria",c:Alphaproteobacteria,o:Rhodospirillales,f:Acetobacteraceae

TACGAAGGGGGCTAGCGTTGCTCGGAATGACTGGGCGTAAAGGGCGCGTAGGCGGCTTGG

TTTGTCGGACGTGAAAGTCCTGGGCTCAACCTGGGGATGGCGTTCGGGACGGCTGGGCTT

GAGTGGGGAAGAGGGTCGTGGAATTCCCAGTGTAGAGGTGAAATTCGTAGATATTGGGAA

GAACACCGGTGGCGAAGGCGGCGACCTGGTCCTTGACTGACGCTGAGGCGCGAGAGCGTG

GGGAGCAAAC

>ASV596 GSL|98.8|AB193172_S000539085;k:Bacteria,p:"Actinobacteria",c:Actinobacteria,o:Actinomycetales,f:Intrasporangiaceae

TACGTAGGGTGCGAGCGTTGTCCGGAATTATTGGGCGTAAAGAGCTTGTAGGCGGTTTGT

CGCGTCTGCTGTGAAATTTCGGGGCTCAACCCCGAACTTGCAGTGGGTACGGGCAGACTA

GAGTGTGGTAGGGGAGACTGGAATTCCTGGTGTAGCGGTGAAATGCGCAGATATCAGGAG

GAACACCGATGGCGAAGGCAGGTCTCTGGGCCACTACTGACGCTGAGAAGCGAAAGCATG

GGGAGCGAAC

>ASV597 SS|0.9700|EF507900_S000859439;k:Bacteria,p:"Bacteroidetes",c:Cytophagia,o:Cytophagales,f:Cytophagaceae,g:Spirosoma

TACGGAGGGTGCAAGCGTTGTCCGGATTTATTGGGTTTAAAGGGTGCGCAGGTGGGTCTG

TAAGTCTGATTTGAAAGCCGGCGGCTCAACCGTCGGATGTGGTTGGAAACTGTGGATCTT

GAATGCGGTAGCGGGAGCCGGAATGGGTCATGTAGCGGTGAAATGCATAGATATGACCCG

GAACACCGATTGCGAAGGCAGGCTCCTTGGCCGTGATTGACACTGAGGCACGAAAGCATG

GGTAGCGAAC

>ASV598 SS|1.0000|GQ454806_S002223697;k:Bacteria,p:"Bacteroidetes",c:Cytophagia,o:Cytophagales,f:Cytophagaceae,g:Hymenobacter

TACGGAGGGTGCGAGCGTTGTCCGGATTTATTGGGTTTAAAGGGTGCGTAGGCGGCCGAT

TAAGTCTGGGGTGAAAGCCCACTGCTCAACAGTGGAACTGCCCTGGAAACTGCTCGGCTT

GAGTACAGACGAGGTTGGCGGAATGGGCACTGTAGCGGTGAAATGCATAGATAGTGCCCA

GAACCCCGATTGCGAAGGCAGCTGACTAGGCTGTAACTGACGCTGAGGCACGAAAGCGTG

GGGAGCGAAC

>ASV599 SS|1.0000|KC560021_S003719248;k:Bacteria,p:"Bacteroidetes",c:Sphingobacteriia,o:"Sphingobacteriales",f:Chitinophagaceae

TACGGAGGGTGCAAGCGTTATCCGGATTCACTGGGTTTAAAGGGTGCGTAGGCGGGCAGG

TAAGTCCGTGGTGAAATCTCTGAGCTTAACTCAGAAACTGCCATGGATACTATTTGTCTT

GAATATTGTGGAGGTAAGCGGAATATGTCATGTAGCGGTGAAATGCTTAGATATGACATA

GAACACCAATTGCGAAGGCAGCTTACTACACAGTTATTGACGCTGAGGCACGAAAGCGTG

GGGATCAAAC

>ASV600 GS|99.6|HE599560_S003258029;k:Bacteria,p:"Actinobacteria",c:Actinobacteria,o:Actinomycetales,f:Nakamurellaceae,g:Nakamurella;

TACGTAGGGTGCAAGCGTTGTCCGGAATTATTGGGCGTAAAGAGCTCGTAGGCGGTCTGT

CGCGTCGACTGTGAAAATCCGAGGCTCAACCTCGGACCTGCATTCGATACGGGCAGACTA

GAGTTCGGTAGGGGAGTCTGGAATTCCTGGTGTAGCGGTGAAATGCGCAGATATCAGGAG

GAACACCGGTGGCGAAGGCGGGACTCTGGGCCGATACTGACGCTGAGGAGCGAAAGCGTG

GGGAGCAAAC

>ASV601 GS|99.6|HQ455786_S002306039;k:Bacteria,p:"Bacteroidetes",c:Sphingobacteriia,o:"Sphingobacteriales",f:Sphingobacteriaceae,g:Mucilaginibacter;

TACGGAGGATCCAAGCGTTATCCGGATTTATTGGGTTTAAAGGGTGCGTAGGCGGCCTAT

TAAGTCAGGGGTGAAAGACGGTAGCTTAACTATCGCAGTGCCTTTGATACTGATGGGCTT

GAATACACTTGAGGTAGGCGGAATGTGACAAGTAGCGGTGAAATGCATAGATATGTCACA

GAACACCAATTGCGAAGGCAGCTTACTAAAGTGTGATTGACGCTGAGGCACGAAAGCGTG

GGGATCAAAC

>ASV602 SS|1.0000|AY140238_S000397413;k:Bacteria,p:"Proteobacteria",c:Alphaproteobacteria,o:Rhodospirillales,f:Acetobacteraceae

TACGAAGGGGGCTAGCGTTGCTCGGAATGACTGGGCGTAAAGGGCGCGTAGGCGGATCTA

TCAGTCGGGCGTGAAATTCCTGGGCTTAACCTGGGGGCTGCGTTCGAGACGGTGGGTCTA

GAGTGGGGAAGAGGGTCGTGGAATTCCCAGTGTAGAGGTGAAATTCGTAGATATTGGGAA

GAACACCGGTGGCGAAGGCGGCGACCTGGTCCTTGACTGACGCTGAGGCGCGAAAGCGTG

GGGAGCAAAC

>ASV603 SS|0.9900|AM162406_S000650674;k:Bacteria,p:"Planctomycetes",c:Planctomycetia,o:Planctomycetales,f:Planctomycetaceae,g:Zavarzinella

GACGAACCGTGCGAACGTTGTTCGGAATCATTGGGCTTAAAGGGCGCGTAGGCGGGCCGC

CAAGTCCGAGGTGAAATCCTCCAGCTCAACTGGAGAACTGCCCCGGATACTGGAGGTCTC

GAGGAAGGTAGGGGCATGCGGAACTGTGGGTGGAGCGGTGAAATGCGTTGATATCCACAG

GAACTCCGGTGGCGAAGGCGGCGTGCTGGACCTTTTCTGACGCTGAGGCGCGAAAGCCAG

GGGAGCAAAC

>ASV604 SS|1.0000|D86513_S000011943;k:Bacteria,p:"Proteobacteria",c:Alphaproteobacteria,o:Rhodospirillales,f:Acetobacteraceae

TACGAAGGGGGCTAGCGTTGCTCGGAATGACTGGGCGTAAAGGGCGCGTAGGCGGATTGC

ACAGTCAGATGTGAAATTCCCGGGCTTAACCTGGGGGCTGCATTTGATACGTGTGGTCTA

GAGTGAGGAAGAGGGTCGTGGAATTCCCAGTGTAGAGGTGAAATTCGTAGATATTGGGAA

GAACACCGGTGGCGAAGGCGGCGACCTGGTCCTTTTACTGACGCTGAGGCGCGAAAGCGT

GGGGAGCAAA

>ASV605 GS|0.0|None;No hit

GTGTCAGCAGCCGCATCCAGAACATCGGCGCGCTGAAGGCAAGGAAGTTGTAGCTCAGCT

CGTTGAACTTCCTACGCTGGCCATCGTGTTCTTCGCCCGGGCGGCTAAGGGCAATCATCC

GCGTGATGAGCGAGACAGTCAGCAGCGCATATAGCGTGCTATTAAAGACATTCCAAAACC

CGATATAGTCCCTCCCAACCTTCCAGAACTTGGCAAACTCATCACATATAAACCCAAACG

TCATGATATA

>ASV606 SS|1.0000|AB072735_S000251887;k:Bacteria

TACAGAGGGTGCGAGCGTTGTCCGGAATCACTGGGCGTAAAGGGCGCGTAGGCGGCCCCA

TAACGCGTGCCGTGAAAGCCCGGGGCTCAACCCCGGGTCGGCGGTGCGAACGGGGGGGCT

GGAGCCGATCAGAGGCGGGCGGAATTCCGGGTGTAGCGGTGGAATGCGTAGAGATCCGGA

AGAACACCGGGGGCGAAGGCGGCCCGCTGGGATCGAGCTGACGCTGAGGCGCGACAGCGT

GGGGAGCAAA

>ASV607 GS|98.0|DQ883810_S000728612;k:Bacteria,p:"Proteobacteria",c:Alphaproteobacteria,o:Rhizobiales,f:"Aurantimonadaceae",g:Aureimonas;

TACGAAGGGGGCTAGCGTTGTTCGGAATTACTGGGCGTAAAGCGCACGTAGGCGGATATT

TAAGTCGGGGGTGAAATCCCGGGGCTCAACCCCGGAACTGCCTTCGATACTGGGTATCTT

GAGTTCGGAAGAGGTGAGTGGAATTGCGAGTGTAGAGGTGAAATTCGTAGATATTCGCAG

GAACACCAGTGGCGAAGGCGGCTCACTGGTCCGACACTGACGCTGAGGTGCGAAAGCGTG

GGGAGCAAAC

>ASV608 GS|100.0|EF166075_S000776495;k:Bacteria,p:"Proteobacteria",c:Gammaproteobacteria,o:Xanthomonadales,f:Xanthomonadaceae,g:Rhodanobacter;

TACGAAGGGTGCAAGCGTTAATCGGAATTACTGGGCGTAAAGGGTGCGTAGGCGGTTAGT

TAAGTCTGTTGTGAAATCCCCGGGCTCAACCTGGGAATGGCAATGGATACTGGCTAGCTA

GAGTGTGTCAGAGGATGGTGGAATTTCCGGTGTAGCGGTGAAATGCGTAGAGATCGGAAG

GAACATCAGTGGCGAAGGCGGCCATCTGGGACAACACTGACGCTGAAGCACGAAAGCGTG

GGGAGCAAAC

>ASV609 GS|97.6|AB267478_S000721192;k:Bacteria,p:"Bacteroidetes",c:Sphingobacteriia,o:"Sphingobacteriales",f:Chitinophagaceae,g:Segetibacter;

TACGGAGGGTGCAAGCGTTATCCGGATTCACTGGGTTTAAAGGGTGCGTAGGTGGGAATG

TAAGTCAGTGGTGAAATCTCCGTGCTTAACATGGAAACTGCCATTGATACTATATTTCTT

GAATTTTCTGGAGGTAAGCGGAATATGTCATGTAGCGGTGAAATGCTTAGATATGACATA

GAACACCAATTGCGAAGGCAGCTTACTACAGGAACATTGACACTGAGGCACGAAAGCGTG

GGGATCAAAC

>ASV610 GS|100.0|AM397630_S000728101;k:Bacteria,p:"Proteobacteria",c:Betaproteobacteria,o:Burkholderiales,f:Oxalobacteraceae,g:Undibacterium;

TACGTAGGGTGCAAGCGTTAATCGGAATTACTGGGCGTAAAGCGTGCGCAGGCGGTTTTA

TAAGTCTGATGTGAAATCCCCGGGCTCAACCTGGGAACTGCATTGGAGACTGTAAGGCTA

GAGTGTGTCAGAGGGGGGTAGAATTCCACGTGTAGCAGTGAAATGCGTAGATATGTGGAG

GAATACCGATGGCGAAGGCAGCCCCCTGGGATAACACTGACGCTCATGCACGAAAGCGTG

GGGAGCAAAC

>ASV611 GSL|75.8|JN175332_S002916776;k:Bacteria,p:"Proteobacteria",c:Gammaproteobacteria,o:"Enterobacteriales",f:Enterobacteriaceae

CTTGGTCATTTAGAGGAAGTAAAAGTCGTAACAAGGTTTCCGTAGGTGAACCTGCGGAAG

GATCATTATCGAGAGGGGTCCCCGGACTCCGGGGGCTTCGGCCCCCTACTCTTCACCCTA

TGTCTACACACCTTTGTTGCTTTGGCGGGCCTCGGGTTCGCCCCGTACCGGCCGTGGGCT

TCCATACCCCGGCCGTCCGTGCCCGTCAGAGGCCCATGAACCCTCGTTTATCAGTGTCGT

CCGAGTCCAA

>ASV612 SS|1.0000|AB561883_S002150744;k:Bacteria,p:"Proteobacteria",c:Alphaproteobacteria,o:Rhodospirillales,f:Acetobacteraceae

TACGAAGGGGGCTAGCGTTGCTCGGAATGACTGGGCGTAAAGGGCGCGTAGGCGGCTTGT

ACAGTCAGGCGTGAAATTCCTGGGCTCAACCTGGGGACTGCGTCTGATACGTGCGAGCTT

GAGTAGGGAAGAGGGTCGTGGAATTTCCAGTGTAGAGGTGAAATTCGTAGATATTGGAAA

GAACACCGGTGGCGAAGGCGGCGACCTGGTCCTTTACTGACGCTGAGGCGCGAAAGCGTG

GGGAGCAAAC

>ASV613 GS|0.0|None;No hit

TTCCAGCTCCAATAGCGTATATTAAAGTTGTTGCAGTTAAAAAGCTCGTAGTTGAAACTT

GGGTCTGGCTGTCCGGTCCGCCTCACCGCGTGCACTGGTTCGGCCGGGCCTTTCCTTCTG

GGGAGCCGCATGCCCTTCATTGGGTGTGCCGGGGAACCAGGACTTTTACTTTGAAAAAAT

TAGAGTGTTCAAAGCAGGCCTATGCTCGAATACATTAGCATGGAATAATAGAATAGGACG

CGTGGTTCTA

>ASV614 SS|1.0000|EF635408_S001095322;k:Bacteria,p:"Deinococcus-Thermus",c:Deinococci,o:Deinococcales,f:Deinococcaceae,g:Deinococcus

TACGGAGGGTGCAAGCGTTACCCGGAATCACTGGGCGTAAAGGGCGTGTAGGCGGTTTGC

CAAGTCTGACTTTAAAGACCGAAGCTCAACTTCGGGCATGGGCCAGAGACTGGCAGACTA

GACGGATGGAGAGGTCACTGGAATTCCTGGTGTAGCGGTGGAATGCGTAGATACCAGGAG

GAACACCAACGGCGAAGGCAGGTGACTGGACATTTAGTGACGCTGAGGCGCGAAAGTGTG

GGGAGCAAAC

>ASV615 GS|0.0|None;No hit

GTGTCAGCAGCCATCTACCTCTCCTTCGAGATAGCTTTGGCACACAGACTAAGTGAGAAT

GGACCAAAGCTTGCTTAAAATGATACACTCTCTGAGCAAGTATGATGGTTCTAAGATGTA

GTCGAACCCAGGATCGAAAGGTCTCGGTACCAGTGGCATGGCCGTTCTTAGTTGGTGGAG

TGATTTGTCTGCTTAATTGCGATAACGAACGAGACCTTAACCTGCTAAATAGCCAGGTCA

GCTTCGGCTG

>ASV616 SS|1.0000|JQ309130_S003619637;k:Bacteria,p:"Acidobacteria",c:Acidobacteria_Gp4

TACGTAGGGACCAAGCGTTGTTCGGATTTACTGGGCGTAAAGGGCGCGTAGGCGGCGTGA

CAAGTCACTTGTGAAATCTCCGAGCTTAACTCGGAACGGTCAAGTGATACTGTTGTGCTA

GAGTATAGAAGGGGCAATCGGAATTCTCGGTGTAGCGGTGAAATGCGTAGATATCGAGAG

GAACACCAGAGGCGAAGGCGGATTGCTAGGCTAATACTGACGCTGAGGCGCGAAAGCTAG

GGTAGCAAAC

>ASV617 SS|1.0000|D86513_S000011943;k:Bacteria,p:"Proteobacteria",c:Alphaproteobacteria,o:Rhodospirillales,f:Acetobacteraceae

TACGAAGGGGGCTAGCGTTGCTCGGAATGACTGGGCGTAAAGGGCGCGTAGGCGGTTTGC

ATAGTCAGATGTGAAATTCCTGGGCTTAACCTGGGGGCTGCATTTGATACGTGCGGGCTT

GAGTGGGGAAGAGGGTCGTGGAATTCCCAGTGTAGAGGTGAAATTCGTAGATATTGGGAA

GAACACCGGTGGCGAAGGCGGCGACCTGGTCCTTTACTGACGCTGAGGCGCGAAAGCGTG

GGGAGCAAAC

>ASV618 GS|0.0|None;No hit

TACGGGGGGGGCGAGCGTTATTCGAAATGATTGGGCGTAAAGGGCACGTAGACGGTTTTT

TGAGTTGACATGCTTGAGGTCGTACAAAGAATACAAAATAAATATTCTTTCGTACGTCTA

AAAGATTAAAATGGAGTGTGGATTTTCACCCTCTATTCTTTGCTTGATAAAAGAAAAGGT

ATTCGGTTTGCTTTACACTTGGGAAAAAGGCCAAGGCTCAACCATGGTGTTTCCCGCTAT

ACTATAAAAC

>ASV619 GS|98.8|AB428568_S001168624;k:Bacteria,p:"Proteobacteria",c:Alphaproteobacteria,o:Sphingomonadales,f:Sphingomonadaceae,g:Sphingomonas;

TACGGAGGGAGCTAGCGTTATTCGGAATTACTGGGCGTAAAGCGCACGTAGGCGGCTTTG

TAAGTAAGAGGTGAAAGCCCAGAGCTCAACTCTGGAATTGCCTTTTAGACTGCATCGCTT

GAATCCAGGAGAGGTGAGTGGAATTCCGAGTGTAGAGGTGAAATTCGTAGATATTCGGAA

GAACACCAGTGGCGAAGGCGGCTCACTGGACTGGTATTGACGCTGAGGTGCGAAAGCGTG

GGGAGCAAAC

>ASV620 SS|1.0000|KF999686_S004084195;k:Bacteria,p:"Bacteroidetes",c:Cytophagia,o:Cytophagales,f:Cytophagaceae,g:Spirosoma

TACGGAGGGTGCGAGCGTTGTCCGGATTTATTGGGTTTAAAGGGTGCGTAGGTGGGTTAC

TAAGTCTGGTTTGAAAGCAGGTGGCTCAACCATCTGATGTGGCTGGAAACTGGGGATCTT

GAATGGGTTGGCGGCTGCCGGAACGGGTCATGTAGCGGTGAAATGCATAGATATGACCCA

GAACACCGATTGCGAAGGCAGGCAGCTACGACCTGATTGACACTGAGGCACGAGAGCCGG

GGTAGCGAAC

>ASV621 SS|0.9900|AB251884_S000650634;k:Bacteria,p:"Bacteroidetes",c:Cytophagia,o:Cytophagales,f:Cytophagaceae,g:Hymenobacter

TACGGAGGGTGCAAGCGTTGTCCGGATTTATTGGGTTTAAAGGGTGCGCAGGCGGCCGAT

TAAGTCCGGGGTGAAAGCCCGTTGCTCAACAACGGAACCGCCCCGGAAACTGGTGGGCTT

GAGTACAGACGAGGTTGGCGGAATGGACGGTGTAGCGGTGAAATGCATAGATACCGTCCA

GAACCCCGATTGCGAAGGCAGCTGACTAGGCTGTGACTGACGCTGAGGCACGAAAGCGTG

GGGAGCGAAC

>ASV622 SS|1.0000|AY140238_S000397413;k:Bacteria,p:"Proteobacteria",c:Alphaproteobacteria,o:Rhodospirillales,f:Acetobacteraceae

TACGAAGGGGGCTAGCGTTGCTCGGAATGACTGGGCGTAAAGGGCGCGTAGGCGGATCTG

GCAGTCGGGCGTGAAATTCCTGGGCTTAACCTGGGGGCTGCGTTCGAGACGTTGGGTCTA

GAGTTTGGAAGAGGGTTGTGGAATTCCCAGTGTAGAGGTGAAATTCGTAGATATTGGGAA

GAACACCGGTGGCGAAGGCGGCGACCTGGTCCTTGACTGACGCTGAGGCGCGAAAGCGTG

GGGAGCAAAC

>ASV623 GSL|99.2|AM501439_S000964802;k:Bacteria,p:"Proteobacteria",c:Betaproteobacteria

TACGTAGGGTGCAAGCGTTAATCGGAATTACTGGGCGTAAAGCGTGCGCAGGCGGTTATG

TAAGACAGAGGTGAAATCCCCGGGCTCAACCTGGGAACTGCCTTTGTGACTGCATAGCTT

GAGTGCGGCAGAGGGGGATGGAATTCCGCGTGTAGCAGTGAAATGCGTAGATATGCGGAG

GAACACCGATGGCGAAGGCAATCCCCTGGGCCTGCACTGACGCTCATGCACGAAAGCGTG

GGGAGCAAAC

>ASV624 SS|0.9800|CP001854_S001872674;k:Bacteria,p:"Actinobacteria",c:Actinobacteria,o:Solirubrobacterales

TACGTAGGGGGCAAGCGTTGTCCGGAATCATTGGGCGTAAAGAGCGTGTAGGCGGCCCGA

TAAGTCCGCTGTGAAAGTCCAGGGCTCAACCCTGGAATGCCGGTGGATACTGTCGGGCTC

GAGTCCGGAAGAGGTGAGTGGAATTCCTGGTGTAGCGGTGAAATGCGCAGATATCAGGAG

GAACACCAATGGCGAAGGCAGCTCACTGGGACGGTACTGACGCTGAGACGCGAAAGCGTG

GGGAGCAAAC

>ASV625 GS|0.0|None;No hit

TACGTAAAAGACTAGTGTTACTCATCTTGAATAGGTTTAAAGGGTACCTAGACAGCCAAT

CAAGCCCCAAAAGGGTACTAATGGGCTAGAGTTTAATATAAGTAGGCTTTAAAGTACTGA

GAATGTTGAGAGATGAAATTCTGTGATACTATATACGGCACTGGTAAAGGCGAAAGCATC

TTATTATGTAAAAACTGACGTTGAGGGACGAAGGCTGTGTGTAGCAAAAAGG

>ASV626 SS|1.0000|HM032898_S002167666;k:Bacteria,p:"Bacteroidetes",c:Cytophagia,o:Cytophagales,f:Cytophagaceae,g:Hymenobacter

TACGGAGGGTGCGAGCGTTGTCCGGATTTATTGGGTTTAAAGGGTGCGTAGGCGGCTTTT

TAAGTCTGGGGTGAAAGCCCGCTGCTCAACAGCGGAACTGCCCTGGATACTGGAGAGCTT

GAGTACAGACGAGGTTGGCGGAATGGACTGAGTAGCGGTGAAATGCATAGATACAGTCCA

GAACCCCGATTGCGAAGGCAGCTGACTAGGTTGTTACTGACGCTGAGGCACGAAAGCGTG

GGGAGCGAAC

>ASV627 GSL|79.2|EU046270_S000903110;k:Bacteria

TACGTAGGTGGCAAGCGTTGTCCGGATTTACTGGGCGTAAAGGGCAAGCAGGCGGACTGT

TAAGTAGGAAGTGAAAGGTTGGAGCTCAACTCCAACATTGCTCCCTATACTGGCAGTCTT

GAGTCTCGGAGAGGAAAGCGGAACGATACGTGTAGCGGTGAAATGCGTTGATATGTATCG

GAACACCAATGGCGAAGGCAGCTTTCTGGACGAGAACTGACGCTCAGTTGCGAAAGCCGA

GGTAGCGAAC

>ASV628 SS|1.0000|EF457480_S000834905;k:Bacteria

TACGTGGGGTCCAAGCGTTGTCCGGAATTACTGGGCGTAAAGGGCGTGCAGGTGGCTACT

CAAGCTTGGCGTGAAAGCCCCCGGCCCAACCGGGGAGGGTCGTCGAGGACTGGGTGGCTT

GAGGGGCGCAGGGGTCGGTGGAATTCCGGGTGTAGTGGTGAAATGCGTAGAGATCCGGAG

GAACACCAGTGGCGAAGGCGGCCGACTGGGCGCACCCTGACACTGAGACGCGAAGGCGTG

GGGAGCGAAC

>ASV629 SS|1.0000|D86513_S000011943;k:Bacteria,p:"Proteobacteria",c:Alphaproteobacteria,o:Rhodospirillales,f:Acetobacteraceae

TACGAAGGGGGCTAGCGTTGCTCGGAATGACTGGGCGTAAAGGGCGCGTAGGCGGATGGC

ACAGTCAGATGTGAAATTCCCGGGCTTAACCTGGGGGCTGCATTTGATACGTGTTGTCTA

GAGTGAGGAAGAGGGTCGTGGAATTCCCAGTGTAGAGGTGAAATTCGTAGATATTGGGAA

GAACACCGGTGGCGAAGGCGGCGACCTGGTCCTTTACTGACGCTGAGGCGCGAAAGCGTG

GGGAGCAAAC

>ASV630 SS|0.9400|EF516412_S000840854;k:Bacteria,p:"Armatimonadetes",c:Armatimonadia,o:Armatimonadales,f:Armatimonadaceae,g:Armatimonas/Armatimonadetes_gp1

TACGTAGGGGGCGAGCGTTGTCCGAAGTTACTGGGCGTAAAGGGCGCGTAGGCGGGTCCT

TAAGTGTGGGGTGAAAGGCTGAGGCTCAACCTCAGCACTGCCCTGCAAACTGGGGGTCTT

GAGTGTCGGAGGGGCGAGTGGAATGGTCGGTGTAGCGGTGAAATGCGTAGATATCGATCG

GAACACCCATGGCGAAGGCAGCTCGCTGGCCGACAACTGACGCTGAGGCGCGAAAGCGTG

GGGAGCAAAC

>ASV631 SS|0.9800|AM231587_S000650722;k:Bacteria,p:"Proteobacteria",c:Alphaproteobacteria,o:Rhodospirillales,f:Acetobacteraceae

TACGAAGGGGGCTAGCGTTGCTCGGAATGACTGGGCGTAAAGGGCGCGTAGGCGGCTTGG

TTAGTTAGGCGTGAAATTCCTGGGCTCAACCTGGGGGCTGCGTTTAATACAGCTAGGCTA

GAGTGGGGAAGAGGGTTGTGGAATTCCCAGTGTAGAGGTGAAATTCGTAGATATTGGGAA

GAACACCAGTGGCGAAGGCGGCAACCTGGTCCTTGACTGACGCTGAGGCGCGAAAGCGTG

GGGAGCAAAC

>ASV632 SS|0.9700|AM947653_S001093907;k:Bacteria,p:"Proteobacteria",c:Alphaproteobacteria,o:Rhodospirillales,f:Acetobacteraceae,g:Acidisoma

TACGAAGGGGGCTAGCGTTGCTCGGAATGACTGGGCGTAAAGGGCGCGTAGGCGGACATA

TTAGTCAGGCGTGAAATTCCTGGGCTCAACCTGGGGGCTGCGTTTGATACGGTATGTCTA

GAGTTTGGAAGAGGGTCGTGGAATTCCCAGTGTAGAGGTGAAATTCGTAGATATTGGGAA

GAACACCGGTGGCGAAGGCGGCGACCTGGTCCTTGACTGACGCTGAGGCGCGAAAGCGTG

GGGAGCAAAC

>ASV633 GSL|100.0|AJ243326_S000433018;k:Bacteria,p:Firmicutes,c:Bacilli,o:Lactobacillales,f:Enterococcaceae,g:Vagococcus

TACGTAGGTGGCAAGCGTTGTCCGGATTTATTGGGCGTAAAGCGAGCGCAGGCGGTTCTT

TAAGTCTGATGTGAAAGCCCCCGGCTCAACCGGGGAGGGTCATTGGAAACTGGAGAACTT

GAGTGCAGAAGAGGAGAGTGGAATTCCATGTGTAGCGGTGAAATGCGTAGATATATGGAG

GAACACCAGTGGCGAAGGCGACTCTCTGGTCTGTAACTGACGCTGAGGCTCGAAAGCGTG

GGGAGCAAAC

>ASV634 SS|1.0000|DQ022076_S000537030;k:Bacteria,p:"Deinococcus-Thermus",c:Deinococci,o:Deinococcales,f:Trueperaceae,g:Truepera

TACGGAGGGTGCAAGCGTTATCCGGAATCACTGGGCGTAAAGGGCGCGTAGGCGGTTTGT

TAAGTCCGATGTTAAAGACCGAGGCTCAACCTCGACACGGCGTTGGATACTGACAAGCTG

GACGGTTGGAGAGGAAGGTAGAATTACCAGTGTAGCGGTGGAATGCGTAGATACTGGTAG

GAATACCCATTGCGAAGGCAGCCTTCTGGACAACACGTGACGCTGAGGCGCGAAAGTGTG

GGGAGCAAAC

>ASV635 SS|1.0000|D30775_S000413730;k:Bacteria,p:"Proteobacteria",c:Alphaproteobacteria,o:Rhodospirillales,f:Acetobacteraceae

TACGAAGGGGGCTAGCGTTGCTCGGAATGACTGGGCGTAAAGGGCGCGTAGGCGGATGGC

ATAGTCAGGCGTGAAATTCCTGGGCTCAACCTGGGGGCTGCGTTTGATACGTGTTGTCTA

GAGTGGGGAAGAGGGTTGTGGAATTCCCAGTGTAGAGGTGAAATTCGTAGATATTGGGAA

GAACACCGGTGGCGAAGGCGGCAACCTGGTCCTTGACTGACGCTGAGGCGCGAAAGCGTG

GGGAGCAAAC

>ASV636 GS|98.8|AY043938_S000354314;k:Bacteria,p:"Verrucomicrobia",c:Spartobacteria,g:Spartobacteria_genera_incertae_sedis;

TACAGAGGTCTCAAGCGTTGTTCGGATTCATTGGGCGTAAAGGGTGCGTAGGTGGCGGGG

TAAGTCGGGTGTGAAATTTCGGAGCTTAACTCCGAAACTGCATTCGATACTGCCTTGCTT

GAGGACTGGAGAGGAGACTGGAATTTACGGTGTAGCAGTGAAATGCGTAGATATCGTAAG

GAAGACCAGTGGCGAAGGCGGGTCTCTGGACAGTTCCTGACACTGAGGCACGAAGGCTAG

GGGAGCAAAC

>ASV637 SS|1.0000|EU135017_S000933611;k:Bacteria

TACGTAGGGTCCGAGCGTTGTCCGGAATTACTGGGCGTAAAGCGTGCGCAGGCGGCCCGG

AAGGTGTCACGTGAAAGCCCCGGGCTCAACCCGGGAGGGTCGTGGCAAACCGGCGGGCTT

GAGGCCGGTAGAGGTGGGTGGAACTCCTGGTGGAGTGGTGAAATGCGTAGAGATCAGGAA

GAACACCCGTGGCGAAGGCGGCCCACTGGGCCGGTACTGACGCTGAGGCACGAAGGCGTG

GGGAGCGAAC

>ASV638 SS|0.9200|X64380_S000749517;k:Bacteria,p:"Verrucomicrobia",c:Spartobacteria,g:Spartobacteria_genera_incertae_sedis

TACAGAGGCCTCAAGCGTTGTTCGGATTCATTGGGCGTAAAGGGAGCGTAGGCGGTCGGG

TAAGTCGGGTGTGAAATCCTGGGGCTCAACCTCAGAACTGCATTCGATACTGCTTGGCTA

GAGGACTGGAGAGGTGAGTGGAATTCACGGTGTAGCAGTGAAATGCGTAGATATCGTGAG

GAAGACCAGCGGCGAAGGCGGCTCACTGGACAGTATCTGACGCTGATGCTCGAAGGCCAG

GGGAGCAAAC

>ASV639 SS|1.0000|CP001854_S001872674;k:Bacteria,p:"Actinobacteria",c:Actinobacteria,o:Solirubrobacterales

TACGTAGGGGGCAAGCGTTGTCCGGAATCATTGGGCGTAAAGAGCGTGTAGGCGGCTCGG

TAAGTCCGCTGTGAAAGTCCAGGGCTCAACCCTGGAATGCCGGTGGAAACTGTCGAGCTA

GAGTCCGGAAGAGGCGAGTGGAATTCCTGGTGTAGCGGTGAAATGCGCAGATATCAGGAG

GAACACCAATGGCGAAGGCAGCTCGCTGGGACGGTACTGACGCTGAGACGCGAAAGCGTG

GGGAGCAAAC

>ASV640 SS|0.9600|AJ519386_S000102346;k:Bacteria,p:"Acidobacteria",c:Acidobacteria_Gp3,g:Gp3

TACGTAGGCAGCGAGCGTTGTTCGGAATTACTGGGCGTAAAGAGTGTGTAGGCGGTGCTC

TAAGTTCGGTGTGAAATCTCCTGGCTTAACTGGGAGGGTGCGCCGGAAACTGGAGTGCTC

GAACGTGGGAGAGGAAAGCGGAATTCCTGGTGTAGCGGTGAAATGCGTAGATATCAGGAG

GAACACCTGCGGTGTAGACGGCTTTCTGGACCATTGTTGACGCTGAGACACGAAAGCGTG

GGTAGCAAAC

>ASV641 GS|97.2|KF999686_S004084195;k:Bacteria,p:"Bacteroidetes",c:Cytophagia,o:Cytophagales,f:Cytophagaceae,g:Spirosoma;

TACGGAGGGTGCGAGCGTTGTCCGGATTTATTGGGTTTAAAGGGTGCGTAGGTGGGTAAC

TAAGTCTGGTTTGAAAGCAGGTGGCTCAACCATCTGATGTGGCTGGAAACTGGTGATCTT

GAATGGGTTGGCGGTAGCCGGAACGGGTCATGTAGCGGTGAAATGCATAGATATGACCCA

GAACACCGATTGCGAAGGCAGGCTACTAGGACTTGATTGACACTGAGGCACGAGAGCATG

GGTAGCGAAC

>ASV642 GS|0.0|None;No hit

GTGTCAGCAGCCATAAGCAAGTTACCAGACCAACAGTGCGACTCAGTGTTGAGCTTGTAA

TGGGAAGCTCGACGTTGAATTAGACAACTGAACATCCCTGACTCGACTAAAAGAAGACTT

TGACCACGCCCCGCGTAGTCC

>ASV643 GS|98.8|FJ948169_S001418440;k:Bacteria,p:"Proteobacteria",c:Alphaproteobacteria,o:Sphingomonadales,f:Sphingomonadaceae,g:Hephaestia;

TACGGAGGGAGCTAGCGTTGTTCGGAATTACTGGGCGTAAAGCGCACGTAGGCGGCTTTG

TAAGTTAGAGGTGAAAGCCCGGGGCTCAACCCCGGAATTGCCTTTAAGACTGCATCGCTT

GAATCCAGGAGAGGTGAGTGGAATTCCGAGTGTAGAGGTGAAATTCGTAGATATTCGGAA

GAACACCAGTGGCGAAGGCGGCTCACTGGACTGGTATTGACGCTGAGGTGCGAAAGCGTG

GGGAGCAAAC

>ASV644 GS|99.6|EF507900_S000859439;k:Bacteria,p:"Bacteroidetes",c:Cytophagia,o:Cytophagales,f:Cytophagaceae,g:Spirosoma;

TACGGAGGGTGCAAGCGTTGTCCGGATTTATTGGGTTTAAAGGGTGCGTAGGTGGGTATT

TAAGTCTGGTTTGAAAGCAGGTGGCTCAACCATCTGATGTGGCTGGAAACTGGATATCTT

GAATGGGTTGGCGGTAGCCGGAATGGGTCATGTAGCGGTGAAATGCATAGATATGACCCG

GAACACCGATTGCGAAGGCAGGCTACTACGACTTGATTGACACTGAGGCACGAGAGCATG

GGTAGCGAAC

>ASV645 SS|0.8300|D86511_S000414138;k:Bacteria,p:"Proteobacteria",c:Alphaproteobacteria,o:Rhodospirillales,f:Acetobacteraceae,g:Acidiphilium

TACGAAGGGGGCTAGCGTTGCTCGGAATGACTGGGCGTAAAGGGCGCGTAGGCGGCATGT

TTCGTCAGGCGTGAAATTCCTGGGCTCAACCTGGGGACTGCGCTTGATACAGGCAGGCTA

GAGTGTGAAAGAGGGTCGTGGAATTTCCAGTGTAGAGGTGAAATTCGTAGATATTGGAAA

GAACACCGGTGGCGAAGGCGGCGACCTGGTTCACAACTGACGCTGAGGCGCGAAAGCGTG

GGGAGCAAAC

>ASV646 SS|1.0000|EF457481_S000834906;k:Bacteria,p:"Acidobacteria",c:Acidobacteria_Gp4

TACGTAGGGACCAAGCGTTGTTCGGATTTACTGGGCGTAAAGGGCGCGTAGGCGGCGTGT

CAAGTCAGTTGTGAAATCTCCGAGCTTAACTCGGAACGGTCAACTGATACTGATGTGCTA

GAGTACAGAAGGGGCAATCGGAATTCTTGGTGTAGCGGTGAAATGCGTAGATATCAAGAG

GAACACCAGAGGCGAAGGCGGATTGCTAGGCTGATACTGACGCTGAGGCGCGAAAGCTAG

GGTAGCAAAC

>ASV647 SS|0.9900|AB847449_S004125734;k:Bacteria,p:"Proteobacteria",c:Deltaproteobacteria,o:Myxococcales

GACAGAGGGTGCAAACGTTGTTCGGAATTACTGGGCGTAAAGCGTGTGTAGGCGGTGAGG

TAAGTCGGATGTGAAAGCCCAGGGCTCAACCCTGGAAGTGCACTCGATACTGCTTCGCTT

GAGTACTGGAGAGGTCGGTGGAATTCTCGGTGTAGAGGTGAAATTCGTAGATATCGAGAG

GAACATCTGTGGCGAAGGCGGCCGACTGGACAGATACTGACGCTGAGACACGAAAGTGCG

GGGAGCAAAC

>ASV648 GS|100.0|GQ352404_S001577790;k:Bacteria,p:"Actinobacteria",c:Actinobacteria,o:Actinomycetales,f:Micrococcaceae,g:Kocuria;

TACGTAGGGCGCAAGCGTTGTCCGGAATTATTGGGCGTAAAGAGCTCGTAGGCGGTTTGT

CGCGTCTGCTGTGAAAGCCCGGGGCTTAACCCCGGGTGTGCAGTGGGTACGGGCAGACTT

GAGTGCAGTAGGGGAGACTGGAATTCCTGGTGTAGCGGTGAAATGCGCAGATATCAGGAG

GAACACCGATGGCGAAGGCAGGTCTCTGGGCTGTTACTGACGCTGAGGAGCGAAAGCATG

GGGAGCGAAC

>ASV649 SS|0.8200|FM998003_S002355582;k:Bacteria,p:"Actinobacteria",c:Actinobacteria,o:Actinomycetales,g:Motilibacter

TACGTAGGGTGCAAGCGTTGTCCGGAATTATTGGGCGTAAAGAGCTCGTAGGTGGCTTGT

CACGTCGGGTGTGAAATCCCGGGGCTTAACCCCGGGTCTGCATTCGATACGGGCTGGCTA

GGGTCCTTCAGGGGAGACTGGAATTCCTGGTGTAGCGGTGAAATGCGCAGATATCAGGAG

GAACACCGGTGGCGAAGGCGGGTCTCTGGGAAGGTACCGACGCTGAGGAGCGAAAGCGTG

GGGAGCGAAC

>ASV650 GSL|99.6|AB245358_S000627894;k:Bacteria,p:"Proteobacteria",c:Betaproteobacteria,o:Burkholderiales,f:Comamonadaceae

TACGTAGGGTGCAAGCGTTAATCGGAATTACTGGGCGTAAAGCGTGCGCAGGCGGTTATG

TAAGACAGTTGTGAAATCCCCGGGCTCAACCTGGGAATTGCATCTGTGACTGCATAGCTA

GAGTACGGTAGAGGGGGATGGAATTCCGCGTGTAGCAGTGAAATGCGTAGATATGCGGAG

GAACACCGATGGCGAAGGCAATCCCCTGGACCTGTACTGACGCTCATGCACGAAAGCGTG

GGGAGCAAAC

>ASV651 GS|0.0|None;No hit

GTGTCAGCAGCCATCGGATGACACTAGATCATCAAGCGGCGATGGCCAACCTCGAATAGA

GAGGCGAAGTTCTGGCGGCGGGATCGCCACACCACCCGTAACGTCACGGCCGAATTCTAG

CAACGCGATTCCCAGGGCTGGTTGACGACTATCCTCTGCCTCCGCATCTTGGCTCGGTGG

GCGACCATGGAACATGGGTGTTGTTGTATGGCATTTGTACACAGGTGGCGCCAGTCGCTG

CTTCTTGGAT

>ASV652 GS|100.0|AJ132943_S000128260;k:Bacteria,p:"Actinobacteria",c:Actinobacteria,o:Actinomycetales,f:Propionibacteriaceae,g:Friedmanniella;

TACGTAGGGTCCGAGCGTTGTCCGGAATTATTGGGCGTAAAGGGCTTGTAGGCGGTCCGT

CGCGTCAGAAGTGAAAACTCAGGGCTTAACCCTGAGCCTGCTTTTGATACGGGCGGACTA

GAGGGATGCAGGGGAGAACGGAATTCCTGGTGGAGCGGTGGAATGCGCAGATATCAGGAG

GAACACCGGTGGCGAAGGCGGTTCTCTGGGCATTACCTGACGCTGAGAAGCGAAAGCGTG

GGGAGCAAAC

>ASV653 SS|1.0000|AY140238_S000397413;k:Bacteria,p:"Proteobacteria",c:Alphaproteobacteria,o:Rhodospirillales,f:Acetobacteraceae

TACGAAGGGGGCTAGCGTTGCTCGGAATGACTGGGCGTAAAGGGCGCGTAGGCGGATAGG

TTTGTCGGGCGTGAAATTCCTGGGCTCAACCTGGGGGCTGCGTTCGAGACGGCTTGTCTT

GAGTGGGGAAGAGGGTCGTGGAATTCCCAGTGTAGAGGTGAAATTCGTAGATATTGGGAA

GAACACCGGTGGCGAAGGCGGCGACCTGGTCCTTGACTGACGCTGAGGCGCGAAAGCGTG

GGGAGCAAAC

>ASV654 SS|0.9900|AY788950_S000610642;k:Bacteria,p:"Proteobacteria",c:Alphaproteobacteria,o:Rhodospirillales,f:Acetobacteraceae

TACGAAGGGGGCTAGCGTTGCTCGGAATGACTGGGCGTAAAGGGCGCGTAGGCGGTTCGG

ACAGTCAGATGTGAAATTCCTGGGCTCAACCTGGGGACTGCATTTGATACGTCCGGGCTT

GAGTGTGGAAGAGGGTTGTGGAATTCCCAGTGTAGAGGTGAAATTCGTAGATATTGGGAA

GAACACCGGTGGCGAAGGCGGCAACCTGGTCCATTACTGACGCTGAGGCGCGAAAGCGTG

GGGAGCAAAC

>ASV655 SS|1.0000|L24105_S000414532;k:Bacteria

TACGTAGGGTCCAAGCGTTATCCGGAATCACTGGGCGTAAAGCGTCTGTAGGCGGCTTGT

TAAGTGTGATGTTAAATCGTGTGGCTCAACCATATCGATGCATCGCATACTGGCAGGCTA

GAGGATGGCAGAGGCAAGTGGAACTAGTAGTGTAGCAGTGAAATGCGTAGAGATTACTAA

GAACACCAATGGCGAAGGCAGCTTGCTGGGCCATATCTGACGCTGAGAGACGAAAGCGTG

GGGAGCGAAC

>ASV656 SS|1.0000|FN391026_S001418752;k:Bacteria,p:"Planctomycetes",c:Planctomycetia,o:Planctomycetales,f:Planctomycetaceae

GACGAACCGTGCGAACGTTATTCGGAATCACTGGGCTTAAAGCGCGTGTAGGCGGGCCGC

CACGTCGAGTGCTGAAATCCCCCGGCTCAACCGGGGAACGGGCATCGATACGAGCGGCCT

GGAGGGAGGTAGGGGGGGCTGGAACTTCCGGTGGAGCGGTGAAATGCGTTGAGATCGGAA

GGAACGCCCGTGGCGAAAGCGAGCCCCTGGACCTTTTCTGACGCTGAGACGCGAAAGCTA

GGGGAGCGAA

>ASV657 SS|1.0000|AM231587_S000650722;k:Bacteria,p:"Proteobacteria",c:Alphaproteobacteria,o:Rhodospirillales,f:Acetobacteraceae

TACGAAGGGGGCTAGCGTTGCTCGGAATGACTGGGCGTAAAGGGCGCGTAGGCGGATTGG

TTAGTCAGACGTGAAATTCCTGGGCTCAACCTGGGGGCTGCGTTTGAAACGGCTAATCTA

GAGTTTGGAAGAGGGTTGTGGAATTCCCAGTGTAGAGGTGAAATTCGTAGATATTGGGAA

GAACACCGGTGGCGAAGGCGGCAACCTGGTCCTTGACTGACGCTGAGGCGCGAAAGCGTG

GGGAGCAAAC

>ASV658 SS|1.0000|EF457349_S000834774;k:Bacteria

TACGTAGGGGGCGAGCGTTGTCCGGATTTATTGGGCGTAAAGGGCGCGTAGGCGGCCTGG

CGCATCCGGCGTGAAATCTCCCCGCTCAACGGGGAGGGGTCGGCGGAGATGGCCAGGCTC

GAGGGCGGTAGAGGCAGATGGAAGTCCCGAAGTAGTGGTGAAATGCGTAGAGATCGGGAG

GAACACCAGAGGCGAAGGCGATCTGCTGGGCCGACCCTGACGCTGAGGCGCGACAGCCAG

GGGAGCGAAC

>ASV659 SS|1.0000|AF385568_S000344685;k:Bacteria,p:Candidatus_Saccharibacteria,g:Saccharibacteria_genera_incertae_sedis

TACGTAGGACCCAAGCGTTATCCGGAATTACTGGGCGTAAAGAGTTGCGTAGGCGGTCTG

TTAAGCGAATAGTGAAATCATGTGGCTCAACCATATTGGCTATTATTCGAACTGGCAGAC

TAGAGAATGGTATGGGTAACTGGAATTTCTAGTGTAGGAGTGAAATCCGTAGATATTAGA

AGGAACACCAATGGCGTAGGCAGGTTACTGGACCATTTCTGACGCTCAGGCACGAAAGCG

TGGGGAGCGA

>ASV660 GS|99.6|EF457477_S000834902;k:Bacteria,p:"Acidobacteria",c:Acidobacteria_Gp6,g:Gp6;

TACAGAGGGGGCAAGCGTTGTTCGGAATTACTGGGCGTAAAGGGCGCGTAGGCGGCCTAC

TAAGTCAGACGTGAAATCCCTCGGCTCAACCGGGGAACTGCGTCTGATACTGGTGGGCTT

GAGTTCGGGAGAGGAATGTGGAATTCCAGGTGTAGCGGTGAAATGCGTAGATATCTGGAG

GAACACCGGTGGCGAAGGCGGCATTCTGGACCGACACTGACGCTGAGGCGCGAAAGCCAG

GGGAGCAAAC

>ASV661 GS|0.0|None;No hit

TACGTAGAAGACTAGTGTTATTCATCTTTAATAGGTTTAAAGGGTACCTAGACGGTAAAT

CAAGCCCATATGGGGACTACTTTACTAGAGTTACTTATGAGGGGGTATTAAAGTACTGCT

GGTGTAGAGATGAAATTCTGTCATACCTCTTTTCGTAGGAAAAATGATGGCACAGGTATA

GGCGAAAGCATCTCCTTATGTGATAACTGACGTTGAAGGACGAAGGCTTTGTGTCGCGAA

CAGG

>ASV662 GS|0.0|None;No hit

GTGTCAGCAGCCGCTTCTGAATTGCCACAGGTCTTTCGCCAGGTCCGAGCTGCCTTTGCA

AATCATGCTTGCGCATGATGATTTGATAATTATCTAATTCGTCCTGCAAGAAGGACTTCT

TGCCAAGTATCTGTAAGATCTCCGCCACAGTGGTGTTGACACCGGTAGAAACTGTGGCGA

AAGTCGAATCGATCCTGAAGATCCGAACACAATGCGGAGTCCCGTCGTCTTCCACCTTCT

GAGGAACGCC

>ASV663 SS|1.0000|JQ309130_S003619637;k:Bacteria,p:"Acidobacteria",c:Acidobacteria_Gp4

TACGTAGGGACCAAGCGTTGTTCGGATTTACTGGGCGTAAAGGGCGCGTAGGCGGCGTGA

CAAGTCACTTGTGAAATCTCCGGGCTTAACTCGGAACGGTCAAGTGATACTGTTGTGCTA

GAGTACAGAAGGGGCAATCGGAATTCTTGGTGTAGCGGTGAAATGCGTAGATATCAAGAG

GAACACCAGAGGCGAAGGCGGATTGCTAGGCTGATACTGACGCTGAGGCGCGAAAGCTAG

GGTAGCAAAC

>ASV664 SS|1.0000|KC560021_S003719248;k:Bacteria,p:"Bacteroidetes",c:Sphingobacteriia,o:"Sphingobacteriales",f:Chitinophagaceae

TACGGAGGGTGCAAGCGTTATCCGGATTCACTGGGTTTAAAGGGTGCGTAGGCGGGCAGG

TAAGTCCGTGGTGAAATCTCTGAGCTTAACTCAGAAACTGCCATGGATACTATTTGTCTT

GAATACTGTGGAGGTAAGCGGAATATGTCATGTAGCGGTGAAATGCTTAGATATGACATG

GAACACCAATTGCGAAGGCAGCTTACTACACAGTTATTGACGCTGAGGCACGAAAGCGTG

GGGATCAAAC

>ASV665 GS|0.0|None;No hit

GACGGTAAAGACTAGTGTTATTCATCTTTAATAGGTTTAAAGGGTACCTAGACGGTGGAT

CTTGCCCCTAAAAGGTACGGATCTTACTAGAGTTTTATGGAAGAGGTTAGATTTAGTATT

AACGGTGGAGAGATGAAATTCTTTGATACTGTTAGGCACTGGTAACGGCGAAGGCGATCC

TCTAAGTAAAAACTGACGTTGAGGGACGAAGGCTTGGGTAGCGAACAGG

>ASV666 SS|1.0000|JF999998_S002914924;k:Bacteria,p:"Bacteroidetes",c:Sphingobacteriia,o:"Sphingobacteriales",f:Sphingobacteriaceae

TACGGAGGATCCAAGCGTTATCCGGATTTATTGGGTTTAAAGGGTGCGTAGGCGGCCTGT

TAAGTCAGGGGTGAAAGACGGTGGCTCAACCATCGCAGTGCCTTTGATACTGACGGGCTT

GAATGCAGCTGAGGTAGGCGGAATGTGACAAGTAGCGGTGAAATGCATAGATATGTCACA

GAACACCAATTGCGAAGGCAGCTTACCAAAGTGTGATTGACGCTGAGGCACGAAAGCGTG

GGGATCAAAC

>ASV667 SS|1.0000|DQ291145_S000625839;k:Bacteria,p:"Actinobacteria",c:Actinobacteria,o:Actinomycetales

TACGTAGGGTGCAAGCGTTGTCCGGAATTATTGGGCGTAAAGAGCTCGTAGGCGGTTTGT

CGCGTCGGCTGTGAAATCTTGGGGCTCAACCCCAAGCCTGCAGTCGATACGGGCAGACTT

GAGTGCGGTAGGGGAGACTGGAATTCCTGGTGTAGCGGTGAAATGCGCAGATATCAGGAG

GAACACCGGTGGCGAAGGCGGGTCTCTGGGCCGTAACTGACGCTGAGGAGCGAAAGCGTG

GGGAGCGAAC

>ASV668 GS|97.6|AB267478_S000721192;k:Bacteria,p:"Bacteroidetes",c:Sphingobacteriia,o:"Sphingobacteriales",f:Chitinophagaceae,g:Segetibacter;

TACGGAGGGTGCAAGCGTTATCCGGATTTACTGGGTTTAAAGGGTGCGTAGGTGGGAATG

TAAGTCAGTGGTGAAATCTCCGTGCTTAACATGGAAACTGCCATTGATACTATGTTTCTT

GAATTTTCTGGAGGTTAGCGGAATATGTCATGTAGCGGTGAAATGCTTAGATATGACATA

GAACACCAATTGCGAAGGCAGCTGACTACAGGAAAATTGACACTGATGCACGAAAGCGTG

GGGATCAAAC

>ASV669 SS|0.9400|EU004198_S001350584;k:Bacteria,p:"Bacteroidetes",c:Cytophagia,o:Cytophagales,f:Cytophagaceae,g:Rhodocytophaga

TACGGAGGGTGCAAGCGTTGTCCGGATTTATTGGGTTTAAAGGGTGCGTAGGCGGCCTGT

TAAGTCAGAGGTGAAAGACTATTGCTCAACAATGGCAGTGCCTTTGATACTGATAGGCTT

GAGTATAGATGAGGCTGGCGGAATGGGTGGTGTAGCGGTGAAATGCATAGATACCATCCA

GAACACCGATTGCGAAGGCAGCCGGCTAAACTACAACTGACGCTGAGGCACGAAAGCGTG

GGGATCGAAC

>ASV670 GS|98.4|HQ687087_S002989302;k:Bacteria,p:"Acidobacteria",c:Acidobacteria_Gp1,g:Granulicella;

TACGAGGGGGGCAAGCGTTGTTCGGAATTATTGGGCGTAAAGGGTGCGTAGGCGGTTTGA

CAAGTCTTATGTGAAATCTTCGGGCTCAACCCGAAGTCTGCATGGGAAACTGTCGGGCTT

GAGTATGGGAGAGGTGAGTGGAATTTCCGGTGTAGCGGTGAAATGCGTAGATATCGGAAG

GAACACCTGTGGCGAAAGCGGCTCACTGGACCATAACTGACGCTGATGCACGAAAGCTAG

GGGAGCAAAC

>ASV671 GS|75.9|X99562_S000381430;k:Archaea,p:"Crenarchaeota",c:Thermoprotei,o:Desulfurococcales,f:Desulfurococcaceae,g:Ignicoccus;

CTTGGTCATTTAGAGGAAGTAAAAGTCGTAACAAGGTTTCCGTAGGTGAACCTGCGGAAG

GATCATTACCGAGAGCGGGGCTAACCCCCCAAACTCCGCCGCCGAAAGGGGTACTCTCCA

CCCTATGTATACATATACCACTCTCGCTTTGGCGGGCTGAAGGCTCTTGCCCTACGCCCG

CCAGTGGCTCAAAAAATTCTGTTCATCAGTGATGTCCGAGTAAAAACCTAATAGTTAAAA

CTTTCAACAA

>ASV672 SS|1.0000|DQ291145_S000625839;k:Bacteria,p:"Actinobacteria",c:Actinobacteria,o:Actinomycetales

TACGTAGGGTGCAAGCGTTGTCCGGAATTATTGGGCGTAAAGAGCTCGTAGGCGGTTTGT

CGCGTCGGCTGTGAAAACTTGGGGCTCAACCCCAAGCCTGCAGTCGATACGGGCAGACTA

GAGTGCGGTAGGGGAGACTGGAATTCCTGGTGTAGCGGTGAAATGCGCAGATATCAGGAG

GAACACCGGTGGCGAAGGCGGGTCTCTGGGCCGTAACTGACGCTGAGGAGCGAAAGCGTG

GGGAGCGAAC

>ASV673 GS|100.0|HQ839787_S002910138;k:Bacteria,p:"Actinobacteria",c:Actinobacteria,o:Actinomycetales,f:Propionibacteriaceae,g:Friedmanniella;

TACGTAGGGTCCGAGCGTTGTCCGGAATTATTGGGCGTAAAGGGCTTGTAGGCGGTCCGT

CGCGTCAGGAGTGAAAACTCGGGGCTTAACCCCGAGCCTGCTTTTGATACGGGCGGACTA

GAGGGATGCAGGGGAGAACGGAATTCCTGGTGGAGCGGTGGAATGCGCAGATATCAGGAG

GAACACCGGTGGCGAAGGCGGTTCTCTGGGCATCACCTGACGCTGAGAAGCGAAAGCGTG

GGGAGCAAAC

>ASV674 SS|1.0000|D86513_S000011943;k:Bacteria,p:"Proteobacteria",c:Alphaproteobacteria,o:Rhodospirillales,f:Acetobacteraceae

TACGAAGGGGGCTAGCGTTGCTCGGAATGACTGGGCGTAAAGGGCGCGTAGGCGGATCGG

ACAGTCAGACGTGAAATTCCCGGGCTCAACCTGGGGGCTGCGTTTGAGACGTTTGGTCTA

GAGTTTGGAAGAGGGTCGTGGAATTCCCAGTGTAGAGGTGAAATTCGTAGATATTGGGAA

GAACACCGGTGGCGAAGGCGGCGACCTGGTCCTTGACTGACGCTGAGGCGCGAAAGCGTG

GGGAGCAAAC

>ASV675 GS|98.0|DQ232612_S000942113;k:Bacteria,p:"Actinobacteria",c:Actinobacteria,o:Actinomycetales,f:Microbacteriaceae,g:Leifsonia;

TACGTAGGGTCCGAGCGTTGTCCGGAATTATTGGGCGTAAAGAGCTCGTAGGCGGTTTGT

CGCGTCTGCTGTGAAAACCCGAGGCTCAACCTCGGGCCTGCAGTGGGTACGGGCAGACTA

GAGTGCGGTAGGGGAGAATGGAACTCCTGGTGTAGCGGTGGAATGCGCAGATATCAGGAA

GAACACCGATGGCGAAGGCAGTTCTCTGGGCCGTAACTGACGCTGAGGAGCGAAAGCATG

GGGAGCGAAC

>ASV676 SS|1.0000|AM231587_S000650722;k:Bacteria,p:"Proteobacteria",c:Alphaproteobacteria,o:Rhodospirillales,f:Acetobacteraceae

TACGAAGGGGGCTAGCGTTGCTCGGAATGACTGGGCGTAAAGGGCGCGTAGGCGGCTTGG

TTAGTCAGACGTGAAATTCCTGGGCTCAACCTGGGGGCTGCGTTTGATACAGCTAGGCTA

GAGTGGGGAAGAGGGTTGTGGAATTCCCAGTGTAGAGGTGAAATTCGTAGATATTGGGAA

GAACACCGGTGGCGAAGGCGGCAACCTGGTCCTTGACTGACGCTGAGGCGCGAAAGCGTG

GGGAGCAAAC

>ASV677 GS|100.0|AF288300_S000390729;k:Bacteria,p:"Proteobacteria",c:Alphaproteobacteria,o:Rhizobiales,f:Bradyrhizobiaceae,g:Bosea;

TACGAAGGGGGCTAGCGTTGCTCGGAATCACTGGGCGTAAAGGGCGCGTAGGCGGACTCT

TAAGTCGGGGGTGAAAGCCCAGGGCTCAACCCTGGAATTGCCTTCGATACTGAGAGTCTT

GAGTTCGGAAGAGGTTGGTGGAACTGCGAGTGTAGAGGTGAAATTCGTAGATATTCGCAA

GAACACCAGTGGCGAAGGCGGCCAACTGGTCCGATACTGACGCTGAGGCGCGAAAGCGTG

GGGAGCAAAC

>ASV678 SS|0.9200|EU682685_S001044889;k:Bacteria,p:"Proteobacteria",c:Alphaproteobacteria,o:Sphingomonadales,f:Sphingomonadaceae

TACGGAGGGGGCTAGCGTTGTTCGGAATTACTGGGCGTAAAGCGCACGTAGGCGGCTTCC

TAAGTCAGAGGTGAAAGCCCGGAGCTCAACTCCGGAATAGCCTTTGAGACTGGGTCGCTT

GAATCCAGGAGAGGTGAGTGGAATTCCGAGTGTAGAGGTGAAATTCGTAGATATTCGGAA

GAACACCAGTGGCGAAGGCGGCTCACTGGACTGGTATTGACGCTGAGGTGCGAAAGCGTG

GGGAGCAAAC

>ASV679 SS|1.0000|FJ817379_S001351635;k:Bacteria,p:"Actinobacteria",c:Actinobacteria,o:Actinomycetales

TACGTAGGGTGCGAGCGTTGTCCGGAATTATTGGGCGTAAAGAGCTTGTAGGTGGTCCTT

CACGTCTGCTGTGAAATCTCAGGGCTCAACCCTGAGCTTGCAGTGGATACGGGAGGACTC

GAGTTCGGCTGGGGAGACTGGAACTCCTGGTGTAGCGGTGGAATGCGCAGATATCAGGAA

GAACACCGGTGGCGAAGGCGGGTCTCTGAACCGATACTGACACTGAGAAGCGAAAGCGTG

GGGAGCAAAC

>ASV680 SS|1.0000|FN391026_S001418752;k:Bacteria,p:"Planctomycetes",c:Planctomycetia,o:Planctomycetales,f:Planctomycetaceae

GACGAACCGTGCGAACGTTATTCGGAATCACTGGGCTTAAAGCGCGTGTAGGCGGGCCGC

CGCGTCGGCTGCTGAAATCCCCCGGCTCAACCGGGGAACGGGCACCGATACGGGCGGCCT

CGAGGGGGGTAGGGGGGTCTGGAACTTCCGGTGGAGCGGTGAAATGCGTTGAGATCGGAA

GGAACGCCCGTGGCGAAAGCGAGACCCTGGACCCTTTCTGACGCTGAGACGCGAAAGCCA

GGGGAGCGAA

>ASV681 GS|99.2|AJ504424_S000251907;k:Bacteria,p:"Actinobacteria",c:Actinobacteria,o:Actinomycetales,f:Corynebacteriaceae,g:Corynebacterium;

TACGTAGGGTGCAAGCGTTGTCCGGAATTACTGGGCGTAAAGAGCTCGTAGGTGGTTTGT

CGCGTCGTCTGTGAAAGCCCGGGGCTTAACTCCGGGTCTGCAGGCGATACGGGCATAACT

AGAGTGCTGTAGGGGAGACTGGAATTCCTGGTGTAGCGGTGAAATGCGCAGATATCAGGA

GGAACACCGATGGCGAAGGCAGGTCTCTGGGCAGTAACTGACGCTGAGGAGCGAAAGCAT

GGGGAGCGAA

>ASV682 GS|98.0|HQ687087_S002989302;k:Bacteria,p:"Acidobacteria",c:Acidobacteria_Gp1,g:Granulicella;

TACGAGGGGGGCAAGCGTTGTTCGGAATTATTGGGCGTAAAGGGTGCGTAGGCGGTTTGA

CAAGTCTTATGTGAAATCTTCGGGCTCAACCCGAAGCCTGCATGGGAAACTGTCGGGCTT

GAGTATGGGAGAGGTGAGTGGAATTTCCGGTGTAGCGGTGAAATGCGTAGATATCGGAAG

GAACACCTGTGGCGAAAGCGGCTCACTGGACCATAACTGACGCTGATGCACGAAAGCTAG

GGGAGCAAAC

>ASV683 GS|0.0|None;No hit

GTGTCAGCAGCCGCAAGGAAACTTTGGGTTTCAGACGTATCGCGGTCCCCCAATCCTAAC

GGCTCGTTCAATTGGGGATCCTCGTTGGTAACCGTCGAAATGCCAGAGAAGTAGGAGTCC

GAGCGCTCCCTGGGTGGCCTGACCAAAGACGCAATTGGGTCTTGAATGAACCTTTCAACC

TCTGGTTGAGTAGGAGTCGTAGGGCTGCTAGATATTGCGCTTGCACTACCATCGGTGTCG

TATCTGAAGC

>ASV684 SS|1.0000|EU861876_S001148181;k:Bacteria,p:"Armatimonadetes",c:Armatimonadia,o:Armatimonadales,f:Armatimonadaceae,g:Armatimonas/Armatimonadetes_gp1

TACGTAGGGGGCCAGCGTTGTCCGAAGTTACTGGGCGTAAAGAGCGCGTAGGCGGACCCT

TAAGTGAGGGGTGAAAGGTTCAGGGCTTAACCCGGACACTGCCTTTCATACTGGGGGTCT

TGAGTGTTGGAGAGGCGAGTGGAATGGTCGGTGTAGCGGTGAAATGCGTAGATATCGATC

GGAACACCCATGGCGAAGGCAGCTCGCTGGCCAACAACTGACGCTGAGGCGCGAAAGCGT

GGGGAGCAAA

>ASV685 SS|1.0000|EF635408_S001095322;k:Bacteria,p:"Deinococcus-Thermus",c:Deinococci,o:Deinococcales,f:Deinococcaceae,g:Deinococcus

TACGGAGGGTGCAAGCGTTACCCGGAATCACTGGGCGTAAAGGGCGTGTAGGCGGTTTGC

CAAGTCTGACTTTAAAGACCGAAGCTCAACTTCGGGAATGGGCCAGAGACTGGCAGACTA

GACGGATGGAGAGGTCACTGGAATTCCTGGTGTAGCGGTGGAATGCGTAGATACCAGGAG

GAACACCAACGGCGAAGGCAGGTGACTGGACATTTAGTGACGCTGAGGCGCGAAAGTGTG

GGGAGCAAAC

>ASV686 GS|0.0|None;No hit

GTGTCAGCAGCCGCAGGACAGCAGAATCATCTTTCGAGAATTTCAAAGAAAGGCAAAGGC

AAAAACAGAGATGAAGGTATCTAAGGTATCCGTACTTCCGGTTTGTCCTTGAGGTACCTT

ATGTTTCGGGGGAAGGGGAAGCAGTTGTGATTGTTGAGCCTCTGACCTTTCACCGGTGAC

CTCTCCTTCCTCCCTGCCCTTTCTTTCCCCTTCACACTCTGTTCTCACCCTCGTCATTTC

CCCTCAAATT

>ASV687 GS|98.8|EU861928_S001148233;k:Bacteria,p:"Armatimonadetes",c:Armatimonadia,o:Armatimonadales,f:Armatimonadaceae,g:Armatimonas/Armatimonadetes_gp1;

TACGTAGGGGGCGAGCGTTGTCCGAAGTTACTGGGCGTAAAGAGCGCGTAGGCGGGTTTT

TAAGTGAGGGGTGAAAGTCCGAGGCTCAACCTCGGAACTGCCTTTCATACTGGAGACCTT

GAGTGCGGGAGAGGCGAGTGGAATGGTTGGTGTAGCGGTGAAATGCGTAGATATCAATCG

GAACACCCATGGCGAAGGCAGCTCGCTGGCCTGTAACTGACGCTGAGGCGCGAAAGCGTG

GGGAGCAAAC

>ASV688 GS|0.0|None;No hit

GTGTCAGCAGCCCAGAGAATTGATTCAAATCCACTAGTCCACCATCAGACTGCATGAATT

CACACCTCGCTCCTTTGGCACCTTCTTCCCTCTTGACGCTCTCTGTGCCCAAACCACCCT

CGAAGCCAGCCTGAAACCCGGCGATATACTTCTTGATGGCGGGAGTCAGGTAGGCATCCG

CGCAGGCAGAGGTAGCTCGAGGAACCAGCTTGATCATCGGCATGAGCTCATGACTCAGAC

TGACATGCGT

>ASV689 GS|0.0|None;No hit

GTGTCAGCAGCCCACTAAGGTATCAAAGGTACCGGGATGCTAAGGAAGTGGACATAACAT

CGACATTATTTACTTTGCGAAAAGTGAGAAGAGTAAAAGTTGACTACGCTAAGGGAAAGA

CTTACATAAGTCACAATTGTTTGACTTTGATGCAAAAAGGGCAATTGTGCCGGGAAGTAG

TCTGATGGGTTAGCAAGCCCTGAAGAACTTCCTGACAAGCAGCTTGATCTGAATTCCTAC

TCGTCGTAGA

>ASV690 SS|1.0000|EF457488_S000834913;k:Bacteria

TACGTAGGAGGCGAGCGTTGTCCGGAATTACTGGGCGTAAAGAGCGCGTAGGCGGCACTG

TACGACATGTGTGAAAGCCCCTGGCTCAACTAGGGAGGGTCACGTGTAACGGCAGAGCTT

TTGAGTGTGGGAGAGGGAAGTGGAACTCCGGGAGTAGCGGTGAAATGCGTAGATACCCGG

AGGAACACCAGTGGCGAAGGCGGCTTCCTGGCCTACAACTGACGCTGAGGCGCGAAAGCT

AGGGGAGCGA

>ASV691 SS|0.9600|EF368368_S000806277;k:Bacteria,p:"Proteobacteria",c:Alphaproteobacteria,o:Rhodospirillales,f:Acetobacteraceae,g:Roseomonas

TACGAAGGGGGCTAGCGTTGCTCGGAATTACTGGGCGTAAAGGGCGCGTAGGCGGCTTGG

TTAGTCAGGCGTGAAATCCTGGGGCTCAACCCTGGGGCTGCGCTTGATACGGCTGGGCTT

GAGGGTAGGAGAGGGTCGCGGAATTCCCAGTGTAGAGGTGAAATTCGTAGATATTGGGAA

GAACACCGGTGGCGAAGGCGGCGACCTGGCCTATTACTGACGCTGAGGCGCGACAGCGTG

GGGAGCAAAC

>ASV692 SS|1.0000|DQ792500_S000713035;k:Bacteria,p:"Actinobacteria",c:Actinobacteria,o:Actinomycetales

TACGTAGGGTGCAAGCGTTGTCCGGAATTATTGGGCGTAAAGAGCTCGTAGGCGGTCTGT

CGCGTCGGCTGTGAAACCCGGAGGCTCAACCTCCGGCCTGCAGTCGATACGGGCAGACTA

GAGTTCGGTAGGGGAGACTGGAATTCCTGGTGTAGCGGTGAAATGCGCAGATATCAGGAG

GAACACCGGTGGCGAAGGCGGGTCTCTGGGCCGATACTGACGCTGAGGAGCGAAAGCGTG

GGGAGCAAAC

>ASV693 GS|0.0|None;No hit

TACGTAGAAGACAAGTGTTATTCATCTTTAATAGGTTTAAAGGGTACTTAGACGGTAAAT

AAAGCCTCCAAAAGGTACTAGTTTGCTAGAGTTTTATGTGAAGGAGTTTTAAAGTACTAC

TGGTGTAGAGATGAAATTCAGTTATACCTTACATGGCACAGGTTAAGGCGAAAGCATCTT

CTTATGTAAAAACTGACGTTGAAGGACGAAGGCTTTGTGTCTCGAACAGG

>ASV694 SS|1.0000|FN600641_S001794056;k:Bacteria,p:"Actinobacteria",c:Actinobacteria,o:Actinomycetales

TACGTAGGGTGCAAGCGTTGTCCGGAATTATTGGGCGTAAAGAGCTCGTAGGCGGTCTGT

CGCGTCGGCTGTGAAAACCTGAGGCTCAACCTCGGGCCTGCAGTCGATACGGGCAGACTA

GAGTTCGGTAGGGGAGACTGGAATTCCTGGTGTAGCGGTGGAATGCGCAGATATCAGGAG

GAACACCGGTGGCGAAGGCGGGTCTCTGGGCCGAAACTGACGCTGAGGAGCGAAAGCGTG

GGTAGCAAAC

>ASV695 GS|0.0|None;No hit

TACGGGGGGGGCAAGCGTTATTCGAAATGATTGGGCGTAAAGAGCACGTAGACGGTTTTT

ATAGTAGACATAGTGGCTATATCTCTGTATTAAACATCTTTCACATTTCAAACATCTACA

TCTTTGACATTTCTCATAAATGTTTAGGATGTTAGGATGTCAGAGAAATGTGAAAGATGT

TTACTTCTATATATTTGCATACTTTTGATGGAGTATCTCTTTGTTCATGTATAAACATGA

ACAAATACTC

>ASV696 GS|99.6|AY218772_S000356842;k:Bacteria,p:"Verrucomicrobia",c:Spartobacteria,g:Spartobacteria_genera_incertae_sedis;

TACAGAGGTCCCAAGCGTTGTTCGGATTCATTGGGCGTAAAGGGTGCGTAGGTGGCGCCG

TAAGTGGGGTGTGAAATTTCGGAGCTTAACTCCGAAACTGCATTCCATACTGCGGTGCTT

GAGGACTGGAGAGGAGACTGGAATTCATGGTGTAGCAGTGAAATGCGTAGAGATCATGAG

GAAGACCAGTGGCGAAGGCGGGTCTCTGGACAGTTCCTGACACTGAGGCACGAAGGCTAG

GGGAGCGAAC

>ASV697 GS|0.0|None;No hit

CACGTAGAAGACAAGTGTTATTCATCTTTATTTGGTATATAGGGTACCTAGACGGATTAC

CAAGCCTTAACAAAGGGACTAGTTTTCTAGAGTTTTATGTGGGAATACACGAATTACCAG

AGGAGAGACAAAATGTTATTATACTGGTAGGACGGGTCACGGCGAAGGCTTTATTCTAAG

TAAAAACTGACGTTGAGGGACGAAGCCTAGGGGAGCGATAAGG

>ASV698 SS|1.0000|AY140238_S000397413;k:Bacteria,p:"Proteobacteria",c:Alphaproteobacteria,o:Rhodospirillales,f:Acetobacteraceae

TACGAAGGGGGCTAGCGTTGCTCGGAATGACTGGGCGTAAAGGGCGCGTAGGCGGATTTG

TCAGTCGGGCGTGAAATTCCCGGGCTTAACCTGGGGGCTGCGTTCGAGACGGCGGGTCTT

GAGTTTGGAAGAGGGTCGTGGAATTCCCAGTGTAGAGGTGAAATTCGTAGATATTGGGAA

GAACACCGGTGGCGAAGGCGGCGACCTGGTCCTGGACTGACGCTGAGGCGCGAAAGCGTG

GGGAGCAAAC

>ASV699 GS|0.0|None;No hit

GTGTCAGCAGCCGAAAGCGATGCGGTCACATTCATTTGCCGCCGCTGCGAGCCCAGTTGT

GACTACGGATACAGCAAATATTGACTCAGTGAACACCGAGACTAAGTATAGCGAGCAGAA

GGGCGCCGTCAGCTCCATGATGGCGATCACTAATCGCTCGCAACCTGCGTCTCCC

>ASV700 SS|0.8800|KJ528316_S004225669;k:Bacteria,p:"Proteobacteria",c:Alphaproteobacteria,o:Sphingomonadales,f:Sphingomonadaceae

TACGGAGGGGGCTAGCGTTGTTCGGAATTACTGGGCGTAAAGCGCACGTAGGCGGCTATC

CAAGTCAGGGGTGAAAGCCCGGAGCTCAACTCCGGAACTGCCTCTGAAACTAGGTAGCTT

GAATCATGGAGAGGCGAGTGGAATTCCGAGTGTAGAGGTGAAATTCGTAGATATTCGGAA

GAACACCAGTGGCGAAGGCGGCTCGCTGGACATGTATTGACGCTGAGGTGCGAAAGCGTG

GGGAGCAAAC

>ASV701 GS|0.0|None;No hit

GTGTCAGCAGCCGCCGTGGAGCGAACTCAAGACGAAAGCCGGCAAAGAGCGTAAACGTCT

TCCTCTGGCTTGTATAGCCTGTCGACGGAAAAAGATCCGTTGTTCCGGCGAGAAACCCGC

GTAGTCC

>ASV702 SS|1.0000|FR773158_S002442234;k:Archaea,p:"Thaumarchaeota",o:Nitrososphaerales,f:Nitrososphaeraceae,g:Nitrososphaera

TACCAGCACCCCGAGTGGTCGGGACGTTTATTGGGCCTAAAGCATCCGTAGCCGGTTCTA

CAAGTCTTCCGTTAAATCCACCTGCTTAACAGATGGGCTGCGGAAGATACTATAGAGCTA

GGAGGCGGGAGAGGCAAGCGGTACTCGATGGGTAGGGGTAAAATCCGTTGATCCATTGAA

GACCACCAGTGGCGAAGGCGGCTTGCCAGAACGCGCTCGACGGTGAGGGATGAAAGCTGG

GGGAGCAAAC

>ASV703 GS|0.0|None;No hit

GTGTCAGCAGCCGTGAGGCAGTGTTGCCTGGAGCTGCTCACCATCTAGTGCCTTGATGCG

GCCAGGCATTGGTCGATTGCTATCTAGGCAGAACTCTCCAGCCCAGCCGAAGTCAAGCCT

CGACGATTGCATATATATCCCG

>ASV704 SS|1.0000|D86512_S000010801;k:Bacteria,p:"Proteobacteria",c:Alphaproteobacteria,o:Rhodospirillales,f:Acetobacteraceae

TACGAAGGGGGCTAGCGTTGCTCGGAATGACTGGGCGTAAAGGGCGCGTAGGCGGATGGC

TTAGTCAGGCGTGAAATTCCCGGGCTTAACCTGGGGGCTGCGTTTGATACGGGTTGTCTA

GAGTTTGGCAGAGGGTCGTGGAATTCCCAGTGTAGAGGTGAAATTCGTAGATATTGGGAA

GAACACCGGTGGCGAAGGCGGCGACCTGGTCCTTGACTGACGCTGAGGCGCGAAAGCGTG

GGGAGCAAAC

>ASV705 GS|99.6|JX275860_S003303907;k:Bacteria,p:"Proteobacteria",c:Alphaproteobacteria,o:Rhodospirillales,f:Acetobacteraceae,g:Roseomonas;

TACGAAGGGGGCTAGCGTTACTCGGAATTACTGGGCGTAAAGGGCGCGTAGGCGGCGCTC

CAAGTTAGGCGTGAAAGTCCTGGGCTCAACCTGGGAACTGCGCTTAAGACTGGAGTGCTT

GAGGATGGAAGAGGGTCGTGGAATTCCCAGTGTAGAGGTGAAATTCGTAGATATTGGGAA

GAACACCGGTGGCGAAGGCGGCGACCTGGTCCATTACTGACGCTGAGGCGCGACAGCGTG

GGGAGCAAAC

>ASV706 SS|1.0000|KJ634465_S004217796;k:Bacteria,p:"Bacteroidetes",c:Sphingobacteriia,o:"Sphingobacteriales",f:Chitinophagaceae

TACGGAGGGTGCAAGCGTTATCCGGATTCACTGGGTTTAAAGGGTGCGTAGGCGGACAGA

TAAGTCAGTGGTGAAATCTCCGGGCTTAACCCGGAAACTGCCGTTGATACTATTTGTCTT

GAATATTGTGGAGGTAAGCGGAATATGTCATGTAGCGGTGAAATGCTTAGATATGACATA

GAACACCAATTGCGAAGGCAGCTTACTACACAATTATTGACGCTGAGGCACGAAAGCGTG

GGGATCAAAC

>ASV707 GS|0.0|None;No hit

GTGTCAGCAGCCGCGGTTCCCCTGATCGGTGCCGGGAATGCCTCCGGTGTCCACCCATAA

AGCACGGCGTTGAACATGCTCTGGAAAAAGTACTCCATGGCGTTCAACCCAATGTTTGAC

GCTTCGGTGTTCACGGTGGCGAAGAGGAAGAGCGAGATGCCCATGAGTGCGGAACTTCCG

ACCATGGCCCACTTACGACCCACTCGAGGGACAGCATACATCAGCGCGCCTAGCATAACA

CCGGGGACAC

>ASV708 GS|0.0|None;No hit

TACGTGGAAGACTAGTGTTATTCATCTTTAATAGGTTTAAAGGGTACCTAGACGGTATAT

CTAGCCCGAAAAGGGTACTGATAAACTAGAGTTTAATGTGAGGGGGGATTAGTACTAATG

GAGTAGAGATGAAATTCTGTCATACCATAAGGAGACAGGTAACGGCGAAAGCAACCCTTT

ATGCAATAACTGACGTTGAAGGACGAAGGCTTGGGTCACAAACAGG

>ASV709 GS|0.0|None;No hit

GTGTCAGCAGCCGCAGCGATGACACCCTACATGCCCTGTCAATTGTCAACCACACTTGCG

AGAACCGTTGGCGCGATCGGCTGCGGCGTCCGCCGCGGAACTCTCGAACACTTGCATTTG

GCAAACTTGAAATCTGACTGCACACTTGAGCATCCCGTCATGATGCCCAATGTCGGTGCA

TGGTTTACAGCTTTGACATGAATTAATCTGTTCGAAGTCGCTTTTCGATTTTCGGCCTGC

CGACTTCGAA

>ASV710 SS|1.0000|EF368368_S000806277;k:Bacteria,p:"Proteobacteria",c:Alphaproteobacteria,o:Rhodospirillales,f:Acetobacteraceae

TACGAAGGGGGCTAGCGTTGCTCGGAATGACTGGGCGTAAAGGGCGCGTAGGCGGACTGA

TTAGTCAGGCGTGAAATTCCCGGGCTTAACCTGGGGGCTGCGTTTGATACGGTTGGTCTA

GAGTTTGGCAGAGGGTCGTGGAATTCCCAGTGTAGAGGTGAAATTCGTAGATATTGGGAA

GAACACCGGTGGCGAAGGCGGCGACCTGGTCCTTGACTGACGCTGAGGCGCGAAAGCGTG

GGGAGCAAAC

>ASV711 SS|1.0000|HM032898_S002167666;k:Bacteria,p:"Bacteroidetes",c:Cytophagia,o:Cytophagales,f:Cytophagaceae,g:Hymenobacter

TACGGAGGGTGCGAGCGTTGTCCGGATTTATTGGGTTTAAAGGGTGCGTAGGCGGCTTCG

TAAGTCTGGGGTGAAAGCCCGCTGCTCAACAGCGGAACTGCCCTGGATACTGCGGAGCTT

GAGGACAGACGAGGTTGGCGGAATAGAGGGTGTAGCGGTGAAATGCATAGATACCCTCTA

GAACCCCGATTGCGAAGGCAGCTGACTAGACTGTATCTGACGCTGAGGCACGAAAGCGTG

GGGAGCGAAC

>ASV712 SS|0.9200|AJ292687_S000017618;k:Bacteria,p:candidate_division_WPS-1,g:WPS-1_genera_incertae_sedis

GACAGAGGTGCCAAGCGTTAGGCGGAATCACTGGGCTTAAAGCGTGTGTAGGCGGATGTC

TAAGTACTTTGTGAAATCCCACGGCTCAACCGTGGAACTGCTCGGTATACTGGACATCTT

GAGGCAATTAGGGGTTACCGGAACAAGTGGTGGAGCGGTGAAATGCGTAGATATCACTTG

GAACGCCAATGGCGAAGGCAGGTAACTGGGATTGTCCTGACGCTGAGACACGAAAGCTAG

GGGAGCAAAC

>ASV713 SS|0.8800|AY140238_S000397413;k:Bacteria,p:"Proteobacteria",c:Alphaproteobacteria,o:Rhodospirillales,f:Acetobacteraceae,g:Acidicaldus

TACGAAGGGGGCTAGCGTTGCTCGGAATGACTGGGCGTAAAGGGCGCGTAGGCGGATGTC

TTTGTCGGGCGTGAAATTCCAGGGCTTAACCTTGGGGCTGCGTTCGAGACGGGGTGTCTA

GAGTTTGGCAGAGGGTCGTGGAATTCCCAGTGTAGAGGTGAAATTCGTAGATATTGGGAA

GAACACCGGTGGCGAAGGCGGCGACCTGGTCCATGACTGACGCTGATGCGCGAAAGCGTG

GGGAGCAAAC

>ASV714 GS|0.0|None;No hit

TACAAGGAAGACTAGTGTTATTCATCTTTACTAGGTTTAAAGGGTACCTAGACGGTTATG

ATAGCTCCGAAAGAGTACGTCAAAACTAGAGTTTGATAGGAGAGGAATATATTAGGGCTA

CTGGTGTAGAGATGAAATTCAGTCATACTAGTAGAACGGATAAAGGCGAAGGCAACCCTC

TATATATAAACTGACGTTGAGGGACGAAGGCTTGGGGAGCAAATAGG

>ASV715 SS|1.0000|AB545808_S002235816;k:Bacteria,p:"Bacteroidetes",c:Sphingobacteriia,o:"Sphingobacteriales",f:"Rhodothermaceae"

TACGGAGGGTCCAAGCGTTGTCCGGAATCACTGGGTGTAAAGGGTGCGCAGGCGGGCGTG

TAAGTCAGAGGTGAAAGCCACCGGCCTAACCGGTGAACTGCCTTTGATACTGCACGTCTT

GAGTCCCGGAGAGGCTATCGGAATTCGTGGTGTAGCGGTGAAATGCGTAAATATCACGAG

GAACACCGGATGCGTAAGCGGATAGCTGGACGGGTACTGACGCTCAGGCACGAAAGCGCG

GGGAGCGAAC

>ASV716 SS|1.0000|HM032898_S002167666;k:Bacteria,p:"Bacteroidetes",c:Cytophagia,o:Cytophagales,f:Cytophagaceae,g:Hymenobacter

TACGGAGGGTGCGAGCGTTGTCCGGATTTATTGGGTTTAAAGGGTGCGTAGGCGGCTTTG

TAAGTCCGGGGTGAAAGCCCGCTGCTCAACAGCGGAACTGCCCTGGATACTGCGGAGCTT

GAGTACAGACGAGGTTGGCGGAATGGACCGAGTAGCGGTGAAATGCATAGATACGGTCCA

GAACACCGATTGCGAAGGCAGCTGACTAGGCTGATACTGACGCTGAGGCACGACAGCGTG

GGGAGCGAAC

>ASV717 SS|1.0000|HM032898_S002167666;k:Bacteria,p:"Bacteroidetes",c:Cytophagia,o:Cytophagales,f:Cytophagaceae,g:Hymenobacter

TACGGAGGGTGCGAGCGTTGTCCGGATTTATTGGGTTTAAAGGGTGCGTAGGCGGCTTTT

TAAGTCTGGGGTGAAAGCCCGCTGCTCAACAGCGGAACTGCCCTGGATACTGGAGAGCTT

GAGTACAGACGAGGTTGGCGGAATGGACTGAGTAGCGGTGAAATGCATAGATACAGTCCA

GAACCCCGATTGCGAAGGCAGCTGACTAGGCTGTTACTGACGCTGAGGCACGAAAGCGTG

GGGAGCGAAC

>ASV718 GSL|97.2|AB193724_S000439756;k:Bacteria,p:"Proteobacteria",c:Betaproteobacteria,o:Methylophilales,f:Methylophilaceae,g:Methylotenera

TACGTAGGGTGCGAGCGTTAATCGGAATTACTGGGCGTAAAGCGTGCGCAGGCGGCTTTA

AAAGTCAGATGTGAAATCCCCGAGCTCAACTTGGGAACTGCGTTTGAAACTCTAAAGCTA

GAATATGTCAGAGGGGGGTAGAATTCCACGTGTAGCAGTGAAATGCGTAGAGATGTGGAG

GAATACCAATGGCGAAGGCAGCCCCCTGGGATAATATTGACGCTCATGCACGAAAGCGTG

GGGAGCAAAC

>ASV719 SS|1.0000|JN090860_S002916046;k:Bacteria,p:"Bacteroidetes",c:Cytophagia,o:Cytophagales,f:Cytophagaceae,g:Hymenobacter

TACGGAGGGTGCGAGCGTTGTCCGGATTTATTGGGTTTAAAGGGTGCGTAGGCGGCCGCG

TAAGTCTGGGGTGAAAGCCCGTTGCTCAACAACGGAACTGCCCTGGAAACTGCGCGGCTT

GAGTCCAGACGAGGTTGGCGGAATGGGCGGTGTAGCGGTGAAATGCATAGATACCGTCCA

GAACCCCGATTGCGAAGGCAGTTGACTAGGCTGGTACTGACGCTGAGGCACGAAAGCGTG

GGGAGCGAAC

>ASV720 SS|0.9100|AJ289983_S000020777;k:Bacteria,p:"Verrucomicrobia",c:Spartobacteria,g:Spartobacteria_genera_incertae_sedis

TACAGAGGTCCCAAGCGTTGTTCGGATTCATTGGGCGTAAAGGGTGCGCAGGCGGTTGGG

TAAGTCAGATGTGAAATCCTGGGGCTCAACCCCAGAACTGCATTTGATACTGCTCGGCTA

GAGGACTGGAGAGGTGATTGGAATTCACGGTGTAGCAGTGAAATGCGTAGATATCGTGAG

GAAGGCCAACGGCGAAGGCAGATCACTGGACAGTTCCTGACGCTCAGGCACGAAGGCCAG

GGGAGCAAAC

>ASV721 GS|0.0|None;No hit

CTTGGTCATTTAGAGGAAGTAAAAGTCGTAACAAGGTTTCCGTAGGTATGCAAGACTGCA

CGTTTGCCTACGGGAGCCCTCGCAGCGACTCTAAACAAGTGCCTCAGCCTGCGCCCCGAG

TGGGACTGGCGACGCCGTCAATCTGCGCTGGGAGGACCCCCTTGGGGCTCAACCAGAAGC

TCTTGAATGAGTTCACAGATCAGACGATGGCGGCCACTTGCGAGTGGTTTAGATATGACC

GGCCCCGGCC

>ASV722 SS|1.0000|FN391026_S001418752;k:Bacteria,p:"Planctomycetes",c:Planctomycetia,o:Planctomycetales,f:Planctomycetaceae

GACGAACCGTGCGAACGTTATTCGGAATTACTGGGCTTAAAGCGCGTGTAGGCGGGCCGG

CACGTCCGACGCTGAAATCCCCCGGCTCAACCGGGGAAGTGGCGGGGATACGACCGGCCT

CGAGGGGGGTAGGGGGACCTGGAACTTCCGGTGGAGCGGTGAAATGCGTTGAGATCGGAA

GGAACGCCCGTGGCGAAAGCGAGGTCCTGGACCCTTTCTGACGCTGAGACGCGAAAGCCA

GGGGAGCGAA

>ASV723 SS|0.9900|FJ177421_S001188350;k:Bacteria,p:"Bacteroidetes"

TACGGAGGGTGCAAGCGTTGTCCGGATTCATTGGGTTTAAAGGGTGCGTAGGCGGGACAT

TAAGTCCGGGGTGAAAGACGGTTGCTCAACAATCGCAGTGCCTTGGATACTGGTGTTCTT

GGGTGAAGTTGAGGTGGGCGGAATTCAAGGTGTAGCGGTGAAATGCATAGATACCTTGAA

GAACTCCGATTGCGAAGGCAGCCCACTGGACTTCTACCGACGCTGAGGCACGAAAGCGTG

GGGATCAAAC

>ASV724 SS|0.9900|AB859260_U010573780;k:Bacteria,p:"Bacteroidetes",c:Cytophagia,o:Cytophagales,f:Cytophagaceae,g:Hymenobacter

TACGGAGGGTGCGAGCGTTGTCCGGATTTATTGGGTTTAAAGGGTGCGTAGGCGGCTTGG

TAAGTCTGGGGTGAAAGCCCGCTGCTCAACAGCGGAACTGCCCTGGATACTGCGGAGCTT

GAGGACAGACGAGGTTGGCGGAATAGAGGGTGTAGCGGTGAAATGCATAGATACCCTCTA

GAACCCCGATTGCGAAGGCAGCTGACTAGACTGTATCTGACGCTGAGGCACGAAAGCGTG

GGGAGCGAAC

>ASV725 GS|0.0|None;No hit

TACGTAGAAGACTAGTGTTATTCATCTTTAATAGGTTTAAAGGGTACCTAGACGGTAAAT

CAAGCCTGAAATATGGGACTAATGTACTAGAGTTACTTACGAGGGGGTATTAAAGTACTG

CTGGTGTAGAGATGAAATTCTGTCATACCTCGTTTCACAGGAAACACTACGGCACAGGTA

TAGGCGAAAGCATCTCCTTATGTGATAACTGACGTTGAAGGACGAAGGCTTTGTGTAGCG

AACAGG

>ASV726 GS|99.6|KM044053_S004224125;k:Bacteria,p:"Actinobacteria",c:Actinobacteria,o:Actinomycetales,f:Nocardiaceae,g:Rhodococcus;

TACGTAGGGTGCAAGCGTTGTCCGGAATTACTGGGCGTAAAGAGCTCGTAGGCGGTTTGT

CGCGTCGACTGTGAAATCCCATGGCTCAACTGTGGGCGTGCAGTCGATACGGGCAGACTT

GAGTACTGCAGGGGAGACTGGAATTCCTGGTGTAGCGGTGAAATGCGCAGATATCAGGAG

GAACACCGGTGGCGAAGGCGGGTCTCTGGGCAGTAACTGACGCTGAGGAGCGAAAGCATG

GGTAGCGAAC

>ASV727 GS|0.0|None;No hit

GACAAGAGAGACTAGTGTTATTCATCTTTATTAGGTTTAAAGGGTACCTAGACTGTAAAT

TTAGCCCCTAAAGGGTACGTATTTACTAGAGTTATATGTGAGAGGAAGATGGAACTATTA

GTGTAAAGATGAAATTTTTTGATACTAATATAACGGGTAACGGCGAAGGCAACCCTCTAT

GTAATAACTGACGTTGAGGGACGAAGGCTCAGGGAGCGAAGAGG

>ASV728 GS|97.2|U88041_S000438700;k:Bacteria,p:"Proteobacteria",c:Alphaproteobacteria,o:Rhodobacterales,f:Rhodobacteraceae,g:Amaricoccus;

TACGGAGGGGGCTAGCGTTGTTCGGAATTACTGGGCGTAAAGCGCACGTAGGCGGATCAG

TCAGTCAGGGGTGAAATCCCGGGGCTCAACCCCGGAACTGCCTTTGATACTGCTGGTCTA

GAGATCATGAGAGGTGAGTGGAATTCCGAGTGTAGAGGTGAAATTCGTAGATATTCGGAG

GAACACCAGTGGCGAAGGCGGCTCACTGGCATGATACTGACGCTGAGGTGCGAAAGTGTG

GGGAGCAAAC

>ASV729 GS|97.6|KJ473428_S004089227;k:Bacteria,p:"Bacteroidetes",c:Cytophagia,o:Cytophagales,f:Cytophagaceae,g:Dyadobacter;

TACGGAGGGTGCGAGCGTTGTCCGGATTTATTGGGTTTAAAGGGTGCGTAGGTGGCCCTG

TAAGTCAGTGGTGAAATACAGCCGCTTAACGGTTGAGGTGCCATTGATACTGCAGGGCTT

GAAACAAGTGGAGGCTGCCGGAATGGATGGTGTAGCGGTGAAATGCATAGATATCATCCA

GAACACCGATTGCGAAGGCAGGTGGCTACGTTTGATTTGACACTGAGGCACGAAAGCATG

GGGAGCAAAC

>ASV730 SS|1.0000|FM252034_S001199926;k:Bacteria,p:"Proteobacteria",c:Alphaproteobacteria,o:Rhizobiales

TACGAAGGGGGCTAGCGTTGTTCGGATTTACTGGGCGTAAAGGGTGCGTAGGCGGATTGT

TAAGTCAGGGGTGAAATCCCGAGGCTCAACCTCGGAACTGCCTTTGATACTGGCGATCTT

GAGTCCGGGAGAGGTGAGTGGAACTGCGAGTGTAGAGGTGAAATTCGTAGATATTCGCAA

GAACACCGGTGGCGAAGGCGGCTCACTGGCCCGGTACTGACGCTGAGGCACGAAAGCGTG

GGGAGCAAAC

>ASV731 GS|98.4|DQ883810_S000728612;k:Bacteria,p:"Proteobacteria",c:Alphaproteobacteria,o:Rhizobiales,f:"Aurantimonadaceae",g:Aureimonas;

TACGAAGGGGGCTAGCGTTGTTCGGAATTACTGGGCGTAAAGCGCACGTAGGCGGATATT

TAAGTCGGGGGTGAAATCCCGGGGCTCAACCCCGGAACTGCCTTCGATACTGGGTATCTT

GAGTTCGGAAGAGGTGAGTGGAATTGCGAGTGTAGAGGTGAAATTCGTAGATATTCGCAG

GAACACCAGTGGCGAAGGCGGCTCACTGGTCCGATACTGACGCTGAGGTGCGAAAGCGTG

GGGAGCAAAC

>ASV732 GS|0.0|None;No hit

GTGTCAGCAGCCGCCCGTGCACCTCCATTGGCGTCGCCATAAGGTGGCTGATATGCCTCT

GGAGTCGCTGGAAGCGTTCGATGCATGGTTGCGAGAACGGTGGAACGAGAAAGAAGTGCT

TCTAGAGCAGCATGCCAAGACTGGCAGCTTCCCATCGGAATCGGAATCGGAACCGATCGT

CACGGAAGTGAAGTTGGGGCACTGGGTGGAACTTTGGGGCATCTGTGGCTTGCTGGCGAG

TCTAGCCATG

>ASV733 SS|1.0000|D86512_S000010801;k:Bacteria,p:"Proteobacteria",c:Alphaproteobacteria,o:Rhodospirillales,f:Acetobacteraceae

TACGAAGGGGGCTAGCGTTGCTCGGAATGACTGGGCGTAAAGGGCGCGTAGGCGGGTGTG

CCAGTCGGGCGTGAAATTCCTGGGCTTAACCTGGGGGCTGCGTTCGAGACGGCATGCCTA

GAGTGGGGAAGAGGGTCGTGGAATTCCCAGTGTAGAGGTGAAATTCGTAGATATTGGGAA

GAACACCGGTGGCGAAGGCGGCGACCTGGTCCTTGACTGACGCTGAGGCGCGAAAGCGTG

GGGAGCAAAC

>ASV734 SS|1.0000|EU861928_S001148233;k:Bacteria,p:"Armatimonadetes",c:Armatimonadia,o:Armatimonadales,f:Armatimonadaceae,g:Armatimonas/Armatimonadetes_gp1

TACGTAGGGGGCGAGCGTTGTCCGAAGTTACTGGGCGTAAAGAGCGCGTAGGCGGCCTCT

TAAGTGTGGGGTGAAAGTCCGAGGCTCAACCTCGGAACTGCCCTGCAAACTGGGGGGCTT

GAGTGCGGGAGGGGCGAGTGGAATGGTCGGTGTAGCGGTGAAATGCGTAGATATCGATCG

GAACACCCATGGCGAAGGCAGCTCGCTGGCCTGTAACTGACGCTGAGGCGCGAAAGCGTG

GGGAGCAAAC

>ASV735 SS|0.9700|Y18189_S000001545;k:Bacteria,p:Firmicutes

TACGTAGGTGGCAAGCGTTGTCCGGATTTACTGGGCGTAAAGCGAACGCAGGCGGATACT

TAAGTAGAGAGTGAAAGGCGGGAGCTCAACTCCTGGACTGCTCCCTATACTGGGTGTCTT

GAGTGGCGGAGAGGAAGATGGAACAAATCGTGTAGCGGTGAAATGCGTAGATATGATTTG

GAACACCAATGGCGAAAGCAATCTTCTGGACGCAAACTGACGCTGAGGTTCGAAAGCCAT

GGTAGCGAAC

>ASV736 SS|1.0000|HM032897_S002167665;k:Bacteria,p:"Bacteroidetes",c:Cytophagia,o:Cytophagales,f:Cytophagaceae,g:Hymenobacter

TACGGAGGGTGCGAGCGTTGTCCGGATTTATTGGGTTTAAAGGGTGCGTAGGCGGCTTTT

TAAGTCCGGGGTGAAAGCCCGCTGCTCAACAGCGGAACTGCCCTGGATACTGGACAGCTT

GAATACAGTGGAGGTTGGCGGAATGGACCGAGTAGCGGTGAAATGCATAGATACGGTCCA

GAACCCCGATTGCGAAGGCAGCTGACTACACTGGCATTGACGCTGAGGCACGACAGCGTG

GGGAGCGAAC

>ASV737 GS|100.0|HM474794_S002230137;k:Bacteria,p:"Proteobacteria",c:Alphaproteobacteria,o:Rhizobiales,f:Hyphomicrobiaceae,g:Devosia;

TACGAAGGGGGCTAGCGTTGTTCGGATTTACTGGGCGTAAAGCGCACGTAGGCGGATTGT

TAAGTGAGGGGTGAAATCCTGGAGCTCAACTCCAGAACTGCCTTTCATACTGGCAATCTA

GAGTCCGGAAGAGGTAAGTGGAACTCCTAGTGTAGAGGTGGAATTCGTAGATATTAGGAA

GAACACCAGTGGCGAAGGCGGCTTACTGGTCCGGTACTGACGCTGAGGTGCGAAAGCGTG

GGGAGCAAAC

>ASV738 GS|100.0|AB245358_S000627894;k:Bacteria,p:"Proteobacteria",c:Betaproteobacteria,o:Burkholderiales,f:Comamonadaceae,g:Variovorax;

TACGTAGGGTGCAAGCGTTAATCGGAATTACTGGGCGTAAAGCGTGCGCAGGCGGTTATG

TAAGACAGTTGTGAAATCCCCGGGCTCAACCTGGGAACTGCATCTGTGACTGCATAGCTA

GAGTACGGTAGAGGGGGATGGAATTCCGCGTGTAGCAGTGAAATGCGTAGATATGCGGAG

GAACACCGATGGCGAAGGCAATCCCCTGGACCTGTACTGACGCTCATGCACGAAAGCGTG

GGGAGCAAAC

>ASV739 GS|0.0|None;No hit

GGCGAAATTCGACTATAGGCTTAACGACGCCCCACCAGTCCCTATTCCGAATCTCGCGAA

AGACTTCAGCGCTGGTGATTCGTATCATACTCCCACCGAATCCGTCTCCTCCAGTGGCTC

CTATGGATCAGATGCTAAAAGCGGAAGCTCACGGTCAAGCCCACCCTTGTCCGATGCGTC

GACACGGCCACTTACAAATTCATTCAACGTAGACCATGTTGGTAACTCAAGGACTGACGT

CCACTCATCT

>ASV740 SS|1.0000|AJ416411_S000018317;k:Bacteria,p:"Proteobacteria",c:Alphaproteobacteria,o:Sphingomonadales,f:Sphingomonadaceae

TACGGAGGGGGCTAGCGTTGTTCGGAATTACTGGGCGTAAAGCGCACGTAGGCGGCTATT

CAAGTCAGAGGTGAAAGCCCGGAGCTCAACTCCGGAACTGCCTTTGAAACTAGGTAGCTC

GAATCATGGAGAGGCGAGTGGAATTCCGAGTGTAGAGGTGAAATTCGTAGATATTCGGAA

GAACACCAGTGGCGAAGGCGGCTCGCTGGACATGTATTGACGCTGAGGTGCGAAAGCGTG

GGGAGCAAAC

>ASV741 SS|0.9100|AF166114_S000498654;k:Bacteria,p:Cyanobacteria/Chloroplast,c:Chloroplast,f:Chloroplast

TACGGGGGATGCAAGTGTTATCCGGAATAATTGGGCGTAAAGCGTCTGTAGGTGGTTGAC

CAAGTCTGTTGTTAAAAATCAGGGCTTAACCCTGATCCGGCAACAGAAACTAGTAAACTT

GAGTACGGTAGGGGCAGAGGGAATTCTCGGTGTAGTGGTGAAATACGTAGATATCGAGAA

GAACACCAATAGCGAAAGCACTCTGCTGGGCCGAAACTGACATTGAGAGACGAAAGCTAG

GGGAGCAAAA

>ASV742 GS|100.0|AJ275038_S000127903;k:Bacteria,p:"Proteobacteria",c:Gammaproteobacteria,o:Pseudomonadales,f:Moraxellaceae,g:Acinetobacter;

TACAGAGGGTGCGAGCGTTAATCGGATTTACTGGGCGTAAAGCGTGCGTAGGCGGCTAAT

TGAGTCGGATGTGAAATCCCCGAGCTTAACTTGGGAATTGCATTCGATACTGGTTAGCTA

GAGTGTGGGAGAGGATGGTAGAATTCCAGGTGTAGCGGTGAAATGCGTAGAGATCTGGAG

GAATACCGATGGCGAAGGCAGCCATCTGGCCTAACACTGACGCTGAGGTACGAAAGCATG

GGGAGCAAAC

>ASV743 SS|1.0000|D86513_S000011943;k:Bacteria,p:"Proteobacteria",c:Alphaproteobacteria,o:Rhodospirillales,f:Acetobacteraceae

TACGAAGGGGGCTAGCGTTGCTCGGAATGACTGGGCGTAAAGGGCGCGTAGGCGGATGGC

ACAGTCAGATGTGAAATTCCCGGGCTTAACCTGGGGGCTGCATTTGATACGTGTTGTCTA

GAGTGAGGAAGAGGGTCGTGGAATTCCCAGTGTAGAGGTGAAATTCGTAGATATTGGGAA

GAACACCGGTGGCGAAGGCGGCGACCTGGTCCTTTTACTGACGCTGAGGCGCGAAAGCGT

GGGGAGCAAA

>ASV744 GS|100.0|DQ178975_S000603877;k:Bacteria,p:"Proteobacteria",c:Alphaproteobacteria,o:Sphingomonadales,f:Sphingomonadaceae,g:Sphingomonas;

TACGGAGGGAGCTAGCGTTGTTCGGAATTACTGGGCGTAAAGCGCACGTAGGCGGCTTTG

TAAGTTAGAGGTGAAAGCCTGGAGCTCAACTCCAGAATTGCCTTTAAGACTGCATCGCTC

GAATCCAGGAGAGGTGAGTGGAATTCCGAGTGTAGAGGTGAAATTCGTAGATATTCGGAA

GAACACCAGTGGCGAAGGCGGCTCACTGGACTGGTATTGACGCTGAGGTGCGAAAGCGTG

GGGAGCAAAC

>ASV745 GS|97.2|EF067921_S001020536;k:Bacteria,p:Cyanobacteria/Chloroplast,c:Chloroplast,f:Chloroplast,g:Bacillariophyta;

GACGGAGGATGCAAGTGTTATCCGGAATCACTGGGCGTAAAGCGTCTGTAGGTGGTTTAA

TAAGTCAACTGTTAAATCTTGAGGCTCAACCTCAAAATCGCAGTCGAAACTATTAGACTA

GAGTATAGTAGGGGTAAAGGGAATTTCCAGTGGAGCGGTGAAATGCGTAGAGATTGGAAA

GAACACCGATGGCGAAAGCACTTTACTAGGCTATTACTGACACTCAGAGACGAAAGCTAG

GGTAGCAAAT

>ASV746 SS|0.9600|AB245334_S000627870;k:Bacteria,p:"Actinobacteria"

TACGTAGGGGGCCAGCGTTGTCCGGAATCATTGGGCGTAAAGAGCGCGTAGGCGGTCCGA

TCAGTCCGCTGTGAAAGTCAGGGGCTCAACCCCTGAAAGCCGGTGGATACTGTCGGGCTA

GAGTCCGGAAGAGGCGAGTGGAATTCCTGGTGTAGCGGTGAAATGCGCAGATATCAGGAG

GAACACCTATGGCGAAGGCAGCTCGCTGGGACGGTACTGACGCTGAGGCGCGAAAGCGTG

GGGAGCAAAC

>ASV747 SS|1.0000|KJ504175_S004089758;k:Bacteria,p:"Actinobacteria",c:Actinobacteria,o:Actinomycetales

TACGTAGGGTGCAAGCGTTGTCCGGAATTATTGGGCGTAAAGAGCTCGTAGGCGGTCTGT

TACGTCGGCTGTGAAAACCTGGGGCTCAACTCCGGGCCTGCAGCCGATACGGGCAGACTA

GAGTTCGGCAGGGGAGACTGGAATTCCTGGTGTAGCGGTGAAATGCGCAGATATCAGGAG

GAACACCGGTGGCGAAGGCGGGTCTCTGGGCCGATACTGACGCTGAGGAGCGAAAGCGTG

GGGAGCAAAC

>ASV748 GS|0.0|None;No hit

TACGCAAAAGACAAGTGTTATTCATCTTAAATGGGTTTAAAGGGTACCTAGACAGGTAAT

TAAGCTGTAGAATGGACTGATTATCTAGAGTTTTATGTGAAAAGGCCGTATTGCCAGAGG

AGAGATGGAATTCTATCATACTGGTAGGACGGGTAAAGGCGAAGGCAACCTTTTATGTAA

AAACTGACGTTGAGGGACGAAGCCTAGGAGATCGATTAGG

>ASV749 SS|1.0000|AB251884_S000650634;k:Bacteria,p:"Bacteroidetes",c:Cytophagia,o:Cytophagales,f:Cytophagaceae,g:Hymenobacter

TACGGAGGGTGCAAGCGTTGTCCGGATTTATTGGGTTTAAAGGGTGCGTAGGCGGCTTTT

TAAGTCTGGGGTGAAAGCCCGTTGCTCAACAACGGAACTGCCCTGGAAACTGGAGAGCTT

GAGTACAGACGAGGGCGGCGGAATGGACGGTGTAGCGGTGAAATGCATAGATACCGTCCA

GAACCCCGATTGCGAAGGCAGCTGCCTAGACTGTAACTGACGCTGAGGCACGAAAGCGTG

GGGAGCGAAC

>ASV750 GS|99.2|AJ252828_S000015744;k:Bacteria,p:"Actinobacteria",c:Actinobacteria,o:Actinomycetales,f:Pseudonocardiaceae,g:Pseudonocardia;

TACGTAGGGTGCGAGCGTTGTCCGGAATTATTGGGCGTAAAGAGCTCGTAGGCGGTCTGT

CGCGTCGGTCGTGAAAACTTGGGGCTTAACCCTGAGCTTGCGGTCGATACGGGCATGACT

TGAGTTCGGTAGGGGAGACTGGAATTCCTGGTGTAGCGGTGAAATGCGCAGATATCAGGA

GGAACACCGGTGGCGAAGGCGGGTCTCTGGGCCGATACTGACGCTGAGGAGCGAAAGCGT

GGGGAGCGAA

>ASV751 SS|1.0000|AM850678_S000925699;k:Bacteria,p:"Planctomycetes",c:Planctomycetia,o:Planctomycetales,f:Planctomycetaceae

GACGAACCGTGCGAACGTTATTCGGAATTACTGGGCTTAAAGCGCGTGTAGGCGGGTTCG

AACGTCCGCTGCTGAAAGCCCCCGGCTCAACCGGGGAAGTGGCACGGATACGGCGAGCCT

GGAGGGGGGTAGAGGGGCCTGGAACTTCCGGTGGAGCGGTGAAATGCGTTGAGATCGGAA

GGAACGCCCGTGGCGAAAGCGAGGCCCTGGACCCTTACTGACGCTGAGACGCGAAAGCCA

GGGGAGCGAA

>ASV752 SS|0.9900|AB847449_S004125734;k:Bacteria,p:"Proteobacteria",c:Deltaproteobacteria,o:Myxococcales

GACAGAGGGTGCAAACGTTGTTCGGAATTACTGGGCGTAAAGCGTGTGTAGGCGGTGAGG

TAAGTCGGATGTGAAAGCCCAGGGCTCAACCCTGGAAGTGCACTCGATACTGCTTCGCTT

GAGTACTGGAGAGGTTGGTGGAATTCTCGGTGTAGAGGTGAAATTCGTAGATATCGAGAG

GAACATCTGTGGCGAAGGCGGCCAACTGGACAGATACTGACGCTGAGACACGAAAGTGTG

GGGAGCAAAC

>ASV753 SS|0.8300|AJ289983_S000020777;k:Bacteria,p:"Verrucomicrobia",c:Spartobacteria,g:Spartobacteria_genera_incertae_sedis

TACAGAGGTCTCAAGCGTTGTTCGGATTCATTGGGCGTAAAGGGAGCGTAGGCGGTCGGG

TAAGTTGGATGTGAAATCCTGGGGCTCAACCTCAGAACTGCATTCAATACTGCTTGGCTA

GAGTACTGGAGAGGTGAGTGGAATTCACGGTGTAGCAGTGAAATGCGTAGATATCGTGAG

GAAGACCAGCGGCGAAGGCGGCTCACTGGACAGTTACTGACGCTGATGCTCGAAGGCCAG

GGGAGCAAAC

>ASV754 SS|1.0000|FN391026_S001418752;k:Bacteria,p:"Planctomycetes",c:Planctomycetia,o:Planctomycetales,f:Planctomycetaceae

GACGAACCGTGCGAACGTTATTCGGAATCACTGGGCTTAAAGCGCGTGTAGGCGGGTCGC

CGCGTCGGCTGCTGAAATCCCCCGGCTCAACCGGGGAACGGGCACCGATACGGGCGGCCT

CGAGGGGGGTAGGGGGGTCTGGAACTTCCGGTGGAGCGGTGAAATGCGTTGAGATCGGAA

GGAACGCCCGTGGCGAAAGCGAGACCCTGGACCCTTTCTGACGCTGAGACGCGAAAGCCA

GGGGAGCGAA

>ASV755 SS|1.0000|JF999998_S002914924;k:Bacteria,p:"Bacteroidetes",c:Sphingobacteriia,o:"Sphingobacteriales",f:Sphingobacteriaceae

TACGGAGGATCCGAGCGTTATCCGGATTTATTGGGTTTAAAGGGTGCGTAGGCGGCCTGT

TAAGTCAGGGGTGAAAGACGGTGGCTCAACCACCGCAGTGCCTTTGATACTGACGGGCTT

GAATGCAGTTGAGGTAGGCGGAATGTGGCAAGTAGCGGTGAAATGCATAGATATGCCACA

GAACACCAATTGCGAAGGCAGCTTACCAAAGTGTGATTGACGCTGAGGCACGAAAGCGTG

GGGATCAAAC

>ASV756 SS|1.0000|D30771_S000413726;k:Bacteria,p:"Proteobacteria",c:Alphaproteobacteria,o:Rhodospirillales,f:Acetobacteraceae

TACGAAGGGGGCTAGCGTTGCTCGGAATGACTGGGCGTAAAGGGCGTGTAGGCGGCTTGG

TGAGTTAGACGTGAAATTCCTGGGCTCAACCTGGGGGCTGCGTTTGATACAGCTAGGCTA

GAGTGGGGAAGAGGGTTGTGGAATTCCCAGTGTAGAGGTGAAATTCGTAGATATTGGGAA

GAACACCGGTGGCGAAGGCGGCAACCTGGTCCTTGACTGACGCTGAGGCGCGAAAGCGTG

GGGAGCAAAC

>ASV757 GS|0.0|None;No hit

GTGTCAGCAGCCGCAGATGTCAAATACCGATCCGGCGCCGTTTTCTACGACTTCCATCAG

GAATCAAATTTCTTCTATCTGACCGGTTTGCTTTCCCCGGCCGGGAGAGAGATGCCTATG

GCTGAGCCTGTCTAGGTTTCAACGAACCAGATGCCGTTGCGGTTATAGGTATGCCACGGC

AACGCATCCAGCCTTGAGGTCGGACGACTCACAGCACCAGGAAAGGCTTCTGACGAGGAT

GACCATACTT

>ASV758 GS|98.4|AJ429239_S000145113;k:Bacteria,p:"Proteobacteria",c:Alphaproteobacteria,o:Sphingomonadales,f:Sphingomonadaceae,g:Sphingomonas;

TACGGAGGGAGCTAGCGTTATTCGGAATTACTGGGCGTAAAGCGCACGTAGGCGGCTTTG

TAAGTAAGAGGTGAAAGCCTGGAGCTCAACTCCAGAATTGCCTTTTAGACTGCATCGCTT

GAATCATGGAGAGGTCAGTGGAATTCCGAGTGTAGAGGTGAAATTCGTAGATATTCGGAA

GAACACCAGTGGCGAAGGCGGCTGACTGGACATGTATTGACGCTGAGGTGCGAAAGCGTG

GGGAGCAAAC

>ASV759 GS|77.6|EF515236_S000839820;k:Bacteria,p:"Armatimonadetes",c:Chthonomonadetes,o:Chthonomonadales,f:Chthonomonadaceae,g:Chthonomonas/Armatimonadetes_gp3;

TACGTAGGTGGCAAGCGTTGTCCGGATTTACTGGGCGTAAAGCGAACGCAGGCGGATGGT

TAAGTAGGAAGTGAAAGGTTGCAGCTCAACTGCGACACTGCTTCTTATACTGGCCGTCTT

GAGTAGCGGAGAGGGAGATGGAACGACACGTGTAGCGGTGAAATGCGTTGATATGTGTCG

GAACACCAATGGCGAAAGCAATCTCCTGGACGCAGACTGACGCTGAGGTTCGAAAGCCAA

GGTAGCAAAC

>ASV760 SS|0.9300|KJ528316_S004225669;k:Bacteria,p:"Proteobacteria",c:Alphaproteobacteria,o:Sphingomonadales,f:Sphingomonadaceae

TACGGAGGGGGCTAGCGTTGTTCGGAATTACTGGGCGTAAAGCGCACGTAGGCGGCTATT

CAAGTCAGGGGTGAAAGCCCGGAGCTCAACTCCGGAACTGCCTCTGAAACTATGTAGCTT

GAATCATGGAGAGGCGAGTGGAATTCCGAGTGTAGAGGTGAAATTCGTAGATATTCGGAA

GAACACCAGTGGCGAAGGCGGCTCGCTGGACATGTATTGACGCTGAGGTGCGAAAGCGTG

GGGAGCAAAC

>ASV761 GSL|98.0|EF532793_S000859919;k:Bacteria,p:"Proteobacteria",c:Betaproteobacteria,o:Burkholderiales,f:Comamonadaceae

TACGTAGGGTGCGAGCGTTAATCGGAATTACTGGGCGTAAAGCGTGCGCAGGCGGTCTTG

TAAGACAGGTGTGAAATCCCCGGGCTCAACCTGGGAATTGCATTTGTGACTGCAAGGCTG

GAGTGCGGCAGAGGGGGATGGAATTCCGCGTGTAGCAGTGAAATGCGTAGATATGCGGAG

GAACACCGATGGCGAAGGCAATCCCCTGGGCCTGCACTGACGCTCATGCACGAAAGCGTG

GGGAGCAAAC

>ASV762 GS|0.0|None;No hit

TACGGGGGGGGCGAGCGTTATTCGAAATGATTGGGCGTAAAGGGCACGTAGACGGTTTTT

TGAGTTGACATGCTTGAGGTCGTACAAAGAATACAAAATAAATATTCTTTCGTACGTCTA

AAAGATTAAAATGGAGTGTGGATTTTCACCCTGTATTCTTTGCTTGACAAAAGAAAAGGT

ATTCGGTTTGCTTTACACTTGGGAAAAAGGCCAAGGCTCAACCATGGTGTTTCCCGCTAT

ACTATAAAAC

>ASV764 SS|1.0000|D86512_S000010801;k:Bacteria,p:"Proteobacteria",c:Alphaproteobacteria,o:Rhodospirillales,f:Acetobacteraceae

TACGAAGGGGGCTAGCGTTGCTCGGAATGACTGGGCGTAAAGGGCGCGTAGGCGGATTGG

TCAGTCAGACGTGAAATTCCTGGGCTTAACCTGGGGGCTGCGTTTGAGACGGCTCGTCTT

GAGTTTGGAAGAGGGTCGTGGAATTCCCAGTGTAGAGGTGAAATTCGTAGATATTGGGAA

GAACACCGGTGGCGAAGGCGGCGACCTGGTCCTGGACTGACGCTGAGGCGCGAAAGCGTG

GGGAGCAAAC

>ASV765 SS|0.8800|AB267478_S000721192;k:Bacteria,p:"Bacteroidetes",c:Sphingobacteriia,o:"Sphingobacteriales",f:Chitinophagaceae,g:Segetibacter

TACGGAGGGTGCAAGCGTTATCCGGATTCACTGGGTTTAAAGGGTGCGTAGGTGGGTCTG

TAAGTCAGTGGTGAAATCTCCGGGCTTAACCCGGAAACTGCCATTGATACTATAGACCTT

GAATTTTCTGGAGGTTAGCGGAATATGTCATGTAGCGGTGAAATGCTTAGATATGACATA

GAACACCAATTGCGAAGGCAGCTAACTACAGGGATATTGACACTGAGGCACGAAAGCGTG

GGGATCAAAC

>ASV766 SS|1.0000|DQ986200_S000736608;k:Bacteria,p:"Planctomycetes",c:Planctomycetia,o:Planctomycetales,f:Planctomycetaceae

GACGAACCGTGCGAACGTTATTCGGAATCACTGGGCTTAAAGCGCGTGTAGGCGGGCCGG

CACGTCGGTCGCTGAAATCCCCCGGCTCAACCGGGGAAGGGGCGCCGATACGACCGGCCT

GGAGGGGAGTAGGGGGGCCTGGAACTTCCGGTGGAGCGGTGAAATGCGTTGAGATCGGAA

GGAACGCCCGTGGCGAAAGCGAGGCCCTGGACTCCTGCTGACGCTGAGACGCGAAAGCCA

GGGGAGCGAA

>ASV767 SS|1.0000|AF166114_S000498654;k:Bacteria,p:Cyanobacteria/Chloroplast

TACGGAGGATGCAAGCGTTATCCGGAATGATTGGGCGTAAAGCGTCTGTAGGTGGGTTGT

AAAGTCTTCTGTTAAAGATCGGGGCTTAACCCAGTTTAAGCAGTGGAAACTTATAACCTA

GAGTACGGTAGGGGCAGAGGGAATTCCCGGTGTAGCGGTGAAATGCGTAGATATCGGGAA

GAACACCGACGGCGAAAGCACTCTGCTGGGCCGAAACTGACACTGAGAGACGAAAGCTAG

GGGATCAAAG

>ASV768 SS|1.0000|AJ519386_S000102346;k:Bacteria,p:"Acidobacteria",c:Acidobacteria_Gp3

TACGTAGGCAGCAAGCGTTGTTCGGAATTACTGGGCGTAAAGAGTGTGTAGGCGGTGCTC

TAAGTTCGGTGTGAAATCTCCCGGCTCAACTGGGAGGGTGCGCCGAAAACTGGAATGCTT

GAACATGGGAGAGGAAAGCGGAATTCCTGGTGTAGCGGTGAAATGCGTAGATATCAGGAG

GAACACCTGCGGTGTAGACGGCTTTCTGGACCATTGTTGACGCTGAGACACGAAAGCGTG

GGTAGCAAAC

>ASV769 SS|1.0000|AF245017_S000498898;k:Bacteria,p:"Actinobacteria",c:Actinobacteria,o:Actinomycetales

TACGTAGGGTGCAAGCGTTGTCCGGAATTATTGGGCGTAAAGAGCTCGTAGGCGGTTTGT

CGCGTCGACTGTGAAAACTCAGGGGCTCAACTCCGAGCTTGCAGTTGATACGGGCAGACT

AGAGTTCGGCAGGGGAGACTGGAATTCCTGGTGTAGCGGTGAAATGCGCAGATATCAGGA

GGAACACCGGTGGCGAAGGCGGGTCTCTGGGCCGATACTGACGCTGAGGAGCGAAAGCGT

GGGGAGCGAA

>ASV770 GS|98.8|HE599560_S003258029;k:Bacteria,p:"Actinobacteria",c:Actinobacteria,o:Actinomycetales,f:Nakamurellaceae,g:Nakamurella;

TACGTAGGGTGCAAGCGTTGTCCGGAATTATTGGGCGTAAAGAGCTCGTAGGCGGTCTGT

CGCGTCGAATGTGAAAATCCGGGGCTCAACCCCGGACCTGTATTCGATACGGGCAGACTA

GAGTTCGGTAGGGGAGTCTGGAATTCCTGGTGTAGCGGTGAAATGCGCAGATATCAGGAG

GAACACCGGTGGCGAAGGCGGGACTCTGGGCCGATACTGACGCTGAGGAGCGAAAGCGTG

GGGAGCAAAC

>ASV771 GS|0.0|None;No hit

GTGTCAGCAGCCGCGACCTCTGAACAGCGCCTCGAGACCTTCTTCAACCAGCAATGGGTC

CAGCAAGGGGAACCCGAGATCCTCTTCATTAAGCGTGCAACACGGACCGGCGACAGGTGG

ACAGGACAAGTCGCATTTCCAGGAGGTGGCAGAGAGCCGAATGACGAGAATGATTGTGCT

GCCAGTGTT

>ASV772 SS|1.0000|AB859260_U010573780;k:Bacteria,p:"Bacteroidetes",c:Cytophagia,o:Cytophagales,f:Cytophagaceae,g:Hymenobacter

TACGGAGGGTGCGAGCGTTGTCCGGATTTATTGGGTTTAAAGGGTGCGTAGGCGGCTTGG

TAAGTCTGGGGTGAAAGCCCGCTGCTCAACAGCGGAACTGCCCTGGATACTGCCCAGCTT

GAGGACAGACGAGGTTGGCGGAATAGAGGGTGTAGCGGTGAAATGCATAGATACCCTCTA

GAACCCCGATTGCGAAGGCAGCTGACTAGACTGTATCTGACGCTGAGGCACGAAAGCGTG

GGGAGCGAAC

>ASV773 SS|1.0000|FN600641_S001794056;k:Bacteria,p:"Actinobacteria",c:Actinobacteria,o:Actinomycetales

TACGTAGGGTGCAAGCGTTGTCCGGAATTATTGGGCGTAAAGAGCTCGTAGGTGGTCTGT

TGCGTCGGCTGTGAAACCCCGAGGCTCAACCTCGGGCCTGCAGCCGATACGGGCAGACTA

GAGTTCGGCAGGGGAGACTGGAATTCCTGGTGTAGCGGTGAAATGCGCAGATATCAGGAG

GAACACCGGTGGCGAAGGCGGGTCTCTGGGCCGATACTGACACTGAGGAGCGAAAGCGTG

GGGAGCAAAC

>ASV774 GS|0.0|None;No hit

TTCCAGCTCCAATAGCGTATATTAAAGTTGTTGCAGTCAAAAAGCTCGTAGTTGAACCTT

GGGCCTGGCTGGCCGGTCCGCCTCACCGCGTGCACTGGTCCGGCCGGGCCTTTCCTTCTG

GGGATCCACATGCCCTTCACTGGGTGTGCCGGGGAACCAGGACTTTTACTTTGAAAAAAT

TAGAGTGTTCAAAGCAGGCCTATGCTCGAATACATTAGCATGGAATAATAGAATAGGACG

TGTGGTTCTA

>ASV775 GS|79.6|AY512588_S004009252;k:Bacteria,p:"Elusimicrobia",c:Endomicrobia,g:Candidatus_Endomicrobium;

TACGTAGGTGGCAAGCGTTGTCCGGATTTACTGGGCGTAAAGCGTATGCAGGCGGATGTT

TAAGTAGGAAGTGAAAGGTTGCGGCTCAACCGCAACACTGCTTCCTATACTGGGCATCTT

GAGTGGCGGAGAGGGAGATGGAACGACACGTGTAGCGGTGAAATGCGTTGATATGTGTCG

GAACACCAATGGCGAAAGCAATCTCCTGGACGCAAACTGACGCTGAGATACGAAAGCTAA

GGTAGCAAAC

>ASV776 GS|0.0|None;No hit

GTGTCAGCAGCCGCCCCGGTTCAGGCAAGGAAATCATCCTCCCGCAGGCCAGCACTTGCC

GCAATGGTTCGCCCAGCACCAGAACTTAAGCCCTACCGCGCAGGAAGATGCCCTTAAGCA

TGAGCCCGGCTTTGCGCATTTGCCAGGAGGCCAGCAACAGCAAGTCATTGACCGCTTGCA

CCGGCTTGACCTGGCACCTCCTGCGCAACGCCAGCGCATGATGGAGCGCAATGAACGCTT

CGAGGCACTG

>ASV777 GS|0.0|None;No hit

TAGAACGCAGCGATACCGGAGTTGATCAGCCCAATAATCGCAAGCGAAACCAGGCCCGAG

TGCAATGCCGCCATAAACACATAGAACTTGCCGAAGAAACCGCCGGTAAAGGGAATGCCG

ATCAACGAGATGAGAAAGAAGGCCATGGCACCGCCCAGCAGCGGCGAGCGGTAACCCAGG

CCCCGGTAGTCC

>ASV778 GS|98.4|AB166881_S000386315;k:Bacteria,p:"Proteobacteria",c:Alphaproteobacteria,o:Caulobacterales,f:Caulobacteraceae,g:Phenylobacterium;

TACGAAGGGGGCTAGCGTTGCTCGGAATTACTGGGCGTAAAGGGCGCGTAGGCGGACAGT

TTAGTCAGAGGTGAAAGCCCAGGGCTCAACCTTGGAACTGCCTTTGATACTGGCTGTCTT

GAGTTCGGGAGAGGTGAGTGGAACTCCGAGTGTAGAGGTGAAATTCGTAGATATTCGGAA

GAACACCGGTGGCGAAGGCGACTCACTGGCCCGATACTGACGCTGAGGCGCGAAAGCGTG

GGGAGCAAAC

>ASV779 SS|0.9900|D86512_S000010801;k:Bacteria,p:"Proteobacteria",c:Alphaproteobacteria,o:Rhodospirillales,f:Acetobacteraceae

TACGAAGGGGGCTAGCGTTGCTCGGAATGACTGGGCGTAAAGGGCGCGTAGGCGGATGTT

TTAGTCAGGCGTGAAAGTCCTGGGCTTAACCTGGGGATTGCGTTTGATACGGGGCATCTA

GAGTTGGGAAGAGGGTCGTGGAATTCCCAGTGTAGAGGTGAAATTCGTAGATATTGGGAA

GAACACCGGTGGCGAAGGCGGCGACCTGGTCCTTGACTGACGCTGAGGCGCGAAAGCGTG

GGGAGCAAAC

>ASV780 GS|98.0|EU861928_S001148233;k:Bacteria,p:"Armatimonadetes",c:Armatimonadia,o:Armatimonadales,f:Armatimonadaceae,g:Armatimonas/Armatimonadetes_gp1;

TACGTAGGGGGCGAGCGTTGTCCGAAGTTACTGGGCGTAAAGAGCGCGTAGGCGGGTTTT

TAAGTGAGGGGTGAAATTTCGAGGCTCAACCTCGGAACTGCCTTTCATACTGGAGACCTT

GAGTGCGGGAGAGGCGAGTGGAATGGTTGGTGTAGCGGTGAAATGCGTAGATATCAATCG

GAACACCCATGGCGAAGGCAGCTCGCTGGCCTGTAACTGACGCTGAGGCGCGAAAGCGTG

GGGAGCAAAC

>ASV781 SS|1.0000|FJ842646_S001352336;k:Bacteria,p:"Actinobacteria",c:Actinobacteria,o:Actinomycetales

TACGTAGGGTGCAAGCGTTGTCCGGAATTATTGGGCGTAAAGGGCTCGTAGGCGGTCCAT

CGCGTCGGGAGTGAAAAGCTGGGGCTTAACCCCAGTCCTGCTTCCGATACGGGTGGACTA

GAGGTAGGTAGGGGAGATCGGAATTCCTGGTGTAGCGGTGAAATGCGCAGATATCAGGAG

GAACACCGGTGGCGAAGGCGGATCTCTGGGCCTTACCTGACGCTGAGGAGCGAAAGCATG

GGGAGCAAAC

>ASV782 SS|1.0000|AJ585986_S000247788;k:Bacteria,p:"Deinococcus-Thermus",c:Deinococci,o:Deinococcales,f:Deinococcaceae,g:Deinococcus

TACGGAGGGTGCAAGCGTTACCCGGAATCACTGGGCGTAAAGGGCGTGTAGGCGGCATCA

CAAGTCTGGTTTTAAAGCCCGCGGCTCAACCGCGGAGTTGGACTGGAGACTGTGAAGCTA

GACCTCTGGAGAGGAAGCTGGAATTCCTGGTGTAGCGGTGGAATGCGTAGATACCAGGAG

GAACACCAATGGCGAAGGCAAGCTTCTGGACAGAAGGTGACGCTGAGGCGCGAAAGTGTG

GGGAGCGAAC

>ASV783 GS|0.0|None;No hit

GTGTCAGCAGCCGCAGTATTGAAAGAGGCGGTAGCAAGTTTGGCATGAGTCCCCTGTTTC

TTGAGCCAATCTTTCAAGGCGGTGCTCTTGGCCTGGGTGTTGACTTGTAGTACAACATTG

CGCTTAGTCTTAACTAAATCGCCGGCGTCAATGCGCAGCTCATAGTCGGCATCGTTCTTT

TGTGTGCGCGATGGCAGTATGCGGCCACCTGATGTATGCGAGGCTTTCAATTTAGTTGCC

ACGTCGAAGT

>ASV784 SS|0.9300|AM162406_S000650674;k:Bacteria,p:"Planctomycetes",c:Planctomycetia,o:Planctomycetales,f:Planctomycetaceae,g:Zavarzinella

TACGAACCGTGCGAACGTTGTTCGGAATCATTGGGCTTAAAGGGCGCGTAGGCGGGCTTC

CAAGTCCGAGGTGAAATCCTCCAGCTCAACTGGAGAACTGCCCCGGATACTGGAGGTCTC

GAGGAGGGTAGGGGCATGCGGAACTGTGGGTGGAGCGGTGAAATGCGTTGATATCCACAG

GAACTCCGGTGGCGAAGGCGGCGTGCTGGACCCTTTCTGACGCTGAGGCGCGAAAGCCAG

GGGAGCAAAC

>ASV785 GS|98.8|HF536497_S003787727;k:Bacteria,p:"Bacteroidetes",c:Sphingobacteriia,o:"Sphingobacteriales",f:Sphingobacteriaceae,g:Pedobacter;

TACGGAGGATCCAAGCGTTATCCGGATTTATTGGGTTTAAAGGGTGCGTAGGCGGCTTAT

TAAGTCAGGGGTGAAAGACGGTGGCTCAACCATCGCAGTGCCTTTGATACTGATGAGCTT

GAATATACTAGAGGTAGGCGGAATGTGACAAGTAGCGGTGAAATGCATAGATATGTCACA

GAACACCGATTGCGAAGGCAGCTTACTATGGTATTATTGACGCTGAGGCACGAAAGCGTG

GGGATCAAAC

>ASV786 SS|1.0000|HM032898_S002167666;k:Bacteria,p:"Bacteroidetes",c:Cytophagia,o:Cytophagales,f:Cytophagaceae,g:Hymenobacter

TACGGAGGGTGCGAGCGTTGTCCGGATTTATTGGGTTTAAAGGGTGCGTAGGCGGCTTCG

TAAGTCTGGGGTGAAAGCCCGTTGCTCAACAACGGAACTGCCCTGGAAACTGCGGGGCTT

GAGTCCAGACGAGGTCGGCGGAATGGGCGGTGTAGCGGTGAAATGCATAGATACCGTCCA

GAACCCCGATTGCGAAGGCAGCTGACTAGGCTGGTACTGACGCTGAGGCACGAAAGCGTG

GGGAGCGAAC

>ASV787 SS|1.0000|EF516412_S000840854;k:Bacteria,p:"Armatimonadetes",c:Armatimonadia,o:Armatimonadales,f:Armatimonadaceae,g:Armatimonas/Armatimonadetes_gp1

TACGTAGGGGGCCAGCGTTGTTCGAAGTTACTGGGCGTAAAGAGCGCGTAGGCGGACTCT

TAAGTGAGGGGTGAAAGGTTCAGGGCTTAACCCGGACACTGCCTTTTATACTGGGGGTCT

TGAGTGTTGGAGAGGCGAGTGGAATGGTCGGTGTAGCGGTGAAATGCGTAGATATCGATC

GGAACACCCATGGCGAAGGCAGCTCGCTGGCCAACAACTGACGCTGAGGCGCGAAAGCGT

GGGGAGCAAA

>ASV788 SS|1.0000|AB192292_S000650534;k:Bacteria,p:"Bacteroidetes",c:Sphingobacteriia,o:"Sphingobacteriales",f:Chitinophagaceae

TACGGAGGGTGCAAGCGTTATCCGGATTCACTGGGTTTAAAGGGTGCGTAGGTGGGCAGT

TAAGTCAGTGGTGAAATCTCCGGGCTCAACCCGGAAACTGCCATTGATACTATCTGTCTT

GAATATCGTTGAGGTAAGCGGAATATGTCATGTAGCGGTGAAATGCTTAGATATGACATA

GAACACCAATTGCGAAGGCAGCTTGCTAACCGAATATTGACACTGAGGCACGAAAGCGTG

GGGATCAAAC

>ASV789 GS|0.0|None;No hit

GTGTCAGCAGCCTCCTATCAATAAGAGACTAATTGGAAAGGTATTATGGCGATGTGAGCA

CCCTCAATGCCCTCACCTTCACCCCCCCCCAGATAACAAGCCAAATAACCCGTCCGCCCC

AGTAGTCC

>ASV790 SS|1.0000|FN600641_S001794056;k:Bacteria,p:"Actinobacteria",c:Actinobacteria,o:Actinomycetales

TACGTAGGGTGCAAGCGTTGTCCGGAATTATTGGGCGTAAAGAGCTCGTAGGCGGTTTGT

CGCGTCGGCTGTGAAACCCTGAGGCTCAACCTCAGGCCTGCAGTCGATACGGGCAGACTA

GAGTCCGGCAGGGGAGACTGGAATTCCTGGTGTAGCGGTGAAATGCGCAGATATCAGGAG

GAACACCGGTGGCGAAGGCGGGTCTCTGGGCCGGAACTGACGCTGAGGAGCGAAAGCGTG

GGGAGCGAAC

>ASV791 SS|0.9900|U65647_S000438120;k:Bacteria,p:"Actinobacteria",c:Actinobacteria,o:Rubrobacterales,f:Rubrobacteraceae,g:Rubrobacter

TACGTAGGGGGCGAGCGTTGTCCGGAATTATTGGGCGTAAAGAGCGTGTAGGCGGTTCGG

TAAGTCTGTCGTGAAAACCTGGGGCTCAACCCCGGGCGTGCGATGGATACTGCCGGGCTA

GAGGGTGGTAGAGGCGAGTGGAATTCCCAGTGTAGCGGTGAAATGCGCAGATATTGGGAG

GAACACCAGTAGCGAAGGCGGCTCGCTGGGCCACACCTGACGCTGAGACGCGAAAGCGTG

GGGAGCAAAC

>ASV792 SS|1.0000|FN600641_S001794056;k:Bacteria,p:"Actinobacteria",c:Actinobacteria,o:Actinomycetales

TACGTAGGGTGCAAGCGTTGTCCGGAATTATTGGGCGTAAAGAGCTCGTAGGCGGTCCGC

TACGTCGGCTGTGAAATCCCGAGGCTCAACCTCGGGCCTGCAGTCGATACGGGCAGACTA

GAGTTCGGTAGGGGAGACTGGAATTCCTGGTGTAGCGGTGAAATGCGCAGATATCAGGAG

GAACACCGGTGGCGAAGGCGGGTCTCTGGGCCGAAACTGACGCTGAGGAGCGAAAGCGTG

GGGAGCAAAC

>ASV793 GS|100.0|AB245356_S000627892;k:Bacteria,p:"Proteobacteria",c:Gammaproteobacteria,o:Pseudomonadales,f:Pseudomonadaceae,g:Rhizobacter;

TACGTAGGGTGCAAGCGTTAATCGGAATTACTGGGCGTAAAGCGTGCGCAGGCGGCTTTG

CAAGACAGATGTGAAATCCCCGGGCTCAACCTGGGAACTGCATTTGTGACTGCATGGCTA

GAGTACGGTAGAGGGGGATGGAATTCCGCGTGTAGCAGTGAAATGCGTAGATATGCGGAG

GAACACCGATGGCGAAGGCAATCCCCTGGACCTGTACTGACGCTCATGCACGAAAGCGTG

GGGAGCAAAC

>ASV794 SS|1.0000|AJ871304_S000460115;k:Bacteria,p:"Actinobacteria",c:Actinobacteria,o:Actinomycetales

TACGTAGGGTGCAAGCGTTGTCCGGAATTATTGGGCGTAAAGAGCTCGTAGGCGGTCTGT

CACGTCGGCTGTGAAAACTTGGGGCTCAACCCCAAGCCTGCAGTCGATACGGGCAGACTA

GAGTGCGGTAGGGGAGACTGGAATTCCTGGTGTAGCGGTGAAATGCGCAGATATCAGGAG

GAACACCGGTGGCGAAGGCGGGTCTCTGGGCCGTAACTGACGCTGAGGAGCGAAAGCGTG

GGGAGCAAAC

>ASV795 SS|0.9700|AJ292687_S000017618;k:Bacteria,p:candidate_division_WPS-1,g:WPS-1_genera_incertae_sedis

GACAGAGGTGCCAAGCGTTAGGCGGAATCACTGGGCTTAAAGCGTGTGTAGGCGGATGTC

TAAGTACCTTGTGAAATCCCACGGCTCAACCGTGGAACTGCTCGGTATACTGGATGTCTT

GAGGCAATTAGGGGTTACTGGAACAAGTGGTGGAGCGGTGAAATGCGTAGATATCACTTG

GAACGCCAATGGCGAAGGCAGGTAACTGGGATTGTCCTGACGCTGAGACACGAAAGCCAG

GGGAGCAAAC

>ASV796 SS|1.0000|FN600641_S001794056;k:Bacteria,p:"Actinobacteria",c:Actinobacteria,o:Actinomycetales

TACGTAGGGTGCAAGCGTTGTCCGGAATTATTGGGCGTAAAGAGCTCGTAGGTGGTCTGT

TGCGTCGGCTGTGAAACCCCGAGGCTCAACCTCGGGCCTGCAGCCGATACGGGCAGACTA

GAGTTCGGCAGGGGAGACTGGAATTCCTGGTGTAGCGGTGAAATGCGCAGATATCAGGAG

GAACACCGGTGGCGAAGGCGGGTCTCTGGGCCGATACTGACACTGAGGAGCGAAAGCGTG

GGGAGCGAAC

>ASV797 SS|1.0000|HM032897_S002167665;k:Bacteria,p:"Bacteroidetes",c:Cytophagia,o:Cytophagales,f:Cytophagaceae,g:Hymenobacter

TACGGAGGGTGCGAGCGTTGTCCGGATTTATTGGGTTTAAAGGGTGCGTAGGCGGCCGTT

TAAGTCTGGGGTGAAAGCCCGTTGCTCAACAGCGGAACTGCCCTGGATACTGAACGGCTT

GAATACAGTGGAGGTTGGCGGAATGGACCGAGTAGCGGTGAAATGCATAGATACGGTCCA

GAACCCCGATTGCGAAGGCAGCTGACTACACTGGTATTGACGCTGAGGCACGACAGCGTG

GGGAGCGAAC

>ASV798 GS|100.0|DQ344632_S000640797;k:Bacteria,p:"Actinobacteria",c:Actinobacteria,o:Actinomycetales,f:Pseudonocardiaceae,g:Pseudonocardia;

TACGTAGGGTGCGAGCGTTGTCCGGAATTATTGGGCGTAAAGAGCTCGTAGGCGGTCTGT

CGCGTCGGTCGTGAAAACTTGGGGCTTAACCCTGAGCTTGCGGTCGATACGGGCATGACT

GGAGTTCGGCAGGGGAGACTGGAATTCCTGGTGTAGCGGTGAAATGCGCAGATATCAGGA

GGAACACCGGTGGCGAAGGCGGGTCTCTGGGCCGATACTGACGCTGAGGAGCGAAAGCGT

GGGGAGCGAA

>ASV799 SS|0.9900|HM032898_S002167666;k:Bacteria,p:"Bacteroidetes",c:Cytophagia,o:Cytophagales,f:Cytophagaceae,g:Hymenobacter

TACGGAGGGTGCGAGCGTTGTCCGGATTTATTGGGTTTAAAGGGTGCGTAGGCGGCTCTT

TAAGTCTGGGGTGAAAGCCCGCTGCTCAACAGCGGAACTGCCCTGGATACTGAAGAGCTT

GAGTACAGACGAGGTTGGCGGAATGGACGGAGTAGCGGTGAAATGCATAGATACCGTCCA

GAACCCCGATTGCGAAGGCAGCTGACTAGGCTGATACTGACGCTGAGGCACGACAGCGTG

GGGAGCGAAC

>ASV800 GSL|99.2|EF553529_S000860302;k:Bacteria,p:"Actinobacteria",c:Actinobacteria,o:Actinomycetales,f:Intrasporangiaceae

TACGTAGGGTGCGAGCGTTGTCCGGAATTATTGGGCGTAAAGAGCTTGTAGGCGGTTTGT

CGCGTCTGCTGTGAAAATCCGGGGCTCAACCCCGGACTTGCAGTGGGTACGGGCAGACTA

GAGTGTGGTAGGGGAGACTGGAATTCCTGGTGTAGCGGTGGAATGCGCAGATATCAGGAG

GAACACCGATGGCGAAGGCAGGTCTCTGGGCCATTACTGACGCTGAGAAGCGAAAGCATG

GGGAGCAAAC

>ASV801 SS|0.9900|AF127407_S000387324;k:Bacteria,p:"Proteobacteria",c:Alphaproteobacteria,o:Rhodospirillales,f:Acetobacteraceae

TACGAAGGGGGCTAGCGTTGCTCGGAATGACTGGGCGTAAAGGGCGCGTAGGCGGATTGG

TCAGTCAGACGTGAAATTCCTGGGCTCAACCTGGGGGCTGCGTTTGAGACGGCTGATCTA

GAGTTTGGAAGAGGGTTGTGGAATTCCCAGTGTAGAGGTGAAATTCGTAGATATTGGGAA

GAACACCGGTGGCGAAGGCGGCAACCTGGTCCTTGACTGACGCTGAGGCGCGAAAGCGTG

GGGAGCAAAC

>ASV803 SS|0.9900|HM032898_S002167666;k:Bacteria,p:"Bacteroidetes",c:Cytophagia,o:Cytophagales,f:Cytophagaceae,g:Hymenobacter

TACGGAGGGTGCGAGCGTTGTCCGGATTTATTGGGTTTAAAGGGTGCGTAGGCGGCCGTT

TAAGTCTGGGGTGAAAGCCCGCTGCTCAACAGCGGAACTGCCCTGGATACTGGATGGCTT

GAGTACAGACGAGGTTGGCGGAATGGACTGAGTAGCGGTGAAATGCATAGATACAGTCCA

GAACACCGATTGCGAAGGCAGCTGACTAGGCTGATACTGACGCTGAGGCACGACAGCGTG

GGGAGCGAAC

>ASV804 SS|1.0000|AB778530_U010573767;k:Bacteria,p:"Proteobacteria",c:Alphaproteobacteria,o:Rhodospirillales,f:Acetobacteraceae

TACGAAGGGGGCTAGCGTTGCTCGGAATGACTGGGCGTAAAGGGCGCGTAGGCGGTTCAC

GCAGTCAGATGTGAAATTCCTGGGCTTAACCTGGGGGCTGCATTTGAGACGCGTAGGCTT

GAGTGTGAAAGAGGGTCGTGGAATTCCCAGTGTAGAGGTGAAATTCGTAGATATTGGGAA

GAACACCGGTGGCGAAGGCGGCGACCTGGTTCACAACTGACGCTGAGGCGCGAAAGCGTG

GGGAGCAAAC

>ASV805 GS|98.8|AF234731_S000340045;k:Bacteria,p:"Acidobacteria",c:Acidobacteria_Gp6,g:Gp6;

TACAGAGGTGGCAAGCGTTGTTCGGAATTACTGGGCGTAAAGGGCGCGTAGGCGGCCTTC

TAAGTCGGACGTGAAAGCCCCAGGCTTAACCTGGGAACTGCGTCTGATACTGGGAGGCTT

GGATTCGGGAGAGGGATGTGGAATTCCAGGTGTAGCGGTGAAATGCGTAGATATCTGGAG

GAACACCGGTGGCGAAGGCGGCATCCTGGACCGAGATCGACGCTGAGGCGCGAAAGCTAG

GGGAGCAAAC

>ASV806 GS|99.2|AY218772_S000356842;k:Bacteria,p:"Verrucomicrobia",c:Spartobacteria,g:Spartobacteria_genera_incertae_sedis;

TACAGAGGTCCCAAGCGTTGTTCGGATTCATTGGGCGTAAAGGGTGCGTAGGTGGCGCCG

TCAGTGGGGTGTGAAATTTCGGAGCTTAACTCCGAAACTGCATTCCATACTGCGGTGCTT

GAGGACTGGAGAGGAGACTGGAATTCATGGTGTAGCAGTGAAATGCGTAGAGATCATGAG

GAAGACCAGTGGCGAAGGCGGGTCTCTGGACAGTTCCTGACACTGAGGCACGAAGGCTAG

GGGAGCGAAC

>ASV807 SS|1.0000|EF635408_S001095322;k:Bacteria,p:"Deinococcus-Thermus",c:Deinococci,o:Deinococcales,f:Deinococcaceae,g:Deinococcus

TACGGAGGGTGCAAGCGTTACCCGGAATCACTGGGCGTAAAGGGCGTGTAGGCCGCACTC

CAAGTCTGGCTTTAAAGACCGAAGCTCAACTTCGGGACTGGGCCGGAAACTGGAGCGCTA

GACGGATGGAGAGGTCACTGGAATTCCTGGTGTAGCGGTGGAATGCGTAGATACCAGGAG

GAACACCAACGGCGAAGGCAGGTGACTGGACATTTTGTGACGCTGAGGCGCGAAAGTGTG

GGGAGCGAAC

>ASV808 SS|1.0000|AY788950_S000610642;k:Bacteria,p:"Proteobacteria",c:Alphaproteobacteria,o:Rhodospirillales,f:Acetobacteraceae

TACGAAGGGGGCTAGCGTTGCTCGGAATGACTGGGCGTAAAGGGCGCGTAGGCGGTTCGT

ACAGTCGGATGTGAAATTCCTGGGCTTAACCTGGGGGCTGCATTCGATACGTGCGGGCTT

GAGTGGGGAAGAGGGTTGTGGAATTCCCAGTGTAGAGGTGAAATTCGTAGATATTGGGAA

GAACACCGGTGGCGAAGGCGGCAACCTGGTCCTTGACTGACGCTGAGGCGCGAAAGCGTG

GGGAGCAAAC

>ASV809 GS|97.2|JN090860_S002916046;k:Bacteria,p:"Bacteroidetes",c:Cytophagia,o:Cytophagales,f:Cytophagaceae,g:Hymenobacter;

TACGGAGGGTGCGAGCGTTGTCCGGATTTATTGGGTTTAAAGGGTGCGTAGGCGGCCTTT

TAAGTCTGGGGTGAAAGCCCGTTGCTCAACAACGGAACTGCCCTGGATACTGGAAGGCTT

GAGTACAGACGAGGTTGGCGGAATGGACGGAGTAGCGGTGAAATGCATAGATACCGTCCA

GAACCCCGATTGCGAAGGCAGCTGACTAGGCTGTTACTGACGCTGAGGCACGACAGCGTG

GGGAGCGAAC

>ASV810 GS|0.0|None;No hit

TTCCAGCTCCAATAGCGTATATTAAAGTTGCTGCAGTTAAAAAGCTCGTAGTTGGATTTC

TGTACGTGACGTGTCTGGCCTTGGCGTGGTTACACGTCCGAGTGCCGGACGTTGTCCGTC

TGTCTTCTCCGTTCTGTCCGTCGTCCTTCACTGGATGTGCGGTACAGGTGGGGATCGTTT

ACTTTGAACAAATTAGAGTGTTTCAAGCAGGTCGTGTGCCTTGTATACCTTAGCATGGAA

TAATCATCTA

>ASV811 SS|1.0000|X75044_S000001387;k:Bacteria,p:Cyanobacteria/Chloroplast,c:Cyanobacteria

GACGGAGGATGCAAGCGTTATCCGGAATGATTGGGCGTAAAGCGTCCGCAGGTGGCAGTT

CAAGTCTGCTGTCAAAGACCGGGGCTCAACCTCGGAAAGGCAGTGGAAACTGAACAGCTA

GAGTATGGTAGGGGCAAAGGGAATTCCTGGTGTAGCGGTGAAATGCGTAGAGATCAGGAA

GAACATCGGTGGCGAAGGCGCTTTGCTGGACCATAACTGACACTCAGGGACGAAAGCTAG

GGGAGCGAAT

>ASV812 SS|1.0000|FN391026_S001418752;k:Bacteria,p:"Planctomycetes",c:Planctomycetia,o:Planctomycetales,f:Planctomycetaceae

GACGAACCGTGCGAACGTTATTCGGAATCACTGGGCTTAAAGCGCGTGTAGGCGGGACGG

CACGTCGGTCGCTGAAATCCCCCGGCTCAACCGGGGAAGTGGCGCCGATACGACCGCCCT

GGAGGGACGTAGGGGGGGCTGGAACTTCCGGTGGAGCGGTGAAATGCGTTGAGATCGGAA

GGAACGCCCGTGGCGAAAGCGAGCCCCTGGACGTCTACTGACGCTGAGACGCGAAAGCCA

GGGGAGCGAA

>ASV813 SS|1.0000|D86513_S000011943;k:Bacteria,p:"Proteobacteria",c:Alphaproteobacteria,o:Rhodospirillales,f:Acetobacteraceae

TACGAAGGGGGCTAGCGTTGCTCGGAATGACTGGGCGTAAAGGGCGCGTAGGCGGTTCGT

ACAGTCGGGTGTGAAATTCCTGGGCTTAACCTGGGGACTGCATTCGAGACGTGCGGGCTT

GAGTGGGGAAGAGGGTTGTGGAATTCCCAGTGTAGAGGTGAAATTCGTAGATATTGGGAA

GAACACCGGTGGCGAAGGCGGCAACCTGGTCCTTGACTGACGCTGAGGCGCGAAAGCGTG

GGGAGCAAAC

>ASV814 SS|1.0000|AY140238_S000397413;k:Bacteria,p:"Proteobacteria",c:Alphaproteobacteria,o:Rhodospirillales,f:Acetobacteraceae

TACGAAGGGGGCTAGCGTTGCTCGGAATGACTGGGCGTAAAGGGCGCGTAGGCGGACATT

TTAGTCGGGCGTGAAATTCCTGGGCTTAACCTGGGGGCTGCGTTCGATACGGGGTGTCTA

GAGTTTGGCAGAGGGTCGTGGAATTCCCAGTGTAGAGGTGGAATTCGTAGATATTGGGAA

GAACACCGGTGGCGAAGGCGGCGACCTGGTCCTTGACTGACGCTGAGGCGCGAAAGCGTG

GGGAGCAAAC

>ASV815 GS|100.0|AB071954_S000334613;k:Bacteria,p:"Proteobacteria",c:Alphaproteobacteria,o:Caulobacterales,f:Caulobacteraceae,g:Brevundimonas;

TACGAAGGGGGCTAGCGTTGCTCGGAATTACTGGGCGTAAAGGGAGCGTAGGCGGACATT

TAAGTCAGGGGTGAAATCCCGGGGCTCAACCTCGGAATTGCCTTTGATACTGGGTGTCTT

GAGTATGAGAGAGGTGTGTGGAACTCCGAGTGTAGAGGTGAAATTCGTAGATATTCGGAA

GAACACCAGTGGCGAAGGCGACACACTGGCTCATTACTGACGCTGAGGCTCGAAAGCGTG

GGGAGCAAAC

>ASV816 SS|1.0000|X70769_S000529355;k:Bacteria,p:Cyanobacteria/Chloroplast,c:Cyanobacteria

GACGGAGGATGCAAGCGTTATCCGGAATGATTGGGCGTAAAGCGTCCGCAGGTGGCAGTT

CAAGTCTGCTGTCAAAGACCGGGGCTTAACCTCGGAAAGGCAGTGGAAACTGAACAGCTA

GAGTATGGTAGGGGCAAAGGGAATTCCTGGTGTAGCGGTGAAATGCGTAGAGATCAGGAA

GAACATCGGTGGCGAAGGCGCTTTGCTGGACCATAACTGACACTCAGGGACGAAAGCTAG

GGGAGCGAAT

>ASV817 SS|1.0000|EF516412_S000840854;k:Bacteria,p:"Armatimonadetes",c:Armatimonadia,o:Armatimonadales,f:Armatimonadaceae,g:Armatimonas/Armatimonadetes_gp1

TACGTAGGGGGCCAGCGTTGTTCGAAGTTACTGGGCGTAAAGAGCGCGTAGGCGGATCCT

TAAGTGAGGGGTGAAAGGTTCAGGGCTTAACCCGGACACTGCCTTTCATACTGGGGATCT

TGAGTGTTGGAGAGGCGAGTGGAATGGTCGGTGTAGCGGTGAAATGCGTAGATATCGATC

GGAACACCCATGGCGAAGGCAGCTCGCTGGCCAACAACTGACGCTGAGGCGCGAAAGCGT

GGGGAGCAAA

>ASV818 SS|0.9400|FJ842646_S001352336;k:Bacteria,p:"Actinobacteria",c:Actinobacteria,o:Actinomycetales,f:Nocardioidaceae

TACGTAGGGTGCAAGCGTTGTCCGGAATTATTGGGCGTAAAGGGCTCGTAGGCGGTCCAT

CGCGTCGGGAGTGAAAAGCTGGGGCTCAACCCCAGTCCTGCTTCCGATACGGGTGGACTA

GAGGTATGCAGGGGAGAACGGAATTCCTGGTGTAGCGGTGAAATGCGCAGATATCAGGAG

GAACACCGGTGGCGAAGGCGGTTCTCTGGGCATTACCTGACGCTGAGGAGCGAAAGCGTG

GGGAGCAAAC

>ASV819 GS|0.0|None;No hit

GTGTCAGCAGCCGCAGAAGGCCTGATGGTTGATGGATGTTGCTGATACGGAGCGGAAGAT

TGGAACGAGCAATGGTTTTCCCACTTCCAAATTGACACACTCGCTCAACCAGCTCATCGC

CAGTAACGAGAGCTGACAGATCATACTCGCGAGCATACAGTCGGCGTCAGACCACGGCGA

GAGTGGGGCGCTCCCTCGGCCTCGGCATTCGGGGCACCTCGGTCTCGCCCATCGAATCTA

TCTTCGGCCA

>ASV820 SS|1.0000|EU861928_S001148233;k:Bacteria,p:"Armatimonadetes",c:Armatimonadia,o:Armatimonadales,f:Armatimonadaceae,g:Armatimonas/Armatimonadetes_gp1

TACGTAGGGGGCGAGCGTTGTCCGAAGTTACTGGGCGTAAAGAGCGCGTAGGCGGGTTTT

TAAGTGAGGGGTGAAATTCCGAGGCTTAACCTCGGAACTGCCTTTCATACTGGAAACCTT

GAGTGTGGGAGAGGCGAGTGGAATGGTCGGTGTAGCGGTGAAATGCGTAGATATCGATCG

GAACACCCATGGCGAAGGCAGCTCGCTGGCCTATAACTGACGCTGAGGCGCGAAAGCGTG

GGGAGCAAAC

>ASV821 SS|1.0000|HM032897_S002167665;k:Bacteria,p:"Bacteroidetes",c:Cytophagia,o:Cytophagales,f:Cytophagaceae,g:Hymenobacter

TACGGAGGGTGCGAGCGTTGTCCGGATTTATTGGGTTTAAAGGGTGCGTAGGCGGCCGTT

TAAGTCTGGGGTGAAAGCCCGCTGCTCAACAGCGGAACTGCCCTGGATACTAGATGGCTT

GAATACAGTGGAGGTTGGCGGAATGGACTGAGTAGCGGTGAAATGCATAGATACAGTCCA

GAACCCCGATTGCGAAGGCAGCTGACTACACTGGTATTGACGCTGAGGCACGACAGCGTG

GGGAGCGAAC

>ASV822 SS|0.9200|AJ289983_S000020777;k:Bacteria,p:"Verrucomicrobia",c:Spartobacteria,g:Spartobacteria_genera_incertae_sedis

TACAGAGGCCTCAAGCGTTGTTCGGATTCATTGGGCGTAAAGGGAGCGTAGGCGGTGGGG

TAAGTTGGATGTGAAATCCTGGGGCTCAACCTCAGAACTGCATTCAATACTGCTCCGCTA

GAGTACTGGAGAGGAGATTGGAATTCACGGTGTAGCAGTGAAATGCGTAGATATCGTGAG

GAAGACCAGTGGCGAAGGCGGATCTCTGGACAGTTACTGACGCTGAGGCTCGAAGGCTAG

GGGAGCAAAC

>ASV823 GS|0.0|None;No hit

CACGTAAAAGACTAGTGTTACTCATCTTTAATAGGTTTAAAGGGTACCTAGACTGCCAAT

CAAGCCCCCAAAAGGGTACTAATTGGCTAGAGTTTAATATAAGGAGGCATGAAAGTACTG

AGAATGTTGAGAGATGAAATTCTGTGATACTATATATGGCACTGGTAAAGGCGAAAGCAT

CTTATTATGTAATAACTGACGTTGAGGGACGAAGGCTGTGTATAGCAAAAAGG

>ASV824 GS|0.0|None;No hit

GTGTCAGCAGCCGCAGTCCAAGTCGGGAGAATTTCGTGATAATACCCGATTGCGCTGAAG

AAGGTATCCGAGGAGACACCATCGGATGTGAAGTTCAGCACGACACCGGTGACAGGGAAA

TCCCGATGTGTCTTGACGGTAAGAGAGTGAACAACACCATACGTACCACCGCCCCCTCCA

CCCAAAGCCCAATACAAATCAGGGTTCTCGGATCTTGAAGCTTTCAAAAGGCGCCCATGA

CCATCCACAA

>ASV825 GS|100.0|DQ911241_S000768873;k:Bacteria,p:Firmicutes,c:Clostridia,o:Clostridiales,f:Peptoniphilaceae,g:Peptoniphilus;

TACGTAGGGGGCTAGCGTTGTCCGGAATCACTGGGCGTAAAGGGTTCGCAGGCGGAAATG

CAAGTCAGATGTAAAAGGCAGTAGCTTAACTACTGTAAGCATTTGAAACTGCATATCTTG

AGAAGAGTAGAGGTAAGTGGAATTTTTAGTGTAGCGGTGAAATGCGTAGATATTAAAAAG

AATACCGGTGGCGAAGGCGACTTACTGGGCTCATTCTGACGCTGAGGAACGAAAGCGTGG

GTAGCAAACA

>ASV826 SS|0.9800|EU135026_S000933620;k:Bacteria,p:"Armatimonadetes",c:Chthonomonadetes,o:Chthonomonadales,f:Chthonomonadaceae,g:Chthonomonas/Armatimonadetes_gp3

TACGGAGGGGGCGAGCGTTGTCCGAAGTTACTGGGCGTAAAGGGCGCGTAGGCGGGTTGG

CAAGTCCGCTGTGAAAGCCCGGCGCTTAACGCCGGAGGGTCGGTGGATACTGTCAGTCTT

GAAGGTGCTAGGGACAGATGGAATTACCAGTGTAGCGGTGAAATGCGTAGATATTGGTAG

GAACACCAGTGGCGAAGGCGGTCTGTTGGAGCACTCTTGACGCTGAGGCGCGAAAGCTGG

GGGAGCGAAC

>ASV827 SS|1.0000|EU714260_S001045469;k:Bacteria,p:"Bacteroidetes",c:Sphingobacteriia,o:"Sphingobacteriales",f:Chitinophagaceae

TACGGAGGGTGCAAGCGTTATCCGGATTCACTGGGTTTAAAGGGTGCGTAGGCGGGCAGG

TAAGTCCGTGGTGAAATCTCTGAGCTTAACTCAGAAACTGCCATGGATACTATTTGTCTT

GAATGTTGTGGAGGTAAGCGGAATATGTCATGTAGCGGTGAAATGCTTAGATATGACATA

GAACACCAATTGCGAAGGCAGCTTACTACACAAATATTGACGCTGAGGCACGAAAGCGTG

GGGATCAAAC

>ASV828 SS|0.8400|AB649056_S002949821;k:Bacteria,p:"Proteobacteria",c:Alphaproteobacteria,o:Sphingomonadales,f:Sphingomonadaceae

TACGGAGGGGGCTAGCGTTGTTCGGAATTACTGGGCGTAAAGCGTCCGTAGGCGGCTTTC

CAAGTCAGAGGTGAAATCCCACGACTCAATCGTGGAACTGCCTTTGAGACTGGTTCGCTT

GAAGATGGGAGAGGTTAGTGGAATTCCGAGTGTAGAGGTGAAATTCGTAGATATTCGGAA

GAACACCAGTGGCGAAGGCGGCTAACTGGACCATTCTTGACGCTGAGGGACGAAAGCGTG

GGGAGCAAAC

>ASV829 GSL|97.2|AB778531_U010573768;k:Bacteria,p:"Proteobacteria",c:Alphaproteobacteria,o:Rhodospirillales,f:Acetobacteraceae

TACGAAGGGGGCTAGCGTTGCTCGGAATGACTGGGCGTAAAGGGCGCGTAGGCGGTTTGG

ACAGTCAGATGTGAAATTCCTGGGCTTAACCTGGGGGCTGCATTTGATACGTCCAGGCTT

GAGTGTGGAAGAGGGTTGTGGAATTCCCAGTGTAGAGGTGAAATTCGTAGATATTGGGAA

GAACACCGGTGGCGAAGGCGGCAACCTGGTCCATTACTGACGCTGAGGCGCGAAAGCGTG

GGGAGCAAAC

>ASV830 GS|98.8|EU888308_S001169258;k:Bacteria,p:"Proteobacteria",c:Betaproteobacteria,o:Burkholderiales,f:Comamonadaceae,g:Delftia;

TACGTAGGGTGCGAGCGTTAATCGGAATTACTGGGCGTAAAGCGTGCGCAGGCGGTGATG

TAAGACAGATGTGAAATCCCCGGGCTCAACCTGGGACCTGCATTTGTGACTGCATCGCTA

GAGTACGGTAGAGGGGGATGGAATTCCGCGTGTAGCAGTGAAATGCGTAGATATGCGGAG

GAACACCGATGGCGAAGGCAATCCCCTGGACCTGTACTGACGCTCATGCACGAAAGCGTG

GGGAGCAAAC

>ASV831 GS|0.0|None;No hit

TACGGGGGGGGCGAGCGTTATTCGAAATGATTGGGCGTAAAGGGCACGTAGACGGTTTTT

TAAGTGGACATCTTCTCTTTTTTGTTCTCTAAGGATAAAAAAGAAAGGATTATGGAAATC

TTTCTGTACTCGGGAAAAGGACCAAGGCTCAACCATGGTGTTTCCCACCAAACTAAAAAA

CTAGAGTAAGTTAGAGGAAAGTGGAATTCCTGGAGGAAAGGTTAAATTTTATGATATCAG

GAGGAACGCC

>ASV832 SS|1.0000|D86512_S000010801;k:Bacteria,p:"Proteobacteria",c:Alphaproteobacteria,o:Rhodospirillales,f:Acetobacteraceae

TACGAAGGGGGCTAGCGTTGCTCGGAATGACTGGGCGTAAAGGGCGCGTAGGCGGATCAC

ACAGTCAGGCGTGAAATTCCTGGGCTTAACCTGGGGGCTGCGTTTGAGACGTGGGGTCTA

GAGTGGGGAAGAGGGTCGTGGAATTCCCAGTGTAGAGGTGAAATTCGTAGATATTGGGAA

GAACACCGGTGGCGAAGGCGGCGACCTGGTCCTTGACTGACGCTGAGGCGCGAAAGCGTG

GGGAGCAAAC

>ASV833 SS|1.0000|FN600641_S001794056;k:Bacteria,p:"Actinobacteria",c:Actinobacteria,o:Actinomycetales

TACGTAGGGTGCAAGCGTTGTCCGGAATTATTGGGCGTAAAGAGCTCGTAGGCGGTCTGC

CACGTCGGCTGTGAAATCCCGAGGCTCAACCTCGGGCCTGCAGTCGATACGGGCAGACTA

GAGTTCGGTAGGGGAGACTGGAATTCCTGGTGTAGCGGTGAAATGCGCAGATATCAGGAG

GAACACCGGTGGCGAAGGCGGGTCTCTGGGCCGAAACTGACGCTGAGGAGCGAAAGCGTG

GGGAGCAAAC

>ASV834 SS|1.0000|EF368368_S000806277;k:Bacteria,p:"Proteobacteria",c:Alphaproteobacteria,o:Rhodospirillales,f:Acetobacteraceae

TACGAAGGGGGCTAGCGTTGCTCGGAATGACTGGGCGTAAAGGGCGCGTAGGCGGCTTGG

TTCGTCAGACGTGAAATTCCTGGGCTCAACCTGGGGGCTGCGTTTGATACAGCTGGGCTA

GAGTGGGGAAGAGGGTTGTGGAATTCCCAGTGTAGAGGTGAAATTCGTAGATATTGGGAA

GAACACCGGTGGCGAAGGCGGCAACCTGGTCCTTGACTGACGCTGAGGCGCGAAAGCGTG

GGGAGCAAAC

>ASV835 GS|0.0|None;No hit

GTGTCAGCAGCCAACACATCACCATATAAGCAGGAGTGAACGCCTTTATCATGAACAGCT

AGACCATCATGTACCTGTCCAAGTACACCGATAGTCTTGACGGACTTAGGGAGCGCCTTG

ATGAACTCCTCCTCGATAAAAGGACGATACACTCGGACATTCAGTACGCCGAGTTTAGCA

CCATCTCGTTCGAGTGATAAGACAACCTGTGTAGCCAAGGAGGATTCTACAGTGCCAAAG

ACTACCAAAA

>ASV836 GS|0.0|None;No hit

GTGTCAGCAGCCGCACGAGCGTGGGGAGGAGTGCATCACGCTATATGATGTCAGTTTTGG

CGAGTCAGGACTTCAGAGATCTACAACCGAATCCTGTGGTTCCTACAAGGTGCTGACCTC

CCAACCGATCAAGCCAGACGTCGATATCTTGATACGTCAAAGCTTTCGGCCGACCTCTCG

AACCATCCCTGATCGCGTTACTTTTCGATCGGTGTCAACGGTGACCGACTCGGCCGAGAT

GCCAAGTGGA

>ASV837 GSL|100.0|JN175341_S002916785;k:Bacteria,p:"Proteobacteria",c:Gammaproteobacteria,o:Pseudomonadales,f:Moraxellaceae,g:Enhydrobacter

TACAGAGGGTGCGAGCGTTAATCGGAATTACTGGGCGTAAAGCGAGTGTAGGTGGCTCAT

TAAGTCACATGTGAAATCCCCGGGCTTAACCTGGGAACTGCATGTGATACTGGTGGTGCT

AGAATATGTGAGAGGGAAGTAGAATTCCAGGTGTAGCGGTGAAATGCGTAGAGATCTGGA

GGAATACCGATGGCGAAGGCAGCTTCCTGGCATAATATTGACACTGAGATTCGAAAGCGT

GGGTAGCAAA

>ASV838 GS|99.2|AB594446_S002949232;k:Bacteria,p:"Actinobacteria",c:Actinobacteria,o:Actinomycetales,f:Dermacoccaceae,g:Branchiibius;

TACGTAGGGTGCGAGCGTTGTCCGGAATTATTGGGCGTAAAGAGCTTGTAGGCGGTTTGT

CGCGTCTGCTGTGAAAGCCCGGGGCTTAACTCCGGGTCTGCAGTGGGTACGGGCAGGCTA

GAGTATGGTAGGGGAGACTGGAATTCCTGGTGTAGCGGTGAAATGCGCAGATATCAGGAG

GAACACCGATGGCGAAGGCAGGTCTCTGGGCCATTACTGACGCTGAGAAGCGAAAGCATG

GGGAGCAAAC

>ASV839 SS|1.0000|EF067920_S001020492;k:Bacteria,p:Cyanobacteria/Chloroplast,c:Chloroplast,f:Chloroplast,g:Bacillariophyta

GACGGGGGATGCAAGTGTTATCCGGAATCACTGGGCGTAAAGCGTCTGTAGGTGGTTAAA

TAAGTCAACTGTTAAATCTTGAGGCTTAACTTCAAAATCGCAGTCGAAACTGTTTGACTA

GAGTATAGTAGGGGTAAAGGGAATTTCCAGTGGAGCGGTGAAATGCGTAGATATTGGAAA

GAACACCGATGGCGAAGGCACTTTACTGGGCTATTACTAACACTGAGAGACGAAAGCTAG

GGTAGCAAAT

>ASV840 SS|1.0000|AB015062_S000012231;k:Bacteria,p:Cyanobacteria/Chloroplast,c:Cyanobacteria,f:Family_IV,g:GpIV

GACGGAGGATGCAAGCGTTATCCGGAATTATTGGGCGTAAAGCGTCCGTAGGTGGTATAG

AAAGTCTGTTGTTAAATCCCACAGCTCAACTGTGGATCGGCAATGGAAACTACTAAACTT

GAGTGTGGTAGGGGTAGAGGGAATTCCTAGTGTAGCGGTGAAATGCGTAGATATTAGGAA

GAACACCAGTGGCGAAGGCGCTCTACTGGGCCATAACTGACACTGATGGACGAAAGCTAG

GGGAGCGAAA

>ASV841 SS|1.0000|JQ819258_S003292119;k:Bacteria,p:"Actinobacteria",c:Actinobacteria,o:Actinomycetales

TACGTAGGGTGCAAGCGTTGTCCGGAATTATTGGGCGTAAAGAGCTCGTAGGTGGTCTGT

TGCGTCGGCTGTGAAAACCCGAGGCTCAACCTCGGGCCTGCAGCCGATACGGGCAGACTA

GAGTTCGGCAGGGGAGACTGGAATTCCTGGTGTAGCGGTGAAATGCGCAGATATCAGGAG

GAACACCGGTGGCGAAGGCGGGTCTCTGGGCCGATACTGACACTGAGGAGCGAAAGCGTG

GGGAGCGAAC

>ASV842 SS|0.9400|AM231587_S000650722;k:Bacteria,p:"Proteobacteria",c:Alphaproteobacteria,o:Rhodospirillales,f:Acetobacteraceae,g:Roseomonas

TACGAAGGGGGCTAGCGTTGCTCGGAATTACTGGGCGTAAAGGGCGCGTAGGCGGCACGA

TTAGTCAGGCGTGAAATTCCTGGGCTCAACCTGGGGACTGCGCTTGATACGGTCGAGCTA

GAGGATGGAAGAGGGTCGCGGAATTCCCAGTGTAGAGGTGAAATTCGTAGATATTGGGAA

GAACACCGGTGGCGAAGGCGGCGACCTGGTCCATTACTGACGCTGAGGCGCGACAGCGTG

GGGAGCAAAC

>ASV843 GS|78.4|EF515236_S000839820;k:Bacteria,p:"Armatimonadetes",c:Chthonomonadetes,o:Chthonomonadales,f:Chthonomonadaceae,g:Chthonomonas/Armatimonadetes_gp3;

TACGTAGGTGGCAAGCGTTGTCCGGATTTACTGGGCGTAAAGCGAACGCAGGCGGACTGT

TAAGTAGGAAGTGAAAGGTTGCAGCTCAACTGCGACACTGCTTCTTATACTGGCCGTCTT

GAGTAGCGGAGAGGGAGATGGAACGACACGTGTAGCGGTGAAATGCGTTGATATGTGTCG

GAACACCAATGGCGAAAGCAATCTCCTGGACGCAGACTGACGCTGAGGTTCGAAAGCCAA

GGTAGCAAAC

>ASV844 GS|98.0|AM229669_S000643543;k:Bacteria,p:"Proteobacteria",c:Alphaproteobacteria,o:Sphingomonadales,f:Sphingomonadaceae,g:Sphingomonas;

TACGGAGGGAGCTAGCGTTATTCGGAATTACTGGGCGTAAAGCGCACGTAGGCGGCTTTG

TAAGTTAGAGGTGAAAGCCCAGAGCTCAACTCTGGAATTGCCTTTAAGACTGCATCGCTC

GAATCCAGGAGAGGTGAGTGGAATTCCGAGTGTAGAGGTGAAATTCGTAGATATTCGGAA

GAACACCAGTGGCGAAGGCGGCTCACTGGACTGGTATTGACGCTGATGTGCGAAAGCGTG

GGGAGCAAAC

>ASV845 SS|0.9600|AB360448_S001014085;k:Bacteria,p:"Actinobacteria",c:Actinobacteria,o:Acidimicrobiales,f:Iamiaceae,g:Iamia

CACGTAGGGGGCAAGCGTTGTCCGGATTTATTGGGCGTAAAGGGCTCGTAGGCGGTTCGA

CAAGTCGGGTGTGAAACCTCCAGGCTCAACCTGGAGTCGCCGCTCGAAACTGTTGTGACT

AGAGTCCGGTAGAGGAGCATGGAATTTGTGGTGTAGCGGTGGAATGCGCAGATATCACAA

GGAACACCAGTAGCGAAGGCGATGCTCTGGGCCGGTACTGACGCTGAGGAGCGAAAGCAT

GGGGAGCGAA

>ASV846 GSL|99.6|X77434_S000003090;k:Bacteria,p:"Actinobacteria",c:Actinobacteria,o:Actinomycetales,f:Microbacteriaceae

TACGTAGGGTGCAAGCGTTGTCCGGAATTATTGGGCGTAAAGAGCTCGTAGGCGGTTTGT

CGCGTCTGCTGTGAAATCCCGAGGCTCAACCTCGGGTCTGCAGTGGGTACGGGCAAACTA

GAGTGCGGTAGGGGAGATTGGAATTCCTGGTGTAGCGGTGGAATGCGCAGATATCAGGAG

GAACACCGATGGCGAAGGCAGATCTCTGGGCCGTAACTGACGCTGAGGAGCGAAAGCATG

GGGAGCGAAC

>ASV847 GS|0.0|None;No hit

TTCCAGCTCCAATAGCGTATATTAAAGTTGTTGCAGTTAAAAAGCTCGTAGTTGAAACTT

GGGCCTGGCTGGCCGGTCCGCCTCACCGCGTGCACTGGTCCGGCCGGGCCTTTCCTTCTG

GGGAGCCGCATGCCCTTCATTGGGTGTGCCGGGGAACCAGGACTTTTACTTTGAAAAAAT

TAGAGTGTTCAAAGCAGGCCTATGCTCGAATACATTAGCATGGAATAATAGAATAGGACG

TGTGGTTCTA

>ASV848 SS|1.0000|AB245334_S000627870;k:Bacteria,p:"Actinobacteria",c:Actinobacteria,o:Solirubrobacterales,f:Solirubrobacteraceae,g:Solirubrobacter

TACGTAGGGGGCTAGCGTTGTCCGGAATTATTGGGCGTAAAGCGCGTGTAGGCGGTTCAG

TAAGTCCGCTGTGAAAGTCGGGGGCTCAACCCTCGAATGCCGGTGGATACTGTTGGACTA

GAGTGCGGAAGAGGCGAGTGGAATTCCTGGTGTAGCGGTGAAATGCGCAGATATCAGGAG

GAACACCAATTGCGAAGGCAGCTCGCTGGGACGTTACTGACGCTGAGACGCGAAAGCGTG

GGGAGCAAAC

>ASV849 GS|100.0|EU888308_S001169258;k:Bacteria,p:"Proteobacteria",c:Betaproteobacteria,o:Burkholderiales,f:Comamonadaceae,g:Delftia;

TACGTAGGGTGCGAGCGTTAATCGGAATTACTGGGCGTAAAGCGTGCGCAGGCGGTTATG

TAAGACAGATGTGAAATCCCCGGGCTCAACCTGGGAACTGCATTTGTGACTGCATGGCTA

GAGTACGGTAGAGGGGGATGGAATTCCGCGTGTAGCAGTGAAATGCGTAGATATGCGGAG

GAACACCGATGGCGAAGGCAATCCCCTGGACCTGTACTGACGCTCATGCACGAAAGCGTG

GGGAGCAAAC

>ASV850 GS|100.0|AM600682_S000858506;k:Bacteria,p:Firmicutes,c:Bacilli,o:Lactobacillales,f:Leuconostocaceae,g:Leuconostoc;

TACGTATGTCCCGAGCGTTATCCGGATTTATTGGGCGTAAAGCGAGCGCAGACGGTTGAT

TAAGTCTGATGTGAAAGCCCGGAGCTCAACTCCGGAATGGCATTGGAAACTGGTTAACTT

GAGTGTTGTAGAGGTAAGTGGAACTCCATGTGTAGCGGTGGAATGCGTAGATATATGGAA

GAACACCAGTGGCGAAGGCGGCTTACTGGACAACAACTGACGTTGAGGCTCGAAAGTGTG

GGTAGCAAAC

>ASV851 SS|1.0000|AB362219_S001043921;k:Bacteria,p:"Proteobacteria",c:Alphaproteobacteria,o:Rhodospirillales,f:Acetobacteraceae

TACGAAGGGGGCTAGCGTTGCTCGGAATGACTGGGCGTAAAGGGCGCGTAGGCGGCTTGT

ACAGTCAGATGTGAAATTCCTGGGCTCAACCTGGGGACTGCATTTGATACGTGCGGGCTA

GAGTTTGGAAGAGGGTCGTGGAATTCCCAGTGTAGAGGTGAAATTCGTAGATATTGGGAA

GAACACCGGTGGCGAAGGCGGCGACCTGGTCCTTGACTGACGCTGAGGCGCGAAAGCGTG

GGGAGCAAAC

>ASV852 GS|0.0|None;No hit

GTGTCAGCAGCCGCCCCCTCAGCCCACTCTACCCTCCTTCCCAGAAGACGACTGGATCAA

TGAGGATATCTATAATACAGGAGACATGTTCAGTGGCGAGGAAGGATTGGATCTTGACTT

CTTCGACTTCTCACACGGGAATCCTCAGGCCTCGCAGCAAGCTCTAATCCAGGTCGATGA

CTGCGACCGACCATTACTGGATCATTTCATCGACAACGTTTTACGGTTGATCTTCCCAAT

TCTCGAAGTC

>ASV853 SS|1.0000|AY743256_S000595378;k:Bacteria,p:"Deinococcus-Thermus",c:Deinococci,o:Deinococcales,f:Deinococcaceae,g:Deinococcus

TACGGAGGGTGCAAGCGTTACCCGGAATCACTGGGCGTAAAGGGCGTGTAGGCGGCGCAC

CAAGTCTGGTTTTAAAGCCCGCAGCTCAACTGCGGAGATGGACTGGAGACTGGTGTGCTT

GACCTCTGGAGAGGAAGCTGGAATTCCTGGTGTAGCGGTGGAATGCGTAGATACCAGGAG

GAACACCAATGGCGAAGGCAGGCTTCTGGACAGAAGGTGACGCTGAGGCGCGAAAGTGTG

GGGAGCGAAC

>ASV854 SS|1.0000|EF516412_S000840854;k:Bacteria,p:"Armatimonadetes",c:Armatimonadia,o:Armatimonadales,f:Armatimonadaceae,g:Armatimonas/Armatimonadetes_gp1

TACGTAGGGGGCCAGCGTTGTCCGAAGTTACTGGGCGTAAAGAGCGCGTAGGCGGACTCT

TAAGTGAGGGGTGAAAGGTTCAGGGCTTAACCCGGACACTGCCTTTCATACTGGGAGTCT

TGAGTGTTGGAGAGGCGAGTGGAATGGTCGGTGTAGCGGTGAAATGCGTAGATATCGATC

GGAACACCCATGGCGAAGGCAGCTCGCTGGCCAACAACTGACGCTGAGGCGCGAAAGCGT

GGGGAGCAAA

>ASV855 SS|1.0000|GQ423055_S001589422;k:Bacteria

TACGTAGGTGGCAAGCGTTGTCCGGATTTACTGGGCGTAAAGCGTGCGCAGGCGGGCGTT

TAAGTAGAAAGTGAAAGGTTGGGGCTCAACCCTGACACTGCTTCCTATACTGGACGCCTT

GAGTAGCGGAGAGGAAAGCGGAACGACACGTGTAGCGGTGAAATGCGTTGAGATGTGTCG

GAACACCAATGGCGAAAGCAGCTTTCTGGACGCAAACTGACGCTCATGCACGAAAGCCAA

GGTAGCGAAC

>ASV856 GS|0.0|None;No hit

GGCGAAATTCGACTATAGGCTTAACGACGCCCCACCAGTCCCTATTCCGAATCTCGCGAA

AGACTTCAGCGCTGGTGATTCGTATCATACTCCCACCGAATCTGTCTCCTCCAGTGGCTC

CTATGGGTCAGATGCTAAAAGCGGAAGCTCACGGTCAAGCCCACCCTTATCCGATGCATC

GACACGGCCACTTACAAATTCATTCAACGTAGACCATGTTGGTAACTCAAGGACTGACGT

CCACTCATCT

>ASV857 SS|1.0000|AB030585_S000469290;k:Bacteria,p:"Actinobacteria",c:Actinobacteria,o:Actinomycetales

TACGTAGGGTGCAAGCGTTGTCCGGAATTATTGGGCGTAAAGAGCTCGTAGGTGGCCTGT

CGCGTCGGCTGTGAAAACTCAGGGCTCAACCCTGAGCCTGCAGTCGATACGGGCAGGCTA

GAGTCCGGCAGGGGAGACTGGAATTCCTGGTGTAGCGGTGAAATGCGCAGATATCAGGAG

GAACACCGGTGGCGAAGGCGGGTCTCTGGGCCGGAACTGACACTGAGGAGCGAAAGCGTG

GGGAGCGAAC

>ASV858 GS|100.0|KC843488_S004044709;k:Bacteria,p:"Proteobacteria",c:Gammaproteobacteria,o:Pseudomonadales,f:Moraxellaceae,g:Acinetobacter;

TACAGAGGGTGCAAGCGTTAATCGGATTTACTGGGCGTAAAGCGTGCGTAGGTGGCCAAT

TAAGTCAAATGTGAAATCCCCGAGCTTAACTTGGGAATTGCATTCGATACTGGTTGGCTA

GAGTATGGGAGAGGATGGTAGAATTCCAGGTGTAGCGGTGAAATGCGTAGAGATCTGGAG

GAATACCGATGGCGAAGGCAGCCATCTGGCCTAATACTGACACTGAGGTACGAAAGCATG

GGGAGCAAAC

>ASV859 SS|1.0000|AB267478_S000721192;k:Bacteria,p:"Bacteroidetes",c:Sphingobacteriia,o:"Sphingobacteriales",f:Chitinophagaceae

TACGGAGGGTGCAAGCGTTATCCGGATTCACTGGGTTTAAAGGGTGCGTAGGTGGGGATG

TAAGTCAGTGGTGAAATCTCCGTGCTTAACACGGAAACTGCCATTGATACTATGTTTCTT

GAATTATCTGGAGGTCAGCGGAATATGTCATGTAGCGGTGAAATGCTTAGATATGACATA

GAACACCAATTGCGAAGGCAGCTGGCTACAGGTGGATTGACACTGAGGCACGAAAGCGTG

GGGATCAAAC

>ASV860 SS|0.9800|EU401905_S001588054;k:Bacteria,p:"Proteobacteria",c:Alphaproteobacteria,o:Rhizobiales

TACGAAGGGGGCTAGCGTTGTTCGGATTAACTGGGCGTAAAGGGCGCGTAGGCGGGTTTT

TAAGTCAGGGGTGAAATCCCGAGGCTCAACCTCGGAACTGCCTTTGATACTGGAAATCTT

GAGTCCGGGAGAGGTGAGTGGAACTGCGAGTGTAGAGGTGAAATTCGTAGATATTCGCAA

GAACACCAGTGGCGAAGGCGGCTCACTGGCCCGGTACTGACGCTGAGGCGCGAAAGCGTG

GGGAGCAAAC

>ASV861 SS|1.0000|FJ842646_S001352336;k:Bacteria,p:"Actinobacteria",c:Actinobacteria,o:Actinomycetales

TACGTAGGGTGCAAGCGTTGTCCGGAATCATTGGGCGTAAAGGGCTCGTAGGCGGTCCAT

CGCGTCGGGAGTGAAAAGCTGGGGCTCAACCCCAGTCCTGCTTCCGATACGGGTGGACTA

GAGGTAGGCAGGGGAGAACGGAATTCCTGGTGTAGCGGTGAAATGCGCAGATATCAGGAG

GAACACCGGTGGCGAAGGCGGTTCTCTGGGCCTTACCTGACGCTGAGGAGCGAAAGCGTG

GGGAGCAAAC

>ASV862 SS|1.0000|KF999686_S004084195;k:Bacteria,p:"Bacteroidetes",c:Cytophagia,o:Cytophagales,f:Cytophagaceae,g:Spirosoma

TACGGAGGGTGCGAGCGTTGTCCGGATTTATTGGGTTTAAAGGGTGCGTAGGTGGGTGGC

TAAGTCTGGTTTGAAAGCAGGTGGCTCAACCATCTGATGTGGCTGGAAACTGGTCATCTT

GAATGGGTTGGCGGTTGCCGGAACGGGTCATGTAGCGGTGAAATGCATAGATATGACCCA

GAACACCGATTGCGAAGGCAGGCAACTAGGACTTGATTGACACTGAGGCACGAGAGCCGG

GGTAGCGAAC

>ASV863 GS|98.8|DQ660892_S000712490;k:Bacteria,p:"Acidobacteria",c:Acidobacteria_Gp1,g:Terriglobus;

TACGAGGGGGGCAAGCGTTGTTCGGAATTATTGGGCGTAAAGGGCGCGTAGGCGGTTTGG

CAAGTTTGGTGTGAAATCTTCGGGCTCAACTCGAAGTCTGCATCGAAAACTGCCGGGCTT

GAGTATGGGAGAGGTGAGTGGAATTTCCGGTGTAGCGGTGAAATGCGTAGATATCGGAAG

GAACACCTGTGGCGAAAGCGGCTCACTGGACCATTACTGACGCTGAGGCGCGAAAGCTAG

GGGAGCAAAC

>ASV864 SS|1.0000|JN090860_S002916046;k:Bacteria,p:"Bacteroidetes",c:Cytophagia,o:Cytophagales,f:Cytophagaceae,g:Hymenobacter

TACGGAGGGTGCGAGCGTTGTCCGGATTTATTGGGTTTAAAGGGTGCGTAGGCGGCCGTT

TAAGTCTGGGGTGAAAGCCCGCTGCTCAACAGCGGAACGGCCCTGGATACTGGATGGCTT

GAGTACAGACGAGGTTGGCGGAATGGACCGAGTAGCGGTGAAATGCATAGATACGGTCCA

GAACCCCGATTGCGAAGGCAGCTGACTAGGCTGCTACTGACGCTGAGGCACGACAGCGTG

GGGAGCGAAC

>ASV865 SS|1.0000|D86513_S000011943;k:Bacteria,p:"Proteobacteria",c:Alphaproteobacteria,o:Rhodospirillales,f:Acetobacteraceae

TACGAAGGGGGCTAGCGTTGCTCGGAATGACTGGGCGTAAAGGGCGCGTAGGCGGATCAT

GTAGTCAGATGTGAAATTCCTGGGCTCAACCTGGGGACTGCATTTGATACGCGTGGTCTA

GAGTTTGGAAGAGGGTCGTGGAATTCCCAGTGTAGAGGTGAAATTCGTAGATATTGGGAA

GAACACCGGTGGCGAAGGCGGCGACCTGGTCCTTGACTGACGCTGAGGCGCGAAAGCGTG

GGGAGCAAAC

>ASV866 GS|0.0|None;No hit

GTGTCAGCAGCCGCGGTTTCGGGATGCGATTCGGCCTTTTCTCTCTGCTACTCATTCGCG

AGTTGAGCTACTACCTTGTCACACAATACTTTTGCCTCGTCATCAGCATCATCTTCACCC

AATTTCCCAAATCACCCAGCGCACTGGTTCGCTTCATCTTCTTCCCATACCCGGTCTCTG

AGTGGTTTTTCATTATCAGGTACGTTACAATTCCTTTGTATCCCTGCTATTCAAGAGCAT

ACTTCTAACG

>ASV867 GS|100.0|EF380353_S001020397;k:Bacteria,p:Cyanobacteria/Chloroplast,c:Chloroplast,f:Chloroplast,g:Streptophyta;

GACAGAGGATGCAAGCGTTATCCGGAATGATTGGGCGTAAAGCGTCTGTAGGTGGCTTTT

CAAGTCCGCCGTCAAATCCCAGGGCTCAACCCTGGACAGGCGGTGGAAACTACCAAGCTG

GAGTACGGTAGGGGCAGAGGGAATTTCCGGTGGAGCGGTGAAATGCGTAGAGATCGGAAA

GAACACCAACGGCGAAAGCACTCTGCTGGGCCGACACTGACACTGAGAGACGAAAGCTAG

GGGAGCAAAT

>ASV868 GS|97.2|AB267478_S000721192;k:Bacteria,p:"Bacteroidetes",c:Sphingobacteriia,o:"Sphingobacteriales",f:Chitinophagaceae,g:Segetibacter;

TACGGAGGGTGCAAGCGTTATCCGGATTCACTGGGTTTAAAGGGTGCGTAGGTGGGAATG

TAAGTCAGTGGTGAAATCTCCGTGCTTAACATGGAAACTGCCATTGATACTATATTTCTT

GAATTTTCTGGAGGTAAGCGGAATATGTCATGTAGCGGTGAAATGCTTAGATATGACATA

GAACACCAATTGCGAAGGCAGCTTACTACAGGAACATTGACACTAAGGCACGAAAGCGTG

GGGATCAAAC

>ASV869 SS|0.8300|FJ177532_S001188354;k:Bacteria,p:"Bacteroidetes",c:Sphingobacteriia,o:"Sphingobacteriales",f:Chitinophagaceae,g:Ferruginibacter

TACGGAGGGTGCAAGCGTTATCCGGATTTACTGGGTTTAAAGGGTGCGTAGGTGGGTCTG

TAAGTCAGTGGTGAAATCTCCGAGCTTAACTCGGAAACTGCCATTGATACTATAGGTCTT

GAATTATCTGGAGGTAAGCGGAATATGTCATGTAGCGGTGAAATGCTTAGATATGACATA

GAACACCAATTGCGAAGGCAGCTTACTACGGATTGATTGACACTGAGGCACGAAAGCGTG

GGGATCAAAC

>ASV870 GS|97.5|AY043938_S000354314;k:Bacteria,p:"Verrucomicrobia",c:Spartobacteria,g:Spartobacteria_genera_incertae_sedis;

TACAGAGGTCTCAAGCGTTGTTCGGATTCATTGGGCGTAAAGGGTGCGTAGGTGGCGCCA

TAAGTCGGGTGTGAAATTTCGGAGCTCAACTCCGAAACTGCATTCGATACTGTGGTGCTT

GAGGACTGGAGAGGAGACTGGAATTCACGGTGTAGCAGTGAAATGCGTAGAGATCGTGAG

GAAGACCAGTGGCGAAGGCGGGTCTCTGGACAGTTCCTGACACTGAGGCACGAAGGCTAG

GGGAGCAAAC

>ASV871 SS|1.0000|HM032897_S002167665;k:Bacteria,p:"Bacteroidetes",c:Cytophagia,o:Cytophagales,f:Cytophagaceae,g:Hymenobacter

TACGGAGGGTGCGAGCGTTGTCCGGATTTATTGGGTTTAAAGGGTGCGTAGGCGGCCGTT

TAAGTCTGGGGTGAAAGCCCGCTGCTCAACAGCGGAACTGCCCTGGATACTGGATGGCTT

GAATACAGTGGAGGTTGGCGGAATGGACTGAGTAGCGGTGAAATGCATAGATACAGTCCA

GAACCCCGATTGCGAAGGCAGCTGACTACACTGGTATTGACGCTCAGGCACGACAGCGTG

GGGAGCGAAC

>ASV872 SS|1.0000|AB072735_S000251887;k:Bacteria,p:"Gemmatimonadetes",c:Gemmatimonadetes,o:Gemmatimonadales,f:Gemmatimonadaceae,g:Gemmatimonas

TACGGAGGGTGCGAGCGTTGTCCGGAATCACTGGGCGTAAAGGGCGCGTAGGCGGTCTGT

TAAGCGTGCGGTGAAAGCCTGGGGCTCAACCCCAGGTCTGCCGTGCGAACTGGTGGACTG

GAGCACTGTAGAGGCAGGTGGAATTCCGGGTGTAGCGGTGGAATGCGTAGAGATCCGGAA

GAACACCAGTGGCGAAGGCGGCCTGCTGGGCAGTTGCTGACGCTGAGGCGCGACAGCGTG

GGGAGCAAAC

>ASV873 SS|0.8500|EF368368_S000806277;k:Bacteria,p:"Proteobacteria",c:Alphaproteobacteria,o:Rhodospirillales,f:Acetobacteraceae,g:Roseomonas

TACGAAGGGGGCTAGCGTTGCTCGGAATTACTGGGCGTAAAGGGCGCGTAGGCGGCATGG

TCAGTCAGGCGTGAAATCCCGGGGCTCAACCTCGGGGCTGCGCTTGATACGGCTGCGCTA

GAGGGTAGGAGAGGGTCGTGGAATTCCCAGTGTAGAGGTGAAATTCGTAGATATTGGGAA

GAACACCGGTGGCGAAGGCGGCGACCTGGCCTATTACTGACGCTGAGGCGCGACAGCGTG

GGGAGCAAAC

>ASV874 GS|98.0|HM032898_S002167666;k:Bacteria,p:"Bacteroidetes",c:Cytophagia,o:Cytophagales,f:Cytophagaceae,g:Hymenobacter;

TACGGAGGGTGCGAGCGTTGTCCGGATTTATTGGGTTTAAAGGGTGCGTAGGCGGCTTTT

TAAGTCCGGGGTGAAAGCCCGTTGCTCAACAACGGAACTGCCCTGGATACTGGGGAGCTT

GAGTACAGACGAGGTTGGCGGAATGGACCGAGTAGCGGTGAAATGCATAGATACGGTCCA

GAACCCCGATTGCGAAGGCAGCTGACTAGGCTGTTACTGACGCTGAGGCACGACAGCGTG

GGGAGCGAAC

>ASV875 SS|1.0000|AF166114_S000498654;k:Bacteria,p:Cyanobacteria/Chloroplast

GACGGAGGATGCAAGCGTTATCCGGAATGATTGGGCGTAAAGCGTCTGTAGGTGGGTTGT

GAAGTCTTCTGTTAAAGATCAGGGCTTAACCCTGTTTAGGCAGTGGAAACTCATAACCTA

GAGTACGGTAGGGGCAGAGGGAATTCCCGGTGTAGCGGTGAAATGCGTAGATATCGGGAA

GAACACCGACGGCGAAAGCACTCTGCTGGGCCGAAACTGACACTGAGAGACGAAAGCTAG

GGGAGCAAAC

>ASV876 SS|1.0000|EF661571_S000893309;k:Bacteria,p:"Proteobacteria",c:Alphaproteobacteria,o:Rhodospirillales,f:Acetobacteraceae

TACGAAGGGGGCAAGCGTTGCTCGGAATGACTGGGCGTAAAGGGCGCGTAGGCGGCTCTG

TTTGTCAGGCGTGAAAGTCCTGGGCTCAACCTGGGGATTGCGCTTGGGACGGCGGGGCTA

GAGTGGGGAAGAGGGTCGTGGAATTCCCAGTGTAGAGGTGAAATTCGTAGATATTGGGAA

GAACACCGGTGGCGAAGGCGGCGACCTGGTCCTTTGACTGACGCTGAGGCGCGAGAGCGT

GGGGAGCAAA

>ASV877 SS|0.9500|AB245334_S000627870;k:Bacteria,p:"Actinobacteria",c:Actinobacteria,o:Solirubrobacterales,f:Solirubrobacteraceae,g:Solirubrobacter

TACGTAGGGGGCTAGCGTTGTCCGGAATTATTGGGCGTAAAGCGCGTGTAGGCGGTTCGA

TAAGTCTGCTGTGAAAGTCGGGGGCTCAACCCTCGAAAGCCGGTGGATACTGTCGGACTA

GAGTGCGGAAGAGGCGAGTGGAATTCCTGGTGTAGCGGTGAAATGCGCAGATATCAGGAG

GAACACCAATTGCGAAGGCAGCTCGCTGGGACGTTACTGACGCTGAGACGCGAAAGCGTG

GGGAGCAAAC

>ASV878 GSL|99.6|EF553529_S000860302;k:Bacteria,p:"Actinobacteria",c:Actinobacteria,o:Actinomycetales,f:Intrasporangiaceae

TACGTAGGGTGCGAGCGTTGTCCGGAATTATTGGGCGTAAAGAGCTTGTAGGCGGTTTGT

CGCGTCTGCTGTGAAAATCCGGGGCTCAACCCCGGACTTGCAGTGGGTACGGGCAGACTA

GAGTGTGGTAGGGGAGACTGGAATTCCTGGTGTAGCGGTGGAATGCGCAGATATCAGGAG

GAACACCGATGGCGAAGGCAGGTCTCTGGGCCATTACTGACGCTGAGAAGCGAAAGCATG

GGGAGCGAAC

>ASV879 SS|1.0000|D30775_S000413730;k:Bacteria,p:"Proteobacteria",c:Alphaproteobacteria,o:Rhodospirillales,f:Acetobacteraceae

TACGAAGGGGGCTAGCGTTGCTCGGAATGACTGGGCGTAAAGGGCGCGTAGGCGGATGGC

ATAGTCAGACGTGAAATTCCTGGGCTCAACCTGGGGGCTGCGTTTGATACGTGTCGTCTA

GAGTTTGGAAGAGGGTTGTGGAATTCCCAGTGTAGAGGTGAAATTCGTAGATATTGGGAA

GAACACCGGTGGCGAAGGCGGCAACCTGGTCCTTGACTGACGCTGAGGCGCGAAAGCGTG

GGGAGCAAAC

>ASV880 GS|0.0|None;No hit

GTGTCAGCAGCCAAATTACCAGATACTTTACTCCCCAAAGGCTGCTGTTTCAATGCCGCA

ATGTTGTCAAATGCGAAGGTCTCGTCCATAATCCGAGAGGATATTCCCAGGTCATCCTCA

TCGCTTTCGGAGGAGTAATCACTGCCCTCTTCGGCCATAGCATAGCGTCGCCTAGGAGAA

TCCTCGTCCGGGGCCTTGAACTGAACGCTGGCGATCCCGAGGAGGGCTTTTGCAACCTCG

AAATGGCGCC

>ASV881 GS|98.8|AF131295_S000387421;k:Bacteria,p:"Proteobacteria",c:Alphaproteobacteria,o:Sphingomonadales,f:Sphingomonadaceae,g:Sphingomonas;

TACGGAGGGAGCTAGCGTTGTTCGGAATTACTGGGCGTAAAGCGCACGTAGGCGGCTTTG

TAAGTTAGAGGTGAAAGCCCGGGGCTCAACTCCGGAACTGCCTTTAAGACTGCATCGCTT

GAATCCAGGAGAGGTGAGTGGAATTCCGAGTGTAGAGGTGAAATTCGTAGATATTCGGAA

GAACACCAGTGGCGAAGGCGGCTCACTGGACTGGTATTGACGCTGAGGTGCGAAAGCGTG

GGGAGCAAAC

>ASV882 SS|0.9600|KC560021_S003719248;k:Bacteria,p:"Bacteroidetes",c:Sphingobacteriia,o:"Sphingobacteriales",f:Chitinophagaceae

TACGGAGGGTGCAAGCGTTATCCGGATTTACTGGGTTTAAAGGGTGTGTAGGCGGGCTAT

TTAAGTCCGTGGTGAAATCTCCGGGCTCAACCCGGAAACTGCCATGGATACTATTAGTCT

TGAATACTCTGGAGGTGGGCGGAACGGGTCATGTAGCGGTGAAATGCATAGATATGACCC

GGAACACCGATTGCGAAGGCAGCCCACTACAGAGTGATTGACGCTGAGGCACGAAAGCGT

GGGGATCAAA

>ASV883 GS|99.2|EF363714_S000806231;k:Bacteria,p:"Proteobacteria",c:Alphaproteobacteria,o:Sphingomonadales,f:Sphingomonadaceae,g:Sphingomonas;

TACGGAGGGAGCTAGCGTTGTTCGGAATTACTGGGCGTAAAGCGCACGTAGGCGGCCTTG

TAAGTTAGAGGTGAAAGCCTGGAGCTCAACTCCAGAATTGCCTTTAAGACTGCATCGCTT

GAATCCAGGAGAGGTGAGTGGAATTCCGAGTGTAGAGGTGAAATTCGTAGATATTCGGAA

GAACACCAGTGGCGAAGGCGGCTCACTGGACTGGTATTGACGCTGAGGTACGAAAGCGTG

GGGAGCAAAC

>ASV884 GS|0.0|None;No hit

GTGTCAGCAGCCGCGAGTTACGCCTCTGAAGTATCCCAAAGTTCTAGTCCCCAGGCATGA

TAAACGCTCTTATGAATAATCCCTGGCAGCGACTGCCTTTGCAATAACACCAGCAAATCT

ACCCGCCATATCCTGGTCTTGCTTAGCAGTCTCTATGCCAAGCAGAATCTTCTTAATCCC

AACGCCGACTTTCTGATCGCTCCTCGTAGTCC

>ASV885 GS|0.0|None;No hit

GTGTCAGCAGCCGCGTCGAGCAAAGCACTAAAACTAAGCGCAGAAGCTCATCACACAGTC

GAAGCGAACATCTGTCGAAGTGGCTCGATGAAGCGGTCTCTGCGGACGAAGGCAGCCCTT

CGCGTCATCGCAGCAACCGCCAACTGTCTCAAAGAGCTAGACGCTCGTCACCCAAGCCAG

CGTTTCCTGACCAGTTCTCCTCCATTGGAGAAGTACTCCATAAGCCCAAGCCCAACGCGC

TCG

>ASV886 GSL|100.0|AF177667_S000388693;k:Bacteria,p:"Proteobacteria",c:Betaproteobacteria,o:Burkholderiales,f:Alcaligenaceae,g:Achromobacter

TACGTAGGGTGCAAGCGTTAATCGGAATTACTGGGCGTAAAGCGTGCGCAGGCGGTTCGG

AAAGAAAGATGTGAAATCCCAGAGCTTAACTTTGGAACTGCATTTTTAACTACCGGGCTA

GAGTGTGTCAGAGGGAGGTGGAATTCCGCGTGTAGCAGTGAAATGCGTAGATATGCGGAG

GAACACCGATGGCGAAGGCAGCCTCCTGGGATAACACTGACGCTCATGCACGAAAGCGTG

GGGAGCAAAC

>ASV887 GS|100.0|M58825_S000436416;k:Bacteria,p:Firmicutes,c:Bacilli,o:Lactobacillales,f:Carnobacteriaceae,g:Carnobacterium;

TACGTAGGTGGCAAGCGTTGTCCGGATTTATTGGGCGTAAAGCGAGCGCAGGCGGTTCTT

TAAGTCTGATGTGAAAGCCCCCGGCTCAACCGGGGAGGGTCATTGGAAACTGGAGAACTT

GAGTGCAGAAGAGGAGAGTGGAATTCCACGTGTAGCGGTGAAATGCGTAGATATGTGGAG

GAACACCAGTGGCGAAGGCGACTCTCTGGTCTGTAACTGACGCTGAGGCTCGAAAGCGTG

GGGAGCAAAC

>ASV888 GS|0.0|None;No hit

GTGTCAGCAGCCGCCCATTCCCAGATATGGGTCGCGATCTCATACTCAGTGTACCCCTCT

TCATCGAGAAGGTCAAAGACGATTTCAAGATCTAGGACCATCCCGACCAGATGATCACAG

ATGAACGTCAGGGTTTCATCTAAAGTACAAGCCCCAGTGTGGTAAGACTTGACATACTGT

GAAAGCTCTTGGATGATCTCTCGCTCGTCTGCGTCTGGCTCCGCAATCTCAGGCTTTTGC

AGCTGACCAT

>ASV889 GS|98.4|EF532793_S000859919;k:Bacteria,p:"Proteobacteria",c:Betaproteobacteria,o:Burkholderiales,f:Comamonadaceae,g:Hydrogenophaga;

TACGTAGGGTGCAAGCGTTAATCGGAATTACTGGGCGTAAAGCGTGCGCAGGCGGTTTTG

TAAGACAGGTGTGAAATCCCCGGGCTTAACCTGGGAATTGCATTTGTGACTGCAAGGCTG

GAGTGCGGCAGAGGGGGATGGAATTCCGCGTGTAGCAGTGAAATGCGTAGATATGCGGAG

GAACACCGATGGCGAAGGCAATCCCCTGGGCCTGCACTGACGCTCATGCACGAAAGCGTG

GGGAGCAAAC

>ASV890 GS|98.8|EF457479_S000834904;k:Bacteria,p:"Acidobacteria",c:Acidobacteria_Gp6,g:Gp6;

TACGGGGGGGGCAAGCGTTGTTCGGAATTACTGGGCGTAAAGGGCTCGTAGGTGGCCAAC

TAAGTCAGACGTGAAATCCCTCGGCTTAACCGGGGAACTGCGTCTGATACTGGATGGCTT

GAGTTCGGGAGAGGGATGCGGAATTCCAGGTGTAGCGGTGAAATGCGTAGATATCTGGAG

GAACACCGGTGGCGAAGGCGGCATCCTGGACCGAGACTGACACTGAGGAGCGAAAGCCAG

GGGAGCAAAC

>ASV891 SS|1.0000|AJ009456_S000115949;k:Bacteria

TACGTAGGTGGCAAGCGTTGTCCGGATTTACTGGGCGTAAAGCGCGCGCAGGCGGACTGT

TAAGTAGAAAGTGAAAGGTTGGAGCTCAACTCCAACATTGCTTCCTATACTGGCAGTCTT

GAGTCCCGGAGGGGAGAGCGGAACAATATGTGTAGCGGTGAAATGCGTTGATATGTATTG

GAACACCAATGGCGAAGGCAGCTCTCTGGACGGGAACTGACGCTCAGGCGCGAAAGCCGA

GGTAGCGAAC

>ASV892 GS|97.2|JN090860_S002916046;k:Bacteria,p:"Bacteroidetes",c:Cytophagia,o:Cytophagales,f:Cytophagaceae,g:Hymenobacter;

TACGGAGGGTGCGAGCGTTGTCCGGATTTATTGGGTTTAAAGGGTGCGTAGGCGGCCTTA

TAAGTCCGAGGTGAAAGCCCGTTGCTCAACAACGGAACTGCCTTGGATACTGTGAGGCTT

GAGTACAGACGAGGTTGGCGGAATGGACCGAGTAGCGGTGAAATGCATAGATACGGTCCA

GAACCCCGATTGCGAAGGCAGCTGACTAGGCTGTTACTGACGCTGAGGCACGACAGCGTG

GGGAGCGAAC

>ASV893 GS|99.2|EF397574_S001044326;k:Bacteria,p:"Proteobacteria",c:Gammaproteobacteria,o:Xanthomonadales,f:Xanthomonadaceae,g:Dyella;

TACGAAGGGTGCAAGCGTTAATCGGAATTACTGGGCGTAAAGCGTGCGTAGGCGGTTTGT

TAAGTCTGCTGTGAAATCCCCGGGCTCAACCTGGGAATGGCAGTGGATACTGGCAAGCTA

GAGTGTGTCAGAGGATGGTGGAATTCCCGGTGTAGCGGTGAAATGCGTAGAGATCGGGAG

GAACATCAGTGGCGAAGGCGGCCATCTGGGACAACACTGACGCTGAGGCACGAAAGCGTG

GGGAGCAAAC

>ASV894 SS|0.8000|KF459924_S004053537;k:Bacteria,p:"Actinobacteria",c:Actinobacteria,o:Solirubrobacterales,f:Solirubrobacteraceae,g:Solirubrobacter

TACGTAGGGGGCAAGCGTTGTCCGGAATCATTGGGCGTAAAGCGCGTGTAGGCGGTTCGA

TAAGTCTGCTGTGAAAGTCCAGGGCTCAACCCTGGGATGCCGGTGGATACTGTCGGGCTA

GAGTACGGAAGAGGCGAGTGGAATTCCCGGTGTAGCGGTGAAATGCGCAGATATCGGGAG

GAACACCAATTGCGAAGGCAGCTCGCTGGGACGTTACTGACGCTGAGACGCGAAAGCGTG

GGGAGCAAAC

>ASV895 GS|98.8|JQ346802_S003290742;k:Bacteria,p:"Actinobacteria",c:Actinobacteria,o:Actinomycetales,f:Cryptosporangiaceae,g:Jatrophihabitans;

TACGTAGGGTGCAAGCGTTGTCCGGAATTATTGGGCGTAAAGAGCTCGTAGGCGGCTTGT

CGCGTCGGCTGTGAAAACCTAGGGCTCAACCCTGGGCCTGCAGCCGATACGGGCAAGCTA

GAATTCGGTAGGGGAGACTGGAATTCCTGGTGTAGCGGTGAAATGCGCAGATATCAGGAG

GAACACCGGTGGCGAAGGCGGGTCTCTGGGCCGATATTGACGCTGAGGAGCGAAAGCGTG

GGGAGCAAAC

>ASV896 SS|1.0000|CP001854_S001872674;k:Bacteria,p:"Actinobacteria",c:Actinobacteria,o:Solirubrobacterales

TACGTAGGGGGCAAGCGTTGTCCGGAATTATTGGGCGTAAAGAGCGTGTAGGCGGCTCGG

TAAGTCTGCTGTGAAAGTCCAGGGCTCAACCCTGGAATGCCGGTGGATACTGTCGGGCTA

GAGTGCGGAAGAGGCGAGTGGAATTCCTGGTGTAGCGGTGAAATGCGCAGATATCAGGAG

GAACACCAATTGCGAAGGCAGCTCGCTGGGACGTTACTGACGCTGAGACGCGAAAGCGTG

GGGAGCAAAC

>ASV897 GS|0.0|None;No hit

GTGTCAGCAGCCGAGAAAGCTTGTAGGGGAAGGAGGGCCAACGCTGTGAGCGTGGGATAG

AGGAGTTTGAGGGCCATGGTAGGGCTAAAGAGCAGCGTAGGGTAATCGAGGAGGCGGGAA

CGAAACCCTAGTAGTCC

>ASV898 GS|99.2|EF457481_S000834906;k:Bacteria,p:"Acidobacteria",c:Acidobacteria_Gp4,g:Gp4;

TACGTAGGGACCAAGCGTTGTTCGGATTTACTGGGCGTAAAGGGCGCGTAGGCGGCGTGT

TAAGTCAGCTGTGAAATCTCCGAGCTTAACTCGGAACGGTCAGCTGATACTGATGTGCTA

GAGTGCAGAAGGGGCAATCGGAATTCTTGGTGTAGCGGTGAAATGCGTAGATATCAAGAG

GAACACCTGAGGTGAAGACGGGTTGCTGGGCTGACACTGACGCTGAGGCGCGAAAGCTAG

GGTAGCAAAC

>ASV899 SS|1.0000|EF635404_S001095318;k:Bacteria,p:"Deinococcus-Thermus",c:Deinococci,o:Deinococcales,f:Deinococcaceae,g:Deinococcus

TACGGAGGGTGCAAGCGTTACCCGGAATCACTGGGCGTAAAGGGCGTGTAGGCGGGGACT

TAAGTCTGGCTTTAAAGACCGGGGCTCAACCTCGGGACTGGGCTGGATACTGGGTTTCTA

GACCTCTGGAGAGGAACTTGGAATTTCTGGTGTAGCGGTGGAATGCGTAGATACCAGAAG

GAACACCGATGGCGAAGGCAGAGTTCTGGACAGAAGGTGACGCTGAGGCGCGAAAGTGTG

GGGAGCGAAC

>ASV900 SS|1.0000|FN391026_S001418752;k:Bacteria,p:"Planctomycetes",c:Planctomycetia,o:Planctomycetales,f:Planctomycetaceae

GACGAACCGTGCGAACGTTATTCGGAATCACTGGGCTTAAAGCGCGTGTAGGCGGGCCGG

CACGTCGGTCGCTGAAATCCCCCGGCTCAACCGGGGAAGGGGCGCCGATACGACCGGCCT

GGAGGGACGTAGGGGGGGCTGGAACTTCCGGTGGAGCGGTGAAATGCGTTGAGATCGGAA

GGAACGCCCGTGGCGAAAGCGAGCCCCTGGACGTCTACTGACGCTGAGACGCGAAAGCCA

GGGGAGCGAA

>ASV901 SS|0.9900|EF457474_S000834899;k:Bacteria,p:"Acidobacteria",c:Acidobacteria_Gp1

TACGAGGGGGGCAAGCGTTGTTCGGAATTATTGGGCGTAAAGGGTGCGTAGGCGGCCCTG

CAAGTCTCGTGTGAAATCCGCAAGCTCAACTTGCGGCCTGCACGGGAAACTGCGGGGCTG

GAGTGTGGGAGAGGTGAGTGGAATTCCCGGTGTAGCGGTGAAATGCGTAGATATCGGGAG

GAACACCTGTGGCGAAAGCGGCTCACTAGACCACAACTGACGCTGAGGCACGAAAGCTAG

GGGAGCAAAC

>ASV902 GS|0.0|None;No hit

GTGTCAGCAGCCGCAAGGACAAAGACCAGCTCTTAGGGAAACGACCTGCTTTCTATGGCG

ACGAGGCTTGCCGATTACTGGAGATTGCTGGGGATGAGCTTATTTCAGAGGCTACCGGAG

CGGCATCTGGCGACAACGTAGTCC

>ASV903 SS|0.9200|AJ252626_S000040852;k:Bacteria,p:"Verrucomicrobia",c:Spartobacteria,g:Spartobacteria_genera_incertae_sedis

TACAGAGGTCCCGAGCGTTGTTCGGATTCATTGGGCGTAAAGGGTGCGTAGGCGGCTGGG

TAAGTTTGATGTGAAATCCTGGGGCTCAACCCTGGAACTGCATTGAATACTGCCCGGCTA

GAGTACTGGAGAGGAAACTGGAATTTACGGTGTAGCAGTGAAATGCGTAGATATCGTAAG

GAAGACCAGTGGCGAAGGCGAGTTTCTGGACAGTTACTGACGCTGAGGCACGAAGGCCAG

GGGAGCAAAC

>ASV904 SS|1.0000|JF803808_S002913330;k:Bacteria,p:"Bacteroidetes",c:Sphingobacteriia,o:"Sphingobacteriales",f:Chitinophagaceae

TACGGAGGGTGCAAGCGTTATCCGGATTCACTGGGTTTAAAGGGTGCGTAGGTGGGTTGG

TAAGTCAGTGGTGAAATCTCCGGGCTTAACCCGGAAACTGCCATTGATACTACTAGTCTT

GAATGTCGTGGAGGTGAGCGGAATATGTCATGTAGCGGTGAAATGCTTAGATATGACATA

GAACACCAATTGCGAAGGCAGCTCACTACACGCATATTGACACTGAGGCACGAAAGCGTG

GGGATCAAAC

>ASV905 SS|0.9900|AB166881_S000386315;k:Bacteria,p:"Proteobacteria",c:Alphaproteobacteria,o:Caulobacterales,f:Caulobacteraceae,g:Phenylobacterium

TACGAAGGGGGCTAGCGTTGCTCGGATTTACTGGGCGTAAAGGGCGCGTAGGCGGACAGT

TTAGTTGGGGGTGAAAGCCCGGGGCTCAACCTCGGAATTGCCTTCAATACTGGCTGTCTT

GAGTACGGGAGAGGTGAGTGGAACTCCGAGTGTAGAGGTGAAATTCGTAGATATTCGGAA

GAACACCAGTGGCGAAGGCGACTCACTGGCCCGTTACTGACGCTGAGGCGCGAAAGCGTG

GGGAGCAAAC

>ASV906 GS|98.8|DQ664244_S000712592;k:Bacteria,p:"Proteobacteria",c:Betaproteobacteria,o:Burkholderiales,f:Burkholderiales_incertae_sedis,g:Piscinibacter;

TACGTAGGGTGCAAGCGTTAATCGGAATTACTGGGCGTAAAGCGTGCGCAGGCGGCTTTG

CAAGACAGATGTGAAATCCCCGGGCTTAACCTGGGAACTGCATTTGTGACTGCATAGCTA

GAGTGCGGCAGAGGGGGATGGAATTCCGCGTGTAGCAGTGAAATGCGTAGATATGCGGAG

GAACACCGATGGCGAAGGCAATCCCCTGGGCCTGCACTGACGCTCATGCACGAAAGCGTG

GGGAGCAAAC

>ASV907 GS|97.6|AM230668_S000749699;k:Bacteria,p:Cyanobacteria/Chloroplast,c:Cyanobacteria,f:Family_I,g:GpI;

TACGGAGGATGCAAGCGTTATCCGGAATGATTGGGCGTAAAGCGTCCGCAGGTGGCGATG

TAAGTCTGCTGTTAAAGAGTGAGGCTCAACCTCATAAGAGCAGTGGAAACTACATAGCTA

GAGTACGTTCGGGGCAGAGGGAATTCCTGGTGTAGCGGTGAAATGCGTAGAGATCAGGAA

GAACACCAGTGGCGAAGGCGCTCTGCTAGGCCGTAACTGACACTGAGGGACGAAAGCTAG

GGGAGCGAAT

>ASV908 GS|98.0|DQ528761_S000701761;k:Bacteria,p:"Acidobacteria",c:Acidobacteria_Gp1,g:Edaphobacter;

TACGAGGGGGGCAAGCGTTGTTCGGAATTATTGGGCGTAAAGGGTGCGTAGGCGGTTTGA

CAAGTCTTATGTGAAATCTCTGGGCTCAACCCAGAGTCTGCATAGGAAACTGTCGGGCTT

GAGTATGGGAGAGGTGAGTGGAATTTCCGGTGTAGCGGTGAAATGCGTAGATATCGGAAG

GAACACCTGTGGCGAAAGCGGCTCACTGGACCATAACTGACGCTGAGGCACGAAAGCTAG

GGGAGCAAAC

>ASV909 SS|1.0000|AY140238_S000397413;k:Bacteria,p:"Proteobacteria",c:Alphaproteobacteria,o:Rhodospirillales,f:Acetobacteraceae

TACGAAGGGGGCTAGCGTTGCTCGGAATGACTGGGCGTAAAGGGCGCGTAGGCGGATCGC

ACAGTCGGGCGTGAAATTCCTGGGCTTAACCTGGGGGCTGCGTTCGAGACGTGGGGTCTA

GAGTGGGGAAGAGGGTCGTGGAATTCCCAGTGTAGAGGTGAAATTCGTAGATATTGGGAA

GAACACCGGTGGCGAAGGCGGCGACCTGGTCCTTGACTGACGCTGAGGCGCGAAAGCGTG

GGGAGCAAAC

>ASV910 SS|1.0000|AY140238_S000397413;k:Bacteria,p:"Proteobacteria",c:Alphaproteobacteria,o:Rhodospirillales,f:Acetobacteraceae

TACGAAGGGGGCTAGCGTTGCTCGGAATGACTGGGCGTAAAGGGCGCGTAGGCGGATGCC

TTAGTCGGGCGTGAAAGTCCTGGGCTTAACCTGGGGATTGCGTTCGATACGGGGTGTCTA

GAGTTGGAAAGAGGGTTGTGGAATTCCCAGTGTAGAGGTGAAATTCGTAGATATTGGGAA

GAACACCGGTGGCGAAGGCGGCGACCTGGTTCTTGACTGACGCTGAGGCGCGAAAGCGTG

GGGAGCAAAC

>ASV911 SS|1.0000|JN090860_S002916046;k:Bacteria,p:"Bacteroidetes",c:Cytophagia,o:Cytophagales,f:Cytophagaceae,g:Hymenobacter

TACGGAGGGTGCGAGCGTTGTCCGGATTTATTGGGTTTAAAGGGTGCGTAGGCGGTCGAT

TAAGTCTGGGGTGAAAGCCCGCTGCTCAACAGCGGAACTGCCCTGGATACTGGTTGACTT

GAGTACAGACGAGGTTGGCGGAATGGACGGAGTAGCGGTGAAATGCATAGATACCGTCCA

GAACCCCGATTGCGAAGGCAGCTGACTAGGCTGATACTGACGCTGAGGCACGACAGCGTG

GGGAGCGAAC

>ASV912 SS|0.9300|AJ289983_S000020777;k:Bacteria,p:"Verrucomicrobia",c:Spartobacteria,g:Spartobacteria_genera_incertae_sedis

TACAGAGGCCTCAAGCGTTGTTCGGATTCATTGGGCGTAAAGGGAGCGTAGGCGGTCGGG

TAAGTCGGATGTGAAATCCTGGGGCTCAACCTCAGAACTGCATTCGATACTGCTTGGCTA

GAGGACTGGAGAGGTGAGTGGAATTCACGGTGTAGCAGTGAAATGCGTAGATATCGTGAG

GAAGACCAGCGGCGAAGGCGGCTCACTGGACAGTATCTGACGCTGATGCTCGAAGGCCAG

GGGAGCAAAC

>ASV913 GS|78.8|AJ009456_S000115949;k:Bacteria,p:"Armatimonadetes",c:Chthonomonadetes,o:Chthonomonadales,f:Chthonomonadaceae,g:Chthonomonas/Armatimonadetes_gp3;

TACGTAGGTGGCAAGCGTTGTCCGGATTTACTGGGCGTAAAGGGCAAGCAGGCGGACTGT

TAAGTAGGAAGTGAAAGGTTGGAGCTCAACTCCAACATTGCTCCCTATACTGGCAGTCTT

GAGTCTCGGAGAGGAAAGCGGAACGATACGTGTAGCGGTGAAATGCGTTGATATGTATCG

GAACACCAATGGCGAAGGCAGCTTTCTGGACGAGAACTGACGCTCATTTGCGAAAGCCGA

GGTAGCGAAC

>ASV914 GS|0.0|None;No hit

TTCCAGCTCCAATAGCGTATATTAAAGTTGTTGCAGTTAAAAAGCTCGTAGTTGAAACTT

GGGCCTGGCTGTCCGGTCCGCCTCACCGCGTGCACTGGTTCGGCCGGGCCTTTCCTTCTG

GGGAGCCGCATGCCCTTCATTGGGTGTGTCGGGGAACCAGGACTTTTACTTTGAAAAAAT

TAGAGTGTTCAAAGCAGGCATATGCTCGAATACATTAGCATGGAATAATAGAATAGGACG

TGTGGTTCTA

>ASV915 SS|1.0000|JQ345500_S003290740;k:Bacteria

GACGTAGGAGGCGAGCGTTGTCCGGAGTTACTGGGCGTAAAGCGCGCGCAGGCGGTCCCG

CAGGTCACCTGTGAAAGCCCCCGGCTCAACCGGGCGGAGGCGGGCGAAACCGCGGGACTG

GAGGGCGGCAGAGGGTCGTGGAATTCCCGGTGTAGTGGTGAAATGCGTAGAGATCGGGAG

GAACACCCGCGGCGAAGGCGGCGACCTGGACCGACCCTGACGCTGAGGCGCGAAGGCCGG

GGGAGCGAAC

>ASV916 GS|100.0|AF542227_S000432293;k:Bacteria,p:Firmicutes,c:Clostridia,o:Clostridiales,f:Clostridiales_Incertae_Sedis_XI,g:Finegoldia;

TACGTATGGAGCGAGCGTTGTCCGGAATTATTGGGCGTAAAGGGTACGCAGGCGGTTTAA

TAAGTCGAATGTTAAAGATCGGGGCTCAACCCCGTAAAGCATTGGAAACTGATAAACTTG

AGTAGTGGAGAGGAAAGTGGAATTCCTAGTGTAGTGGTGAAATACGTAGATATTAGGAGG

AATACCAGTAGCGAAGGCGACTTTCTGGACACAAACTGACGCTGAGGTACGAAAGCGTGG

GGAGCAAACA

>ASV917 GS|0.0|None;No hit

GTGTCAGCAGCCCTGTGTATGTCTCATCACCATCTTCGTCGCTCATCGGCCAGGCGAAAA

TGGTCTTATCTTTGCTCTGCTGAATGATAGCTCTCTGCAGTTGGAGGAAAGCCGCTTCCC

GTTCTCCGTATATCTGTGGCATGCTGATGGCGAAAATACCTAACAACGAGTAGTCC

>ASV918 GS|0.0|None;No hit

CTTCAAATTTGGAGACCACCCTGAGTGAACGACTATCGTGGCTTGAATCCGTCTTCGCGA

CTATCAACCCGAACGTAAGTGTCACACAATTTTCCGATCCATCGTGTAACGCTAATCCGG

GTTCAGGATCCCGAGCTCCACGATGTTGGTGCACGCATAATGGAAGTCCTCCGCGAACGC

CTGGAATCCGGCTTCATGCAGATATCTTTAGCCAAGCCCGGCGAACCTGCGCTCCGACGA

ATTCCACCCC

>ASV919 GS|0.0|None;No hit

TTGTCATAGAGAAGGTAACCAGCGAGGATCTCCCACTCCTTAACTTCTGGGAAACCTTCG

TAGACTGCTTGGGCCGCAAGTGCAAATCTCGATTCAACTACCGAGCCTGGCAAGACTGCA

ATCGTCTCTGGCAAGGTTGGCGCTTCCCCTTCGTCACCATCTTCGGAGCTGTAGGACTGT

AAGTTTTCAGCAAGGCATTTATACTTAAGCCATGCCCTCCGGGGCATTTCGAAGTTCTCC

GTGTCTTCTT

>ASV920 GS|98.0|JQ772481_S003313779;k:Bacteria,p:"Proteobacteria",c:Alphaproteobacteria,o:Sphingomonadales,f:Sphingomonadaceae,g:Sphingomonas;

TACGGAGGGGGCTAGCGTTGTTCGGAATTACTGGGCGTAAAGCGCATGTAGGCGGCTTTG

TAAGTTAGAGGTGAAAGCCCGGGGCTCAACTCCGGAATTGCCTTTAAGACTGCATCGCTT

GAACATCGGAGAGGTAAGTGGAATTCCGAGTGTAGAGGTGAAATTCGTAGATATTCGGAA

GAACACCAGTGGCGAAGGCGACTTACTGGACGATTGTTGACGCTGAGATGCGAAAGCGTG

GGGAGCAAAC

>ASV921 GS|99.2|HM583567_S002988298;k:Bacteria,p:"Proteobacteria",c:Betaproteobacteria,o:Burkholderiales,f:Comamonadaceae,g:Polaromonas;

TACGTAGGGTGCGAGCGTTAATCGGAATTACTGGGCGTAAAGCGTGCGCAGGCGGTGATG

CAAGACAGTTGTGAAATCCCCGGGCTTAACCTGGGAATTGCATCTGTGACTGCATCGCTA

GAGTACGGTAGAGGGGGATGGAATTCCGCGTGTAGCAGTGAAATGCGTAGATATGCGGAG

GAACACCGATGGCGAAGGCAATCCCCTGGACCTGTACTGACGCTCATGCACGAAAGCGTG

GGGAGCAAAC

>ASV922 GS|0.0|None;No hit

GTGTCAGCAGCCGCATCTACCCTCTTCGACGCCCCCACGTCTCTACCTTCATTGACCGTA

TTTCCGCAATTCTCCACTCTCCTCCCCACATTCATCGGTGTCCCAACTCCCAACAGCGCA

GGCTCCGAGCCTCCGAGCCTGATCGACGCTGTGCTTTTCCTAGGGACATACATTCTCTCC

ACAACCTCCATCGACGCACCCGAATCAGACGAAGCCTTCAACGAAACCCTCCAACGACTA

TCGCTCCTCT

>ASV923 GS|0.0|None;No hit

GTGTCAGCAGCCGCAGAAGGCCTGATGGTTGATGGATGTTGTTGATACGGAGCGGAAGAT

TGGAACGAGCAATGGTTTTCCCACTTCCAAATTGACACACTCGCTCAACCAGCTCATCGC

CAGTAACGAGAGCTGACAGATCATACTCGCGAGCATACAGTCGGCGTCAGACCACGGCGA

GAGTGGGGCGCTCCCTCGGCCTCGGCATTCGGGGCACCTCGGTCTCGCCCATCGAATCTA

TCTTCGGCCA

>ASV924 SS|0.9200|AF137029_S000470279;k:Bacteria,p:"Bacteroidetes",c:Cytophagia,o:Cytophagales,f:Cytophagaceae

TACGGAGGGTGCGAGCGTTGTCCGGATTTATTGGGTTTAAAGGGTGCGCAGGTGGCCCGG

TCAGTCAGTGGTGAAATGCGGCCGCTCAACGGTCGAACTGCCATTGATACTGCCGGGCTT

GAGAGGGGTGGAGGCTGCCGGAACGGACGGTGTAGCGGTGAAATGCATAGATATCGTCCA

GAACGCCGATTGCGAAGGCAGGTGGCCACGCCTCATCTGACACTGAGGCACGAAAGCGTG

GGGAGCGAAC

>ASV925 GS|100.0|KM044053_S004224125;k:Bacteria,p:"Actinobacteria",c:Actinobacteria,o:Actinomycetales,f:Nocardiaceae,g:Rhodococcus;

TACGTAGGGTGCAAGCGTTGTCCGGAATTACTGGGCGTAAAGAGCTCGTAGGCGGTTTGT

CACGTCGACTGTGAAATCCCATGGCTCAACTGTGGGCGTGCAGTCGATACGGGCAGACTT

GAGTACTGCAGGGGAGACTGGAATTCCTGGTGTAGCGGTGAAATGCGCAGATATCAGGAG

GAACACCGGTGGCGAAGGCGGGTCTCTGGGCAGTAACTGACGCTGAGGAGCGAAAGCATG

GGTAGCGAAC

>ASV926 GS|0.0|None;No hit

TACGTAGAAGACTAGTGTTATTCATCTTTAATAGGTTTAAAGGGTACCTAGACGGTATAT

CAAGCCTGCAATACGGGACTAATGTACTAGAGTTACTTACGAGGGGGTATTAAAGTACTG

CTGGTGTAGAGATGAAATTCTGTCATACCTCATTTCGTAAGAAATACTACGGCACAGGTA

TAGGCGAAAGCATCCCCTTATGTGATAACTGACGTTGAAGGACGAAGGCTTTGTGTAGCG

AACAGG

>ASV927 SS|0.8900|AM162406_S000650674;k:Bacteria,p:"Planctomycetes",c:Planctomycetia,o:Planctomycetales,f:Planctomycetaceae,g:Zavarzinella

GACGAACCGTGCGAACGTTGTTCGGAATCATTGGGCTTAAAGGGCGCGTAGGCGGGCTTT

CAAGTCCGGGTTGAAATACTCCAGCTTAACTGGAGAACTGGCCTGGATACTGAAGGTCTC

GAGGAAGGTAGGGGCATGCGGAACTAATGGTGGAGCGGTGAAATGCGTTGATATCATTAG

GAACTCCGGTGGCGAAGGCGGCGTGCTGGACCTTTTCTGACGCTGAGGCGCGAAAGCCAG

GGGAGCGAAC

>ASV928 SS|1.0000|EU861928_S001148233;k:Bacteria,p:"Armatimonadetes",c:Armatimonadia,o:Armatimonadales,f:Armatimonadaceae,g:Armatimonas/Armatimonadetes_gp1

TACGTAGGGGGCGAGCGTTGTCCGAAGTTACTGGGCGTAAAGAGCGCGTAGGCGGGTTCT

TAAGTGAGGGGTGAAATTCCGGGGCTCAACCCCGGAACTGCCTTTCATACTGGGAACCTT

GAGTGCGGGAGAGGCGAGTGGAATGGTCGGTGTAGCGGTGAAATGCGTAGATATCGATCG

GAACACCCATGGCGAAGGCAGCTCGCTGGCCTGTAACTGACGCTGAGGCGCGAAAGCGTG

GGGAGCAAAC

>ASV929 SS|1.0000|KJ528316_S004225669;k:Bacteria,p:"Proteobacteria",c:Alphaproteobacteria,o:Sphingomonadales,f:Sphingomonadaceae

TACGGAGGGGGCTAGCGTTGTTCGGAATTACTGGGCGTAAAGCGCACGTAGGCGGCTATT

CAAGTCAGAGGTGAAAGCCCGGAGCTCAACTCCGGAACTGCCTTTGAAACTAGATAGCTT

GAATCATGGAGAGGCGGGTGGAATTCCGAGTGTAGAGGTGAAATTCGTAGATATTCGGAA

GAACACCAGTGGCGAAGGCGGCCCGCTGGACATGTATTGACGCTGAGGTGCGAAAGCGTG

GGGAGCAAAC

>ASV930 SS|1.0000|JX949238_S003747851;k:Bacteria,p:"Bacteroidetes",c:Sphingobacteriia,o:"Sphingobacteriales",f:Sphingobacteriaceae

TACGGAGGATCCGAGCGTTATCCGGATTTATTGGGTTTAAAGGGTGCGTAGGCGGCCTGT

TAAGTCAGGGGTGAAATTTTTCGGCTCAACCGGAAAATTGCCTTTGATACTGATGGGCTT

GAATGCAGCTGAGGTAGGCGGAATGTGACAAGTAGCGGTGAAATGCATAGATATGTCACA

GAACACCAATTGCGAAGGCAGCTTACTAAAGTGTGATTGACGCTGAGGCACGAAAGCGTG

GGGATCAAAC

>ASV931 SS|1.0000|EU861876_S001148181;k:Bacteria,p:"Armatimonadetes",c:Armatimonadia,o:Armatimonadales,f:Armatimonadaceae,g:Armatimonas/Armatimonadetes_gp1

TACGTAGGGGGCCAGCGTTGTCCGAAGTTACTGGGCGTAAAGAGCGCGTAGGCGGACCCT

TAAGTGAGGGGTGAAAGGTTCAGGGCTTAACCCGGACACTGCCTTTCATACTGGGGGTCT

TGAGTGTTGGAGAGGCGAGTGGAATGGTCGGTGTAGCGGTGAAATGCGTAGATATCGATC

GGAACACCTATGGCGAAGGCAGCTCGCTGGCCAACAACTGACGCTGAGGCGCGAAAGCGT

GGGGAGCAAA

>ASV932 GS|0.0|None;No hit

GTGTCAGCAGCCGAGAAAGCTTGTAGGGGAAGGAGGGCCAACGCTGTGAGCGTGGGATAG

AGGAGTTTGAGGGCCATGGTAGGGCTAAAGAGTAGCGTAGGGTAATCGAGGAGGCGGGAA

CGAAACCCTTGTAGTCC

>ASV933 SS|1.0000|EU861928_S001148233;k:Bacteria,p:"Armatimonadetes",c:Armatimonadia,o:Armatimonadales,f:Armatimonadaceae,g:Armatimonas/Armatimonadetes_gp1

TACGTAGGGGGCGAGCGTTGTCCGAAGTTACTGGGCGTAAAGAGCGCGTAGGCGGGTTCT

TAAGTGAGGGGTGAAATTCCGGGGCTCAACCCCGGAACTGCCTCTCATACTGGGGACCTT

GAGTGCGGGAGAGGCGAGTGGAATGGTCGGTGTAGCGGTGAAATGCGTAGATATCGATCG

GAACACCCATGGCGAAGGCAGCTCGCTGGCCTGTAACTGACGCTGAGGCGCGAAAGCGTG

GGGAGCAAAC

>ASV934 GS|0.0|None;No hit

GTGTCAGCAGCCGCCCATGTATGTTCGAGCACATCTGACAGCCGTAAAAGCTTAGCAGTG

ATAACCTTTGAGTGCTGTAATGCTTATATTGTGTAGTCGATGCAAAGTAACTATGAGTTT

CCGAAATATATTAAAGTTAGTATGGAATAATATTCAGAGGTTCTGTAGTGTGGAGCAATT

ACTTTAGGAATCCGCGTTTCCGTTCGGAAACCCAAGGCCCTTCTAGTTAAATCCCCGTCT

TGGTAAACCC

>ASV935 GS|0.0|None;No hit

GTGTCAGCAGCCGCCGTGGAGCGAACTCAAGACGAAAGCCGGCAAAGAGCGTAAACGTCT

TCCTCTGGCTTGTATAGCCTGTCGACGGAAAAAGATCCGTTGTTCCGGCGAGAAACCCTA

GTAGTCC

>ASV936 GS|98.8|JQ309130_S003619637;k:Bacteria,p:"Acidobacteria",c:Acidobacteria_Gp4,g:Blastocatella;

TACGTAGGGACCAAGCGTTGTTCGGATTTACTGGGCGTAAAGGGCGCGTAGGCGGCAATT

CAAGTCAGTTGTGAAATCTCCGAGCTTAACTCGGAACGGTCAACTGATACTGTTTTGCTA

GAGTACAGAAGGGGCAATCGGAATTCTTGGTGTAGCGGTGAAATGCGTAGATATCAAGAG

GAACACCTGAGGTGAAGACGGGTTGCTGGGCTGATACTGACGCTGAGGCGCGAAAGCTAG

GGTAGCAAAC

>ASV937 SS|1.0000|D86512_S000010801;k:Bacteria,p:"Proteobacteria",c:Alphaproteobacteria,o:Rhodospirillales,f:Acetobacteraceae

TACGAAGGGGGCTAGCGTTGCTCGGAATGACTGGGCGTAAAGGGCGCGTAGGCGGATGGC

ACAGTCGGGCGTGAAATTCCTGGGCTTAACCTGGGGGCTGCGTTCGAGACGTGTTGTCTA

GAGTGGGGAAGAGGGTCGTGGAATTCCCAGTGTAGAGGTGAAATTCGTAGATATTGGGAA

GAACACCGGTGGCGAAGGCGGCGACCTGGTCCTTGACTGACGCTGAGGCGCGAAAGCGTG

GGGAGCAAAC

>ASV938 GS|78.5|GQ487984_S001683783;k:Bacteria,p:"Armatimonadetes",c:Chthonomonadetes,o:Chthonomonadales,f:Chthonomonadaceae,g:Chthonomonas/Armatimonadetes_gp3;

TACGTAGGTGGCAAGCGTTGTCCGGATTTACTGGGTGTAAAGGGTGCGCAGGCGGGCCAT

TAAGTAGAAAGTGAAAGGTTGGAGCTCAACTCCGACACTGCTCTCTATACTGGTGGCCTT

GAGTCTTGGAGGGGGAAGCGGAACGACACGTGTAGCGGTGAAATGCGTTGATATGTGTCG

GAACACCAATGGCGAAAGCAGCTTCCTGGACAAGTACTGACGCTCAGGCACGAAAGCCAA

GGTAGCAAAC

>ASV939 GS|78.8|AJ009456_S000115949;k:Bacteria,p:"Armatimonadetes",c:Chthonomonadetes,o:Chthonomonadales,f:Chthonomonadaceae,g:Chthonomonas/Armatimonadetes_gp3;

TACGTAGGTGGCAAGCGTTGTCCGGATTTACTGGGCGTAAAGGGCAAGCAGGCGGACTGT

TAAGTAGAAAGTGAAAGGTTGGGGCTCAACCCCAACACTGCTTTCTATACTGGCAGTCTT

GAATCCCGGAGGGGAAAGCGGAACAATACGTGTAGCGGTGAAATGCGTTGATATGTATTG

GAACACCAATGGCGAAGGCAGCTTTCTGGACGGGGATTGACGCTCATTTGCGAAAGCCGA

GGTAGCGAAC

>ASV940 GS|0.0|None;No hit

GTGTCAGCAGCCGAAGTAGTAGTGTGAAGTGGTTGGGTTGGGTTGGGTTGATGACCTTGC

TTGCTTCCTTTTGCCCTTGCCTTCGCTTCATATGCAGCAGTCGATCTTTTCATAGGCTAT

GTAAGCGTCAAGTCATGCTTCTTGATATCGTGAAATCGTAGCATTTCATAGAATTTTGTT

CGAGAAAGAAAGGATTTCGTGGTGACGTCCGATTCGACTCCTTTGCCCTAGAGCAAAACT

GTCCTCACGA

>ASV941 SS|1.0000|GQ454806_S002223697;k:Bacteria,p:"Bacteroidetes",c:Cytophagia,o:Cytophagales,f:Cytophagaceae,g:Hymenobacter

TACGGAGGGTGCAAGCGTTGTCCGGATTTATTGGGTTTAAAGGGTGCGTAGGCGGTTCTT

TAAGTCCGGGGTGAAAGCCCACTGCTCAACAGTGGAACTGCCCTGGAAACTGGAGGACTT

GAGTACAGACGAGGGTGGCGGAATGGATACTGTAGCGGTGAAATGCATAGATAGTATCCA

GAACACCGATTGCGAAGGCAGCTGCCTAGACTGTAACTGACGCTGAGGCACGAAAGCGTG

GGGAGCGAAC

>ASV942 GS|74.1|AF357197_S000011733;k:Bacteria,p:"Proteobacteria",c:Epsilonproteobacteria,o:Nautiliales,f:Nautiliaceae,g:Nautilia;

AACGGAGGGGGCAAGTGTTACTCGTAAGGACTGGGCGTAAAGGGTTCGTAGGCGGTTTGT

AGTAAGTTAGGTGTTAAATACTAGGTCTCAAGCTTATTTTAGGCATCTAAAACTGACAAT

ACTAGAGTTTGATAGAGGGCAAGGGTACTTTTGATGGAGAGGTAGAATTTGCAGATATCA

ATAGGGACCACCAACTGGCGAAGGCGCTTGTCTGGGTCAAACTGACGCTGAGGAACGAAA

GCGCGGGGAG

>ASV943 GS|98.4|DQ321750_S000636629;k:Bacteria,p:"Actinobacteria",c:Actinobacteria,o:Actinomycetales,f:Nakamurellaceae,g:Nakamurella;

TACGTAGGGTGCAAGCGTTGTCCGGAATTATTGGGCGTAAAGAGCTCGTAGGCGGTTTGT

CGCGTCGAATGTGAAAACTCGGGGCTTAACCCCGGGCCTGCATTCGATACGGGCAGACTA

GAGTTCGGTAGGGGAGACTGGAATTCCTGGTGTAGCGGTGAAATGCGCAGATATCAGGAG

GAACACCGGTGGCGAAGGCGGGTCTCTGGGCCGATACTGACGCTGAGGAGCGAAAGCGTG

GGGAGCAAAC

>ASV944 GS|97.6|AB272165_S000824049;k:Bacteria,p:"Bacteroidetes",c:Cytophagia,o:Cytophagales,f:Cytophagaceae,g:Persicitalea;

TACGGAGGGTGCGAGCGTTGTCCGGATTTATTGGGCTTAAAGGGTGCGCAGGTGGCCCCG

CAAGCCAGTGGTGAAATCCCCCCGCTCAACGGGGGGCGTGCCATTGGAACTGTGGGGCTT

GAGTTCGGCCGAGGCCGCCGGAACGGGCGGTGTAGCGGTGAAATGCATAGATATCGCCCA

GAACGCCGATTGCGAAGGCAGGCGGCCAGGCCGACACTGACACTGAGGCACGAAAGCGTG

GGGAGCGAAC

>ASV945 SS|1.0000|HM032897_S002167665;k:Bacteria,p:"Bacteroidetes",c:Cytophagia,o:Cytophagales,f:Cytophagaceae,g:Hymenobacter

TACGGAGGGTGCGAGCGTTGTCCGGATTTATTGGGTTTAAAGGGTGCGTAGGCGGCTATT

TAAGTCCGGGGTGAAAGCCCGCTGCTCAACAGCGGAACTGCCCTGGATACTGGACAGCTT

GAATACAGTGGAGGTTGGCGGAATGGACCGAGTAGCGGTGAAATGCATAGATACGGTCCA

GAACCCCGATTGCGAAGGCAGCTGACTACACTGGCATTGACGCTGAGGCACGACAGCGTG

GGGAGCGAAC

>ASV946 GS|0.0|None;No hit

GTGTCAGCAGCCACACCAACAGCAATGGTATCCATATCAGCAAATACCTGTCTCCATGGC

AAGGCCATACCAGCATTATCCACCGATGATGAACCCTTCCTACCAAACCTATAGCCCGCA

ACATCCTCCACCACATTTACATCCTCGGCCTCAAATGCCCCTAAACCCGCCCTCGCTATC

ATCCATCCCCTCGCGTCATGACGTCCTTTCCCCTGCCTCGTCCAATGCCTCCCTTCATGT

TCCTCCTTCA

>ASV947 GS|98.4|JQ309130_S003619637;k:Bacteria,p:"Acidobacteria",c:Acidobacteria_Gp4,g:Blastocatella;

TACGTAGGGACCAAGCGTTGTTCGGATTTACTGGGCGTAAAGGGCGCGTAGGCGGTAATT

CAAGTCAGTTGTGAAATCTCCGAGCTTAACTCGGAACGGTCAACTGATACTGTTTTGCTA

GAGTACAGAAGGGGCAATCGGAATTCTTGGTGTAGCGGTGAAATGCGTAGATATCAAGAG

GAACACCTGAGGTGAAGACGGGTTGCTGGGCTGATACTGACGCTGAGGCGCGAAAGCTAG

GGTAGCAAAC

>ASV948 SS|1.0000|DQ986200_S000736608;k:Bacteria,p:"Planctomycetes",c:Planctomycetia,o:Planctomycetales,f:Planctomycetaceae

GACGAACCGTGCGAACGTTATTCGGAATCACTGGGCTTAAAGCGCGTGTAGGCGGGCCGT

CCCGTCGGTCGCTGAAATCCCCCGGCTCAACCGGGGAAGTGGCGTCGATACGAGCGGCCT

GGAGGGGAGTAGGGGGGCCTGGAACTTCCGGTGGAGCGGTGAAATGCGTTGAGATCGGAA

GGAACGCCCGTGGCGAAAGCGAGGCCCTGGACTCTTTCTGACGCTGAGACGCGAAAGCCA

GGGGAGCGAA

>ASV949 GS|98.4|HF558380_S003722571;k:Bacteria,p:"Proteobacteria",c:Betaproteobacteria,o:Burkholderiales,f:Burkholderiaceae,g:Lautropia;

TACGTAGGGTGCAAGCGTTAATCGGAATTACTGGGCGTAAAGCGTGCGCAGGCGGTTTTG

TAAGACCGATGTGAAATCCCCGGGCTTAACCTGGGAACTGCATTGGTGACTGCAAGGCTT

GAGTGTGTCAGAGGGAGGTGGAATTCCGCGTGTAGCAGTGAAATGCGTAGATATGCGGAG

GAACACCGATGGCGAAGGCAGCCTCCTGGGATAACACTGACGCTCATGCACGAAAGCGTG

GGGAGCAAAC

>ASV950 GS|98.8|AB512285_S001610744;k:Bacteria,p:"Actinobacteria",c:Actinobacteria,o:Actinomycetales,f:Kineosporiaceae,g:Angustibacter;

TACGTAGGGTGCAAGCGTTGTCCGGAATTATTGGGCGTAAAGAGCTCGTAGGCGGTTTGT

CGCGTCTGCTGTGAAAATCCAGGGCTTAACCCTGGACCTGCAGTGGGTACGGGCAGACTA

GAGTGCGGTAGGGGAGATCGGAATTCCTGGTGTAGCGGTGAAATGCGCAGATATCAGGAG

GAACACCGGTGGCGAAGGCGGATCTCTGGGCCGTAACTGACGCTGAGGAGCGAAAGCATG

GGGAGCGAAC

>ASV951 SS|1.0000|AB778530_U010573767;k:Bacteria,p:"Proteobacteria",c:Alphaproteobacteria,o:Rhodospirillales,f:Acetobacteraceae

TACGAAGGGGGCTAGCGTTGCTCGGAATGACTGGGCGTAAAGGGCGCGTAGGCGGTTCAC

GCAGTCAGATGTGAAATTCCTGGGCTTAACCTGGGGGCTGCATTTGAGACGCGTGGGCTT

GAGTGTGAAAGAGGGTCGTGGAATTCCCAGTGTAGAGGTGAAATTCGTAGATATTGGGAA

GAACACCGGTGGCGAAGGCGGCGACCTGGTTCATAACTGACGCTGAGGCGCGAAAGCGTG

GGGAGCAAAC

>ASV952 GS|0.0|None;No hit

GTGTCAGCAGCCGCTAAACCATTAAAAGATTGCGCAAAATTGAGGCGTTGTGTTGAACTT

TTTGCATCGCCCAAAGATGAAATGTAAGGATTGGCAGCGGTTTCTAAAATTGTCAGCCCG

CAGGCAATGATGAACAGTGCAACCAGGAAAAAGCTGTATTGCTGCGTGTTGGCCGCCGGA

ATAAACAGGTAAGCACCAAAAGCAAAAAGCAGCAAACCGGTGATGATACCGGTTTTGTAG

CCATACTTTT

>ASV953 GS|97.2|JN090860_S002916046;k:Bacteria,p:"Bacteroidetes",c:Cytophagia,o:Cytophagales,f:Cytophagaceae,g:Hymenobacter;

TACGGAGGGTGCGAGCGTTGTCCGGATTTATTGGGTTTAAAGGGTGCGTAGGCGGCCTTT

TAAGTCTGGGGTGAAAGCCCGTTGCTTAACAACGGAACTGCCCTGGATACTGGAAGGCTT

GAGTACAGACGAGGTTGGCGGAATGGACCGAGTAGCGGTGAAATGCATAGATACGGTCCA

GAACCCCGATTGCGAAGGCAGCTGACTAGGCTGATACTGACGCTGAGGCACGACAGCGTG

GGGAGCGAAC

>ASV954 GS|99.2|EU622978_S001098404;k:Bacteria,p:"Deinococcus-Thermus",c:Deinococci,o:Deinococcales,f:Deinococcaceae,g:Deinococcus;

TACGGAGGGTGCAAGCGTTACCCGGAATCACTGGGCGTAAAGGGCGTGTAGGCGGCCTGC

CAAGTCTGGTTTTAAAGCCTGCGGCTCAACCGCAGATCTGGACTGGAGACTGGTAGGCTA

GACCTCTGGAGAGAGAACTGGAATTCCTGGTGTAGCGGTGGAATGCGTAGATACCAGGAG

GAACACCGATGGCGAAGGCAGGTTCTTGGACAGAAGGTGACGCTGAGGCGCGAAAGTGTG

GGGAGCAAAC

>ASV955 SS|0.9200|EF174497_S000776520;k:Bacteria,p:"Proteobacteria",c:Alphaproteobacteria,o:Rhizobiales

TACGAAGGGGGCTAGCGTTGCTCGGAATCACTGGGCGTAAAGGGCGCGTAGGCGGCCGAT

CAAGTCAGAGGTGAAAGCCCAAGGCTCAACCTTGGAATTGCCTTTGATACTGTTCGGCTA

GAGACCGGAAGAGGTTAGTGGAACTGCGAGTGTAGAGGTGAAATTCGTAGATATTCGCAA

GAACACCAGTGGCGAAGGCGGCTAACTGGTCCGGTTCTGACGCTGAGGCGCGAAAGCGTG

GGGAGCAAAC

>ASV956 SS|1.0000|EU861928_S001148233;k:Bacteria,p:"Armatimonadetes",c:Armatimonadia,o:Armatimonadales,f:Armatimonadaceae,g:Armatimonas/Armatimonadetes_gp1

TACGTAGGGGGCGAGCGTTGTCCGAAGTTACTGGGCGTAAAGAGCGCGTAGGCGGGTTCT

TAAGTGAGGGGTGAAATTCCGAGGCTTAACCTCGGAACTGCCTTTCATACTGGGAATCTT

GAGTGTGGGAGAGGCGAGTGGAATGGTCGGTGTAGCGGTGAAATGCGTAGATATCGATCG

GAACACCCATGGCGAAGGCAGCTCGCTGGCCCATAACTGACGCTGAGGCGCGAAAGCGTG

GGGAGCAAAC

>ASV957 GS|0.0|None;No hit

GTGTCAGCAGCCGACAGTGGAAAGACCTCGAGACAGCAACAGTCACGTTTAGAGAGCCTA

AACAAGCAGCCTGAAGGGCGTAAGACGCGAAGCTCAACAGAAAAGGAAGAGAAAAGGAAA

GAGGCGAGGGTGAATTCTATGTTGAACAAATTCGTAGAATCGGAGCCAGGTCATTCCAAT

ATGGGCCAGCCTTCAAAGCCAACATGCCAGAGTCCAAAGCGCCTCCAAAACAAAGCTGAA

CCAAAACGAC

>ASV958 SS|1.0000|FN391026_S001418752;k:Bacteria,p:"Planctomycetes",c:Planctomycetia,o:Planctomycetales,f:Planctomycetaceae

GACGAACCGTGCGAACGTTATTCGGAATTACTGGGCTTAAAGCGCGTGTAGGCGGGCCGG

CACGTCCGACGCTGAAATCCCCCGGCTCAACCGGGGAAGTGGCGGGGATACGACCGGCCT

GGAGGGGGGTAGGGGGACCTGGAACTTCCGGTGGAGCGGTGAAATGCGTTGAGATCGGAA

GGAACGCCCGTGGCGAAAGCGAGGTCCTGGACCCTTTCTGACGCTGAGACGCGAAAGCCA

GGGGAGCGAA

>ASV959 GS|98.8|AJ289983_S000020777;k:Bacteria,p:"Verrucomicrobia",c:Spartobacteria,g:Spartobacteria_genera_incertae_sedis;

TACAGAGGTCTCAAGCGTTGTTCGGATTCATTGGGCGTAAAGGGTGCGCAGGCTGCGGGG

TAAGTCGGATGTGAAATTTAGGGGCTCAACCTCTAAACTGCATTCGATACTGCTCTGCTA

GAGGACTGTAGAGGAGATTGGAATTCACGGTGTAGCAGTGAAATGCGTAGATATCGTGAG

GAAGACCAGTGGCGAAGGCGAATCTCTGGGCAGTTCCTGACGCTCATGCACGAAGGCCAG

GGGAGCAAAC

>ASV960 GS|100.0|L14326_S000414422;k:Bacteria,p:Firmicutes,c:Bacilli,o:Bacillales,f:Bacillales_Incertae_Sedis_XI,g:Gemella;

TACGTAGGTGGCAAGCGTTGTCCGGAATTATTGGGCGTAAAGCGCGCGCAGGTGGTTTAA

TAAGTCTGATGTGAAAGCCCACGGCTCAACCGTGGAGGGTCATTGGAAACTGTTAAACTT

GAGTGCAGGAGAGAAAAGTGGAATTCCTAGTGTAGCGGTGAAATGCGTAGAGATTAGGAG

GAACACCAGTGGCGAAGGCGGCTTTTTGGCCTGTAACTGACACTGAGGCGCGAAAGCGTG

GGGAGCAAAC

>ASV961 GS|0.0|None;No hit

TCAAACACCACTGATGCTAGCCGCTGGTCTGAGTTTAAGAGAGATAGTAGCCATATTAGT

GAACAAGCCTGAGCTTGACATAAATCTTCAGGGCGGTGAGTCCCAAACAACGGCACTTCA

TTGTGCCGTAGAGTCAGGTAGCGCTCGGAATGCGAGGATGATTTTGAGTCACCCCGAGAT

CGAAGTCAACAAGGGAAACCGTTGGTGCACGCCCTTGATATCGGCAGCAACGAGTGGCTA

TACTTCTGTA

>ASV962 GS|99.6|Y18833_S000019071;k:Bacteria,p:"Bacteroidetes",c:Cytophagia,o:Cytophagales,f:Cytophagaceae,g:Hymenobacter;

TACGGAGGGTGCAAGCGTTGTCCGGATTTATTGGGTTTAAAGGGTGCGTAGGTGGCCTGT

TAAGTCCGGGGTGAAAGCCCACAGCTCAACTGTGGAACTGCCCTGGATACTGGCAGGCTT

GAGAGTAGTCGAGGGTGGCGGAATGGACGGTGTAGCGGTGAAATGCATAGATACCGTCCA

GAACCCCAATTGCGTAGGCAGCTGCCTAGGCTACATCTGACACTGAGGCACGAAAGCGTG

GGGAGCGAAC

>ASV963 SS|1.0000|AJ420142_S000145622;k:Bacteria

GACGTAGGAGACGAGCGTTGTCCGGATTTACTGGGCGTAAAGCGCGCGCAGGCGGCCCCG

CAGGTCGGTCGTGAAAGCCCCCGGCTCAACCGGGCGGAGGCGAGCGAAACCGCGGGGCTG

GAGGCCGGCAGAGGGTGGTGGAATTCCCGGTGTAGTGGTGAAATGCGTAGAGATCGGGAG

GAACACCCGCGGCGAAGGCGGCCACCTGGACCGGGCCTGACGCTGAGGCGCGAAGGCCGG

GGGAGCGAAC

>ASV964 SS|0.8900|GQ421847_S001589376;k:Bacteria,p:"Bacteroidetes",c:Sphingobacteriia,o:"Sphingobacteriales",f:Chitinophagaceae,g:Segetibacter

TACGGAGGGTGCGAGCGTTATCCGGATTCACTGGGTTTAAAGGGTGCGTAGGTGGGTTGG

TAAGTCAGTGGTGAAATCTCCGTGCTTAACATGGAAACTGCCATTGATACTACTGGTCTT

GAATTTTCTGGAGGTTAGCGGAATATGTCATGTAGCGGTGAAATGCTTAGATATGACATA

GAACACCAATTGCGAAGGCAGCTAGCTACAGGGATATTGACACTGAGGCACGAAAGCGTG

GGGATCAAAC

>ASV965 GS|100.0|X71120_S000007272;k:Bacteria,p:"Proteobacteria",c:Gammaproteobacteria,o:Aeromonadales,f:Aeromonadaceae,g:Aeromonas;

TACGGAGGGTGCAAGCGTTAATCGGAATTACTGGGCGTAAAGCGCACGCAGGCGGTTGGA

TAAGTTAGATGTGAAAGCCCCGGGCTCAACCTGGGAATTGCATTTAAAACTGTCCAGCTA

GAGTCTTGTAGAGGGGGGTAGAATTCCAGGTGTAGCGGTGAAATGCGTAGAGATCTGGAG

GAATACCGGTGGCGAAGGCGGCCCCCTGGACAAAGACTGACGCTCAGGTGCGAAAGCGTG

GGGAGCAAAC

>ASV966 GS|78.4|X78017_S000008814;k:Bacteria,p:Firmicutes,c:Negativicutes,o:Selenomonadales,f:Acidaminococcaceae,g:Acidaminococcus;

TACGTAGGTGGCAAGCGTTGTCCGGATTTACTGGGCGTAAAGGGCAGGCAGGCGGACTGT

TAAGTAGAAAGTGAAAGGTTGGGGCTTAACCCCAACACTGCTTTCTATACTGGCAGTCTT

GAATCCCGGAGGGGAAAGCGGAACAATACGTGTAGCGGTGAAATGCGTTGATATGTATTG

GAACACCAATGGCGAAGGCAGCTTTCTGGACGGGGATTGACGCTCATCTGCGAAAGCCGA

GGTAGCGAAC

>ASV967 SS|1.0000|HM032898_S002167666;k:Bacteria,p:"Bacteroidetes",c:Cytophagia,o:Cytophagales,f:Cytophagaceae,g:Hymenobacter

TACGGAGGGTGCGAGCGTTGTCCGGATTTATTGGGTTTAAAGGGTGCGTAGGCGGCTCTT

TAAGTCTGGGGTGAAAGCCCGCTGCTCAACAGCGGAACTGCCCTGGATACTGGAGAGCTT

GAGTACAGACGAGGTTGGCGGAATGGACGGAGTAGCGGTGAAATGCATAGATACCGTCCA

GAACCCCGATTGCGAAGGCAGCTGACTAGGCTGATACTGACGCTGAGGCACGACAGCGTG

GGGAGCGAAC

>ASV968 SS|1.0000|EU861928_S001148233;k:Bacteria,p:"Armatimonadetes",c:Armatimonadia,o:Armatimonadales,f:Armatimonadaceae,g:Armatimonas/Armatimonadetes_gp1

TACGTAGGGGGCGAGCGTTGTCCGAAGTTACTGGGCGTAAAGAGCGCGTAGGCGGACTCT

TAAGTGAGGGGTGAAAGTCCGAGGCTCAACCTCGGAACTGCCTTTCATACTGGGAGCCTT

GAGTGCGGGAGAGGCGAGTGGAATGGTTGGTGTAGCGGTGAAATGCGTAGATATCAATCG

GAACACCCATGGCGAAGGCAGCTCGCTGGCCTGTAACTGACGCTGAGGCGCGAAAGCGTG

GGGAGCAAAC

>ASV969 GS|0.0|None;No hit

GTGTCAGCAGCCGCGCAGGAAGTTAATCTTAGTAGATGTGTATCACTTTCTTCTCCTGGC

TGCTGGTAAAGACGAGGGTGCCCATCTCCCGGTAAATCGGCCATCTAATTGCTGGTCATA

CTTTCGAAACGTGAAGAACTCAGTCGGTCCGCTGAGTTTTACGTCTCACTCCAGGACGAT

TTTCAAAGGACCGTCCGCAAGGGTCCCGGCATTCTCTGCGGAAAGCAACAGAGGTGTGAG

CGCAATTATT

>ASV970 SS|1.0000|D86513_S000011943;k:Bacteria,p:"Proteobacteria",c:Alphaproteobacteria,o:Rhodospirillales,f:Acetobacteraceae

TACGAAGGGGGCTAGCGTTGCTCGGAATGACTGGGCGTAAAGGGCGCGTAGGCGGATCGA

TCAGTCGGGCGTGAAATTCCCGGGCTCAACCTGGGGACTGCGTTCGAGACGGTTGGTCTA

GAGTGAGGAAGAGGGTCGTGGAATTCCCAGTGTAGAGGTGAAATTCGTAGATATTGGGAA

GAACACCGGTGGCGAAGGCGGCGACCTGGTCCTTGACTGACGCTGAGGCGCGAAAGCGTG

GGGAGCAAAC

>ASV971 GS|99.6|AY677186_S000574646;k:Bacteria,p:"Actinobacteria",c:Actinobacteria,o:Actinomycetales,f:Corynebacteriaceae,g:Corynebacterium;

TACGTAGGGTGCGAGCGTTGTCCGGAATTACTGGGCGTAAAGAGCTCGTAGGTGGTTTGT

CGCGTCGTTTGTGTAATACCGCAGCTTAACTGCGGGGTTGCAGGCGATACGGGCATAACT

TGAGTGCTGTAGGGGAGACTGGAATTCCTGGTGTAGCGGTGGAATGCGCAGATATCAGGA

GGAACACCGATGGCGAAGGCAGGTCTCTGGGCAGTAACTGACGCTGAGGAGCGAAAGCAT

GGGTAGCGAA

>ASV972 GS|0.0|None;No hit

GTGTCAGCAGCCGCACCAAGCAGCCCGAAGGCGAGGTTCTTGAAGTTCCCCATTGAGAGT

ATTGAGTAGGGAGGAGCGATTGGAGTGTGATGGTATGGGCCCCGATACACCAGTGATGTA

GATGGGAAAAGAGTGTGGGGAGGAATGGGTCGGATTGTTGAGGACAGGATACTGAATTCG

AAGTTGACAGATATTGAACTTCTCGGACCCCCCTGGAATTTCTTAGCTGTGGCGCTAGTA

GGCCCAGCCG

>ASV973 GS|0.0|None;No hit

GTGTCAGGCAGGTGTGTAGCCTTGTCCAACGAGCTTCTCTTAAGAGCGAGCCGACCCCTC

TTCCAGCCTTGTCCATCGGACTTCTCTCTACTCTACCCCCGTAGTCC

>ASV974 SS|1.0000|AF173005_S000388573;k:Bacteria,p:"Actinobacteria",c:Actinobacteria,o:Actinomycetales

TACGTAGGGTGCAAGCGTTGTCCGGAATTACTGGGCGTAAAGAGCTCGTAGGCGGTGTGT

CGCGTCGTCTGTGAAATCCCGTGGCTCAACCACGGGCTTGCAGGCGATACGGGCAGACTC

GAGTATTGCAGGGGAGACTGGAATTCCTGGTGTAGCGGTGAAATGCGCAGATATCAGGAG

GAACACCGGTGGCGAAGGCGGGTCTCTGGGCAAATACTGACGCTGAGGAGCGAAAGCATG

GGTAGCAAAC

>ASV975 SS|0.9900|AB072735_S000251887;k:Bacteria,p:"Gemmatimonadetes",c:Gemmatimonadetes,o:Gemmatimonadales,f:Gemmatimonadaceae,g:Gemmatimonas

TACGGAGGGTGCAAGCGTTGTCCGGAATCACTGGGCGTAAAGGGCGCGTAGGCGGCTCAT

AAAGGCTGTGGTGAAAGTTCGGGGCTCAACCTCGAATCGGCCGTGGCGACTTGTGGGCTG

GAGCACTGTAGAGGCAGGTGGAATTCCGGGTGTAGCGGTGGAATGCGTAGAGATCCGGAA

GAACACCAGTGGCGAAGGCGGCCTGCTGGGCAGTGGCTGACGCTGAGGCGCGACAGCGTG

GGGAGCAAAC

>ASV976 SS|0.9900|AB025317_S000022213;k:Bacteria,p:"Actinobacteria",c:Actinobacteria,o:Actinomycetales

TACGTAGGGTGCGAGCGTTGTCCGGAATTATTGGGCGTAAAGAGCTCGTAGGCGGTACAT

CACGTCTGCTGTGAAATCTCGGGGCTCAACCCCGAGCGTGCAGTGGATACGGGTGAACTC

GAGTTCGGATGGGGAGACTGGAATTCCTGGTGTAGCGGTGGAATGCGCAGATATCAGGAG

GAACACCGGTGGCGAAGGCGGGTCTCTGCACCGATACTGACGCTGAGGAGCGAAAGCGTG

GGGAGCAAAC

>ASV977 GS|98.0|AM229669_S000643543;k:Bacteria,p:"Proteobacteria",c:Alphaproteobacteria,o:Sphingomonadales,f:Sphingomonadaceae,g:Sphingomonas;

TACGGAGGGAGCTAGCGTTATTCGGAATTACTGGGCGTAAAGCGCACGTAGGCGGCTTTG

TAAGTTAGAGGTGAAAGCCCAGAGCTCAACTCTGGAACTGCCTTTAAGACTGCATCGCTC

GAATCCAGGAGAGGTGAGTGGAATTCCGAGTGTAGAGGTGAAATTCGTAGATATTCGGAA

GAACACCAGTGGCGAAGGCGGCTCACTGGACTGGTATTGACGCTGAGGTGCGAAAGCGTG

GGGAGCAAAC

>ASV978 SS|0.9800|CP001854_S001872674;k:Bacteria,p:"Actinobacteria",c:Actinobacteria,o:Solirubrobacterales

TACGTAGGGGGCAAGCGTTGTCCGGAATCATTGGGCGTAAAGAGCGTGTAGGCGGCCCGA

TAAGTCTGCCGTGAAAGTCCAGGGCTCAACCCTGGAATGCCGGTGGATACTGTCGGGCTA

GAGTCCGGAAGGGGCGAGTGGAATTCCTGGTGTAGCGGTGAAATGCGCAGATATCAGGAG

GAACACCAATGGCGAAGGCAGCTCGCTGGGACGGTACTGACGCTGAGACGCGAAAGCGTG

GGGAGCAAAC

>ASV979 SS|0.8800|AM231587_S000650722;k:Bacteria,p:"Proteobacteria",c:Alphaproteobacteria,o:Rhodospirillales,f:Acetobacteraceae,g:Acidisoma

TACGAAGGGGGCTAGCGTTGCTCGGAATGACTGGGCGTAAAGGGCGCGTAGGCGGATTGG

TTAGTCAGACGTGAAATTCCTGGGCTCAACCTGGGGGCTGCGTTTGATACGGCTGGTCTA

GAGTTTGGAAGAGGGTCGTGGAATTCCCAGTGTAGAGGTGAAATTCGTAGATATTGGGAA

GAACACCGGTGGCGAAGGCGGCGACCTGGTCCTTGACTGACGCTGAGGCGCGAAAGCGTG

GGGAGCAAAC

>ASV980 GS|0.0|None;No hit

TACGGGGGGGGCGAGCGTTATTCGAAATGATTGGGCGTAAAGAGCACGTAGACGGTTTTT

TAAGTGGACATTATATCTTTTTTGTTCTCTAAGGATAAAAAAGAAAGGATTATGGAAATA

TTTCTGTACTCGGGAAAAAGACCAAGGCTCAACCATGGTGTTTCCCGCCAAACTAAAAAA

CTAGAGTAAGTTAGAGGAAAGTGGAATTCCTGGAGGAAAGGTTAAATTTTATGAGATCAG

GAGGAAGGCC

>ASV981 SS|1.0000|JX294485_S003614212;k:Bacteria,p:"Bacteroidetes",c:Cytophagia,o:Cytophagales,f:Cytophagaceae,g:Hymenobacter

TACGGAGGGTGCAAGCGTTGTCCGGATTTATTGGGTTTAAAGGGTGCGTAGGCGGCCGTT

TAAGTCCGGGGTGAAAGCCCGTTGCTCAACAACGGAACTGCCCCGGAAACTGGAGGGCTT

GAGTACAGACGAGGGCGGCGGAATGGACGGTGTAGCGGTGAAATGCATAGATACCGTCCA

GAACCCCGATTGCGAAGGCAGCTGCCTAGACTGTGACTGACGCTGAGGCACGAAAGCGTG

GGGAGCGAAC

>ASV982 GS|0.0|None;No hit

GTGTCAGCAGCCTCCGTTAGCAAAGCTGACCTCGCCCTTAGCAGTTTTGACCAACTCTCA

GGGAGCGAAAAAGGAAGCAGGACGTAAAATCTGATCACTCTAGTGGGTATAGAGAATGCT

CATGGGCGGTTCAACCGCAGTATCTTCTTGTATACCCATCCTGCTTTATTCTCTGATGAA

CAGGCTCGCGAACCCCATTCCAGTCCCAACGCAGGCACTCACCACTCCGACCTCCAATCC

CCTCCTCTCC

>ASV983 GS|99.6|AJ132943_S000128260;k:Bacteria,p:"Actinobacteria",c:Actinobacteria,o:Actinomycetales,f:Propionibacteriaceae,g:Friedmanniella;

TACGTAGGGTCCGAGCGTTGTCCGGAATTATTGGGCGTAAAGGGCTTGTAGGCGGTTCGT

CGCGTCAGAAGTGAAAACTCAGGGCTTAACCCTGAGCCTGCTTTTGATACGGGCGGACTA

GAGGGATGCAGGGGAGAACGGAATTCCTGGTGGAGCGGTGGAATGCGCAGATATCAGGAG

GAACACCGGTGGCGAAGGCGGTTCTCTGGGCATTACCTGACGCTGAGAAGCGAAAGCGTG

GGGAGCAAAC

>ASV984 SS|0.9900|AY289372_S000405842;k:Bacteria,p:"Acidobacteria",c:Acidobacteria_Gp4

TACGTAGGGACCAAGCGTTGTTCGGATTTACTGGGCGTAAAGGGTGCGTAGGCGGCGTGA

CAAGTCACTTGTGAAATCTCCGAGCTTAACTCGGAACTGCCAAGTGATACTGTCGTGCTA

GAGTACAGAAAGGGTAACTGGAATTCTTGGTGTAGCGGTGAAATGCGTAGATATCAAGAG

GAACACCTGAGGCGAAGGCGAGTTACTAGGCTGATACTGACGCTGAGGCACGAAAGCTAG

GGGAGCGAAC

>ASV985 SS|0.8100|EF067860_S000769040;k:Bacteria,p:"Bacteroidetes",c:Sphingobacteriia,o:"Sphingobacteriales",f:Chitinophagaceae

TACGGAGGGTGCAAGCGTTATCCGGATTTACTGGGTTTAAAGGGTGTGTAGGCGGACTTT

TAAGTCAGAGGTGAAATCCCGGGGCTCAACCCCGGAACTGCCCCTGATACTATTGGTCTT

GAATACTGATGAGGTGGGCGGAACGGGTCATGTAGCGGTGAAATGCTTAGAGATGACCCG

GAACACCGATTGCGAAGGCAGCTCACTGGGCAGTTATTGACGCTGAGGCACGAAAGCGTG

GGGATCAAAC

>ASV986 GS|100.0|DQ886273_S001020496;k:Bacteria,p:Cyanobacteria/Chloroplast,c:Chloroplast,f:Chloroplast,g:Streptophyta;

GACAGAGGATGCAAGCGTTATCCGGAATGATTGGGCGTAAAGCGTCTGTAGGTGGCTTTT

TAAGTTCGCCGTCAAATCCCAGGGCTCAACCCTGGACAGGCGGTGGAAACTACCAAGCTG

GAGTACGGTAGGGGCAGAGGGAATTTCCGGTGGAGCGGTGAAATGCGTAGAGATCGGAAA

GAACACCAACGGCGAAAGCACTCTGCTGGGCCGACACTGACACTGAGAGACGAAAGCTAG

GGGAGCGAAT

>ASV987 GS|98.4|AB428568_S001168624;k:Bacteria,p:"Proteobacteria",c:Alphaproteobacteria,o:Sphingomonadales,f:Sphingomonadaceae,g:Sphingomonas;

TACGGAGGGAGCTAGCGTTATTCGGAATTACTGGGCGTAAAGCGCACGTAGGCGGCTTTG

TAAGTTAGAGGTGAAAGCCCAGAGCTCAACTCTGGAACTGCCTTTAAGACTGCATCGCTT

GAATCCAGGAGAGGTGAGTGGAATTCCGAGTGTAGAGGTGAAATTCGTAGATATTCGGAA

GAACACCAGTGGCGAAGGCGGCTCACTGGACTGGTATTGACGCTGAGGTGCGAAAGCGTG

GGGAGCAAAC

>ASV988 SS|1.0000|FN391026_S001418752;k:Bacteria,p:"Planctomycetes",c:Planctomycetia,o:Planctomycetales,f:Planctomycetaceae

GACGAACCGTGCGAACGTTATTCGGAATCACTGGGCTTAAAGCGCGTGTAGGCGGCTCGG

CACGTCGGACGTTGAAATCCCCCGGCTCAACCGGGGAAGTGGCGCCGATACGGCCGGGCT

GGAGGGGGGTAGGGGGGTCTGGAACTTCCGGTGGAGCGGTGAAATGCGTTGAGATCGGAA

GGAACGCCCGTGGCGAAAGCGAGGCCCTGGACCCTTTCTGACGCTGAGACGCGAAAGCCA

GGGGAGCGAA

>ASV989 GS|100.0|AF512827_S000430787;k:Bacteria,p:"Proteobacteria",c:Betaproteobacteria,o:Burkholderiales,f:Burkholderiaceae,g:Burkholderia;

TACGTAGGGTGCGAGCGTTAATCGGAATTACTGGGCGTAAAGCGTGCGCAGGCGGTTCGT

TAAGACAGATGTGAAATCCCCGGGCTTAACCTGGGAACTGCATTTGTGACTGGCGAGCTA

GAGTATGGCAGAGGGGGGTAGAATTCCACGTGTAGCAGTGAAATGCGTAGAGATGTGGAG

GAATACCGATGGCGAAGGCAGCCCCCTGGGCCAATACTGACGCTCATGCACGAAAGCGTG

GGGAGCAAAC

>ASV990 SS|1.0000|D86513_S000011943;k:Bacteria,p:"Proteobacteria",c:Alphaproteobacteria,o:Rhodospirillales,f:Acetobacteraceae

TACGAAGGGGGCTAGCGTTGCTCGGAATGACTGGGCGTAAAGGGCGCGTAGGCGGATAAC

ACAGTCAGATGTGAAATTCCCGGGCTTAACCTGGGGGCTGCATTTGATACGTGTGATCTA

GAGTGAGGAAGAGGGTCGTGGAATTCCCAGTGTAGAGGTGAAATTCGTAGATATTGGGAA

GAACACCGGTGGCGAAGGCGGCGACCTGGTCCTTGACTGACGCTGAGGCGCGAAAGCGTG

GGGAGCAAAC

>ASV991 SS|1.0000|AY140238_S000397413;k:Bacteria,p:"Proteobacteria",c:Alphaproteobacteria,o:Rhodospirillales,f:Acetobacteraceae

TACGAAGGGGGCTAGCGTTGCTCGGAATGACTGGGCGTAAAGGGCGCGTAGGCGGATCTC

ACAGTCGGGCGTGAAATTCCTGGGCTTAACCTGGGGGCTGCGTTTGAGACGTGGGGTCTA

GAGTTTGGAAGAGGGTCGTGGAATTCCCAGTGTAGAGGTGAAATTCGTAGATATTGGGAA

GAACACCGGTGGCGAAGGCGGCGACCTGGTCCTGGACTGACGCTGAGGCGCGAAAGCGTG

GGGAGCAAAC

>ASV992 SS|0.9500|FJ842646_S001352336;k:Bacteria,p:"Actinobacteria",c:Actinobacteria,o:Actinomycetales,f:Nocardioidaceae

TACGTAGGGTGCAAGCGTTGTCCGGAATTATTGGGCGTAAAGGGCTCGTAGGCGGTCCAT

CGCGTCGGGAGTGAAAAGCTGGGGCTTAACCCCAGTCCTGCTTCCGATACGGGTGGACTA

GAGGTATGCAGGGGAGAACGGAATTCCTGGTGTAGCGGTGAAATGCGCAGATATCAGGAG

GAACACCGGTGGCGAAGGCGGTTCTCTGGGCATTACCTGACGCTGAGGAGCGAAAGCGTG

GGGAGCAAAC

>ASV993 GS|75.9|X99562_S000381430;k:Archaea,p:"Crenarchaeota",c:Thermoprotei,o:Desulfurococcales,f:Desulfurococcaceae,g:Ignicoccus;

CTTGGTCATTTAGAGGAAGTAAAAGTCGTAACAAGGTTTCCGTAGGTGAACCTGCGGAAG

GATCATTACCGAGATAGGGTCCCCCGGGCCCGACCCTCCACCCGCTGCGTACCTACCTTT

TGTTGCTTTGGCGGGCCGCGGGGCCCCCGGCCCCCCGTCGACCCCGGTGGGCGAGCGCCC

GCCAGAGACCCCCCCAACCCGGTTGATCAGTGACGTCCGAGCCCACGAGACATCTCAGGA

TCTCGTATGC

>ASV994 GS|0.0|None;No hit

GTGTCAGCAGCCGAATGGAAGGCAAAAGTGTGGGAATTGAGGAAGGATATGAAATACTGT

ACGGAGTAGAATAGGCTGATTGAACGGTAGTACTTATAAAGGGTTTTTTTAGGCGTGCTG

TTGTCTTATAAGGAAACAAAAATCTAAGTTGAAGAATTATCTGCGAAACCGACAGGTATA

GAATTTATAGTACGAAAAATGTAGTTATAAAATAAAAGTATTTAATGTAGAATAAAGGAT

GTCTAAGCTG

>ASV995 SS|1.0000|EU622978_S001098404;k:Bacteria,p:"Deinococcus-Thermus",c:Deinococci,o:Deinococcales,f:Deinococcaceae,g:Deinococcus

TACGGAGGGTGCAAGCGTTACCCGGAATCACTGGGCGTAAAGGGCGTGTAGGCGGCTGTC

CAAGTCTGGTTTTAAAGACTGCGGCTCAACCGCAGAACTGGACTGGAGACTGGACGGCTA

GACCTCTGGAGAGGAAGCTGGAATTCCTGGTGTAGCGGTGGAATGCGTAGATACCAGGAG

GAACACCAATGGCGAAGGCAGGCTTCTGGACAGAAGGTGACGCTGAGGCGCGAAAGTGTG

GGGAGCGAAC

>ASV996 GS|0.0|None;No hit

GTGTCAGCAGCCACATCCTCATCAAGCAAACAACGAAGAGGGATGCTGAGCCGACAGCAA

TCGGCGTCTGCAACGCCGCAGGGTCACGATGTCAACAACCCCCCATCTGTCCCCGGTAGT

CC

>ASV997 SS|0.9800|CP000473_S002290405;k:Bacteria,p:"Acidobacteria",c:Acidobacteria_Gp3

TACGTAGGCAGCAAGCGTTGTTCGGAGTTACTGGGCGTAAAGGGTGTGTAGGCGGCCTTC

TAAGTTTGGTGTGAAATCTCCCGGCTCAACCGGGAGGGTGCGCCGAAGACTGGGGGGCTA

GAGTATGGGATGGGAAAGTGGAATTCCTGGTGTAGCGGTGAAATGCGTAGATATCAGGAG

GAACACCTGCGGTGTAGACGGCTTTCTGGACCATAACTGACGCTGAGACACGAAAGCGTG

GGTAGCAAAC

>ASV998 GS|100.0|D83358_S000413959;k:Bacteria,p:Firmicutes,c:Bacilli,o:Bacillales,f:Staphylococcaceae,g:Staphylococcus;

TACGTAGGTGGCAAGCGTTATCCGGAATTATTGGGCGTAAAGCGCGCGTAGGCGGTTTTT

TAAGTCTGATGTGAAAGCCCACGGCTCAACCGTGGAGGGTCATTGGAAACTGAAAAACTT

GAGTGCAGAAGAGGAAAGTGGAATTCCATGTGTAGCGGTGAAATGCGCAGAGATATGGAG

GAACACCAGTGGCGAAGGCGACTTTCTGGTCTGTAACTGACGCTGATGTGCGAAAGCGTG

GGGATCAAAC

>ASV999 SS|1.0000|FJ817379_S001351635;k:Bacteria,p:"Actinobacteria",c:Actinobacteria,o:Actinomycetales

TACGTAGGGTGCGAGCGTTGTCCGGAATTATTGGGCGTAAAGAGCTTGTAGGTGGTCCTT

CACGTCCGCTGTGAAAACTCAGGGCTCAACCCTGAGCTTGCAGTGGATACGGGAGGACTC

GAGTTCGGCTGGGGAGACTGGAACTCCTGGTGTAGCGGTGGAATGCGCAGATATCAGGAA

GAACACCGGTGGCGAAGGCGGGTCTCTGAACCGATACTGACACTGAGAAGCGAAAGCGTG

GGGAGCAAAC

>ASV1000 GS|0.0|None;No hit

GACGGTAAAGACTAGTGTTATTCATCTTTAATAGGTTTAAAGGGTACCTAGACGGTGGAT

CTTGCCCCTAAAAGGAACGGATCTTACTAGAGTTTTATGGAAGAGGTTAGATTTAGTATT

AACGGTGGAGAGATGAAATTCTTTGATACTGTTAGGAACTGGTAACGGCGAAGGCGATCC

TCTAAGTAAAAACTGACGTTGAGGGACGAAGGCTTGGGTAGCGAACAGG

>ASV1001 SS|1.0000|EF635408_S001095322;k:Bacteria,p:"Deinococcus-Thermus",c:Deinococci,o:Deinococcales,f:Deinococcaceae,g:Deinococcus

TACGGAGGGTGCAAGCGTTACCCGGAATCACTGGGCGTAAAGGGCGTGTAGGCGGTTTGC

CAAGTCTGACTTTAAAGACCGAAGCTCAACTTCGGGCCTGGGTTGGAGACTGGCAGACTA

GACGGATGGAGAGGTCACTGGAATTCCTGGTGTAGCGGTGGAATGCGTAGATACCAGGAG

GAACACCAACGGCGAAGGCAGGTGACTGGACATTTAGTGACGCTGAGGCGCGAAAGTGTG

GGGAGCAAAC

>ASV1002 GS|98.4|DQ321750_S000636629;k:Bacteria,p:"Actinobacteria",c:Actinobacteria,o:Actinomycetales,f:Nakamurellaceae,g:Nakamurella;

TACGTAGGGTGCAAGCGTTGTCCGGAATTATTGGGCGTAAAGAGCTCGTAGGCGGTCTGT

CGCGTCGAATGTGAAAACCCGGGGCTTAACCCCGGGCCTGCATTCGATACGGGCAGACTA

GAGTTCGGTAGGGGAGACTGGAATTCCTGGTGTAGCGGTGAAATGCGCAGATATCAGGAG

GAACACCGGTGGCGAAGGCGGGTCTCTGGGCCGATACTGACGCTGAGGAGCGAAAGCGTG

GGGAGCAAAC

>ASV1003 SS|1.0000|AB362219_S001043921;k:Bacteria,p:"Proteobacteria",c:Alphaproteobacteria,o:Rhodospirillales,f:Acetobacteraceae

TACGAAGGGGGCTAGCGTTGCTCGGAATGACTGGGCGTAAAGGGCGCGTAGGCGGCTTGG

TCGGTCAGACGTGAAATTCCTGGGCTCAACCTGGGGGCTGCGTTTGATACAGCTGGGCTA

GAGTGGGGAAGAGGGTTGTGGAATTCCCAGTGTAGAGGTGAAATTCGTAGATATTGGGAA

GAACACCGGTGGCGAAGGCGGCAACCTGGTCCTTGACTGACGCTGAGGCGCGAAAGCGTG

GGGAGCAAAC

>ASV1004 GSL|97.2|AB021390_S000015591;k:Bacteria,p:"Proteobacteria",c:Betaproteobacteria,o:Burkholderiales

TACGTAGGGTGCGAGCGTTAATCGGAATTACTGGGCGTAAAGCGTGCGCAGGCGGTTATG

CAAGACAGATGTGAAATCCCCGGGCTCAACCTGGGAATTGCATCAGTGACTGCATAGCTT

GAGTGCGGCAGAGGGGGATGGAATTCCGCGTGTAGCAGTGAAATGCGTAGATATGCGGAG

GAACACCAATGGCGAAGGCAATCCCCTGGGCCTGCACTGACGCTCATGCACGAAAGCGTG

GGGAGCAAAC

>ASV1005 GS|78.4|AJ312385_S000087730;k:Bacteria,p:Firmicutes,c:Clostridia,o:Clostridiales,f:Lachnospiraceae,g:Roseburia;

TACGTAGGTGGCAAGCGTTGTCCGGATTTACTGGGTGTAAAGGGTGCGCAGGCGGACCTT

TAAGTAGAAAGTGAAAGGTTGGAGCTCAACTCCGACACTGCTTCCTATACTGGGGGTCTT

GAGTTTCGGAGGGGGAAGCGGAACGACACGTGTAGCGGTGAAATGCGTTGATATGTGTCG

GAACACCAATGGCGAAAGCAGCTTCCTGGACGAATACTGACGCTCAGGCACGAAAGCCAA

GGTAGCAAAC

>ASV1006 GS|100.0|AB021405_S000010261;k:Bacteria,p:"Proteobacteria",c:Gammaproteobacteria,o:Xanthomonadales,f:Xanthomonadaceae,g:Stenotrophomonas;

TACGAAGGGTGCAAGCGTTACTCGGAATTACTGGGCGTAAAGCGTGCGTAGGTGGTCGTT

TAAGTCCGTTGTGAAAGCCCTGGGCTCAACCTGGGAACTGCAGTGGATACTGGGCGACTA

GAATGTGGTAGAGGGTAGCGGAATTCCTGGTGTAGCAGTGAAATGCGTAGAGATCAGGAG

GAACATCCATGGCGAAGGCAGCTACCTGGACCAACATTGACACTGAGGCACGAAAGCGTG

GGGAGCAAAC

>ASV1007 GS|0.0|None;No hit

GTGTCAGCAGCCGCAGAAGAATCTGTCGTCCTCGTGAATGTCGAAAACGTATTTACCGGT

CGCTGCTGCACCGAGGAGATAGCCTGCTGTTGTATGCATTACACCCTTCGGTTTACCTGT

TGACCCAGAGGTATAGAGCAGGAAAAGCGGGTCTTCCGAGTTCATAGACTCCGGCGGAAT

GTAGTTTGGATACTTCTCCAGCTCTTCATGCCACCACCAATCGCGACCGTTCGTCCAGGG

AACATCGGCG

>ASV1008 GS|0.0|None;No hit

GTGTCAGCAGCCGCAGCATCAGCCATCACCTGAGGTCCAAGCCAGTGGCGTTGAAGAAGC

CCCTCTGTATGTGAACGCCAAACAATTTCACCGAATATTGAAGCGGCGGGTCGCGCGACA

AAAATTGGAAGAAGCCCTCCGCCTTACATCCAAGGGCCGAAAACCATACCTCCATGAGTC

GCGACACAAACATGCGATGCGCAGACCTCGAGGCCCGGGTAGTCC

>ASV1009 GS|97.5|AY043938_S000354314;k:Bacteria,p:"Verrucomicrobia",c:Spartobacteria,g:Spartobacteria_genera_incertae_sedis;

TACAGAGGTCTCAAGCGTTGTTCGGATTCATTGGGCGTAAAGGGTGCGTAGGTGGCGCCG

TAAGTGGGGTGTGAAATTTCGGAGCTTAACTCCGAAACTGCATTCCATACTGCGGTGCTT

GAGGACTGGAGAGGAGACTGGAATTTACGGTGTAGCAGTGAAATGCGTAGAGATCGTAAG

GAAGACCAGTGGCGAAGGCGGGTCTCTGGACAGTTCCTGACACTGAGGCACGAAGGCTAG

GGGAGCAAAC

>ASV1010 SS|0.9700|JQ309130_S003619637;k:Bacteria,p:"Acidobacteria",c:Acidobacteria_Gp4,g:Blastocatella

TACGTAGGGACCAAGCGTTGTTCGGATTTACTGGGCGTAAAGGGCGCGTAGGCGGCGTAA

CAAGTCAGTTGTGAAATCTCCGAGCTTAACTCGGAACGGTCAACTGATACTGTTATGCTA

GAGTACAGAAGGGGCAATCGGAATTCTTGGTGTAGCGGTGAAATGCGTAGATATCAAGAG

GAACACCTGAGGTGAAGACGGGTTGCTGGGCTGATACTGACGCTGAGGCGCGAAAGCTAG

GGTAGCAAAC

>ASV1011 GS|0.0|None;No hit

GTGTCAGCAGCCGCAAGGAAACTTTGGGTTTCAGACGTATCGCGGTCCCCCAATCCTAAC

GGCTCGTTCAATTGGGGATCCCCGTTGGTAACCGTCGAAACGCCAGAGAAGTAGGAGTCC

GAGCGCTCCCTGGGTGGCCTGACCAAAGACGCAGTTGGGTCTTGAATGAACCTTTCAACC

TCTGGTTGAGTAGGAGTTGTAGGGCTGCTAGATATTGCGCTTGCACTACCATCGGTGTCG

TATCTGAAGC

>ASV1012 GS|100.0|JN160681_S002916632;k:Bacteria,p:"Actinobacteria",c:Actinobacteria,o:Actinomycetales,f:Intrasporangiaceae,g:Janibacter;

TACGTAGGGTGCGAGCGTTGTCCGGAATTATTGGGCGTAAAGAGCTTGTAGGCGGTCTGT

CGCGTCTGCTGTGAAAATCCGGGGCTCAACCCCGGACTTGCAGTGGGTACGGGCAGACTA

GAGTGTGGTAGGGGAGACTGGAATTCCTGGTGTAGCGGTGAAATGCGCAGATATCAGGAG

GAACACCGATGGCGAAGGCAGGTCTCTGGGCCACTACTGACGCTGAGAAGCGAAAGCATG

GGGAGCGAAC

>ASV1013 SS|0.8000|FJ479425_S001235278;k:Bacteria,p:"Armatimonadetes"

GACGTAGGGGGCGAGCGTTGTCCGGAATTACTGGGCGTCAAGCGCGTGTAGGCGGGCCGG

TAGGCGGCGTGTGAAAGGCGGGGGCTCAACCCCCGCATGTCGCGCCGAACCGCCGGTCTT

GGGACGGCGAGGGGCAACTGGAATGGTCGGAGGAGCGGTGAAATGCGTAGAGCCCGATCA

GAACACCGGTGGCGAAGGCGGGTTGCTGGAGCCGGGCCGACGCTGAGACGCGAAAGCGCG

GGGAGCGAAC

>ASV1014 SS|1.0000|EF457420_S000834845;k:Bacteria

TACGTGGGAGGCGAGCGTTGTCCGGAATTACTGGGCGTGAAGAGCGCGTAGGCGGCGCGG

TGCGACCTGTGTGAAAGCCCCCGGCCCAACCGGGGAGGGTCACAGGTTACGGCCGTGCTC

GGAGTGCGGAAGAGGGGAGTGGAACTCCGGGAGTAGCGGTGAAATGCGTAGATACCCGGA

GGAACACCAGTGGCGAAGGCGGCTCCCTGGTCCGCGACTGACGCTGTGGCGCGAAAGCTG

GGGGAGCGAA

>ASV1015 GS|0.0|None;No hit

GTGTCAGCAGCCGCGTTTGCCTTGTCCCGTTCGATCTGGCTTAAGAGATGAGCACCTTCA

GATCTTCGATTGCTTAGGAAAGTCTCGACGTTCTTCTCGAGGCTCTCCACGGTGTCCTTC

ATTTCAGAGTAGAAGCTTTGAGCCCTCATCAGACCATCGATAAGGTCGTTAAAGGCTTGG

AAGATCTTCCTGTATCTTGTTAAGACCATGGTGCGCTGCCGGTTATAGCTTTCGTATCTG

CTCTGTTCGG

>ASV1016 GS|99.2|KF387628_S004052143;k:Bacteria,p:"Proteobacteria",c:Gammaproteobacteria,o:Xanthomonadales,f:Xanthomonadaceae,g:Pseudoxanthomonas;

TACGAAGGGTGCAAGCGTTACTCGGAATTACTGGGCGTAAAGCGTGCGTAGGTGGTTGTT

TAAGTCTGTTGTGAAAGCCCTGGGCTCAACCTGGGAATTGCAATGGATACTGGGCGACTA

GAGTGTGGTAGAGGACAGTGGAATTTCCGGTGTAGCAGTGAAATGCGTAGAGATCGGAAG

GAACATCTGTGGCGAAGGCGACTGTCTGGGCCAACACTGACACTGAGGCACGAAAGCGTG

GGGAGCAAAC

>ASV1017 GSL|99.2|AF144383_S000388105;k:Bacteria,p:"Proteobacteria",c:Betaproteobacteria,o:Burkholderiales,f:Comamonadaceae

TACGTAGGGTGCAAGCGTTAATCGGAATTACTGGGCGTAAAGCGTGCGCAGGCGGTGATG

TAAGACAGATGTGAAATCCCCGGGCTCAACCTGGGAACTGCATTTGTGACTGCATCGCTG

GAGTGCGGCAGAGGGGGATAGAATTCCGCGTGTAGCAGTGAAATGCGTAGATATGCGGAG

GAACACCGATGGCGAAGGCAATCCCCTGGGCCTGCACTGACGCTCATGCACGAAAGCGTG

GGGAGCAAAC

>ASV1018 GS|0.0|None;No hit

GTGTCAGCAGCCTGGAAAGTACGCTGGATCTGGGGCGCCTCCTGCACCTGGGTGGCGTGC

TTGAACTTTCCAAATGATTATTTTCCGAAGTTTGGCAGGGGCATCTTGTAATGACCATGA

TGGTAAATGATTGGTATGGACTTTCGTAGCCGTTTGTCTTACTCGTGGAGTGGCCTCGAG

CATGTGATAATTAAGGTAAGTCCTCGGTCGTAGTATTTAGAAATGAATCATCTTCATATA

TACTGTCGCC

>ASV1019 GS|97.5|AY043938_S000354314;k:Bacteria,p:"Verrucomicrobia",c:Spartobacteria,g:Spartobacteria_genera_incertae_sedis;

TACAGAGGTCCCAAGCGTTGTTCGGATTCATTGGGCGTAAAGGGTGCGTAGGTGGCGTGG

TAAGTCGGGTGTGAAATTTCGGAGCTTAACTCCGAAACTGCATTCGATACTGCCGTGCTT

GAGGACTGGAGAGGAGACTGGAATTTACGGTGTAGCAGTGAAATGCGTAGAGATCGTAAG

GAAGACCAGTGGCGAAGGCGGGTCTCTGGACAGTTCCTGACACTGAGGCACGAAGGCTAG

GGGAGCAAAC

>ASV1020 SS|1.0000|AB778531_U010573768;k:Bacteria,p:"Proteobacteria",c:Alphaproteobacteria,o:Rhodospirillales,f:Acetobacteraceae

TACGAAGGGGGCTAGCGTTGCTCGGAATGACTGGGCGTAAAGGGCGCGTAGGCGGATTGA

TCAGTCAGATGTGAAATTCCTGGGCTTAACCTGGGGGCTGCATTTGAGACGGTTAGTCTA

GAGTGTGAAAGAGGGTCGTGGAATTTCCAGTGTAGAGGTGAAATTCGTAGATATTGGGAA

GAACACCGGTGGCGAAGGCGGCGACCTGGTTCATAACTGACGCTGAGGCGCGAAAGCGTG

GGGAGCAAAC

>ASV1021 GS|97.6|JQ309130_S003619637;k:Bacteria,p:"Acidobacteria",c:Acidobacteria_Gp4,g:Blastocatella;

TACGTAGGGACCAAGCGTTGTTCGGATTTACTGGGCGTAAAGGGCGCGTAGGCGGCAATT

CAAGTCAGTTGTGAAATCTCCGAGCTTAACTCGGAACGGTCAACTGATACTGCTTTGCTA

GAGTACAGAAGGGGCAATCGGAATTCTTGGTGTAGCGGTGAAATGCGTAGATATCAAGAG

GAACACCAGAGGCGAAGGCGGATTGCTGGGCTGATACTGACGCTGAGGCGCGAAAGCTAG

GGTAGCAAAC

>ASV1022 GS|0.0|None;No hit

GTGTCAGCAGCCAAGCATCGATTTCGGCTCGAGCCTCAGATACGGCAGTGCAATGGATGG

GGGTAGAGATGTTAAAACTGACAGCCCCAGTGATCTCTGCACAAGGCATCGGGAAGATCG

TCCACTCAAGCTGGTTCTCAAGCTTGTTTATAGTGTAGCGGATACCCCGGTAGTCC

>ASV1023 SS|1.0000|HM032898_S002167666;k:Bacteria,p:"Bacteroidetes",c:Cytophagia,o:Cytophagales,f:Cytophagaceae,g:Hymenobacter

TACGGAGGGTGCGAGCGTTGTCCGGATTTATTGGGTTTAAAGGGTGCGTAGGCGGCTTTT

TAAGTCTAGGGTGAAAGCCCGCTGCTCAACAGCGGAACTGCCCTGGATACTGGAGAGCTT

GAGTACAGACGAGGTTGGCGGAATGGACAGAGTAGCGGTGAAATGCATAGATACCGTCCA

GAACCCCGATTGCGAAGGCAGCTGACTAGGCTGTTACTGACGCTGAGGCACGAAAGCGTG

GGGAGCGAAC

>ASV1024 GS|99.2|AJ009957_S000012709;k:Bacteria,p:"Proteobacteria",c:Alphaproteobacteria,o:Caulobacterales,f:Caulobacteraceae,g:Caulobacter;

TACGAAGGGGGCTAGCGTTGCTCGGAATTACTGGGCGTAAAGGGAGCGTAGGCGGACTGT

TAAGTTAGAGGTGAAAGCCCAGGGCTCAACCTTGGAATTGCCTTTGATACTGGCAGTCTT

GAGTACGGAAGAGGTATGTGGAACTCCGAGTGTAGAGGTGAAATTCGTAGATATTCGGAA

GAACACCAGTGGCGAAGGCGACATACTGGTCCGTTACTGACGCTGAGGCTCGAAAGCGTG

GGGAGCAAAC

>ASV1025 SS|0.9000|AJ289983_S000020777;k:Bacteria,p:"Verrucomicrobia",c:Spartobacteria,g:Spartobacteria_genera_incertae_sedis

TACAGAGGCCTCAAGCGTTGTTCGGATTCATTGGGCGTAAAGGGAGCGTAGGCGGTCGGG

TAAGTCGGATGTGAAATCCTGGGGCTCAACCTCAGAACTGCATTCGATACTGCTTGGCTA

GAGGACTGGAGAGGTGAGTGGAATTCACGGTGTAGCAGTGAAATGCGTAGATATCGTGAG

GAAGACCAGCGGCGAAGGCGGCTCACTGGACAGTTTCTGACGCTGATGCTCGAAGGCCAG

GGGAGCAAAC

>ASV1026 GS|97.6|AB594446_S002949232;k:Bacteria,p:"Actinobacteria",c:Actinobacteria,o:Actinomycetales,f:Dermacoccaceae,g:Branchiibius;

TACGTAGGGTGCGAGCGTTGTCCGGAATTATTGGGCGTAAAGAGCTTGTAGGCGGTTTGT

CACGTTCGCTGTGAAAGCCCGGGGCTTAACTCCGGGTCTGCAGTGGATACGGGCAGGCTA

GAGTATGGTAGGGGAGACTGGAATTCCTGGTGTAGCGGTGAAATGCGCAGATATCAGGAG

GAACACCGATGGCGAAGGCAGGTCTCTGGGCCATTACTGACGCTGAGAAGCGAAAGCATG

GGGAGCAAAC

>ASV1027 SS|0.9900|AB251884_S000650634;k:Bacteria,p:"Bacteroidetes",c:Cytophagia,o:Cytophagales,f:Cytophagaceae,g:Hymenobacter

TACGGAGGGTGCGAGCGTTGTCCGGATTTATTGGGTTTAAAGGGTGCGTAGGCGGCTGGT

TAAGTCCGGGGTGAAAGCCCGTTGCTCAACAACGGAACGGCCCTGGATACTGGCCAGCTT

GAATCCAGTCGAGGGTGGCGGAATAGAGGCTGTAGCGGTGAAATGCATAGATAGCCTCTA

GAACCCCAATTGCGTAGGCAGCTGCCTAGACTGGCATTGACGCTCAGGCACGAAAGCGTG

GGGAGCGAAC

>ASV1028 GS|100.0|AF064460_S000004663;k:Bacteria,p:"Proteobacteria",c:Gammaproteobacteria,o:Pseudomonadales,f:Pseudomonadaceae,g:Pseudomonas;

TACAGAGGGTGCAAGCGTTAATCGGAATTACTGGGCGTAAAGCGCGCGTAGGTGGTTAGT

TAAGTTGGATGTGAAATCCCCGGGCTCAACCTGGGAACTGCATTCAAAACTGACTGACTA

GAGTATGGTAGAGGGTGGTGGAATTTCCTGTGTAGCGGTGAAATGCGTAGATATAGGAAG

GAACACCAGTGGCGAAGGCGACCACCTGGACTGATACTGACACTGAGGTGCGAAAGCGTG

GGGAGCAAAC

>ASV1029 GS|0.0|None;No hit

GTGTCAGCAGCCGCTGTCCAACATCTCAGCCATCTCGTCATCTGGTAGAGTGGCTTCGCC

CTCATCAAAGCTTCTGTCAACGGCGTTCCTCAATTCATCATTTTCCCCGAGAGCTACAGC

CTATGCCTCGCTTACATACTTGAGCCAACCAACGGGTGGATCGTCTAACAGCCTAAGCCA

TGCCCCAATCGGTTACAATTATACAGCAAAACAAGGCCCCACCGTTTCGAGAAGTAGGAT

AACCGGCATC

>ASV1030 GS|100.0|FJ194436_S001188468;k:Bacteria,p:"Proteobacteria",c:Alphaproteobacteria,o:Sphingomonadales,f:Sphingomonadaceae,g:Sphingomonas;

TACGGAGGGGGCTAGCGTTGTTCGGAATTACTGGGCGTAAAGCGCACGTAGGCGGCTTTG

TAAGTTAGAGGTGAAAGCCTGGAGCTCAACTCCAGAACTGCCTTTAAGACTGCATCGCTT

GAATCCAGGAGAGGTGAGTGGAATTCCGAGTGTAGAGGTGAAATTCGTAGATATTCGGAA

GAACACCAGTGGCGAAGGCGGCTCACTGGACTGGTATTGACGCTGAGGTGCGAAAGCGTG

GGGAGCAAAC

>ASV1031 SS|1.0000|AB245334_S000627870;k:Bacteria,p:"Actinobacteria"

TACGTAGGGGGCTAGCGTTGTCCGGAATCATTGGGCGTAAAGAGCGTGTAGGCGGTCCGG

TAAGTCCGTTGTGAAAGTCAAGGGCTCAACCCTTGAATGCCGGCGGATACTGTCGGGCTA

GAGTCCGGAAGAGGCGAGTGGAATTCCCGGTGTAGCGGTGAAATGCGCAGATATCGGGAG

GAACACCTATGGCGAAGGCAGCTCGCTGGGACGGTACTGACGCTGAGACGCGAAAGCGTG

GGGAGCAAAC

>ASV1032 SS|0.9000|X75272_S000016392;k:Bacteria,p:Firmicutes

TACGTAGGTGGCAAGCGTTGTCCGGATTTACTGGGCGTAAAGCGTATGCAGGCGGATACT

TAAGTAGAGAGTGAAAGGCGGGAGCTCAACTCCTGGACTGCTCCCTATACTGGGTGTCTT

GAGTGGCGGAGAGGAAGATGGAACTCCATGTGTAGCGGTGAAATGCGTAGATATATGGAG

GAACACCAATGGCGAAAGCAATCTTCTGGACGCAAACTGACGCTGAGATACGAAAGCCAT

GGTAGCGAAC

>ASV1033 SS|1.0000|AY140238_S000397413;k:Bacteria,p:"Proteobacteria",c:Alphaproteobacteria,o:Rhodospirillales,f:Acetobacteraceae

TACGAAGGGGGCTAGCGTTGCTCGGAATGACTGGGCGTAAAGGGCGCGTAGGCGGATTGG

TCAGTCGGGCGTGAAATTCCTGGGCTTAACCTGGGGGCTGCGTTCGAGACGGCTGGTCTA

GAGTTTGGAAGAGGGTCGTGGAATTCCCAGTGTAGAGGTGAAATTCGTAGATATTGGGAA

GAACACCGGTGGCGAAGGCGGCGACCTGGTCCTTGACTGACGCTGAGGCGCGAAAGCGTG

GGGAGCAAAC

>ASV1034 SS|0.9400|KJ155688_S004086269;k:Bacteria,p:"Bacteroidetes",c:Cytophagia,o:Cytophagales,f:Cytophagaceae,g:Spirosoma

TACGGAGGGTGCAAGCGTTGTCCGGATTTATTGGGTTTAAAGGGTGCGCAGGTGGTTCTG

TAAGTCTGATTTGAAAGCTAGCGGCTTAACCGTTAGATGTGGTTGGAAACTGTGGAACTT

GAATAGCGTAGCGGGAGCCGGAATGGGTCATGTAGCGGTGAAATGCATAGATATGACCCG

GAACACCGATTGCGAAGGCAGGCTCCTGGGCGCTGATTGACACTGAGGCACGAAAGCATG

GGTAGCGAAC

>ASV1035 GS|98.0|AJ429239_S000145113;k:Bacteria,p:"Proteobacteria",c:Alphaproteobacteria,o:Sphingomonadales,f:Sphingomonadaceae,g:Sphingomonas;

TACGGAGGGAGCTAGCGTTATTCGGAATTACTGGGCGTAAAGCGCACGTAGGCGGCTTTG

TAAGTAAGAGGTGAAAGCCTGGAGCTCAACTCCAGAATTGCCTTTTAGACTGCATCGCTC

GAATCATGGAGAGGTCAGTGGAATTCCGAGTGTAGAGGTGAAATTCGTAGATATTCGGAA

GAACACCAGTGGCGAAGGCGGCTGACTGGACATGTATTGACGCTGAGGTGCGAAAGCGTG

GGGAGCAAAC

>ASV1036 GS|98.8|EF466117_S000843257;k:Bacteria,p:"Actinobacteria",c:Actinobacteria,o:Actinomycetales,f:Nocardioidaceae,g:Nocardioides;

TACGTAGGGTGCGAGCGTTGTCCGGAATTATTGGGCGTAAAGGGCTCGTAGGCGGTTTGT

CACGTCGAAAGTGAAAACTCAGGGCTTAACCCTGAGCCTGCTTCCGATACGGGCAGACTA

GAGGTATGCAGGGGAGAACGGAATTCCTGGTGTAGCGGTGAAATGCGCAGATATCAGGAG

GAACACCGGTGGCGAAGGCGGTTCTCTGGGCATTACCTGACGCTGAGGAGCGAAAGTGTG

GGGAGCGAAC

>ASV1037 SS|0.8800|EF516651_S000841093;k:Bacteria,p:"Armatimonadetes",c:Armatimonadia,o:Armatimonadales,f:Armatimonadaceae,g:Armatimonas/Armatimonadetes_gp1

TACGTAGGGGGCGAGCGTTGTCCGAAGTTACTGGGCGTAAAGCGCGTGTAGGCGGTCTTT

TAAGTTTGGGGTGAAAGGTTCACGGCTCAACCGGAACAGTGCCTTGAAAACTGGGAGACT

TGAATGTGGCAGGGGAAAGCGGAATTCCAGGTGTAGCGGTGAAATGCGTAGATATCTGGA

GGAACACCGATGGCGAAGGCAGCTTTCTGGGCTAACATTGACGCTGAGACGCGAAAGCGT

GGGGAGCGAA

>ASV1038 SS|0.8100|CP001854_S001872674;k:Bacteria,p:"Actinobacteria",c:Actinobacteria,o:Solirubrobacterales,f:Conexibacteraceae,g:Conexibacter

TACGTAGGGGGCAAGCGTTGTCCGGAATCATTGGGCGTAAAGCGCGTGTAGGCGGGTCGT

TAAGTCTGCTCTGAAAGTCCAAGGCTCAACCTTGGGATGCGGGTGGATACTGGCGACCTC

GAGTCCGGAAGAGGCGGGTGGAATTCCTGGTGTAGCGGTGAAATGCGCAGATATCAGGAG

GAACACCAATGGCGAAGGCAGCCCGCTGGGACGTGACTGACGCTGAGACGCGAAAGCGTG

GGGAGCAAAC

>ASV1039 SS|0.9800|KF999686_S004084195;k:Bacteria,p:"Bacteroidetes",c:Cytophagia,o:Cytophagales,f:Cytophagaceae,g:Spirosoma

TACGGAGGGTGCAAGCGTTGTCCGGATTTATTGGGTTTAAAGGGTGCGTAGGTGGGTCTT

TAAGTCTGGTTTGAAAGCAGGCGGCTCAACCGTCTGATGTGGCTGGAAACTGGGGGTCTT

GAATGGGATGGCGGCTGCCGGAACGGGTCATGTAGCGGTGAAATGCATAGATATGACCCA

GAACACCGATTGCGAAGGCAGGCAGCTAGGTCCTGATTGACACTGAGGCACGAGAGCATG

GGGAGCCAAC

>ASV1040 GS|100.0|AY362908_S000368472;k:Bacteria,p:"Proteobacteria",c:Gammaproteobacteria,o:Pasteurellales,f:Pasteurellaceae,g:Haemophilus;

TACGGAGGGTGCGAGCGTTAATCGGAATAACTGGGCGTAAAGGGCACGCAGGCGGTGACT

TAAGTGAGGTGTGAAAGCCCCGGGCTTAACCTGGGAATTGCATTTCATACTGGGTCGCTA

GAGTACTTTAGGGAGGGGTAGAATTCCACGTGTAGCGGTGAAATGCGTAGAGATGTGGAG

GAATACCGAAGGCGAAGGCAGCCCCTTGGGAATGTACTGACGCTCATGTGCGAAAGCGTG

GGGAGCAAAC

>ASV1041 SS|1.0000|AB545808_S002235816;k:Bacteria,p:"Bacteroidetes",c:Sphingobacteriia,o:"Sphingobacteriales",f:"Rhodothermaceae"

TACGGAGGGTCCAAGCGTTGTCCGGAATCACTGGGTGTAAAGGGTGCGCAGGCGGGCGTG

TAAGTCAGAGGTGAAAGCCACCGGCCTAACCGGTGAACGGCCTTTGATACTGCACGTCTT

GAGTCCCGGAGAGGCTATCGGAATTCGTGGTGTAGCGGTGAAATGCGTAAATATCACGAG

GAACACCGGATGCGTAGGCGGATAGCTGGACGGGTACTGACGCTCAGGCACGAAAGCGCG

GGGAGCAAAC

>ASV1042 GS|0.0|None;No hit

TACGTAGAAGACGTGCGTTATTCATCTTTATTAGGTTTAAAGGGTACCTAGACGGTTATT

TCAGTCGTAAAAGATACGTTTTAACTAGAGTTATATGTGGGGAGGCGAGAATTTAAAGAG

TAGAGTTGAAATTCTGTAATACTTTAAGGACTGGTCAAGGTGAAGGCGACCTCTTATGTA

ATAACTGACGTTGAGGGACGAAGGCTTGGGTAGCGAATAGG

>ASV1043 GS|0.0|None;No hit

GTGTCAGCAGCCCACGAACCCCCTATTTTCCATATCTTGGCACGCTAATGCATGCGCGGC

CCGTCAGATTGAAGCCGAGGTTCAACAGATCCCGACCTCCCATTTTCTAACCATGACCAG

CTCCTCAATGCCATTTGCGCGAATTGTGGTAATGAAAGGAGCTTGGAATATACGACGGTA

AGCTGAACAGAGCTGTTGGCCAGCCGAACTACCAAAGCGCTGCTCATCCCGCCCTCAACA

TGGTTCGAGA

>ASV1044 GS|0.0|None;No hit

GTGTCAGCAGCCGCCCGTGCACCTCCATTGGCGTCGCCATAAGGTGGCTGATATGCCTCT

GGAGTCGCTGGAAGCGTTCGATGCATGGTTGCGAGAACGGTGGAACGAGAAAGAAGTGCT

TCTAGAACAGCATGCCAAGACTGGCAGCTTCCCATCGGAATCGGAATCGGAACCGATCGT

CACGGAAGTGAAGTTGGGGCACTGGGTGGAACTTTGGGGCATCTGTGGCTTGCTGGCGAG

TCTAGCCATG

>ASV1045 GS|99.6|FR753034_S002442178;k:Bacteria,p:"Proteobacteria",c:Alphaproteobacteria,o:Rhizobiales,f:Bradyrhizobiaceae,g:Tardiphaga;

TACGAAGGGGGCTAGCGTTGCTCGGAATCACTGGGCGTAAAGGGTGCGTAGGCGGGTTTT

TAAGTCAGAGGTGAAATCCTGGAGCTCAACTCCAGAACTGCCTTTGATACTGAAAGTCTT

GAGTATGGGAGAGGTGAGTGGAACTGCGAGTGTAGAGGTGAAATTCGTAGATATTCGCAA

GAACACCAGTGGCGAAGGCGGCTCACTGGCCCATTACTGACGCTGAGGCACGAAAGCGTG

GGGAGCAAAC

>ASV1046 GSL|97.2|AB196784_S000568191;k:Bacteria,p:Firmicutes,c:Bacilli,o:Bacillales

TACGTAGGTGGCAAGCGTTGTCCGGAATTATTGGGCGTAAAGCGCGCGCAGGCGGTCTCT

TAAGTCTGATGTGAAATCTCGCGGCTCAACCGCGAGCGGCCATTGGAAACTGGGAGGCTT

GAGTGCAGAAGAGGAGAGTGGAATTCCATGTGTAGCGGTGAAATGCGTAGATATATGGAG

GAACACCAGTGGCGAAGGCGACTCTCTGGTCTGTAACTGACGCTGAGGCGCGAAAGCGTG

GGGAGCAAAC

>ASV1047 GS|78.8|EF515236_S000839820;k:Bacteria,p:"Armatimonadetes",c:Chthonomonadetes,o:Chthonomonadales,f:Chthonomonadaceae,g:Chthonomonas/Armatimonadetes_gp3;

TACGTAGGTGGCAAGCGTTGTCCGGATTTACTGGGCGTAAAGCGAACGCAGGCGGACTGT

TAAGTAGGAAGTGAAAGGTTGCAGCTCAACTGCGACACTGCTTCTTATACTGGCAGTCTT

GAGTAGCGGAGAGGGAGATGGAACGACACGTGTAGCGGTGAAATGCGTTGATATGTGTCG

GAACACCAATGGCGAAAGCAATCTCCTGGACGCAGACTGACGCTGAGGTTCGAAAGCCAA

GGTAGCAAAC

>ASV1048 GS|79.6|AJ009456_S000115949;k:Bacteria,p:"Armatimonadetes",c:Chthonomonadetes,o:Chthonomonadales,f:Chthonomonadaceae,g:Chthonomonas/Armatimonadetes_gp3;

TACGTAGGTGGCAAGCGTTGTCCGGATTTACTGGGCGTAAAGGGCAGGCAGGCGGACTGT

TAAGTAGAAAGTGAAAGGTTGGAGCTCAACTCCAACATTGCTTTCTATACTGGCAGTCTT

GAATCCCGGAGGGGAAAGCGGAACGACACGTGTAGCGGTGAAATGCGTTGATATGTGTCG

GAACACCAATGGCGAAGGCAGCTTTCTGGACGGGGATTGACGCTCATCTGCGAAAGCCGA

GGTAGCGAAC

>ASV1049 GS|0.0|None;No hit

GTGTCAGCAGCAGCAGGGTCAGCGCGTCTACCATGGTGAGGCAGATGAAAACAAGAATCT

TGATCCTGATAATAATGGTTATGAGAACGACCTCGGCGCCCCAGAGTCTGCGGAACAGGA

AGAATCTCATACGAATGACTACTACGATGGTCAGAATGAACCTGACGTAAAAGGATATTT

CCTGCGAACCCCGTAGTCC

>ASV1050 GS|0.0|None;No hit

GTGTCAGCAGCGAGCAGACCTTCATTGCCTGACAGCTCTGCTCATAGTCAACATGTTTCT

TACCGATCTACGCCGGGGGAGCCCTCGTCACAGCCGCATGGCGATGCGCCAGGGATACAT

ACCGGAAGCACGAGCCGCGGTGAGGCGAAATTCGACTATAGGCTTAACGACGCCCCACCA

GTCCCTATTCCGAATCTCGCGAAAGACTTCAGCGCTGGTGATTCGTATCATACTCCCACC

GAATCCGTCT

>ASV1051 SS|0.9700|AB072735_S000251887;k:Bacteria,p:"Gemmatimonadetes",c:Gemmatimonadetes,o:Gemmatimonadales,f:Gemmatimonadaceae,g:Gemmatimonas

TACAGAGGGTGCGAGCGTTGTCCGGAATCACTGGGCGTAAAGGGCGCGTAGGTGGTCCTG

TGCGCGTGCCGTGAAAGCCTGGGGCTCAACCCCAGGTCGGCGGTGCGAACGGCGGGACTG

GAGCATGCGAGAGGCAGGCGGAATTCCGGGTGTAGCGGTGGAATGCGTAGAGATCCGGAA

GAACACCGGGGGCGAAGGCGGCCTGCTGGCGCAGTAGCTGACACTGAGGCGCGACAGCGT

GGGGAGCAAA

>ASV1052 GS|98.0|AB018439_S000439484;k:Bacteria,p:"Proteobacteria",c:Alphaproteobacteria,o:Sphingomonadales,f:Sphingomonadaceae,g:Sphingomonas;

TACGGAGGGAGCTAGCGTTATTCGGAATTACTGGGCGTAAAGCGCACGTAGGCGGCTTTG

TAAGTTAGAGGTGAAAGCCCAGAGCTCAACTCTGGAATTGCCTTTAAGACTGCATCGCTT

GAATCCAGCAGAGGTGAGTGGAATTCCGAGTGTAGAGGTGAAATTCGTAGATATTCGGAA

GAACACCAGTGGCGAAGGCGGCTCACTGGACTGGTATTGACGCTGAGGTGCGAAAGCGTG

GGGAGCAAAC

>ASV1053 GS|97.6|AB251884_S000650634;k:Bacteria,p:"Bacteroidetes",c:Cytophagia,o:Cytophagales,f:Cytophagaceae,g:Hymenobacter;

TACGGAGGGTGCAAGCGTTGTCCGGATTTATTGGGTTTAAAGGGTGCGTAGGCGGCCGAT

TAAGTCTGGGGTGAAAGCCCGTTGCTCAACAACGGAACTGCCCTGGAAACTGGTTGGCTT

GAGTACAGACGAGGGTGGCGGAATGGACGGTGTAGCGGTGAAATGCATAGATACCGTCCA

GAACCCCGATTGCGAAGGCAGCTGCCTAGACTGTAACTGACGCTGAGGCACGAAAGCGTG

GGGAGCGAAC

>ASV1054 SS|0.8800|AB649056_S002949821;k:Bacteria,p:"Proteobacteria",c:Alphaproteobacteria,o:Sphingomonadales,f:Sphingomonadaceae

TACGGAGGGGGCTAGCGTTGTTCGGAATTACTGGGCGTAAAGCGTCCGTAGGCGGCTTGC

CAAGTCAGAGGTGAAATCCCACGACTCAATCGTGGAACTGCCTTTGAGACTGGTTCGCTT

GAAGATGGGAGAGGTTAGCGGAATTCCGAGTGTAGAGGTGAAATTCGTAGATATTCGGAA

GAACACCAGTGGCGAAGGCGGCTAACTGGACCATTCTTGACGCTGAGGGACGAAAGCGTG

GGGAGCAAAC

>ASV1055 SS|0.8900|X56305_S000006614;k:Bacteria,p:"Planctomycetes",c:Planctomycetia,o:Planctomycetales,f:Planctomycetaceae,g:Gemmata

GACGAACCGTGCGAACGTTATTCGGAATCACTGGGCTTAAAGGGAGCGTAGGCGGGCTAT

CAAGTCTGGGGTGAAATCCCACGGCTCAACCGTGGAACTGCCTCAGATACTGACGGCCTC

GAGGGAGATAGGGGCATGCGGAACTGTAGGTGGAGCGGTGAAATGCGTTGATATCTACAG

GAACTCCGGTGGCGAAAGCGGCGTGCTGGATCTCTTCTGACGCTGAGGCTCGAAAGCTAG

GGGAGCAAAC

>ASV1056 GS|98.8|AY218772_S000356842;k:Bacteria,p:"Verrucomicrobia",c:Spartobacteria,g:Spartobacteria_genera_incertae_sedis;

TACAGAGGTCCCAAGCGTTGTTCGGATTCATTGGGCGTAAAGGGTGCGTAGGTGGCGCCG

TCAGTGGGGTGTGAAATTTCGGAGCTTAACTCCGAAACTGCATTCCATACTGCGGTGCTC

GAGGACTGGAGAGGAGACTGGAATTCATGGTGTAGCAGTGAAATGCGTAGAGATCATGAG

GAAGACCAGTGGCGAAGGCGGGTCTCTGGACAGTTCCTGACACTGAGGCACGAAGGCTAG

GGGAGCGAAC

>ASV1057 GS|100.0|EF397574_S001044326;k:Bacteria,p:"Proteobacteria",c:Gammaproteobacteria,o:Xanthomonadales,f:Xanthomonadaceae,g:Dyella;

TACGAAGGGTGCAAGCGTTAATCGGAATTACTGGGCGTAAAGCGTGCGTAGGCGGTTTGT

TAAGTCTGCTGTGAAATCCCCGGGCTCAACCTGGGAATGGCAGTGGATACTGGCAAGCTA

GAGTGTGTCAGAGGGTGGTGGAATTCCCGGTGTAGCGGTGAAATGCGTAGAGATCGGGAG

GAACATCAGTGGCGAAGGCGGCCACCTGGGACAACACTGACGCTGAGGCACGAAAGCGTG

GGGAGCAAAC

>ASV1058 GS|0.0|None;No hit

TACGGGGGGGGCGAGCGTTATTCGAAATGATTGGGCGTAAAGAGCACGTAGACGGTTTTT

TAAGTGGACATTATATCTTTTTTGTTCTCTAAGGATAAAAAAGAAAGGATTATGGAAATA

TTTCTGTACTCGGGAAAAAGACCAAGGCTCAACCATGGTGTTTCCCGCTATACTATAAAA

CTAGAGTAAGTATGAGGAAAGTGGAATTCCTGGAGGAAAGGTTAAATTTTATGATATCAG

GAGGAACGCC

>ASV1059 GS|97.6|CP001854_S001872674;k:Bacteria,p:"Actinobacteria",c:Actinobacteria,o:Solirubrobacterales,f:Conexibacteraceae,g:Conexibacter;

TACGTAGGGGGCAAGCGTTGTCCGGAATCATTGGGCGTAAAGCGCGTGTAGGCGGCTCGG

TAAGTCTGCTGTGAAAGTCCAGGGCTCAACCCTGGAATGCCGGTGGATACTGTCGGGCTA

GAGTACGGAAGAGGCGAGTGGAATTCCTGGTGTAGCGGTGAAATGCGCAGATATCAGGAG

GAACACCAATGGCGAAGGCAGCTCGCTGGGACGTAACTGACGCTGAGACGCGAAAGCGTG

GGGAGCAAAC

>ASV1060 SS|1.0000|AB705486_S003658309;k:Bacteria,p:"Actinobacteria",c:Actinobacteria,o:Actinomycetales

TACGTAGGGTGCAAGCGTTGTCCGGATTTATTGGGCGTAAAGAGCTCGTAGGCGGTTTGT

CACGTCGGCTGTGAAAATCCGGGGCTCAACCCCGGACCTGCAGTCGATACGGGCAGACTA

GAGTTCGGCAGGGGAGACTGGAATTCCTGGTGTAGCGGTGAAATGCGCAGATATCAGGAG

GAACACCGGTGGCGAAGGCGGGTCTCTGGGCCGAAACTGACGCTGAGGAGCGAAAGCGTG

GGGAGCAAAC

>ASV1061 GSL|98.4|U49757_S000843932;k:Bacteria,p:"Proteobacteria",c:Betaproteobacteria,o:Burkholderiales,f:Oxalobacteraceae

TACGTAGGGTGCAAGCGTTAATCGGAATTACTGGGCGTAAAGCGTGCGCAGGCGGTTATG

TAAGACAGATGTGAAATGCCCGGGCTCAACCTGGGAACTGCATTTGTGACTGCATGGCTA

GAGTGTGTCAGAGGGGGGTAGAATTCCACGTGTAGCAGTGAAATGCGTAGATATGTGGAG

GAATACCGATGGCGAAGGCAGCCCCCTGGGATAACACTGACGCTCATGCACGAAAGCGTG

GGGAGCAAAC

>ASV1062 GS|100.0|DQ347958_S000641302;k:Bacteria,p:Cyanobacteria/Chloroplast,c:Chloroplast,f:Chloroplast,g:Streptophyta;

TACAGAGGATGCAAGCGTTATCCGGAATGATTGGGCGTAAAGCGTCTGTAGGTGGCTTTT

TAAGTCCGCCGTCAAATCCCAGGGCTCAACCCTGGACAGGCGGTGGAAACTACCAAGCTG

GAGTACGGTAGGGGCAGAGGGAATTTCCGGTGGAGCGGTGAAATGCGTAGAGATCGGAAA

GAACACCAACGGCGAAAGCACTCTGCTGGGCCGACACTGACACTGAGAGACGAAAGCTAG

GGGAGCGAAT

>ASV1063 SS|0.9900|EU861928_S001148233;k:Bacteria,p:"Armatimonadetes",c:Armatimonadia,o:Armatimonadales,f:Armatimonadaceae,g:Armatimonas/Armatimonadetes_gp1

TACGTAGGGGGCGAGCGTTGTCCGAAGTTACTGGGCGTAAAGAGCGCGTAGGCGGGTTTT

TAAGTGAGGGGTGAAATTCCGAGGCTCAACCTCGGAACTGCCTTTCATACTGGGAATCTT

GAGTGTGGGAGAGGCGAGTGGAATGGTCGGTGTAGCGGTGAAATGCGTAGATATCGATCG

GAACACCCATGGCGAAGGCAGCTCGCTGGCCCATAACTGACGCTGAGGCGCGAAAGCGTG

GGGAGCAAAC

>ASV1064 GS|99.6|HM583567_S002988298;k:Bacteria,p:"Proteobacteria",c:Betaproteobacteria,o:Burkholderiales,f:Comamonadaceae,g:Polaromonas;

TACGTAGGGTGCGAGCGTTAATCGGAATTACTGGGCGTAAAGCGTGCGCAGGCGGTGATG

CAAGACAGTTGTGAAATCCCCGGGCTCAACCTGGGAATTGCATCTGTGACTGCATCGCTA

GAGTACGGTAGAGGGGGATGGAATTCCGCGTGTAGCAGTGAAATGCGTAGATATGCGGAG

GAACACCGATGGCGAAGGCAATCCCCTGGACCTGTACTGACGCTCATGCACGAAAGCGTG

GGGAGCAAAC

>ASV1065 GS|100.0|EU791281_S001153684;k:Bacteria,p:"Proteobacteria",c:Gammaproteobacteria,o:Pseudomonadales,f:Pseudomonadaceae,g:Pseudomonas;

TACAGAGGGTGCAAGCGTTAATCGGAATTACTGGGCGTAAAGCGCGCGTAGGTGGTTTGT

TAAGTTGGATGTGAAAGCCCCGGGCTCAACCTGGGAACTGCATCCAAAACTGGCAAGCTA

GAGTACGGTAGAGGGTGGTGGAATTTCCTGTGTAGCGGTGAAATGCGTAGATATAGGAAG

GAACACCAGTGGCGAAGGCGACCACCTGGACTGATACTGACACTGAGGTGCGAAAGCGTG

GGGAGCAAAC

>ASV1066 GS|98.8|FM998003_S002355582;k:Bacteria,p:"Actinobacteria",c:Actinobacteria,o:Actinomycetales,g:Motilibacter;

TACGTAGGGTGCAAGCGTTGTCCGGAATTATTGGGCGTAAAGAGCTCGTAGGTGGCAGGT

CACGTCGGGTGTGAAAGCCCGGGGCTTAACCCCGGGTCTGCATTCGATACGGGCTTGCTA

GGGTCCGGCAGGGGAGACTGGAATTCCTGGTGTAGCGGTGAAATGCGCAGATATCAGGAG

GAACACCGGTGGCGAAGGCGGGTCTCTGGGCCGGTACCGACGCTGAGGAGCGAAAGCATG

GGGAGCAAAC

>ASV1067 GS|0.0|None;No hit

GTGTCAGCCGAGATGTACAAGTATAAAGCAAGGAATCTATAGATTAGAGGTAACGAATAC

GAACCTGTAAAGCTATTTGCGGTCCCTAGGACTCAGCATGAGCTCGGGGCGAACGTGTTT

GTCAAAGTCCTCTTCGGACAGAGCCTTCAATTCCATCGCACTCTCTTTGAGCGTCAAGCC

CTTTTTATGCGCATTCTTAGCGACTTTGGAAGCCATGTCGTATCCGATGACCGGGTTCAA

ACAGGTCACA

>ASV1068 SS|0.8400|KF193526_S004048651;k:Bacteria,p:"Chloroflexi"

TACGTAGGGGGCGAGCGTTGTCCGGAGTGACTGGGCGTAAAGGGCCCGCAGGCGGTCGTG

CGCGTTTTGAGTGACAGCTCCCGGCTTCACTGGGAGAGGGTTCAGAAGACGGCACGACTT

GAGGGCCAGAGAGGGACACGGAATTCCGGGTGTAGTGGTGAAATGCGTAGATATCCGGAG

GAACACCGAAGGCGAAGGCAGTGTCCTGGCTGGTACCTGACGCTGAGGGGCGAAAGCTAG

GGGAGCGAAC

>ASV1069 SS|1.0000|AM947653_S001093907;k:Bacteria,p:"Proteobacteria",c:Alphaproteobacteria,o:Rhodospirillales,f:Acetobacteraceae

TACGAAGGGGGCTAGCGTTGCTCGGAATGACTGGGCGTAAAGGGCGCGTAGGCGGTCACG

ATAGTCAGATGTGAAATTCCTGGGCTTAACCTGGGGACTGCATTTGATACTGTGTGGCTT

GAGTGTGGAAGAGGGTCGTGGAATTCCCAGTGTAGAGGTGAAATTCGTAGATATTGGGAA

GAACACCGGTGGCGAAGGCGGCGACCTGGTCCATAACTGACGCTGAGGCGCGAAAGCGTG

GGGAGCAAAC

>ASV1070 GS|99.2|AM778124_S000941857;k:Bacteria,p:"Actinobacteria",c:Actinobacteria,o:Actinomycetales,f:Nakamurellaceae,g:Nakamurella;

TACGTAGGGTGCAAGCGTTGTCCGGAATTATTGGGCGTAAAGAGCTCGTAGGCGGTCTGT

CGCGTCGAATGTGAAACCCCGAGGCTCAACTTCGGGCCTGCATTCGATACGGGCAGACTA

GAGTTCGGTAGGGGAGTCTGGAATTCCTGGTGTAGCGGTGAAATGCGCAGATATCAGGAG

GAACACCGGTGGCGAAGGCGGGACTCTGGGCCGATACTGACGCTGAGGAGCGAAAGCGTG

GGGAGCAAAC

>ASV1071 GS|0.0|None;No hit

GTGTCAGCCGCCGCAAGGACAAAGACCAGCTCTTAGGGAAACGACCTGCTTTCTATGGCG

ACGAGGCTTGCCGATTACTGGAGATTGCTGGGGATGAGCTTATTTCAGAGGCTACCGGAG

CGGCACCTGGCGACAACGTAGTCC

>ASV1072 GS|0.0|None;No hit

TTCCAGCTCCAATAGCGTATATTAAAGTTGTTGCAGTTAAAAAGCTCGTAGTTGAAACTT

GGGCCTGGCTGGCCGGTCCGCCTCACCGCGTGCACTGGTCCGGCCGGGCCTTTCCTTCTG

GGGAGCCGCATGCCCTTCATTGGGTGTGCCGGGGAACCAGGACTTTTACTTTGAAAAAAT

TAGAGTGTTCAAAGCAGGCCTATGCTCGAATACATTAGCATGGAATAATGGAATAGGACG

TGTGGTTCTA

>ASV1073 SS|1.0000|AB921558_S004078667;k:Bacteria,p:"Bacteroidetes"

TACGGAGGGTGCAAGCGTTATCCGGATTCATTGGGTTTAAAGGGTGCGTAGGCGGAATAT

TAAGTCAGTGGTGAAATCCTGCAGCTCAACTGTAGACTTGCCATTGATACTGGTATTCTT

GAGTGCGCTTGAAGTAGGCGGAATGTGCCGTGTAGCGGTGAAATGCTTAGATATGGCACA

GAACACCAATTGCGAAGGCAGCTTACTAAGGCGATACTGACGCTGAGGCACGAAAGCGTG

GGGATCGAAC

>ASV1074 GSL|99.2|AB245358_S000627894;k:Bacteria,p:"Proteobacteria",c:Betaproteobacteria,o:Burkholderiales,f:Comamonadaceae

TACGTAGGGTGCAAGCGTTAATCGGAATTACTGGGCGTAAAGCGTGCGCAGGCGGTTATA

TAAGACAGTTGTGAAATCCCCGGGCTCAACCTGGGAACTGCATCTGTGACTGTATAGCTA

GAGTACGGTAGAGGGGGATGGAATTCCGCGTGTAGCAGTGAAATGCGTAGATATGCGGAG

GAACACCGATGGCGAAGGCAATCCCCTGGACCTGTACTGACGCTCATGCACGAAAGCGTG

GGGAGCAAAC

>ASV1075 SS|0.9900|FN391026_S001418752;k:Bacteria,p:"Planctomycetes",c:Planctomycetia,o:Planctomycetales,f:Planctomycetaceae

GACGAACCGTGCGAACGTTATTCGGAATCACTGGGCTTAAAGCGAGTGTAGGCGGGCCGG

CACGTCGAGTGCTGAAATGCCCCGGCTTAACCGGGGCACTGGCATCGATACGACCGGCCT

TGAGGGGAGTAGGGGGGTCGGGAACTTCCGGTGGAGCGGTGAAATGCGTTGAGATCGGAA

GGAACGCCCGTGGCGAAAGCGCGGCCCTGGACTCTTTCTGACGCTGAGACTCGAAAGCTA

GGGGAGCGAA

>ASV1076 SS|1.0000|AM180156_S000701194;k:Bacteria

TACGTAGGGGGCGAGCGTTGTCCGGATTTATTGGGCGTAAAGCGCACGCAGGCGGTCGTG

TAAGTTTGGGGTGACAGCCGTCGGCTTAACCGAGGGAGTGTCCCAAAGACTGCAGGACTT

GAGGGCCAAAGAGGAACACAGAATTCCGGGTGTAGTGGTGAAATGCGTAGATATCCGGAG

GAATACCAAAGGCGAAGGCAGTGTTCTGGGTGGCTTCTGACGCTCAGGTGCGAAAGCTAG

GGGAGCGAAC

>ASV1077 SS|1.0000|AB859260_U010573780;k:Bacteria,p:"Bacteroidetes",c:Cytophagia,o:Cytophagales,f:Cytophagaceae,g:Hymenobacter

TACGGAGGGTGCGAGCGTTGTCCGGATTTATTGGGTTTAAAGGGTGCGTAGGCGGCTTGG

TAAGTCTGGGGTGAAAGCCCGCTGCTCAACAGCGGAACTGCCCTGGATACTGCGAAGCTT

GAGGACAGACGAGGTTGGCGGAATGGAGGGTGTAGCGGTGAAATGCATAGATACCCTCCA

GAACCCCGATTGCGAAGGCAGCTGACTAGACTGTAACTGACGCTGAGGCACGAAAGCGTG

GGGAGCGAAC

>ASV1078 GS|0.0|None;No hit

TACGGGGGGGGCAAGCGTTATTCGAAATGATTGGGCGTAAAGGGCACGTAGACGGTTTTT

TAAGTGGCCATCCTTGTTTTTGTTTTTCCCTTTCAACTCTATTATAAATAAAATACATAG

AAGGGAAACTGGAAAAGGGAAGCAAAGATTCAAATGGAGTGTGGATTCTCTTTATTTTTA

TAATCCCTTATTTGTATAAATTATTTATAATGAAAACAAATAAGGGATAACAAGATTCTA

CACTTGGGAA

>ASV1079 SS|0.8600|AM231587_S000650722;k:Bacteria,p:"Proteobacteria",c:Alphaproteobacteria,o:Rhodospirillales,f:Acetobacteraceae,g:Acidisoma

TACGAAGGGGGCTAGCGTTGCTCGGAATGACTGGGCGTAAAGGGCGCGTAGGCGGATTGG

TCAGTCAGACGTGAAATTCCTGGGCTCAACCTGGGGACTGCGTTTGAGACGGCTGGTCTA

GAGTTTGGAAGAGGGTCGTGGAATTCCCAGTGTAGAGGTGAAATTCGTAGATATTGGGAA

GAACACCGGTGGCGAAGGCGGCGACCTGGTCCTTGACTGACGCTGAGGCGCGAAAGCGTG

GGGAGCAAAC

>ASV1080 SS|1.0000|EF457488_S000834913;k:Bacteria

TACGTAGGAGGCGAGCGTTGTCCGGAATTACTGGGCGTAAAGAGCGCGTAGGCGGCGCCG

TACGACTCGTGTGAAAGCCCCCGGCTCAACTGGGGAGGGTCACGAGTAACGGCGGTGCTT

GGAGTGCAGGAGAGGGAAGTGGAACTCCGGGAGTAGCGGTGAAATGCGTAGATACCCGGA

GGAACACCAGTGGCGAAGGCGGCTTCCTGGTCTGCGACTGACGCTGTGGCGCGAAAGCTA

GGGGAGCGAA

>ASV1081 GS|99.6|FM998003_S002355582;k:Bacteria,p:"Actinobacteria",c:Actinobacteria,o:Actinomycetales,g:Motilibacter;

TACGTAGGGTGCAAGCGTTGTCCGGAATTATTGGGCGTAAAGAGCTCGTAGGTGGCGGGT

CACGTCGGGTGTGAAAGCCCGGGGCTTAACCCCGGGTCTGCATTCGATACGGGCTTGCTA

GGGTCCGGCAGGGGAGACTGGAATTCCTGGTGTAGCGGTGAAATGCGCAGATATCAGGAG

GAACACCGGTGGCGAAGGCGGGTCTCTGGGCCGGTACCGACGCTGAGGAGCGAAAGCGTG

GGGAGCAAAC

>ASV1082 GS|97.6|AB267476_S000721190;k:Bacteria,p:"Bacteroidetes",c:Sphingobacteriia,o:"Sphingobacteriales",f:Chitinophagaceae,g:Flavisolibacter;

TACGGAGGGTGCAAGCGTTATCCGGATTCACTGGGTTTAAAGGGTGCGTAGGAGGGCAGG

TAAGTCAGTGGTGAAATCTCCGAGCTTAACTTGGAAACTGCCGTTGATACTATCTGTCTT

GAATACCGTGGAGGTGAGCGGAATATGTCATGTAGCGGTGAAATGCTTAGATATGACATA

GAACACCAATTGCGAAGGCAGCTCACTACACGCATATTGACTCTGAGGCACGAAAGCGTG

GGGATCAAAC

>ASV1083 GS|97.6|GQ161990_S001575468;k:Bacteria,p:"Bacteroidetes",c:Sphingobacteriia,o:"Sphingobacteriales",f:Sphingobacteriaceae,g:Pedobacter;

TACGGAGGATCCAAGCGTTATCCGGATTTATTGGGTTTAAAGGGTGCGTAGGCGGCTTAT

TAAGTCAGGGGTGAAAGACGGTGGCTCAACCATCGCAGTGCCTTTGATACTGATAAGCTT

GAATGTACATGAGGTAGGCGGAATGTGACAAGTAGCGGTGAAATGCATAGATATGTCACA

GAACACCGATTGCGAAGGCAGCTTACTAAAGTATAATTGACGCTGAGGCACGAAAGCGTG

GGGATCAAAC

>ASV1084 GS|99.6|EF466120_S000843260;k:Bacteria,p:"Actinobacteria",c:Actinobacteria,o:Actinomycetales,f:Nocardioidaceae,g:Marmoricola;

TACGTAGGGTGCGAGCGTTGTCCGGAATTATTGGGCGTAAAGGGCTCGTAGGCGGTCTGT

TGCGTCAGGAGTGAAAACTCGGGGCTTAACCCCGAGCCTGCTTCTGATACGGGCAGACTA

GAGGTATGCAGGGGAGAACGGAATTCCTGGTGTAGCGGTGAAATGCGCAGATATCAGGAG

GAACACCGGTGGCGAAGGCGGTTCTCTGGGCATTACCTGACGCTGAGGAGCGAAAGTGTG

GGGAGCGAAC

>ASV1085 SS|1.0000|JF803808_S002913330;k:Bacteria,p:"Bacteroidetes",c:Sphingobacteriia,o:"Sphingobacteriales",f:Chitinophagaceae

TACGGAGGGTGCAAGCGTTATCCGGATTCACTGGGTTTAAAGGGTGCGTAGGTGGGTTGG

TAAGTCAGTGGTGAAATCTCCGGGCTTAACCCGGAAACTGCCATTGATACTACTGGTCTT

GAATGTCGTGGAGGTGAGCGGAATATGTCATGTAGCGGTGAAATGCTTAGATATGACATA

GAACACCAATTGCGAAGGCAGCTCACTACACGCATATTGACACTGAGGCACGAAAGCGTG

GGGATCAAAC

>ASV1086 GS|98.8|AB778259_S003784776;k:Bacteria,p:"Actinobacteria",c:Actinobacteria,o:Actinomycetales,f:Intrasporangiaceae,g:Janibacter;

TACGTAGGGTGCGAGCGTTGTCCGGATTTATTGGGCGTAAAGAGCTTGTAGGCGGTCTGT

CGCGTCTGCTGTGAAAATCCGAGGCTCAACCTCGGACTTGCAGTGGGTACGGGCAGACTA

GAGTGTGGTAGGGGAGACTGGAATTCCTGGTGTAGCGGTGAAATGCGCAGATATCAGGAG

GAACACCGATGGCGAAGGCAGGTCTCTGGGCCACTACTGACGCTGAGAAGCGAAAGCATG

GGGAGCGAAC

>ASV1087 SS|0.8100|AY140238_S000397413;k:Bacteria,p:"Proteobacteria",c:Alphaproteobacteria,o:Rhodospirillales,f:Acetobacteraceae,g:Acidisoma

TACGAAGGGGGCTAGCGTTGCTCGGAATGACTGGGCGTAAAGGGCGCGTAGGCGGATTAG

ATAGTCGGGCGTGAAATTCCTGGGCTCAACCTGGGGACTGCGTTCGAGACGTCTGGTCTA

GAGTTTGGAAGAGGGTCGTGGAATTCCCAGTGTAGAGGTGAAATTCGTAGATATTGGGAA

GAACACCGGTGGCGAAGGCGGCGACCTGGTCCTTGACTGACGCTGAGGCGCGAAAGCGTG

GGGAGCAAAC

>ASV1088 SS|1.0000|HQ436499_S002339679;k:Bacteria,p:"Bacteroidetes",c:Sphingobacteriia,o:"Sphingobacteriales",f:Chitinophagaceae

TACGGAGGGTGCAAGCGTTATCCGGATTCACTGGGTTTAAAGGGTGCGTAGGTGGGTCTG

TAAGTCAGTGGTGAAATCTCCAAGCTTAACTTGGAAACTGCCGTTGATACTATAGCTCTT

GAATGTTGTGGAGGTGAGCGGAATATGTCATGTAGCGGTGAAATGCTTAGATATGACATA

GAACACCAATTGCGAAGGCAGCTCACTACACAAATATTGACACTGAGGCACGAAAGCGTG

GGGATCAAAC

>ASV1089 SS|1.0000|FN600641_S001794056;k:Bacteria,p:"Actinobacteria",c:Actinobacteria,o:Actinomycetales

TACGTAGGGTGCAAGCGTTGTCCGGAATTATTGGGCGTAAAGAGCTCGTAGGCGGTCTGT

CGCGTCGGCTGTGAAAACTCGAGGCTCAACCTCGGGCCTGCAGTCGATACGGGCAGACTA

GAGTTCGGTAGGGGAGACTGGAATTCCTGGTGTAGCGGTGGAATGCGCAGATATCAGGAG

GAACACCGGTGGCGAAGGCGGGTCTCTGGGCCGAAACTGACGCTGAGGAGCGAAAGCGTG

GGGAGCAAAC

>ASV1090 SS|0.8800|KJ528316_S004225669;k:Bacteria,p:"Proteobacteria",c:Alphaproteobacteria,o:Sphingomonadales,f:Sphingomonadaceae

TACGGAGGGGGCTAGCGTTGTTCGGAATTACTGGGCGTAAAGCGCACGTAGGCGGCTATT

CAAGTCAGGGGTGAAAGCCCGGAGCCCAACTCCGGAACTGCCTCTGAAACTAGGTAGCTT

GAATCATGGAGAGGCGAGTGGAATTCCGAGTGTAGAGGTGAAATTCGTAGATATTCGGAA

GAACACCAGTGGCGAAGGCGGCTCGCTGGACATGTATTGACGCTGAGGTGCGAAAGCGTG

GGGAGCAAAC

>ASV1091 SS|0.8600|KF360052_S004051835;k:Bacteria,p:"Proteobacteria",c:Alphaproteobacteria,o:Caulobacterales,f:Caulobacteraceae,g:Caulobacter

TACGAAGGGGGCTAGCGTTGCTCGGAATTACTGGGCGTAAAGGGAGCGTAGGCGGATGCT

TAAGTTAGAGGTGAAAGCCCAGGGCTCAACCTTGGAACTGCCTTTGATACTGGGCATCTT

GAGTGTGGGAGAGGTAAGCGGAACTCCGAGTGTAGAGGTGAAATTCGTAGATATTCGGAA

GAACACCAGTGGCGAAGGCGGCTTACTGGCCCATTACTGACGCTGAGGCTCGAAAGCGTG

GGGAGCAAAC

>ASV1092 GS|97.2|EU181225_S000966508;k:Bacteria,p:"Actinobacteria",c:Actinobacteria,o:Actinomycetales,f:Geodermatophilaceae,g:Modestobacter;

TACGTAGGGTGCAAGCGTTGTCCGGAATTATTGGGCGTAAAGAGCTCGTAGGCGGTCTGT

CGCGTCGGCTGTGAAAACTCGGGGCTCAACCCCGAGCCTGCAGTCGATACGGGCAGACTA

GAGTGCTGTAGGGGAGACTGGAATTCCTGGTGTAGCGGTGAAATGCGCAGATATCAGGAG

GAACACCGGTGGCGAAGGCGGGTCTCTGGGCAGTAACTGACGCTGAGGAGCGAAAGCGTG

GGGAGCGAAC
[truncated: 5,195,621 more chars]
